# Unravelling Hidden Trophic Interactions Among Sea Urchin Juveniles and Macroinvertebrates by DNA Amplification

**DOI:** 10.1111/mec.70163

**Published:** 2025-11-13

**Authors:** Alberto Sutera, Chiara Bonaviri, Francesco Di Trapani, Francesca La Bella, Vesna Macic, Olivera Marković, Bernat Hereu, Roberto De Michele

**Affiliations:** ^1^ Institute of Biosciences and Bioresources (IBBR), Italian National Research Council (CNR) Palermo Italy; ^2^ Department of Earth and Sea Sciences University of Palermo Palermo Italy; ^3^ Fano Marine Center, Department of Integrative Marine Ecology Stazione Zoologica Anton Dohrn Fano Italy; ^4^ Institute of Marine Biology University of Montenegro Kotor Montenegro; ^5^ Department of Evolutionary Biology, Ecology and Environmental Sciences Institut de Recerca de la Biodiversitat (IRBIO), University of Barcelona Barcelona Spain

**Keywords:** *Arbacia lixula*, barren, metabarcoding, micropredation, molecular forensic, mtDNA, *Paracentrotus lividus*, sea urchins

## Abstract

Rocky reefs may shift between two distinct stable states: productive algal forests, characterised by high abundance and biodiversity of macrofauna, and impoverished barrens, dominated by overgrazing sea urchins. Barren states may persist despite the recovery of adult sea urchin predators, suggesting additional stabilising mechanisms. Sea urchin settlers equally colonise forests and barrens in large numbers, but in forests only a few of them reach adult size, suggesting that post‐settlement predation might play a crucial role in determining sea urchin population density. Visual assessment of predation events in the field is unfeasible due to the microscopic scale of both predators and prey and the complexity of the arena. In this study, we designed and tested specific primers for the detection of mtDNA of settlers of the Mediterranean sympatric sea urchin species *Paracentrotus lividus* and *Arbacia lixula* in the stomach content of macroinvertebrates. By testing 360 invertebrates collected in algal forests during an urchin settling event at five mtDNA loci, we identified 60 (17%) samples positive for *P. lividus* DNA. Presence of urchin DNA was confirmed by sequencing and NGS metabarcoding analyses. Our results suggest that predation by macroinvertebrates may represent an important process in controlling sea urchin population density and maintaining the forest state in temperate rocky reefs.

## 1 Introduction

On rocky reefs, one of the most striking transformations is the shift from lush macroalgal forest to barren ground caused by sea urchin overgrazing (Ling et al. 2015). Forests, characterised by erect algae, boast complex architecture and high species diversity, whereas barrens are structurally simple, with low diversity and dominated by sea urchins and encrusting organisms (Graham 2004; Pinna et al. 2020; Steneck et al. 2002; Taylor 1998). Transitions between these states occur globally due to various factors such as the loss of top‐down control over sea urchins, habitat destruction, increases in seawater temperatures and heat waves and the consequent changes in species physiology and distribution (Bonaviri et al. 2017; Jackson et al. 2001; Mann 1982; Sala et al. 1998; Steneck et al. 2002). Once an ecosystem shifts, hysteresis mechanisms maintain the new state, even if initial conditions are restored (Baskett and Salomon 2010; Scheffer and Carpenter 2003; Suding et al. 2004). Upon establishment, barren states often see high sea urchin density and biomass, further stabilising the state (Bonaviri et al. 2011; Gianguzza et al. 2010; Knowlton 2004; Ling et al. 2009; Steneck et al. 2002). Research indicates that large predators of adult sea urchins can reverse urchin barrens and sustain forests (Bernstein et al. 1981; Clemente et al. 2009; Guidetti and Sala 2007; Jackson et al. 2001; Ling et al. 2009; Mann 1982; Sala et al. 1998; Shears and Babcock 2002, 2003). However, despite the recovery of adult sea urchin predators, high sea urchin densities and barren communities can persist for years (Babcock et al. 2010; Foster 1990; Pinnegar et al. 2000), suggesting the presence of other stabilising mechanisms (Agnetta et al. 2024; Ling et al. 2015; Sala 2004). As a matter of fact, although the role of sea urchins in structuring rocky reef communities is well known, drivers of sea urchins' demographics are poorly understood. Recruitment and post‐settlement survival are fundamental processes in sea urchin population dynamics (Hereu et al. 2012). Since urchin recruits are particularly vulnerable to predation, their mortality may represent one of the most important drivers determining urchin population density (Andrew and Choat 1985; Harrold et al. 1991; Jennings and Hunt 2010; Prog et al. 1987; Rowley 1989). After the planktonic larval stage, settlement induction and recruits density appear independent of community state or conspecific abundance, with young urchin settlers equally present in both forest and barren (Balch and Scheibling 2001; Cameron and Schroeter 1980; Hereu et al. 2004; Hernández et al. 2010; McNaught Douglas Colin 1999; Prado et al. 2009; Privitera et al. 2011; Rowley 1989; Schroeter et al. 1996). In the Mediterranean, two sea urchin species, *Paracentrotus lividus* and *Arbacia lixula* , have a key role in the dynamics between algal forests and barren grounds. At high densities, *P. lividus* can form large areas of barren grounds by grazing on erect algae thallus (Agnetta et al. 2015). Barren is then maintained by the co‐occurring thermophilus species *A. lixula* (Bonaviri et al. 2011; Gianguzza et al. 2011; Privitera et al. 2011). The population of *P. lividus* juveniles rapidly drops in algal forests, despite the presence of structural refuges in forests protects small urchins from predation by fishes (Boada et al. 2018; Hereu et al. 2004). Conversely, little is known about *A. lixula* juveniles survival. As a result of these processes, adults of both species are typically rare in forests, and abundant in barrens. It has long been postulated that invertebrate micropredators are responsible for the control of urchin settlers (Bonaviri et al. 2012; Clemente et al. 2013; Hereu et al. 2005; Jennings and Hunt 2010; McNaught Douglas Colin 1999; Scheibling and Hamm 1991; Scheibling and Robinson 2008; Steneck et al. 2013). Predation is a significant cause of early benthic invertebrate mortality (Gosselin and Qian 1997; Griffiths and Gosselin 2008; Hunt and Scheibling 1997; Osman and Whitlatch 1992, 1995, 2004; Paine 1992; Sala and Graham 2002). Algal forests, due to their greater habitat complexity, host a larger abundance and diversity of microfauna, hence potentially micropredators, than structurally simple barren grounds (Christie et al. 2009; Hauser et al. 2006; Norderhaug et al. 2012; Pinna et al. 2020). Consequently, the disappearance of urchin settlers in forests might be due to predation by invertebrates (Steneck et al. 2013).

Experiments in aquaria showed that several decapod species are able to feed on urchin settlers, especially larger specimens (Bonaviri et al. 2012; Fagerli et al. 2014; Feehan et al. 2014). However, the number and variety of invertebrates tested are limited, and aquaria are artificial, oversimplified systems which poorly encompass the real trophic dynamics in the field. Traditional techniques for in situ identification of predation events involving marine invertebrates face paramount challenges, due to the small scale of both predators and prey, the presence of algal canopy, frequent nocturnal activity and disturbance by the observer. Mounting cameras on the seabed may ease some of these problems, but it still presents a limited temporal and spatial arena, often deprived of canopy and other organisms, or where prey are tethered to the substratum. As a result, predation rates measured by visual scoring might be overestimated (Fagerli et al. 2014). Analysis of gut content is problematic as well, since many invertebrates are fluid feeders, avoid consuming indigestible remains, or fully digest samples leaving no recognisable material.

In the last decades, several studies have sequenced environmental DNA (eDNA) to study the trophic interactions in invertebrates (Clare 2014; Cuff et al. 2022; Pompanon et al. 2012; Symondson 2002). Amplification of prey's DNA by polymerase chain reaction (PCR) allows the detection of trace amounts of undigested prey material in predator gut content and in faeces.

Here, we applied molecular techniques to untangle the trophic interactions involving urchin settlers and their potential micropredators. We considered the sea urchins *Paracentrotus lividus* and *Arbacia lixula* , which cause the shift from algal forest to barren grounds in Mediterranean rocky reefs (Agnetta et al. 2015; Bonaviri et al. 2011; Hereu et al. 2008).

In particular, we aimed to: (1) design specific primers for the detection of DNA of the Mediterranean urchin species *P. lividus* and *A. lixula* in degraded samples; (2) identify the invertebrates belonging to different taxa collected during the urchin settlement stage, by both visual assignment and molecular barcoding; (3) detect potential consumers of urchin settlers, by selective amplification of urchin DNA contained in the stomachs of macroinvertebrates.

The extent of micropredation as a process controlling urchin populations in temperate reefs is discussed.

## 2 Materials and Methods

This study focuses on the detection of potential predation events on urchin settlers by macroinvertebrates through molecular analyses. Samples were collected in the field and analysed in the laboratory to individuate and visually identify urchin settlers and their potential predators from which DNA was extracted and analysed.

### 2.1 Sample Collection

**FIGURE 1:**
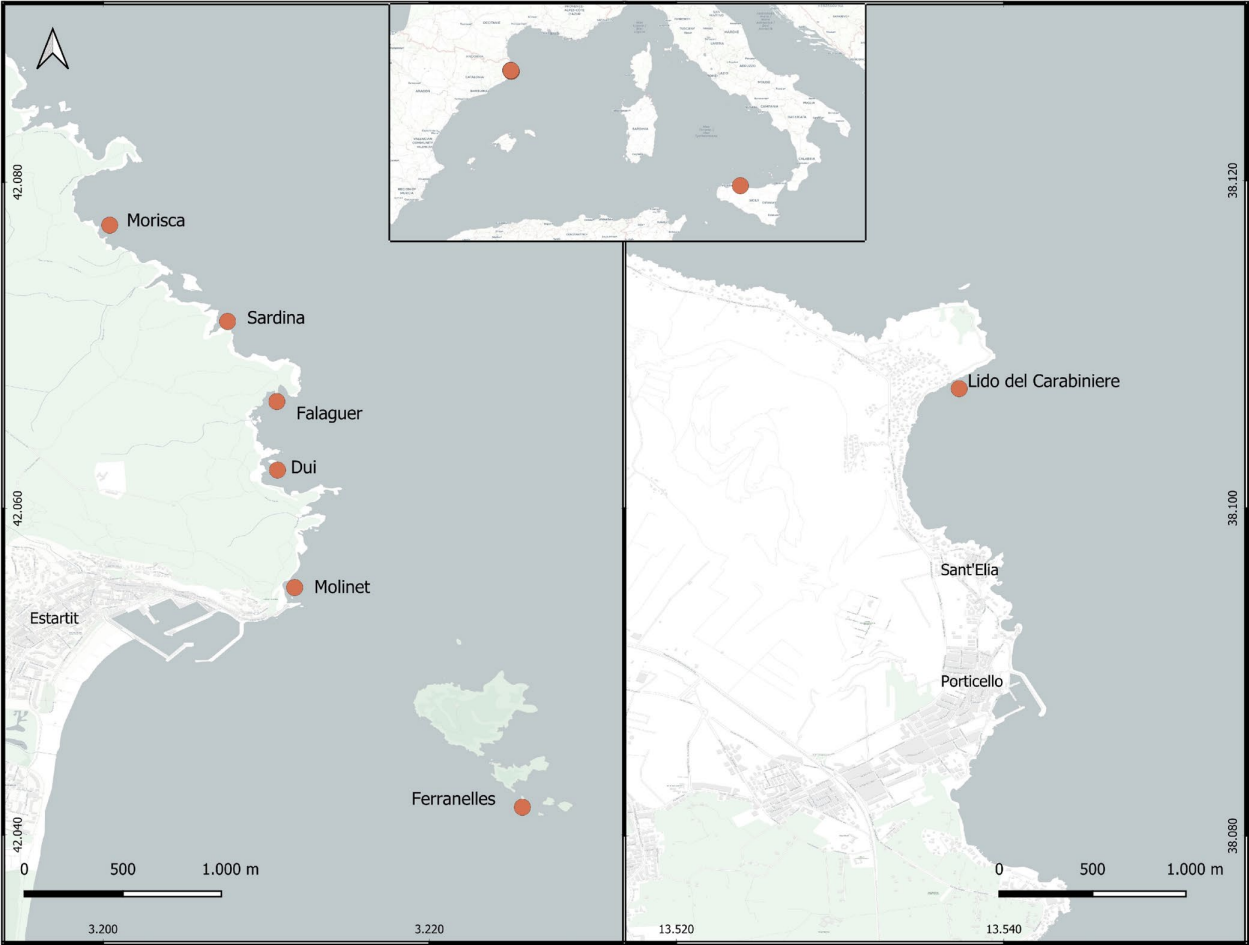
Maps of the sampling sites in Spain and Italy.

Sampling was carried out by SCUBA diving at six sites along the Montgrí massif (42.8160 N, 03.8130 E), Spain, northwestern Mediterranean Sea, and at one site on the Tyrrhenian coast of Sicily (38.1086 N, 13.5382 E), Italy, central Mediterranean Sea (Figure 1).

The communities studied are characterised by a variability of habitats, mainly photophilic algal communities dominated by *Gongolaria elegans*, alternating with turf communities dominated by filamentous algae and sea urchin barrens (Table 1).

**TABLE 1:** Sampling sites and features.

Site	Habitat	Depth (m)	Date
Molinet (Spain)	Dictyotaceae turf	5–7	14‐15‐19‐27‐28/07/2017
Morisca (Spain)	Dictyotaceae/turf	8	15/07/2017
Sardina (Spain)	Gongolaria/Dictyotaceae	6	13/07/2017
Falaguer (Spain)	Dictyotaceae turf	6–8	13‐14‐19‐20‐24‐27‐28/07/2017
Ferranelles‐Medes (Spain)	Dictyotaceae turf	7	24/07/2017
Dui (Spain)	Turf/barren	8	19/07/2017
Lido del Carabiniere (Italy)	Dictyotaceae turf	5	04/07/2017

As the settlement of *P. lividus* occurs in early July, albeit with some variability (Hereu et al. 2004), weekly prospective sampling started in June to be sure that we would detect the peak of settlement. From July 4th to 28th, 2017, we observed a large number of sea urchin recruits on algal assemblages at 5–8 m depth. Small invertebrates (1–35 mm) and urchin recruits were then collected by scraping a small area (25 × 25 cm^2^) of algae‐covered rocky substrate by SCUBA diving, then sorted under a stereomicroscope in the laboratory on the same day of collection. Specimens were individually stored in plastic tubes in 1–10 mL of 70% ethanol, depending on the animal size and kept at −20°C until use. We paid attention to avoid cross‐contamination between samples, by changing gloves and cleaning tweezers for each specimen.

In order to collect urchin recruits, the scraped material was placed in a salver containing a thin layer of seawater and covered with a plastic grid. After a few hours, the small sea urchins climbed actively out of the salver onto the surface of the plastic grid, probably in response to oxygen shortage (Bonaviri et al. 2012). Sea urchins were then carefully collected with tweezers and individually stored as described above. As a precaution to avoid DNA contamination with micropredators, the tubes containing sea urchins were stored separately in a different box, in a different freezer.

Adults of *P. lividus* and *A. lixula* , whose DNA was used as positive controls in primer tests, were collected at the Sicilian locality.

All animals collected in this study were invertebrates and non‐cephalopods; therefore not subject to Directive 2010/63/EU restrictions. None of them belonged to endangered or protected species.

### 2.2 Taxonomic Identification

Invertebrates were visually classified in two rounds: at the moment of collection, and before DNA extraction. Samples were individually visualised under a stereomicroscope in the laboratory and photographed. With the aid of manuals (Falciai and Minervini 2008; Riedl 2010; Trainito and Baldacconi 2014), we identified the specimens at the lowest possible taxonomic level (Figures S1–S7; Tables S1–S6).

For molecular taxonomic identification of both urchin juveniles and potential predators by DNA barcoding, we used the ‘universal’ *cytochrome oxidase subunit 1* (*COI1*) degenerate primer pair jgLCO1490/jgHCO2198 (Table 2) described by (Geller et al. 2013), particularly effective for marine invertebrates.

**TABLE 2:** Primers used in this study.

Species	Locus	Name of primer	Sequence (5′‐3′)	Amplicon size	References
Universal	*COI1*	jgLCO1490	titciaciaaycayaargayattgg	400–1000	(Geller et al. 2013)
		jgHCO2198	taiacytciggrtgiccraaraayca		
Universal	*COI1*	mICOIintF	ggwacwggwtgaacwgtwtayccycc	313	(Geller et al. 2013; Leray et al. 2013)
		jgHCO2198	taiacytciggrtgiccraaraayca		
*P. lividus*	*COI1*	Pliv‐COI‐A‐for	actacccggatttggaatgatt	63	This study
		Pliv‐COI‐A‐rev	aaaggttctcgcttccctga		
*P. lividus*	*COI1*	Pliv‐COI‐B‐for	ttctcactccatcttgcggg	93	This study
		Pliv‐COI‐B‐rev	aaaagacattcccggcgttc		
*P. lividus*	*COI1*	Pliv‐COI‐C‐for	actatgcttctaacagaccgt	178	This study
		Pliv‐COI‐C‐rev	tctcgcttccctgagtagtg		
*P. lividus*	*COI1*	Pliv‐COI‐D‐ for	gcaccagatatggccttccc	103	This study
		Pliv‐COI‐D‐ rev	cggctcctctttctactcctg		
*P. lividus*	*COI1*	Pliv‐COI‐E‐ for	gaacgccgggaatgtctttt	173	This study
		Pliv‐COI‐E‐ rev	aattgggtctcctcctcctg		
*P. lividus*	*CYTb*	Pliv‐CYTb‐A‐for	tctggtggaaattcggctct	155	This study
		Pliv‐CYTb‐A‐rev	tcgaagcagtcacccgtaat		
*P. lividus*	*CYTb*	Pliv‐CYTb‐B‐for	ttccgtcccctatctcaagc	86	This study
		Pliv‐CYTb‐B‐rev	ggaagccaacctgtagaaca		
*P. lividus*	*CYTb*	Pliv‐CYTb‐C‐for	agacaatgccactctaactcg	119	This study
		Pliv‐CYTb‐C‐rev	cctactgggttgttggctcc		
*P. lividus*	*16S*	Pliv‐16S‐for	aggcggagggtaaaatcgtt	161	This study
		Pliv‐16S‐rev	gcttcttttactccgcggtt		
*A. lixula*	*COI1*	Alix‐COI‐A‐for	atgctgggaagagagaacca	97	This study
		Alix‐COI‐A‐rev	aacatgtggtgggctcaaac		
*A. lixula*	*COI1*	Alix‐COI‐B‐for	tgggagcagtcttcgctatt	76	This study
		Alix‐COI‐B‐rev	agtgggtggaagctgtatcc		
*A. lixula*	*COI1*	Alix‐COI‐C‐for	cccctaatgattggtgcccc	112	This study
		Alix‐COI‐C‐rev	ctctttctaccccggcagaa		
*A. lixula*	*COI1*	Alix‐COI‐D‐for	cagcaggtggaggagacc	137	This study
		Alix‐COI‐D‐rev	tggttctctcttcccagcat		
*A. lixula*	*COI1*	Alix‐COI‐E‐for	ccggtgcctcttccatctt	89	This study
		Alix‐COI‐E‐rev	ggcaaacggtcaaaagaga		
*A. lixula*	*16S*	Alix‐16S‐A‐for	tgtgacccgcttatttaggc	153	This study
		Alix‐16S‐A‐rev	acagaccaacccttaaaagct		
*A. lixula*	*16S*	Alix‐16S‐B‐for	ggcaaccacggagaaaataa	110	This study
		Alix‐16S‐B‐rev	gcctaaataagcgggtcaca		

### 2.3 DNA Extraction

For DNA extraction from both the potential predators and sea urchins, we used the Tissue DNA Kit by Ezna (VWR). Samples were removed from the ethanol bath, weighed and rinsed in 1–10 mL distilled water, depending on the animal size. For animals weighing more than 100 mg, we chopped the sample into several pieces by using a disposable blade and processed the parts independently. Since the urchin DNA would be potentially present only in the stomach of the predators, legs and claws were also removed from larger animals, whenever possible, in order to minimise the amount of host DNA recovered. Each sample was put in a 2 mL plastic tube with 3 tungsten beads and homogenised by shaking for 15 s at 300 rpm at room temperature in the TissuLyser machine (Qiagen). Immediately after shaking, we added TL buffer and proceeded according to the kit protocol for tissue. DNA was resuspended in 10 mM Tris buffer, pH 9.0. DNA concentration and extraction quality were measured at a fluorometer (Synergy H1, Biotek). DNA integrity was checked by an electrophoretic run in a 1% agarose gel.

As a test for DNA quality of the sample, we used the primer pair jgLCO1490/jgHCO2198 (Table 2) that was shown to robustly amplify a large range of marine invertebrate taxa (Geller et al. 2013).

### 2.4 Primer Design

For molecular identification of potential feeders of sea urchins, we focused on mitochondrial DNA (mtDNA), since each diploid cell contains only two copies of nuclear loci, but thousands of circular mtDNA molecules (Zhang et al. 1993), which are less prone to degradation, making it easier to detect in digested samples. Additionally, sequences of mtDNA loci are widely available in public databases for a large variety of species, facilitating the design of prey‐specific primers. We selected three loci within the mtDNA: cytochrome oxidase subunit 1 (*COI1*); cytochrome b (*CYTb*); and *16S*. *COI1*, being the barcode standard, is highly polymorphic and sequences are available for a large number of individuals within species, accounting for intraspecific variability. *COI1* is also the most commonly used locus for assessing trophic relationships among species, reviewed in (King et al. 2008; Pompanon et al. 2012). *CYTb* has been used for insects, arachnida and fishes (Parsons et al. 2005; Pons 2006). Additionally, *CYTb* sequences have been used to discern the phylogeographic distribution of *P. lividus* populations in the Mediterranean (Maltagliati et al. 2010). The *16S* locus is also polymorphic, and among other species, it has been used to detect DNA of the sea urchins *Centrostephanus rodgersii* and *Heliocidaris erythrogramma* in lobster faecal samples in Tasmania (Redd et al. 2014; Smith et al. 2023).

Due to the high polymorphism of *COI1*, we were concerned that different urchin accessions might also show sequence variations. In order to design primers that would work for any *P. lividus* and *A. lixula* sequence, we aligned by ClustalW (BioEdit) 254 *COI1* sequences available on NCBI, including the complete mitochondrial genome for *P. lividus* (accessions 49036145‐49036397; 1028338044‐1028338084; 365735528‐365735742; 564282614; 564282618; J04815.1) and 328 *COI1* sequences available for *A. lixula* (JQ745096.1‐JQ745256.1; JN603630.1‐JN603633.1; AF030998.1‐AF031011.1; JF772935.1‐JF773074.1; KU172486.1‐KU172488.1; HE800533.1‐HE800538.1). In the alignment, we also included the *COI1* sequences of marine invertebrate representatives of the major taxonomic group found in this survey: *Alpheus dentipes* (AF309893.1); *Amphipholis squamata* (NSECH002‐13); *Ampithoe ramondi* (GBCM8446‐17); *Dorvillea sp* (BAMPOL0439); *Galathea intermedia* (BNSC183‐10); *Nereis pelagica* ; *Ophiothrix fragilis* (LOBO010‐12); *Pagurus prideaux* (JSDUK158); *Palola cf. siciliensis* (USNM1120744); *Pilumnus villosissimus* (GBCMD18798‐19); *Platynereis dumerilii* (GBAN12514‐19); *Synalpheus gambarelloides* (GBCDA161‐12); and *Thelepus cincinnatus* (HUNTSPOL0372), in order to avoid conserved regions during primer design.

We designed nine different primer pairs for *P. lividus* and seven for *A. lixula* , spanning different regions of each locus and amplifying bands ranging from 63 to 178 bp in size. We intentionally selected small amplification sizes in order to maximise chances of recovery of digested and fragmented prey DNA in the guts (King et al. 2008). Details of the primers are given in Table 2.

### 2.5 Amplification

In order to minimise the risk of cross‐contamination, pre‐packed, aerosol‐resistant and DNA‐free pipette tips were used for assembling PCR reactions and loading samples in gels. Different pipette sets and laboratory rooms were used when handling urchin specimens and their DNA. Pre‐PCR and post‐PCR setups were located in different rooms, as recommended in forensic studies (King et al. 2008).

All PCR reactions were assembled with 0.4 μM each primer, 1% w/v BSA and 2 units MyTaq polymerase (Bioline) in a reaction volume of 25 μL. Amplifications were performed in 96‐well plates. The conditions of PCRs with the urchin‐specific primers were: initial denaturation for 3 min at 95°C, followed by 35 cycles of denaturation for 60 s at 95°C, annealing for 30 s at 56°C, extension for 25 s at 72°C; a final extension step was set for 10 min at 72°C.

Templates in the specificity test PCR included DNA extracted from gonads of adult *P. lividus* and *A. lixula* (0.1 ng), and from invertebrates of different taxonomic groups (100 ng). In particular, we extracted DNA from the Sipunculid *Phascolosoma granulatum* ; the crab *Pilumnus hirtellus* ; the shrimp *Synalpheus gambarelloides* ; the Ophiuridae *Amphipholis squamata* ; a Terebellidae *Thelepus cincinnatus* ; the whelk *Euthria cornea*. With the exception of the Ophiuridae and the Polychaete, either too small or unstructured to be dissected, we extracted DNA from appendices of invertebrate samples, with the aim to minimise contamination with DNA from other species contained in the gut.

Templates in the sensitivity tests consisted of DNA from adults of either *P. lividus* or *A. lixula* , diluted in water from 10 ng to 10 fg (10^6^ folds dilution). For PCRs testing the inhibition effect of excess predator DNA, templates contained 0.1 ng urchin DNA and 100 ng (1000‐fold) of predator DNA. For screening all samples for the presence of urchin DNA with the urchin‐specific primers, we used 50 ng DNA as a template in each reaction, as previously done in a similar survey (Redd et al. 2014). Each reaction plate contained one positive control (0.1 ng *P. lividus* DNA) and 4–5 negative controls (reaction mixture with no template).

As test for quality and ability to amplify of all samples, we used Geller's *COI1* universal primers jgLCO1490/jgHCO2198 (Geller et al. 2013). PCR conditions were: initial denaturation 3 min at 95°C, followed by a touch down setup with 35 cycles of denaturation 60 s at 95°C, annealing 60 s at 52°C–48°C (−1°C/cycle for the first four cycles, followed by 48°C for 31 cycles), extension 60 s at 72°C; a final extension step was set for 10 min at 72°C. Additionally, amplicons from this test were sequenced and used for molecular taxonomic identification.

PCR products were loaded in a 2% agarose gel and visualised by GelRed (Biotium) staining. Band presence and intensities were automatically detected by the software GelAnalyzer 23.1.1 (www.gelanalyzer.com) and manually checked.

### 2.6 Sequencing

Bands amplified by both urchin‐specific primers and *COI1* universal primers, were manually excised from the gels and the DNA was extracted by using the Gel Extraction Kit (Ezna, VWR) according to the manufacturer's instructions. The purified DNAs were directly sequenced by forward primers (Table 2). Sanger sequencing was performed by Eurofins Genomics (Germany). The resulting sequences were blasted in the NCBI database (BLASTn) to retrieve the identity of the specimens (Tables S1–S6). The BLAST hit with the lowest E value score was retained as the putative assignment, and its identity score was annotated. Only in three cases (samples 195, 201, 243) was the second hit annotated since the first ones were clearly misassignments in the database (different Phyla). Additional taxonomic information for each species (Class, Family) was obtained from the World Register of Marine Species (WORMS https://www.marinespecies.org).

### 2.7 Next Generation Sequencing

A thorough amplicon sequencing of the locus *COI1* in invertebrate samples was performed with the Illumina‐tagged primers mICOIintF and jgHCO2198, which amplify a short fragment of around 313 bp, optimal for NGS sequencing with the MiSeq platform (Illumina) (Leray et al. 2013) (Table 2). PCR reactions were assembled with 100 ng template, 0.3 μM each primer, 1% w/v BSA and 4 units MyTaq polymerase (Bioline) in a reaction volume of 50 μL. PCR conditions were modified from (Leray et al. 2013): initial denaturation for 3 min at 95°C, followed by 16 touchdown cycles of denaturation for 45 s at 95°C, annealing for 1 min at 62°C–46°C (−1°C/cycle), extension for 1 min at 72°C; 25 more cycles with the annealing temperature fixed at 46°C; a final extension step was set for 10 min at 72°C. For each sample, five replicate amplifications were pooled and purified together in order to reduce stochastic misrepresentation of rare templates. Bands of the expected size were gel‐purified as described above and sequenced through the MiSeq platform by Eurofins Genomics. Primer trimming and merging of paired‐end reads were performed by Eurofins Genomics through Cutadapt and FLASH software, respectively. Pairs were merged with a minimum overlap size of 10 bp to reduce false positive merges. Libraries were then processed by FROGS through the Galaxy server to remove chimaeras, cluster the OTUs and assign taxonomy, based on Midori's marine database (Abueg et al. 2024; Escudié et al. 2018). Affiliation aggregation was performed on a minimum of 99% identity, the default value of FROGS. Sequencing and data processing statistics are presented in Tables S1–S6.

### 2.8 Statistics

Differences between taxonomic assignments based on microscopy vs. molecular barcoding were tested at the Class and Family levels by Kruskal–Wallis test (alpha = 0.05). The numbers of positive samples detected by the different urchin primer sets were compared by Chi‐square test of independence, since the outcome was binary (alpha = 0.05). Chi‐square was also used to compare the number of positive samples to either all urchin loci or to *16S* + *CYTB‐A* + *COI‐C*, both at the Class and at the Family levels (alpha = 0.05). For the latter tests, sample 537 was not considered because barcoding sequencing failed to provide identification at the Family level. Statistical results are presented in Tables S1–S6.

The Venn diagram used to compare the distribution of positive samples among the different loci (Figure 3b) was constructed using the webtool: https://bioinformatics.psb.ugent.be/webtools/Venn/.

## 3 Results

### 3.1 Invertebrate Assemblages

**FIGURE 2:**
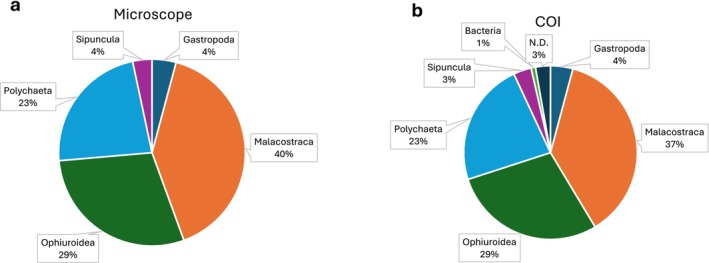
Distribution of collected samples among Classes according to visual assignment (a) and *COI1* barcode (b). ND, not determined.

A total of 360 individuals of potential predator invertebrates were collected and visualised at a stereomicroscope for taxonomic identification (Tables S1–S6). Pictures of each invertebrate are shown in Figures S1–S7. Overall, most of the collected invertebrates were crustaceans (Malacostraca, ~40%). Brittle stars (Ophiuridea) and worms (Polychaeta) were also abundant (~29% and ~23% of samples, respectively). The other invertebrates belonged to Gastropoda (~4%) and Sipuncula (~4%) (Figure 2a).

Only for ~22% of specimens we were confident enough to assign a morphological identification at the species level, especially for crustaceans (46% of all samples from Class Malacostraca). For other samples, we only reached the Genus (~18%), Family/Superfamily (~19%), Order/Infraorder (~14%) or even just the Class (~27%) taxon level. Our taxonomic confidence was especially low for Gastropods, Sipuncula or Polychaeta, to whom we could not assign any specific name. Due to our lack of taxonomic expertise for many organisms, we relied on molecular barcoding as an aid to species identification (Antil et al. 2023).

### 3.2 DNA Quality and Barcoding

To check for the integrity of the DNA extracted from preserved invertebrate samples, we analysed a subset of randomly selected samples by electrophoresis. Most samples showed an extended smear of low molecular weight DNA, indicative of degradation (Figures S1–S7). Very little high molecular weight genomic DNA was visible. Degradation was likely a result of sample storage and/or handling during the extraction protocol. Concerned about the quality of the samples, we first tested all 360 potential predators for their ability to support PCR amplification. To this end, we used Geller's ‘universal’ *COI1* primer pair that had been shown to amplify a wide range of invertebrate taxa (Geller et al. 2013). Accordingly, the vast majority (~97%) of samples gave a clear and strong band, indicating that the handling and storage of the animal and the extraction protocol did not affect the ability of the DNA to amplify (Figure S3). Only 10 invertebrate samples failed to amplify (Tables S1–S6). We excluded that the PCR failure was due to incompatibility of the primer set with the sample species, since other members of the same species showed a clear band in other reactions. More likely, the negative result for these 10 samples was due to the presence of PCR inhibitors, or to insufficient DNA quality. In all the other 350 invertebrate samples clear bands were visible with sizes ranging between 400 and 1000 bp, indicating that the recovered DNA was not fully degraded, and that a sufficient amount of nucleic acid was long enough to serve as a template (Figure S3). Since the primers for urchins were designed on a much smaller region (< 200 bp) and for genes encoded in the mtDNA, present in high copy number, we were confident that our strategy had the potential to work even when the target DNA was present in trace amounts.

The bands amplified in the *COI1* region by Geller's primers were purified and sequenced to serve as barcodes for molecular identification of invertebrates and urchins. Out of 350 invertebrate sequences, three failed to provide a reliable identification, due to misassignment to Bacteria. For these three, we relied only on the visual identification. Tables S1–S6 reports the sequences, the names of species most closely related to sequences for each sample, and the identity scores. Most of the sequences (70%) retrieved a high‐confidence hit (identity ≥ 95%). Only a few samples (5%) showed low identity confidence (75%–85%), and they were especially abundant among Polychaetes. Overall, molecular barcoding confirmed the coarse visual assignment and provided a finer labelling, in most cases (91%) to the Species level. At the Class level, the molecular identification agreed with the visual assignment for all samples. At the Family level, 16 samples (5%) showed a disagreement between *COI1* sequencing and visual assignment, especially among Gastropods. Differences between microscopy‐based assignment and barcoding were statistically not significant (Tables S1–S6). Class distribution was similar to the one obtained by visual assignment (Figure 2b); minor deviations were due to the 13 specimens that failed to provide a valuable barcode sequence.

### 3.3 Barcoding of Urchin Juveniles

In the study area, the two most abundant sea urchin species are *P. lividus* and *A. lixula* . Adults are easily recognised by colour, form and behaviour. However, small juveniles (< 5 mm) are indistinguishable, since spines are still colourless and the shape of the urchin is uniform. In our surveys we collected 55 sea urchin juveniles. Most of them (45, ~82%) were smaller than 1 mm, the rest being sized between 1 and 5 mm (Table S4). In order to identify the urchin species, we sequenced the *COI1* loci of all juveniles (Table S4). Only one sample failed to provide a reliable identification, being assigned to Bacteria. The remaining 54 juveniles were all assigned to *P. lividus* with high confidence (identity score > 92%), suggesting that the settlement event that spanned our surveys was attributed to that species.

### 3.4 Primer Efficiency

Ideal primer sets for assessing predator–prey interaction strongly amplify the prey DNA but not other organisms' DNA. In order to design primers specific for *P. lividus* , yet able to amplify *P. lividus* accessions from different Mediterranean regions, we aligned 254 mtDNA sequences of *P. lividus* from different Mediterranean areas. To ensure specificity, the alignment included 328 sequences of the sympatric sea urchin species *A. lixula* and those of their potential predators, belonging to different taxonomic groups. We then selected the genetic regions that were conserved within the same species, but that differed between the two sea urchin species, and also with other invertebrates. In particular, for *P. lividus* we designed five primer pairs for the locus of the *cytochrome oxidase c subunit 1* (*COI1*), three pairs for *cytochrome oxidase b* (*CYTb*) and one pair for the *16S* locus; for *A. lixula* , we designed five pairs for *COI1* and two pairs for *16S* (Table 2). Each primer pair was tested for specificity of amplification in a panel comprising positive controls, that is, genomic DNA of adult *P. lividus* and *A. lixula* , and negative controls, chosen among potential predator taxa. The expected band sizes were in the 63–178 bp range, in order to amplify degraded DNA.

We first tested for primer specificity. Primer sets amplified urchin DNA with different efficiencies, as observed by band intensity (Figures S1–S7). For *P. lividus* specific primers, all primers produced a strong band with *P. lividus* DNA, with the exception of Pliv‐CYTb‐B, which failed to amplify. Pliv‐COI‐D and Pliv‐COI‐E showed unspecific bands in the predator samples. Conversely, Pliv‐COI‐A, Pliv‐COI‐B, Pliv‐COI‐C, Pliv‐CYTb‐A, Pliv‐CYTb‐C and Pliv‐16S, only showed the specific band when *P. lividus* DNA was present in the template. The short 63 bp amplicon of Pliv‐COI‐A, however, was faint and too close to the primer bands. For this reason, we excluded Pliv‐COI‐A from further analyses. For *A. lixula* , all primers produced a band in the presence of *A. lixula* DNA. However, Alix‐COI‐A, Alix‐COI‐D, Alix‐COI‐E and Alix‐16S‐B also produced unspecific bands in the presence of predator DNA. Only Alix‐COI‐B, Alix‐COI‐C and Alix‐16S‐A were specific. For their specificity in the reaction, we used primers Pliv‐COI‐B, Pliv‐COI‐C, Pliv‐CYTb‐A, Pliv‐CYTb‐C and Pliv‐16S, as *P. lividus* specific primers, and Alix‐COI‐B, Alix‐COI‐C and Alix‐16S‐A as *A. lixula* specific primers in all subsequent experiments.

Once we selected specific primers for *P. lividus* and *A. lixula* , we tested for their sensitivity, by diluting urchin DNA from 1 ng to 10 fg for *P. lividus* and from 10 ng to 100 fg for *A. lixula* (100,000 fold). A strong band was visible for all primer pairs with as little as 10 pg DNA, and fainter bands, yet detected by the automatic image analyser, up to 1 pg for Pliv‐COI‐B and Pliv‐16S (Figures S1–S7; Tables S1–S6). The selected primers therefore are sensitive enough to detect the small amount of urchin DNA present in the stomach of predators. Our tests were performed on purified DNA. However, in homogenates from whole predators and their gut content, the presence of an excess amount of predator's DNA might interfere with prey's DNA amplification. To test this hypothesis, we diluted a small amount (100 pg) of urchin DNA with a thousand‐fold excess of invertebrate DNA, selected from a representative of each major taxonomic group. All the primers amplified the corresponding urchin DNA with no interference from invertebrate DNA (Figures S1–S7), indicating that they are suitable for detecting the prey within the predators' body.

### 3.5 Detection of Urchin DNA in Invertebrate Samples

The 55 sea urchin juveniles all strongly amplified with *P. lividus* primers Pliv‐COI‐B, Pliv‐CYTb‐A and Pliv‐16S, as evidenced by the intense bands of the expected sizes (Figures S1–S7). Conversely, the same samples did not produce any band when *A. lixula‐*specific primers Alix‐16S‐A were used. This result confirmed the barcoding results, indicating that all the juveniles belonged to the species *P. lividus* .

**FIGURE 3:**
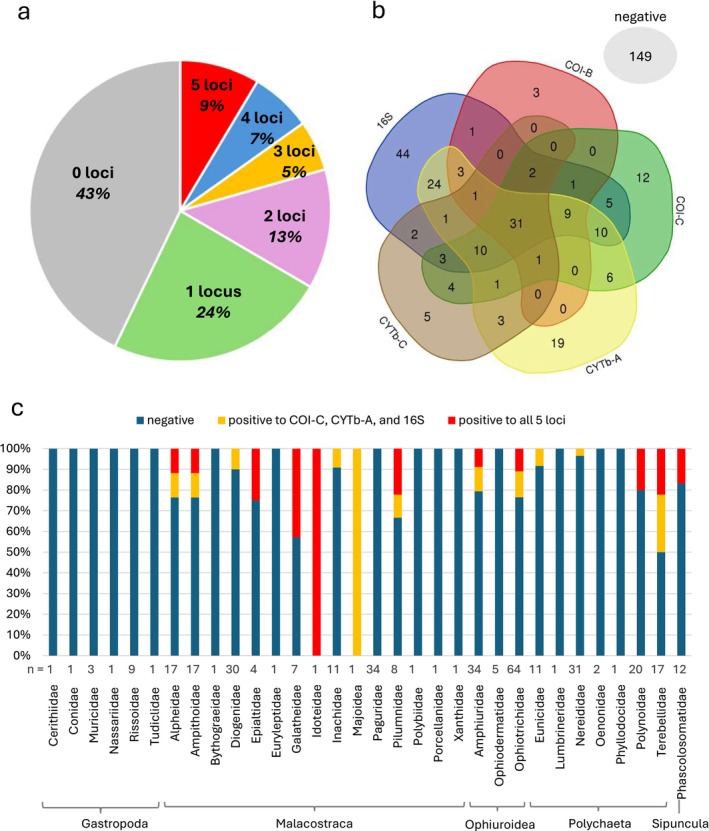
Distribution of samples showing positive response to 0–5 *P. lividus* loci (a). Distribution of samples detected by each locus (b). Proportion of samples positive to all five loci or to the three best performing primer pairs, over the total collected samples, expressed as a percentage within each Family (c). Number of samples is presented for each Family.

Next, we then tested the invertebrates' DNA with the five *P. lividus* specific primers Pliv‐COI‐B, Pliv‐COI‐C, Pliv‐CYTb‐A, Pliv‐CYTb‐C and Pliv‐16S. The 10 invertebrate samples that had failed in the amplification with Geller's universal primers also did not amplify with *P. lividus* primers, confirming that they might contain PCR inhibitors or that their DNA quality was too low. Among the remaining 350 samples, 31 (9%) scored positive for all five loci, 54 samples (15%) for at least four loci, 73 (21%) for at least three loci. An additional 45 (13%) and 83 (24%) samples showed a band for just two or one locus, respectively. Conversely, 149 samples (43%) did not show any band for any primer set (Figure 3a,b and Tables S1–S6). Pliv‐16S was the most sensitive locus, with 147 positive samples (42%), followed by Pliv‐CYTb‐A (*n* = 119, 34%), Pliv‐COI‐C (*n* = 95, 27%), Pliv‐CYTb‐C (*n* = 64, 18%) and Pliv‐COI‐B (*n* = 52, 15%) (Figure 3b). The underperforming primers Pliv‐ CYTb‐C and Pliv‐COI‐B were also the only ones showing no statistically significant differences in the numbers of positive samples with each other (Tables S1–S6). Excised and gel‐purified bands from eight positive samples and the positive control (adult *P. lividus* ) amplified with the Pliv‐16S, Pliv‐CYTb‐A and Pliv‐COI‐B primer pairs were sequenced. Though short, all sequences belonged to *P. lividus* , confirming that the DNA of this urchin species was present in the samples (Tables S1–S6).

Considering only the most conservative results from the 31 samples that strongly amplified all five *P. lividus* loci, positive invertebrates belonged to all Classes but Gastropoda, with 11 positive Malacostraca (8% of all samples from the same Class), ten Ophiuroidea (10%), eight Polychaeta (10%) and two Sipuncula (17%) (Table 3). Among Families, the rate of positive invertebrates ranged widely, with statistically significant differences (Tables S1–S6). Particularly represented were shrimps from Alpheidae and Ampithoidae (both *n* = 2, 12%); crabs from Galatheidae, Idoteidae and Pilumnidae (*n* = 3, 1 and 2, 43%, 100% and 25%, respectively); brittle stars from Ophiotrichidae and Amphiuridae (*n* = 7 and 3%, 11% and 9%, respectively); worms from Polynoidae and Terebellidae (*n* = 4 each, 20% and 22%, respectively) (Table 3; Figure 3c).

**TABLE 3:** List of samples positive to all five *P. lividus* loci.

N. samples	*COI1* identification	Class	Family
2	*Synalpheus gambarelloides*	Malacostraca	Alpheidae
1	*Ampithoe rubricata*	Malacostraca	Ampithoidae
1	*Ampithoe ramondi*	Malacostraca	Ampithoidae
1	*Pisa tetraodon*	Malacostraca	Epialtidae
3	*Galathea intermedia*	Malacostraca	Galatheidae
1	*Failed* ^a^	Malacostraca	Idoteidae
2	*Pilumnus villosissimus*	Malacostraca	Pilumnidae
3	*Amphipholis squamata*	Ophiuroidea	Amphiuridae
7	*Ophiothrix fragilis*	Ophiuroidea	Ophiotrichidae
1	*Harmothoe impar*	Polychaeta	Polynoidae
2	*Harmothoe spinifera*	Polychaeta	Polynoidae
1	*Lepidonotus clava*	Polychaeta	Polynoidae
4	*Thelepus cincinnatus*	Polychaeta	Terebellidae
2	*Phascolosoma granulatum*	Sipuncula	Phascolosomatidae

^a^ For this sample, Class and Family assignments were based on visual identification.

If we do not consider the two loci which underperformed, Pliv‐CYTb‐C and Pliv‐COI‐B, the number of samples positive in all three remaining loci was 60 (17%), distributed among Malacostraca (*n* = 21, 16% of all samples from the same Class), Ophiuroidea (*n* = 22, 21%), Polychaeta (*n* = 15, 18%) and Sipuncula (*n* = 2, 17%) (Table 4). The represented Families were similar to the distribution with five loci, but with more samples, especially among the shrimps Alpheidae and Ampithoidae, which doubled their representation, the brittle stars Ophiotrichidae and Amphiuridae and the worms Terebellidae, which increased even more (*n* = 15, 7 and 9, respectively, corresponding to 23%, 21% and 50% within each Family). Few positive samples were also present among the crabs Diogenidae, Inachidae and Majoidea and the worms Eunicidae and Nereididae (Table 4; Figure 3c). Once again, differences in the rates of positivity were statistically significant among Families (Tables S1–S6).

**TABLE 4:** Additional samples that scored positive for the three best performing primer pairs Pliv‐COI‐C, Pliv‐CYTb‐A and Pliv‐16S.

N. samples	*COI1* identification	Class	Family
2	*Synalpheus gambarelloides*	Malacostraca	Alpheidae
2	*Ampithoe rubricata*	Malacostraca	Ampithoidae
3	*Calcinus tubularis*	Malacostraca	Diogenidae
1	*Inachus aguiarii*	Malacostraca	Inachidae
1	*Herbstia condyliata*	Malacostraca	Majoidea
1	*Pilumnus hirtellus*	Malacostraca	Pilumnidae
4	*Amphipholis squamata*	Ophiuroidea	Amphiuridae
8	*Ophiothrix fragilis*	Ophiuroidea	Ophiotrichidae
1	*Palola valida*	Polychaeta	Eunicidae
1	*Platynereis sp*	Polychaeta	Nereididae
5	*Thelepus cincinnatus*	Polychaeta	Terebellidae

### 3.6 Next Generation Sequencing of COI1 Assemblages

As a further, independent test for the presence of urchin DNA within the invertebrate bodies, we performed a full Next Generation Sequencing (NGS) of the *COI1* locus amplified with Leray's universal primers for a subset of invertebrate samples, namely three brittle stars (*Ophiothrix* sp.), three shrimps ( *Synalpheus gambarelloides* ), and two crabs ( *Galathea intermedia* ) (Table 5, Tables S1–S6). Though costly, NGS allows us to infer the presence of all species present in a sample, with their relative abundances (Clare 2014; Pompanon et al. 2012).

**TABLE 5:** NGS *COI1* results for a subset of samples.

Sample code	Organism	Positivity to *P. lividus* primers	Host reads (%)	*P. lividus* reads (%)	Minimum *P. lividus* identity (%)
408	*Ophiothrix* sp.	Yes	96.03	0.24	98.81
409	*Ophiothrix* sp.	Yes	70.81	0.02	99.68
410	*Ophiothrix* sp.	No	95.59	0	
274	*S. gambarelloides*	Yes	80.85	0.5	99.68
264	*S. gambarelloides*	Yes	96.07	0.07	97.75
269	*S. gambarelloides*	No	52.66	0	
253	*G. intermedia*	Yes	92.59	0.02	94.6
252	*G. intermedia*	No	95.6	0	

As expected, the majority of reads (53%–96%) within each sample belonged to the host organism. Yet, sequencing of *COI1* amplicons for all the specimens which had showed a strong band with all five *P. lividus* primers, also revealed the presence of *P. lividus* reads in their full COI1 assemblages (0.02%–0.5%), with a high identity score (> 94%). Conversely, the brittle star, shrimp and crab samples which had resulted negative with *P. lividus* primers, did not present any urchin COI1 read.

## 4 Discussion

The dynamics between algal forests and barren grounds in Mediterranean rocky reefs relies on the regulation of the populations of the sea urchins *P. lividus* and *A. lixula* . *P. lividus* exerts significant ecological influence by overgrazing on macroalgae, driving the transformation of productive macrolagal forests into structurally impoverished barrens which are then maintained by both sea urchin species (Sala et al. 1998; Guidetti and Sala 2007; Bonaviri et al. 2011). Understanding the mechanisms controlling urchin populations is therefore critical for predicting ecosystem stability and resilience. Predation is a key factor in this regulation, yet estimates of predation pressure have historically focused on fish and large invertebrates, overlooking the potential role of smaller benthic invertebrates. Our study sheds light on the contribution of these micropredators to urchin populations.

### 4.1 Molecular Markers Untangle Trophic Interactions

Estimates of predation pressure on sea urchins suggest that small juveniles may efficiently hide in crevices, avoiding predation by fishes and large invertebrates such as lobsters (Fagerli et al. 2014; Ling and Johnson 2012; Sala and Zabala 1996; Shears and Babcock 2002; Tegner and Dayton 1981). Yet, in Mediterranean algal forests, more than 75% of *P. lividus* juveniles disappear within 6 months of settlement, suggesting intense predation pressure (Sala and Zabala 1996). This contrasts with barrens, where juvenile survival seems to be significantly higher for both *P. lividus* and *A. lixula* , possibly due to lower predator abundance. A possible explanation is that small, crawling invertebrates might efficiently extract urchin juveniles from crevices, exerting the most prominent control of urchin populations (Bonaviri et al. 2012; Clemente et al. 2013; McNaught Douglas Colin 1999; Pavlova 2009; Scheibling and Hamm 1991; Scheibling and Robinson 2008; Smith et al. 2023; Steneck et al. 2013).

By addressing this hypothesis with a molecular strategy, we were able to overcome the technical difficulties of detecting predation events at such a small scale. In this study, we show that juvenile urchins may face intense predation pressure from small invertebrates in algal forests, where predator diversity is highest. In the Mediterranean Sea, macroalgal forests host an abundant and diversified fauna, with a high number of trophic guilds (Antoniadou and Chintiroglou 2006; Pinna et al. 2020). Accordingly, in our survey we collected 360 invertebrates in algal forests, belonged to five major taxonomic groups: Malacostraca, Ophiuroidea, Polychaeta, Gastropoda and Sipuncula. The presence of these organisms suggests a diverse predator community capable of exerting significant top‐down control on early‐stage urchin populations.

Our findings highlight specific predator groups with significant roles in controlling urchin settler populations. Crustaceans from the Alpheidae, Pilumnidae and Galatheidae families, abundant in algal forests compared with barren grounds (Pinna et al. 2020), had high predation rates (24%, 33% and 43% positive samples, respectively), corroborating previous aquarium trials that identified *Alpheus dentipes* and *Pilumnus* species as urchin predators (Bonaviri et al. 2012). Moreover, field surveys in the Atlantic observed a negative correlation between the abundance of sea urchin settlers and that of *Alpheus macrocheles* , and other decapods (Cano et al. 2024; Williams et al. 2011). Crabs from the Xanthidae Family were major predators in aquaria tests (Bonaviri et al. 2012), but unfortunately, we only found two specimens in our survey, which resulted negative. Conflicting evidence in aquaria tests indicates hermit crabs (Paguridae) as potential predators of sea urchin juveniles, with some studies showing predation and others not (Bonaviri et al. 2012; Clemente et al. 2013; Fagerli et al. 2014; Scheibling and Robinson 2008). In our survey, despite the Paguridae Family being largely represented, with 33 samples, none were positive. Most likely then, hermit crabs are not effective predators of urchin settlers and the predation events measured in aquaria represent artificial effects due to limited food choice and captivity conditions. In agreement with this hypothesis, *Pagurus bernardus* mostly fed on settlers of the urchin *Strongylocentrus droebachiensis* in aquaria only when other food sources, such as clams, were not present (Fagerli et al. 2014). Moreover, in situ video recording showed that hermit crabs, though abundant, did not feed on tethered urchins along the Norwegian coast (Fagerli et al. 2014). It remains to be tested whether crabs from the Galatheidae, Idoteidae and Majoidea families, among the most common positive samples in our survey (43%, 100% and 100%, respectively), are effective predators in aquaria trials.

Whelks, carnivorous Gastropods, were also all negative. This finding is in agreement with other studies, that showed that the whelk *Buccinum undatum* interacted, without feeding, with tethered urchins (Fagerli et al. 2014) nor did it feed on urchin juveniles in captivity trials (Scheibling and Hamm 1991; Scheibling and Robinson 2008). However, our survey only retrieved a limited number of molluscs, and mostly (11 out of 13) located in Sicily. Although suggesting that whelks are not primary predators of *P. lividus* juveniles, we cannot exclude that this observation derives from the few specimens analysed.

Several positive invertebrates were found among two families of Ophiuridae collected in our survey: Amphiuridae and Ophiotricidae (21% and 23%, respectively). Surprisingly, brittle stars, which have been found to be abundant in Mediterranean algal forests (Pinna et al. 2020), have been overlooked so far as potential predators of young sea urchins, although our findings suggest they might represent a major component of this community. These findings highlight a previously underappreciated component of sea urchin predation networks, warranting further investigation.

Polychaetes also showed varied predation potential, with Polynoidae (20%), Eunicidae (8%) and Nereididae (3%) testing positive for *P. lividus* DNA. In aquaria tests, *Nereis* sp. did not feed on *S. droebachiensis* settlers (Fagerli et al. 2014; Scheibling and Robinson 2008), although the tested size of the urchin was larger than the size of *P. lividus* settlers we commonly found in our surveys, which might act as a size exclusion threshold for this Class of predators.

It is very important to stress that direct predation is not the only event leading to the presence of urchin DNA in samples. Contamination is an obvious reason, though a careful handling procedure, use of separate machinery for samples and standard *P. lividus* DNA, and inclusion of several negative controls in each PCR run, let us believe that we prevented contamination from occurring in our analyses. Accordingly, the largest share of samples (43%) did not amplify at any locus. Urchin DNA can be present within an invertebrate body as a result of secondary predation, i.e., when a predator eats another predator that had fed on *P. lividus* . Another level of trophic interaction that cannot be excluded by molecular analyses is scavenging on dead urchin material, rather than direct predation on settlers. Finally, urchin DNA can be present in the environment through their faeces or disintegration of their remains. This aspect is particularly concerning, since direct testing of unconsolidated sediments in Tasmanian reefs found urchin DNA in 20%–100% of samples (Redd et al. 2014), and sediments are known to be repositories for eDNA (Bowman and McCuaig 2003). However, only a minority of lobsters fed with those sediments or directly with urchin faeces scored positive, probably because digestion eliminated the already degraded urchin DNA present in the sediment and urchin faeces, suggesting that environmental DNA might represent a minor issue (Redd et al. 2014). Accordingly, a large portion of our samples was negative. Yet, it is possible that the presence of *P. lividus* DNA in some of our positive samples derives from sediment or particulate feeding. This is especially likely for the Terebellidae and Sipuncula specimens, known to feed by sediment filtration and accumulate organic material. All the other positive invertebrates are carnivorous, and events of direct predation on *P. lividus* settlers are the most likely scenario.

### 4.2 Strengths and Limits of Molecular Taxonomy

Species assignment requires a thorough level of taxonomic expertise, with scholars usually specialising in one or a few Classes, and/or limited geographical regions. Moreover, taxonomic knowledge is a field suffering a severe decline in practitioners (Drew 2011). Visual identification of a wide collection of invertebrates is, therefore, a daunting task requiring the involvement of multiple, uncommon experts. When samples are preserved for a long time or are not carefully handled, they may also lose structural details (such as colour and appendages) that make taxonomic identification more challenging. Accordingly, several of our samples presented fragmented bodies (Figures S1–S7), due to the processing of samples and their transport from the field to the dissecting and molecular laboratories, located in different countries.

Over the years, *cytochrome c oxidase 1* (*COI1*) has become the standard locus for barcoding of Metazoans, with thousands of annotated sequences deposited in public databases, such as BOLD (Barcode of Life data system). For three samples, *COI1* sequencing resulted in a *Photobacterium* spp. assignment rather than the related crabs. *Photobacterium* species are marine microorganisms, some known as pathogens for fishes and crabs (Rivas et al. 2013), but they can also constitute the major component of the crab microbiome (Jiang et al. 2023). It is therefore possible that these three specimens were infected by the bacterium, whose DNA abundance predominantly outcompeted the amplification reactions.

Overall, barcoding strongly correlated with visual assignment. Minor disagreement at the Family level (5%) was most likely due to visual misidentification. Though generally considered a potent aid in taxonomic identification, it must be noted that molecular barcoding may suffer from inherent limitations. First, it relies on sequence comparison of a single locus of mtDNA, characterised by a high mutation rate and no recombination. As a result, geographically or sexually separated populations of the same species may diverge in sequence (intraspecific polymorphism), leading to an overestimation of the number of effective species (Eberle et al. 2020). Conversely, for some taxa *COI1* sequence is not variable enough to discriminate between morphologically different species (Carew and Hoffmann 2015). Finally, a bias in the sequence deposition into repositories leads to overrepresentation of species from certain areas of the world. In our survey, most of the invertebrates (70%) were assigned to species with high confidence (> 95% identity). For the specimen with a lower identity score, it is safer to restrict the identification to higher taxonomic levels, such as Genus or even Family.

### 4.3 Development of Specific Markers for Two Key Urchin Species

In the Mediterranean Sea, shallow rocky reefs are dominated by two sympatric species, *P. lividus* and *A. lixula* . At the settler stage, these urchins are indistinguishable. Sequencing of the *COI1* locus for a subset of the collected settlers revealed that they all belonged to *P. lividus* . Yet, we designed and tested specific primers for both sea urchin species, in order to provide tools for molecular trophic studies in future spawning events in the Mediterranean and Atlantic.

Due to PCR sensitivity, caution is required when performing and interpreting molecular analyses, with multiple tests and controls needed (reviewed by King et al. 2008). The specific primers for *P. lividus* and *A. lixula* were designed by aligning hundreds of accessions of both urchin species from across the Mediterranean and Atlantic. Since these primers amplified DNA from urchins collected both in Catalonia and Sicily, located in distant areas of the Mediterranean, they proved not to be affected by intraspecific genetic variability. Primers were also tested for specificity, both in silico and by PCR, including invertebrates encompassing the taxa collected in our surveys. We then selected five primer pairs specific for *P. lividus* and three primer pairs specific for *A. lixula* . Sensitivity tests showed that these primers were able to detect as little as 1–10 pg urchin total DNA. With a nuclear genome of 927.4 Mb and 2000–20,000 copies of 15.697 Kb mtDNA per cell, 1 pg total DNA corresponds to about 1 single cell, or 1400–20,000 mtDNA copies (Cantatore et al. 1989; Marlé Taz et al. 2023). However, estimates in samples with degraded DNA may be skewed. Primer efficiency was also not affected by a thousand‐fold excess of exogenous DNA, from invertebrates belonging to different Classes, mimicking the scenario occurring in micropredators feeding on urchin settlers. Having passed all the control tests, the selected primer pairs can be confidently used as a molecular tool to study trophic interactions involving the sea urchins *P. lividus* and *A. lixula* .

Quantitative assessment of dietary intake in predator–prey interactions is a challenging task. Molecular techniques have been used to quantify the amount of prey consumed in captivity trials (Deagle and Tollit 2007), but evaluation from field samples is not straightforward (Nejstgaard et al. 2008; Redd et al. 2014; Troedsson et al. 2007; Weber and Lundgren 2009). The amount of prey DNA is dependent on the time of ingestion and, in the case of different predator species, on their rate of digestion. Therefore, it is usually not possible to discern between low levels of recent ingestion and high levels of past ingestion, when most of the DNA has been degraded. For this reason, it is safer to adopt a qualitative approach with a binary outcome, positive or negative, regarding the presence of prey DNA. It must be stressed, however, that qualitative assessments are also subject to a certain degree of subjectivity in the choice of the threshold separating positive from negative samples. Threshold values can be based on the band intensity, as in this and other studies (Smith et al. 2023), or on Ct values in the case of qPCR analyses (Redd et al. 2014). In the present work, we relied on the automatic detection of bands, over the gel background, by specific software. To be more conservative in the result interpretation, the threshold was set high enough to discard the ambiguous bands, visible but of faint intensity. Though this is a common approach, it still retains an arbitrary component that needs to be taken into account. Additionally, the choice of small amplicon sizes, instrumental in detecting degraded prey's DNA in gut content, presents the risk of spurious bands resulting from primer dimerization, which may affect interpretation. Longer electrophoresis separation and careful size interpolation may facilitate the discrimination between genuine and artifactual bands. In our analyses, the largest share of samples (43%) was negative, showing no visible bands. Therefore, the issue of dimer bands probably does not apply to our amplification conditions, as it would affect samples regardless of the presence of template.

As an additional measure of confidence in result interpretation, we decided to analyse multiple loci. For *COI1* and *CYTb*, we also used two primer pairs spanning different regions of the loci. The number of positive bands was correlated with the amplicon size. Pliv‐COI1‐C, Pliv‐CYTb‐A and Pliv‐16S showed the highest number of positive samples, and their amplicon sizes were 178, 155 and 161 bp, respectively. The amplicons Pliv‐COI‐B and Pliv‐CYTb‐C, in contrast, were present in fewer samples, and their sizes were smaller (93 and 119 bp, respectively). It is possible then that the higher stringency of Pliv‐COI‐B and Pliv‐CYTb‐C derives from the more difficult discrimination of amplicon bands from primers. The assignment of the amplified bands of all three loci to *P. lividus* DNA was further confirmed by sequencing.

### 4.4 Concluding Remarks

This study makes several key contributions to understanding the dynamics of sea urchin populations in the Mediterranean and the role of micropredators in shaping these populations. First, it shows the potential of the molecular tools developed in this study in providing new opportunities to investigate predator–prey interactions in marine food webs, contributing to a more comprehensive understanding of the factors governing urchin population dynamics.

Moreover, the findings emphasise the potential role of smaller invertebrates in controlling the population density of urchins. Our survey revealed that, in addition to well‐established predators of adult urchins such as large crustaceans and fishes, a variety of less‐observed macroinvertebrate species across multiple taxonomic groups may also play a crucial role in regulating sea urchin populations. The ability of these micropredators to target urchin settlers suggests they may be a primary factor in preventing population outbreaks and maintaining ecosystem balance, particularly in algal forests. Future work should explore the functional roles of brittle stars and other overlooked invertebrates in regulating sea urchin populations.

Additionally, the study highlights the importance of refining molecular techniques, particularly to distinguish between active predation and passive DNA uptake from environmental sources. The use of molecular markers, although powerful, must be carefully interpreted, especially when considering secondary predation, scavenging or the influence of environmental DNA.

## Author Contributions

R.D.M. and C.B. conceived the idea and analysed the data. R.D.M. designed and coordinated the research, developed the primers and wrote the manuscript. F.D.T. and B.H. collected the samples. F.D.T., V.M. and O.M. performed the morphological identification. A.S. and F.L.B. performed the DNA extraction and amplification. R.D.M., F.D.T. and V.M. acquired the funding. All authors read and approved the final version of the manuscript.

## Conflicts of Interest

The authors declare no conflicts of interest.

## Supporting information


**Figure S1–S7:** mec70163‐sup‐0003‐FigureS1–S7.zip.

**Table S1–S6:** mec70163‐sup‐0004‐TableS1–S6.zip.

## Data Availability

The genetic data supporting this study can be found in Tables S1, S4 and S6 (*COI1* sequences of macropredators and urchins, respectively), and at NCBI‐SRA with the reference number PRJNA1336498 (NGS sequences).
