# Supplementary material for: Unravelling Hidden Trophic Interactions Among Sea Urchin Juveniles and Macroinvertebrates by DNA Amplification
Source: Mol Ecol. 2025 Nov 13;34(24):e70163. doi: 10.1111/mec.70163 (PMC12717973; doi:10.1111/mec.70163)

Supplemental Information for:

Unravelling hidden trophic interactions among sea urchin juveniles and macroinvertebrates by DNA amplification

Alberto Sutera, Chiara Bonaviri, Francesco Di Trapani, Francesca La Bella, Vesna Macic, Olivera Marković, Bernat Hereu, Roberto De Michele

Fig. S4. Specificity test. Gels showing amplification of representative samples from different taxa, including positive controls P. lividus and A. lixula and negative controls (NC). Marker: 50 bp.

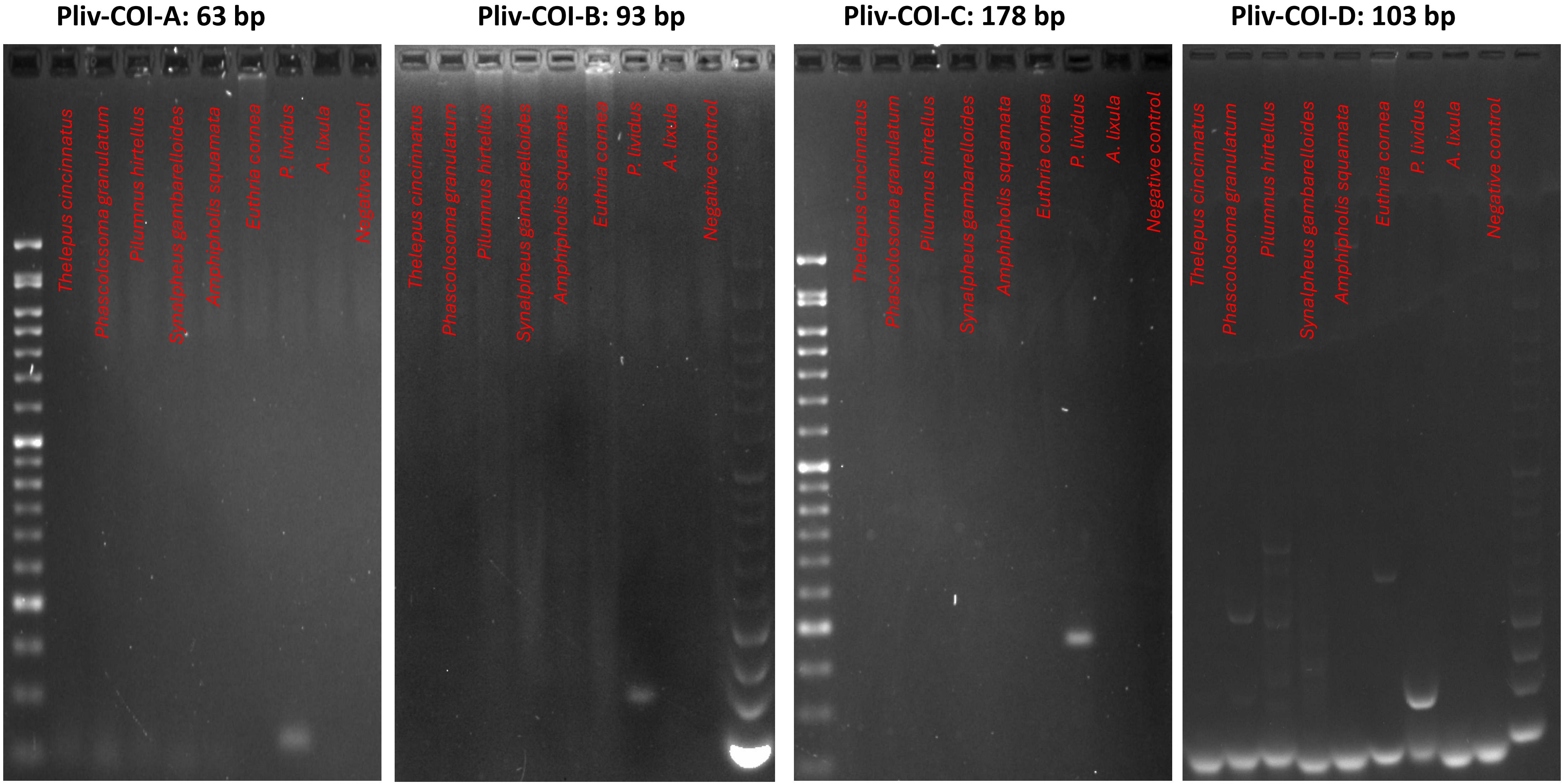

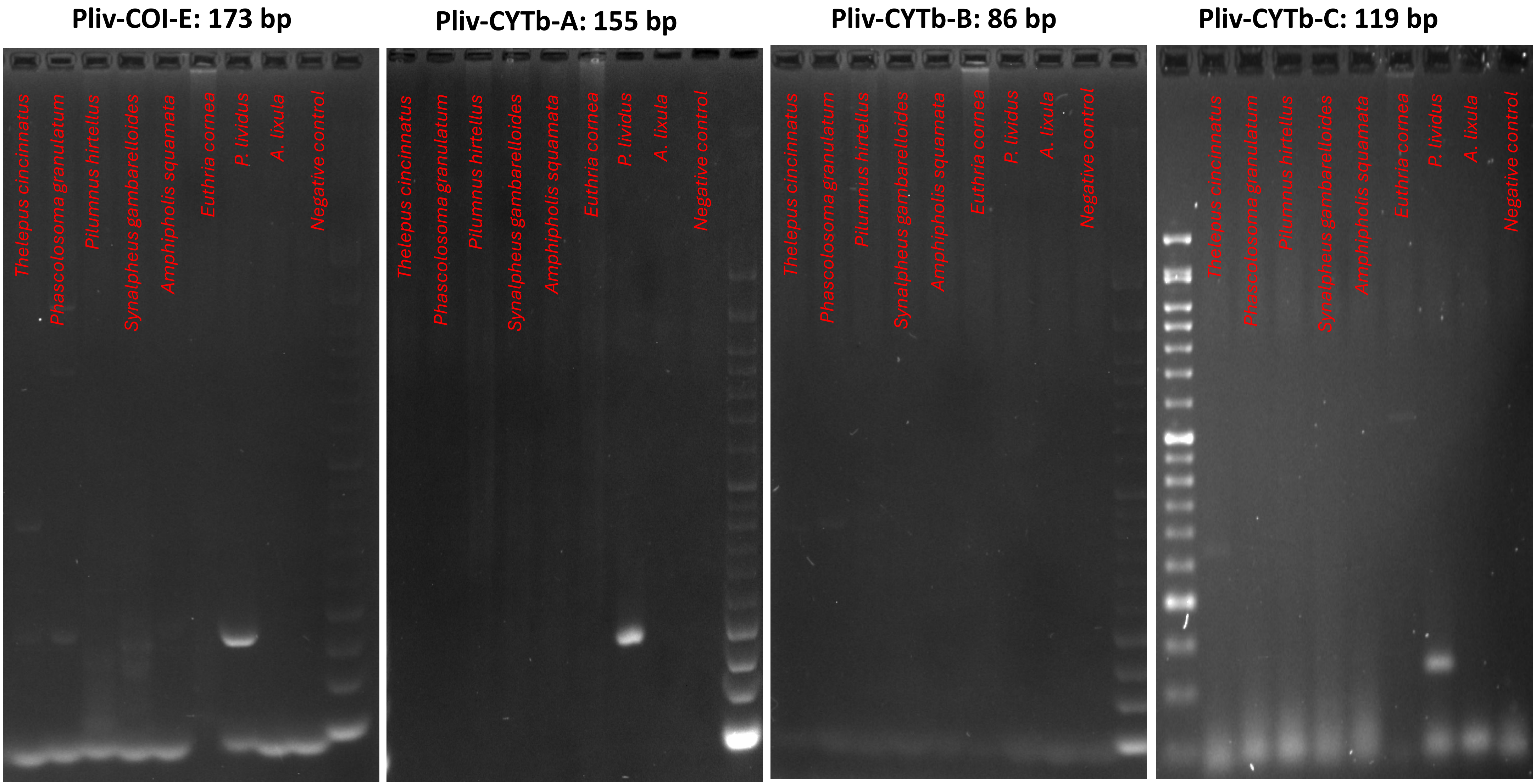
Pliv-COI-E: 173 bp
Pliv-CYTb-A: 155 bp
Pliv-CYTb-B: 86 bp
Pliv-CYTb-C: 119 bp
Thelepus cincinnatus
Phascolosoma granulatum
Pilumnus hirtellus
Synalpheus gambarelloides
Amphipholis squamata
Euthria cornea
P. lividus
A. lixula
Negative control

Pliv-16S: 161 bp

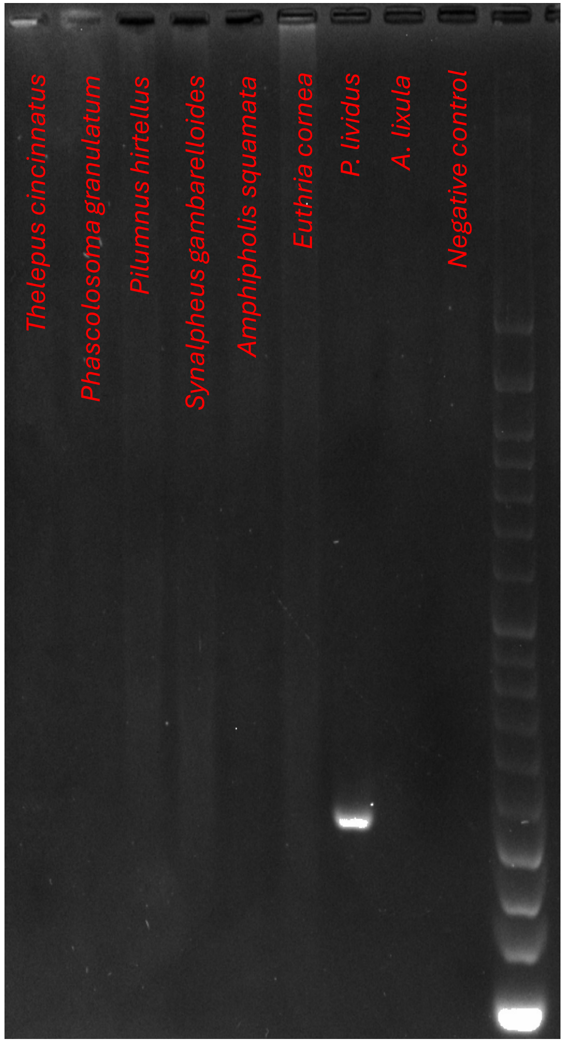

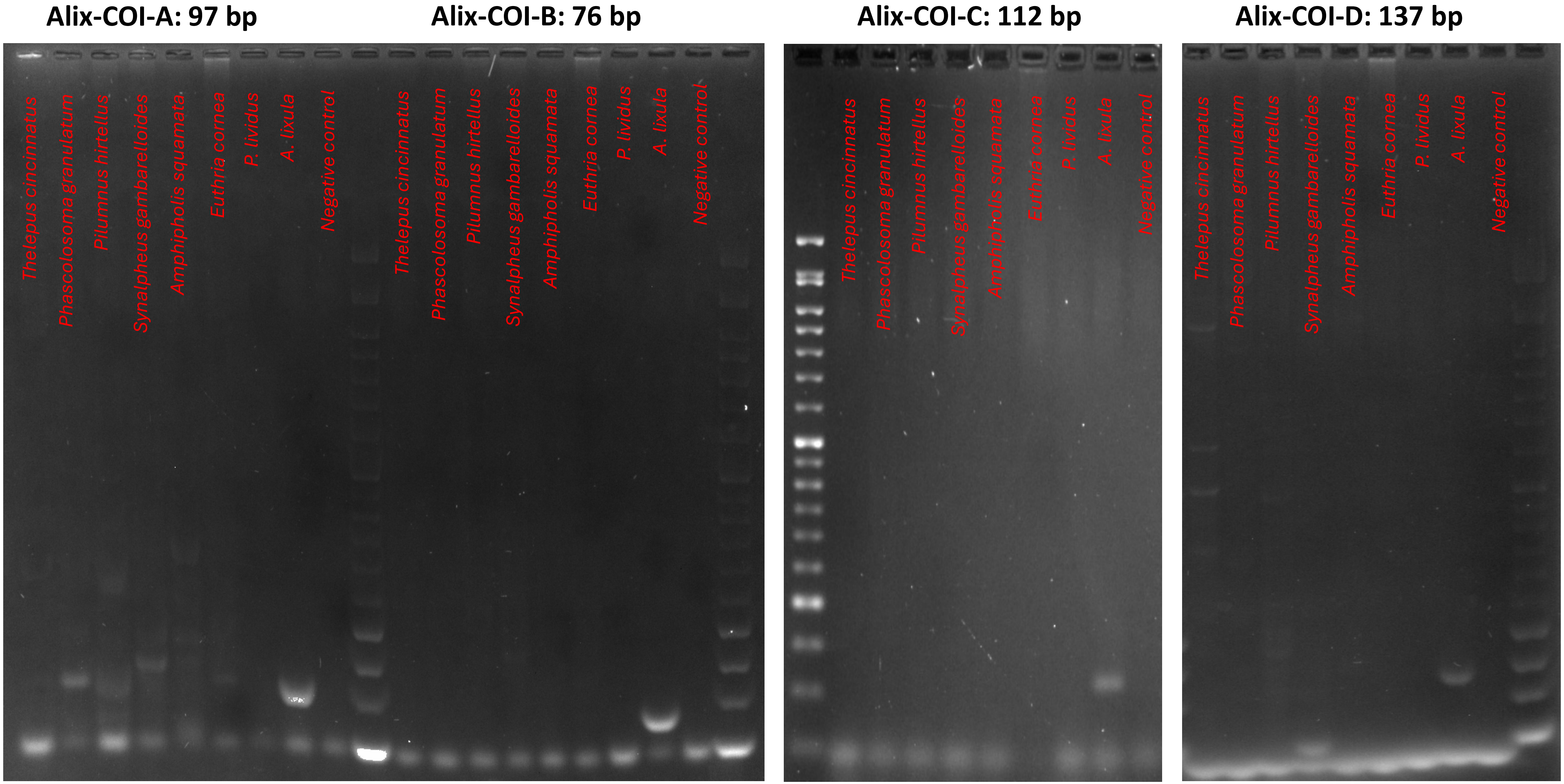
Alix-COI-A: 97 bp
Alix-COI-B: 76 bp
Alix-COI-C: 112 bp
Alix-COI-D: 137 bp
Thelepus cincinnatus
Phascolosoma granulatum
Pilumnus hirtellus
Synalpheus gambarelloides
Amphipholis squamata
Euthria cornea
P. lividus
A. lixula
Negative control

Alix-COI-E: 89 bp **Alix-16S-A: 153 bp** **Alix-16S-B: 110 bp**

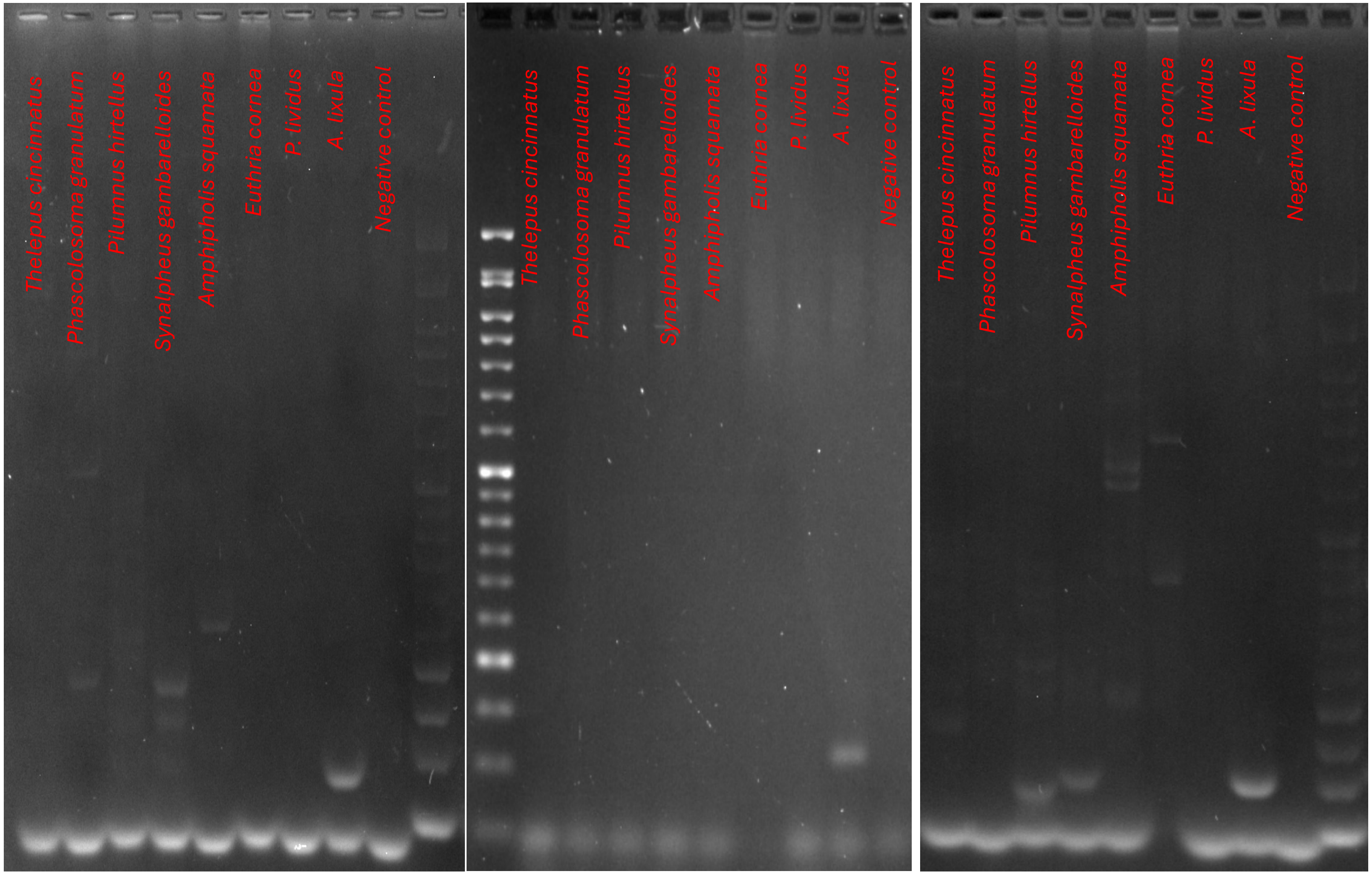

Supplement: Supplementary file 1 — Figure S1–S7: mec70163‐sup‐0003‐FigureS1–S7.zip. [file MEC-34-e70163-s002.zip › mec70163-sup-0001-FigureS1-S7/FigureS4.pdf]
