# Supplementary material for: Unravelling Hidden Trophic Interactions Among Sea Urchin Juveniles and Macroinvertebrates by DNA Amplification
Source: Mol Ecol. 2025 Nov 13;34(24):e70163. doi: 10.1111/mec.70163 (PMC12717973; doi:10.1111/mec.70163)
Supplement: Supplementary file 1 — Figure S1–S7: mec70163‐sup‐0003‐FigureS1–S7.zip. [file MEC-34-e70163-s002.zip › mec70163-sup-0001-FigureS1-S7/FigureS5.pdf]

Supplemental Information for:

Unravelling hidden trophic interactions among sea urchin juveniles and macroinvertebrates by DNA amplification
Alberto Sutera, Chiara Bonaviri, Francesco Di Trapani, Francesca La Bella, Vesna Macic, Olivera Marković, Bernat Hereu, Roberto De Michele

Fig. S5. Sensitivity test. Gels showing amplification of *P. lividus* and *A. lixula* DNA in a dilution concentration series. NC= negative controls. Marker: 50 bp.

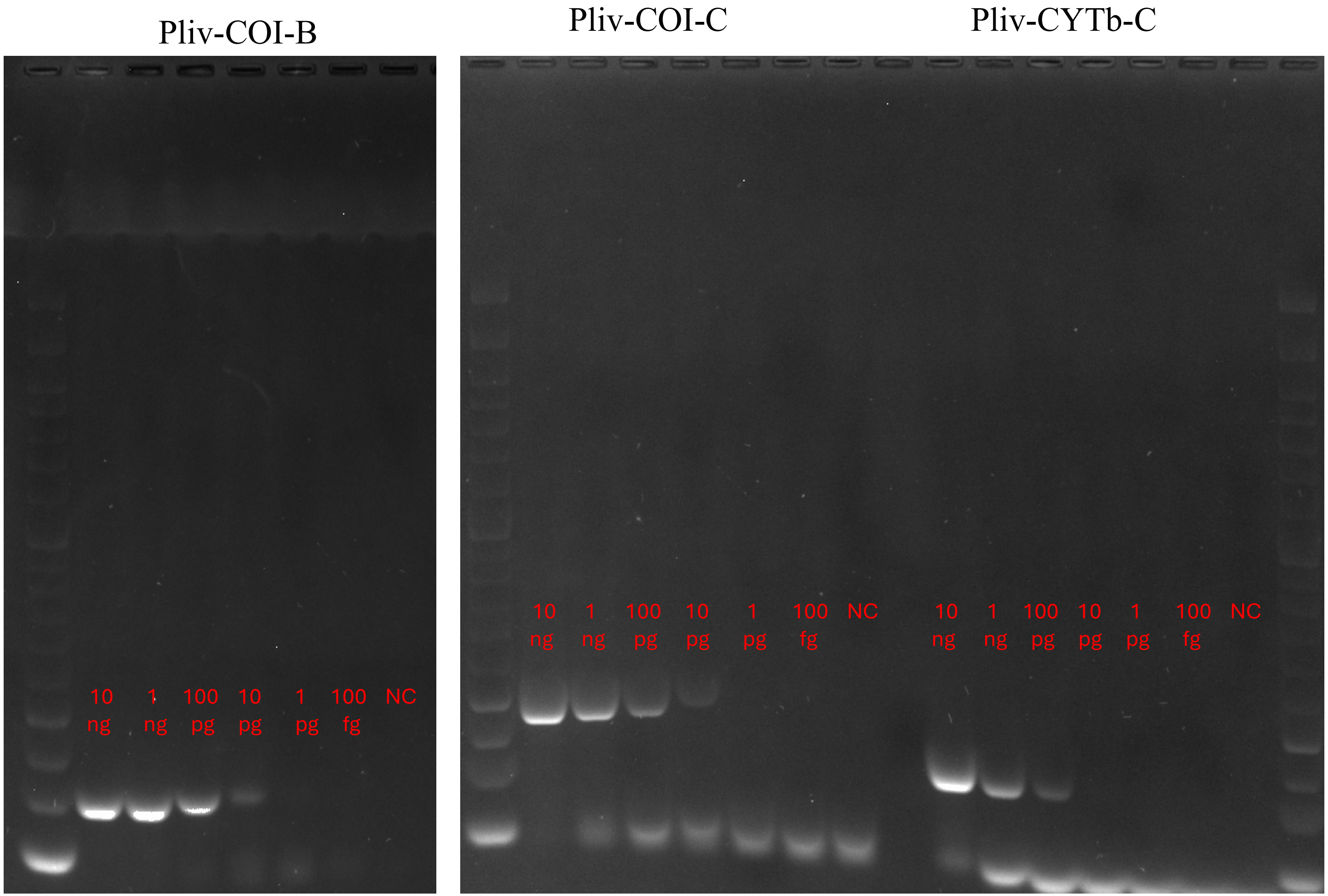

Pliv-16S Pliv-CYTb-A

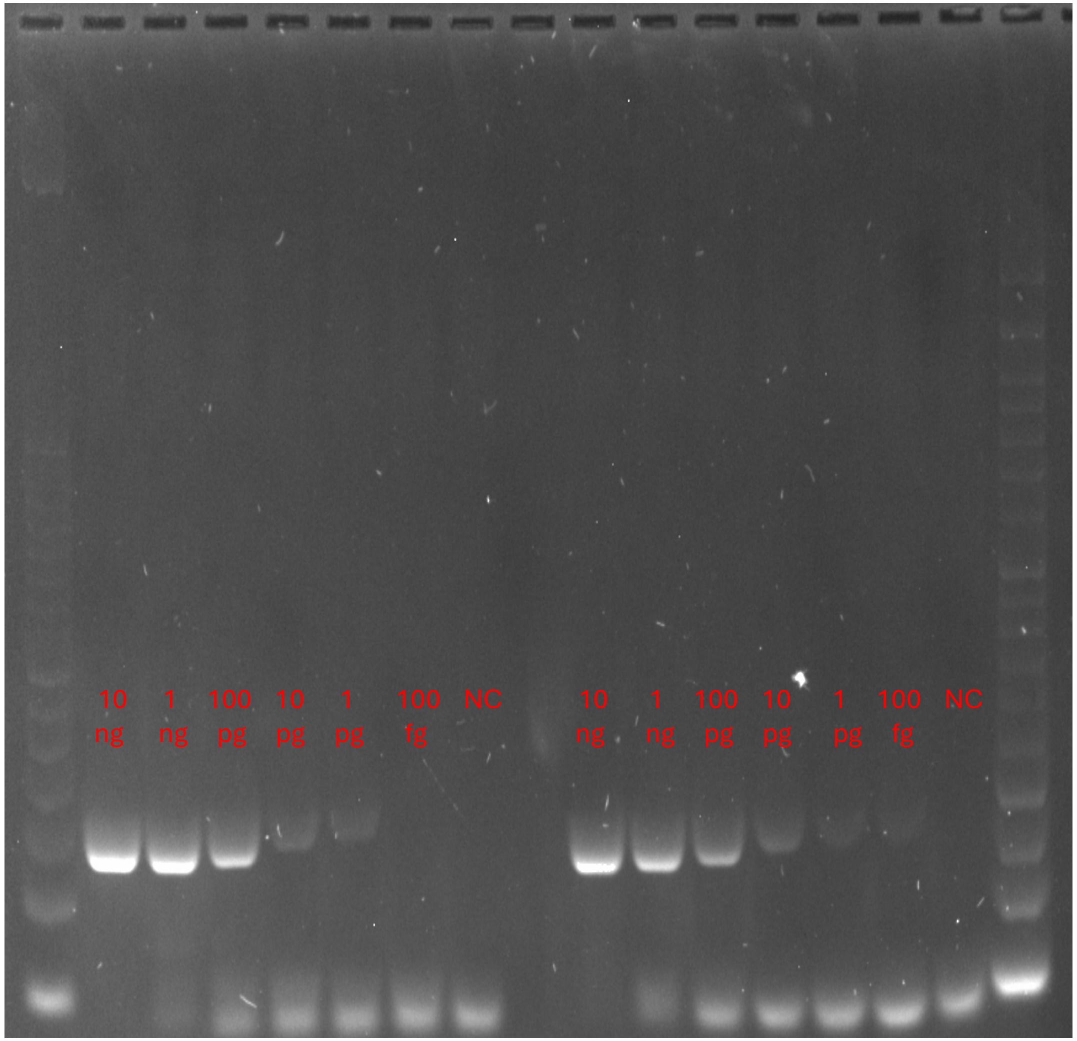

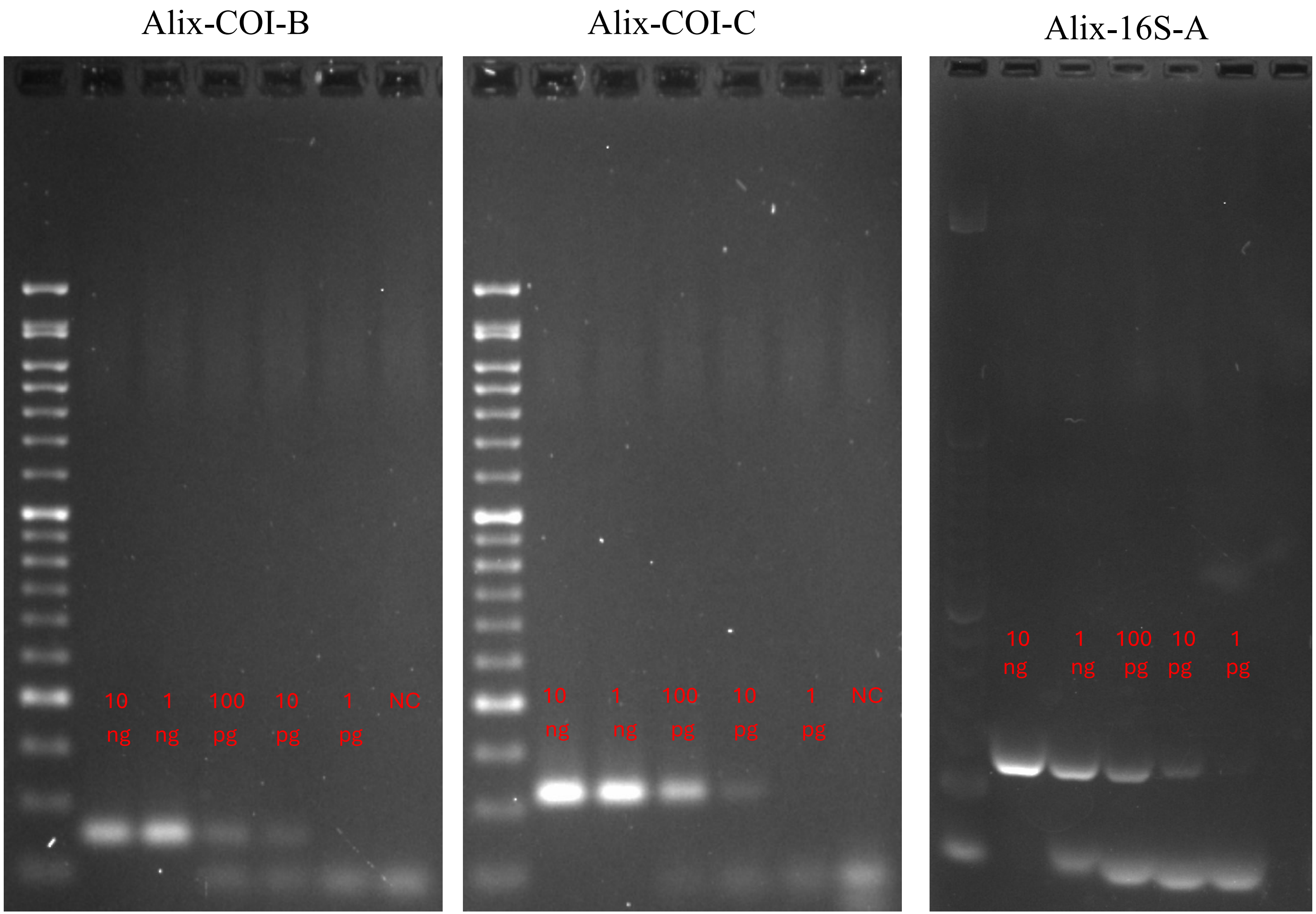
Alix-COI-B
10 ng
1 ng
100 pg
10 pg
1 pg
NC
Alix-COI-C
10 ng
1 ng
100 pg
10 pg
1 pg
NC
Alix-16S-A
10 ng
1 ng
100 pg
10 pg
1 pg
