# Supplementary material for: Unravelling Hidden Trophic Interactions Among Sea Urchin Juveniles and Macroinvertebrates by DNA Amplification
Source: Mol Ecol. 2025 Nov 13;34(24):e70163. doi: 10.1111/mec.70163 (PMC12717973; doi:10.1111/mec.70163)

Supplemental Information for:

Unravelling hidden trophic interactions among sea urchin juveniles and macroinvertebrates by DNA amplification

Alberto Sutera, Chiara Bonaviri, Francesco Di Trapani, Francesca La Bella, Vesna Macic, Olivera Marković, Bernat Hereu, Roberto De Michele

Fig. S6. Inhibition test. Gels showing amplification of P. lividus and A. lixula DNA when mixed with 1,000 thousand-fold excess of predator's DNA. NC= negative controls. Marker: 50 bp.

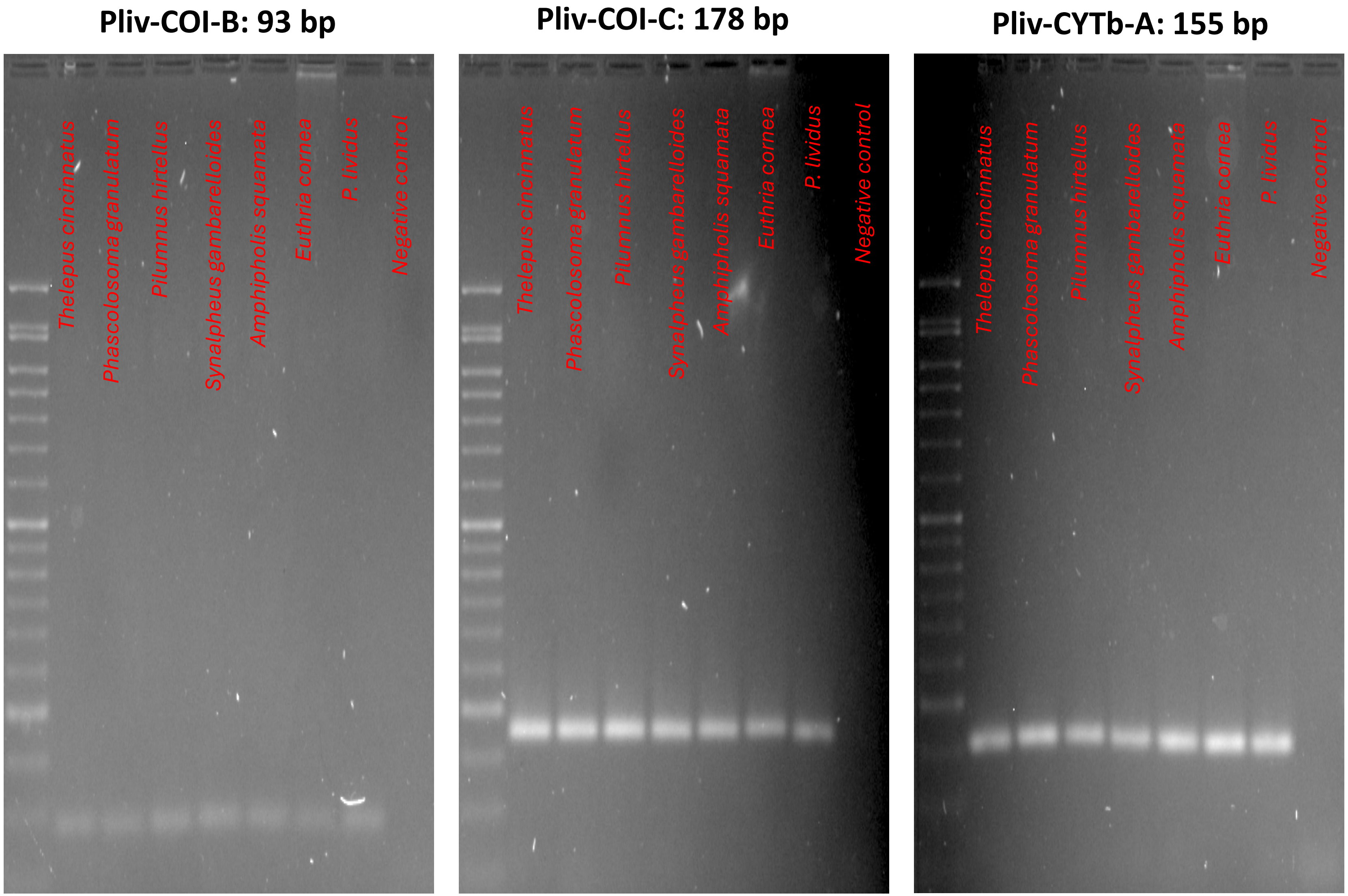

Pliv-CYTb-C: 119 bp

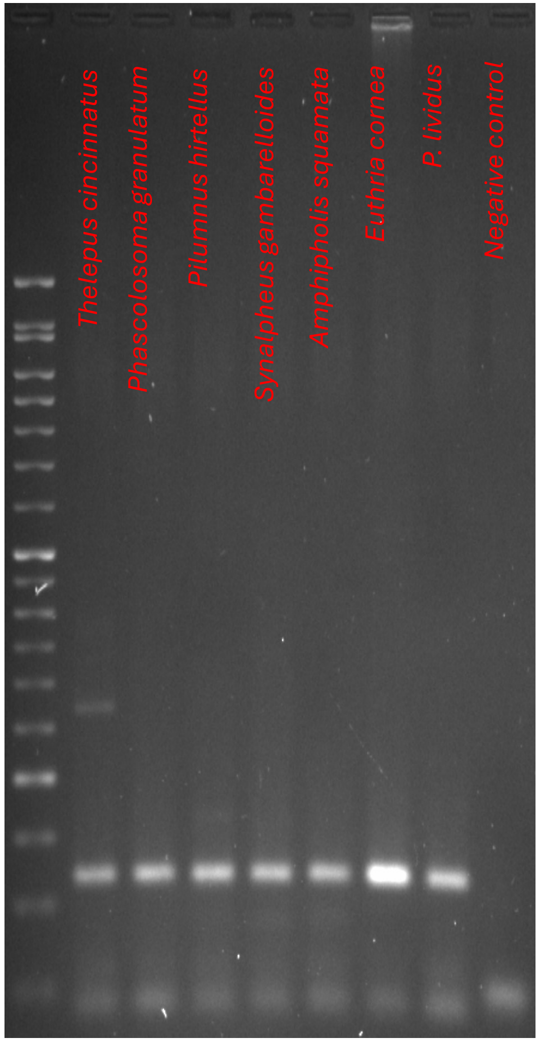

Pliv-16S: 161 bp

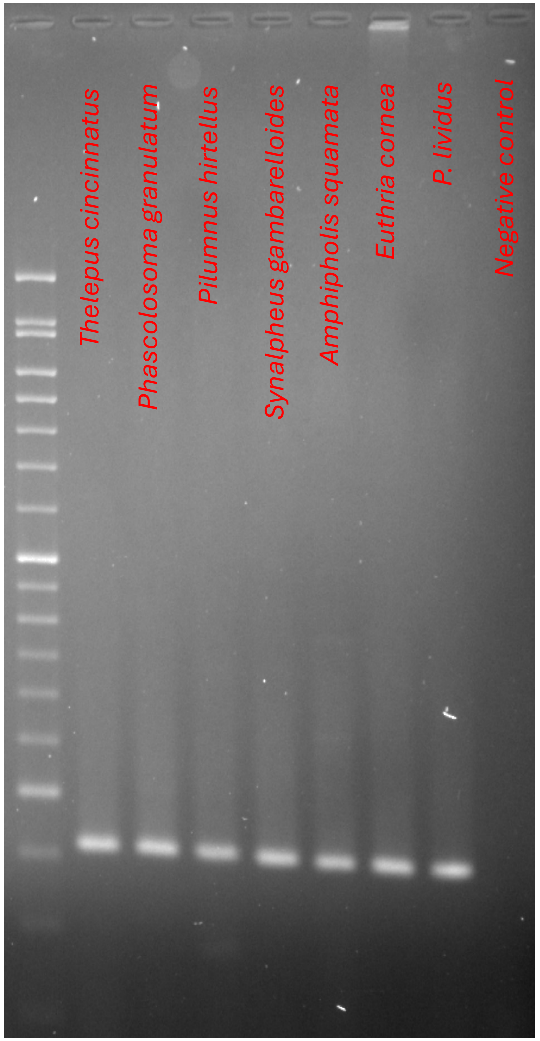

Alix-COI-B: 76 bp **Alix-COI-C: 112 bp** **Alix-16S-A: 153 bp**

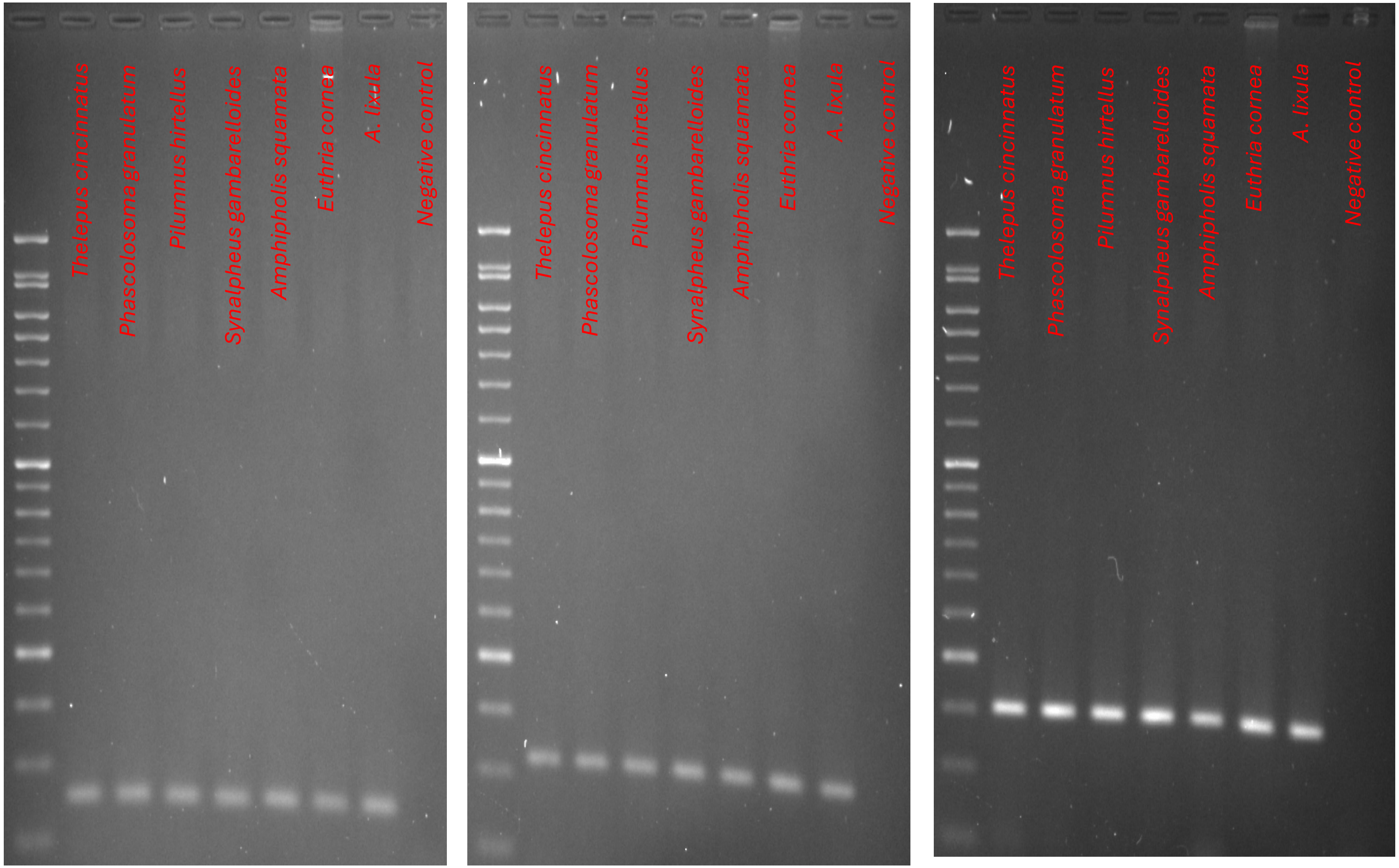

Supplement: Supplementary file 1 — Figure S1–S7: mec70163‐sup‐0003‐FigureS1–S7.zip. [file MEC-34-e70163-s002.zip › mec70163-sup-0001-FigureS1-S7/FigureS6.pdf]
