# Supplementary material for: Unravelling Hidden Trophic Interactions Among Sea Urchin Juveniles and Macroinvertebrates by DNA Amplification
Source: Mol Ecol. 2025 Nov 13;34(24):e70163. doi: 10.1111/mec.70163 (PMC12717973; doi:10.1111/mec.70163)
Supplement: Supplementary file 1 — Figure S1–S7: mec70163‐sup‐0003‐FigureS1–S7.zip. [file MEC-34-e70163-s002.zip › mec70163-sup-0001-FigureS1-S7/FigureS7.pdf]

Fig. S7. Gels showing amplification from urchin settlers' DNA with *P. lividus* and *A. lixula* specific primers. NC= negative controls. Marker: 50 bp.

Pliv-16S

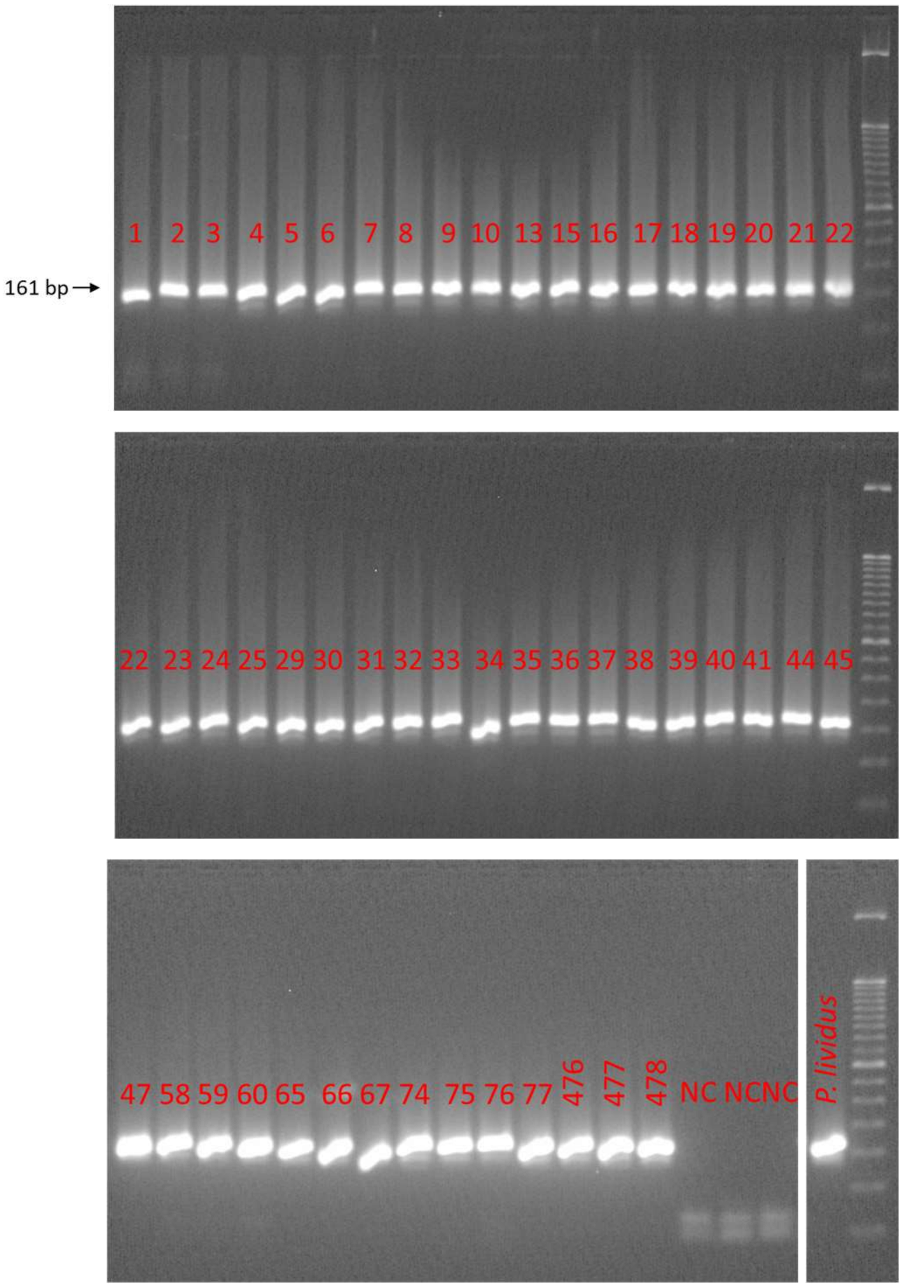

Pliv-CYTb-A

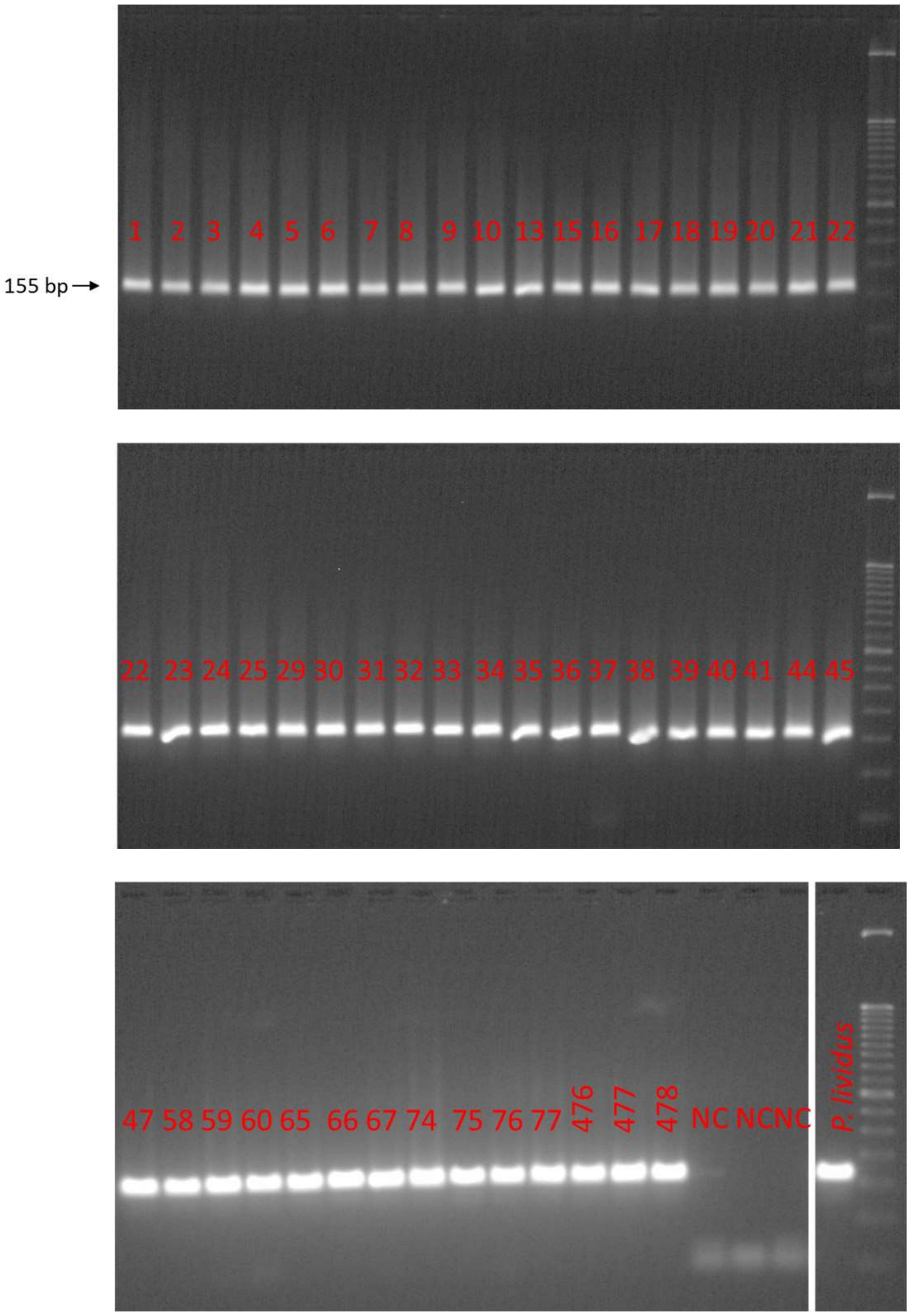
155 bp
1 2 3 4 5 6 7 8 9 10 13 15 16 17 18 19 20 21 22
22 23 24 25 29 30 31 32 33 34 35 36 37 38 39 40 41 44 45
47 58 59 60 65 66 67 74 75 76 77 476 477 478 NC NC NC
P. lividus

Pliv-COI-B

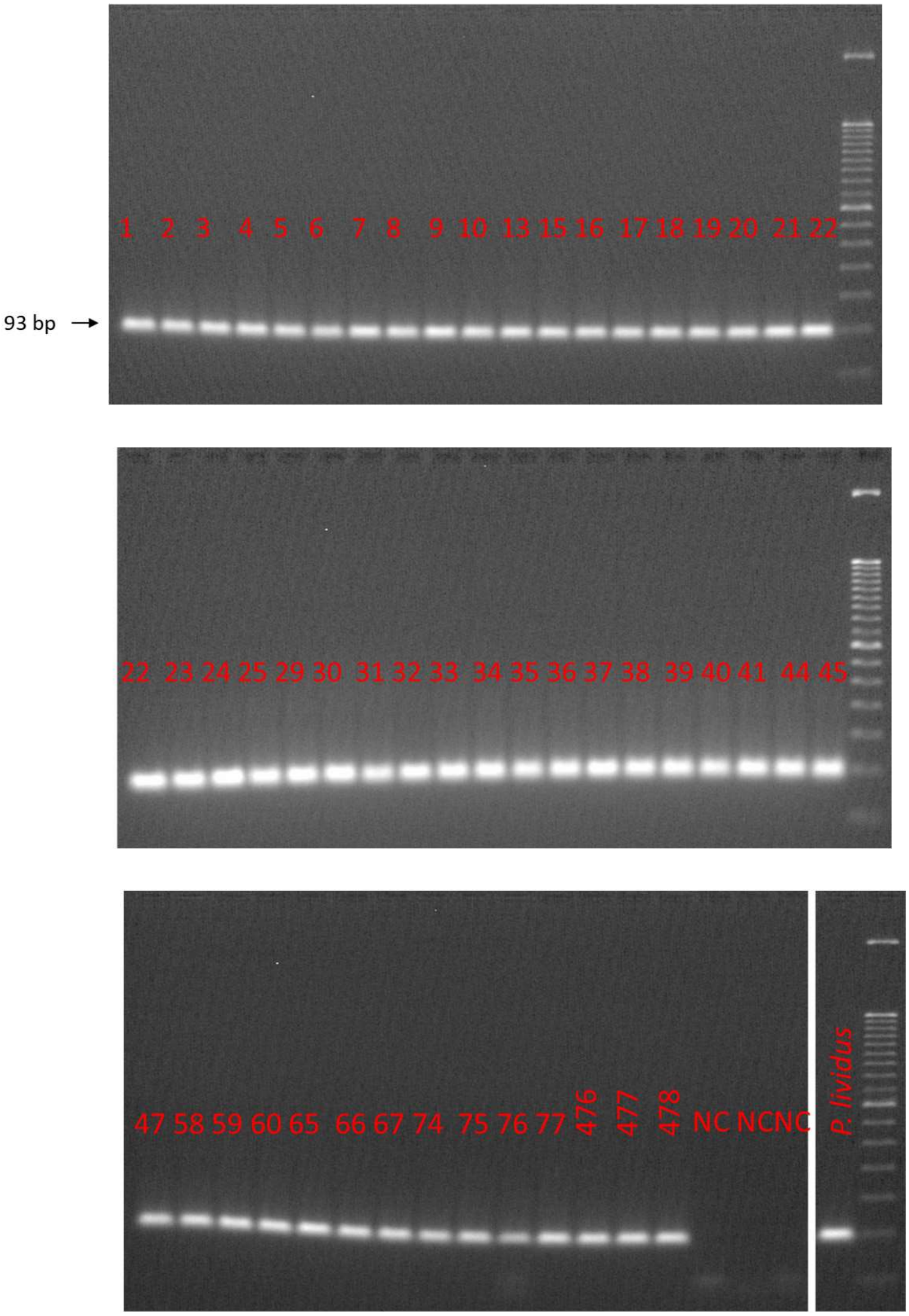
93 bp
1 2 3 4 5 6 7 8 9 10 13 15 16 17 18 19 20 21 22
22 23 24 25 29 30 31 32 33 34 35 36 37 38 39 40 41 44 45
47 58 59 60 65 66 67 74 75 76 77 476 477 478 NC NC NC
P. lividus

Al-16S- A

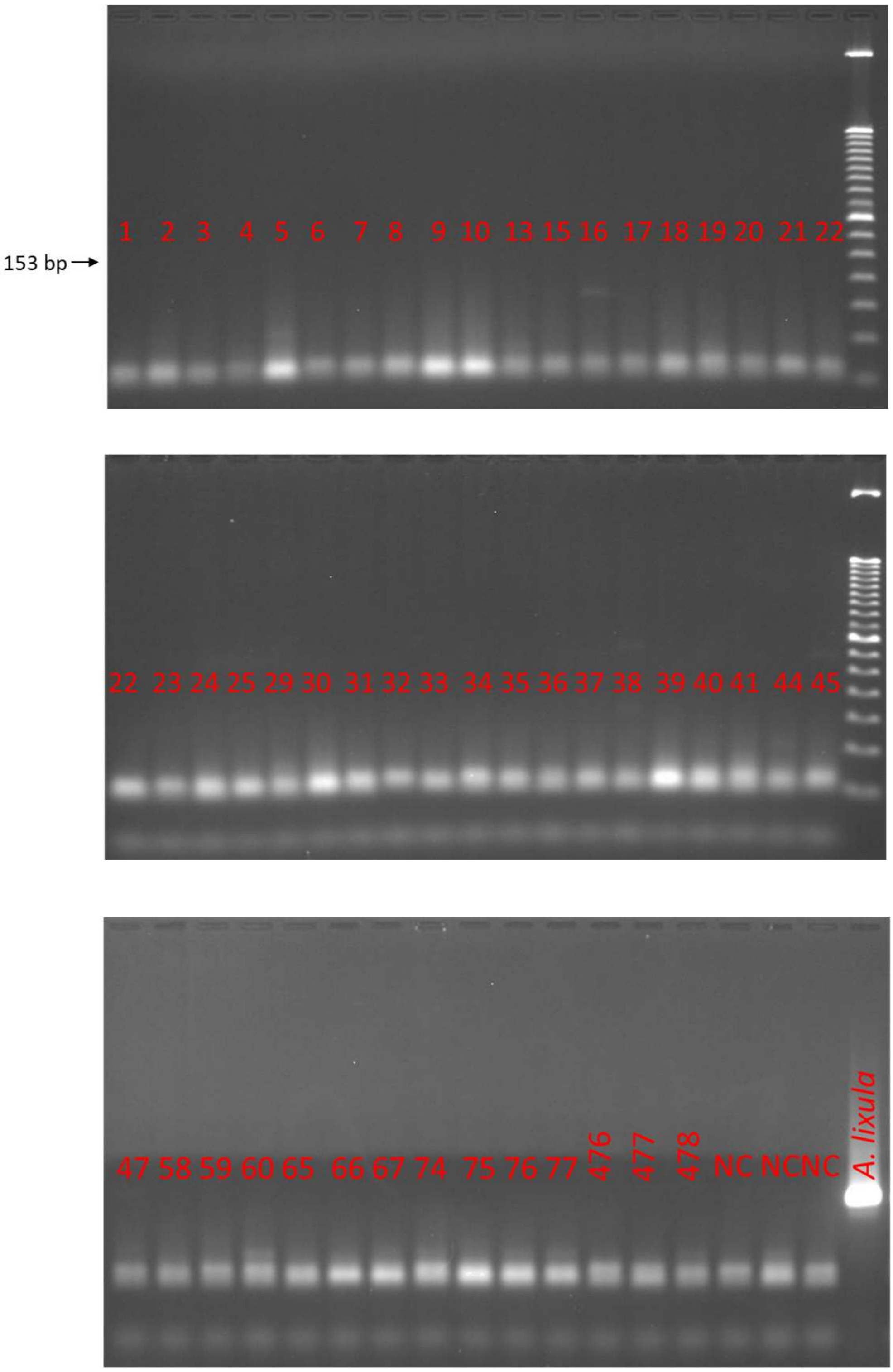
153 bp
1 2 3 4 5 6 7 8 9 10 13 15 16 17 18 19 20 21 22
22 23 24 25 29 30 31 32 33 34 35 36 37 38 39 40 41 44 45
47 58 59 60 65 66 67 74 75 76 77 476 477 478 NC NC NC A. lixula
