# Supplementary figures and images for: Unravelling Hidden Trophic Interactions Among Sea Urchin Juveniles and Macroinvertebrates by DNA Amplification
Source: Mol Ecol. 2025 Nov 13;34(24):e70163. doi: 10.1111/mec.70163 (PMC12717973; doi:10.1111/mec.70163)

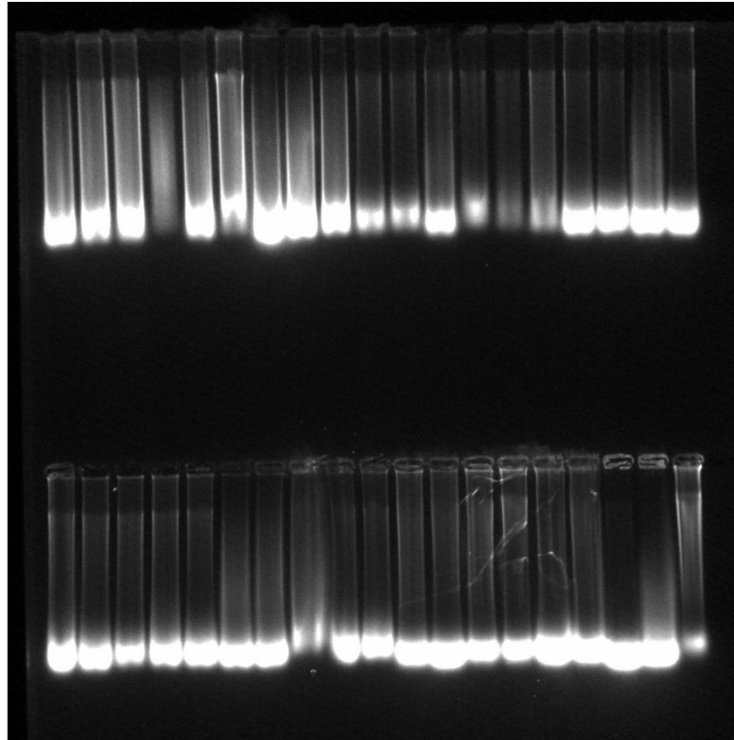

Fig. S2. Gel showing degraded total DNA extracted from randomly selected invertebrate samples.

Supplement: Supplementary file 1 — Figure S1–S7: mec70163‐sup‐0003‐FigureS1–S7.zip. [file MEC-34-e70163-s002.zip › mec70163-sup-0001-FigureS1-S7/FigureS2.pdf]
