# Supplementary material for: Unravelling Hidden Trophic Interactions Among Sea Urchin Juveniles and Macroinvertebrates by DNA Amplification
Source: Mol Ecol. 2025 Nov 13;34(24):e70163. doi: 10.1111/mec.70163 (PMC12717973; doi:10.1111/mec.70163)
Supplement: Supplementary file 1 — Figure S1–S7: mec70163‐sup‐0003‐FigureS1–S7.zip. [file FigureS1.pdf › mec70163-sup-0001-FigureS1-S7/FigureS1.pdf]

MOLECULAR ECOLOGY

Supplemental Information for:

Unravelling hidden trophic interactions among sea urchin juveniles and macroinvertebrates by DNA amplification

Alberto Sutera, Chiara Bonaviri, Francesco Di Trapani, Francesca La Bella, Vesna Macic, Olivera Marković, Bernat Hereu, Roberto De Michele

Fig. S1. Pictures of collected invertebrates.

Table of Contents:

Sample code	Page
86	1-2
87	3-4
88	5
89	6-7
90	8-9
91	10-11
92	12-13
93	14
94	15-16
95	17-18
96	19-20
98	21-22
99	23-24
100	25-26
101	27-28
102	29-30
103	31-32
104	33-34
105	35-36
106	37
107	38-40

**108**	41-42
**109**	43-44
**111**	45-46
**112**	47
**114**	48
**115**	49
**117**	50-53
**118**	54-55
**119**	56-57
**120**	58
**121**	59-60
**122**	61
**123**	62-63
**124**	64
**125**	65
**126**	66-67
**127**	68
**128**	69
**129**	70-71
**130**	72-73
**131**	74-75
**132**	76
**133**	77-78
**134**	79
**135**	80-81
**136**	82
**137**	83-84
**138**	85-86
**141**	87-88
**142**	89-90

MOLECULAR ECOLOGY

**143**	91-92
**144**	93-94
**145**	95-96
**146**	97-98
**147**	99-100
**148**	101-102
**149**	103-104
**150**	105-106
**151**	107-108
**152**	109
**154**	110
**156**	111
**157**	112
**159**	113-114
**160**	115
**161**	116
**162**	117-118
**163**	119
**167**	120
**165**	121
**166**	122
**167**	123
**168**	124
**169**	125
**170**	126-127
**171**	128-129
**172**	130-131
**173**	132-133
**174**	134
**182**	135-136

MOLECULAR ECOLOGY

**183**	137-138
**184**	139-140
**186**	141-142
**189**	143
**190**	144
**192**	145
**193**	146
**194**	147
**198**	148
**197**	149
**200**	150-151
**201**	152-153
**202**	154-155
**203**	156
**204**	157
**205**	158
**206**	159
**207**	160
**208**	161
**209**	162
**210**	163
**211**	164
**212**	165
**213**	166
**214**	167
**215**	168-169
**216**	170-171
**217**	172-173
**218**	174-175
**219**	176-177

**220**	178-179
**221**	180-182
**222**	183-185
**227**	186
**229**	187
**230**	188
**231**	189
**232**	190
**233**	191
**234**	192
**235**	193
**236**	194
**238**	195-196
**239**	197-198
**240**	199
**241**	200-201
**242**	202
**243**	203-204
**244**	205-206
**245**	207-208
**246**	209-210
**247**	211-212
**248**	213-214
**249**	215
**250**	216-217
**251**	218-220
**252**	221-222
**253**	223
**254**	224-225
**256**	226

MOLECULAR ECOLOGY

**257**	227-228
**258**	229-231
**259**	232-234
**260**	235-236
**261**	237-238
**262**	239-240
**263**	241
**264**	242
**265**	243
**266**	244
**267**	245-246
**268**	247-248
**269**	249-250
**270**	251
**271**	252
**272**	253
**273**	254
**274**	255-256
**275**	257-258
**276**	259-260
**282**	261
**284**	262-263
**285**	264
**286**	265
**287**	266
**288**	267
**340**	268-269
**341**	270
**342**	271-272
**345**	273-274

**347**	275-276
**348**	277
**349**	278
**351**	279
**353**	280
**354**	281
**255**	282-284
**356**	285
**357**	286
**358**	287
**359**	288
**360**	289
**362**	290
**363**	291
**364**	292
**365**	293-294
**366**	295
**368**	296
**370**	297
**371**	298
**372**	299
**373**	300
**376**	301-302
**377**	303-304
**379**	305
**380**	306
**381**	307
**382**	308
**383**	309
**384**	310

**385**	311-312
**386**	313-314
**387**	315-316
**390**	317
**391**	318
**392**	319
**394**	320
**395**	321-322
**396**	323
**397**	324
**400**	325-326
**401**	327-329
**402**	330
**403**	331-332
**404**	333-334
**405**	335-336
**406**	337-338
**407**	339
**408**	340
**409**	341
**410**	342
**411**	343
**412**	344
**413**	345
**414**	346
**415**	347
**416**	348
**417**	349
**418**	350-351
**419**	352-353

MOLECULAR ECOLOGY

**420**	354
**421**	355
**422**	356
**423**	357
**424**	358
**425**	359
**426**	360
**427**	361
**428**	362
**429**	363-364
**430**	365-366
**431**	367-368
**432**	369
**438**	370-371
**439**	372
**440**	373-374
**441**	375-376
**442**	377-378
**443**	379
**445**	380
**446**	381
**447**	382
**448**	383
**450**	384
**451**	385
**455**	386-387
**456**	388-389
**457**	390-391
**458**	392-393
**459**	394-395

MOLECULAR ECOLOGY

**460**	396-397
**461**	398-399
**462**	400-401
**463**	402-403
**464**	404-405
**465**	406
**466**	407-407
**467**	409-410
**468**	411
**469**	412
**470**	413
**471**	414
**472**	415-416
**473**	417-418
**474**	419-420
**475**	421-422
**479**	423-424
**480**	425-427
**481**	428-429
**482**	430-431
**483**	432
**484**	433-434
**485**	435-436
**486**	437-438
**487**	439-440
**488**	441-442
**489**	443-444
**490**	445-446
**491**	447-448
**493**	449-450

**494**	451
**495**	452-453
**496**	454-455
**498**	456
**499**	457
**500**	458
**501**	459
**502**	460-461
**503**	462
**504**	463
**505**	464-465
**506**	466-467
**507**	468-469
**508**	470-471
**509**	472-473
**510**	474-475
**511**	476-477
**512**	478-479
**513**	480-481
**514**	482-483
**515**	484-485
**516**	486-487
**517**	488-489
**518**	490-491
**519**	492
**520**	493
**521**	494
**522**	495
**523**	496-497
**524**	498

**525**	499
**526**	500-501
**527**	502-503
**528**	504
**529**	505
**530**	506
**531**	507
**532**	508
**533**	509
**534**	510
**535**	511
**536**	512
**537**	513
**538**	514
**539**	515
**540**	516-517
**541**	518-519
**542**	520-521
**543**	522-524
**544**	525-526
**545**	527-528
**546**	529-530
**547**	531-532
**548**	533-534
**549**	535-536
**550**	537-538
**551**	539
**552**	540
**553**	541
**554**	542

**555**	543
**556**	544-545
**557**	546
**558**	547
**559**	548
**560**	549
**561**	550-551
**562**	552-553
**563**	554
**564**	555
**565**	556
**566**	557
**567**	558
**568**	559
**569**	560
**570**	561
**571**	562
**572**	563
**573**	564
**574**	565
**576**	566
**577**	567
**578**	568

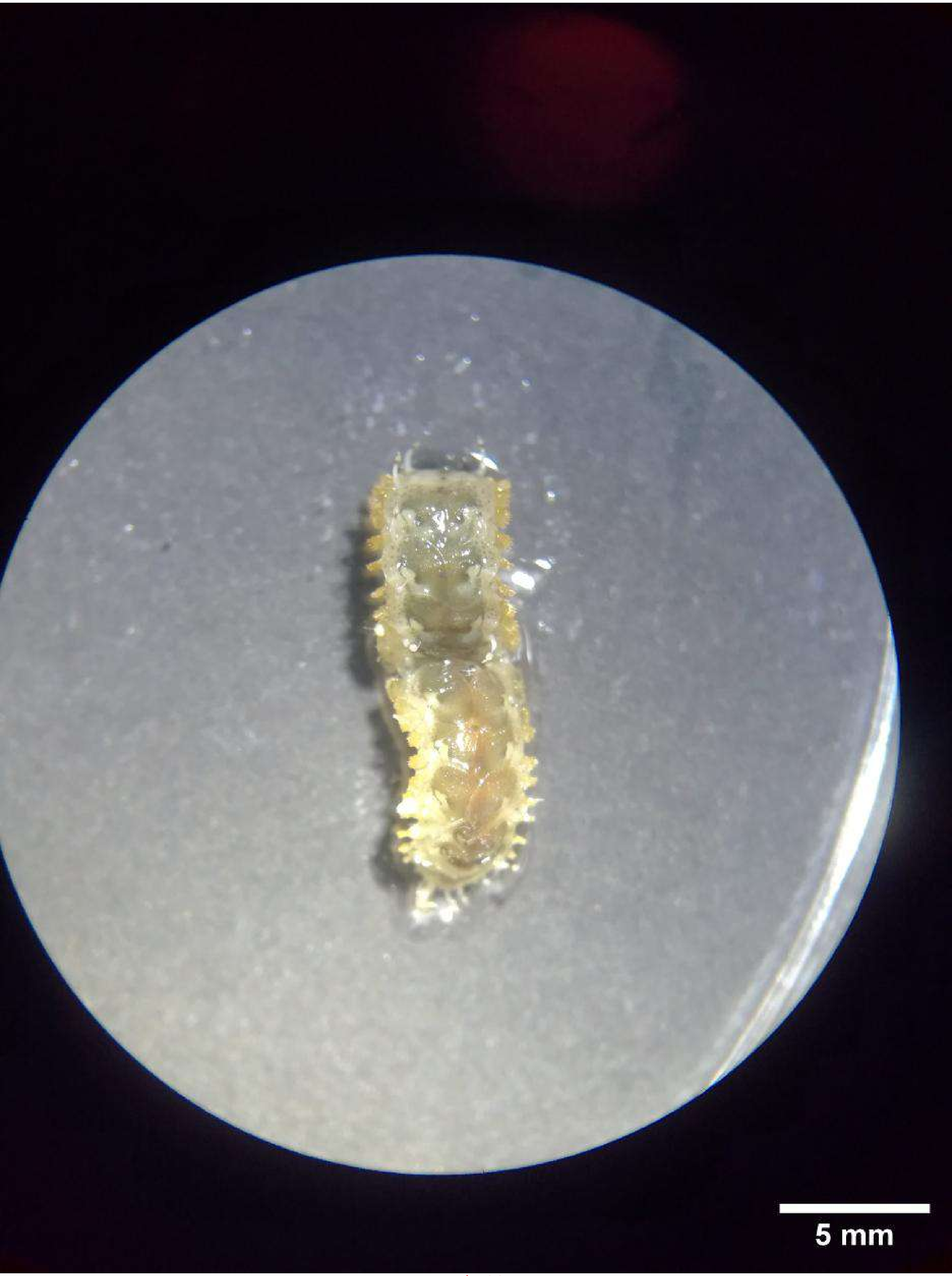
5 mm

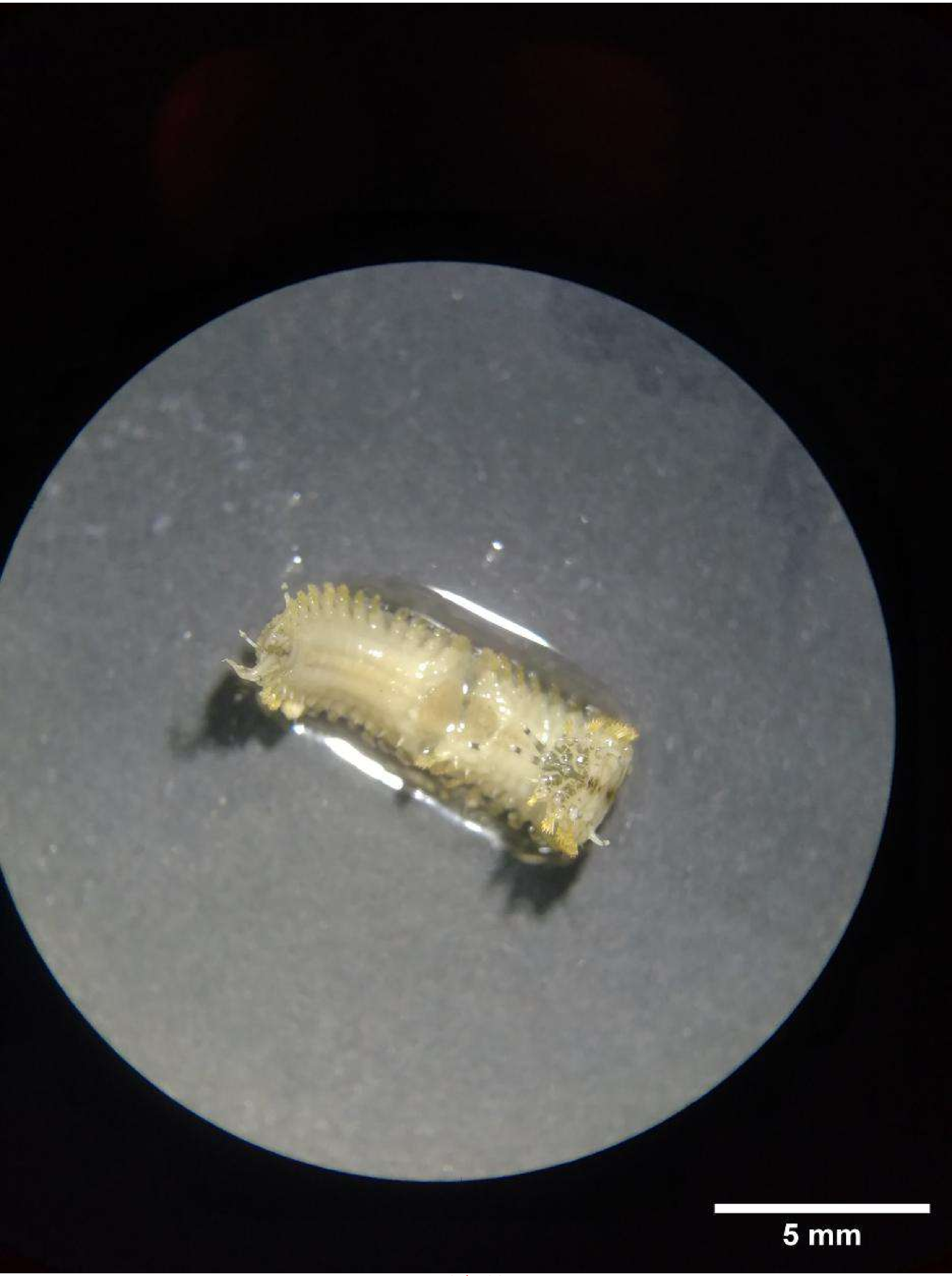
5 mm

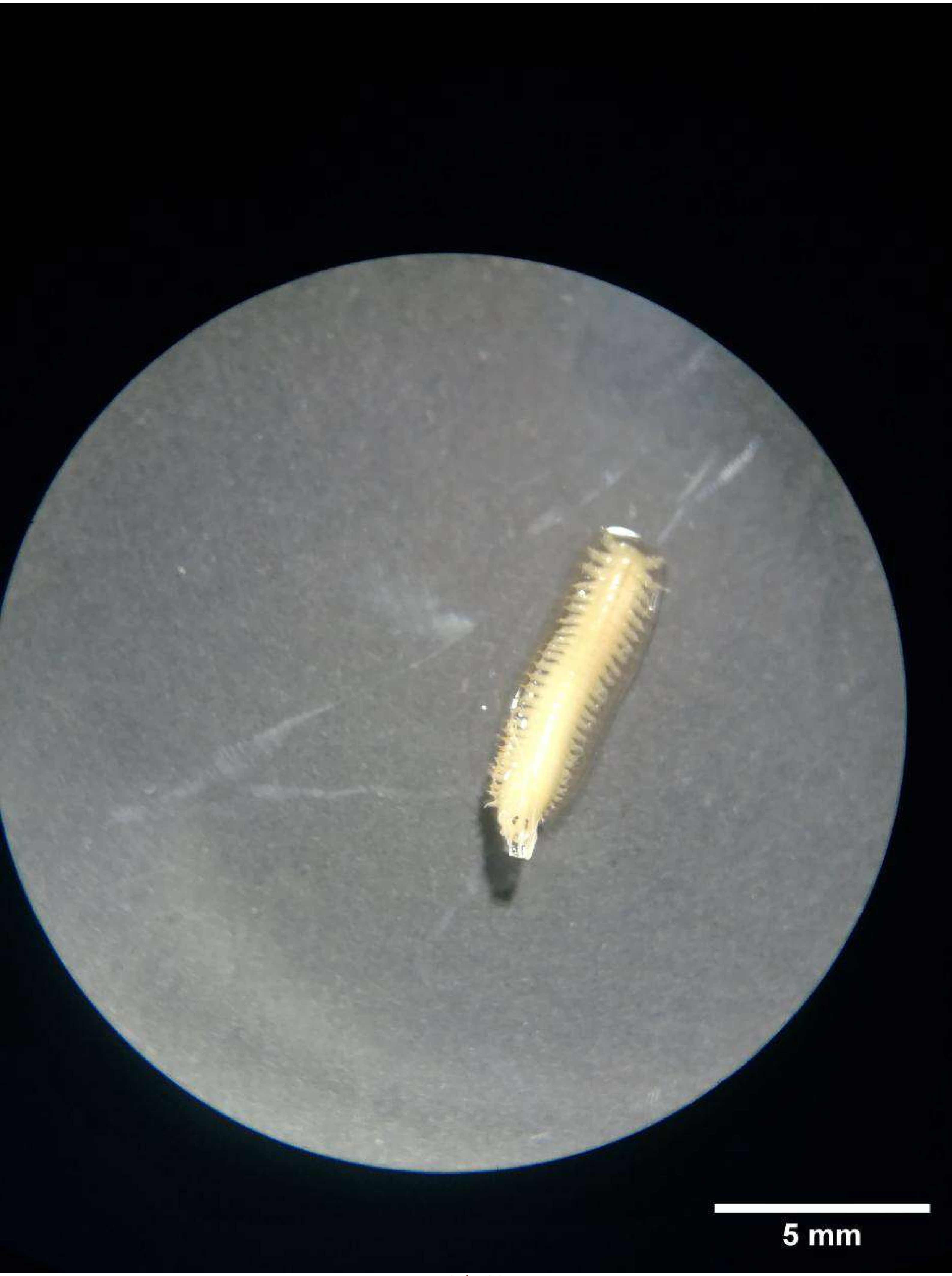
5 mm

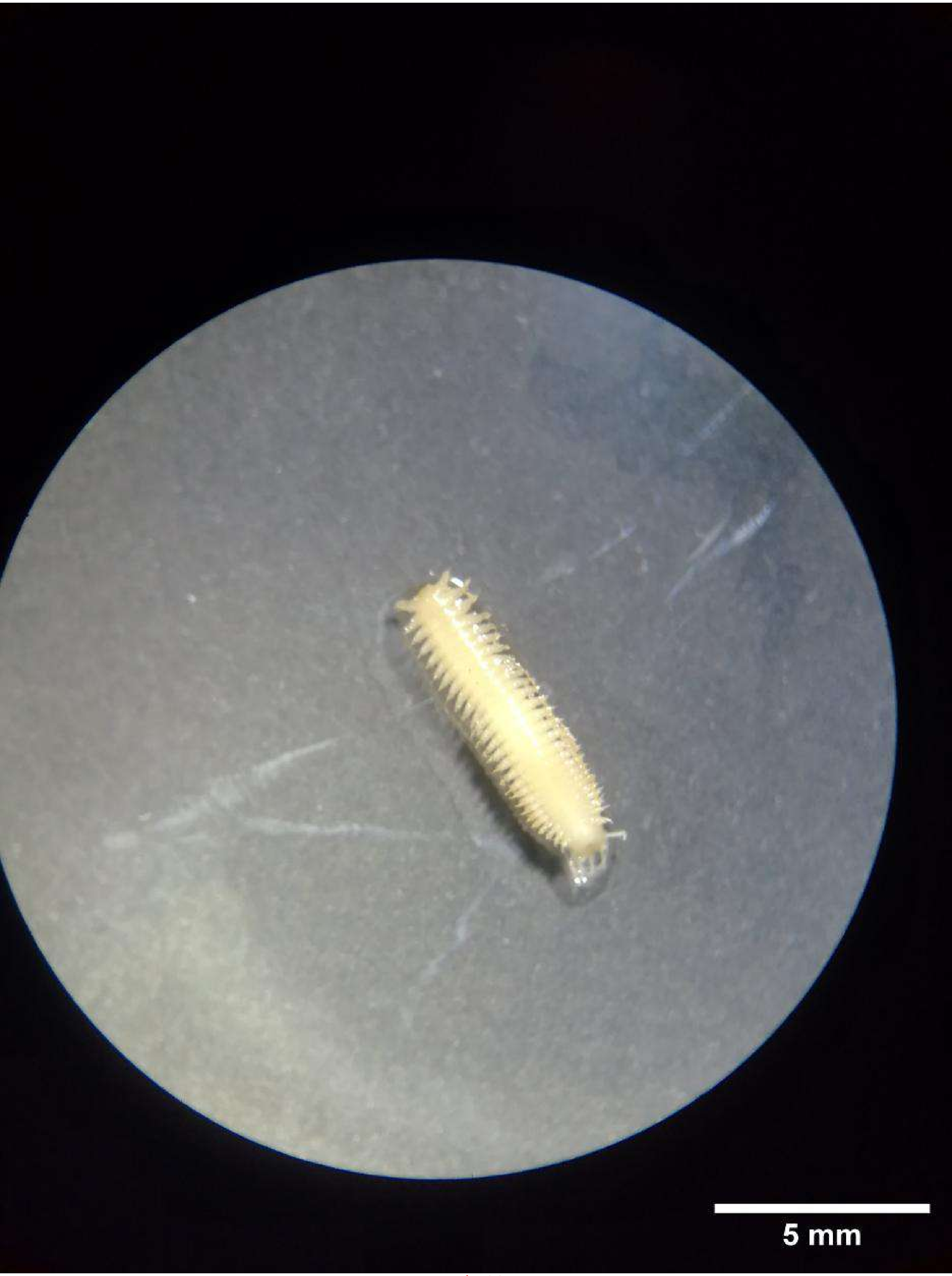
5 mm

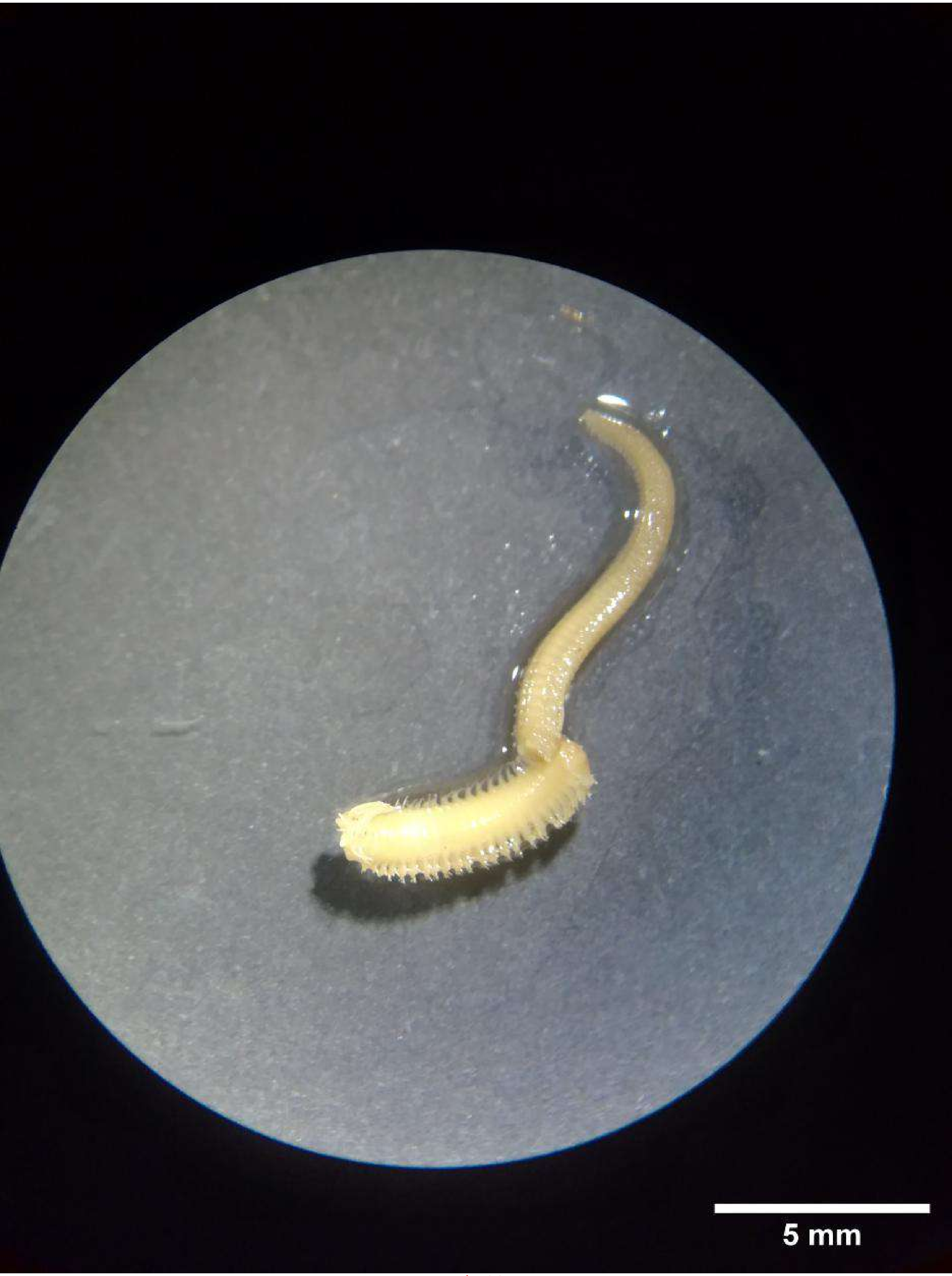
5 mm

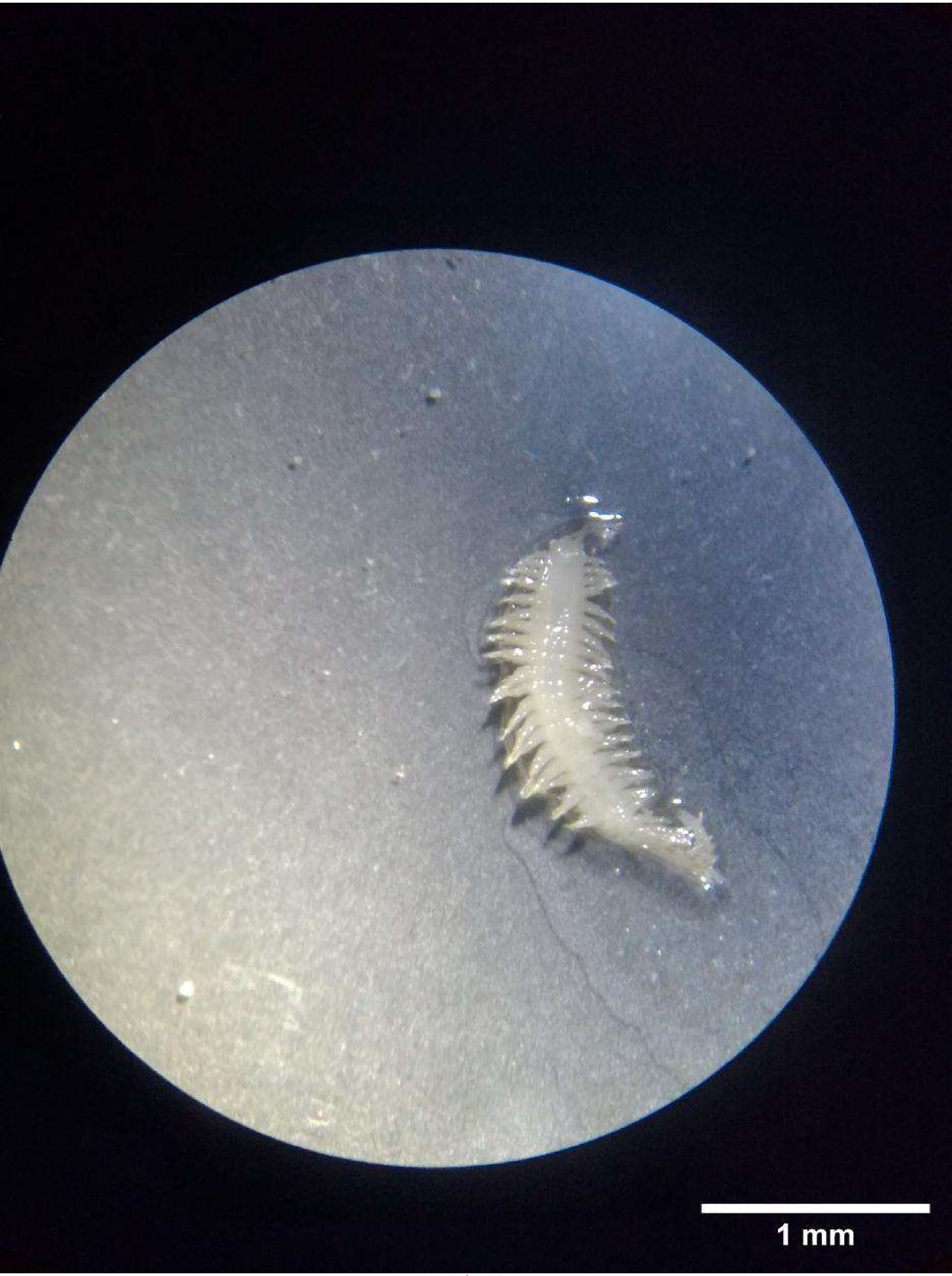
1 mm

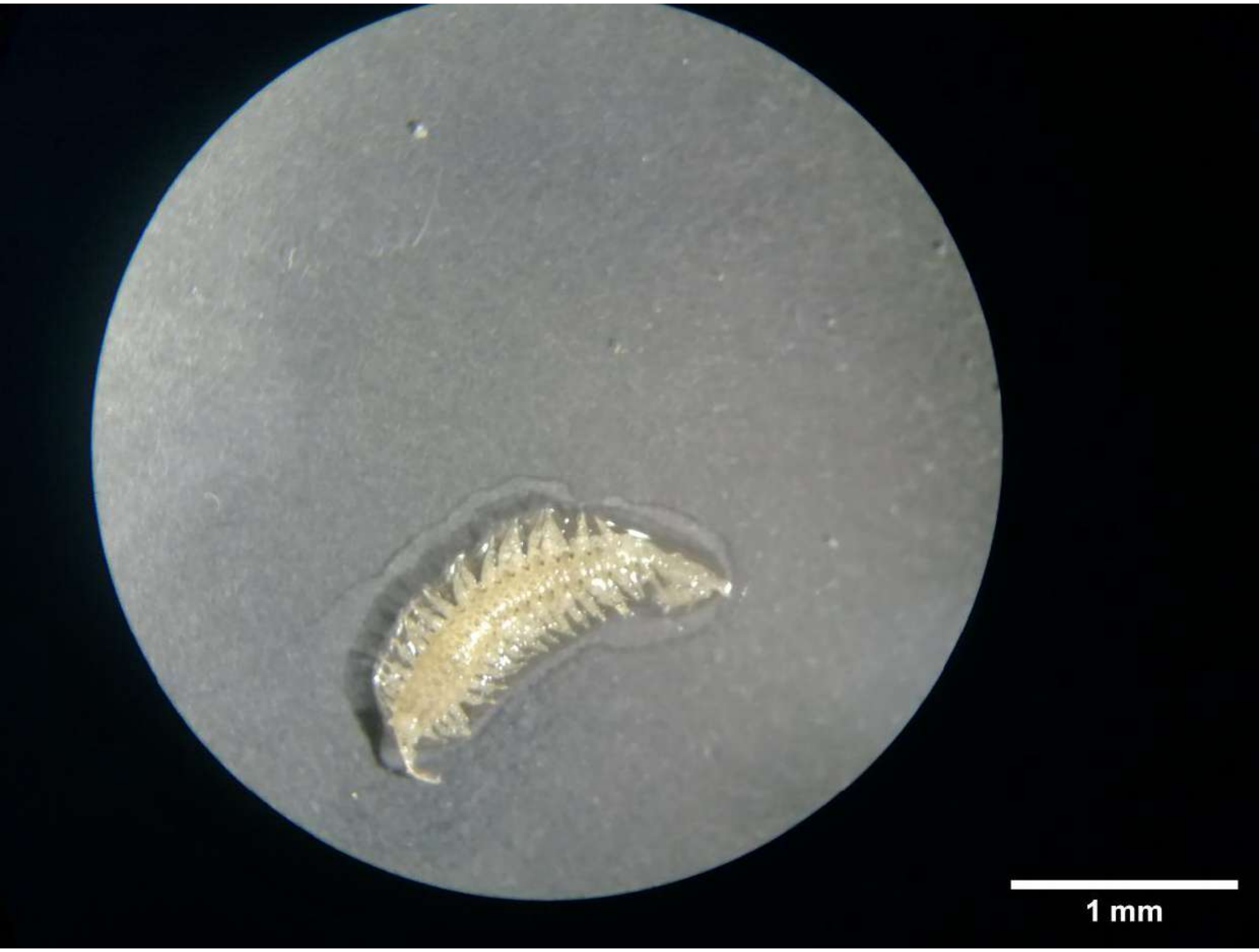
1 mm

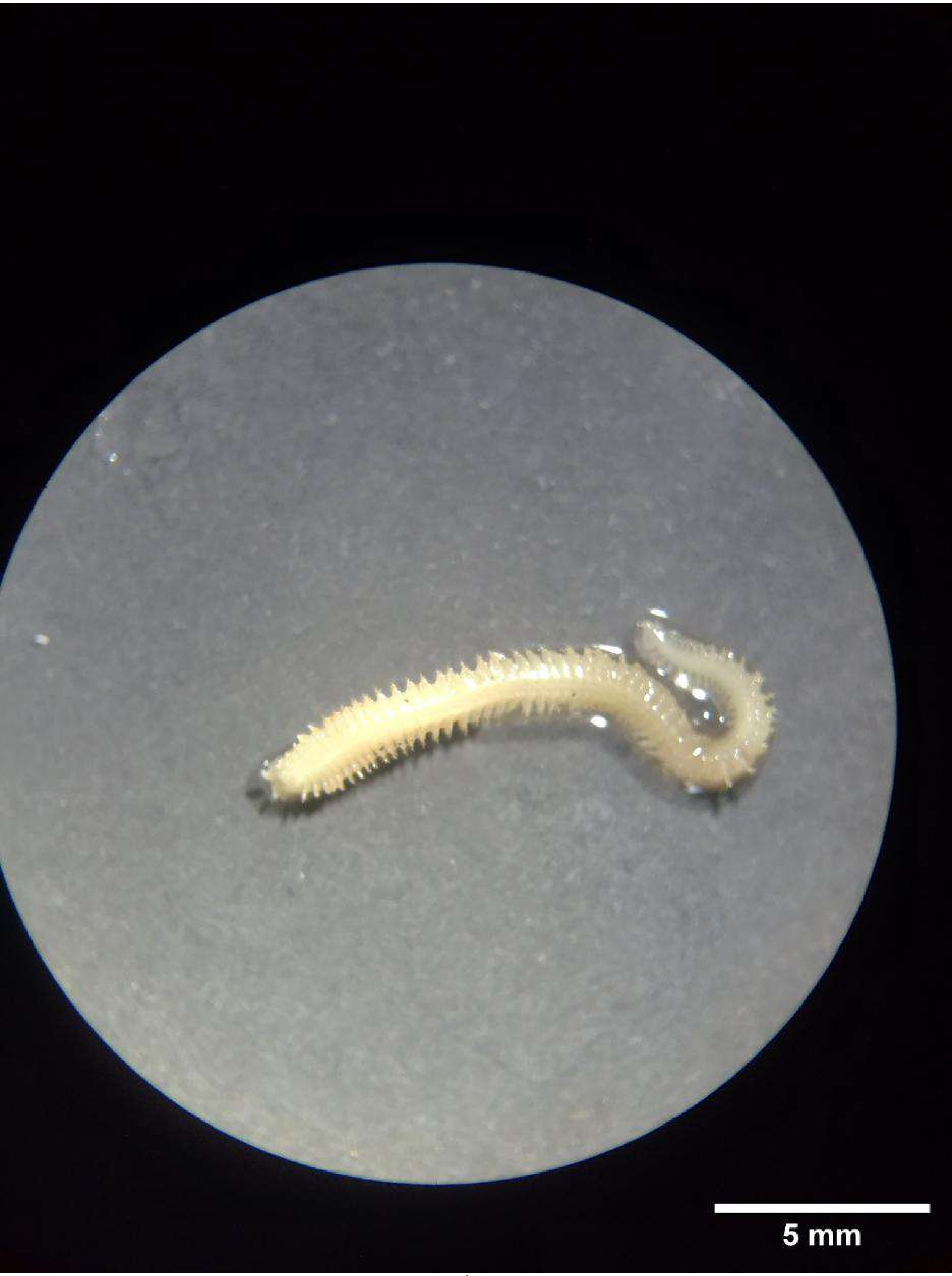
5 mm

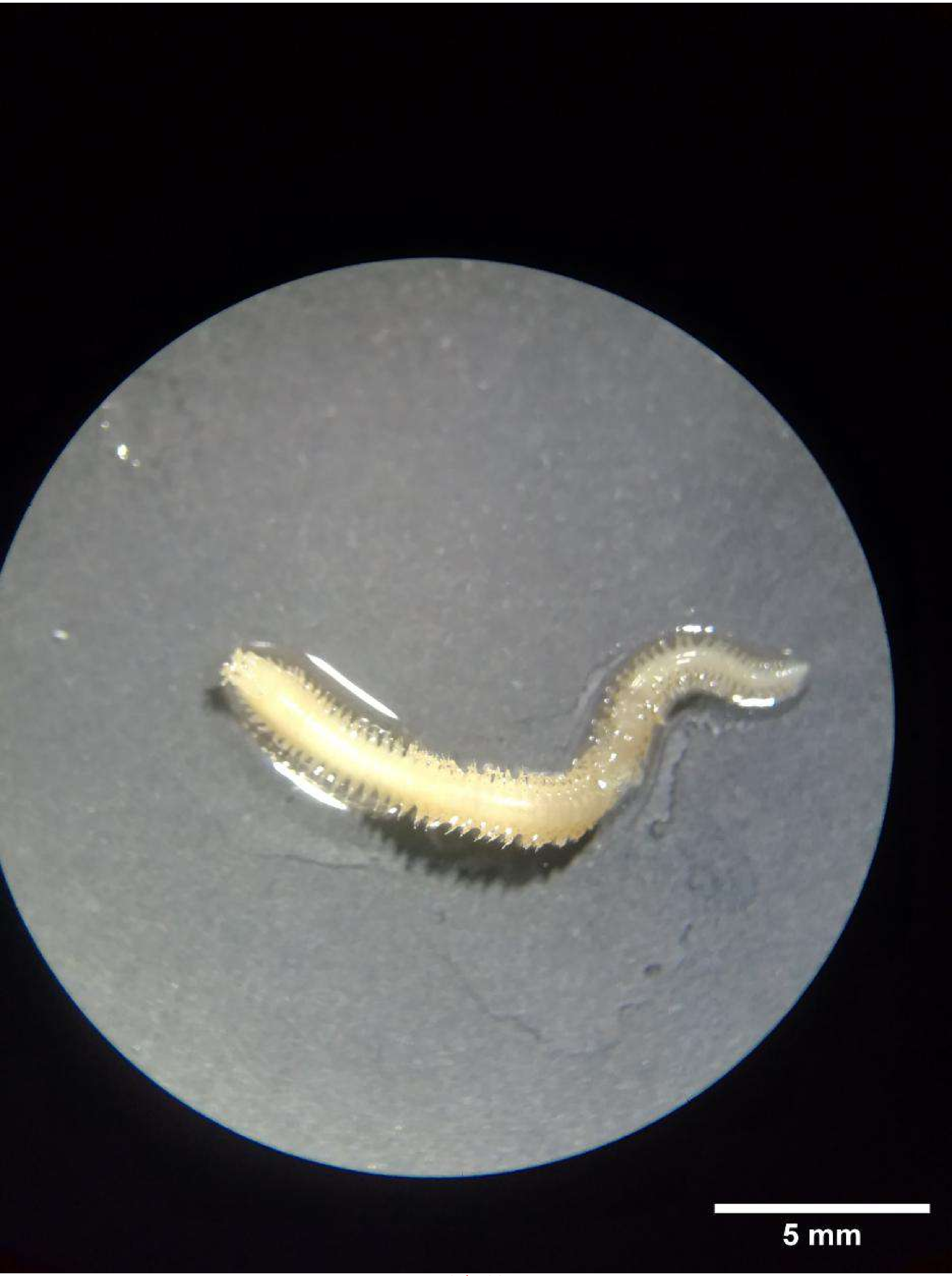
5 mm

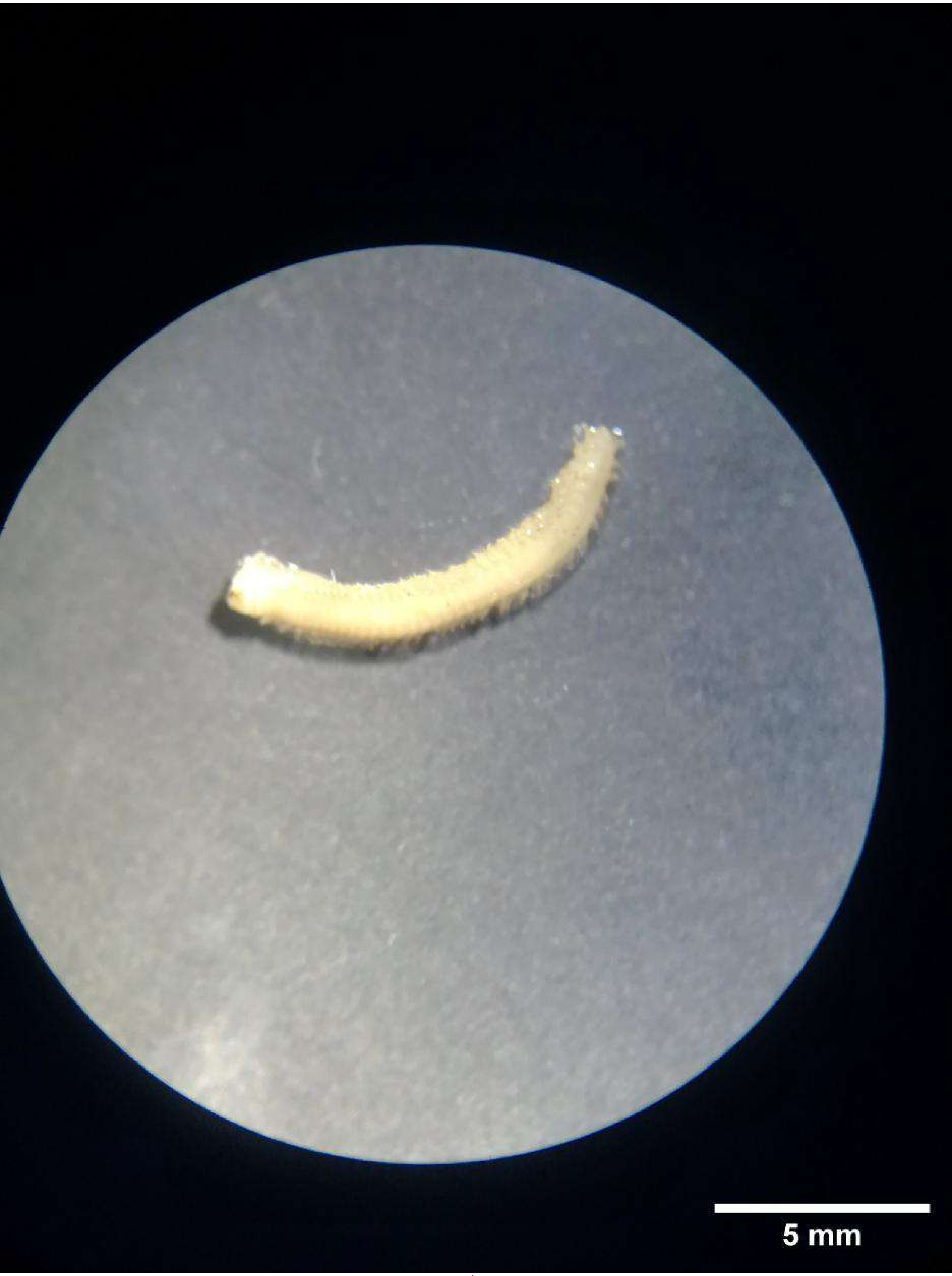
5 mm

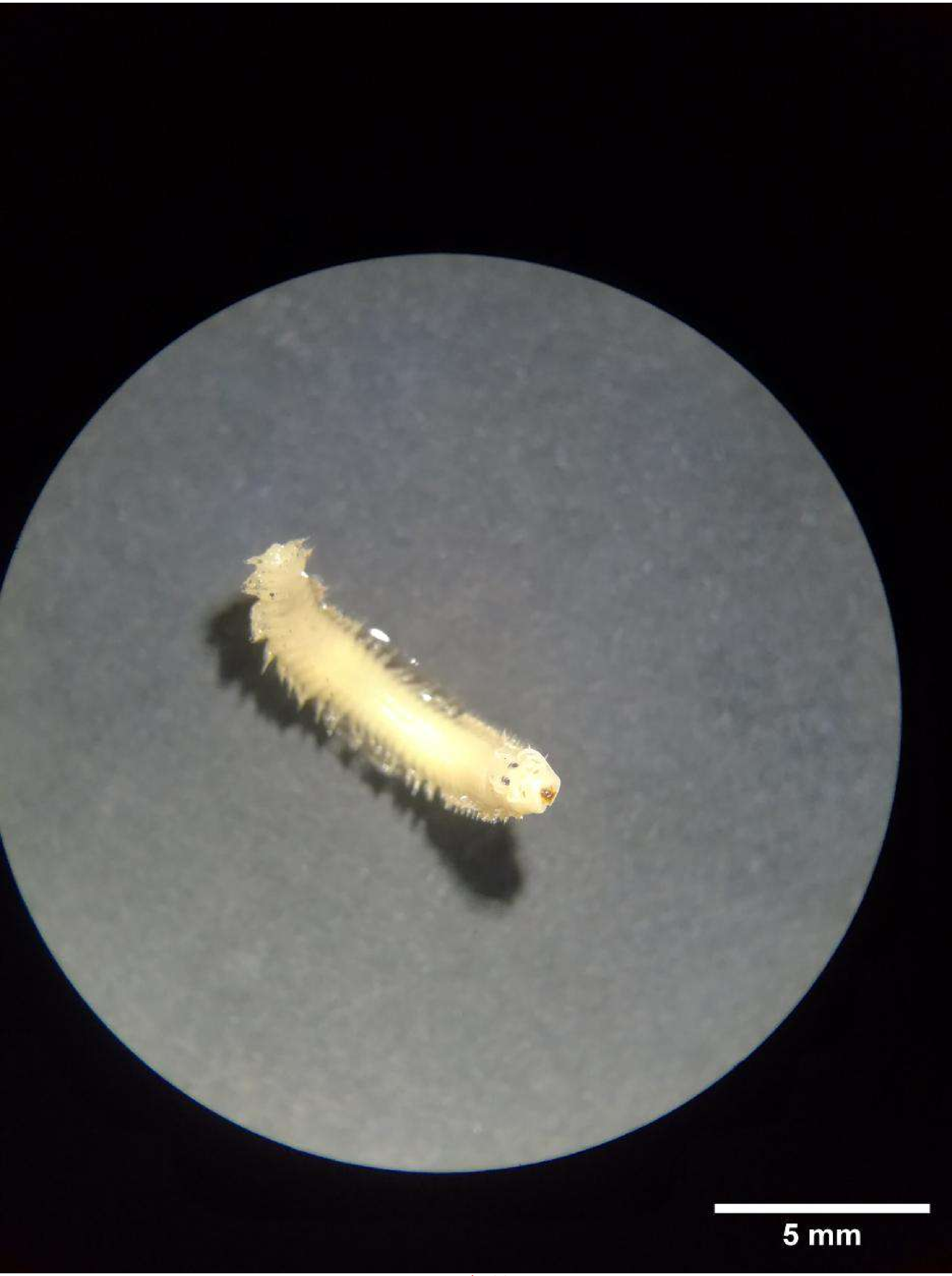
5 mm

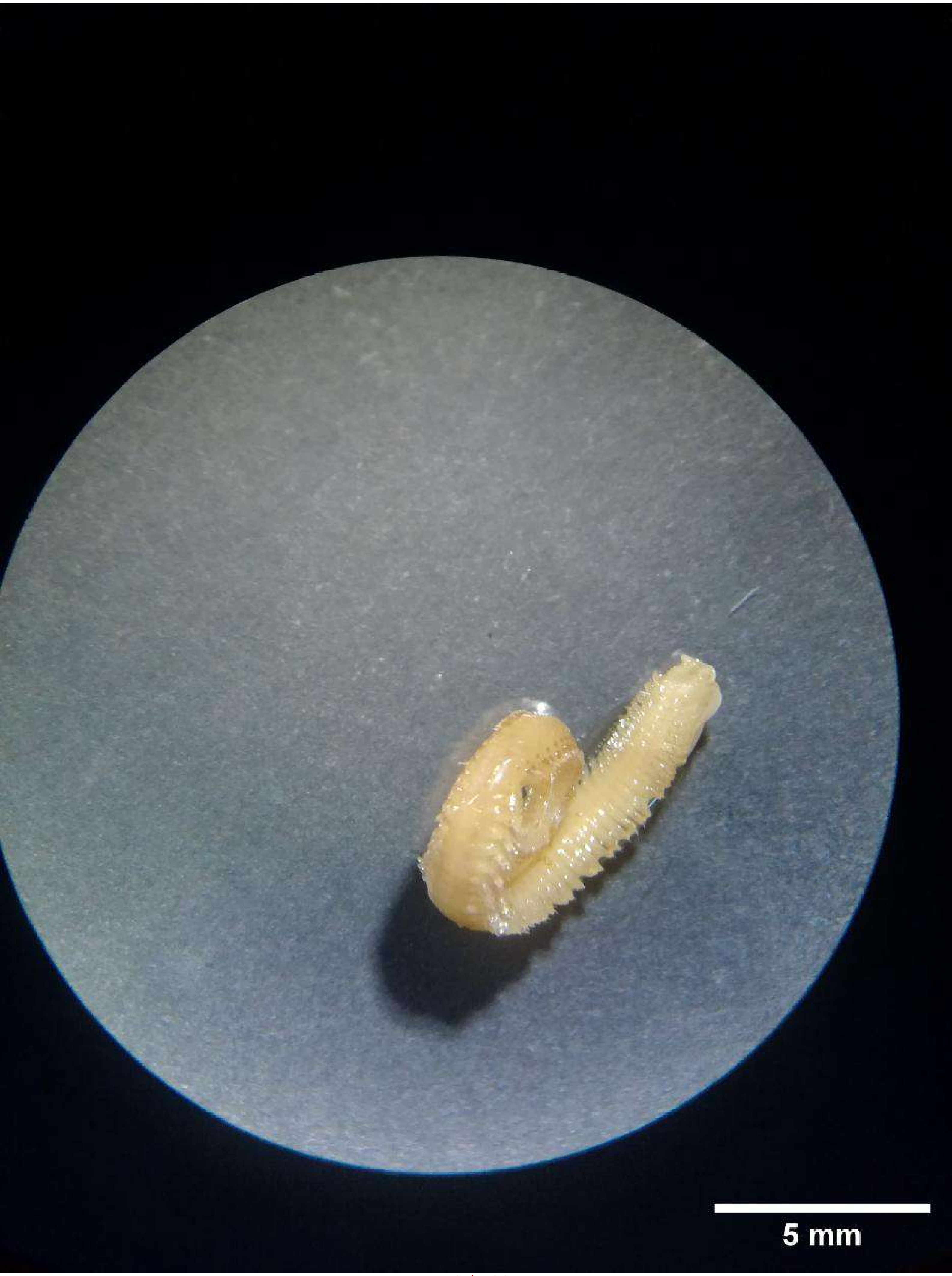
5 mm

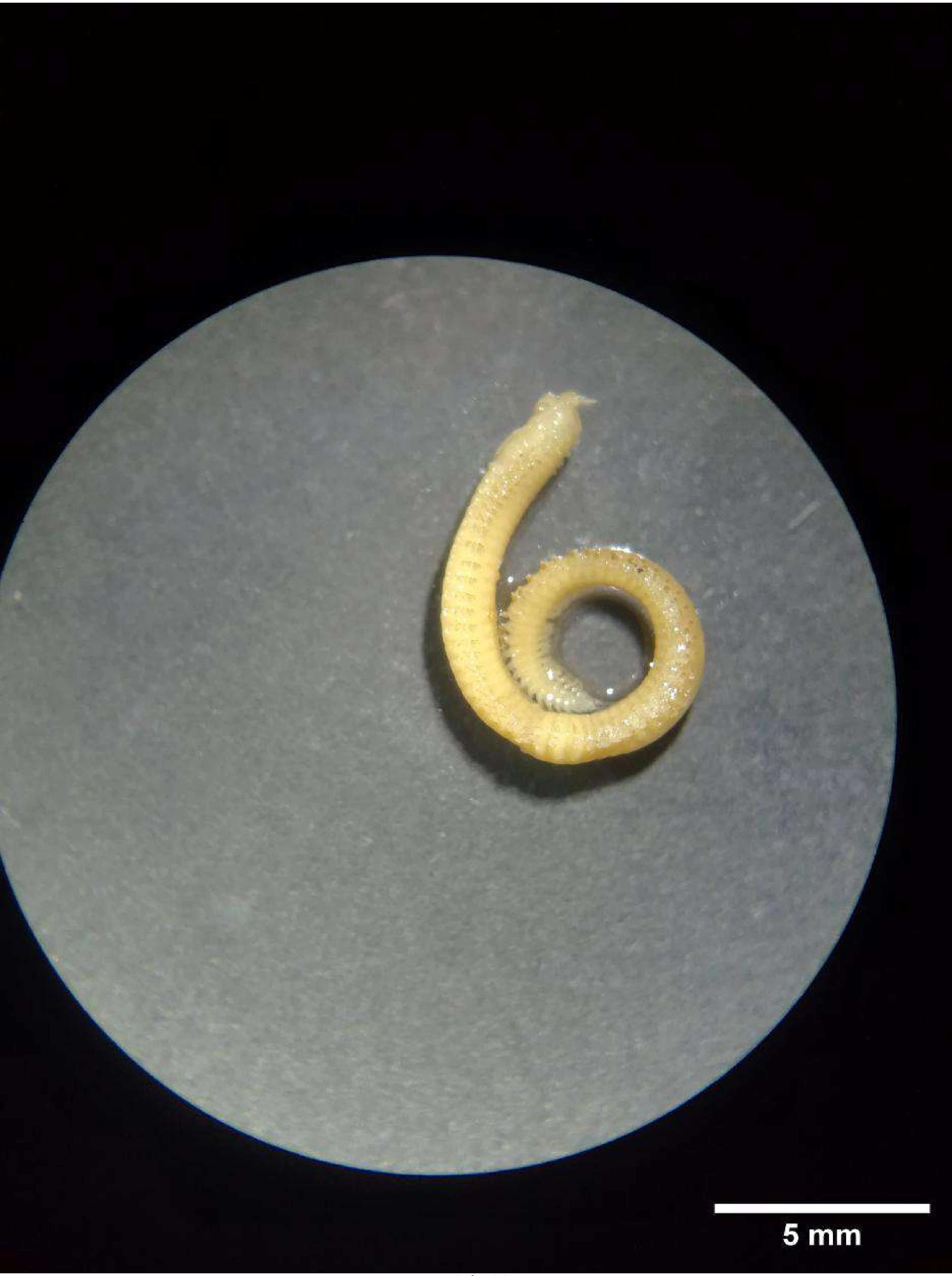
5 mm

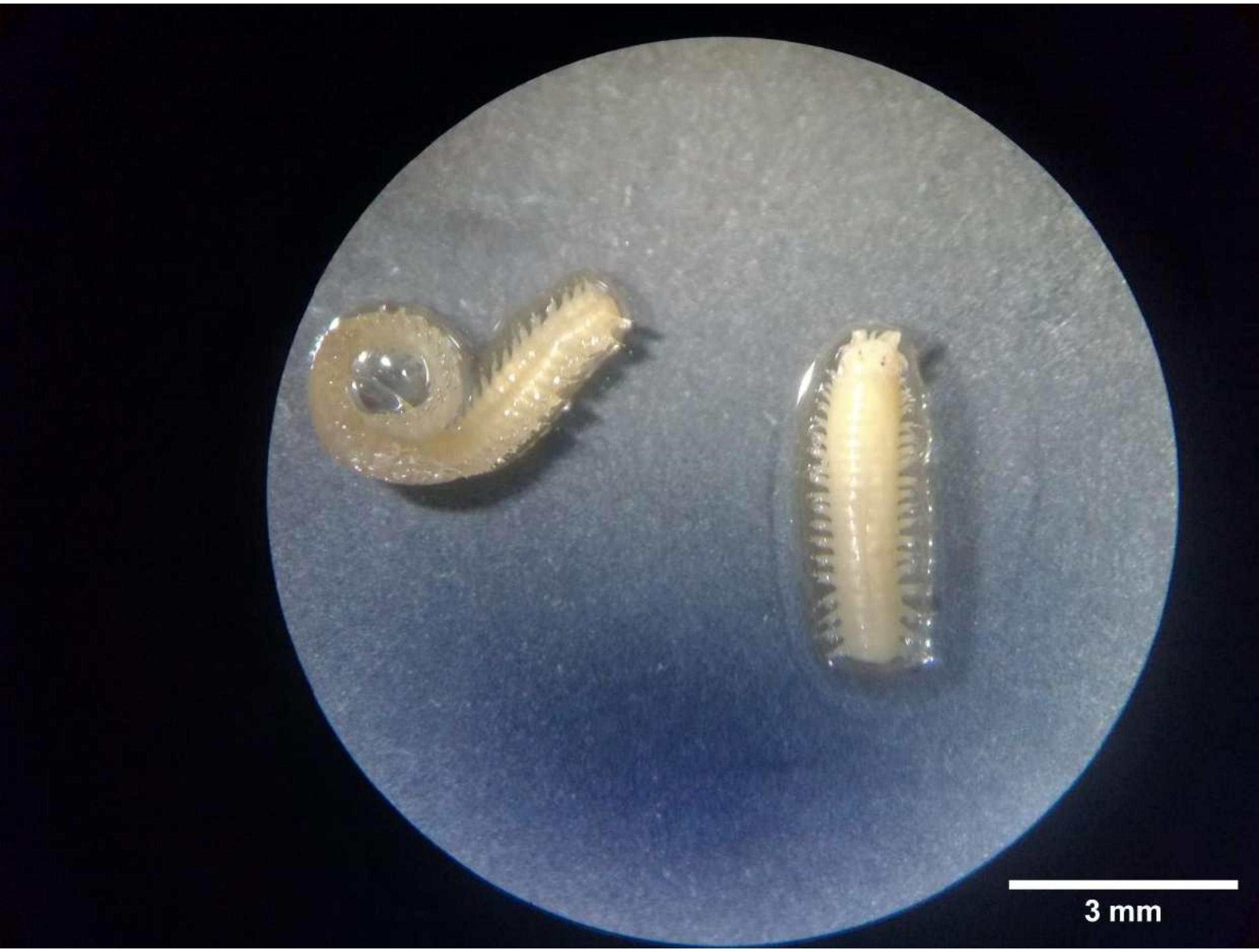
3 mm

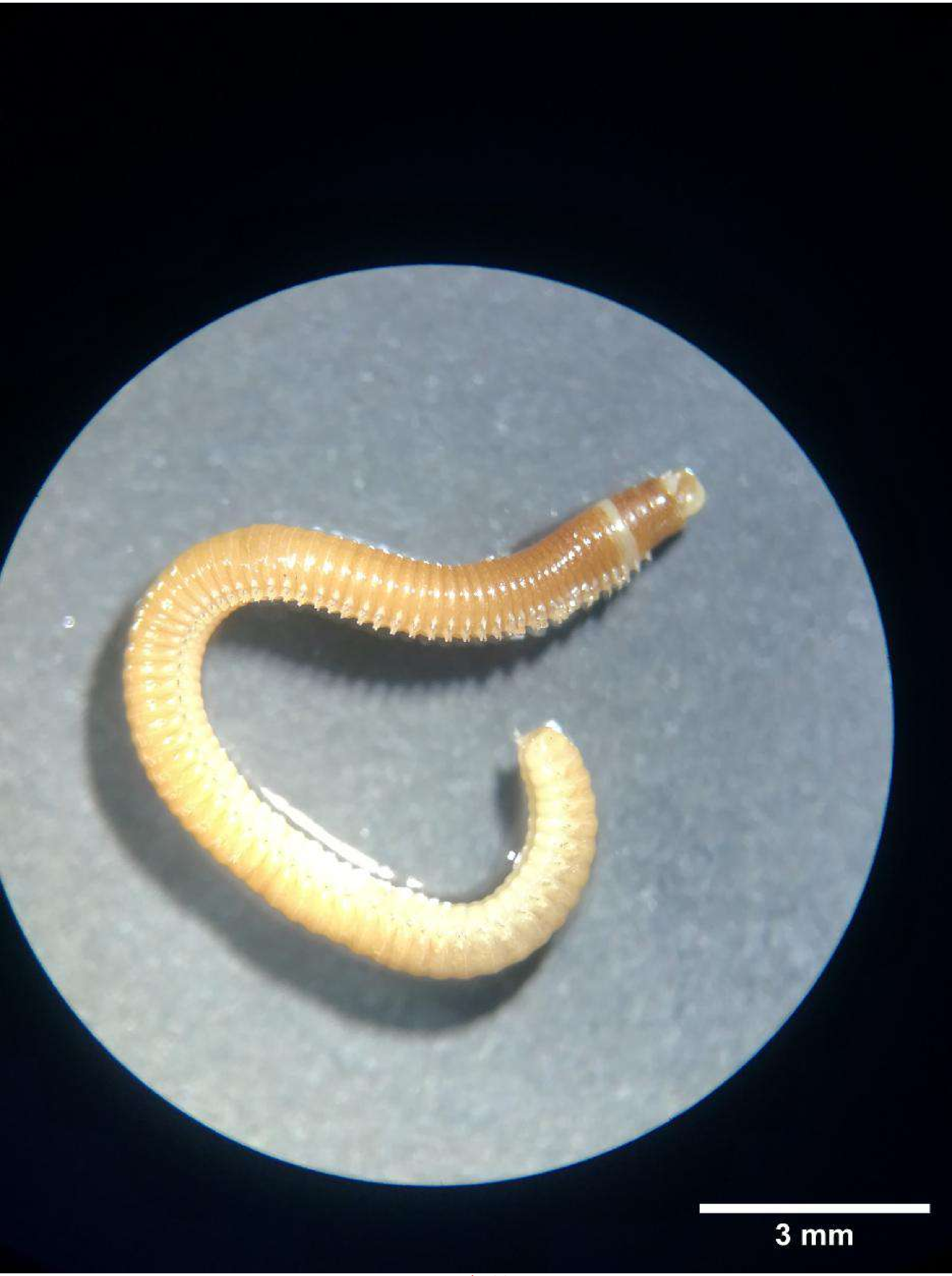
3 mm

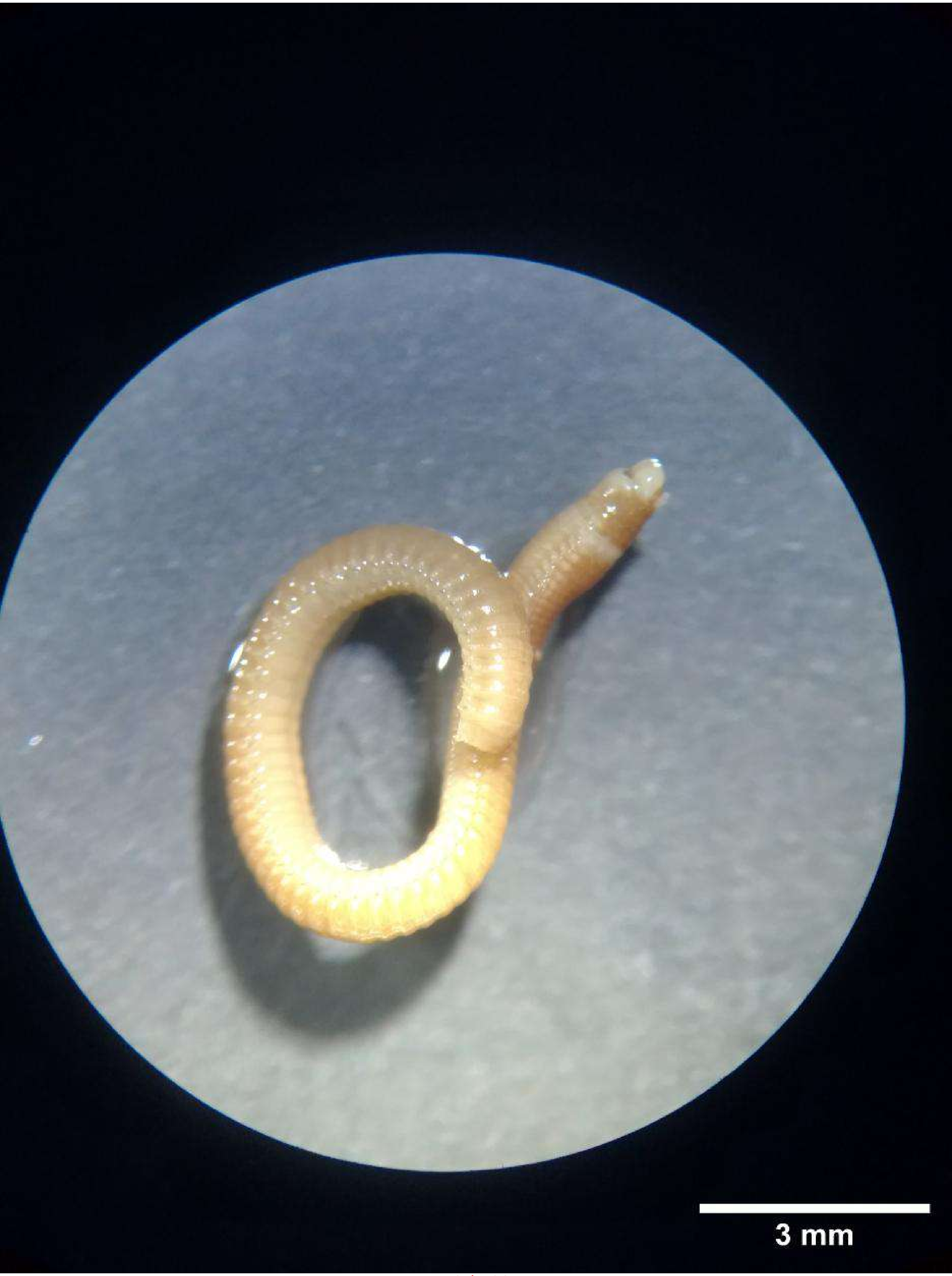
3 mm

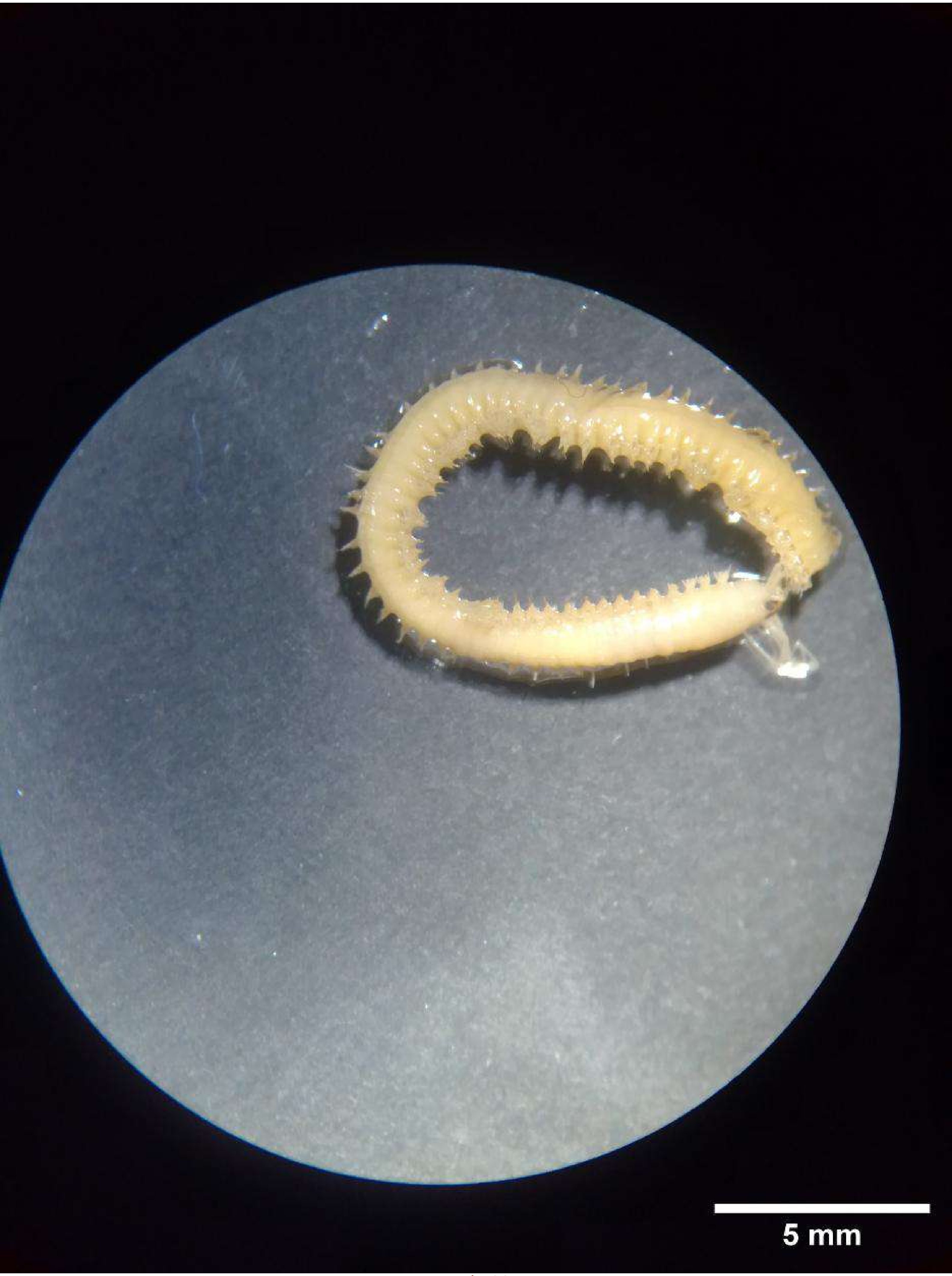
5 mm

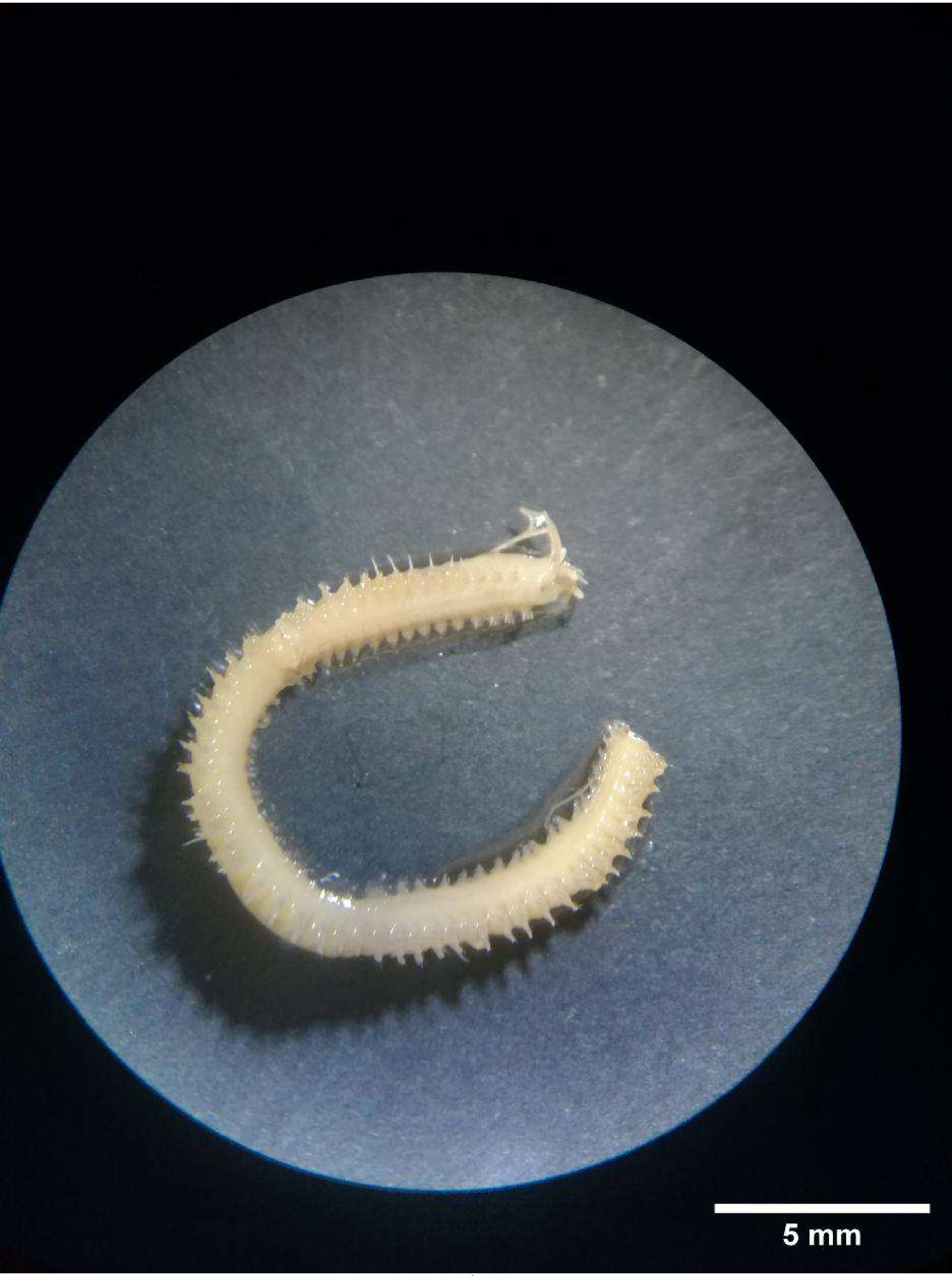
5 mm

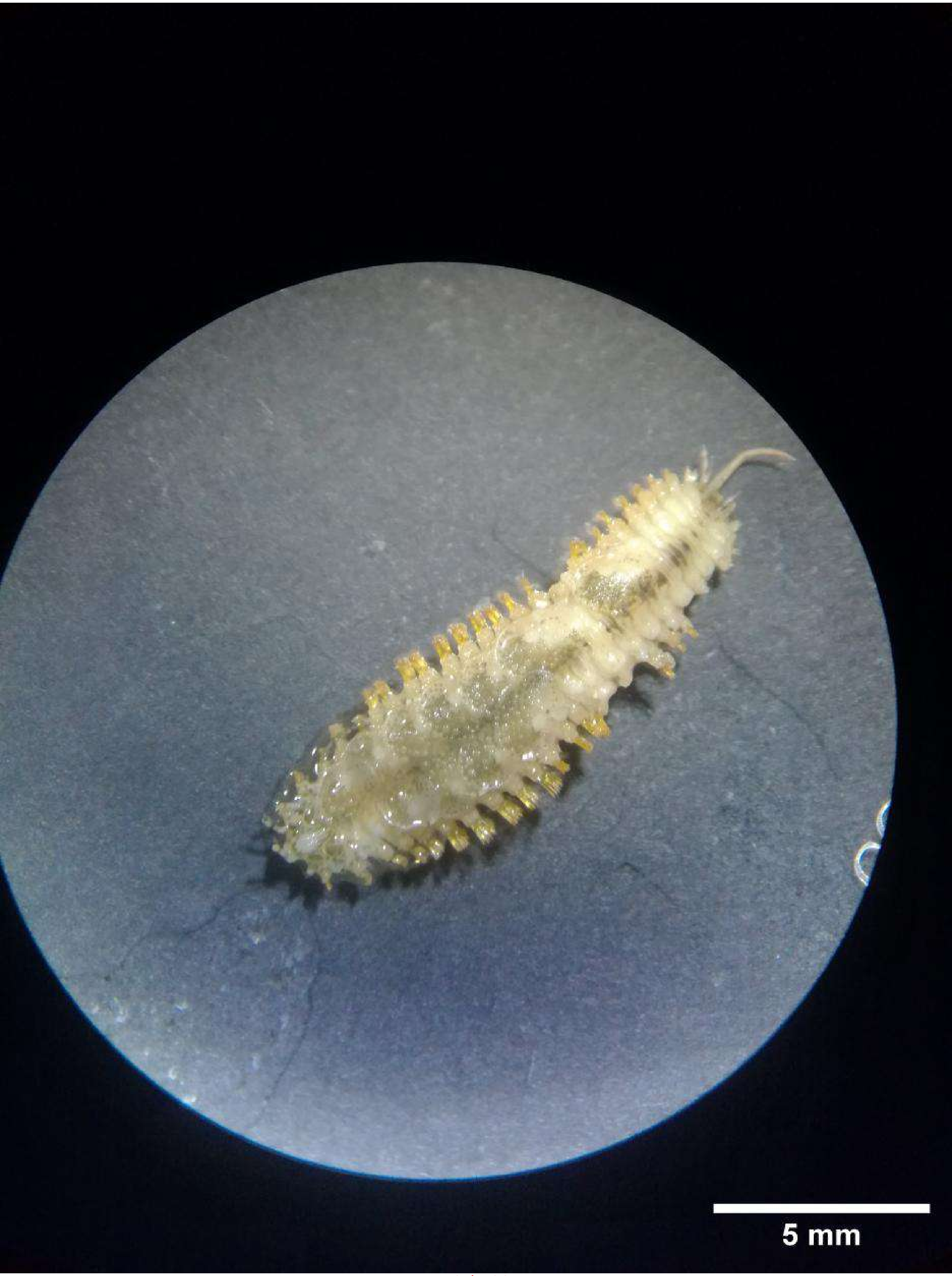
5 mm

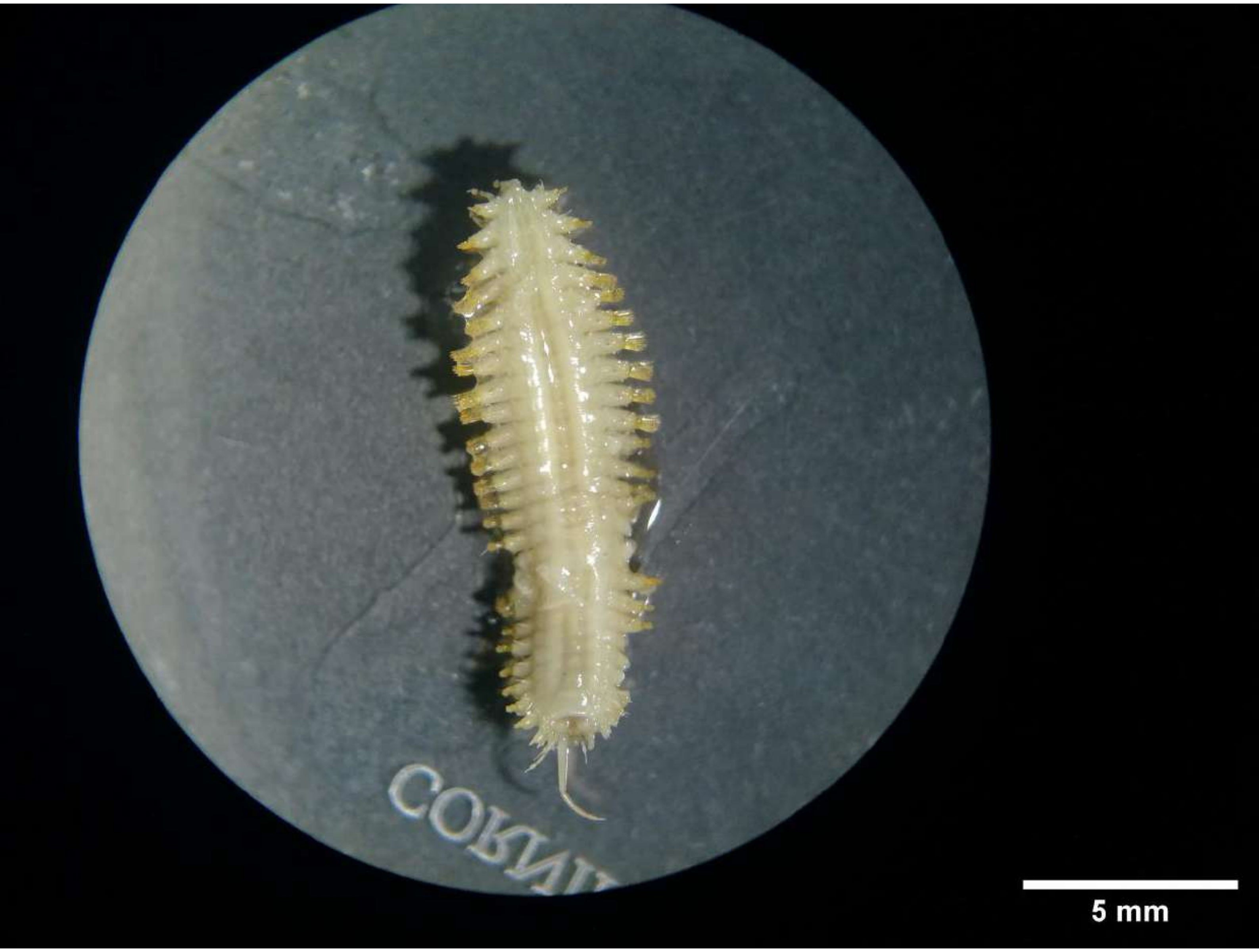
5 mm

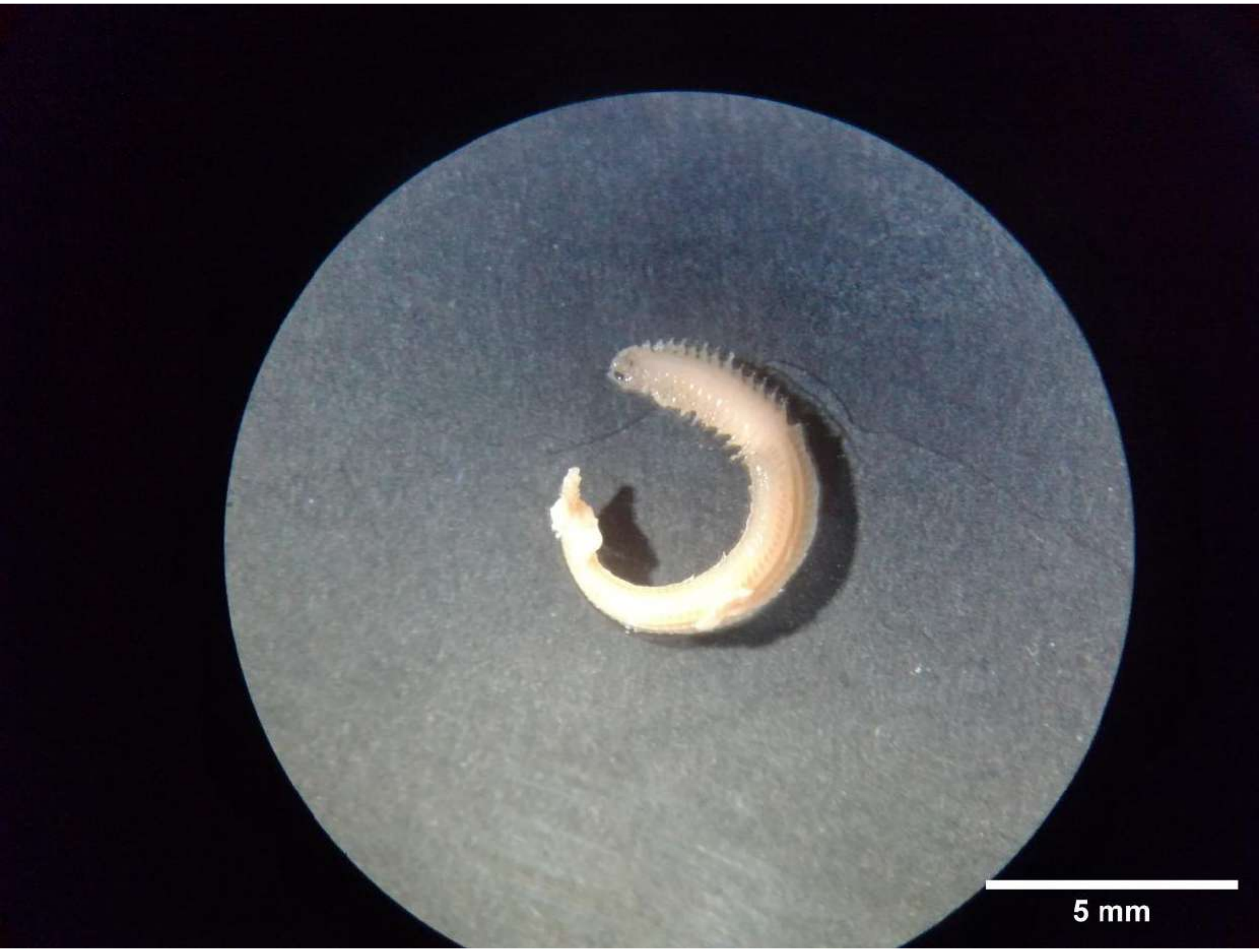
5 mm

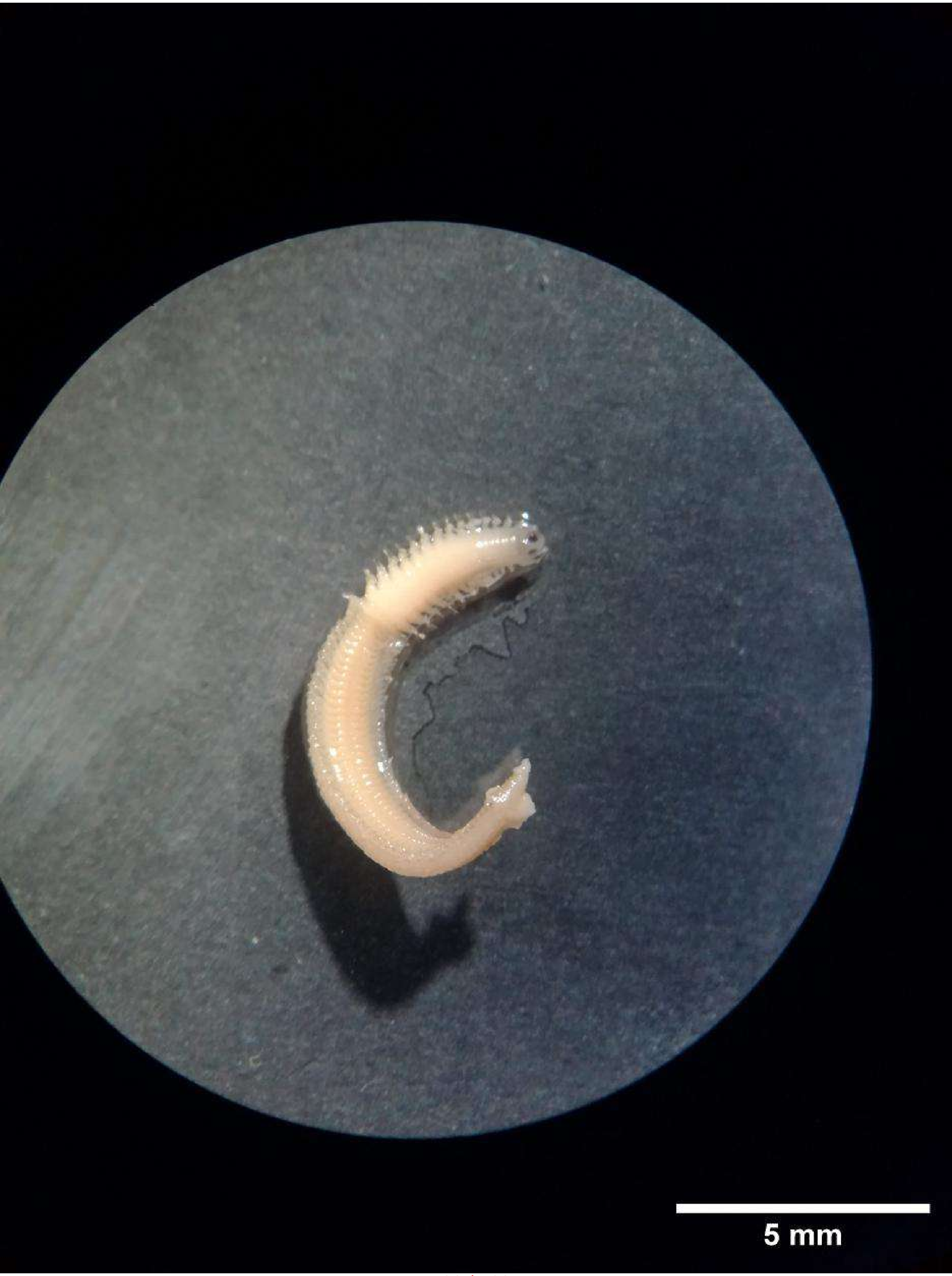
5 mm

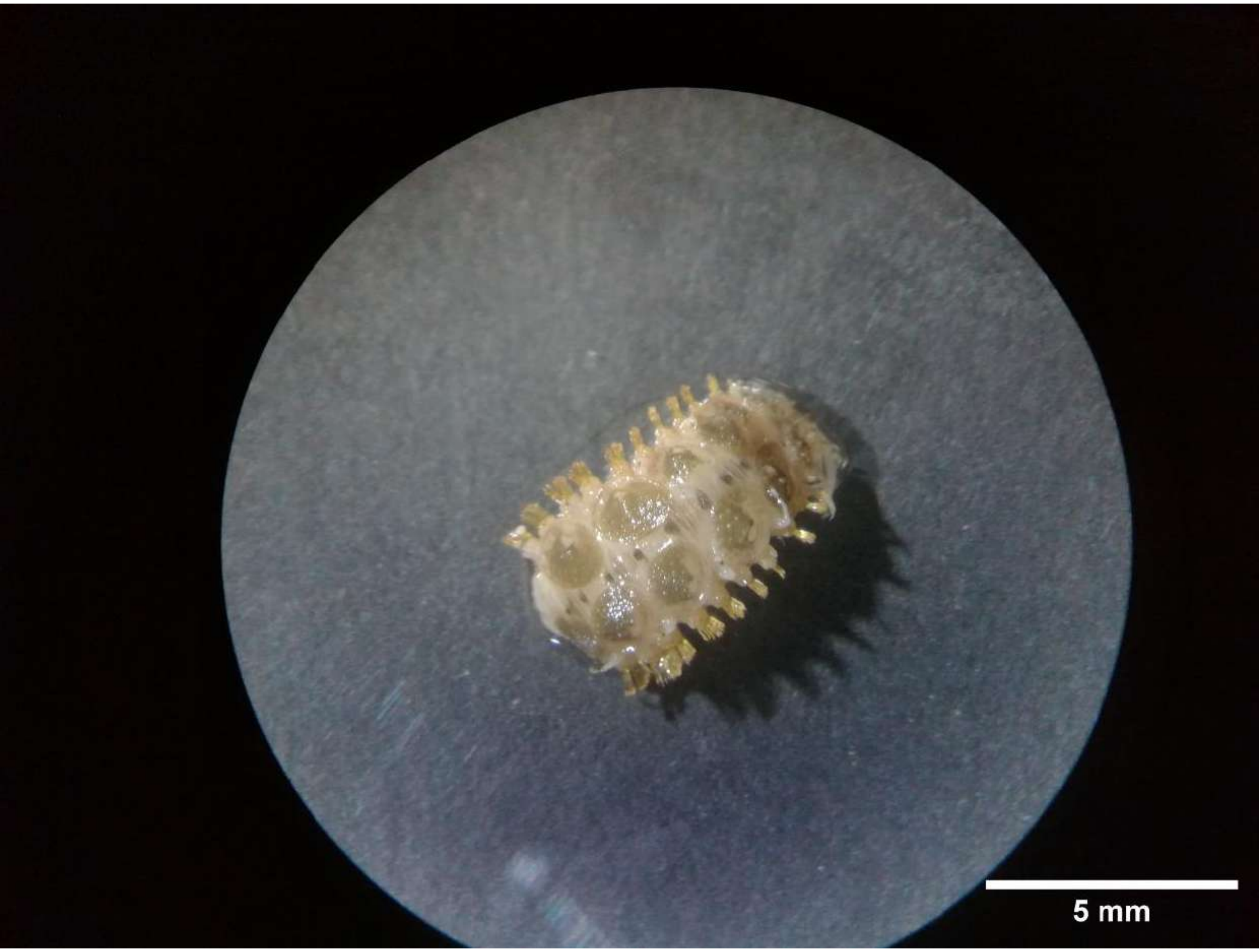
5 mm

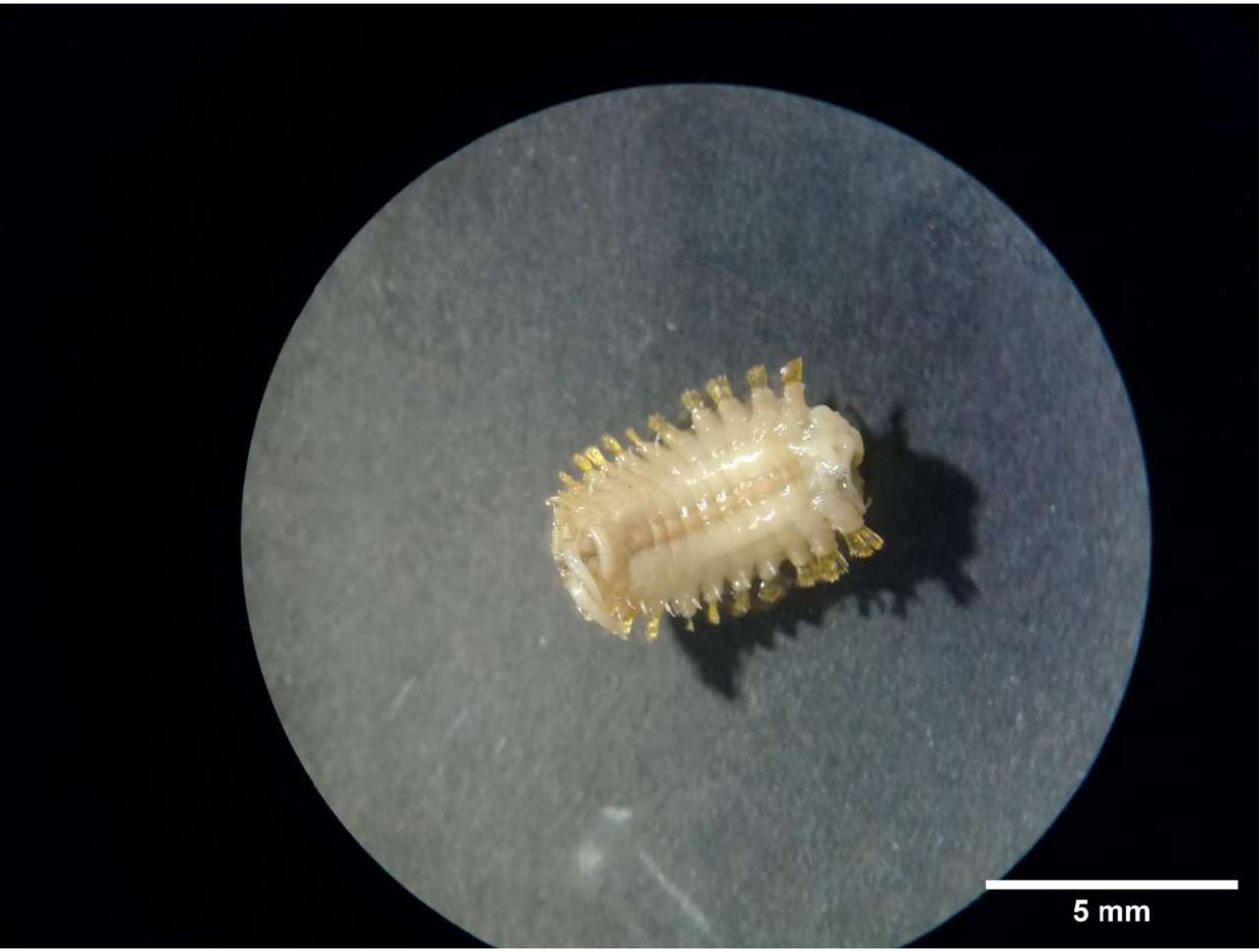
5 mm

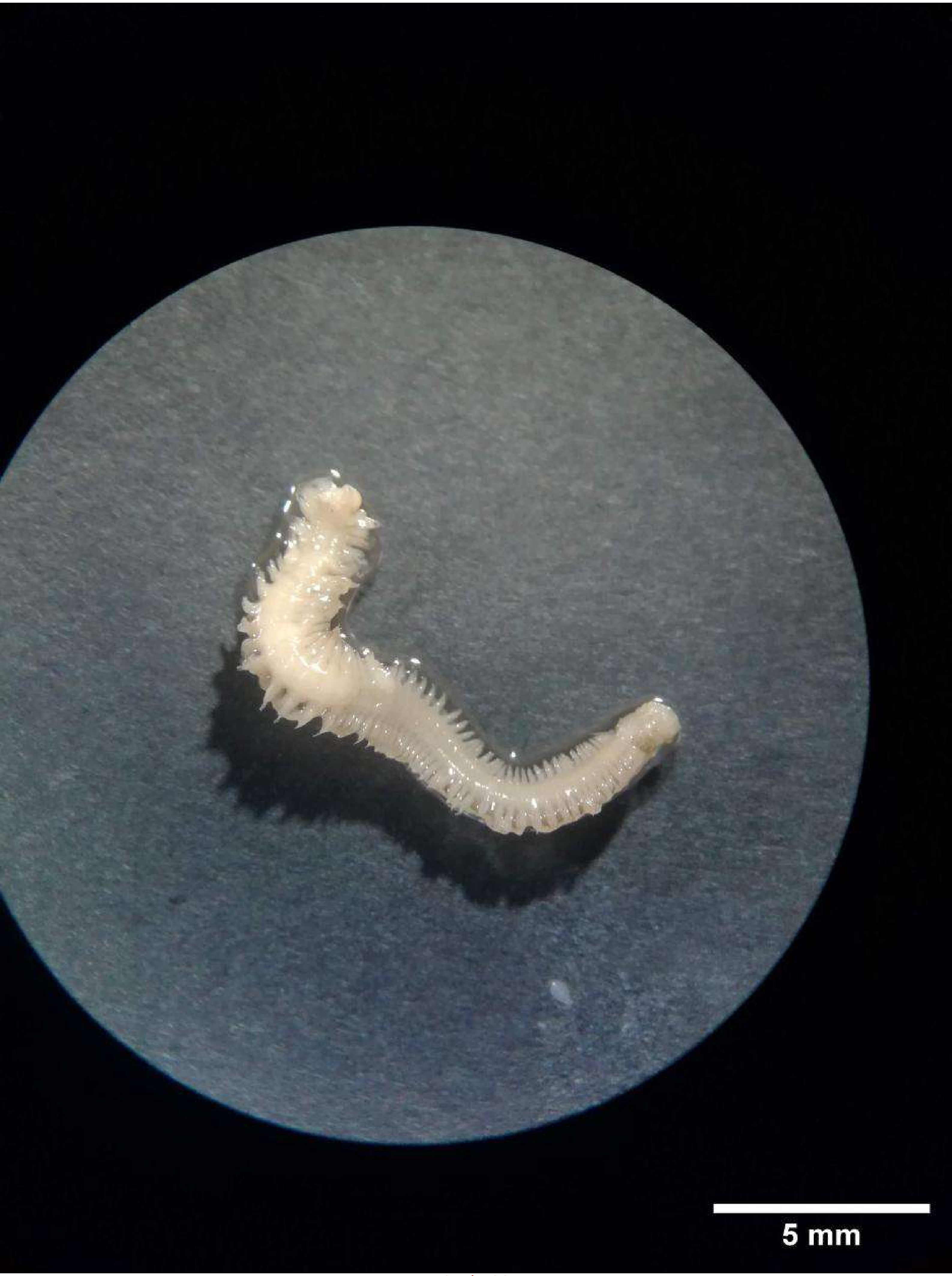
5 mm

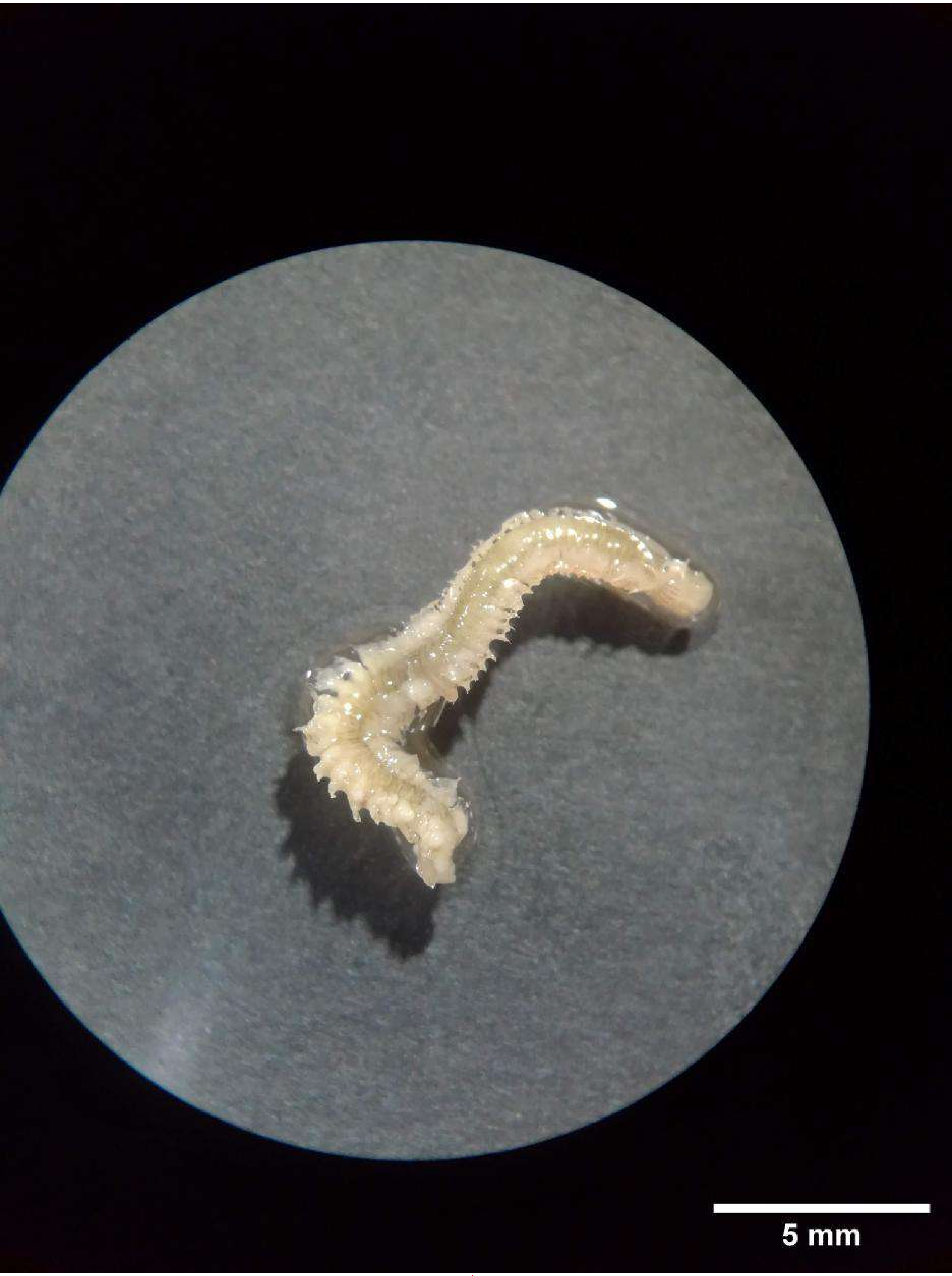
5 mm

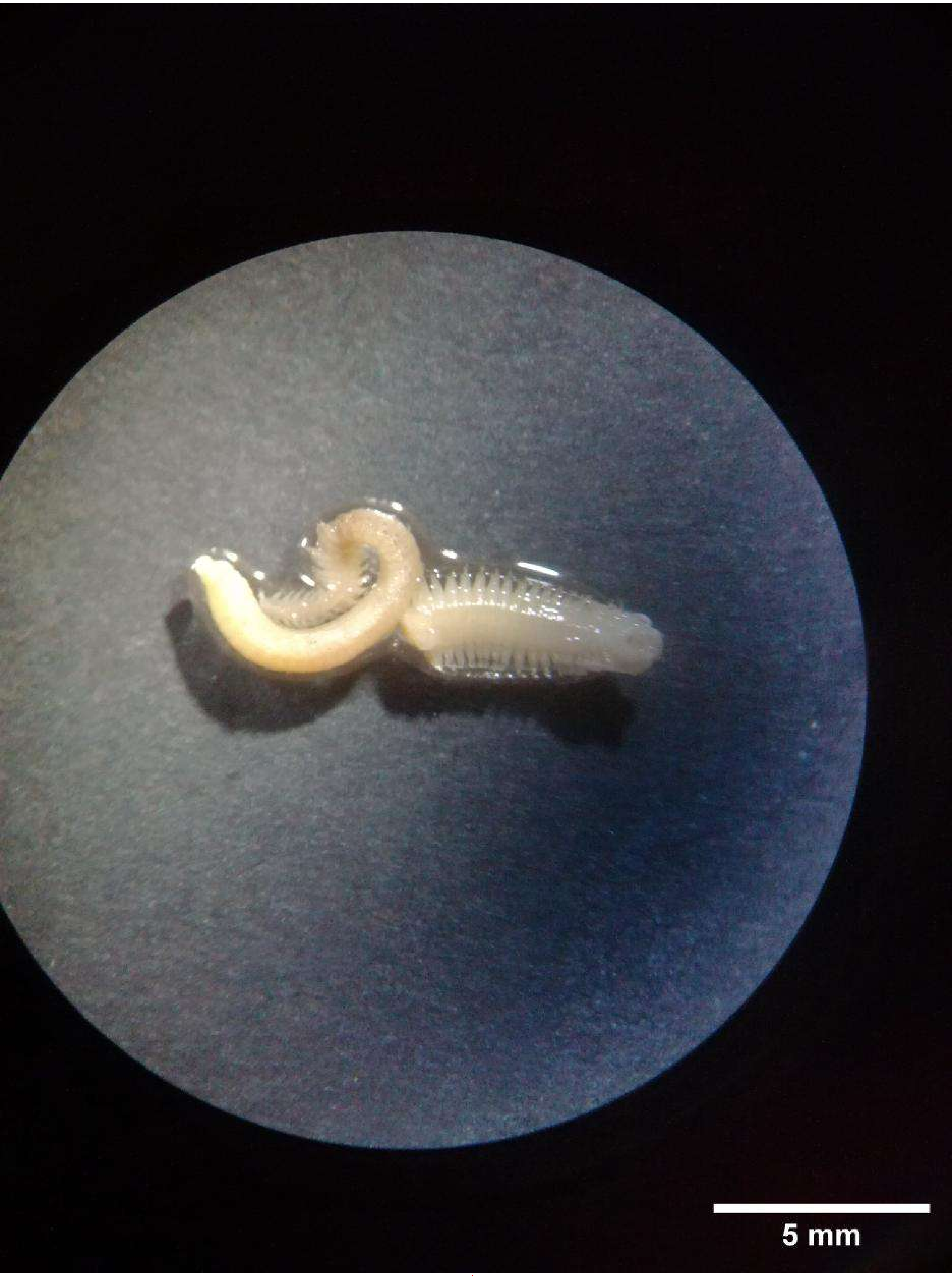
5 mm

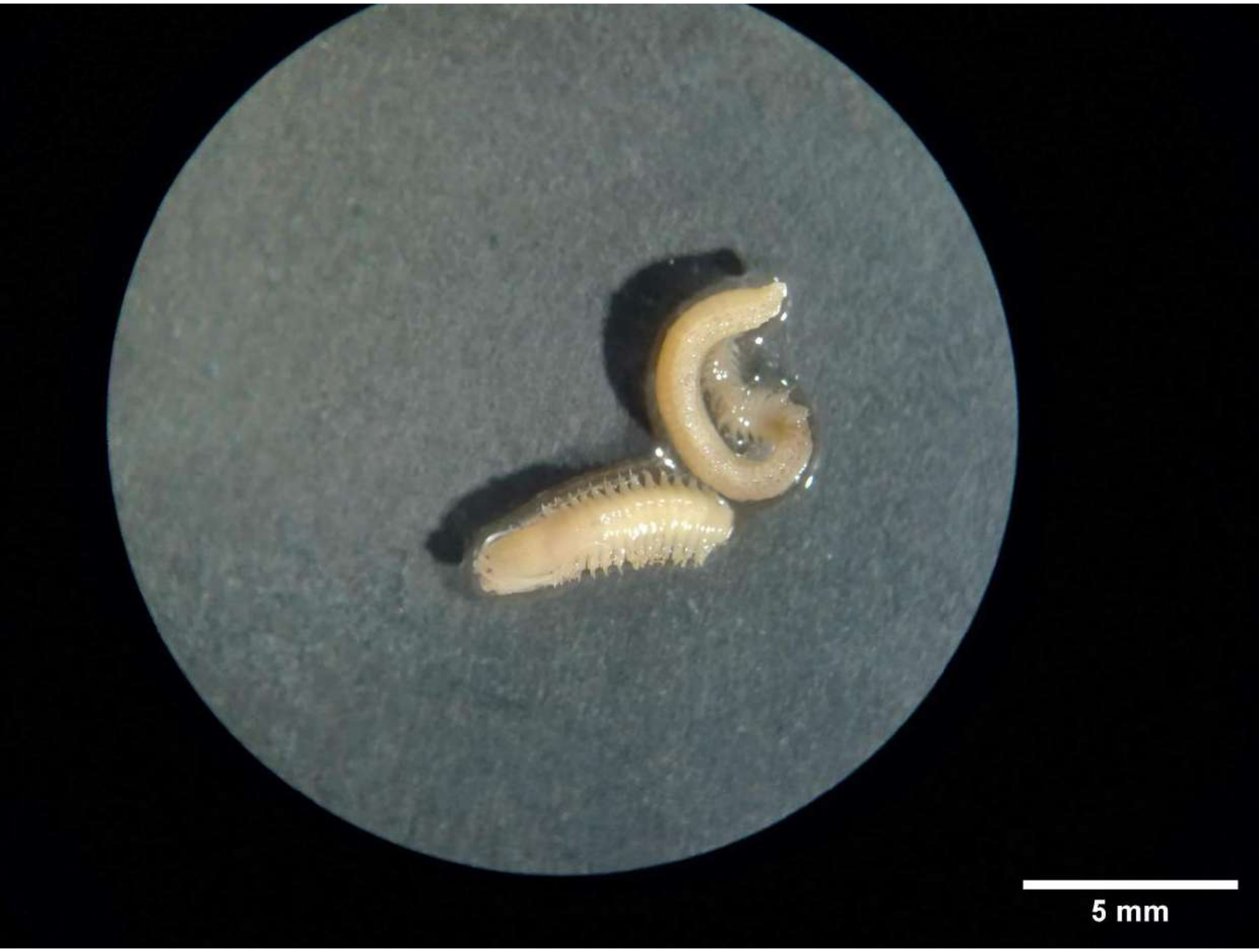
5 mm

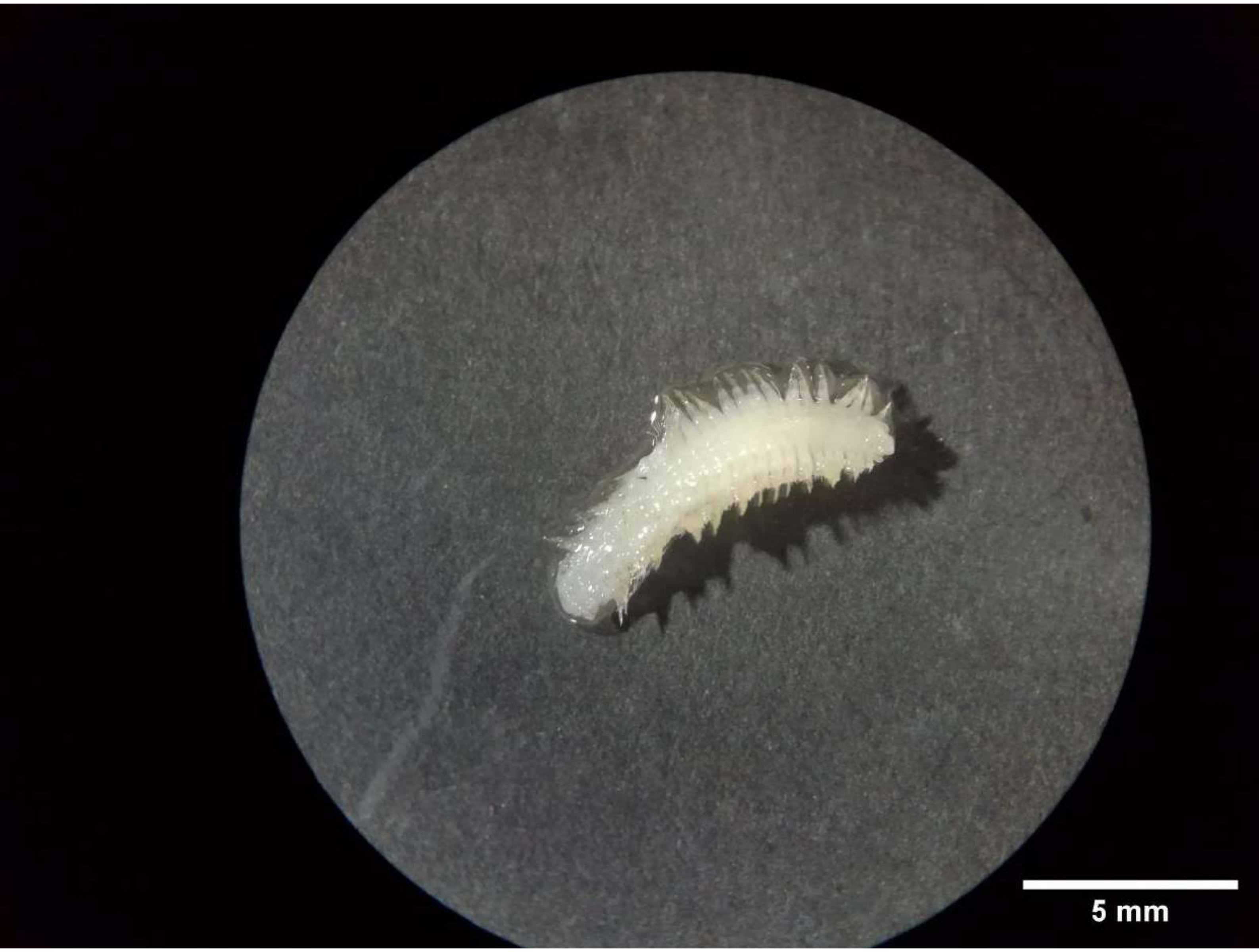
5 mm

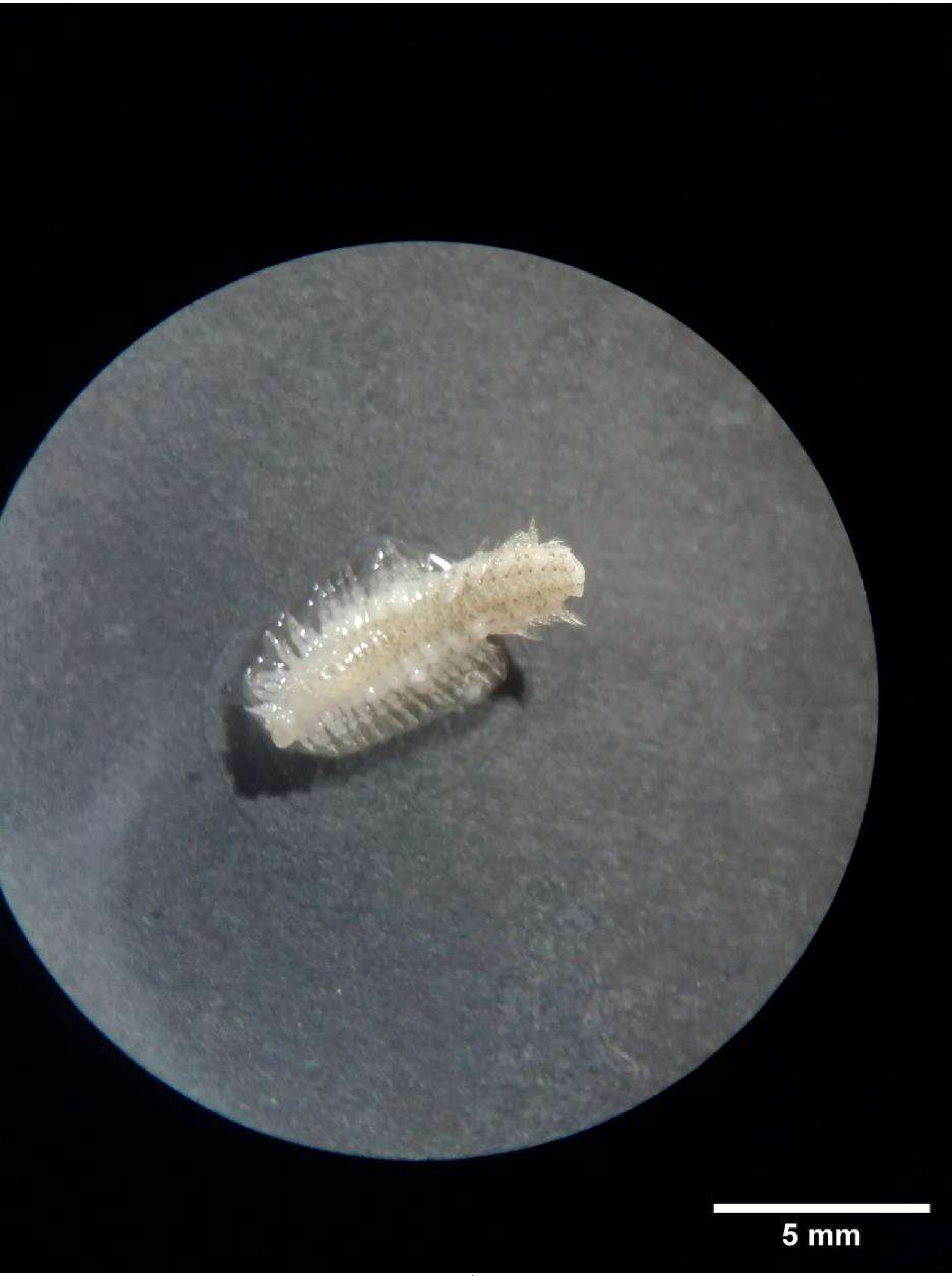
5 mm

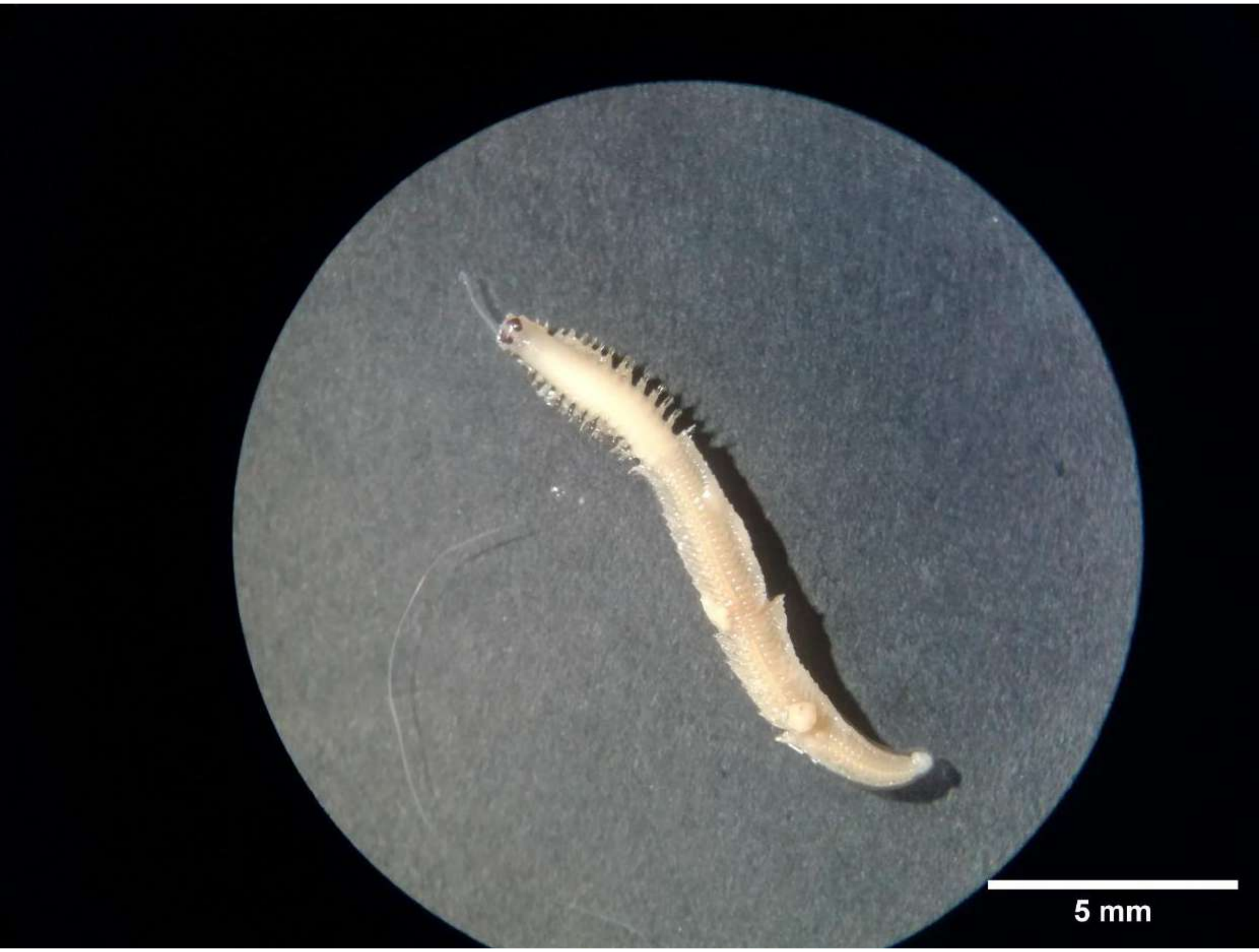
5 mm

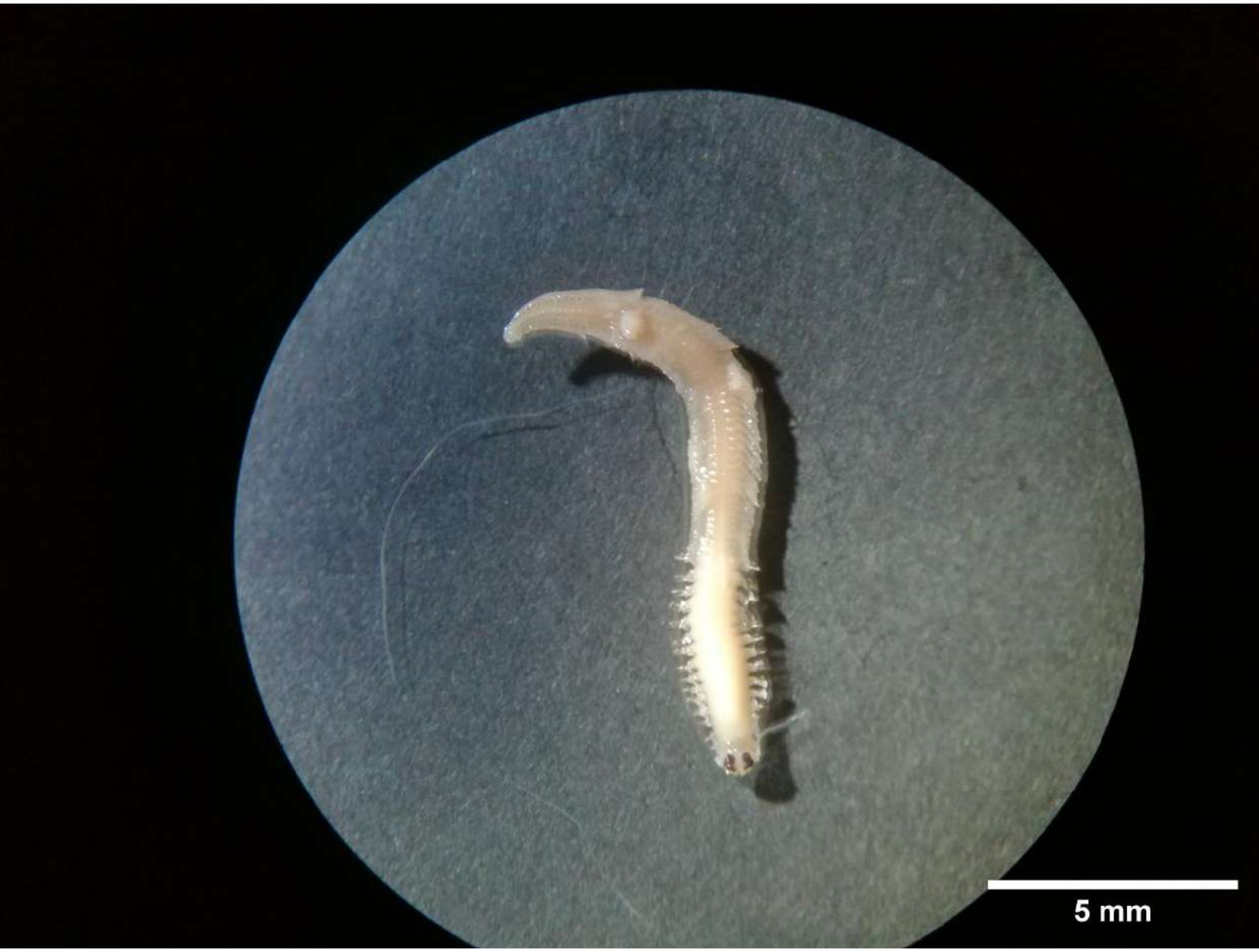
5 mm

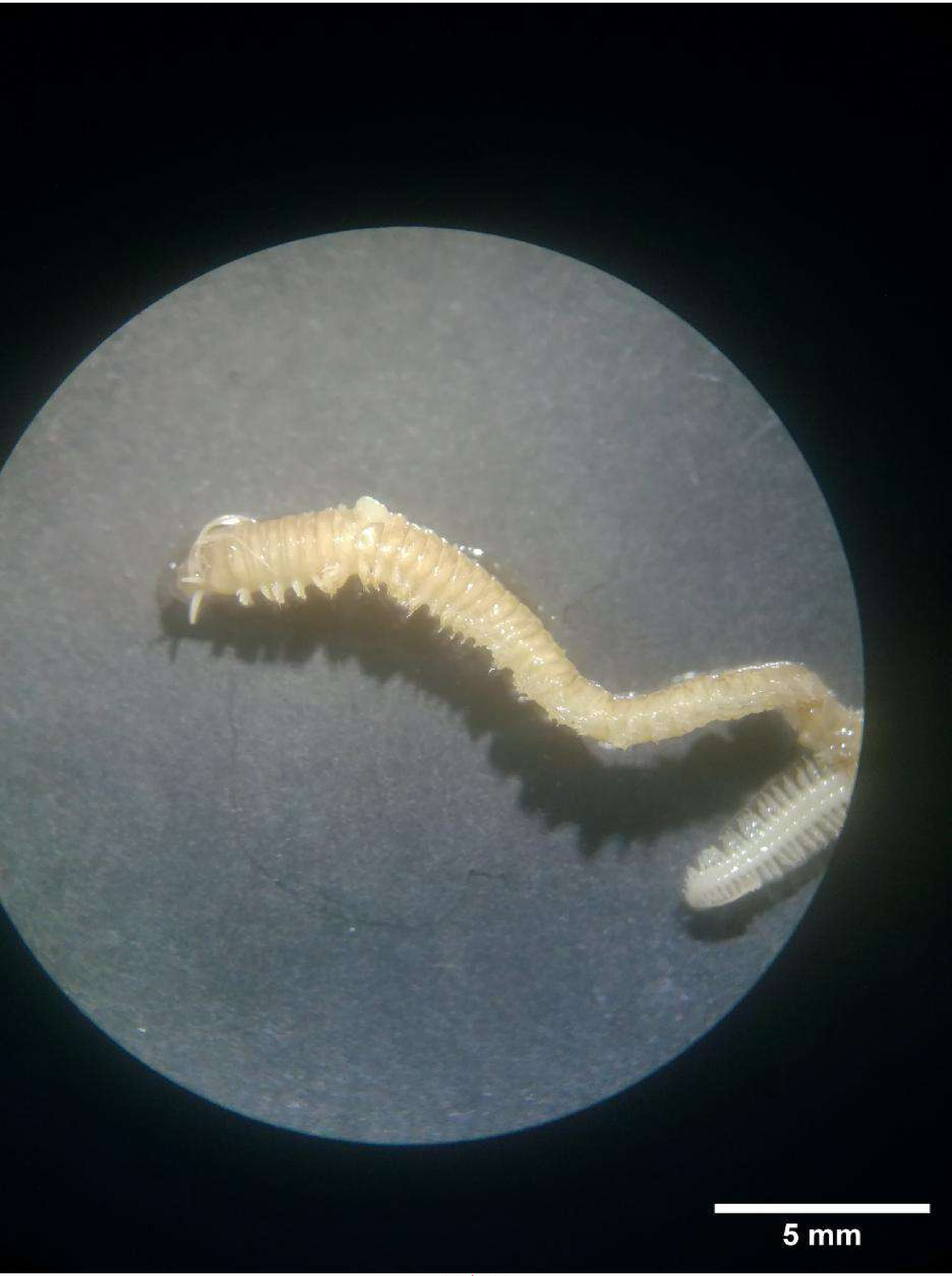
5 mm

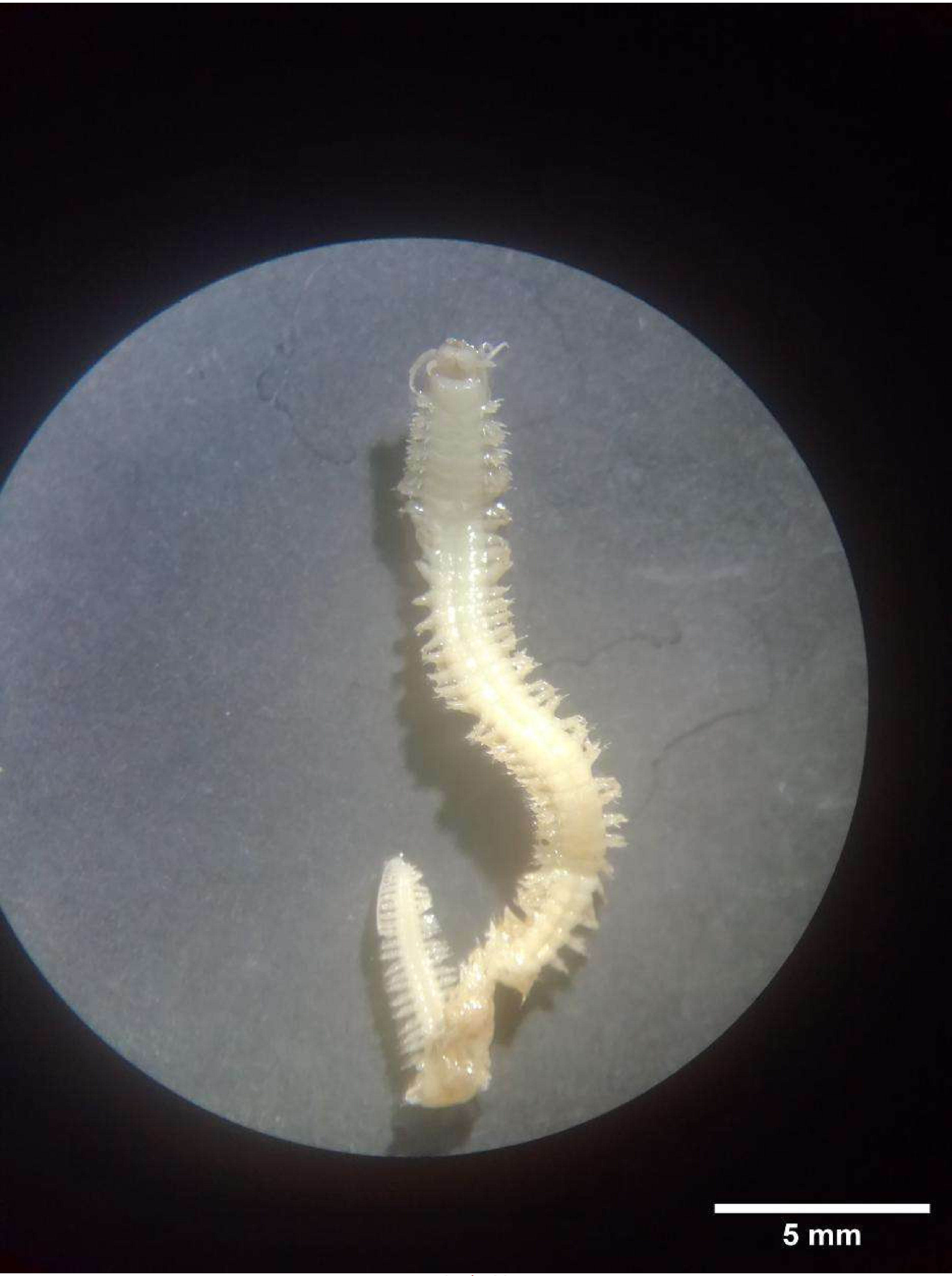
5 mm

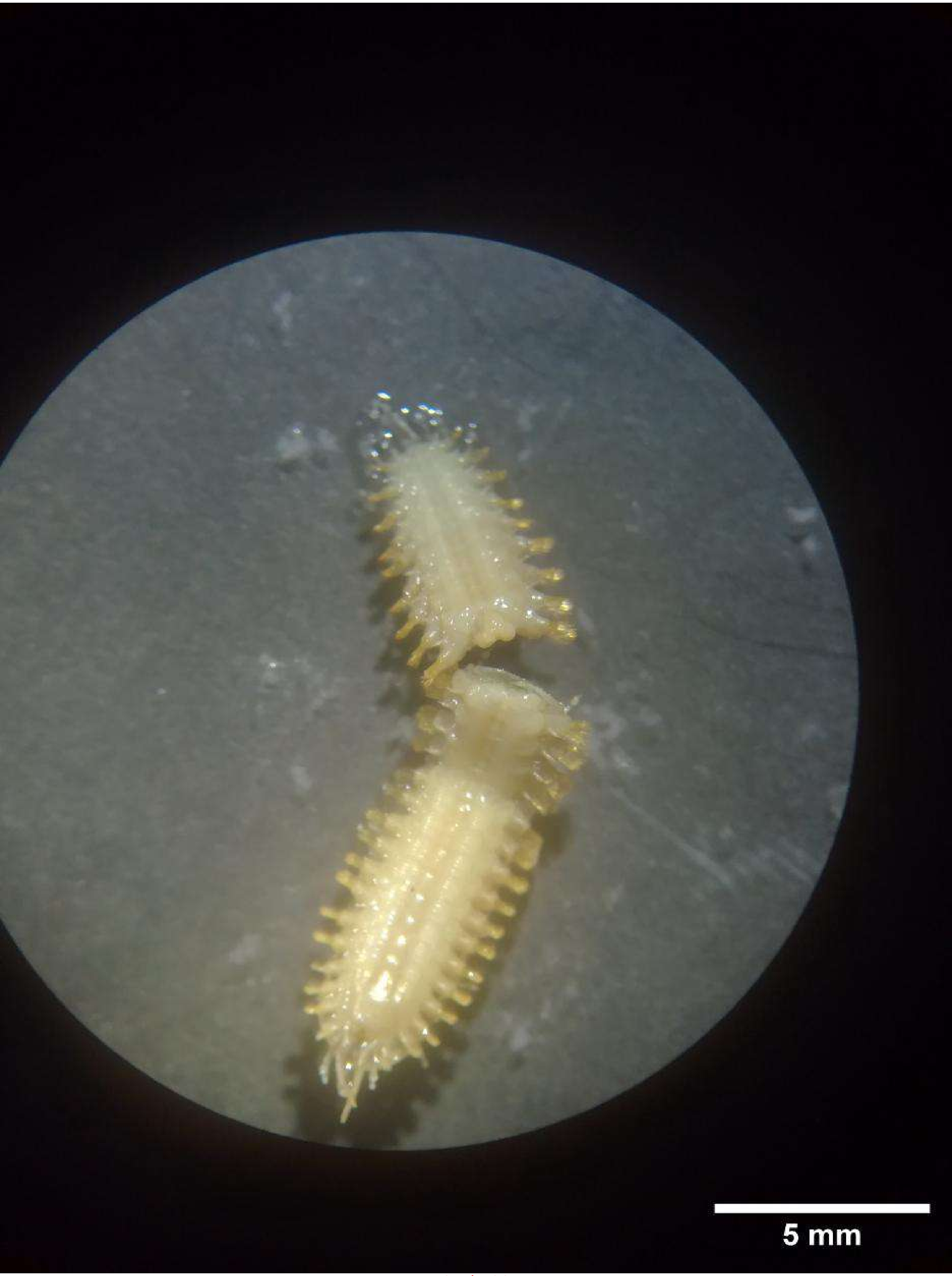
5 mm

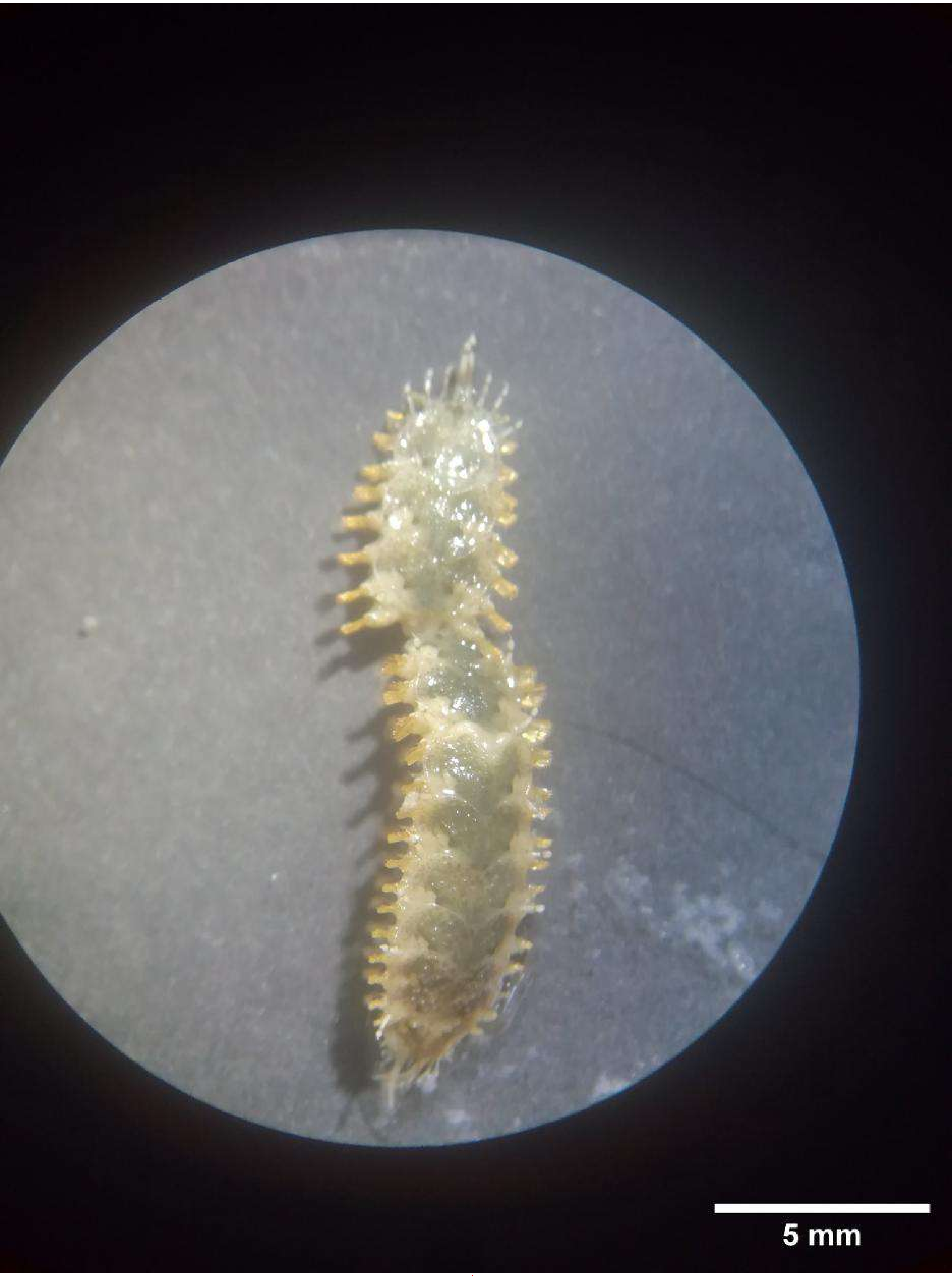
5 mm

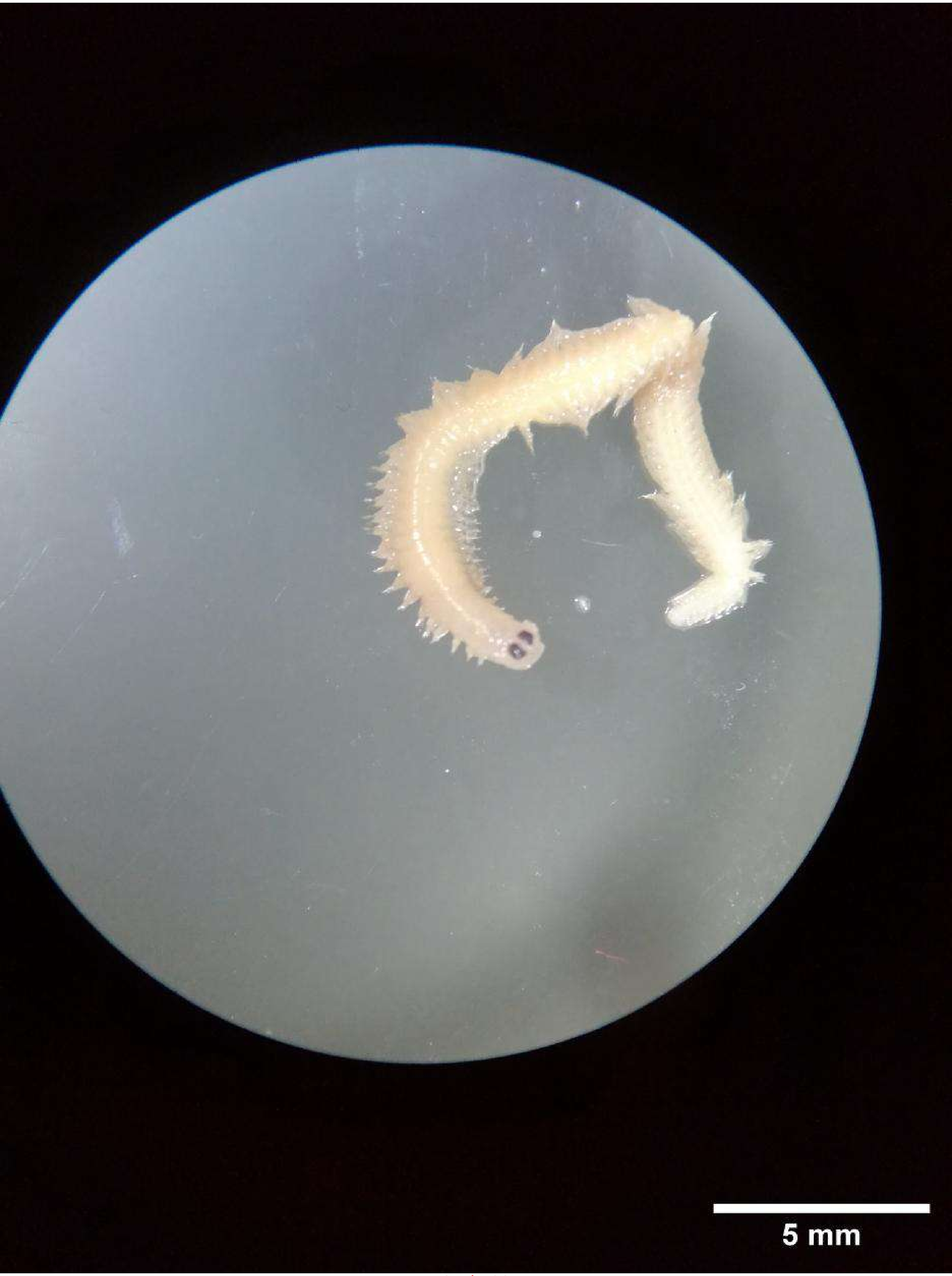
5 mm

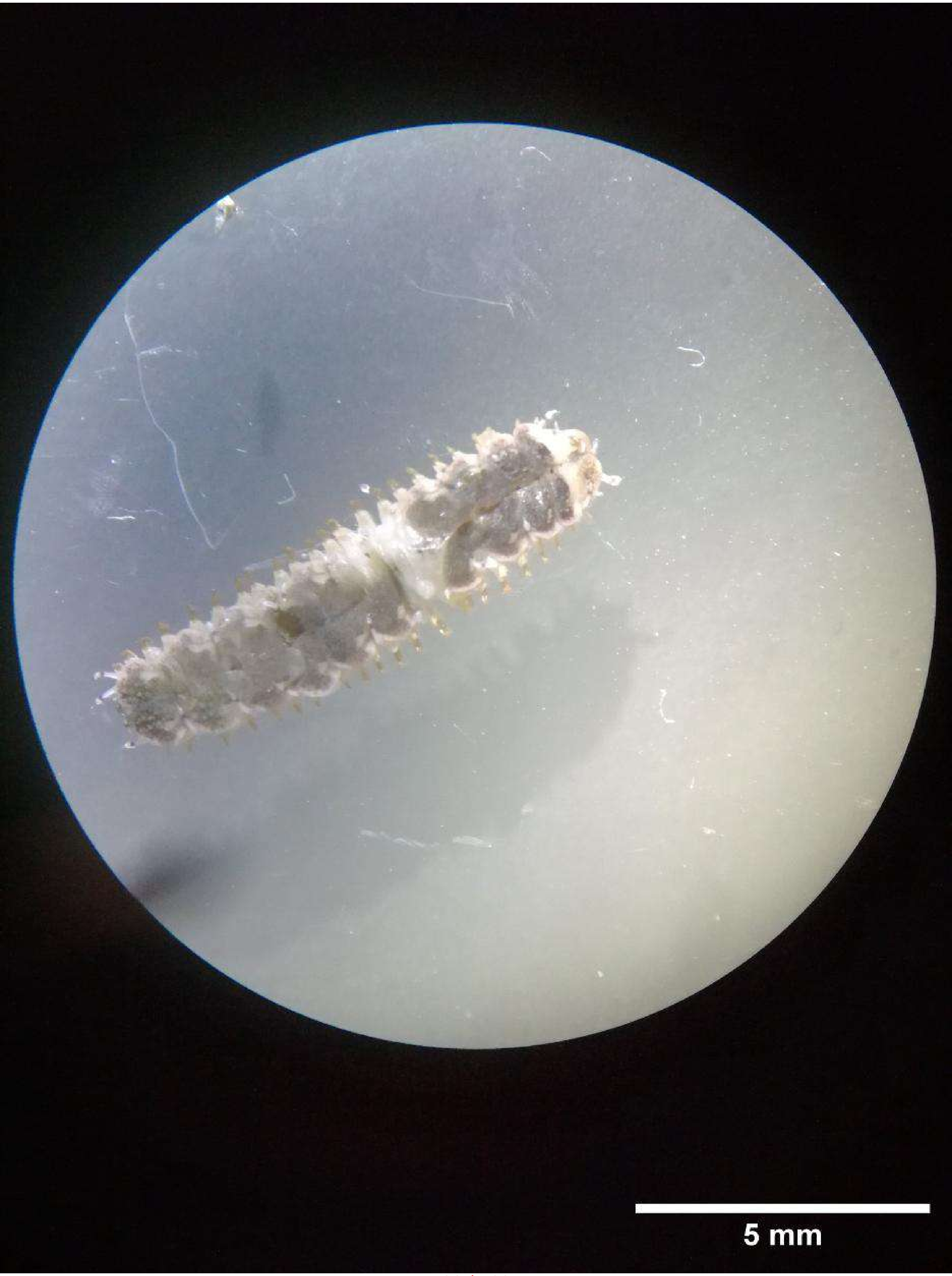
5 mm

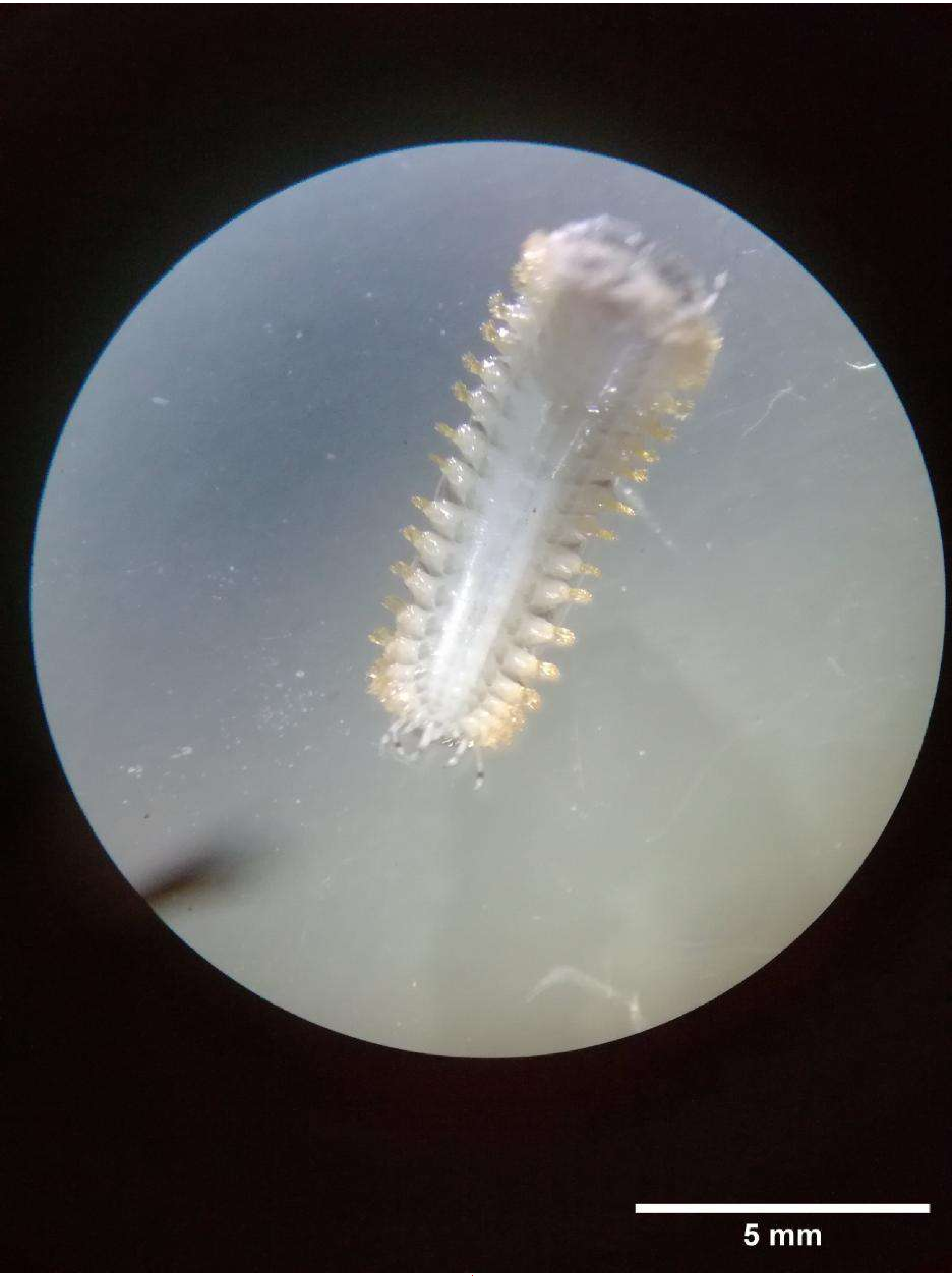
5 mm

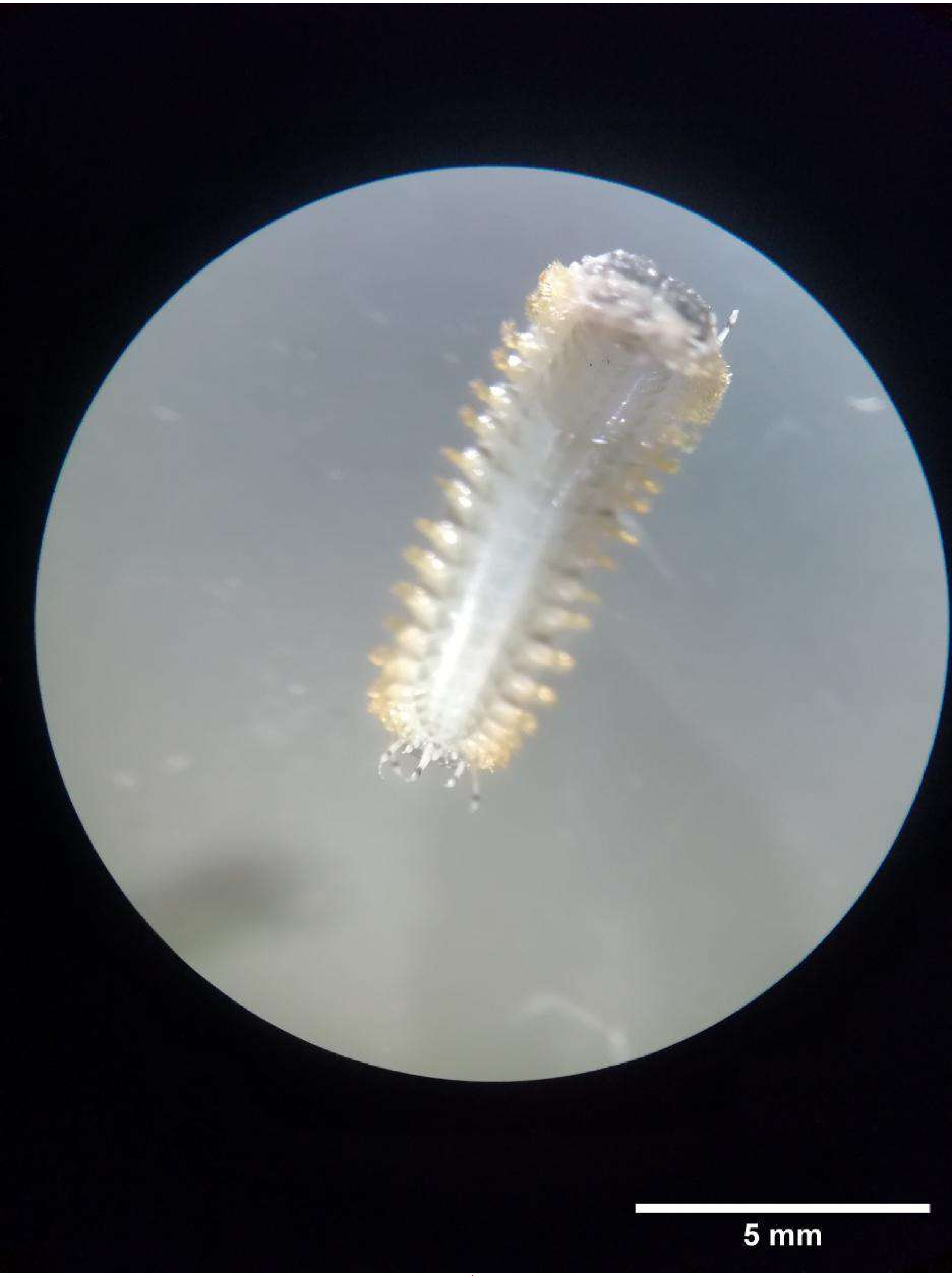
5 mm

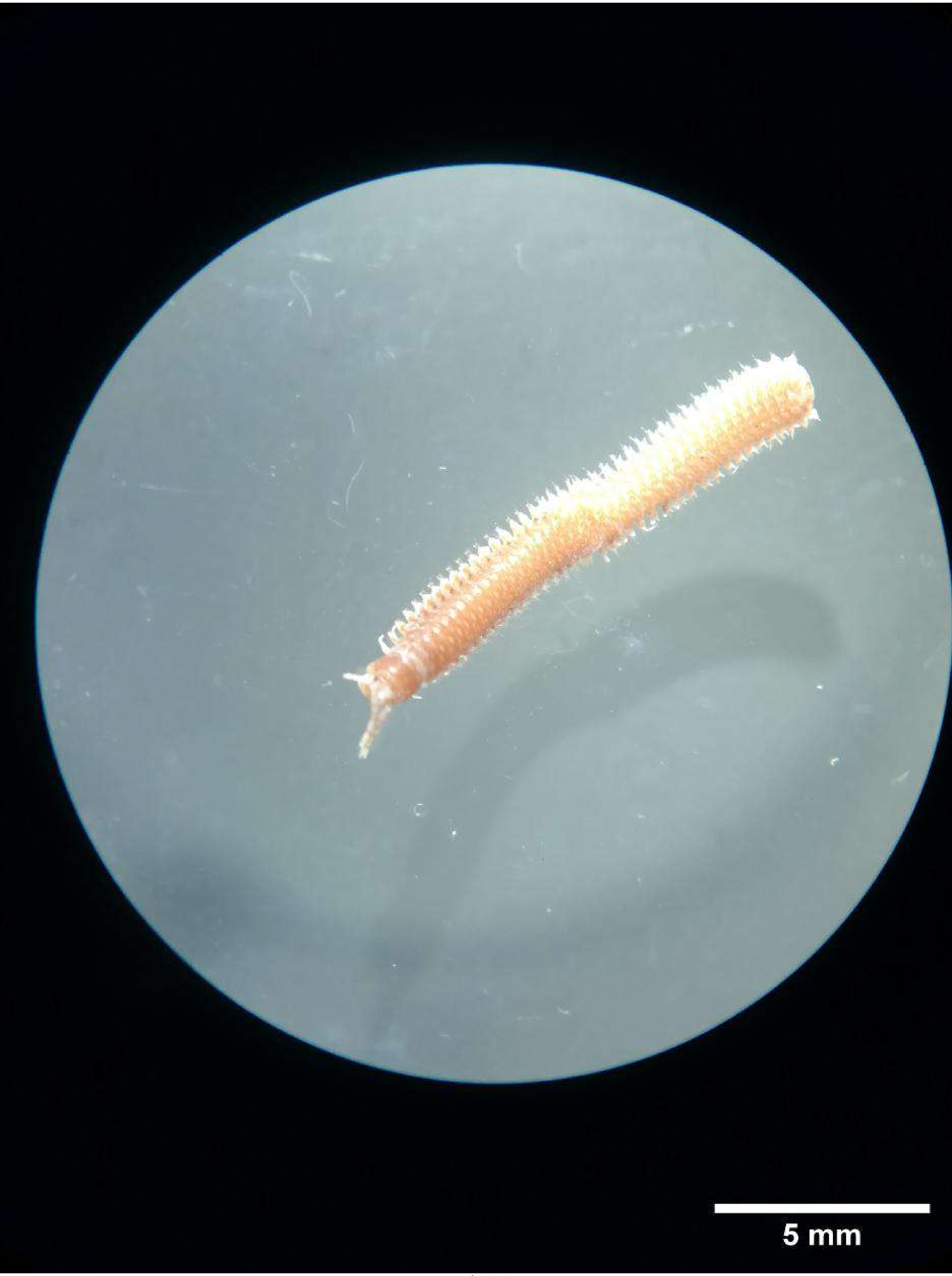
5 mm

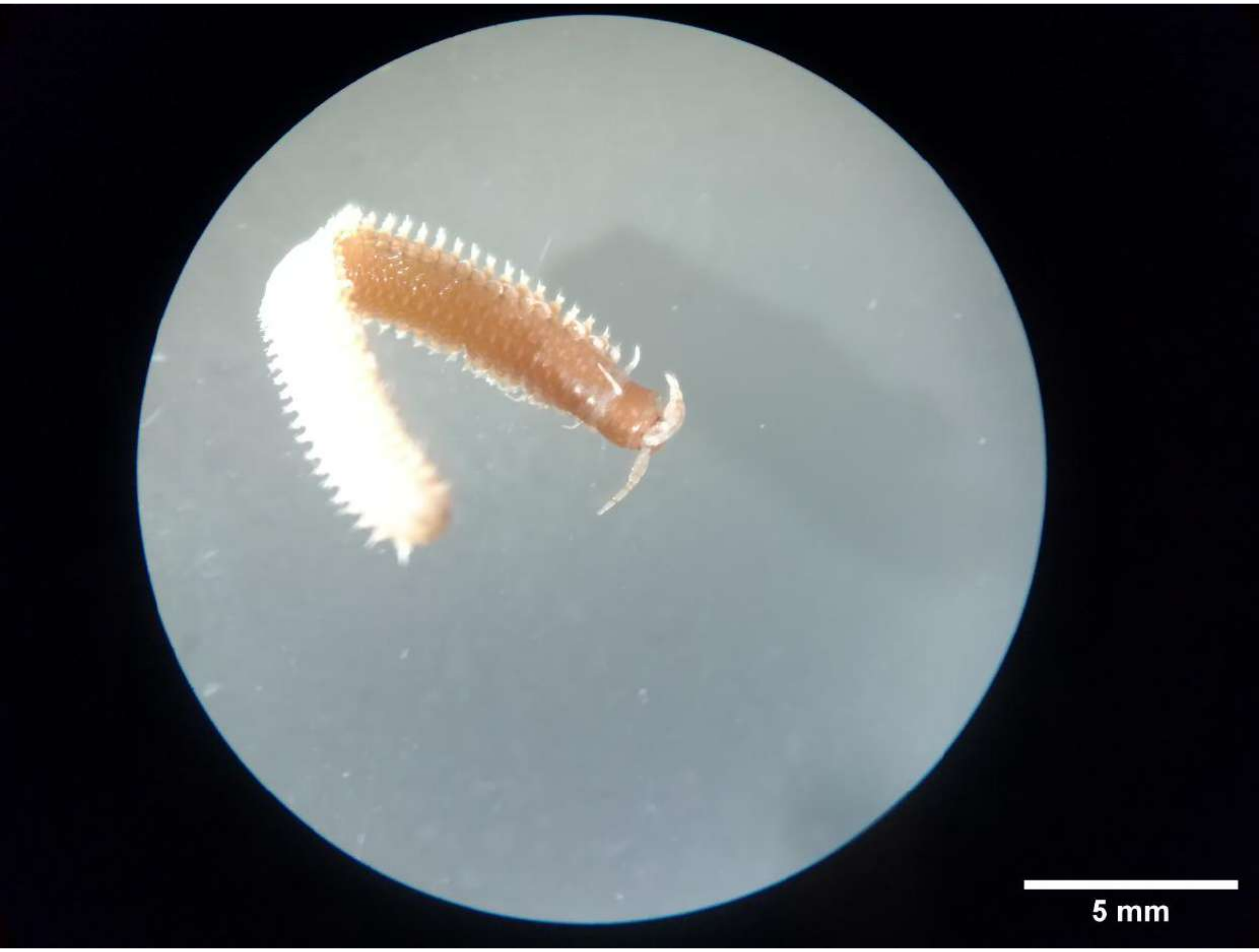
5 mm

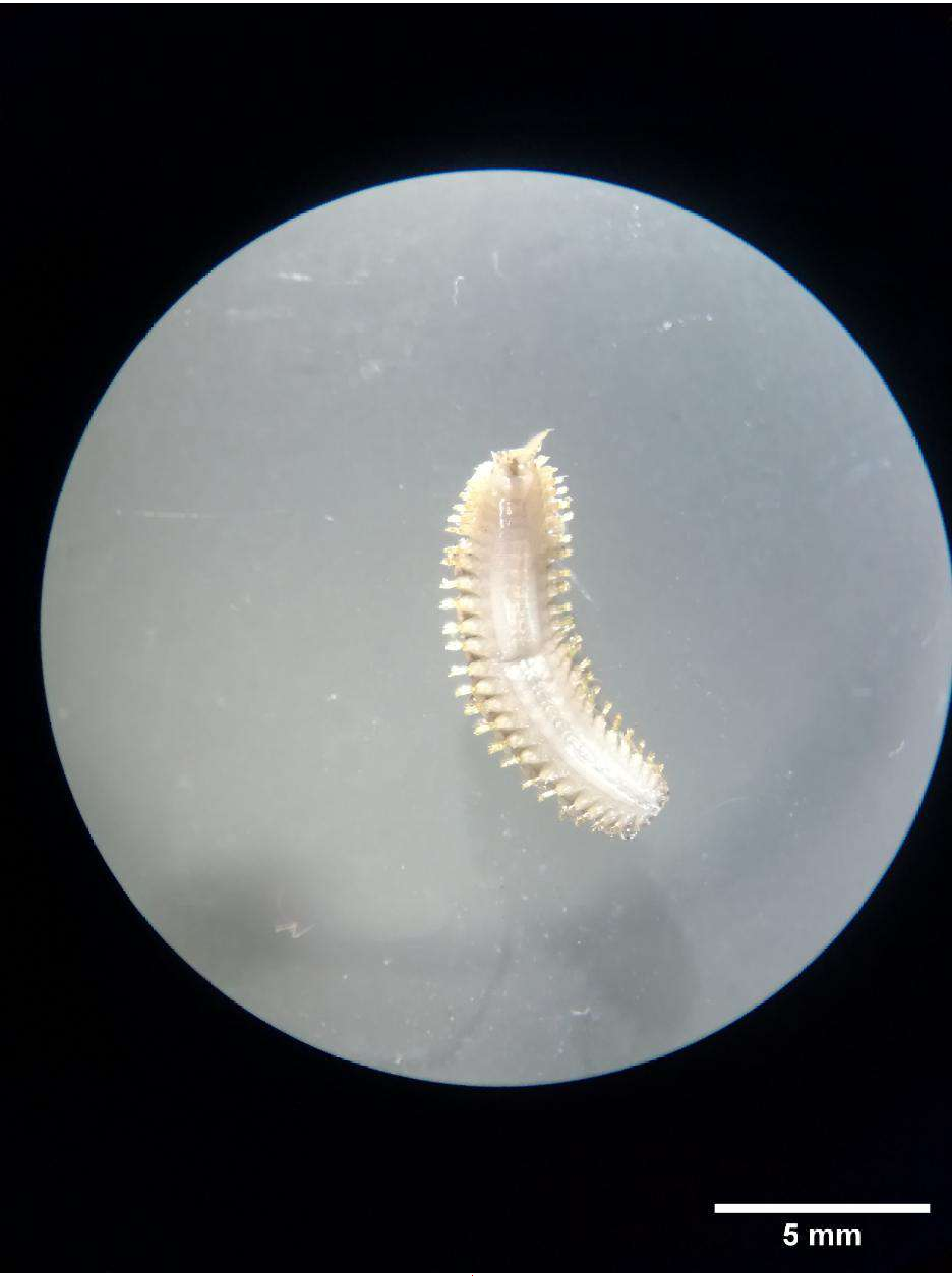
5 mm

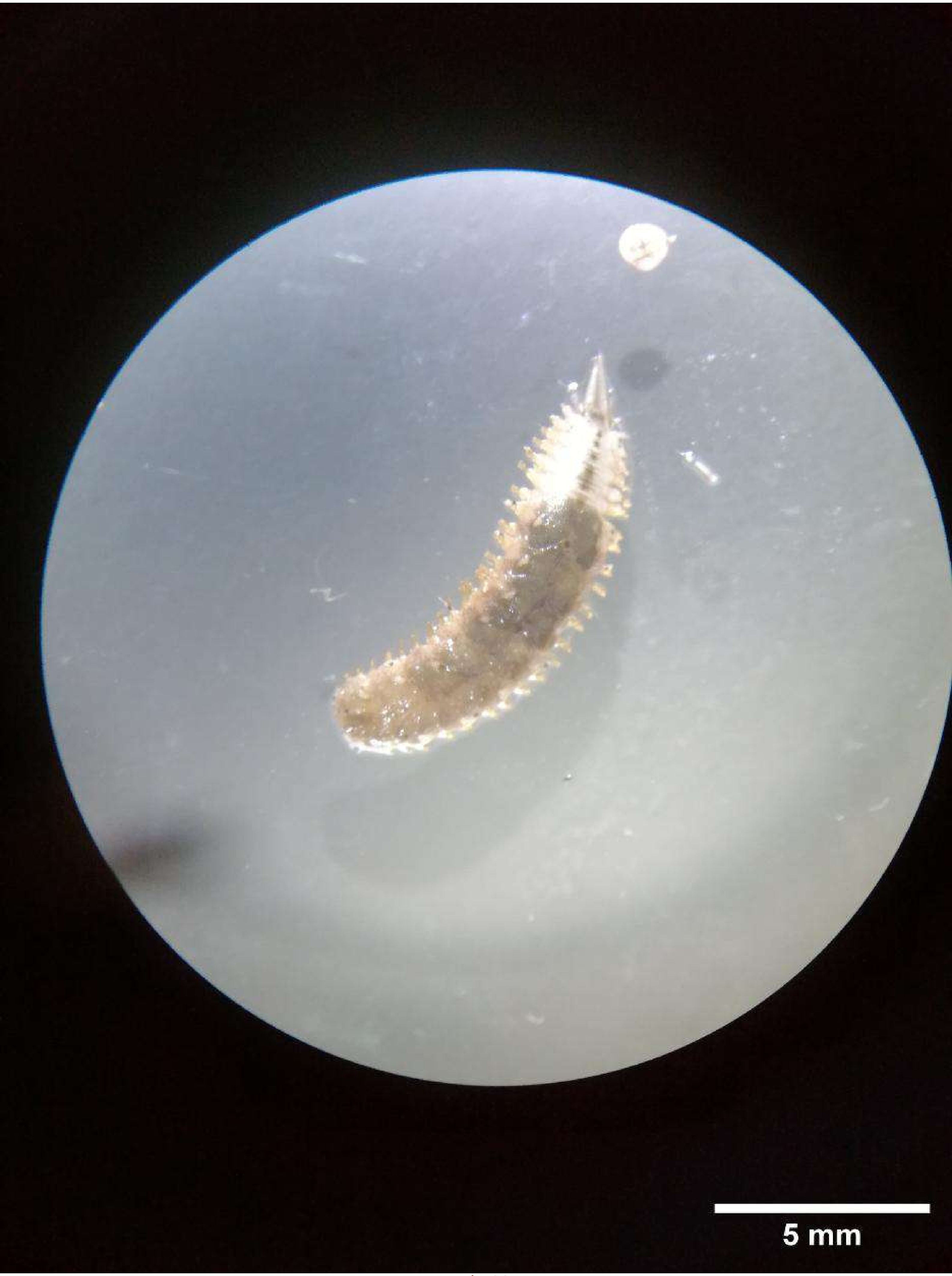
5 mm

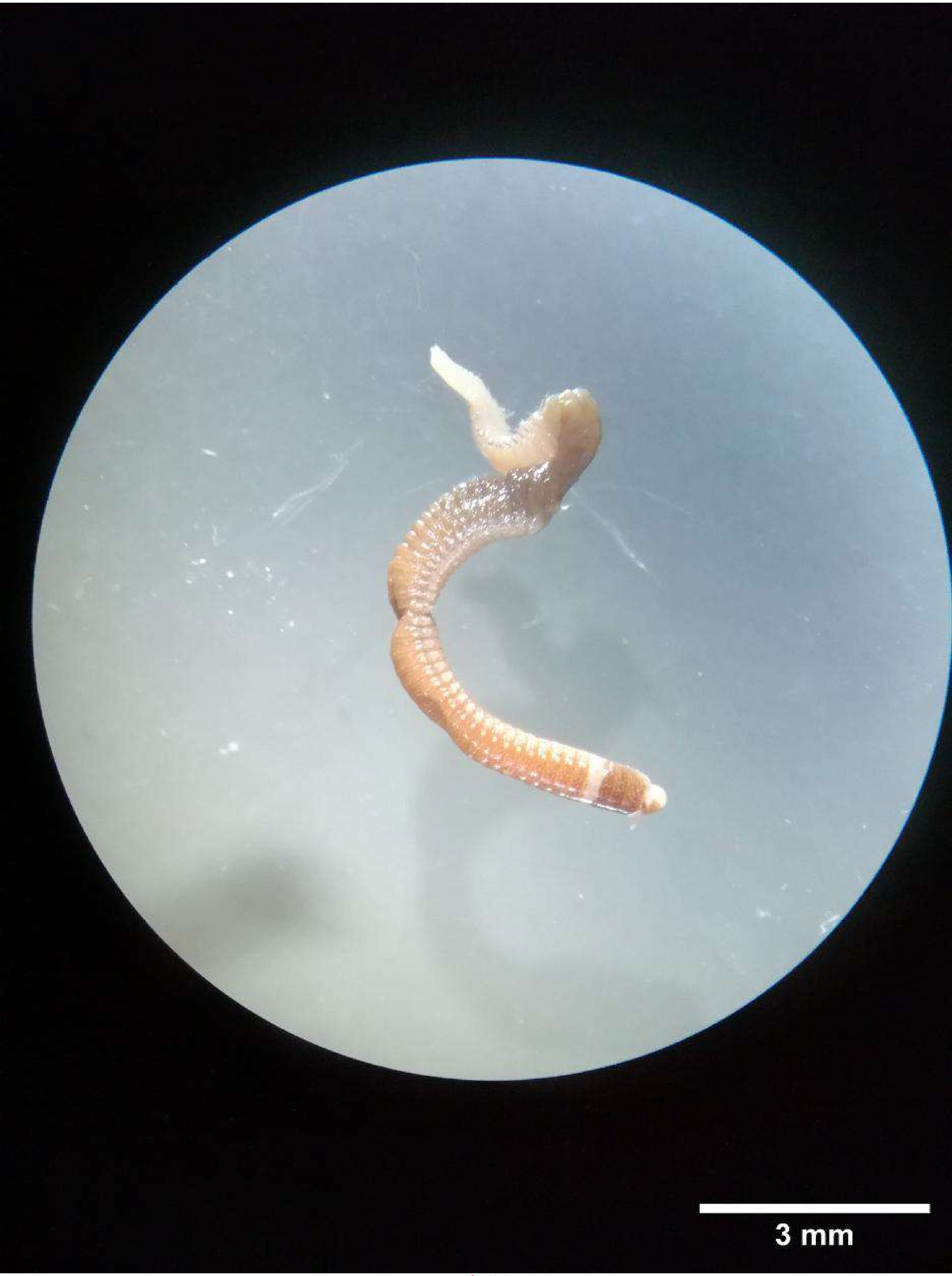
3 mm

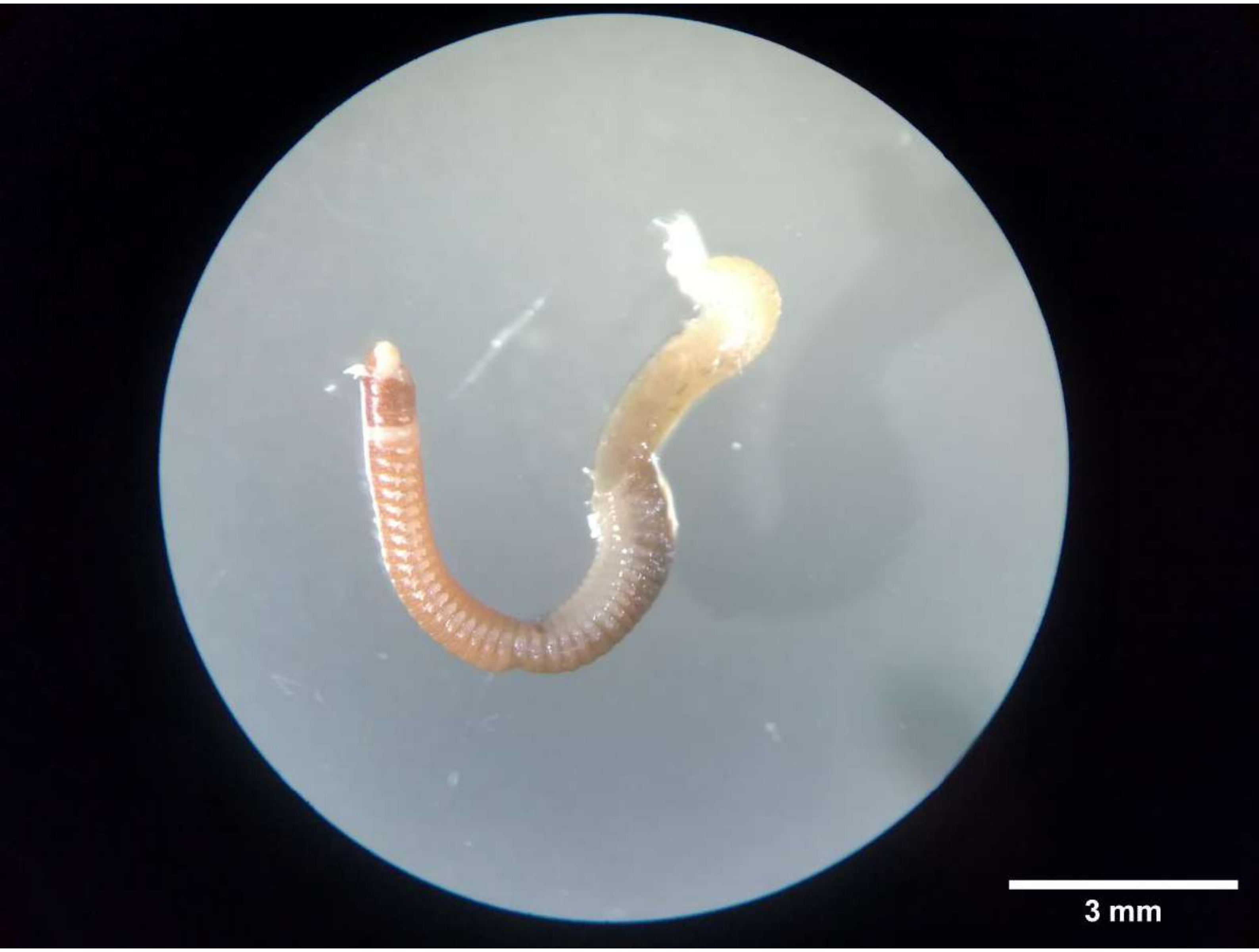
3 mm

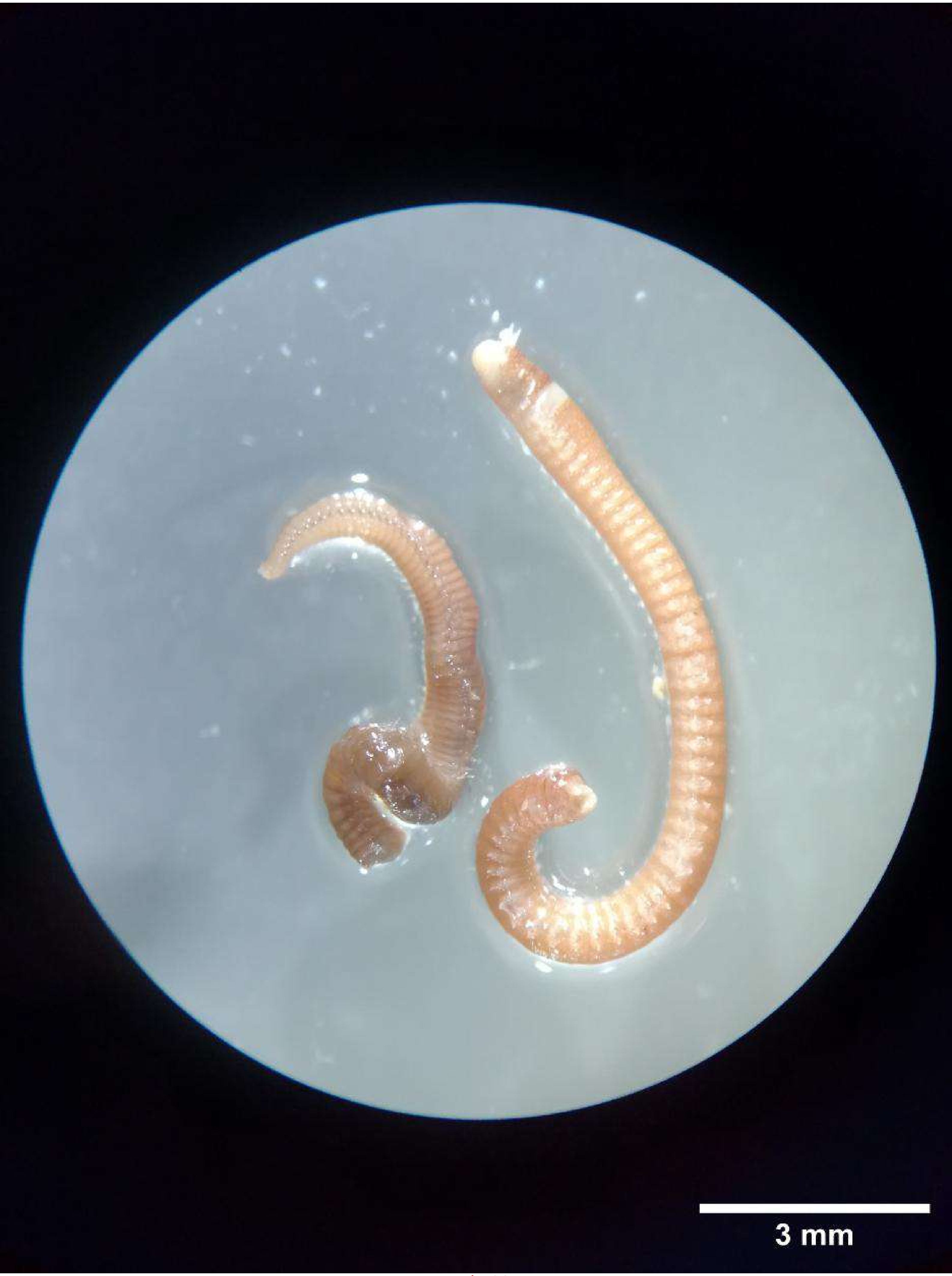
3 mm

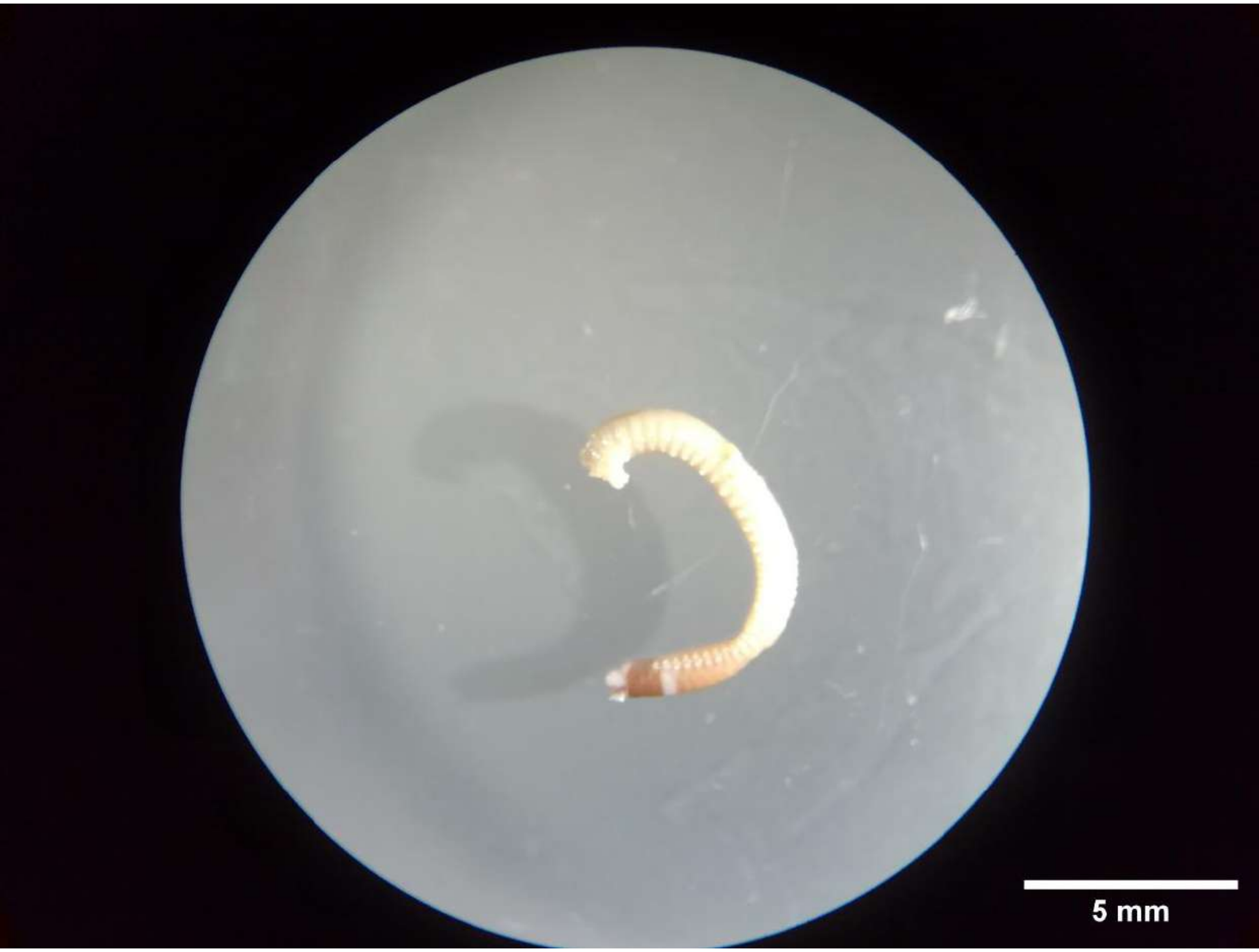
5 mm

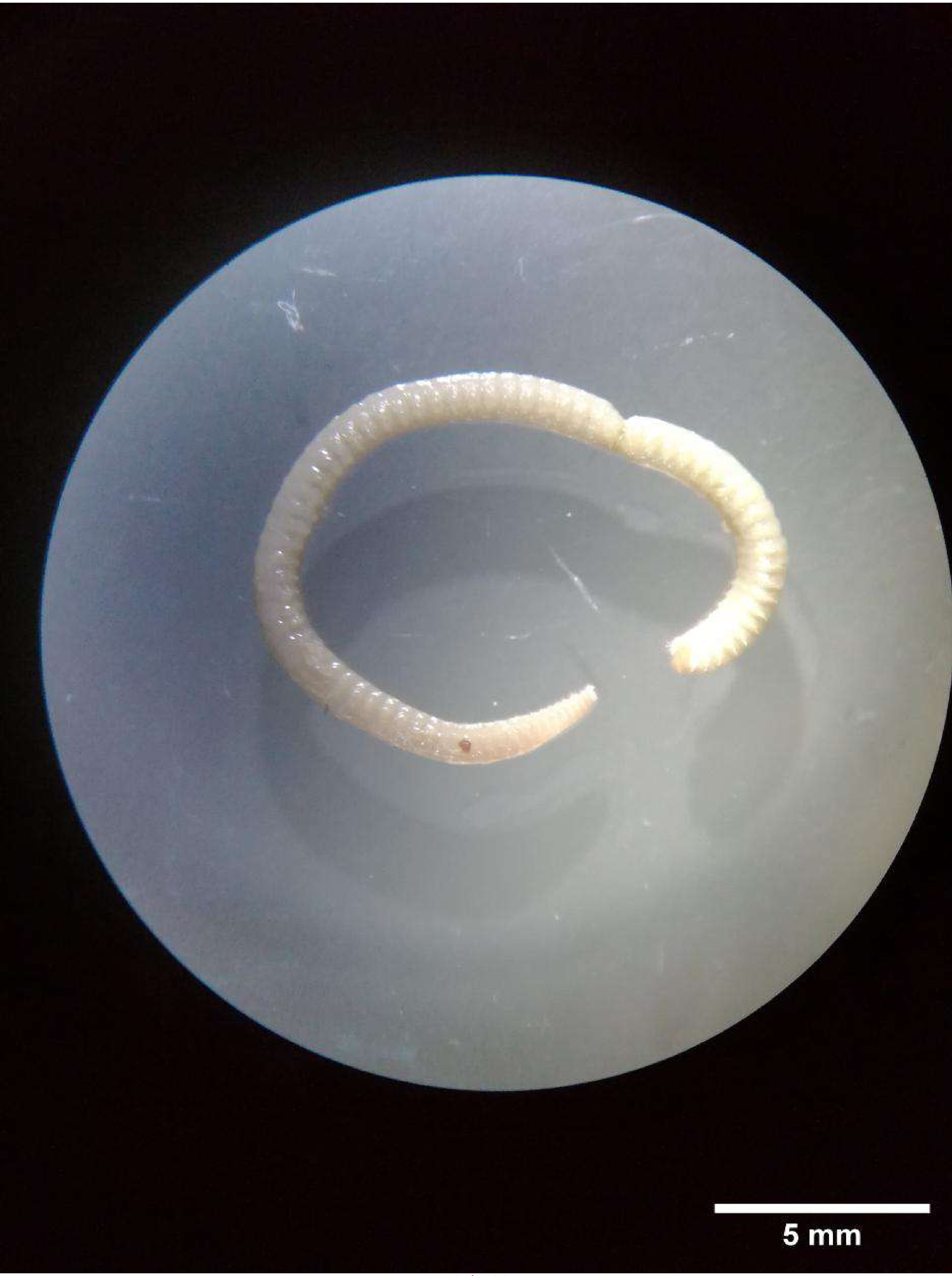
5 mm

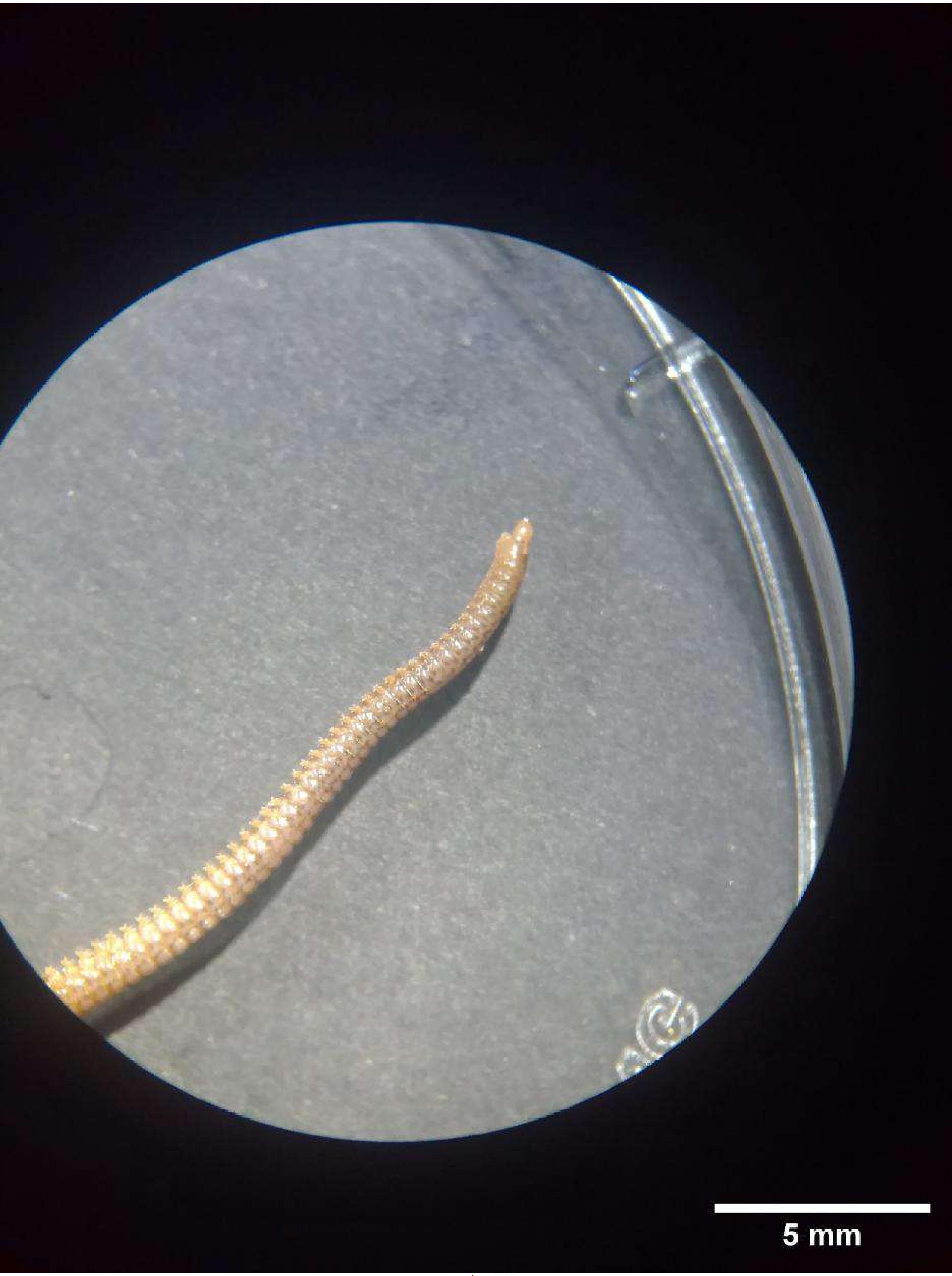
5 mm

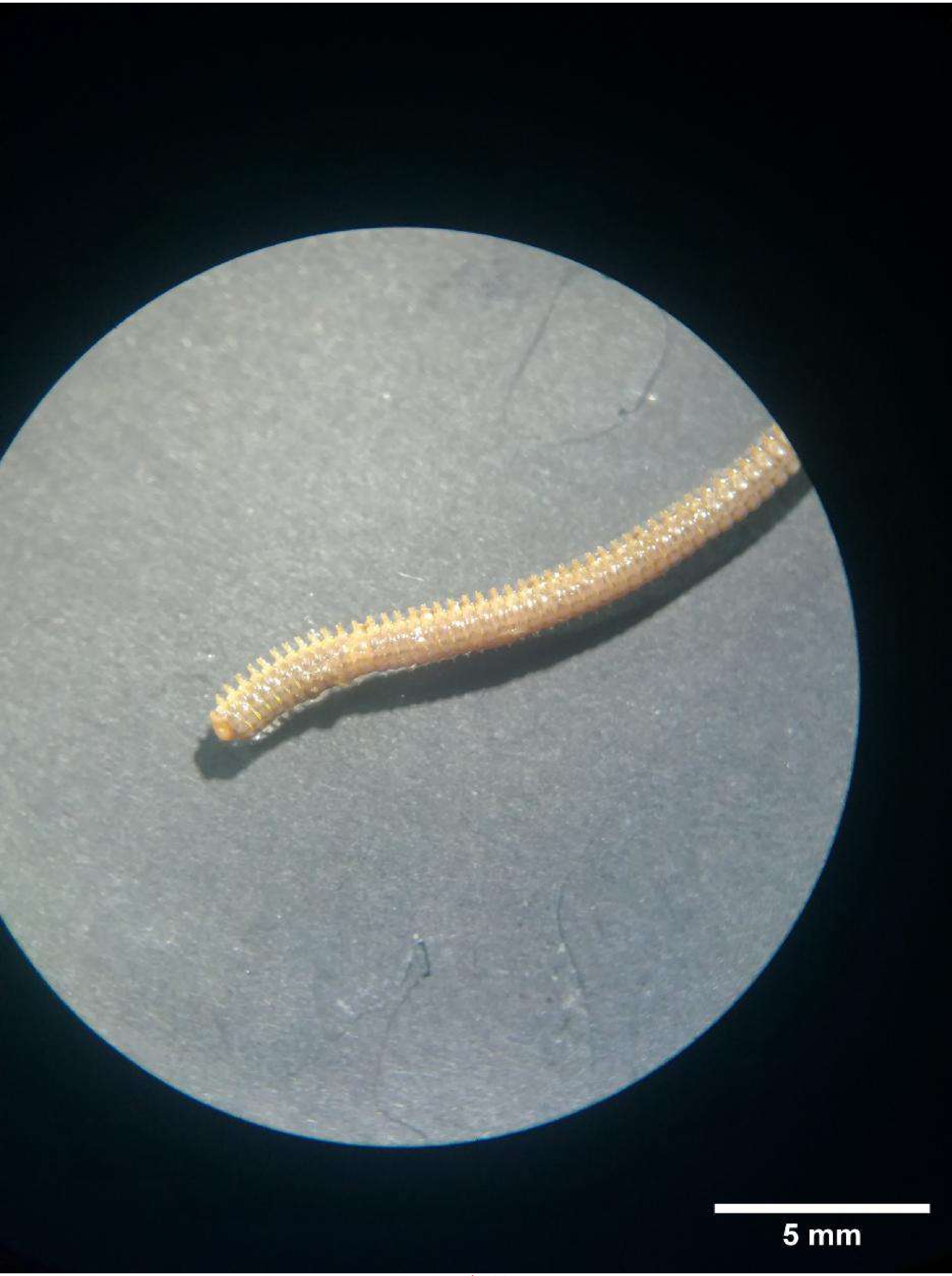
5 mm

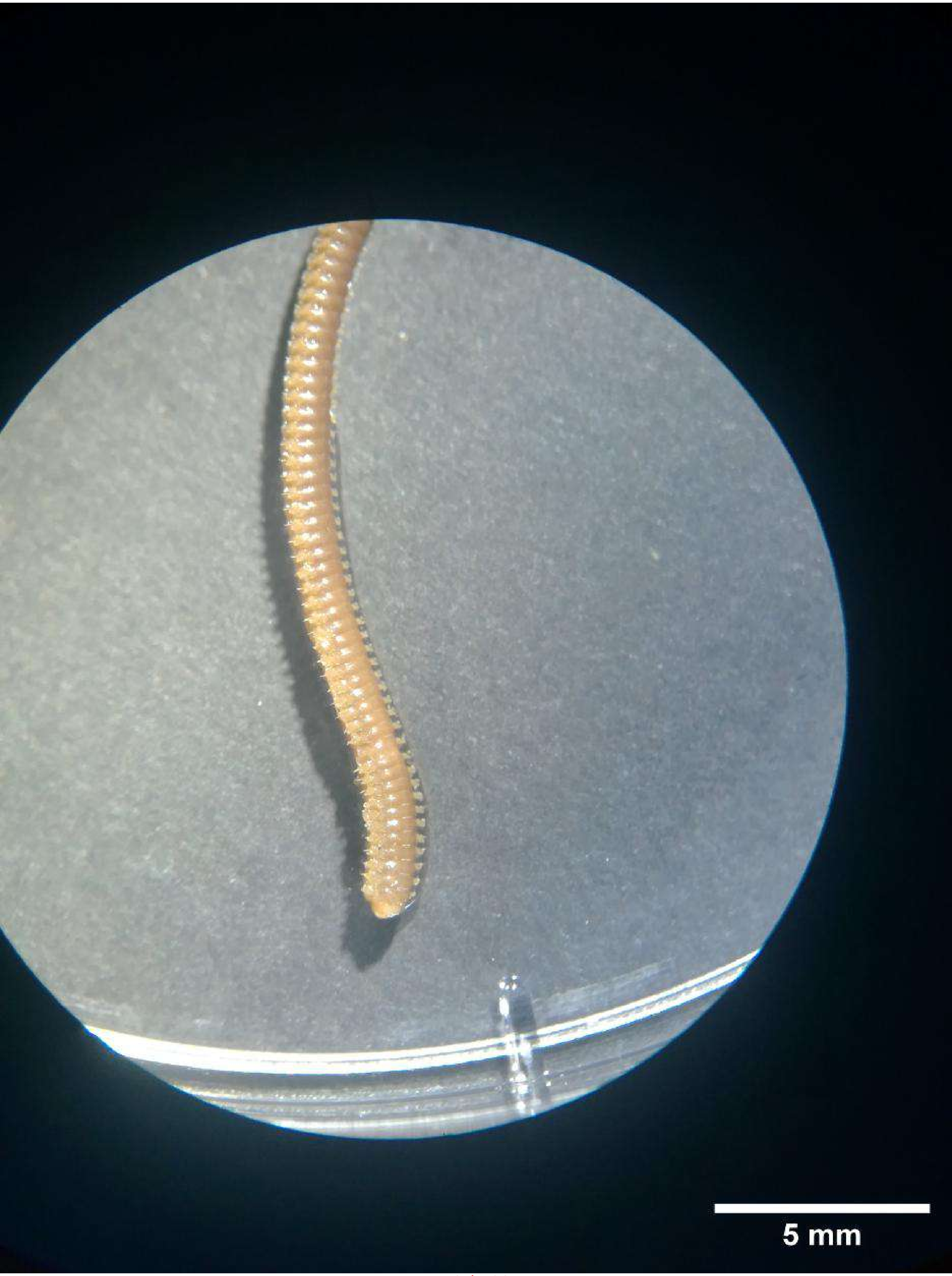
5 mm

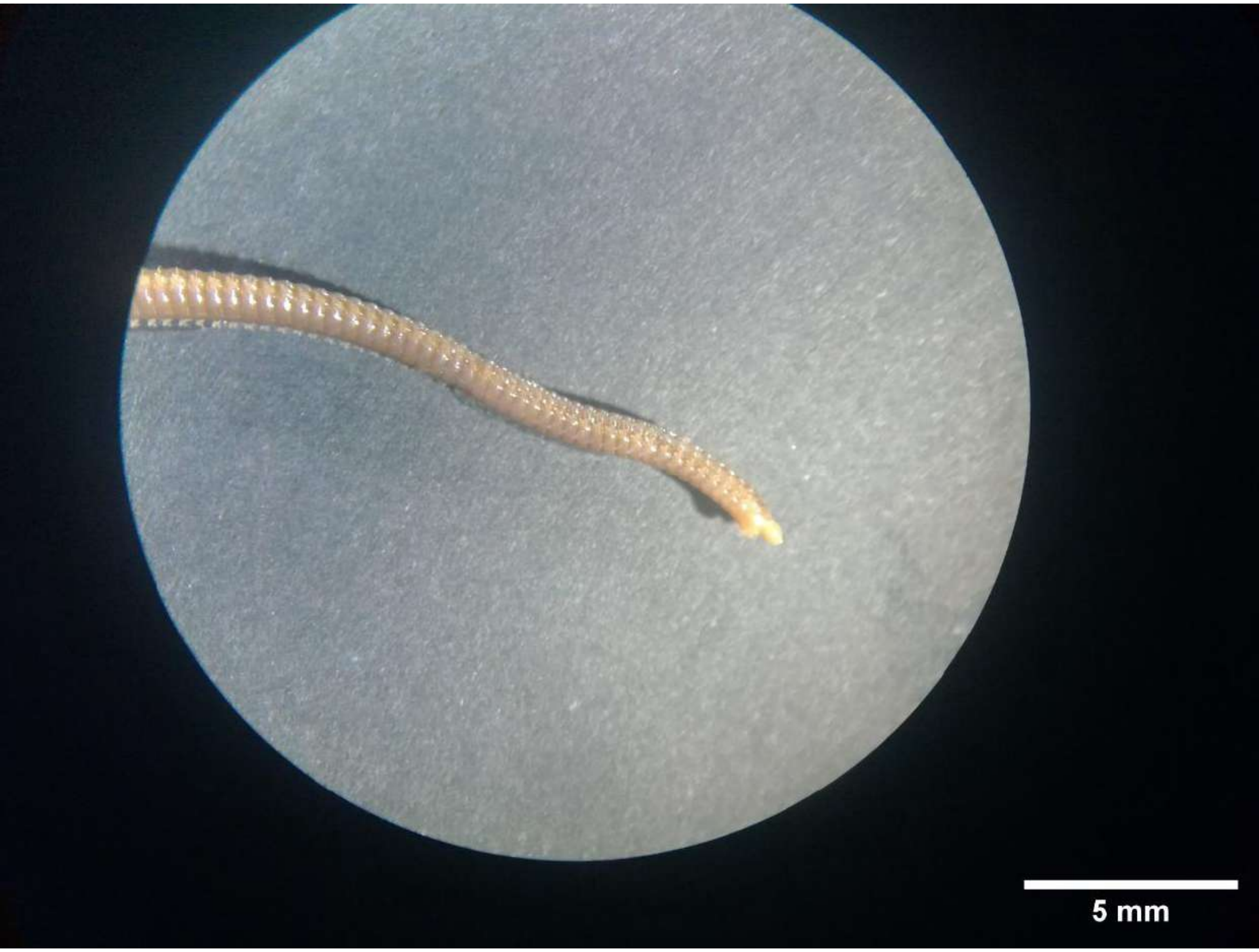
5 mm

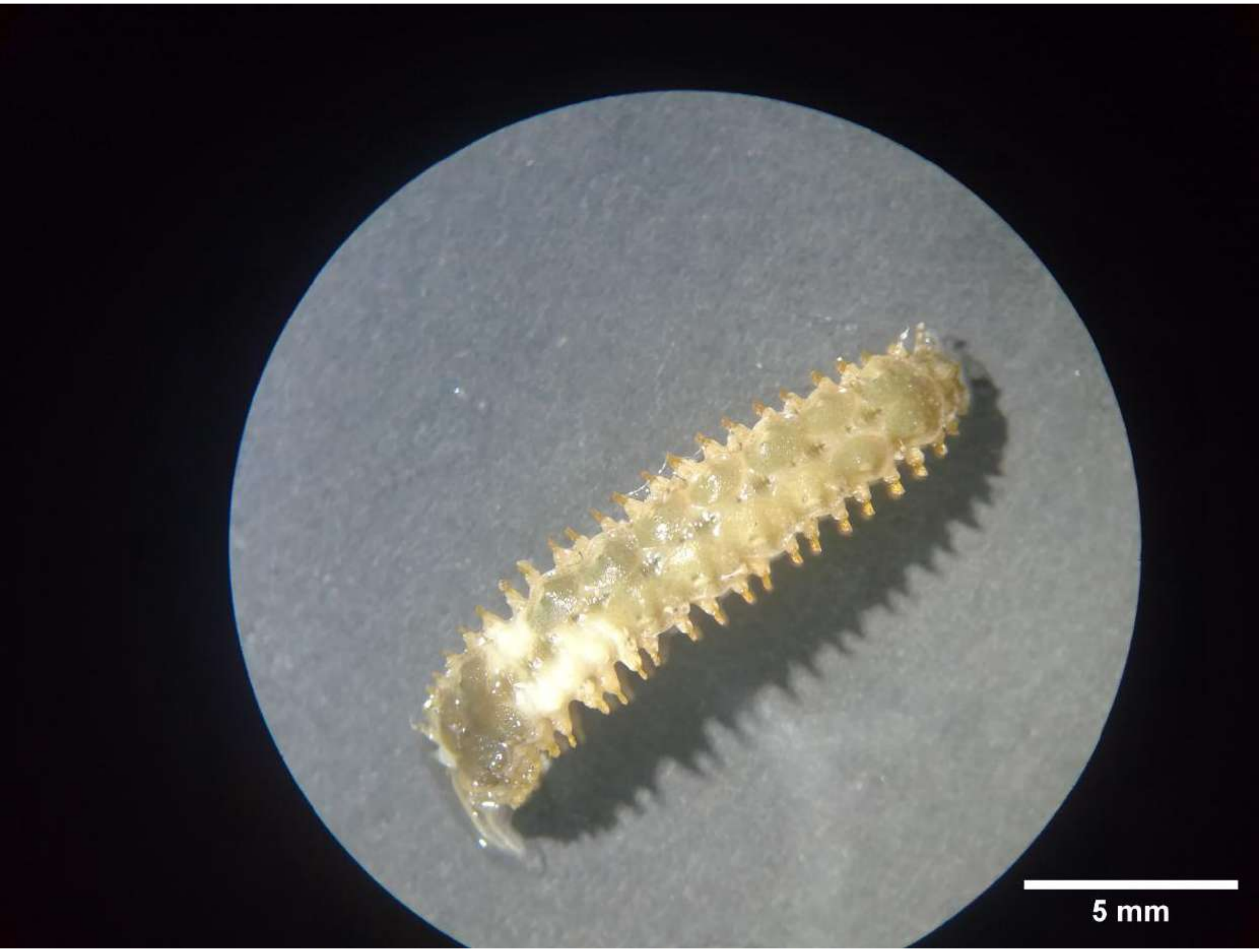
5 mm

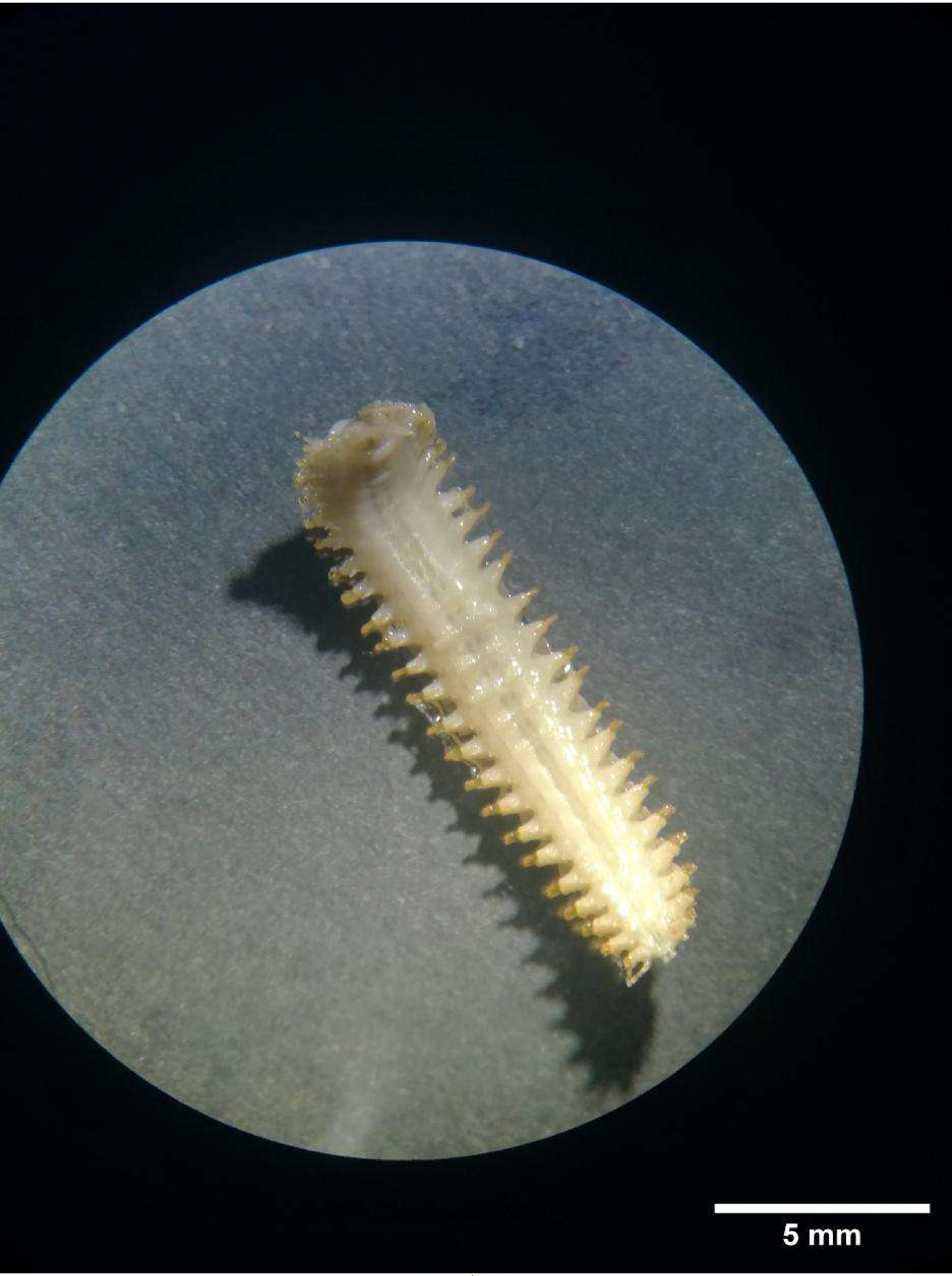
5 mm

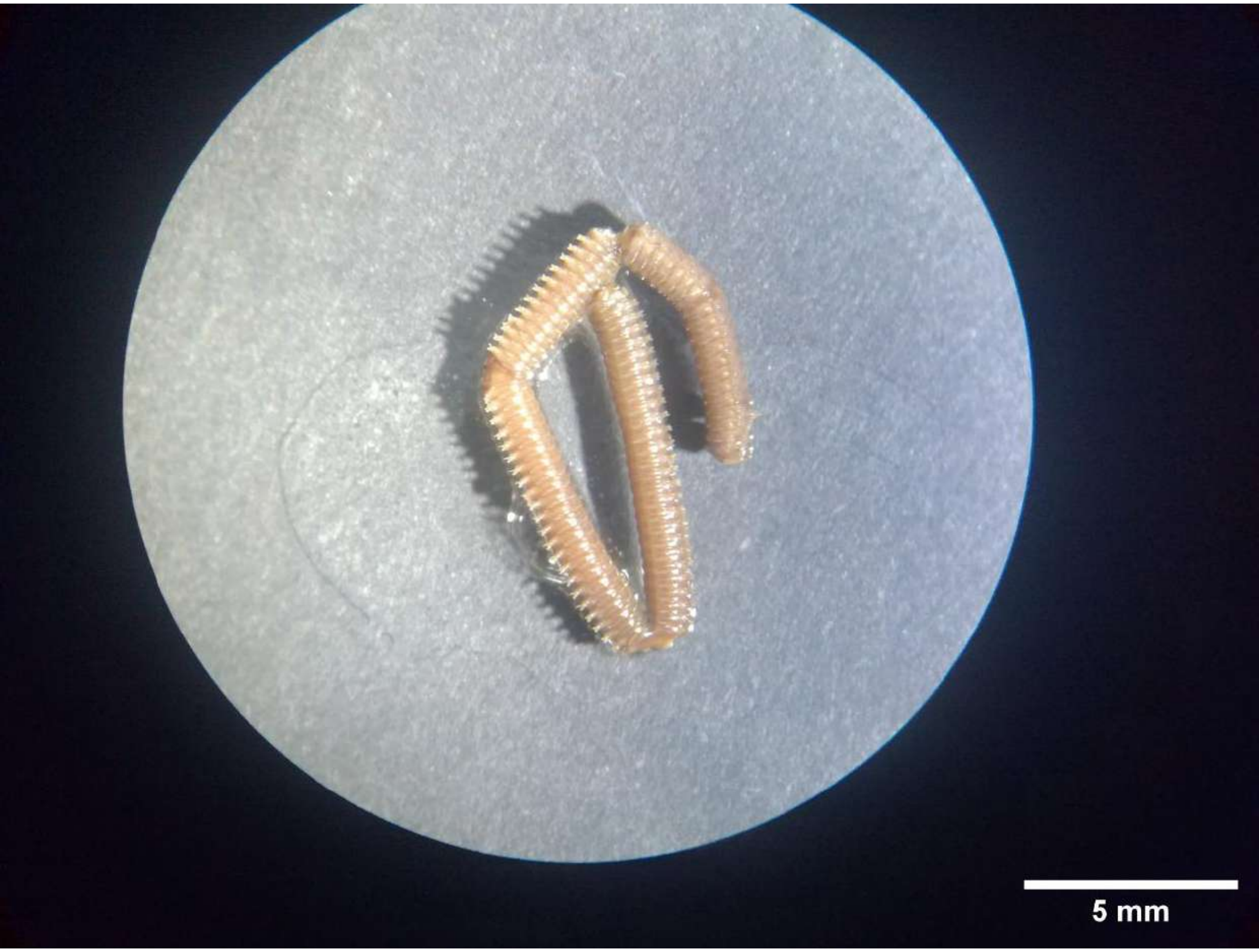
5 mm

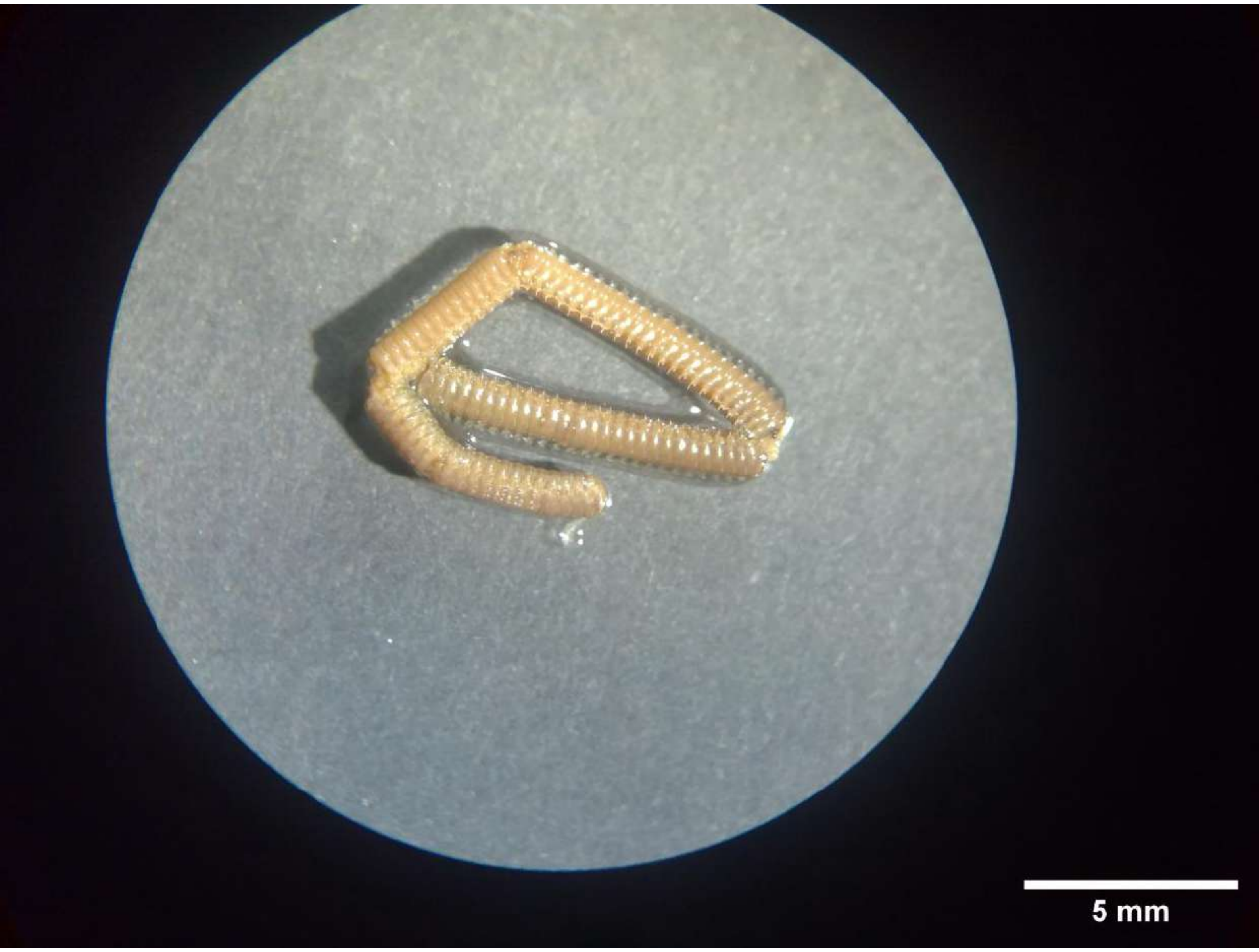
5 mm

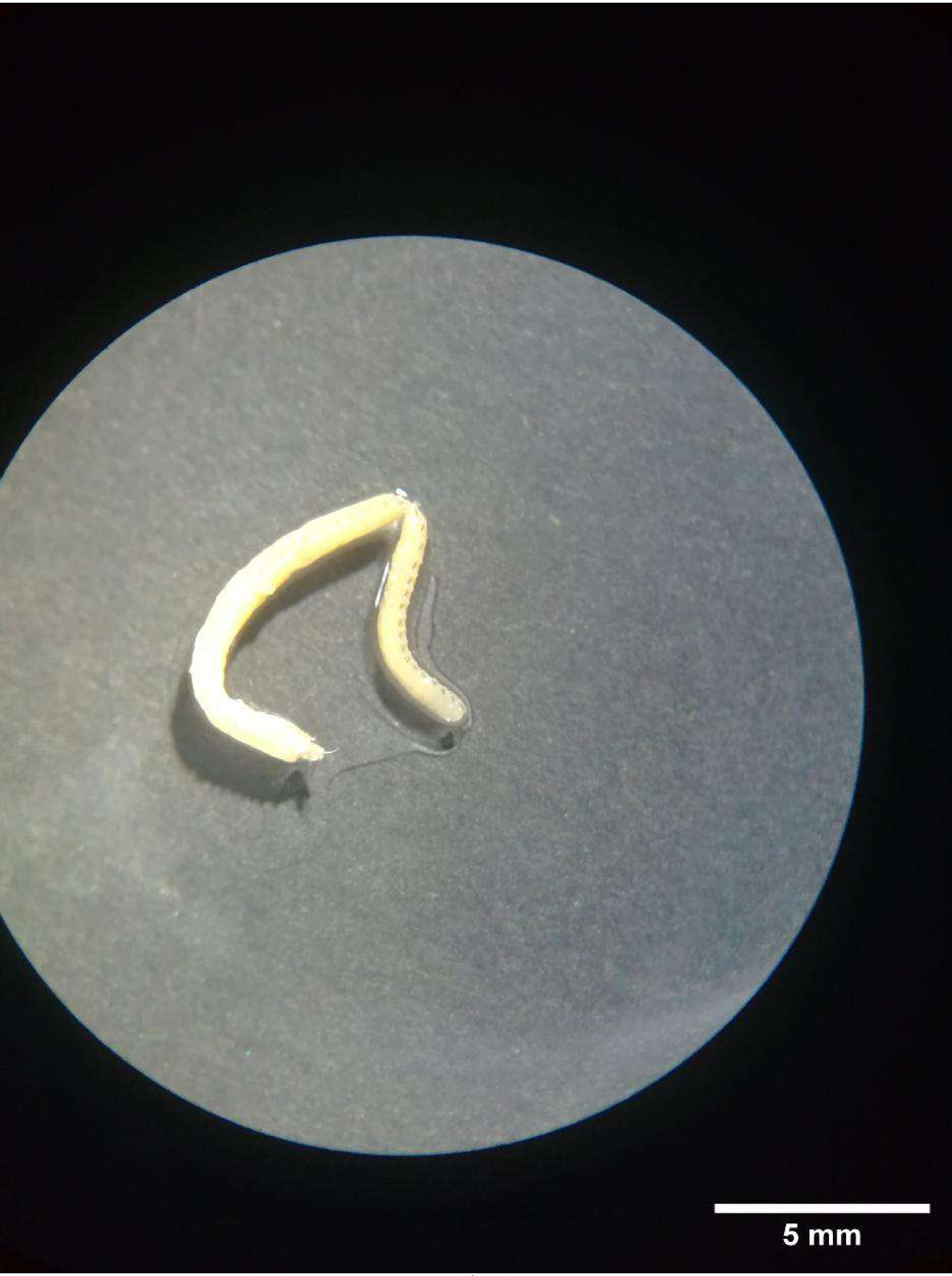
5 mm

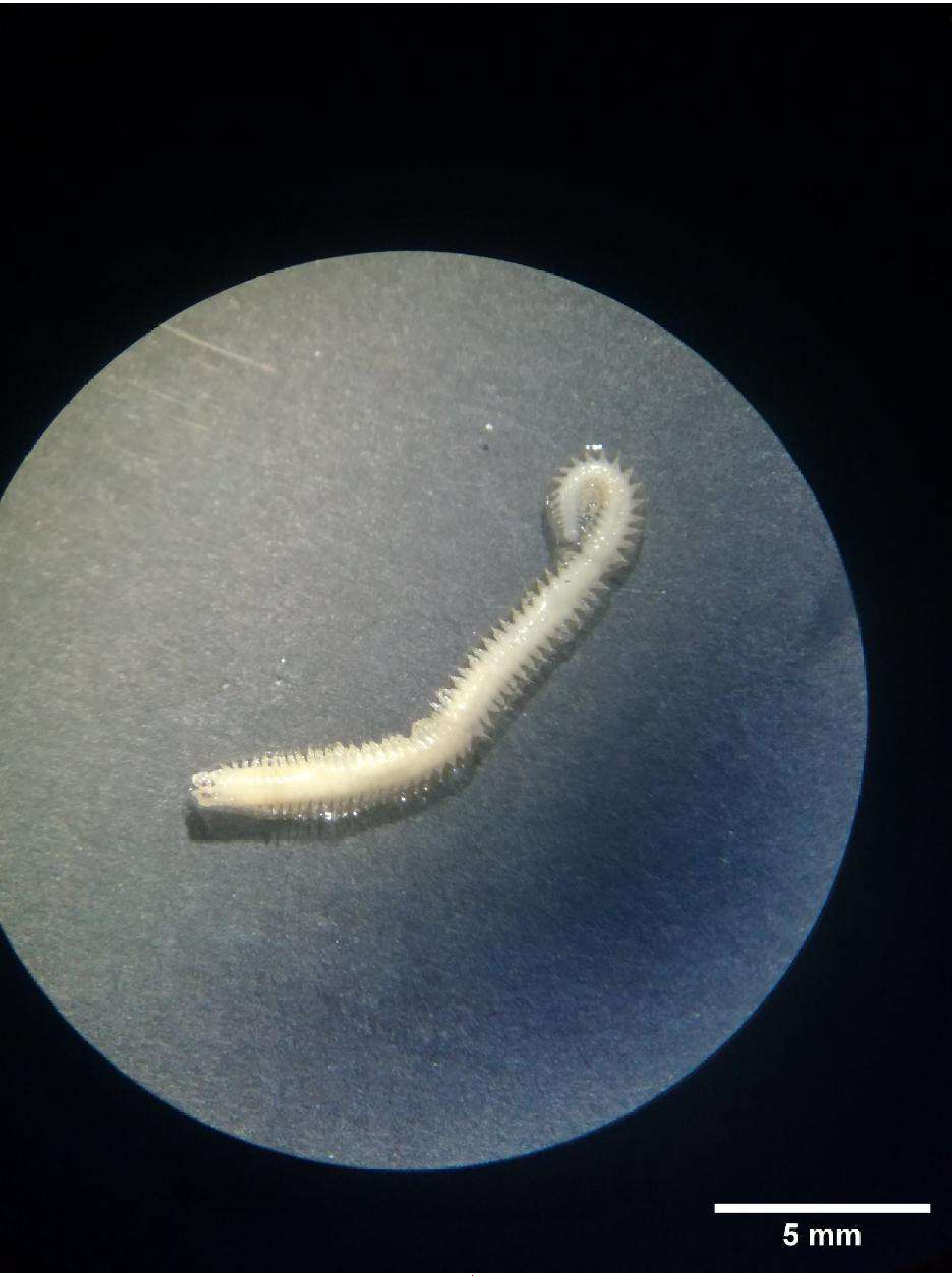
5 mm

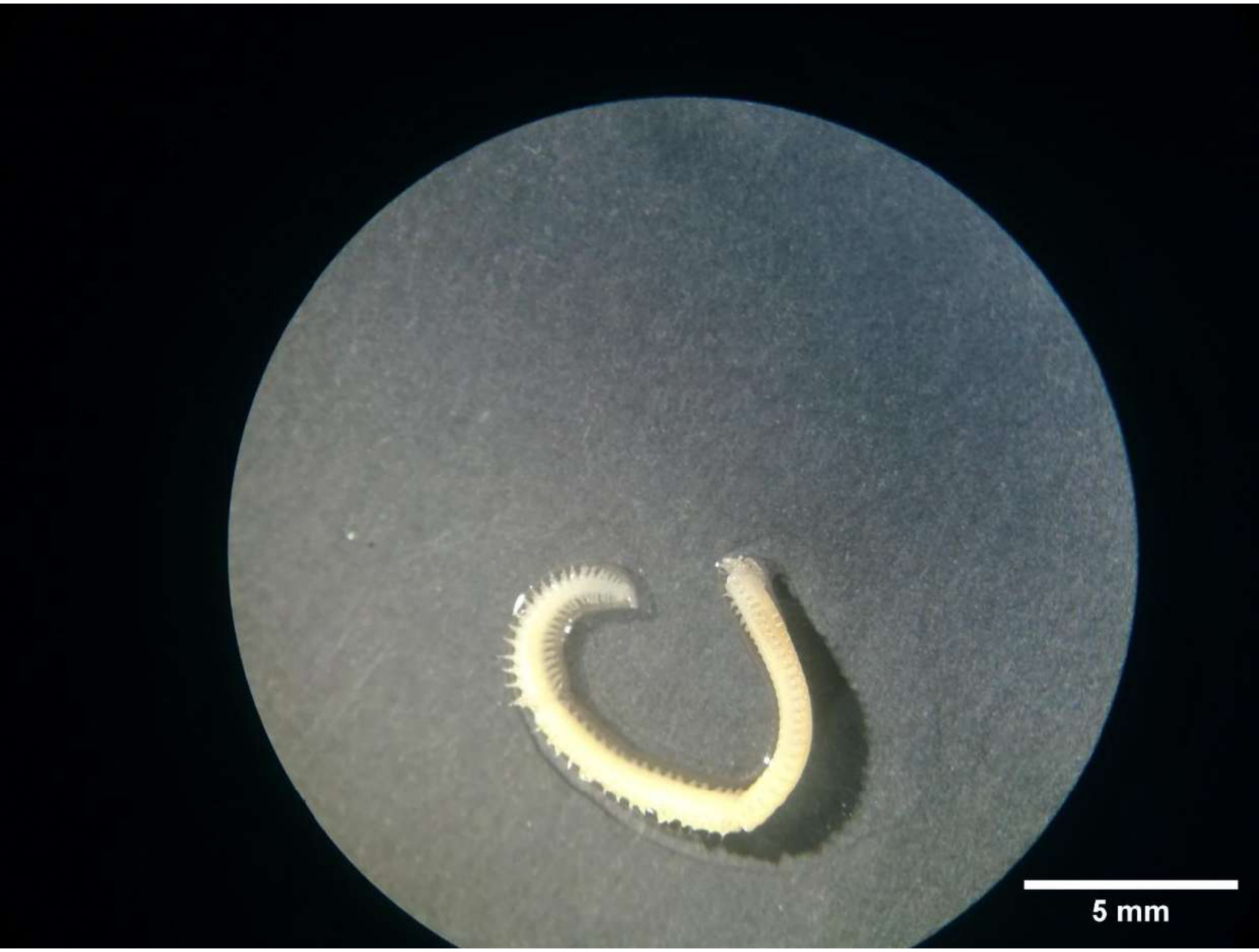
5 mm

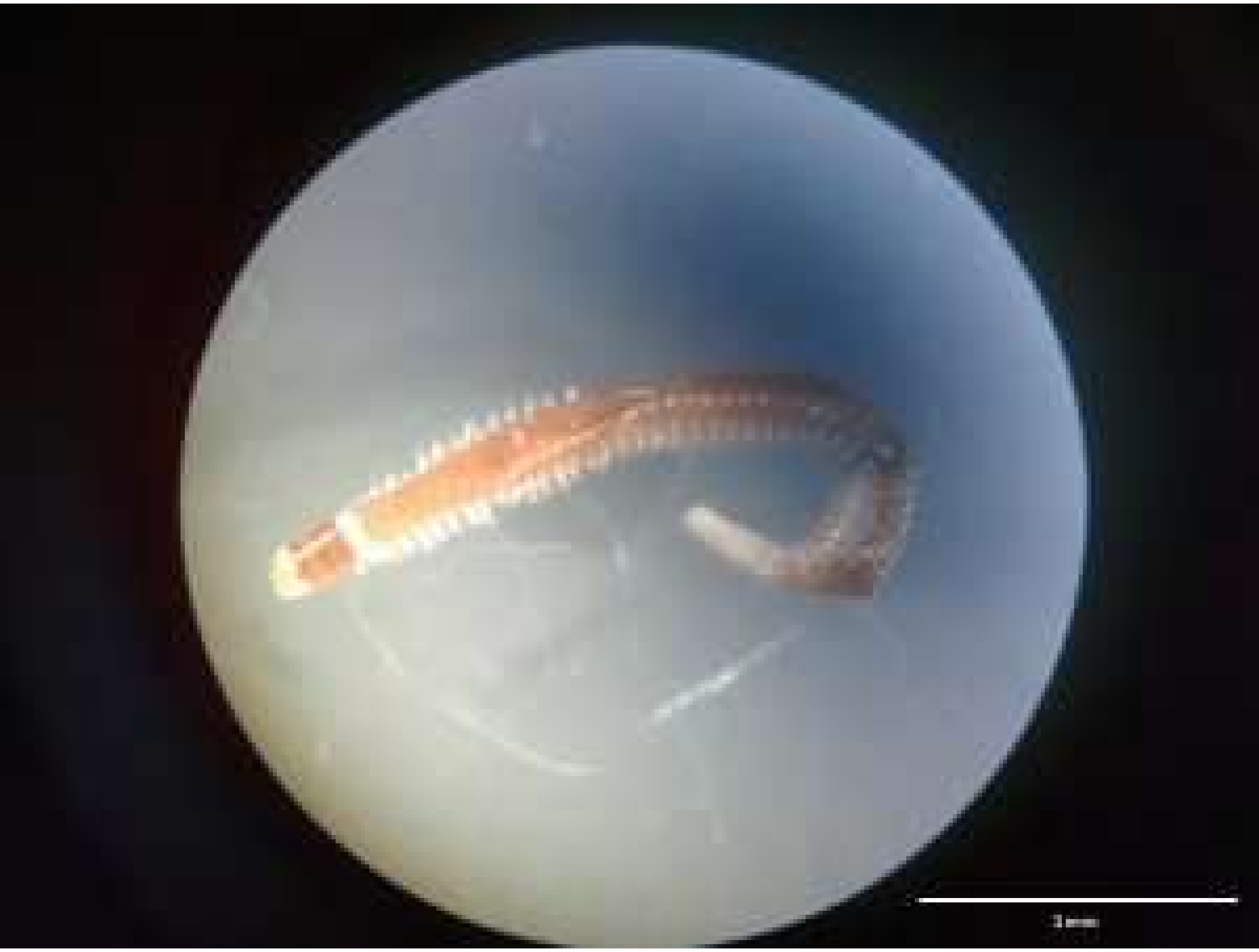

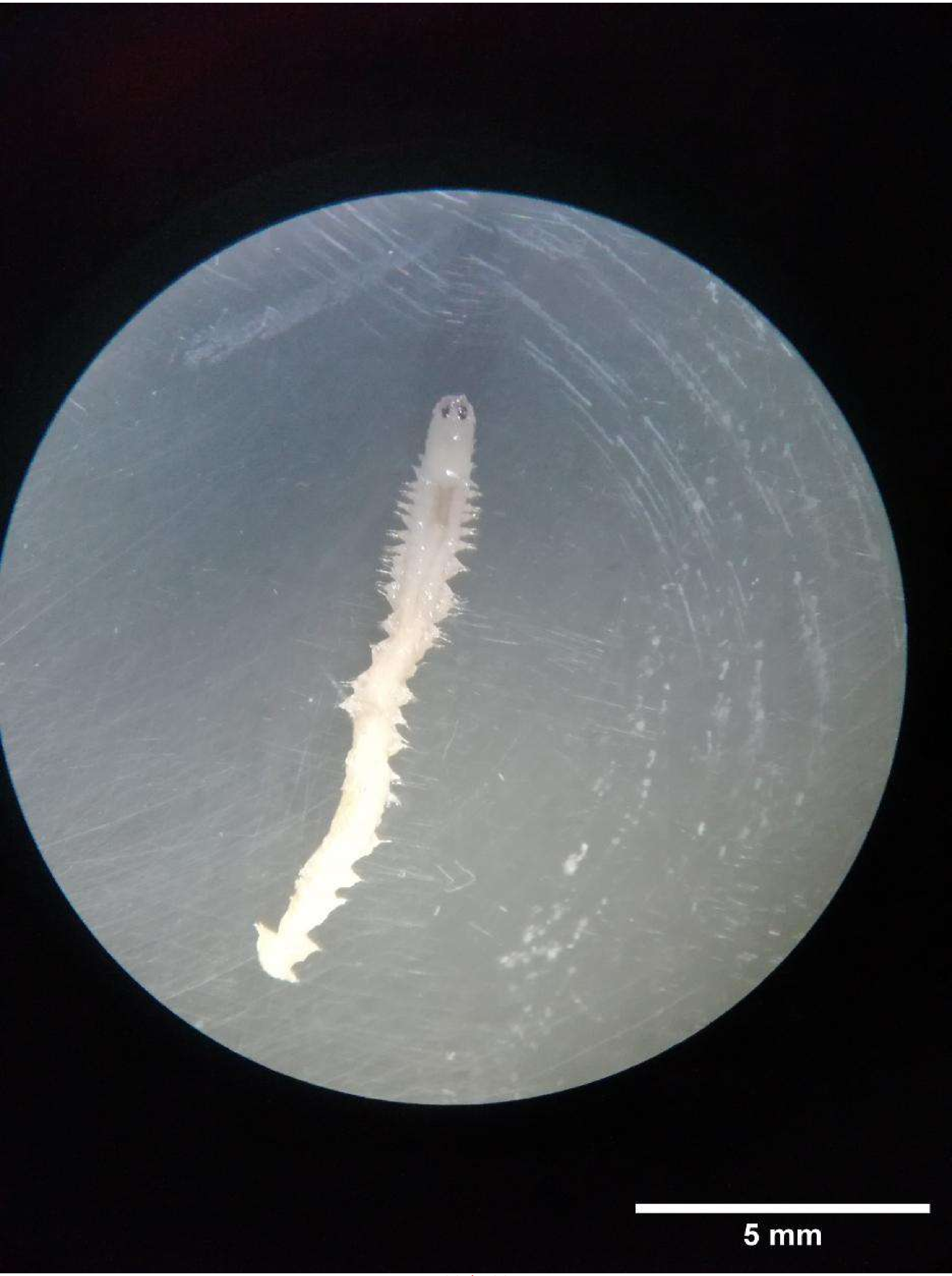
5 mm

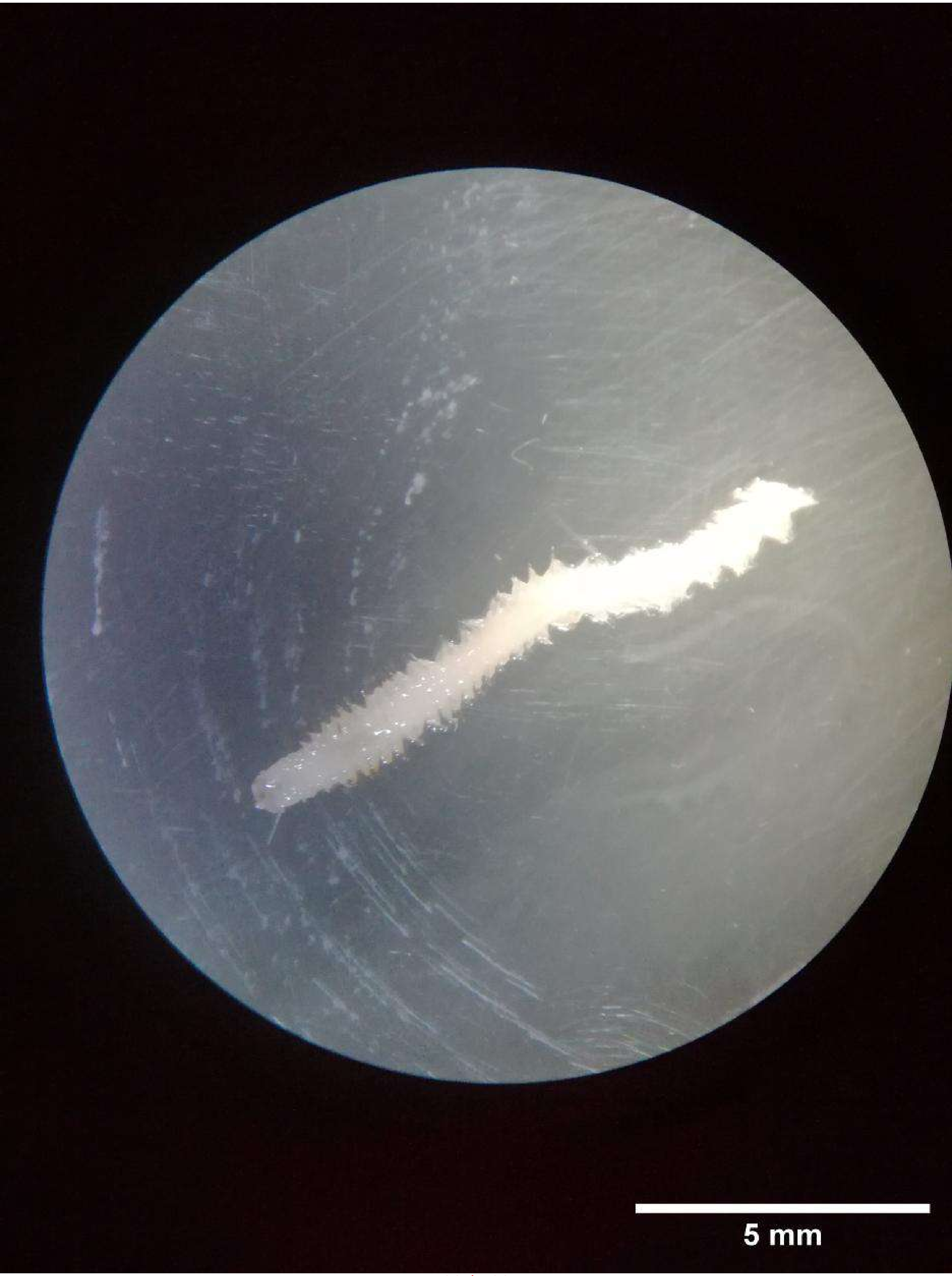
5 mm

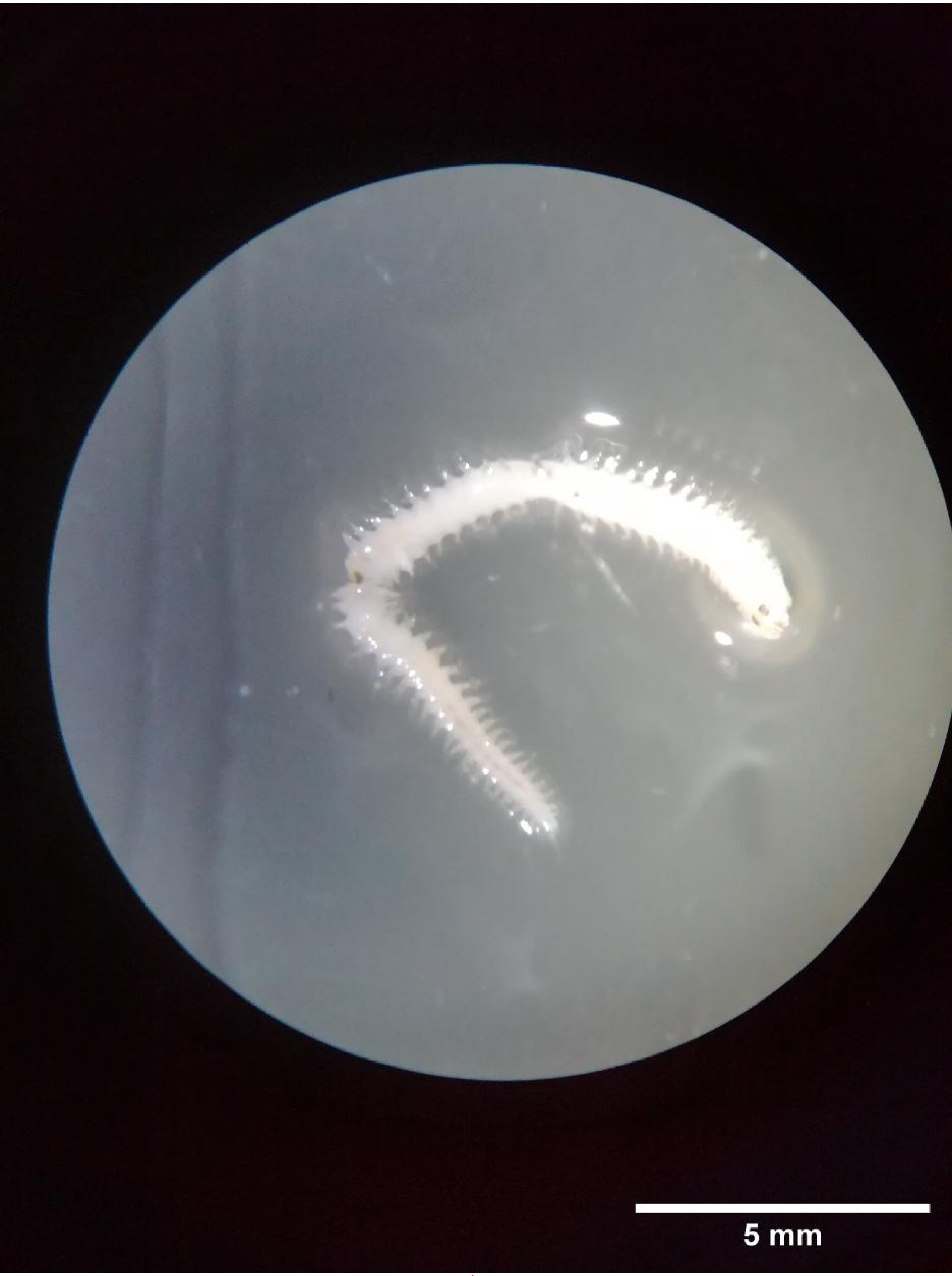
5 mm

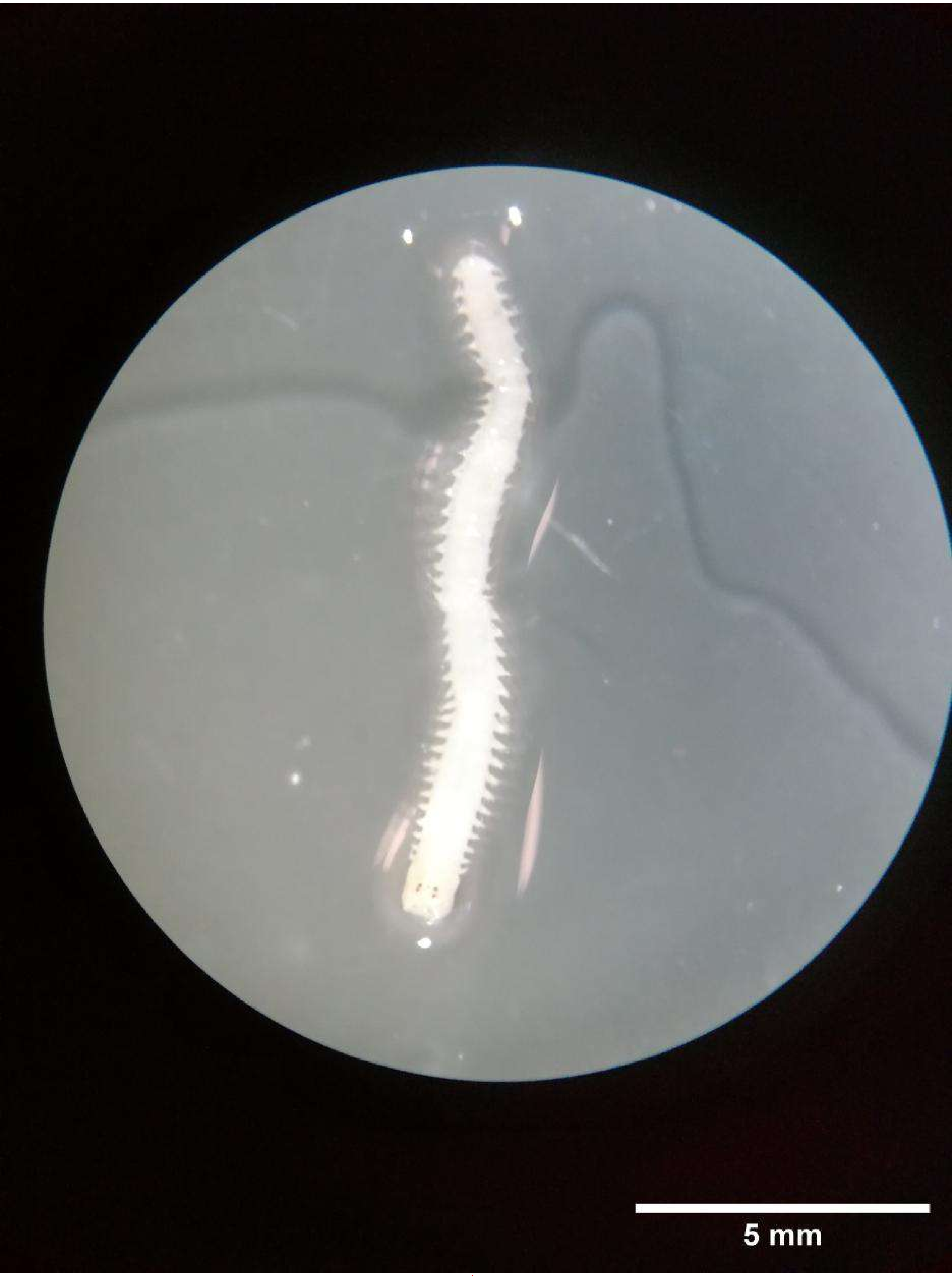
5 mm

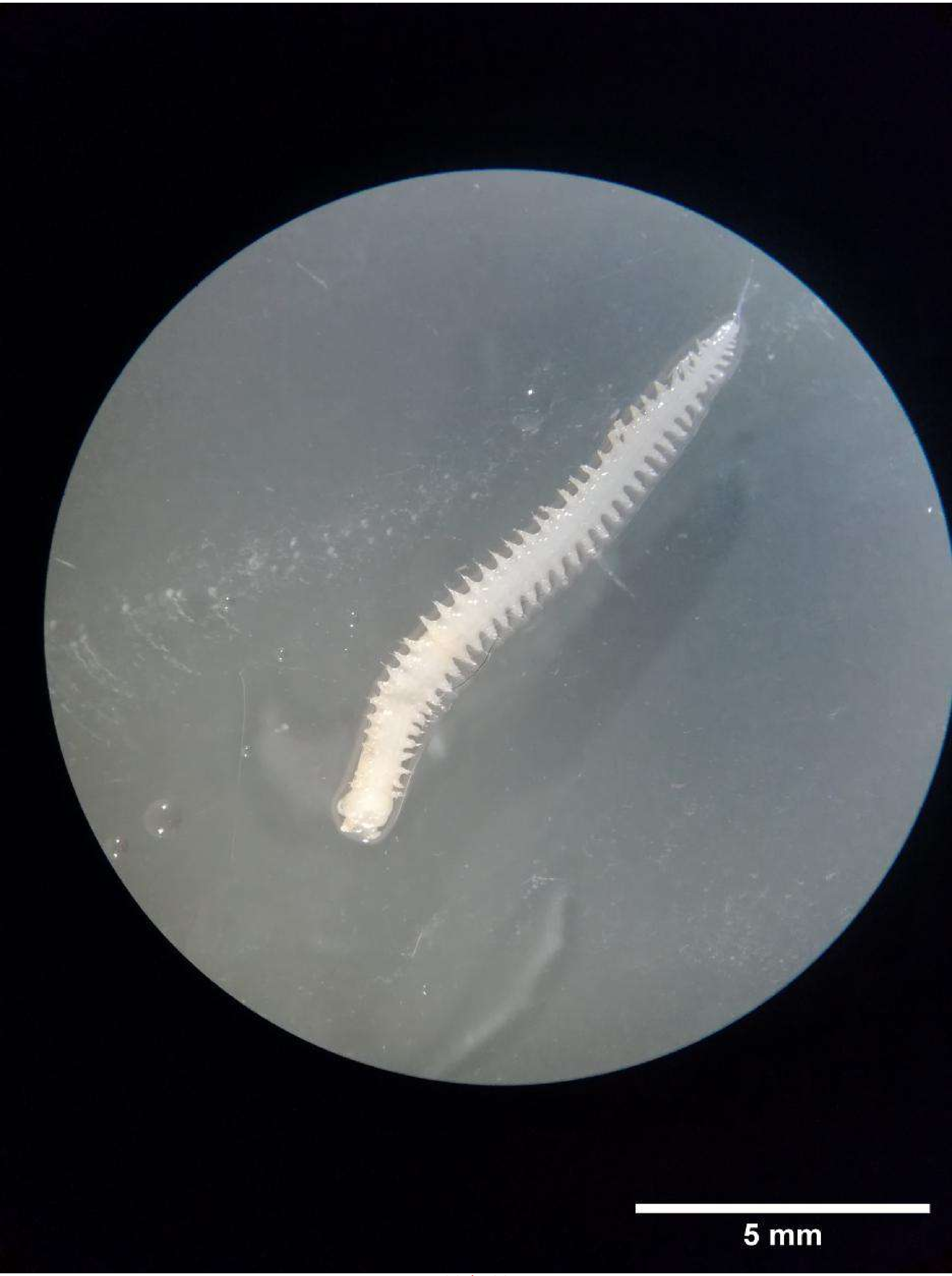
5 mm

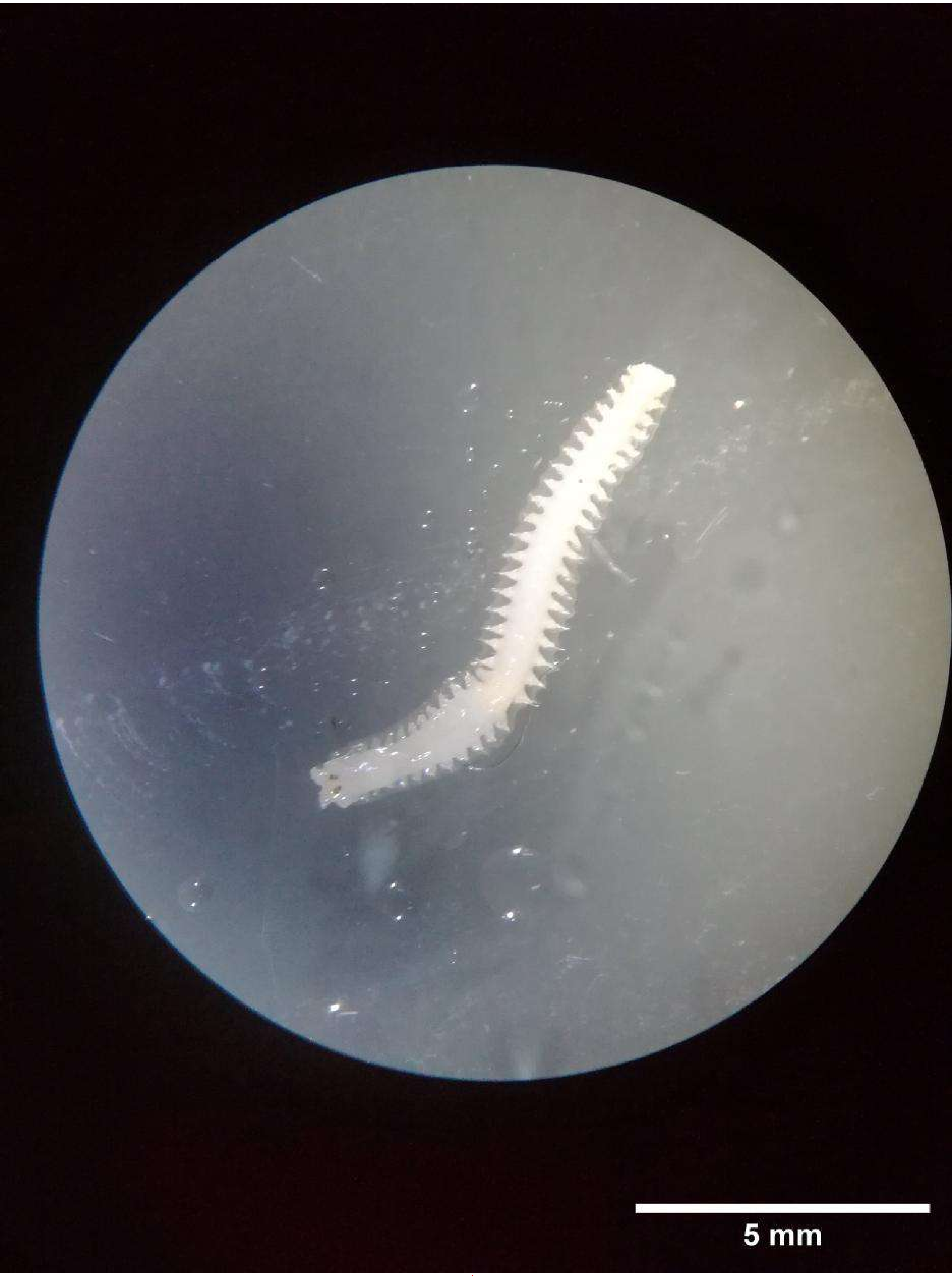
5 mm

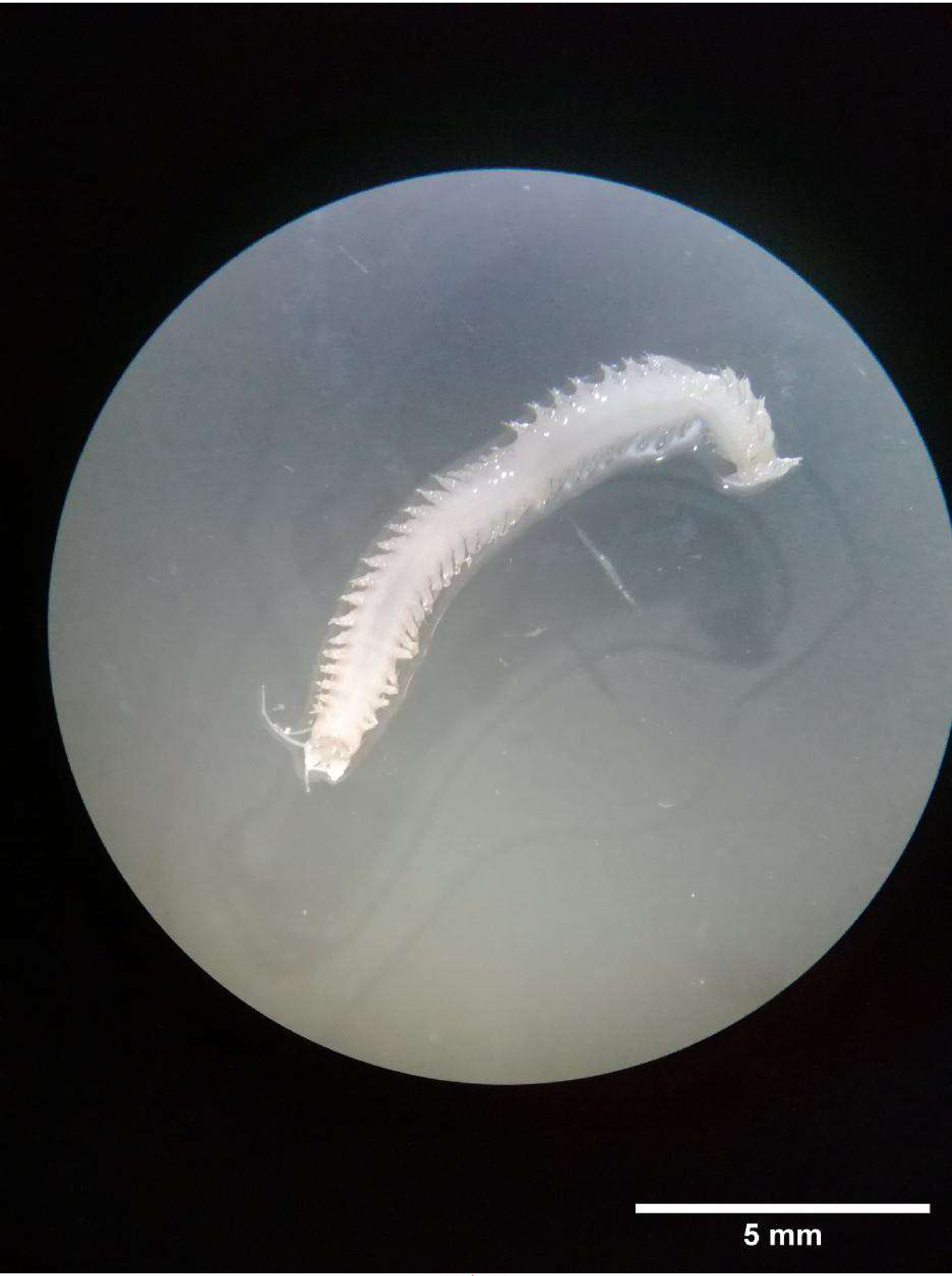

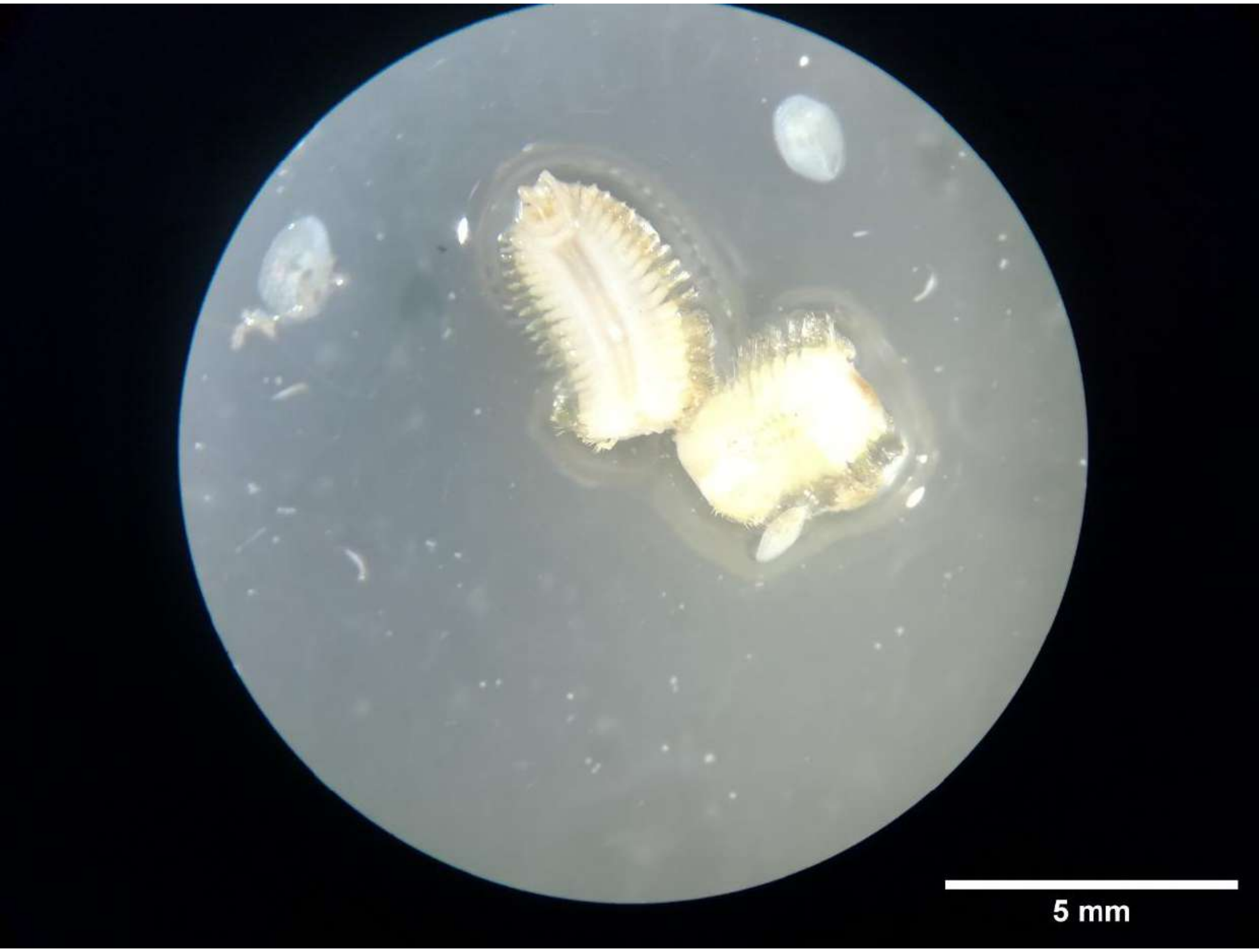
5 mm

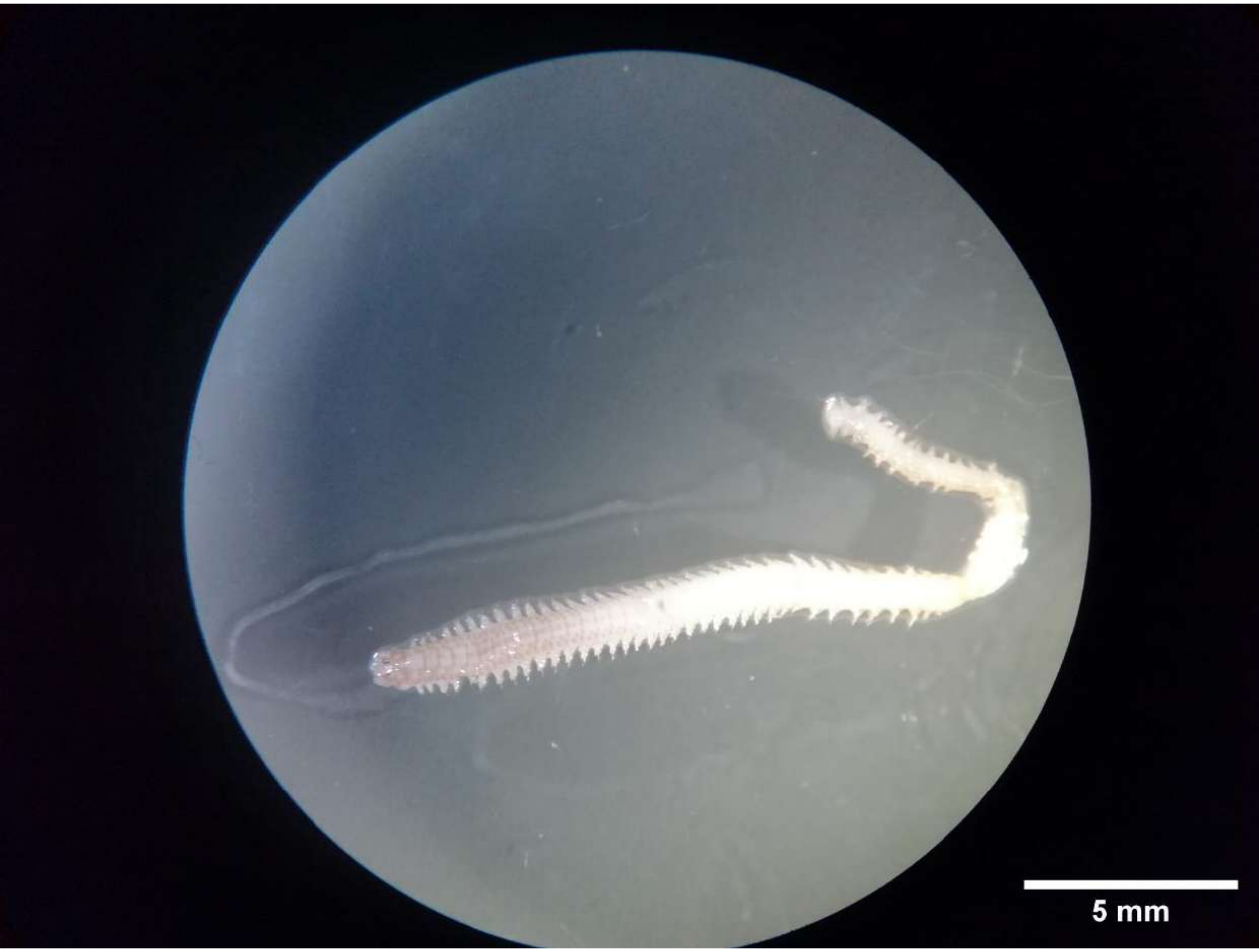
5 mm

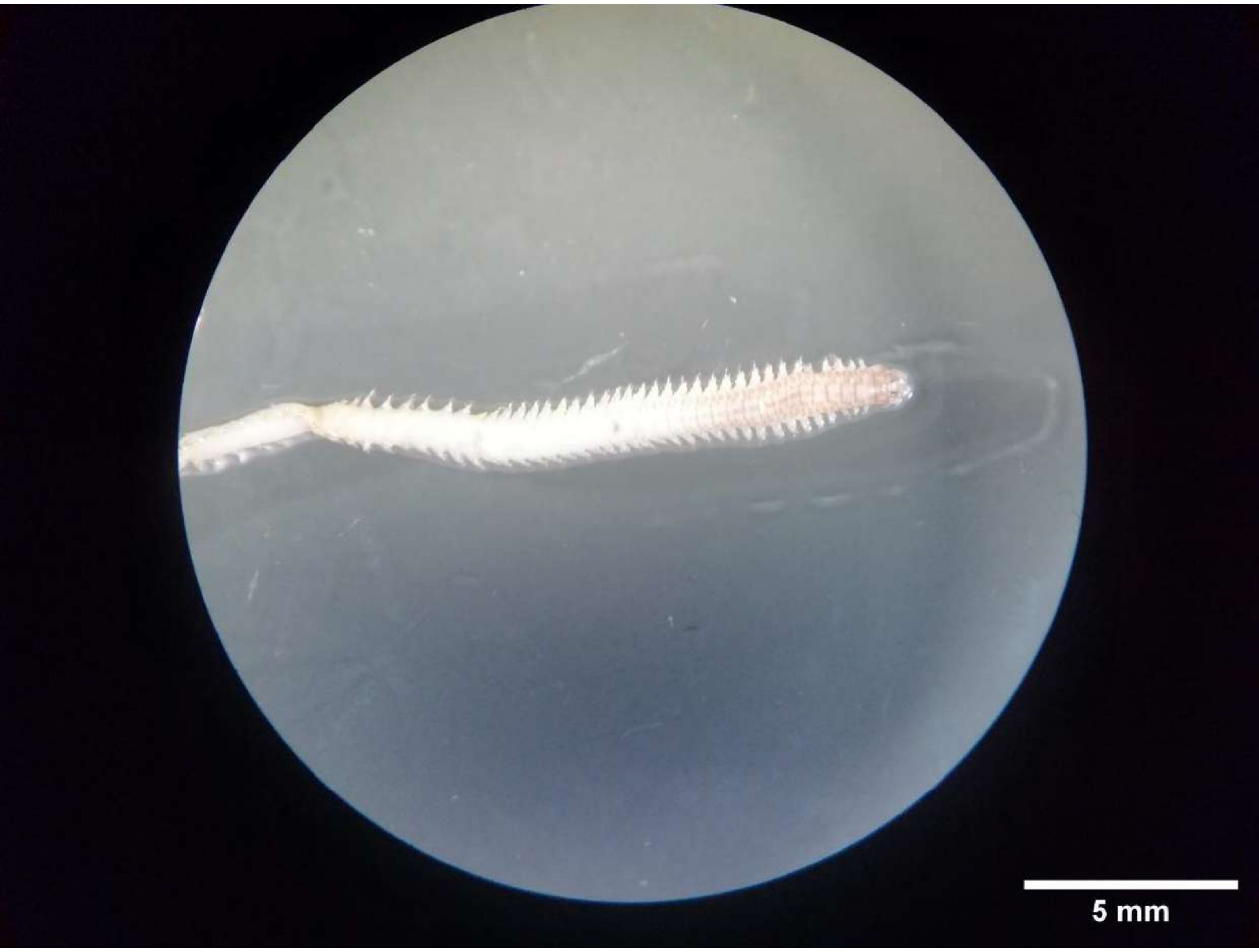
5 mm

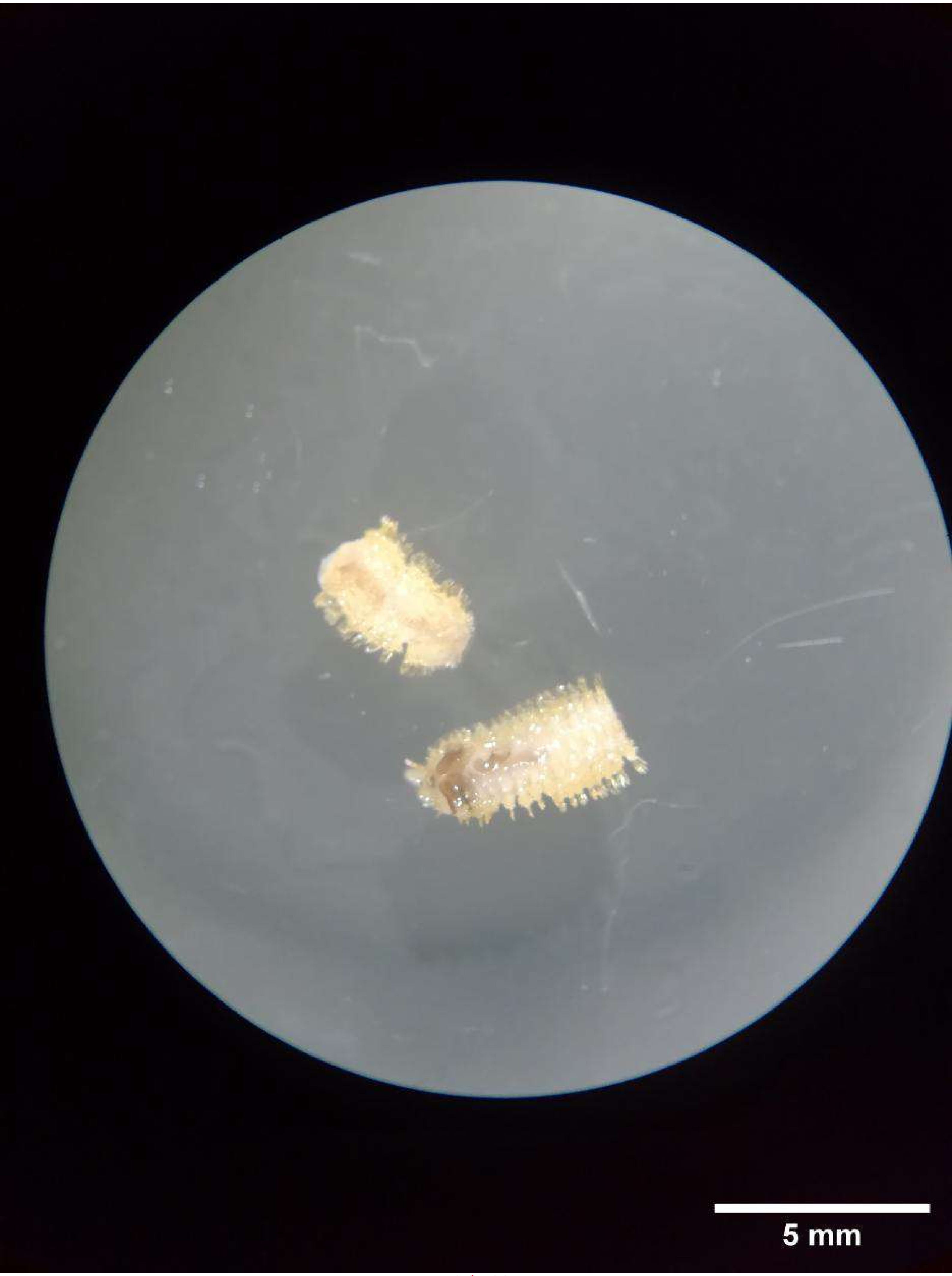
5 mm

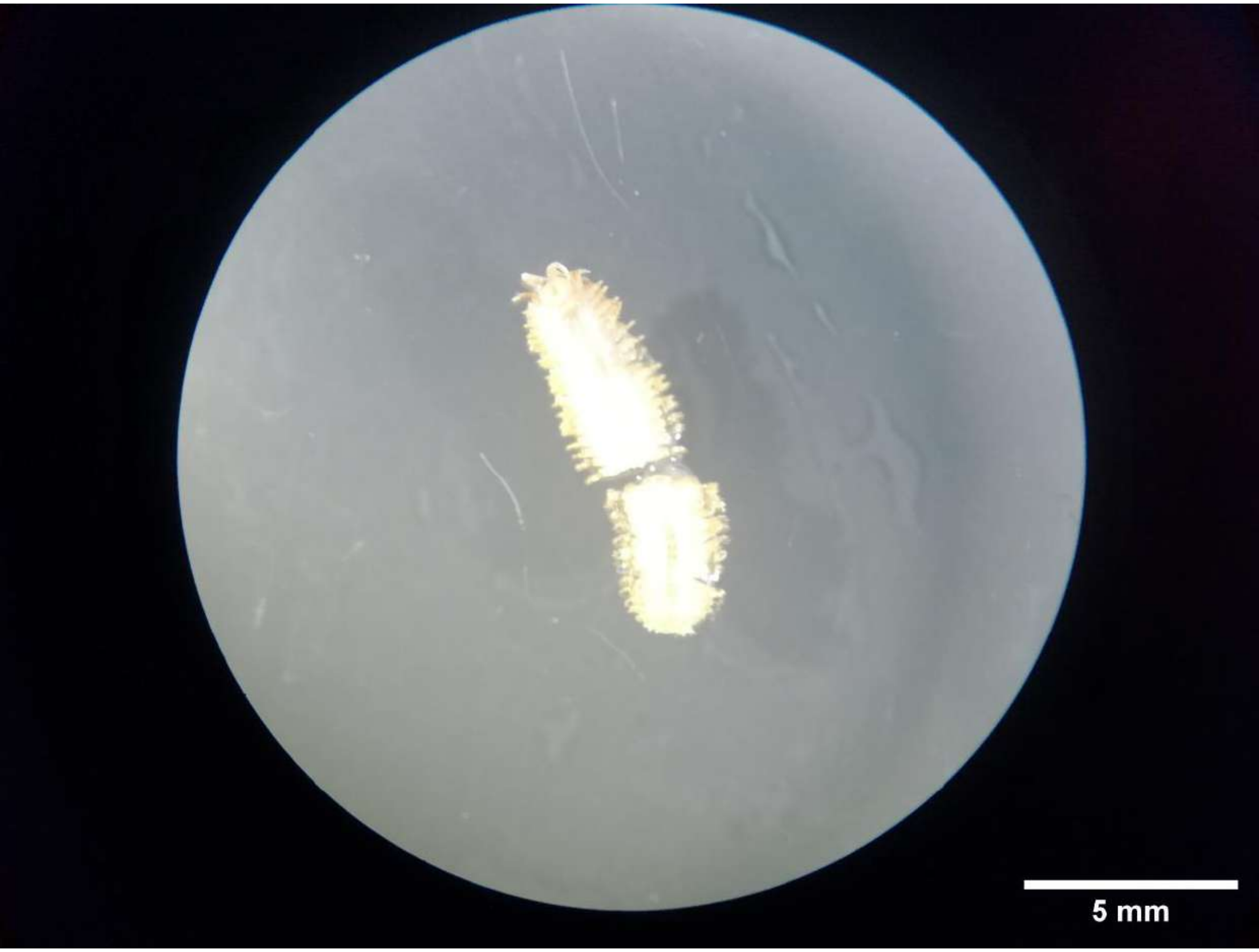
5 mm

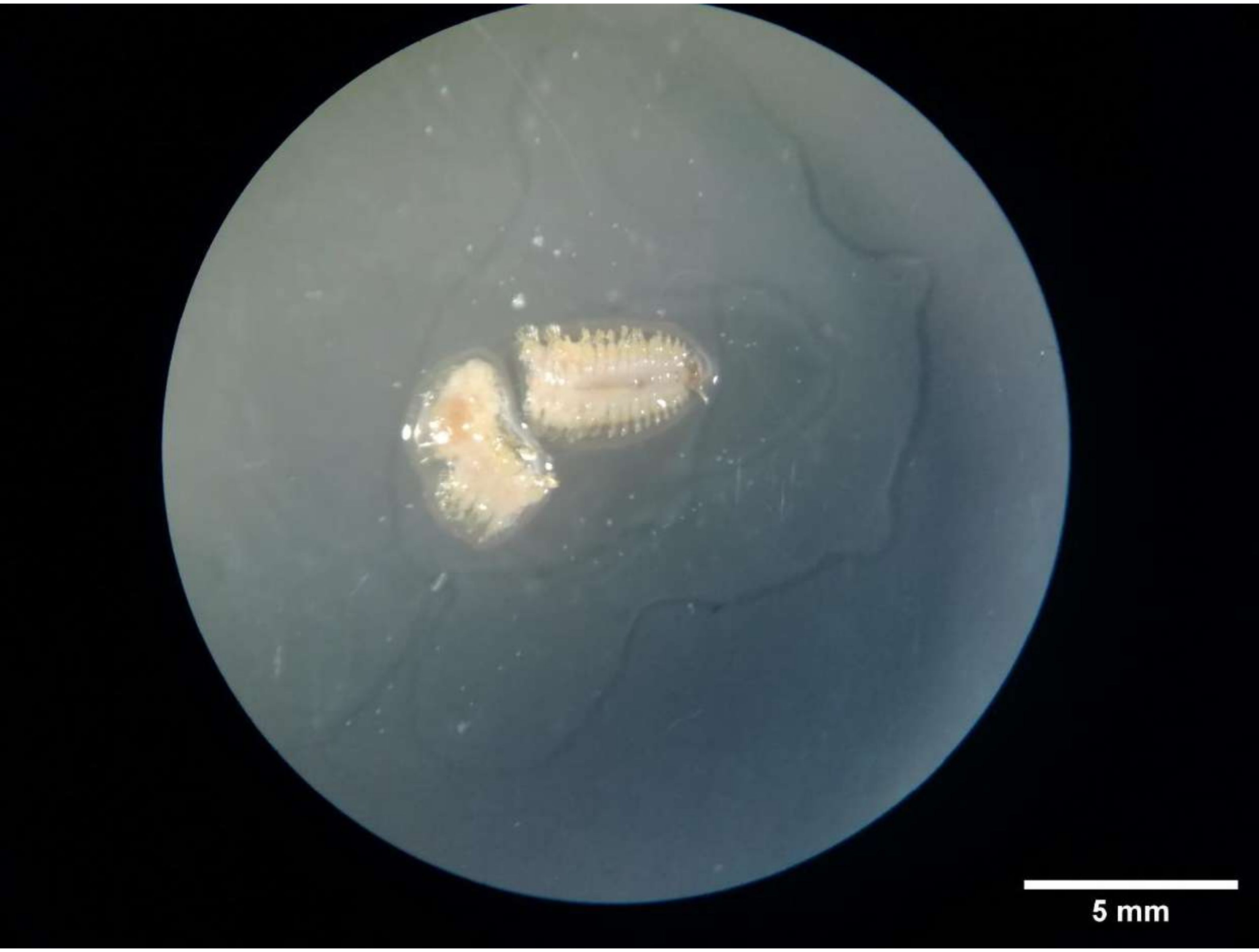
5 mm

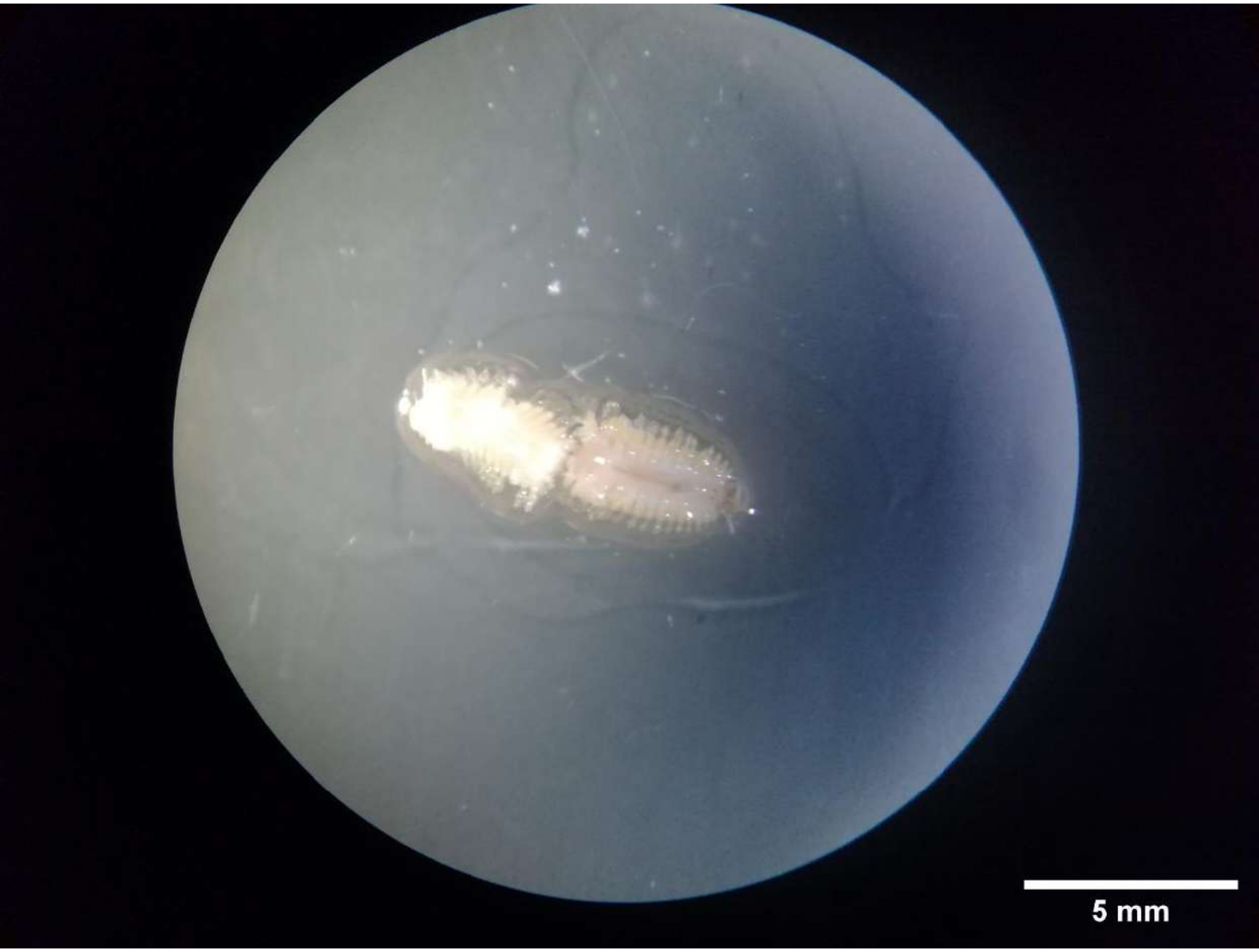
5 mm

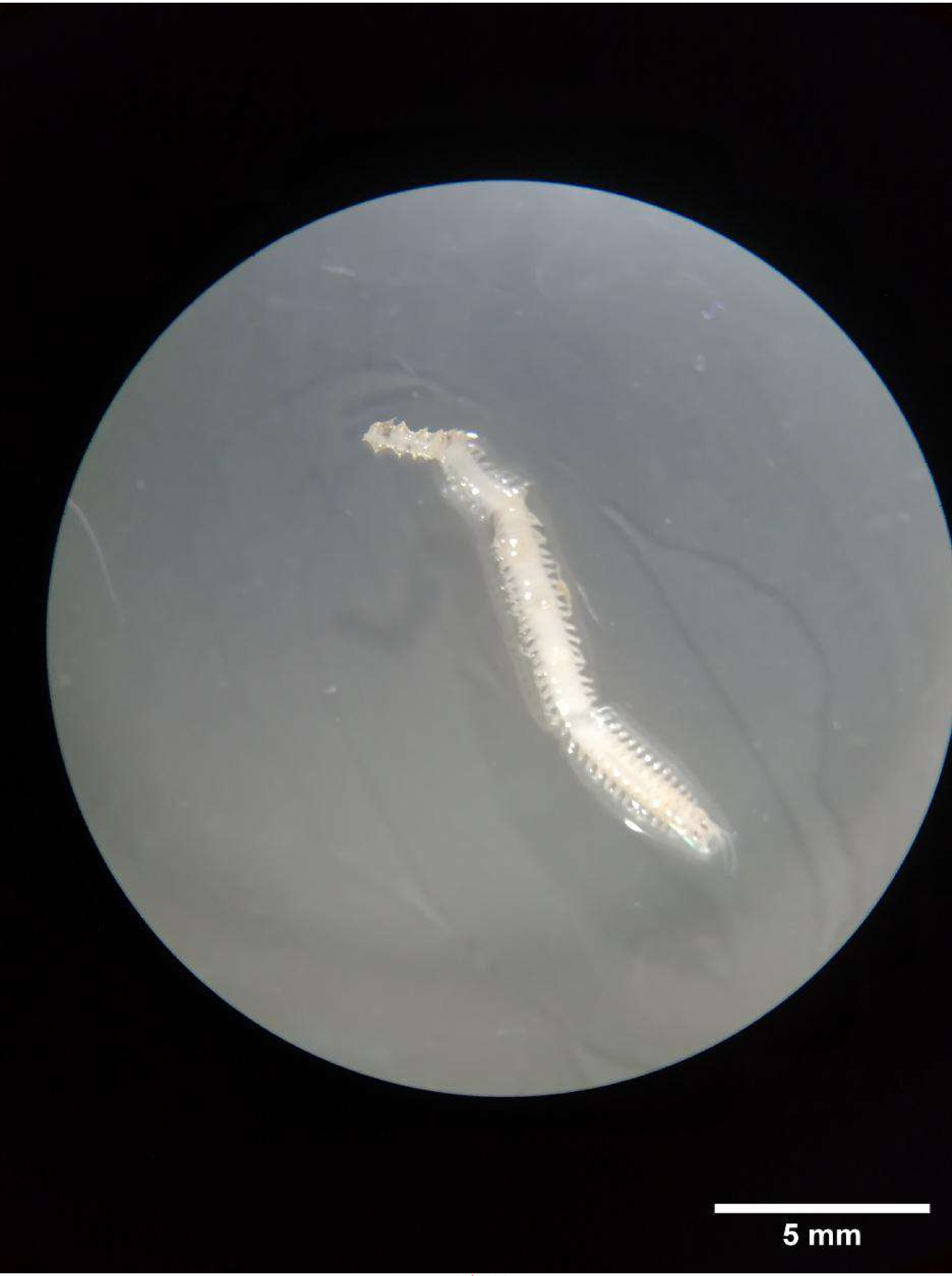
5 mm

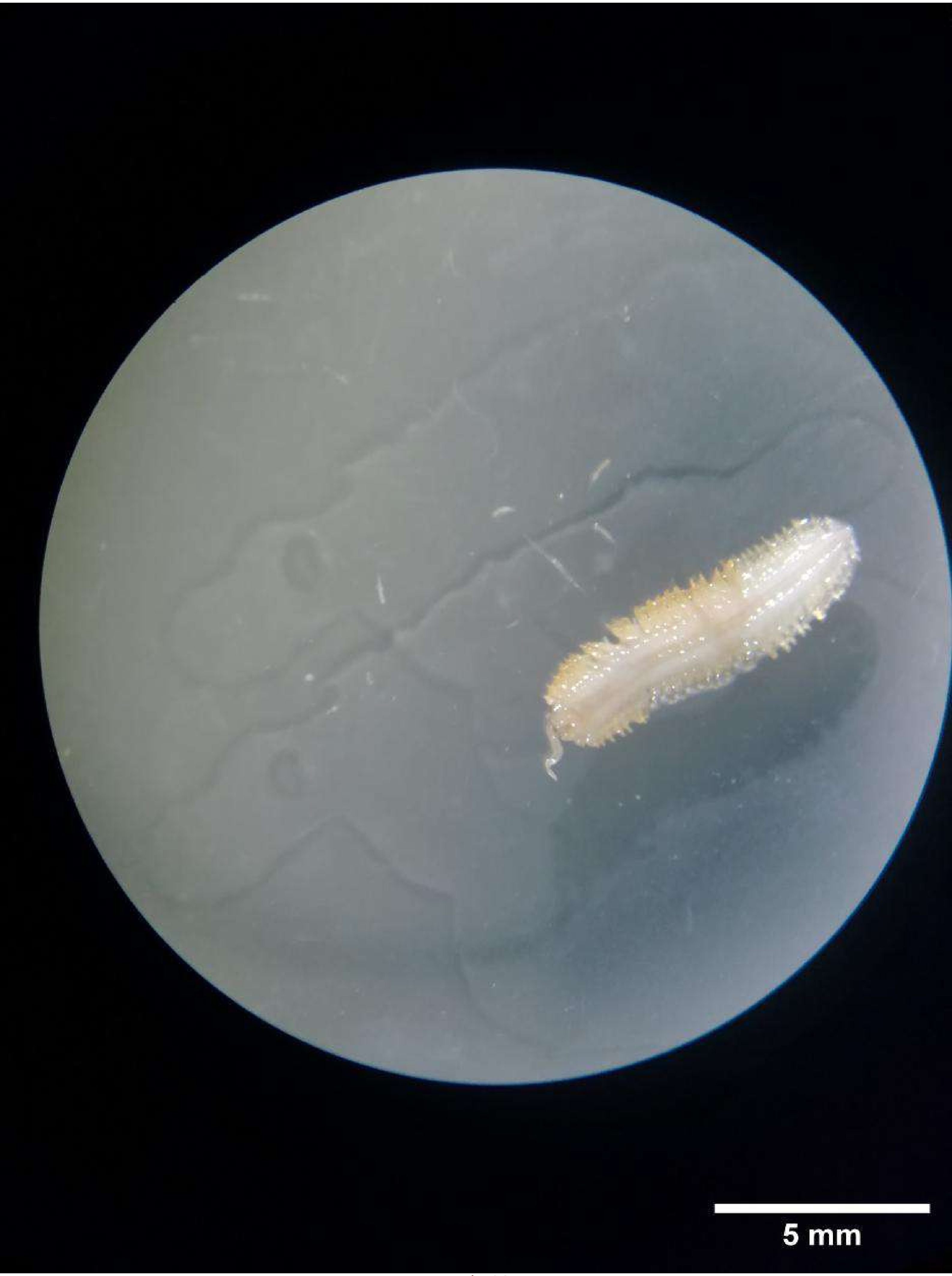
5 mm

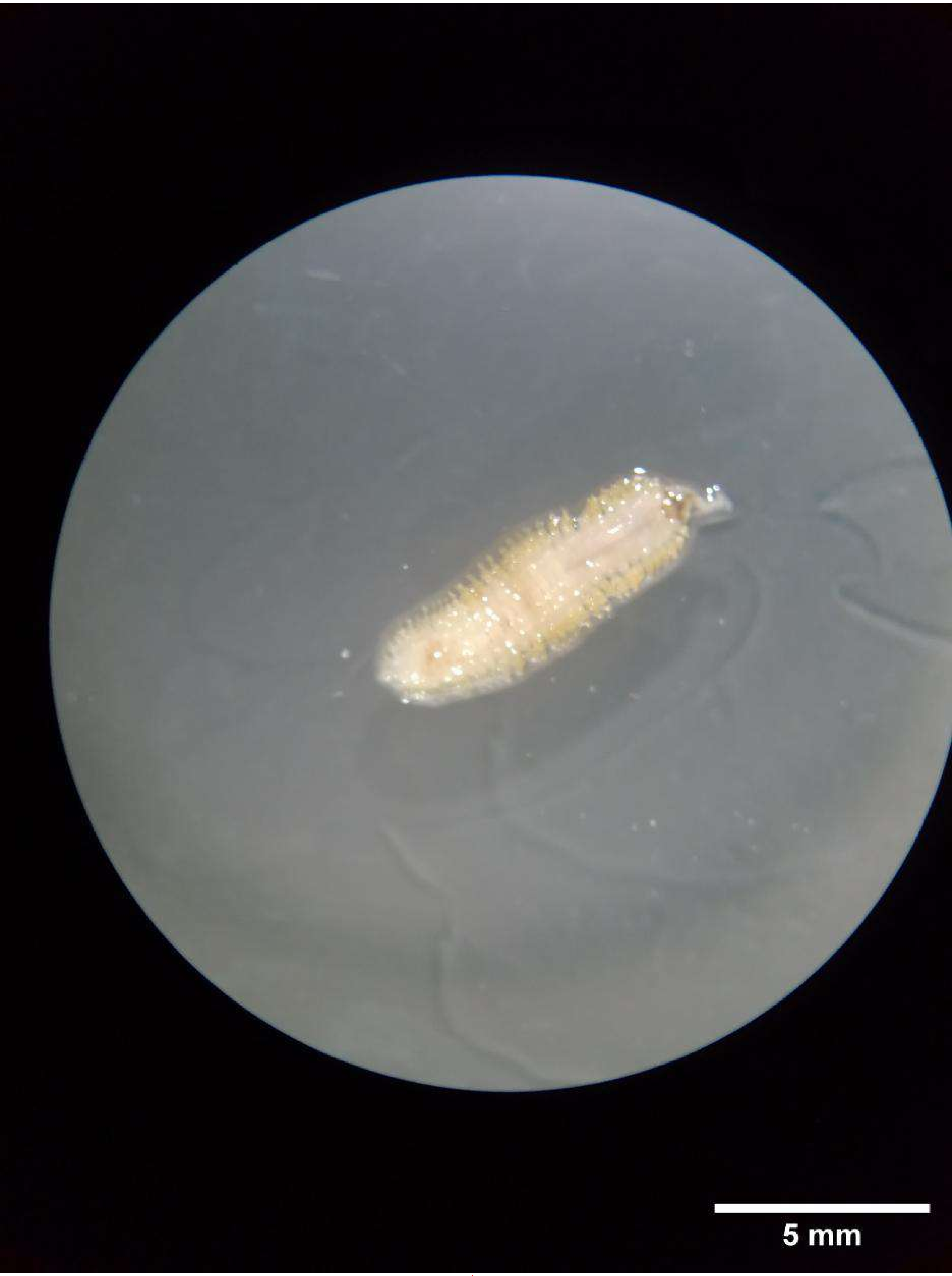
5 mm

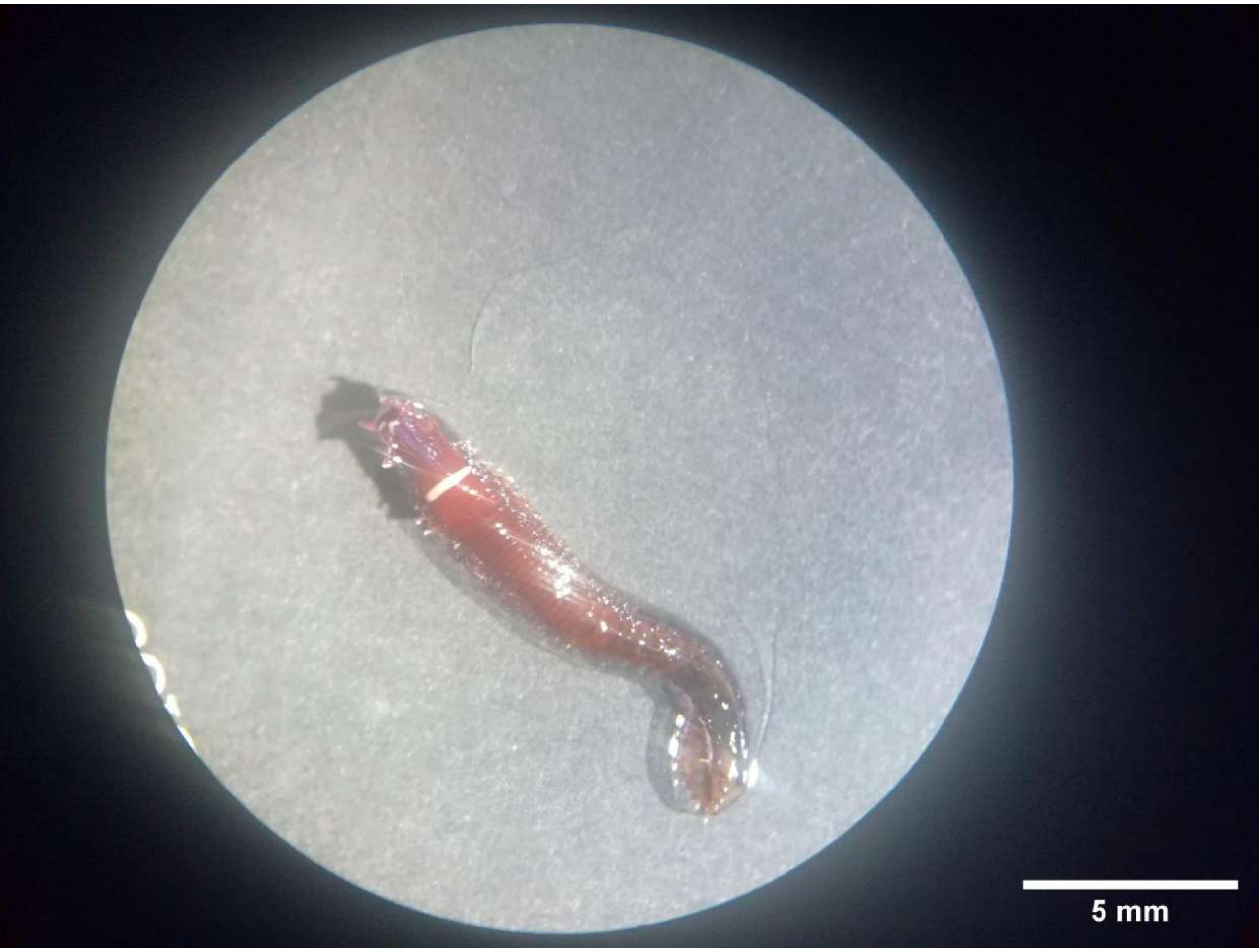
5 mm

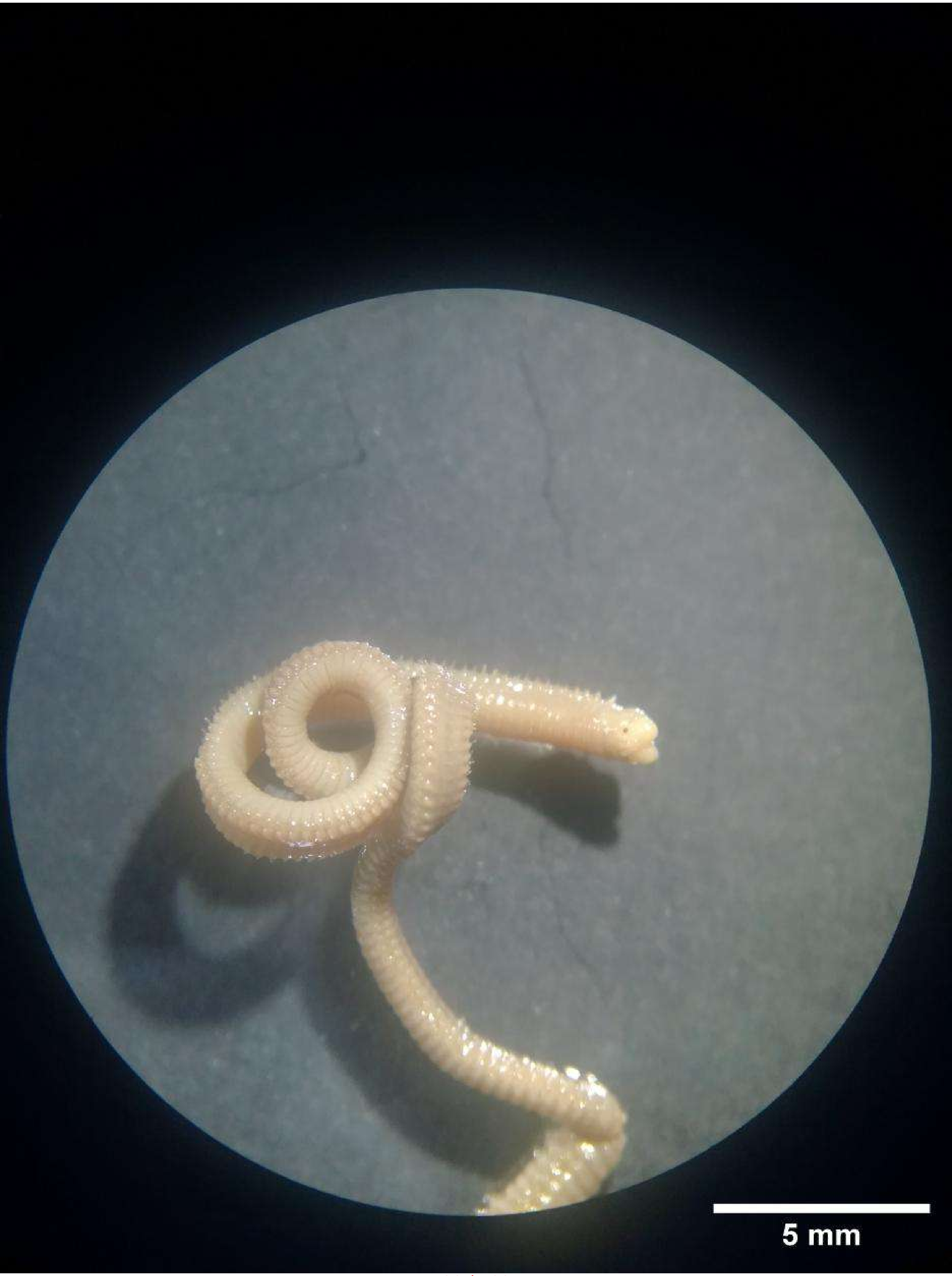
5 mm

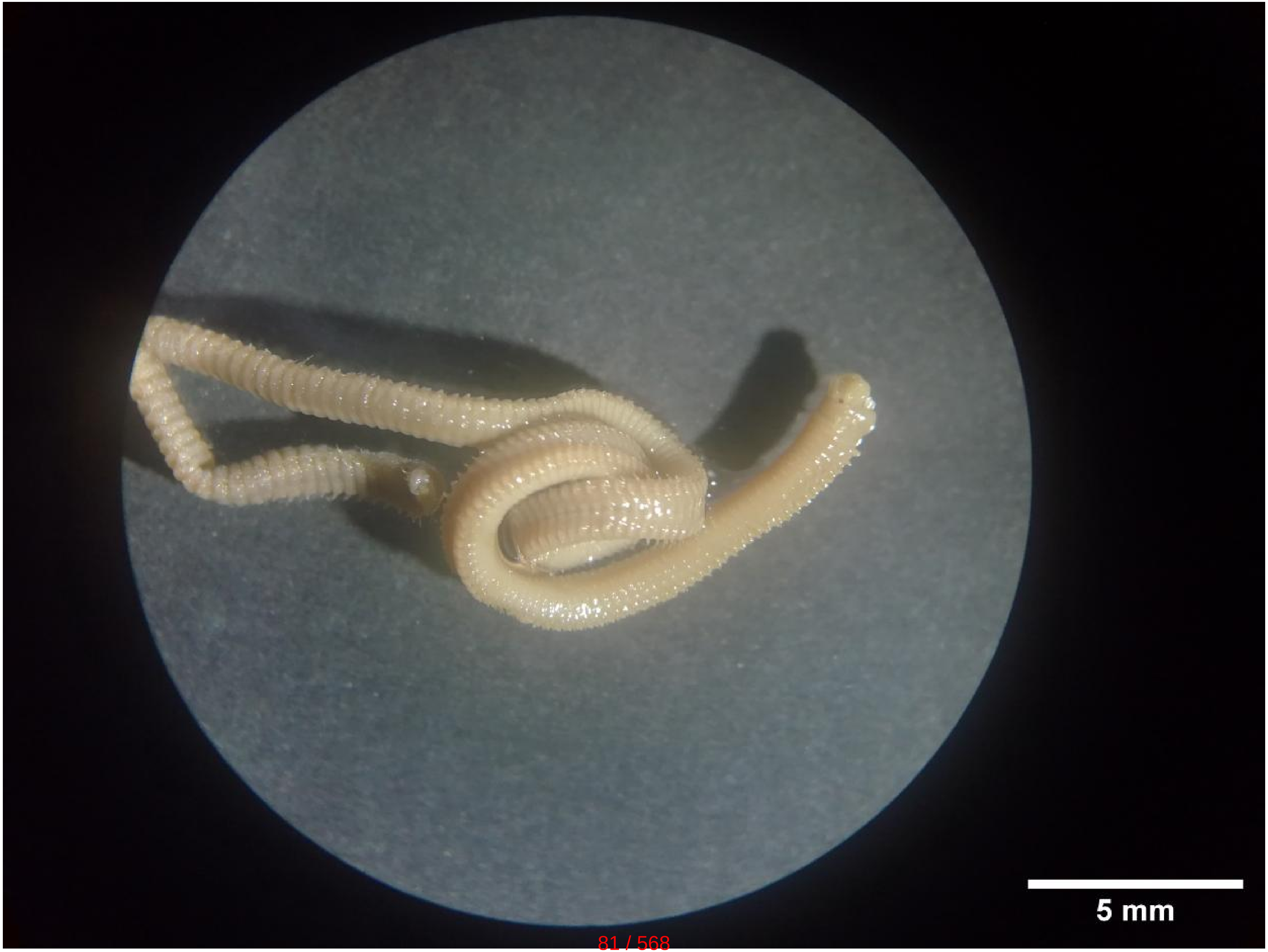
5 mm

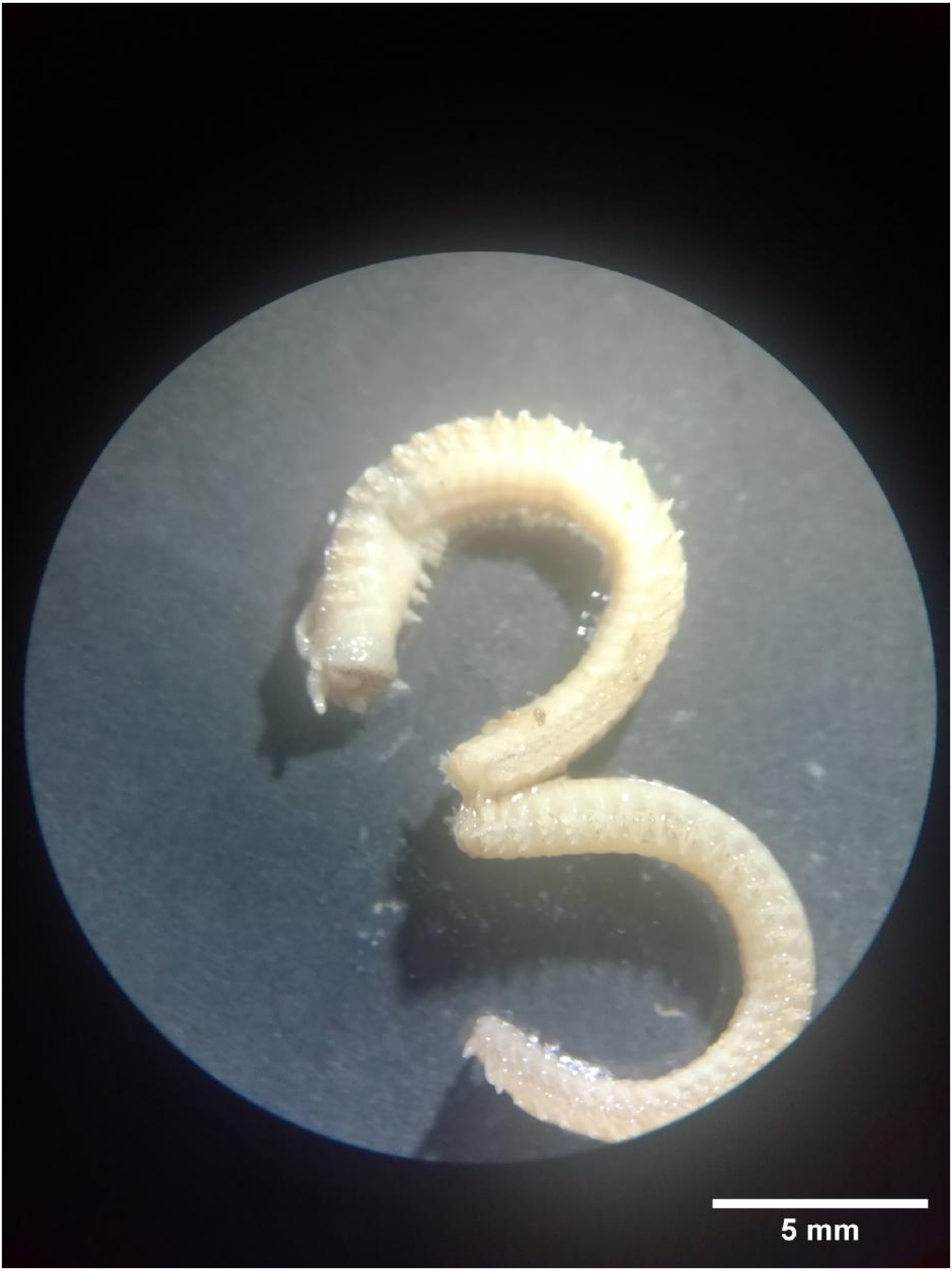
5 mm

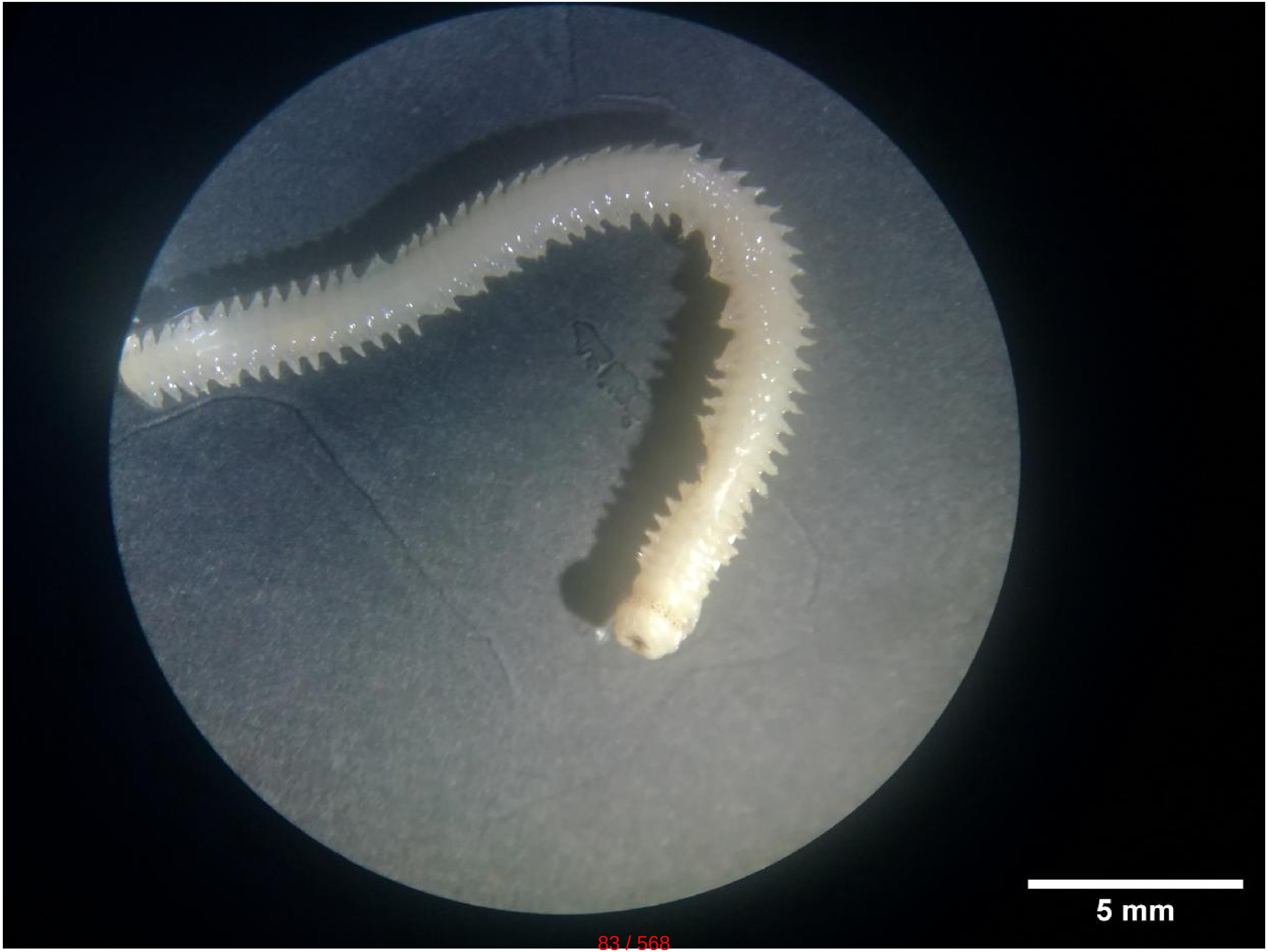
5 mm

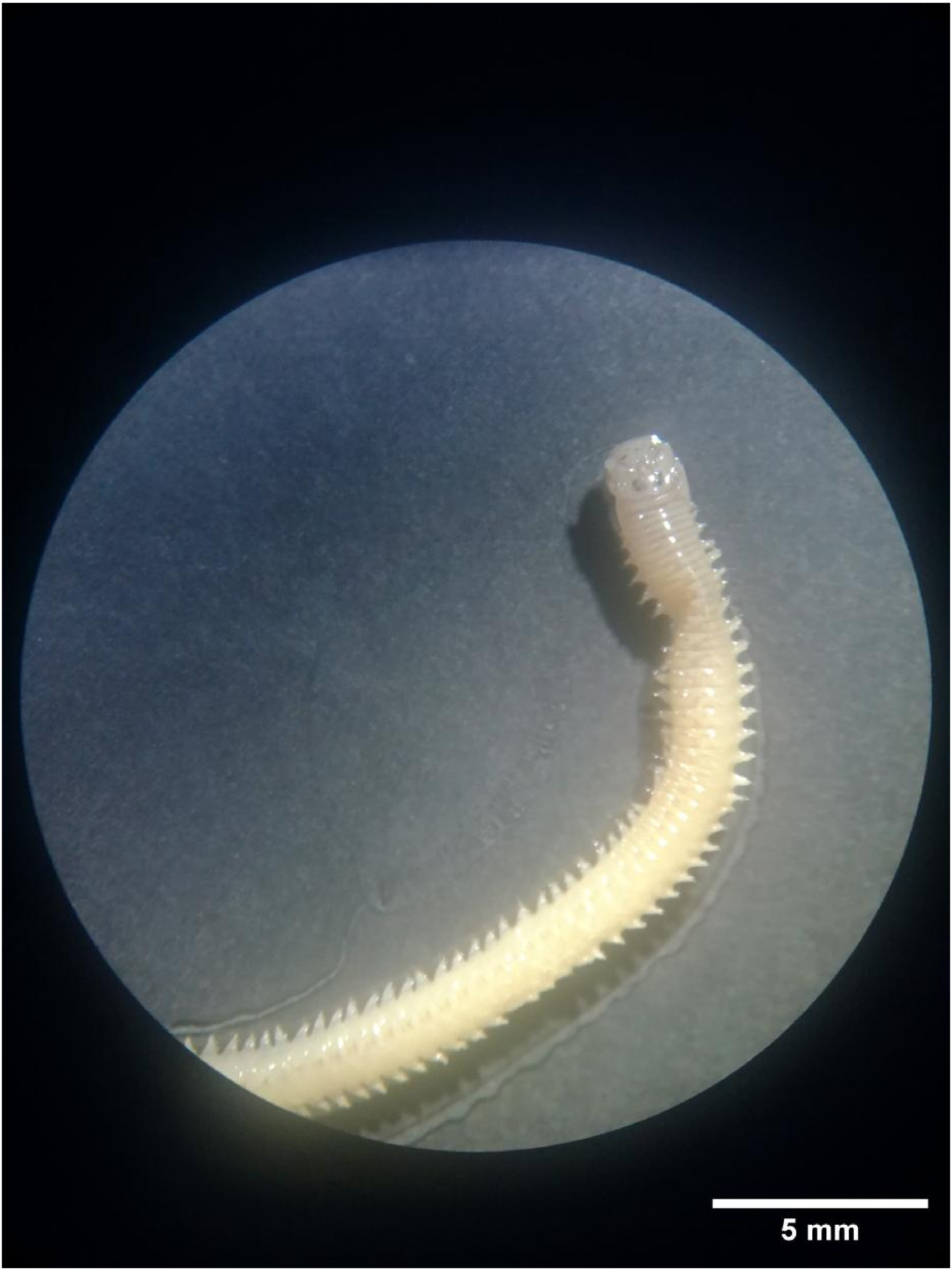
5 mm

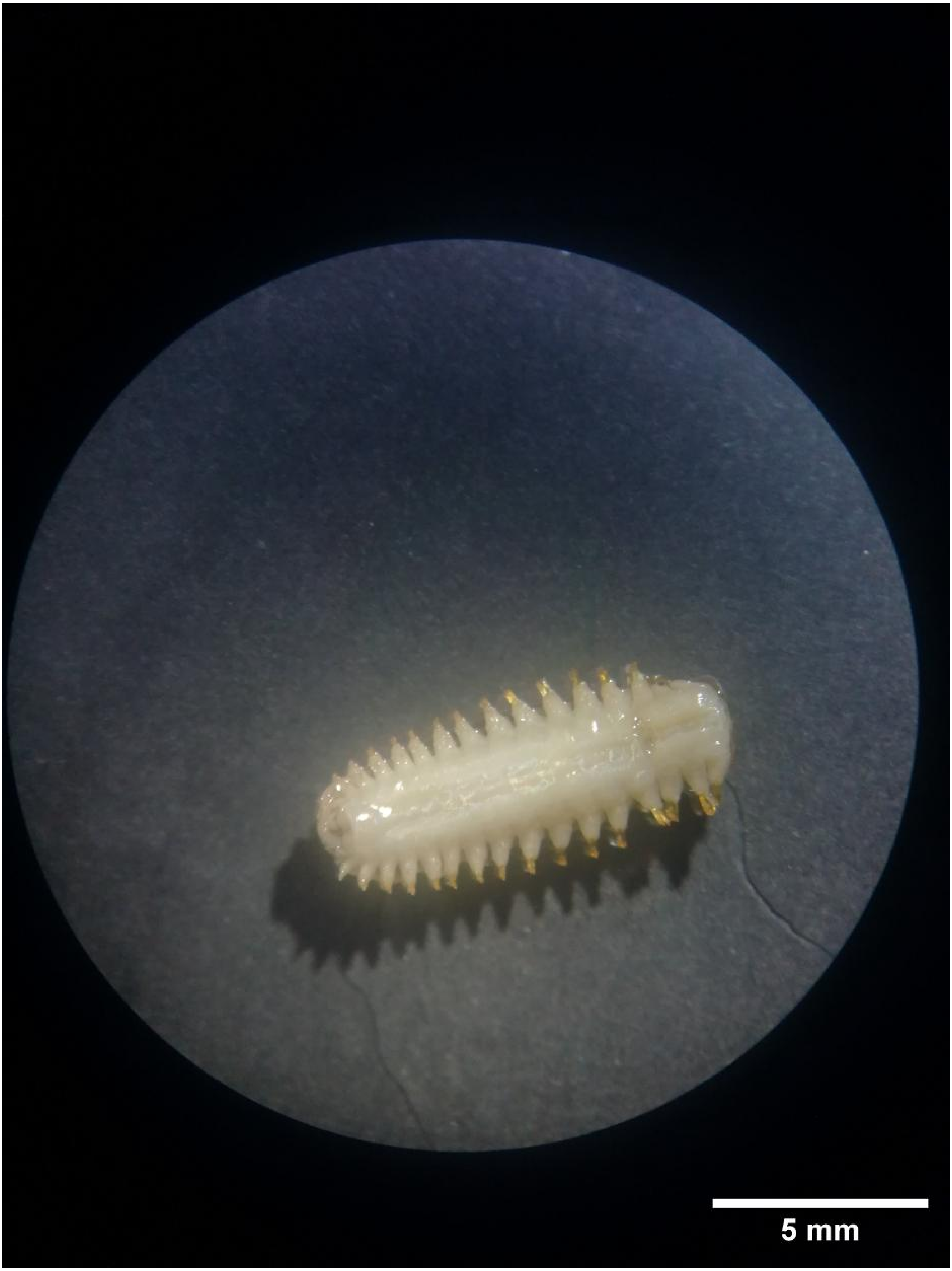
5 mm

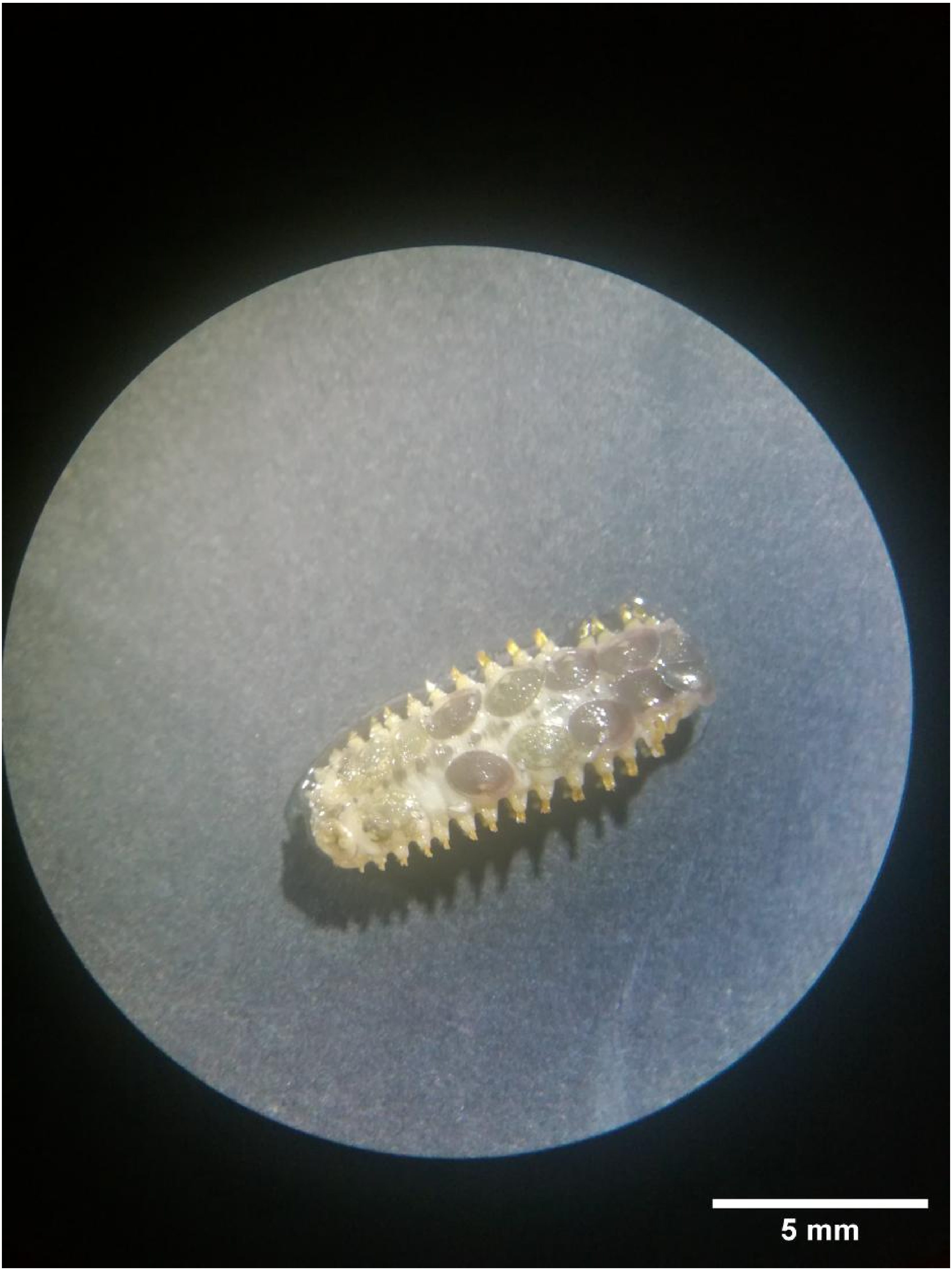
5 mm

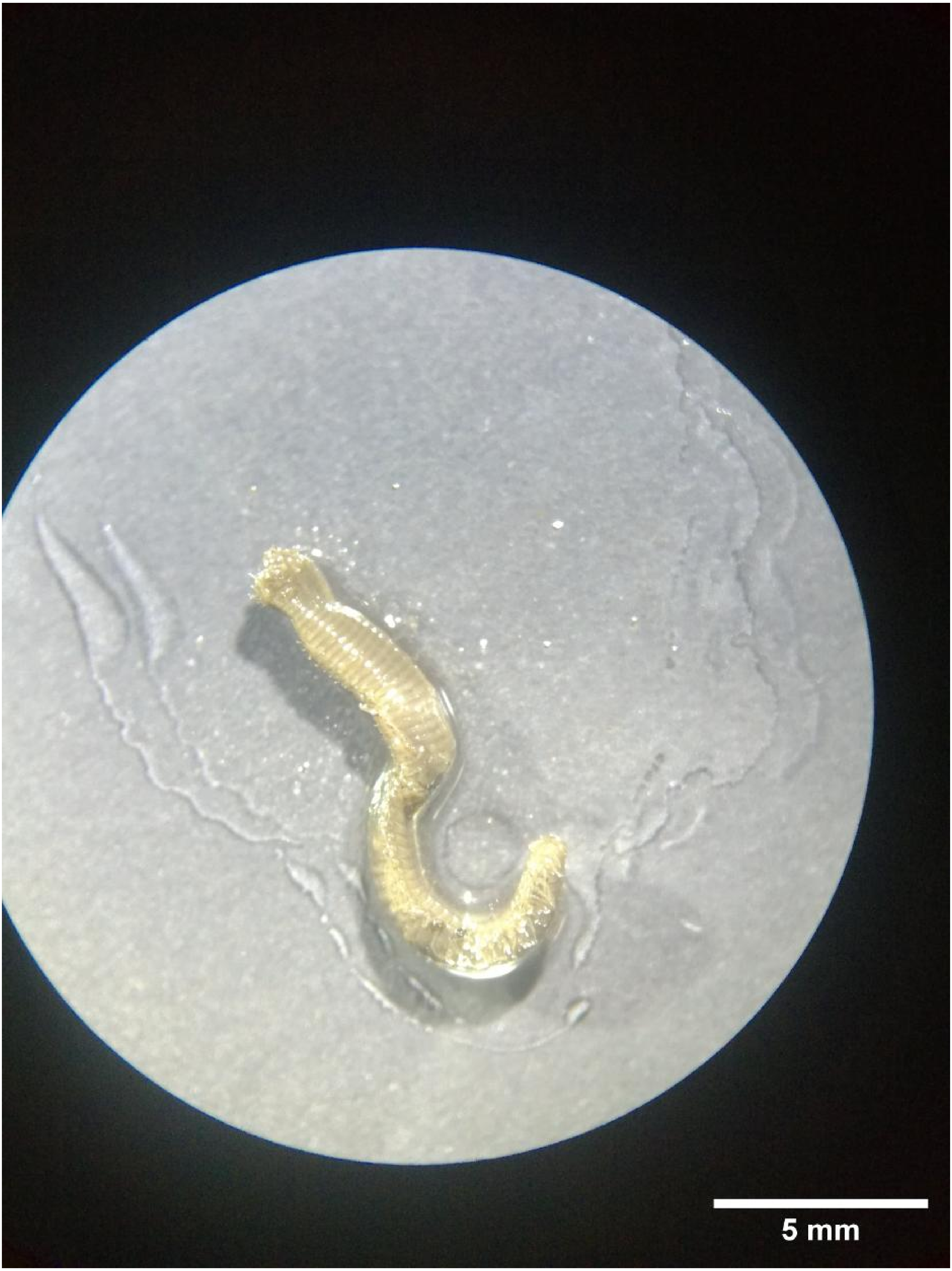
5 mm

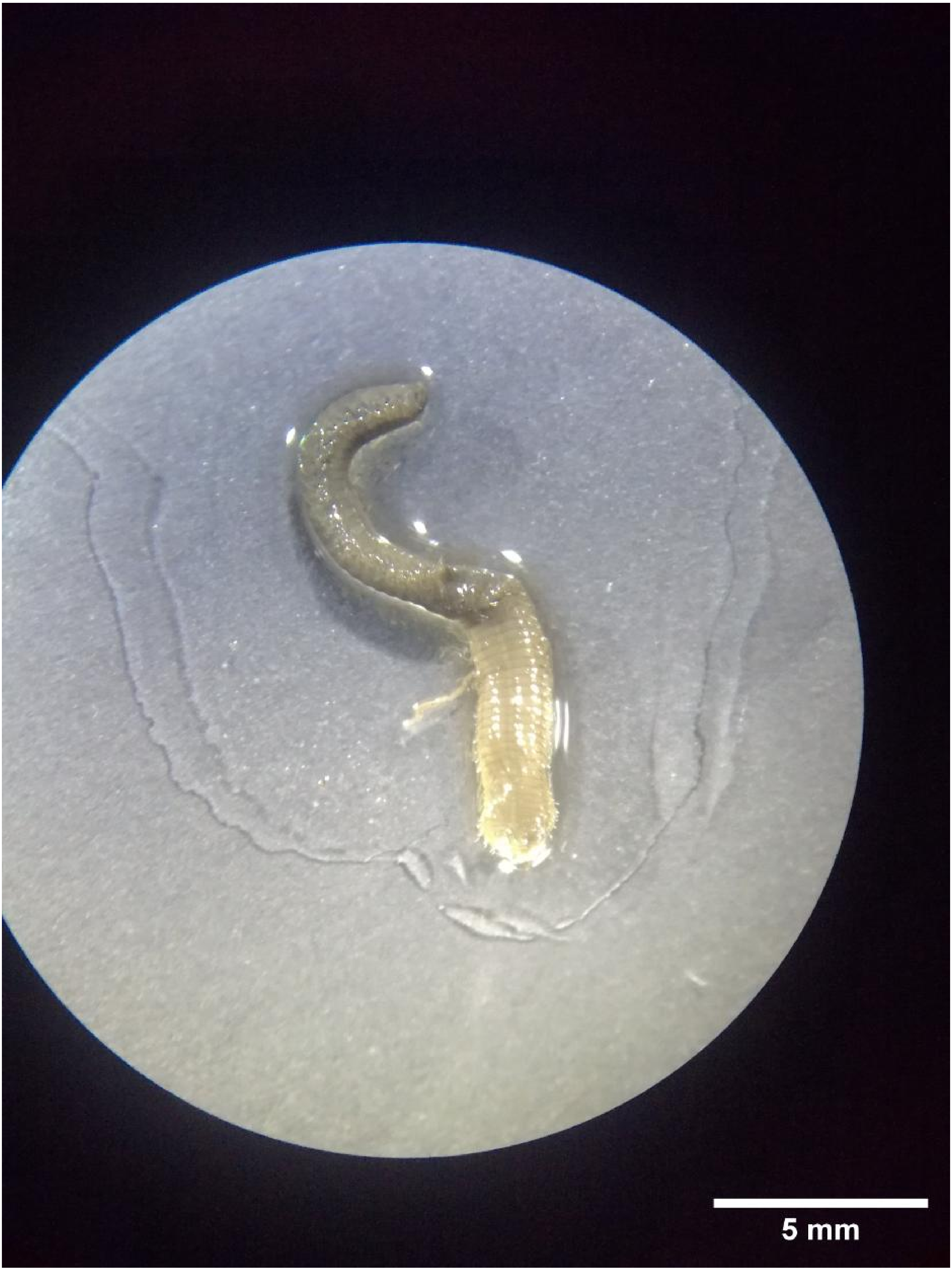
5 mm

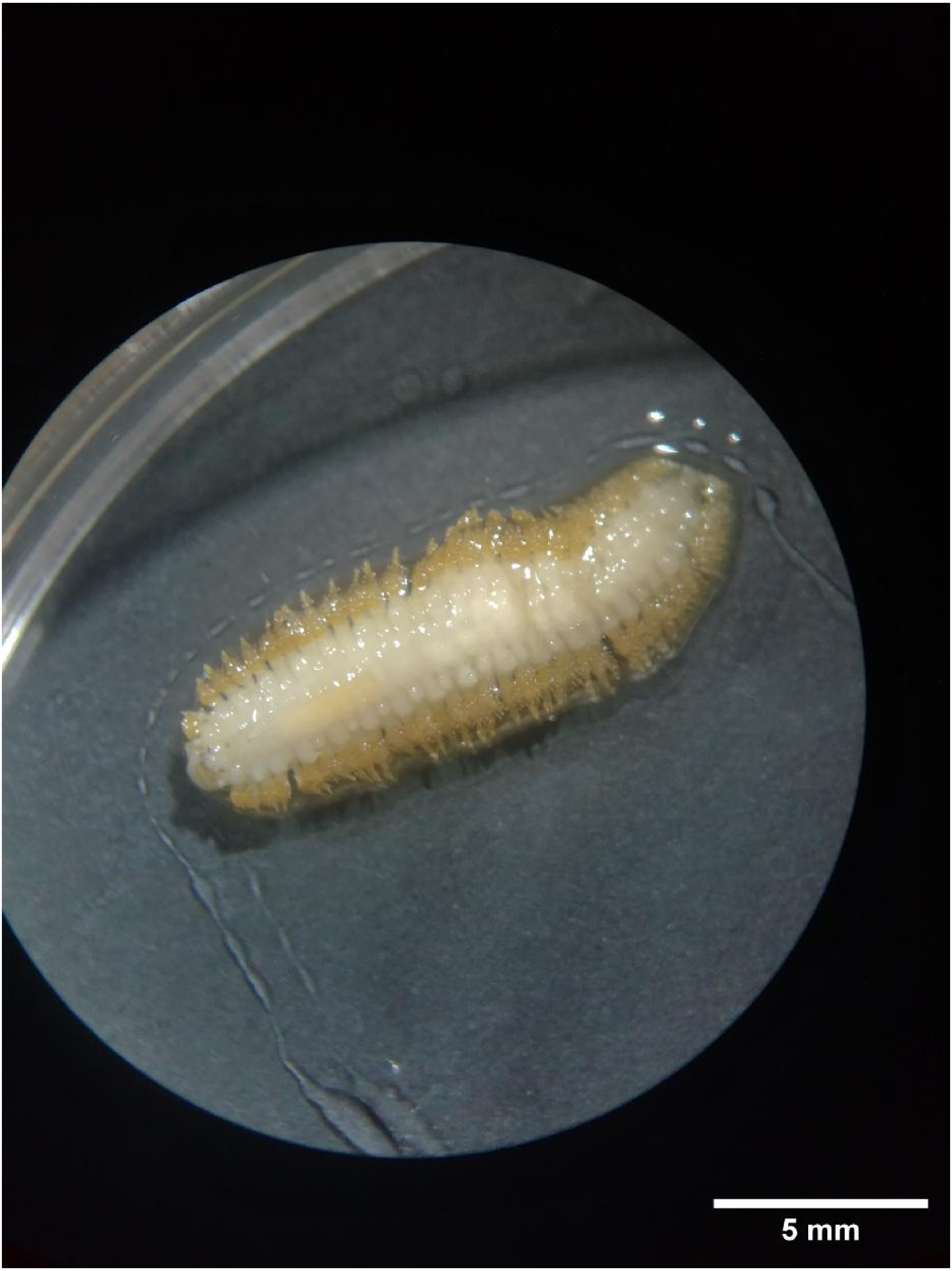
5 mm

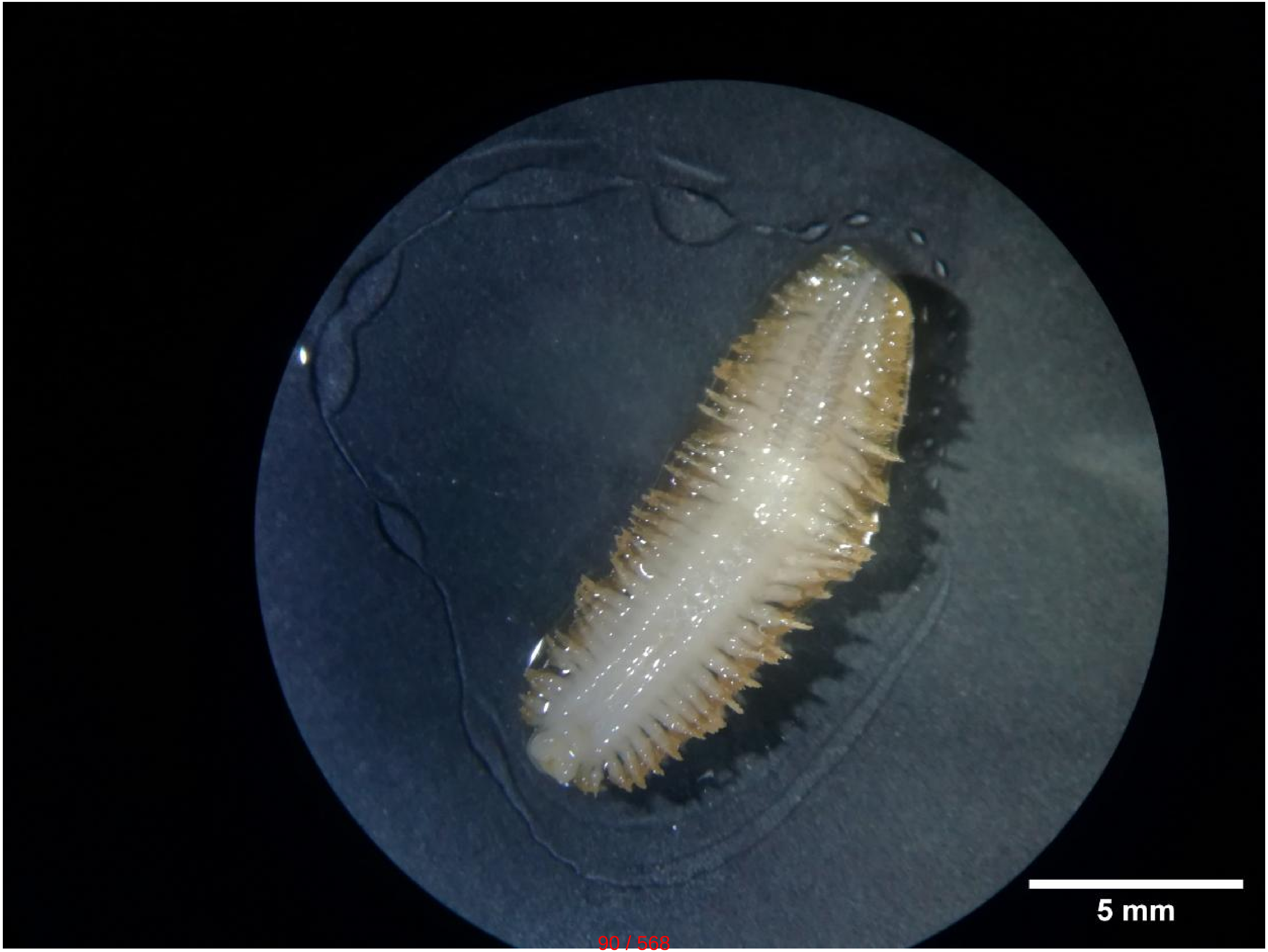
5 mm

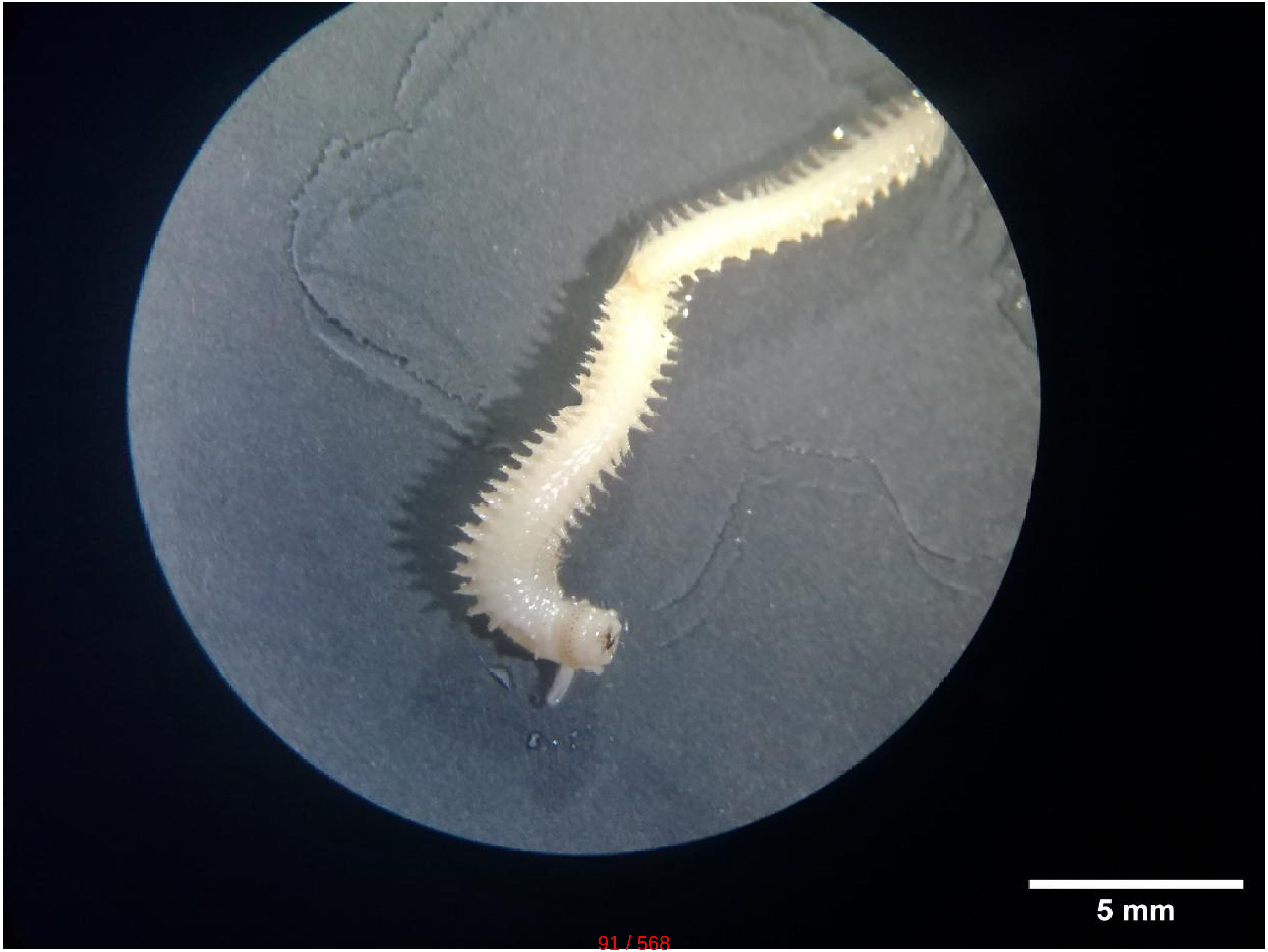
5 mm

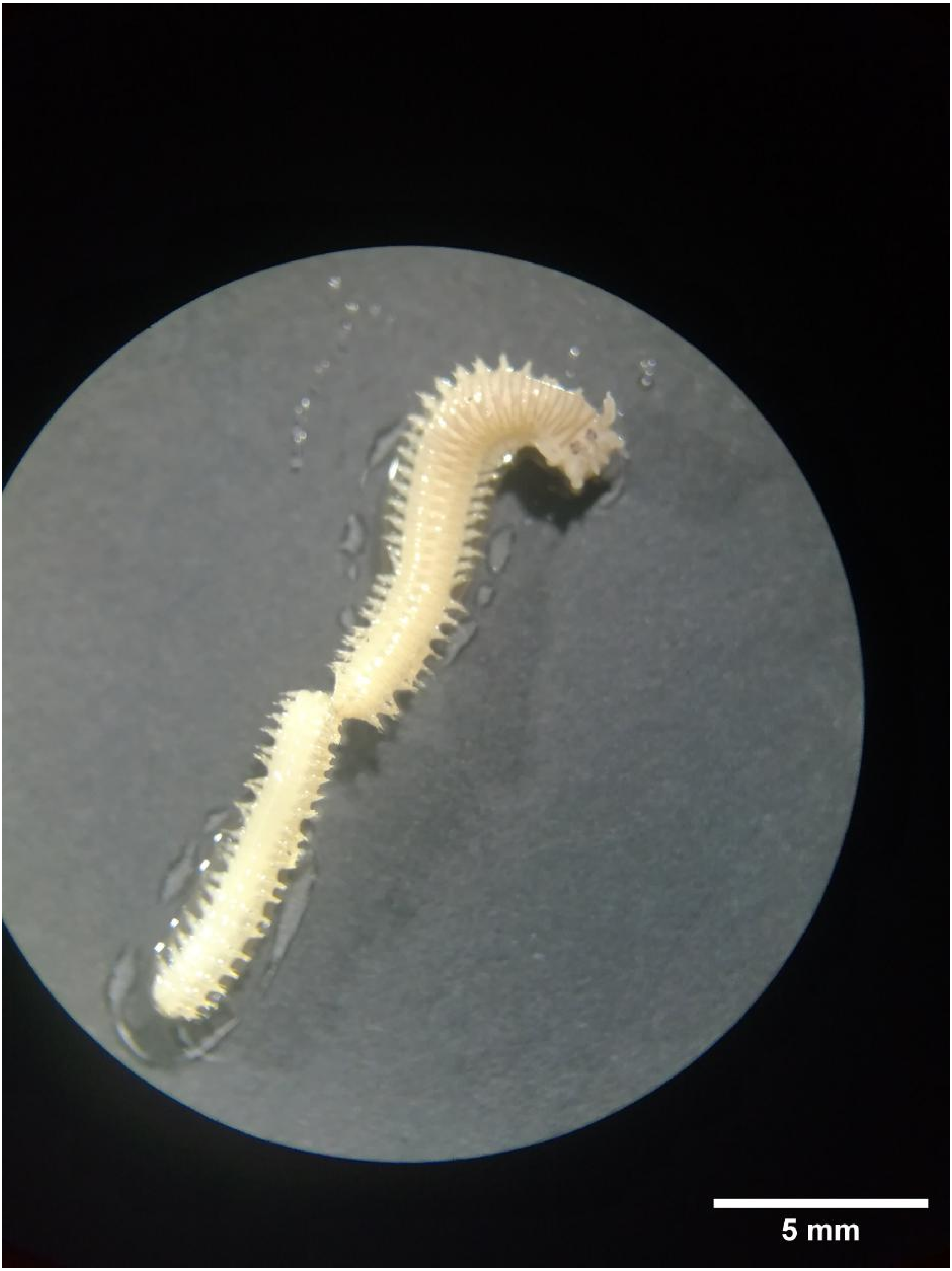
5 mm

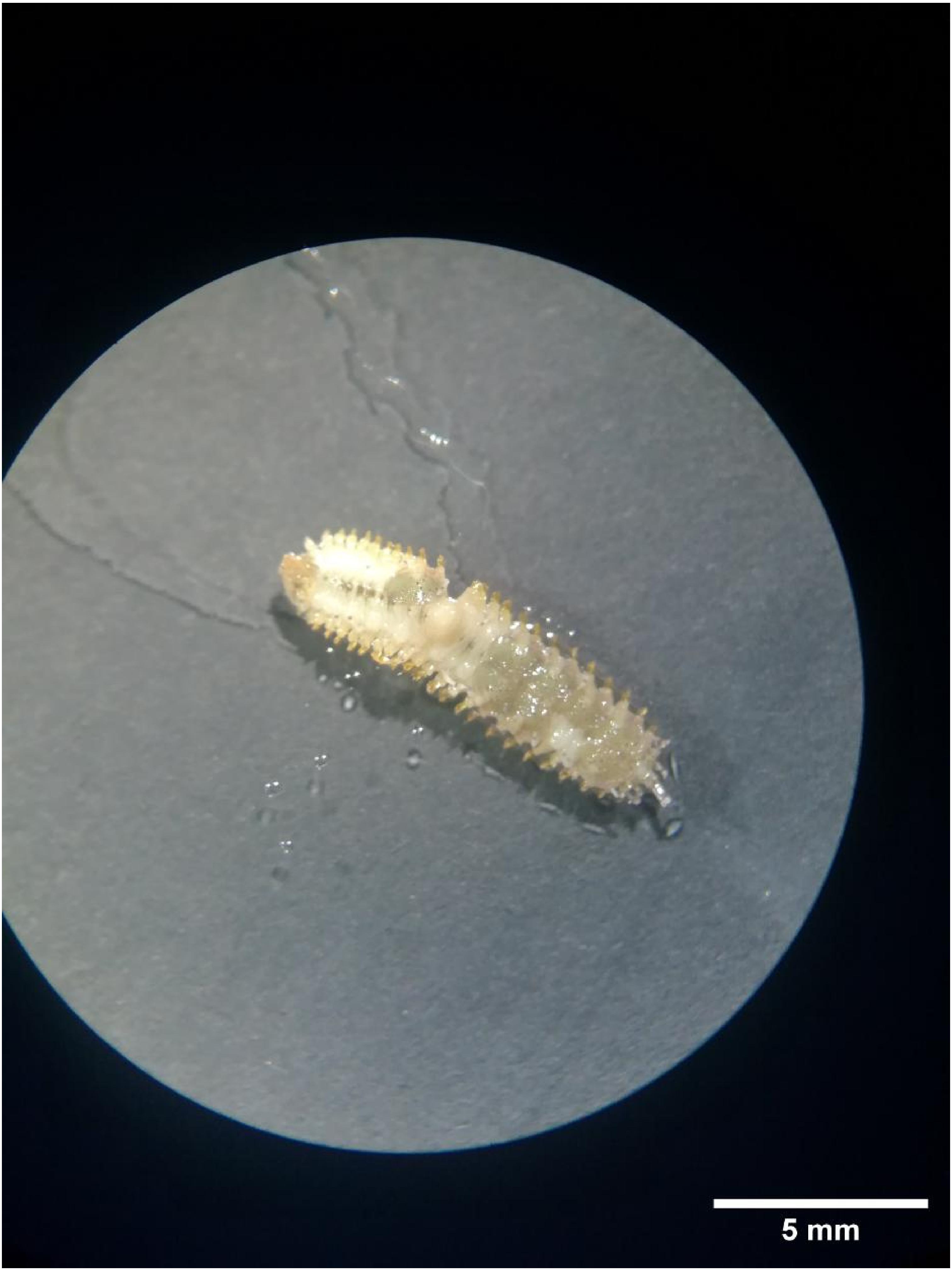
5 mm

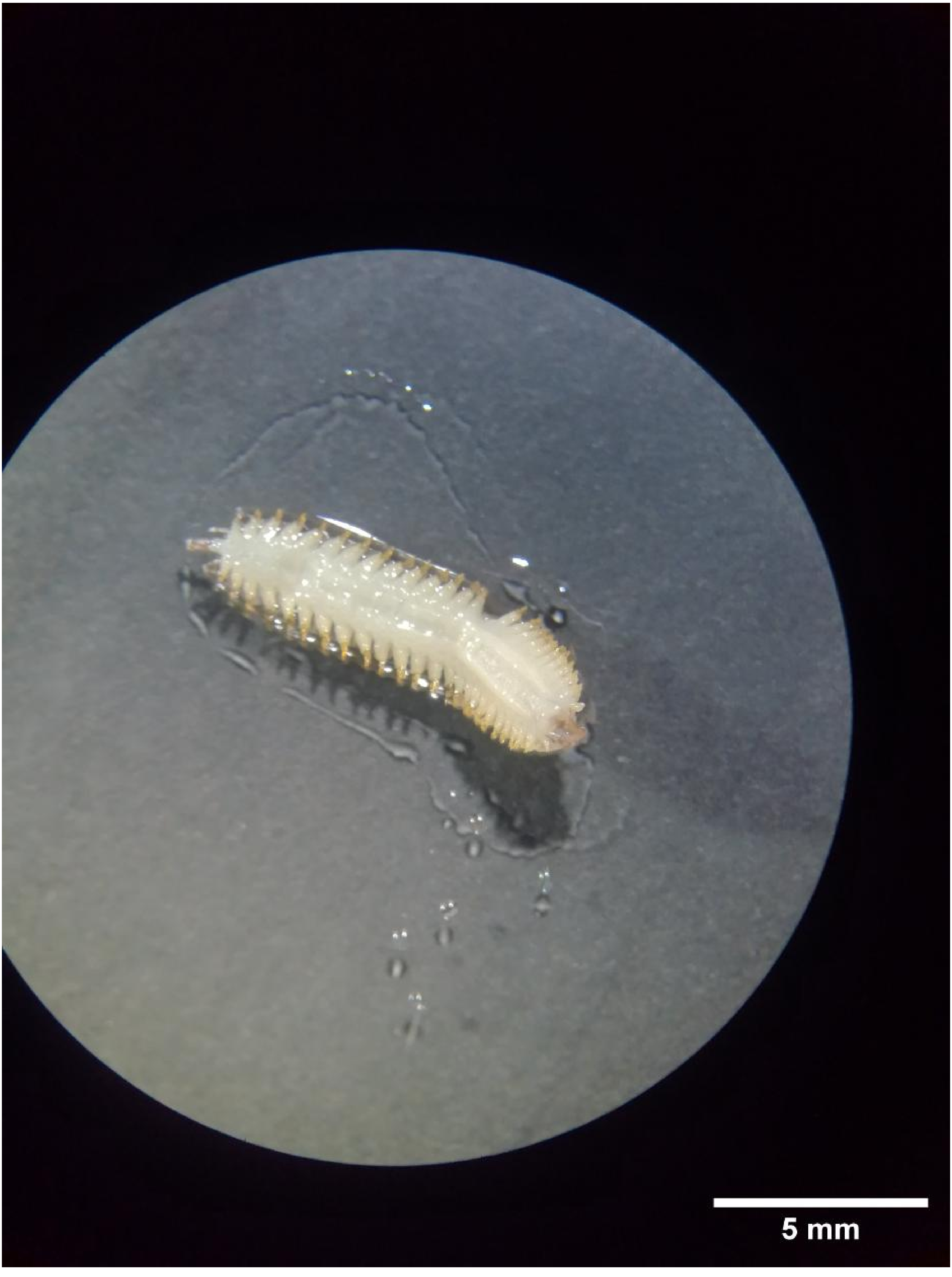
5 mm

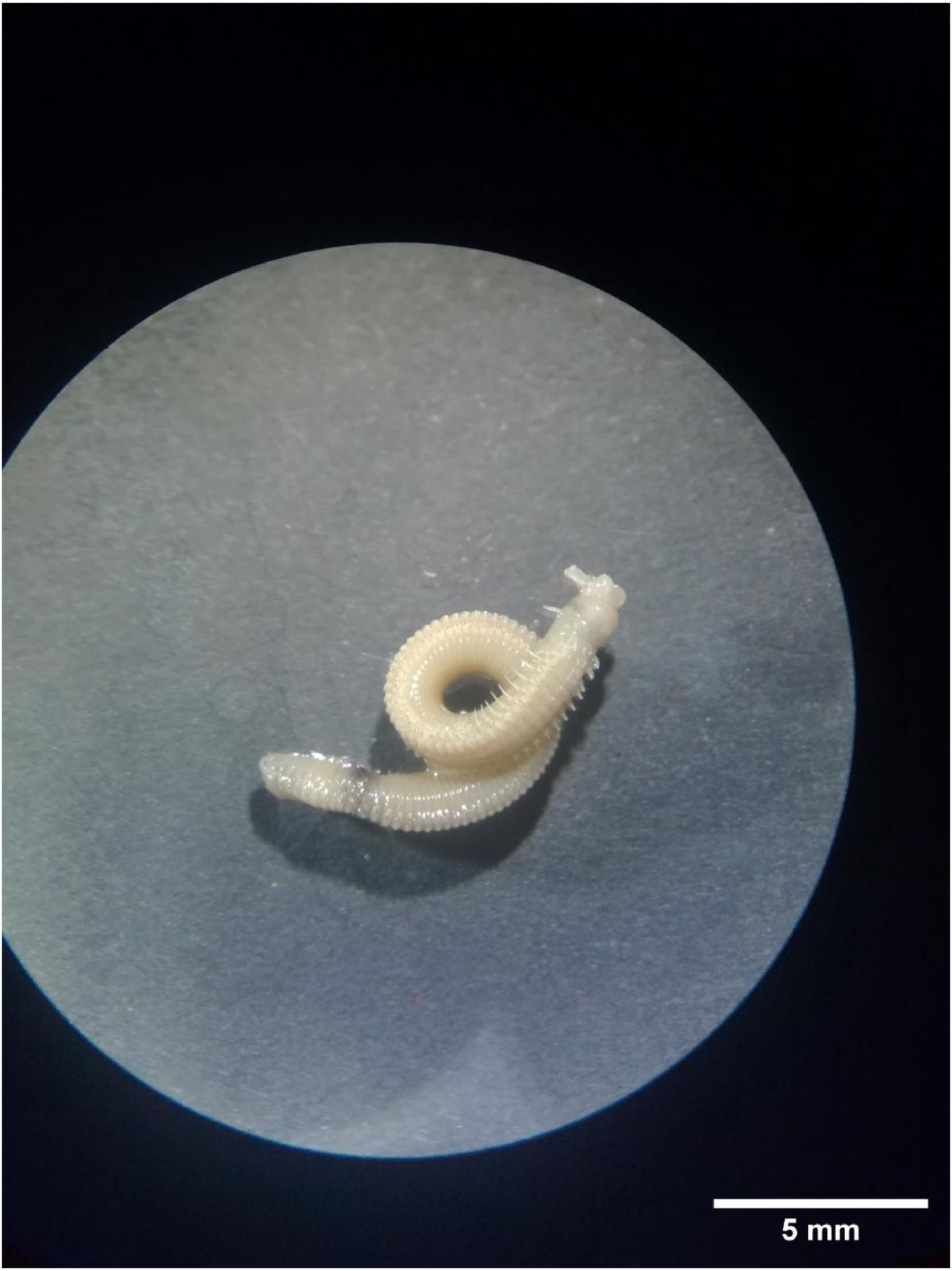
5 mm

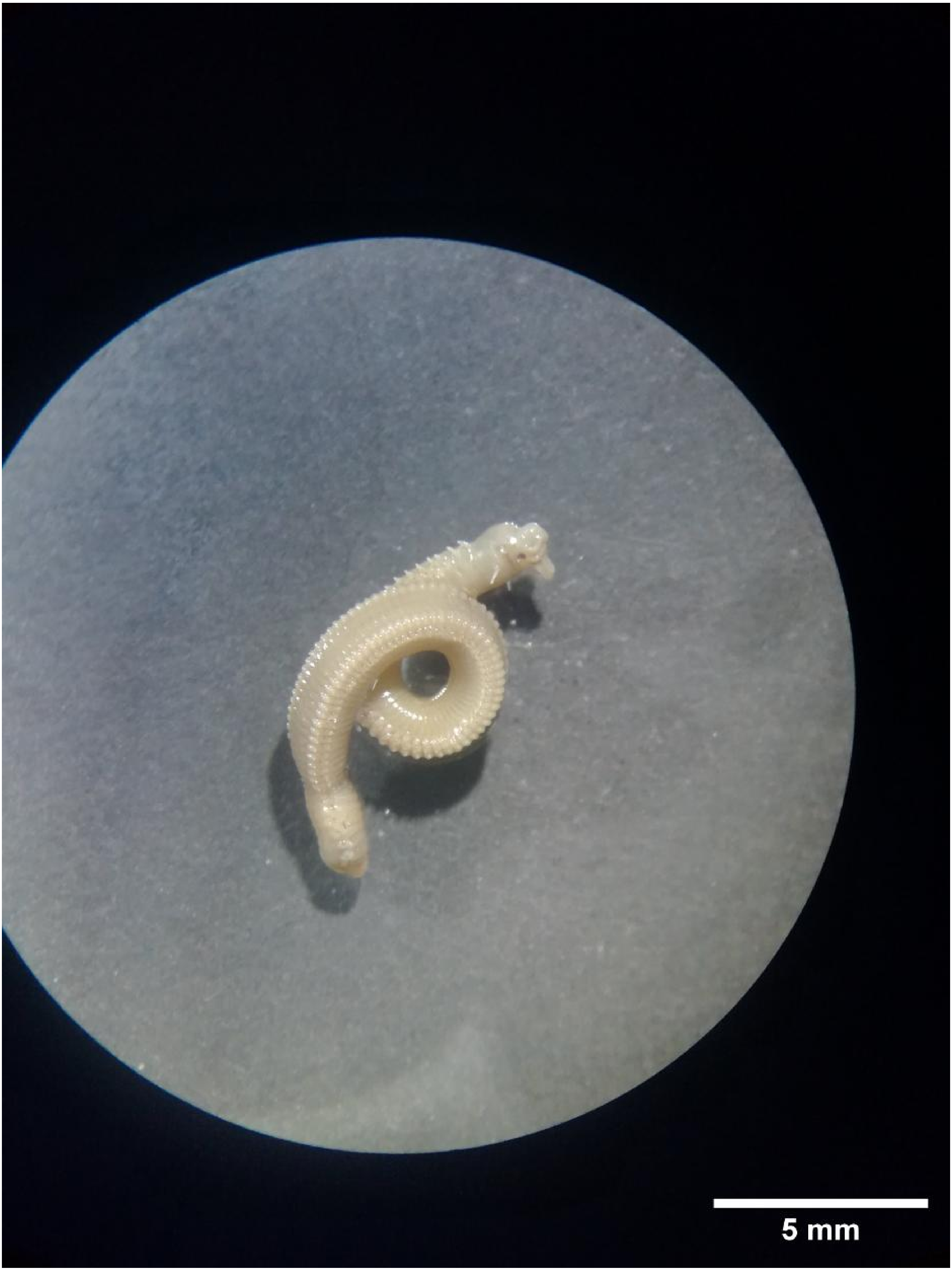
5 mm

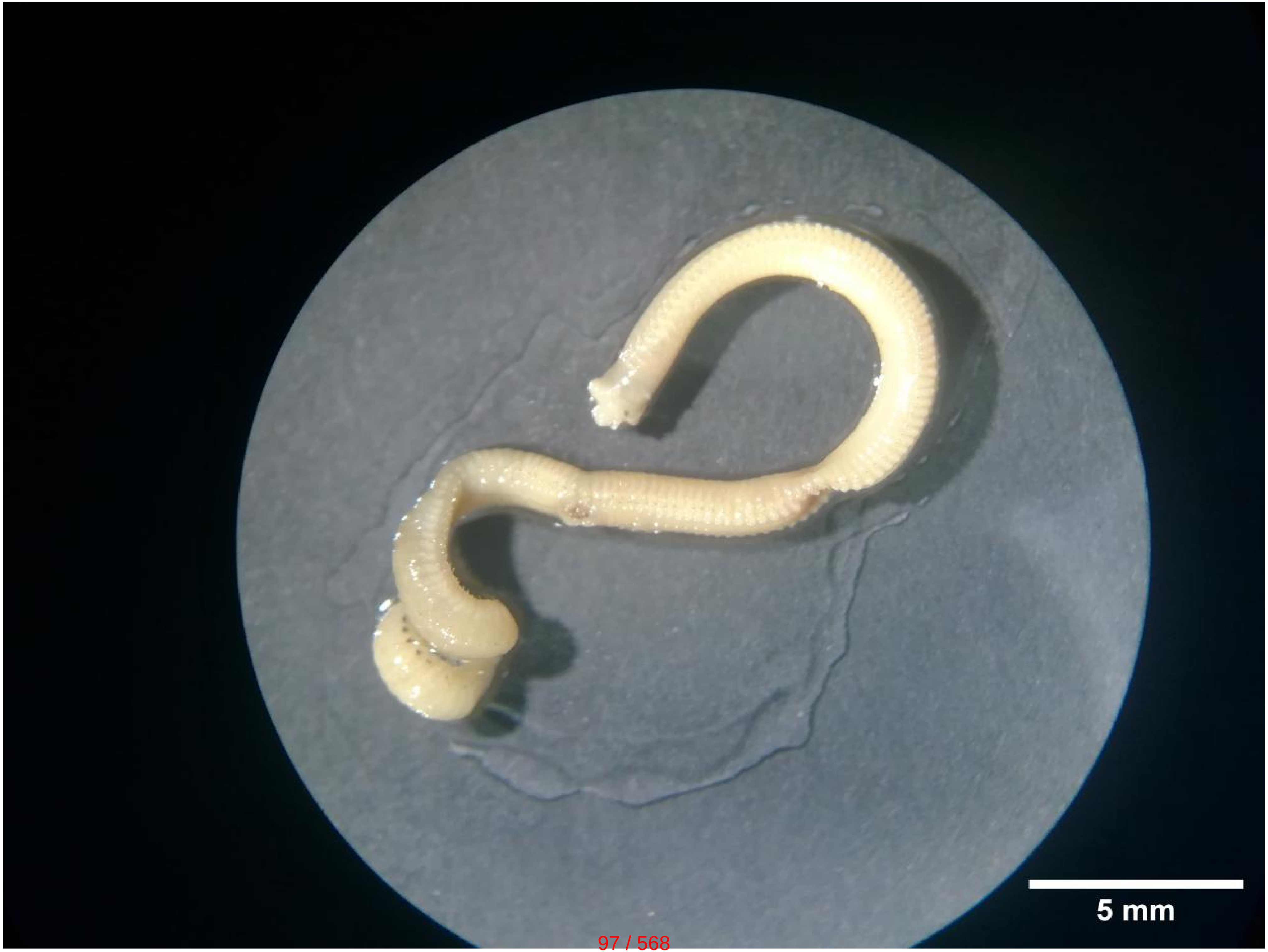
5 mm

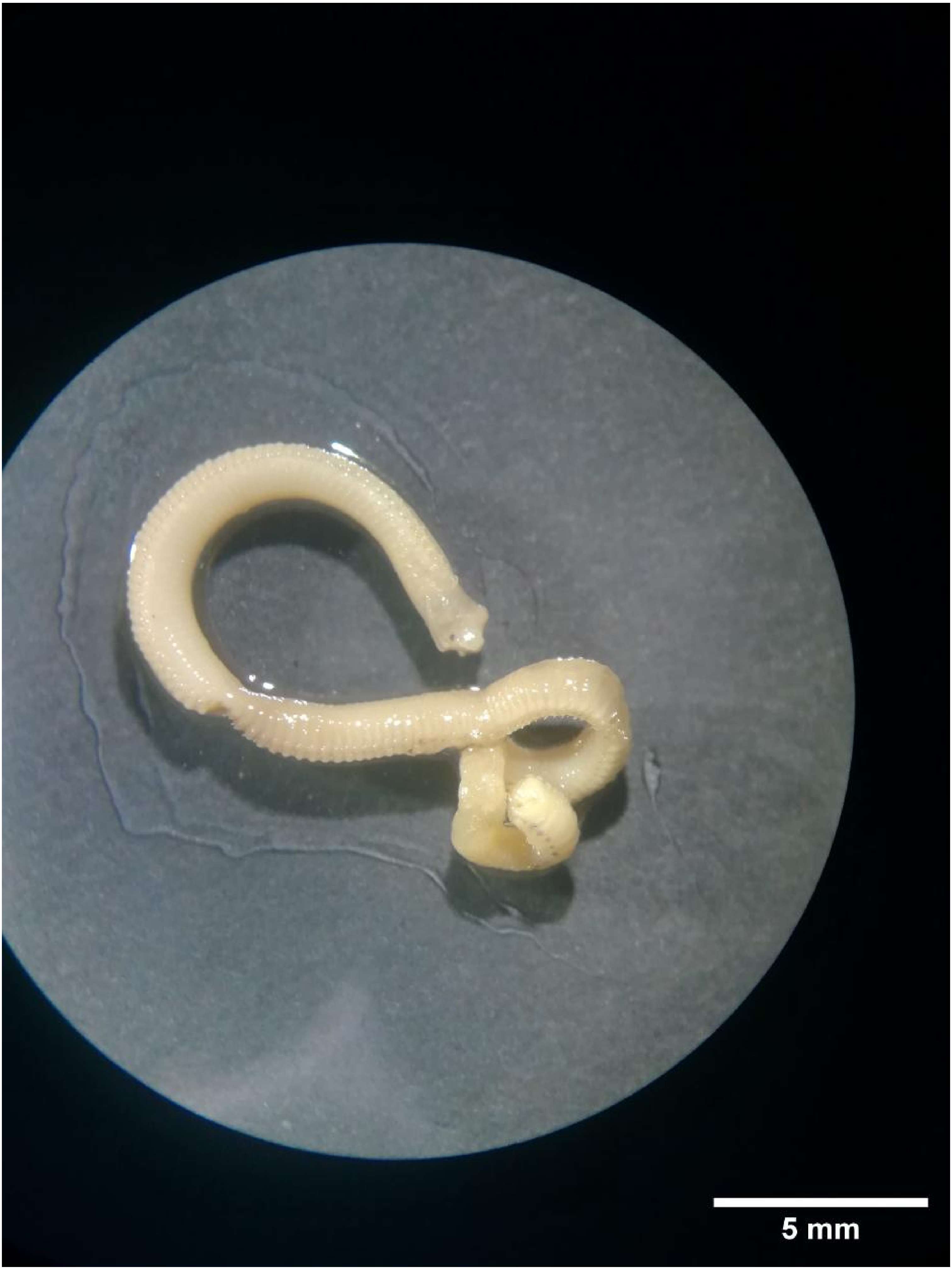
5 mm

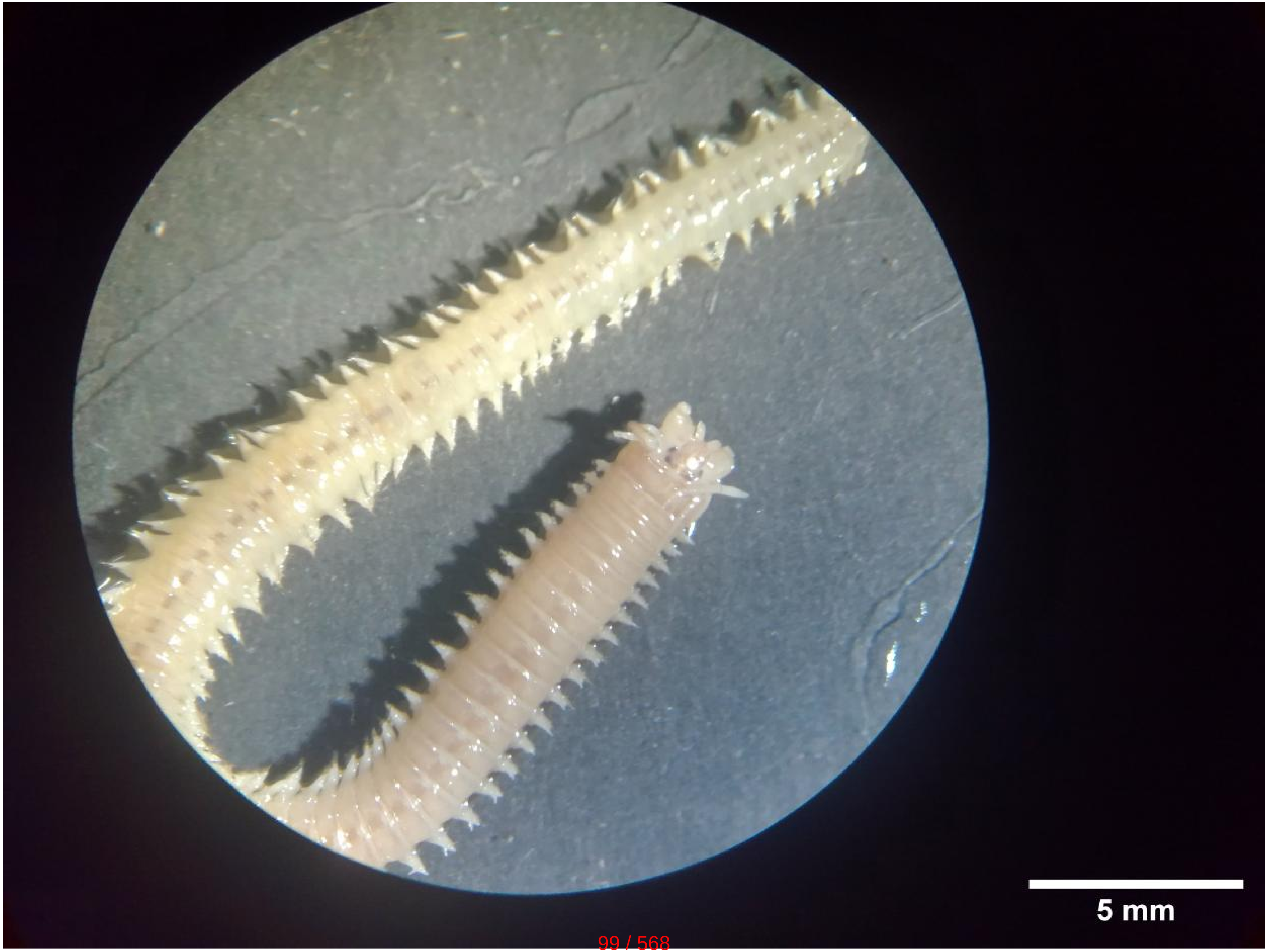
5 mm

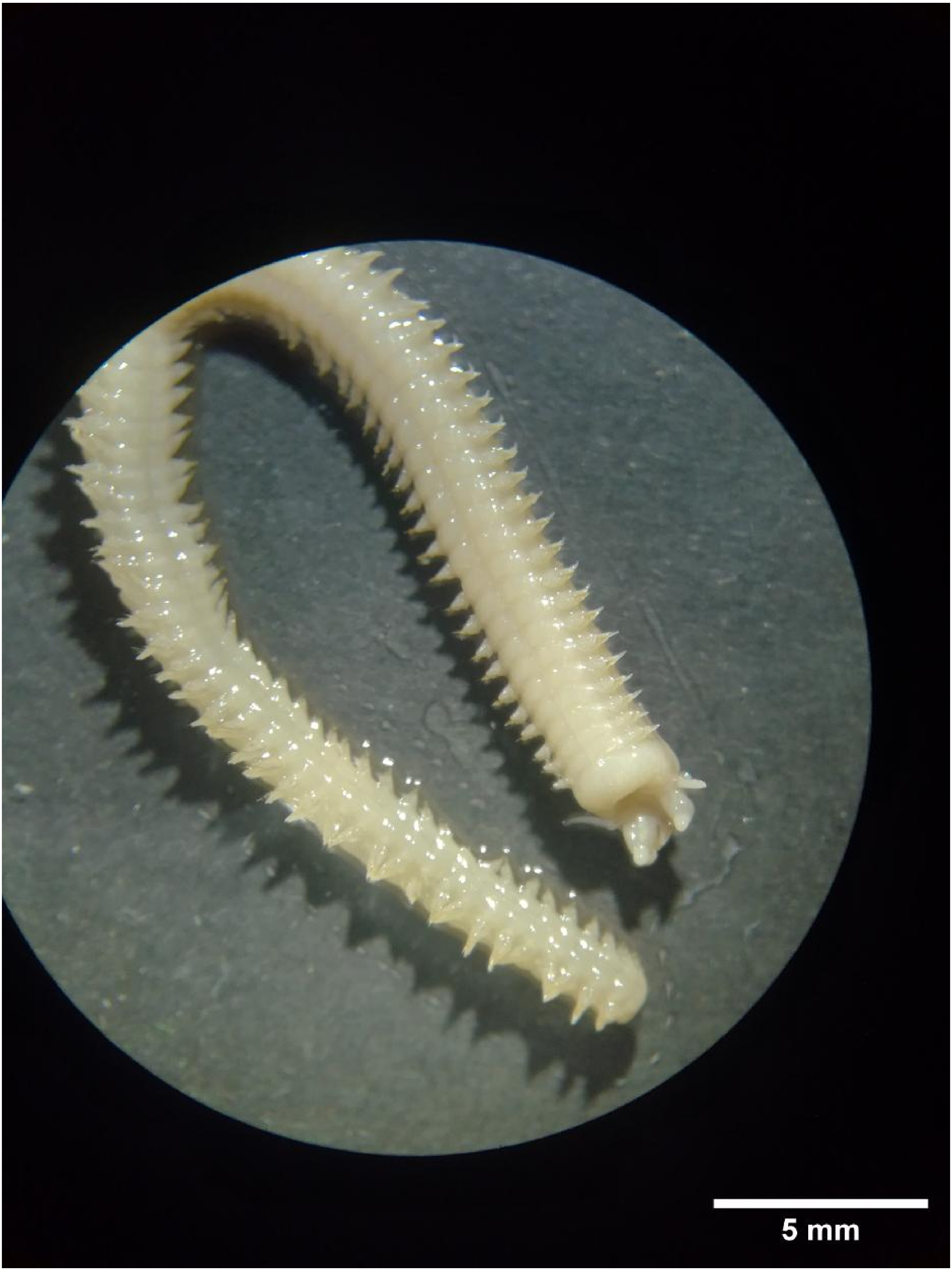
5 mm

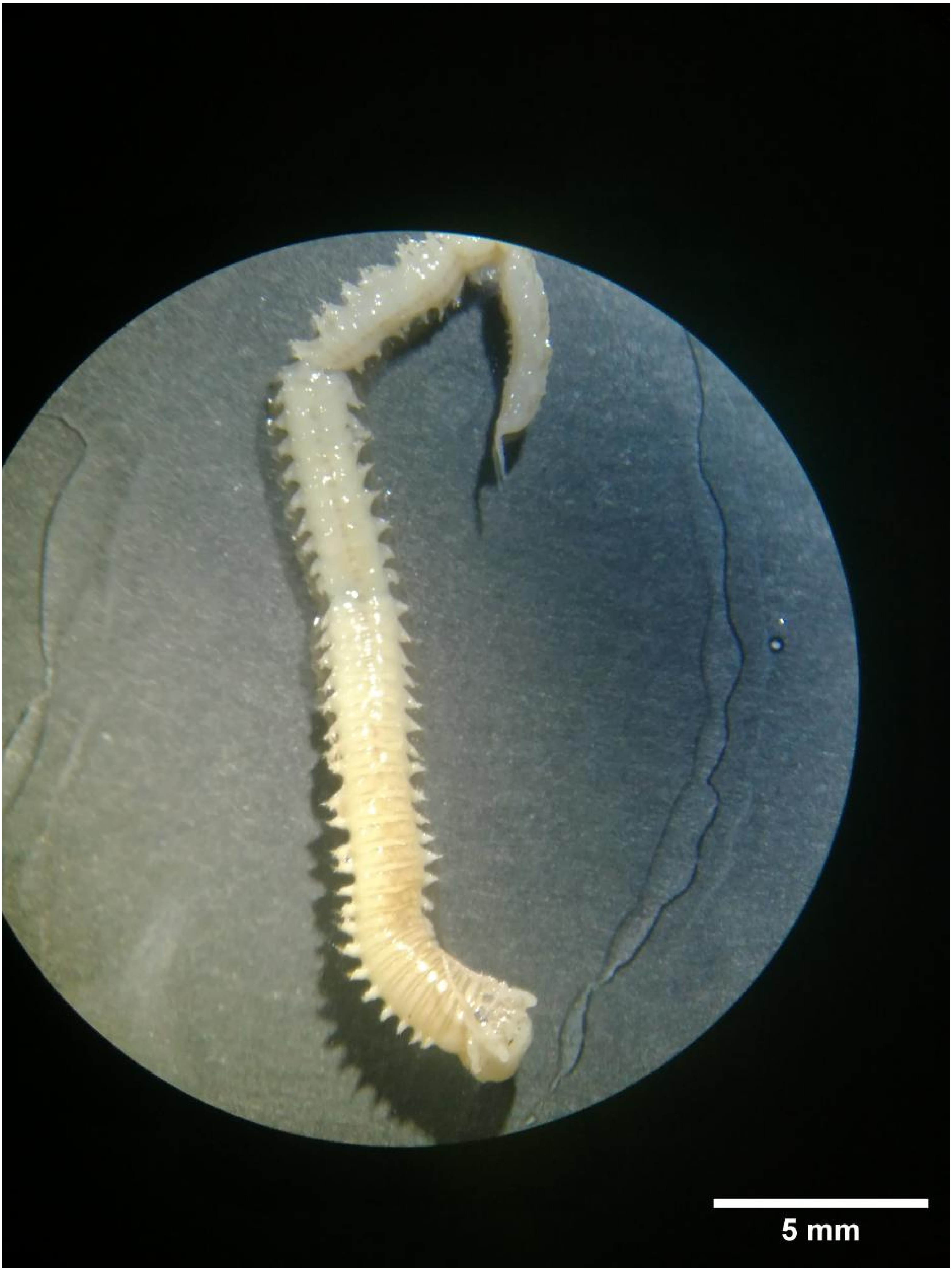
5 mm

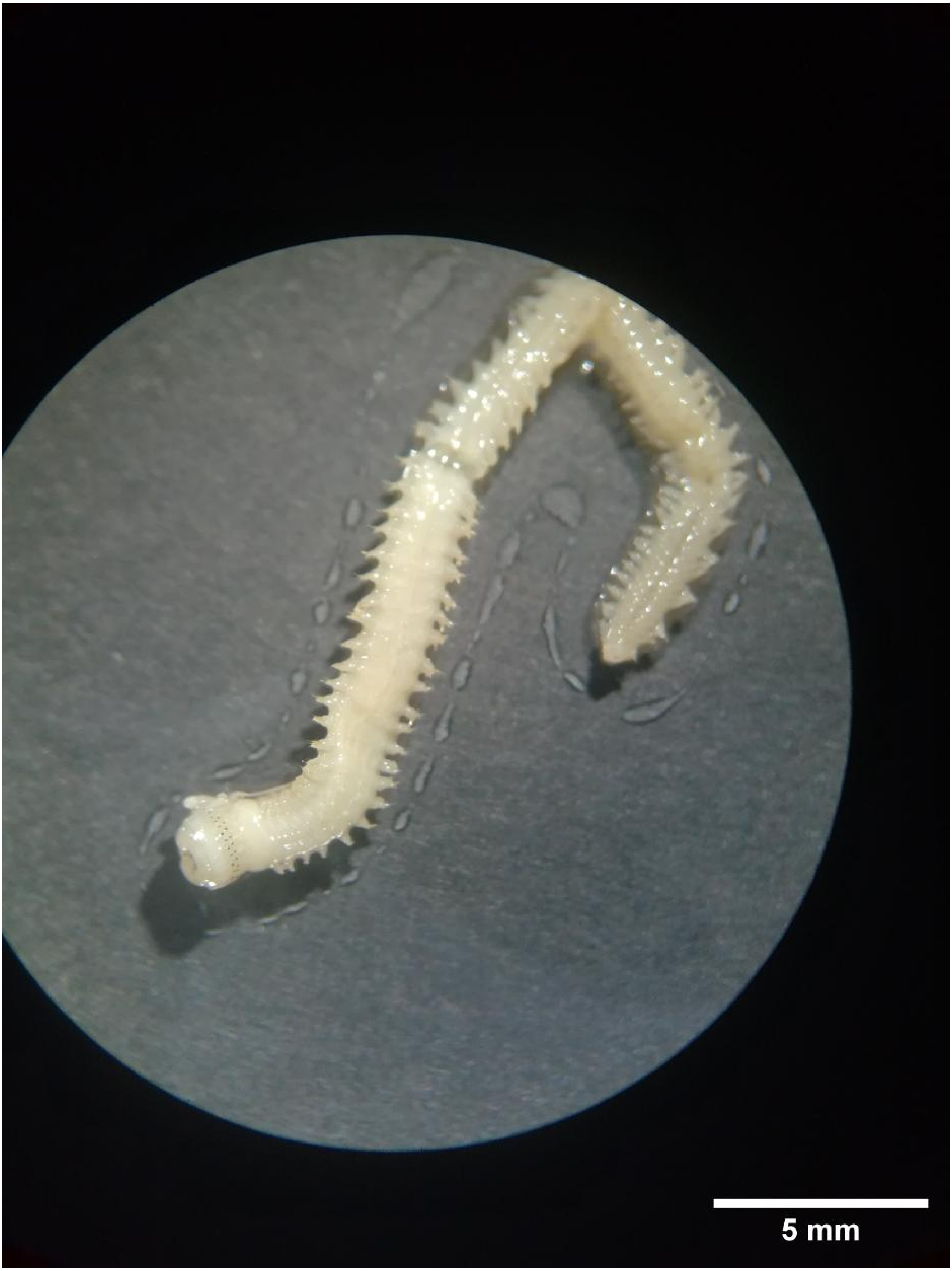
5 mm

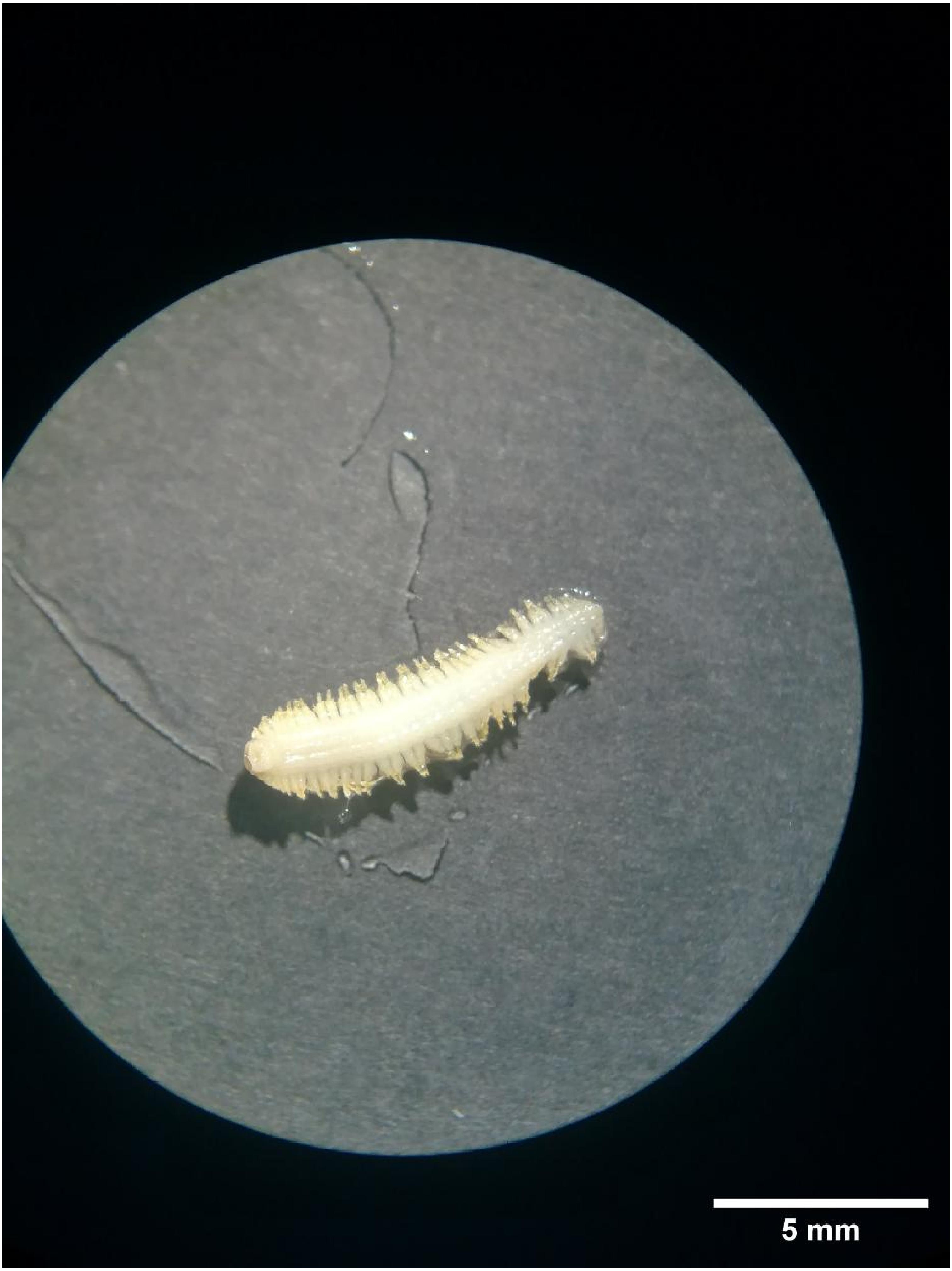
5 mm

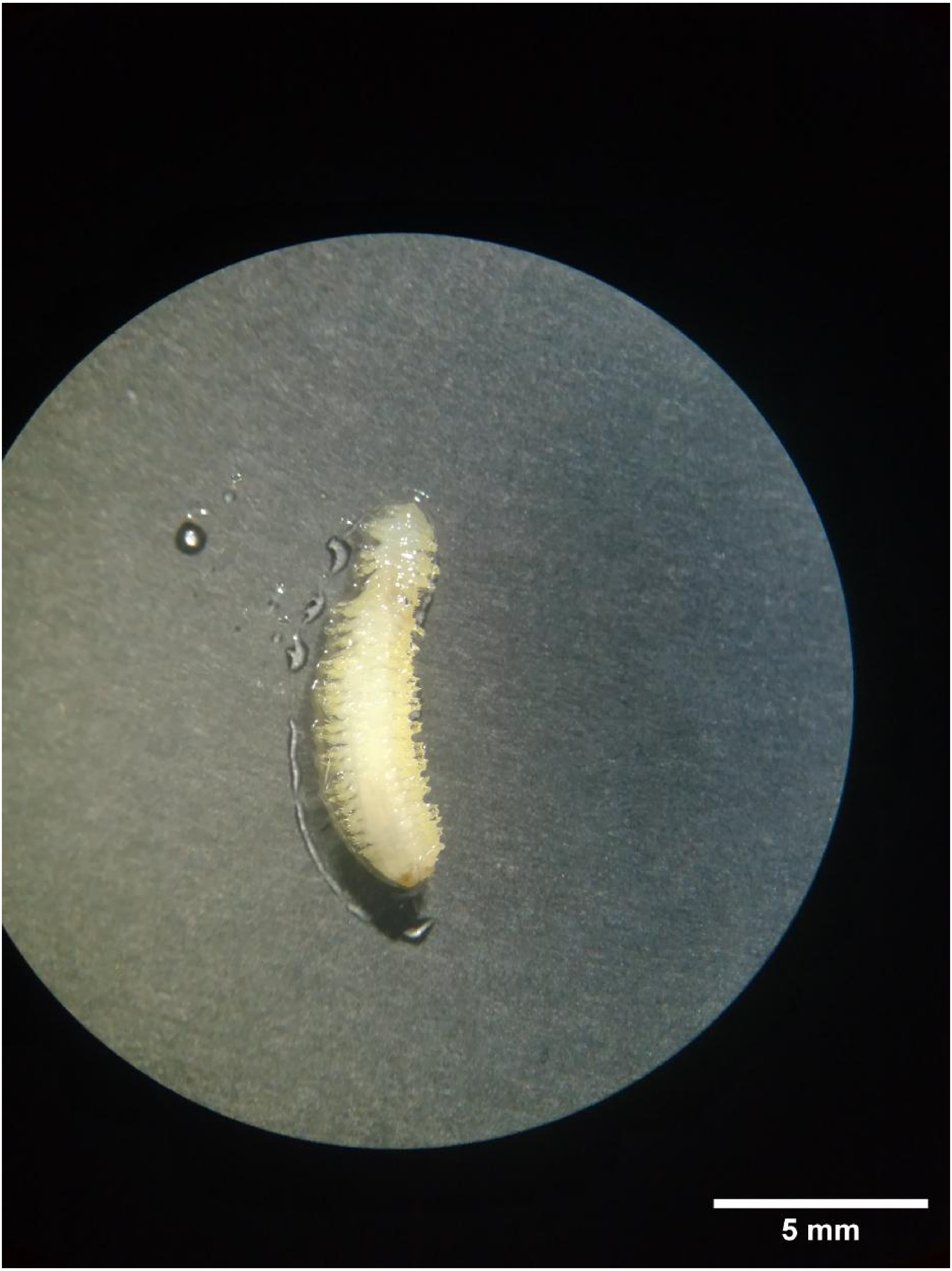
5 mm

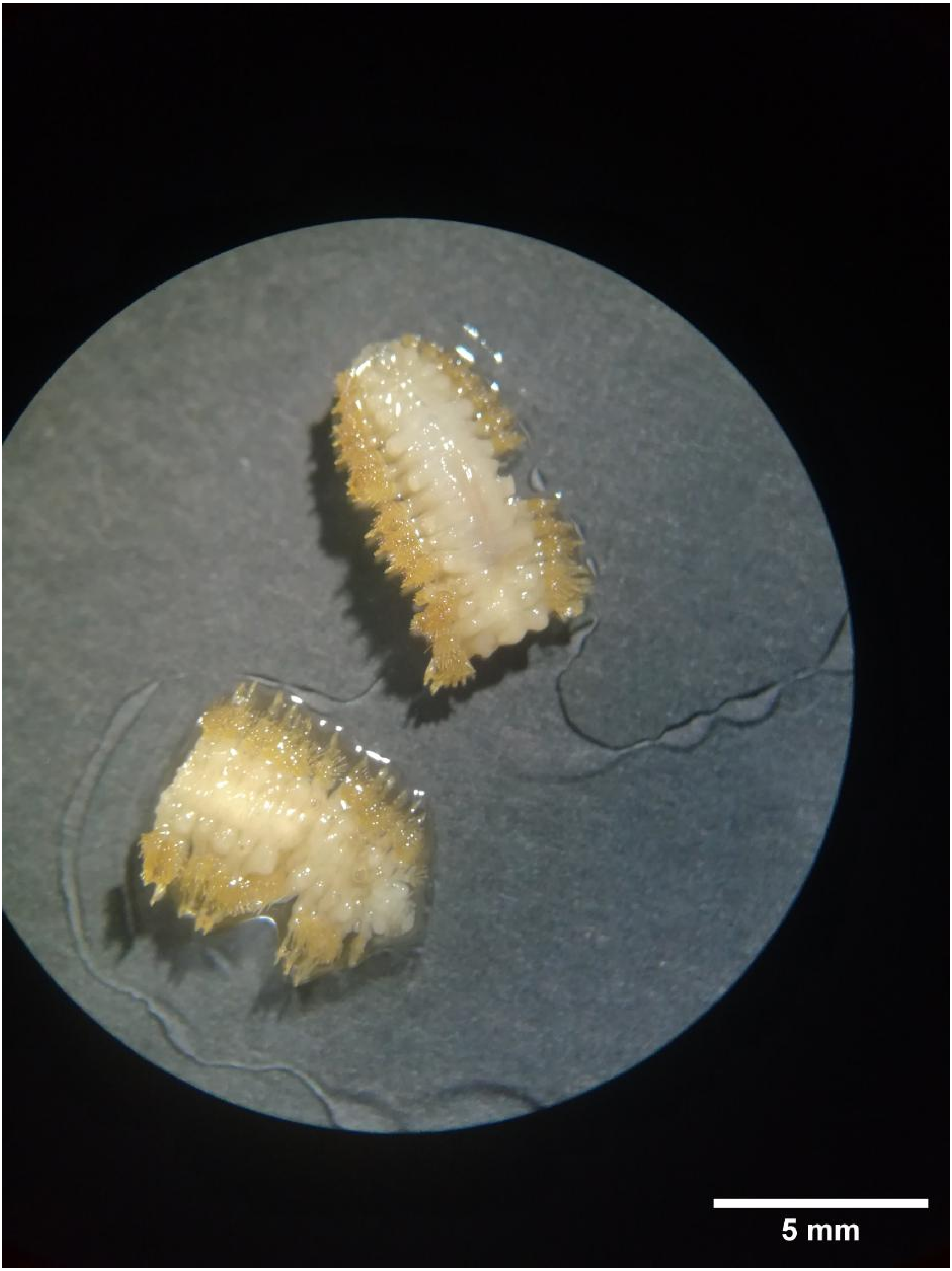
5 mm

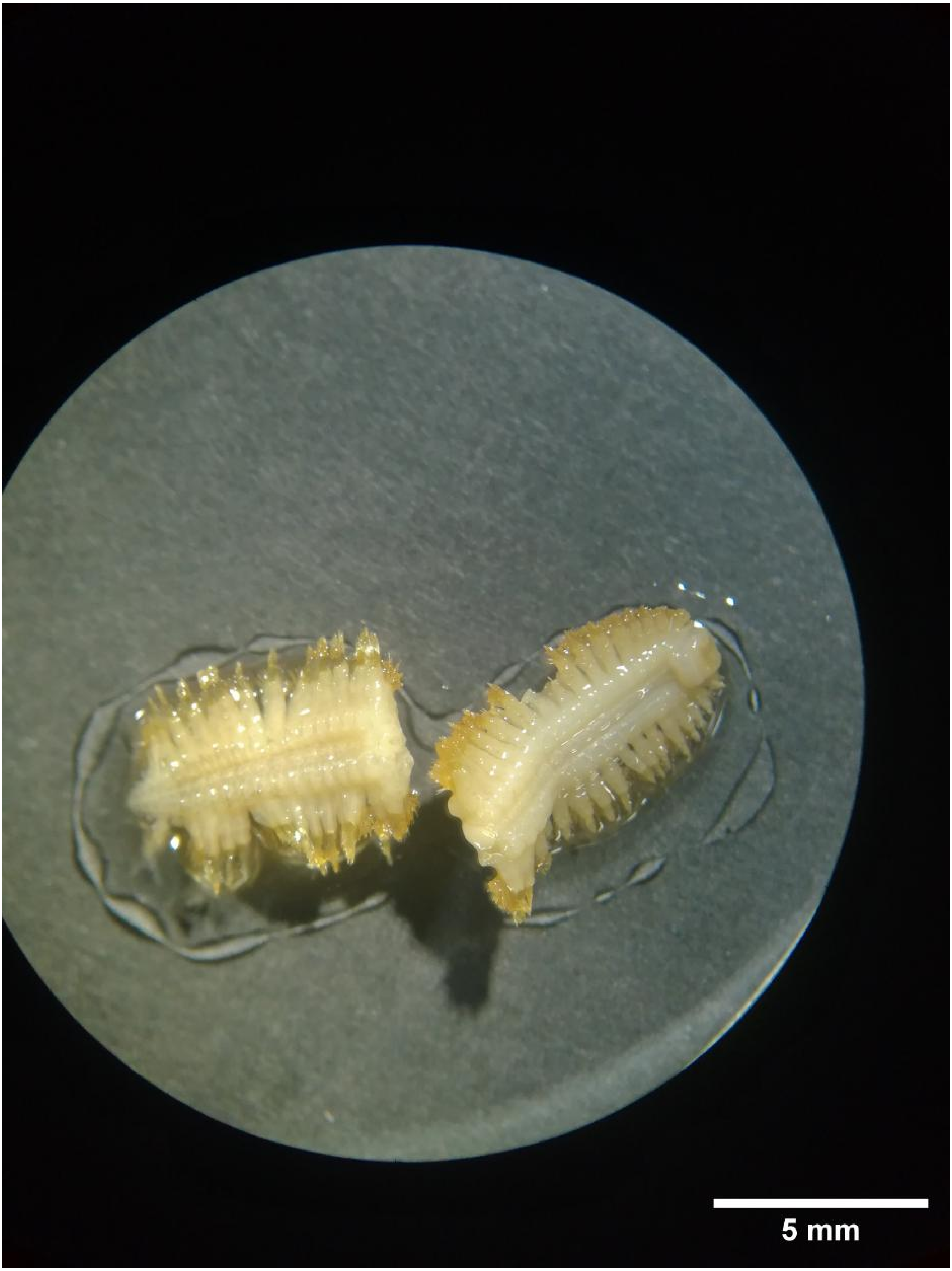
5 mm

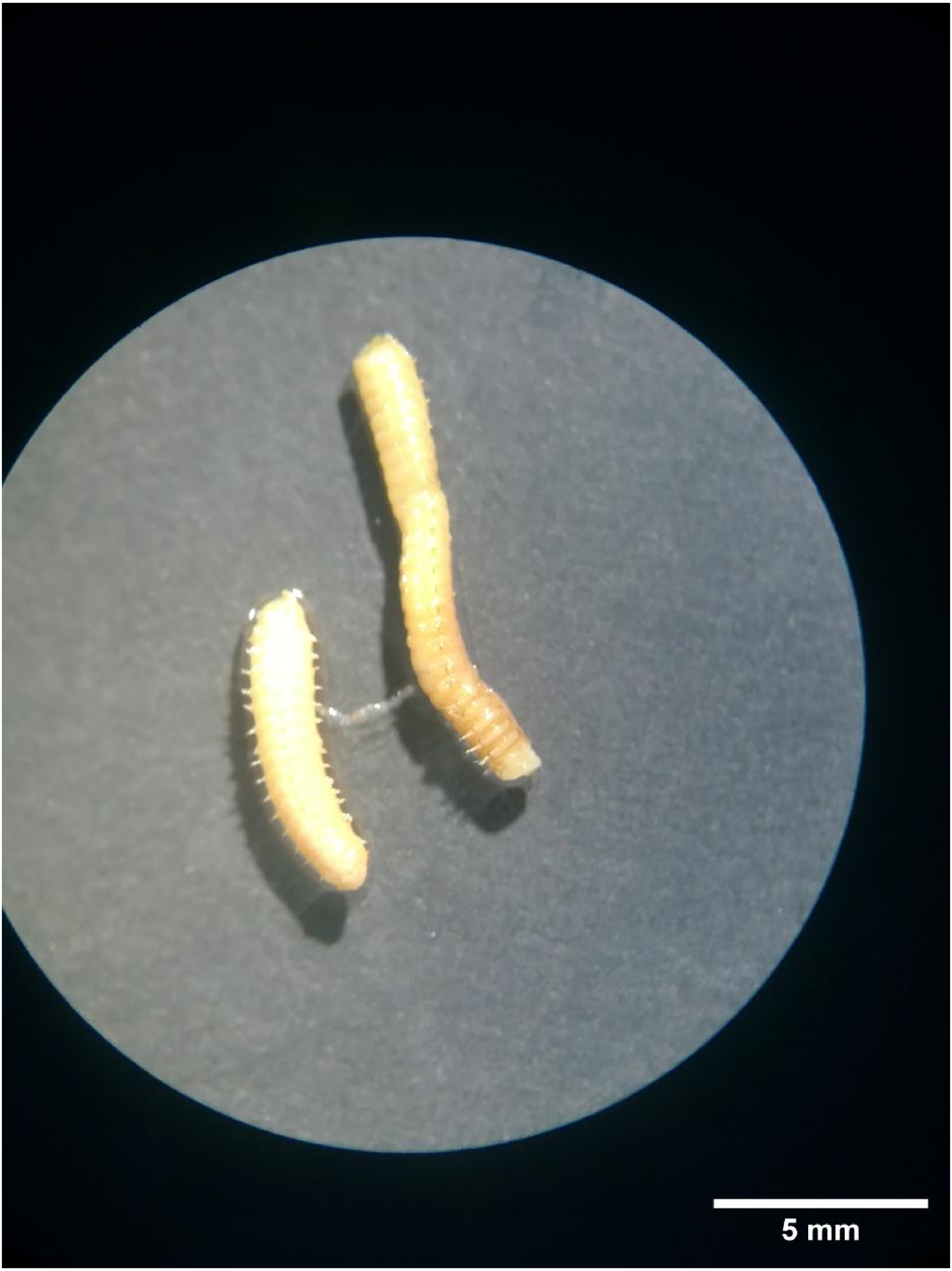
5 mm

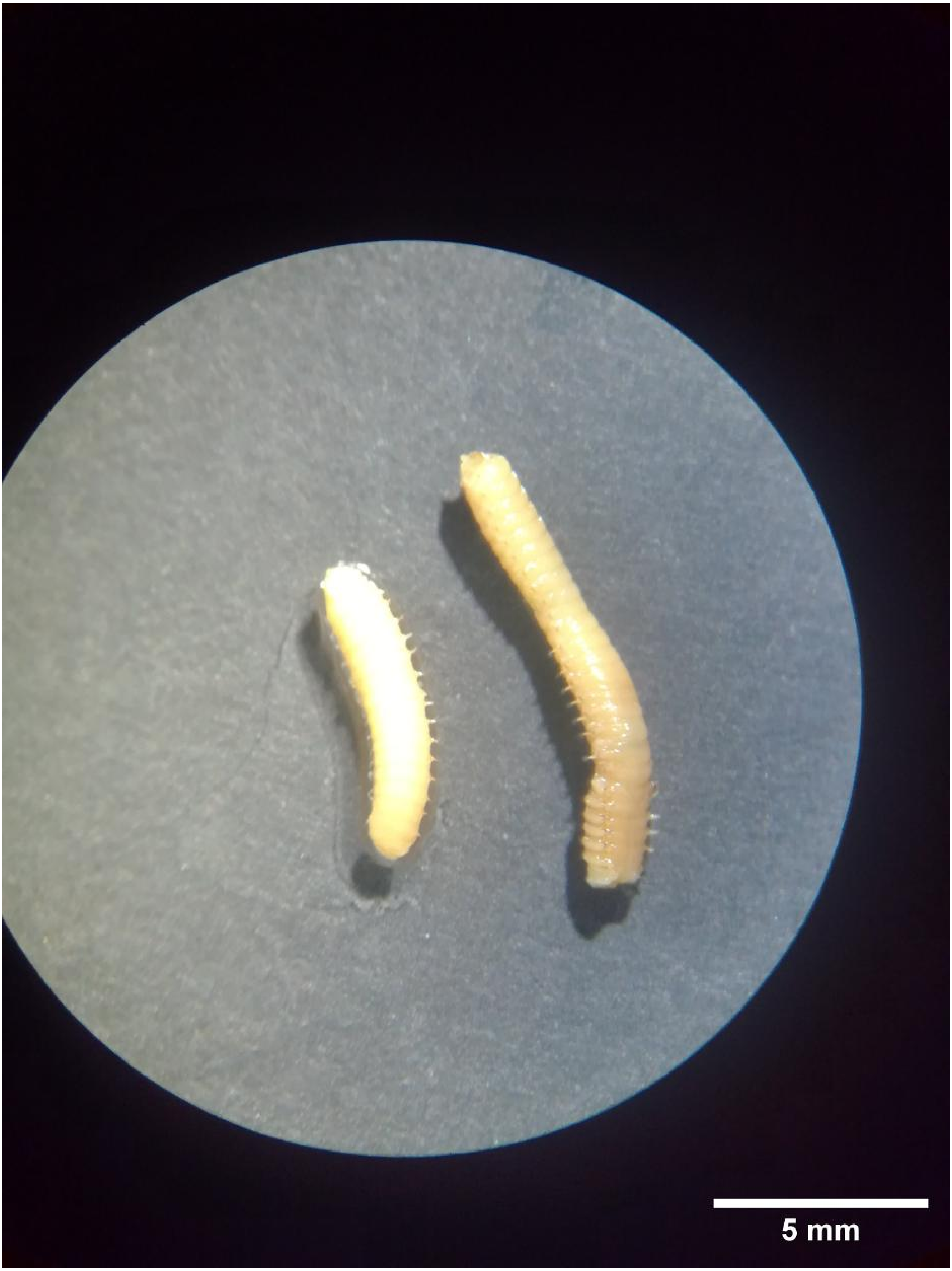
5 mm

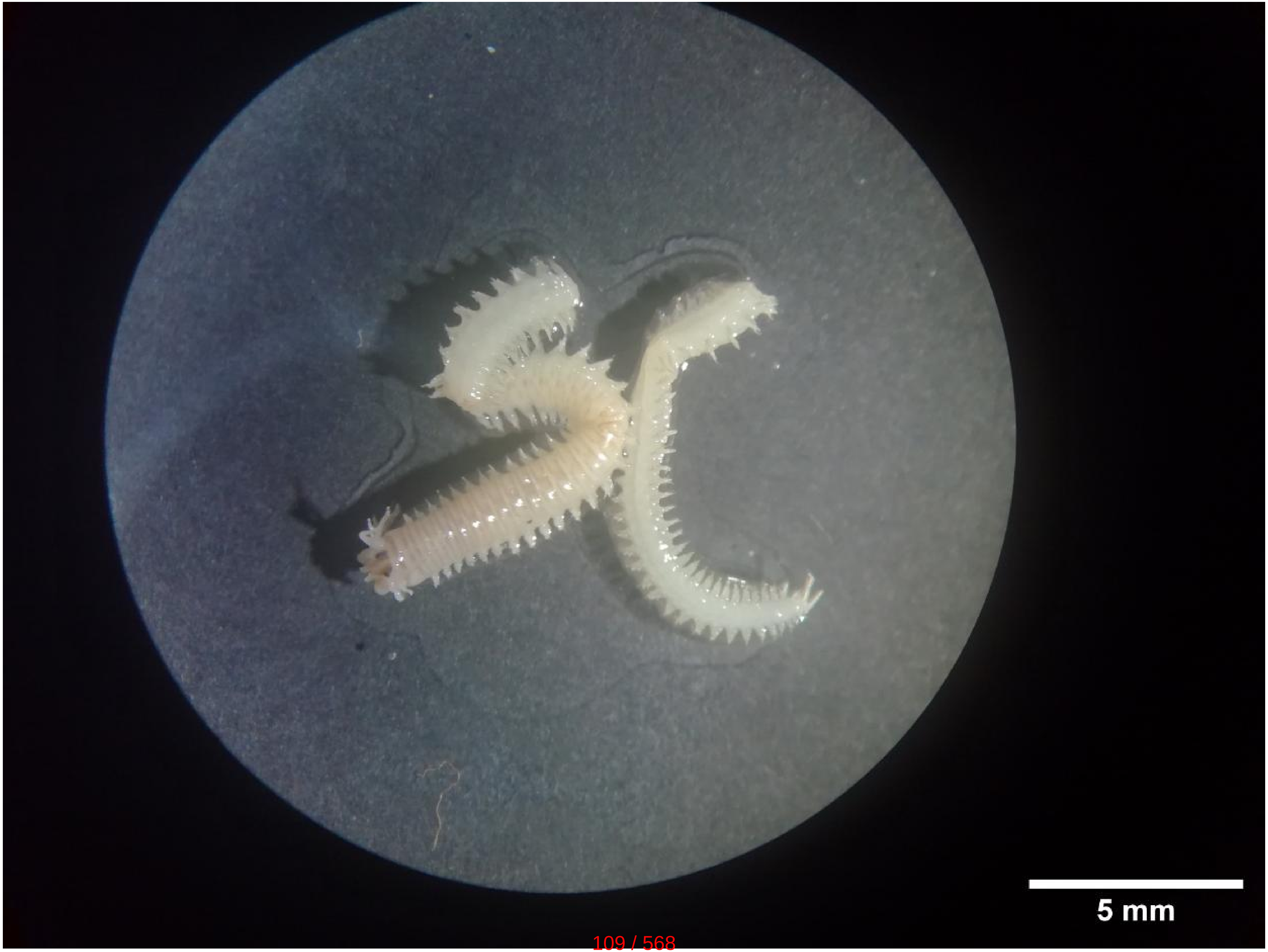
5 mm

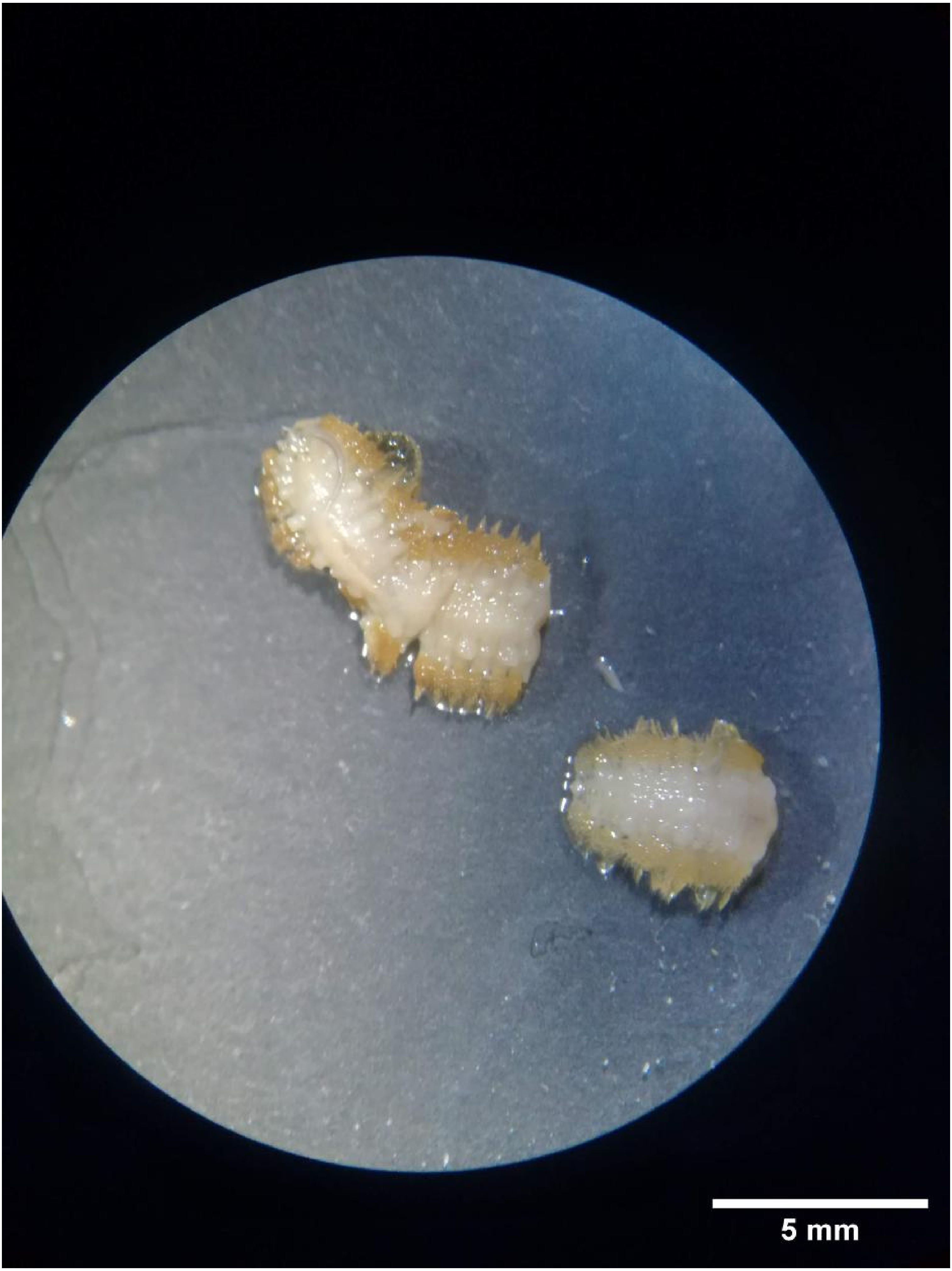
5 mm

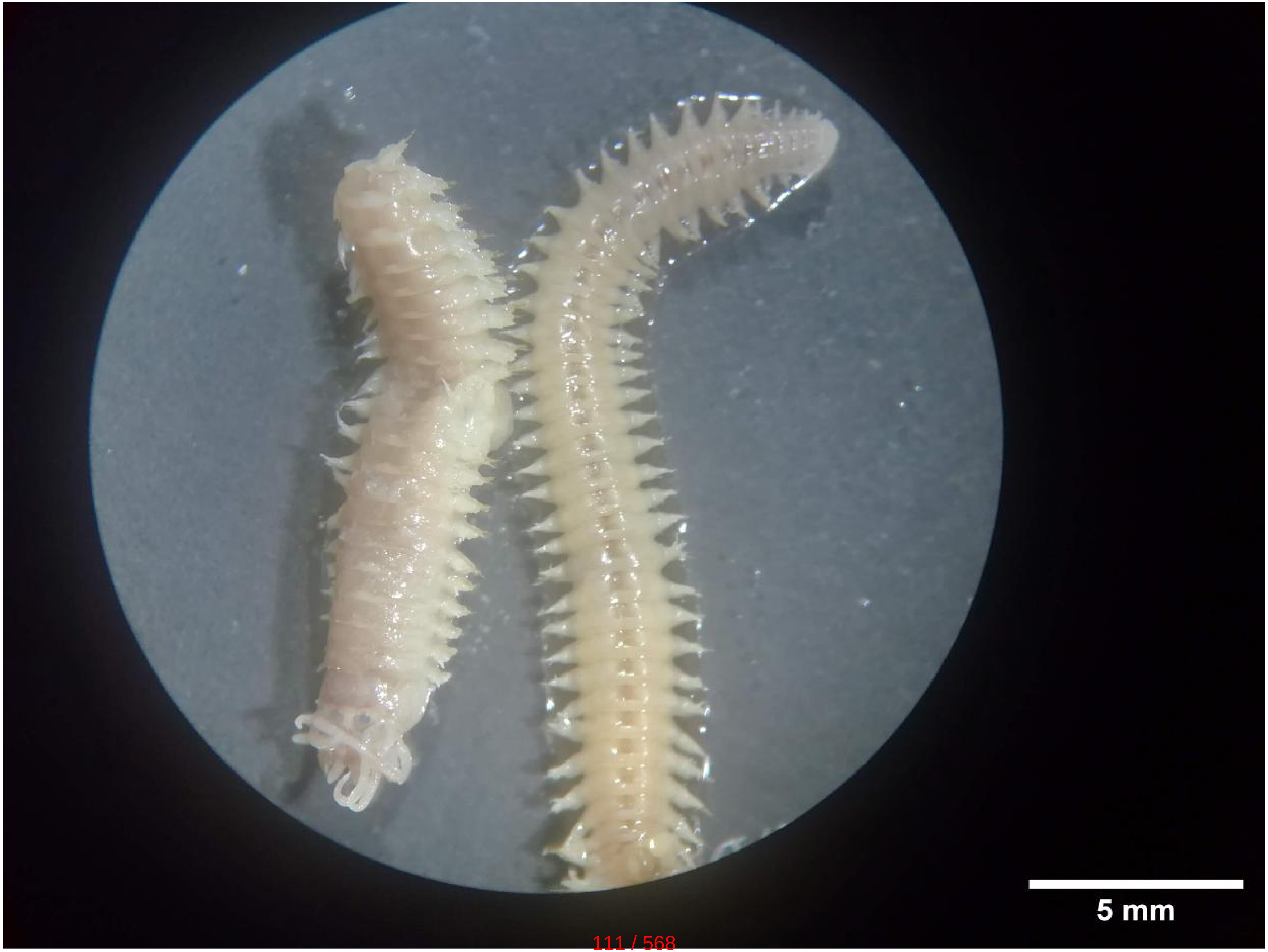
5 mm

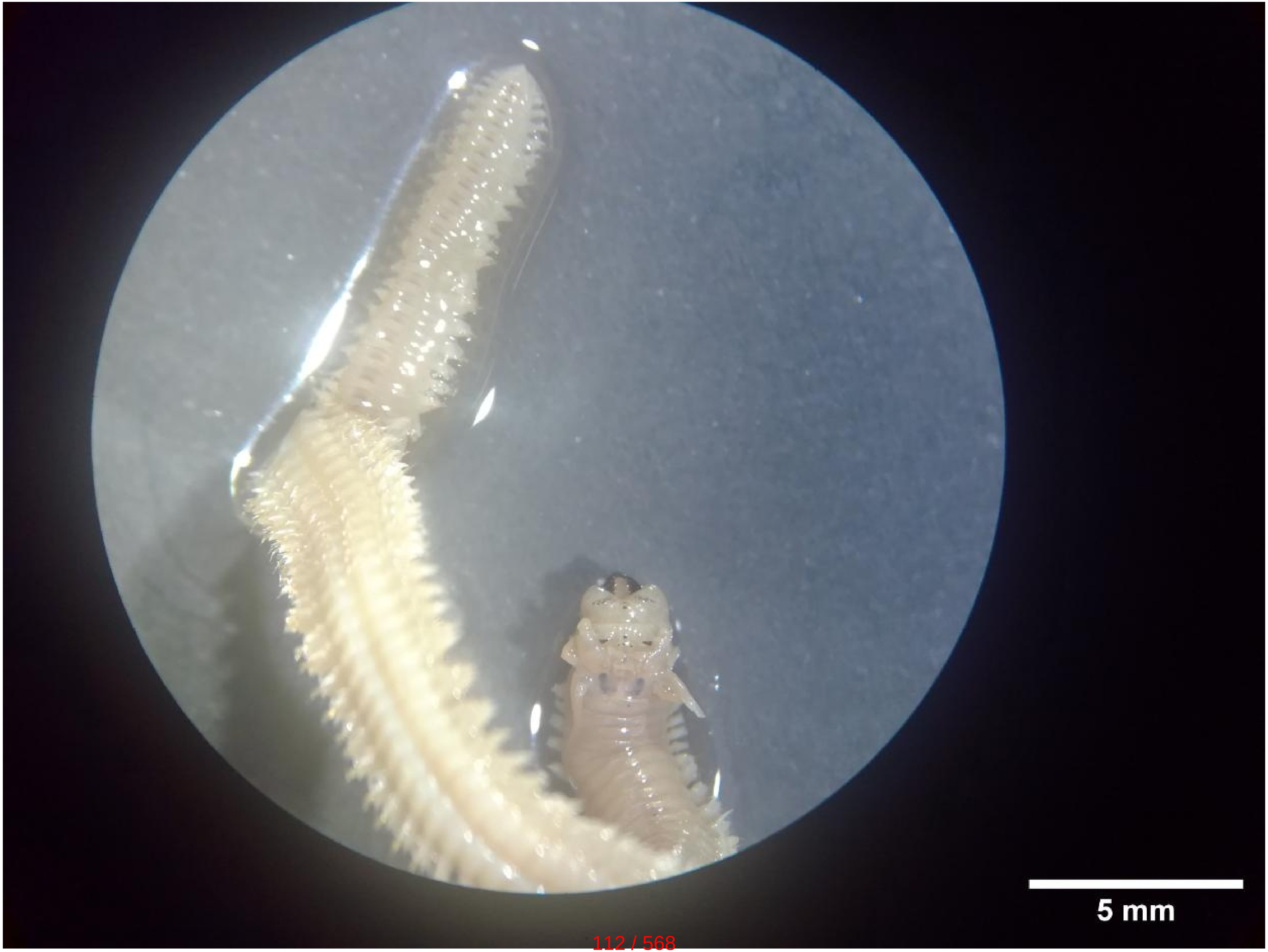
5 mm

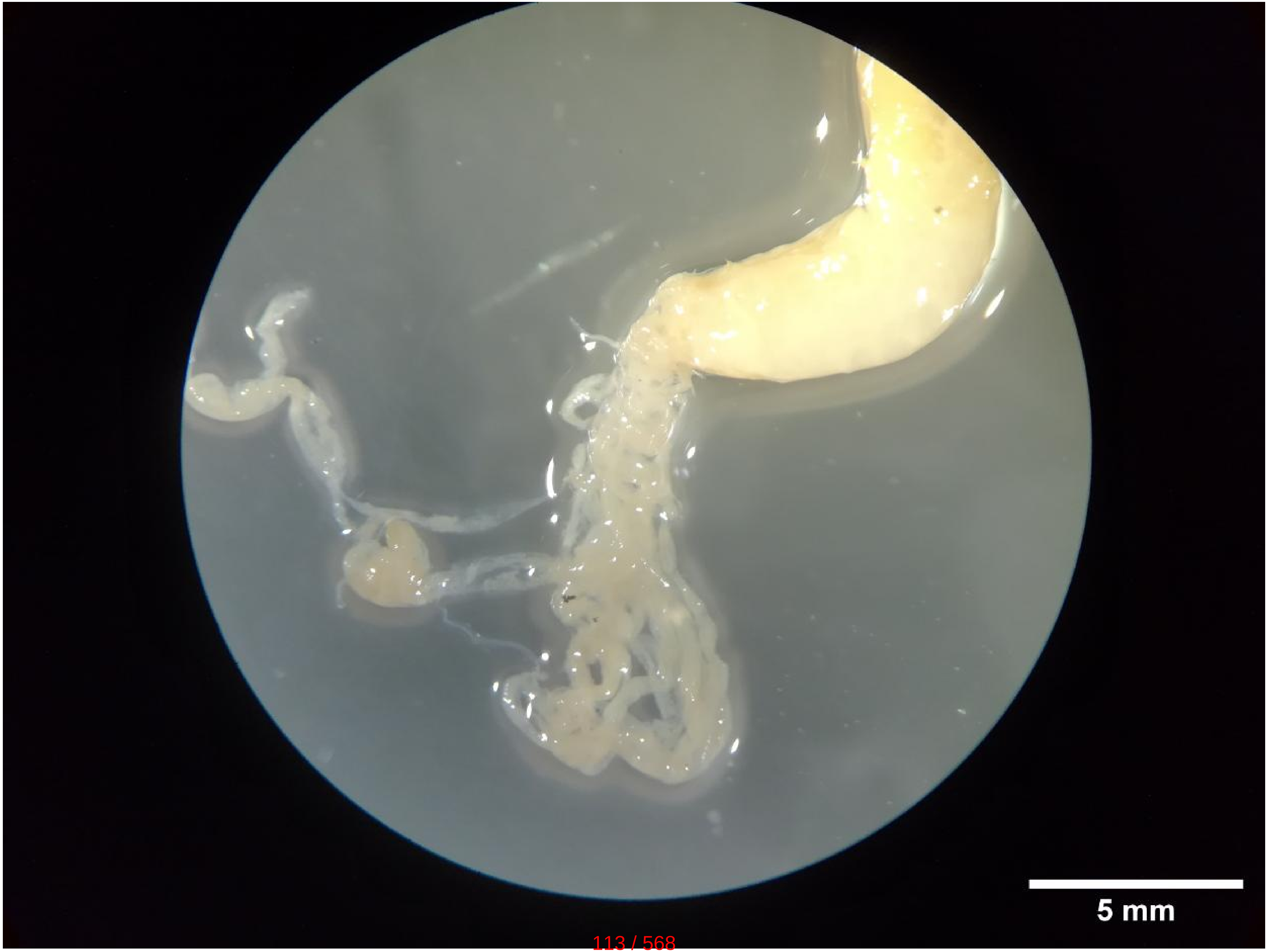
5 mm

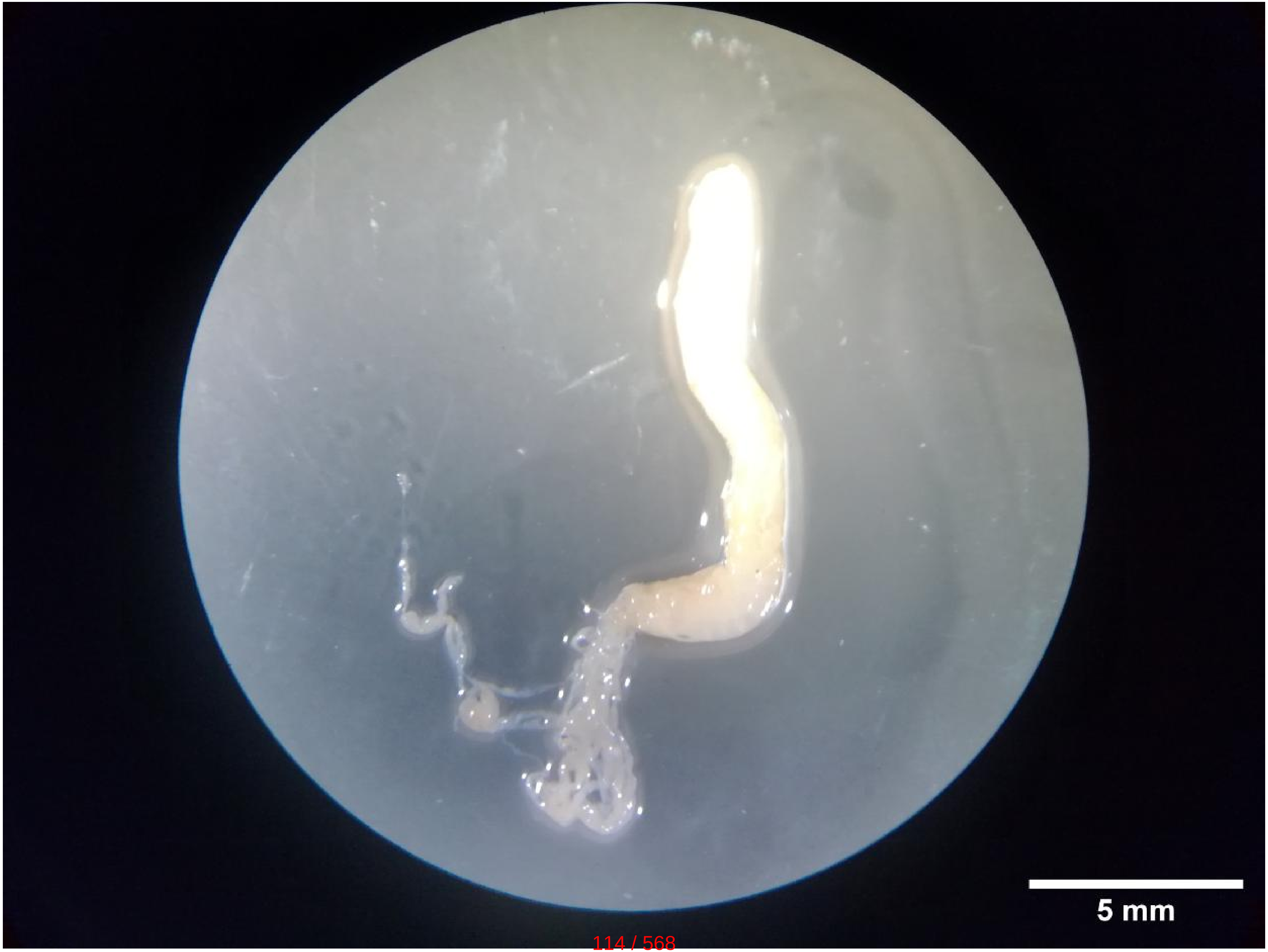
5 mm

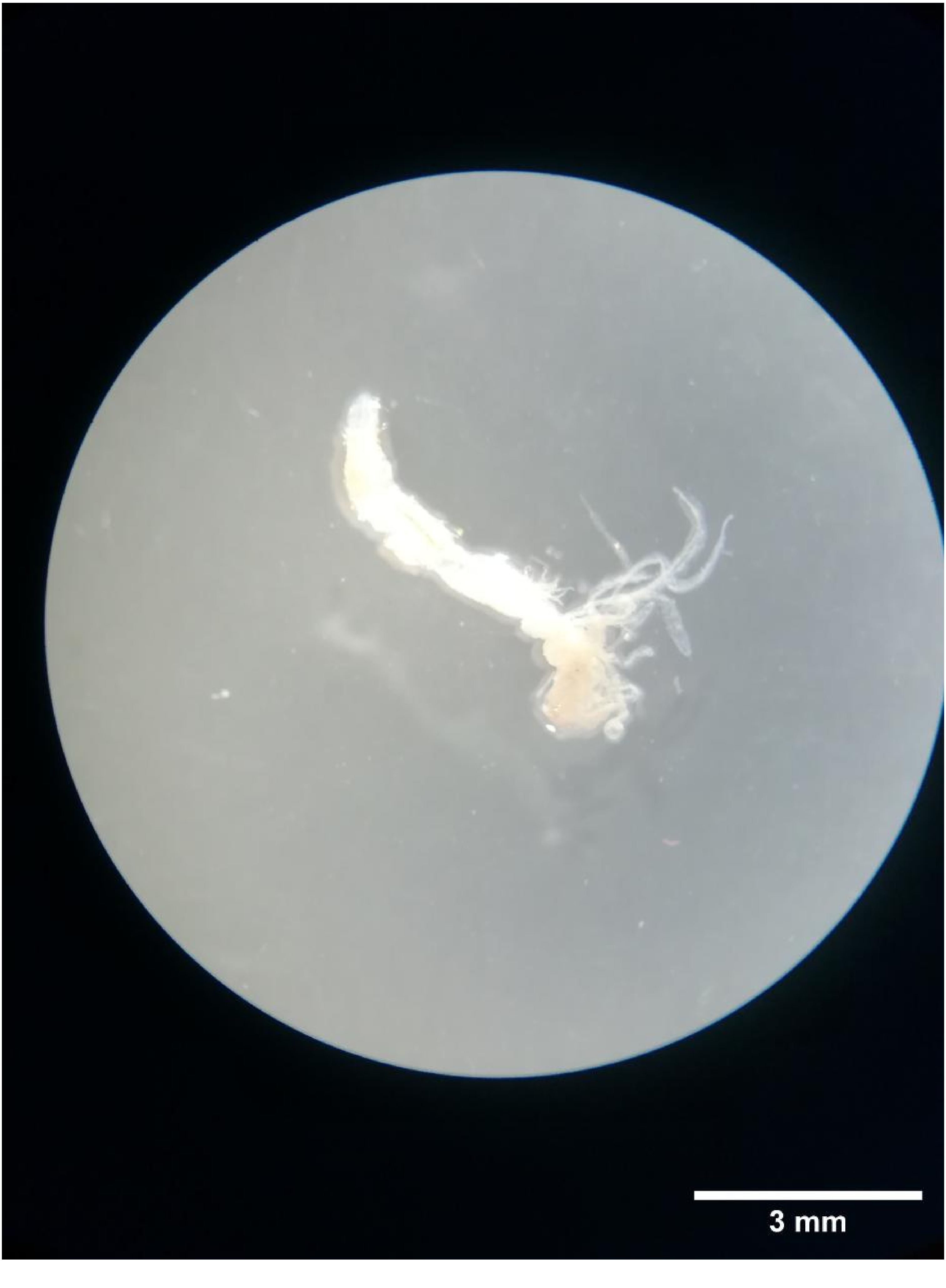
3 mm

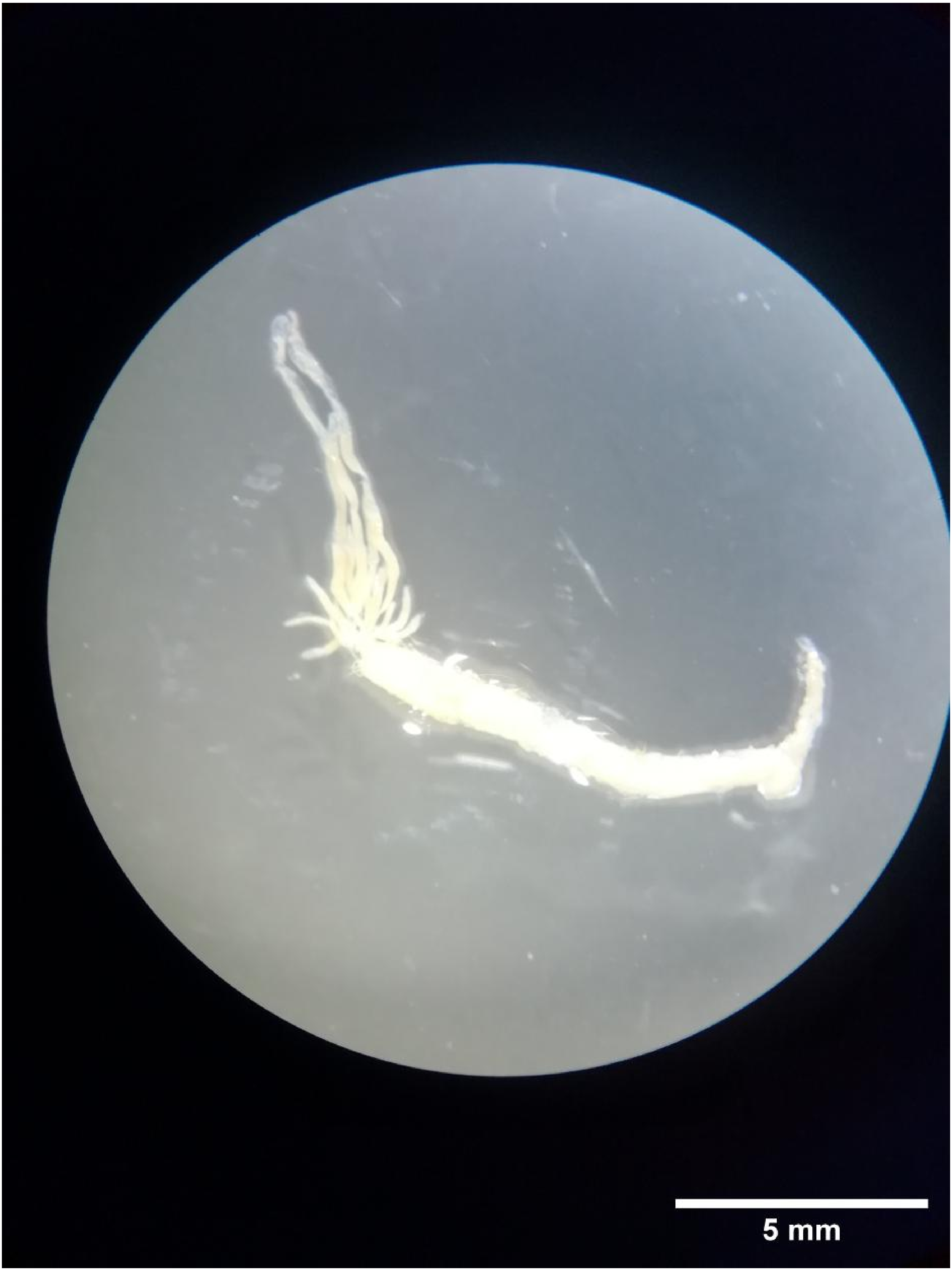
5 mm

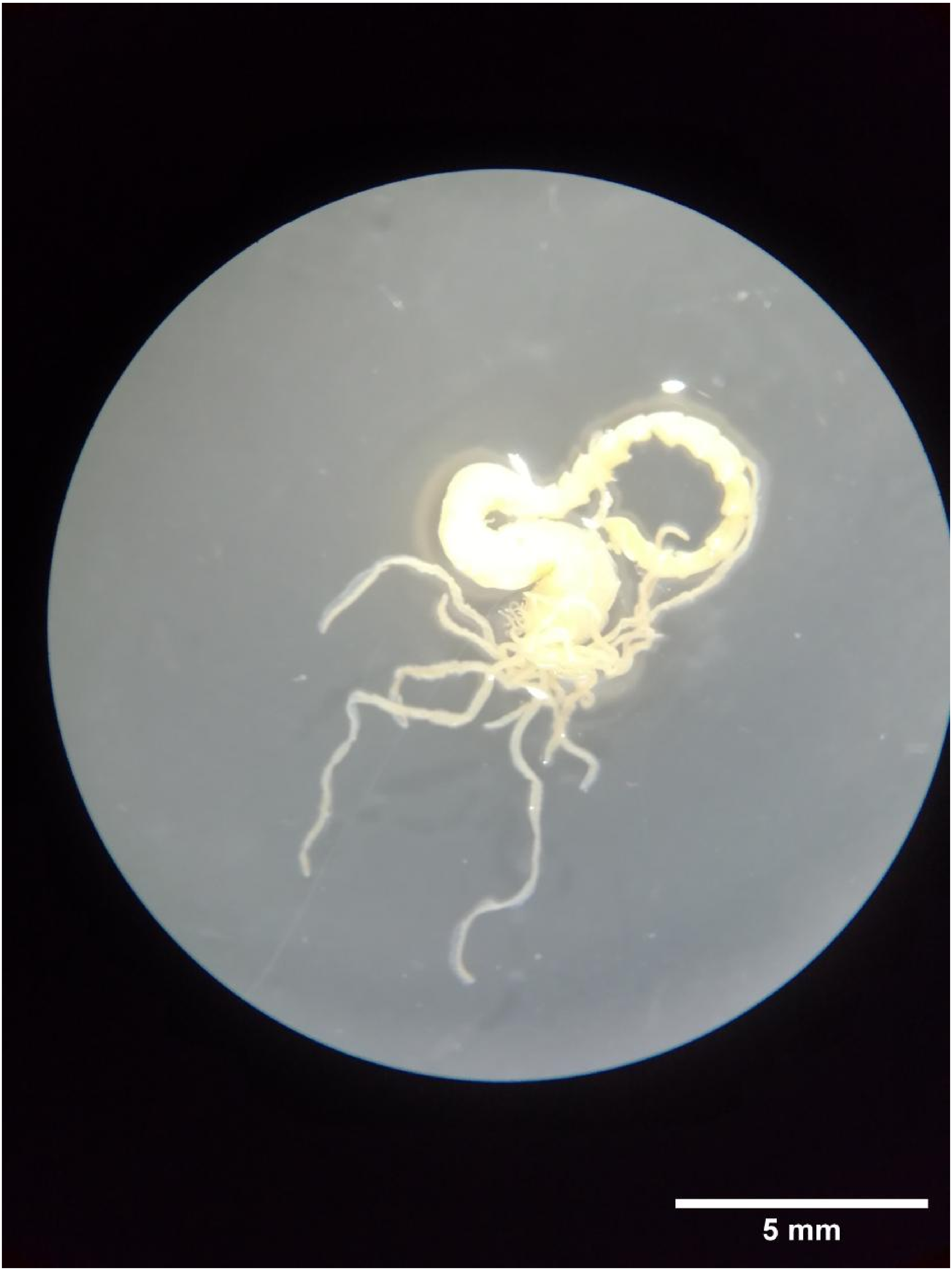
5 mm

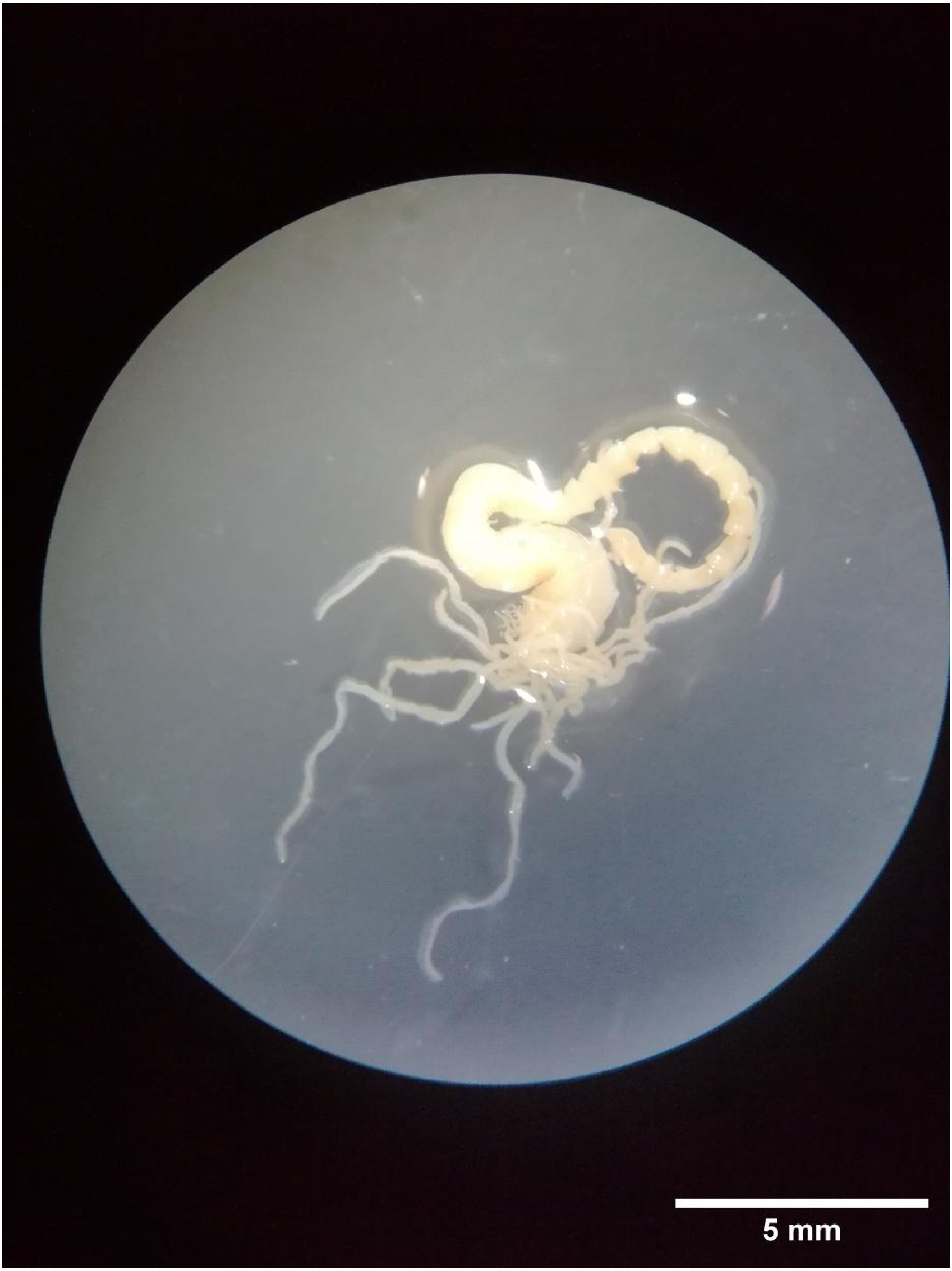
5 mm

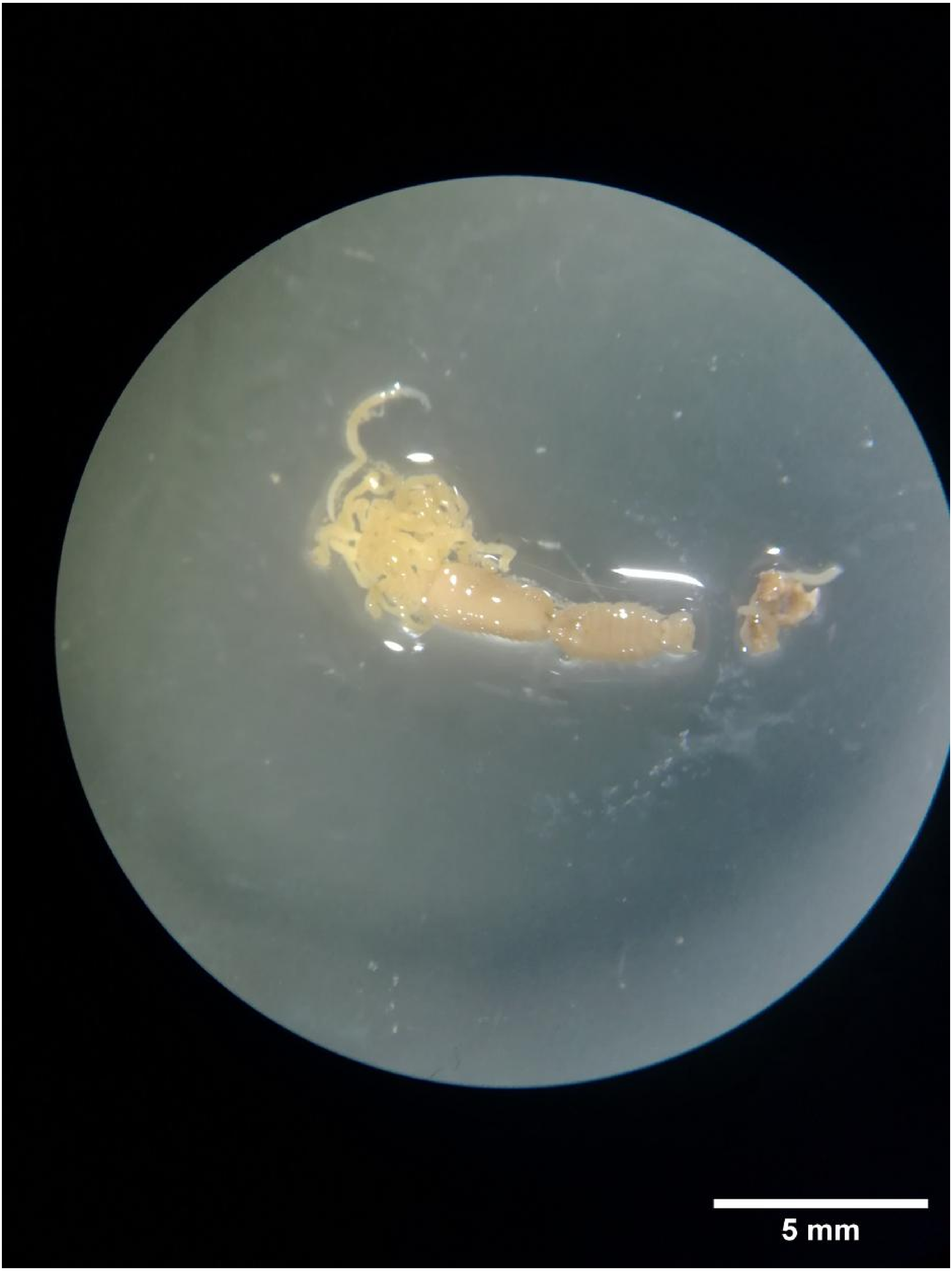
5 mm

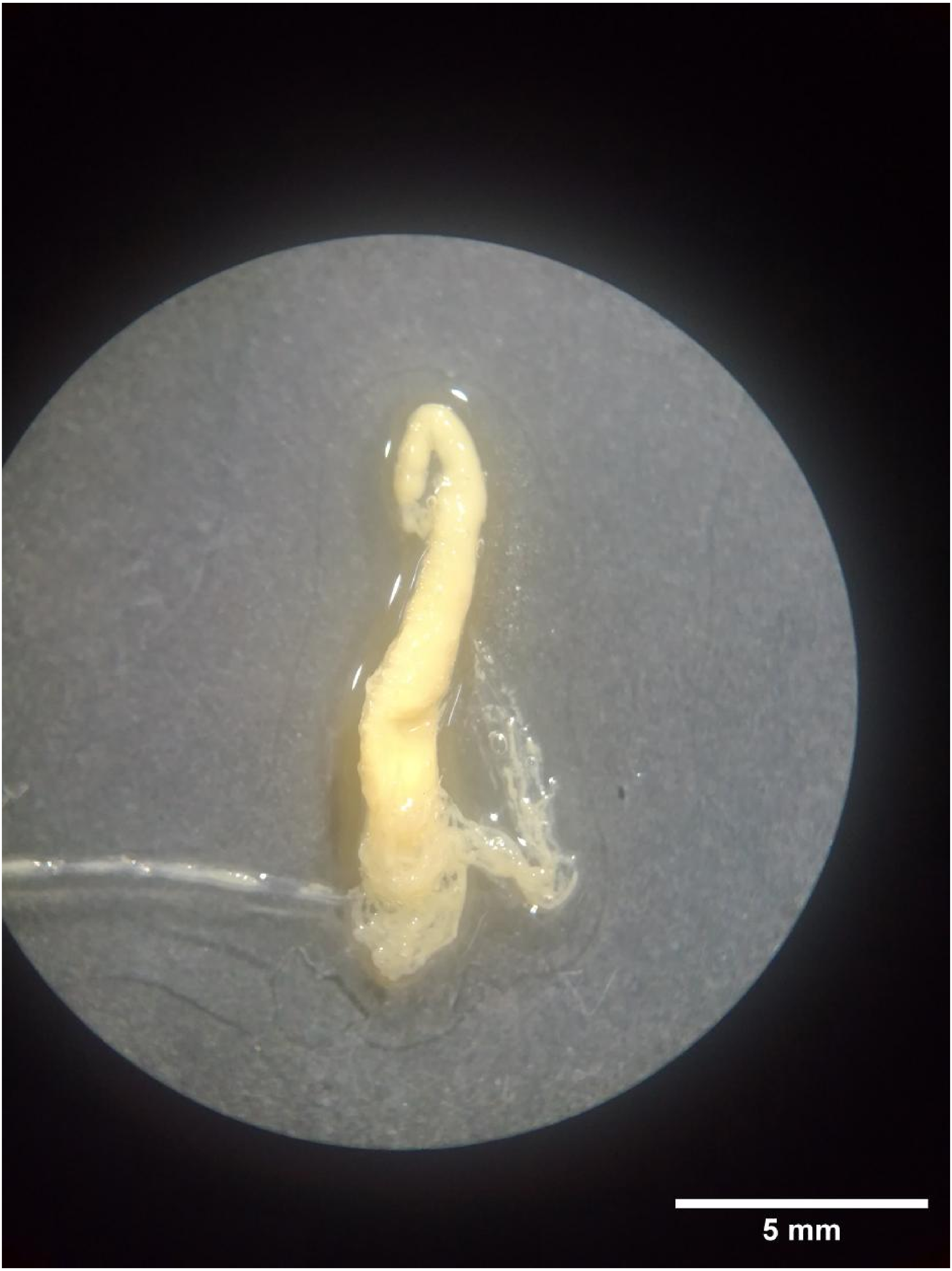
5 mm

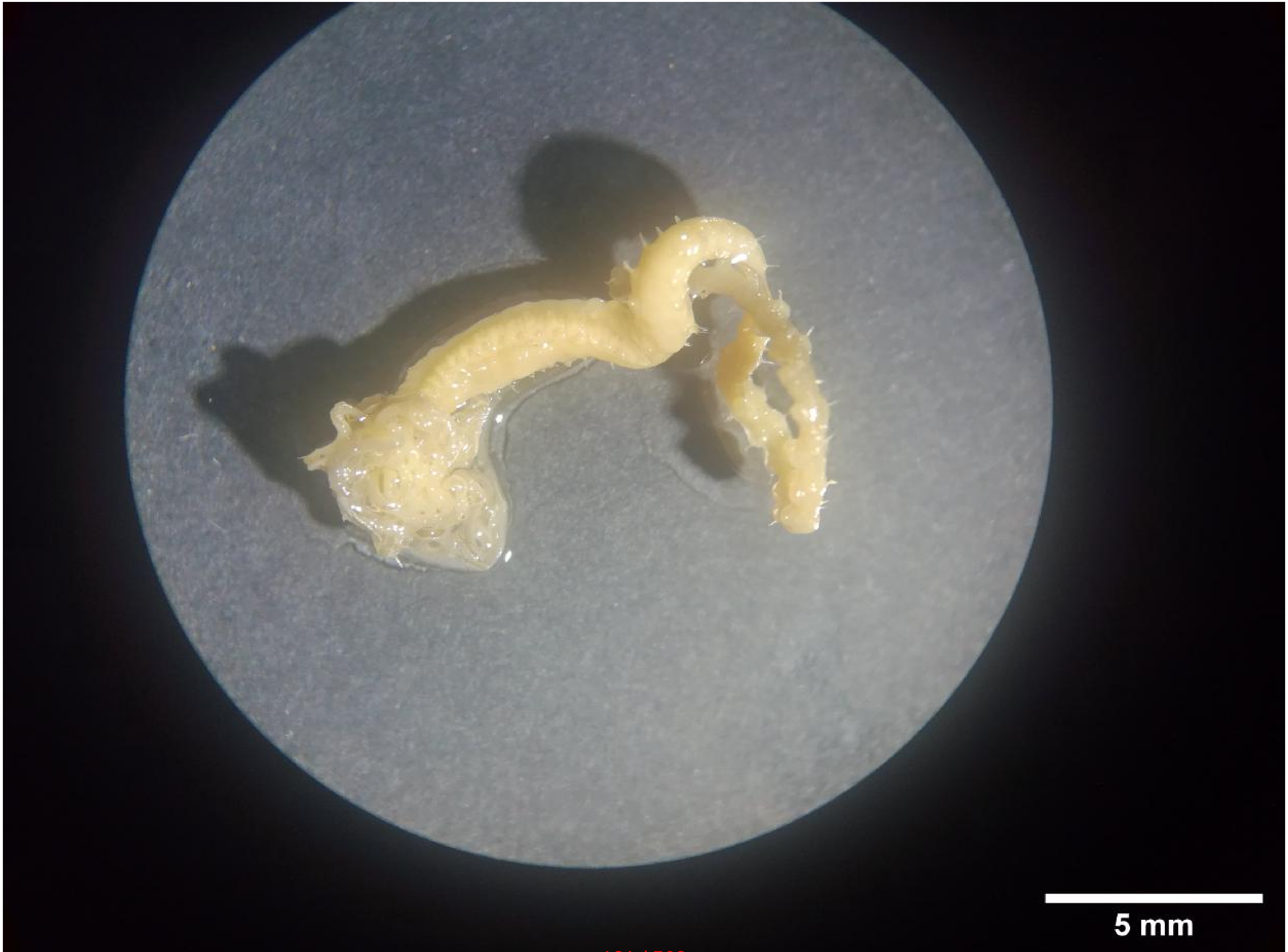
5 mm

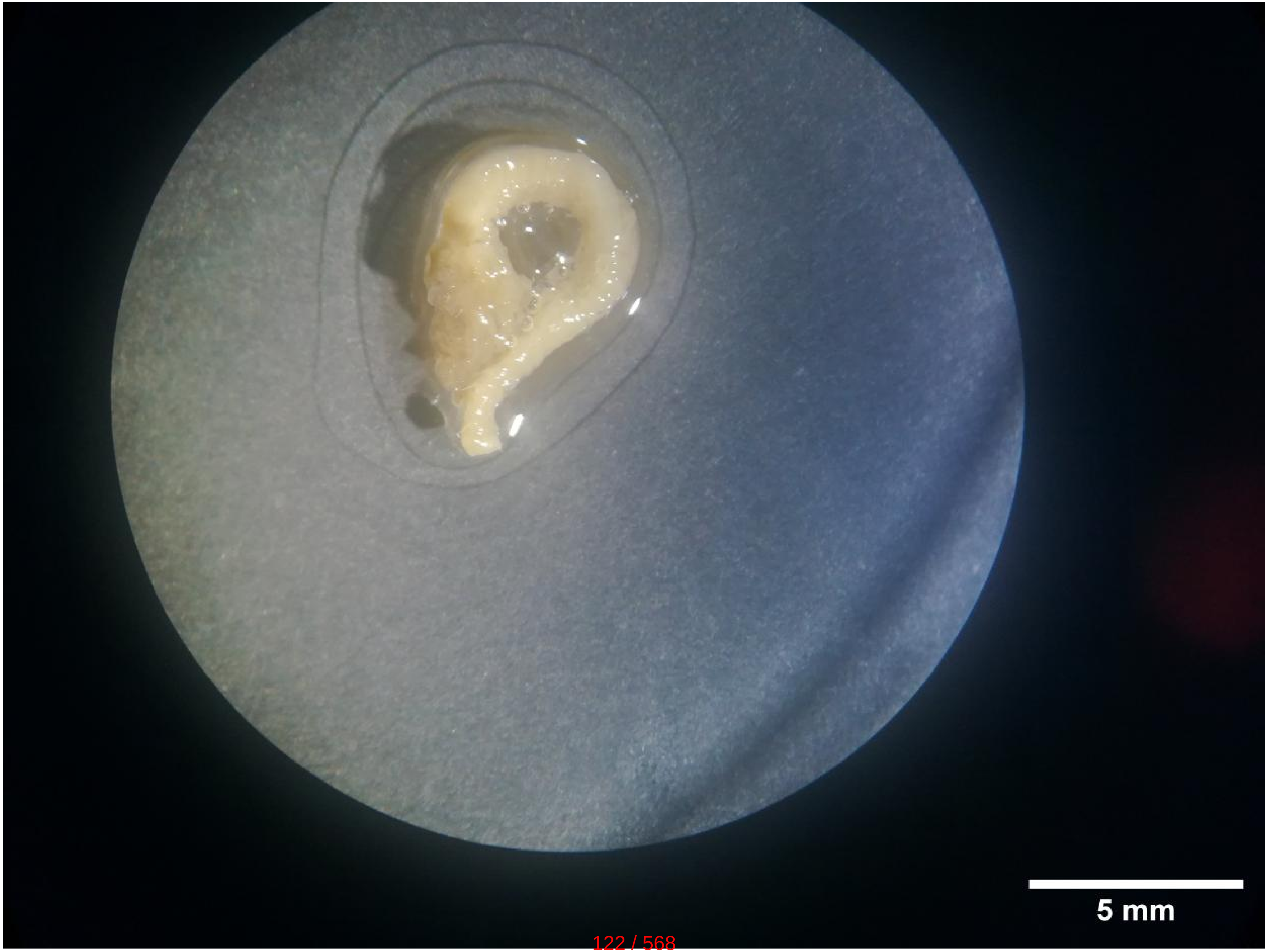
5 mm

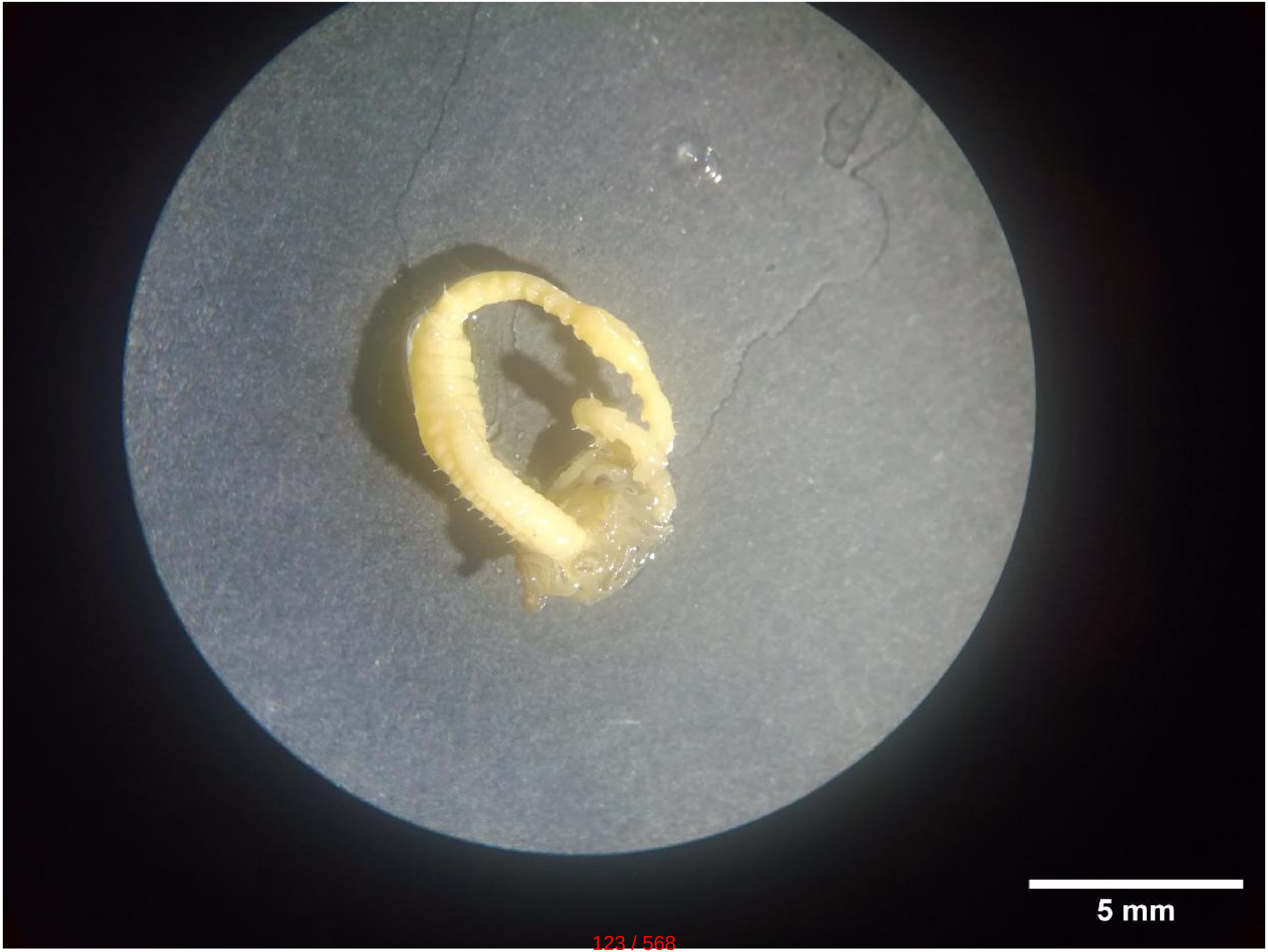
5 mm

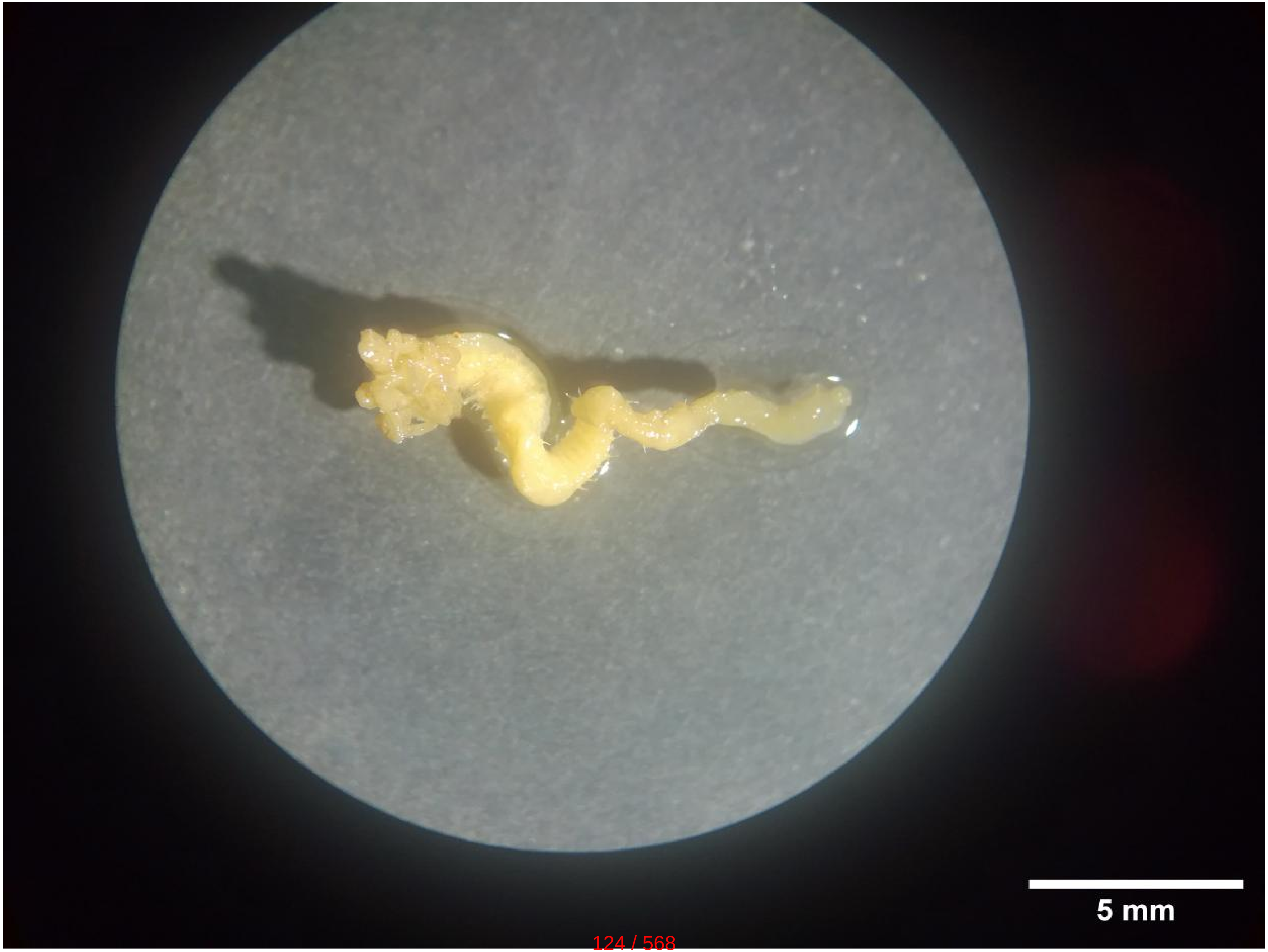
5 mm

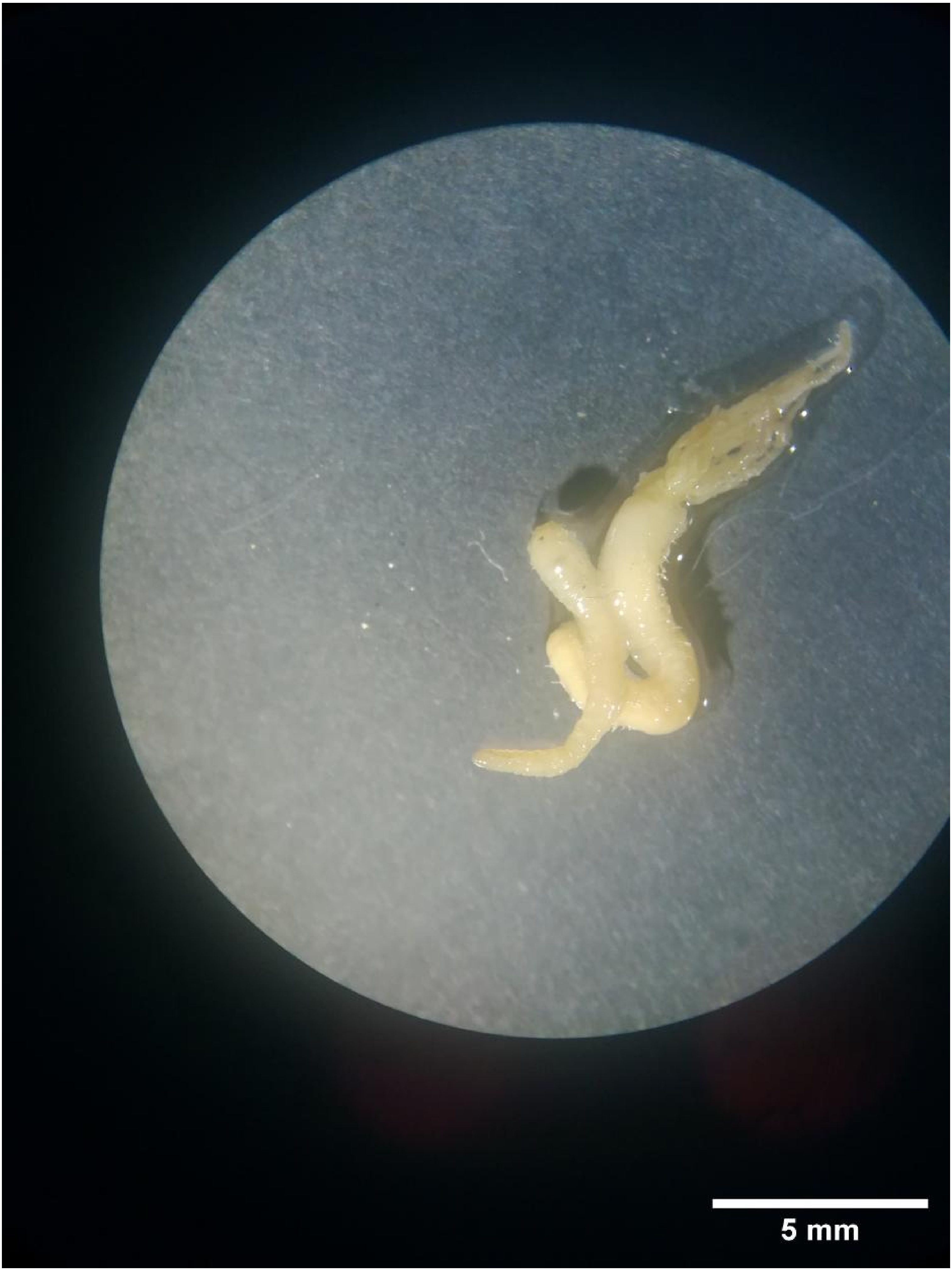
5 mm

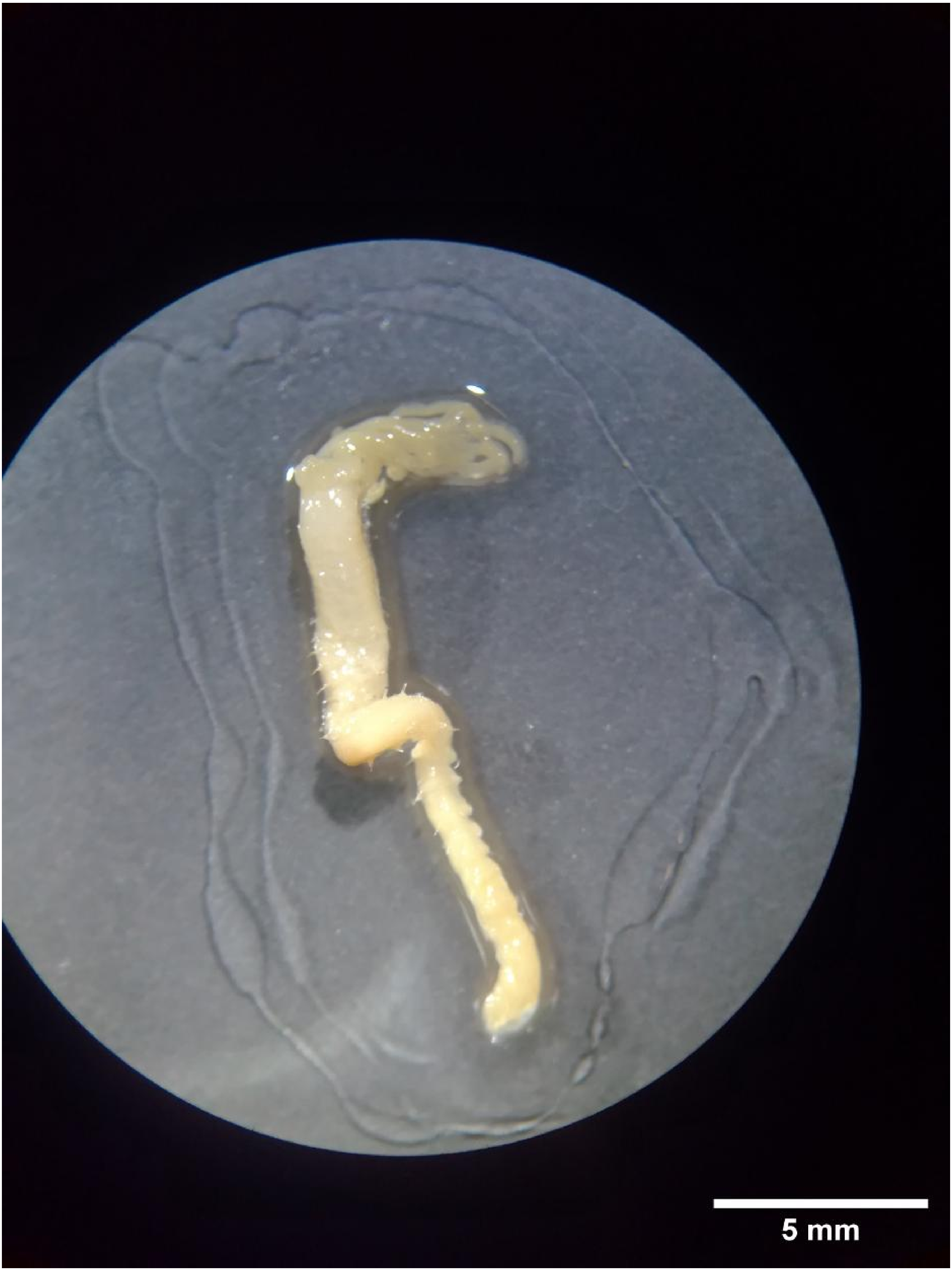
5 mm

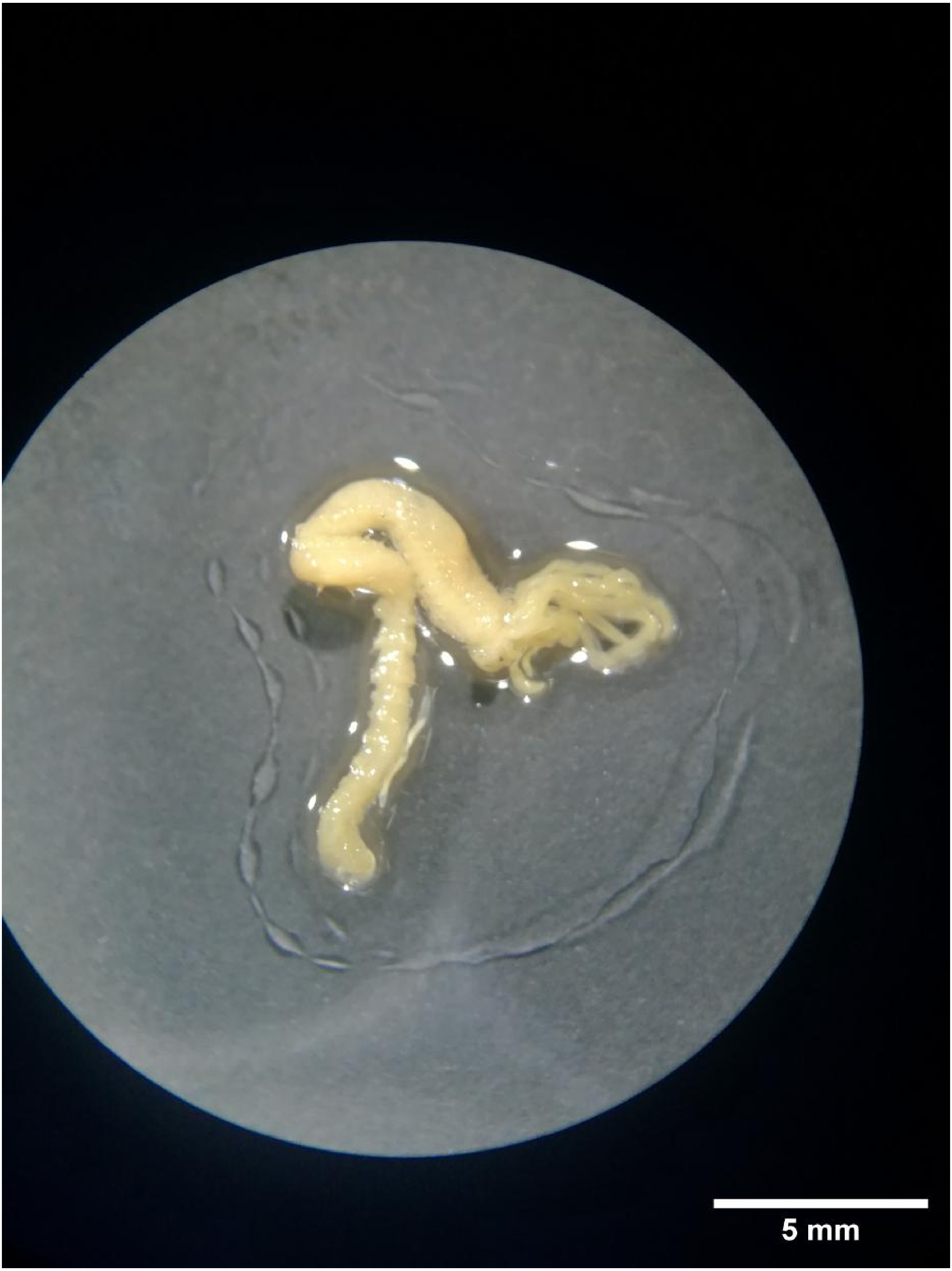
5 mm

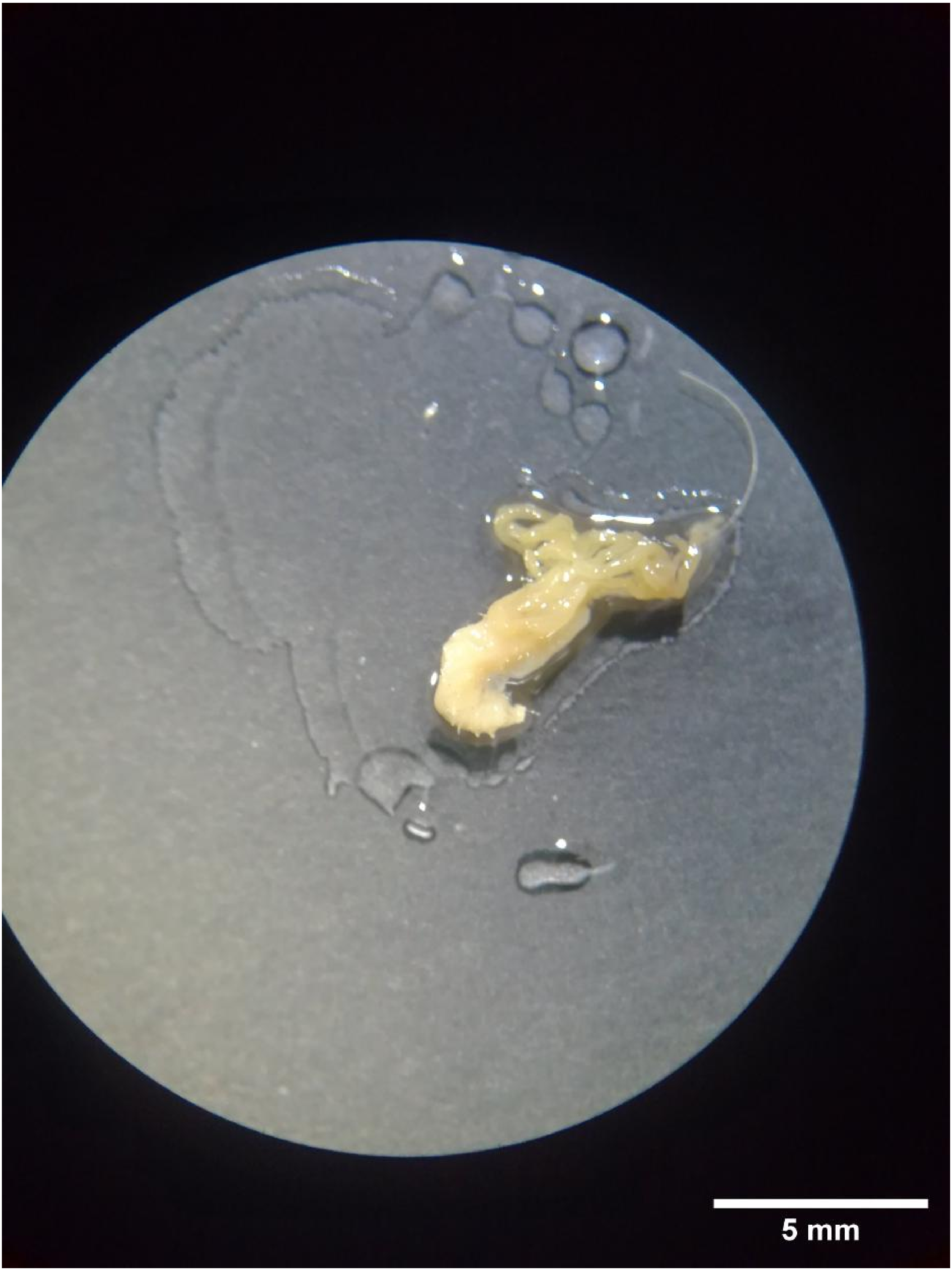
5 mm

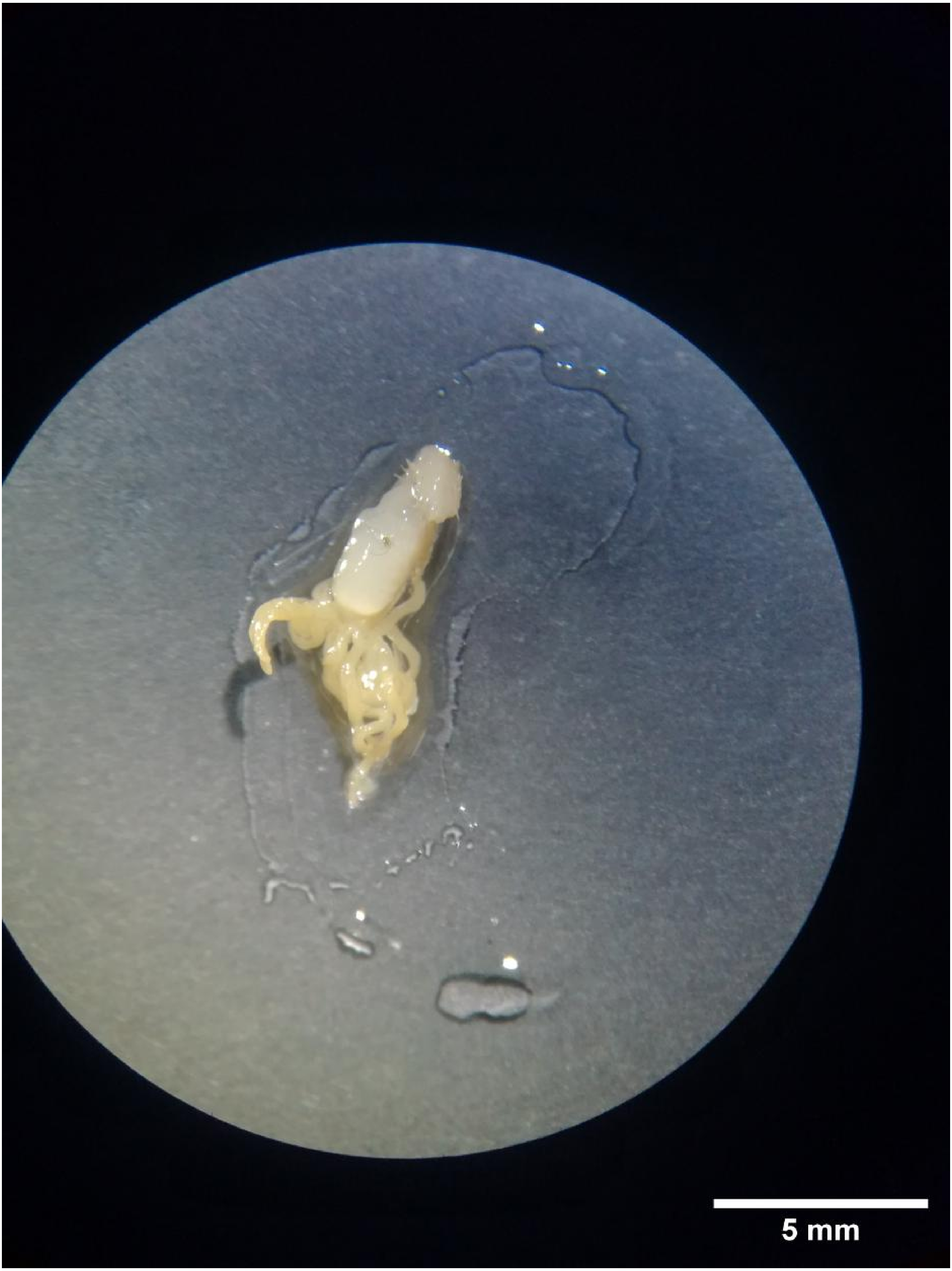
5 mm

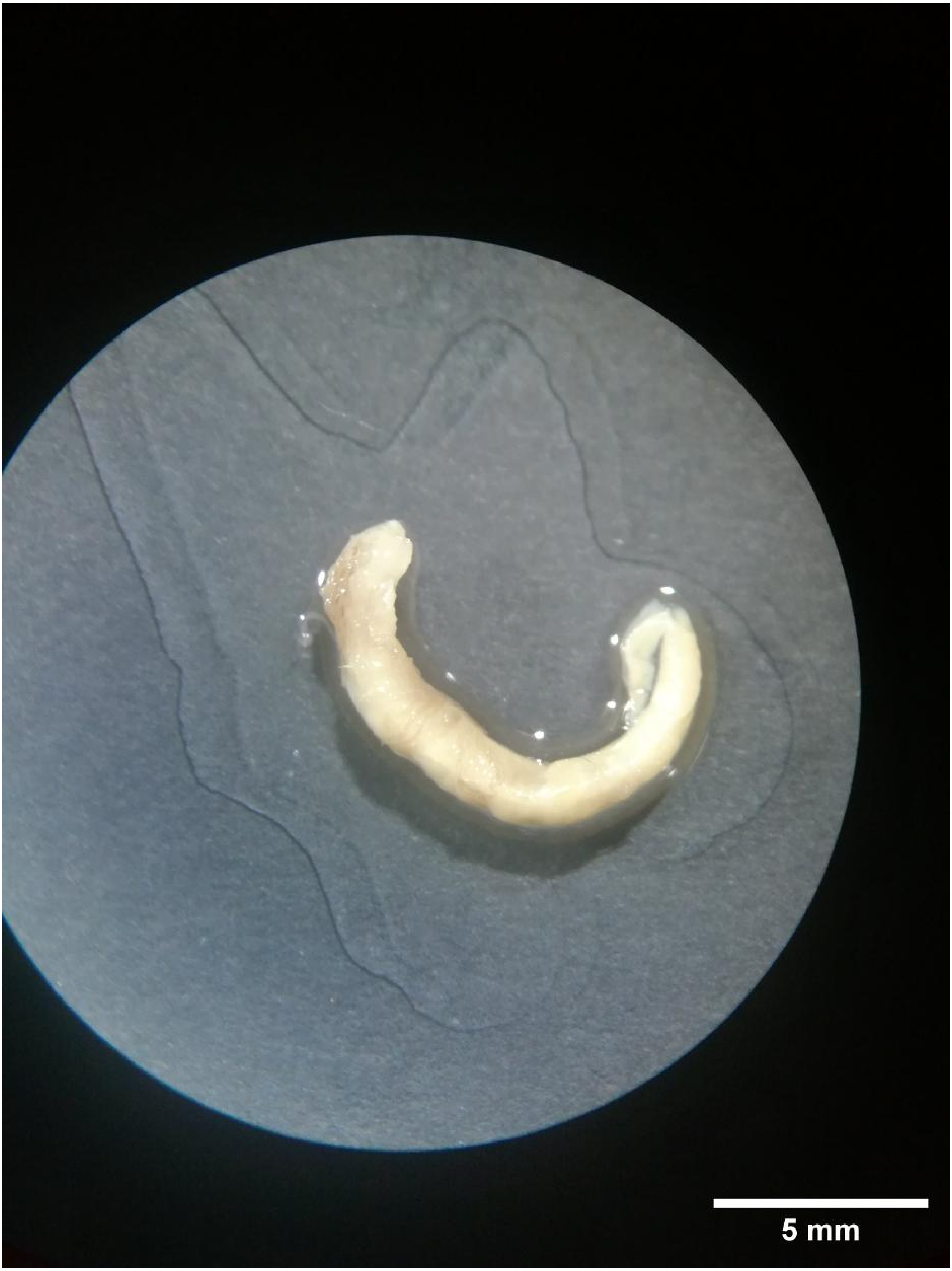
5 mm

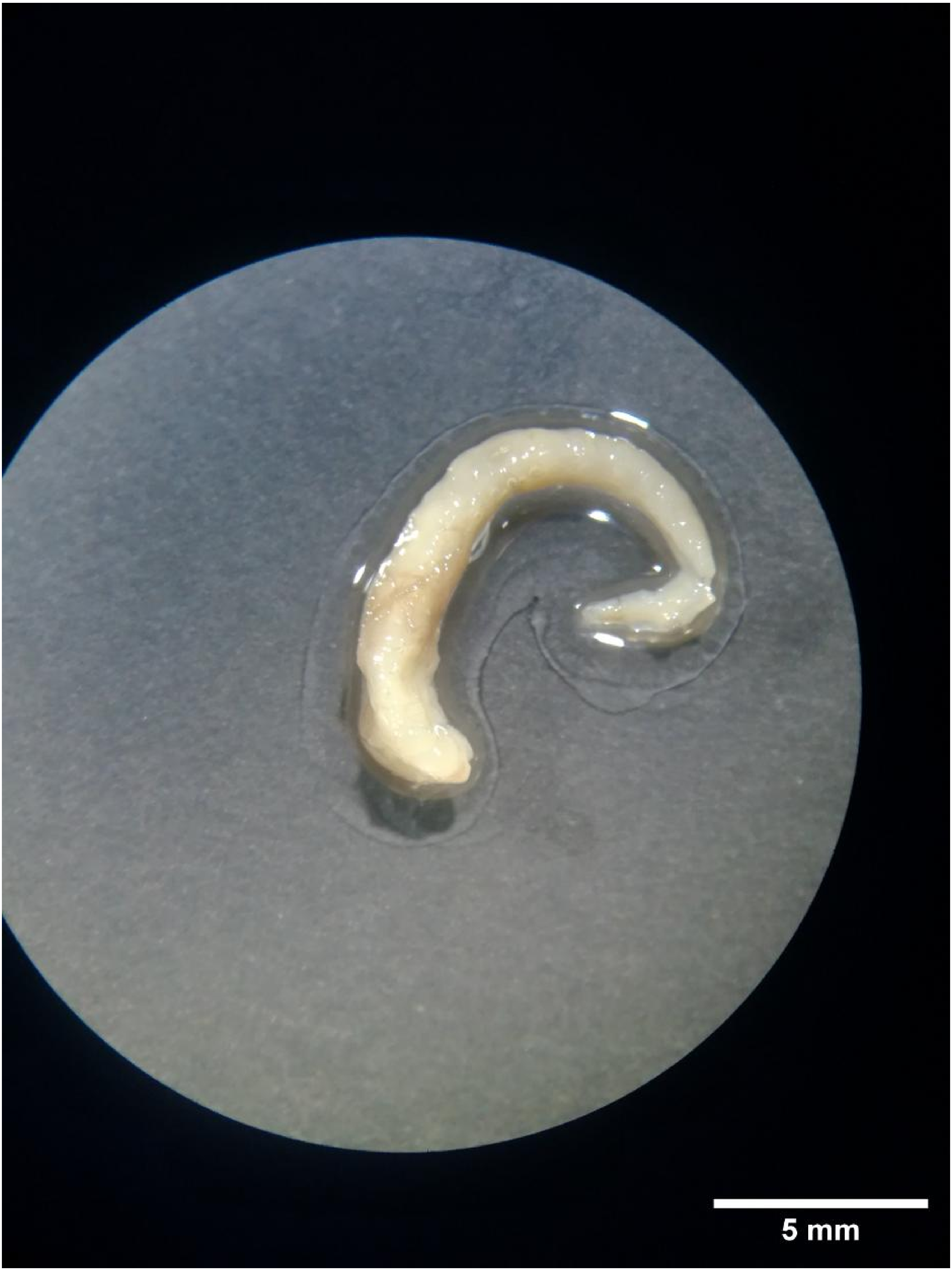
5 mm

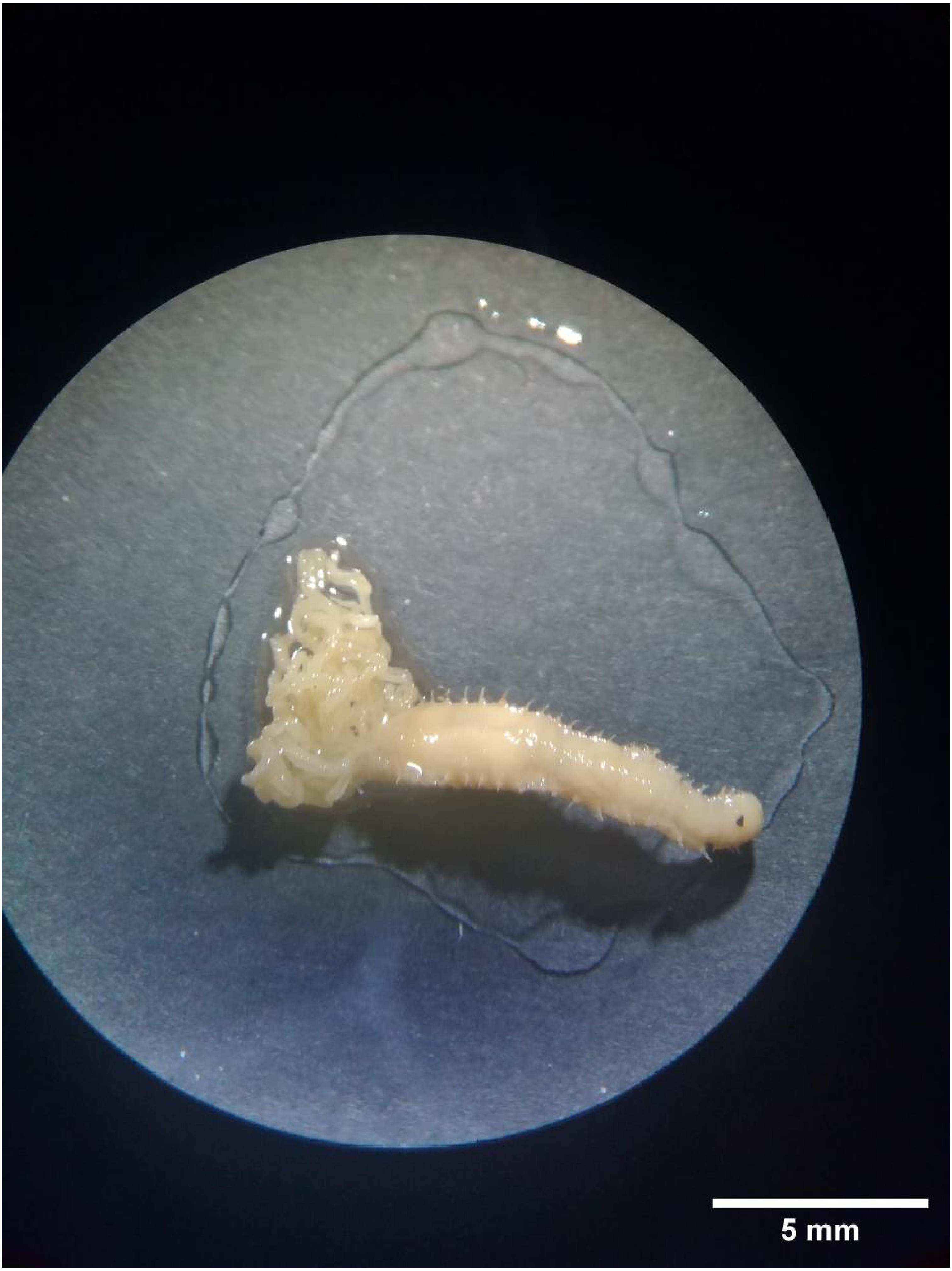
5 mm

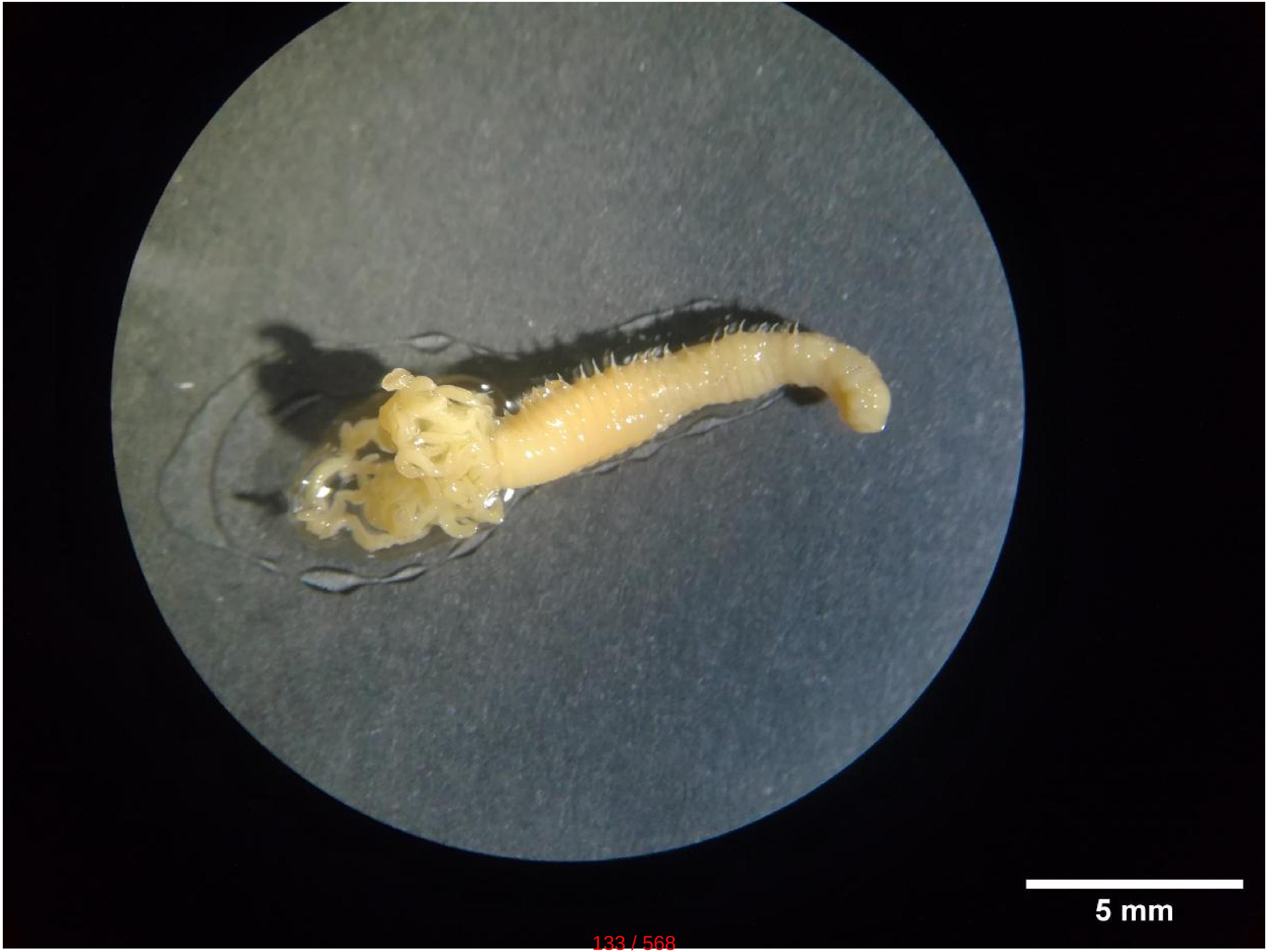
5 mm

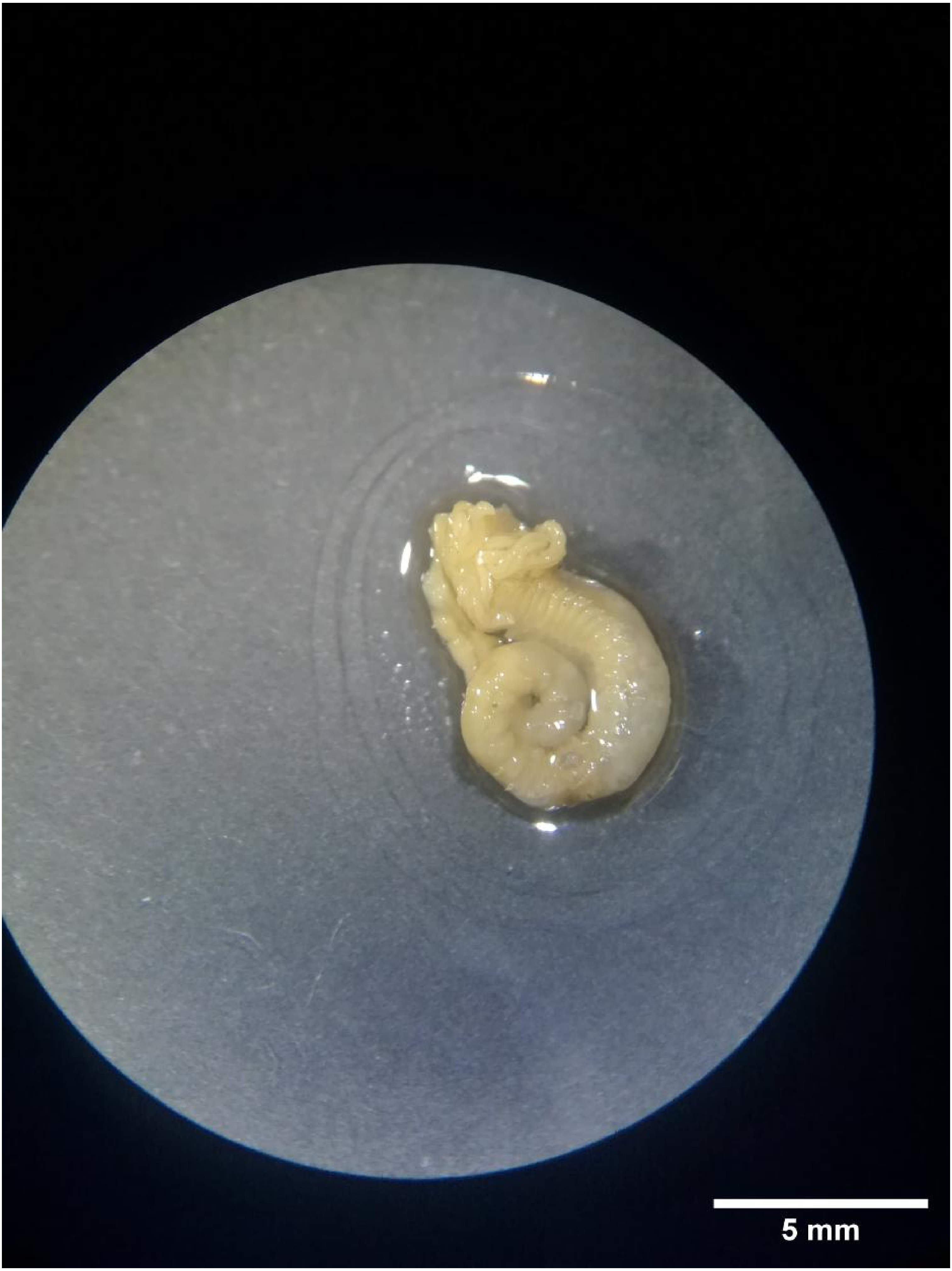
5 mm

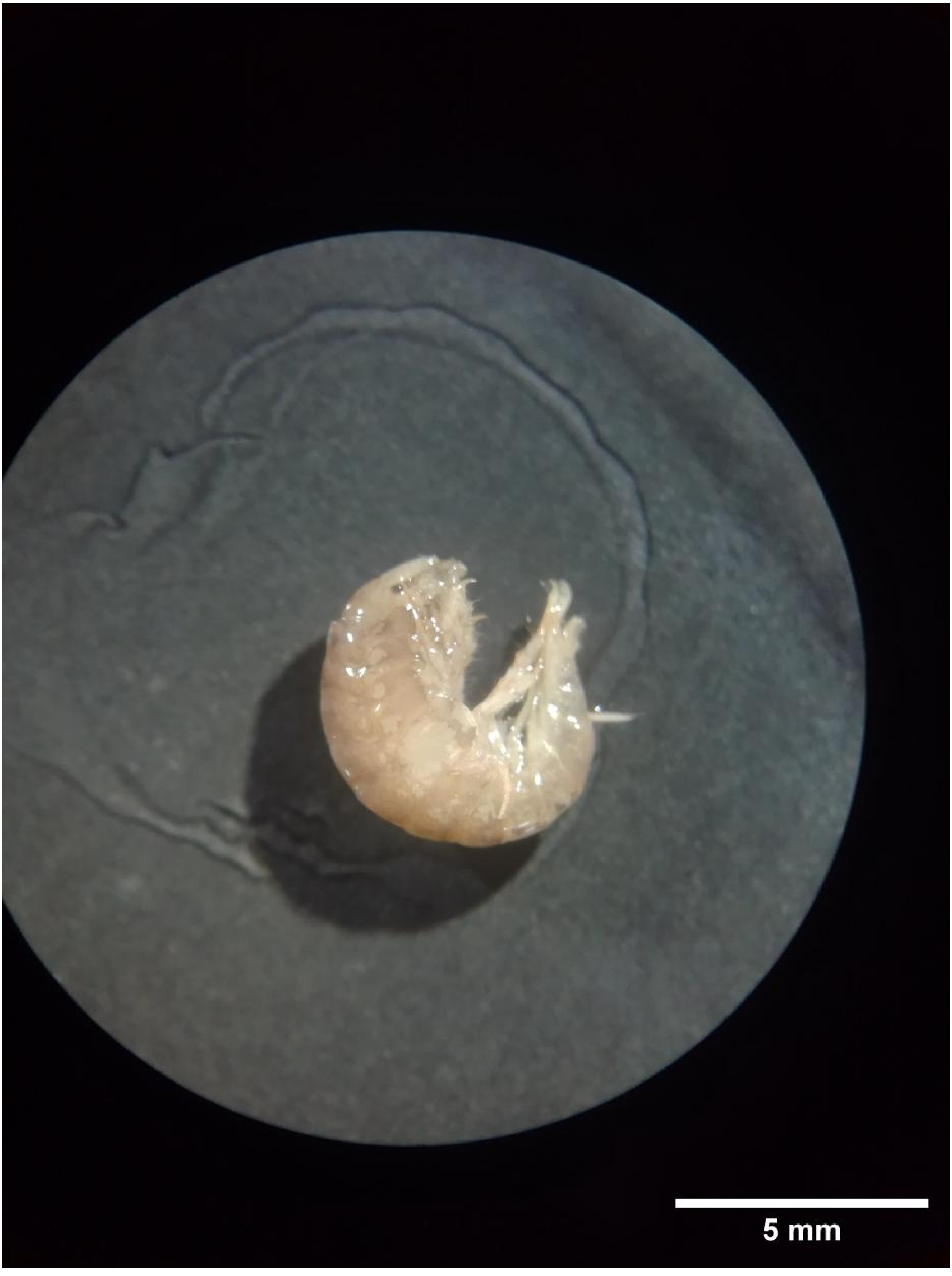
5 mm

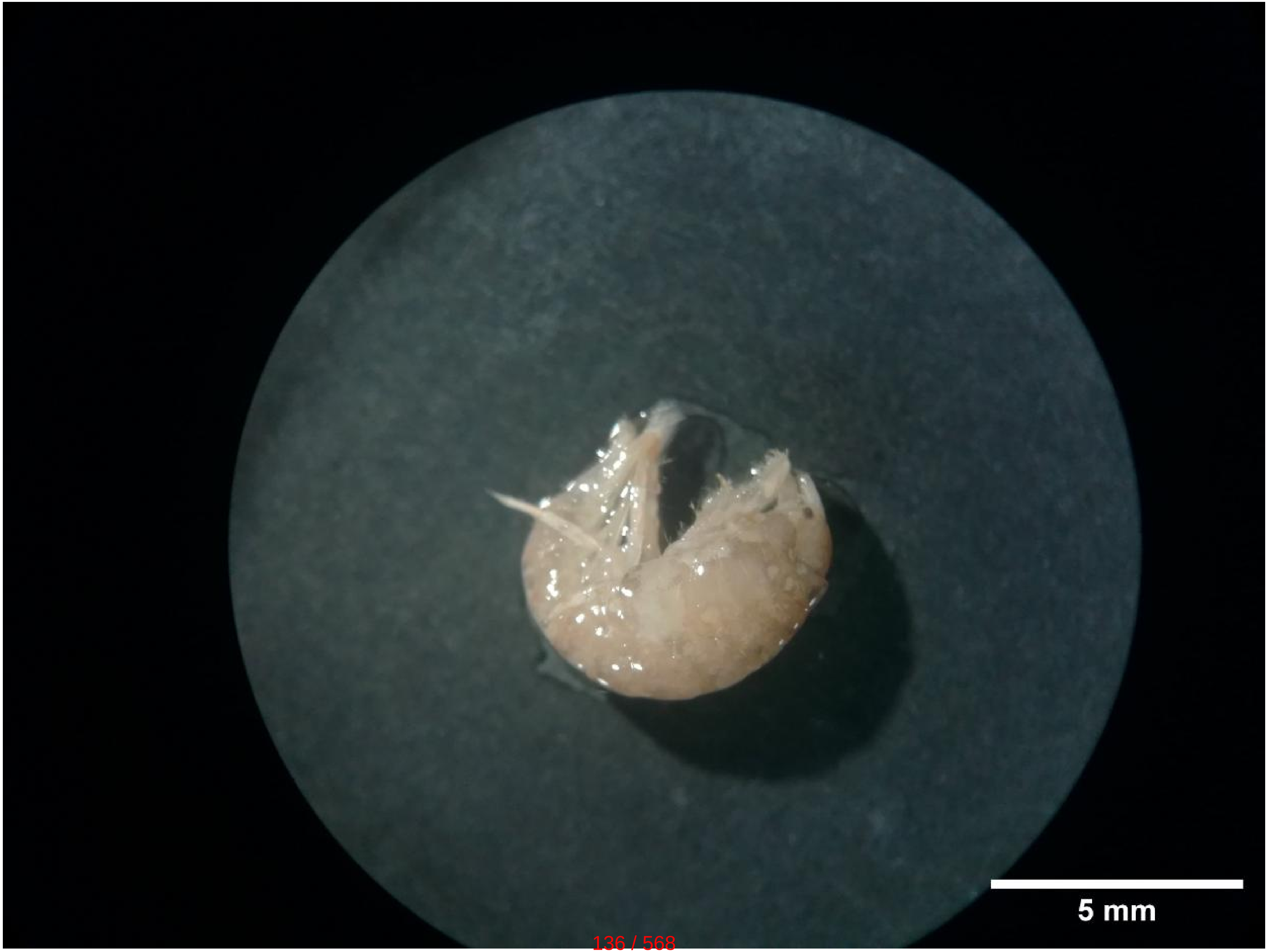
5 mm

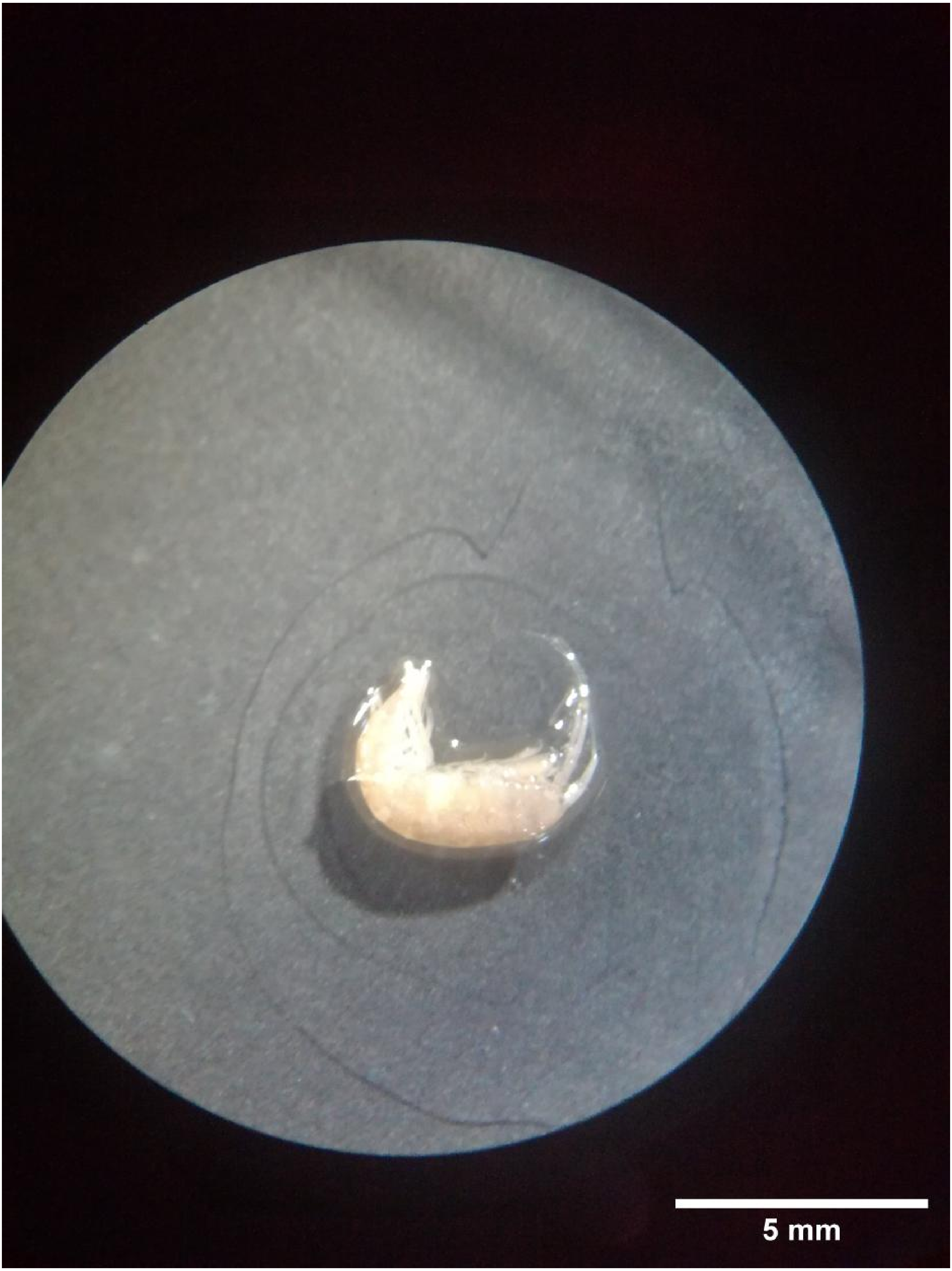
5 mm

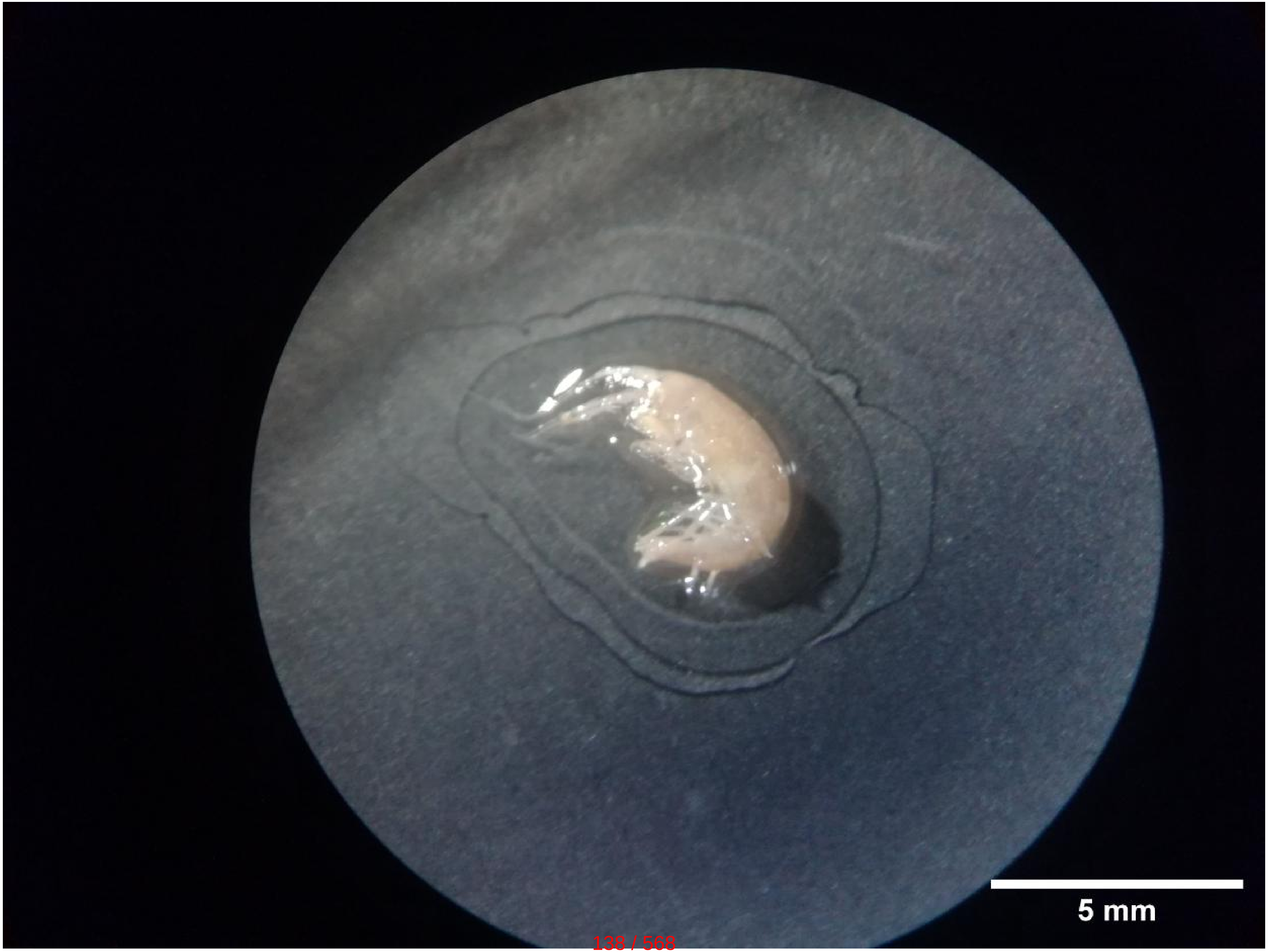
5 mm

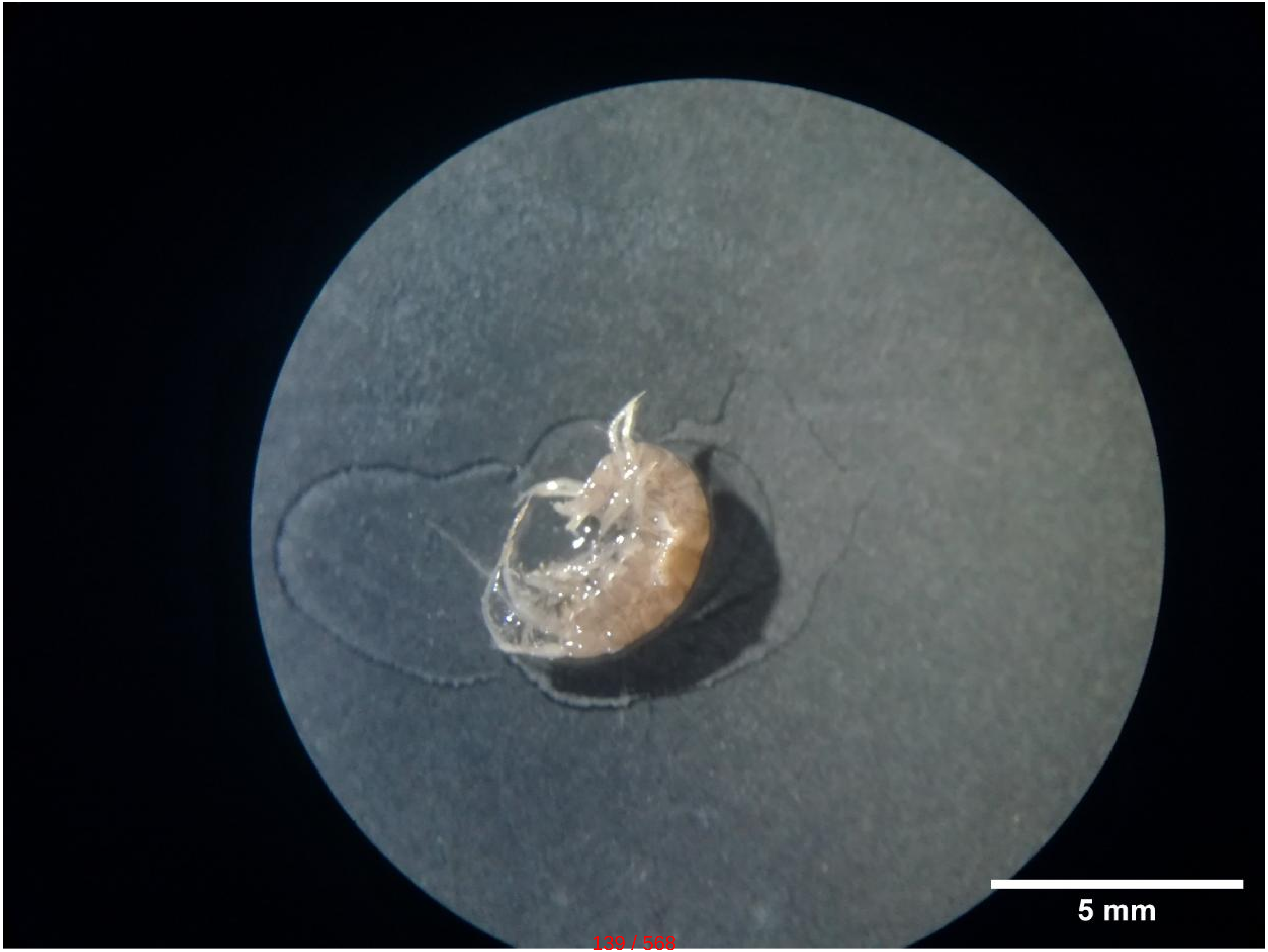
5 mm

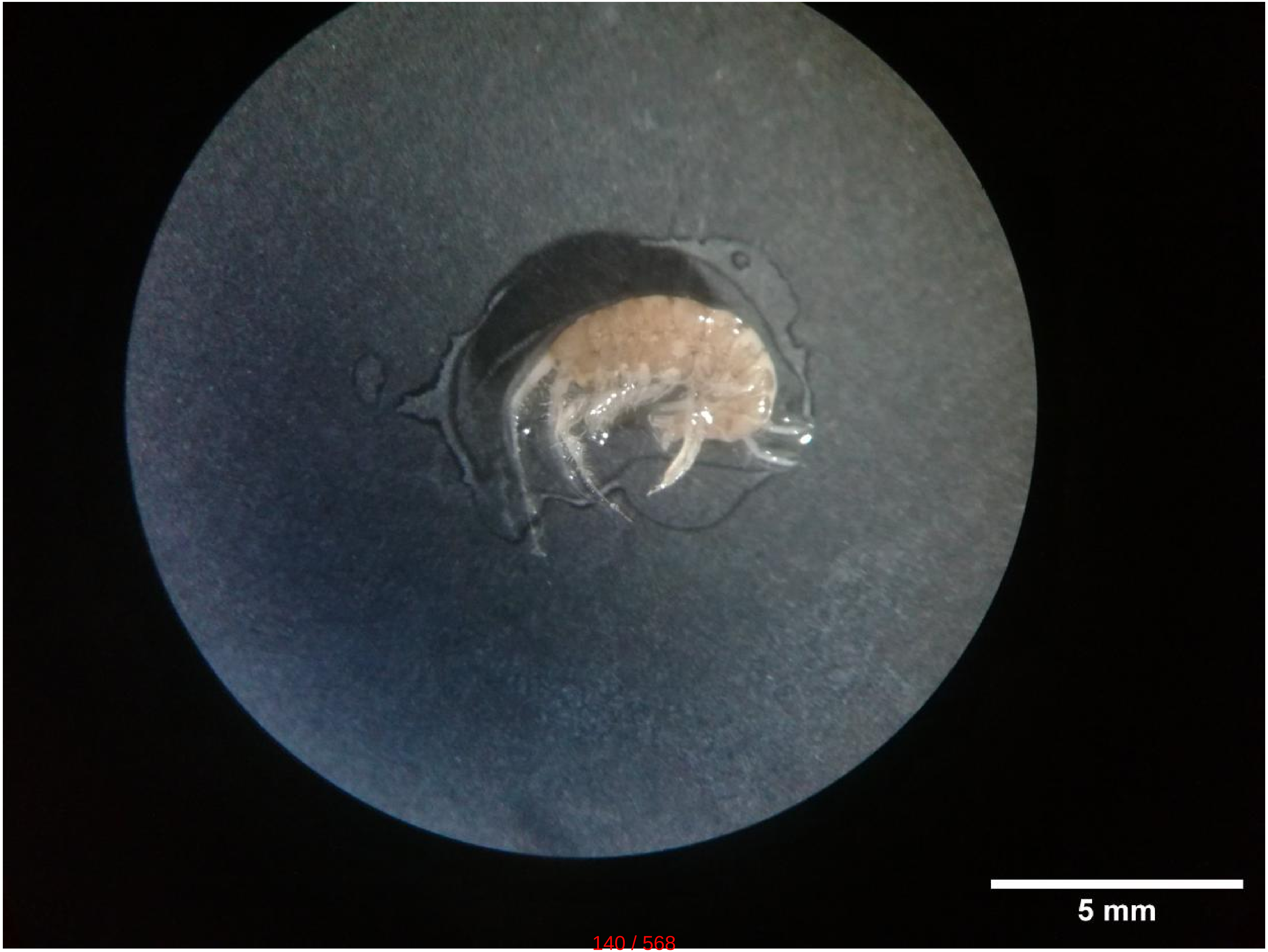
5 mm

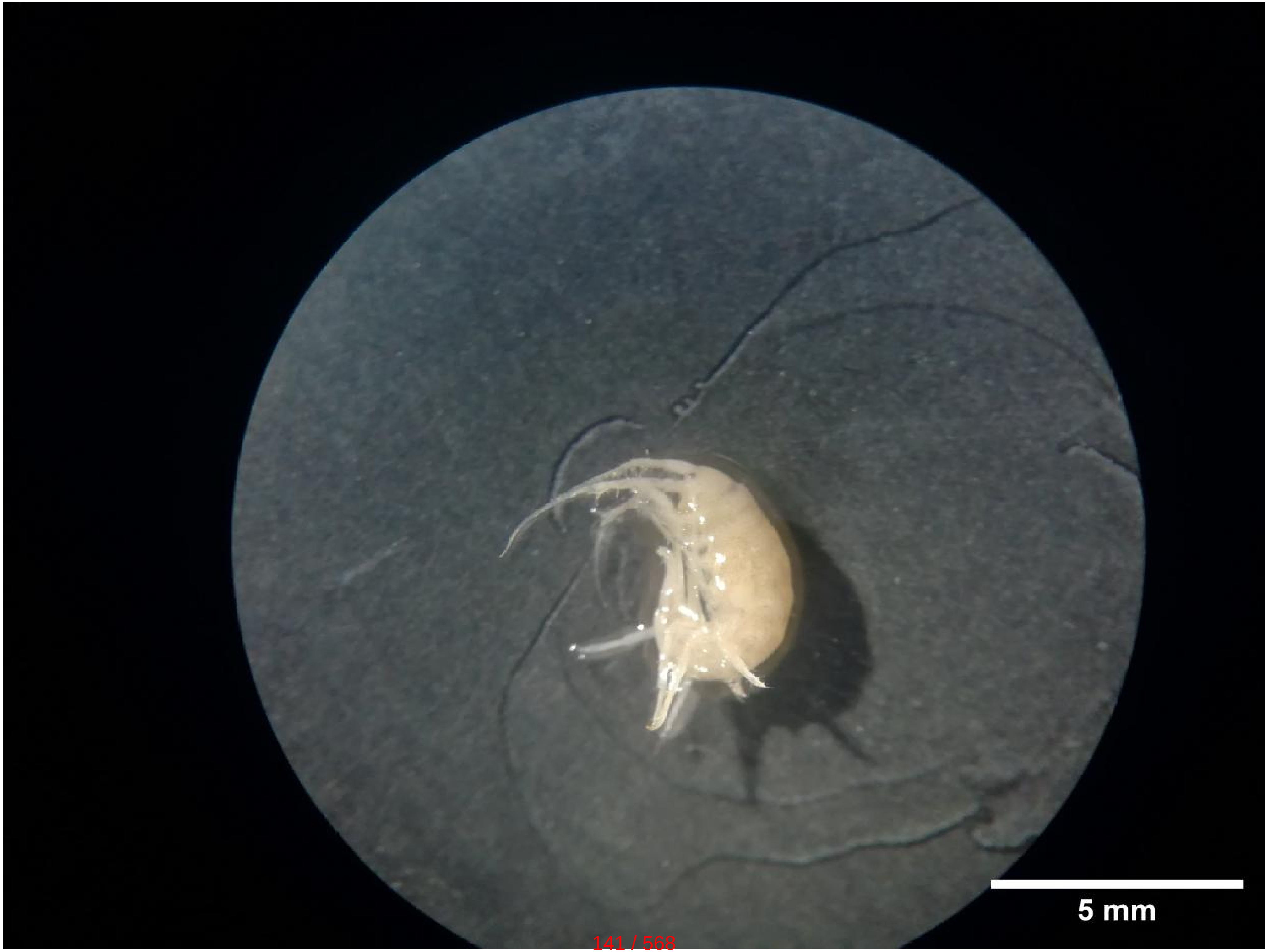
5 mm

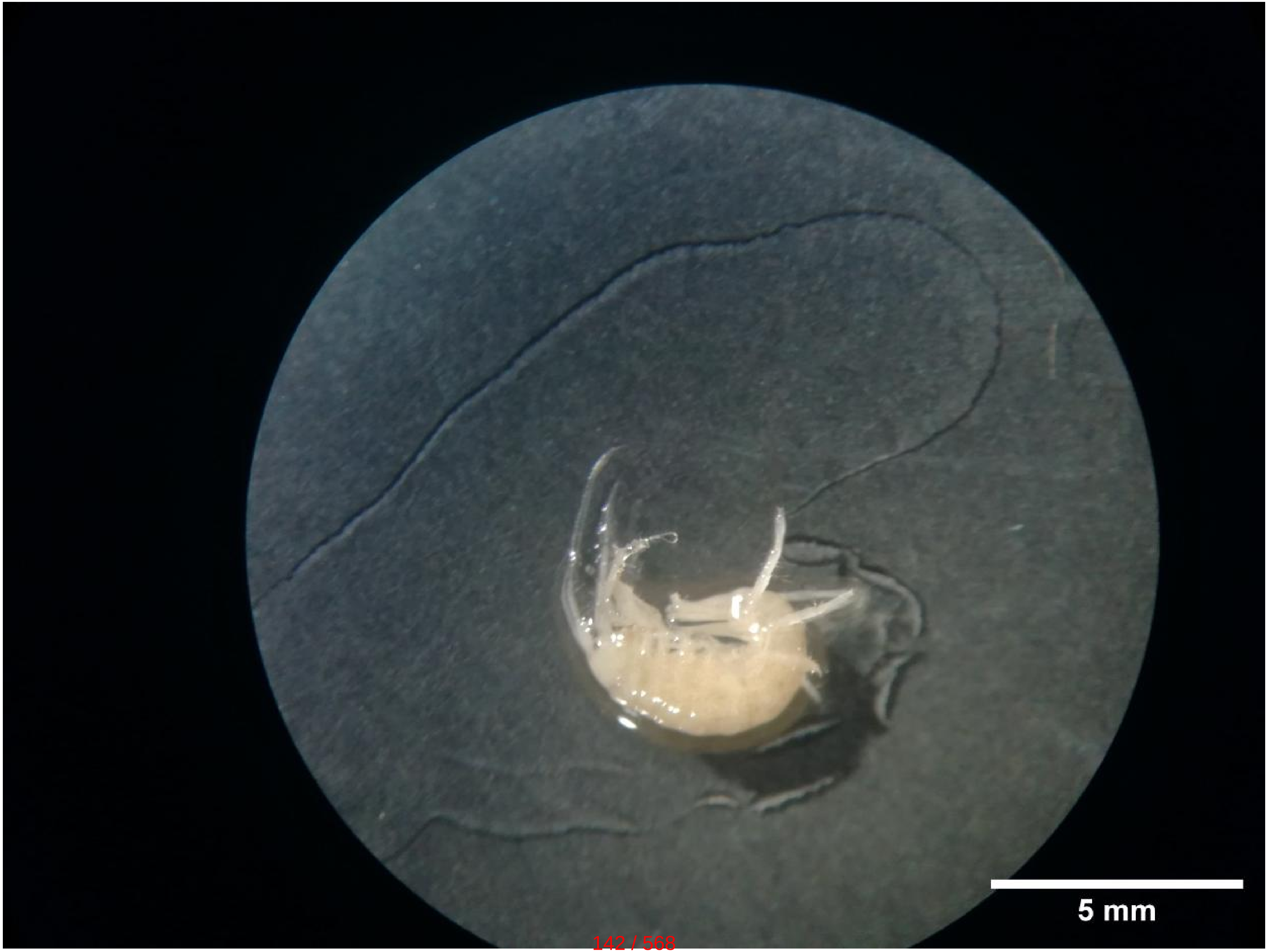
5 mm

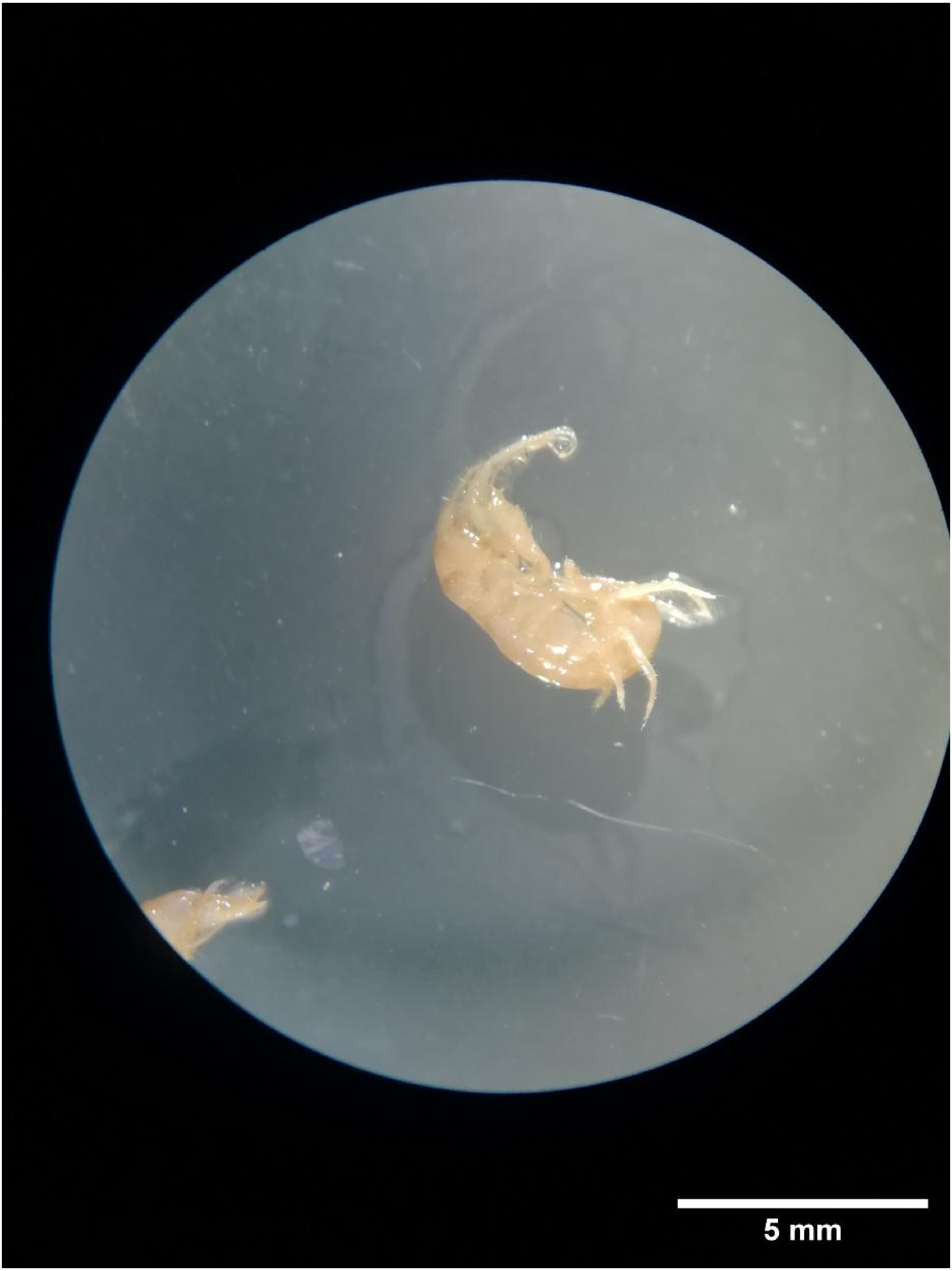
5 mm

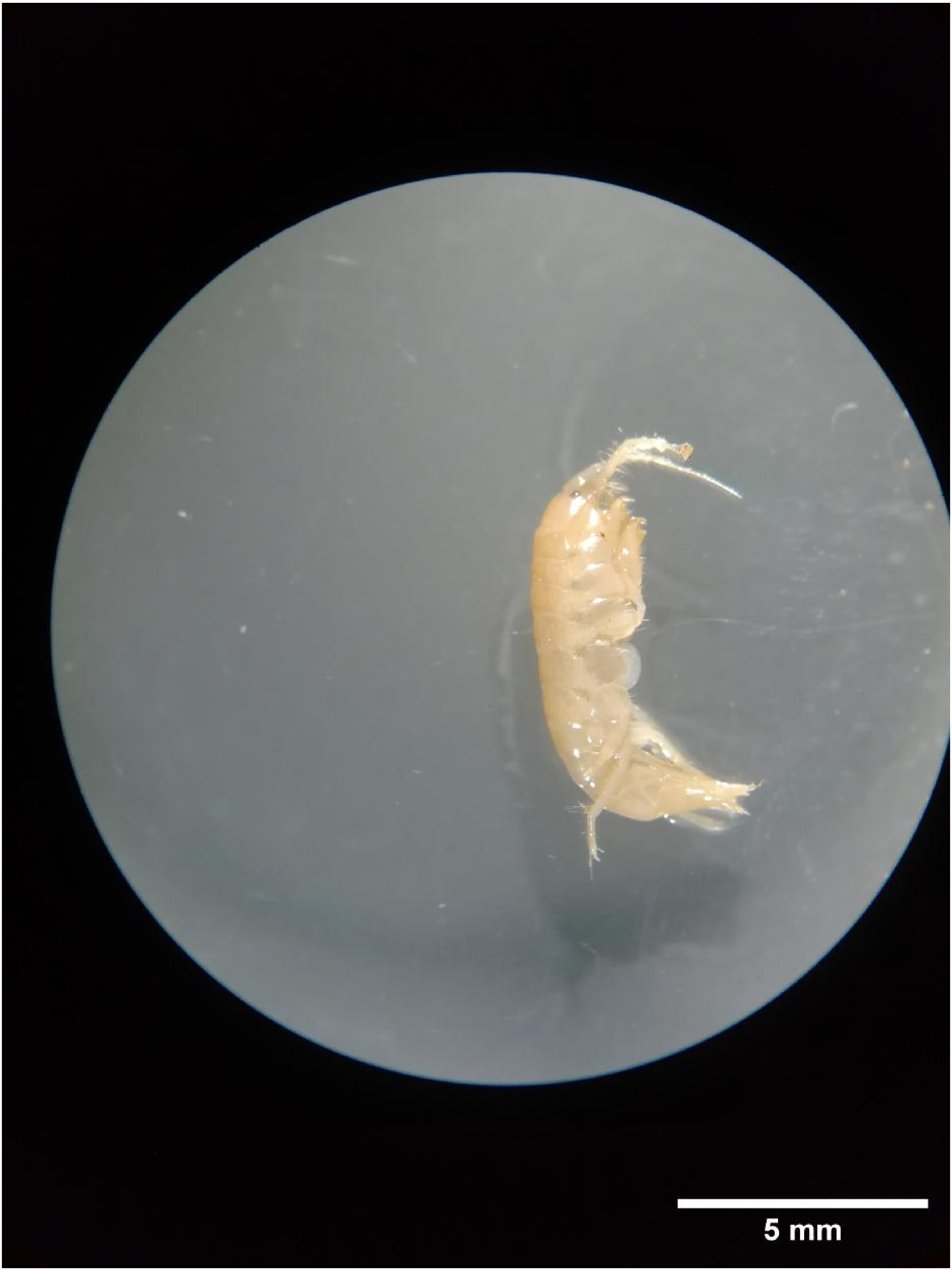
5 mm

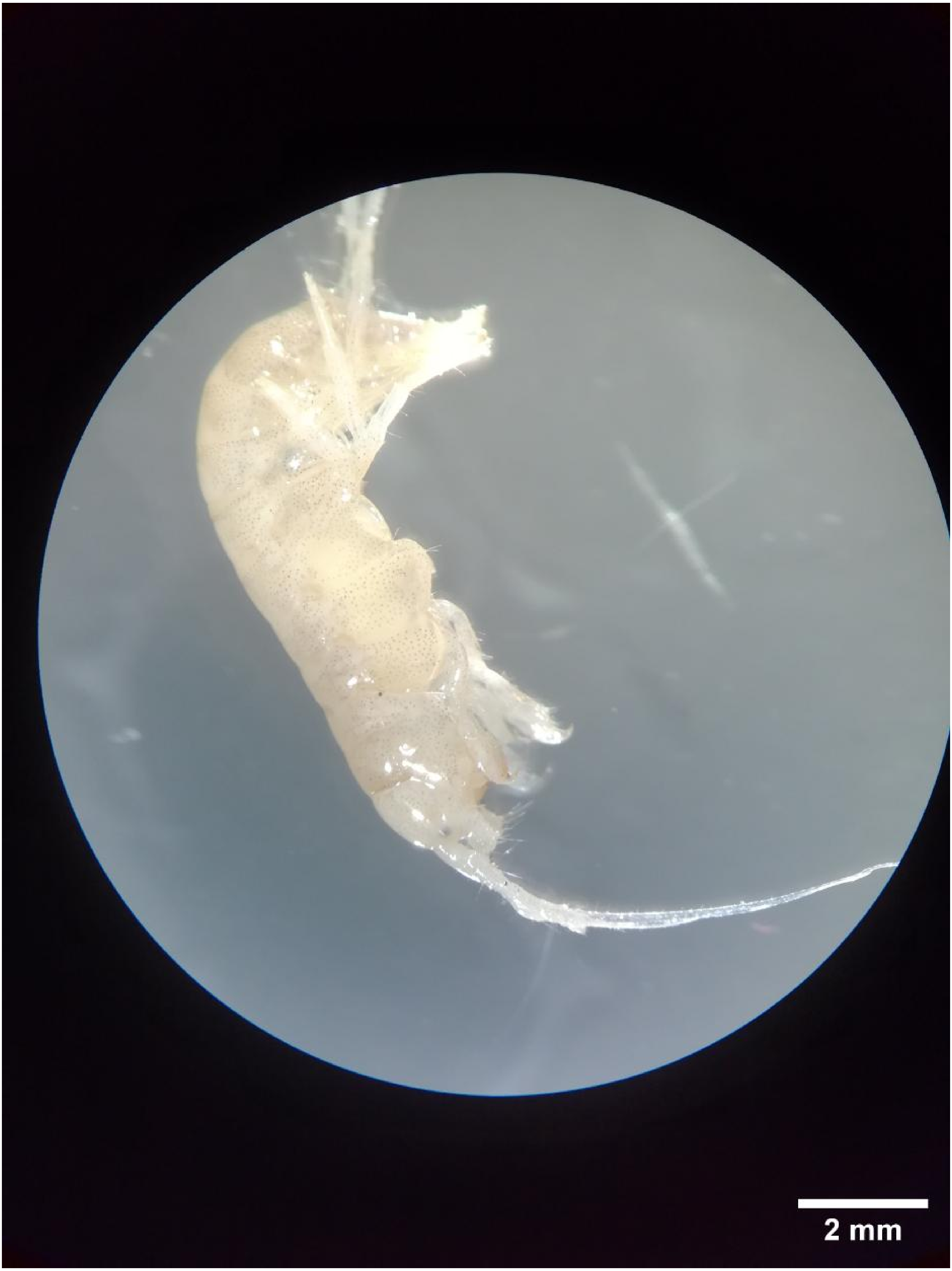
2 mm

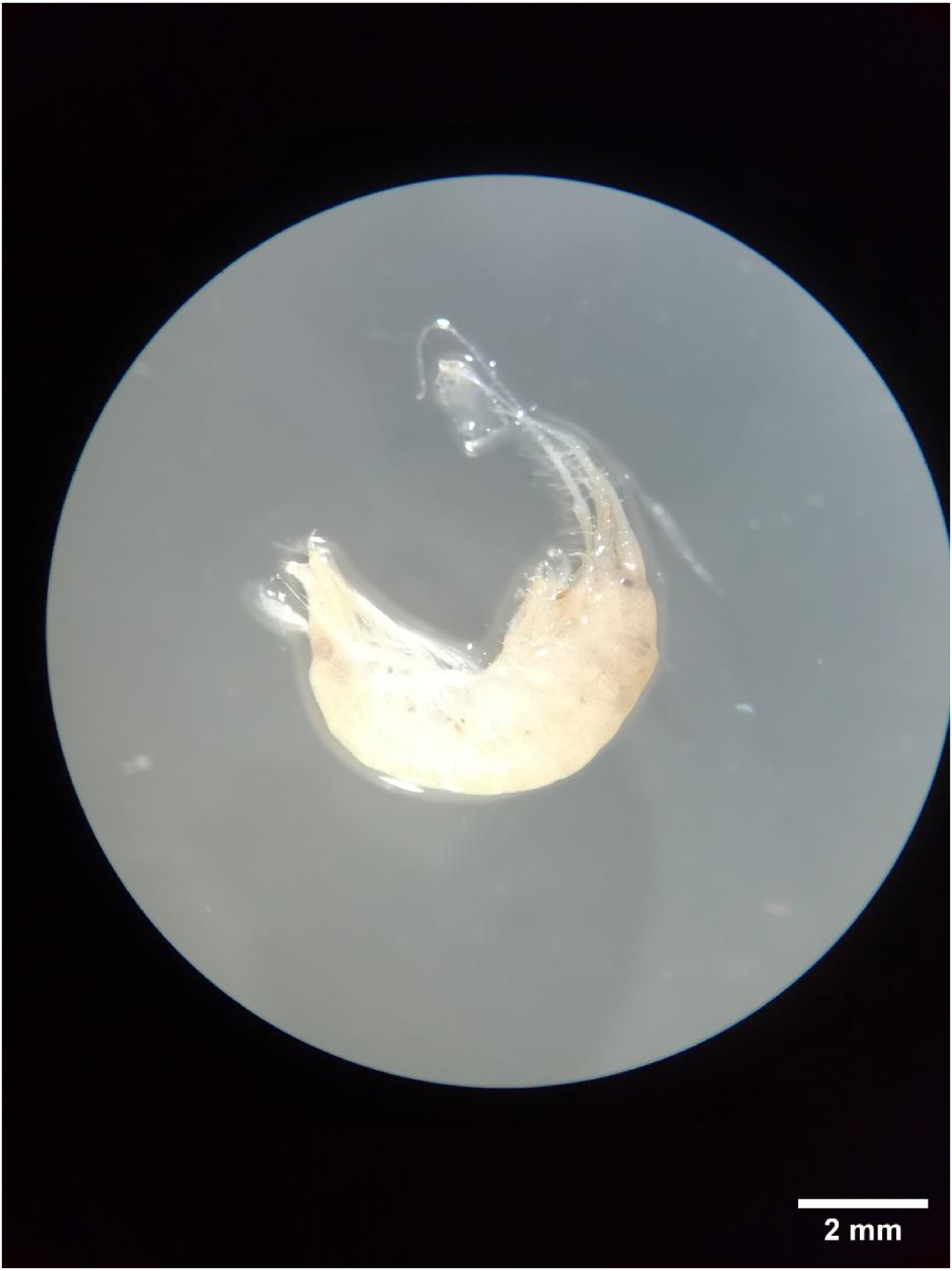
2 mm

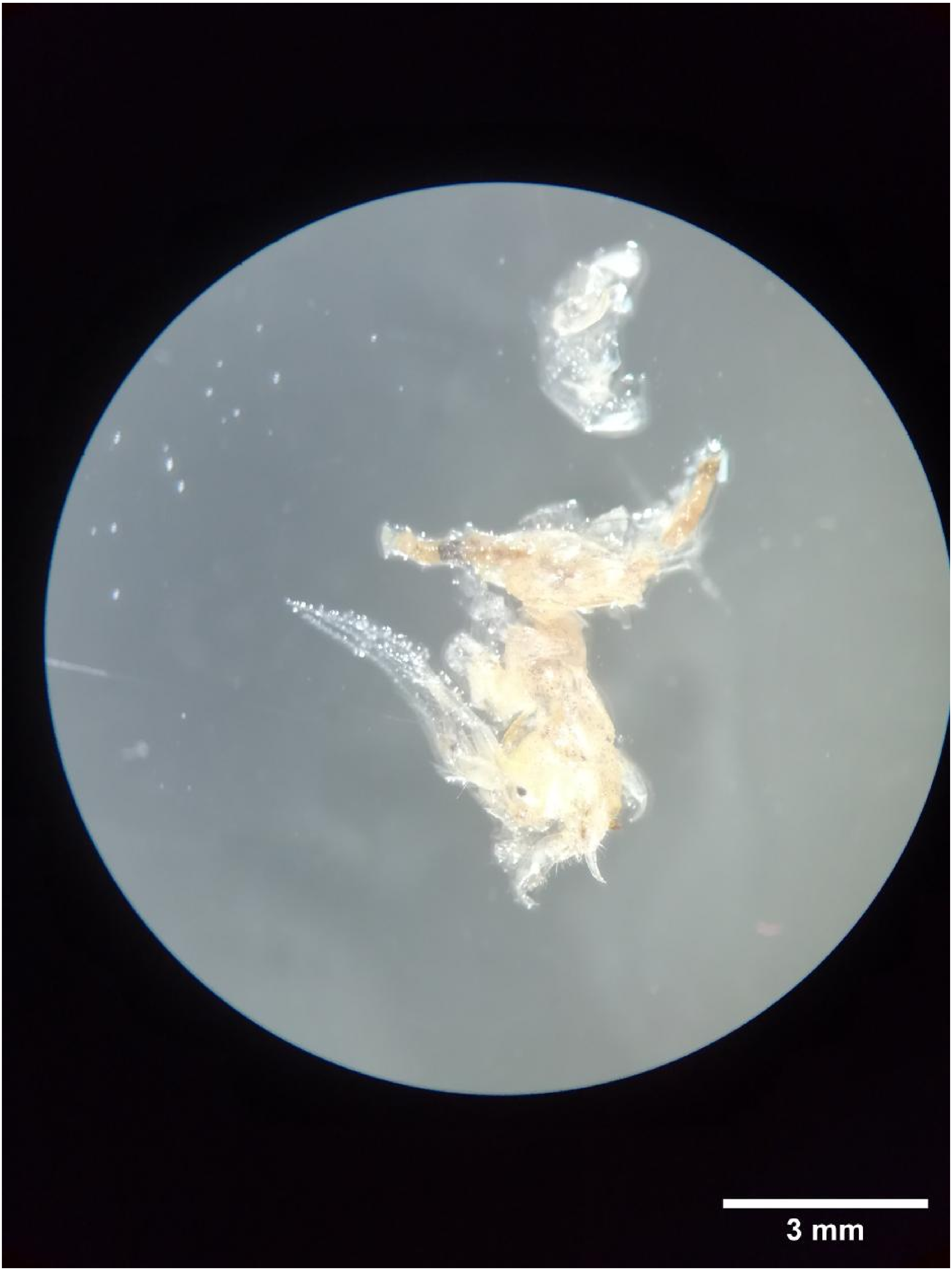
3 mm

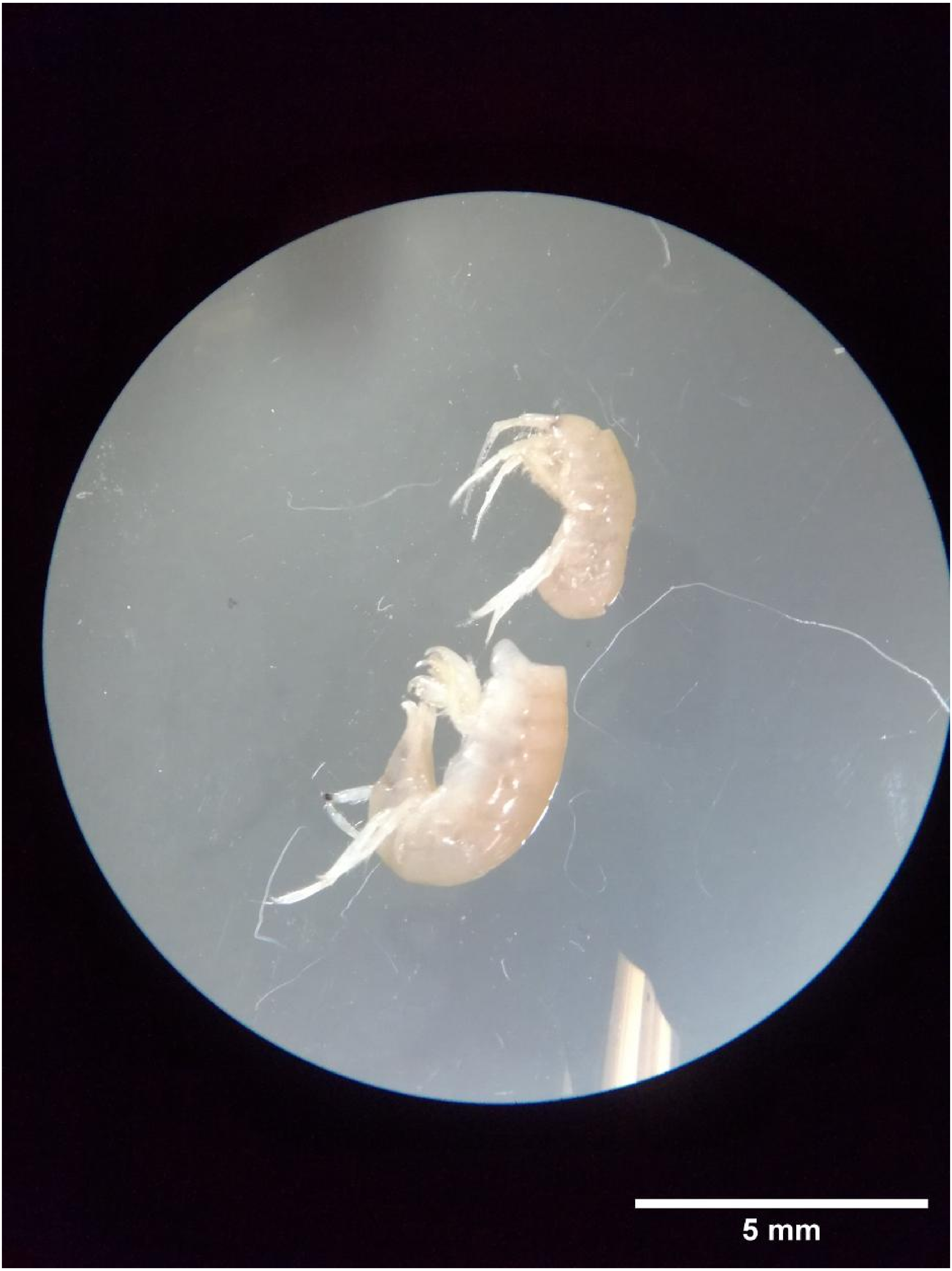
5 mm

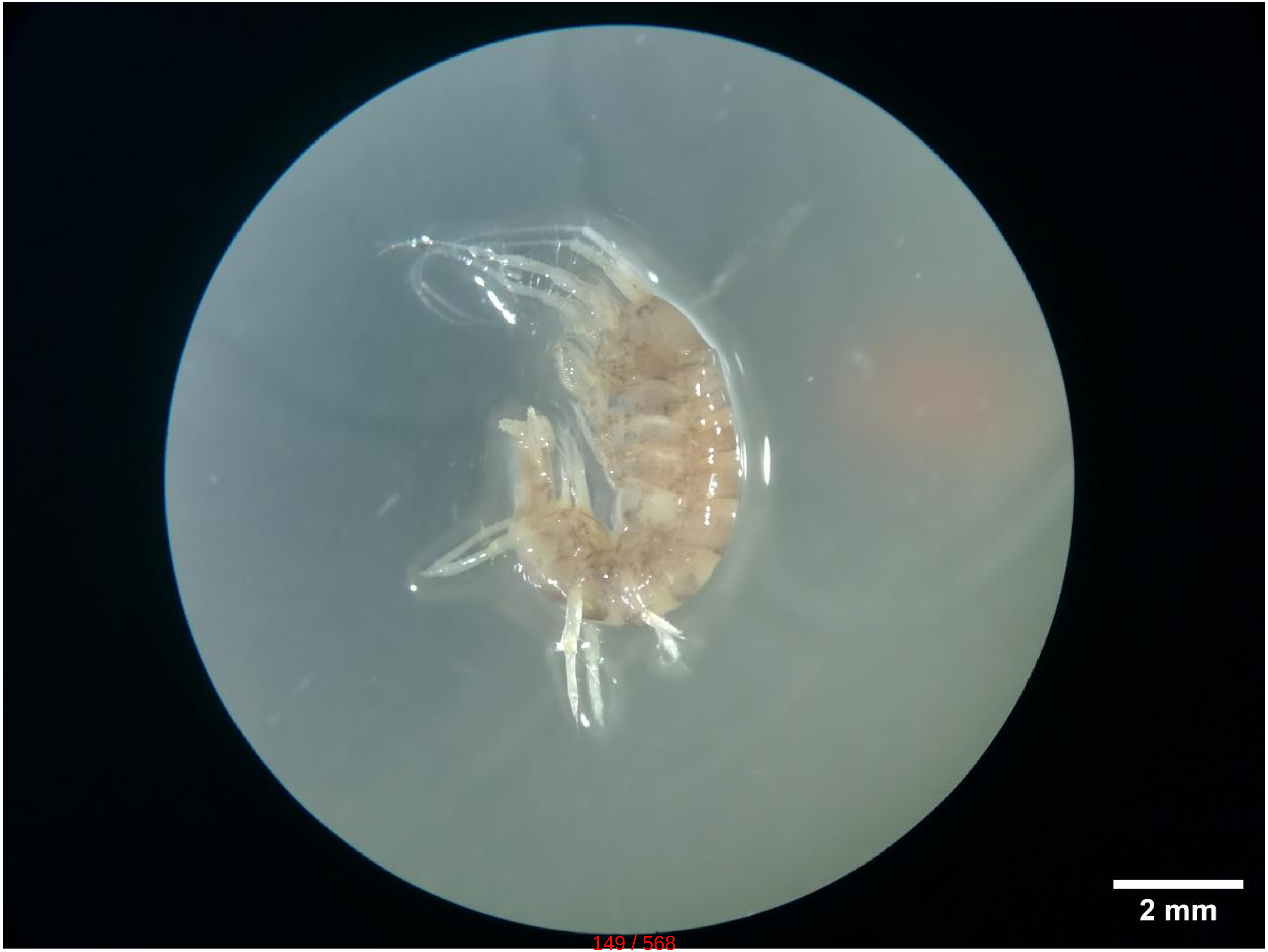
2 mm

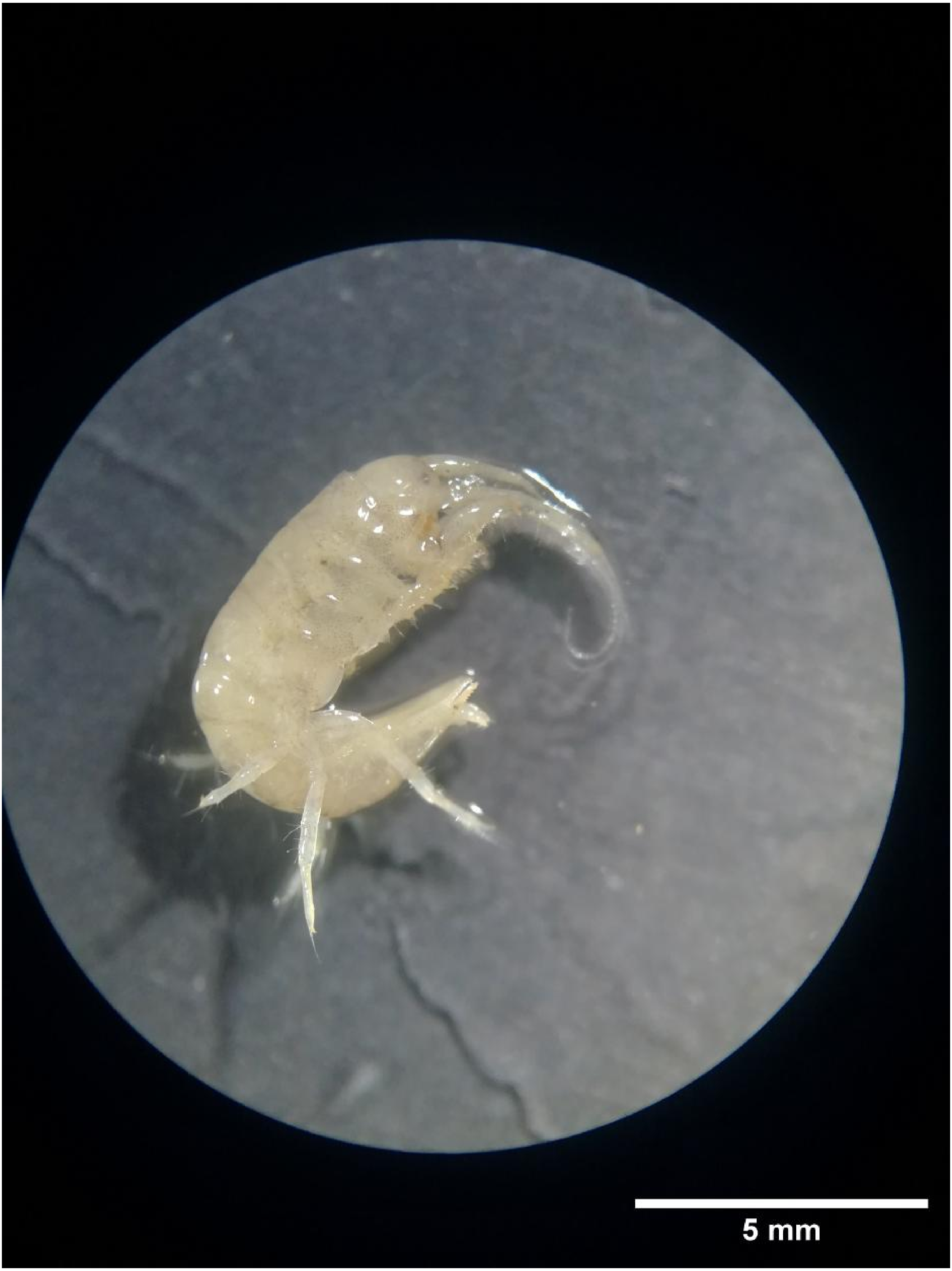
5 mm

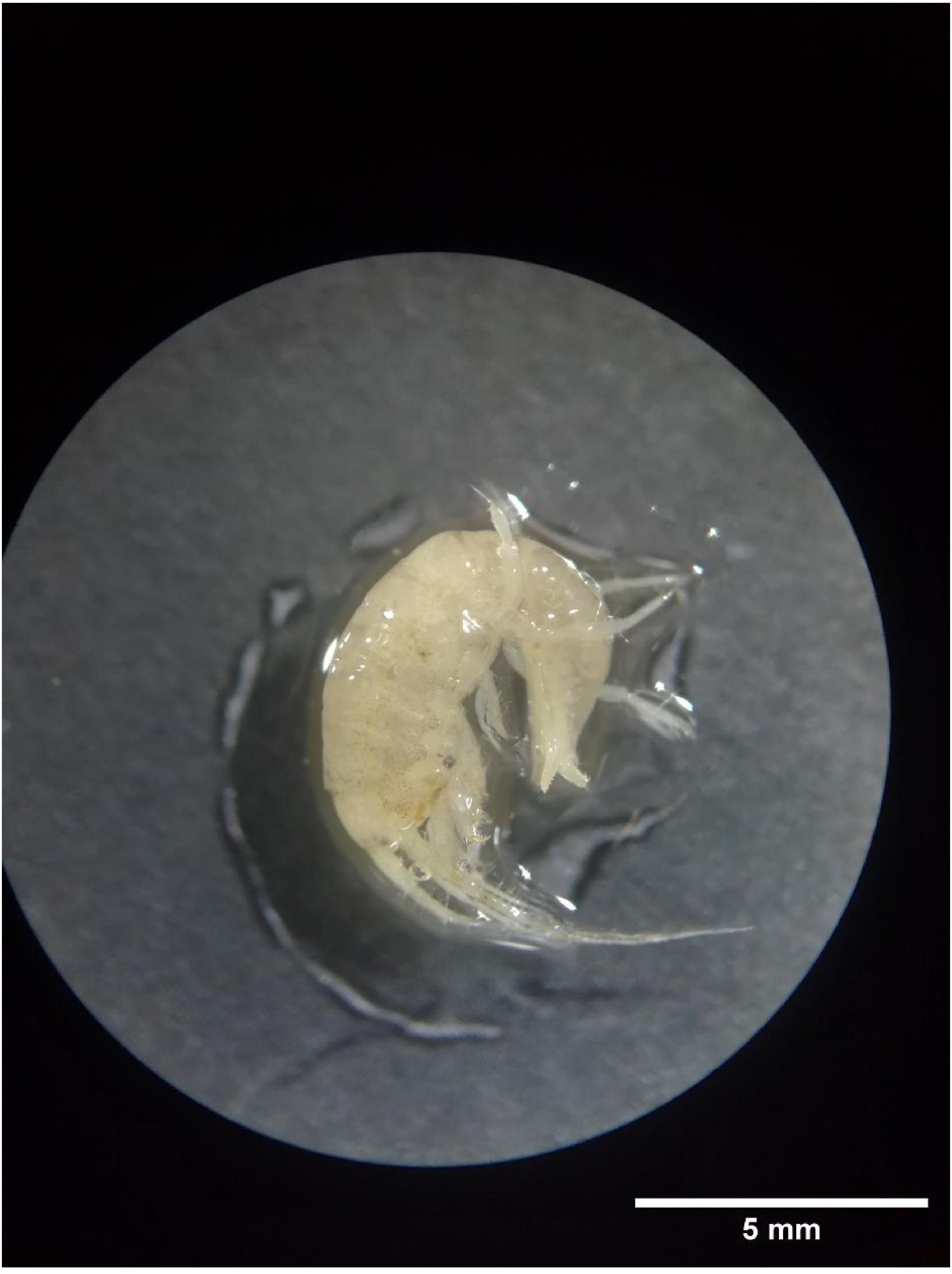
5 mm

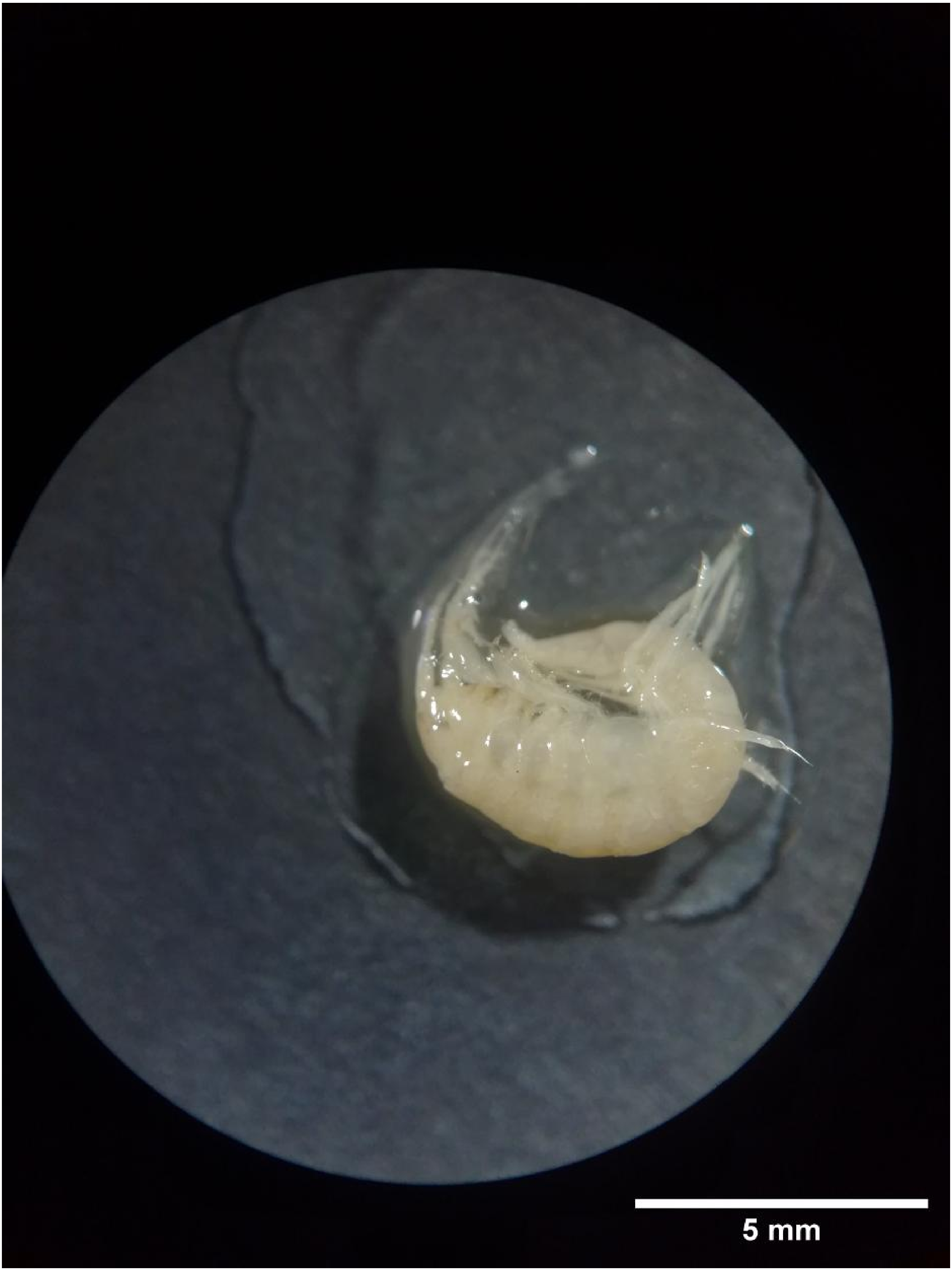
5 mm

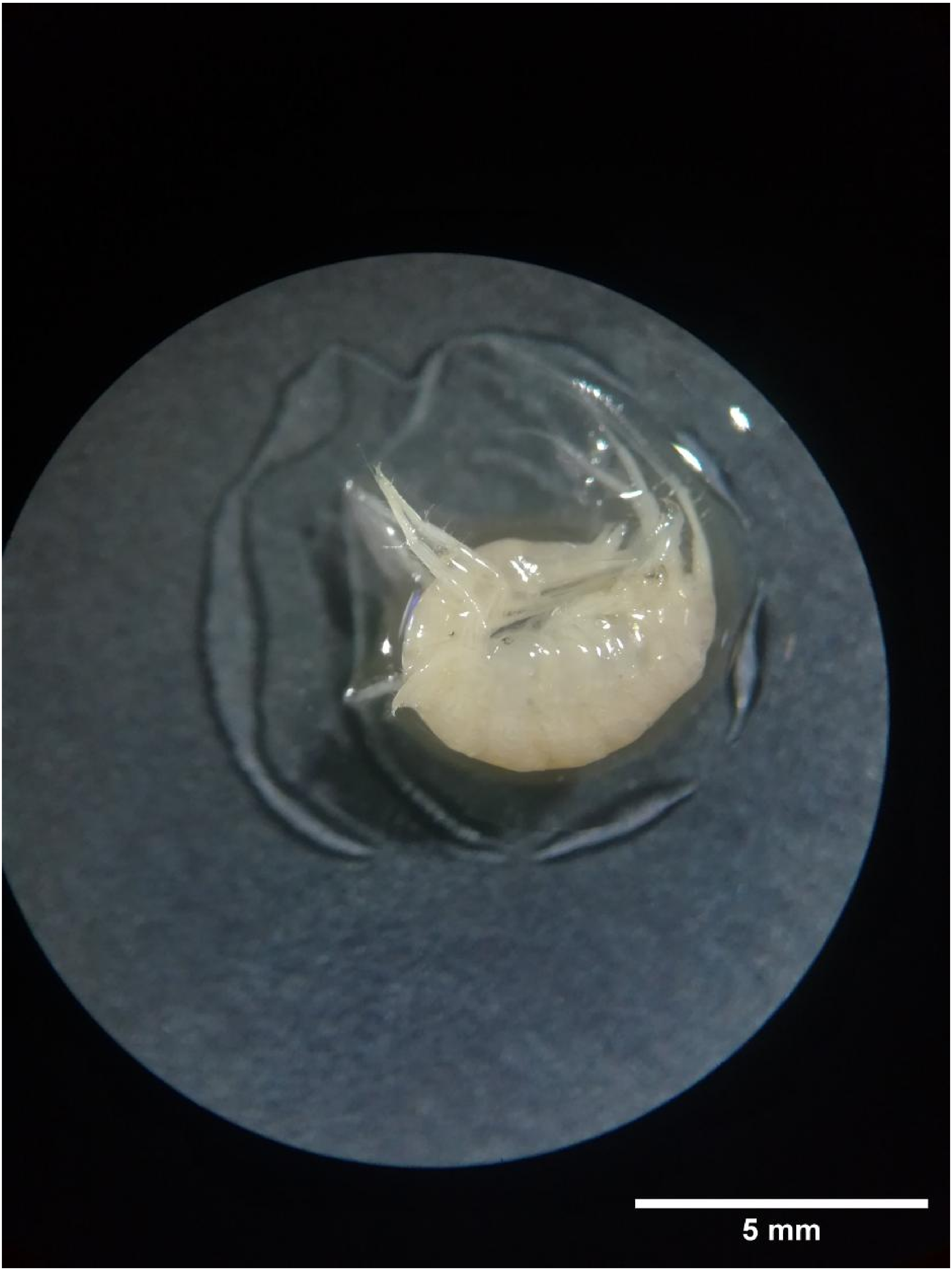
5 mm

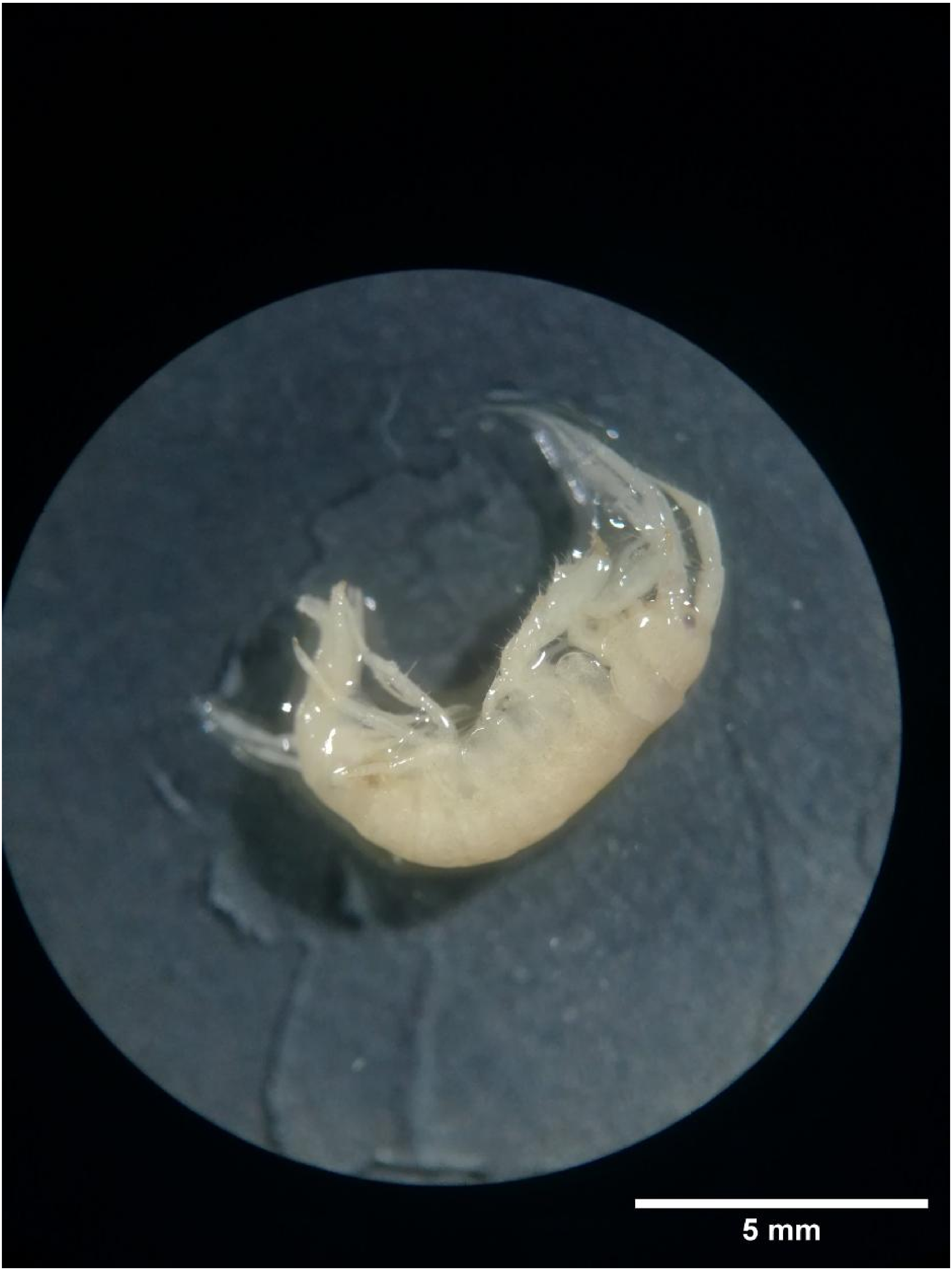
5 mm

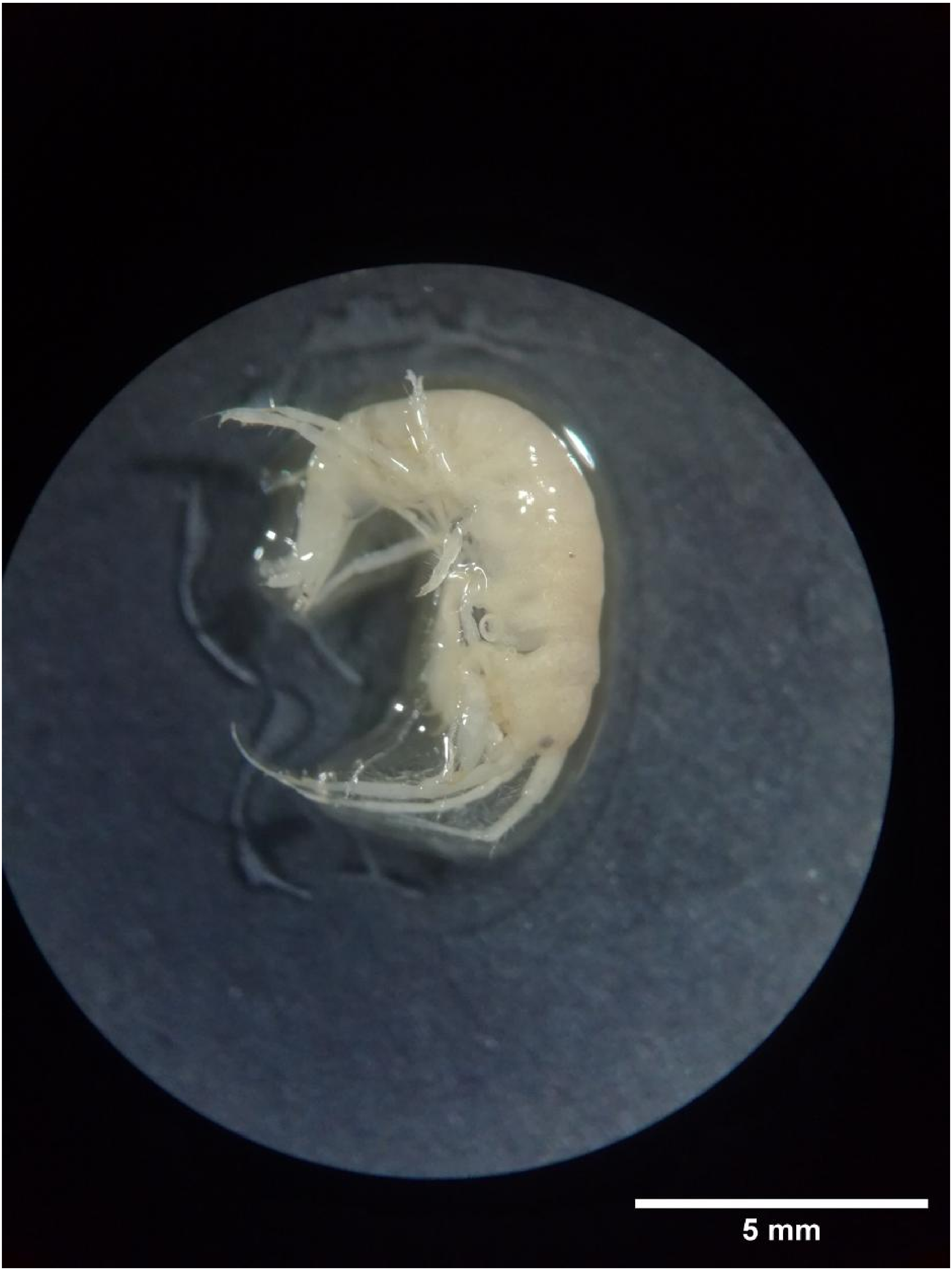
5 mm

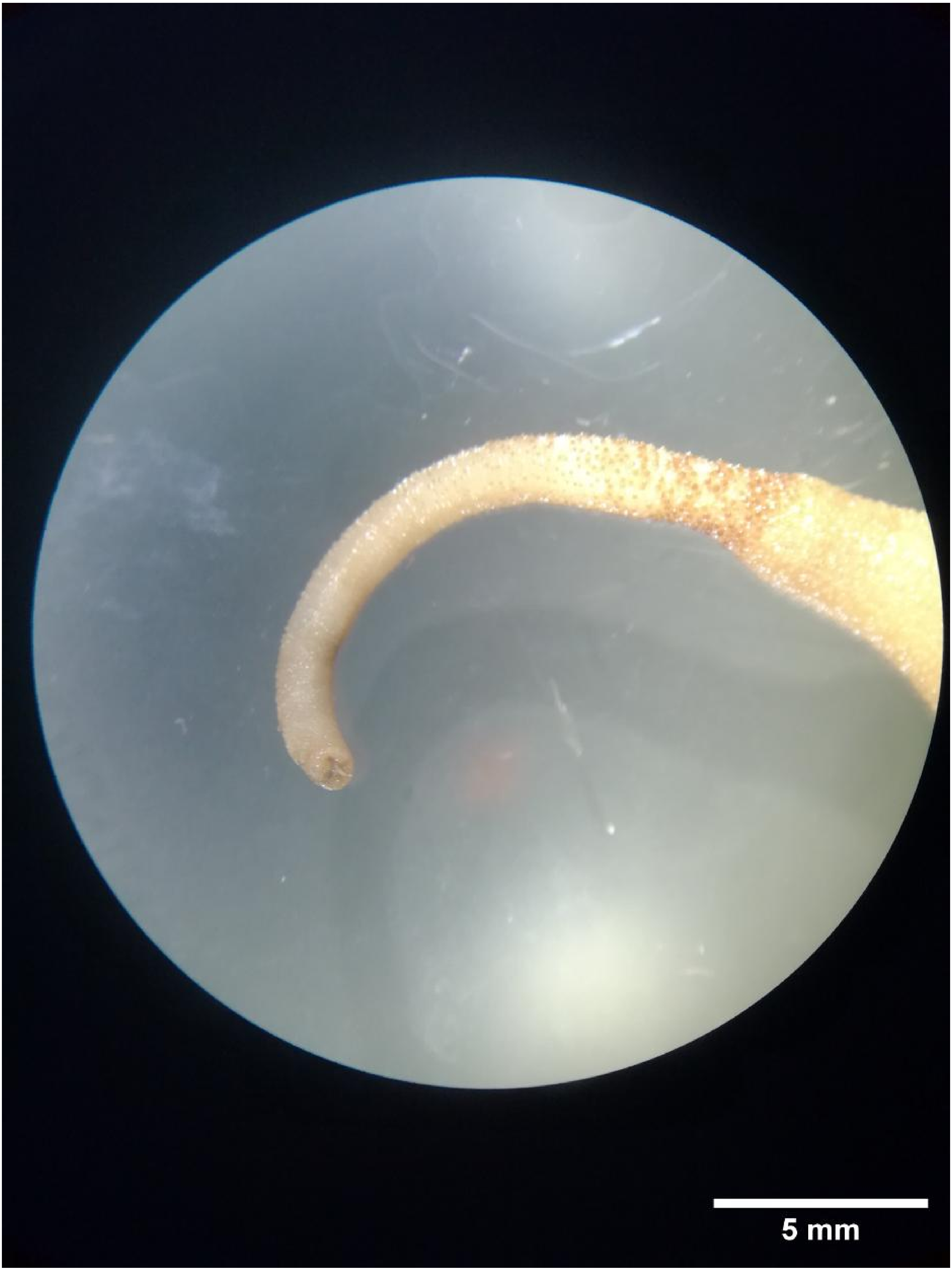
5 mm

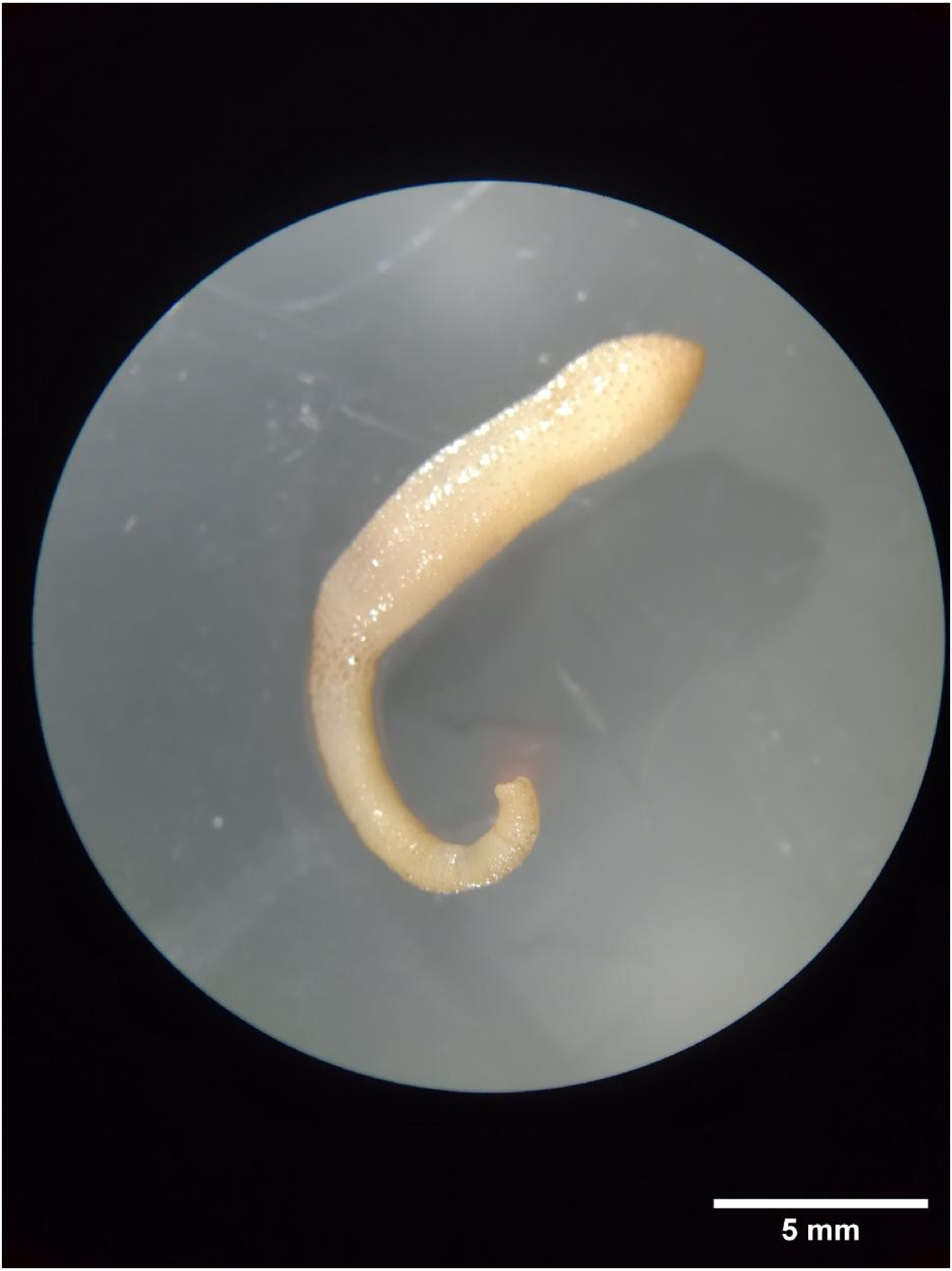
5 mm

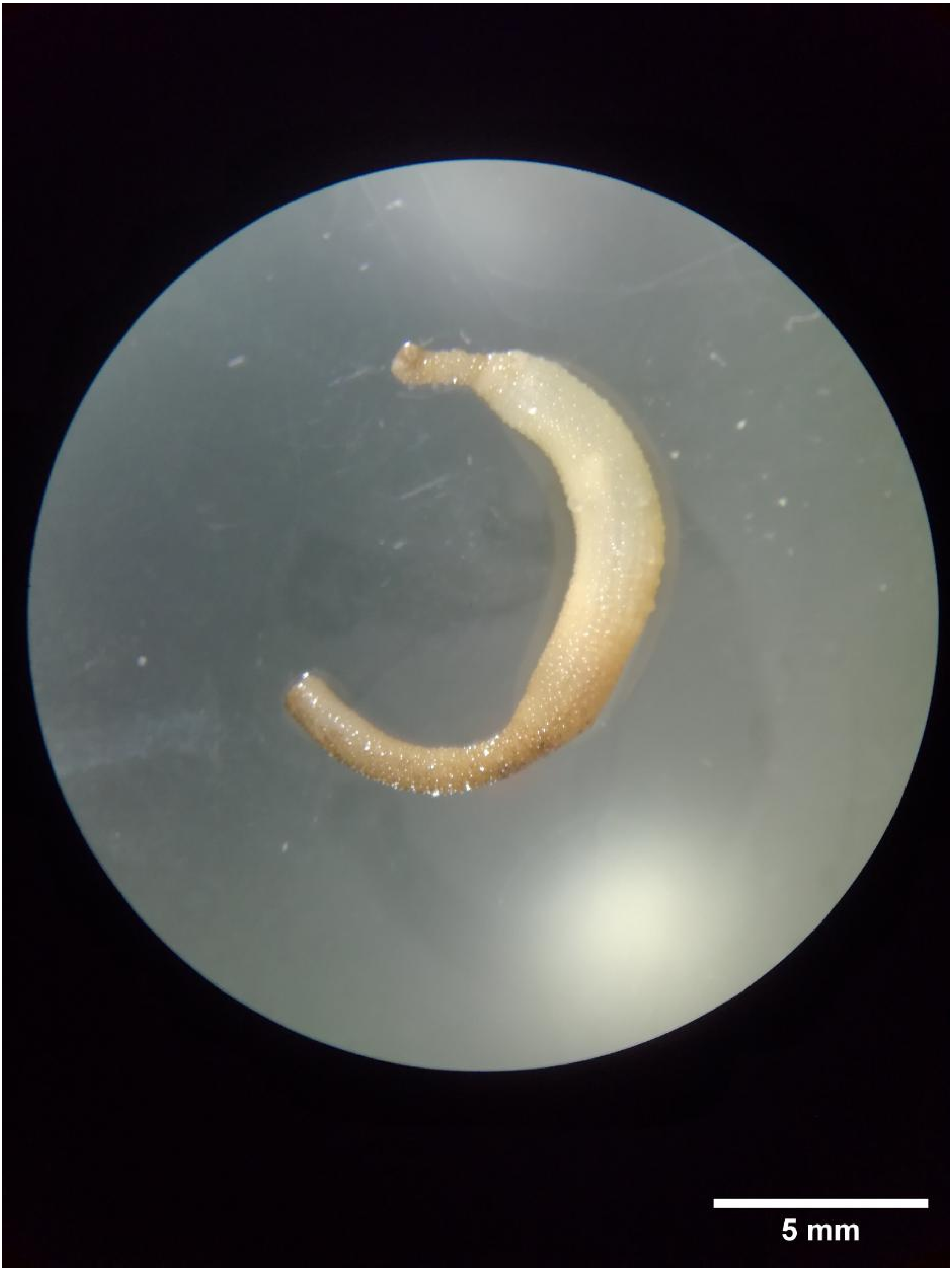
5 mm

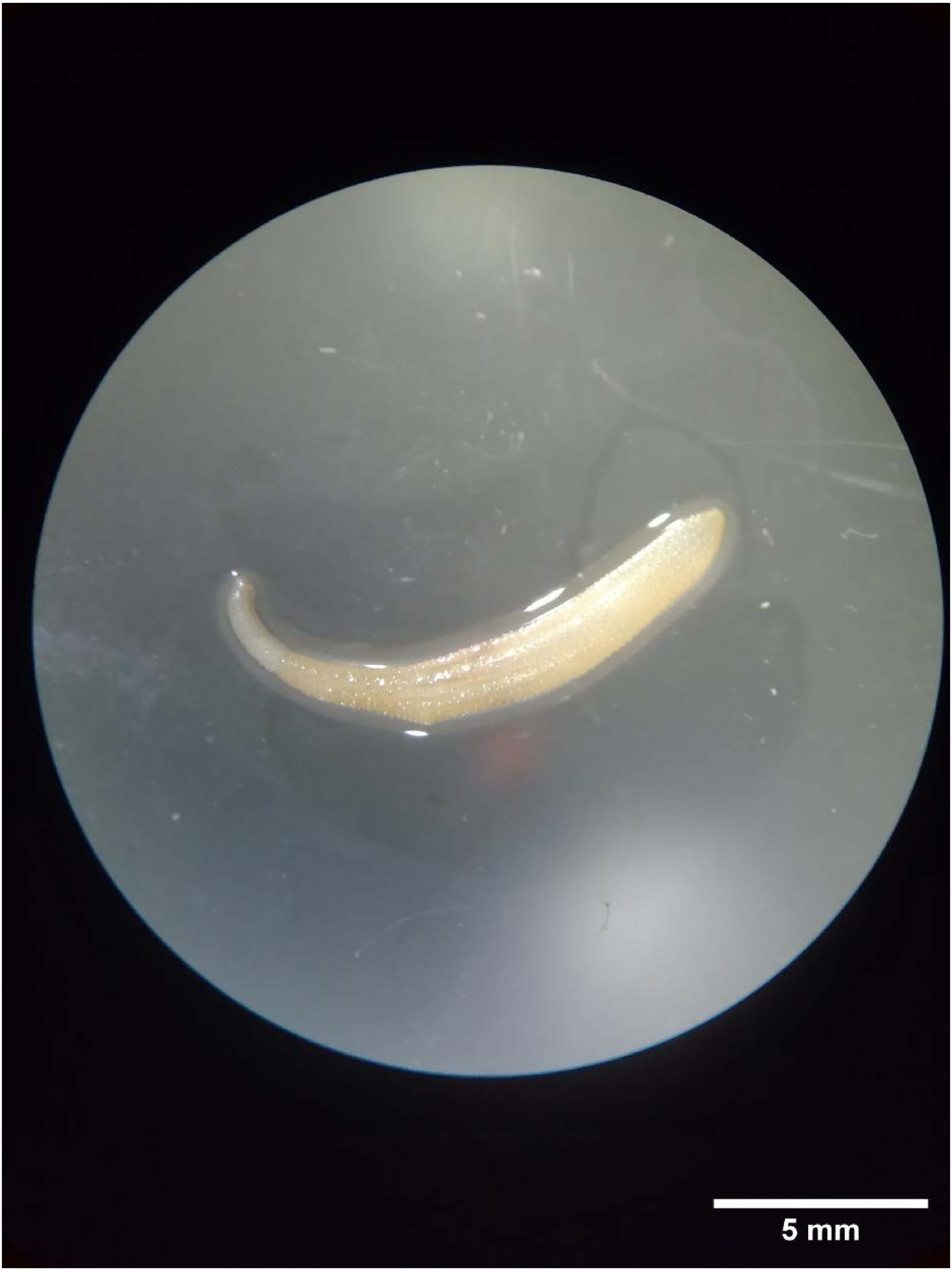
5 mm

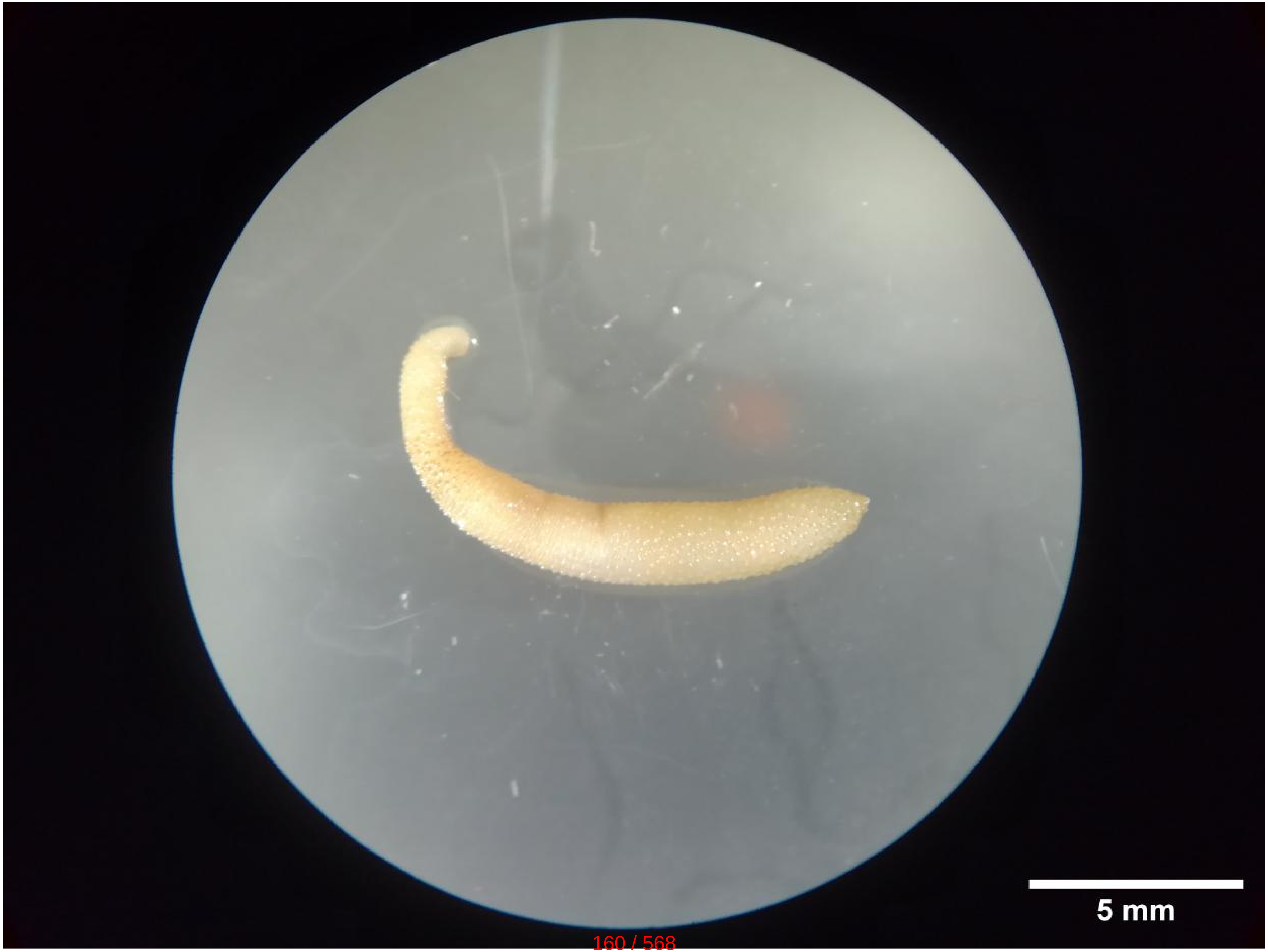
5 mm

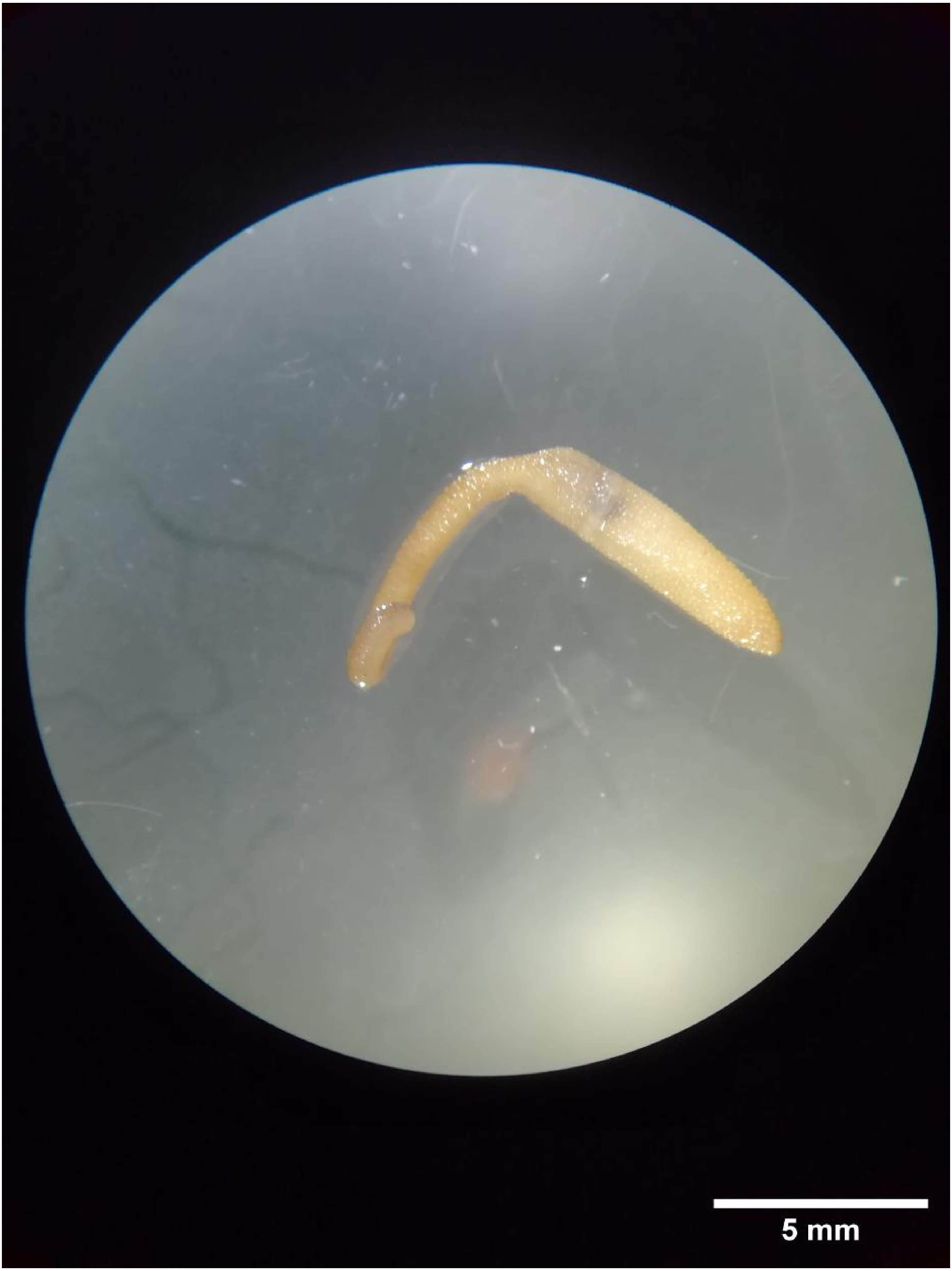
5 mm

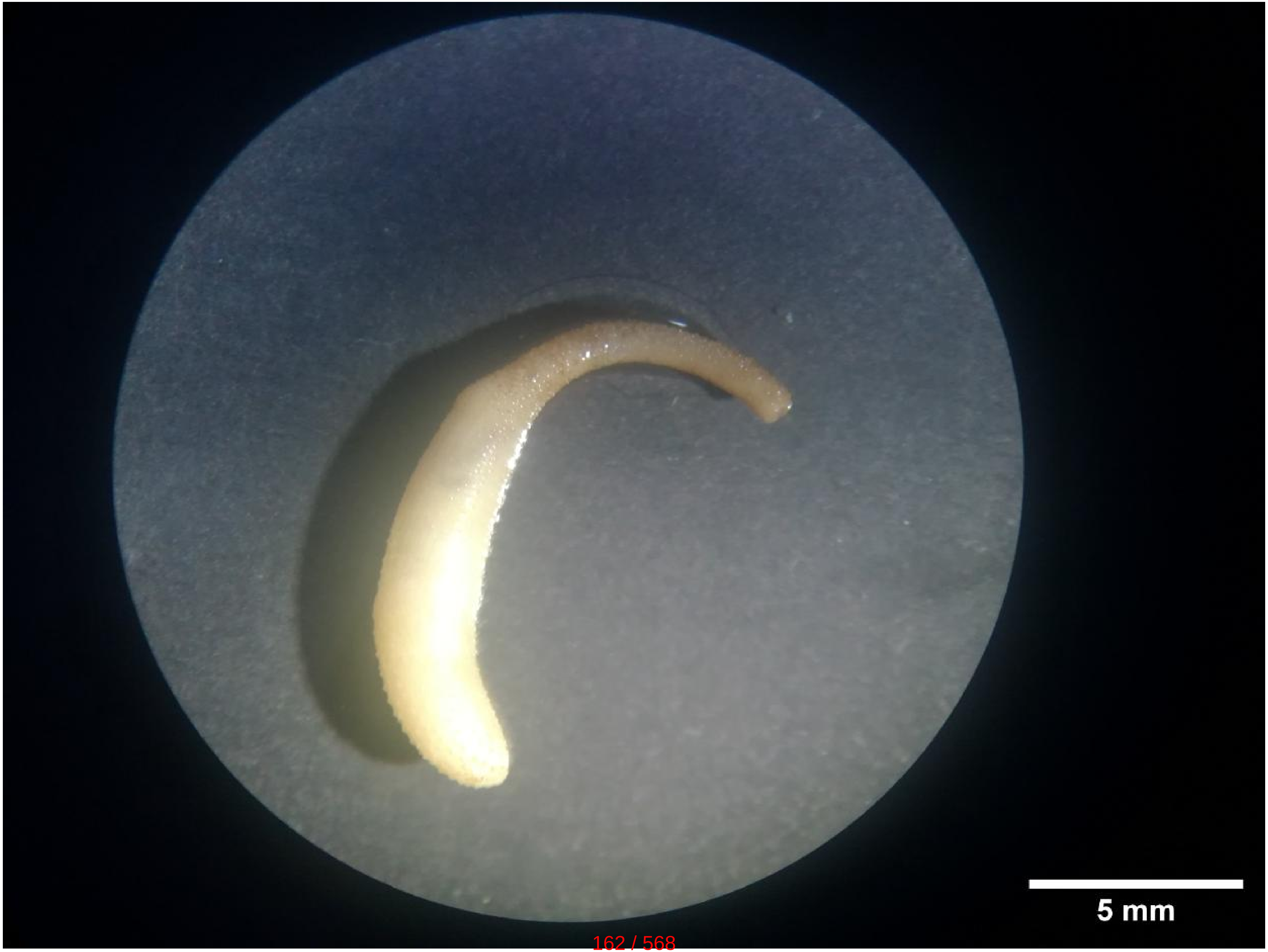
5 mm

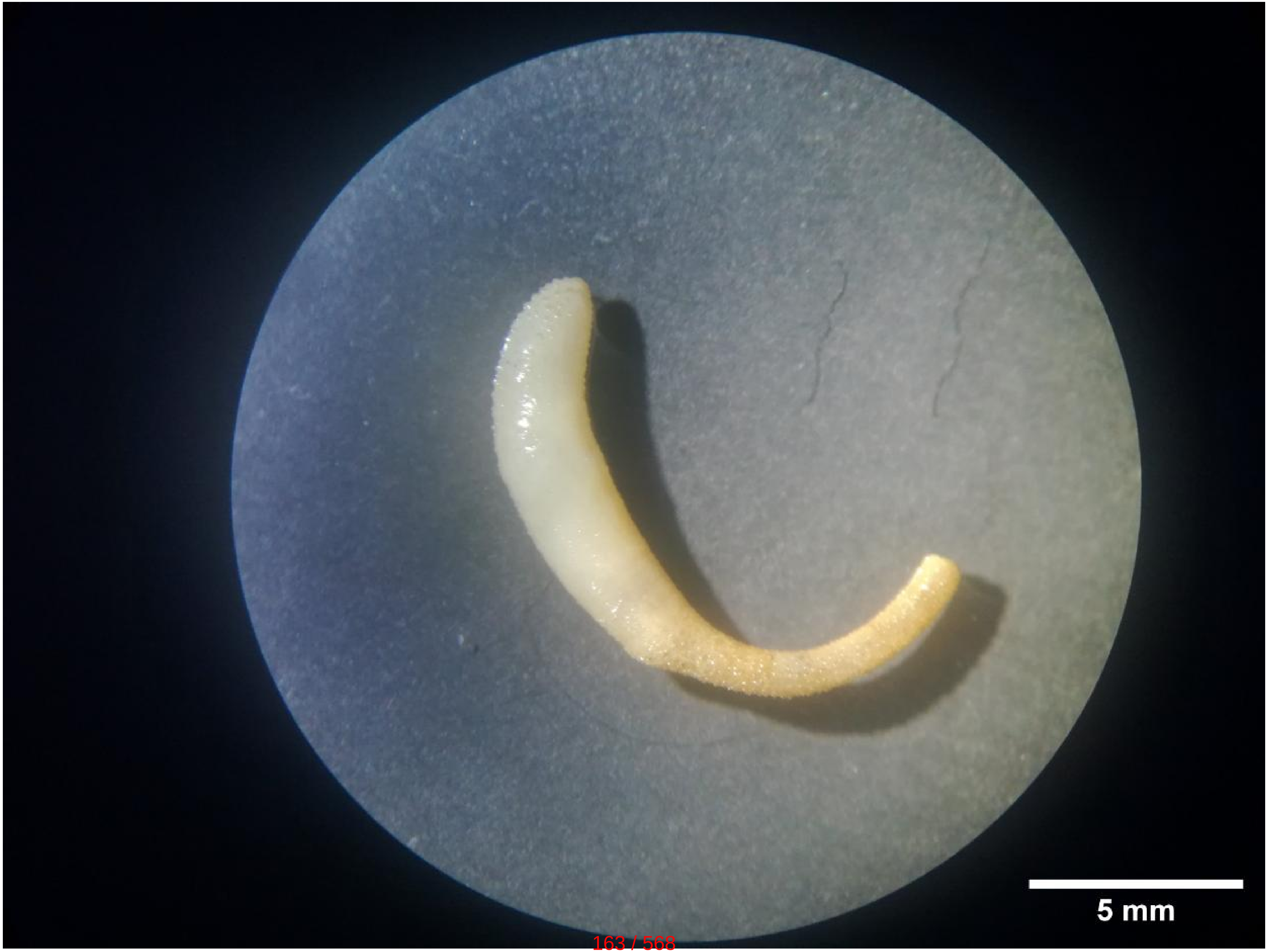
5 mm

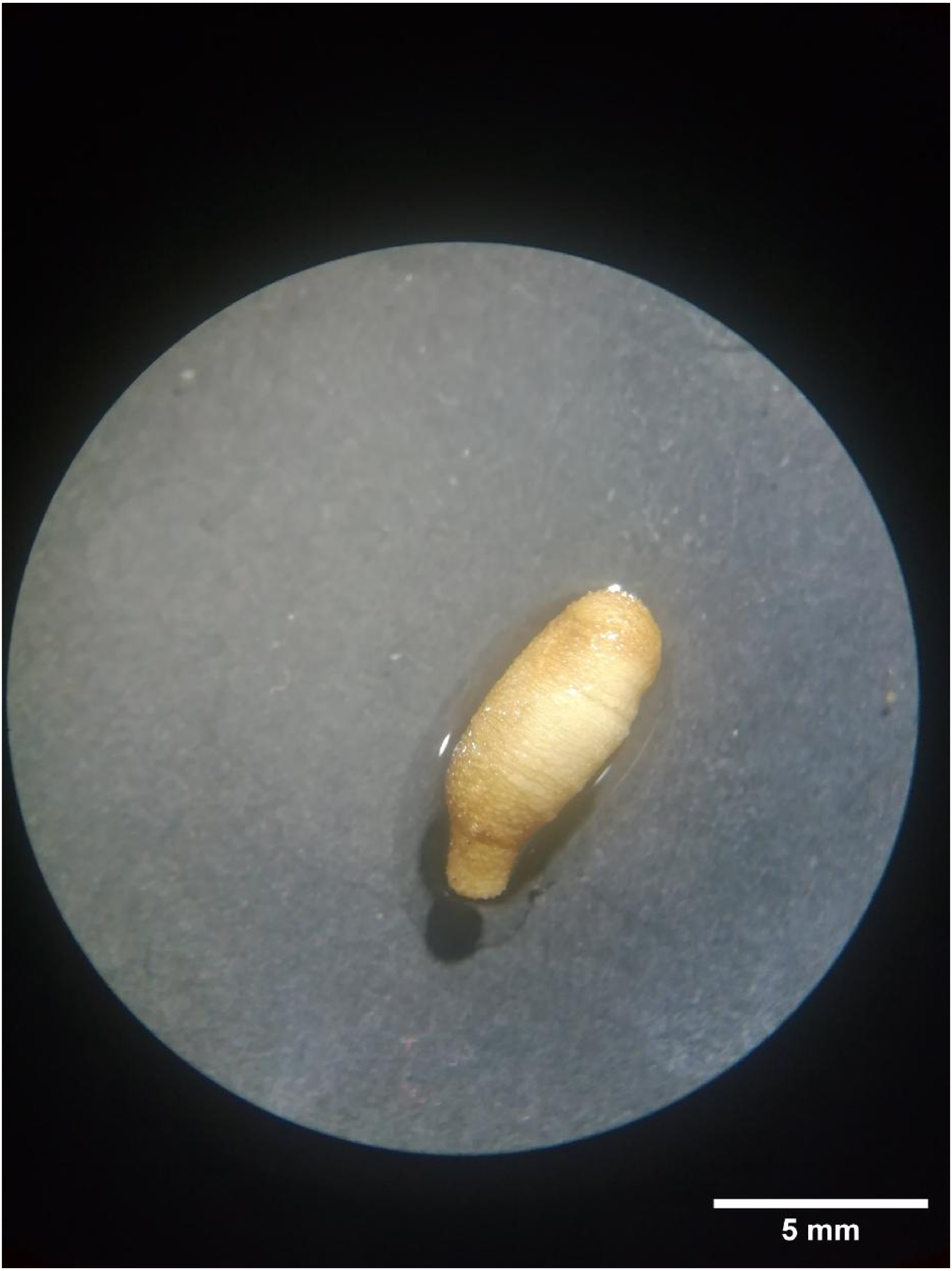
5 mm

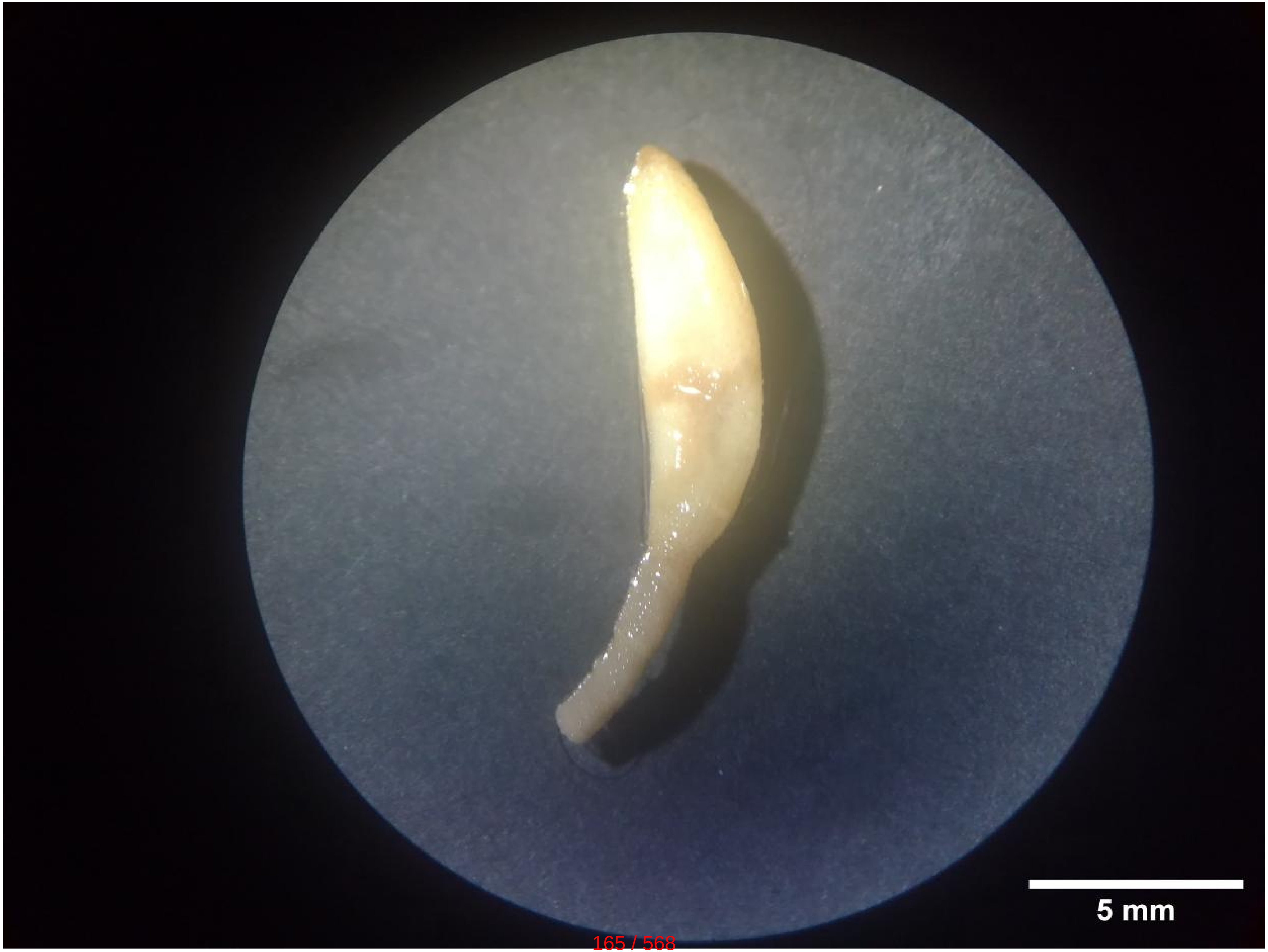
5 mm

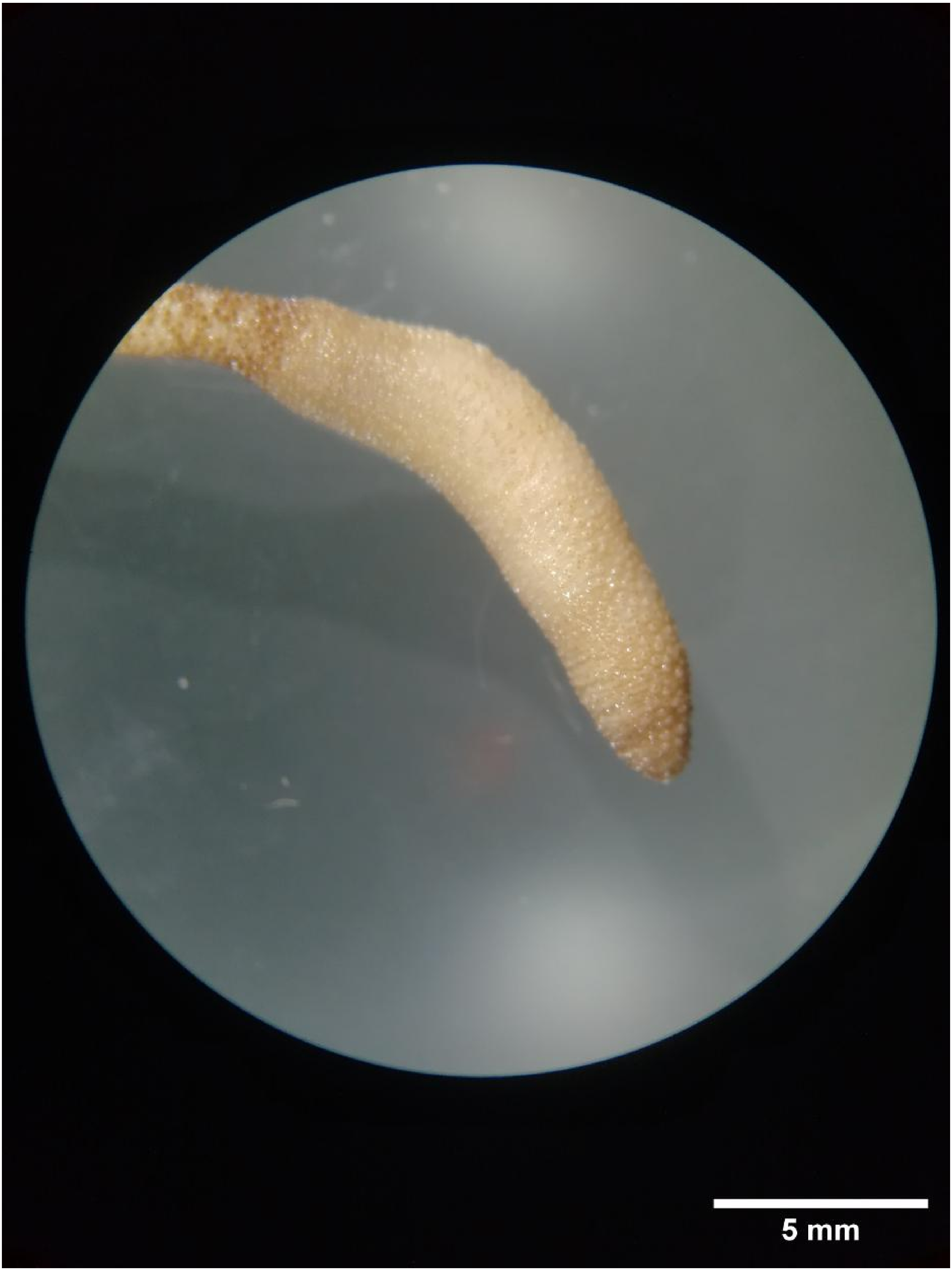
5 mm

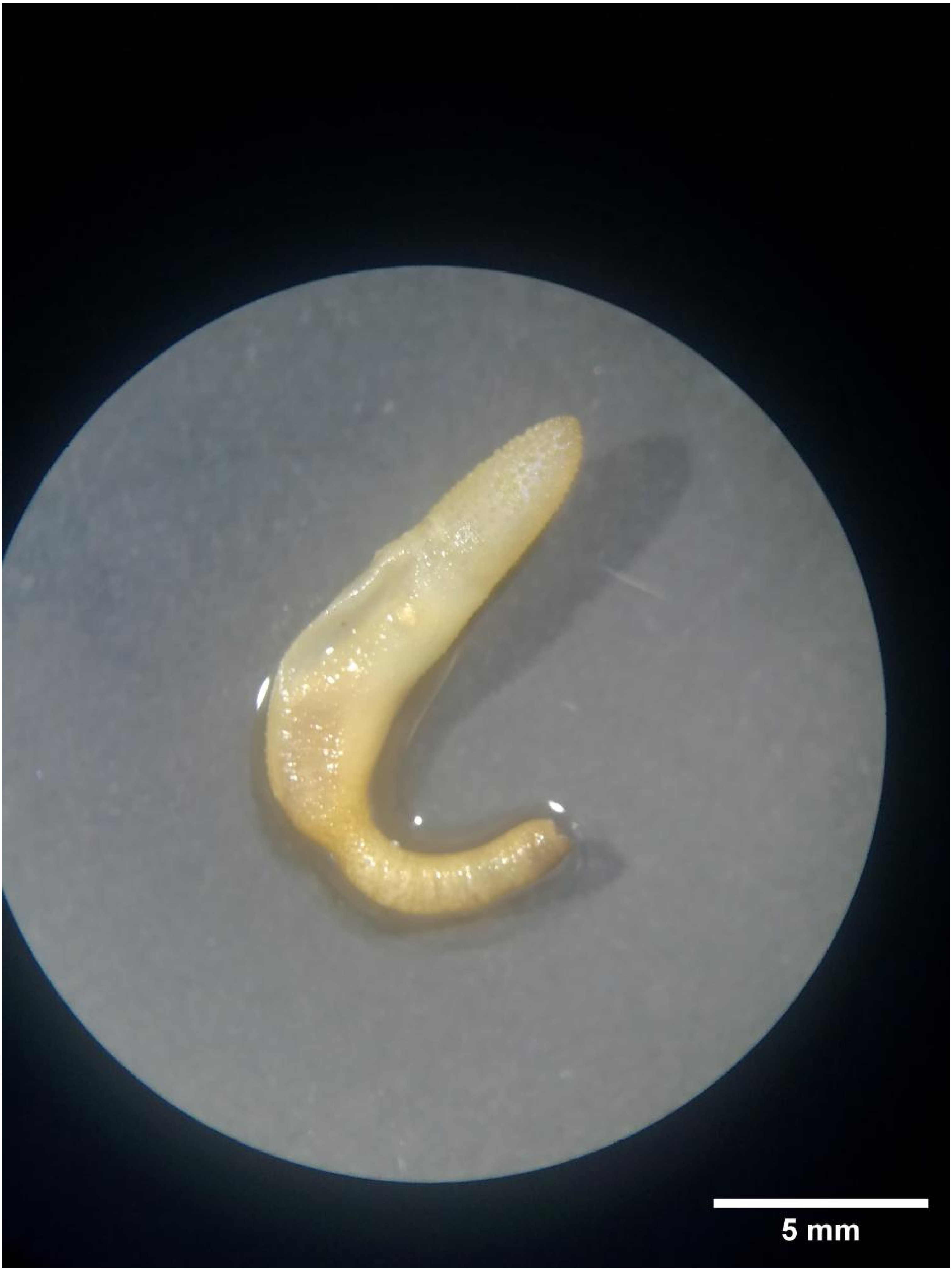
5 mm

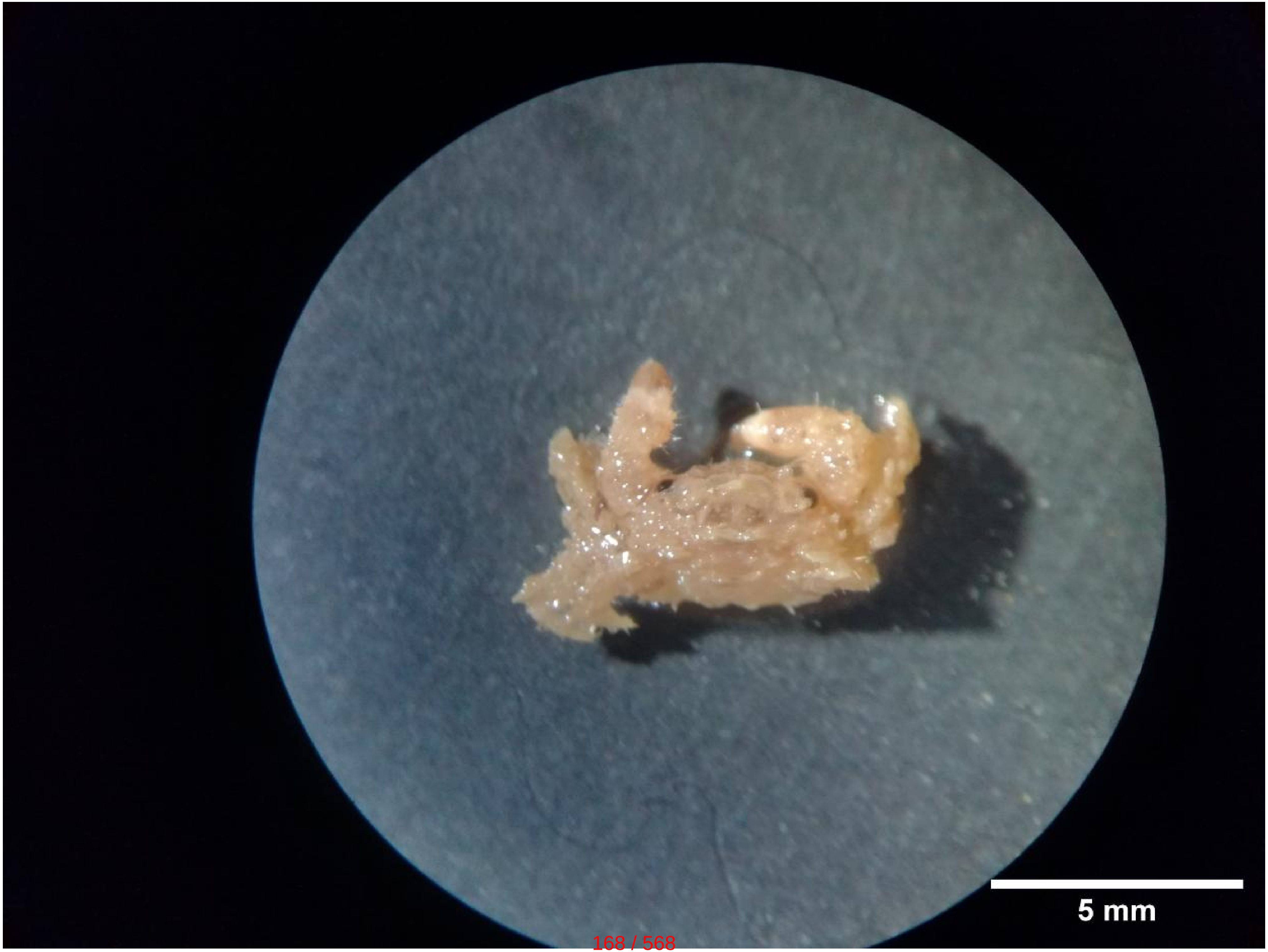
5 mm

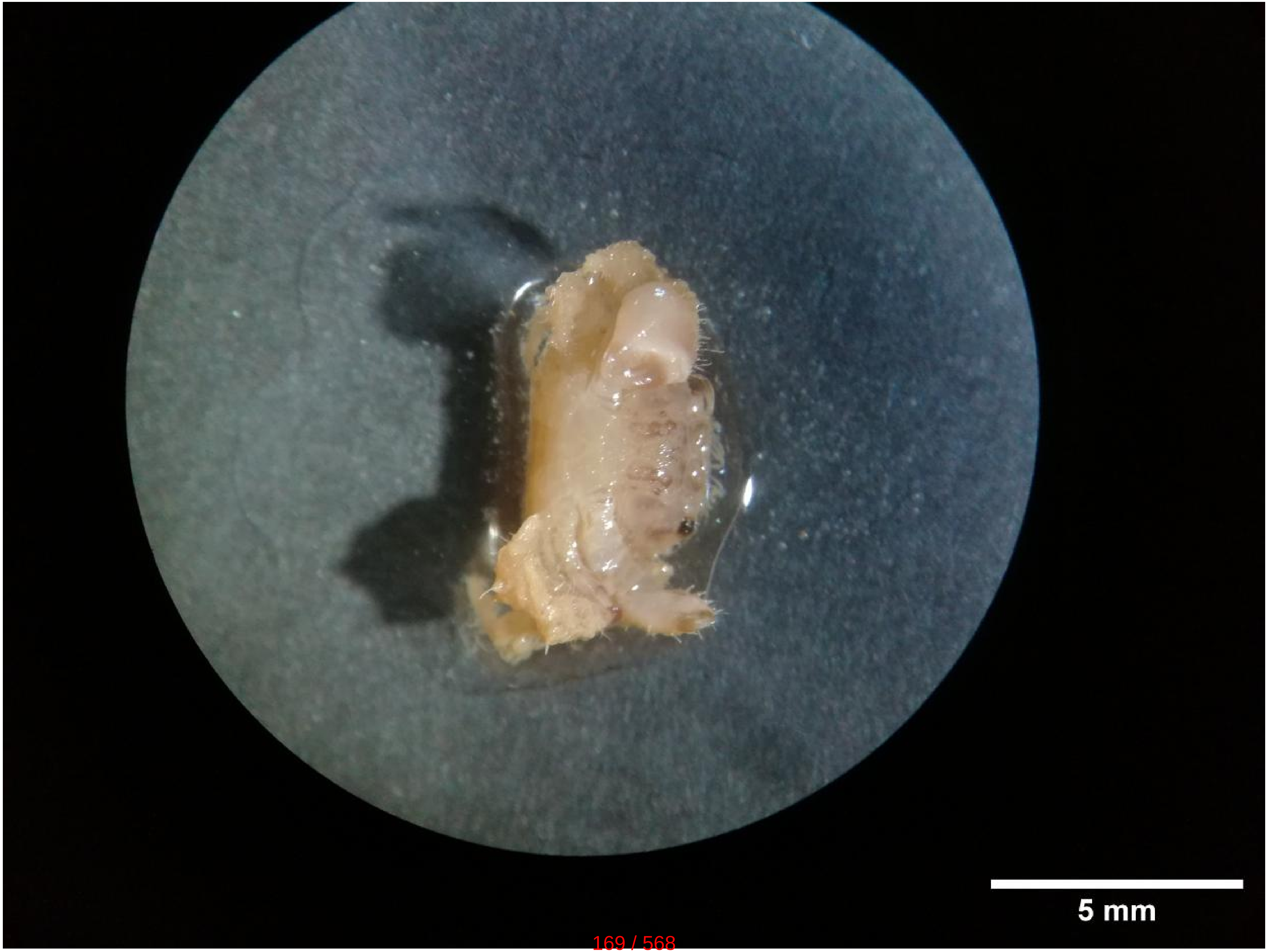
5 mm

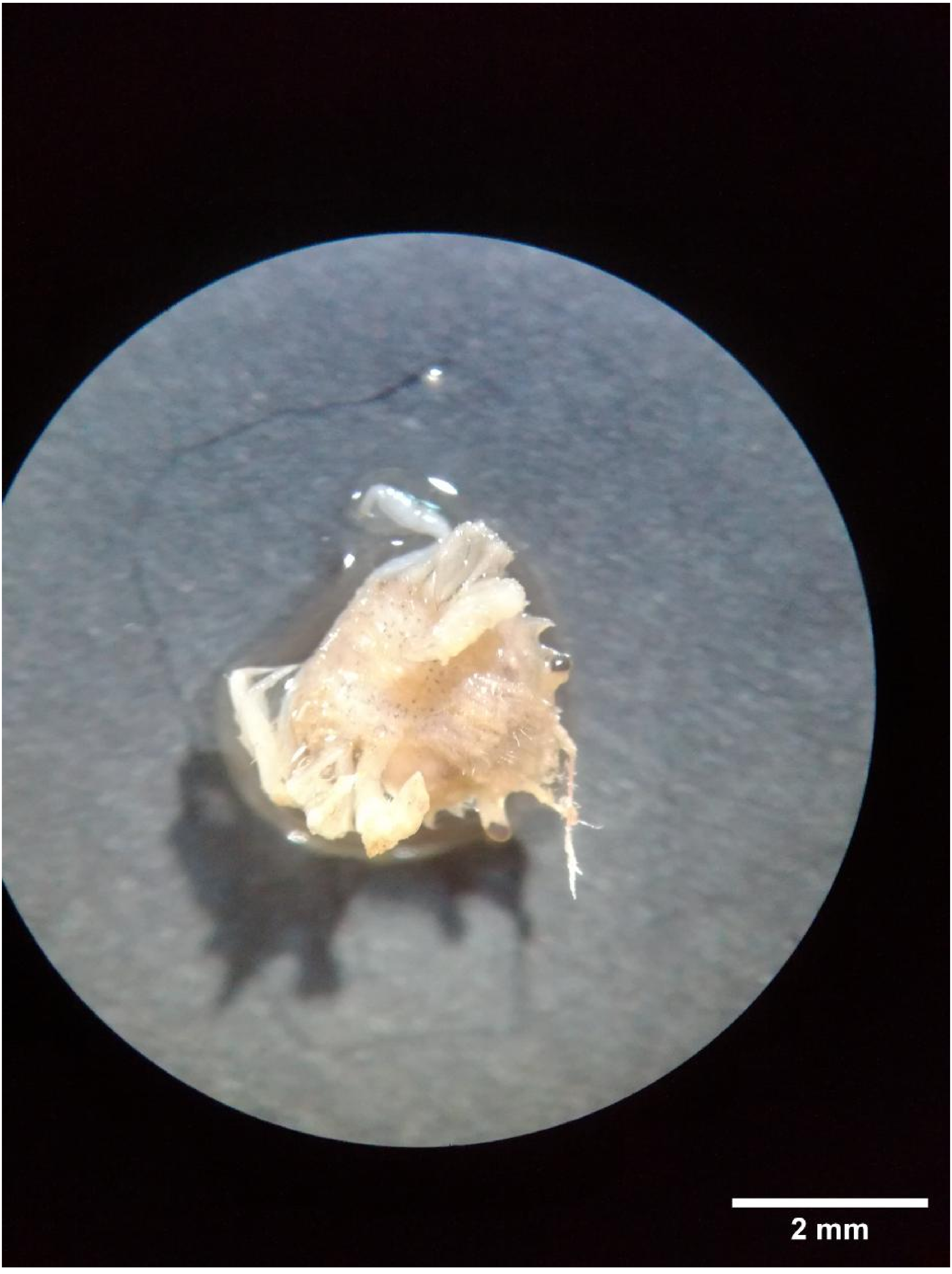
2 mm

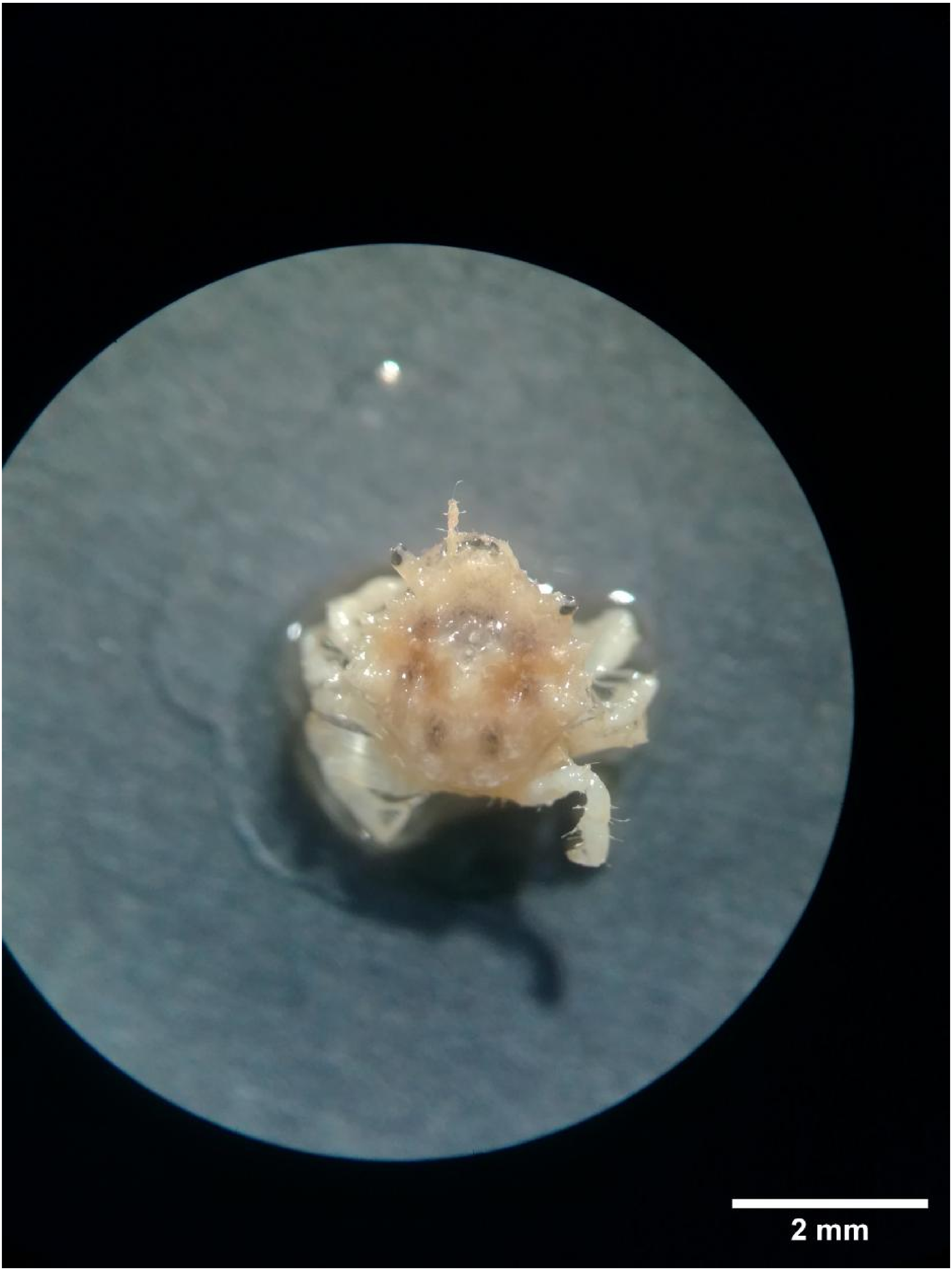
2 mm

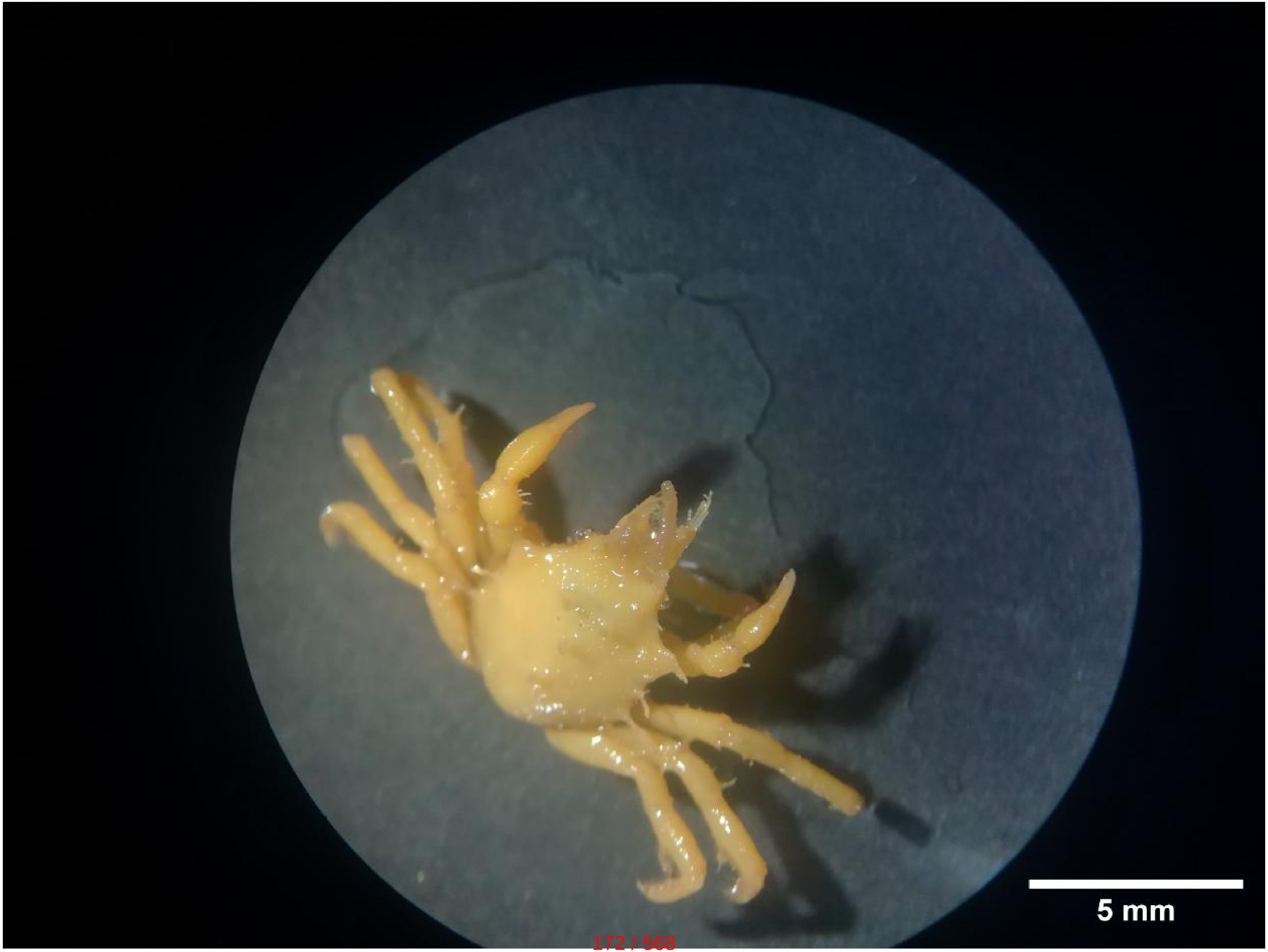
5 mm

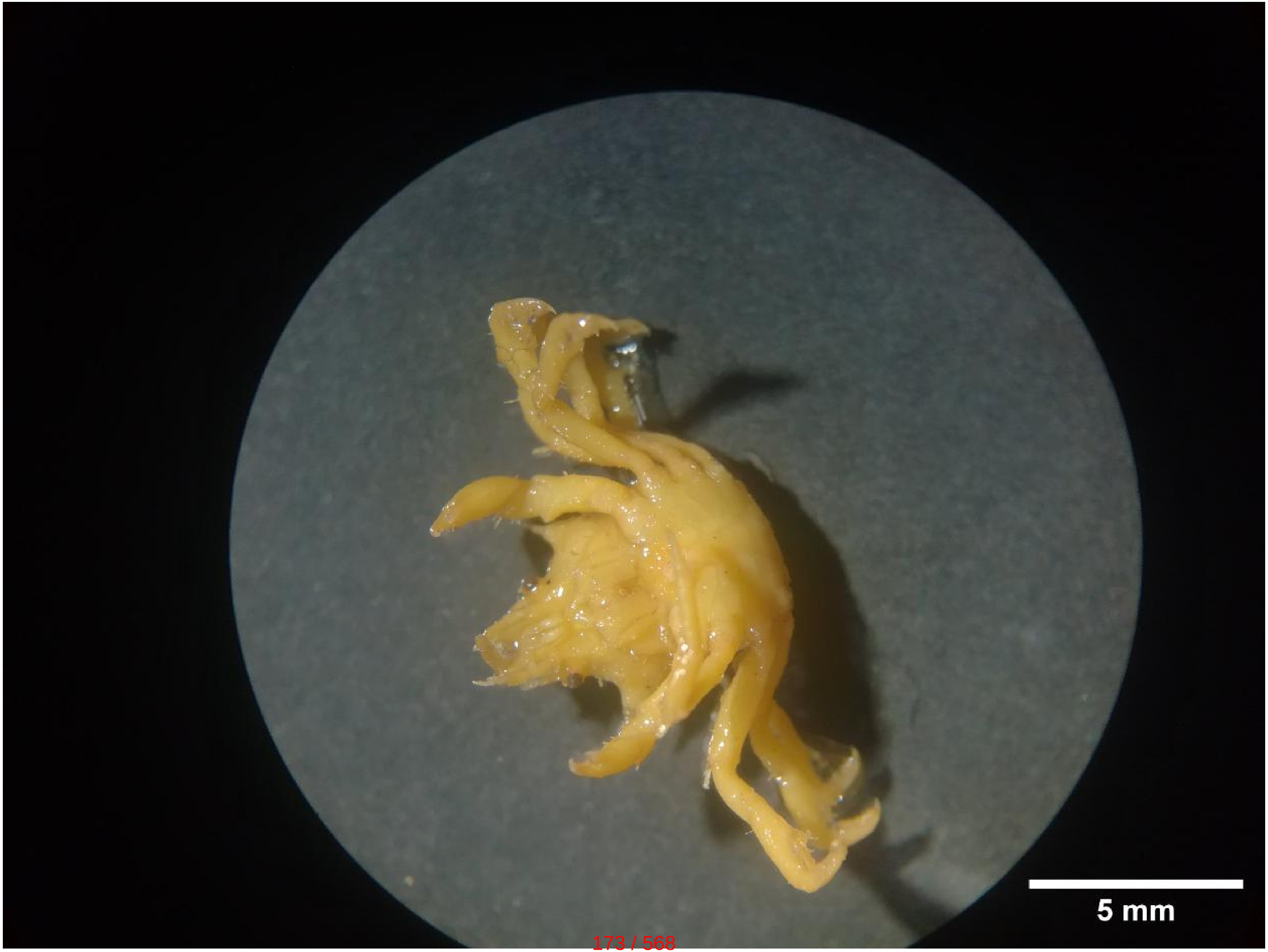
5 mm

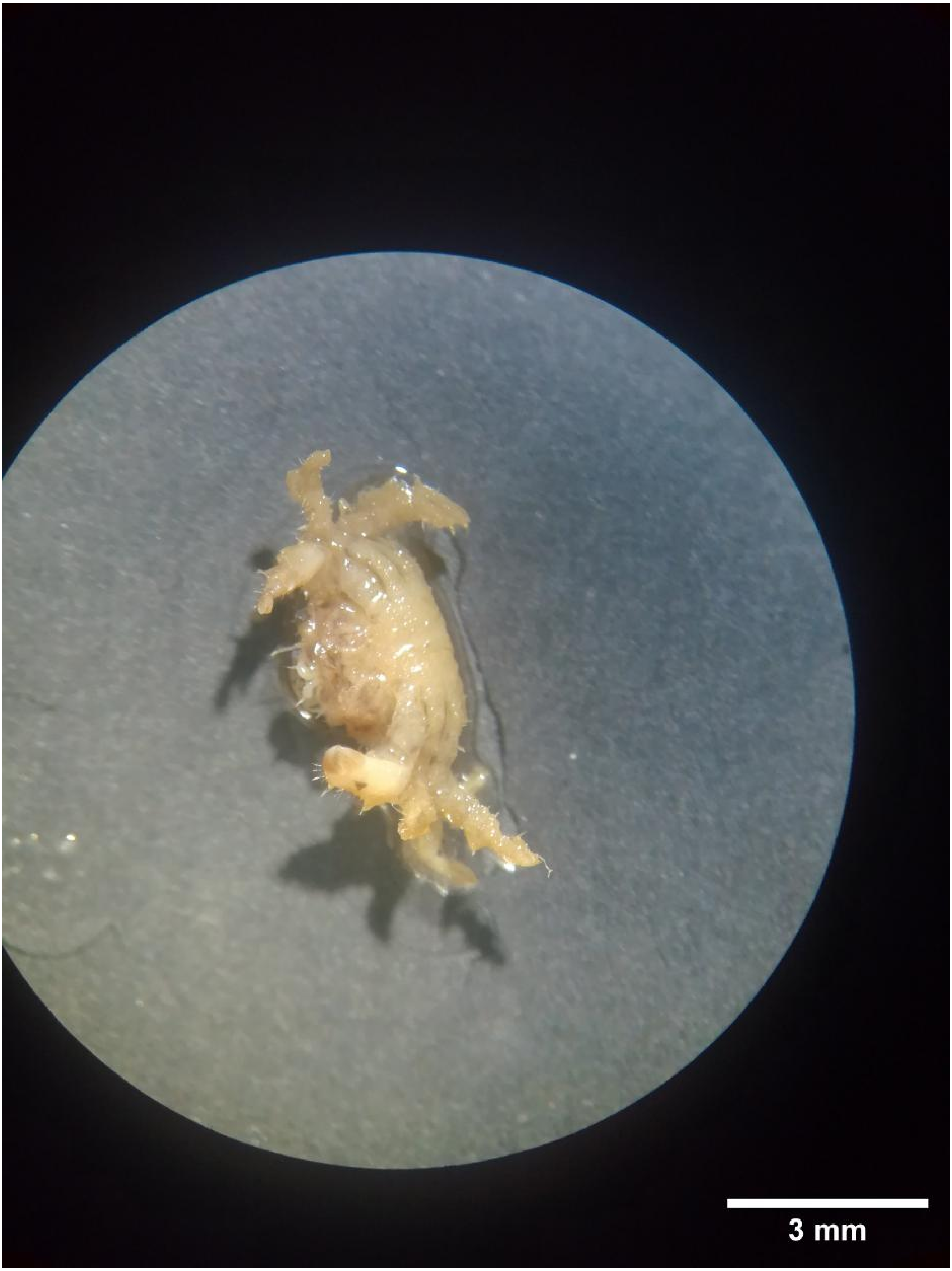
3 mm

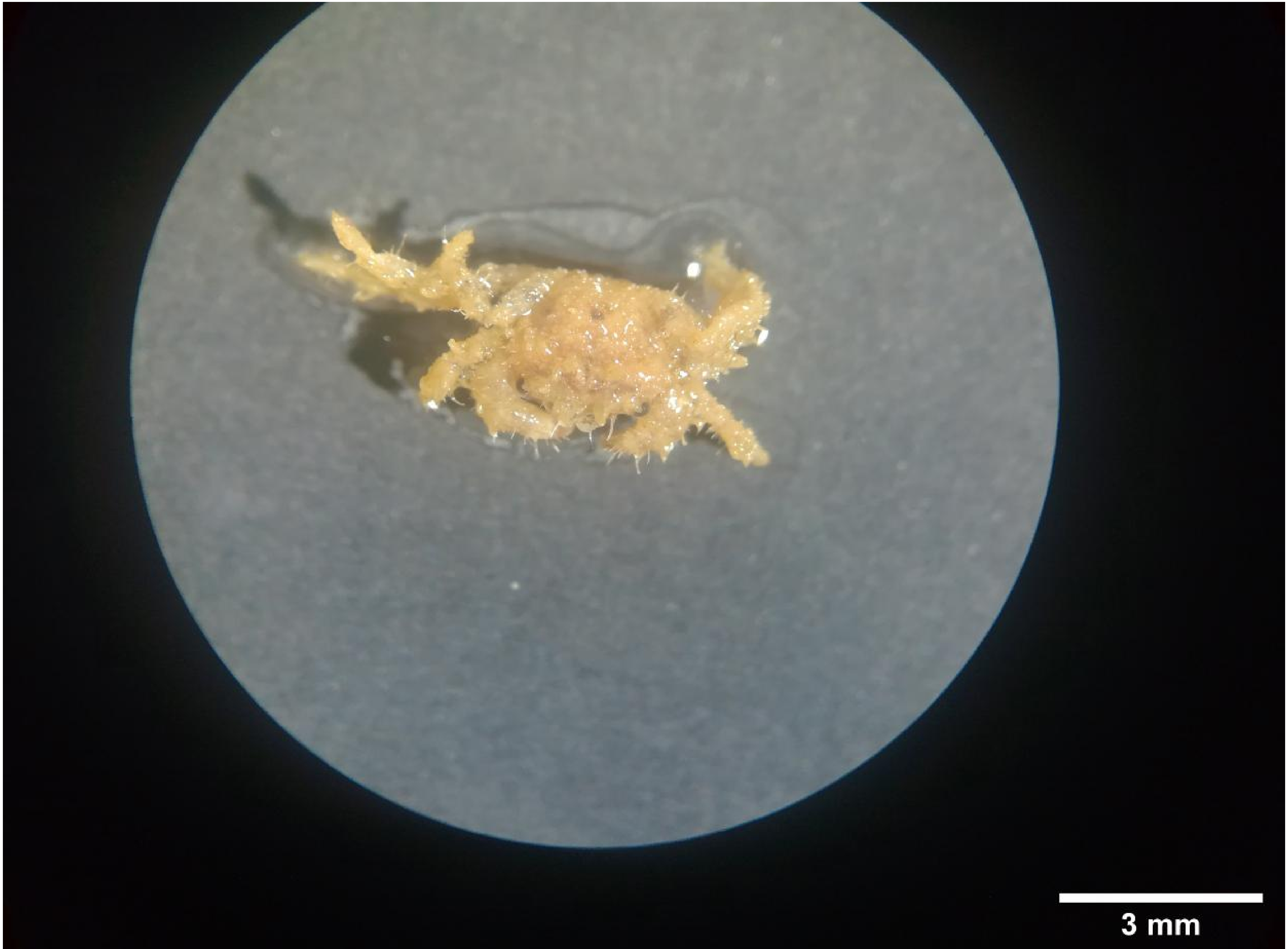
3 mm

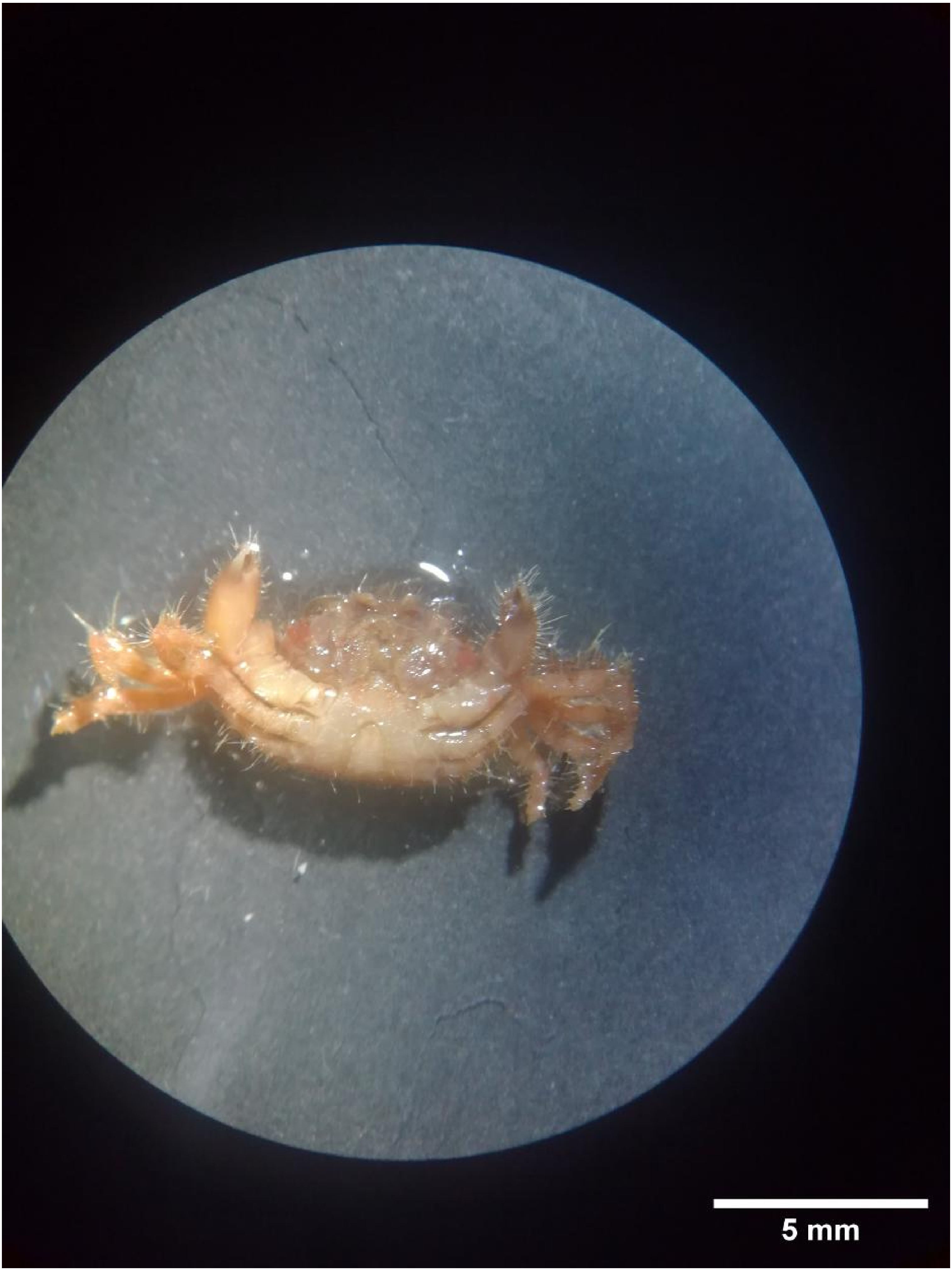
5 mm

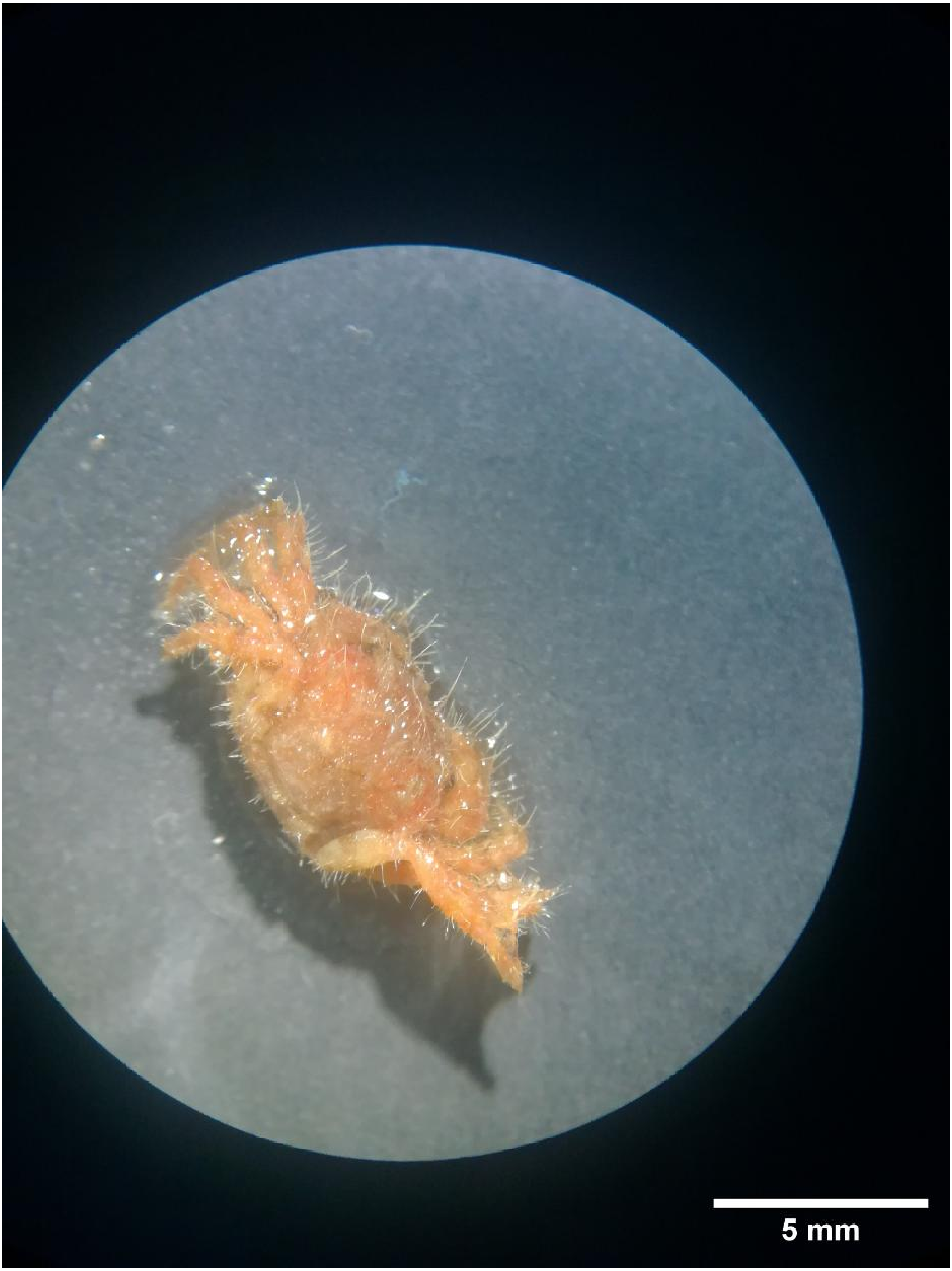
5 mm

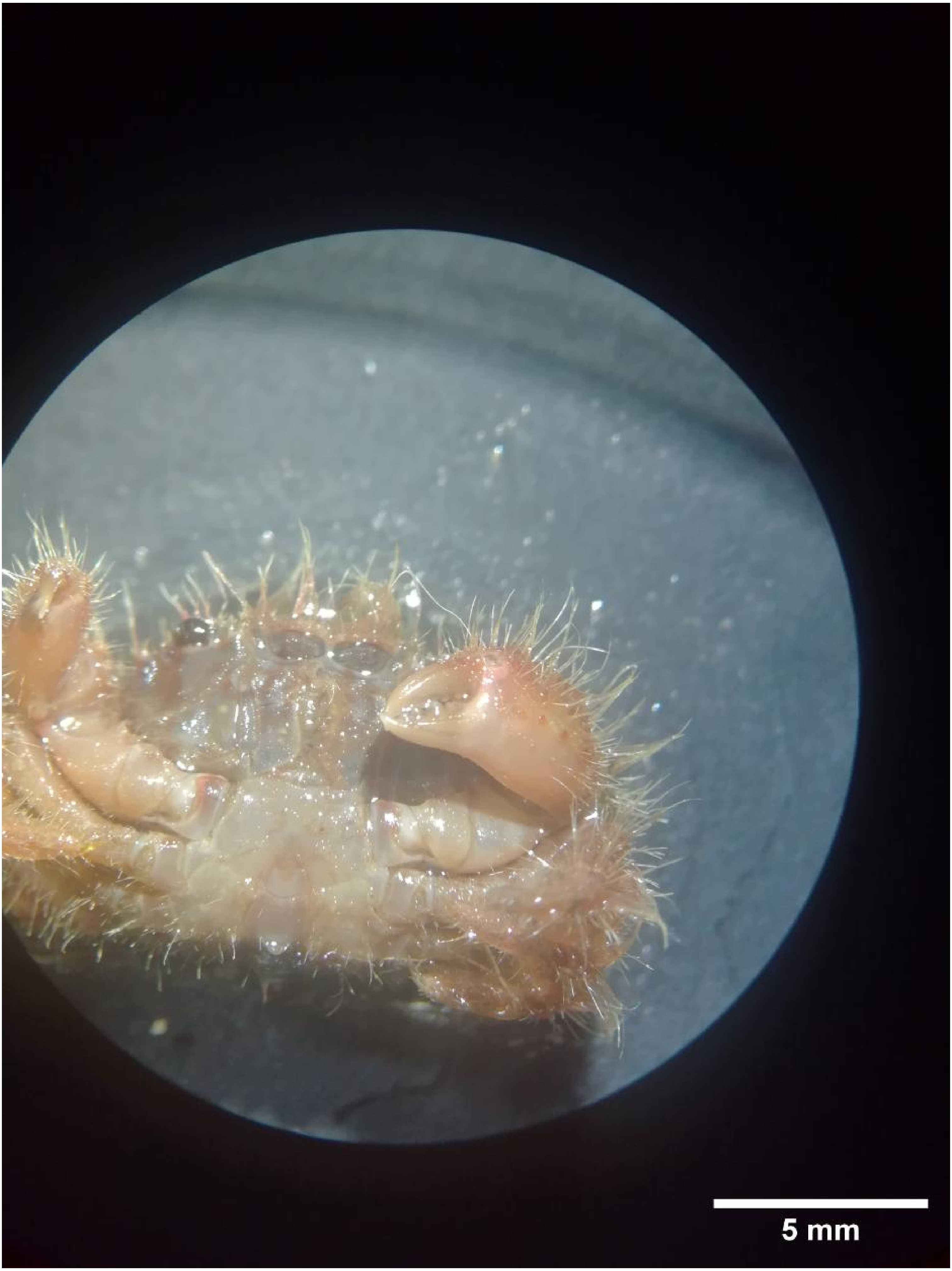
5 mm

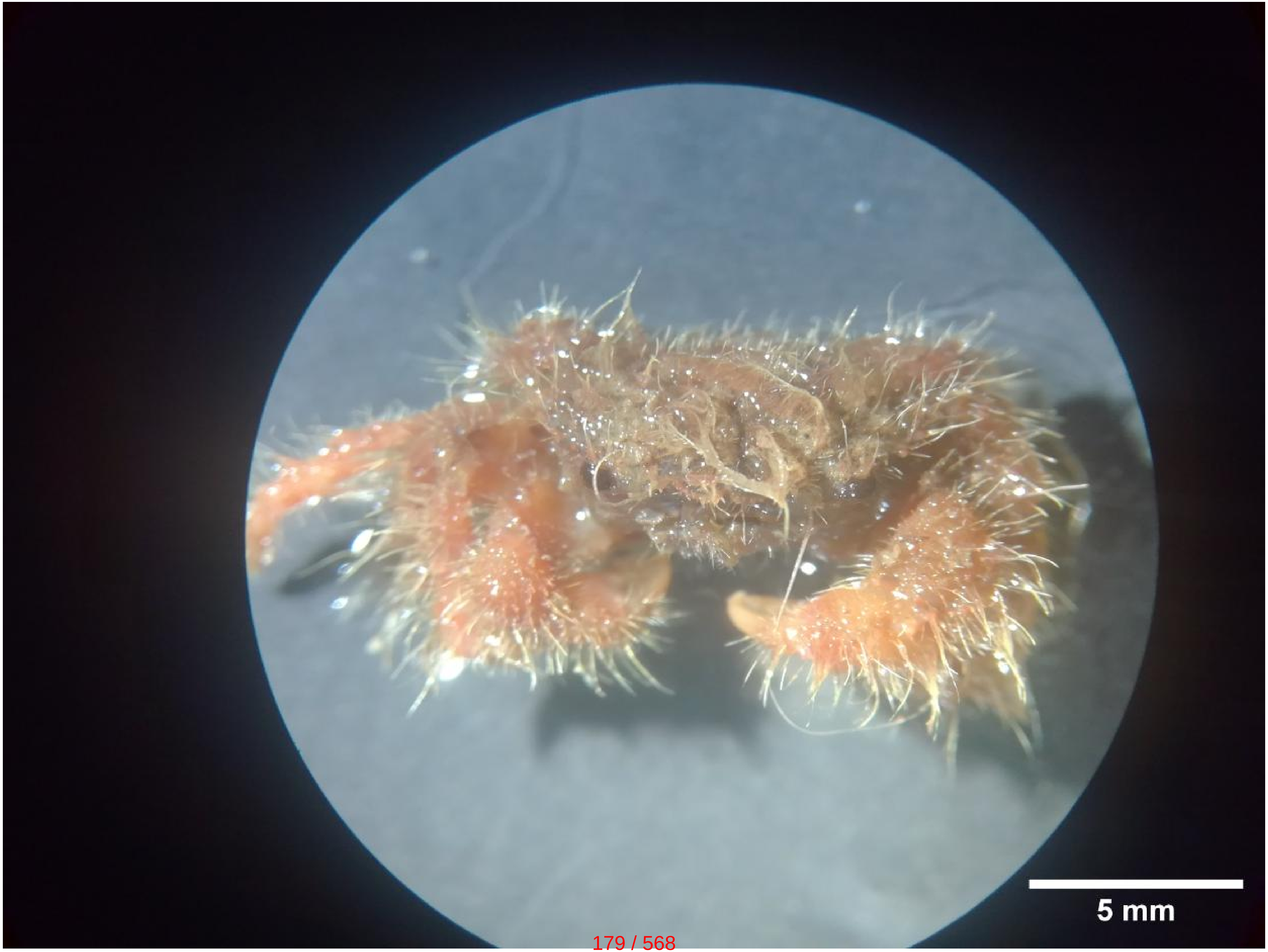
5 mm

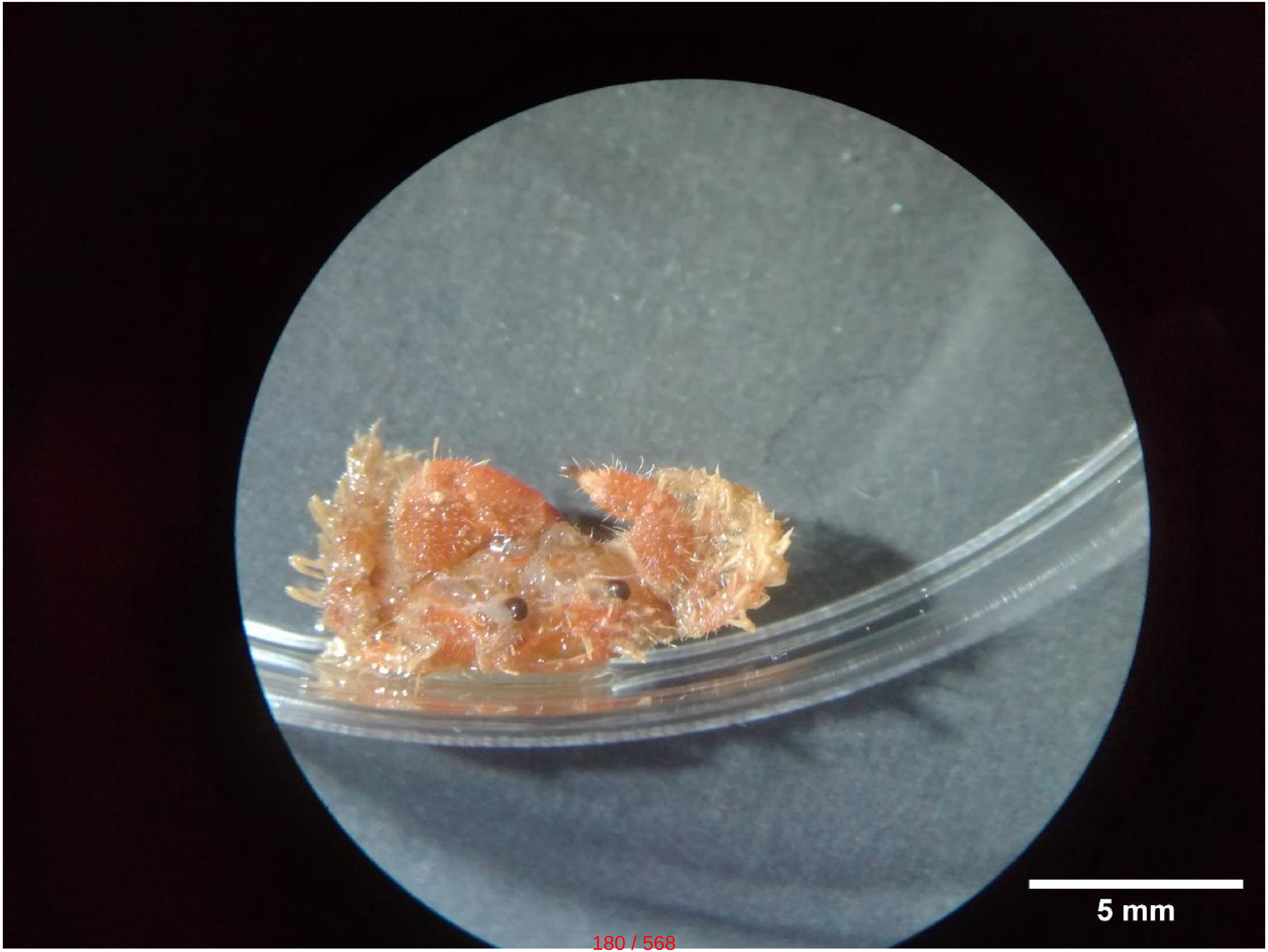
5 mm

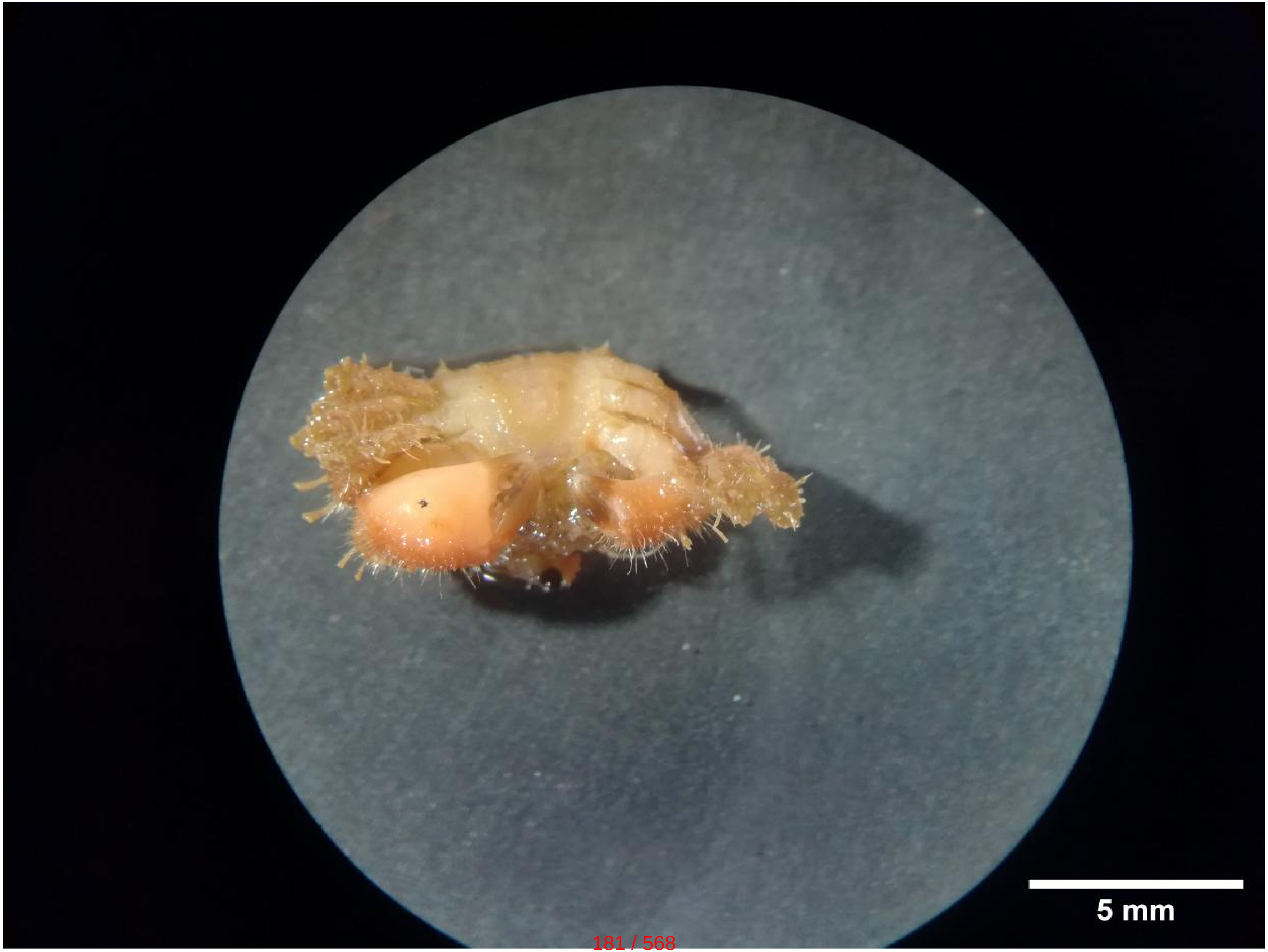
5 mm

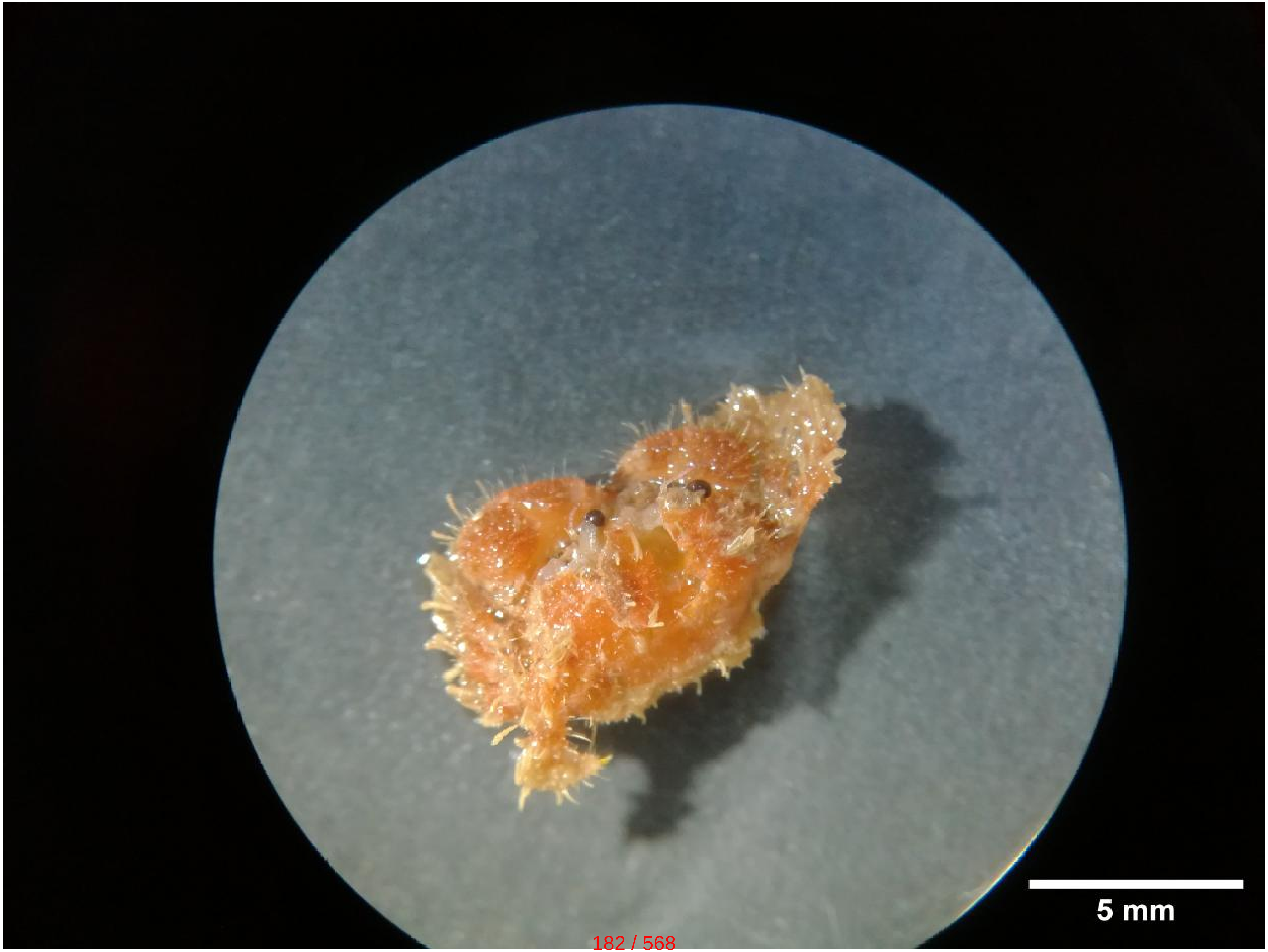
5 mm

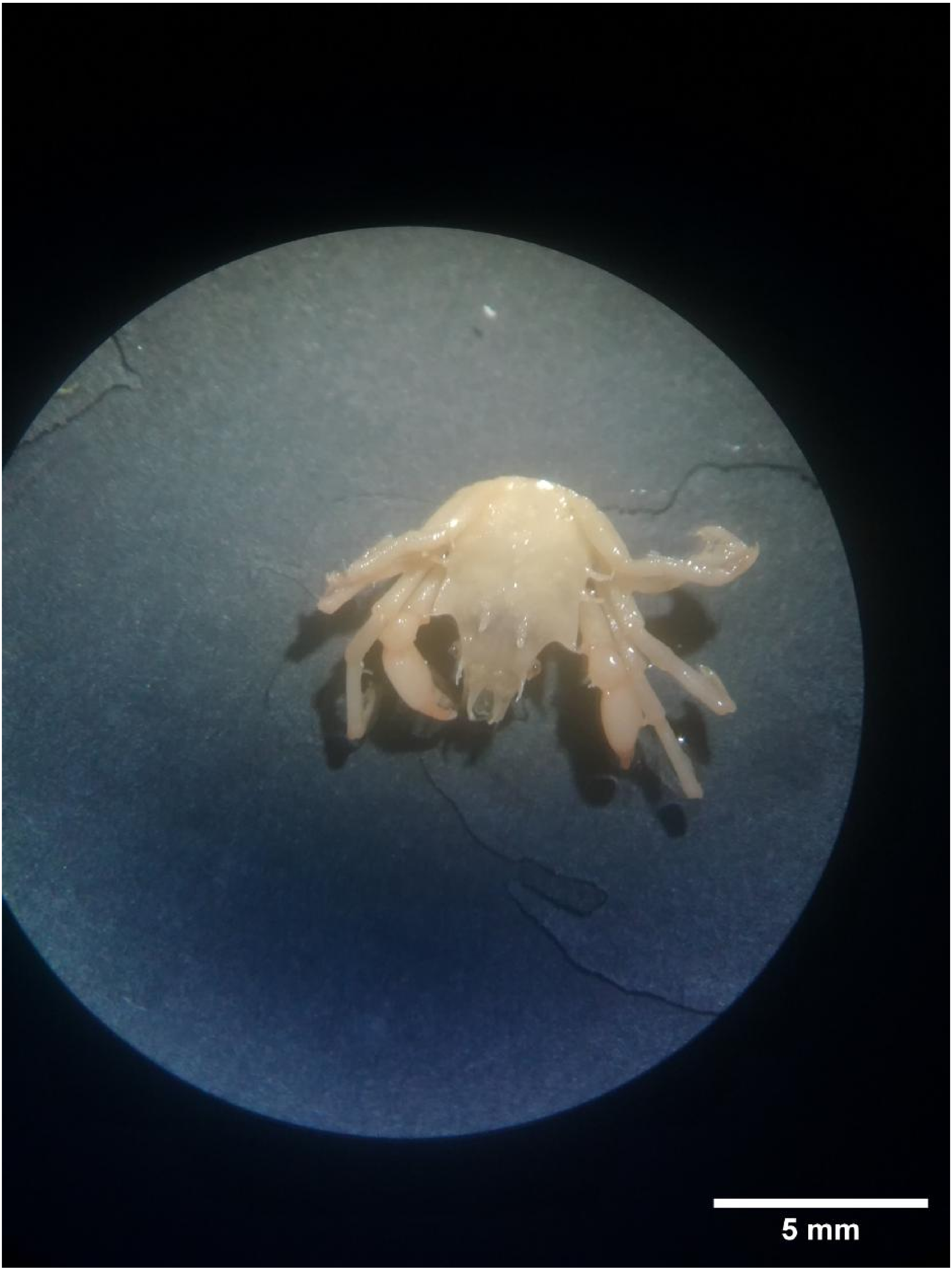
5 mm

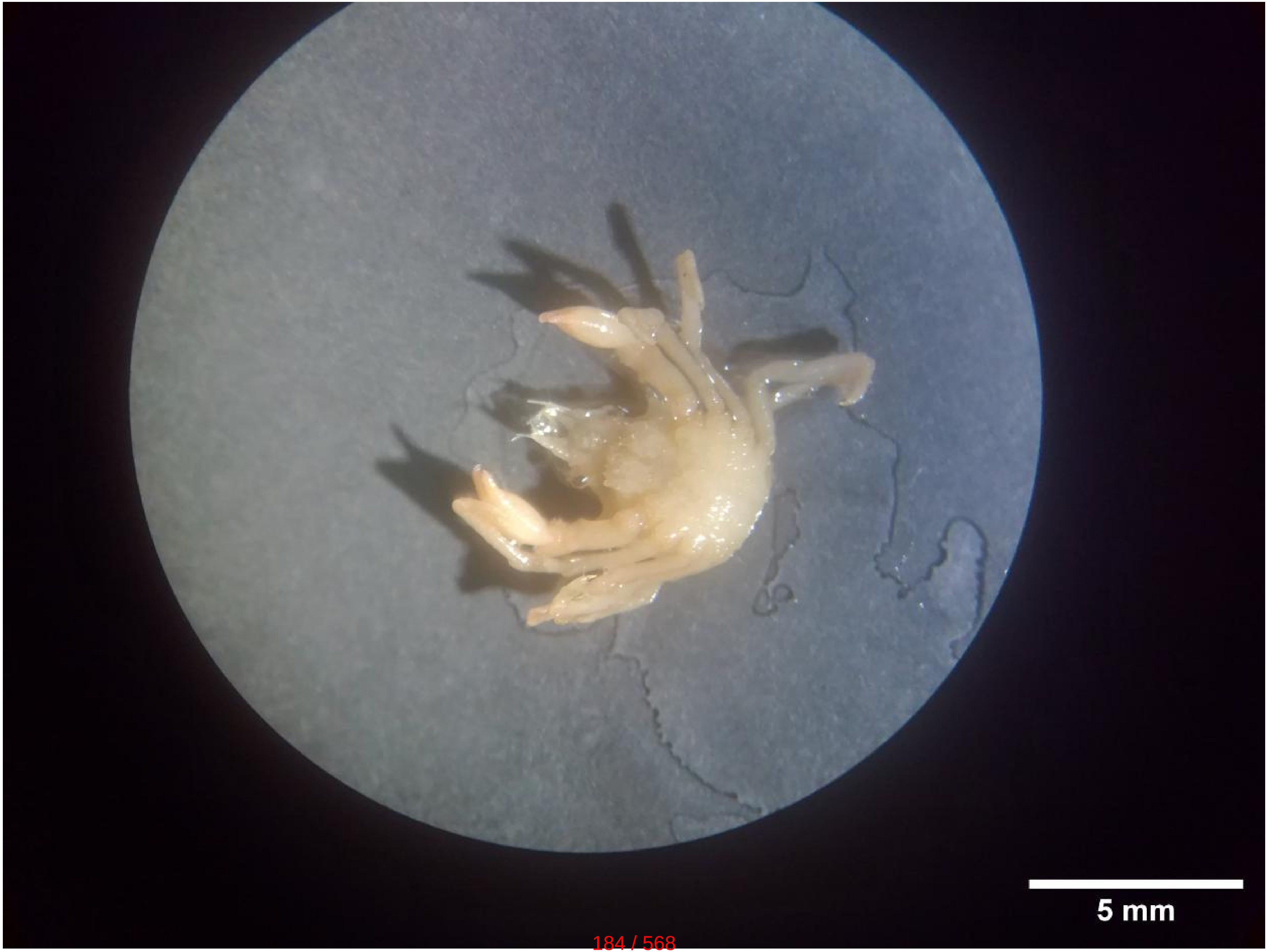
5 mm

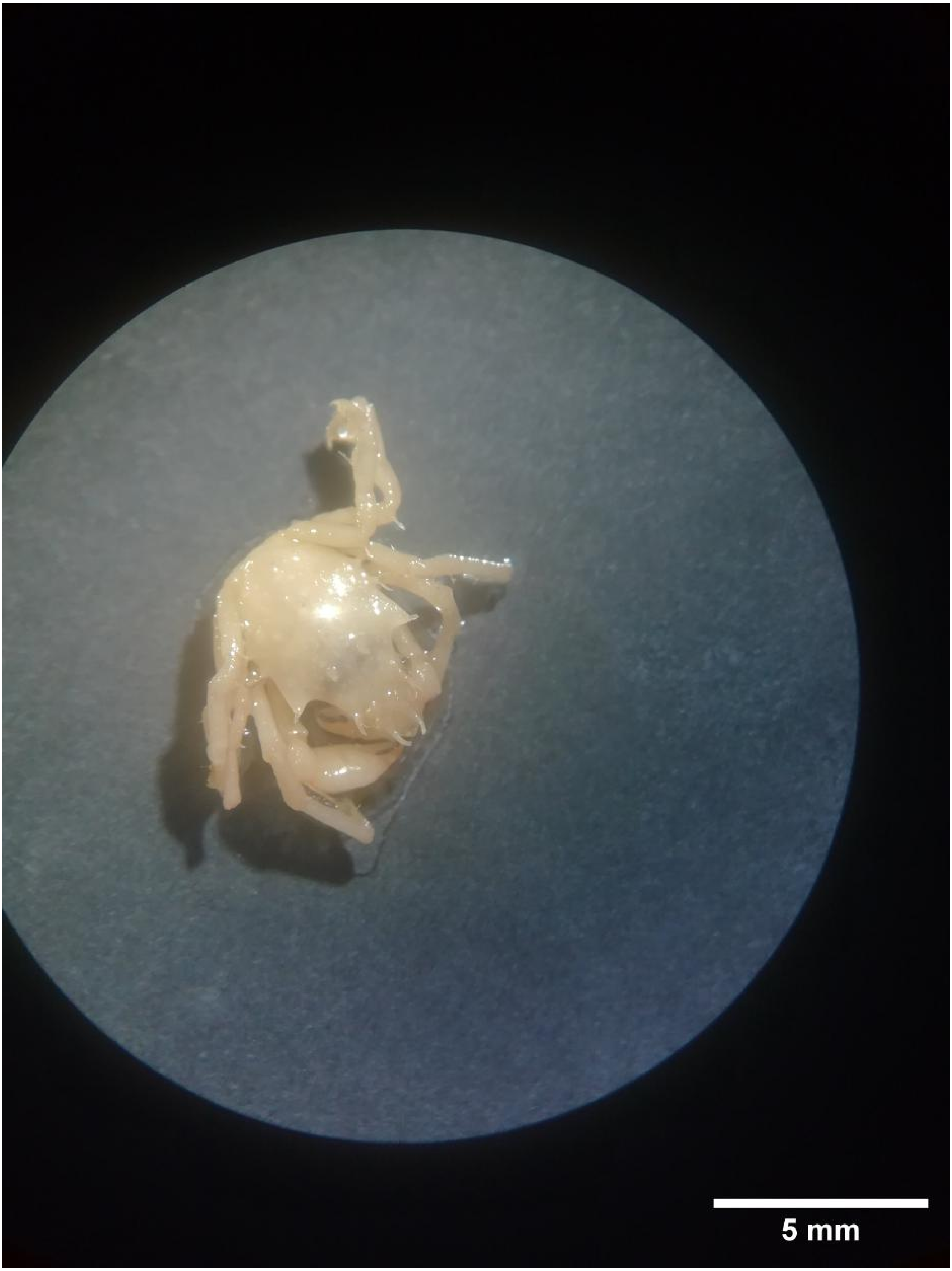
5 mm

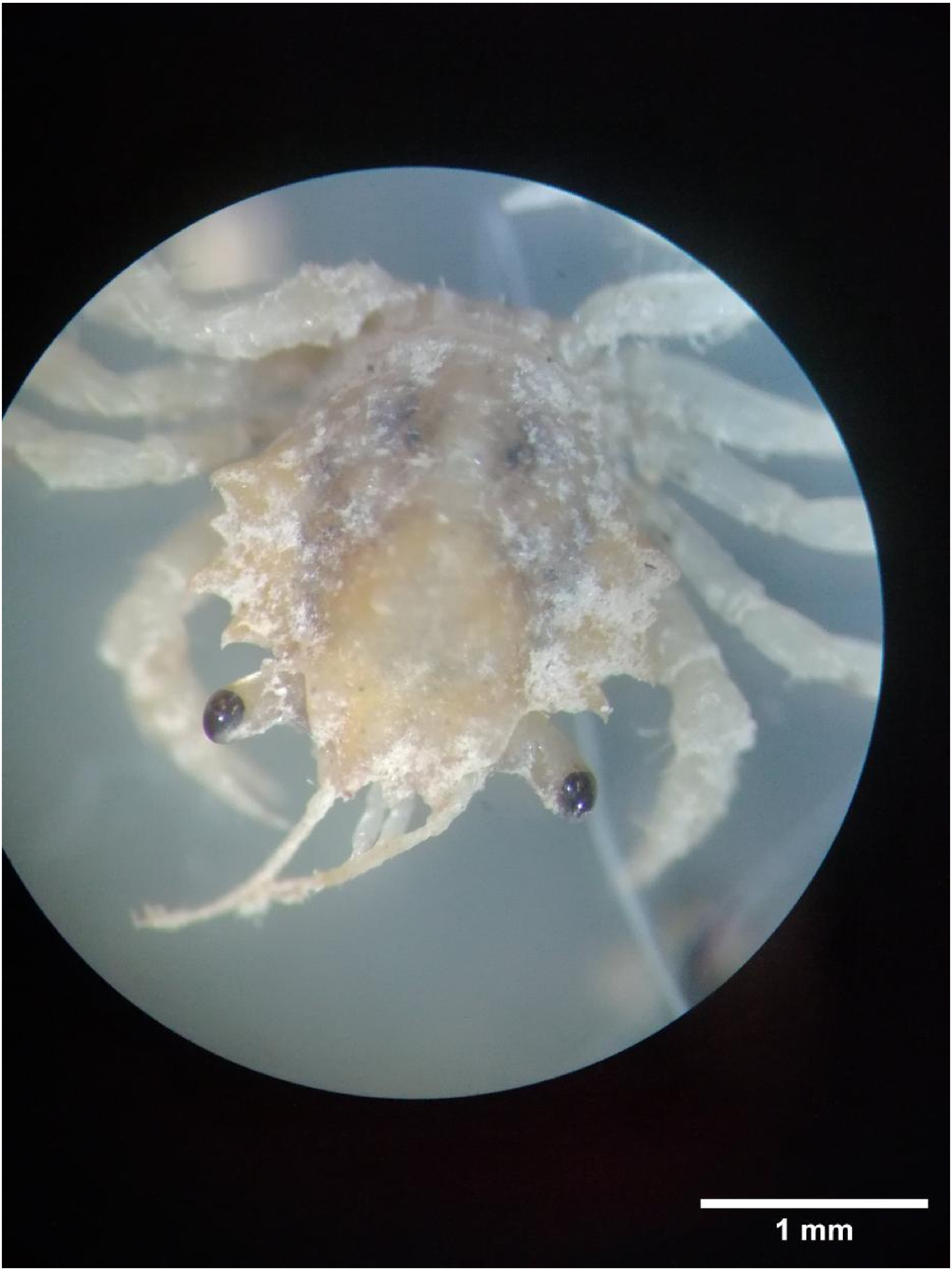
1 mm

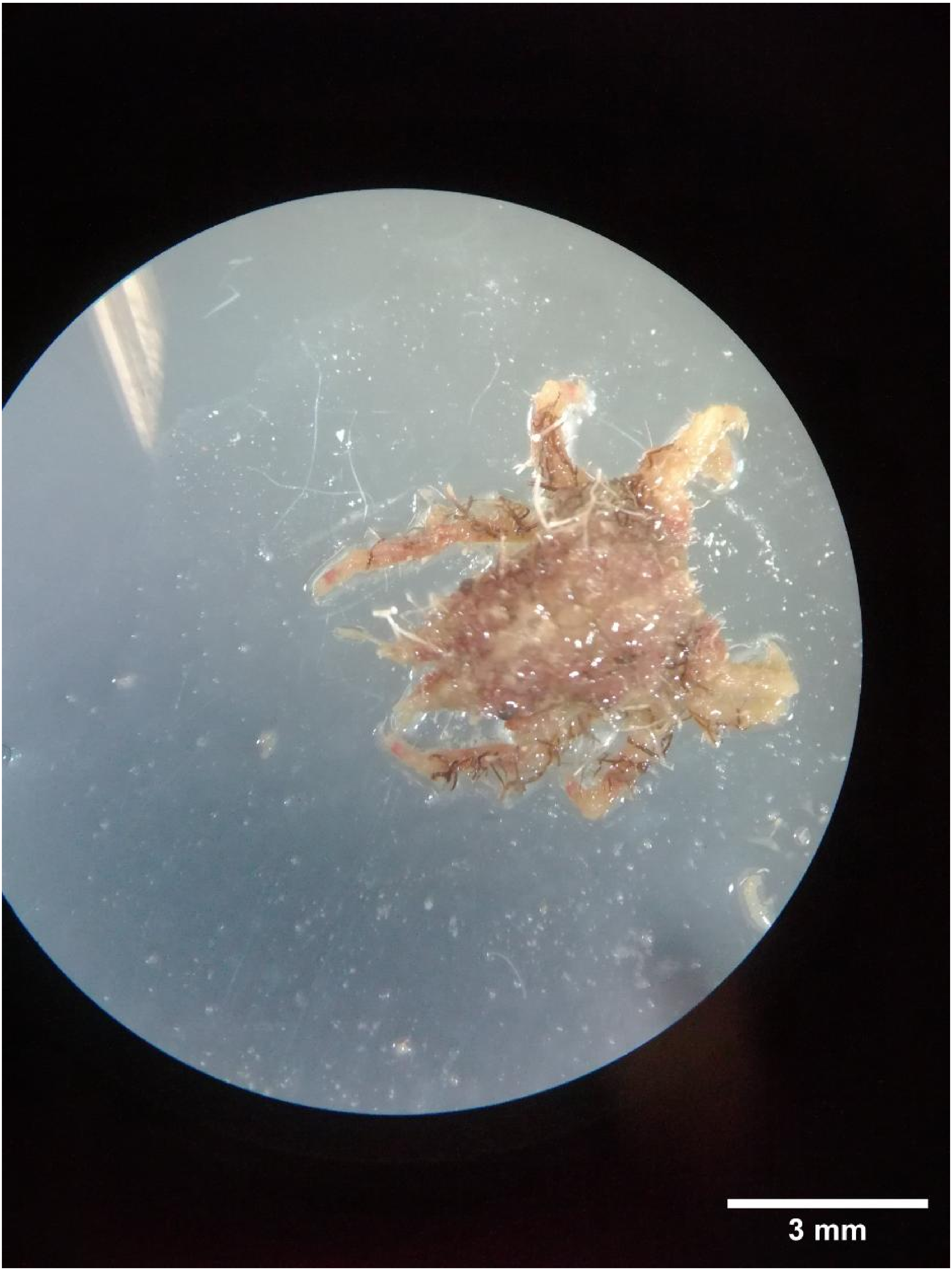
3 mm

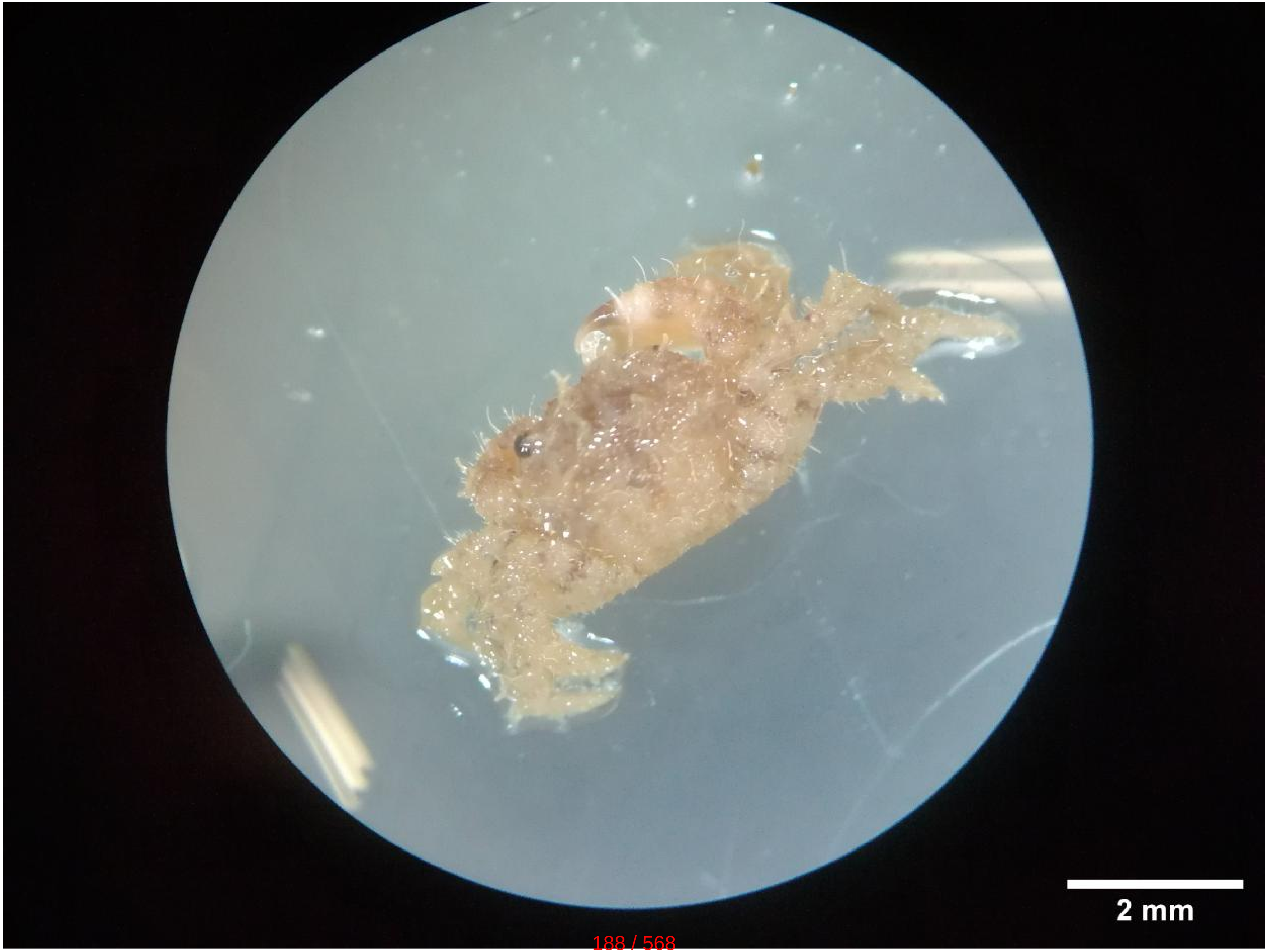
2 mm

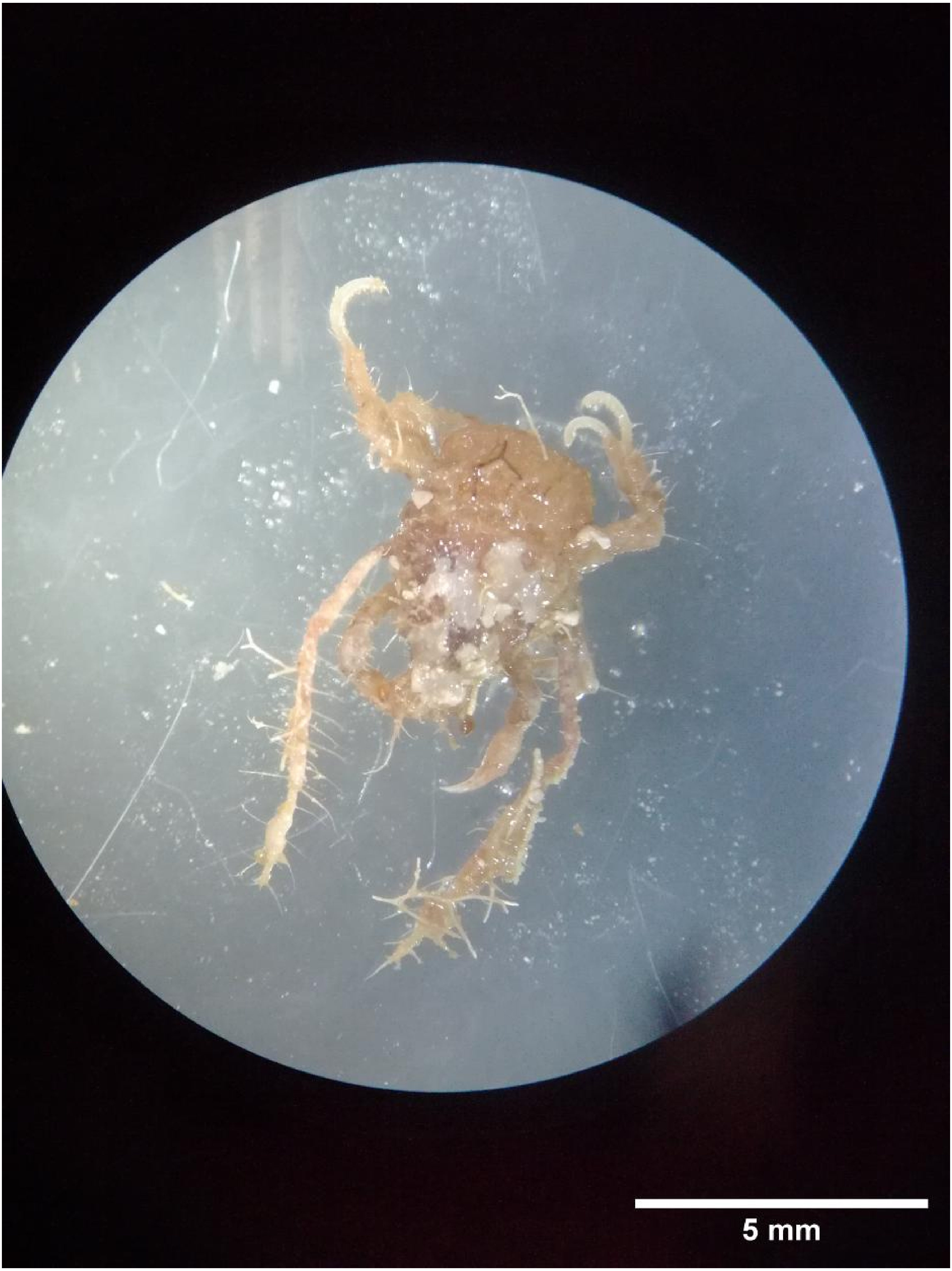
5 mm

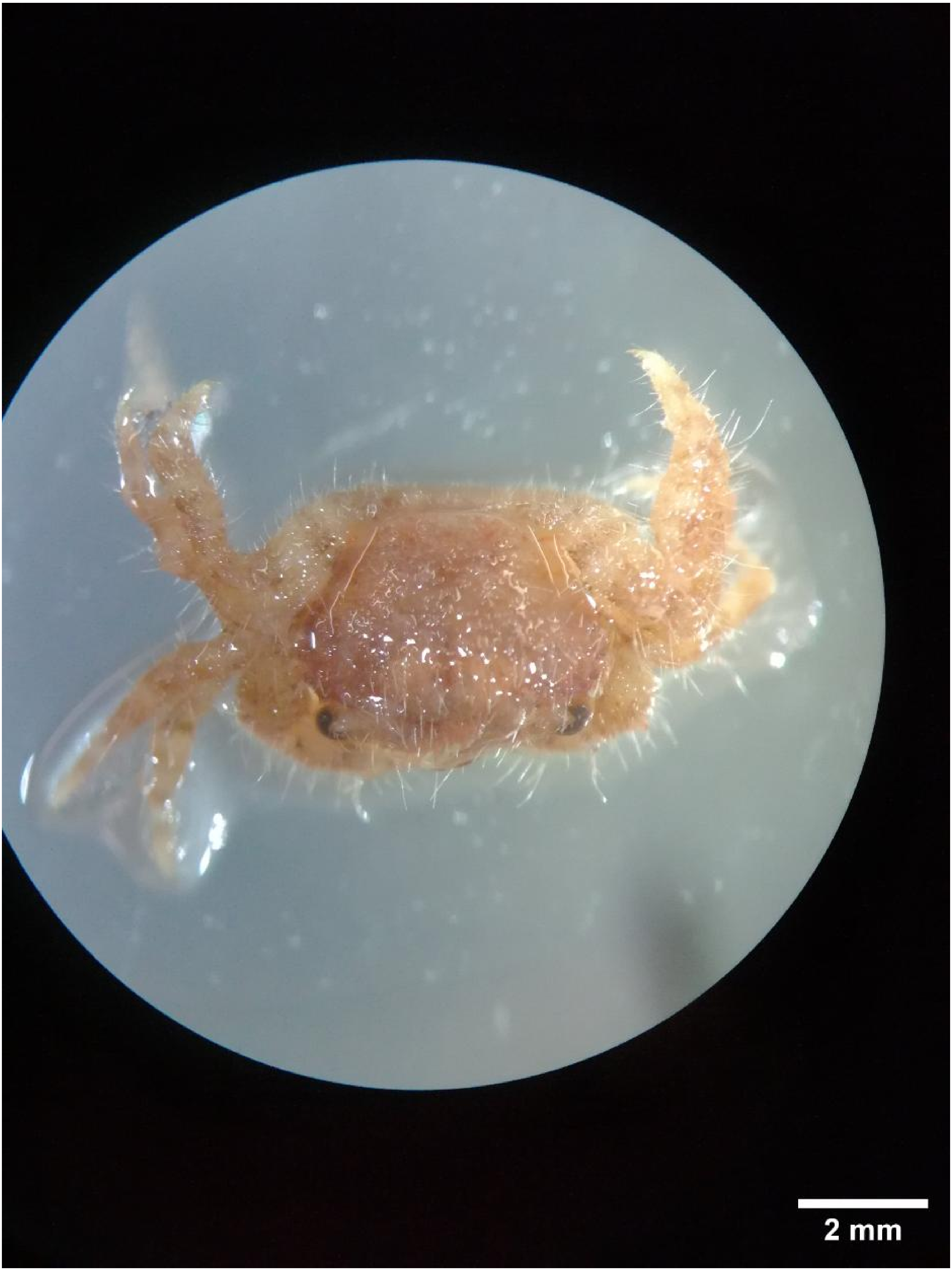
2 mm

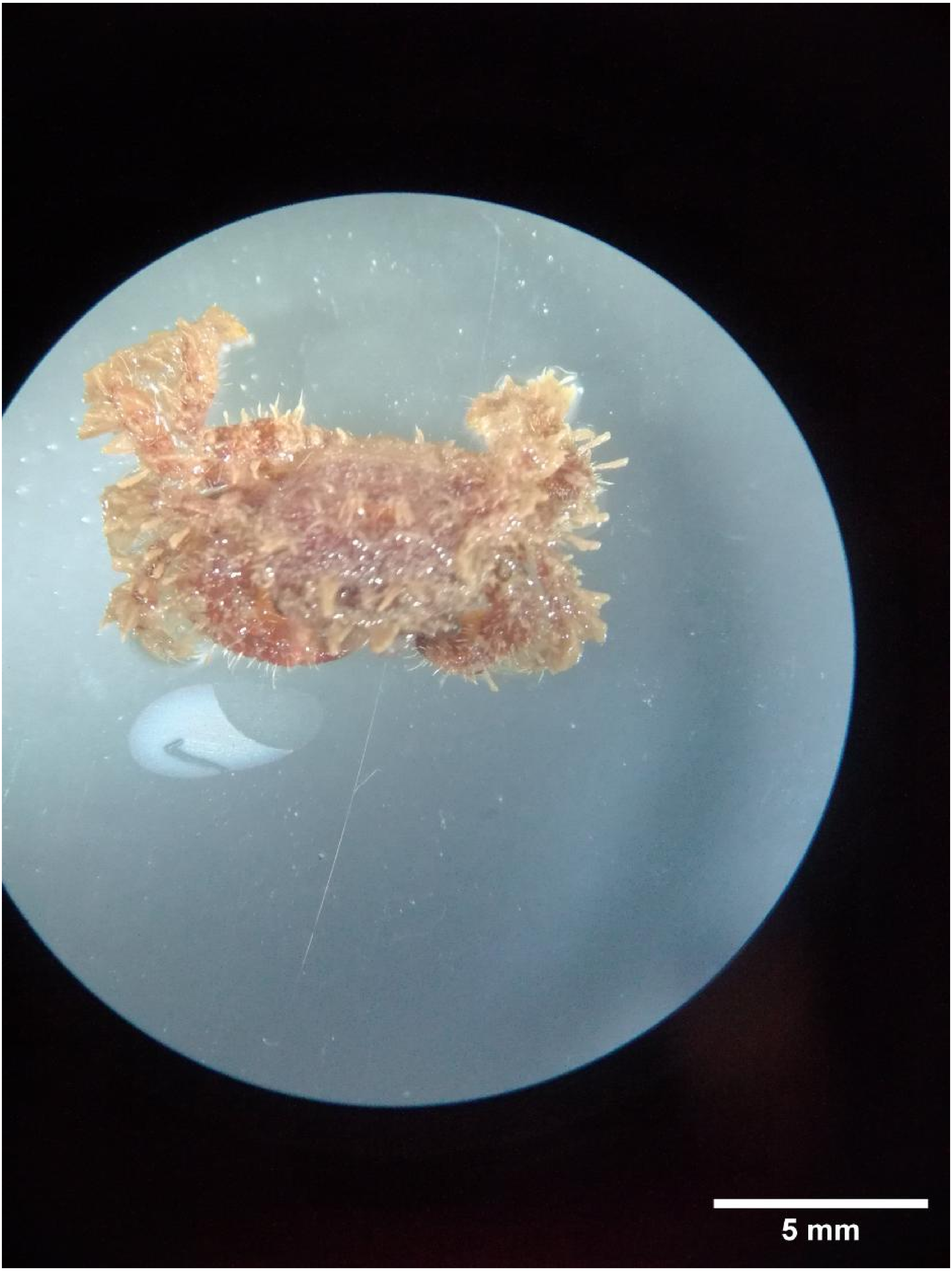
5 mm

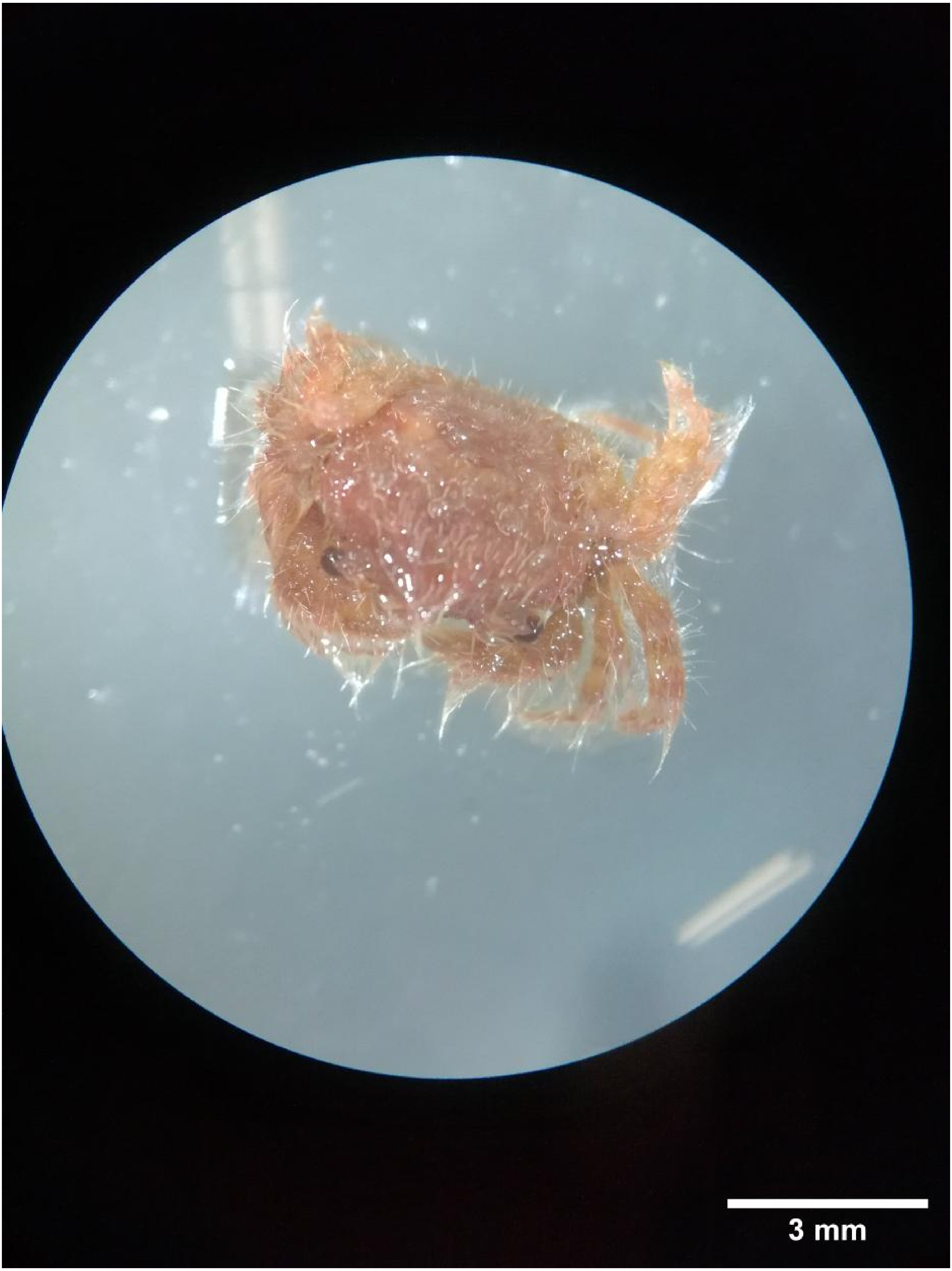
3 mm

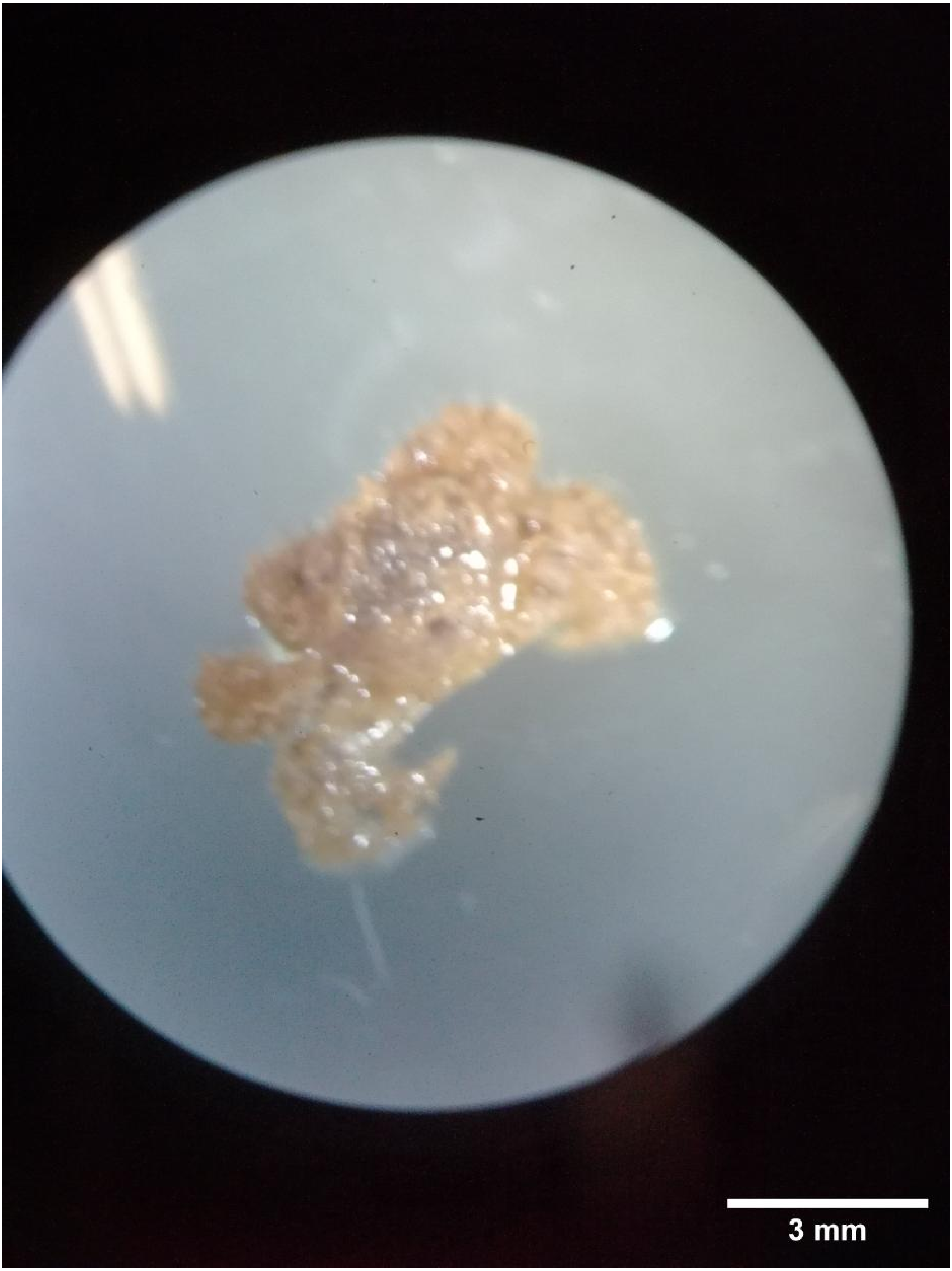
3 mm

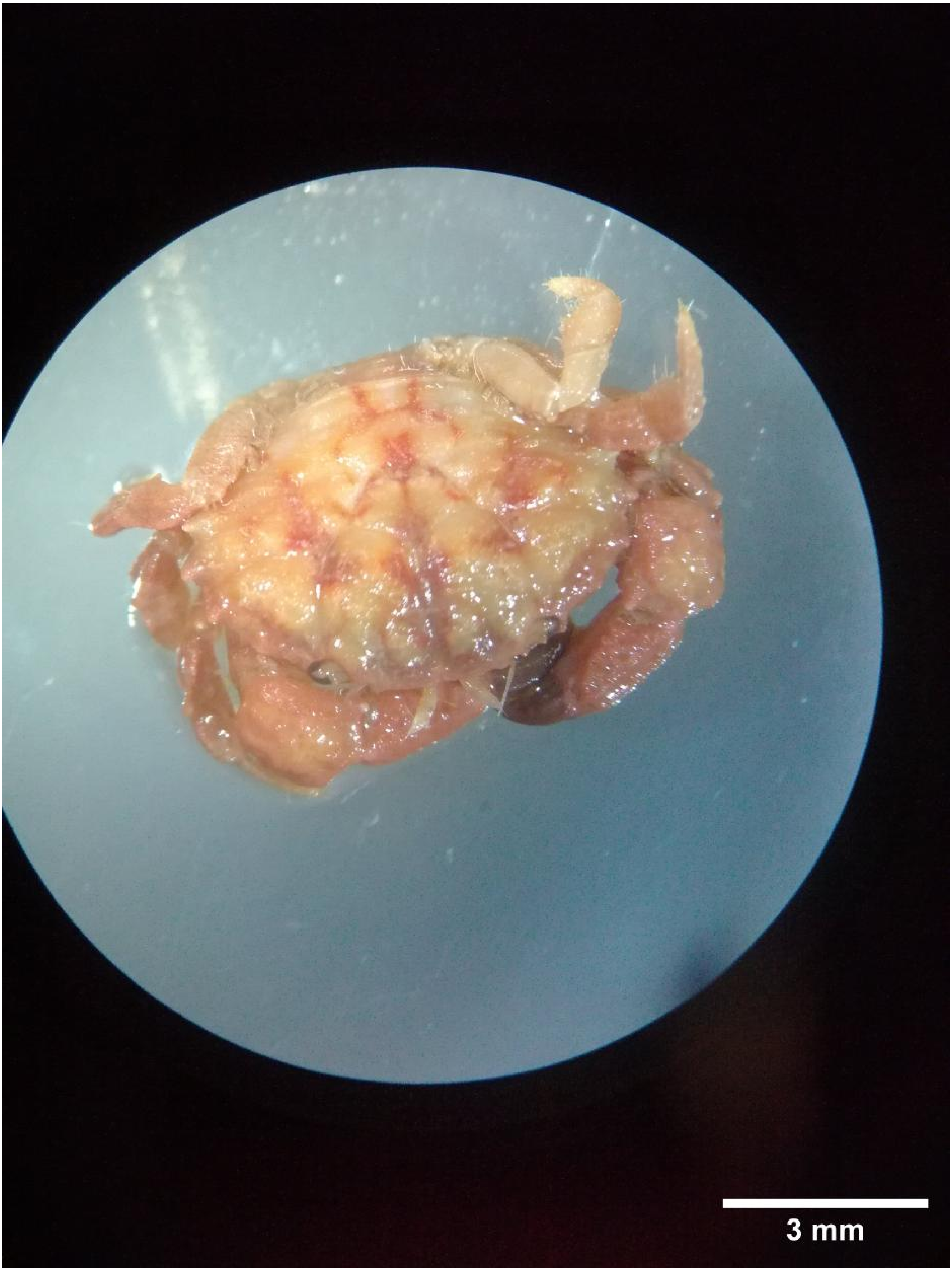
3 mm

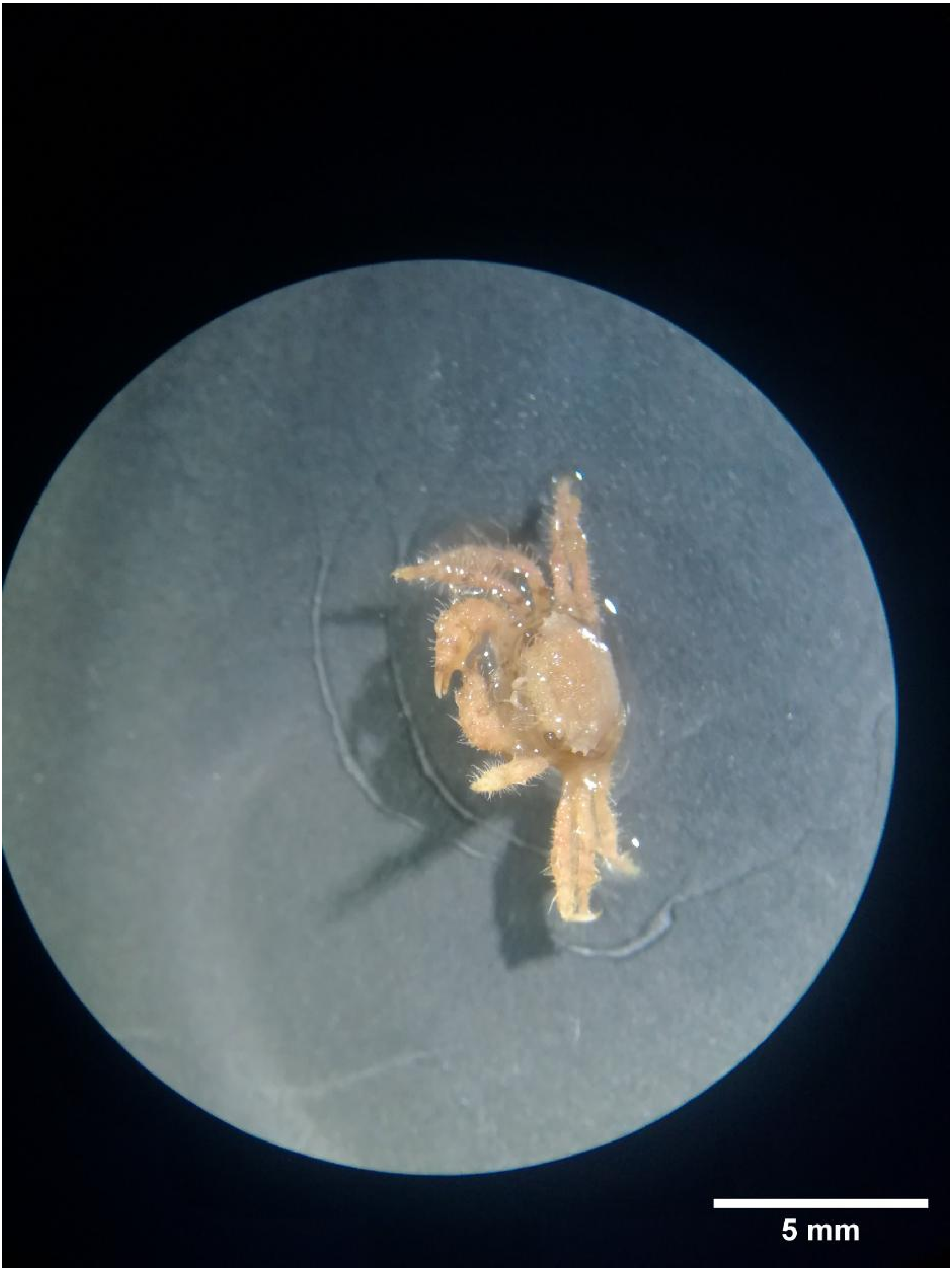
5 mm

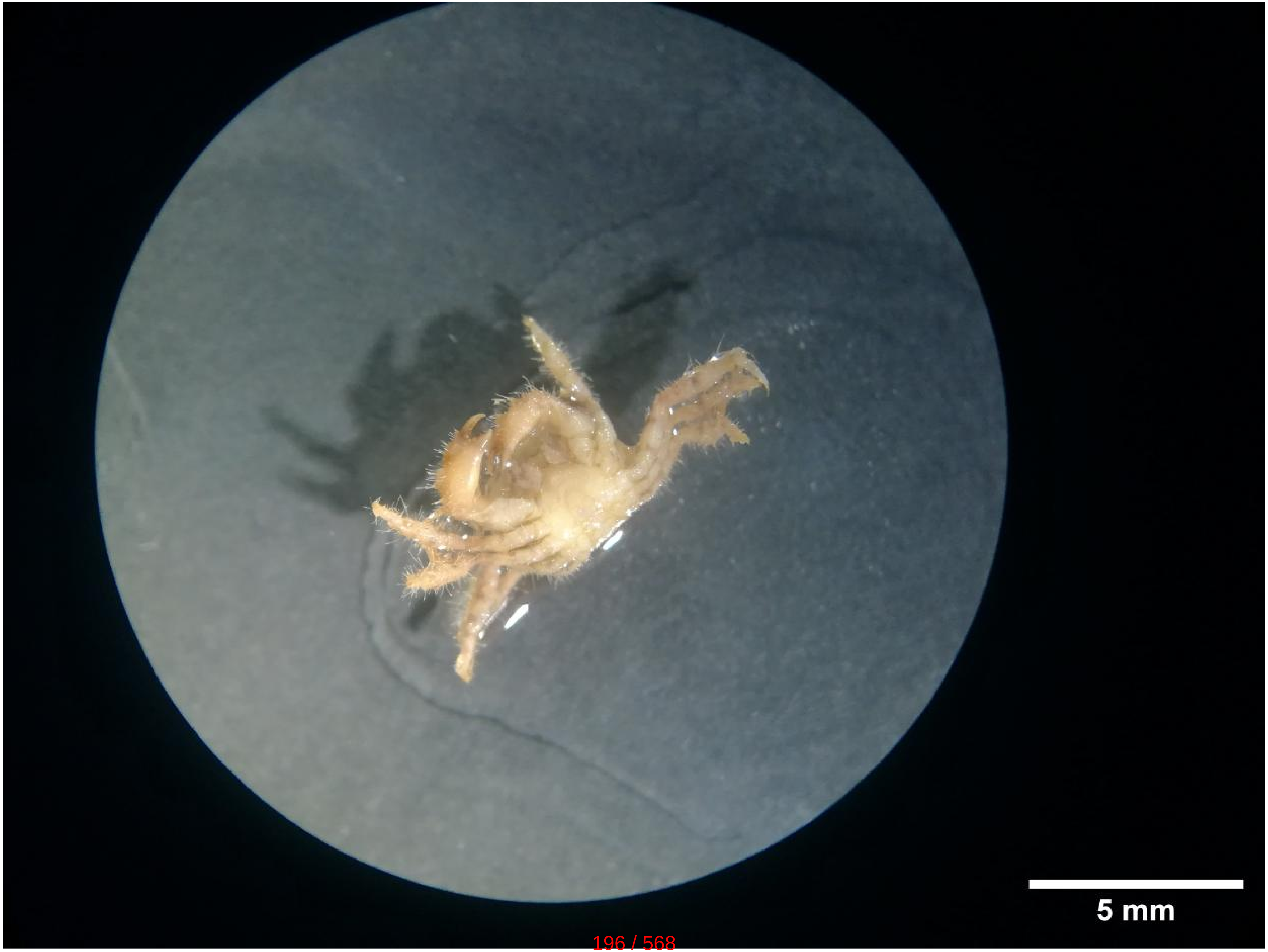
5 mm

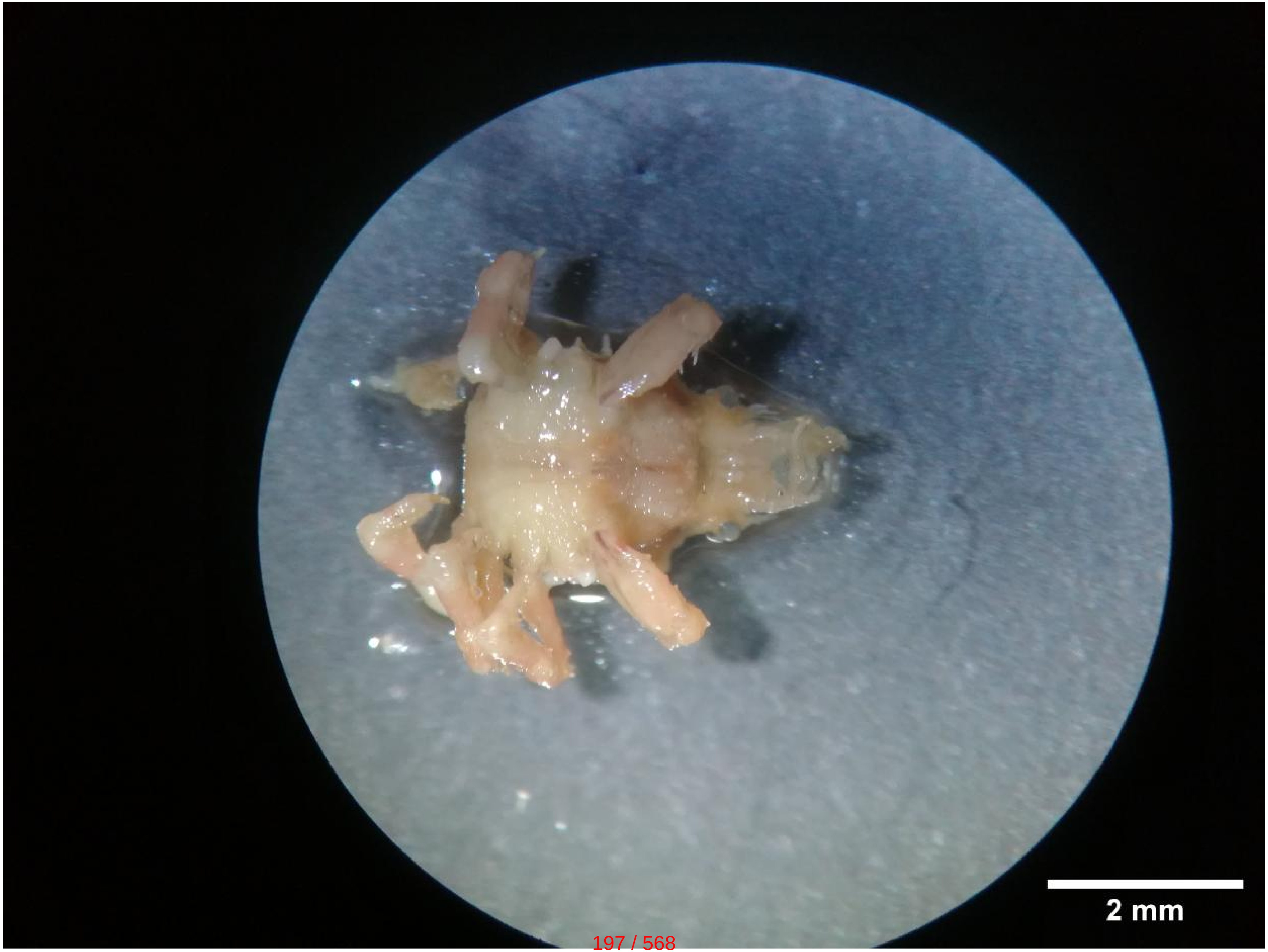
2 mm

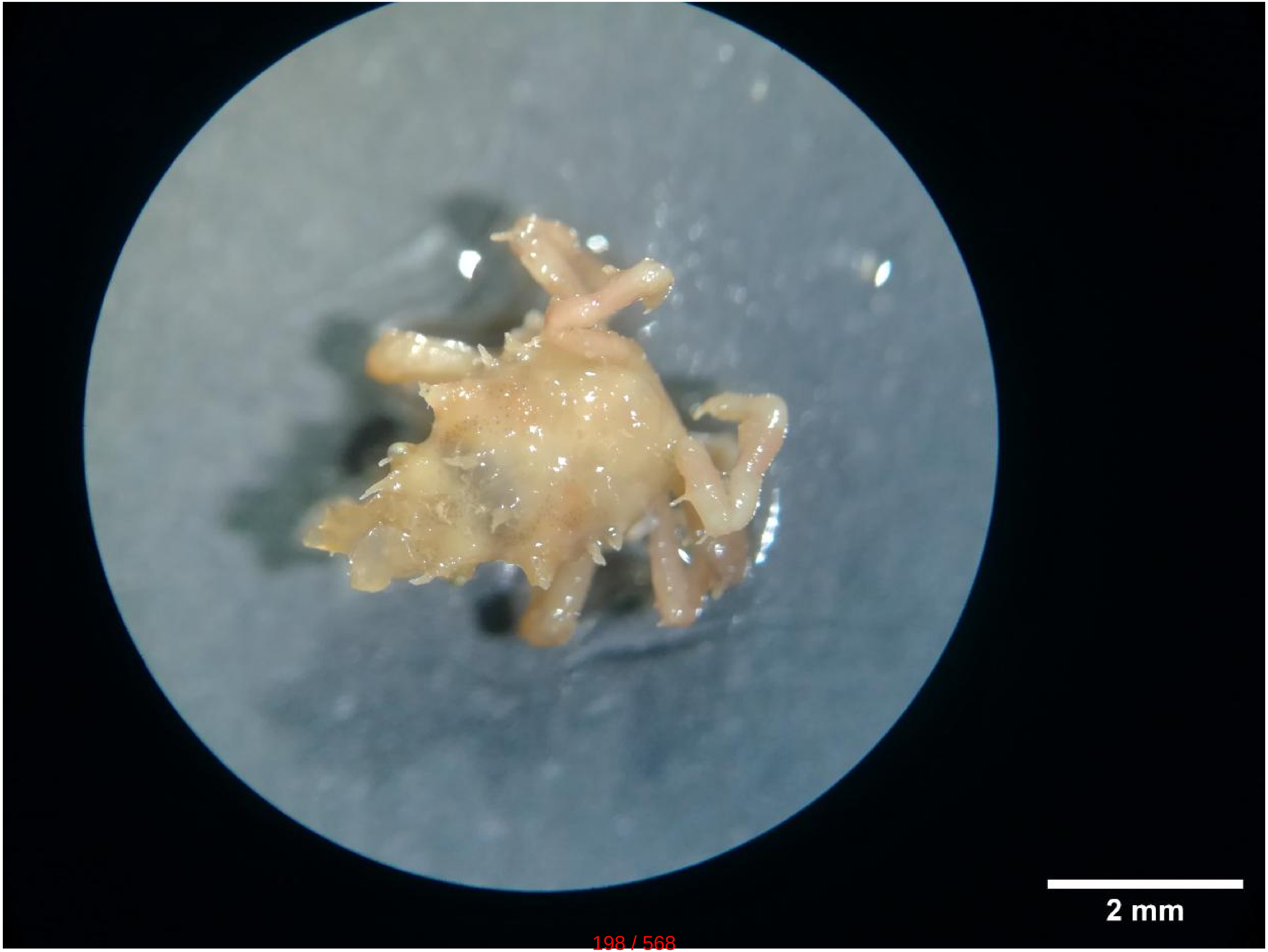
2 mm

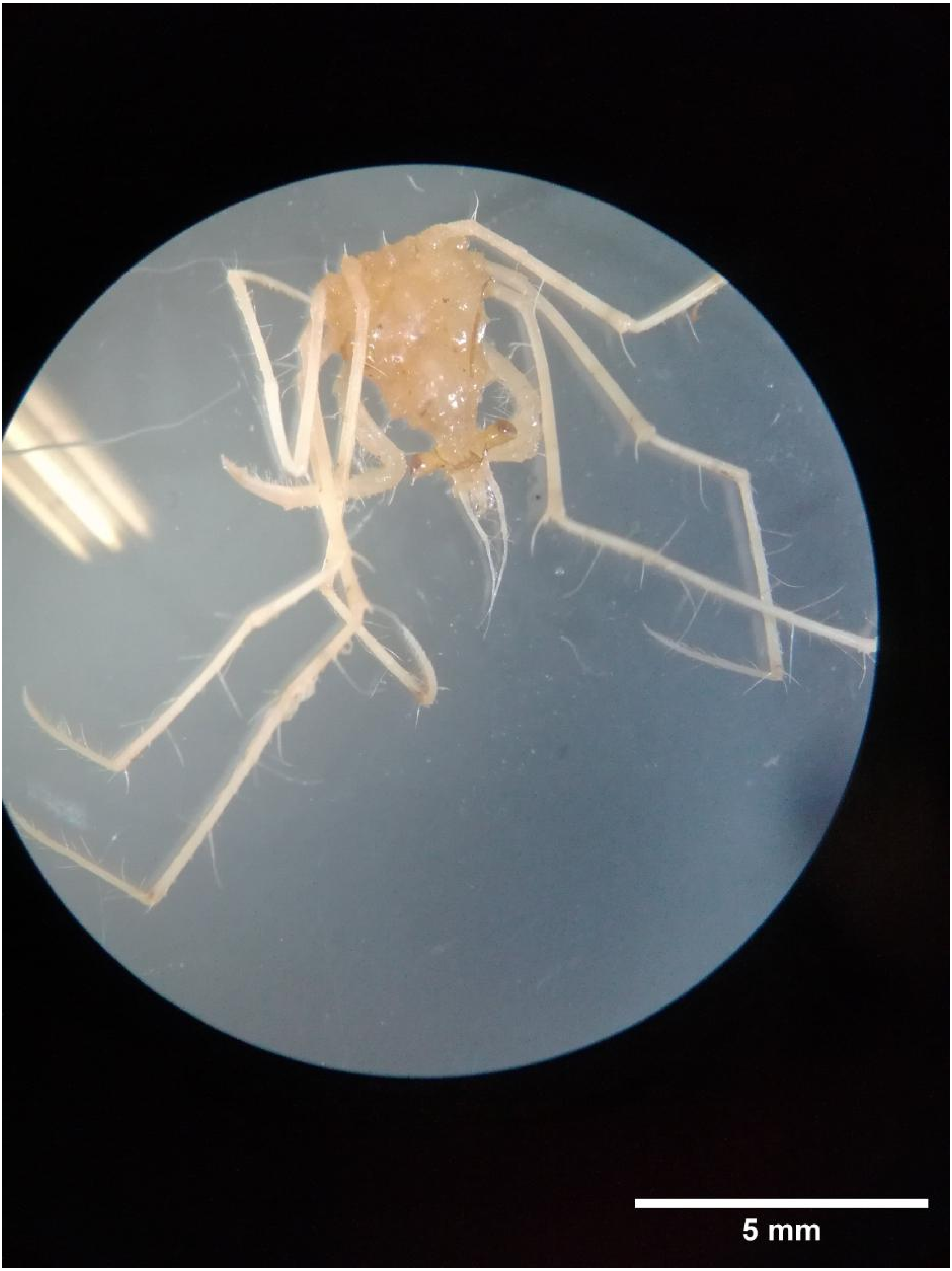
5 mm

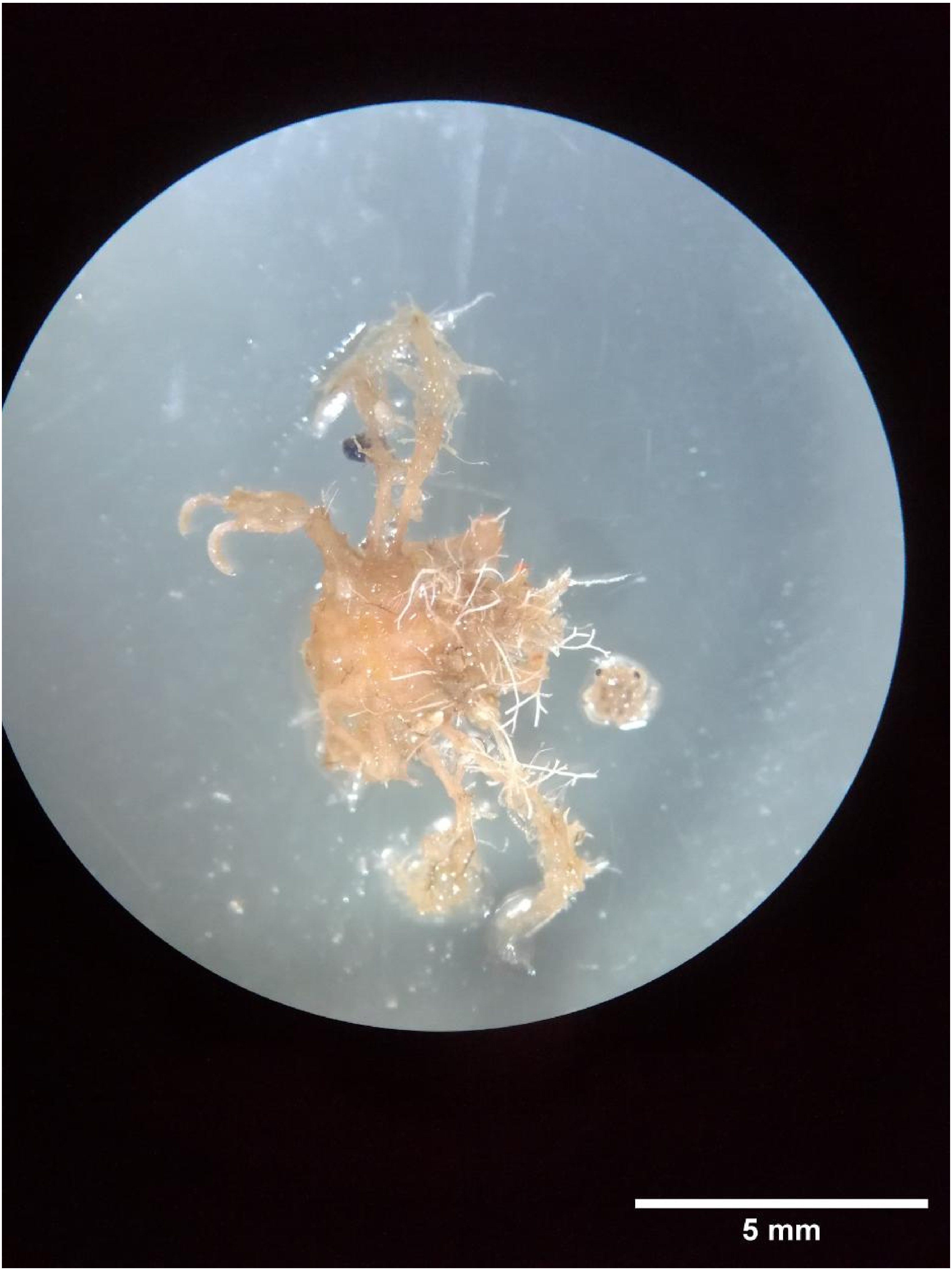
5 mm

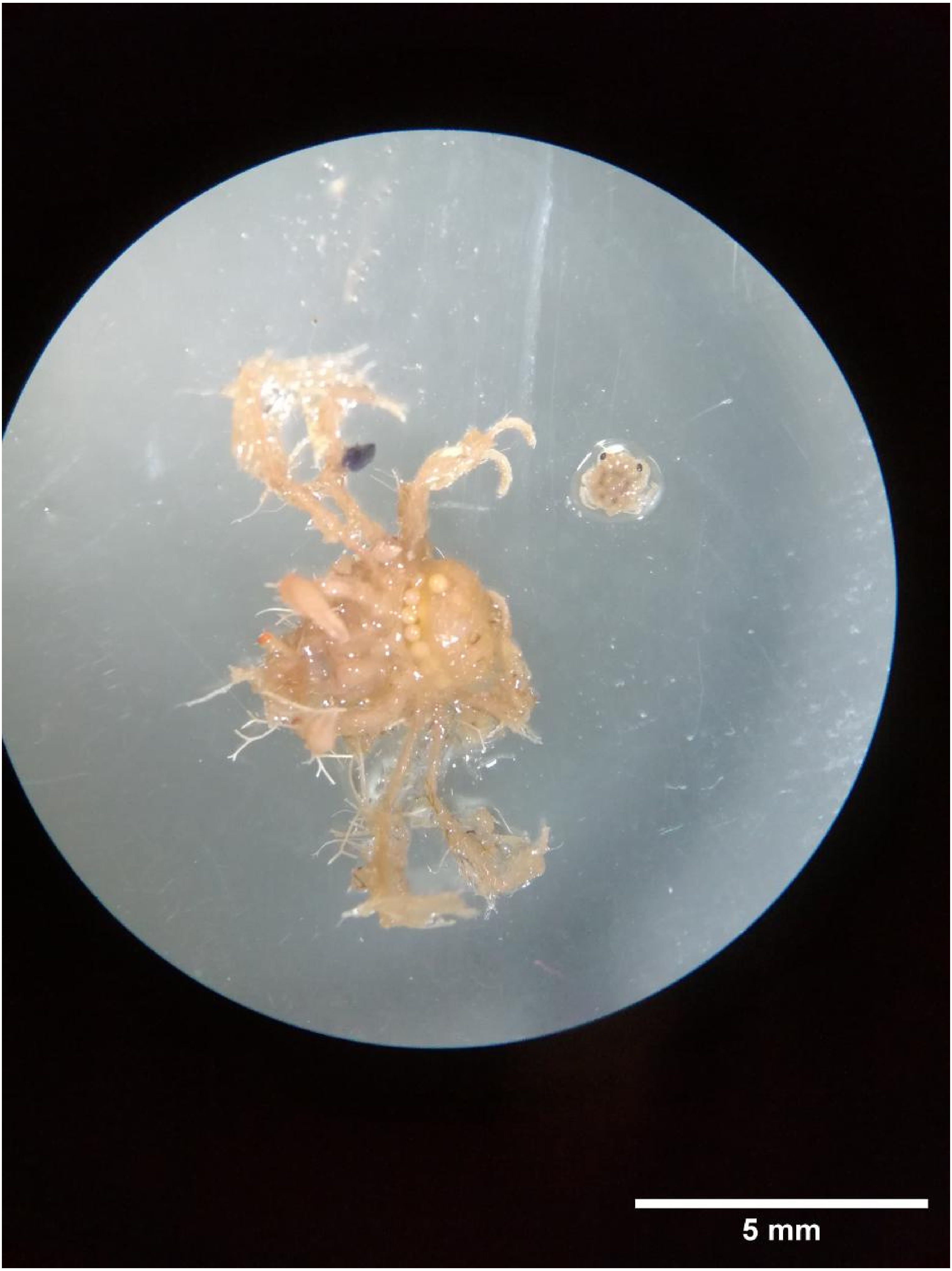
5 mm

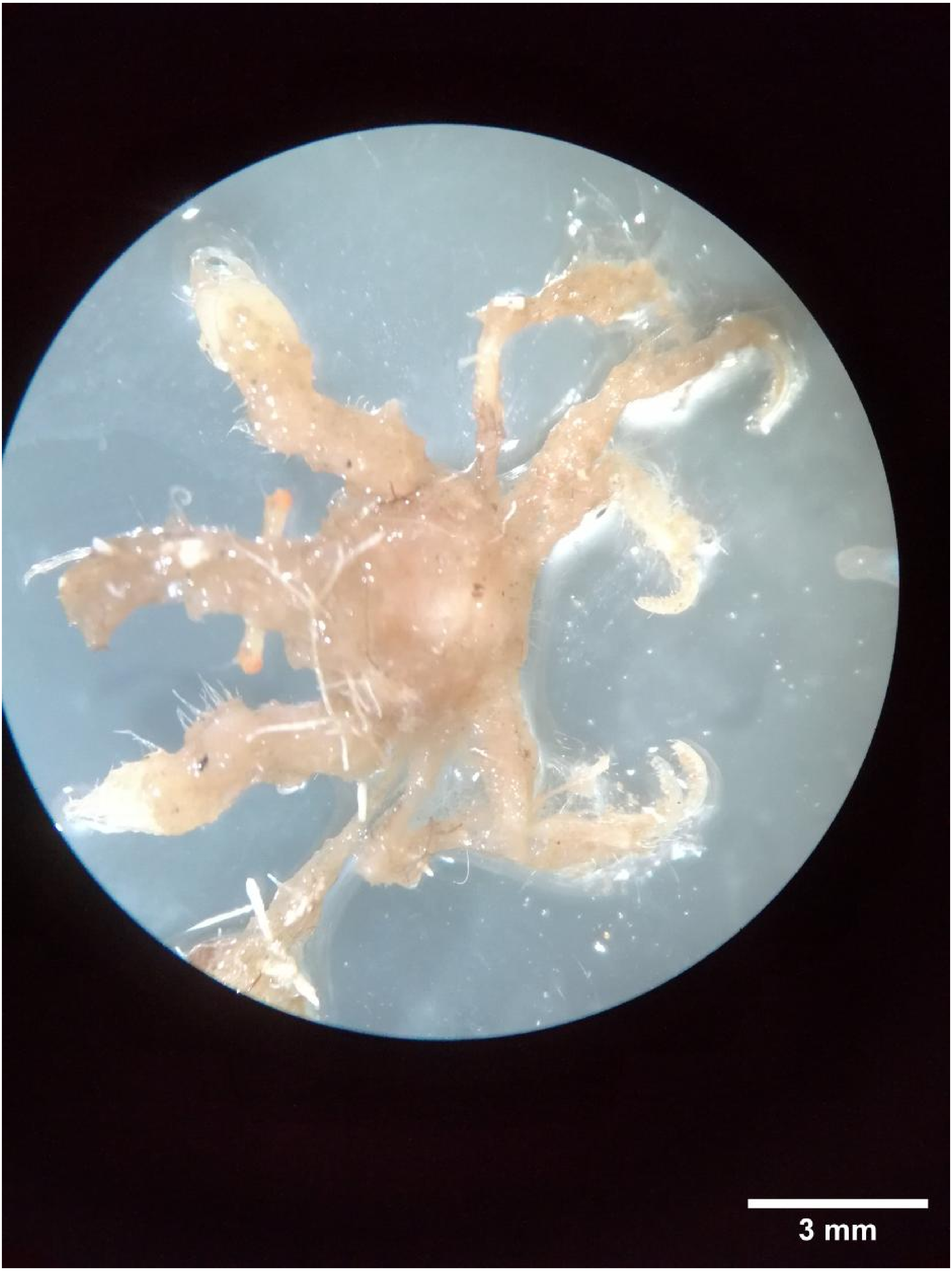
3 mm

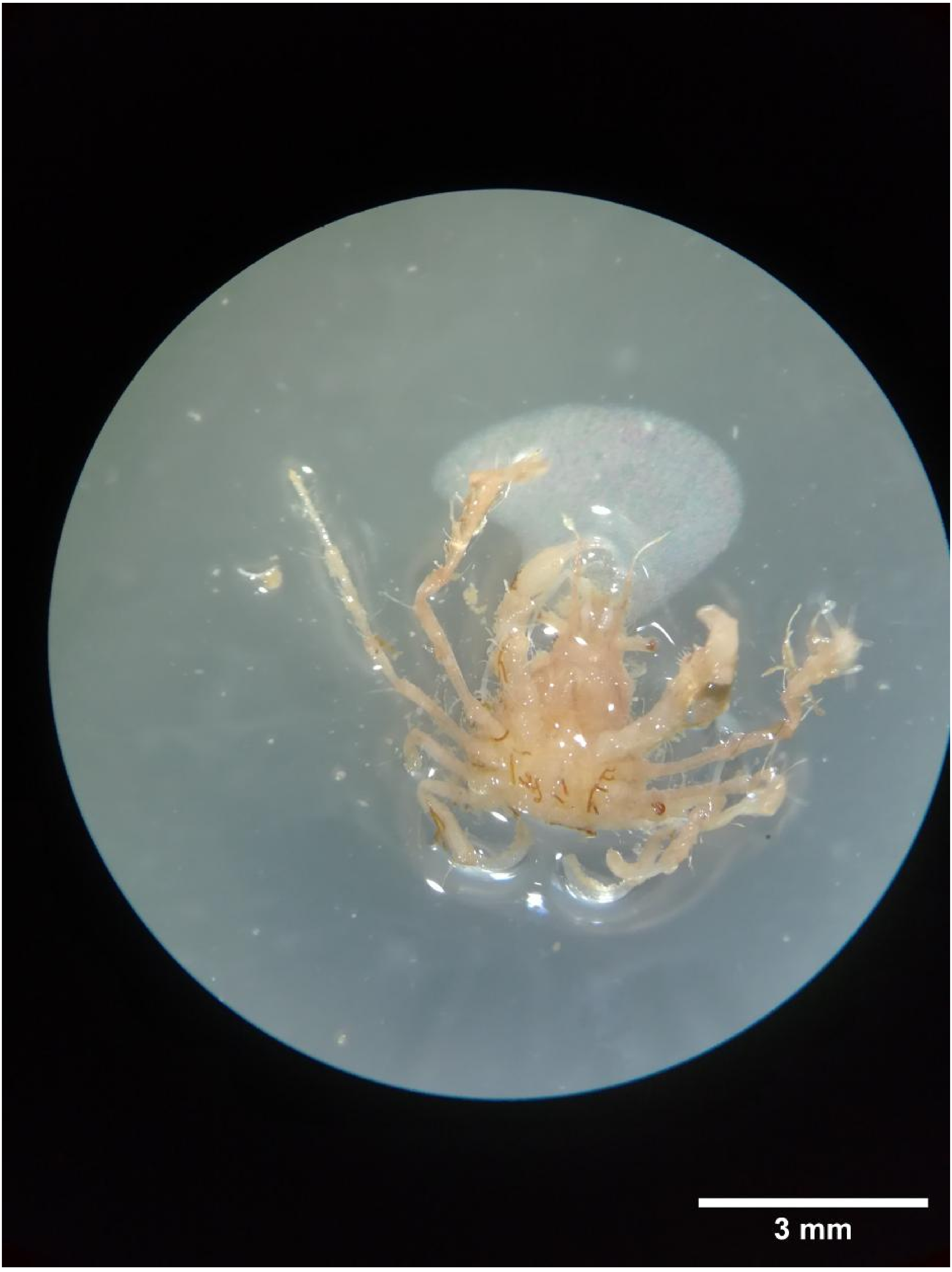
3 mm

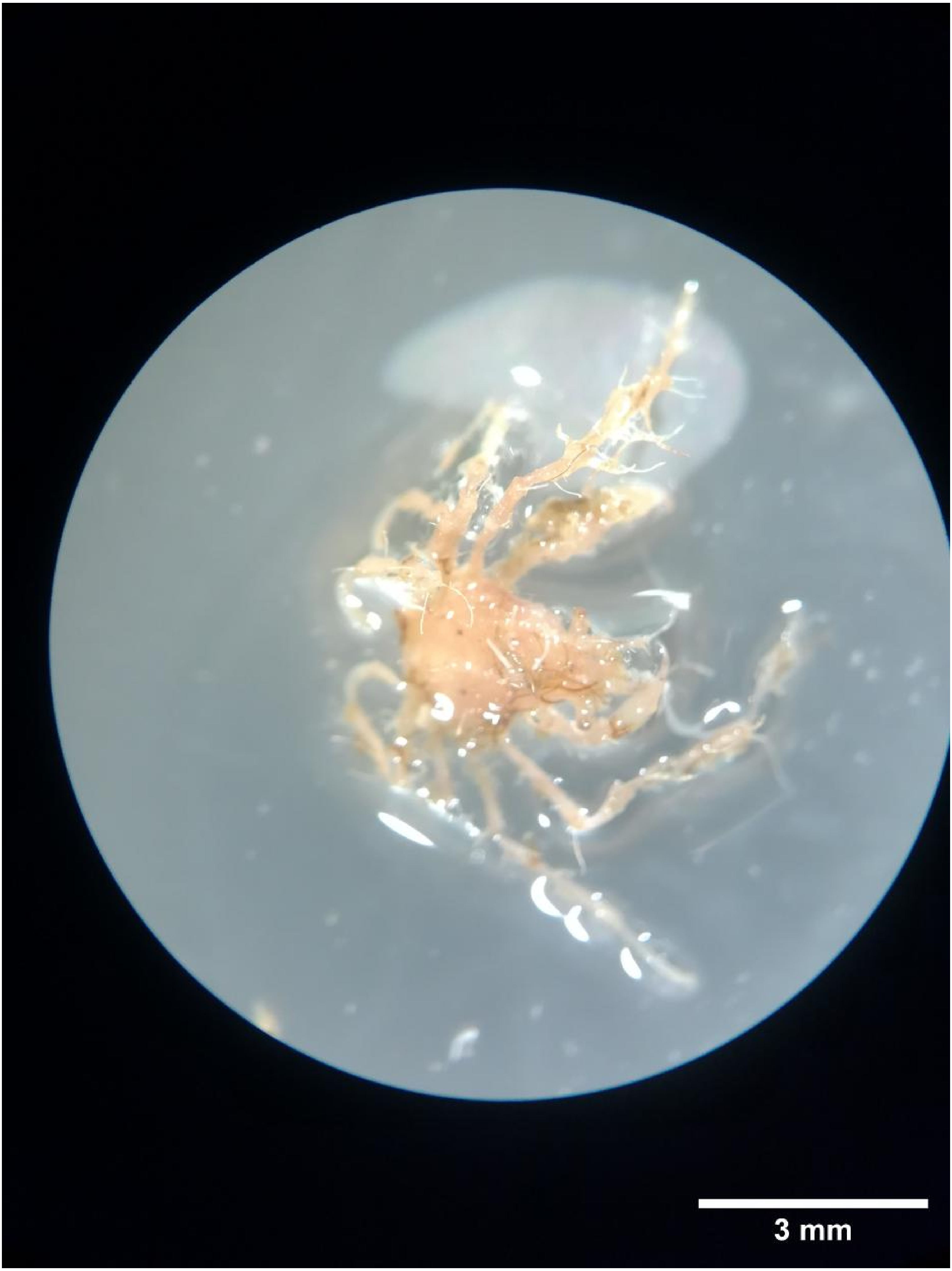
3 mm

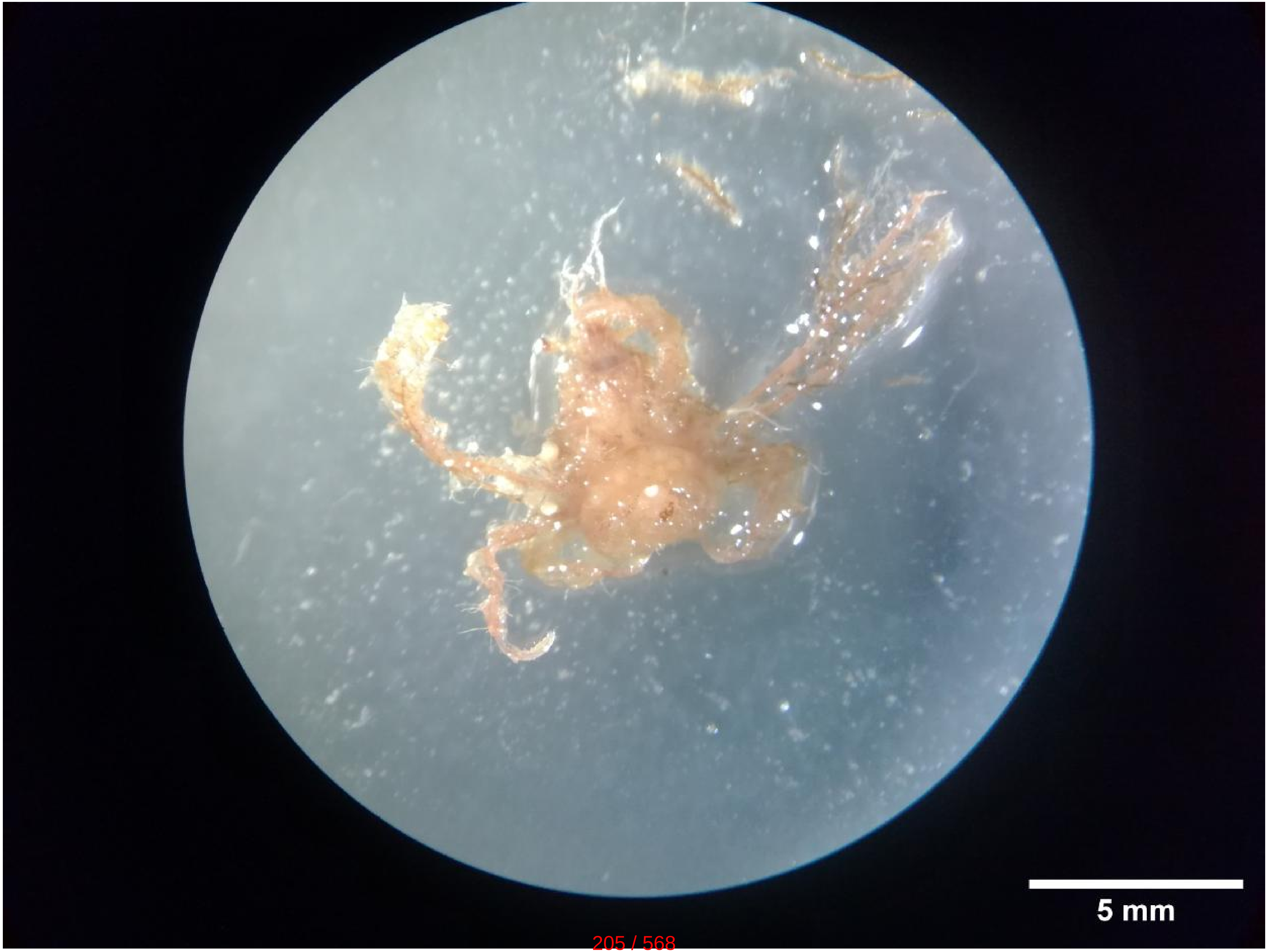
5 mm

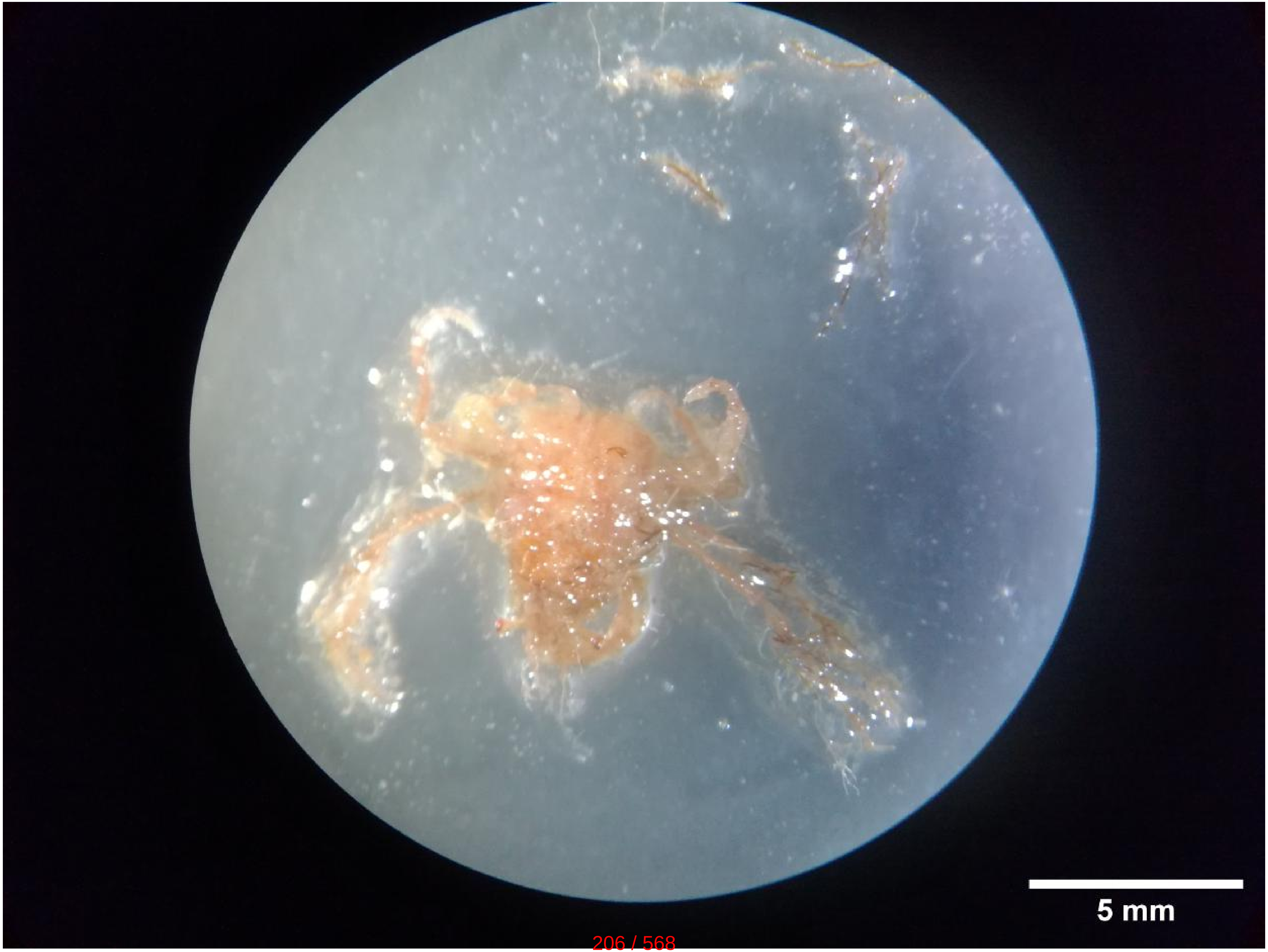
5 mm

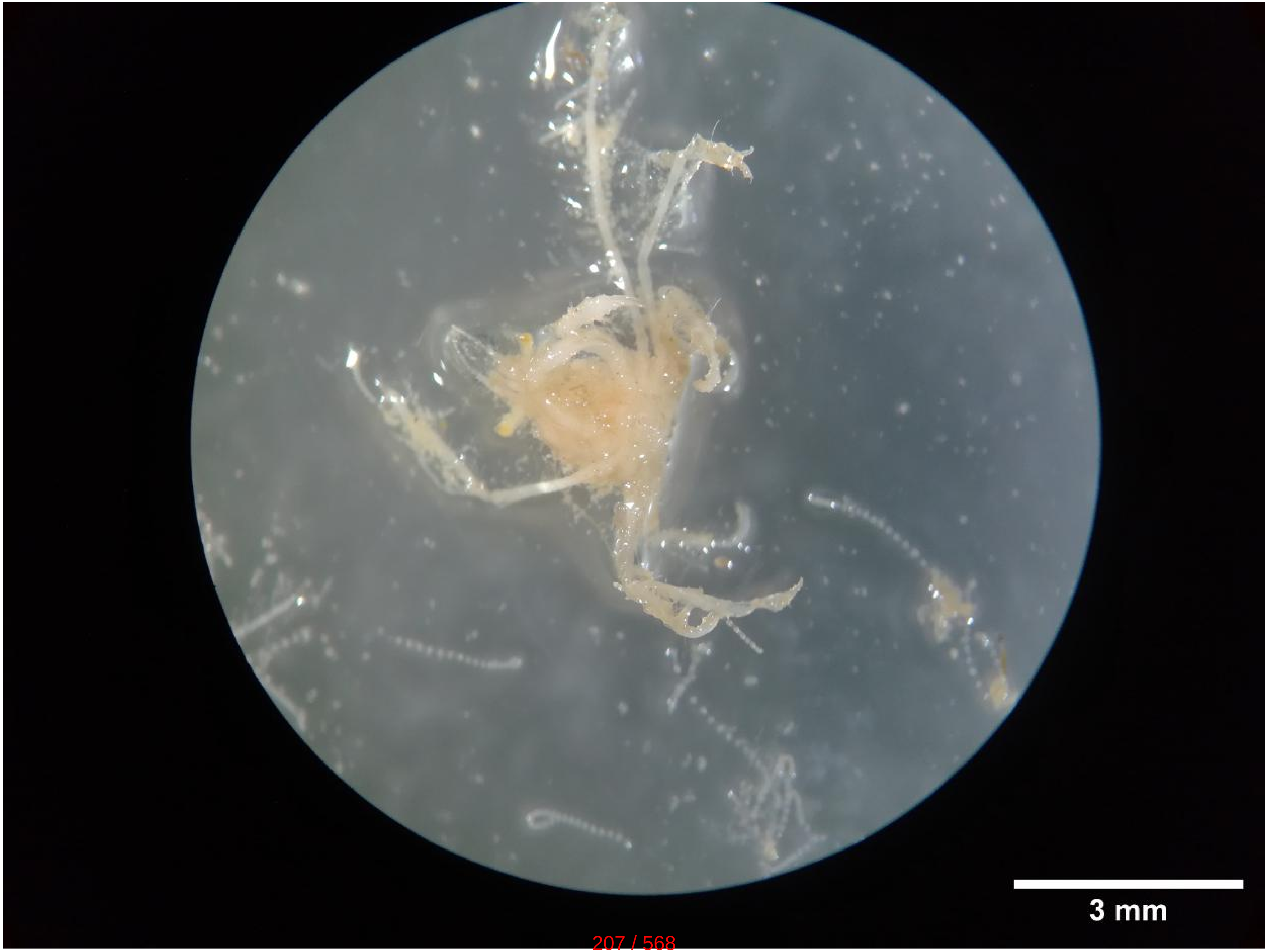
3 mm

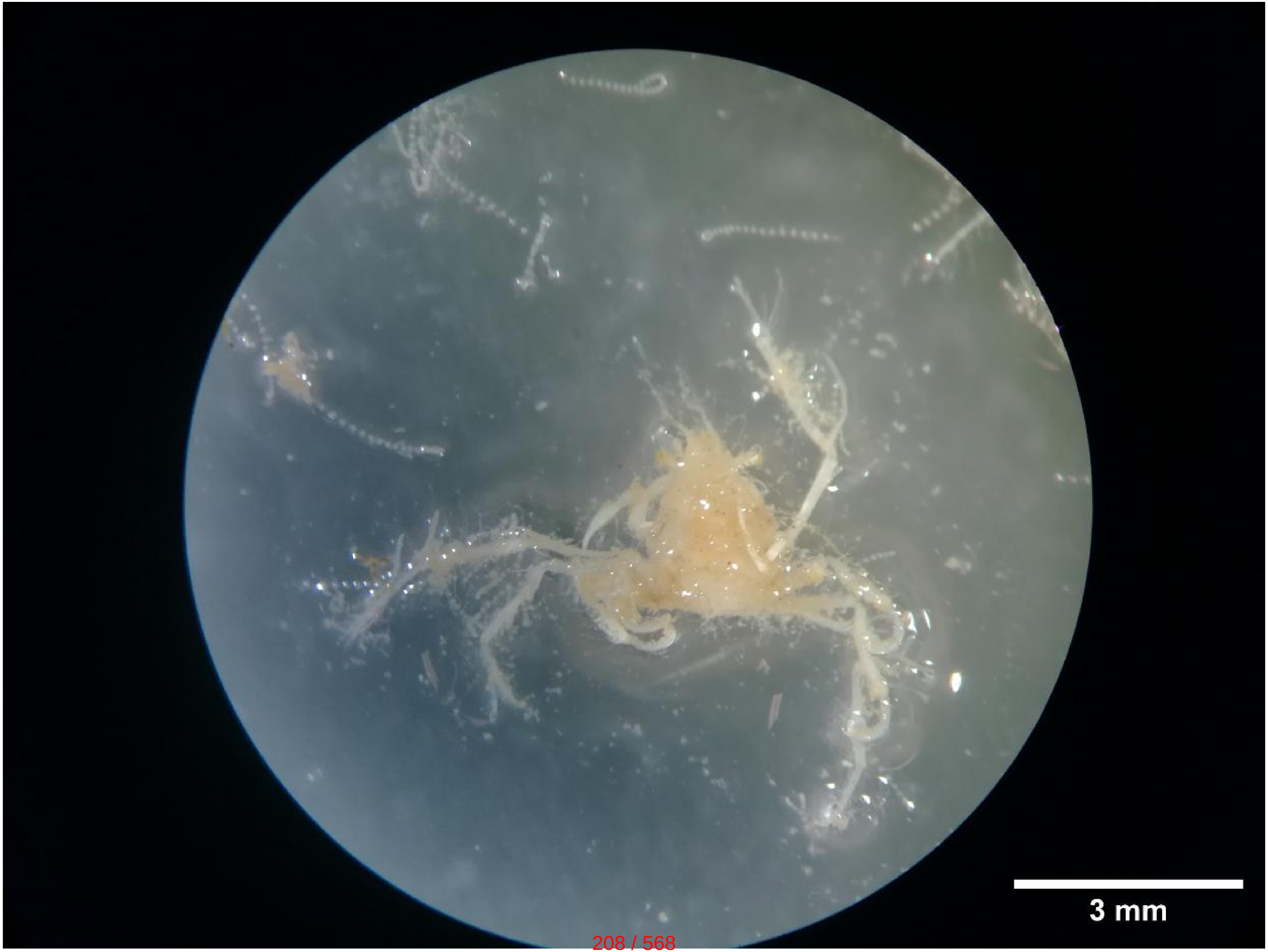
3 mm

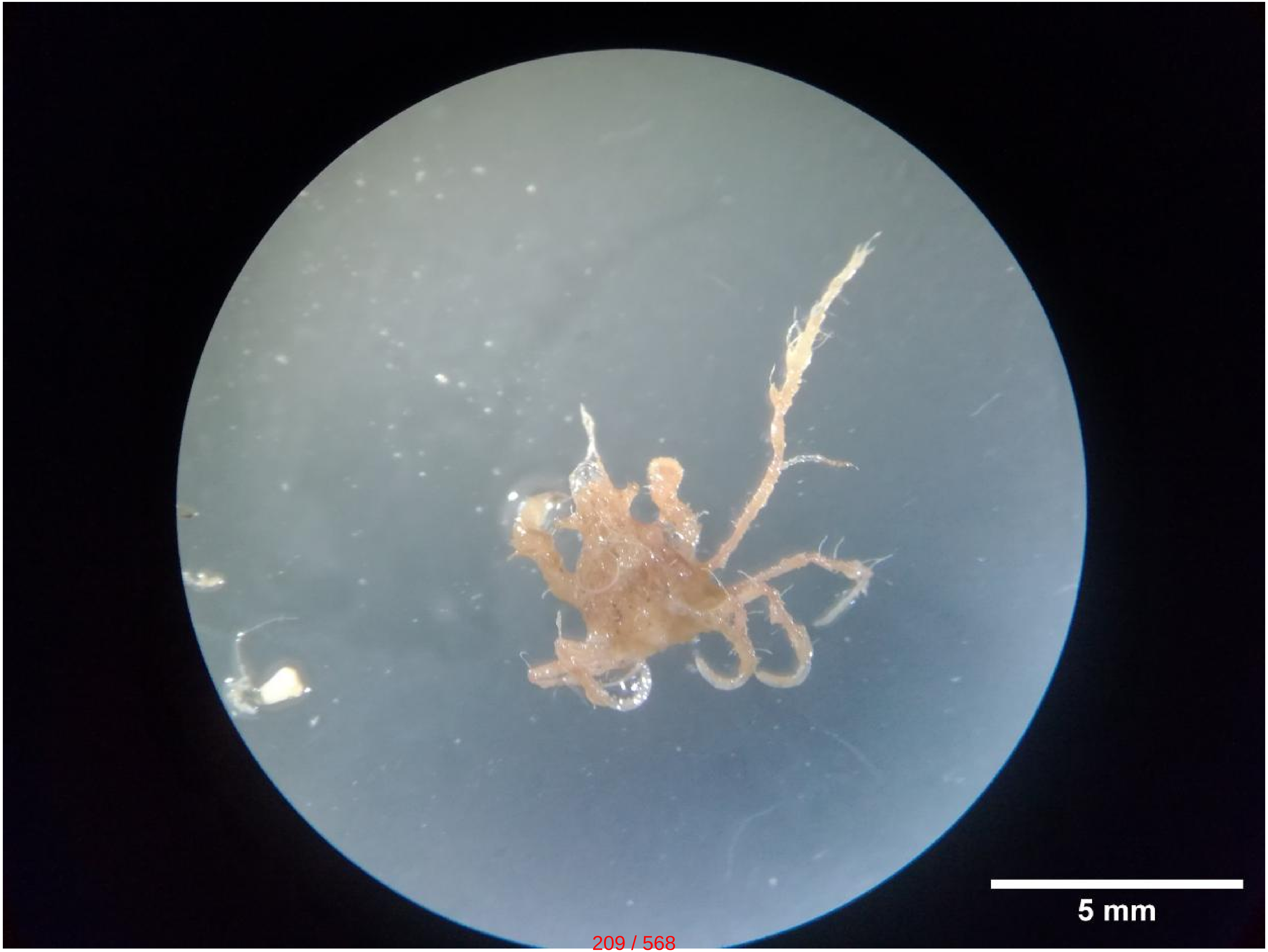
5 mm

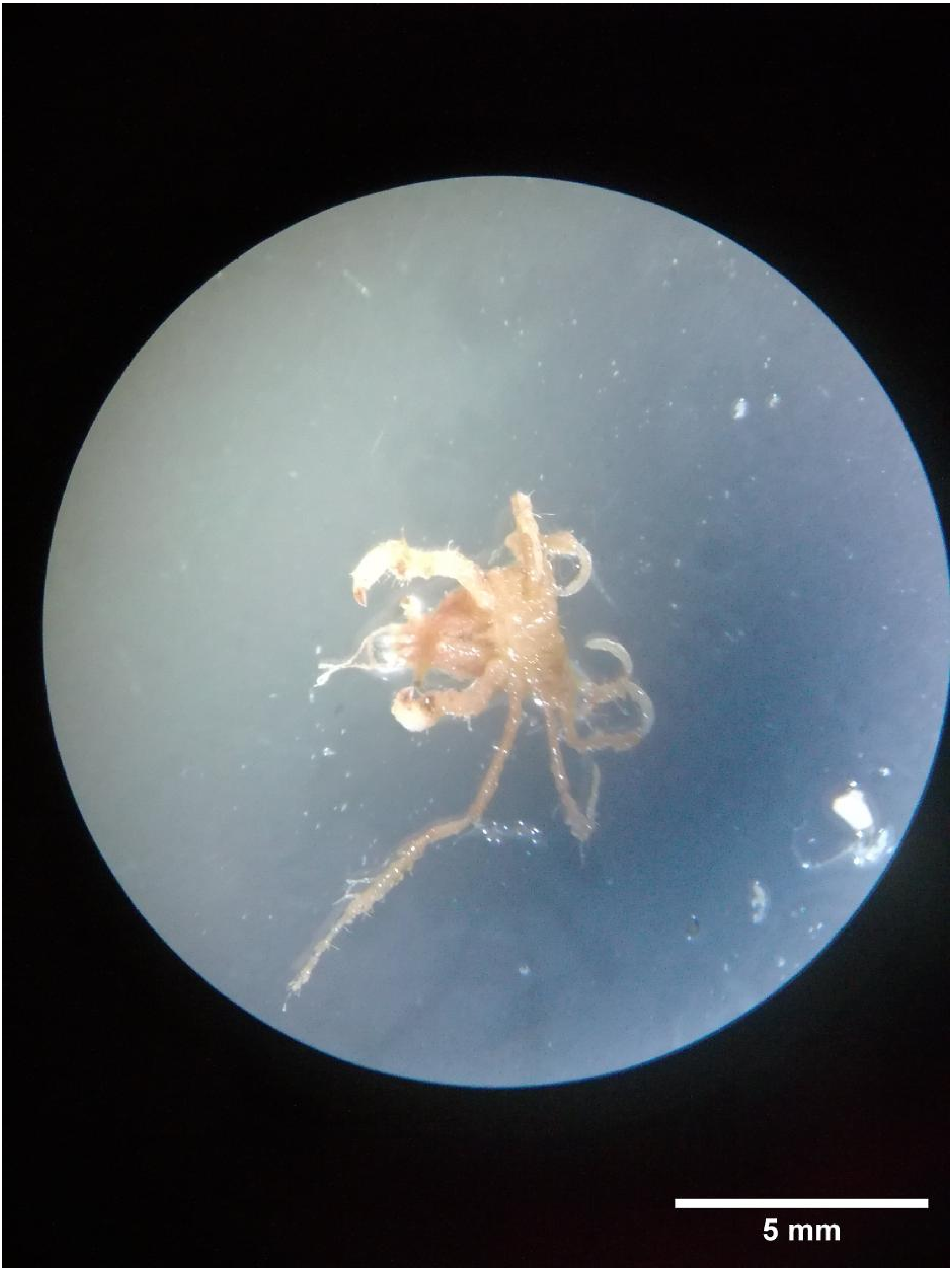
5 mm

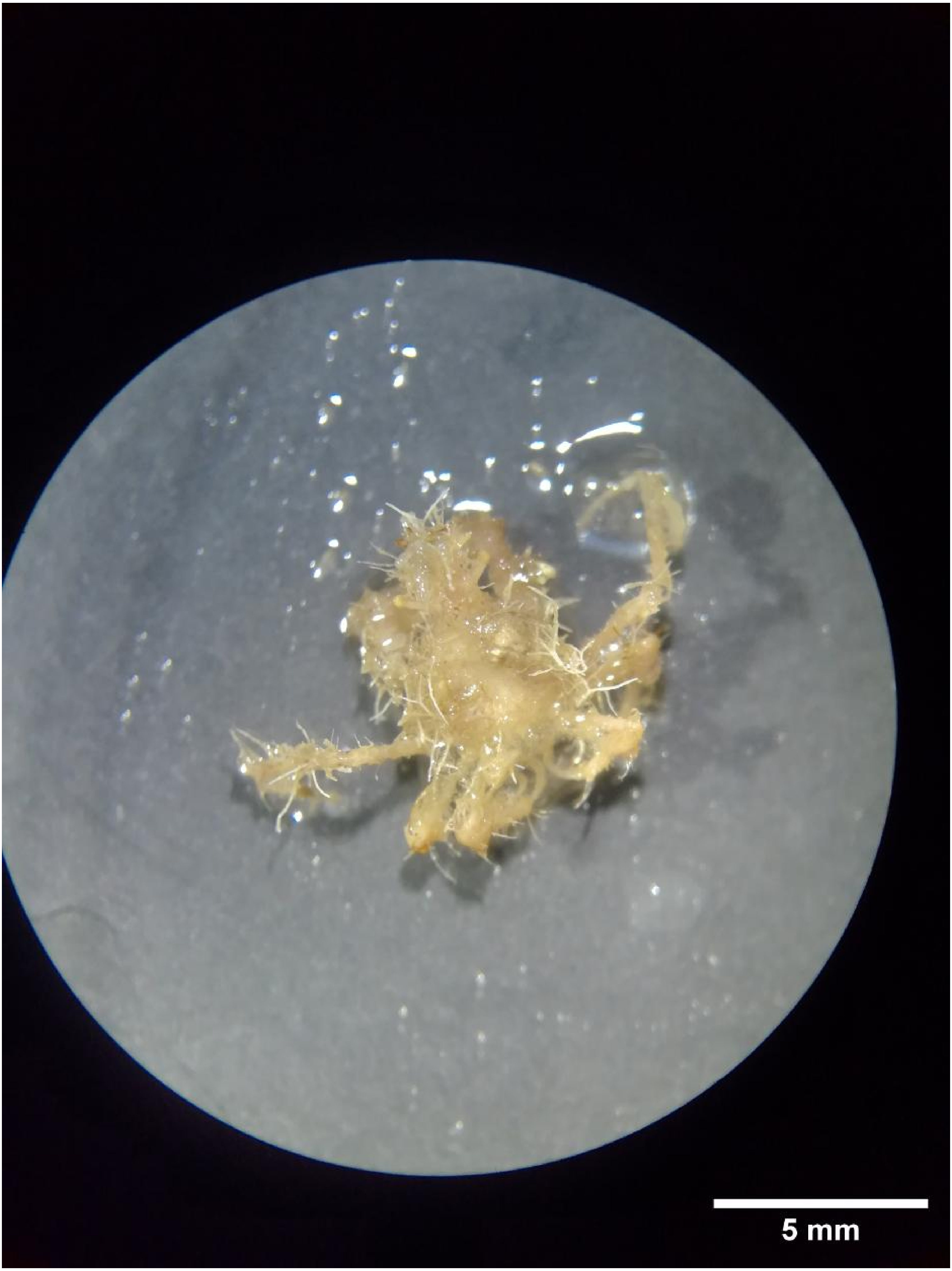
5 mm

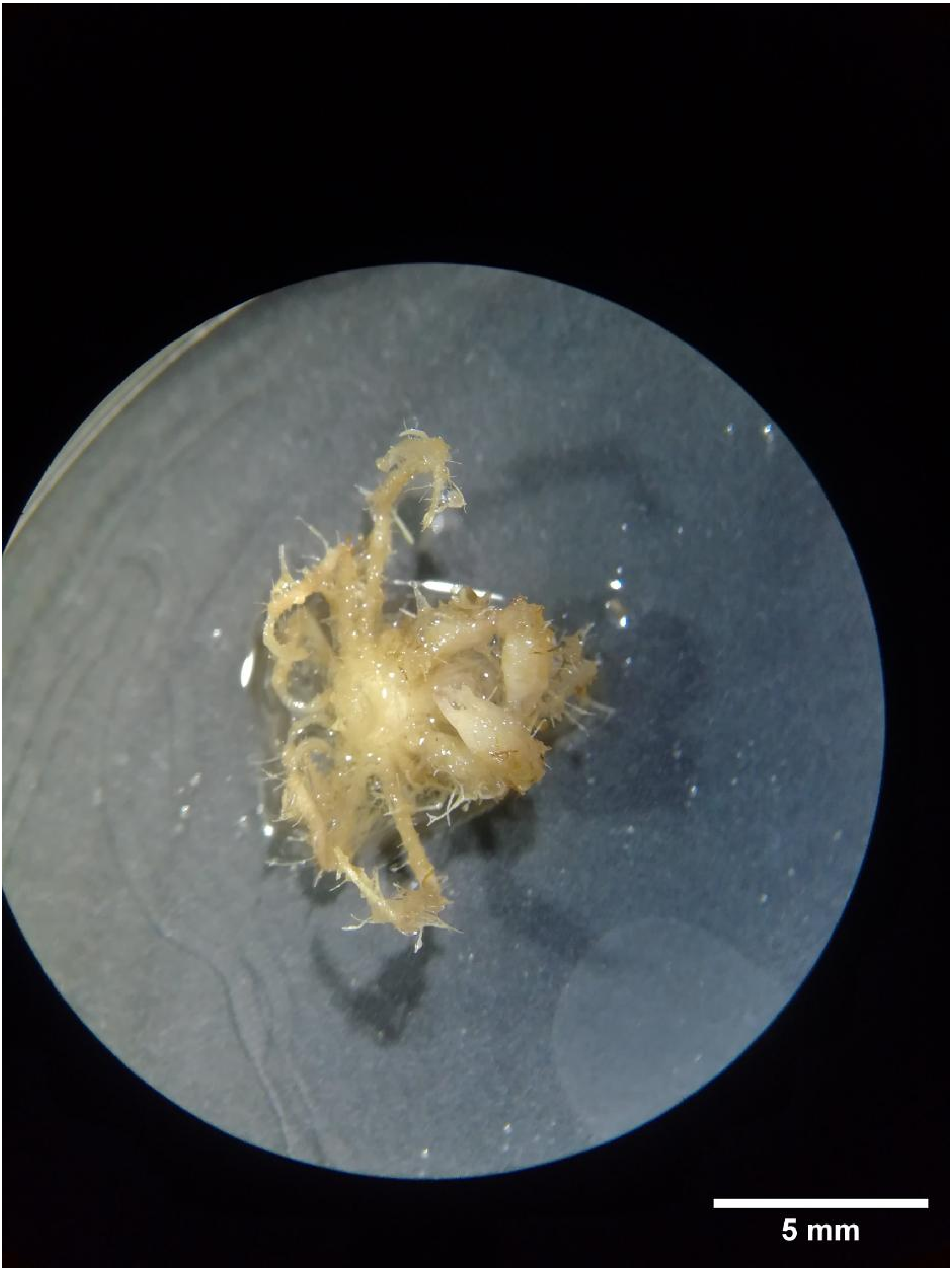
5 mm

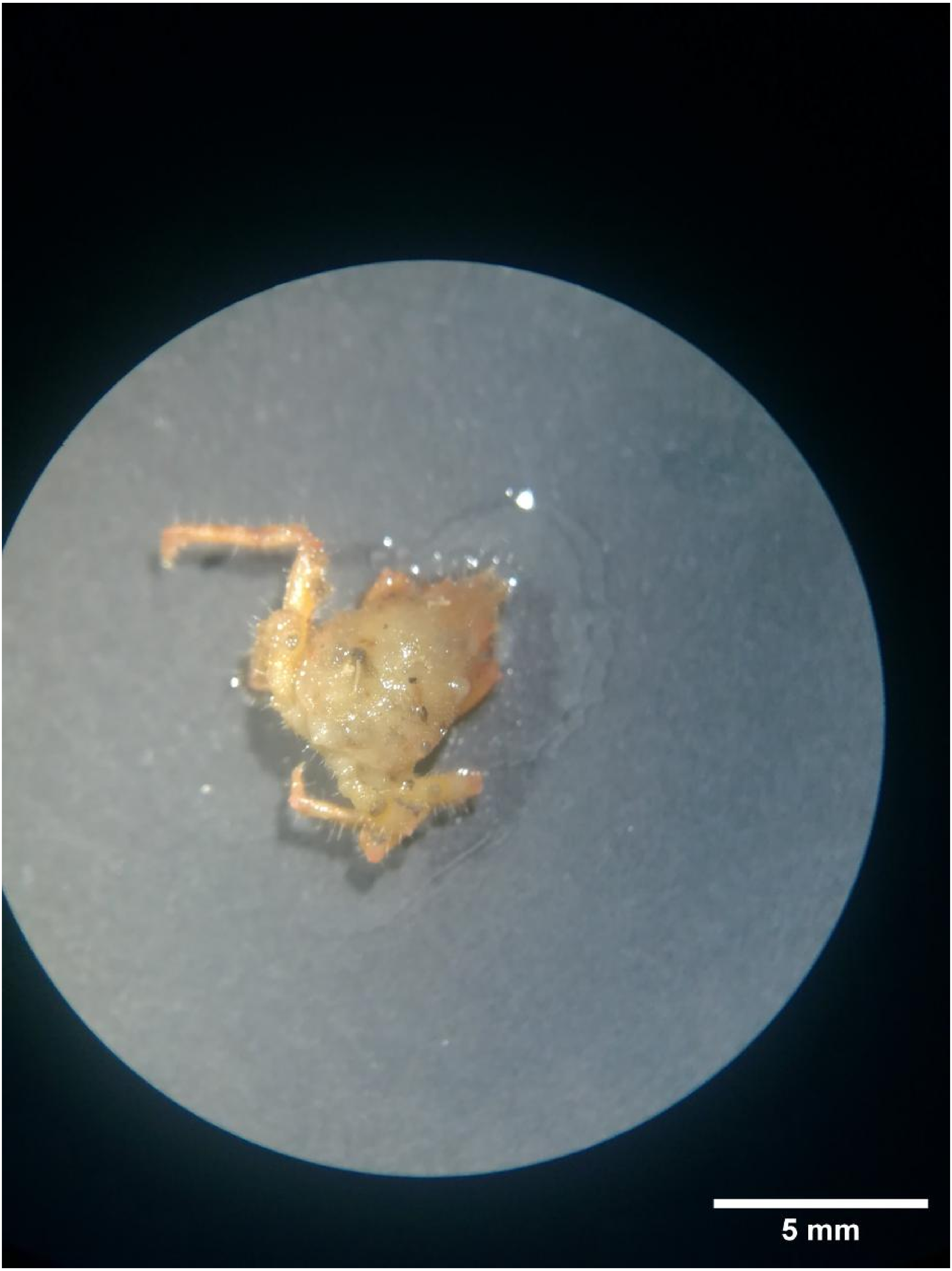
5 mm

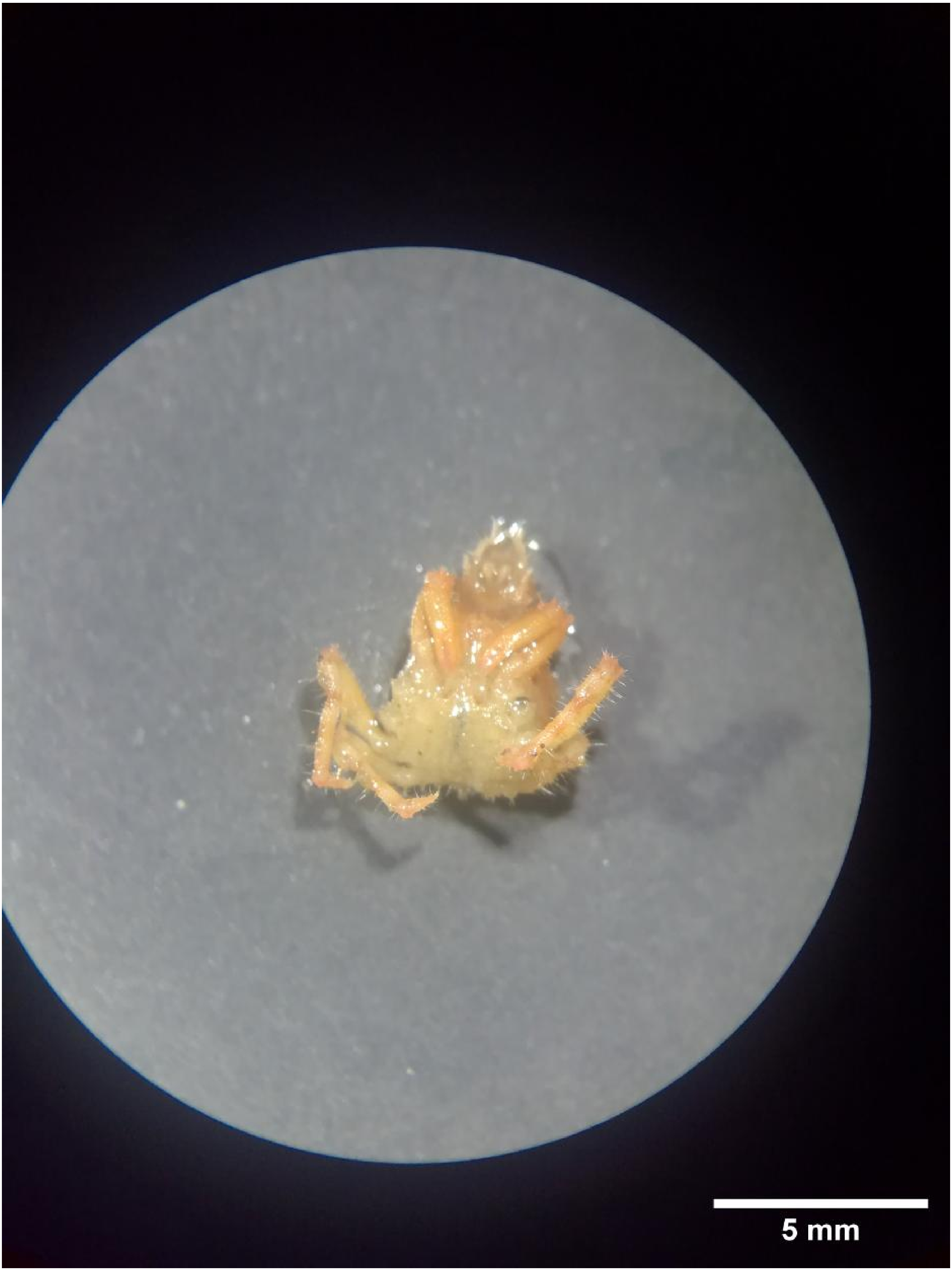
5 mm

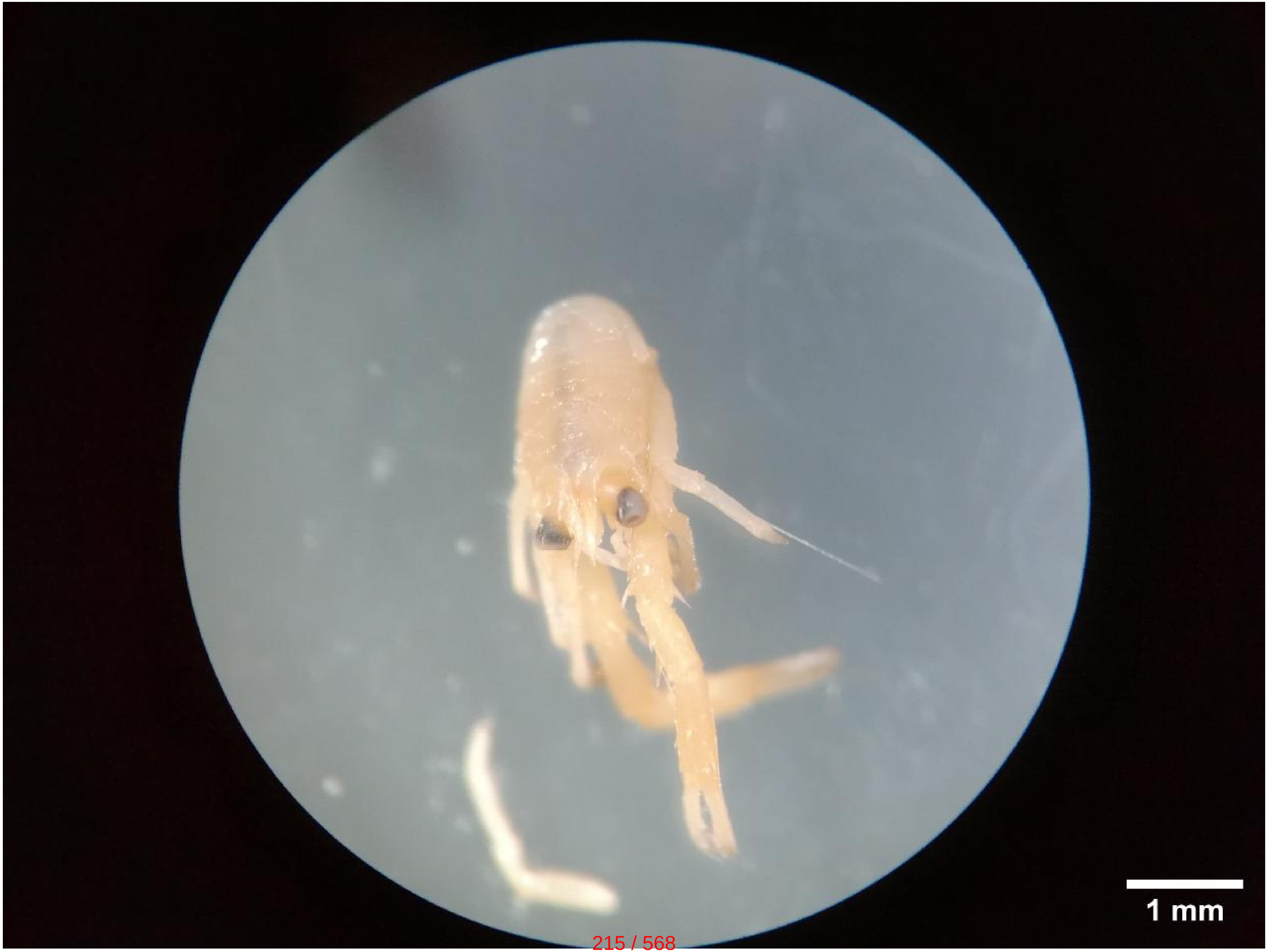
1 mm

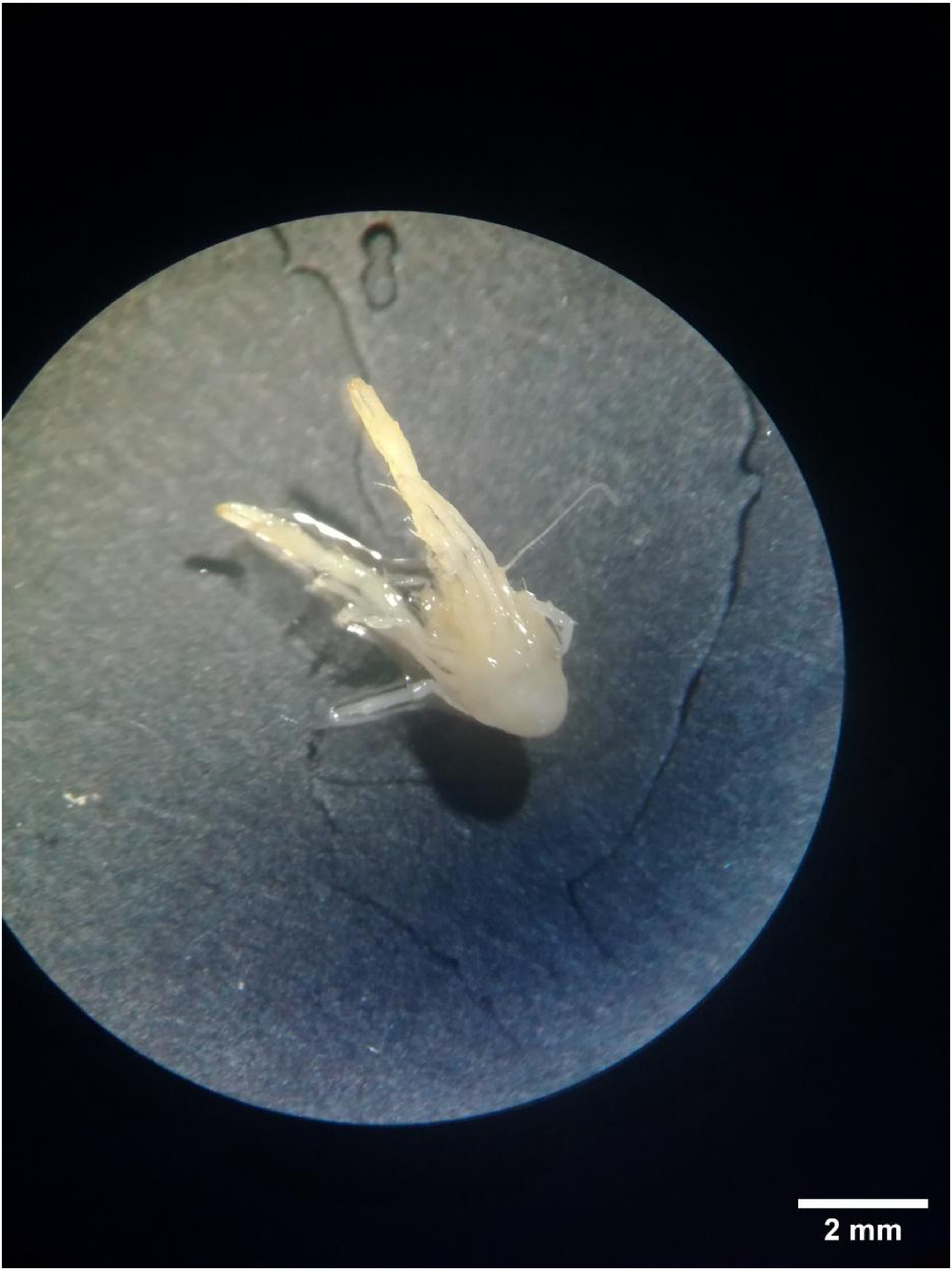
2 mm

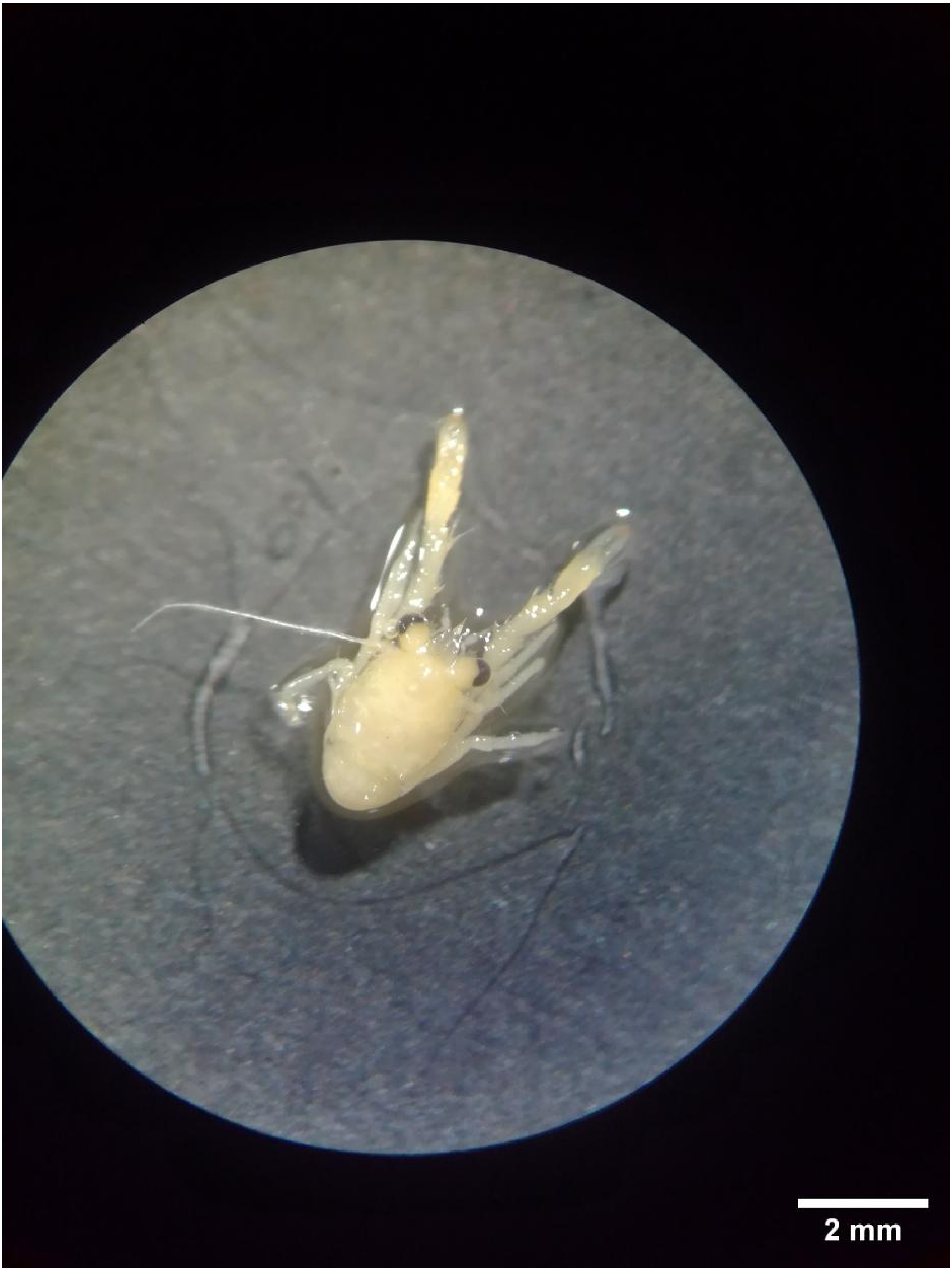
2 mm

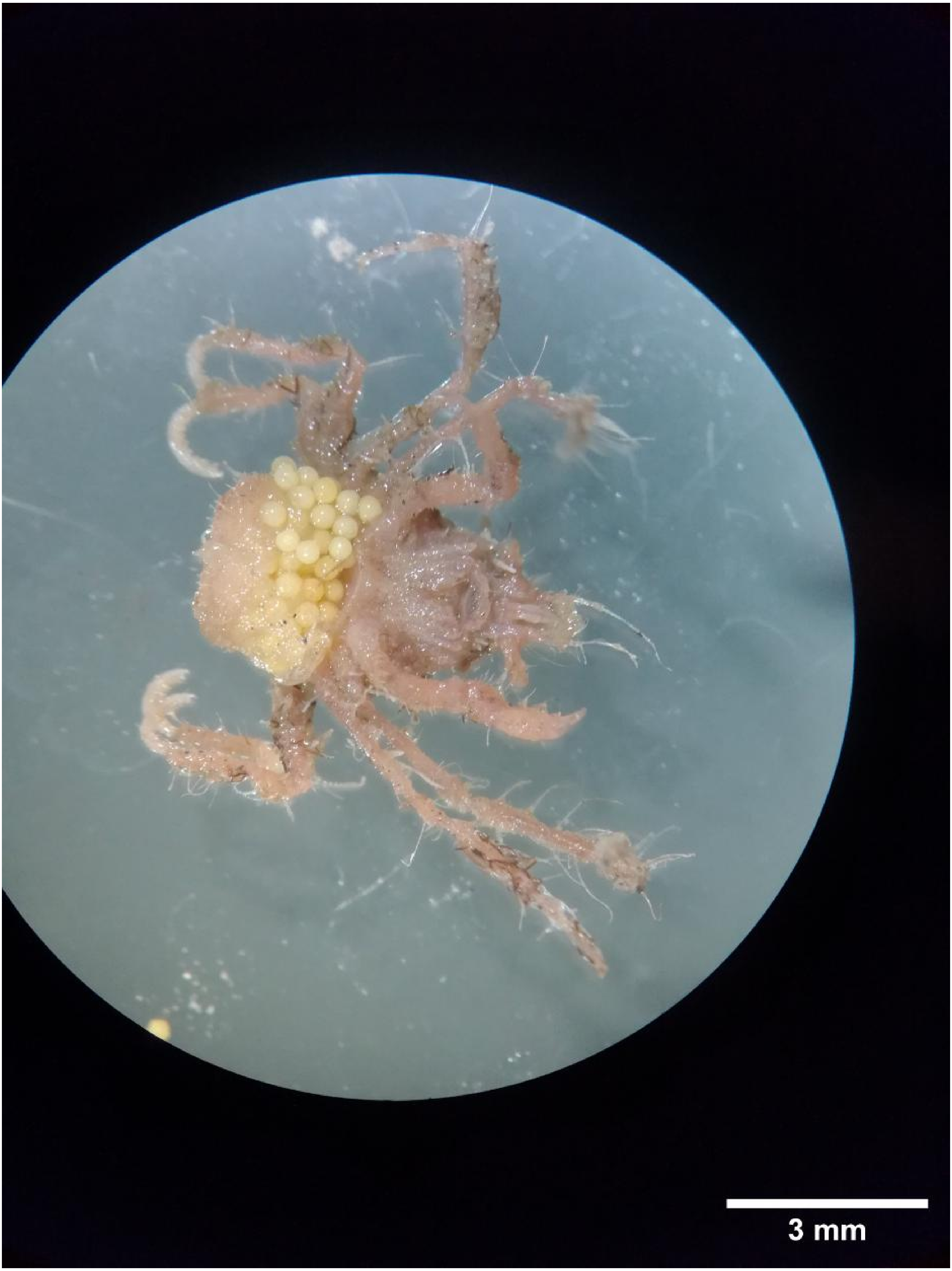
3 mm

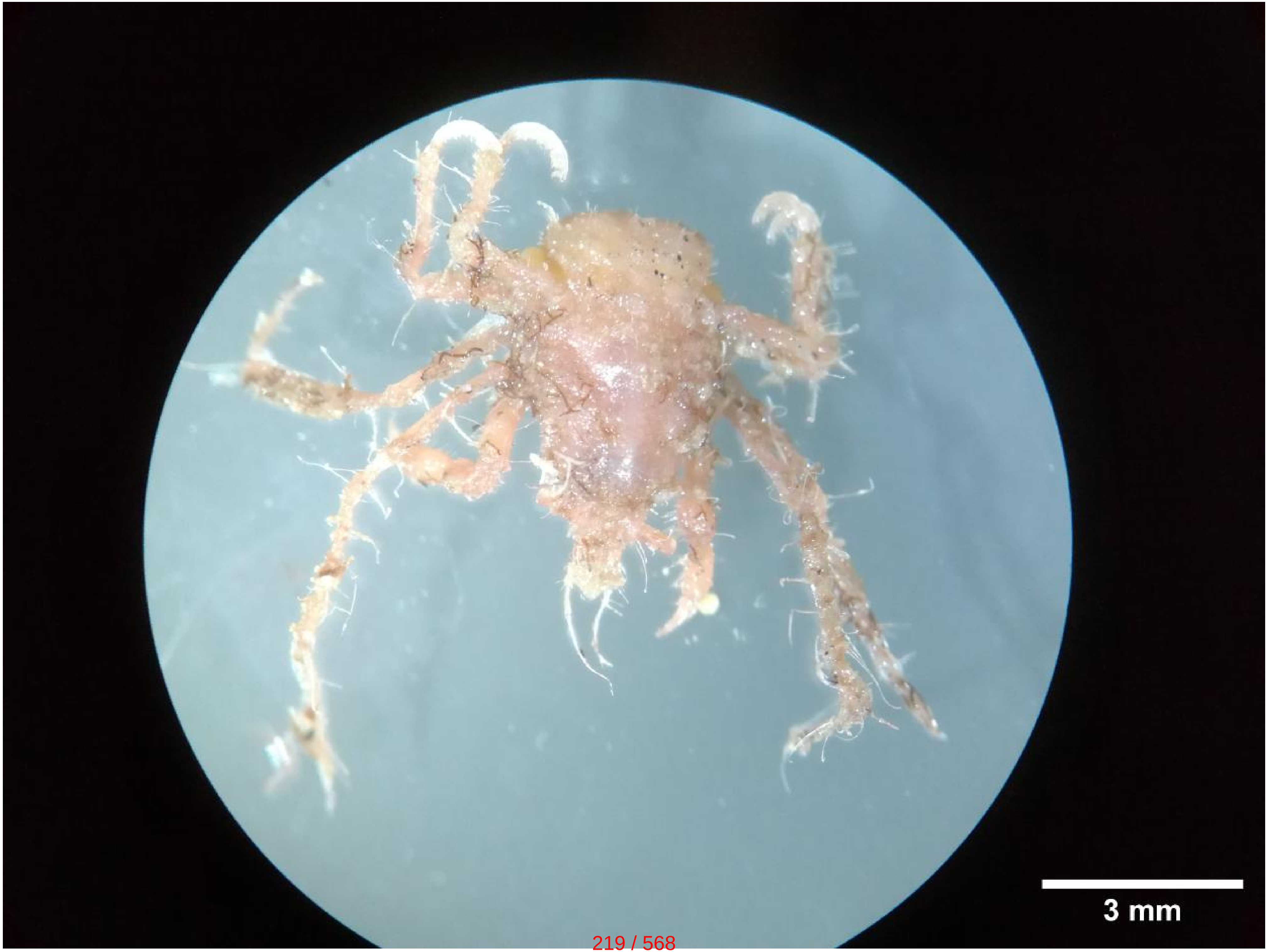
3 mm

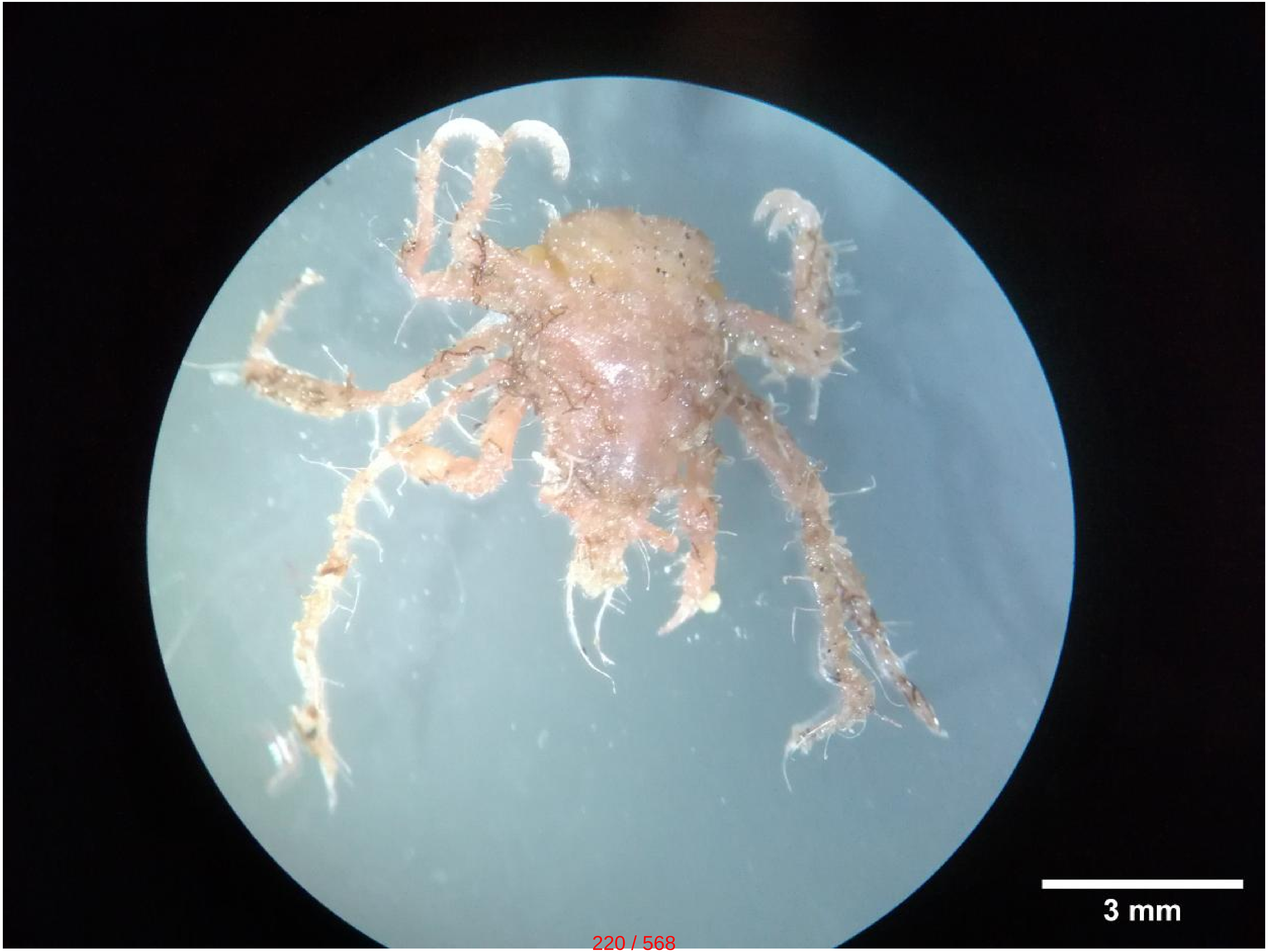
3 mm

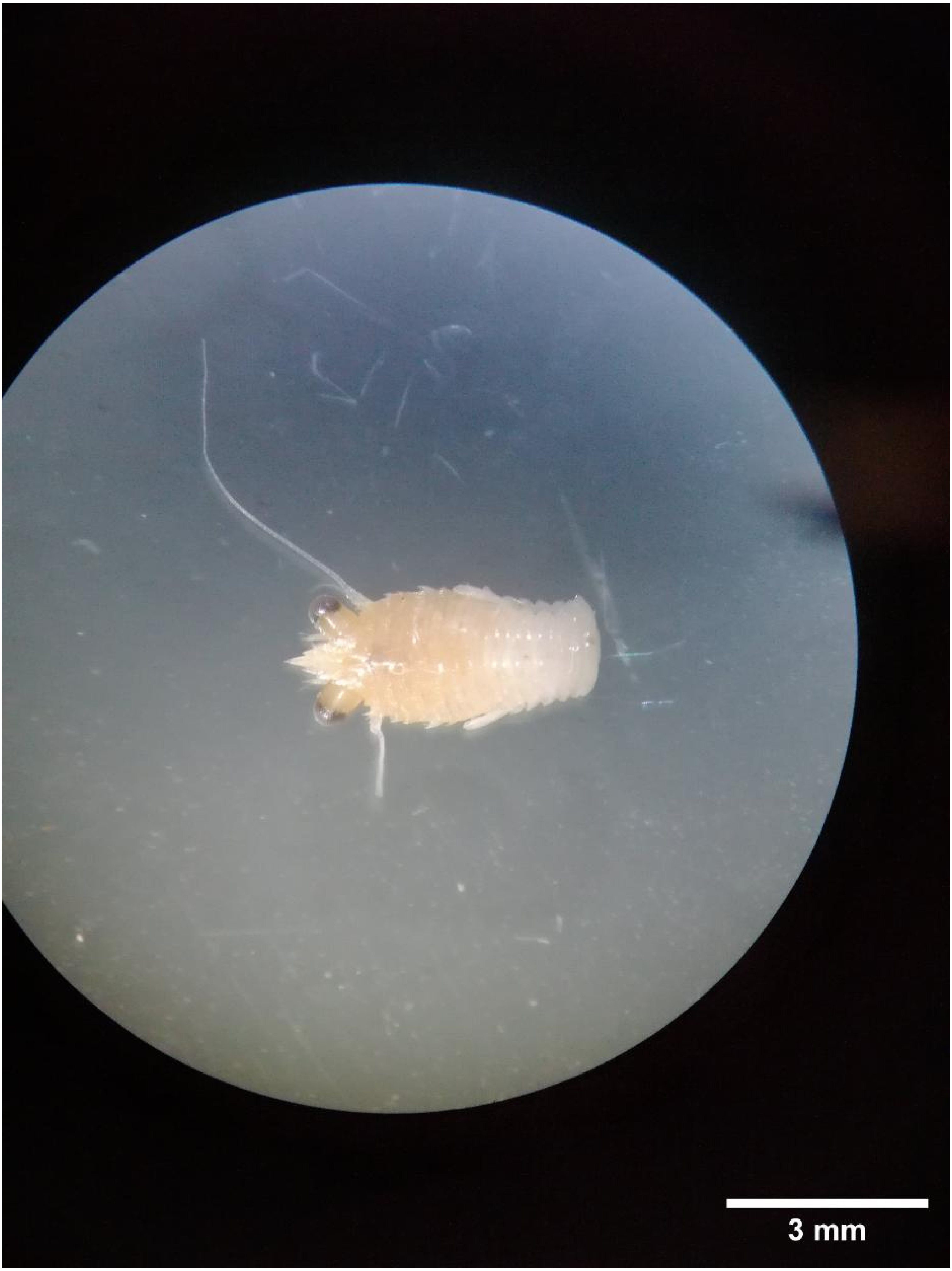
3 mm

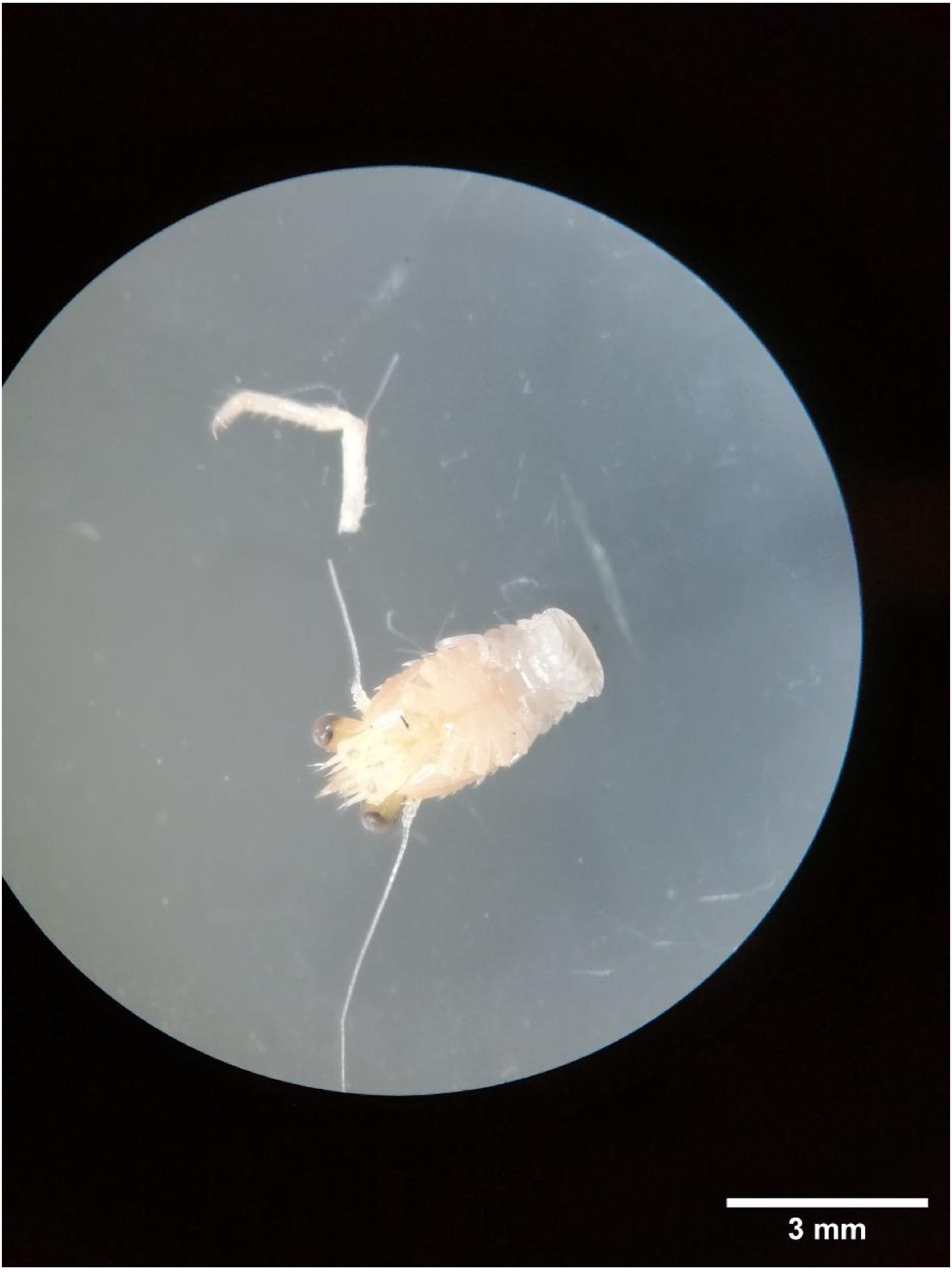
3 mm

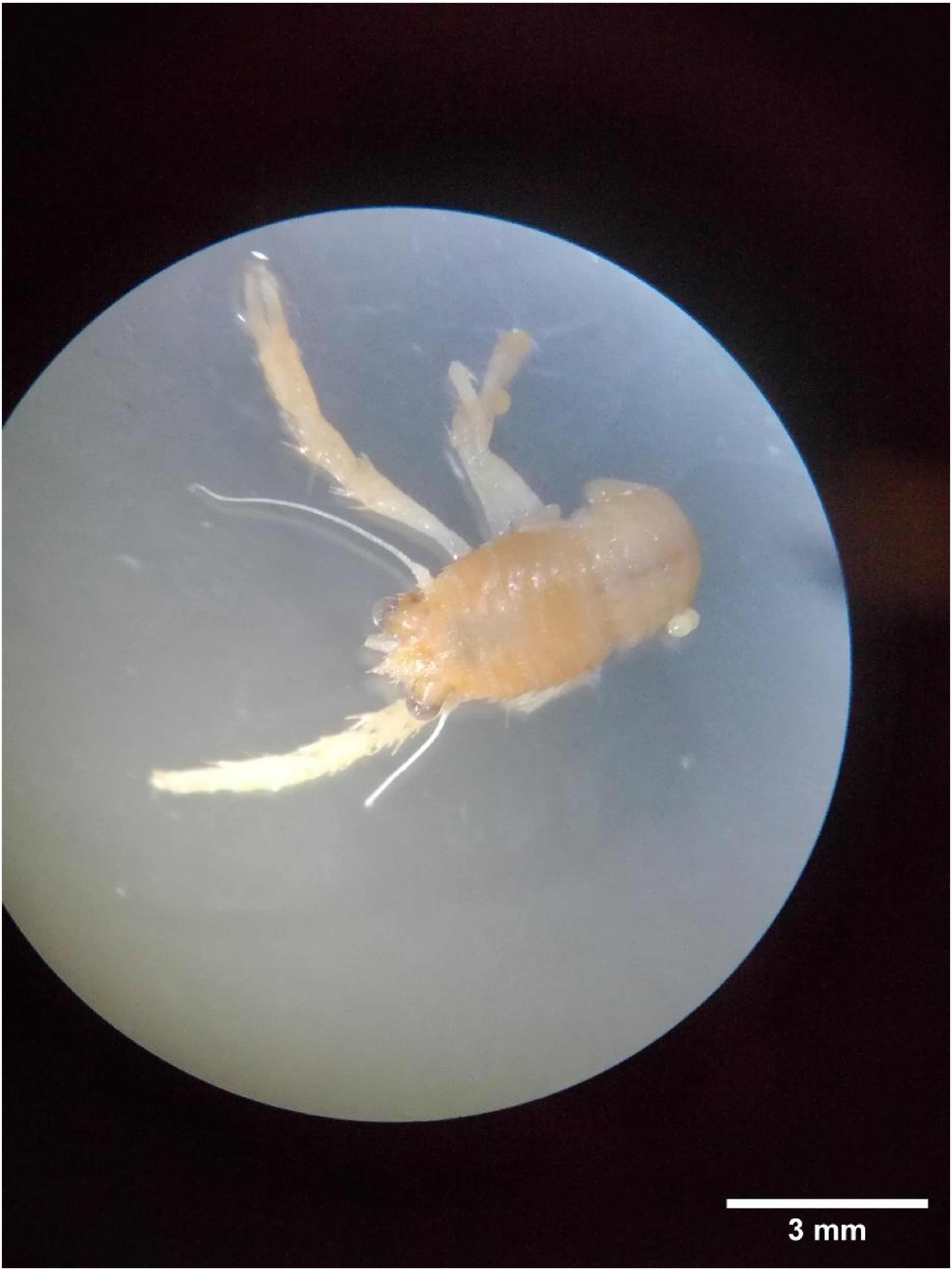
3 mm

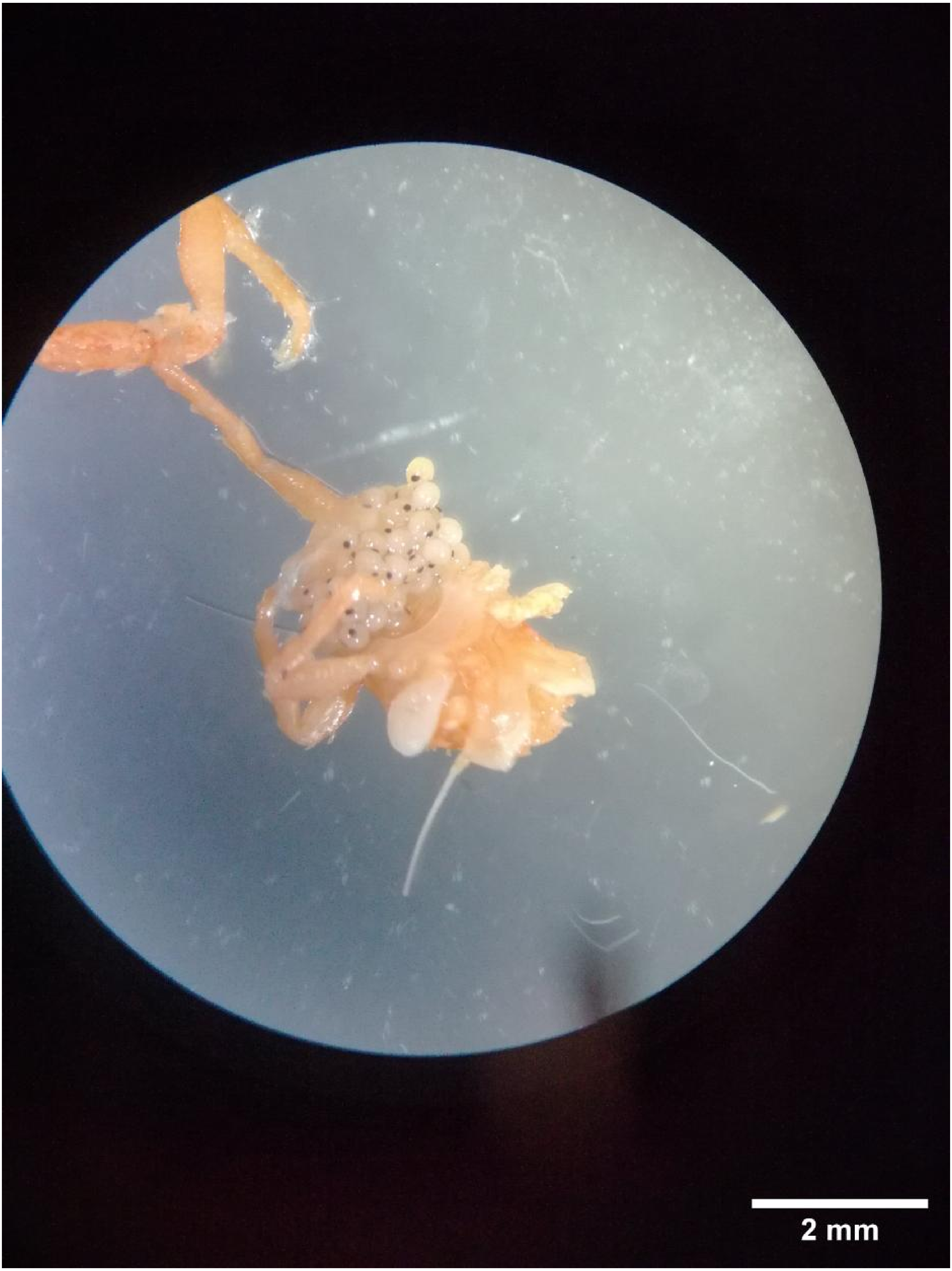
2 mm

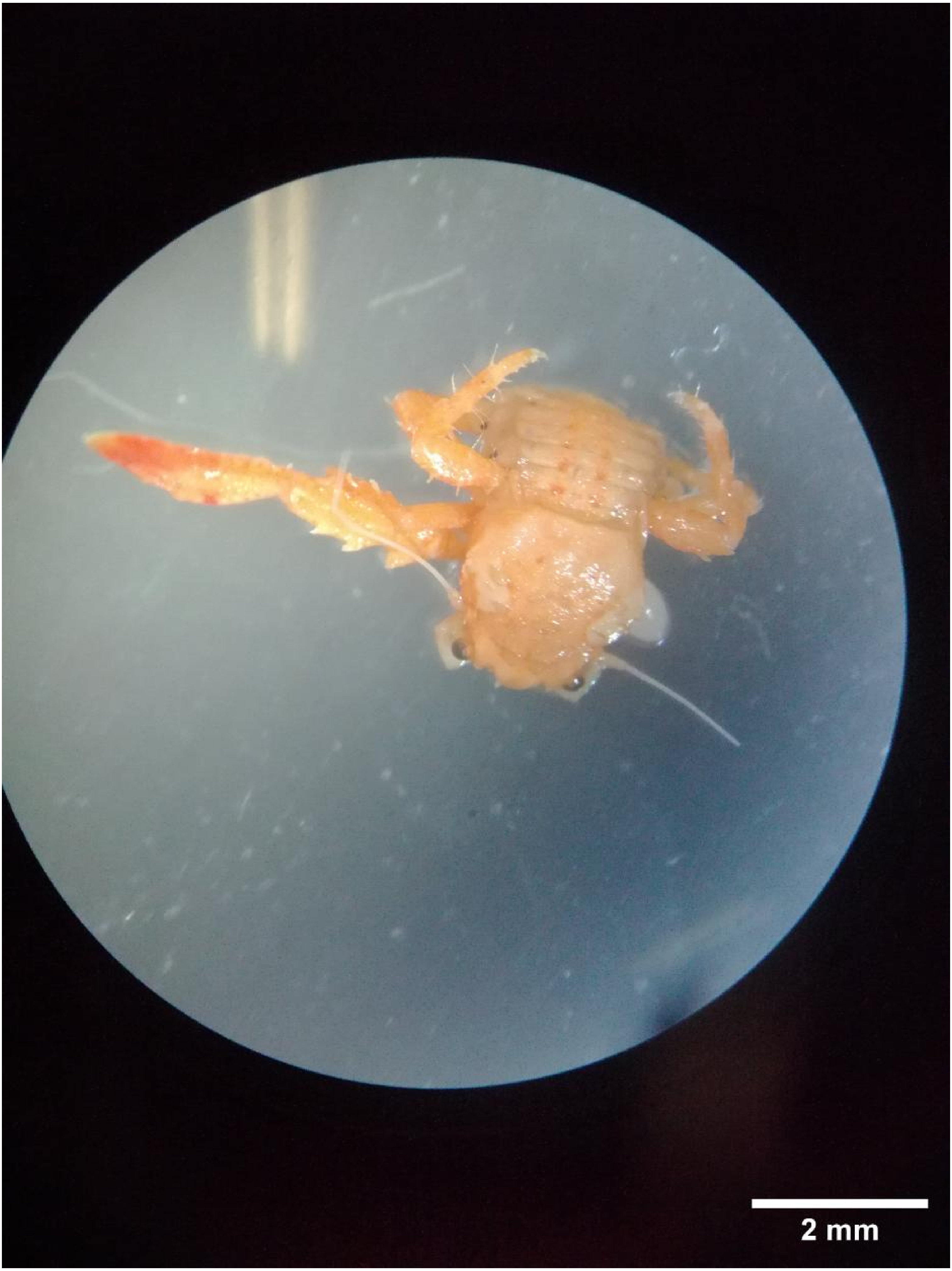
2 mm

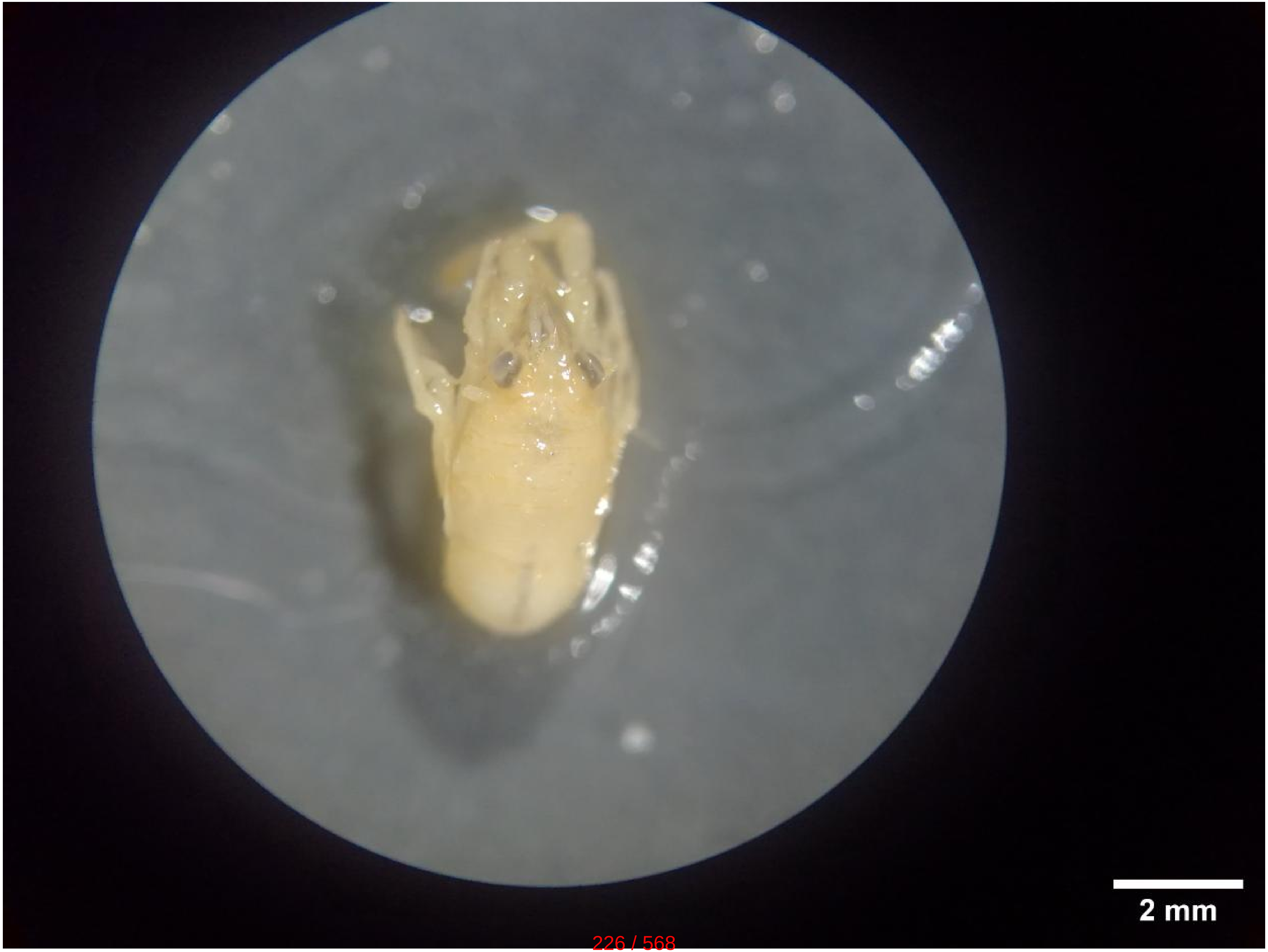
2 mm

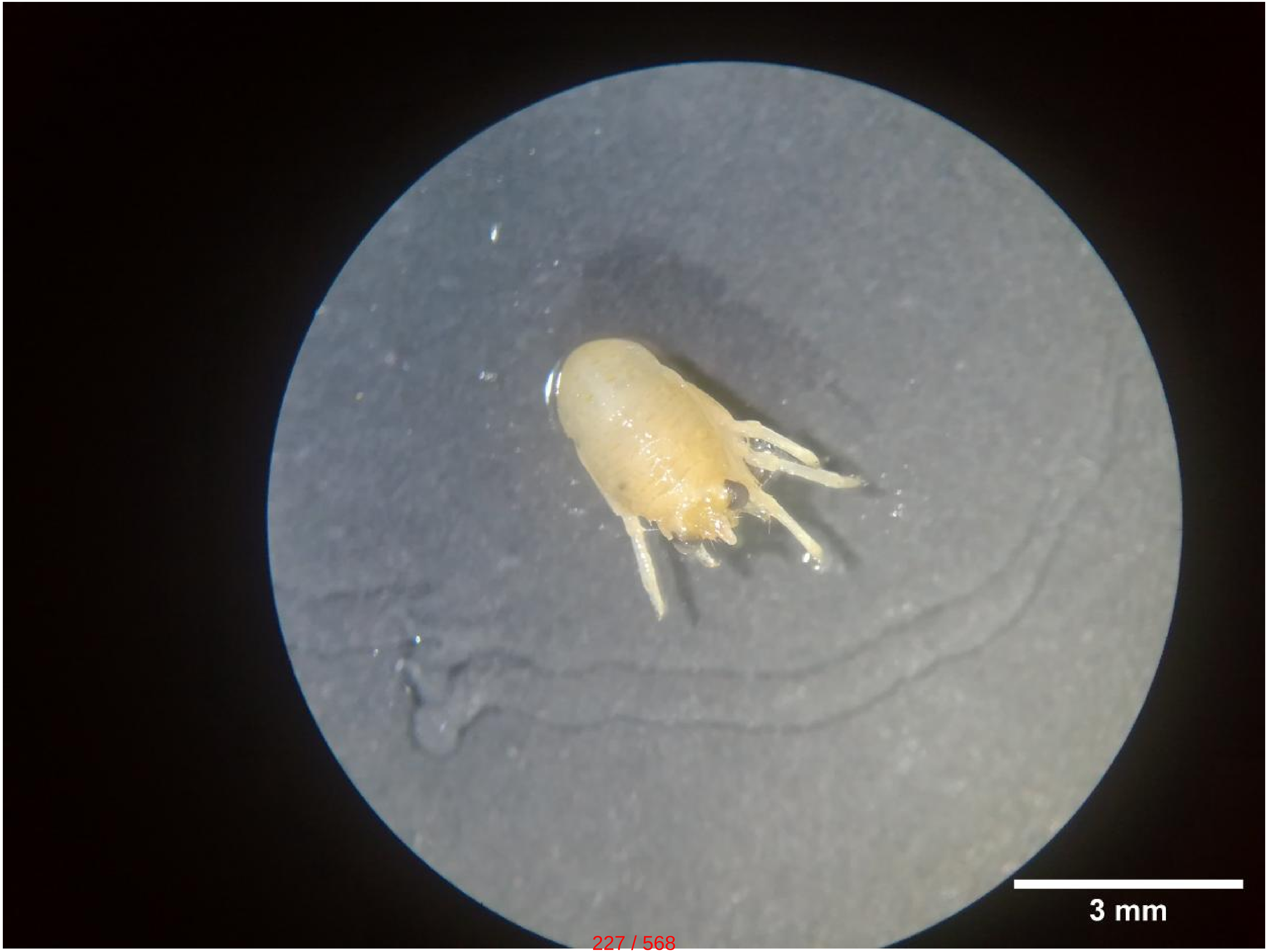
3 mm

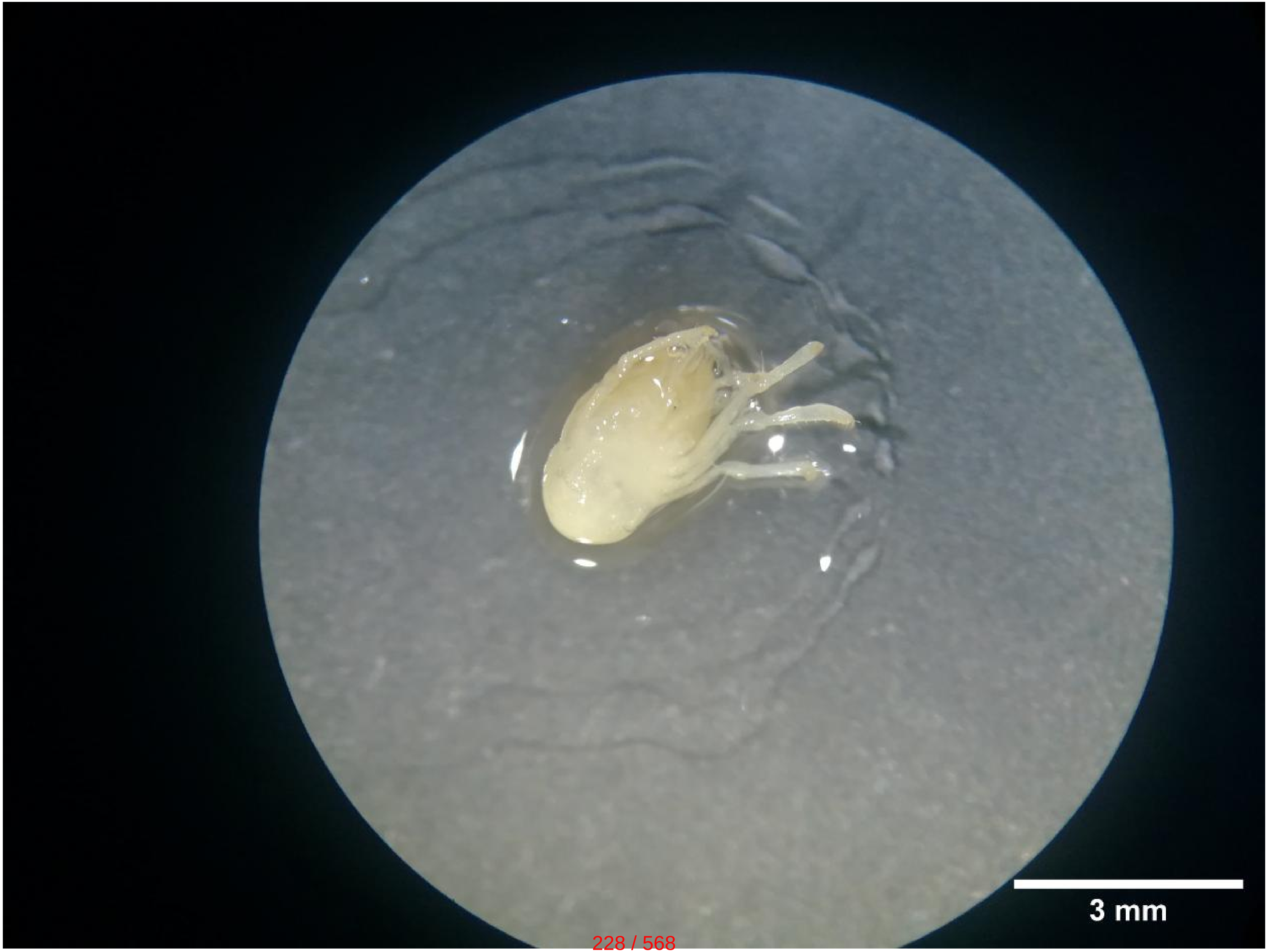
3 mm

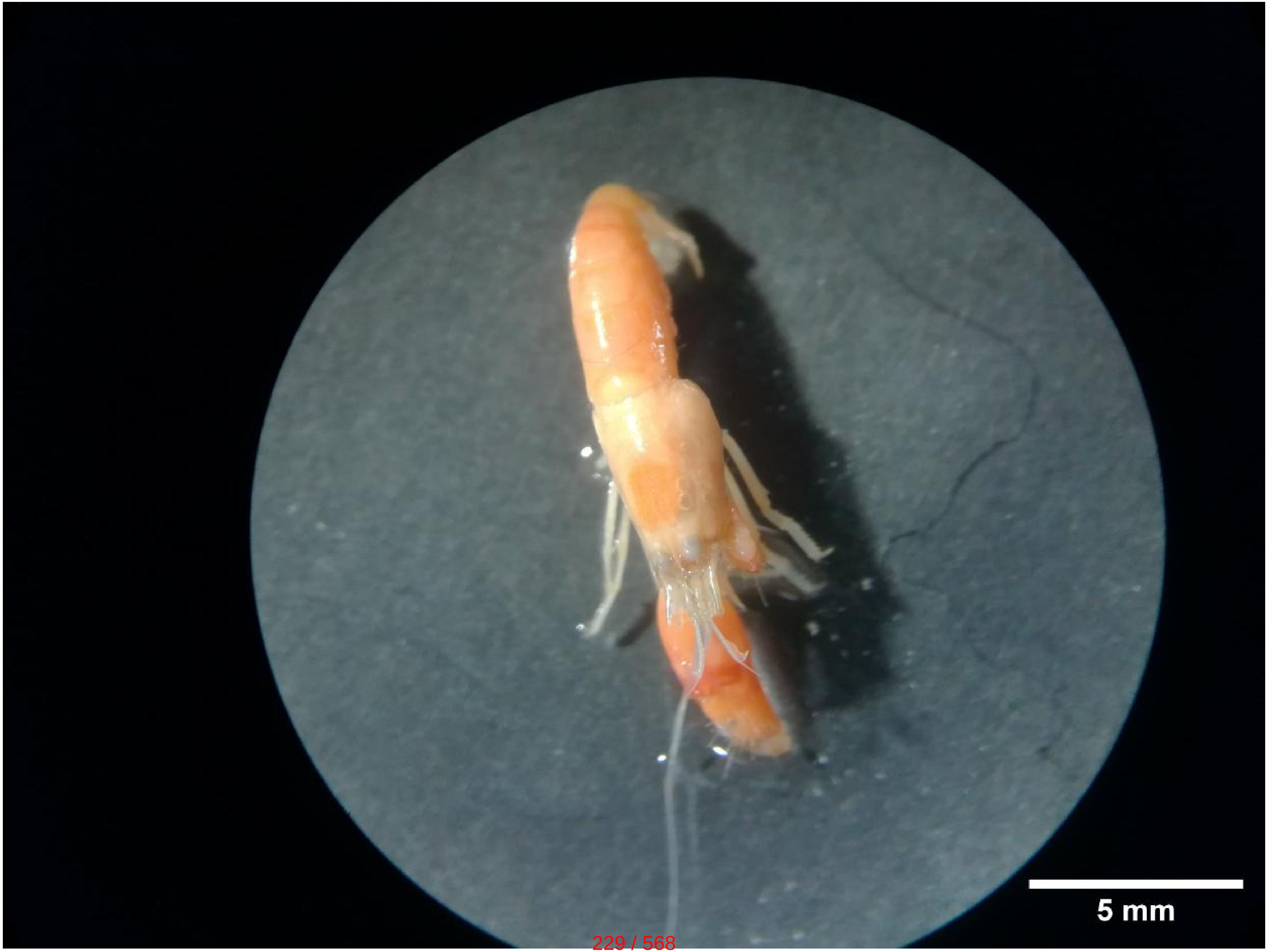
5 mm

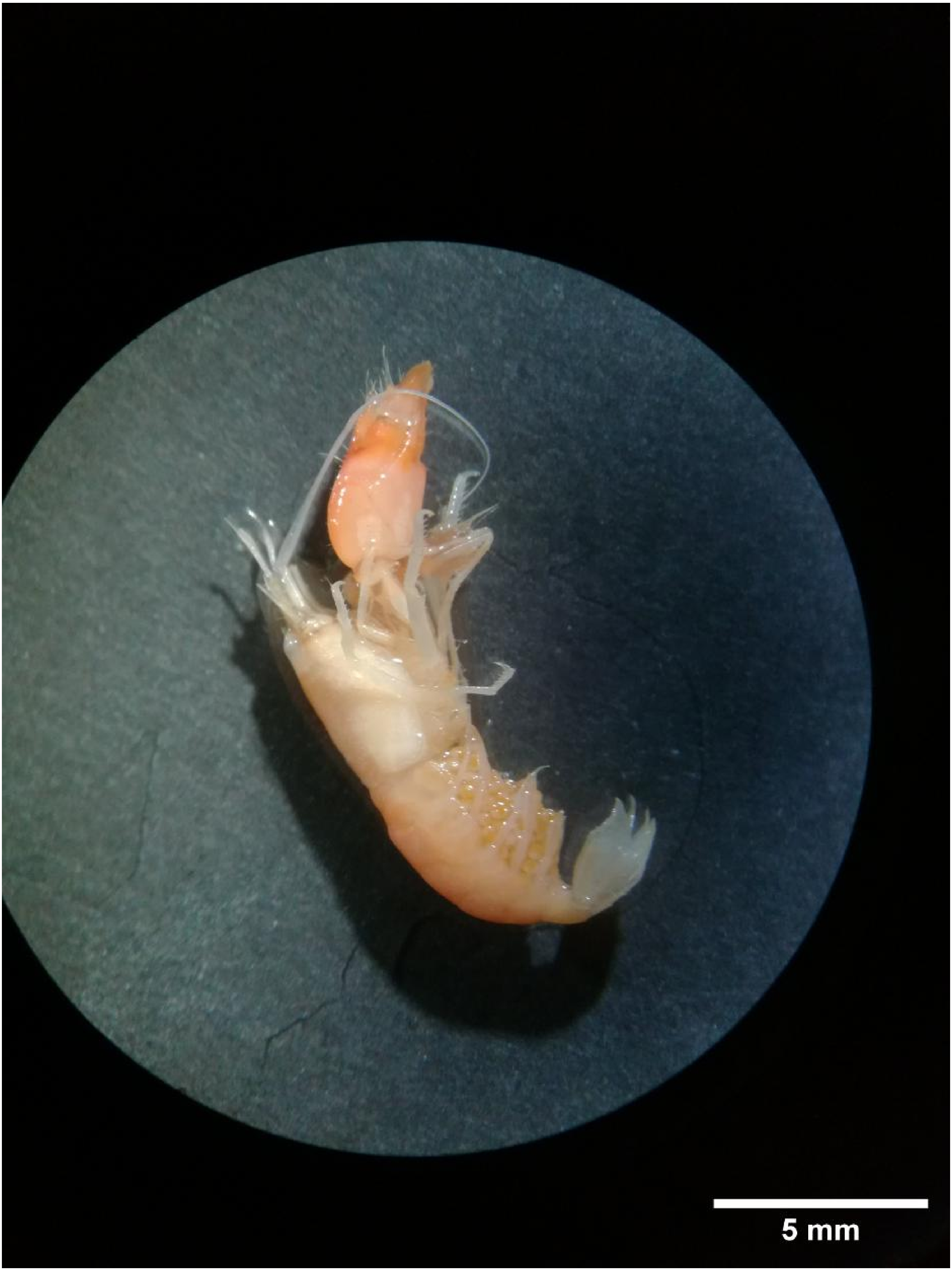
5 mm

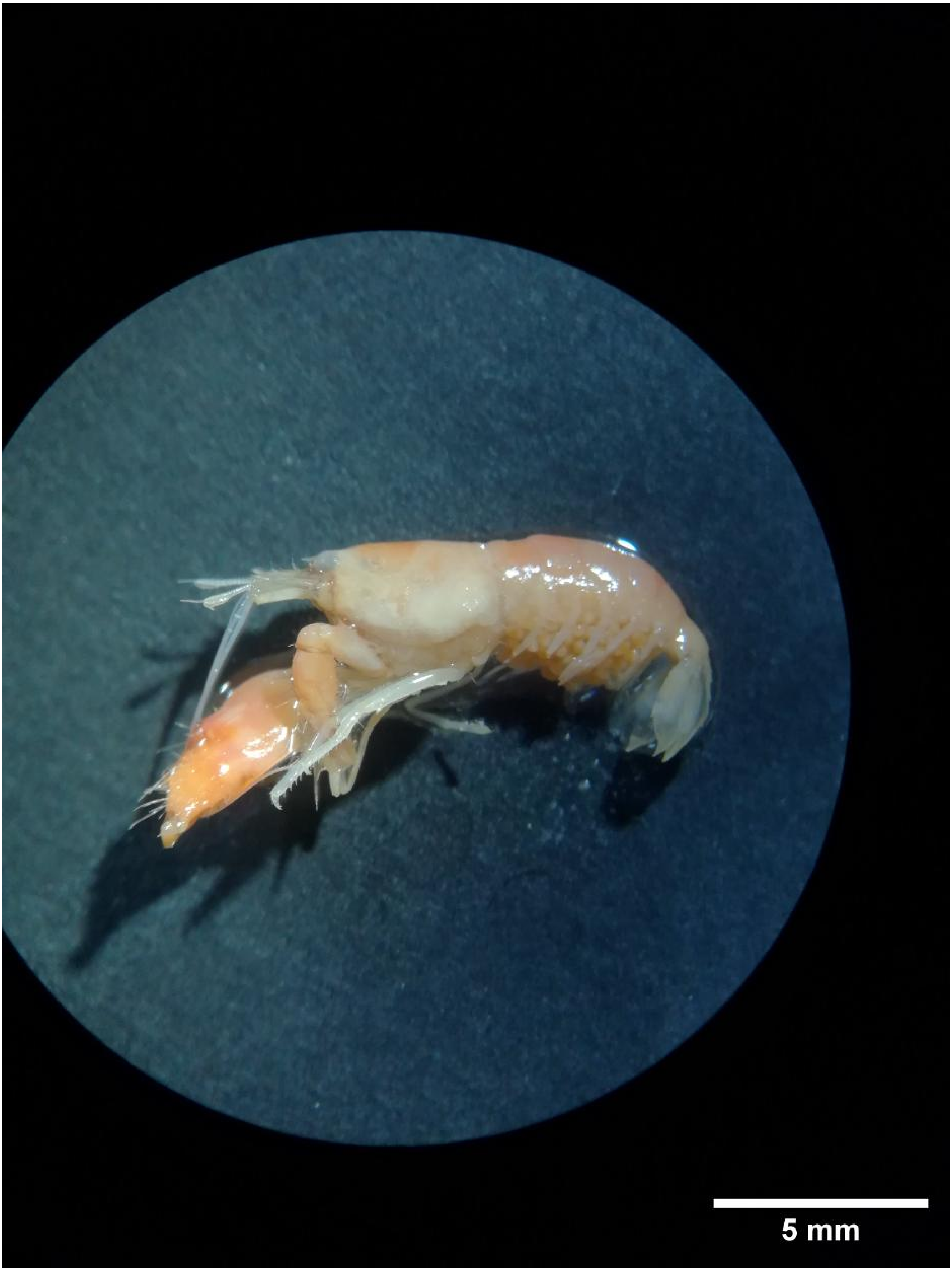
5 mm

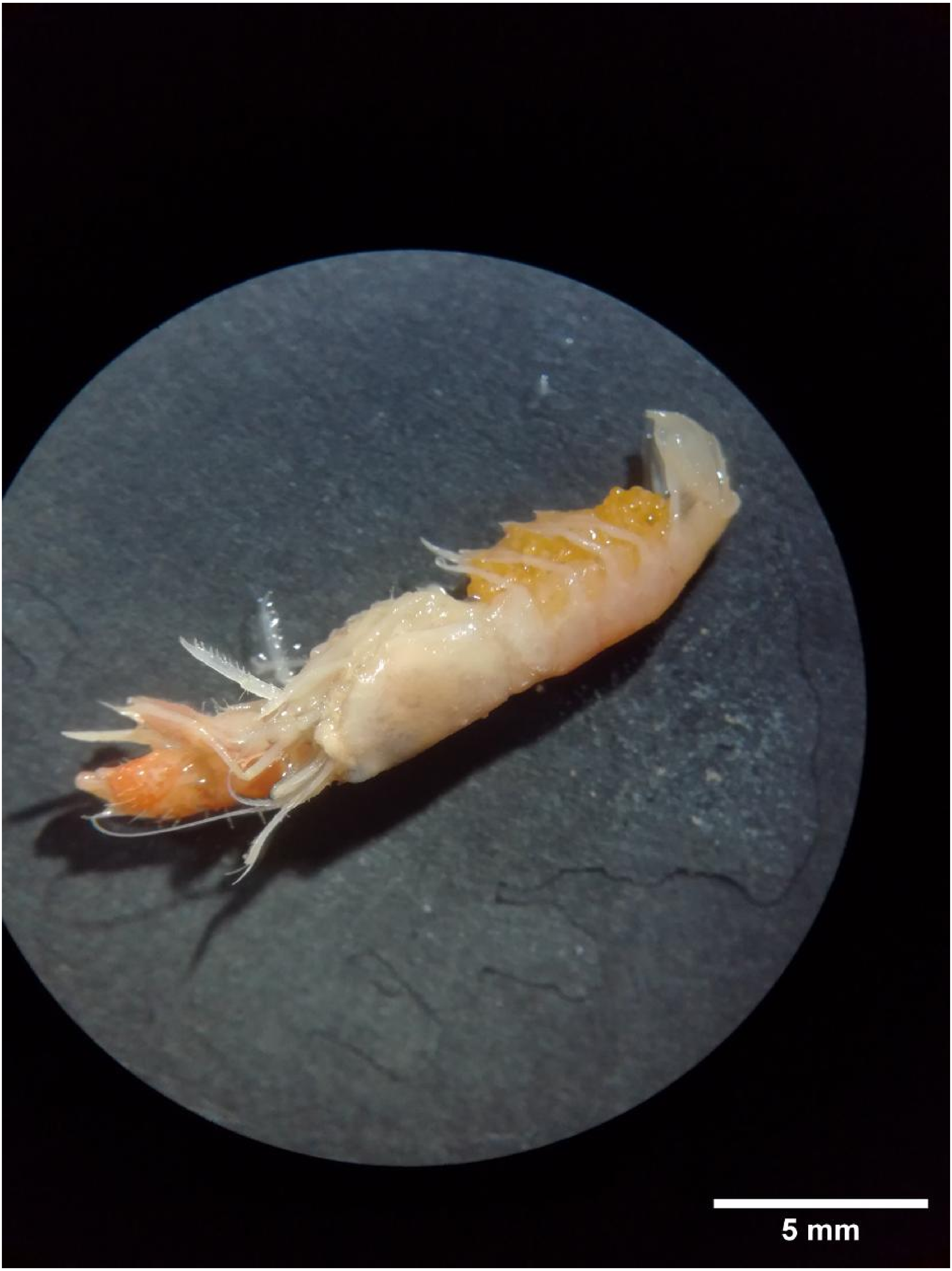
5 mm

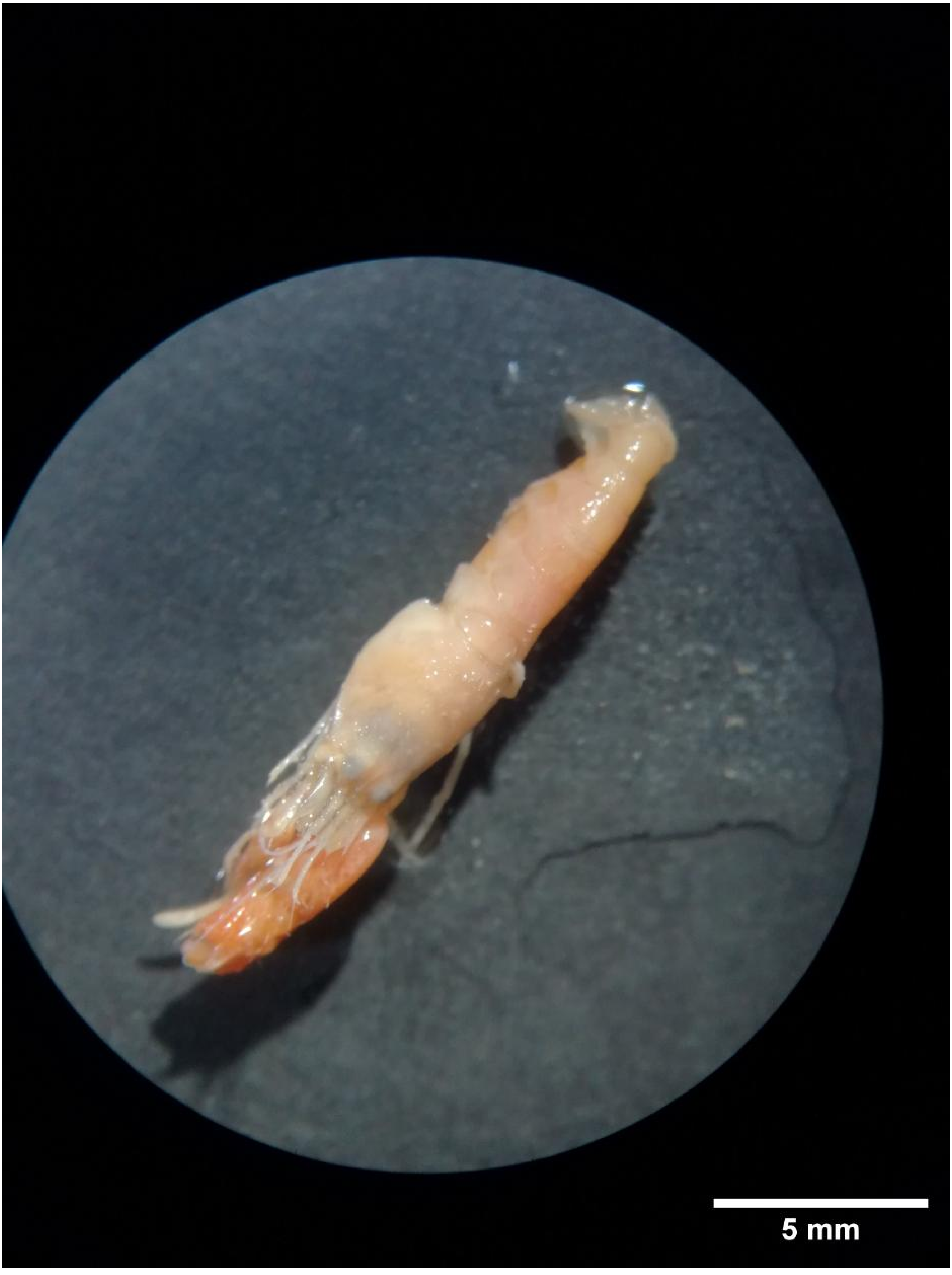
5 mm

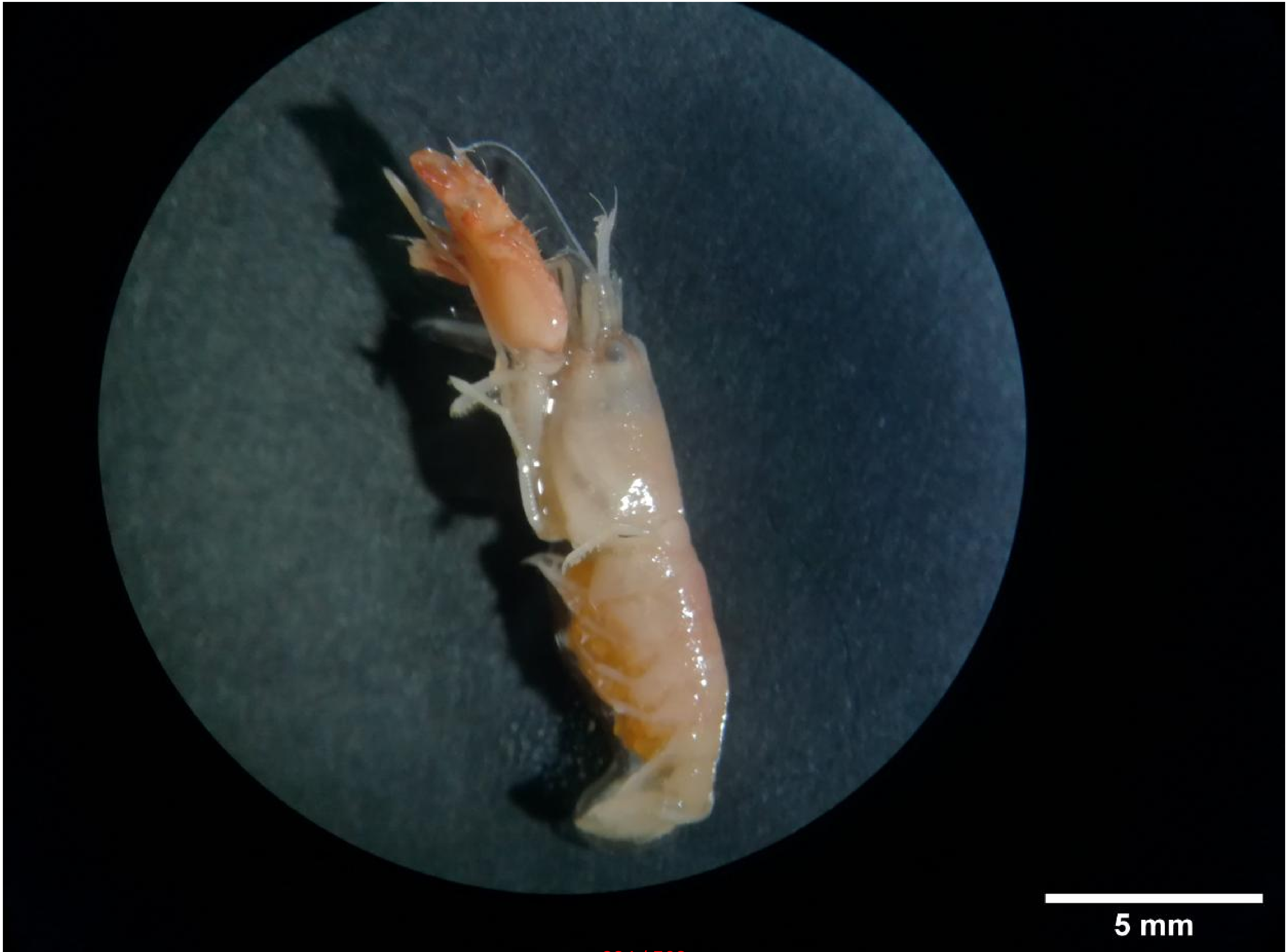
5 mm

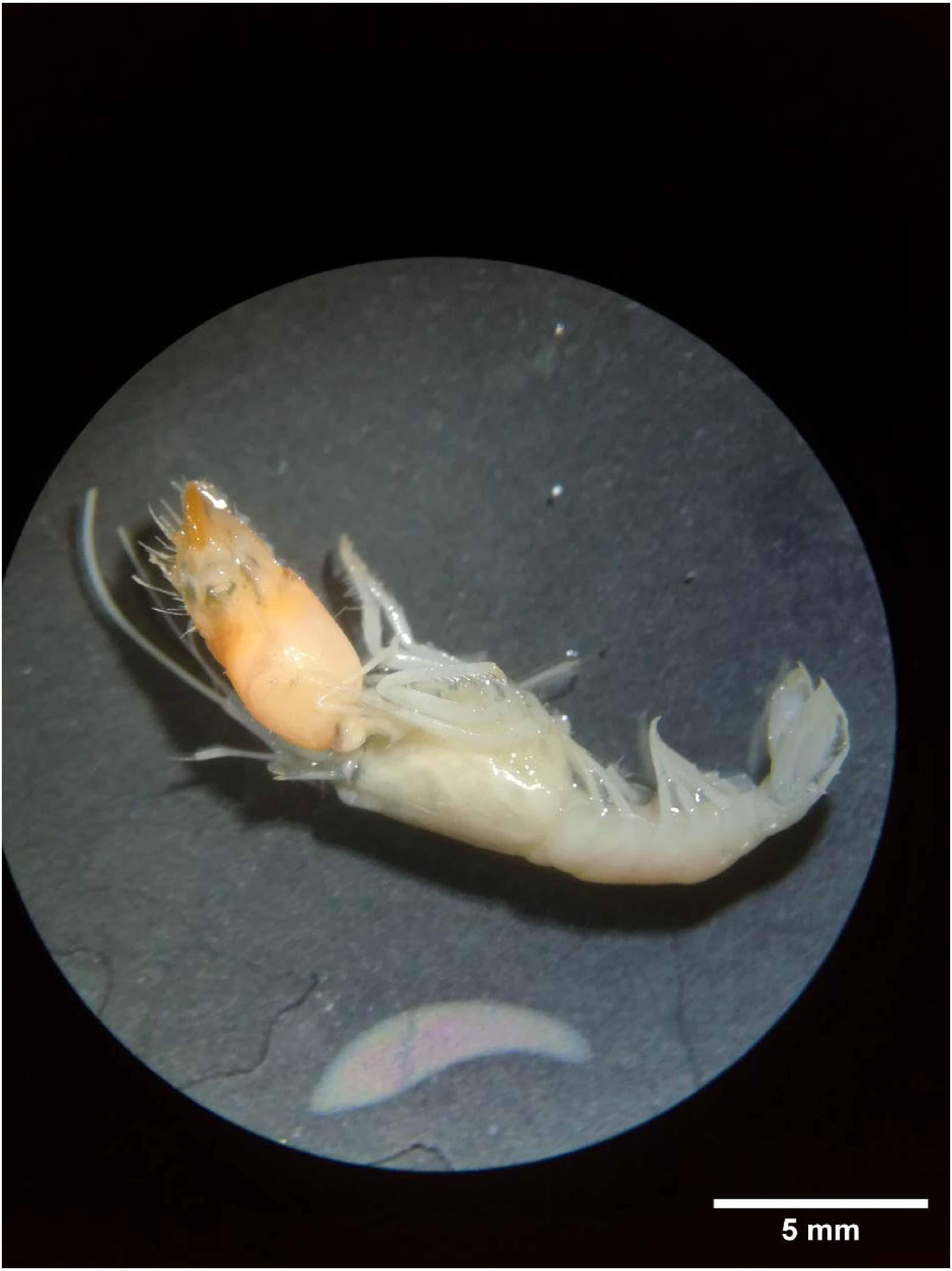
5 mm

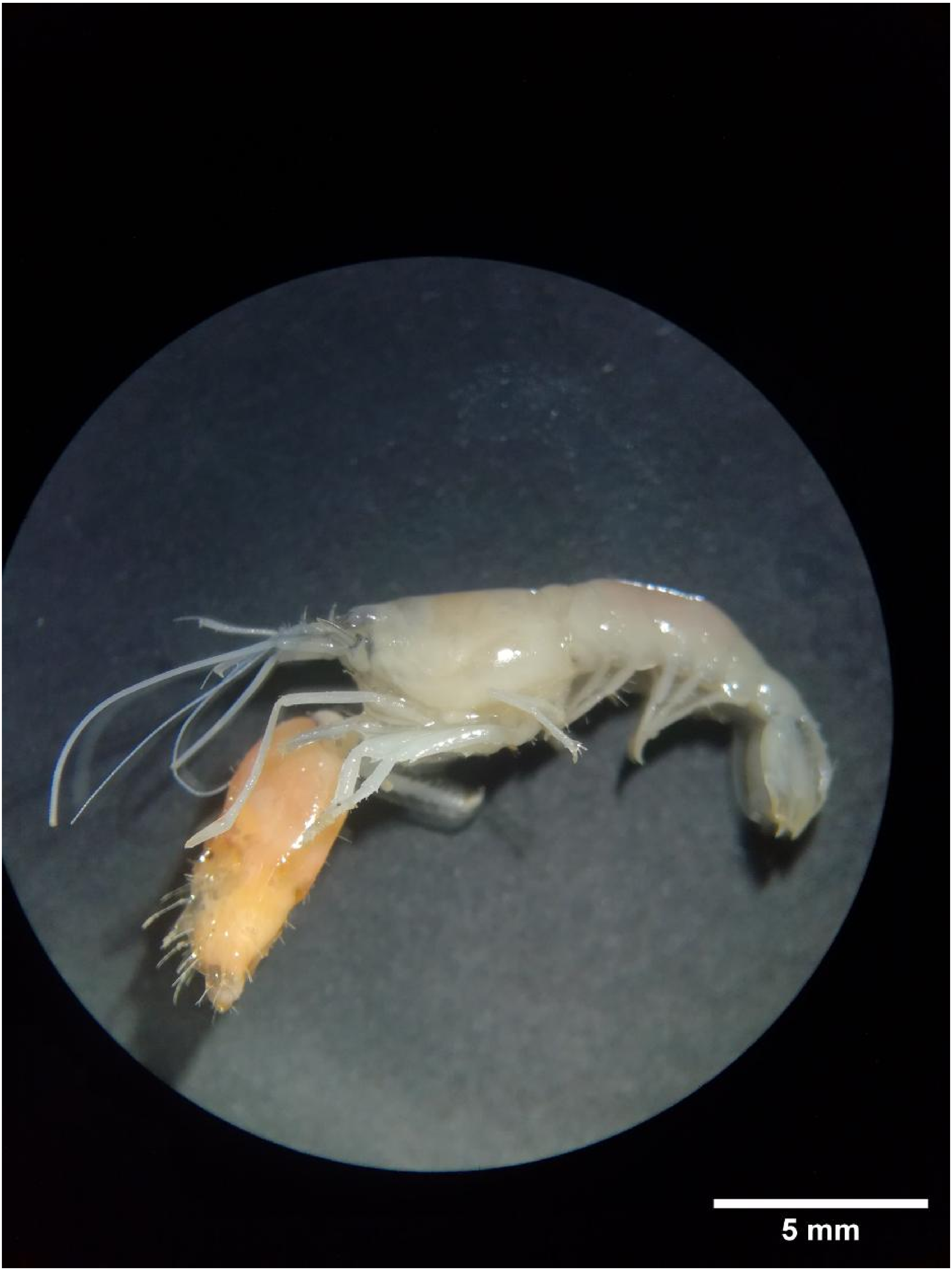
5 mm

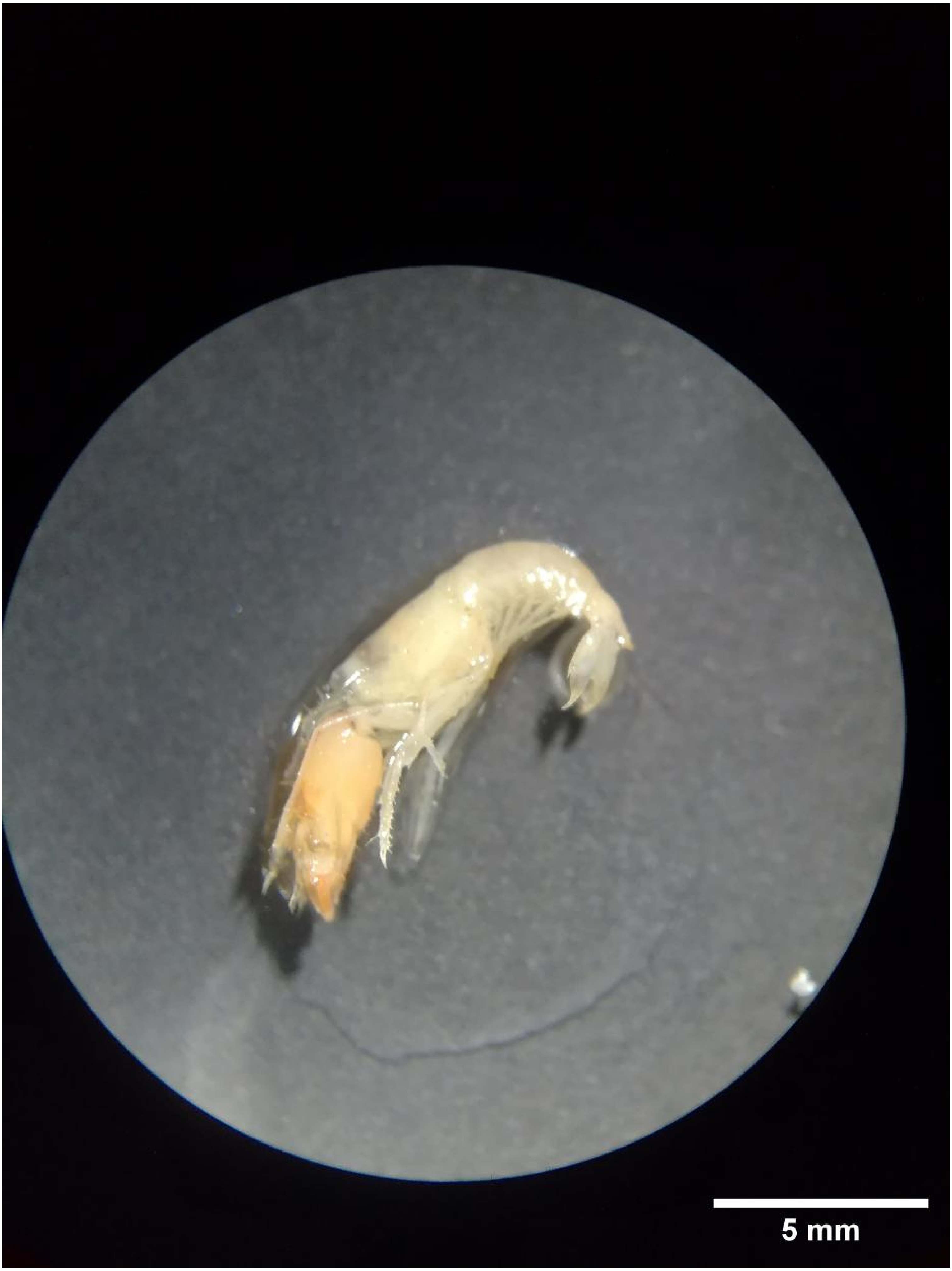
5 mm

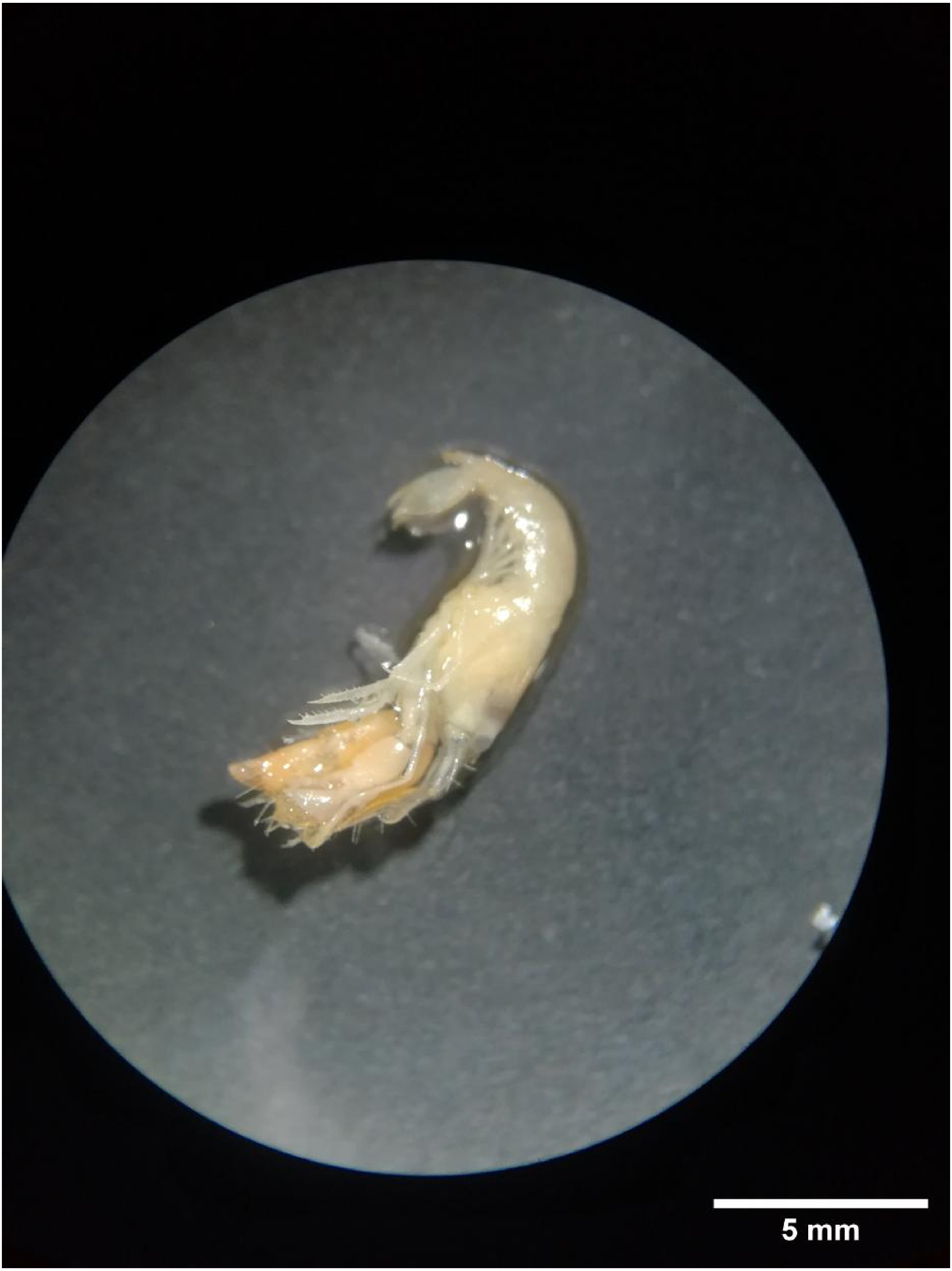
5 mm

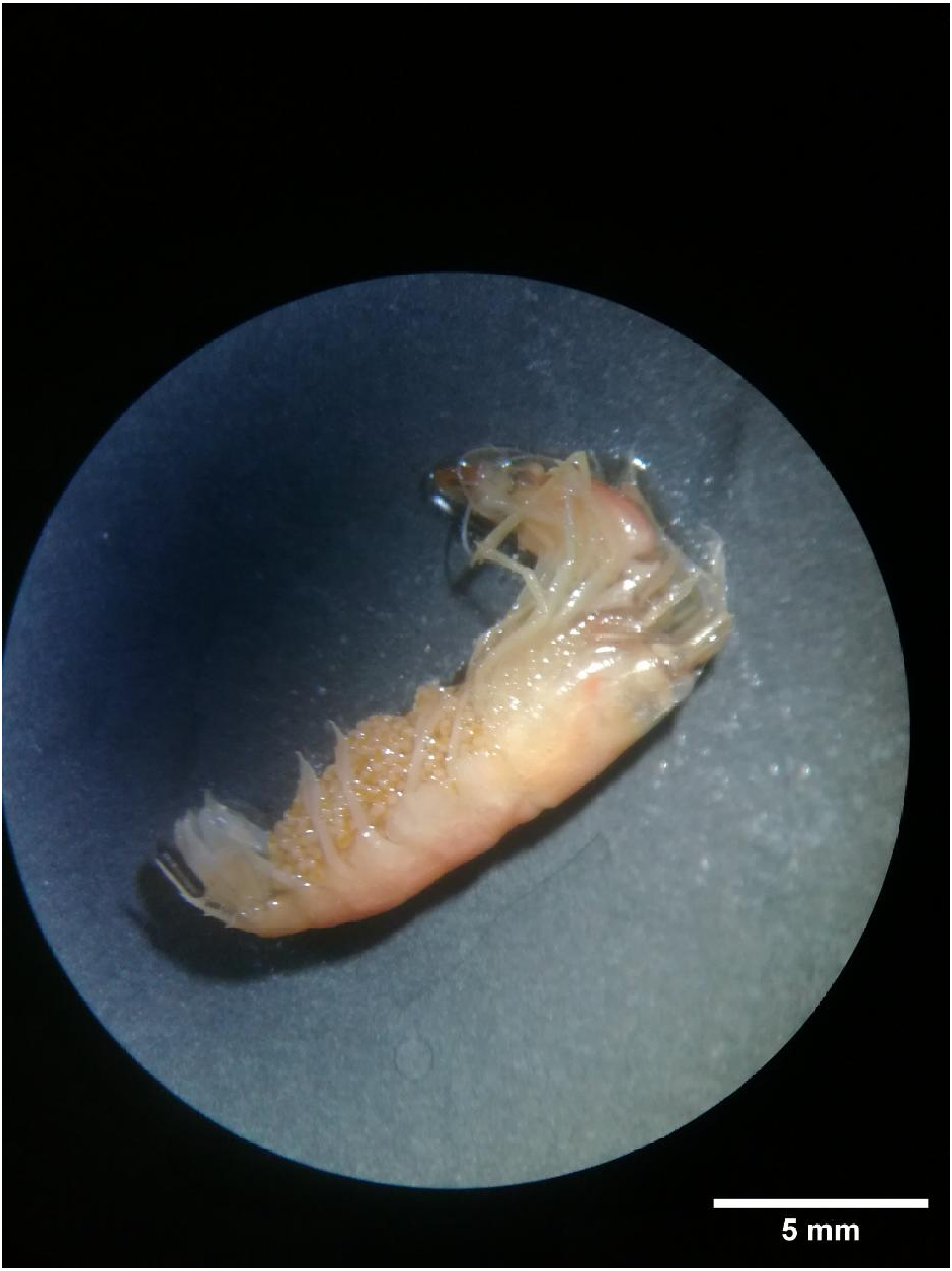
5 mm

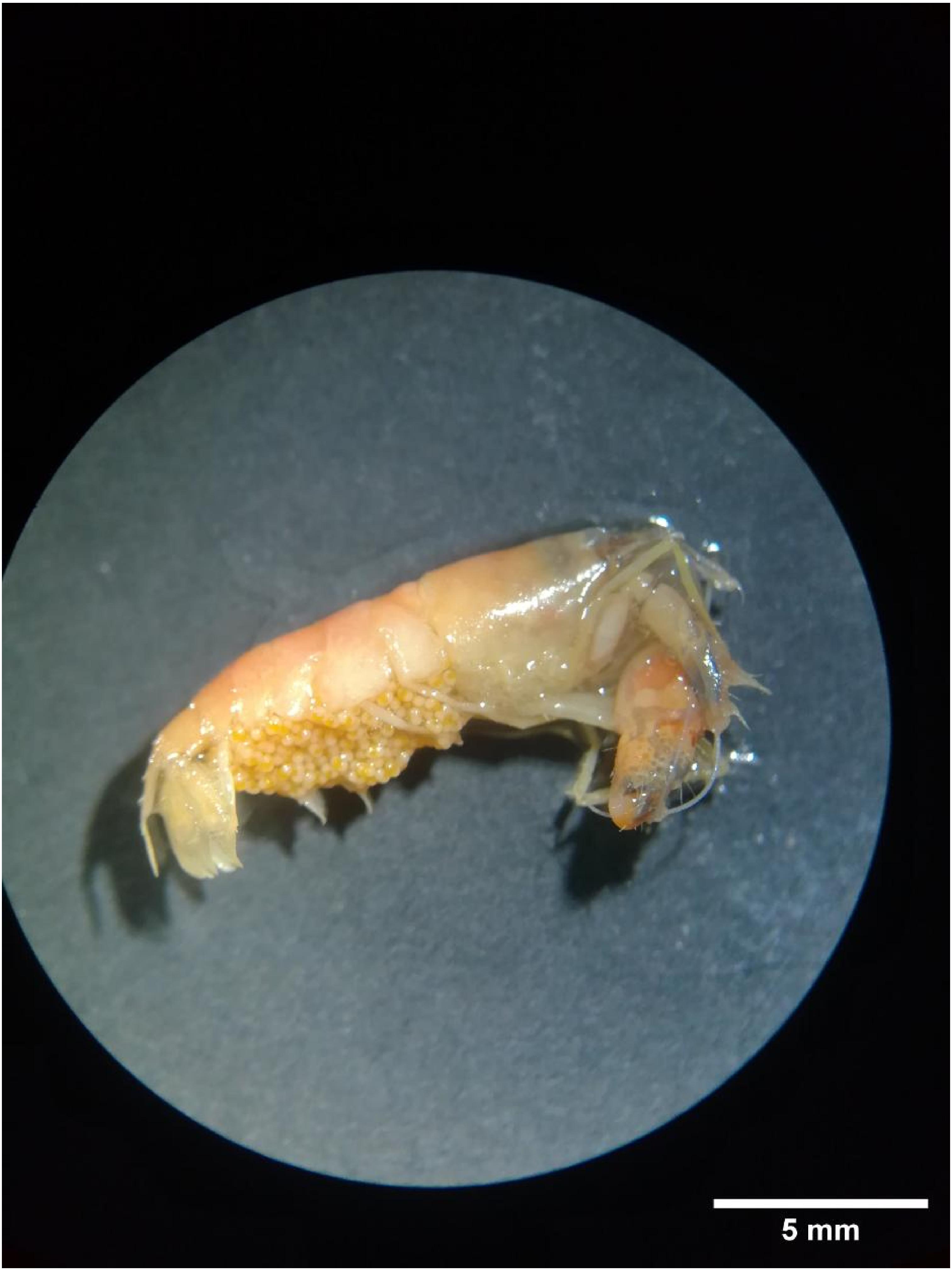
5 mm

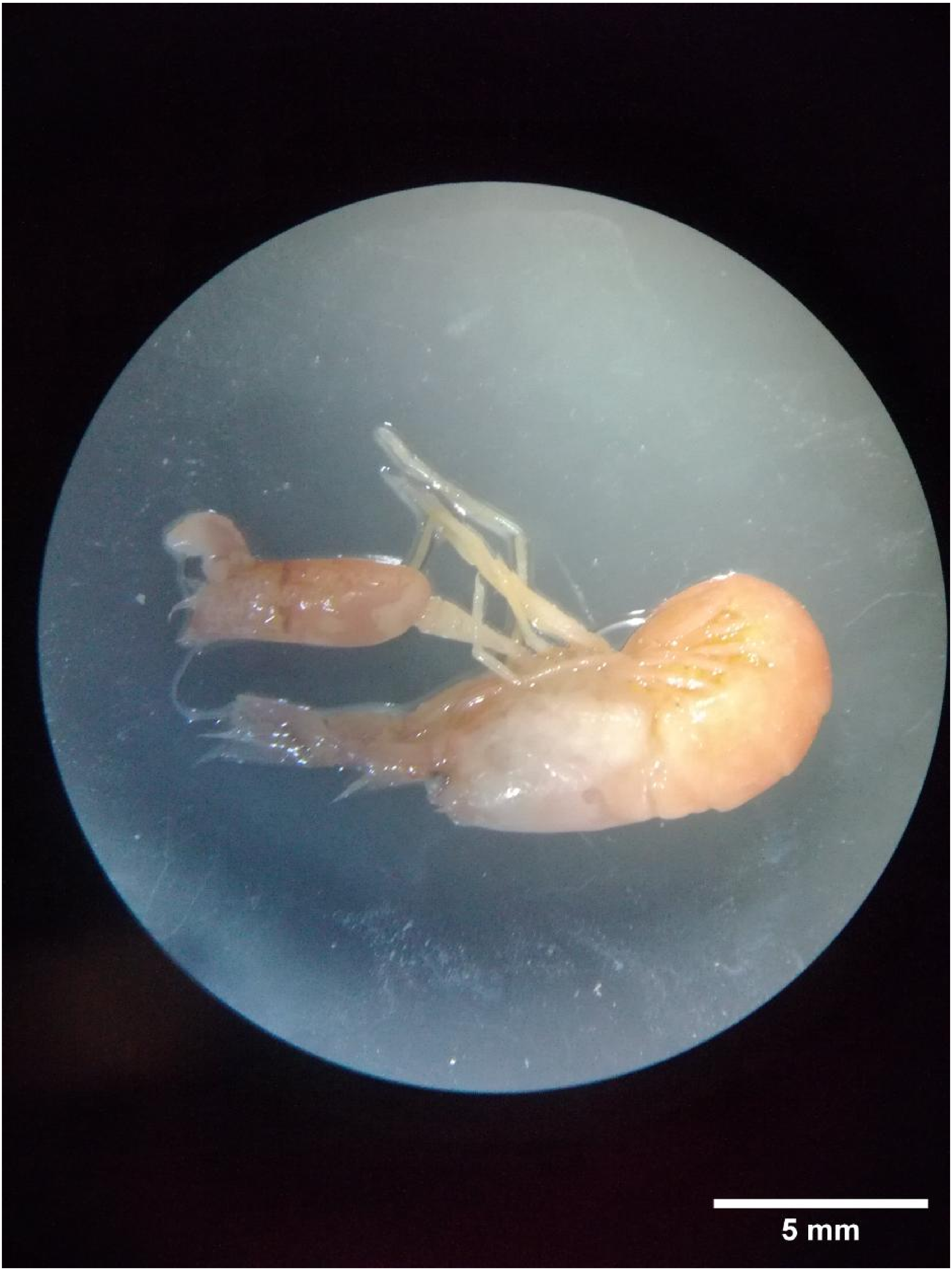
5 mm

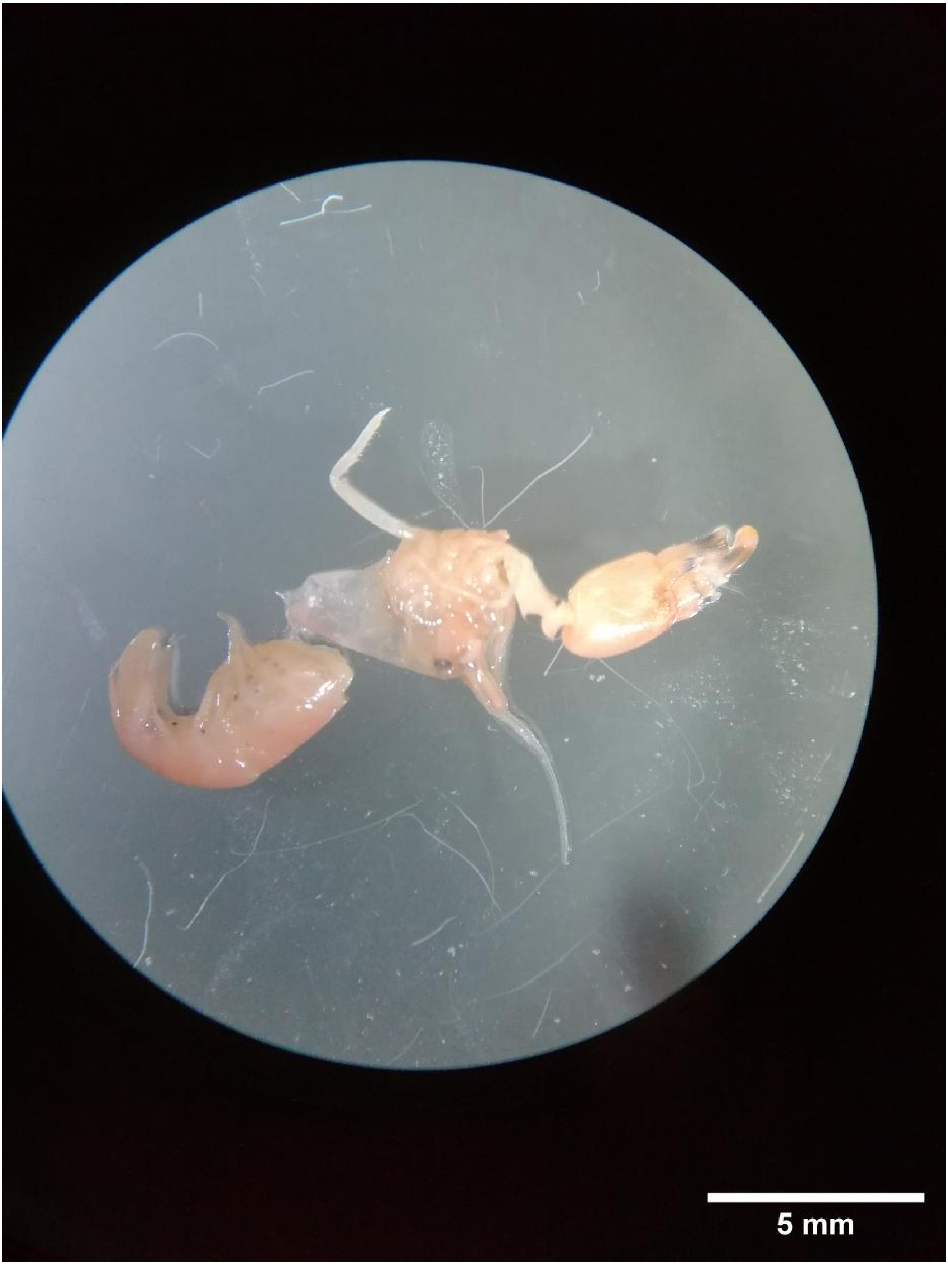
5 mm

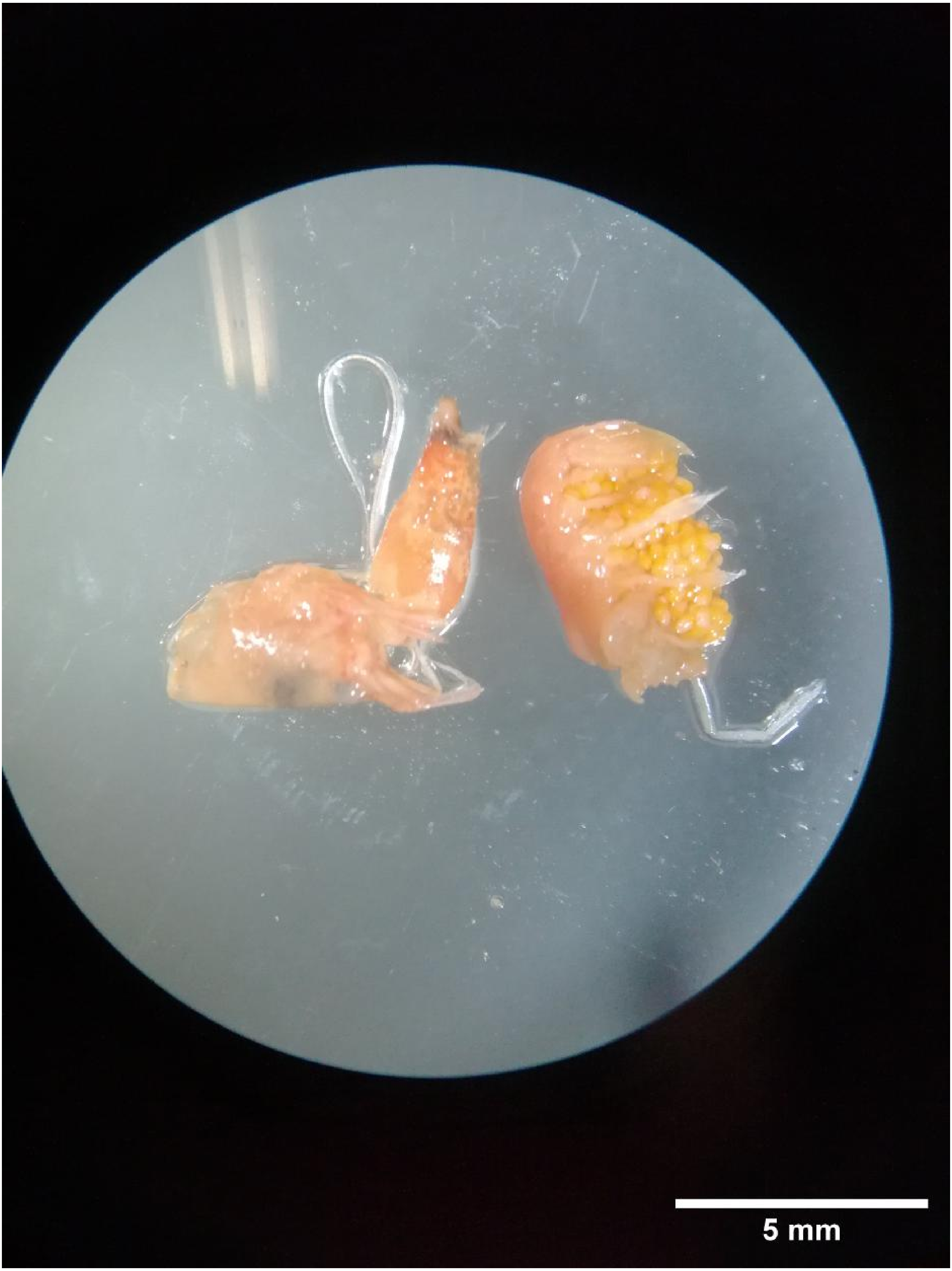
5 mm

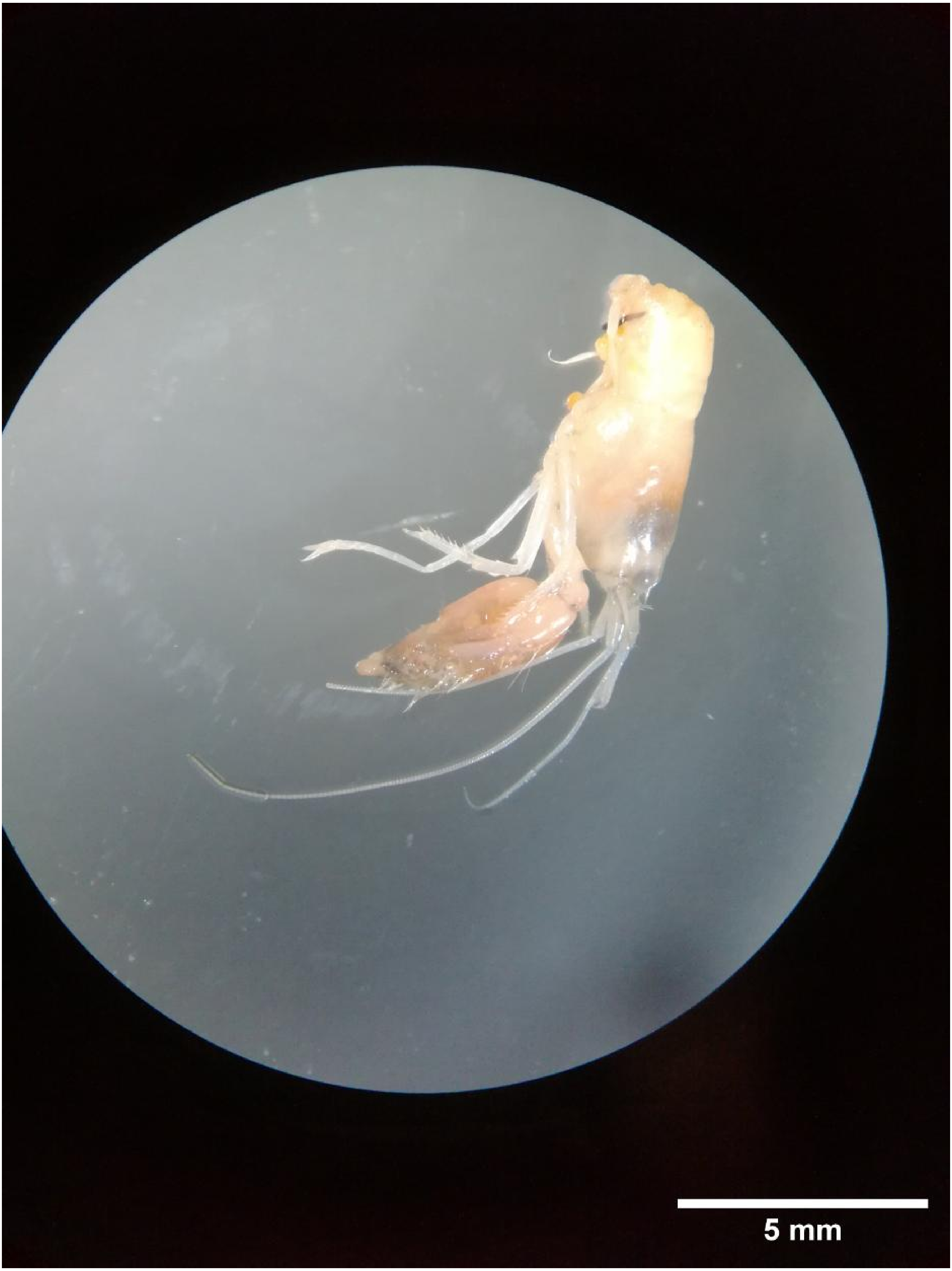
5 mm

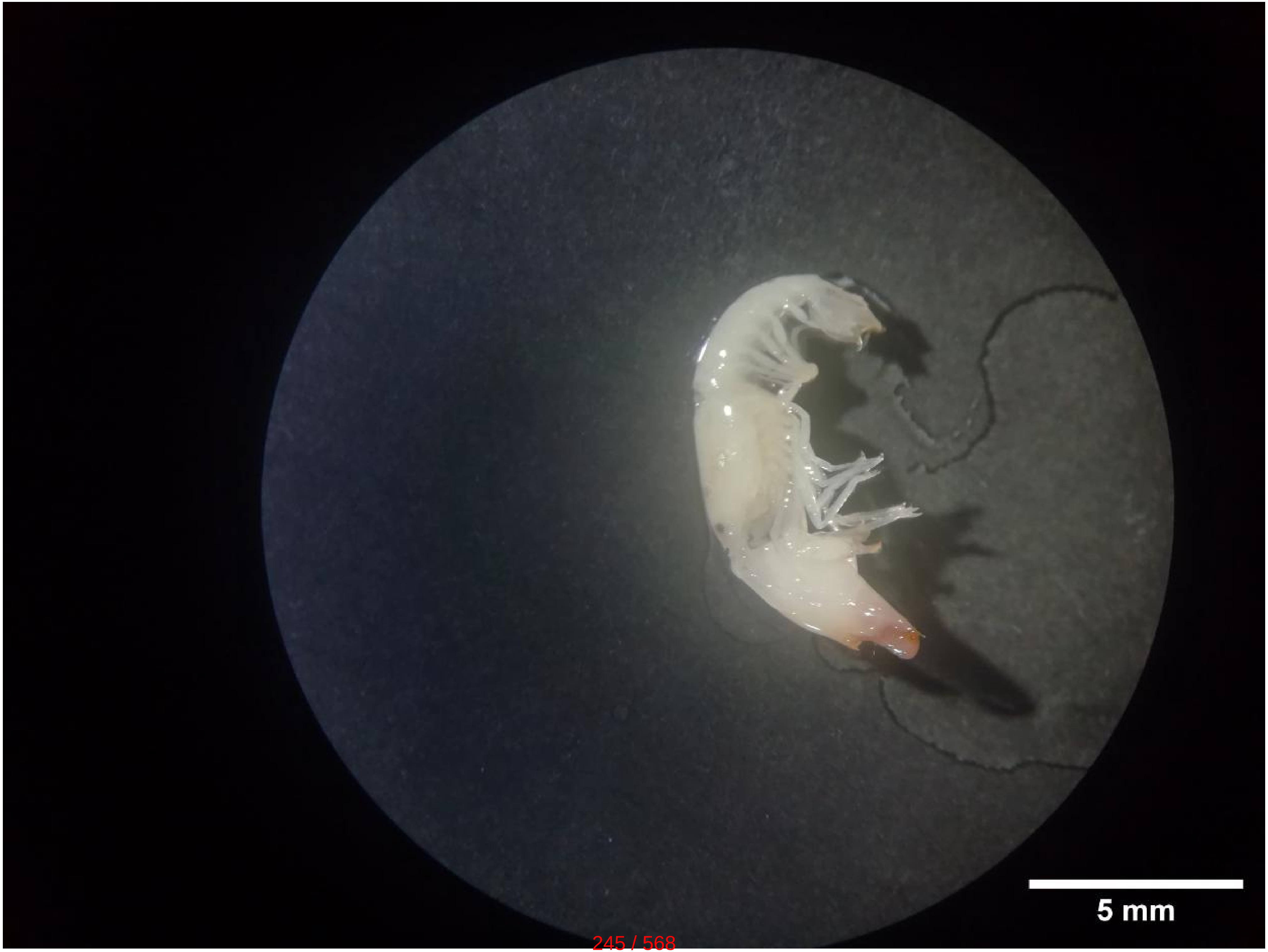
5 mm

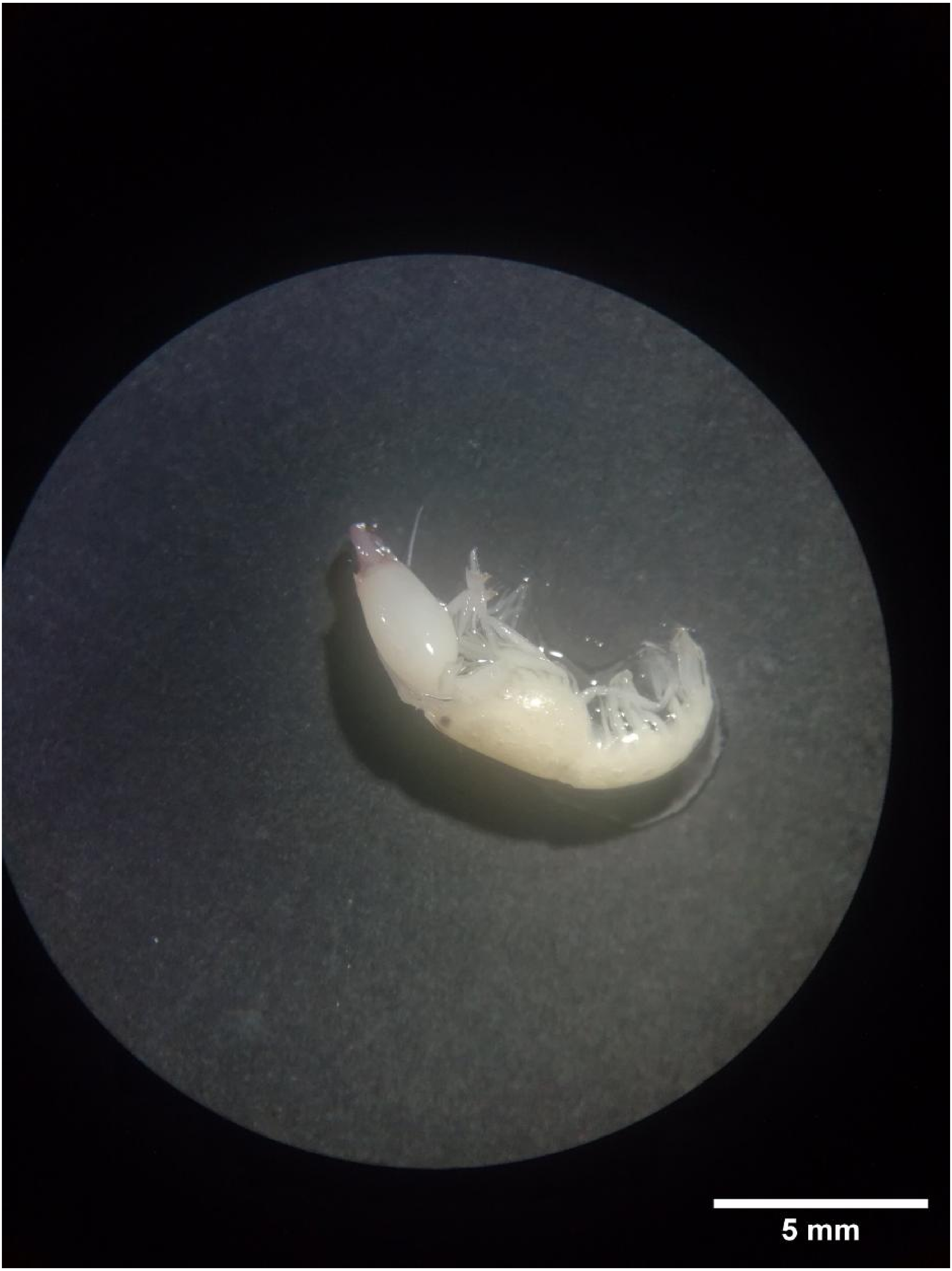
5 mm

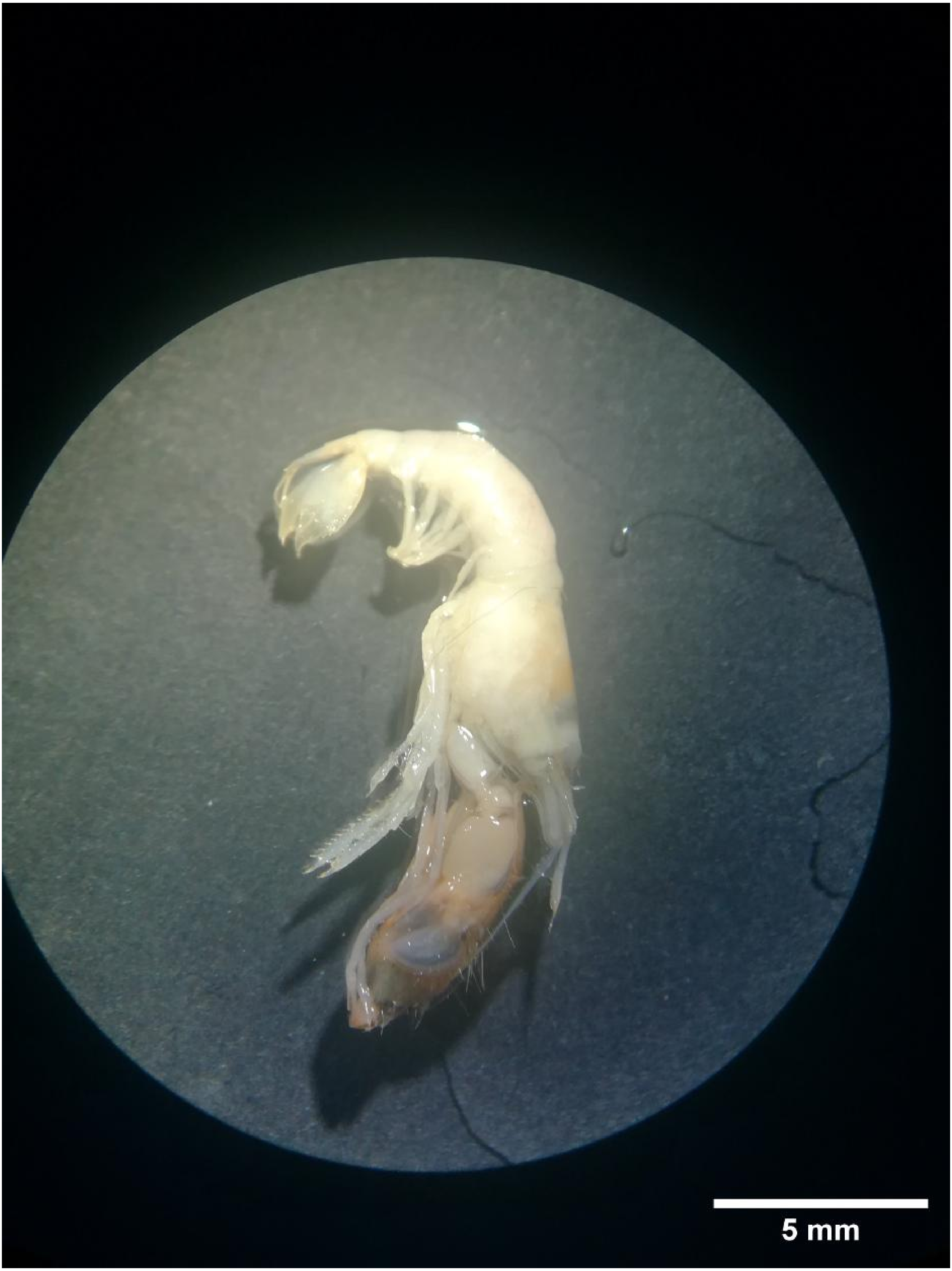
5 mm

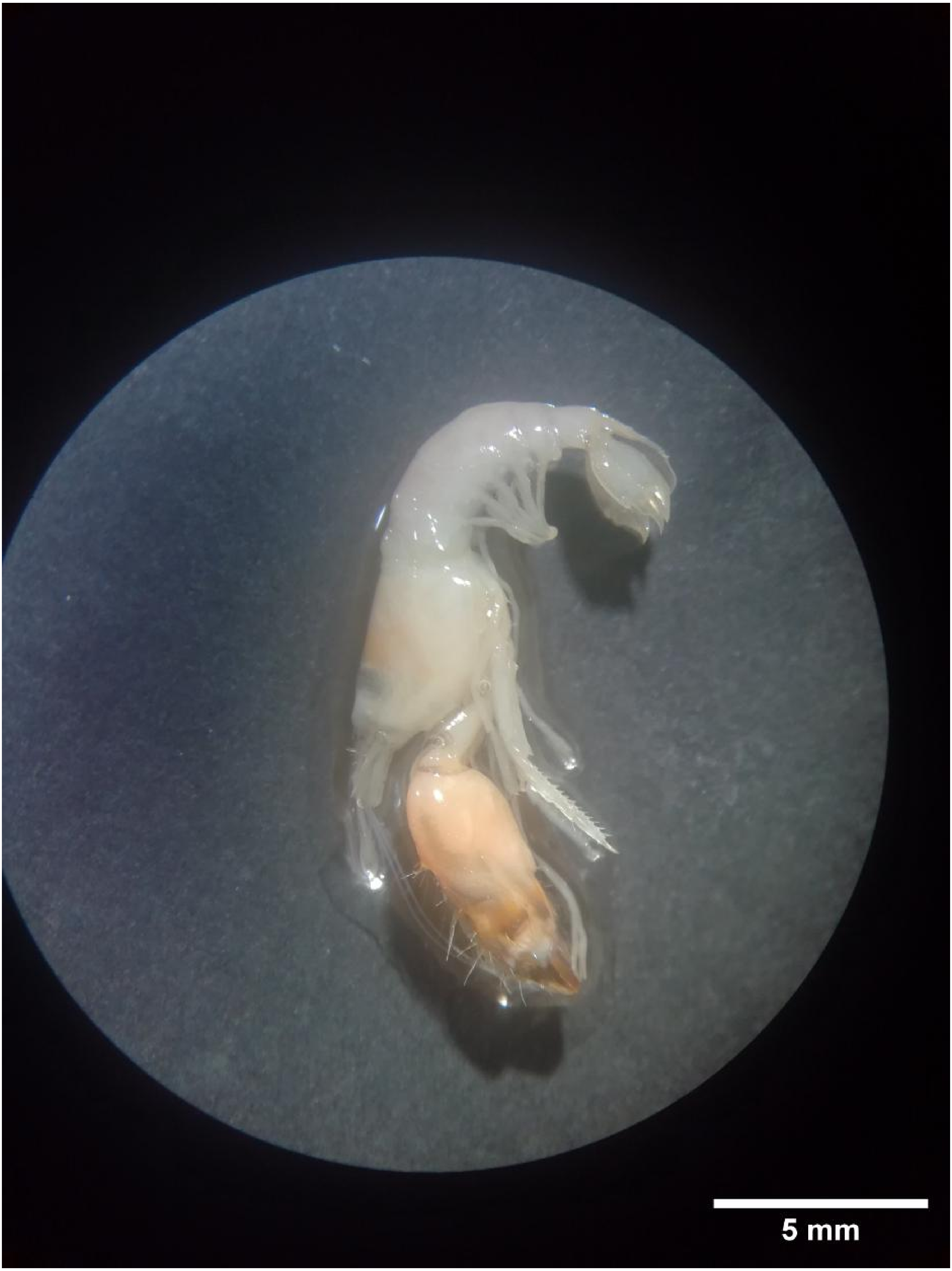
5 mm

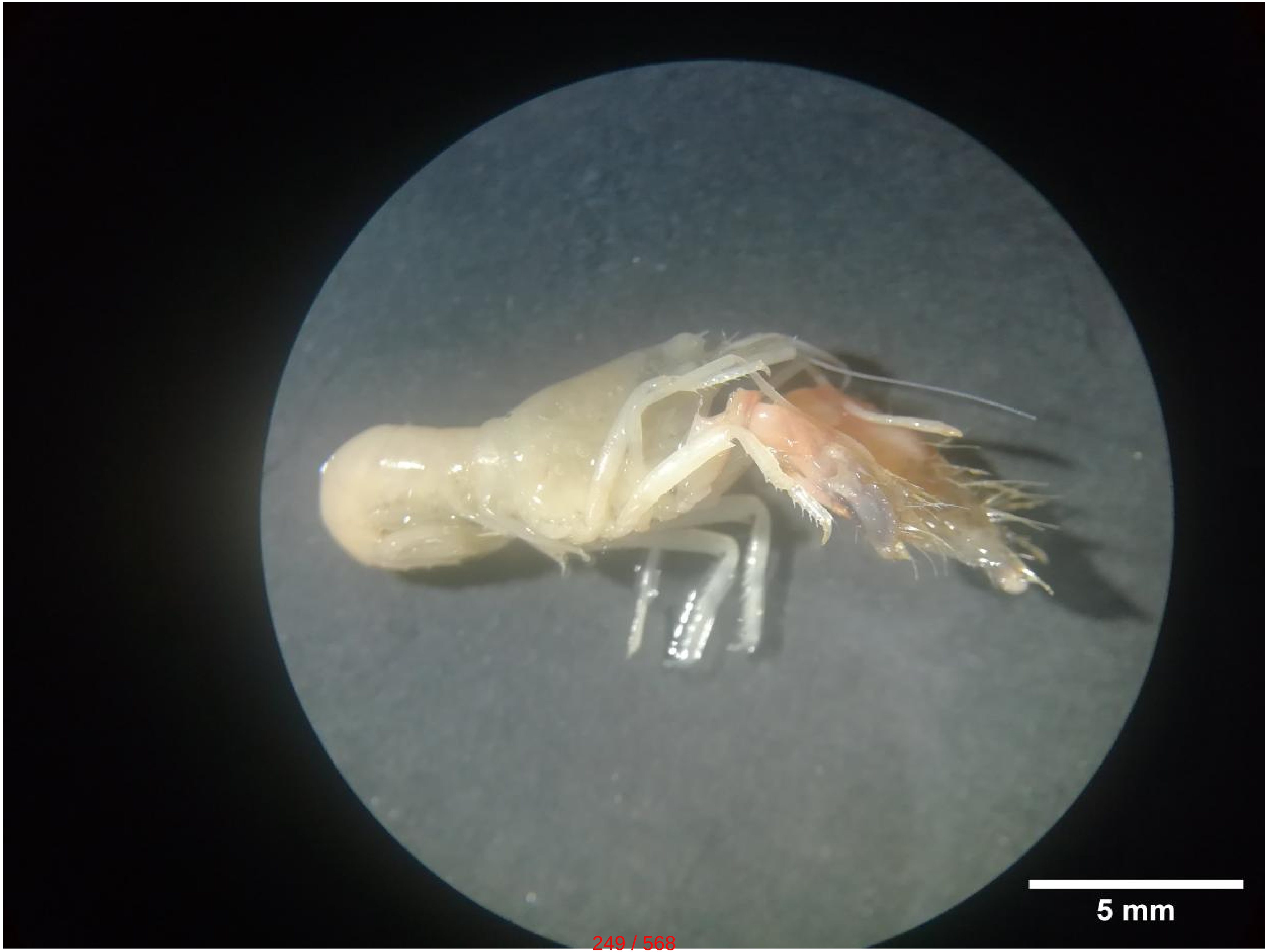
5 mm

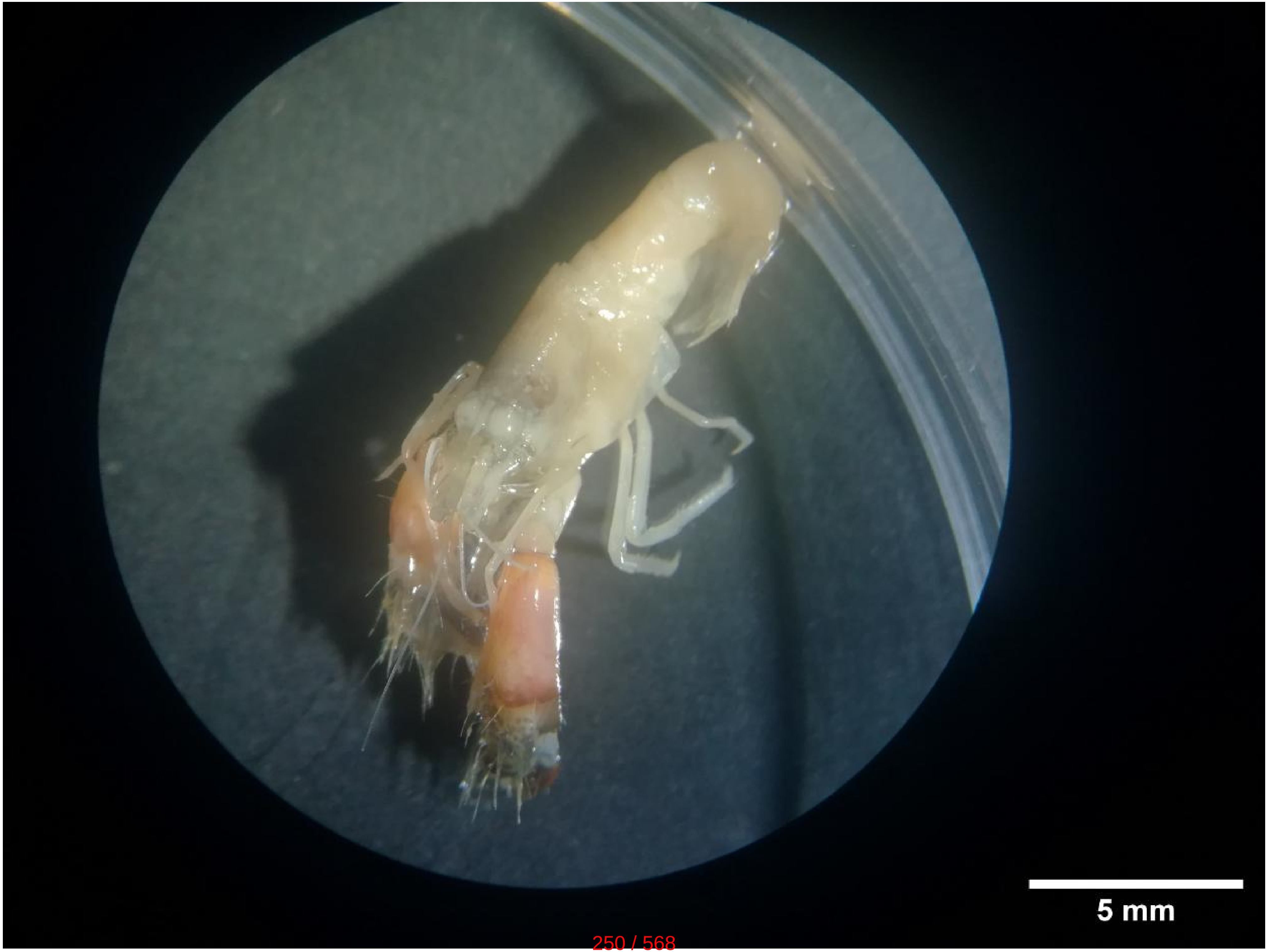
5 mm

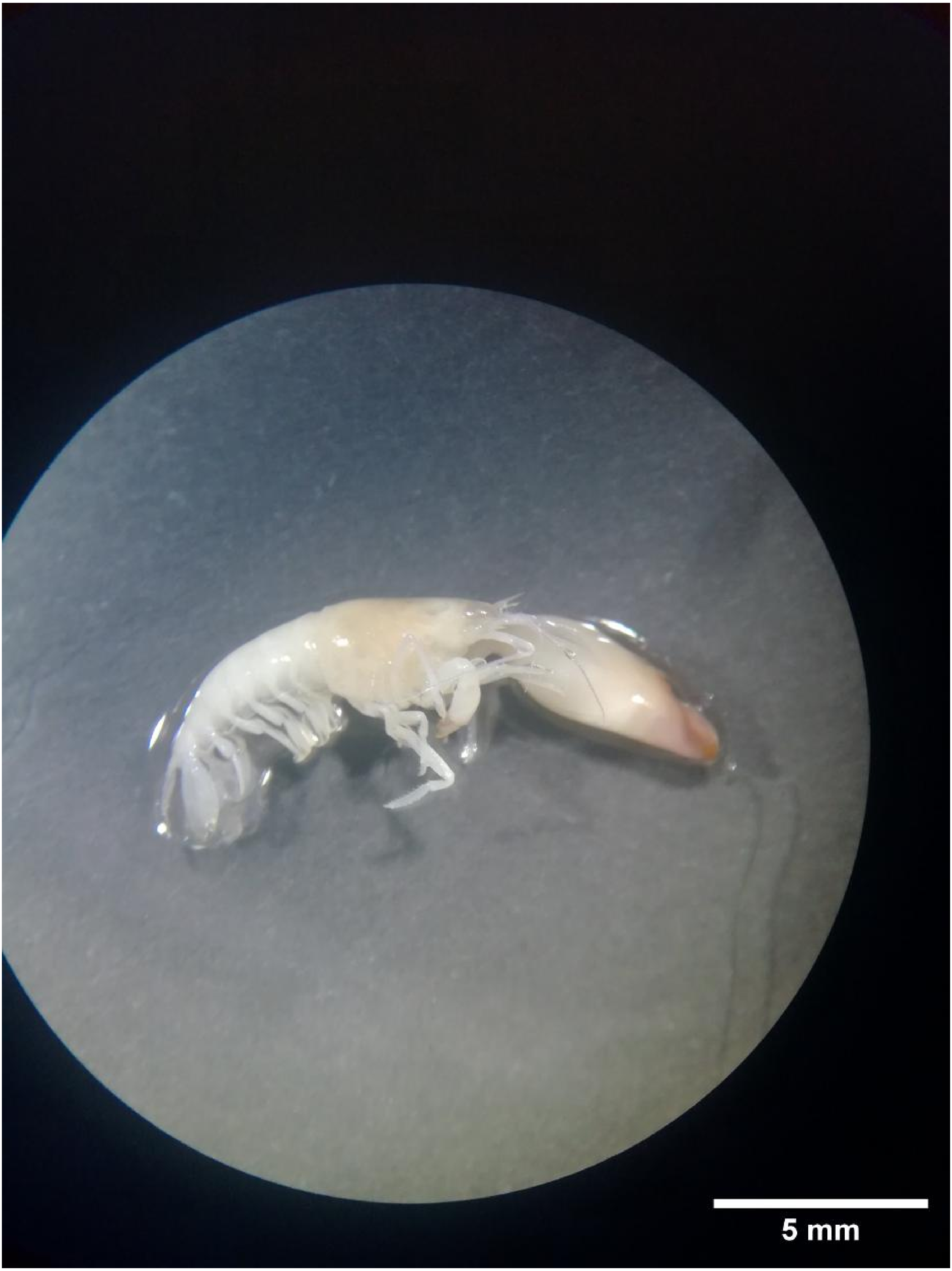
5 mm

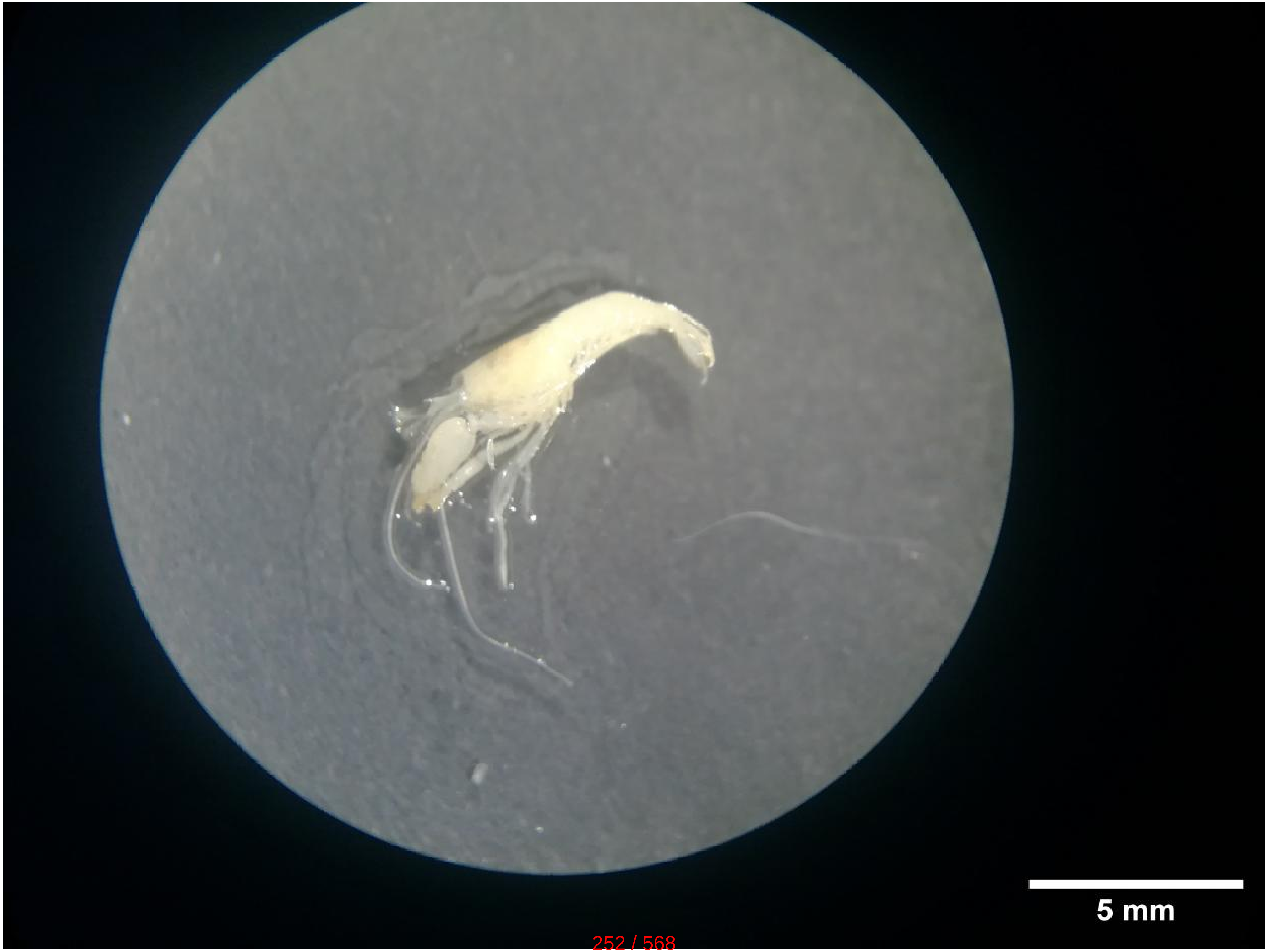
5 mm

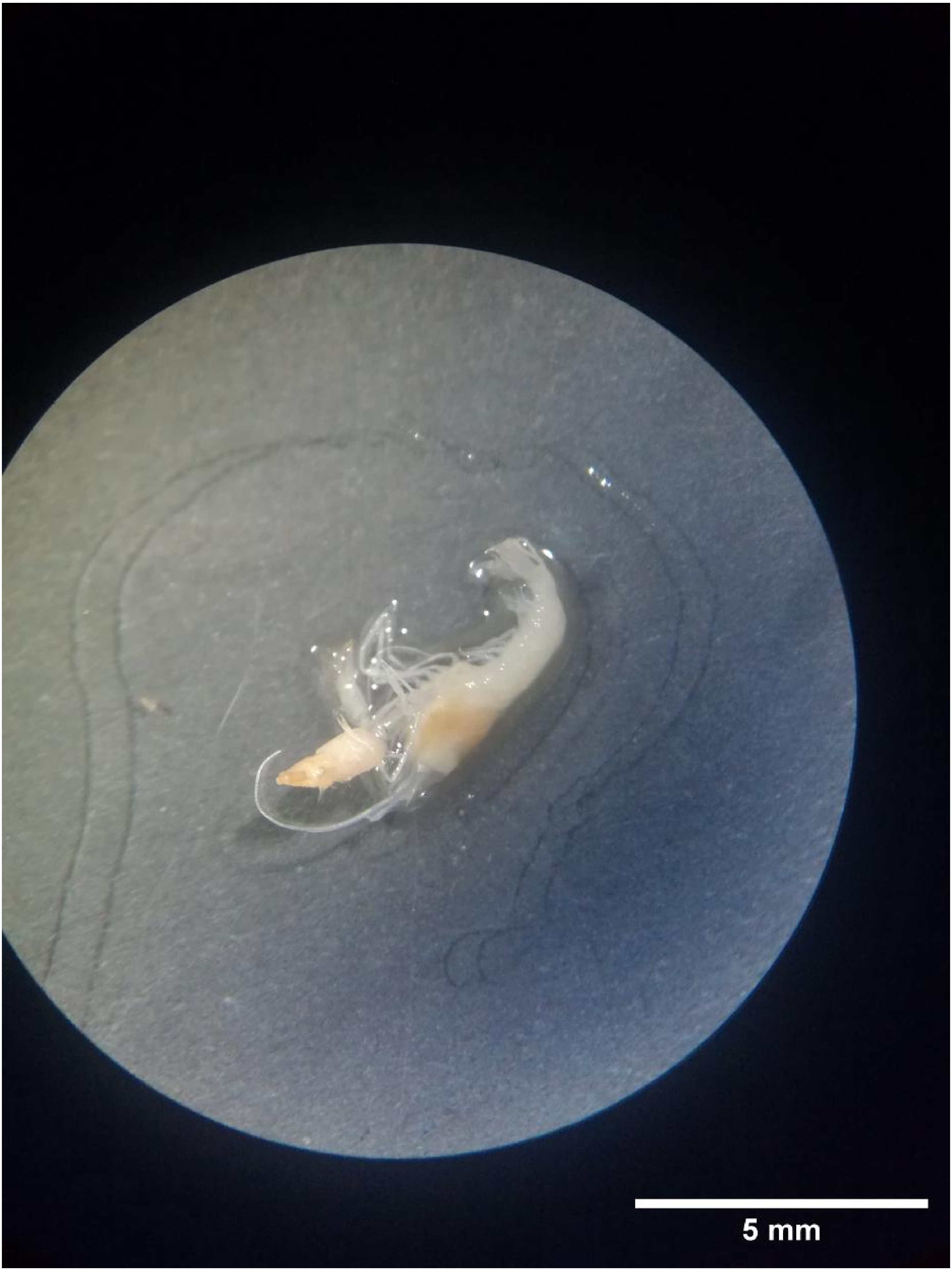
5 mm

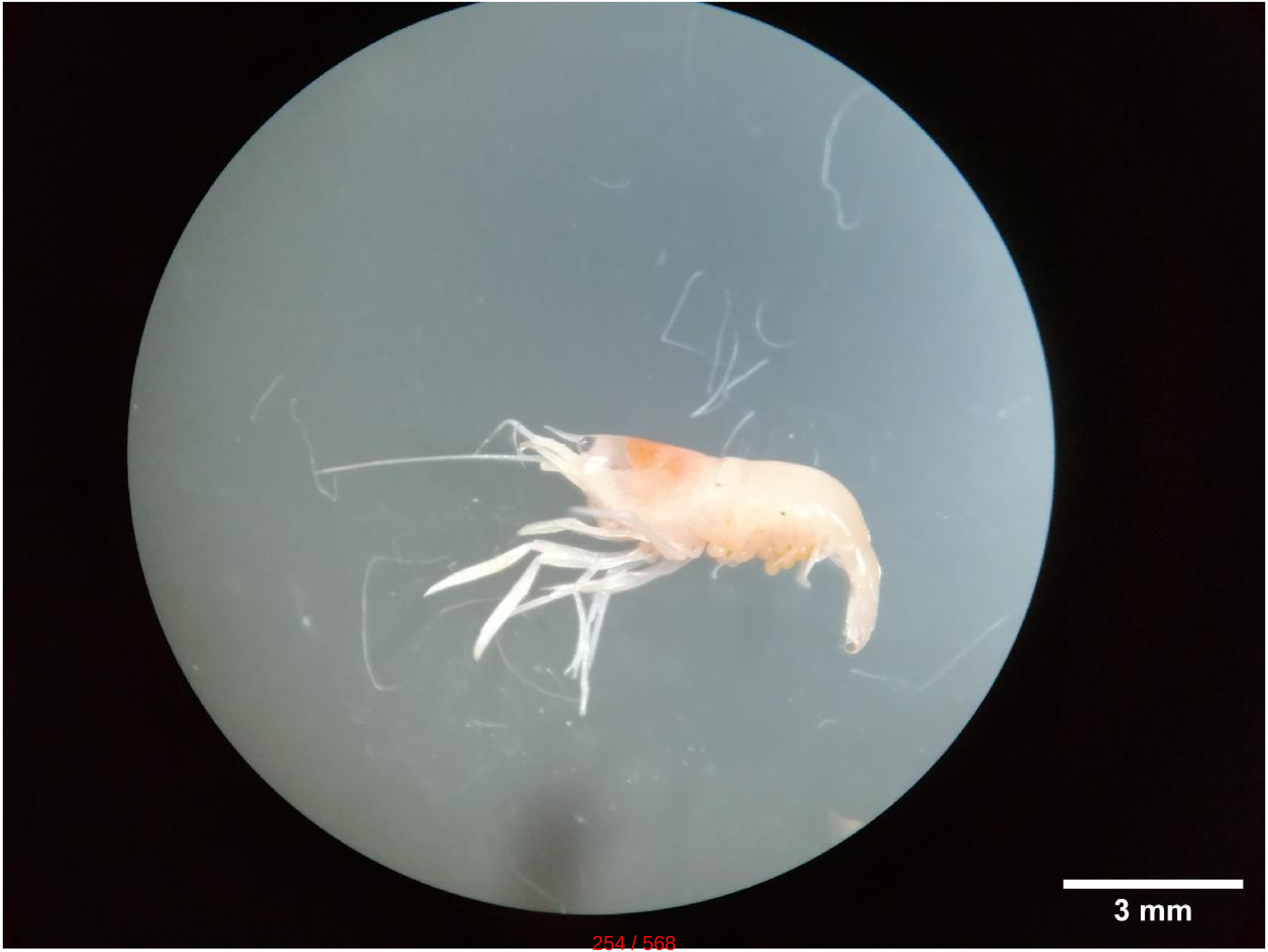
3 mm

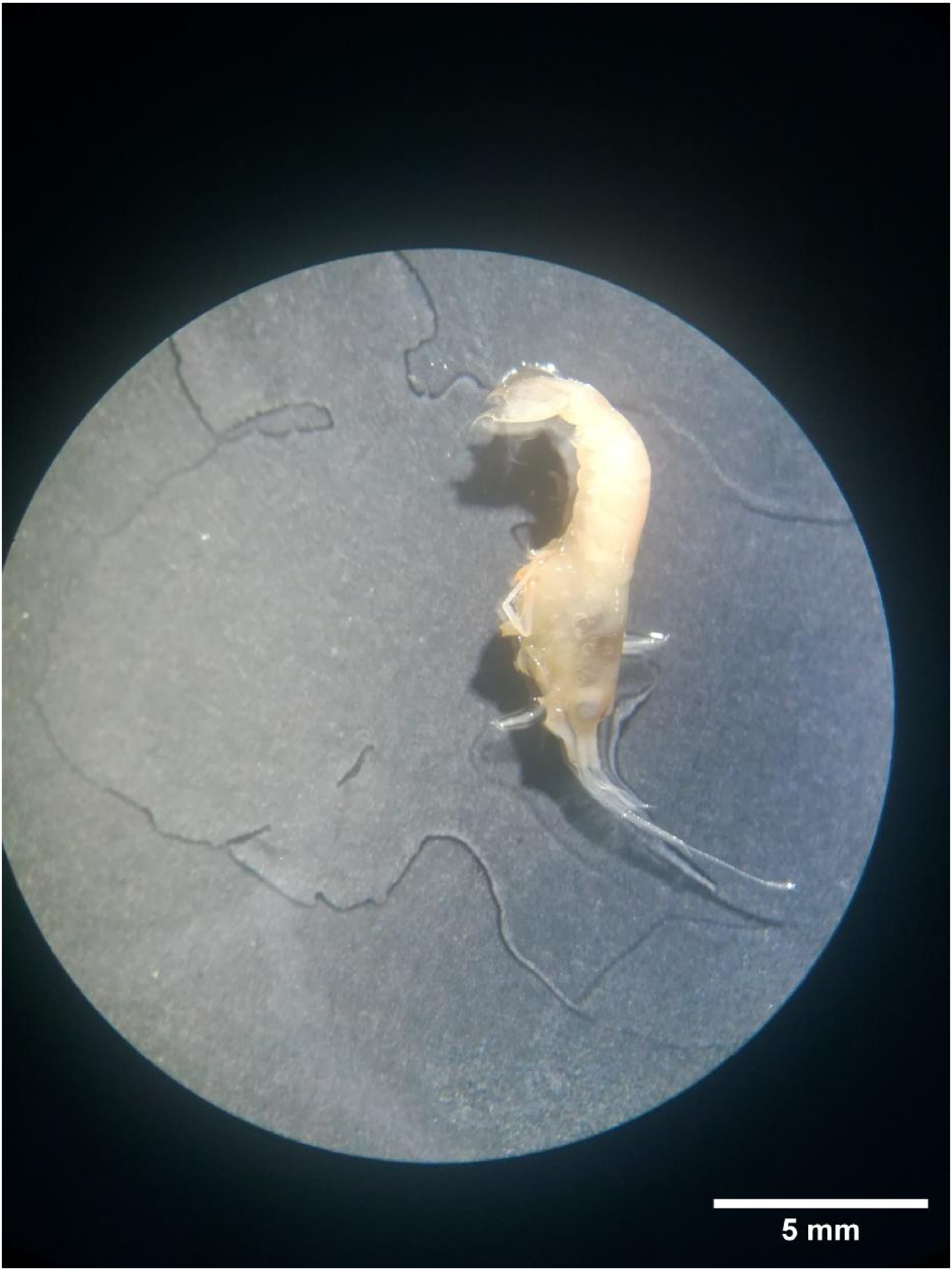
5 mm

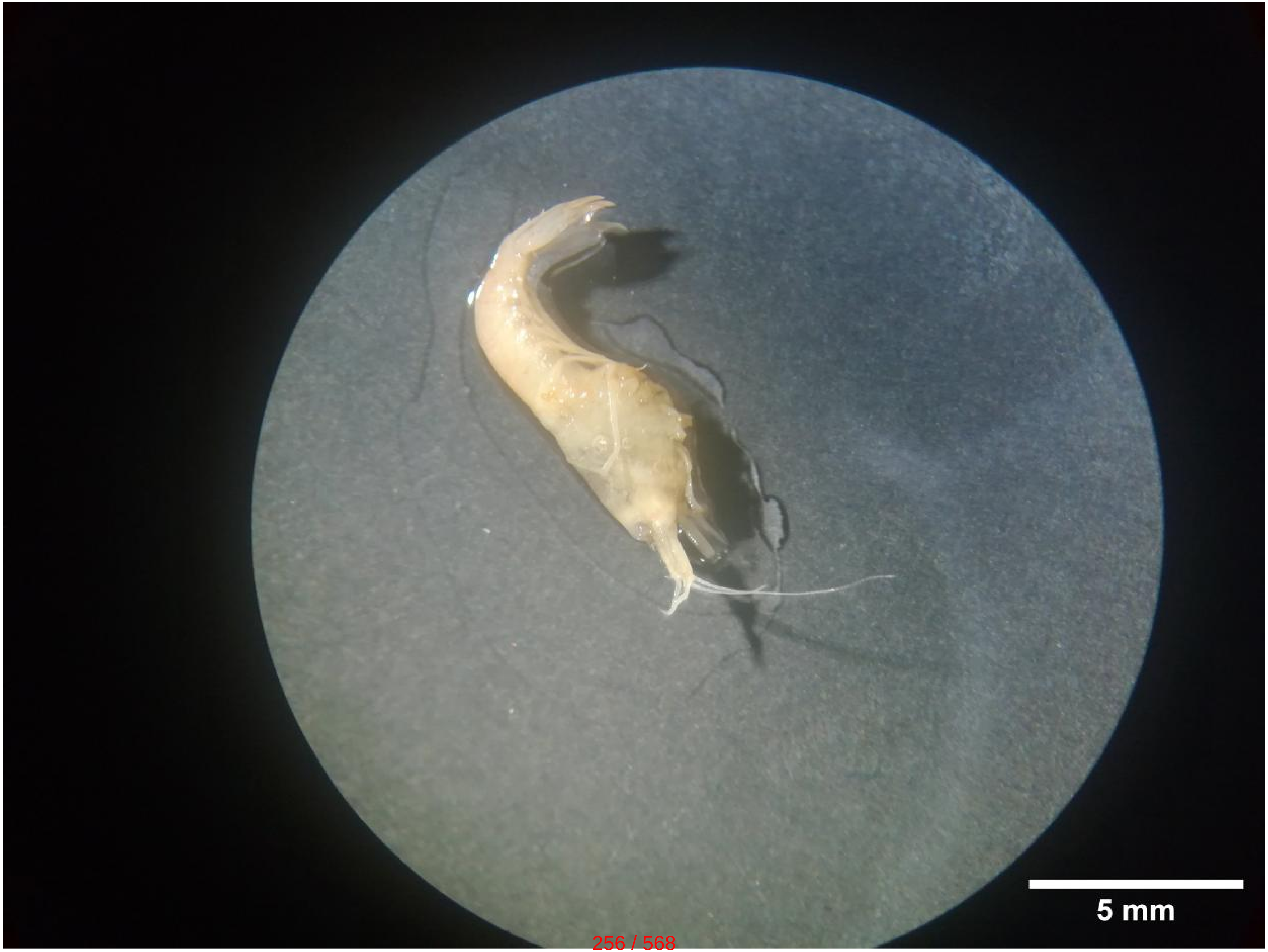
5 mm

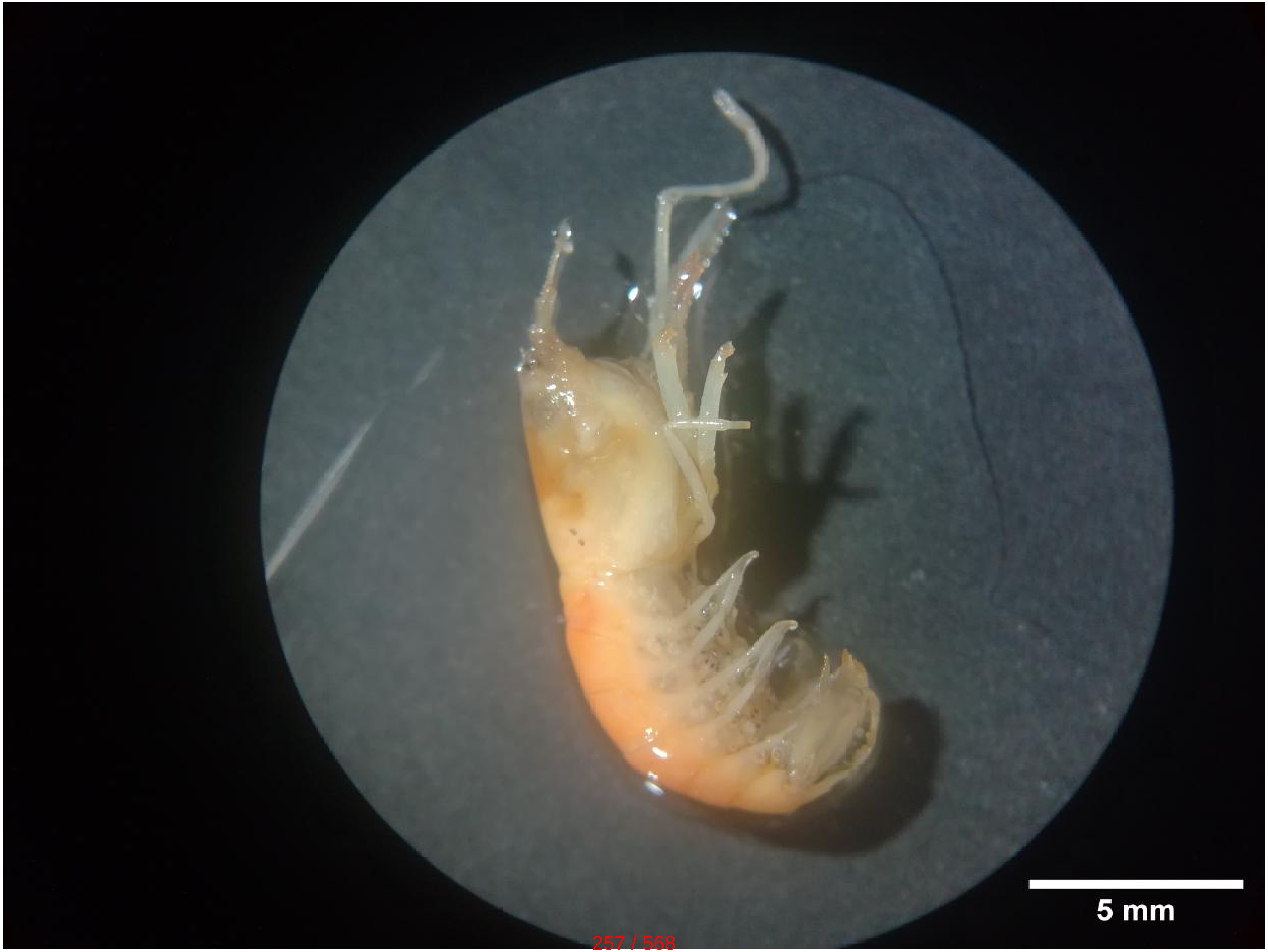
5 mm

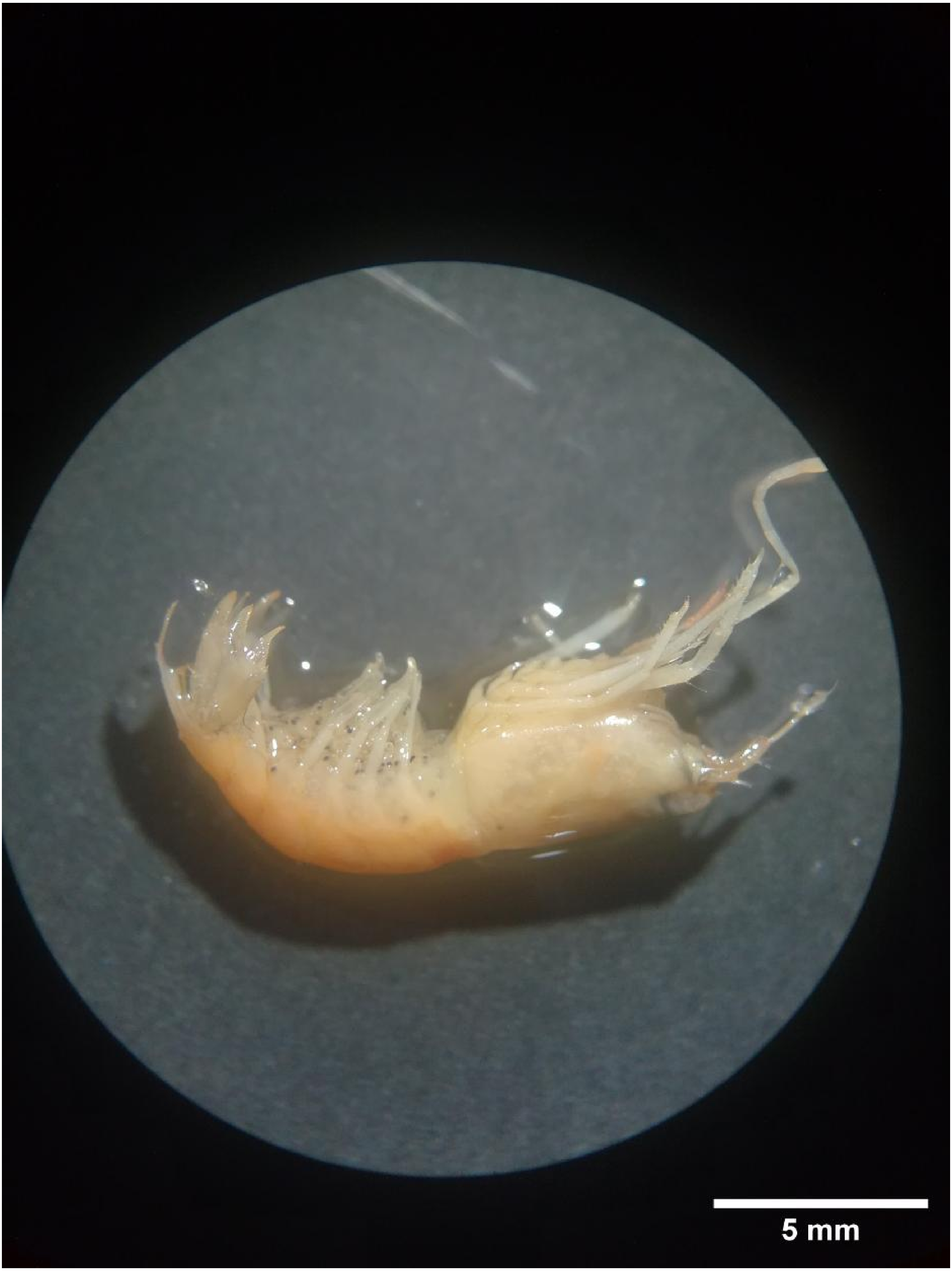
5 mm

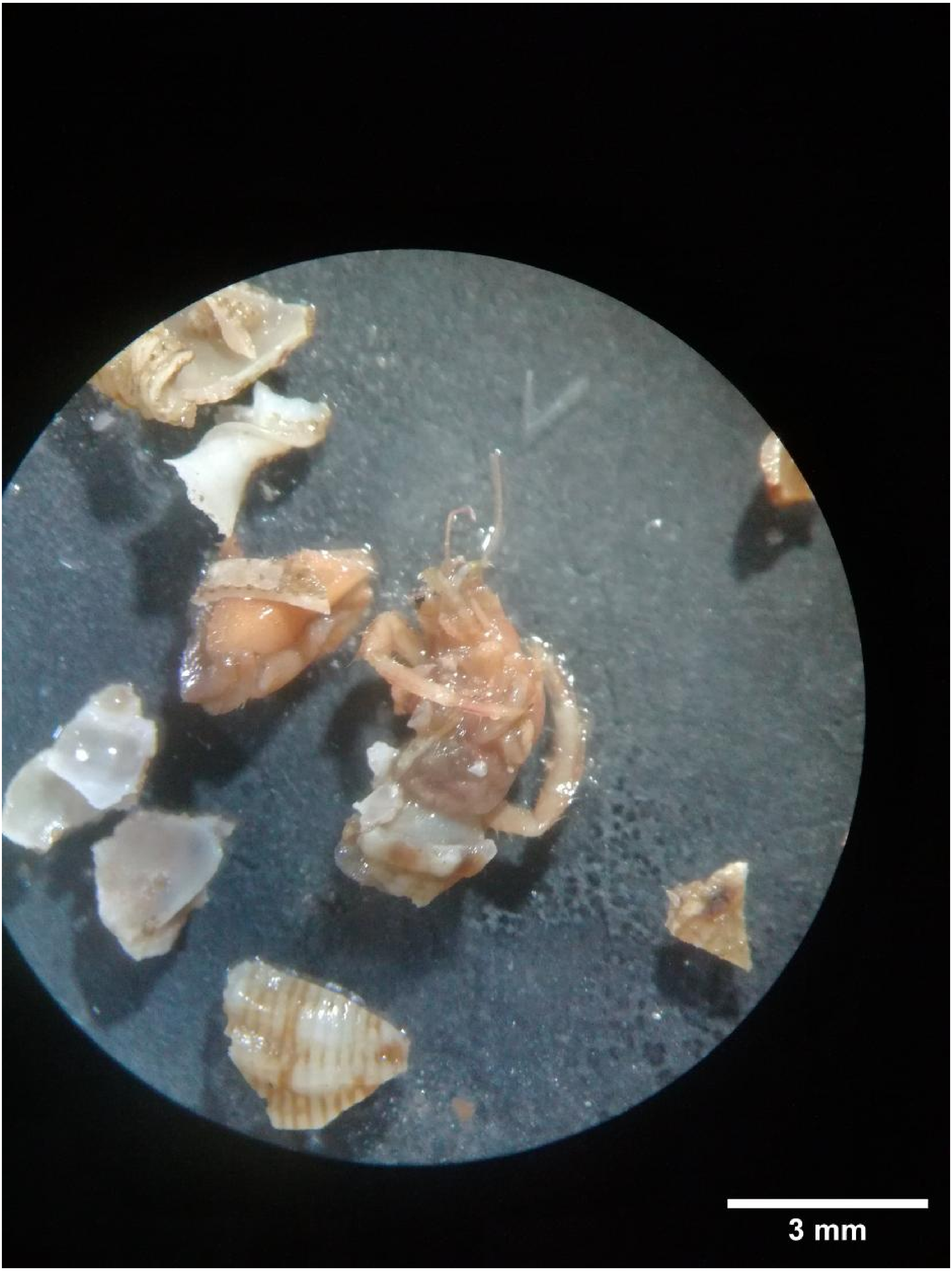
3 mm

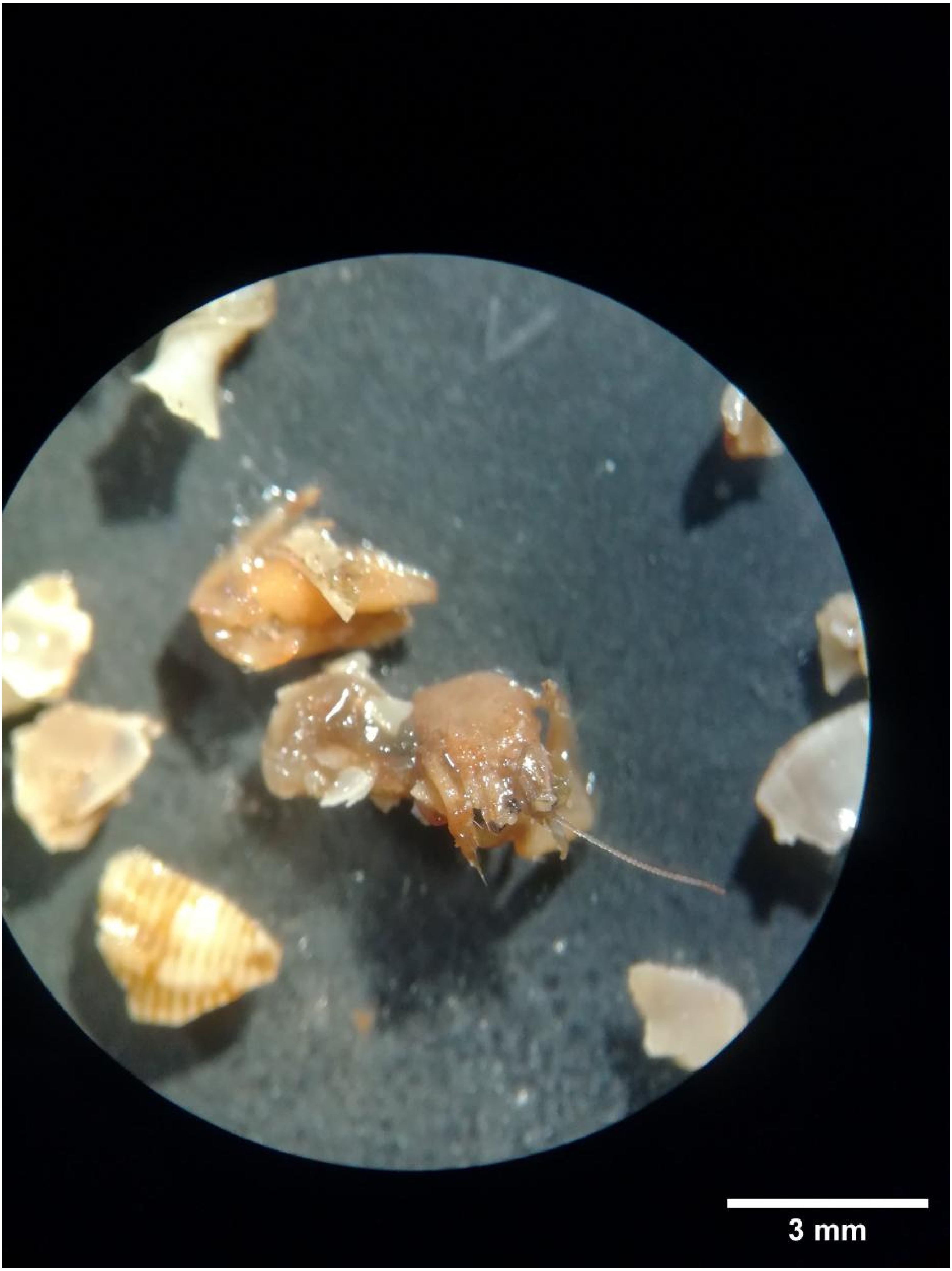
3 mm

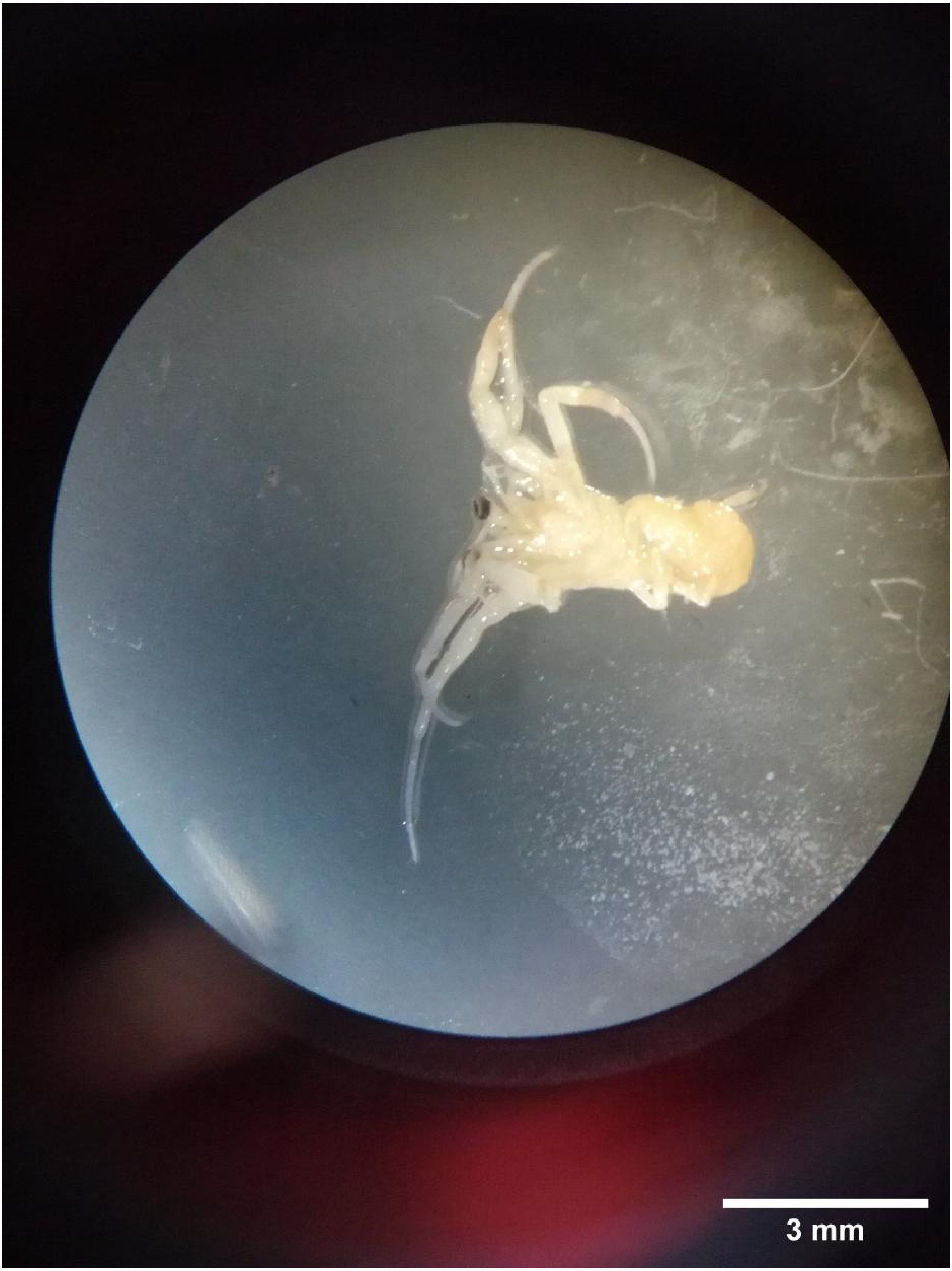
3 mm

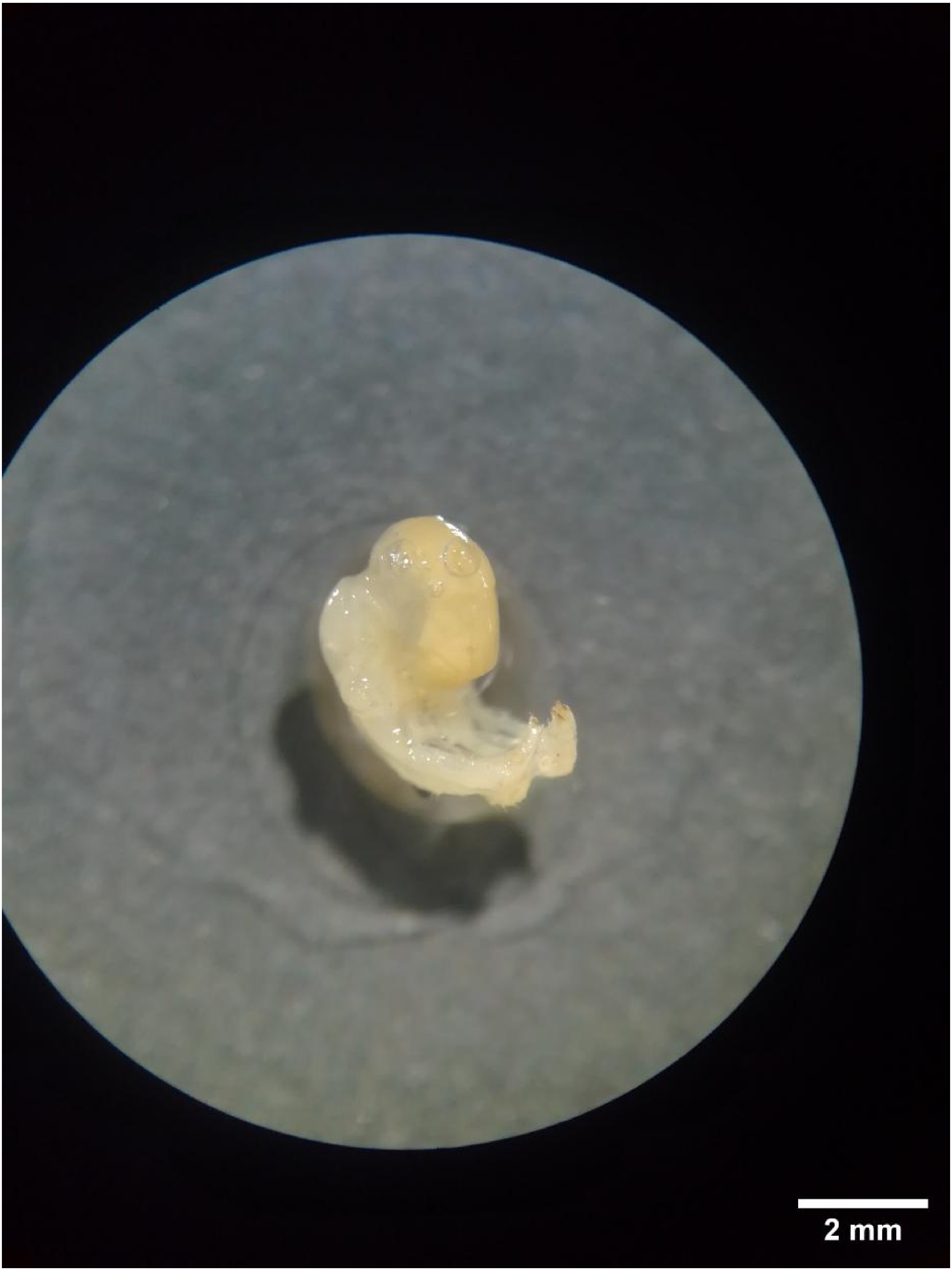
2 mm

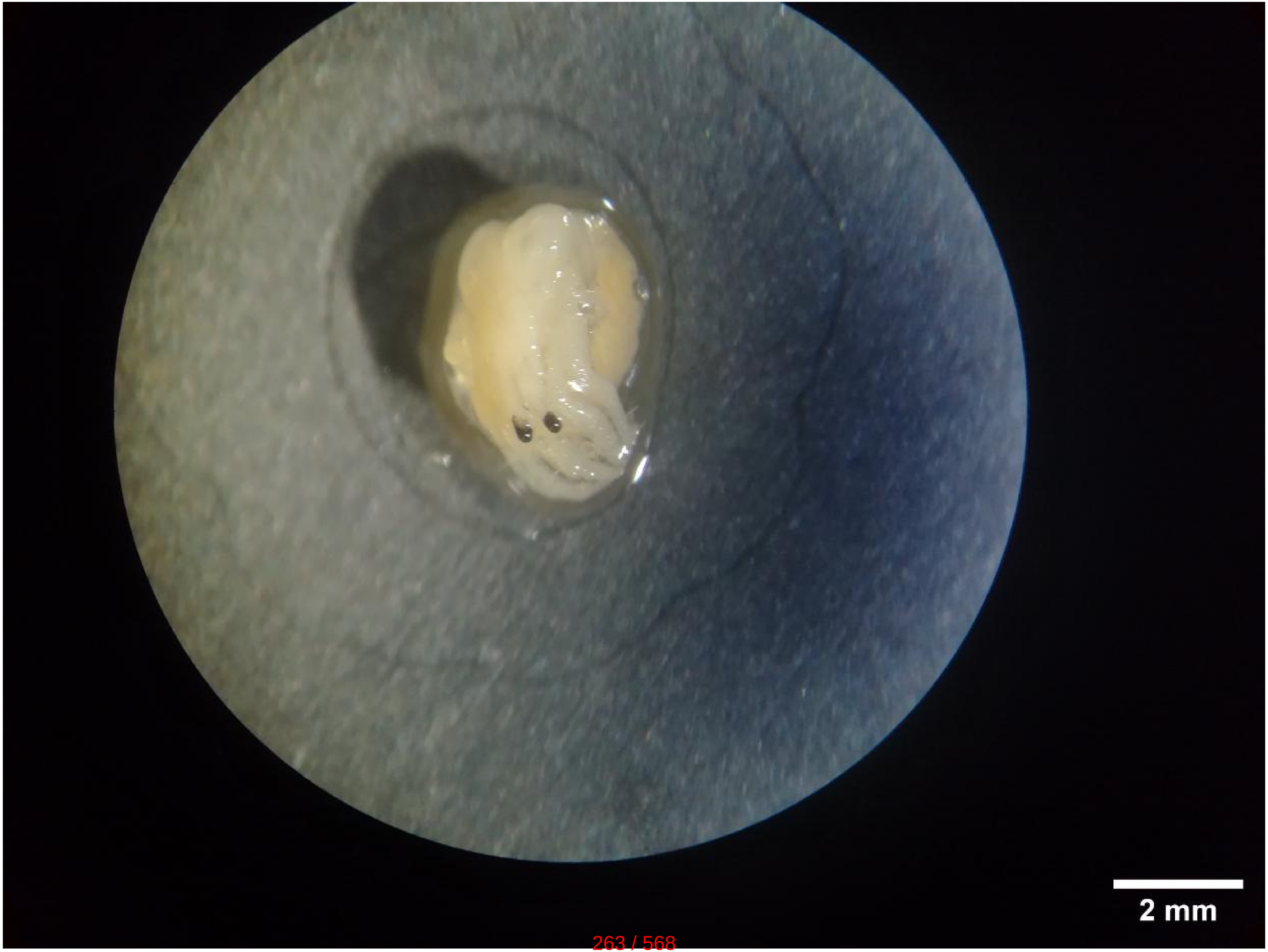
2 mm

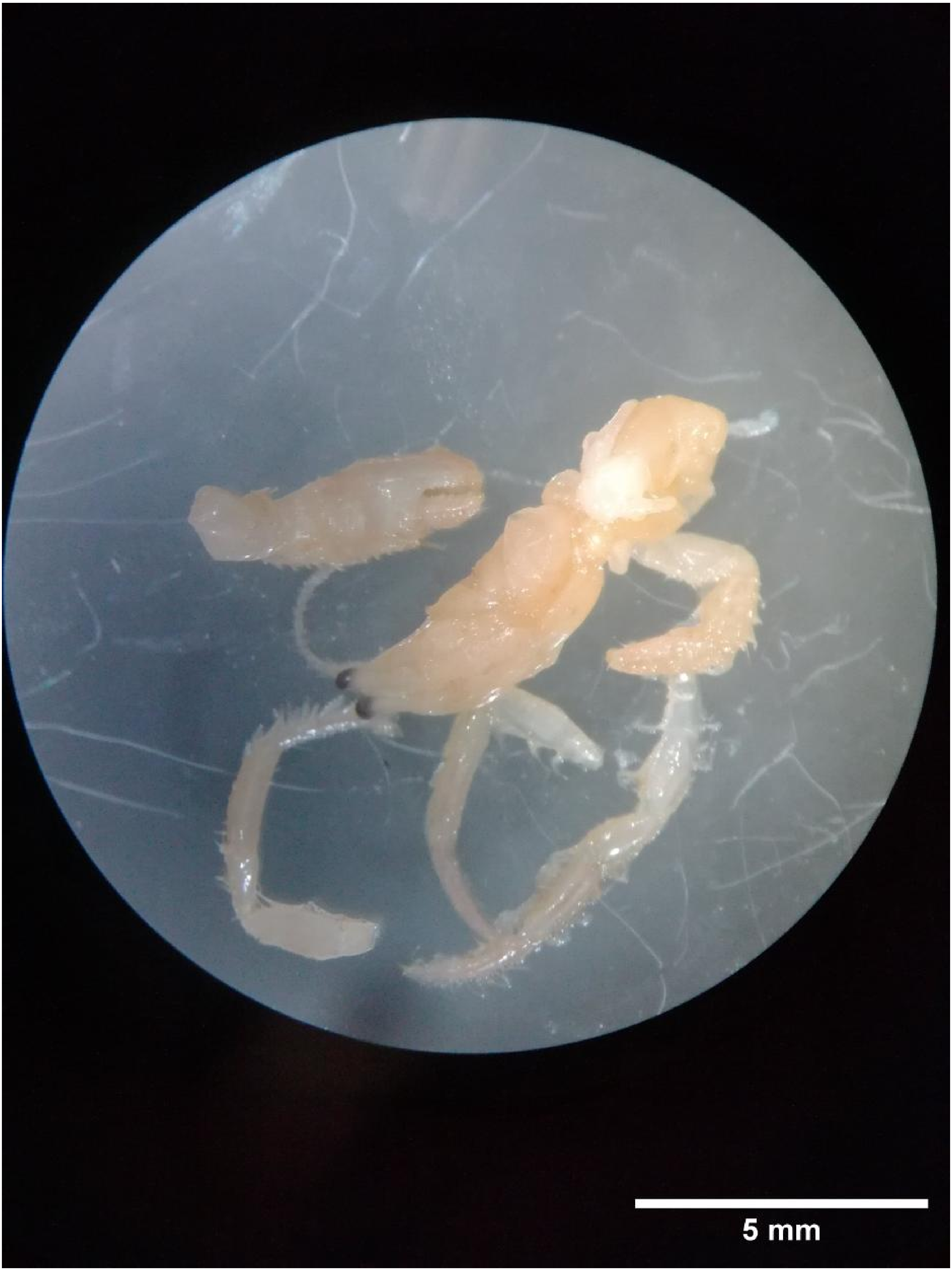
5 mm

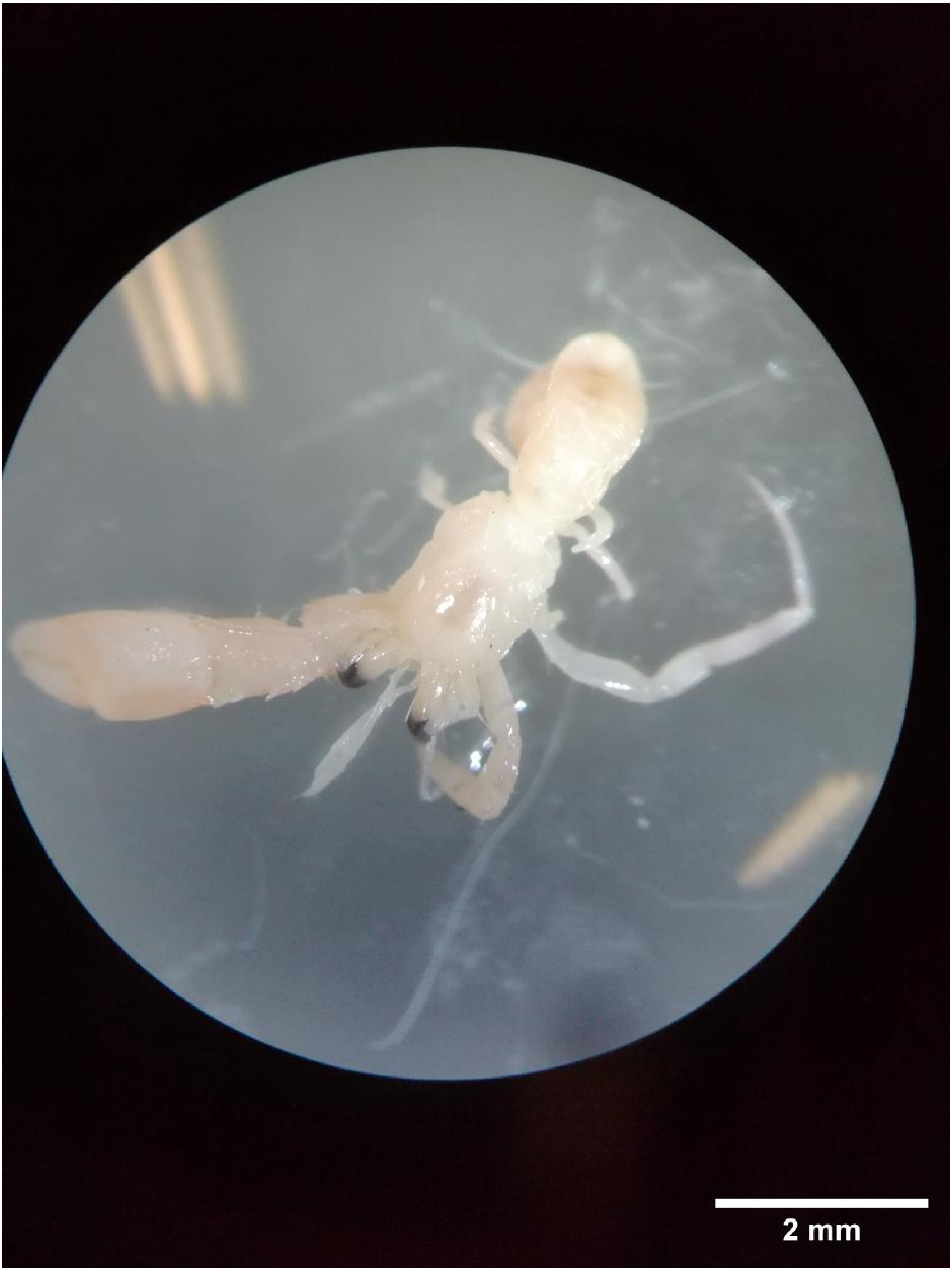
2 mm

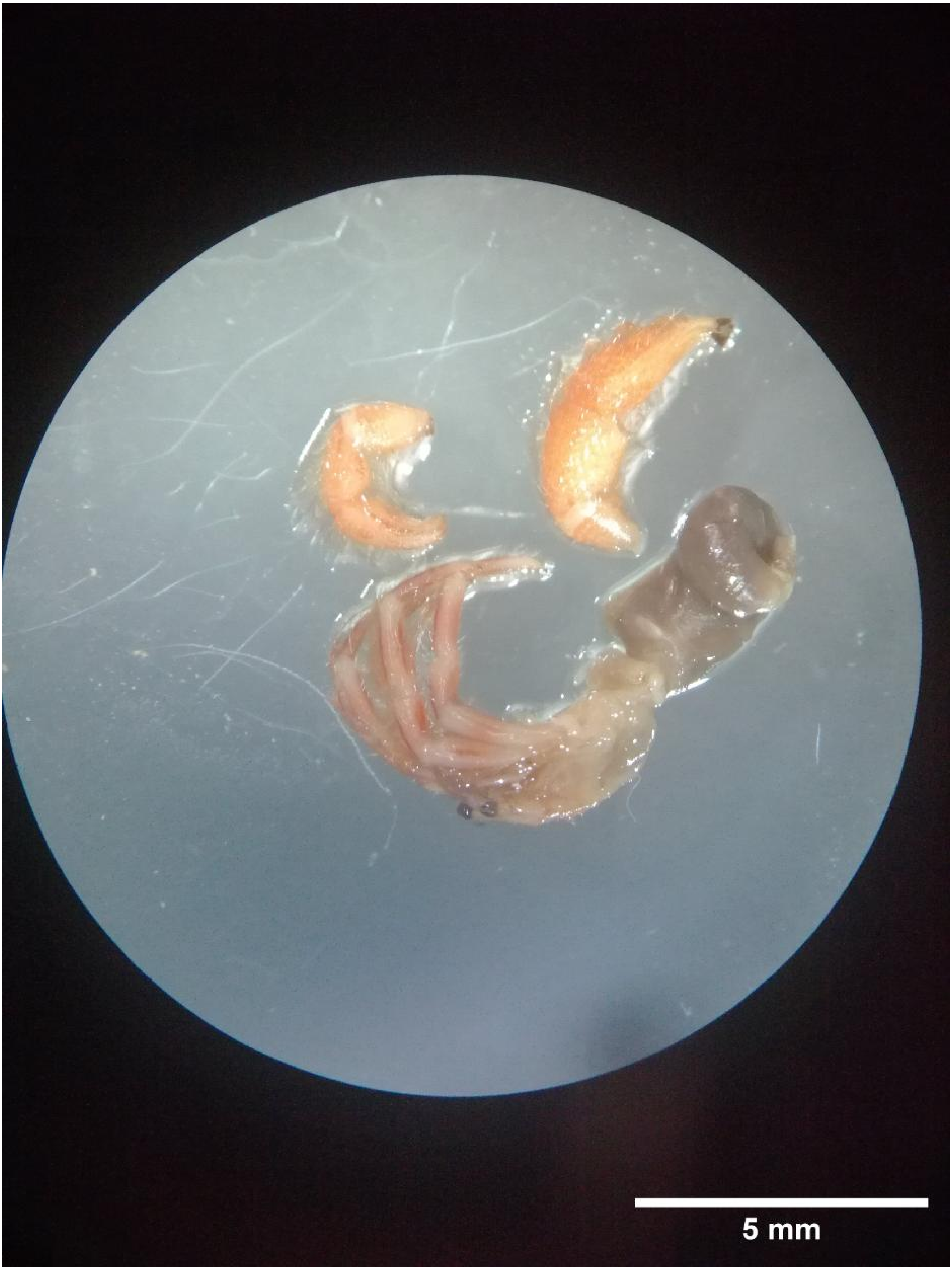
5 mm

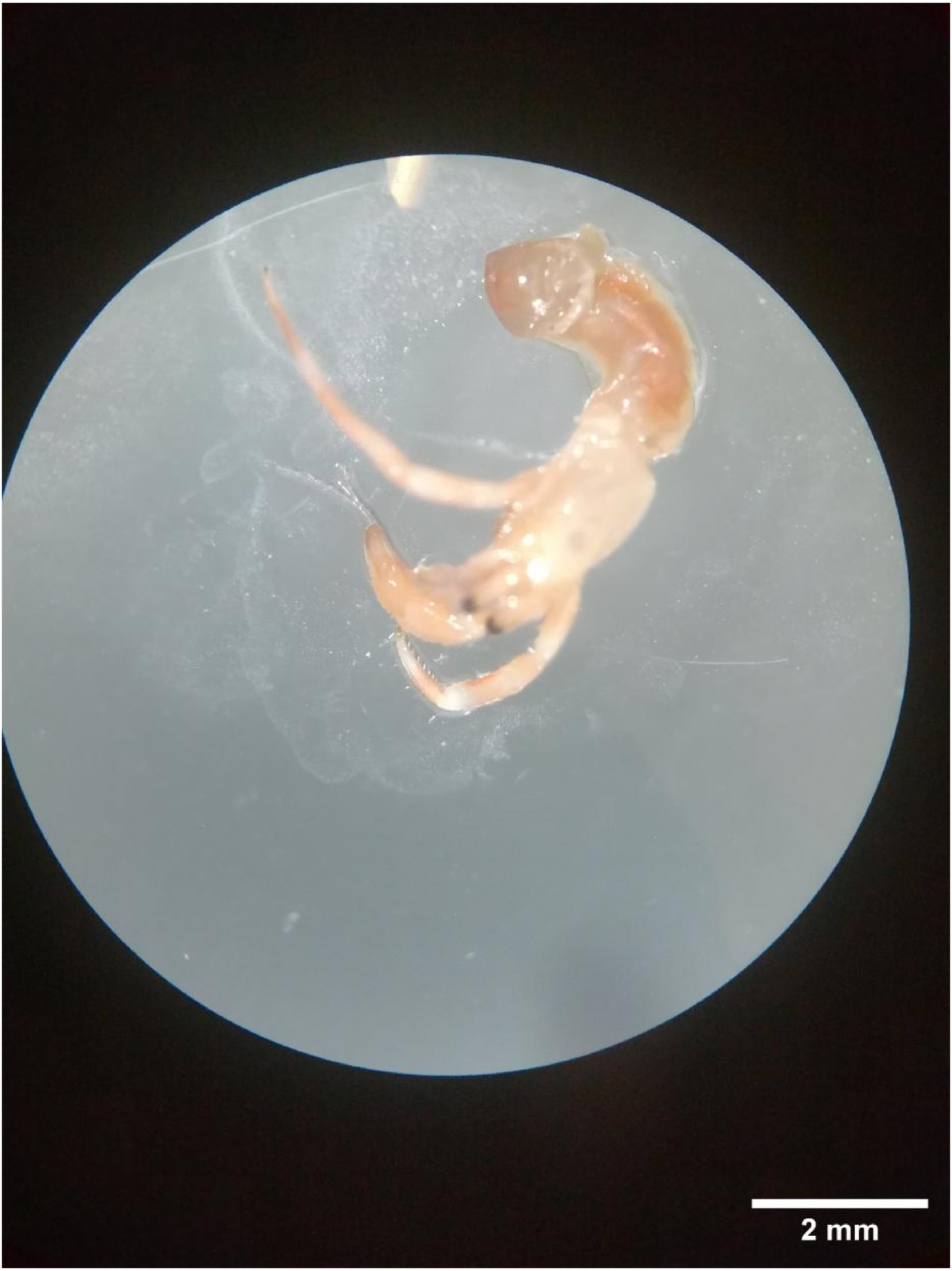
2 mm

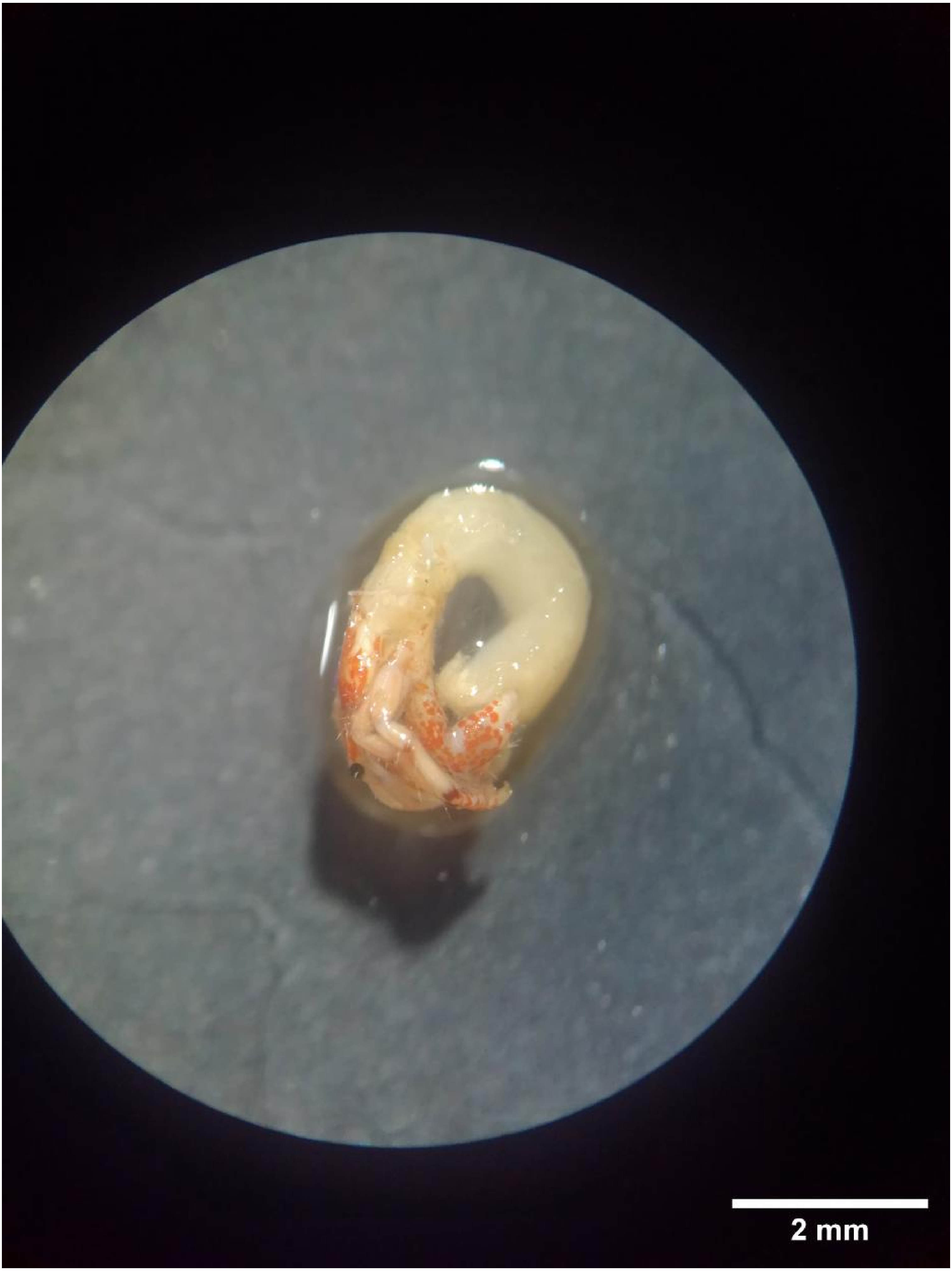
2 mm

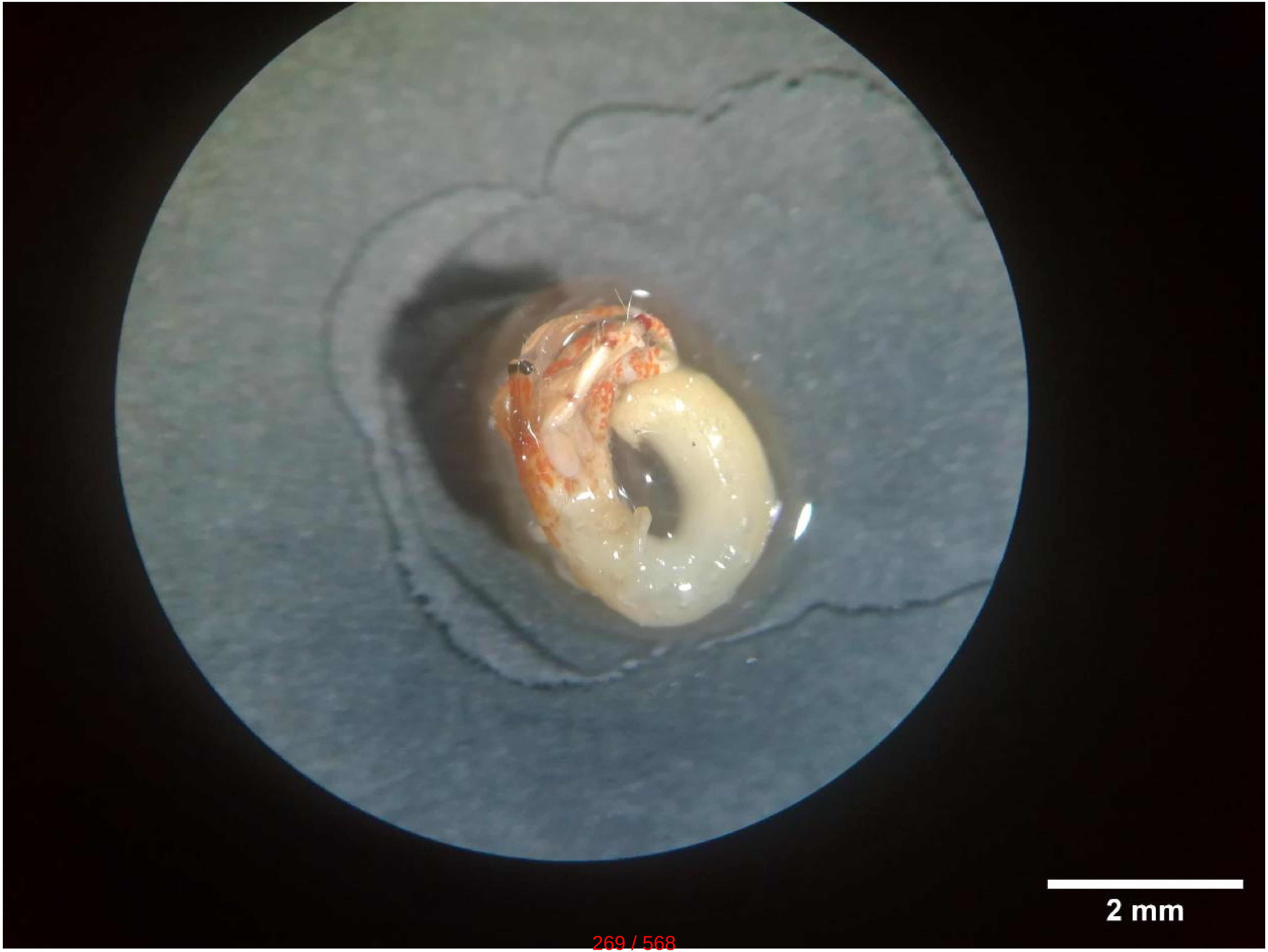
2 mm

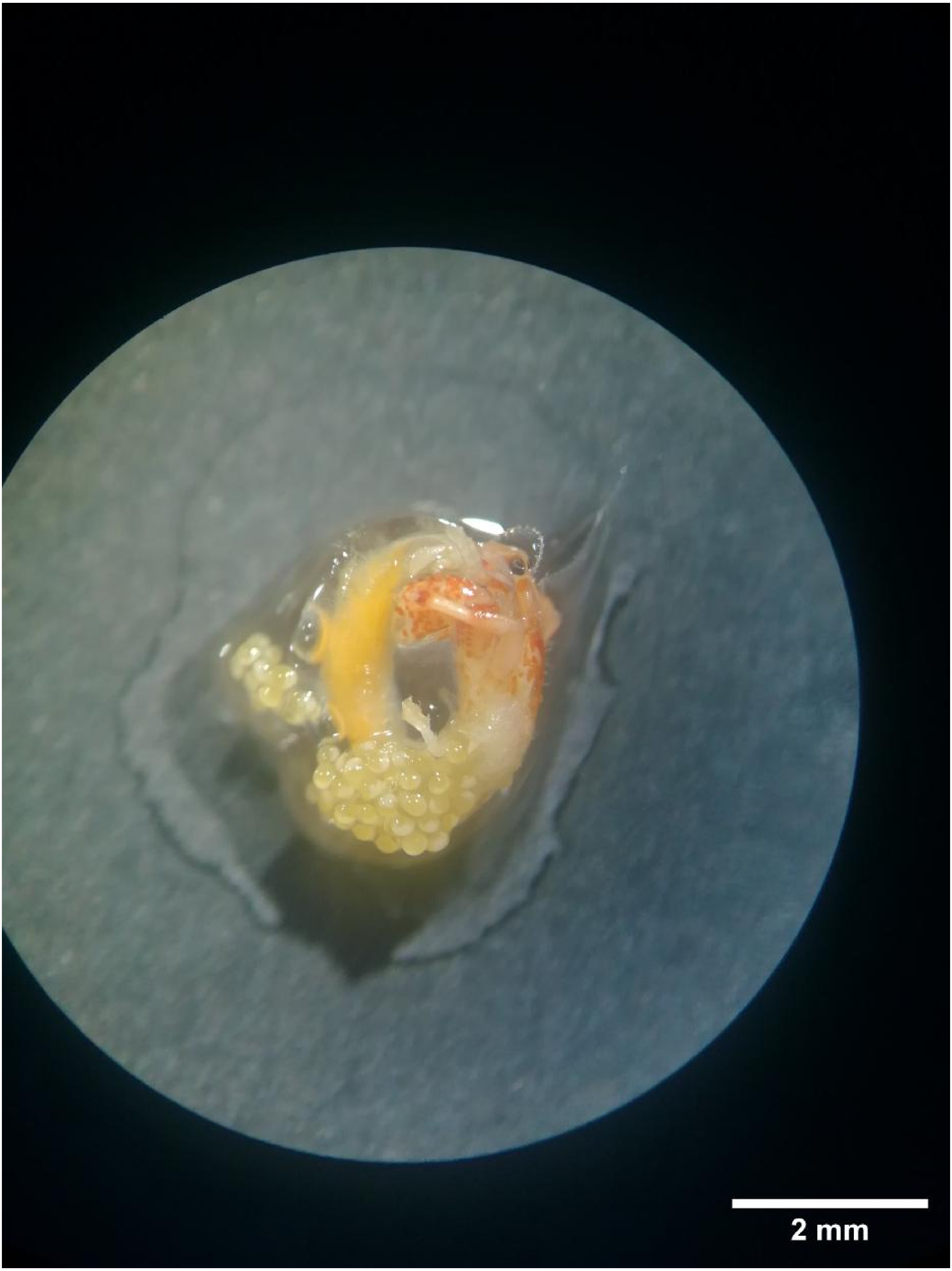
2 mm

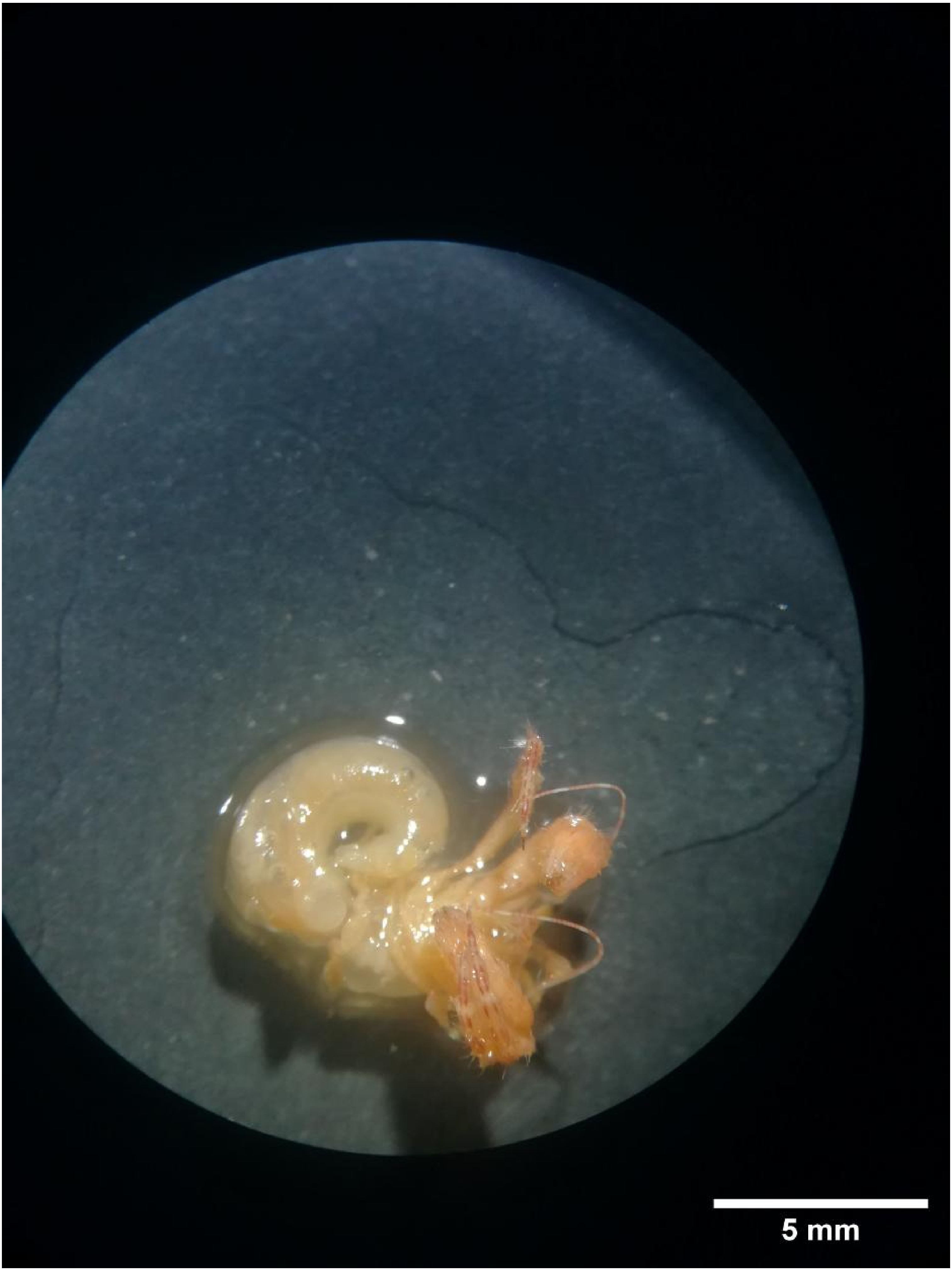
5 mm

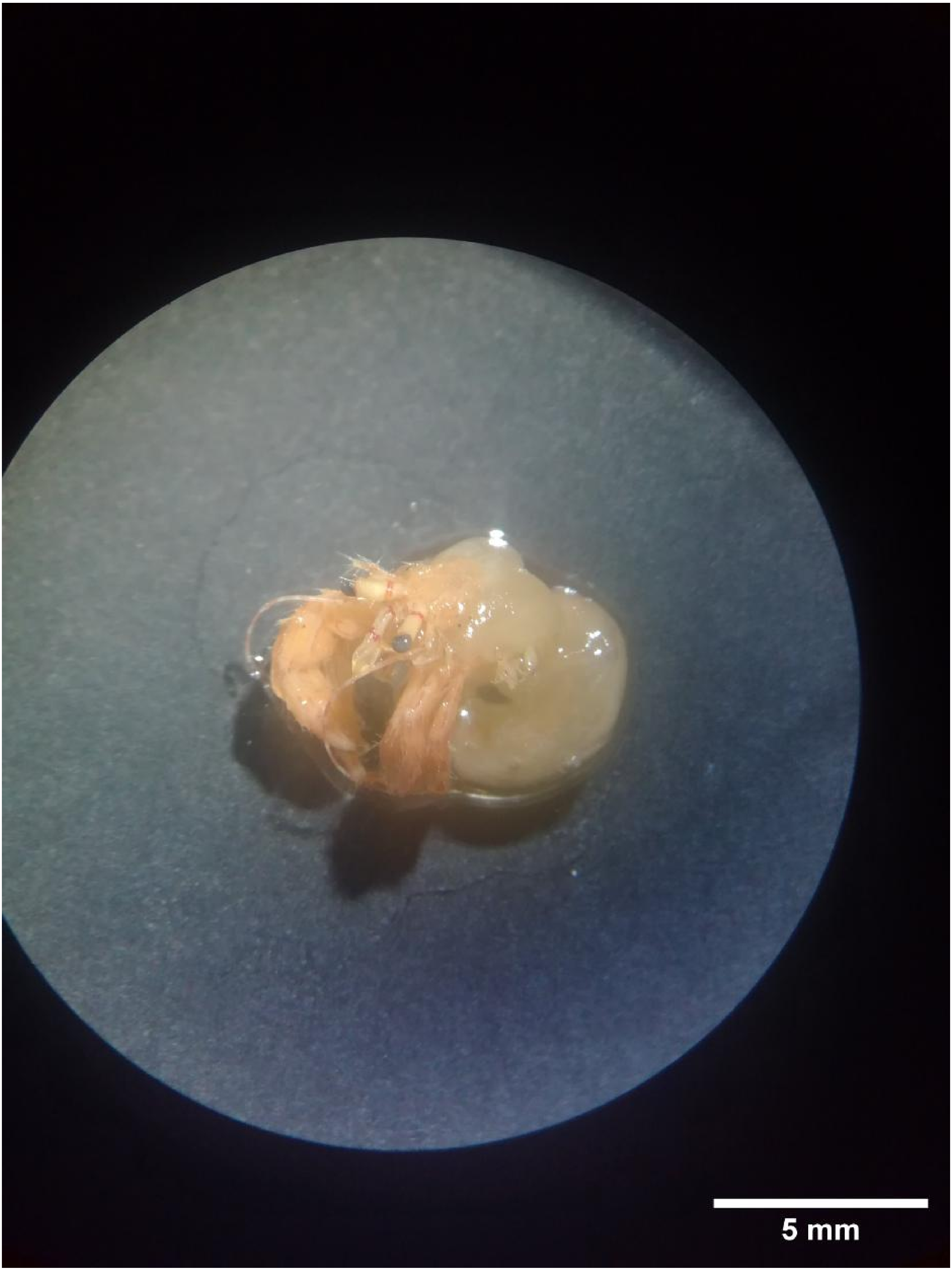
5 mm

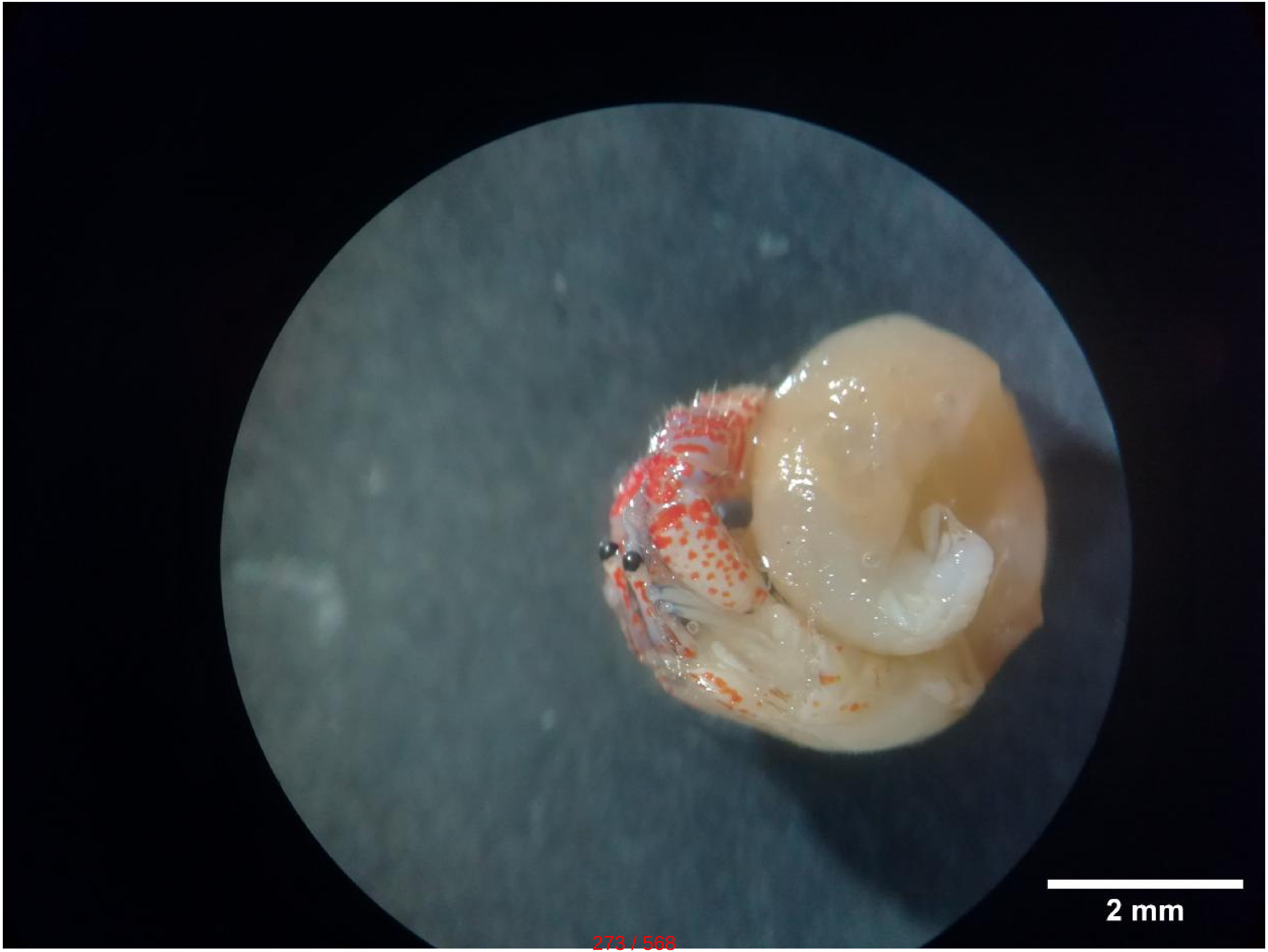
2 mm

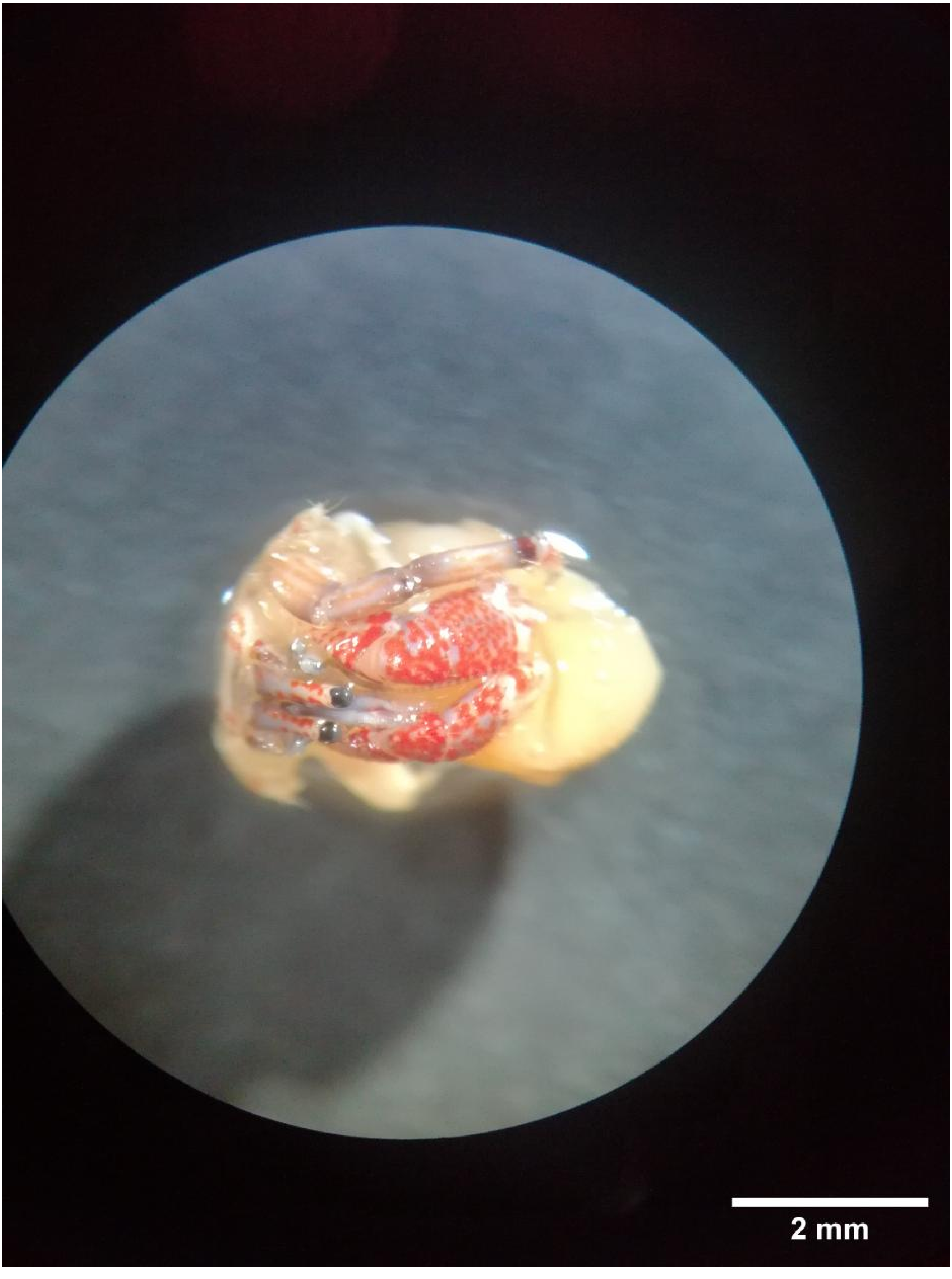
2 mm

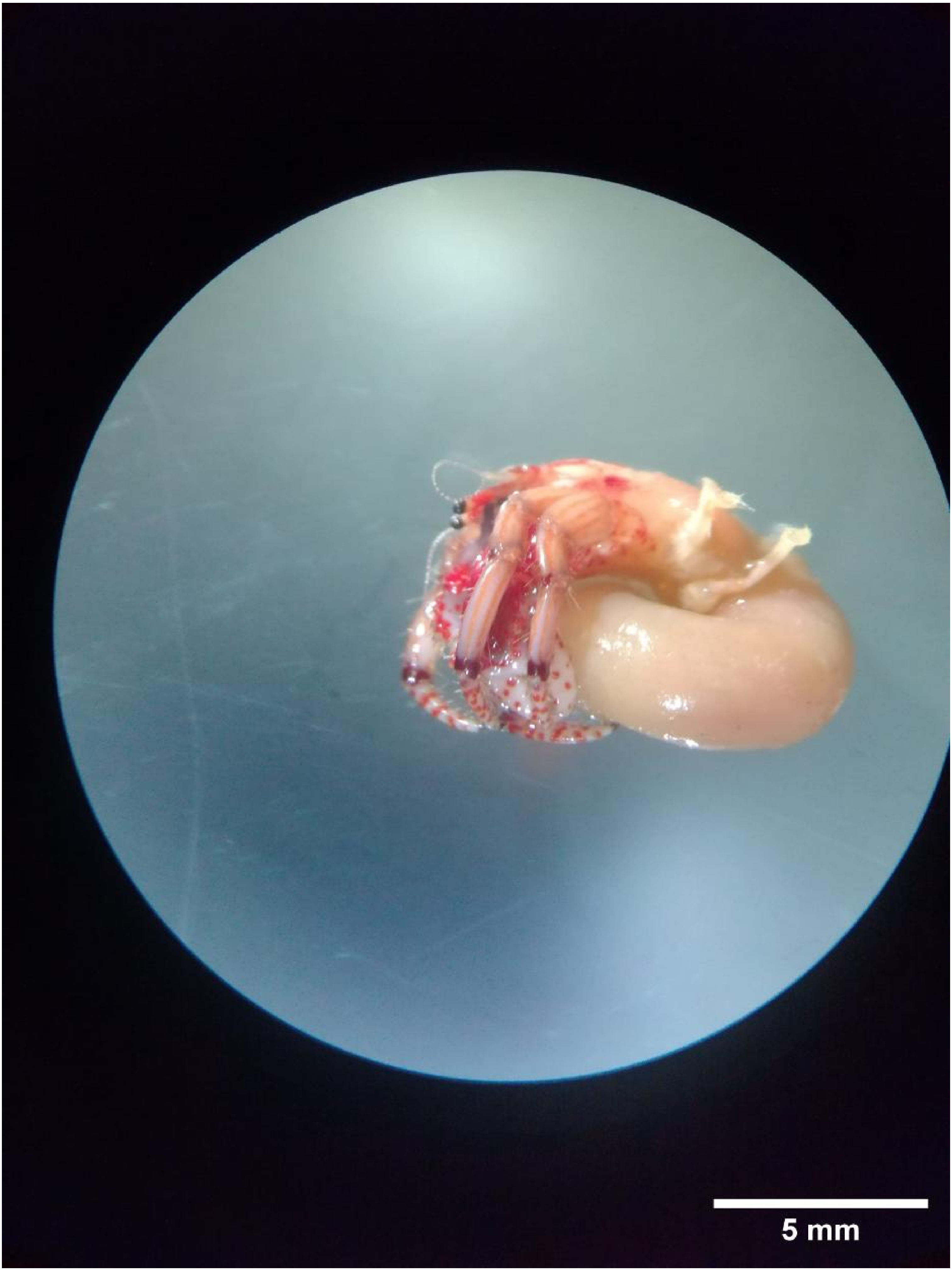
5 mm

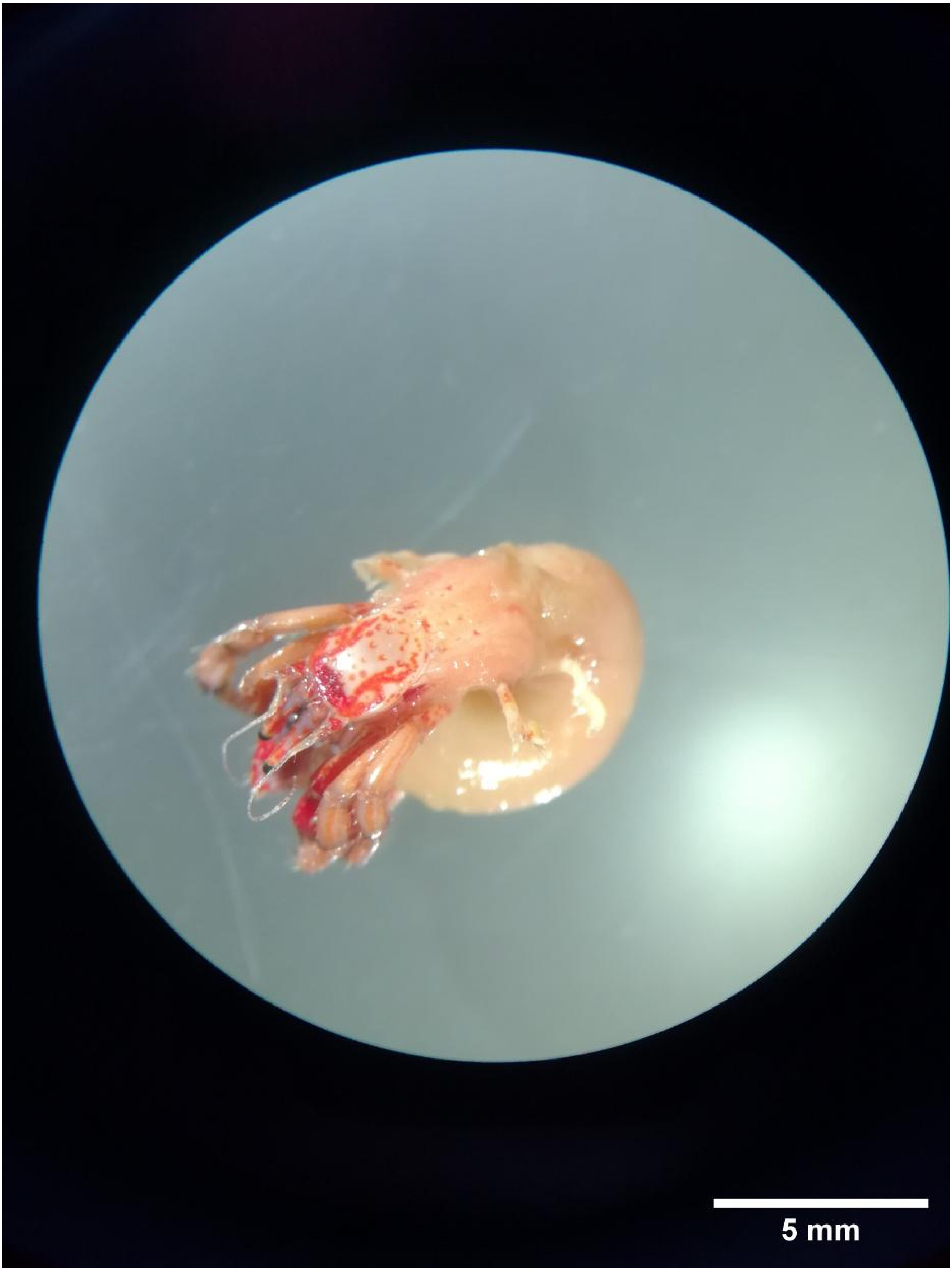
5 mm

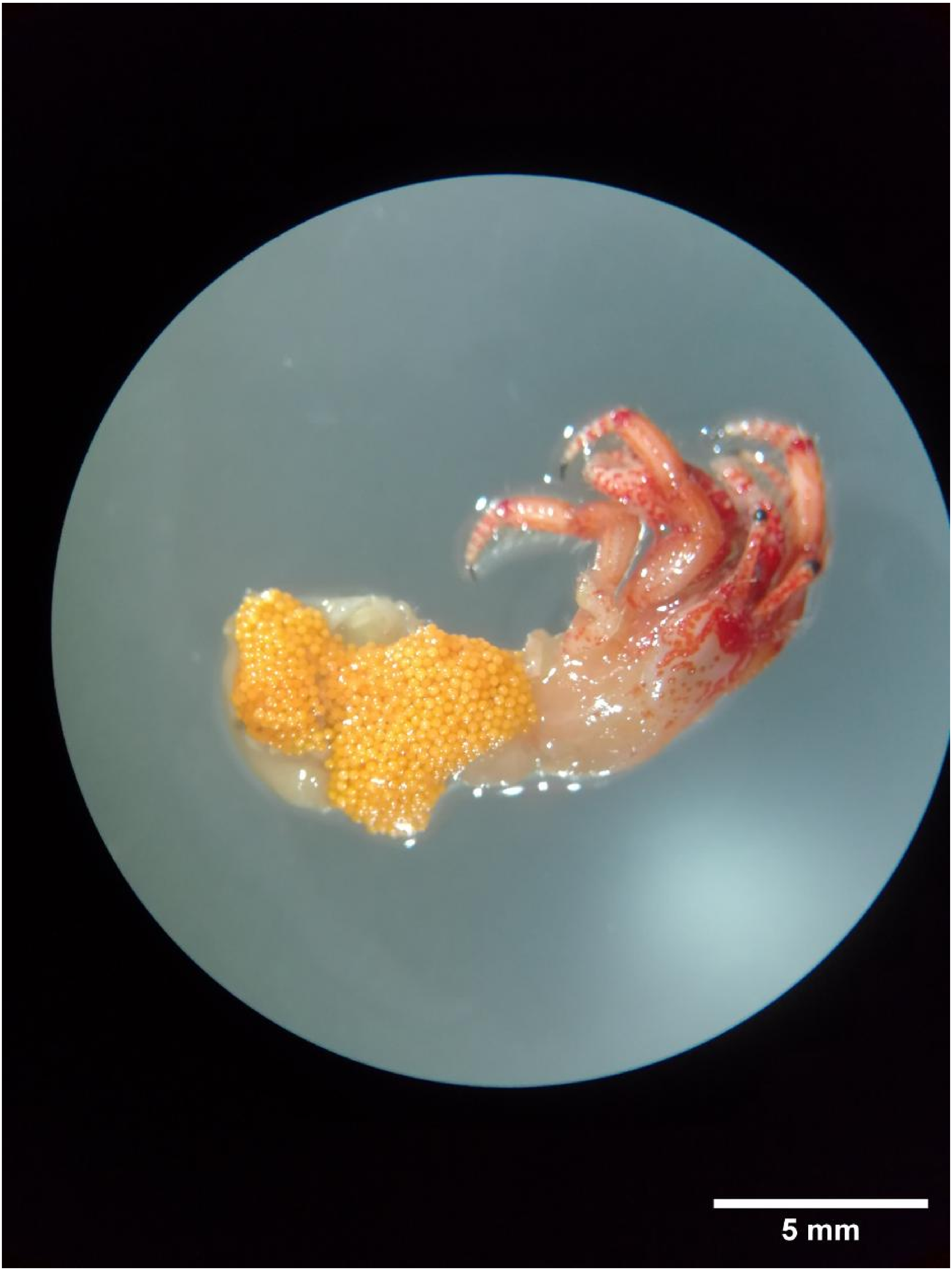
5 mm

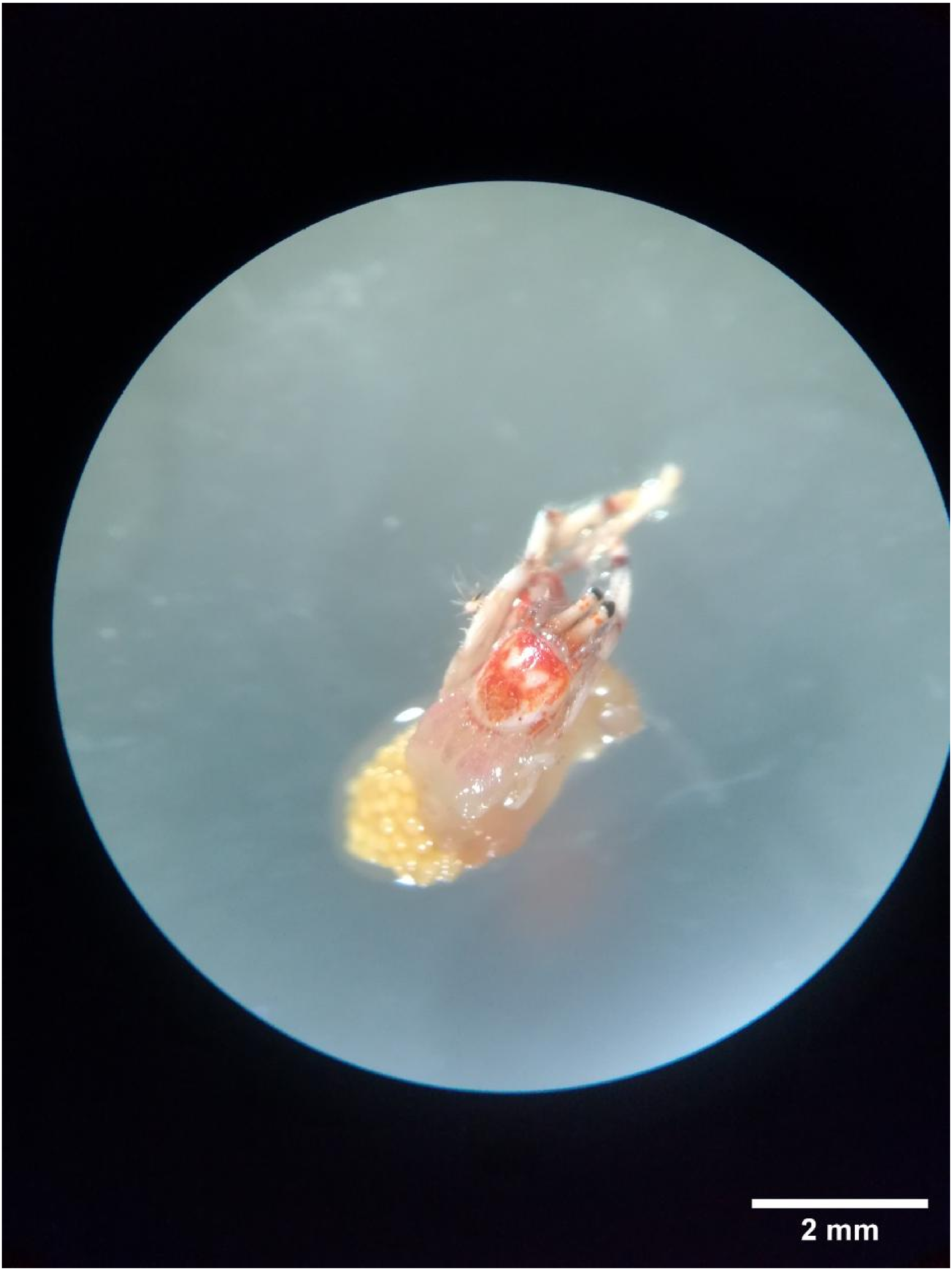
2 mm

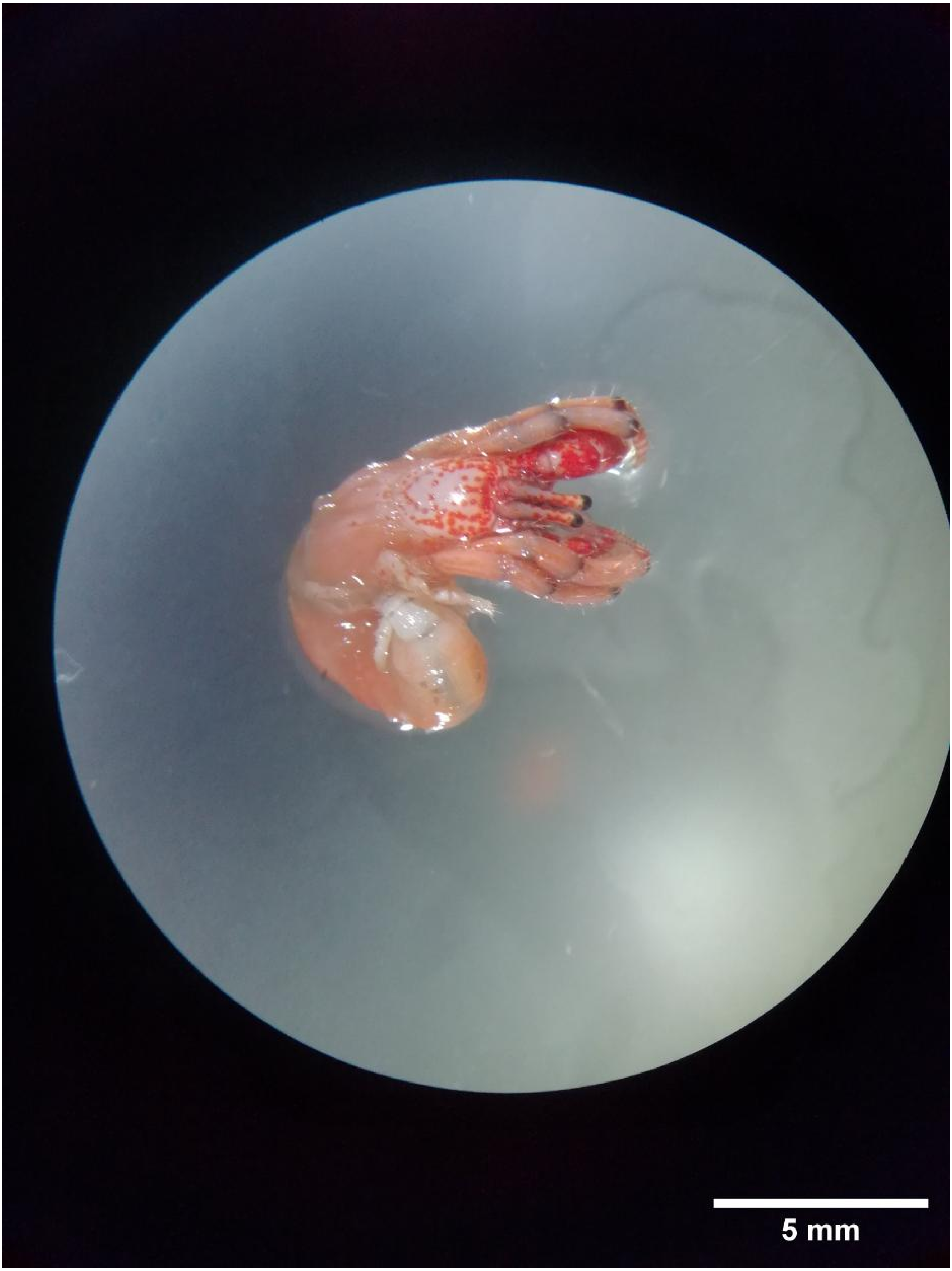
5 mm

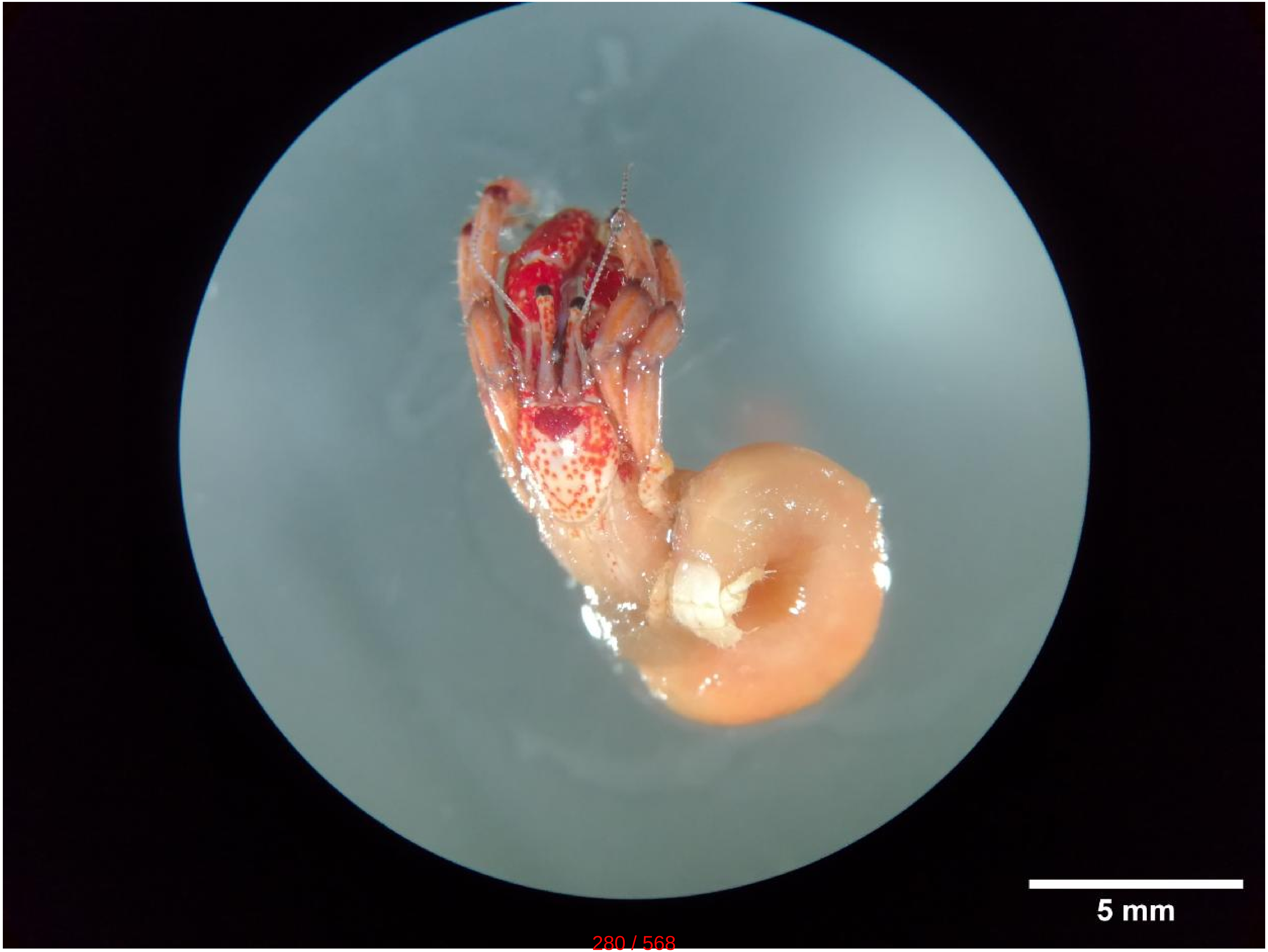
5 mm

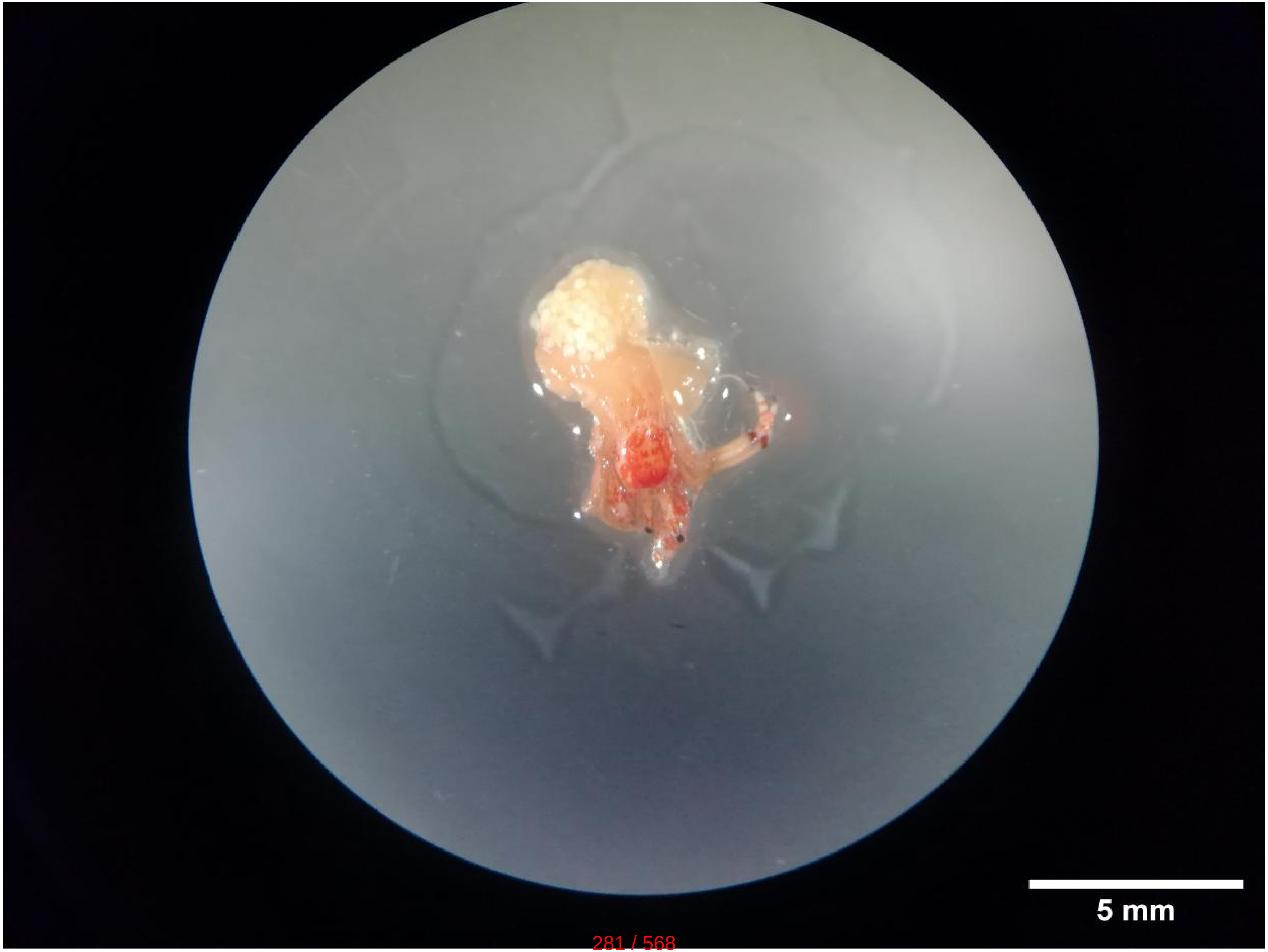
5 mm

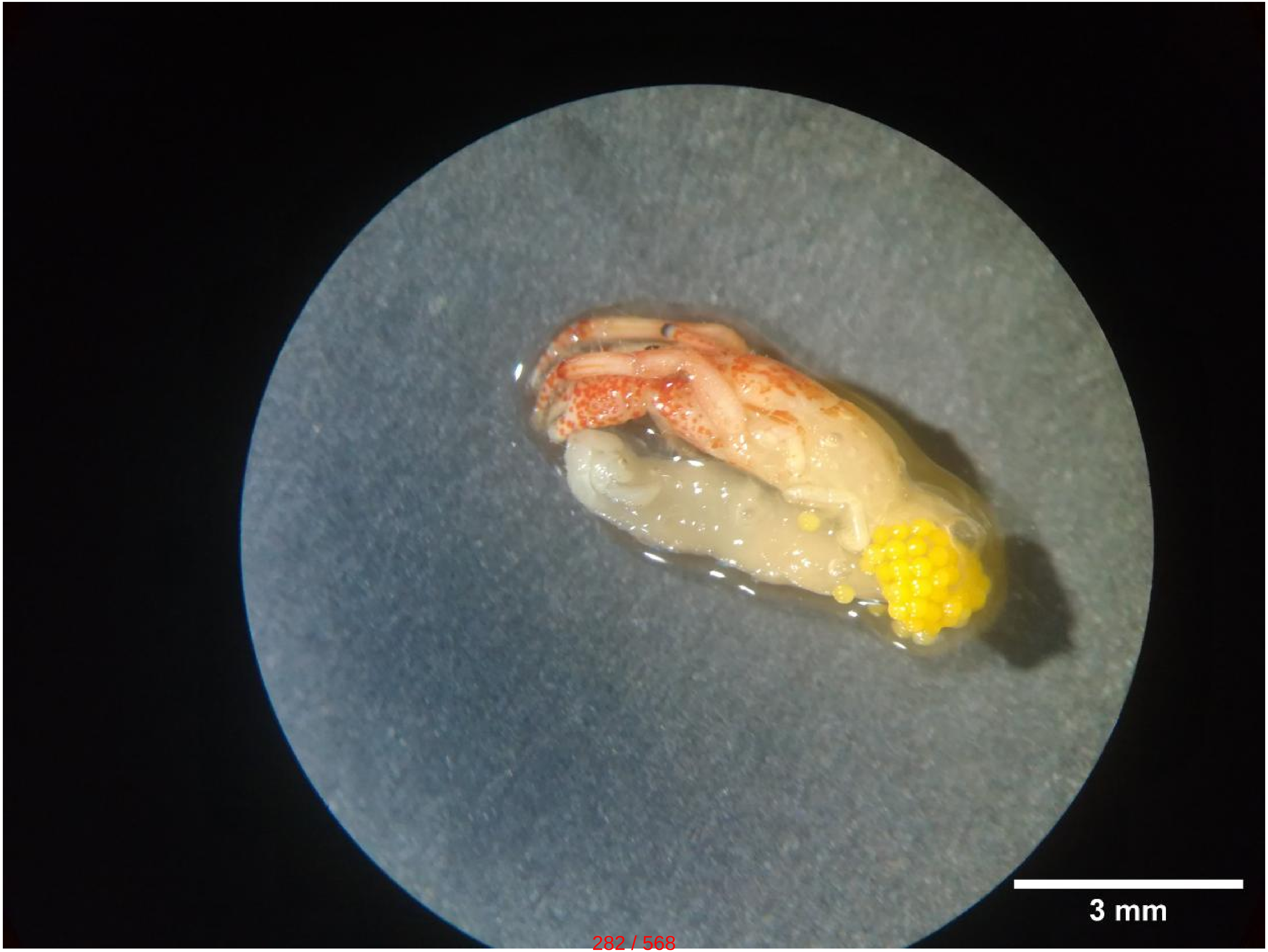
3 mm

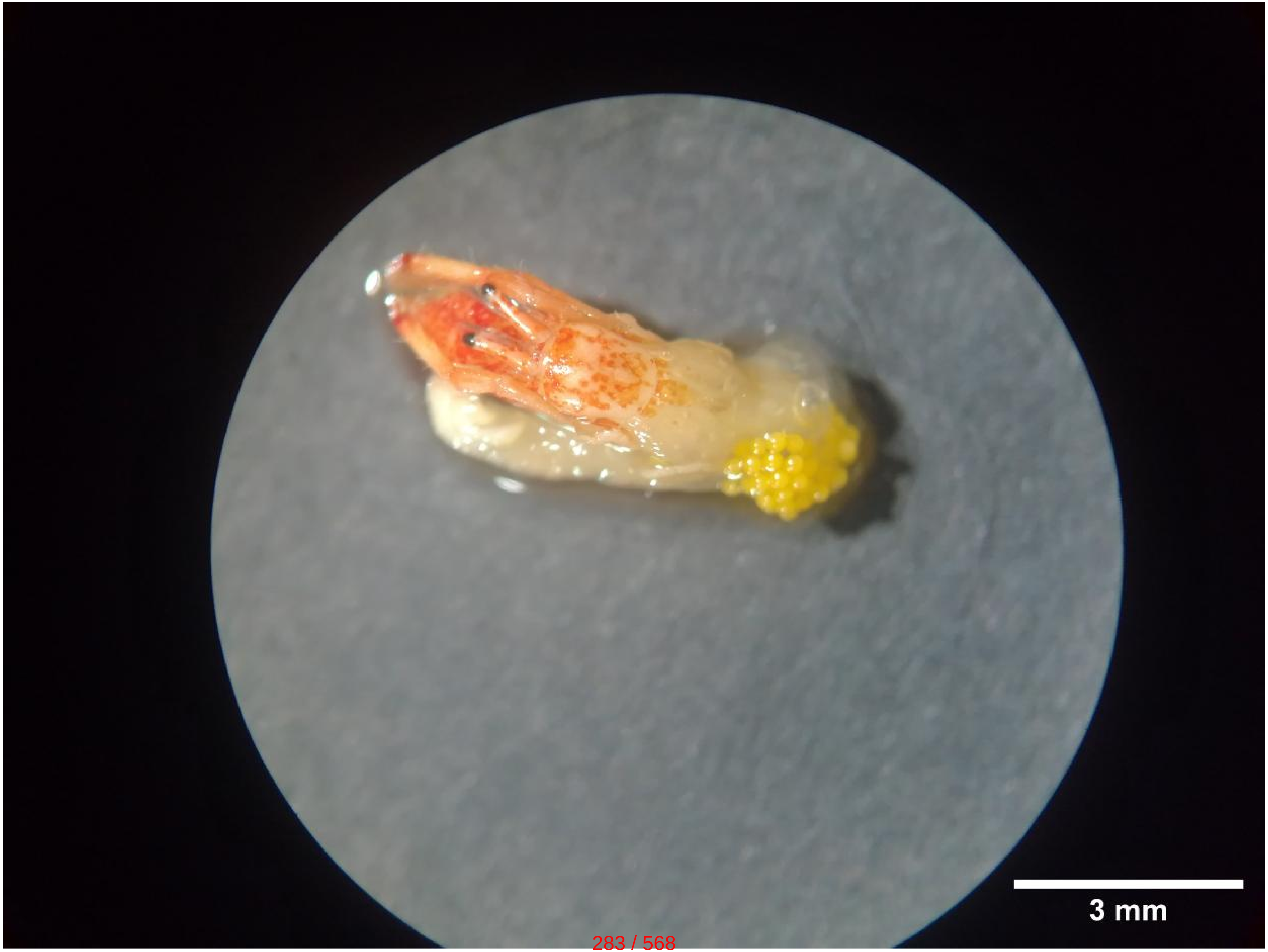
3 mm

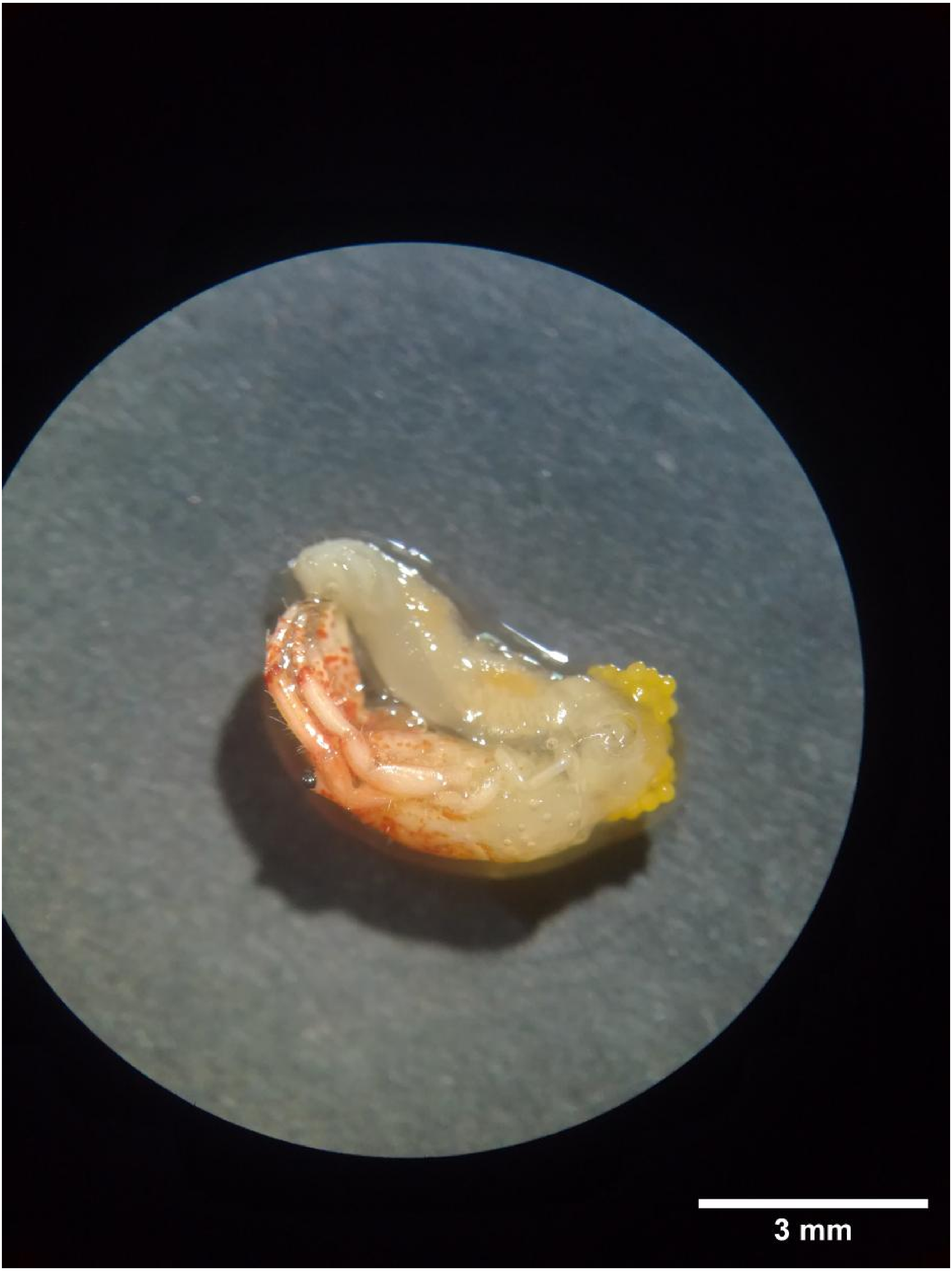
3 mm

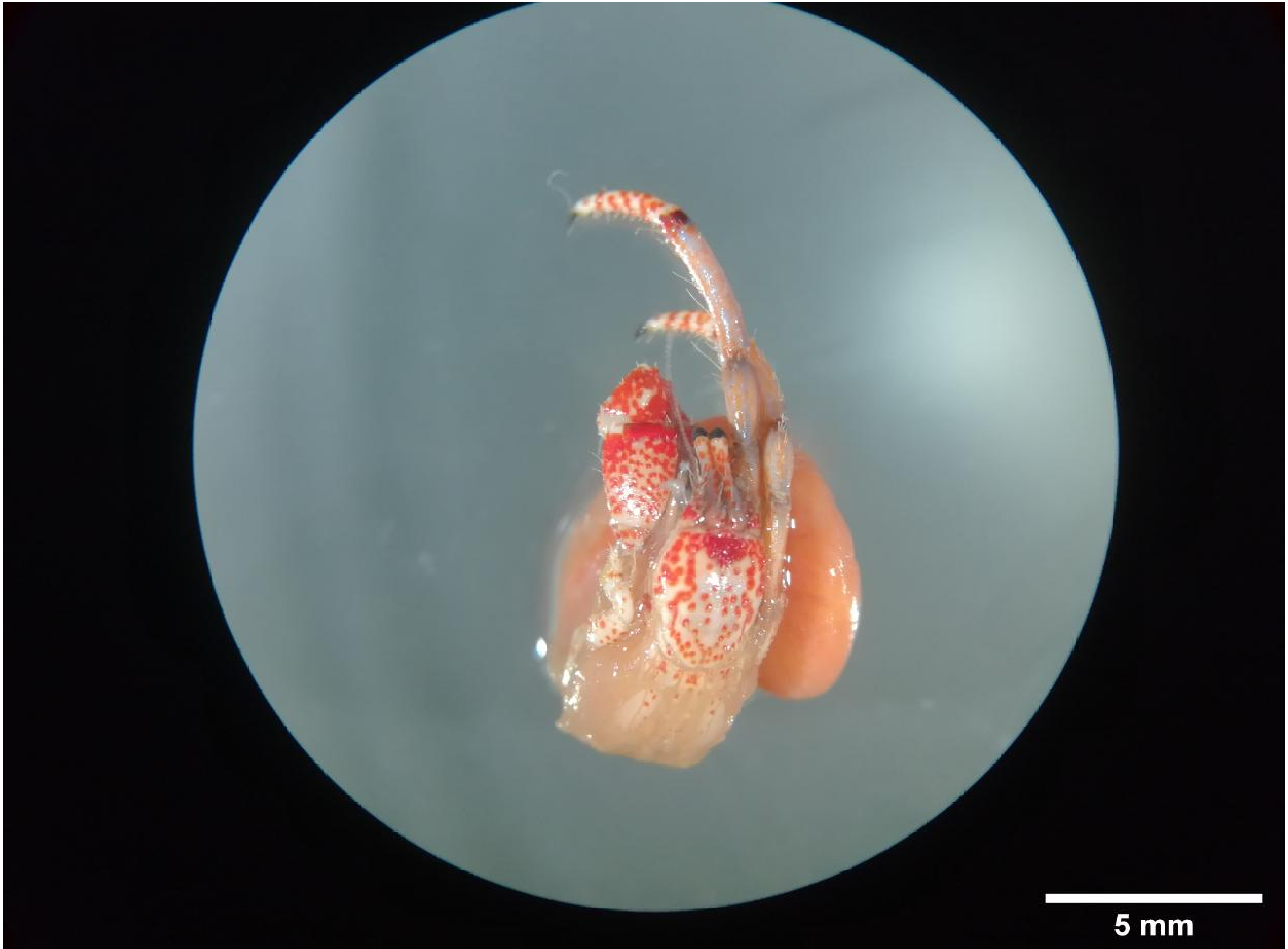
5 mm

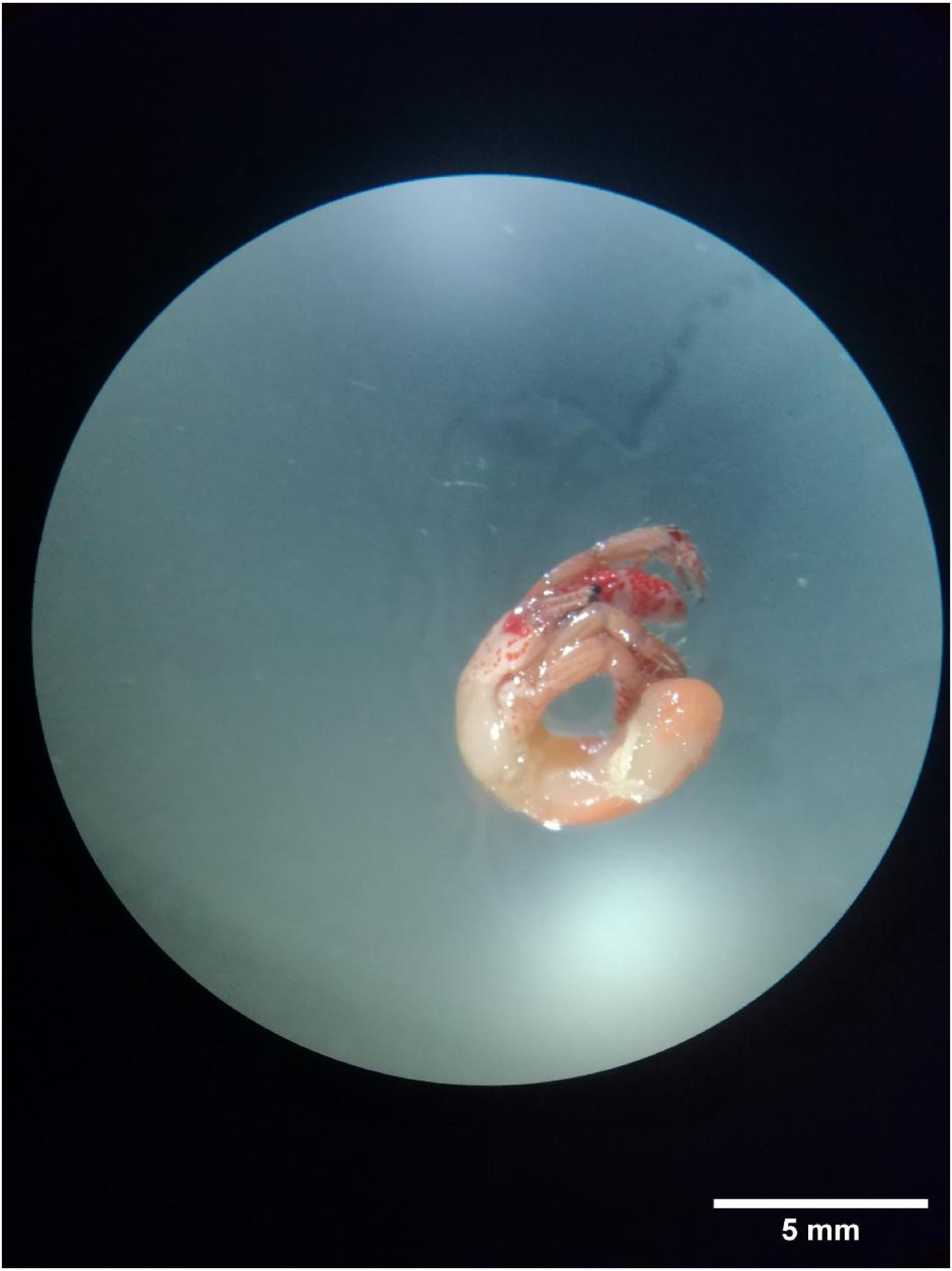
5 mm

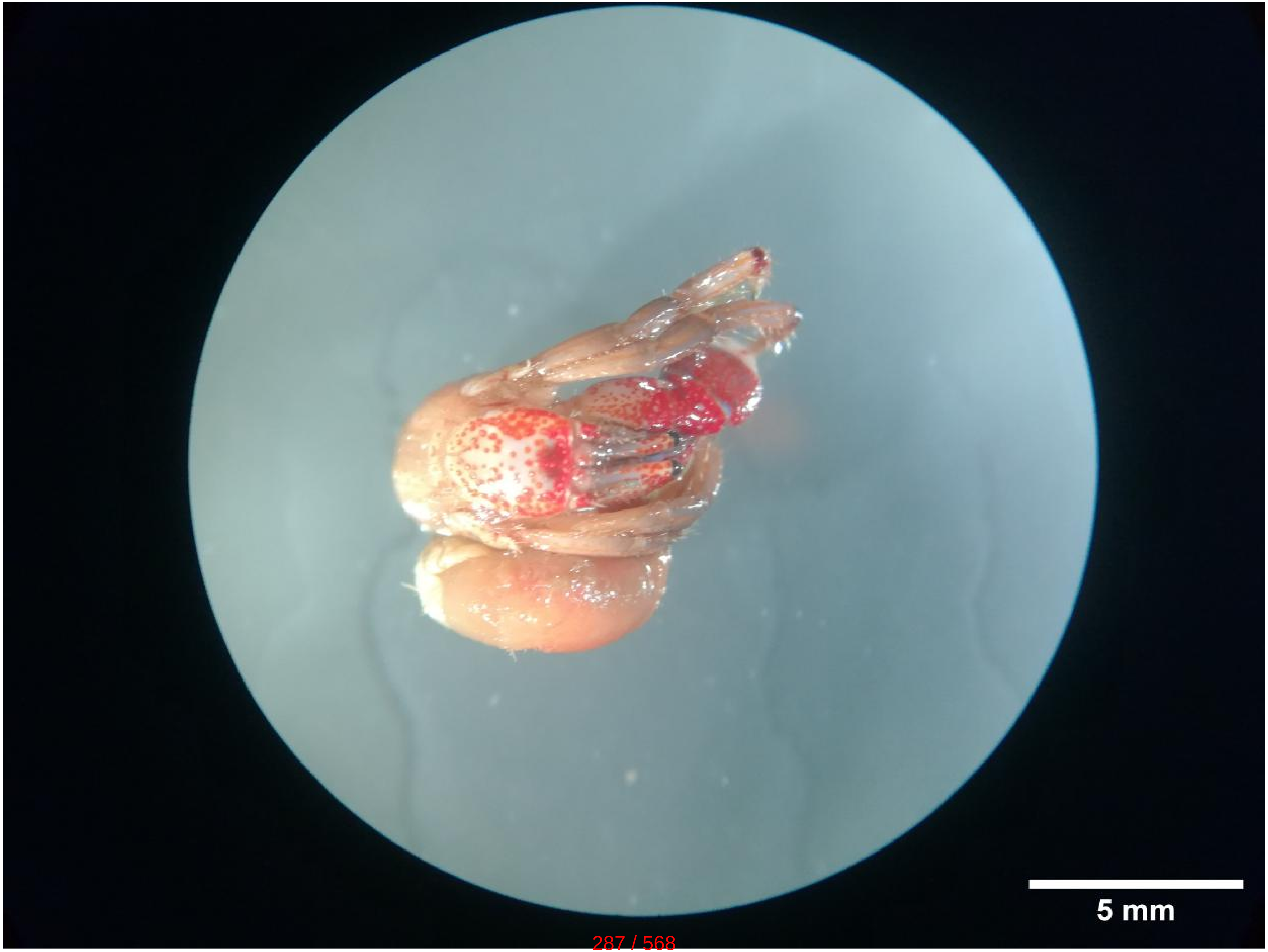
5 mm

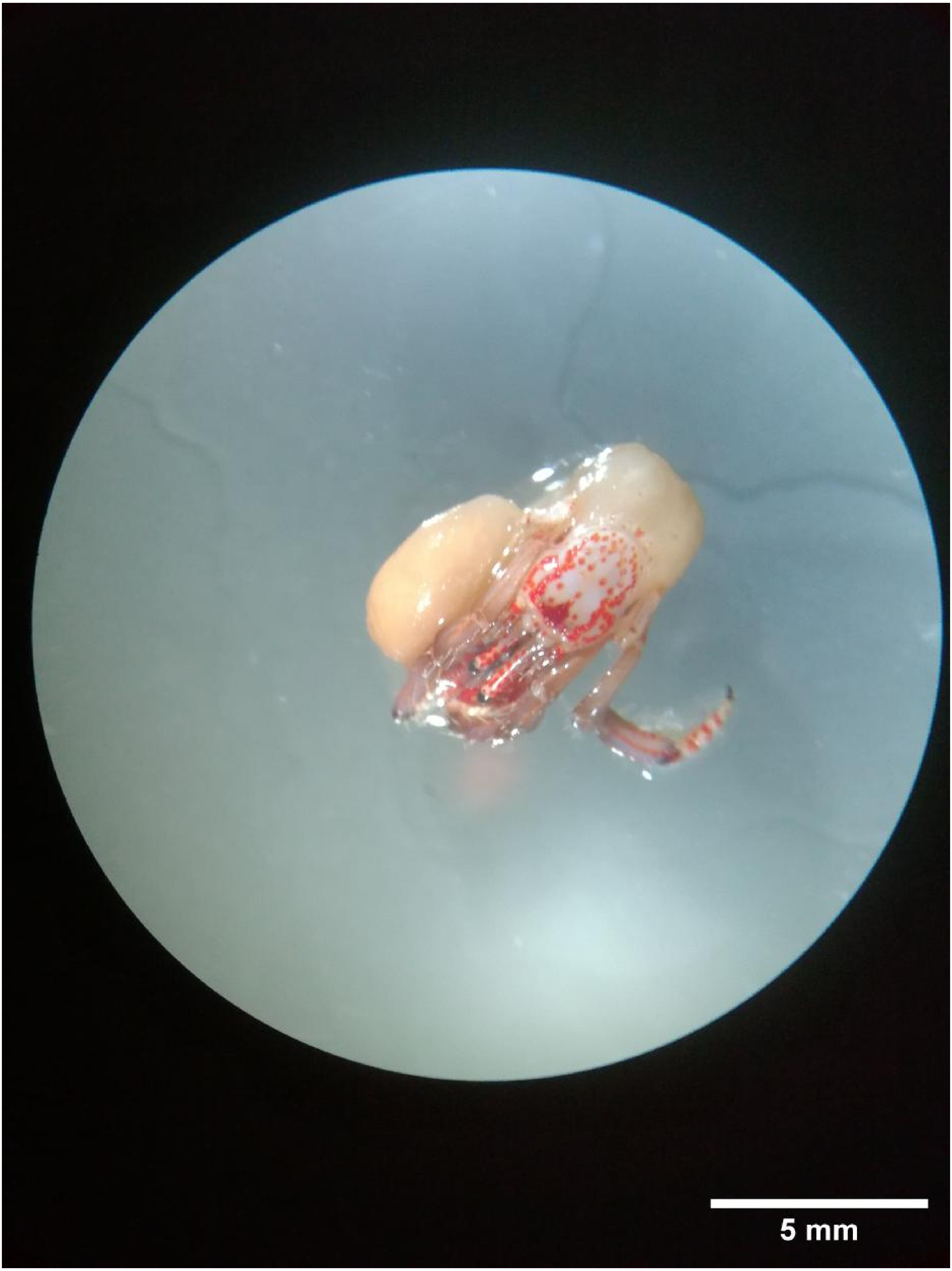
5 mm

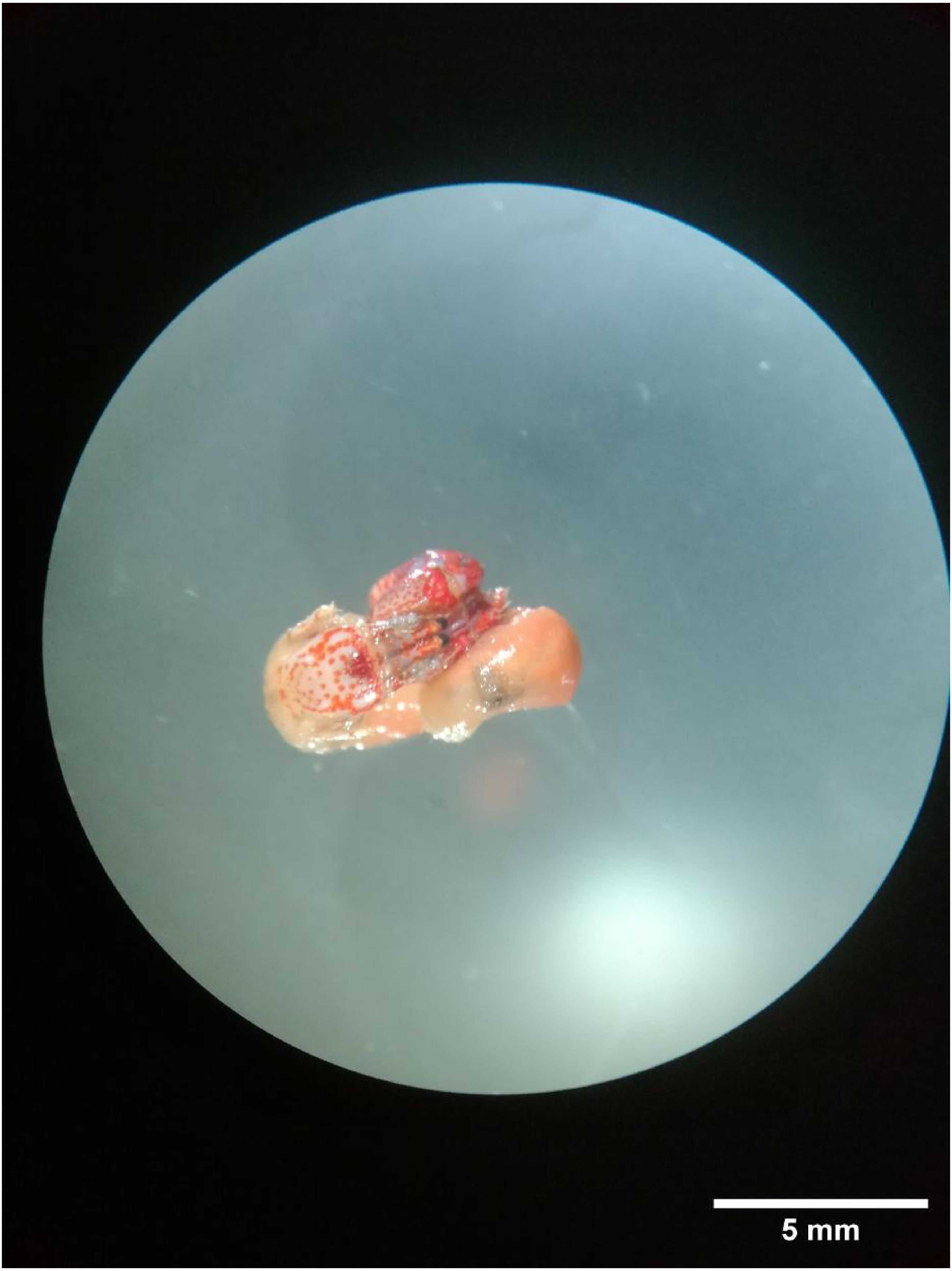
5 mm

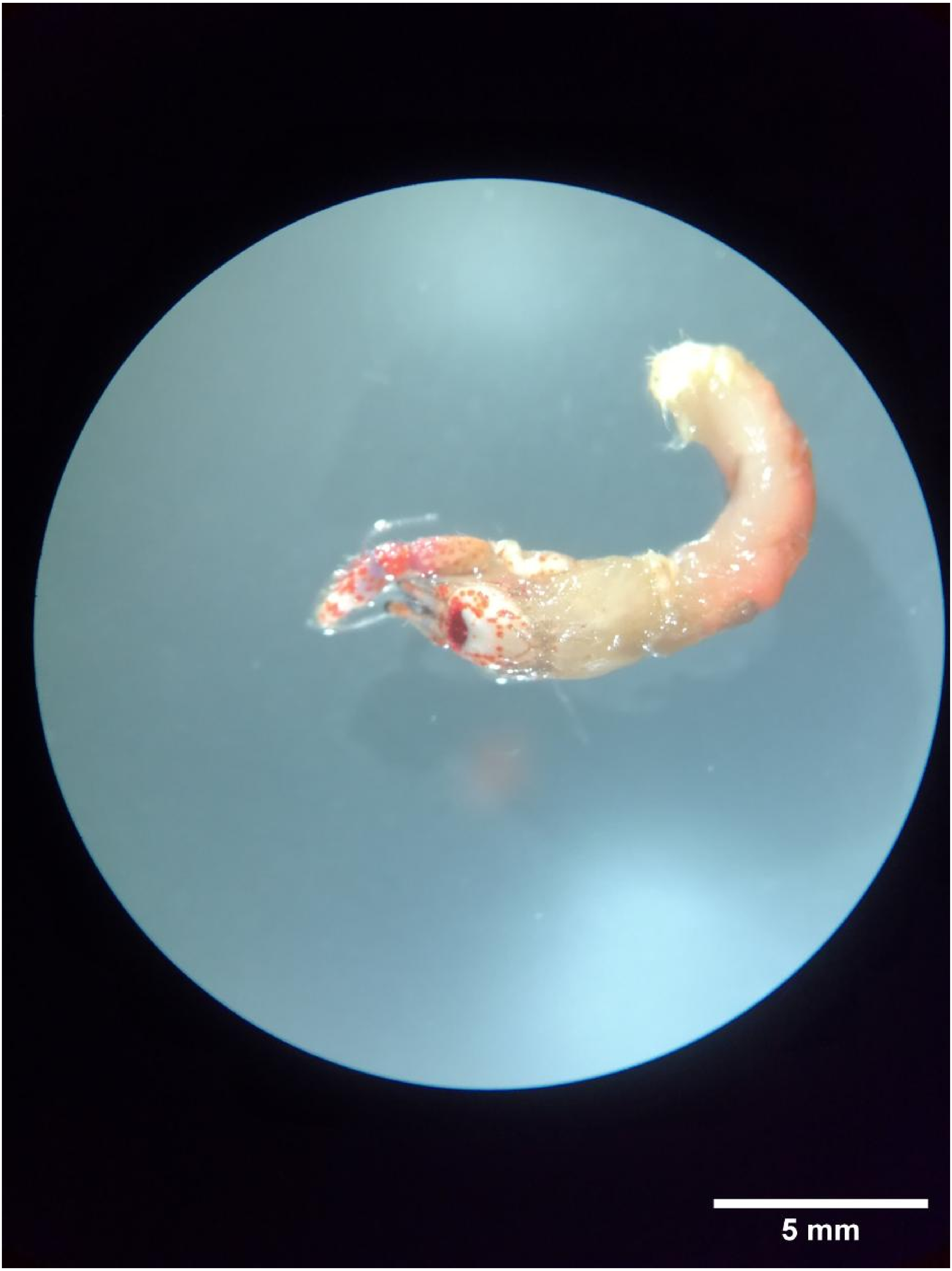
5 mm

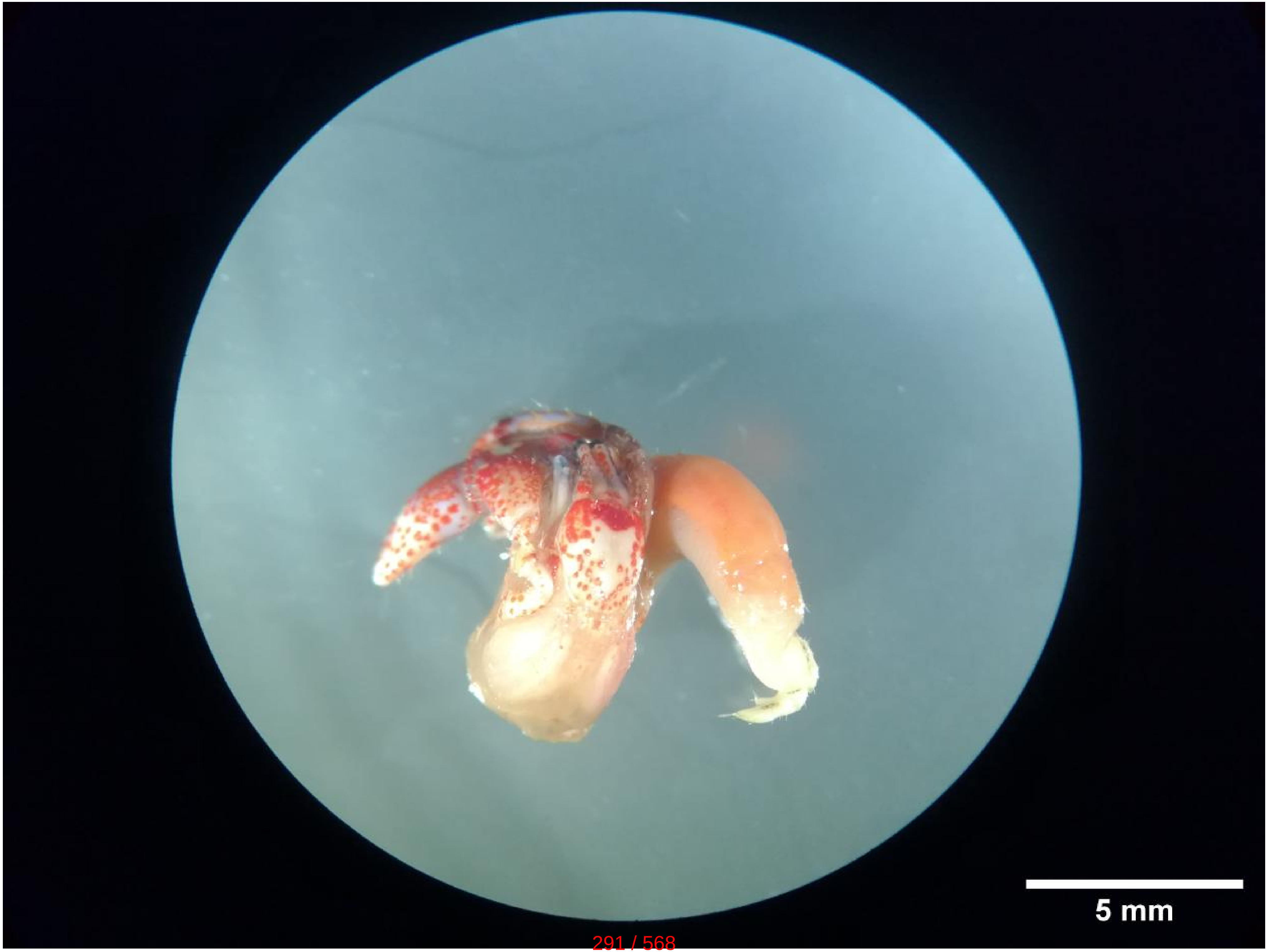
5 mm

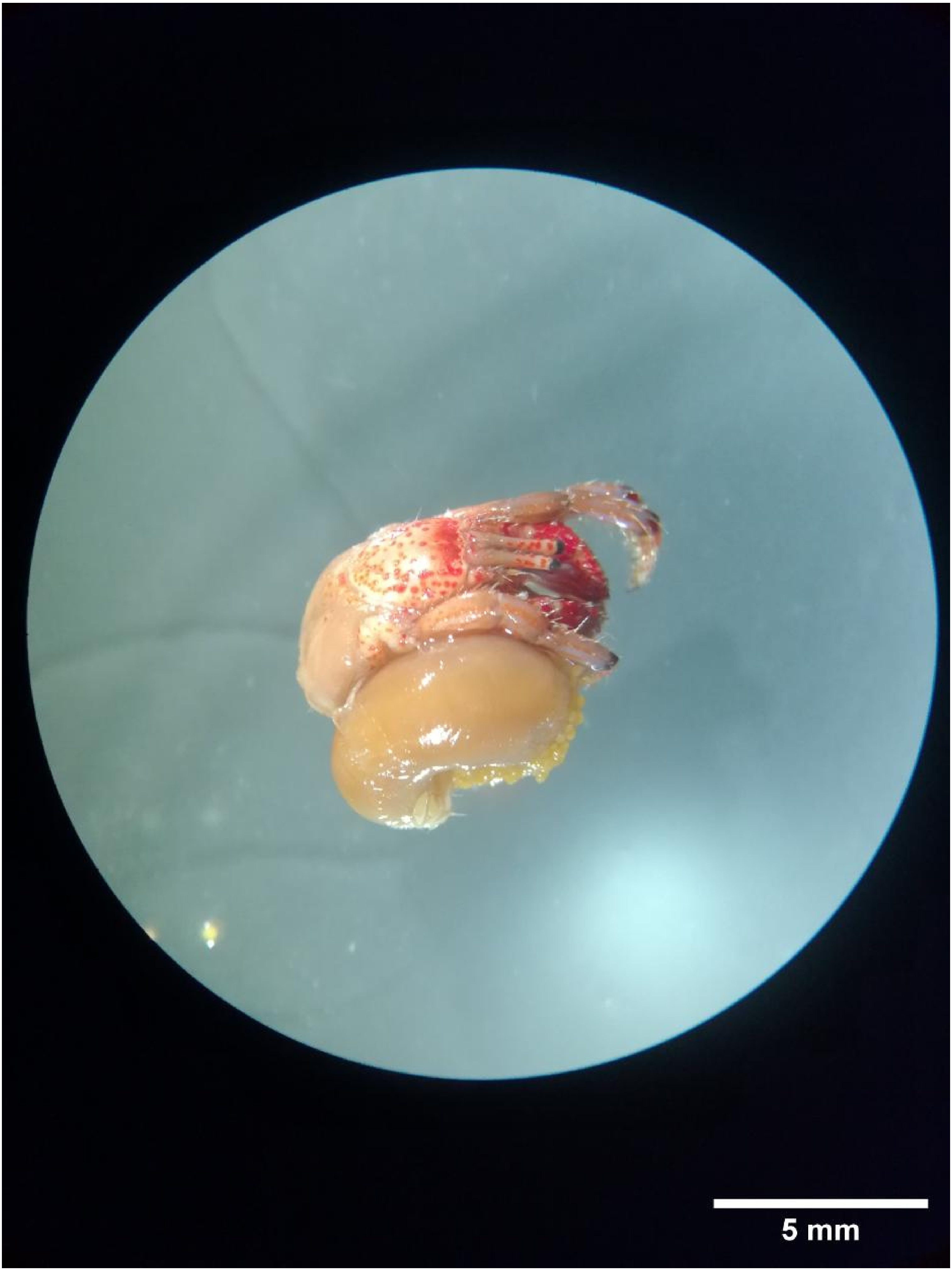
5 mm

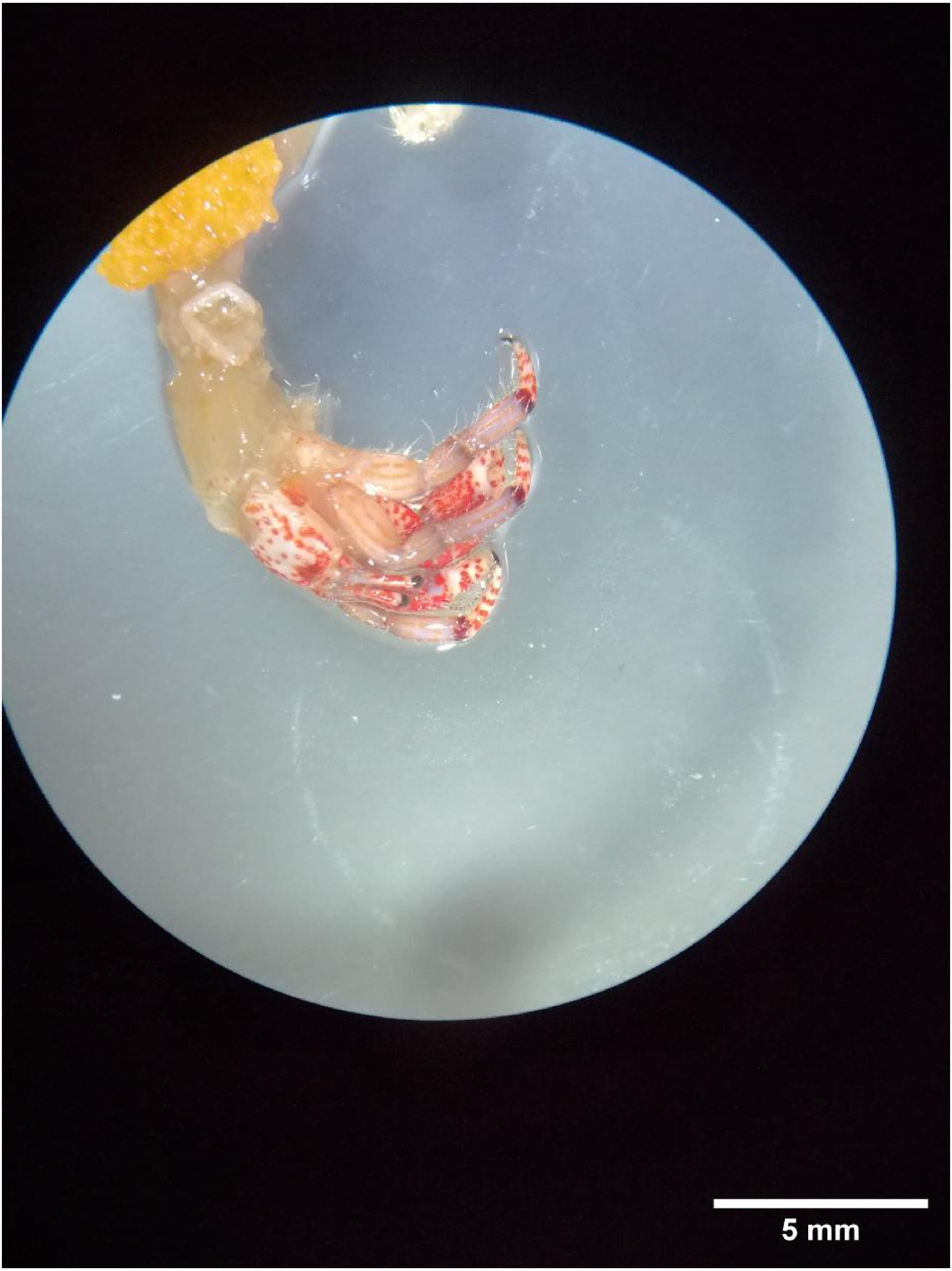
5 mm

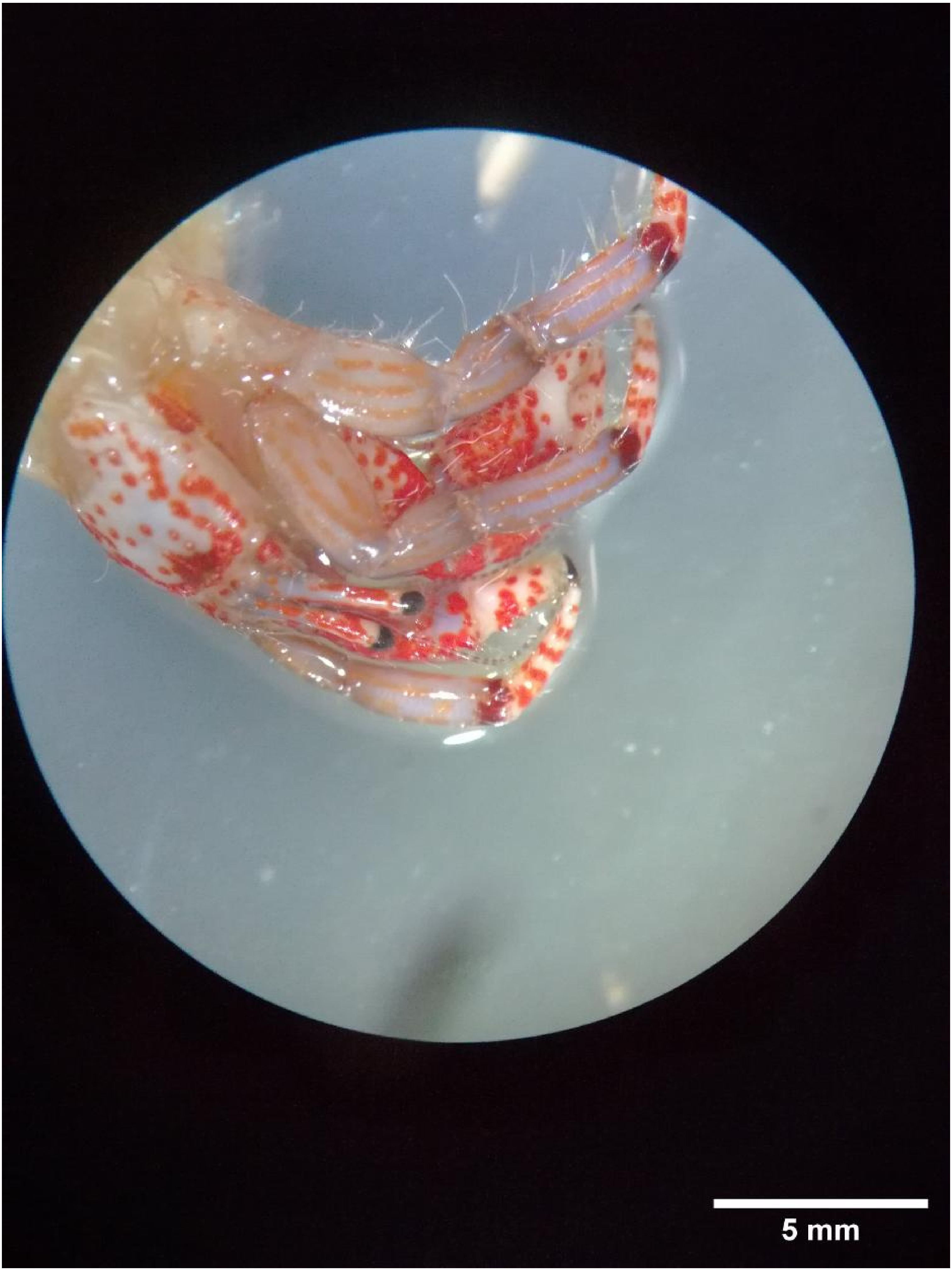
5 mm

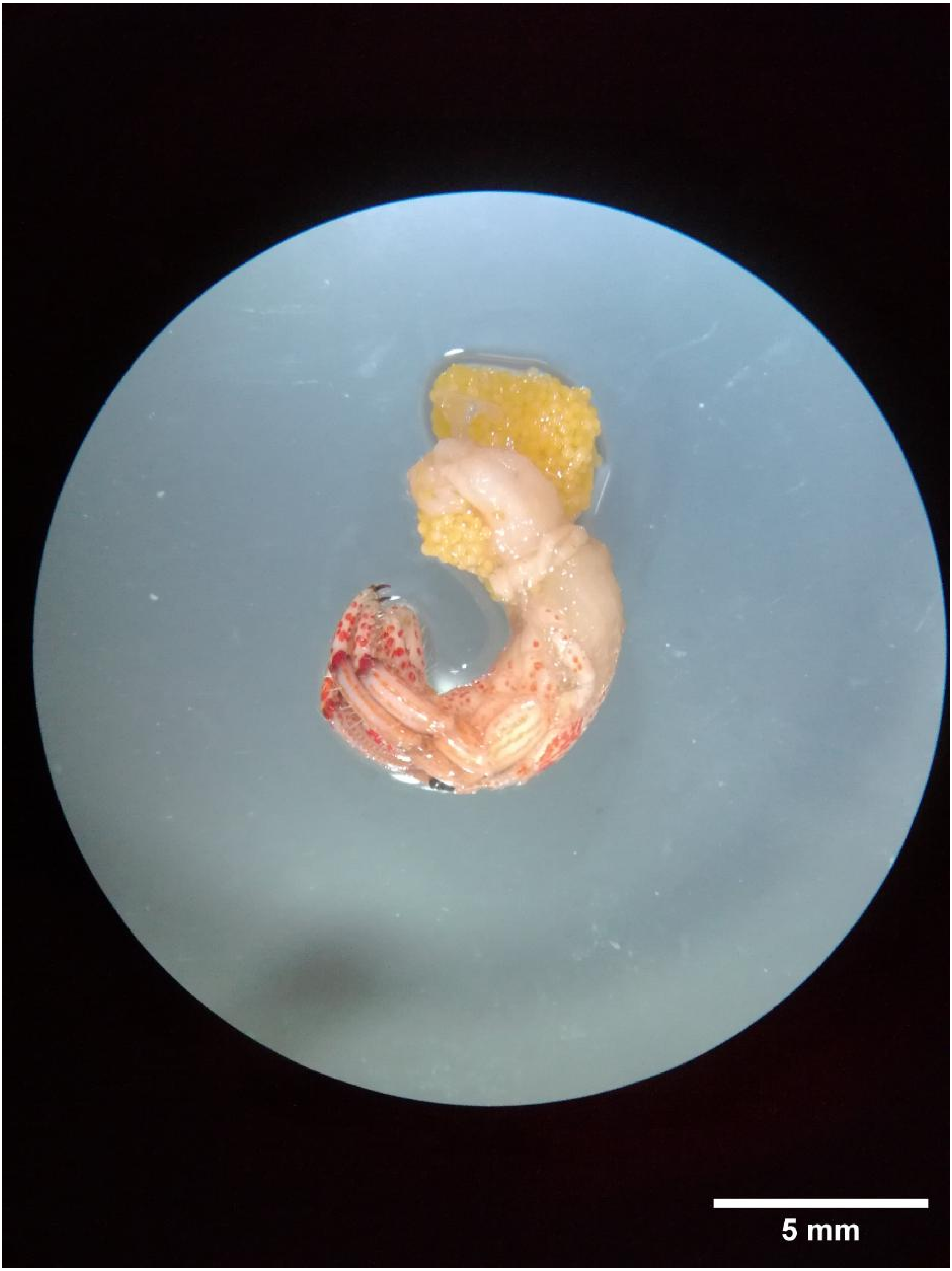
5 mm

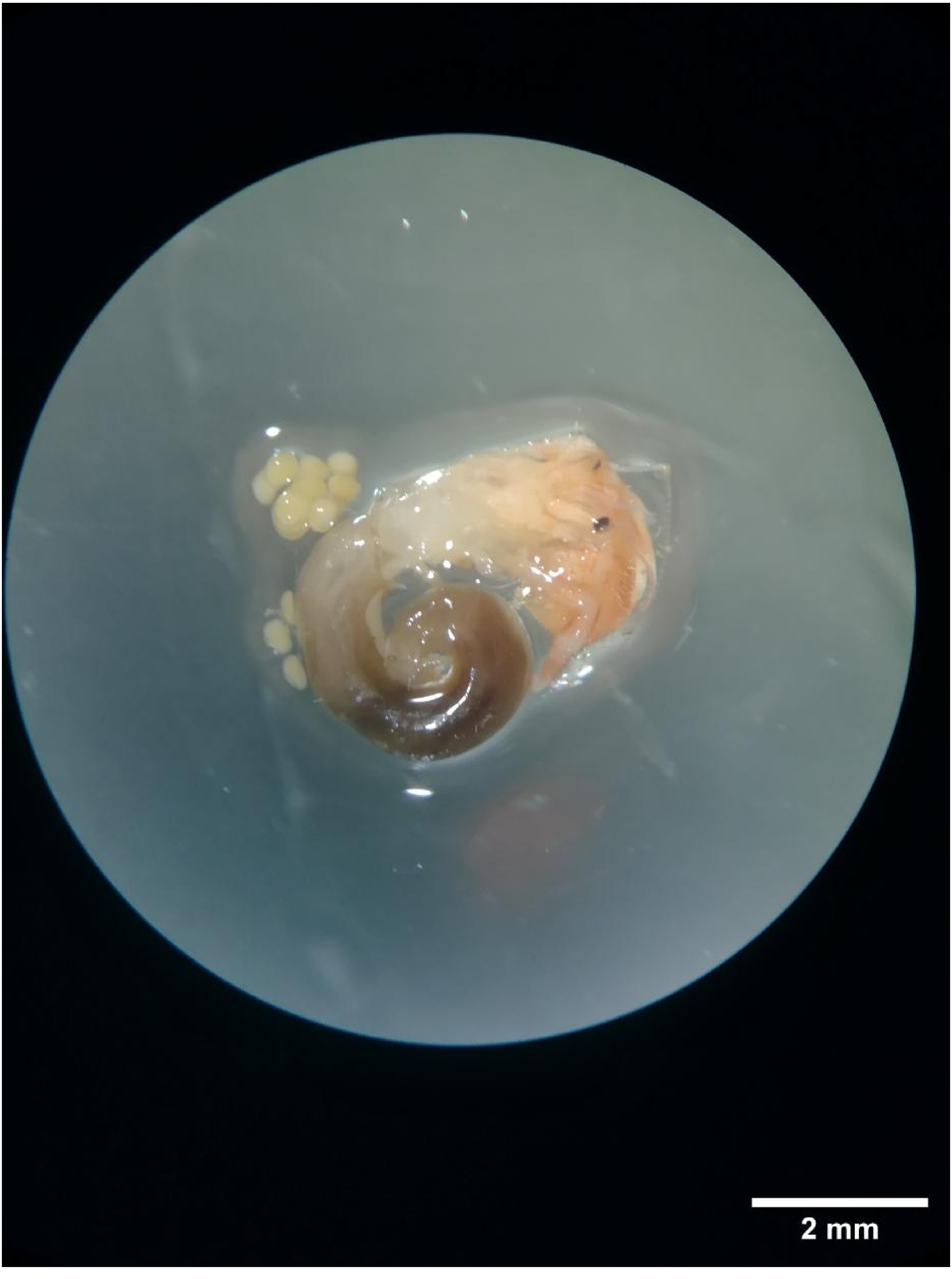
2 mm

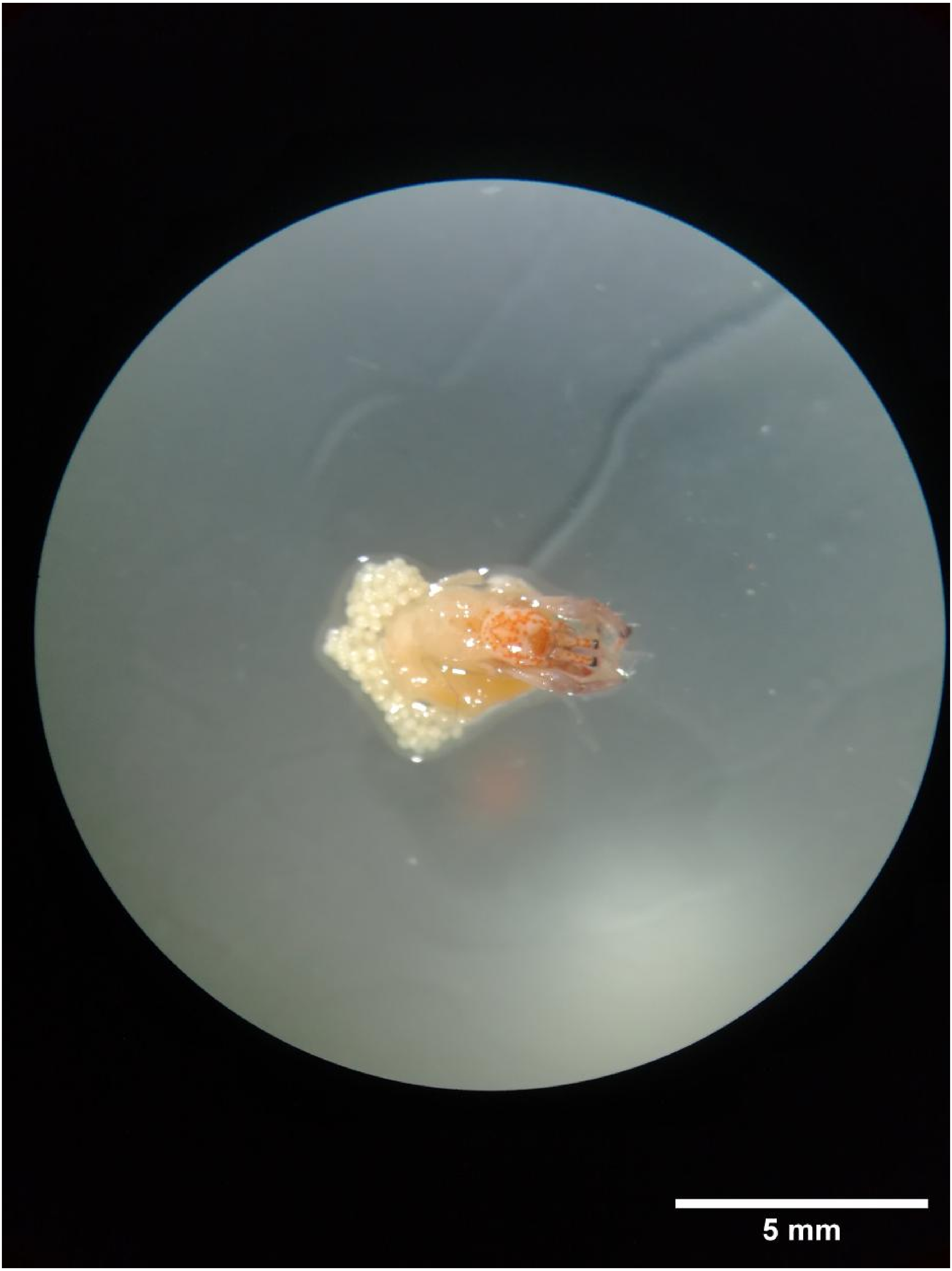
5 mm

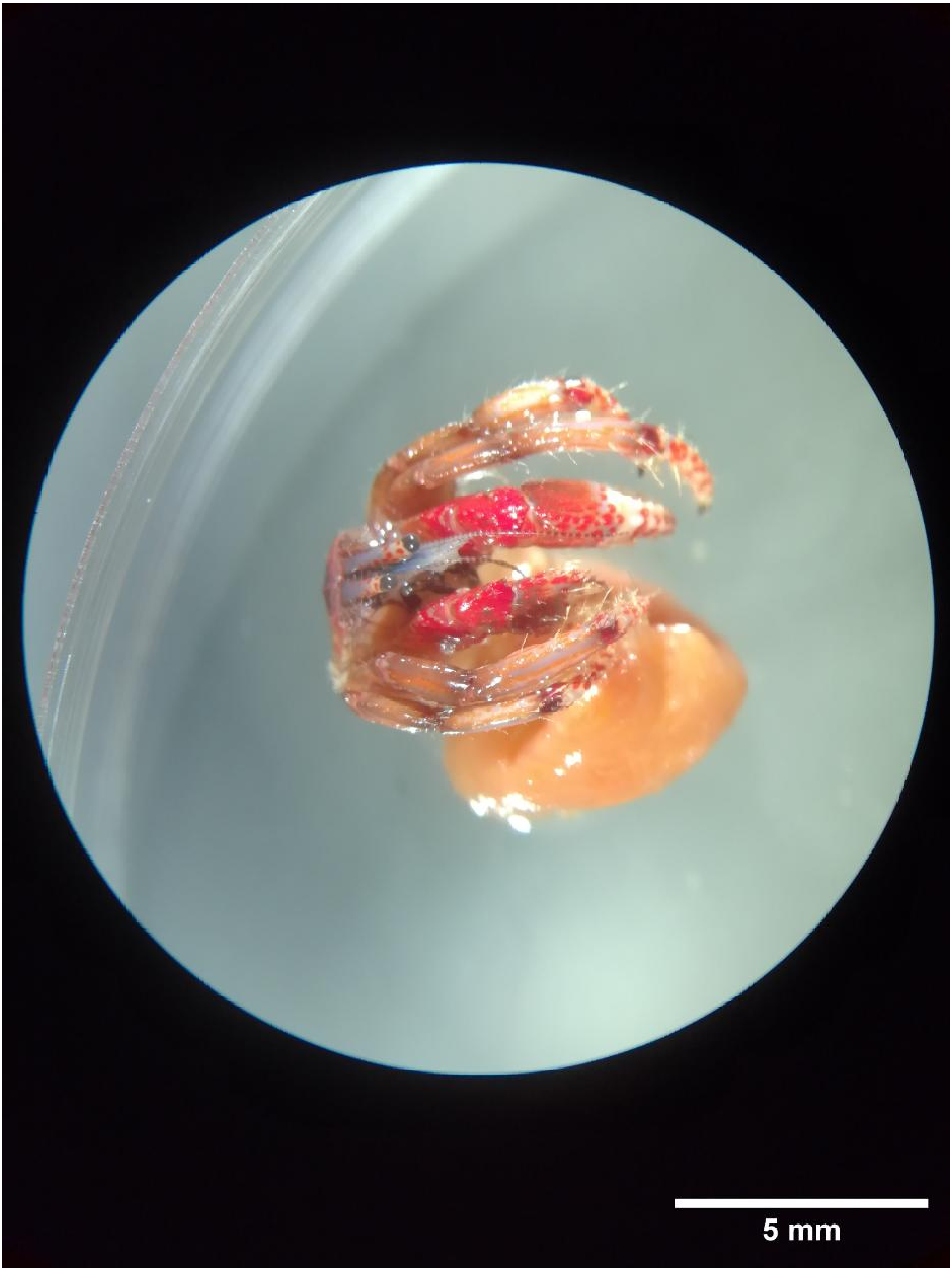
5 mm

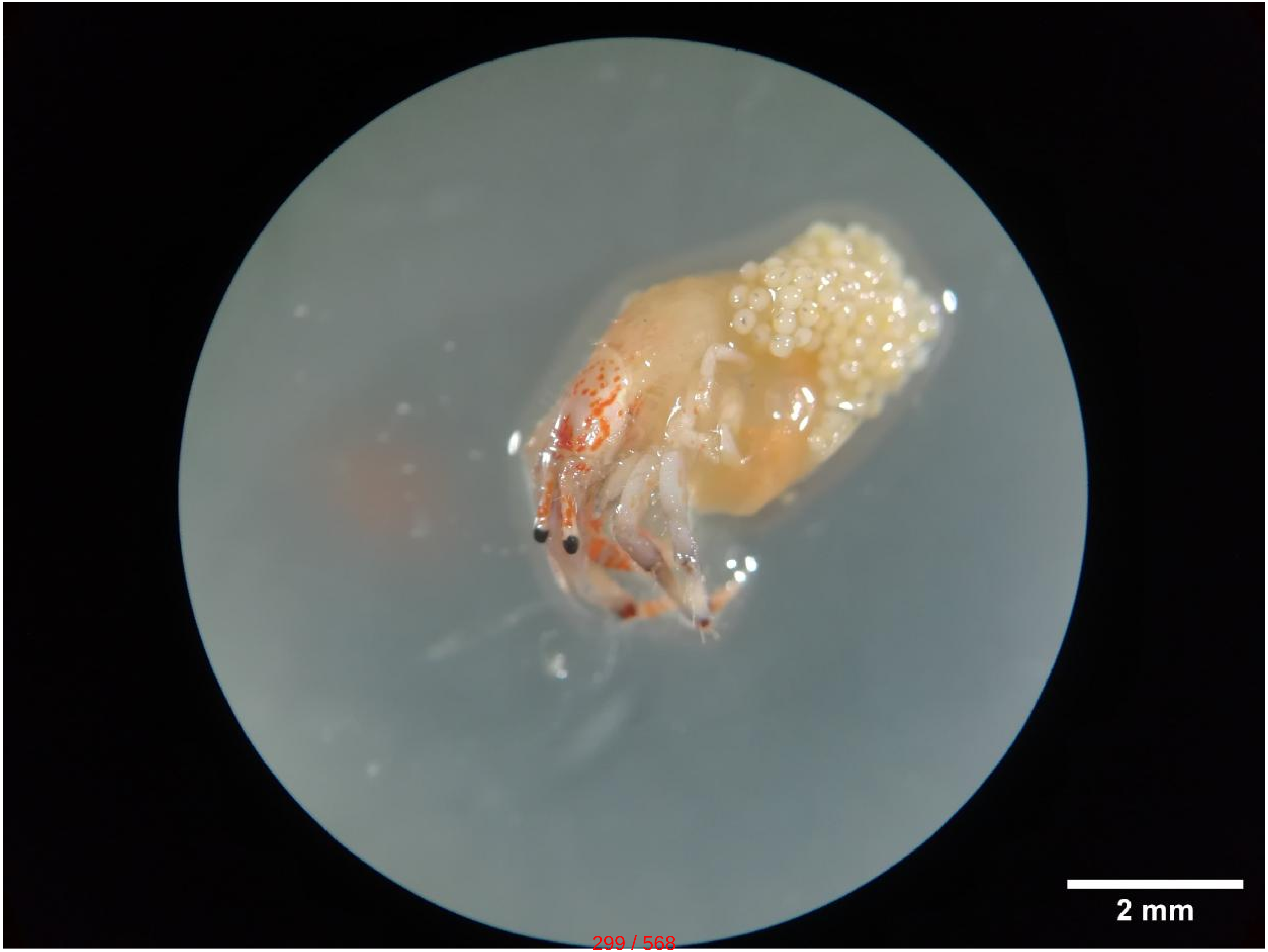
2 mm

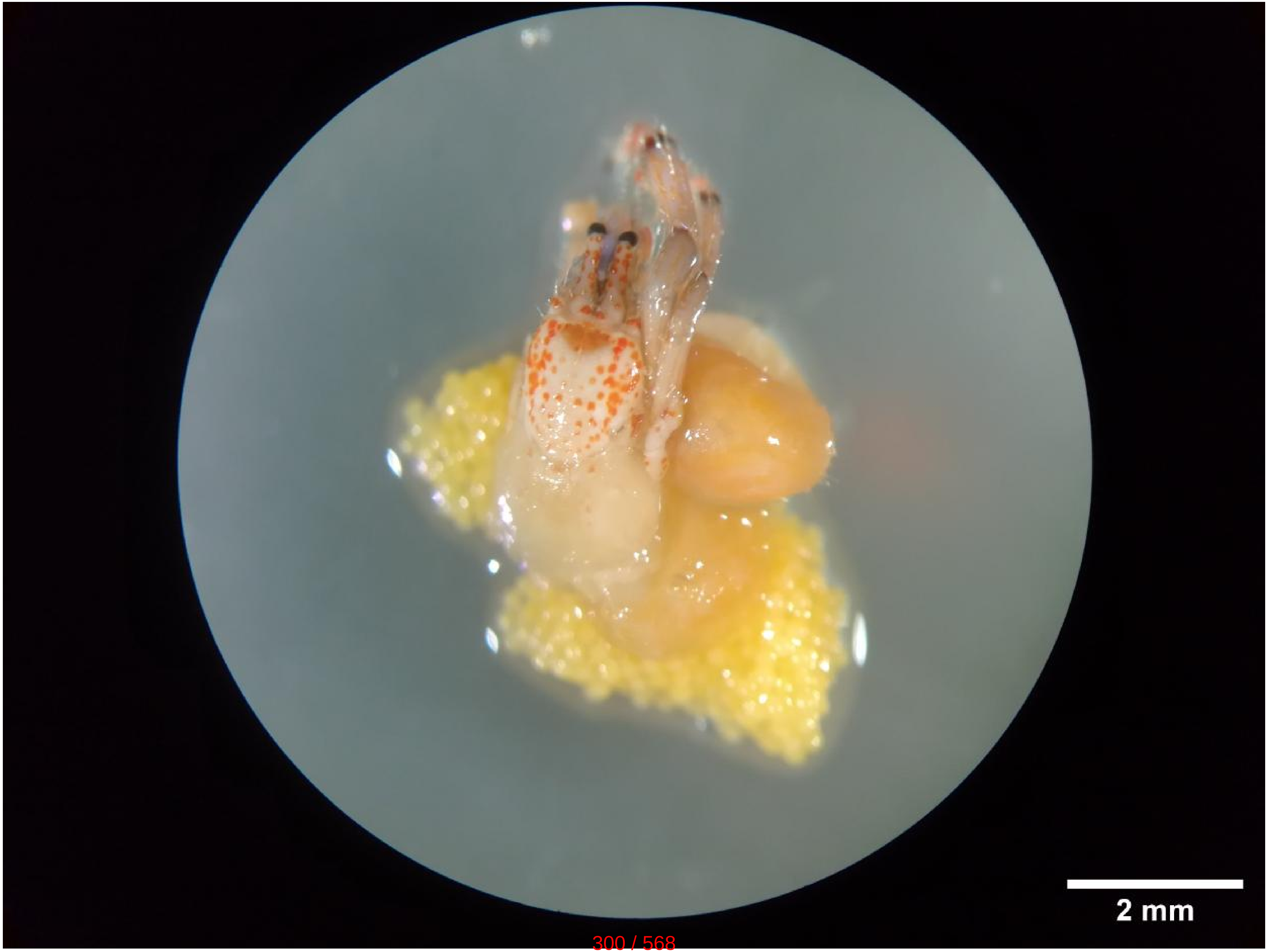
2 mm

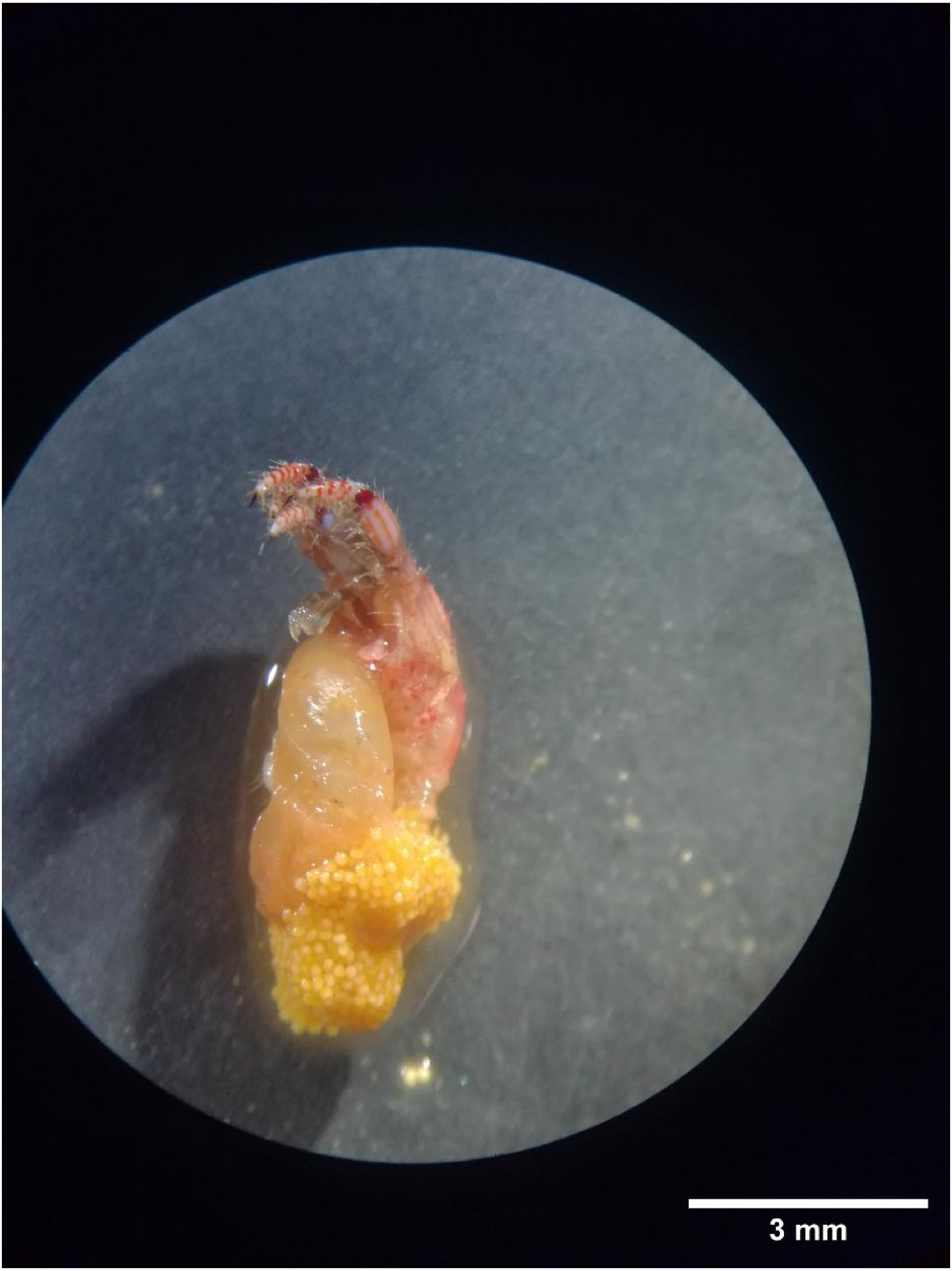
3 mm

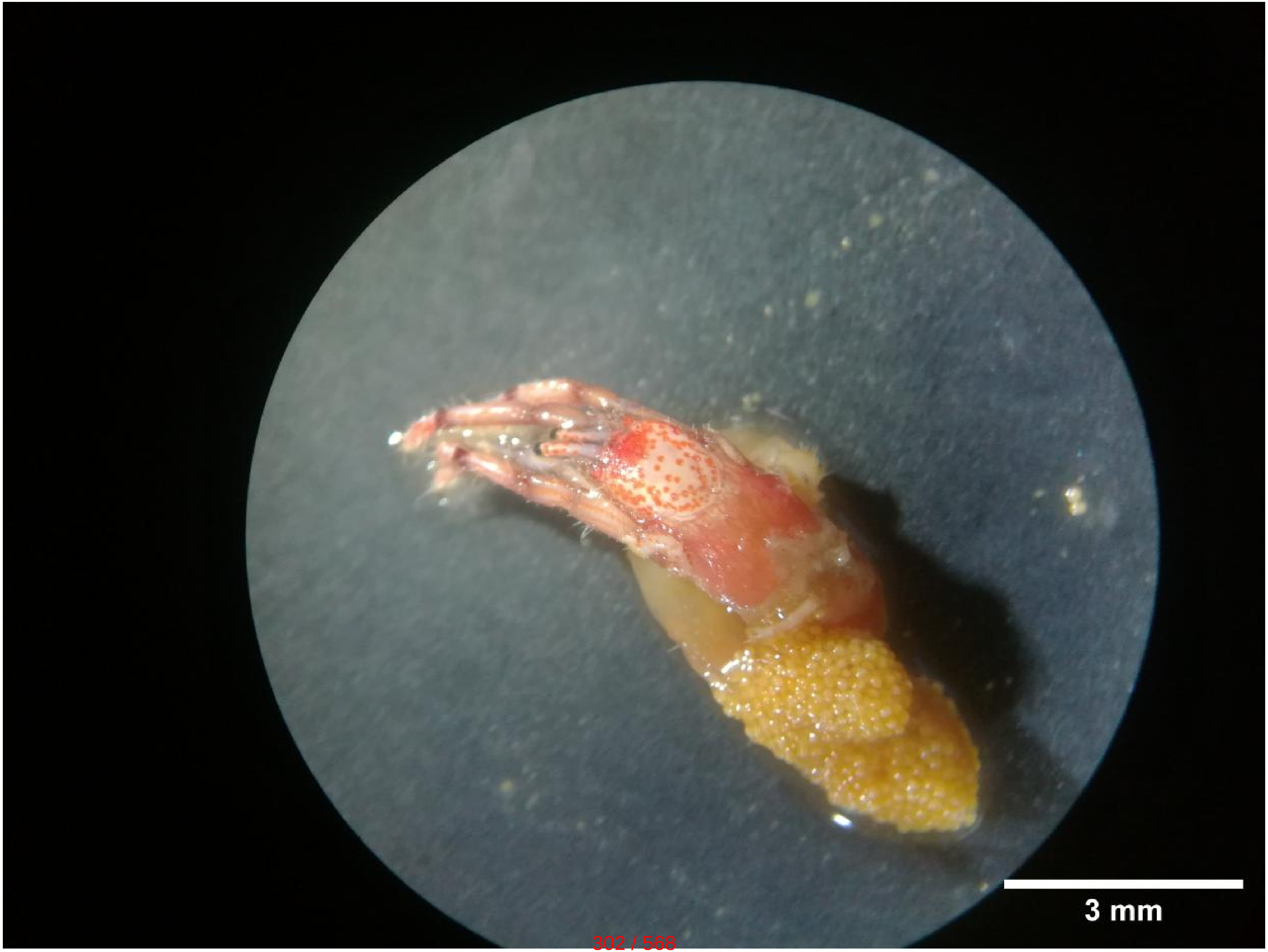
3 mm

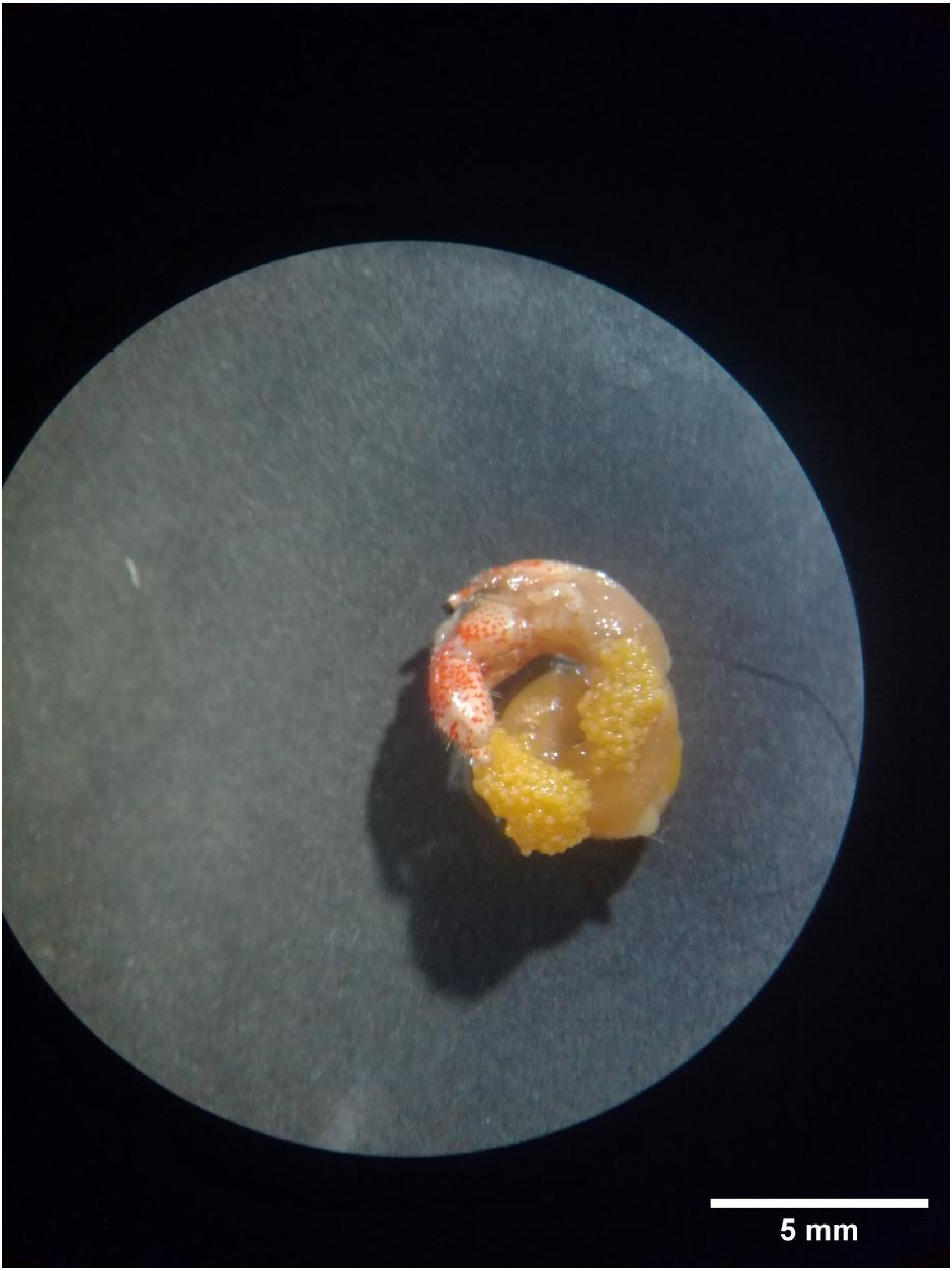
5 mm

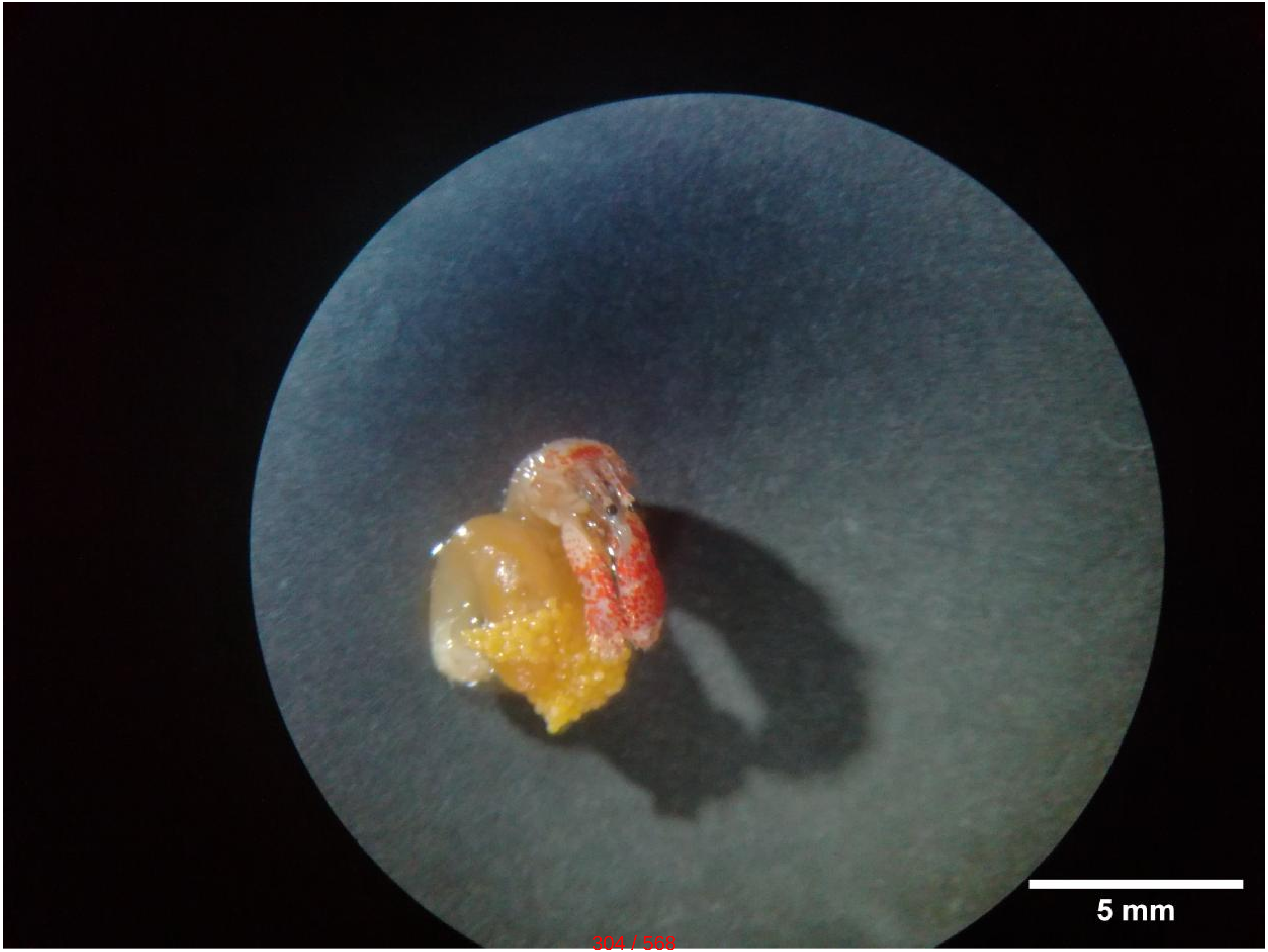
5 mm

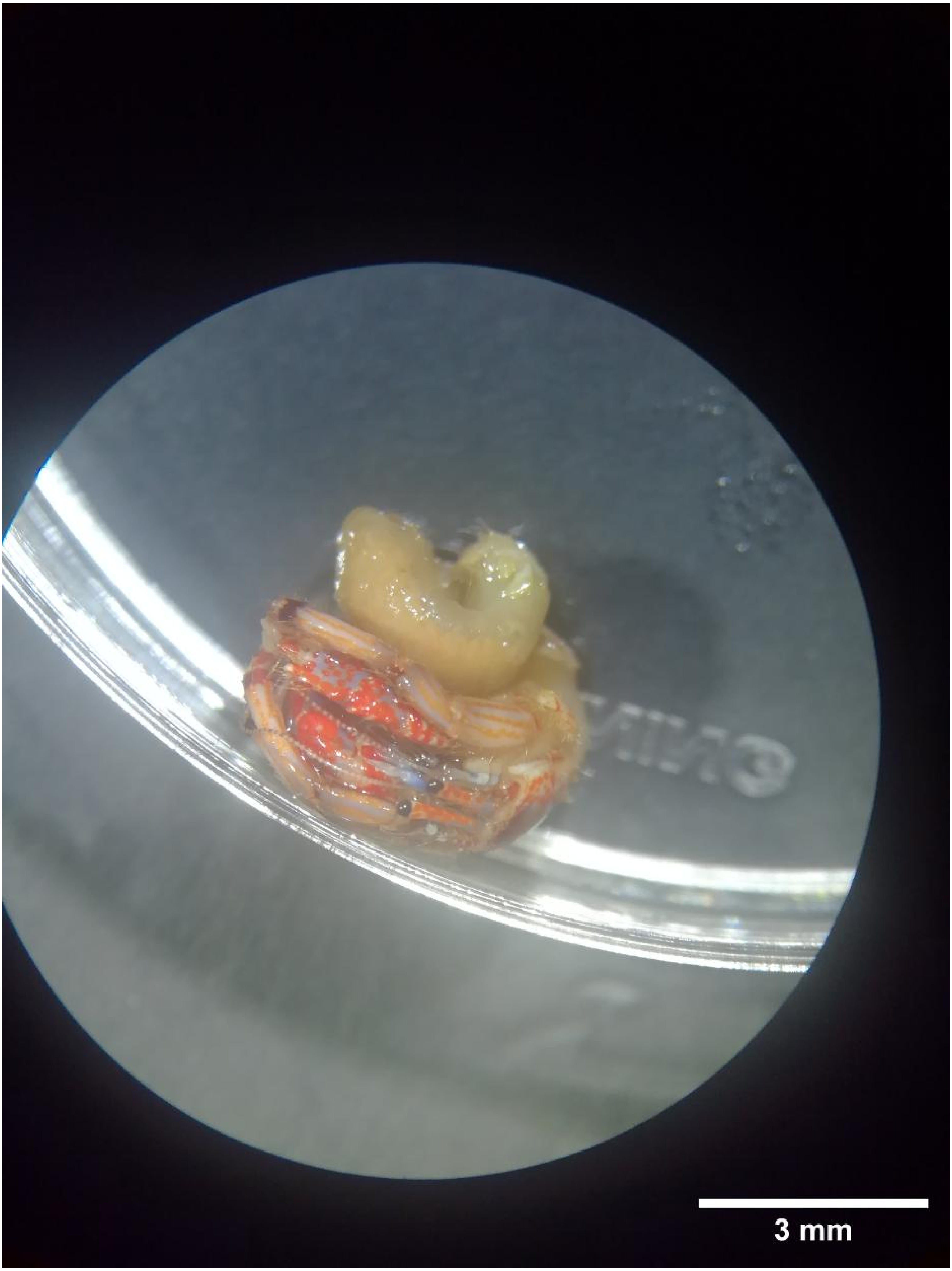
3 mm

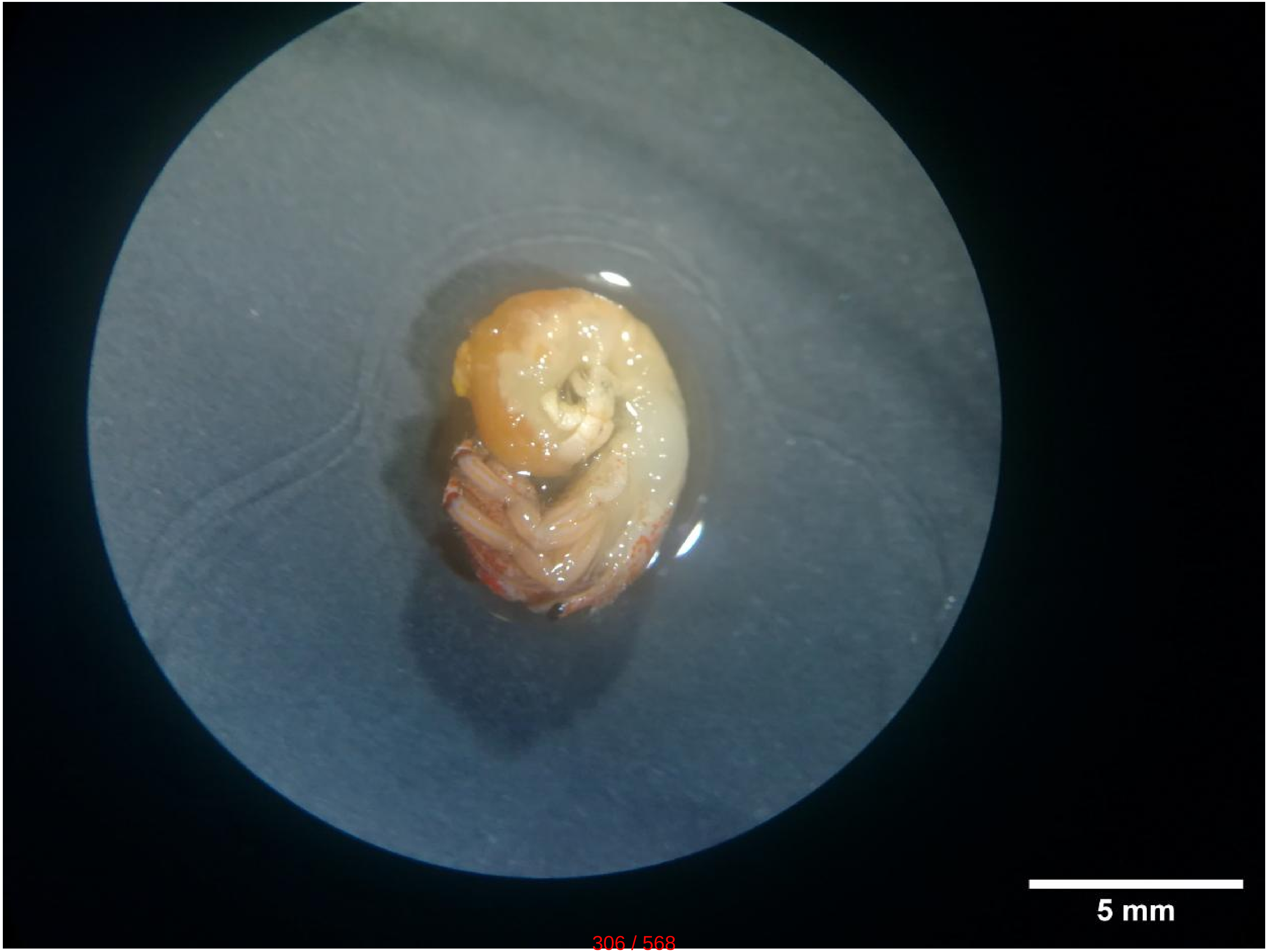
5 mm

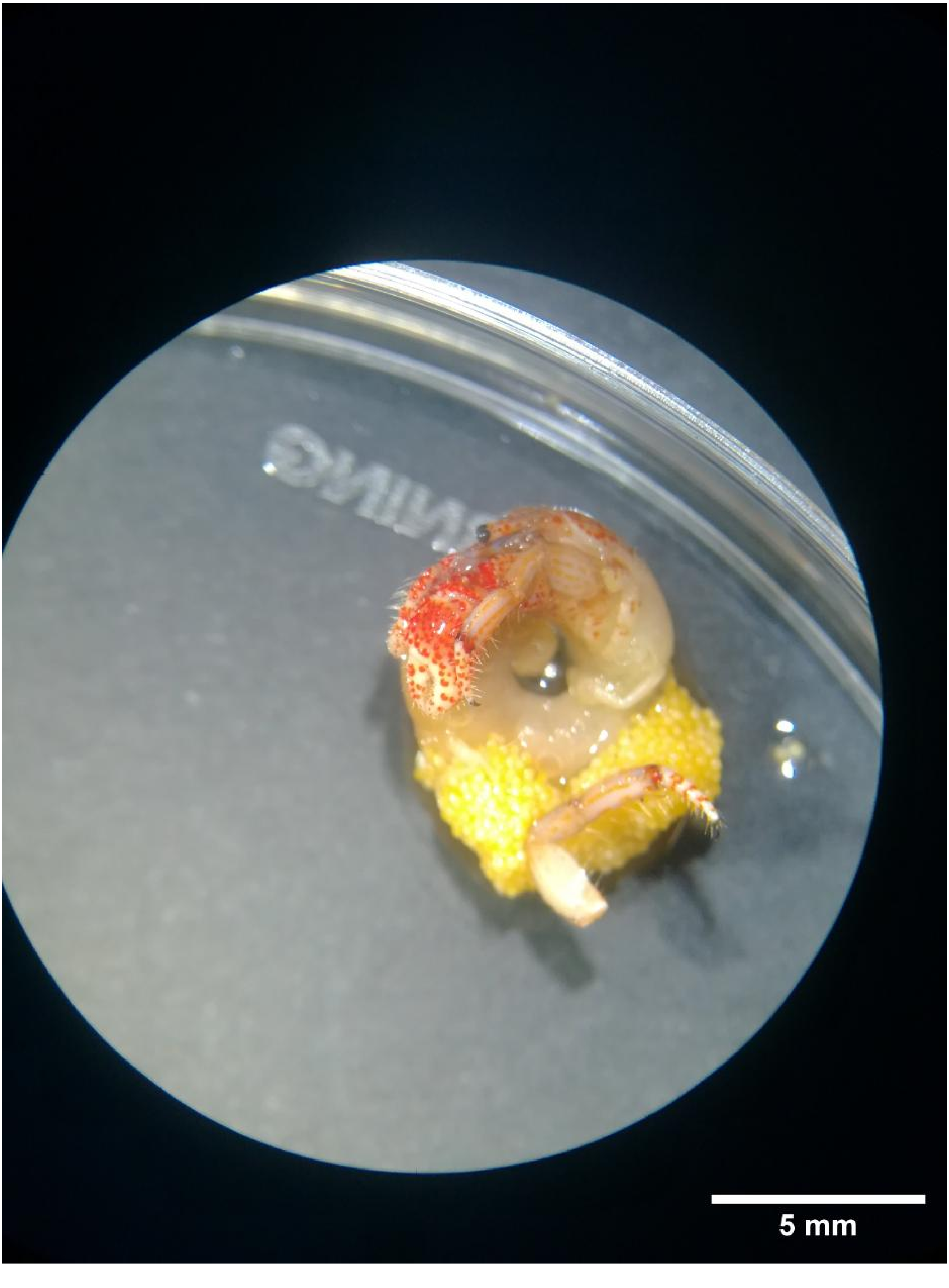
5 mm

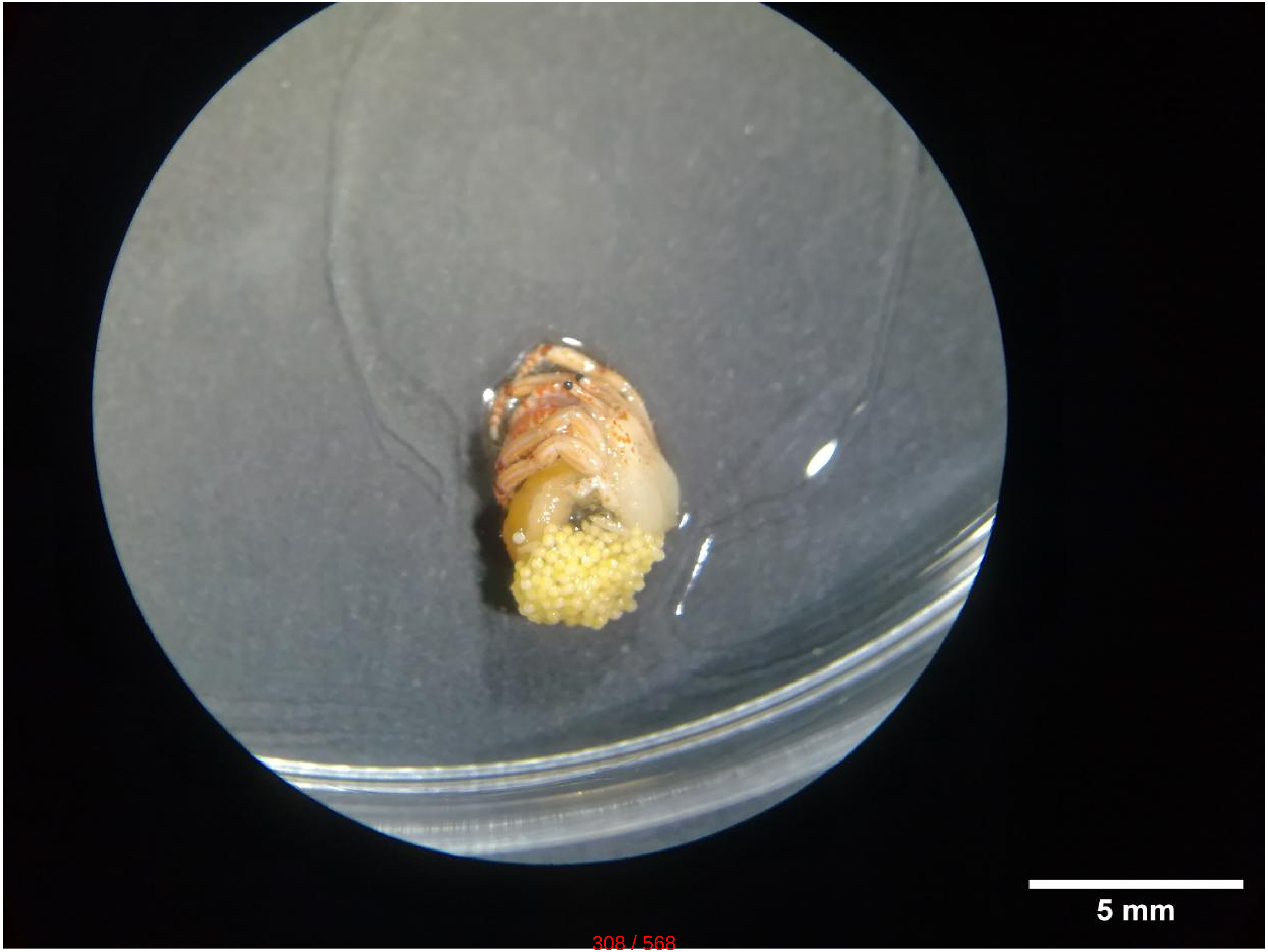
5 mm

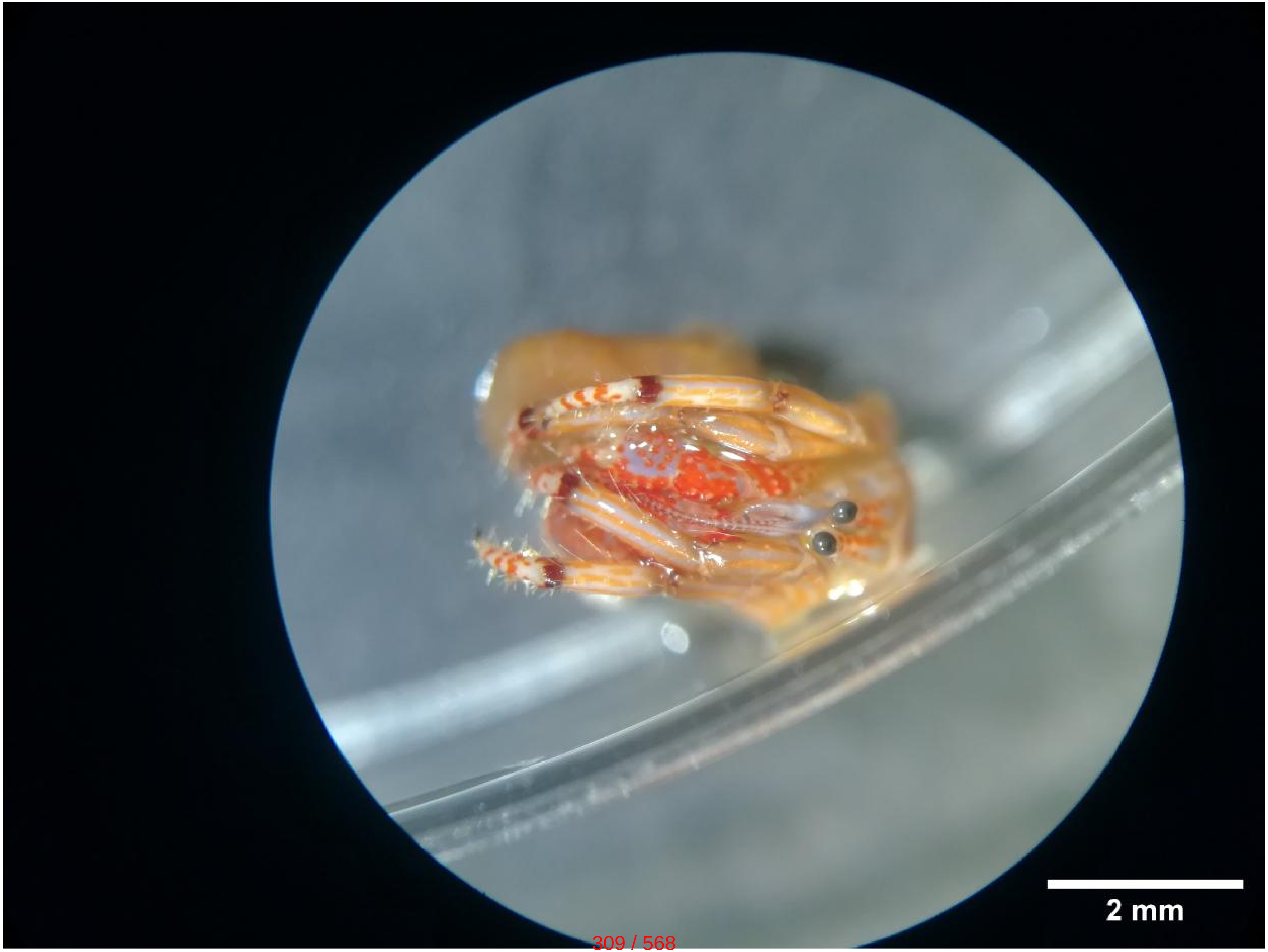
2 mm

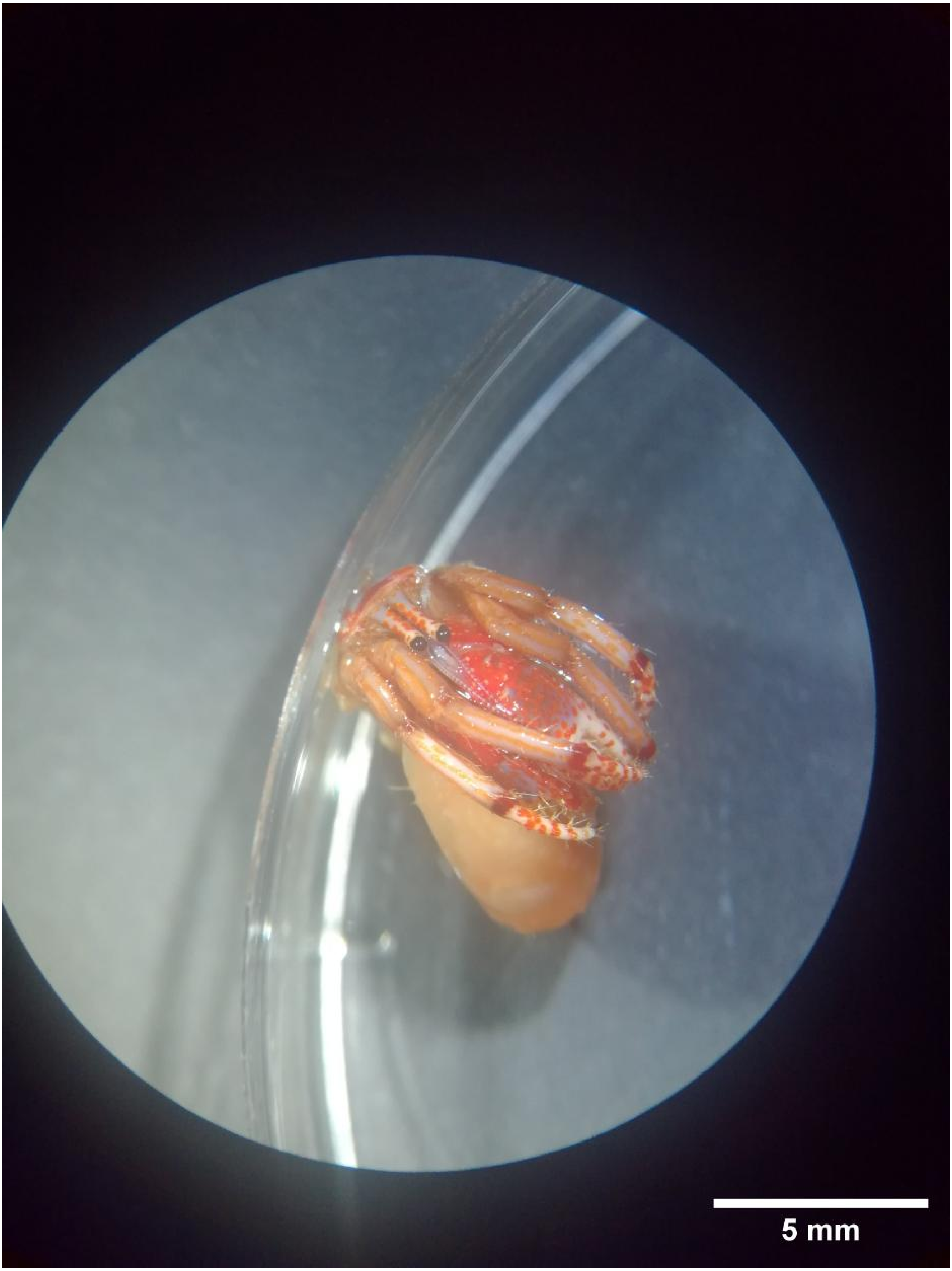
5 mm

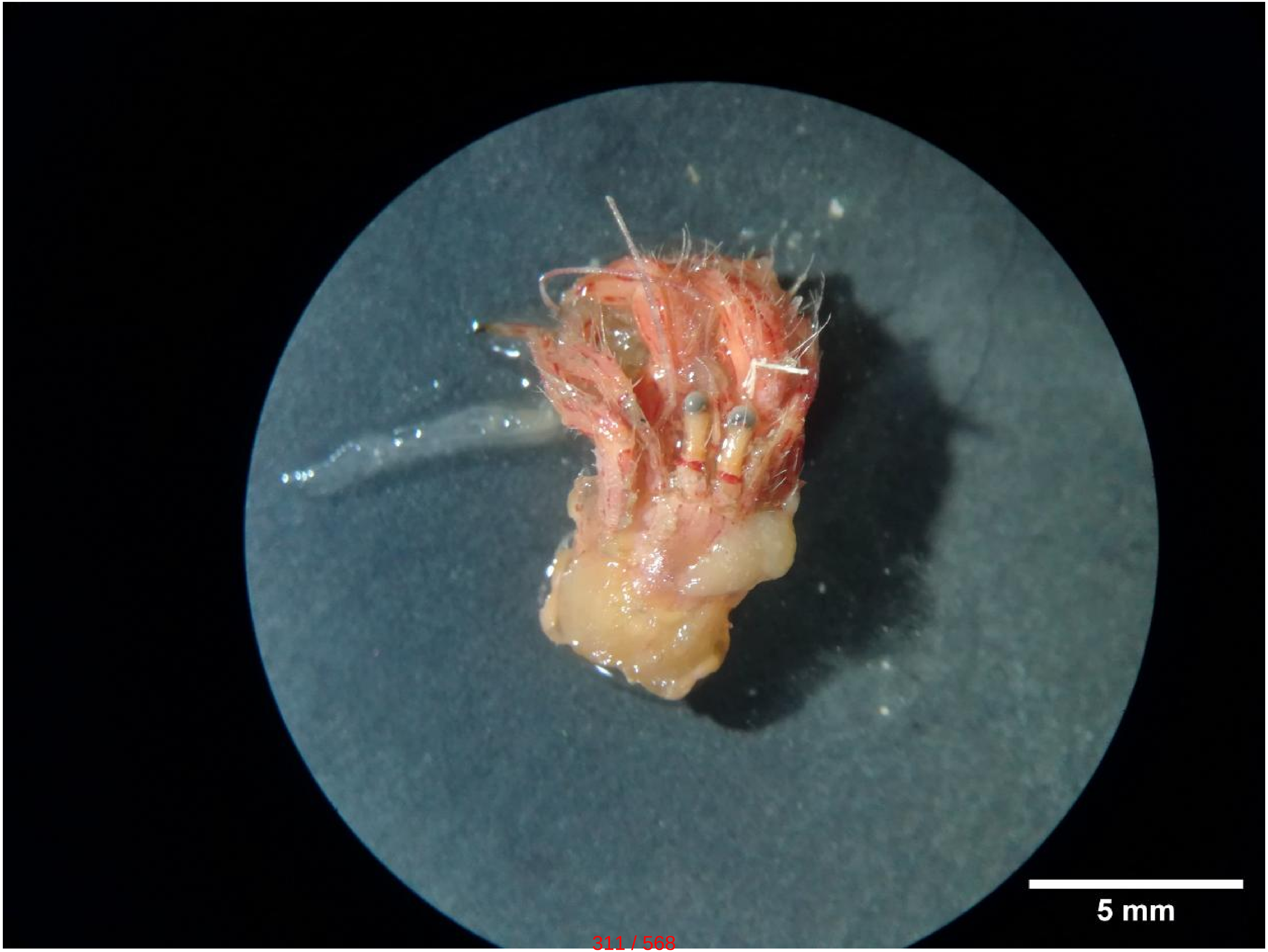
5 mm

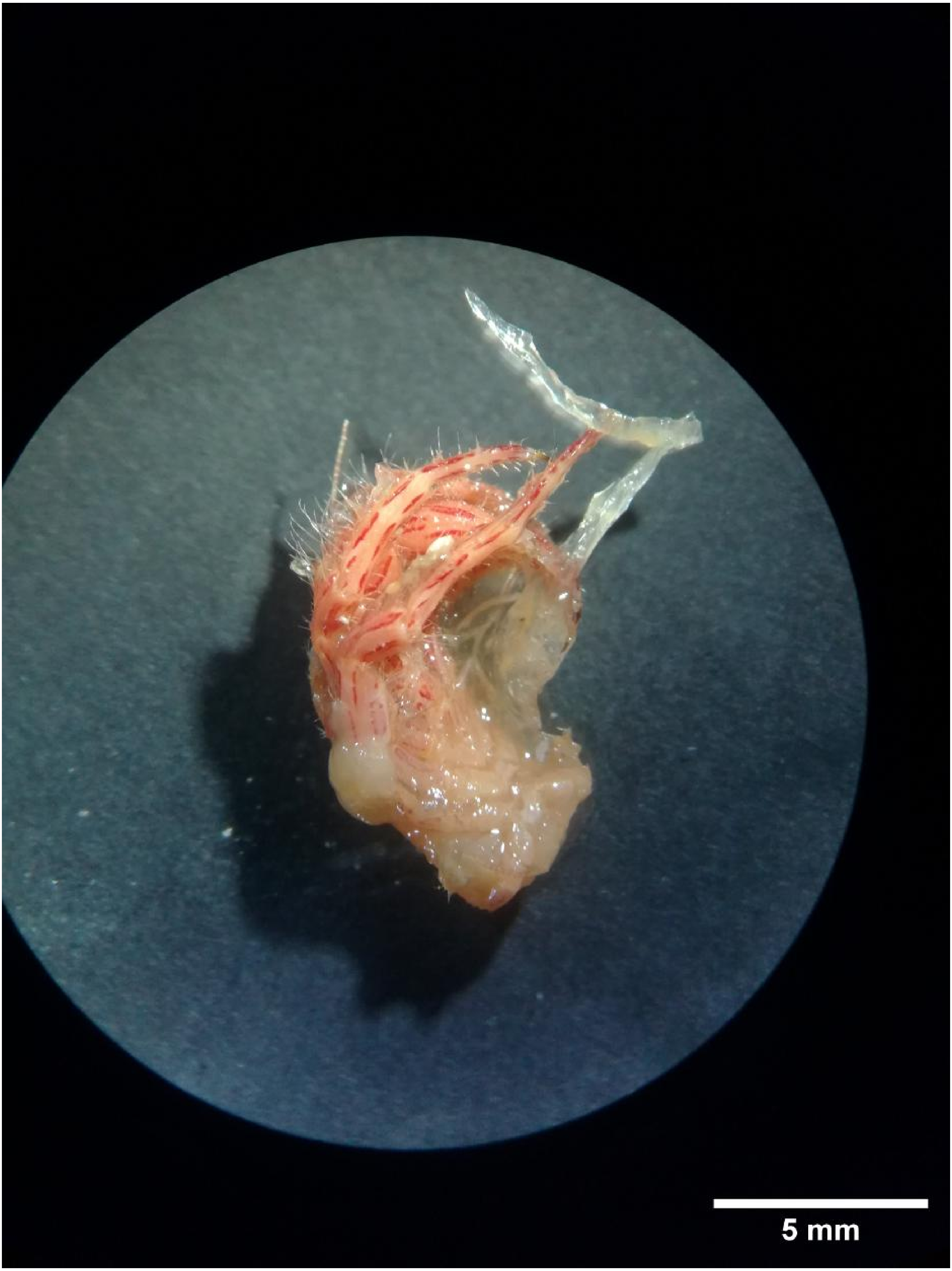
5 mm

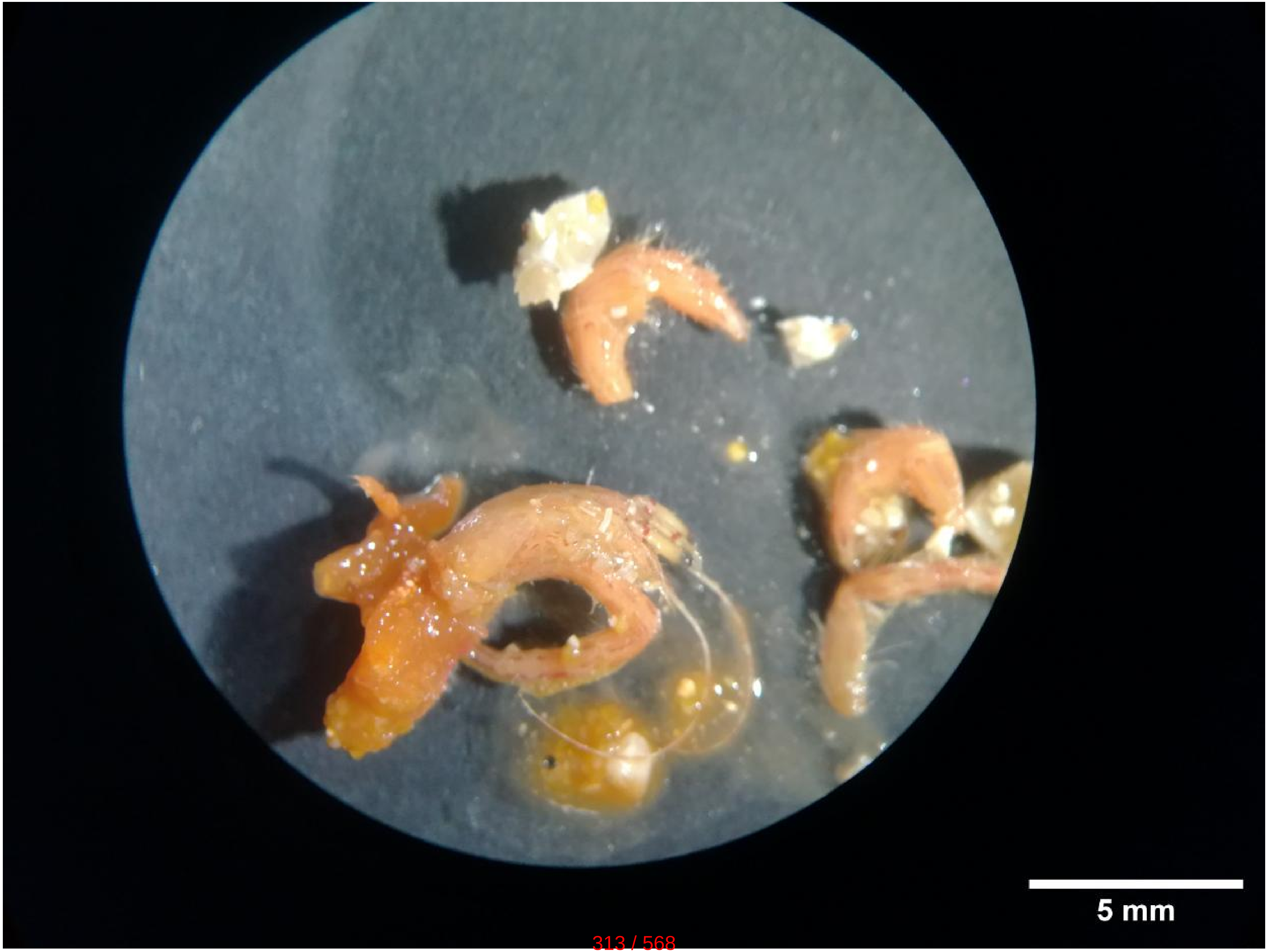
5 mm

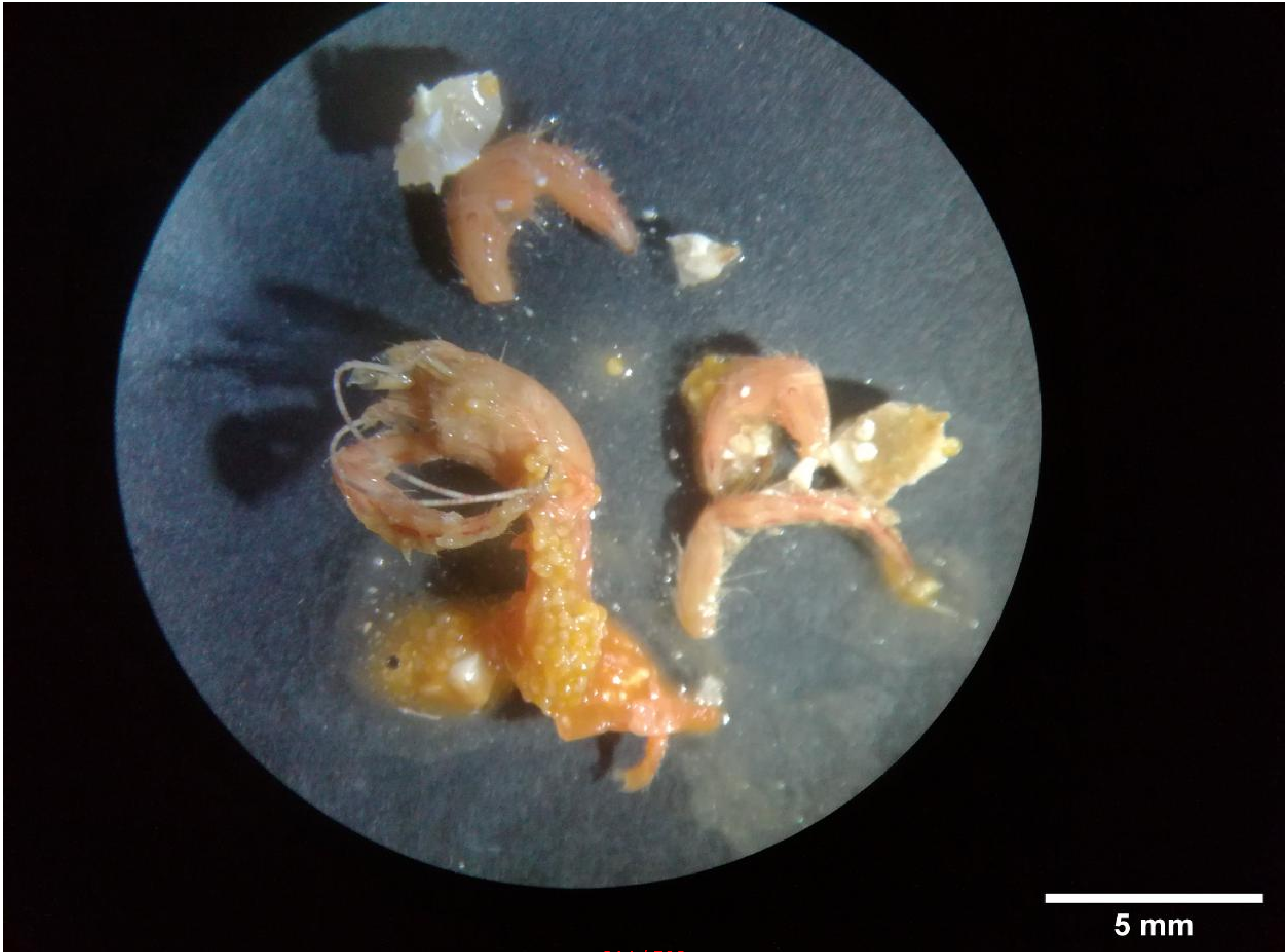
5 mm

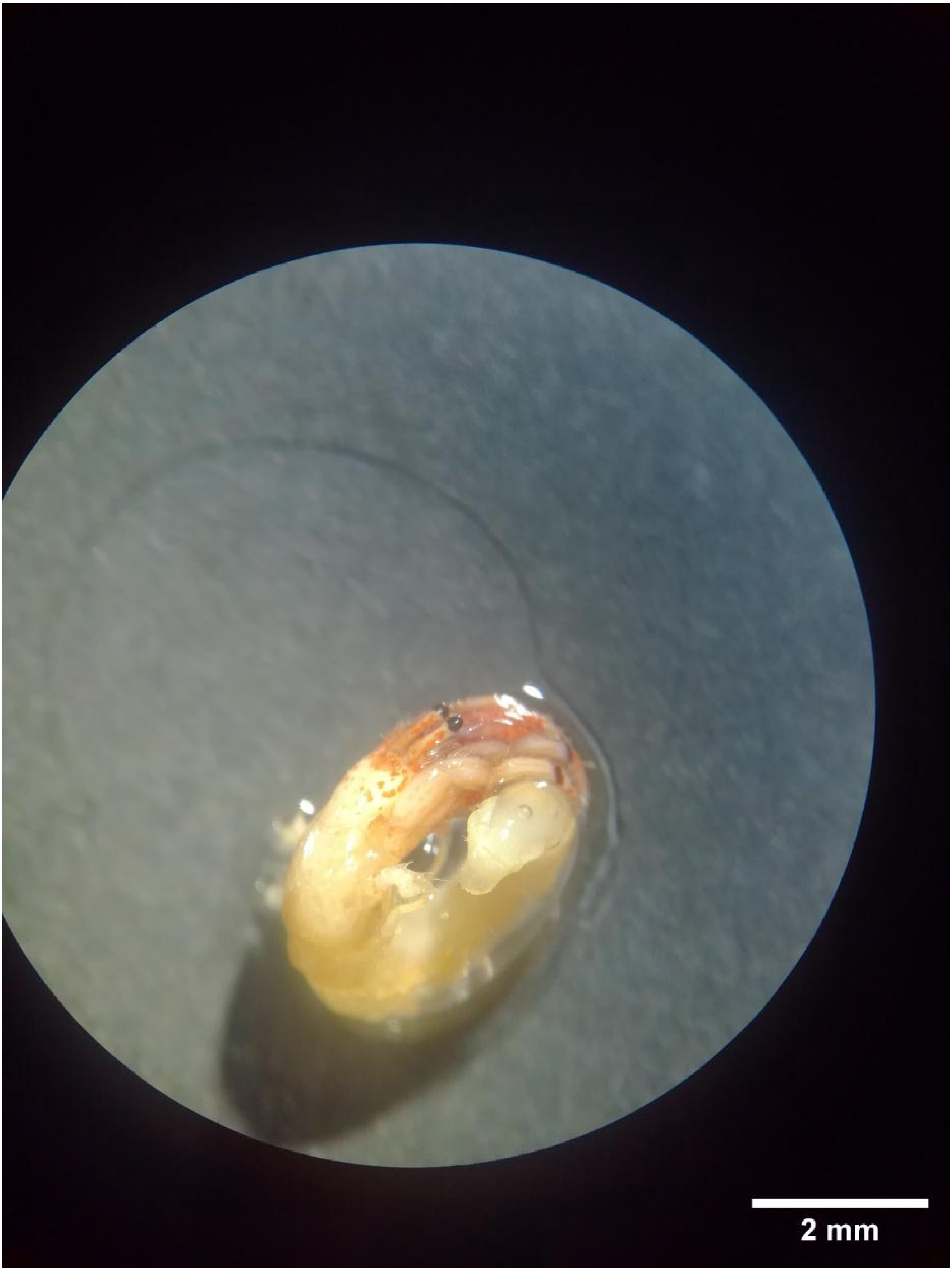
2 mm

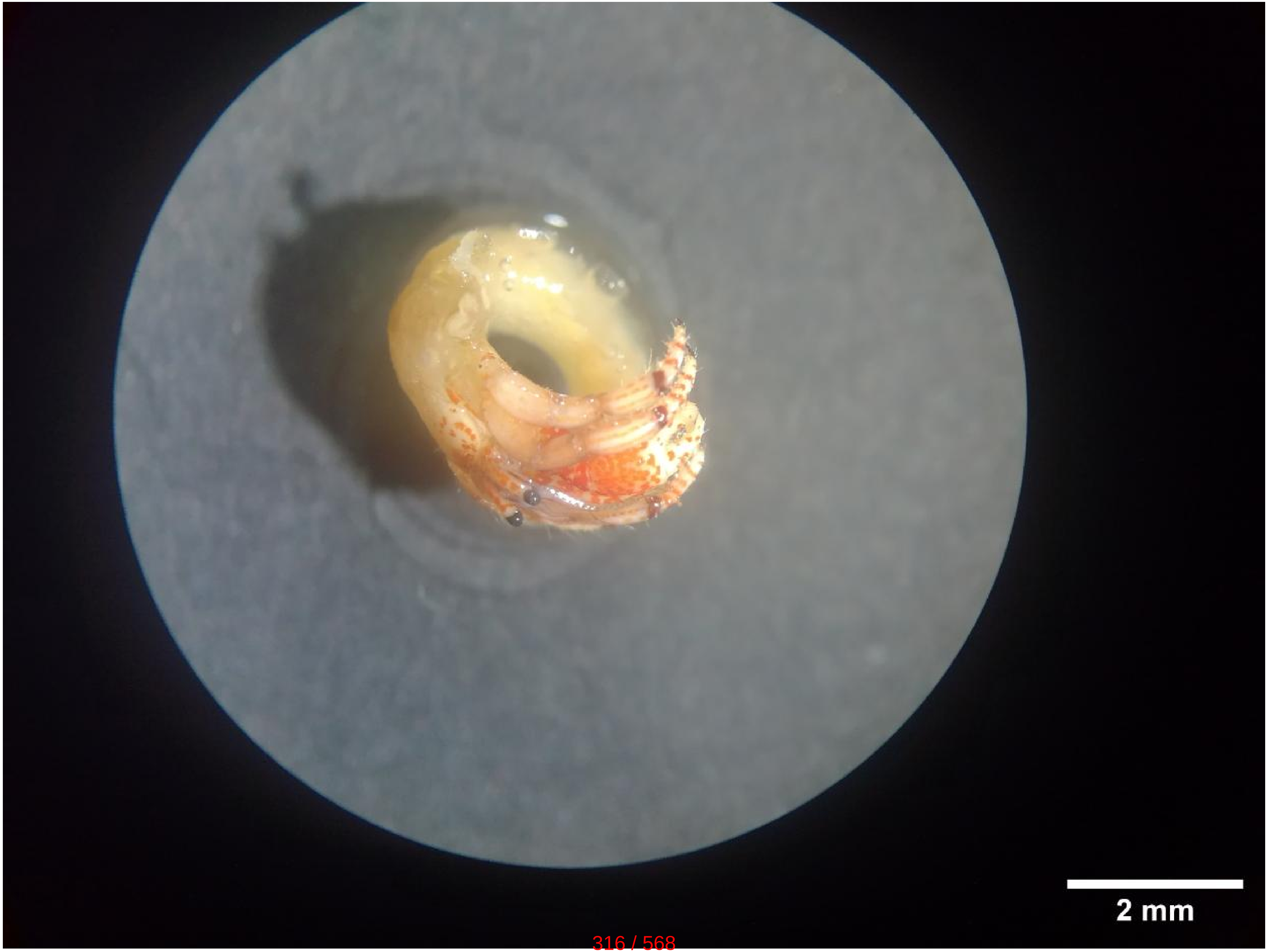
2 mm

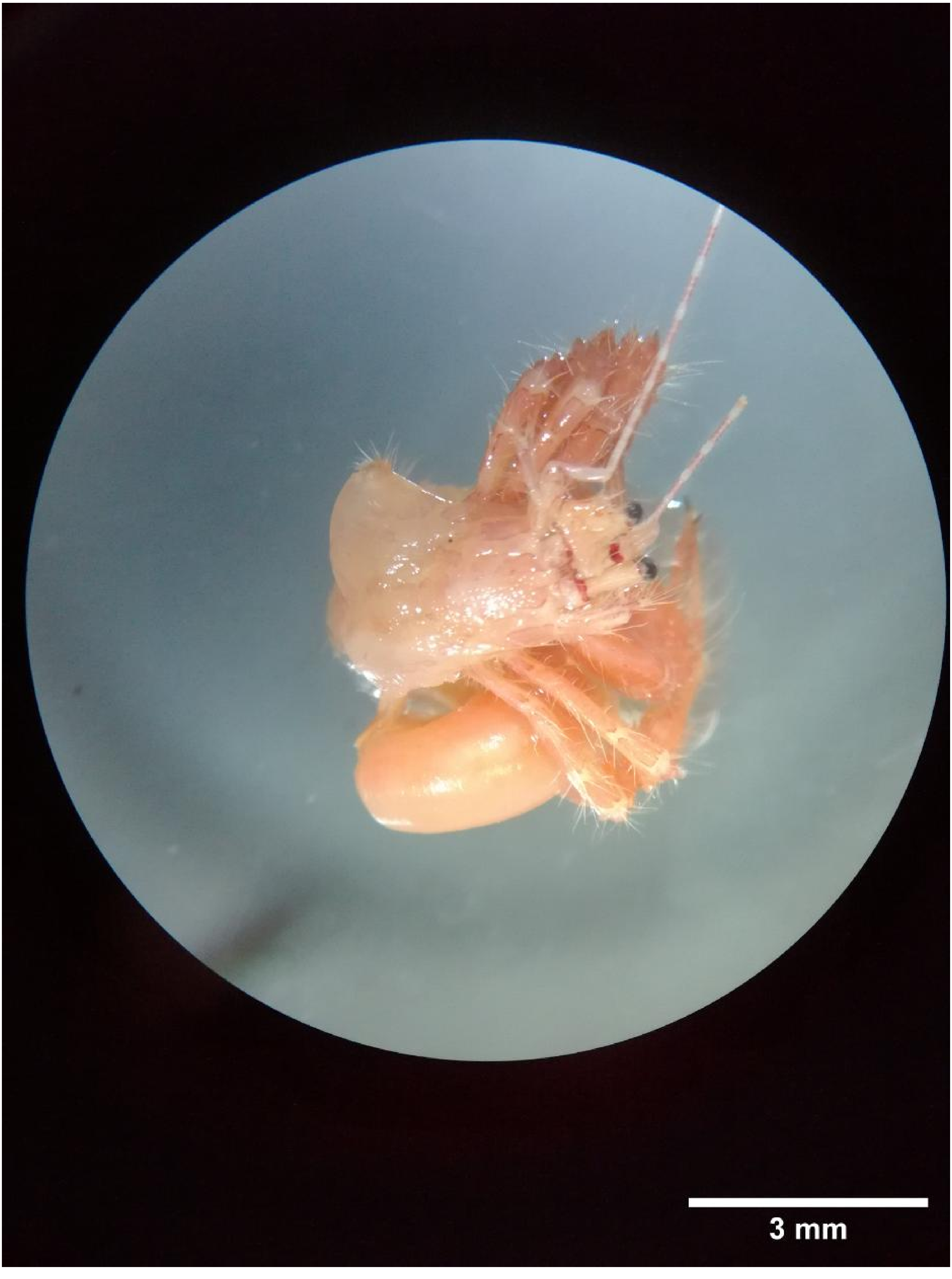
3 mm

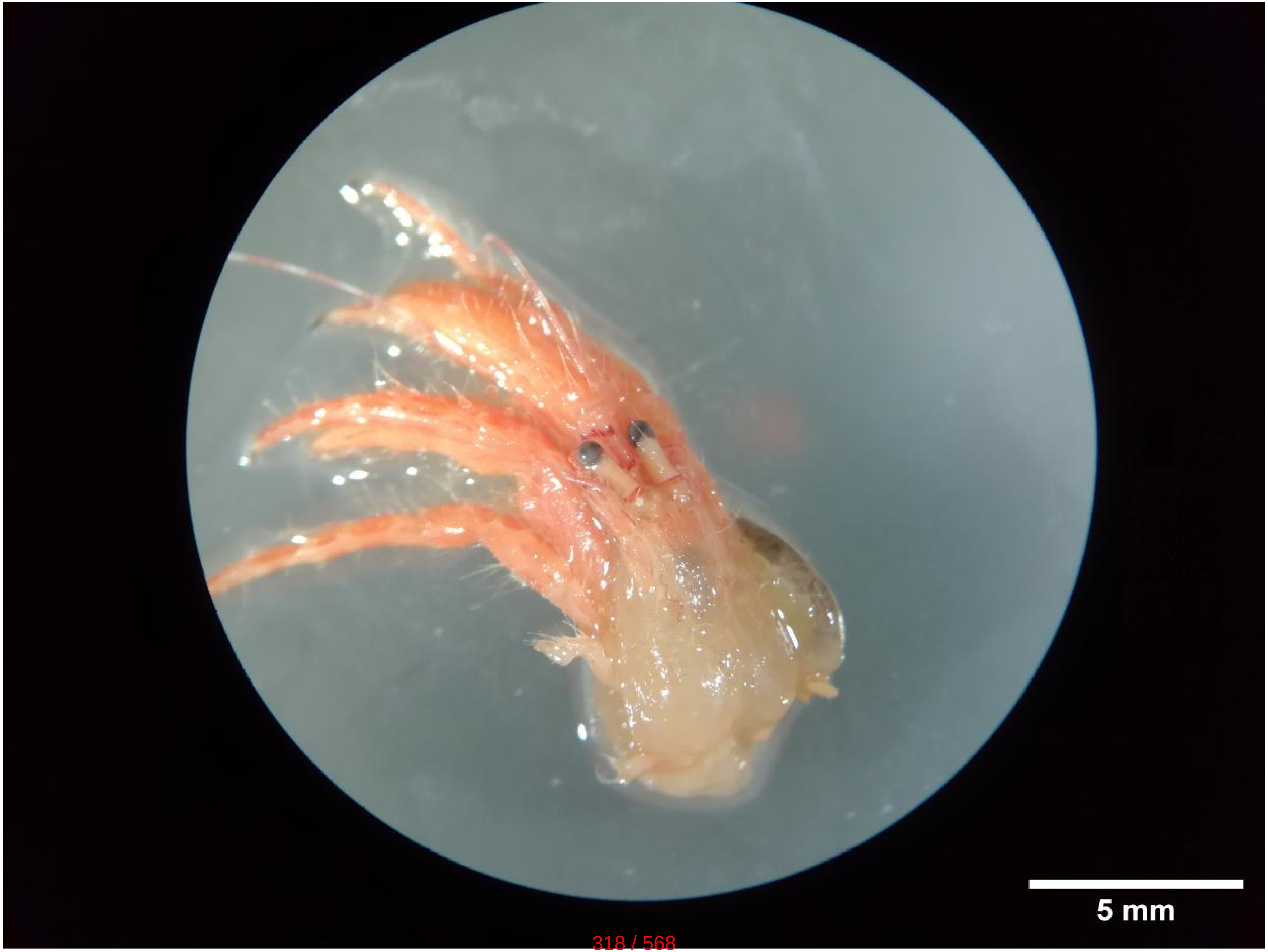
5 mm

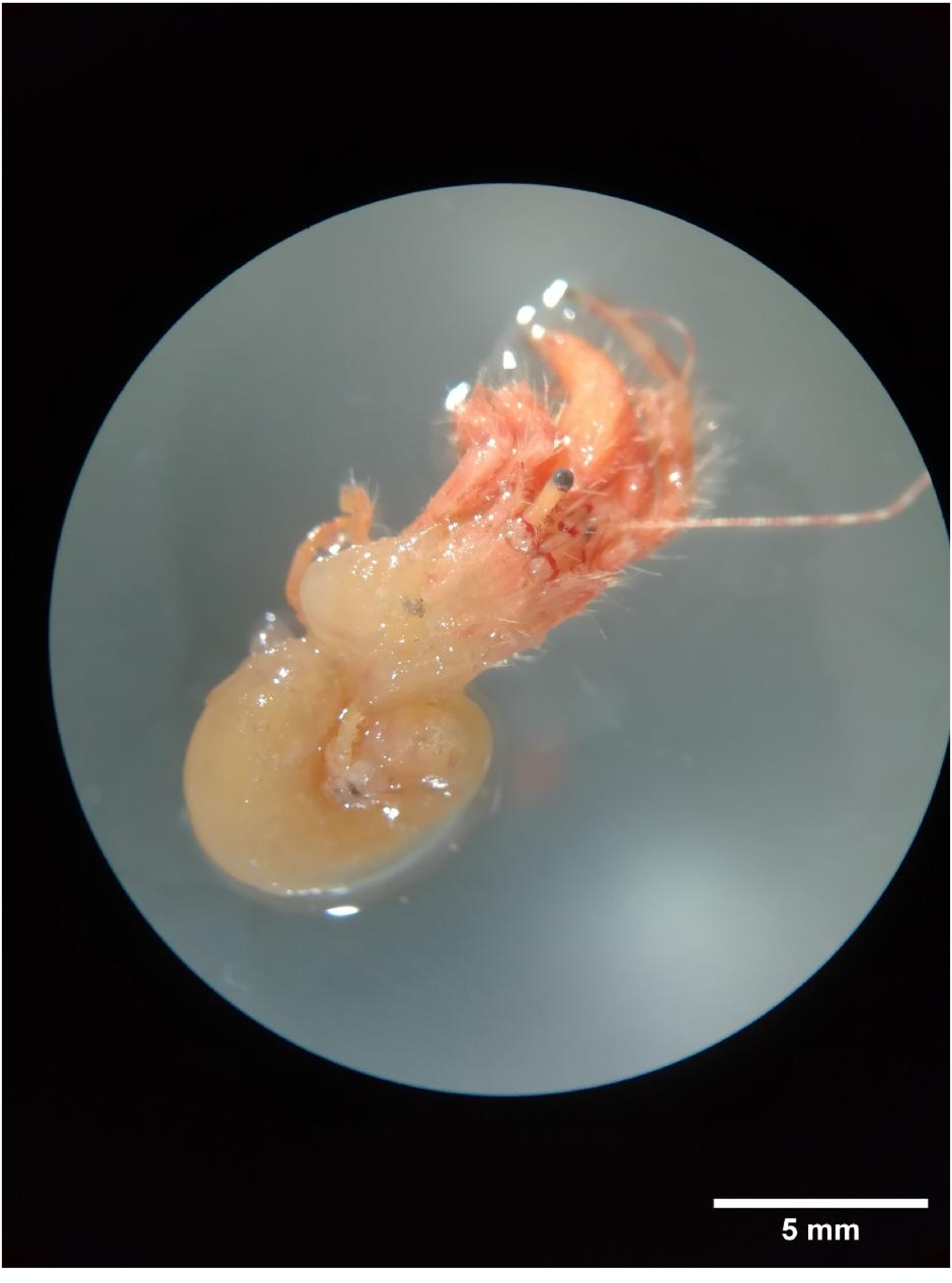
5 mm

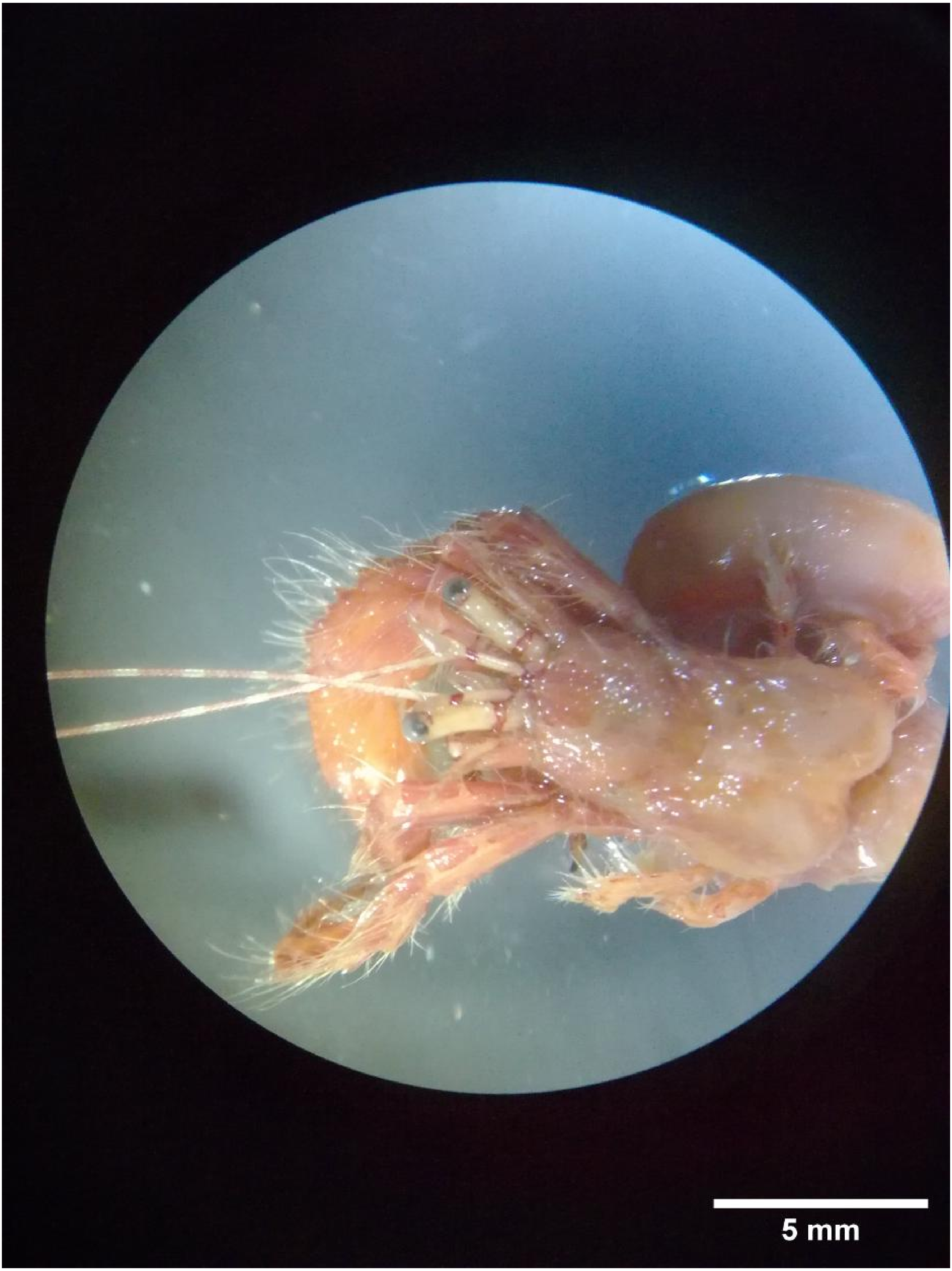
5 mm

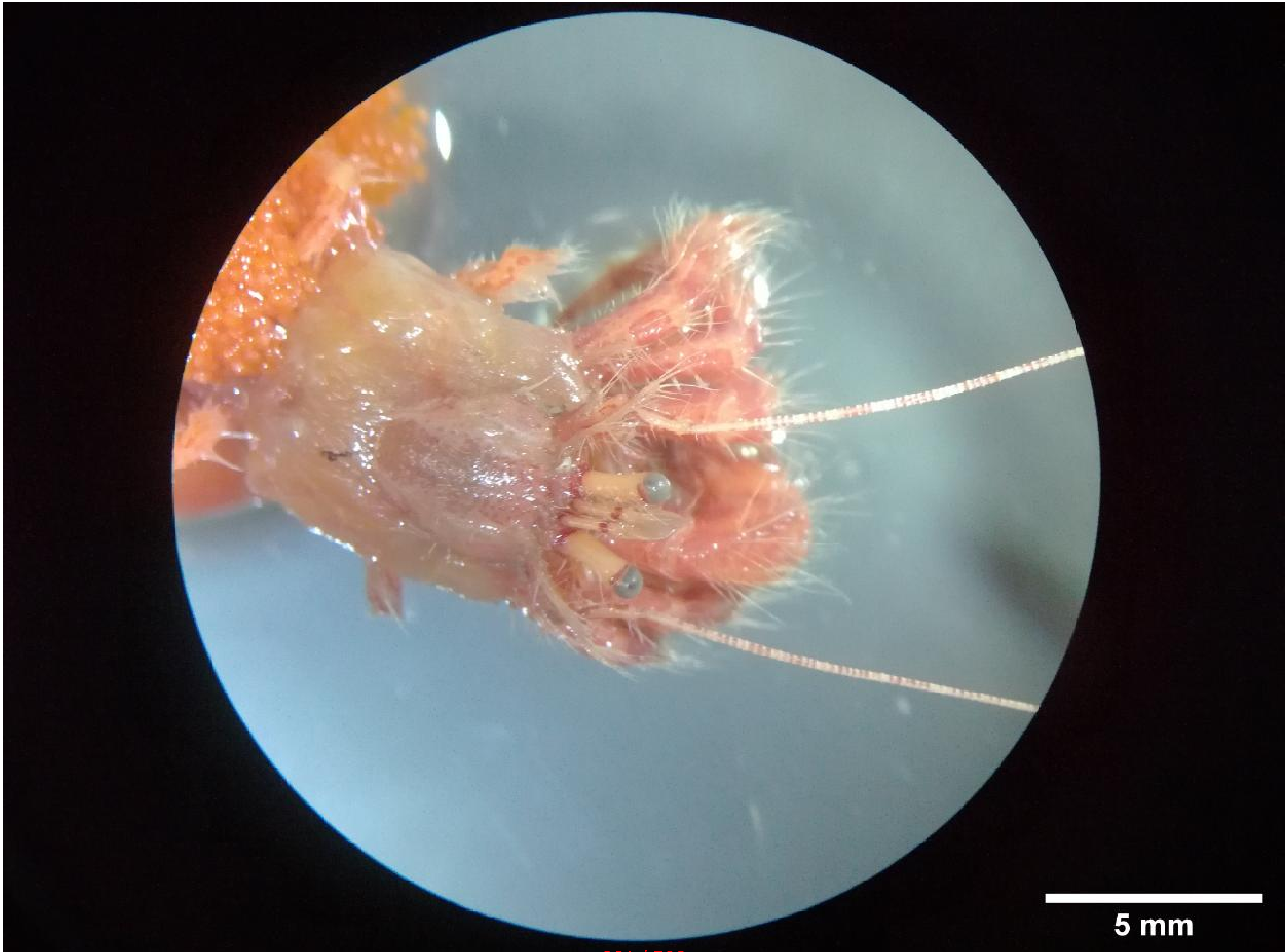
5 mm

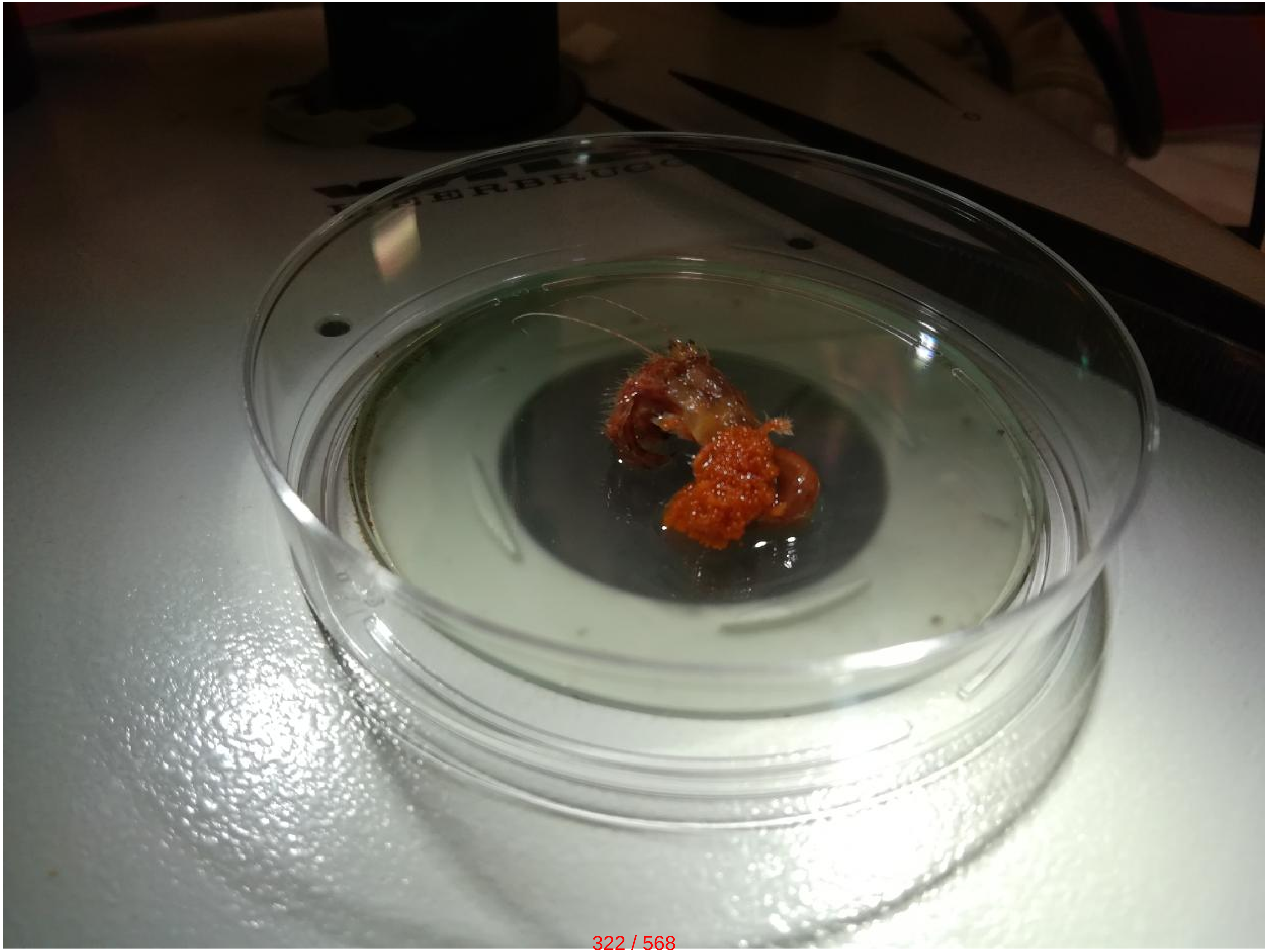

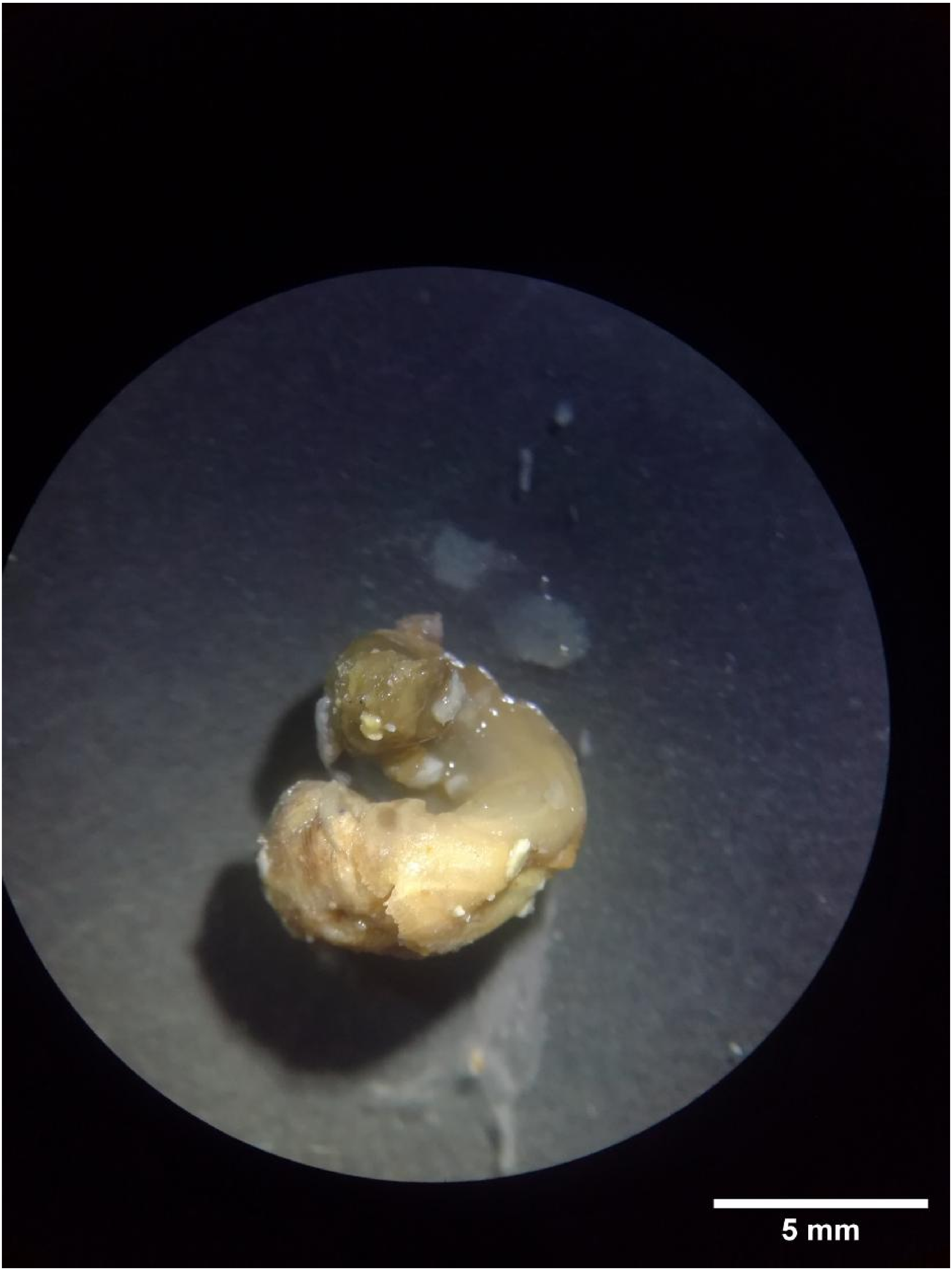
5 mm

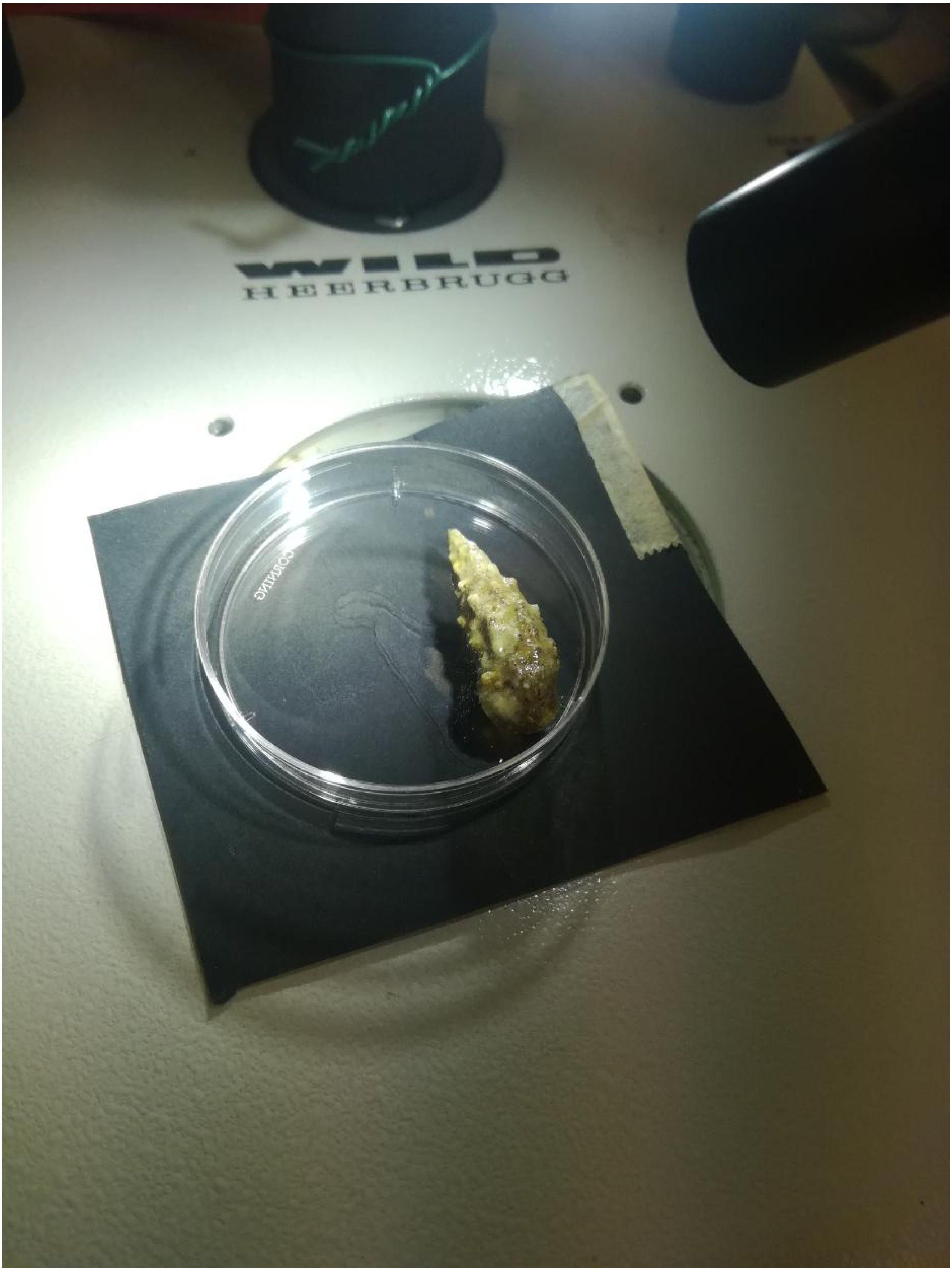
WILD
HEERBRUGG

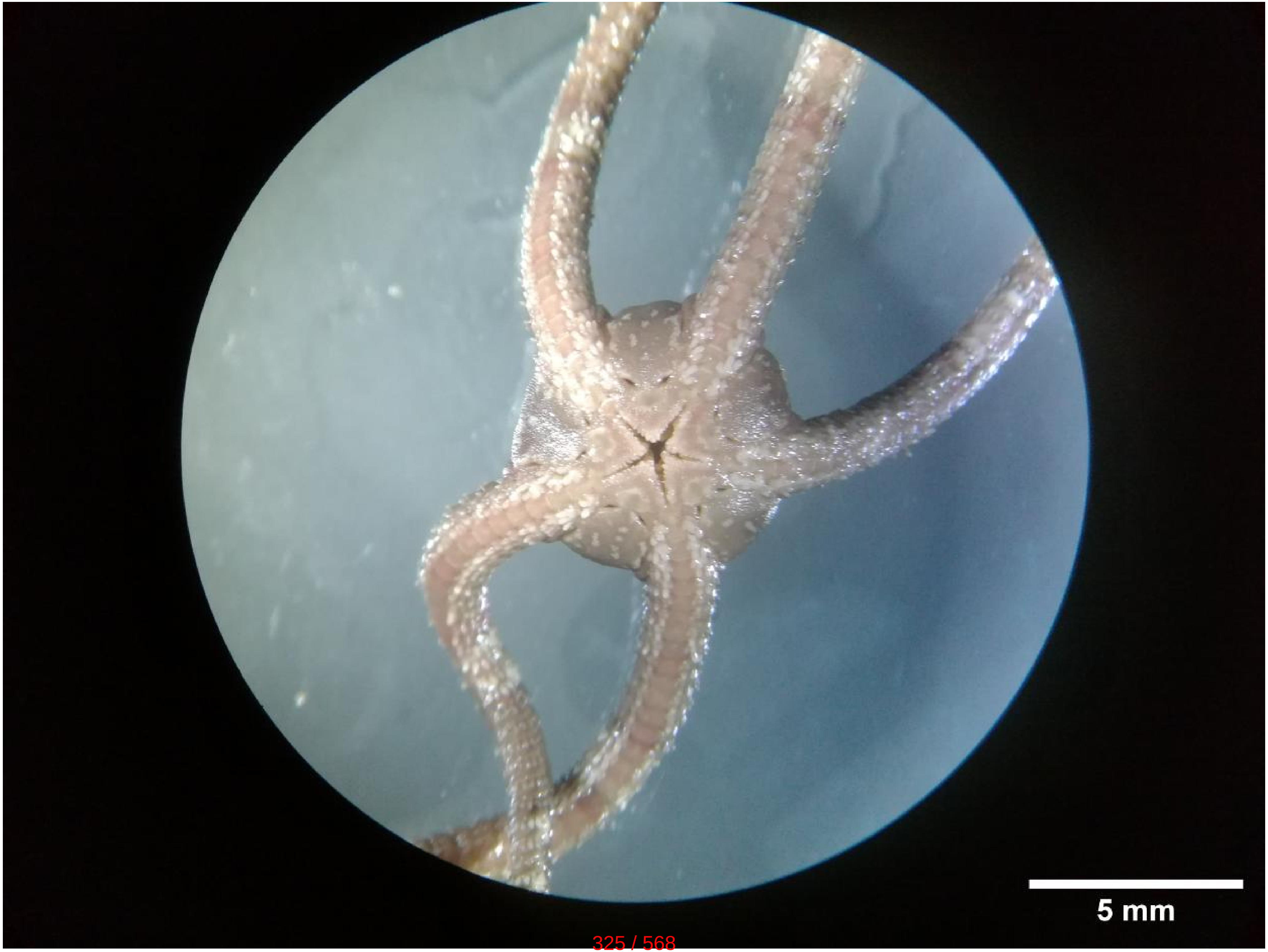
5 mm

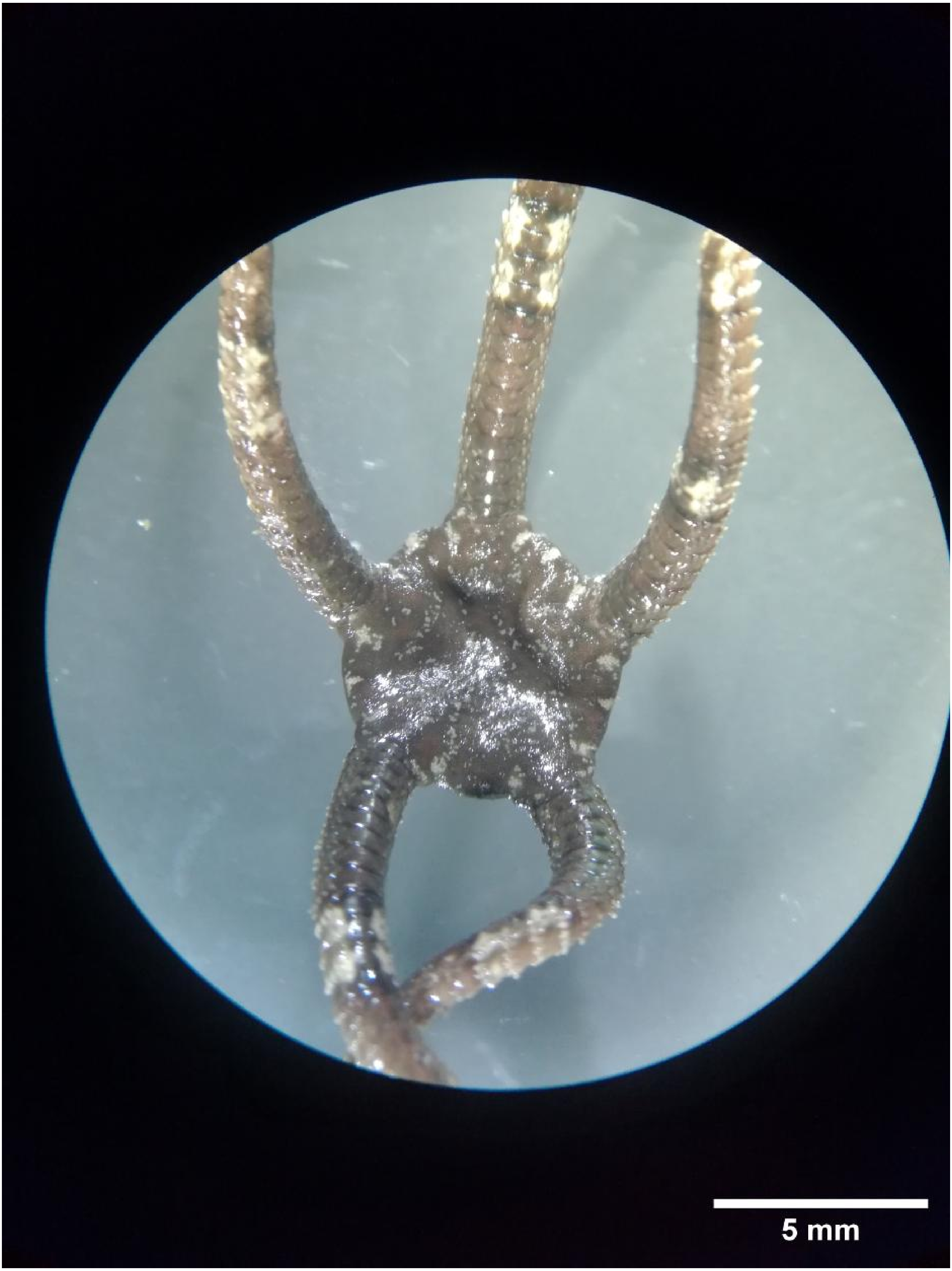
5 mm

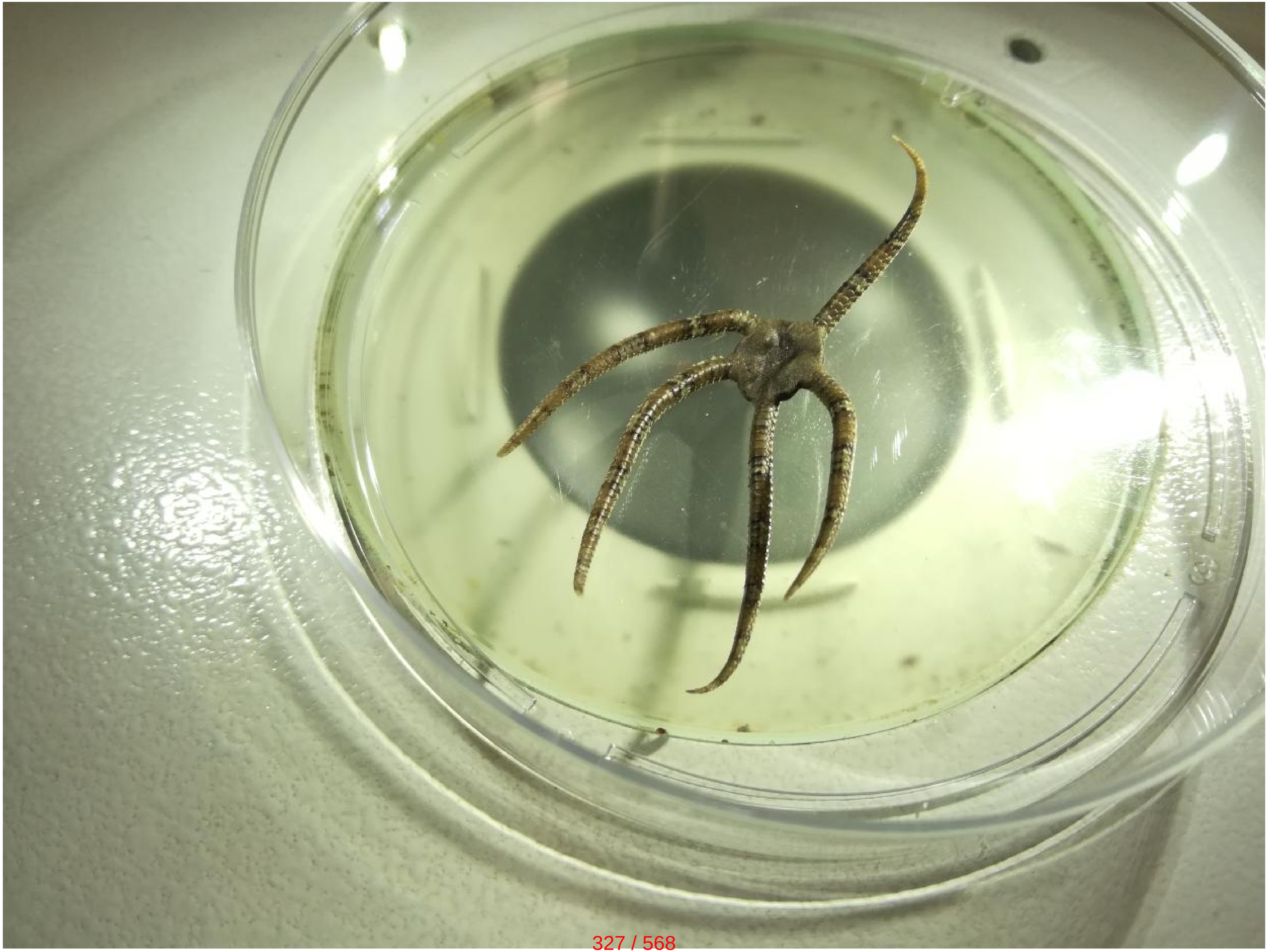

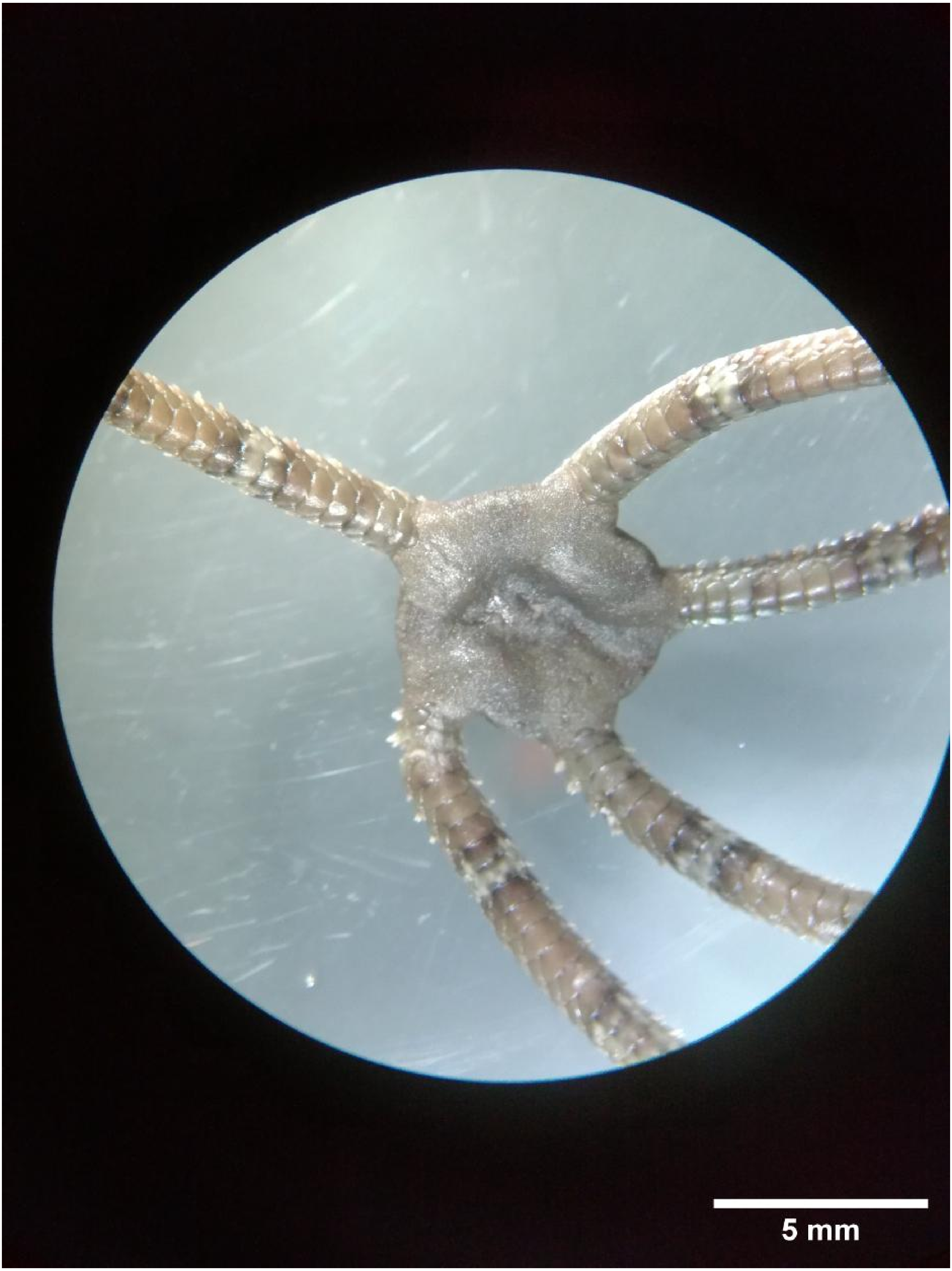
5 mm

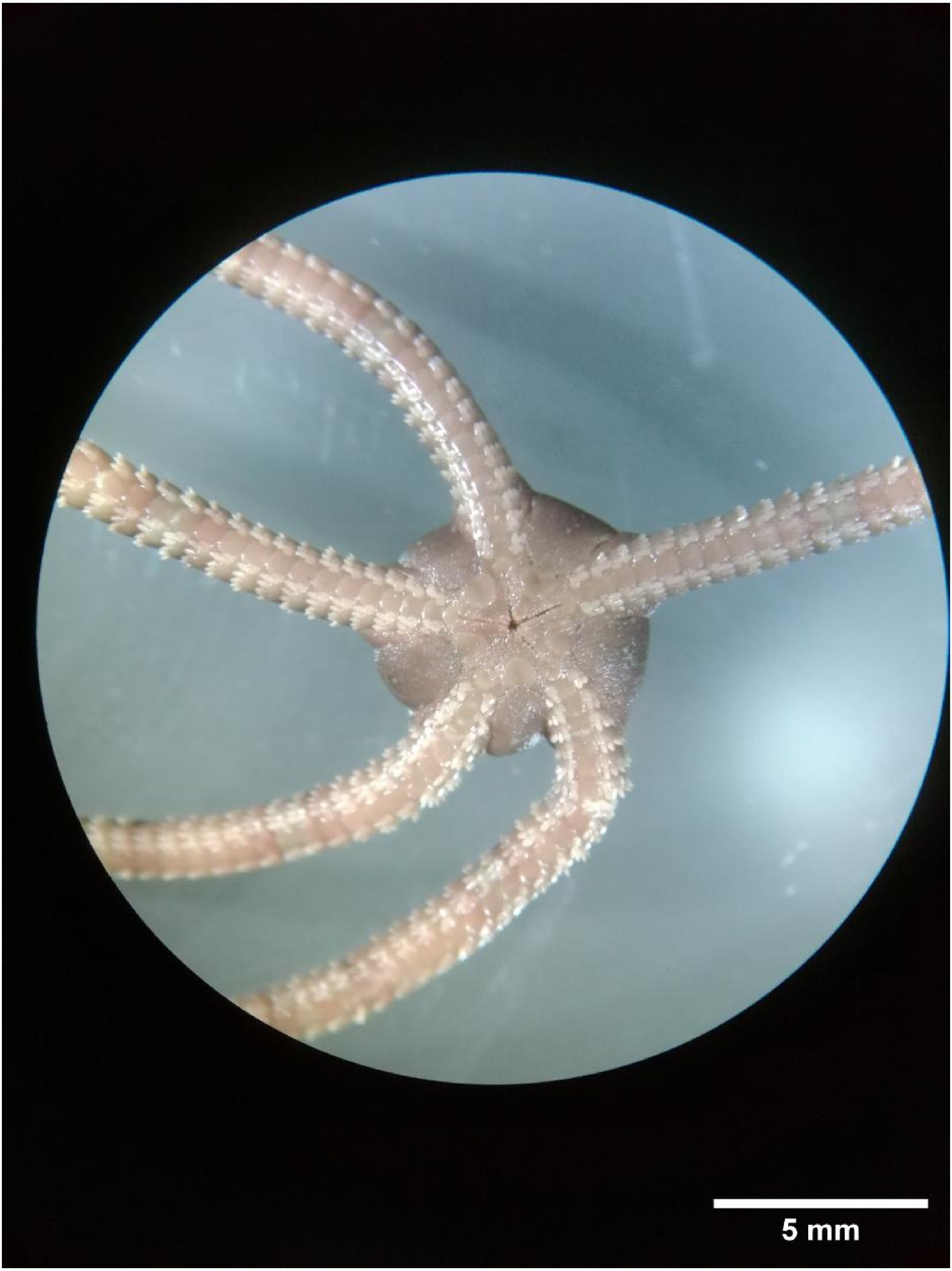
5 mm

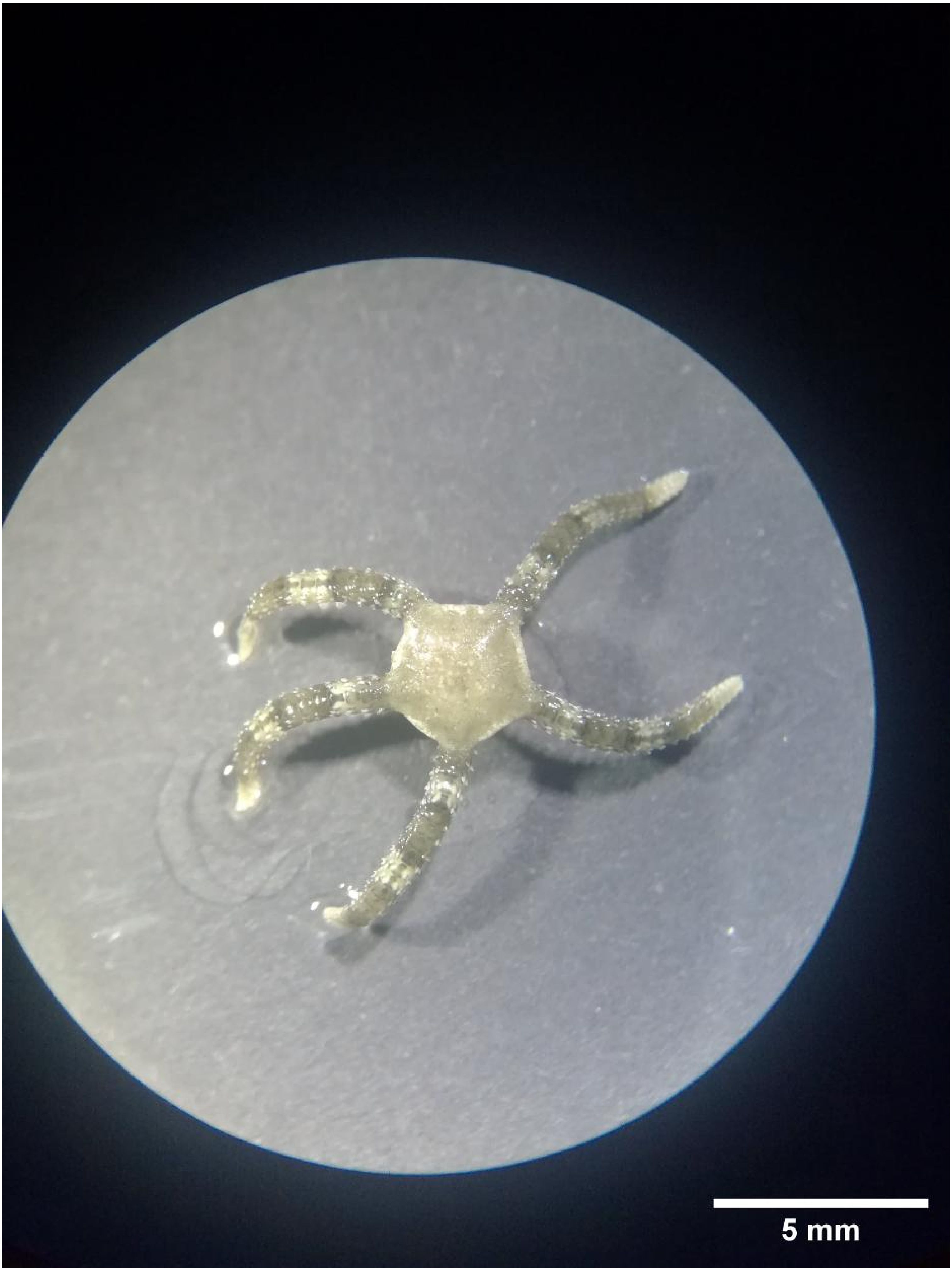
5 mm

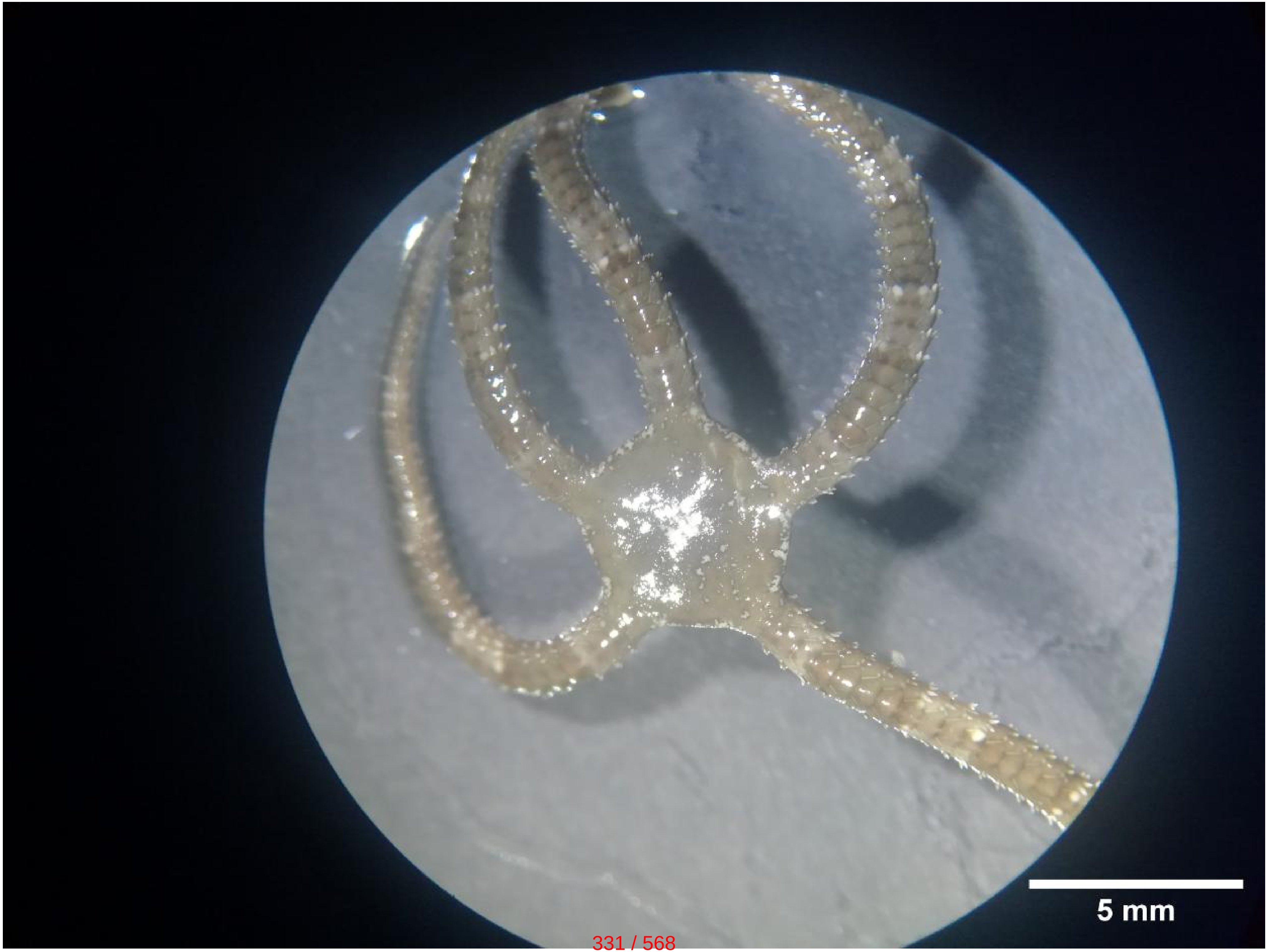
5 mm

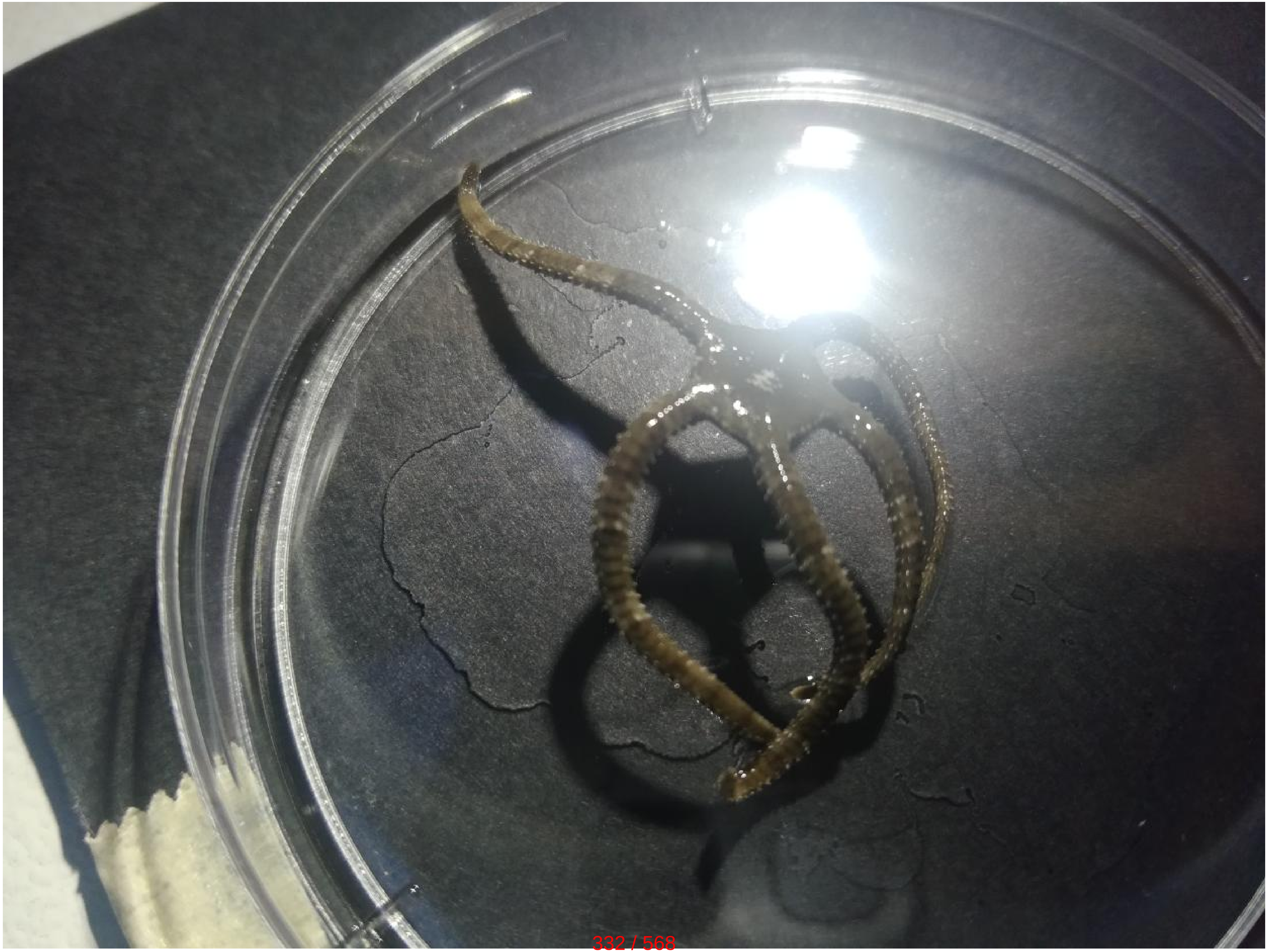

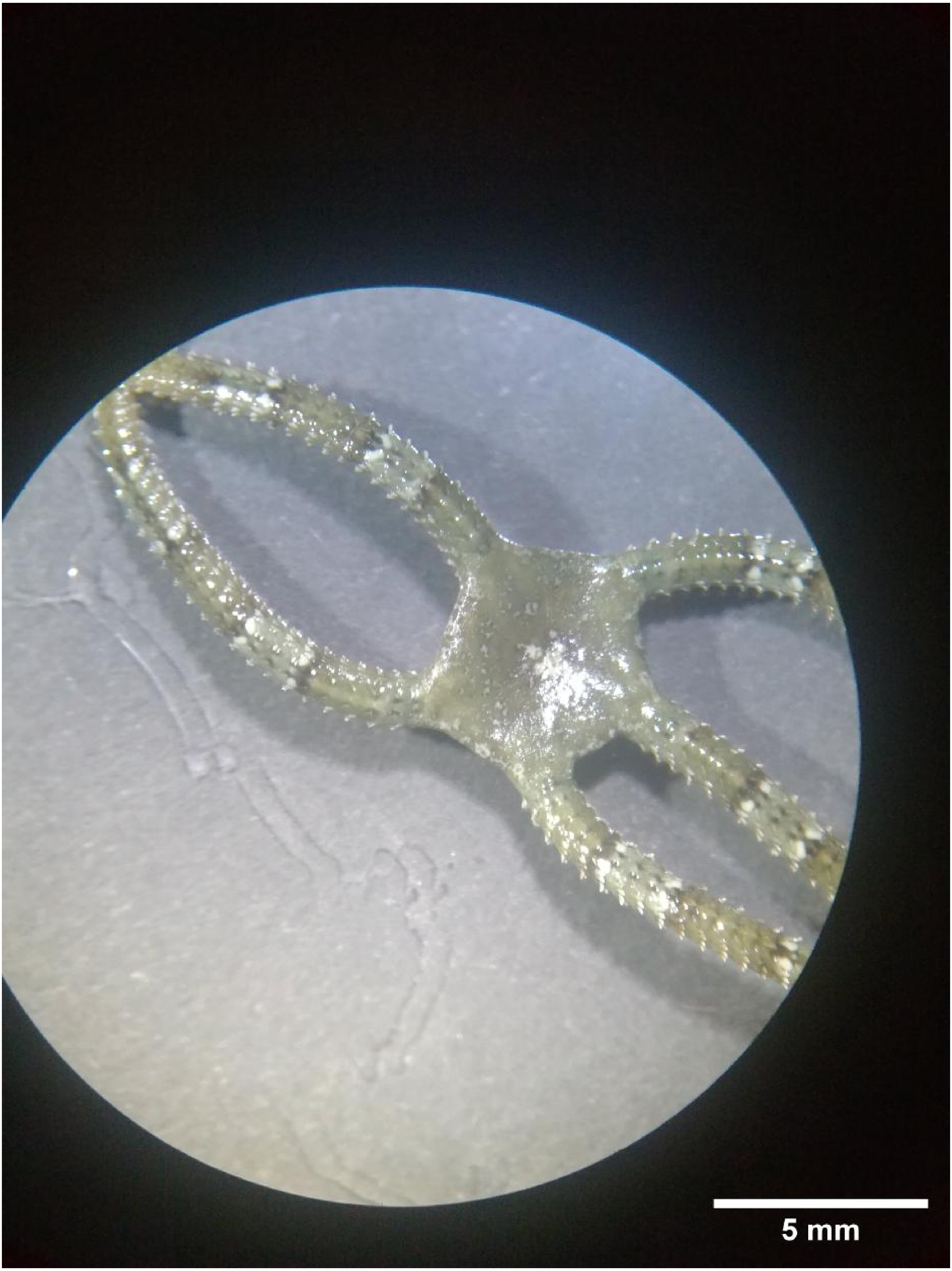
5 mm

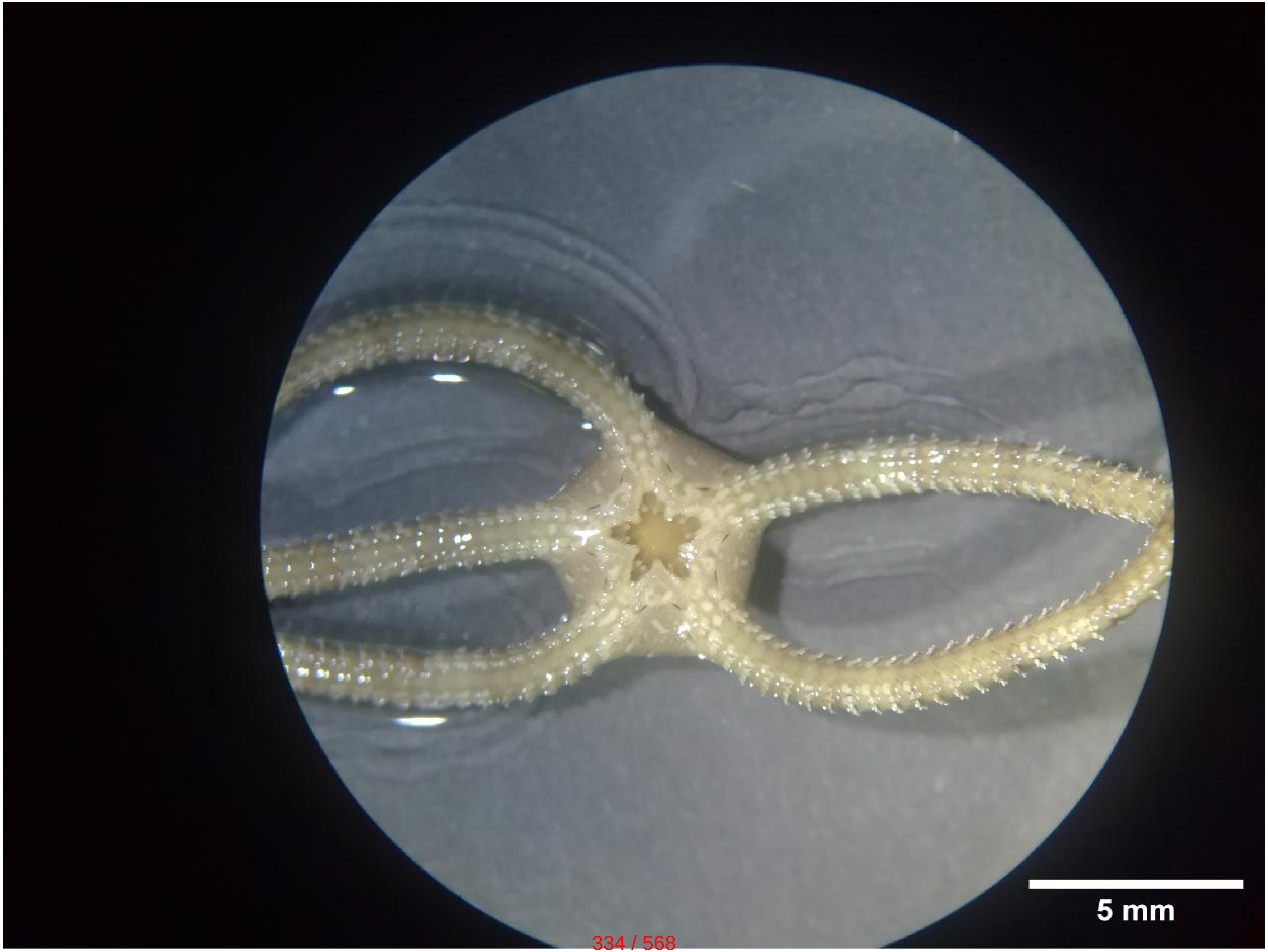
5 mm

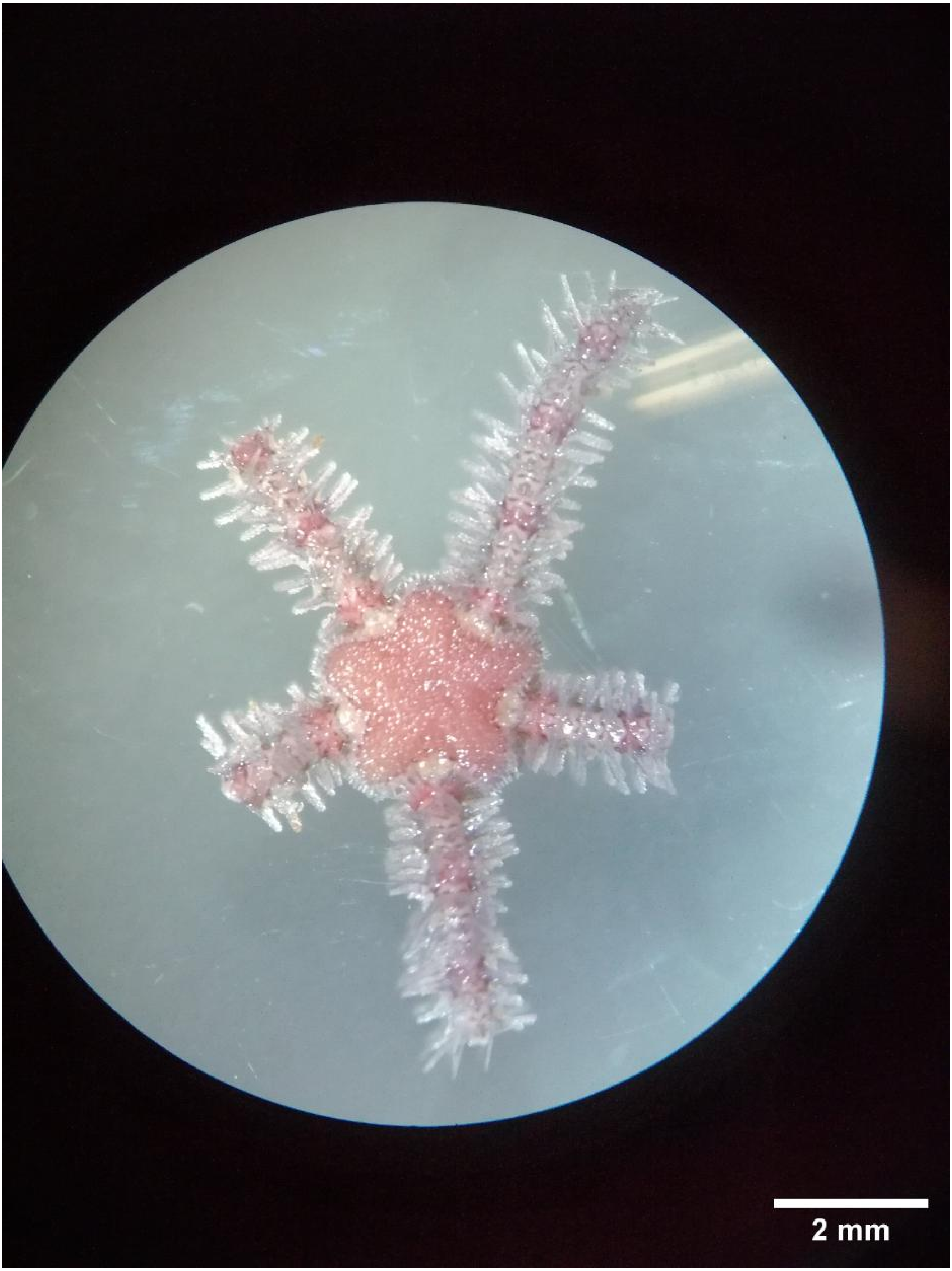
2 mm

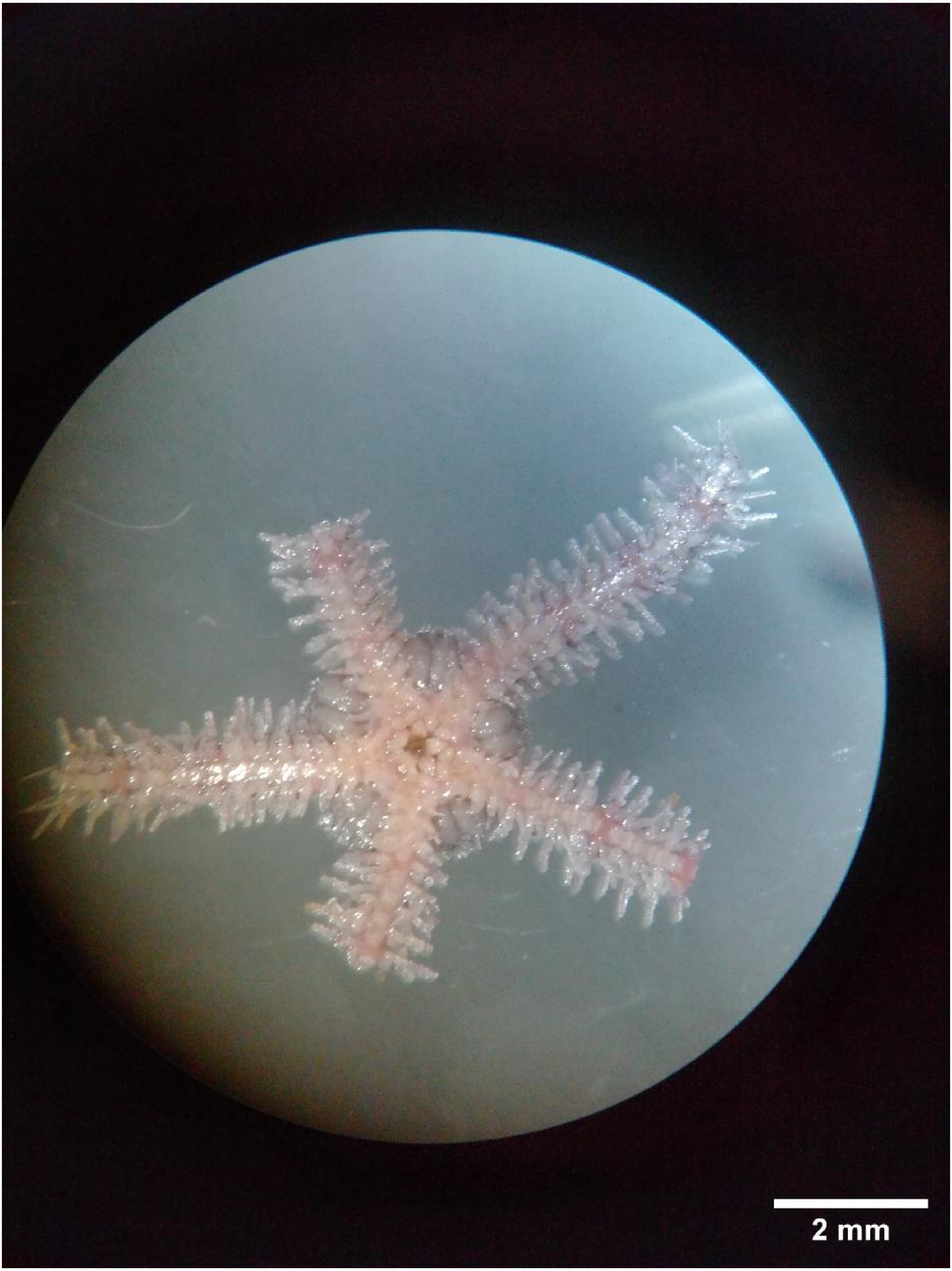
2 mm

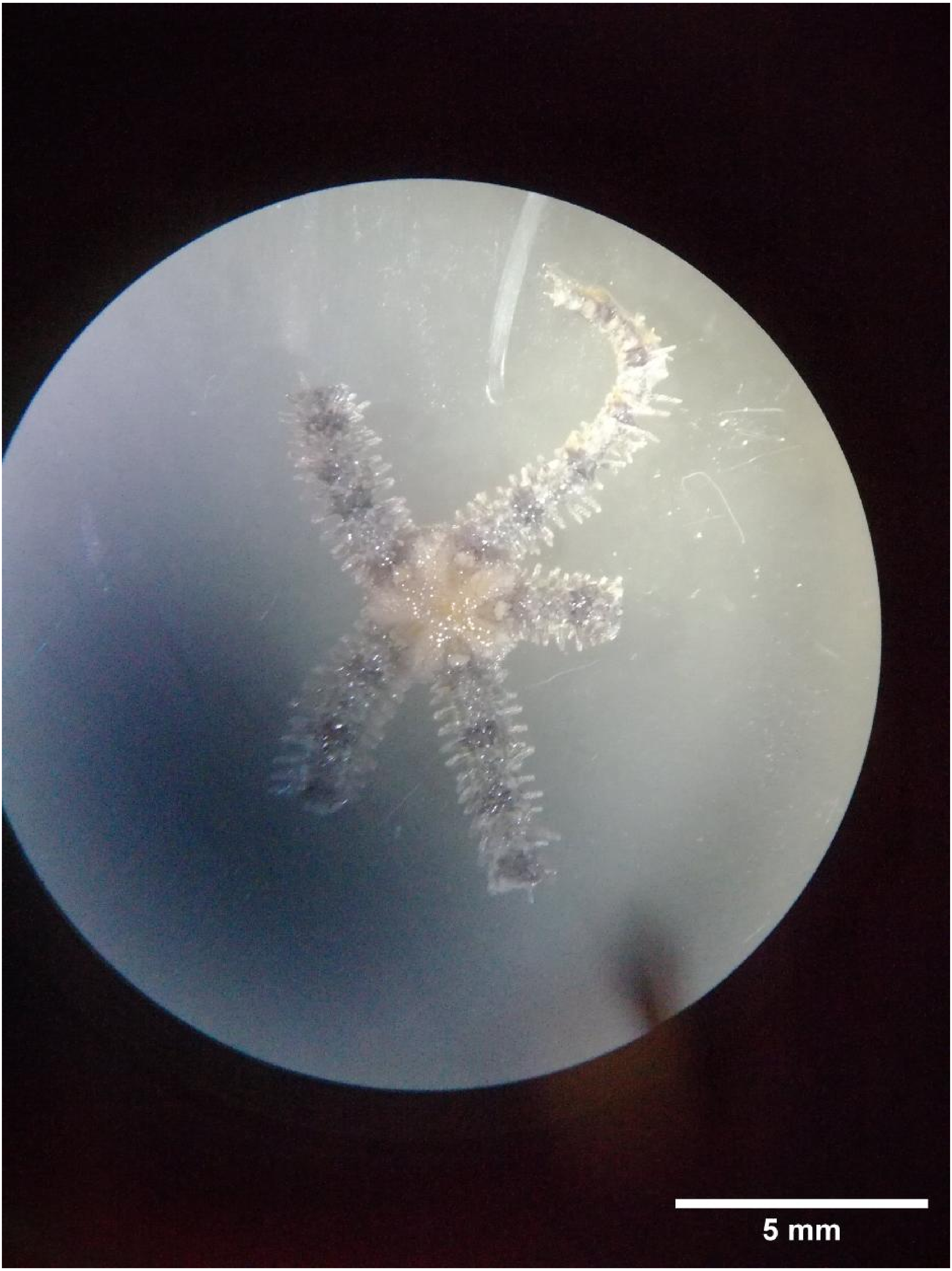
5 mm

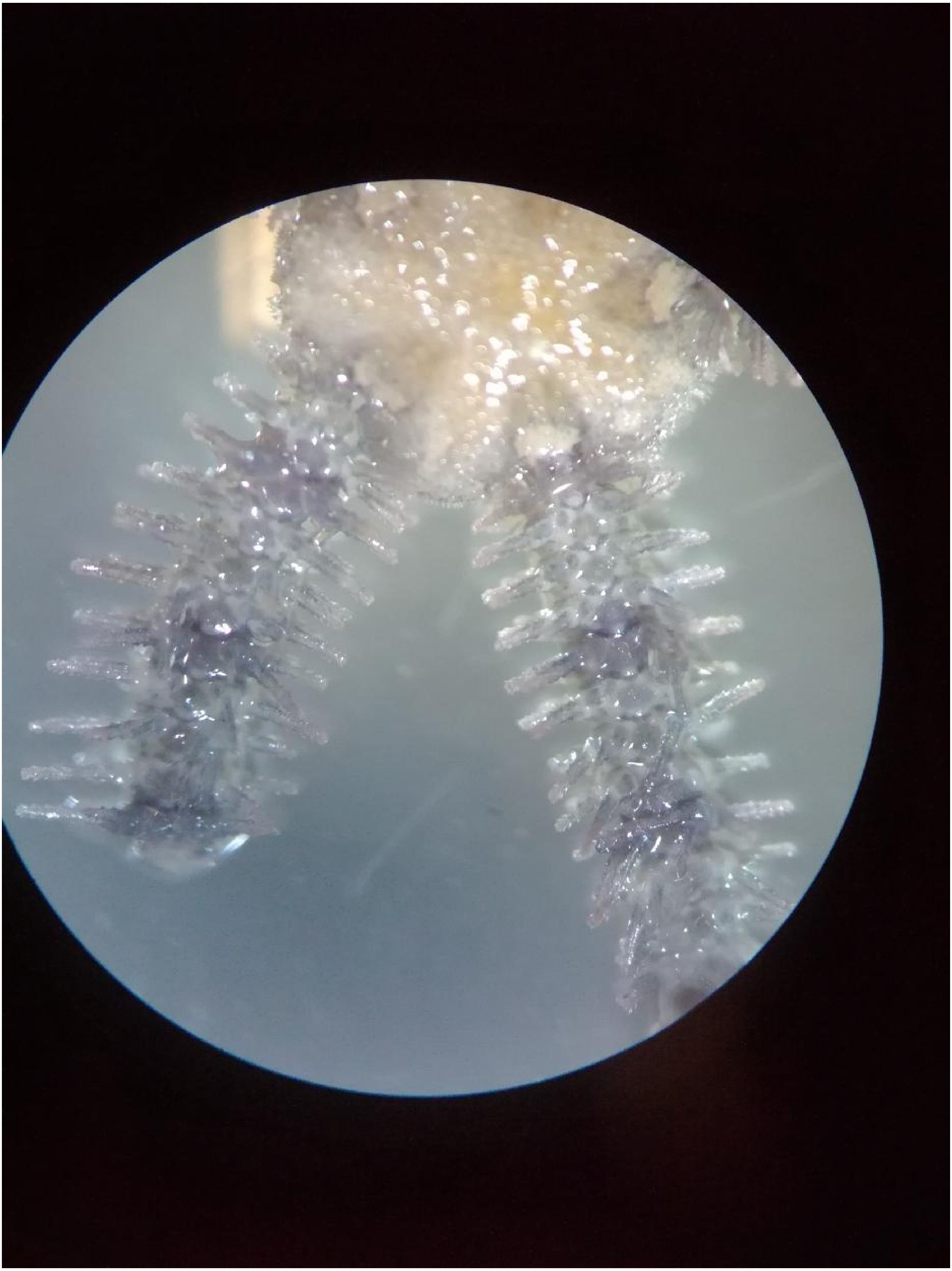

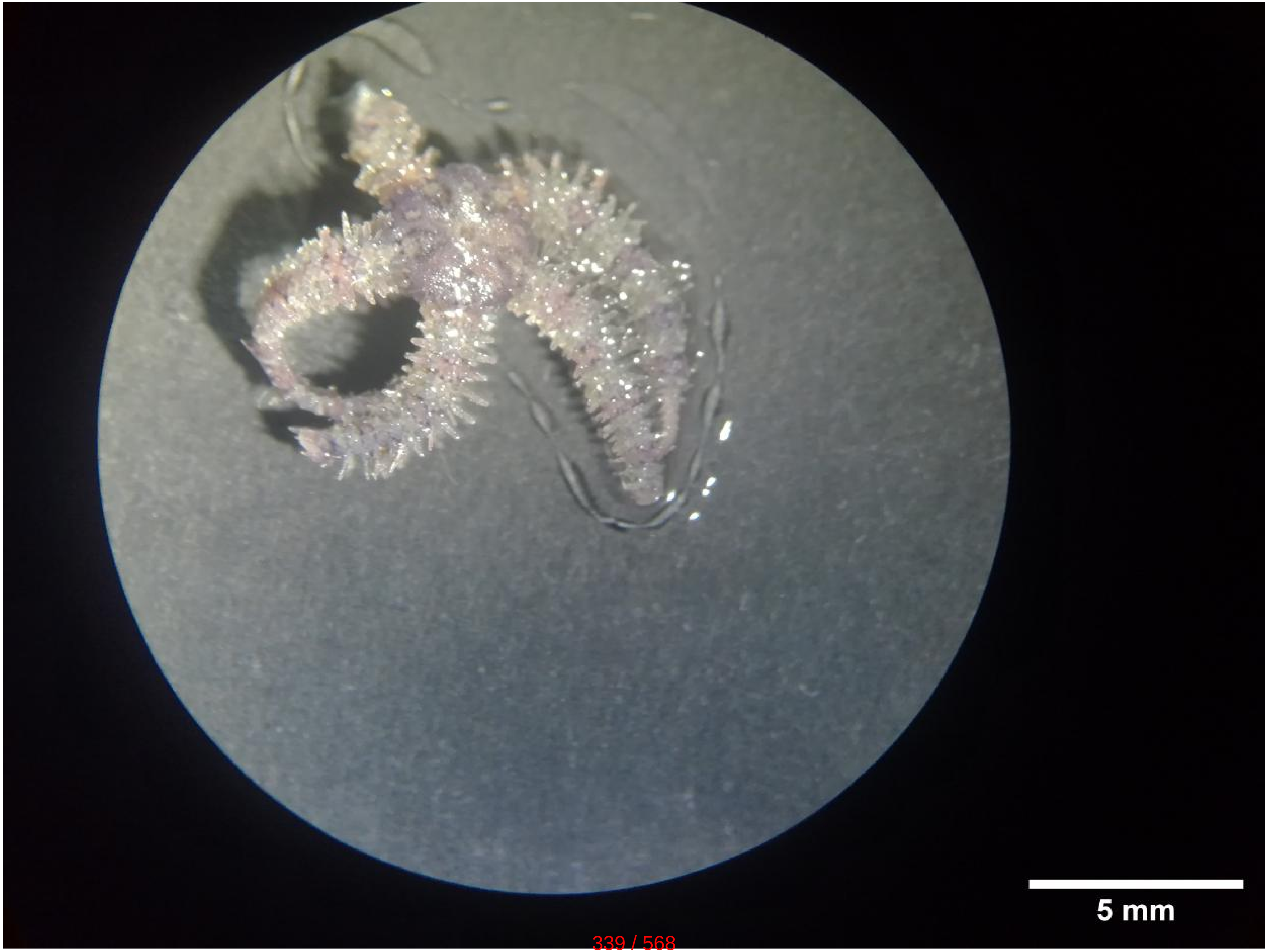
5 mm

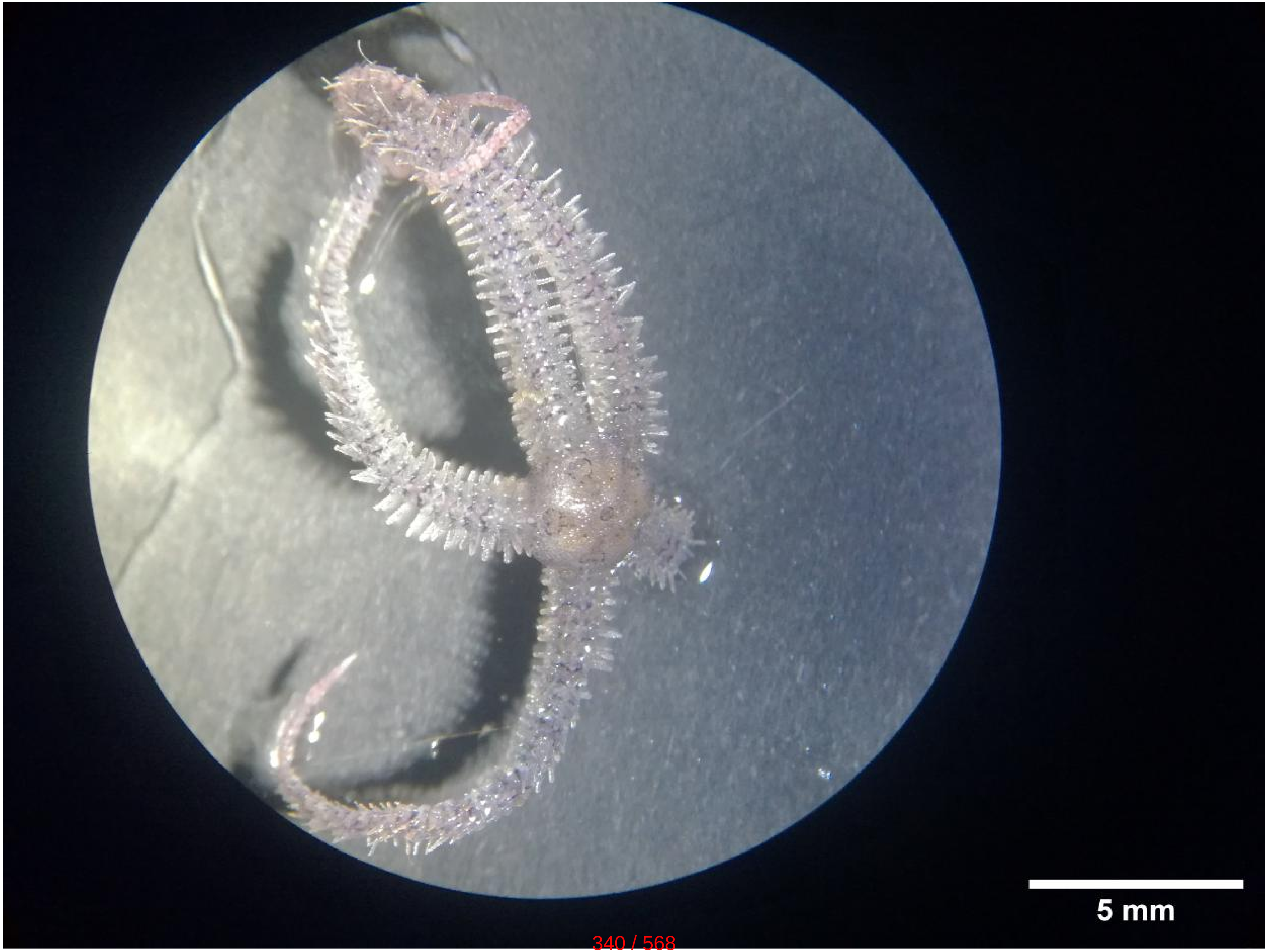
5 mm

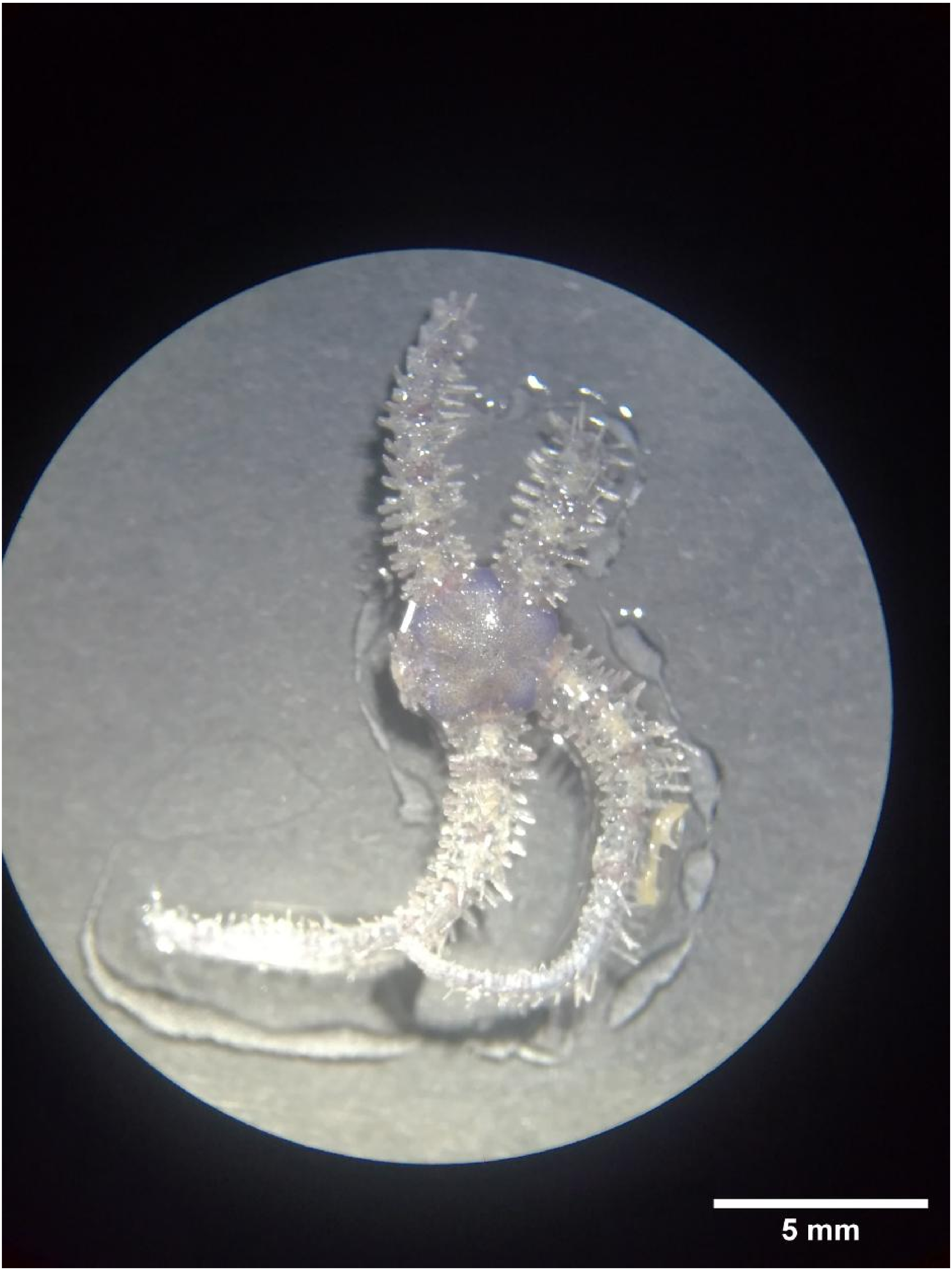
5 mm

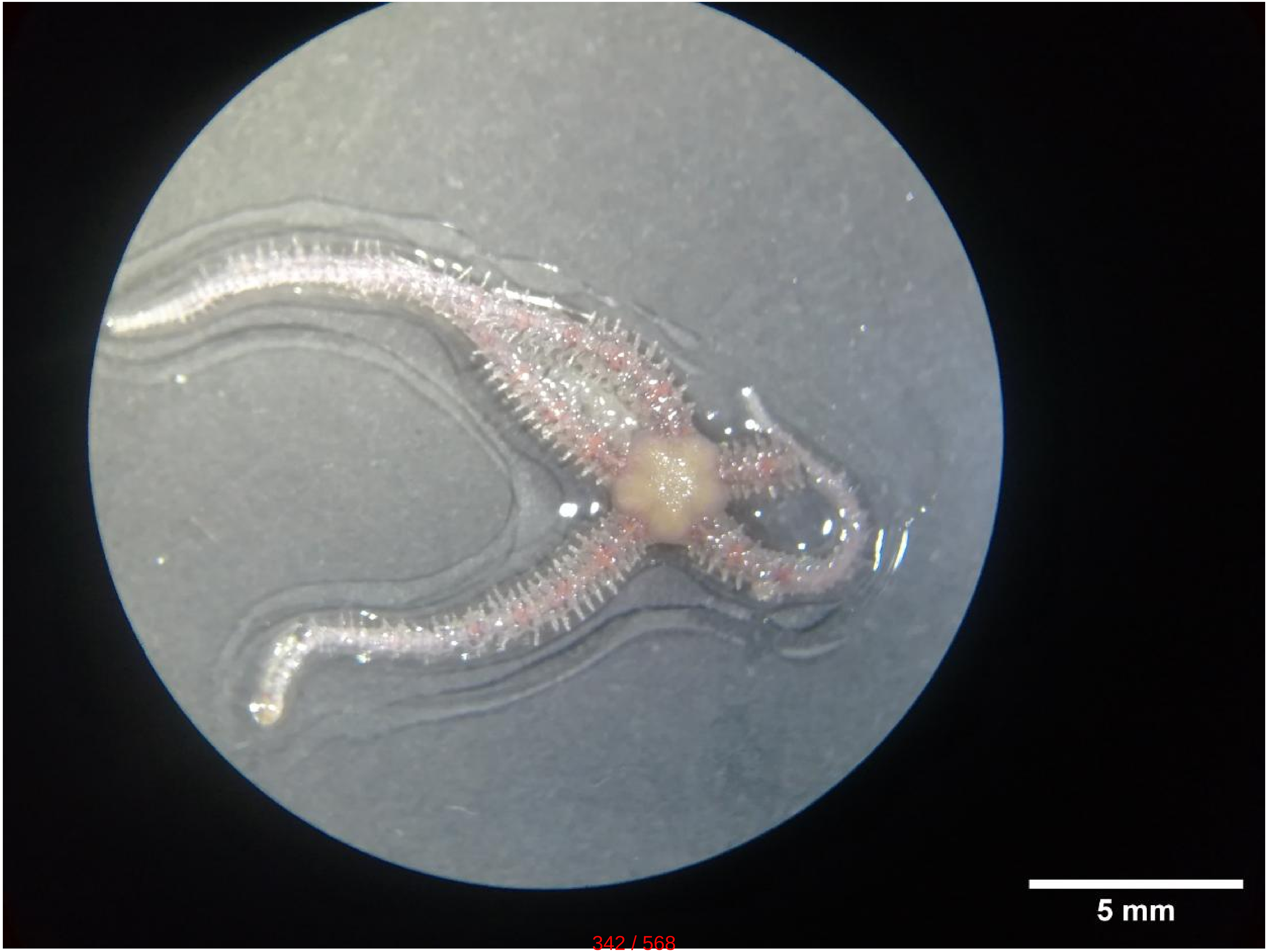
5 mm

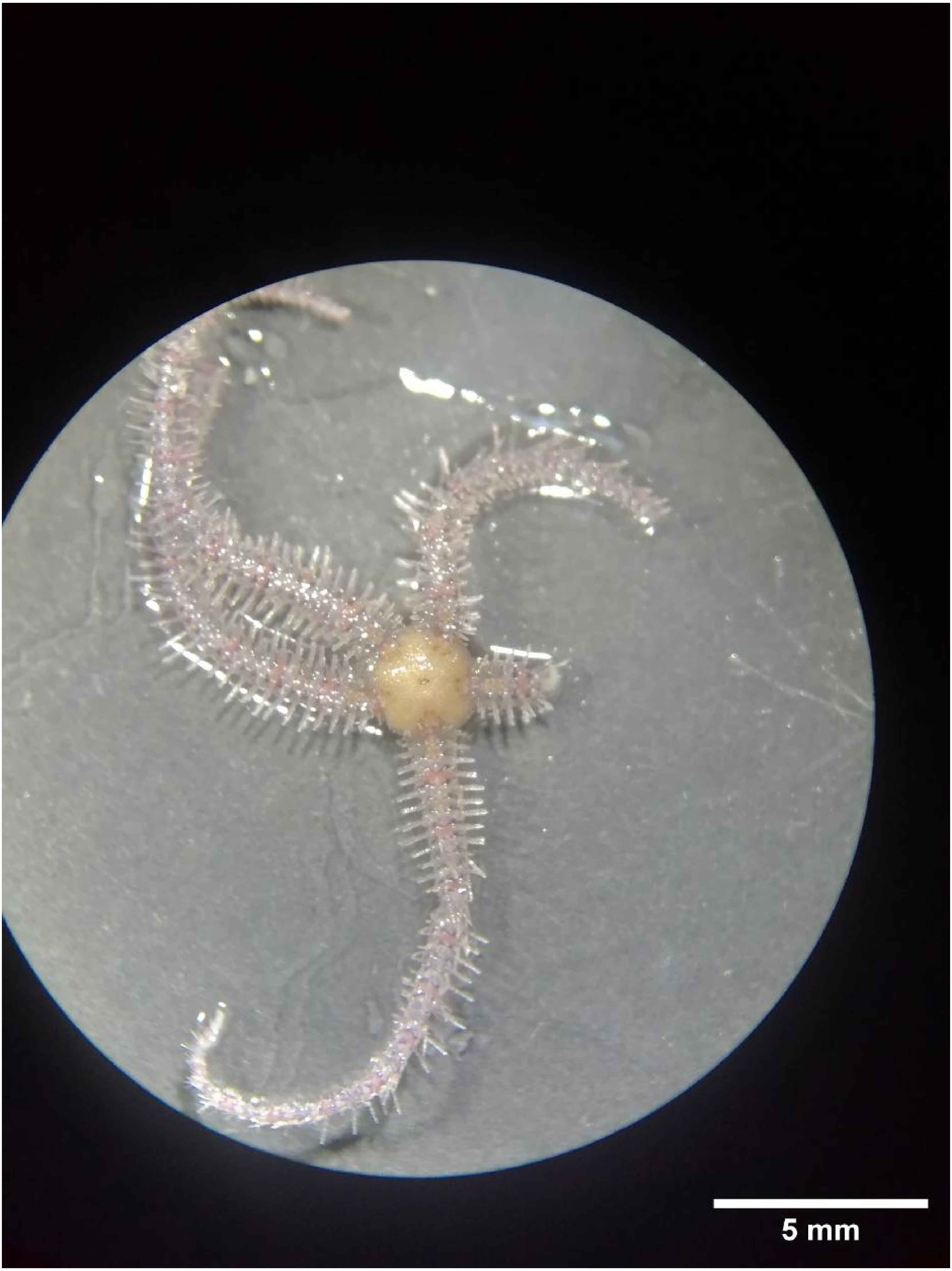
5 mm

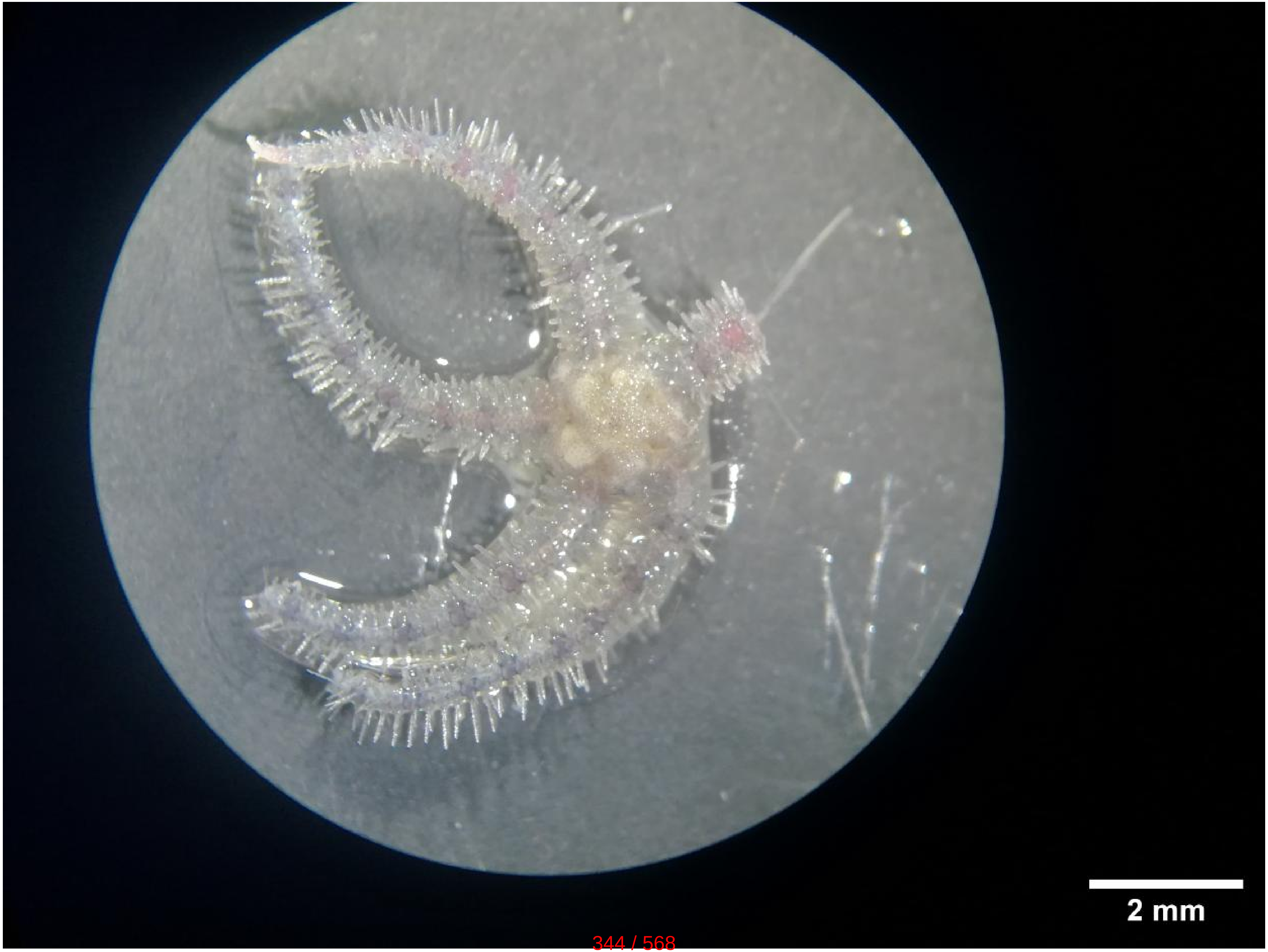
2 mm

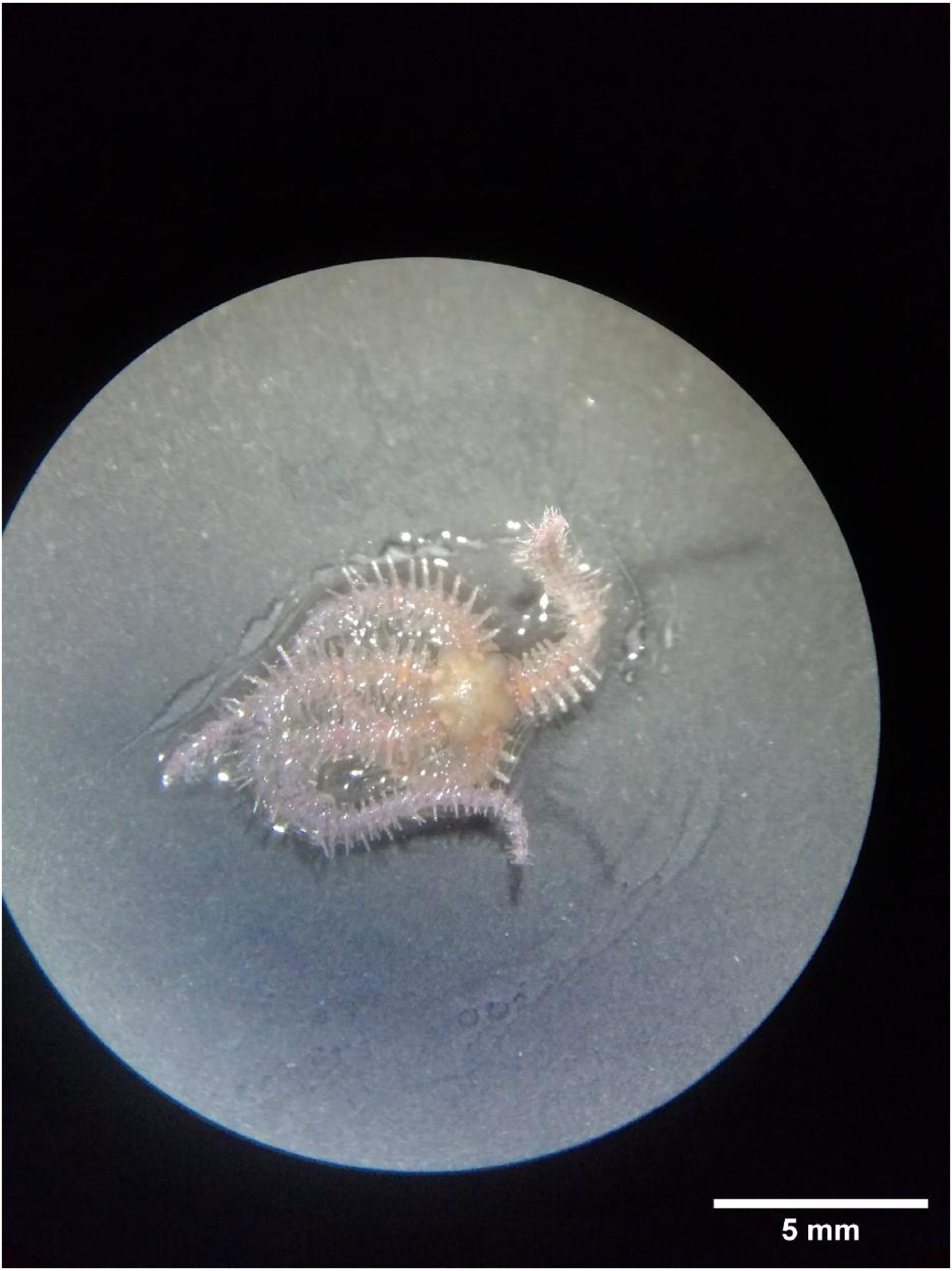
5 mm

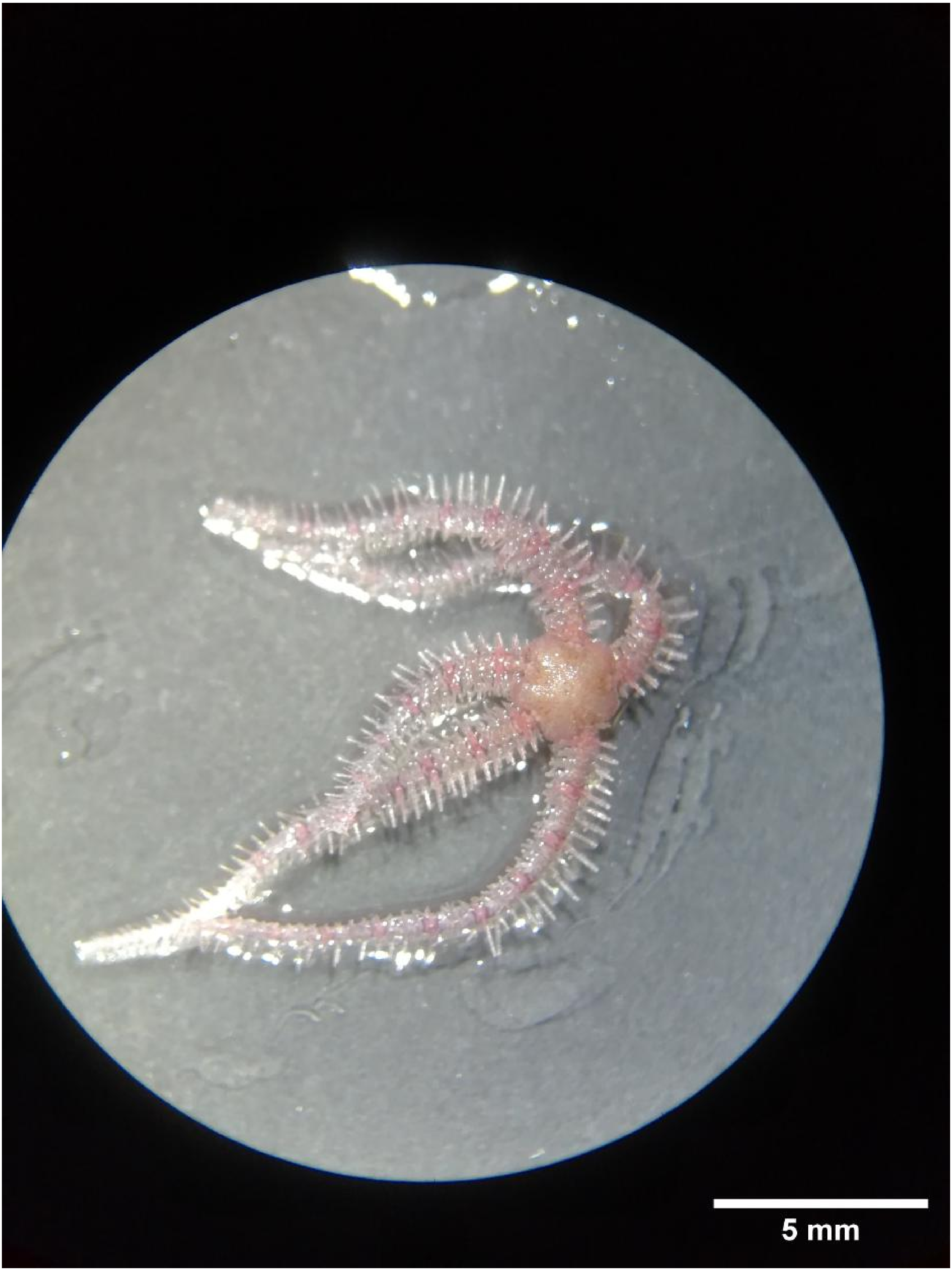
5 mm

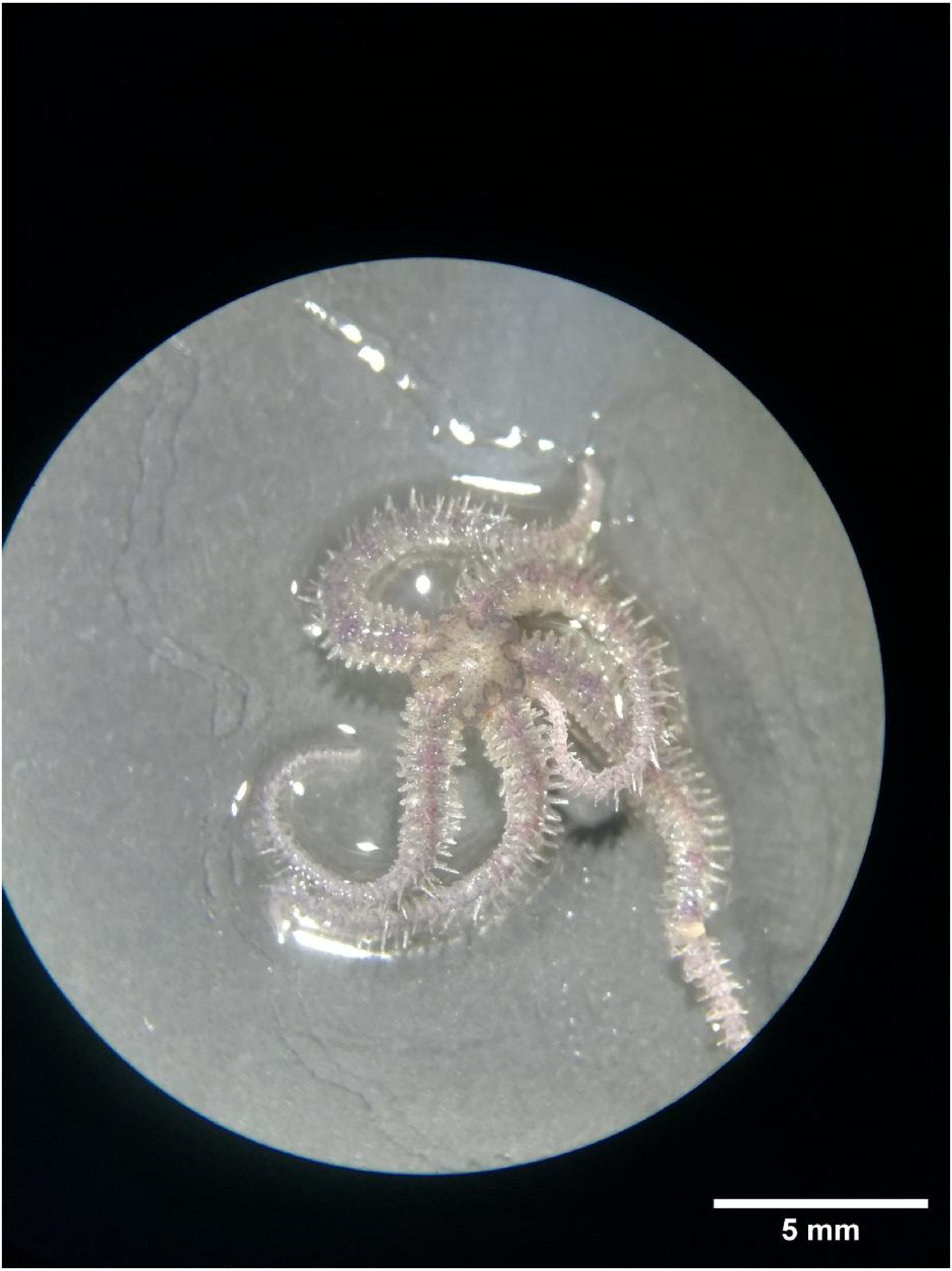
5 mm

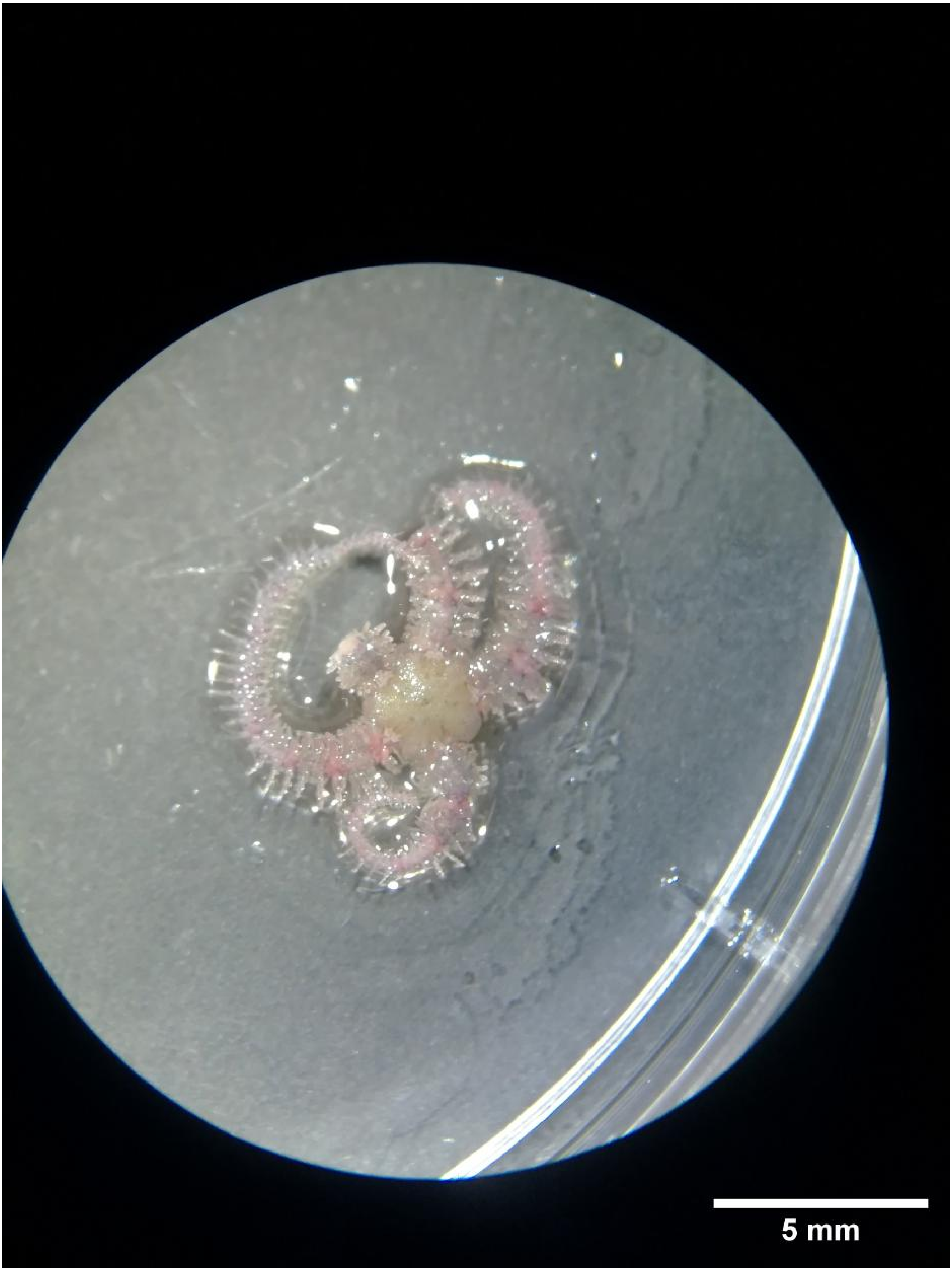
5 mm

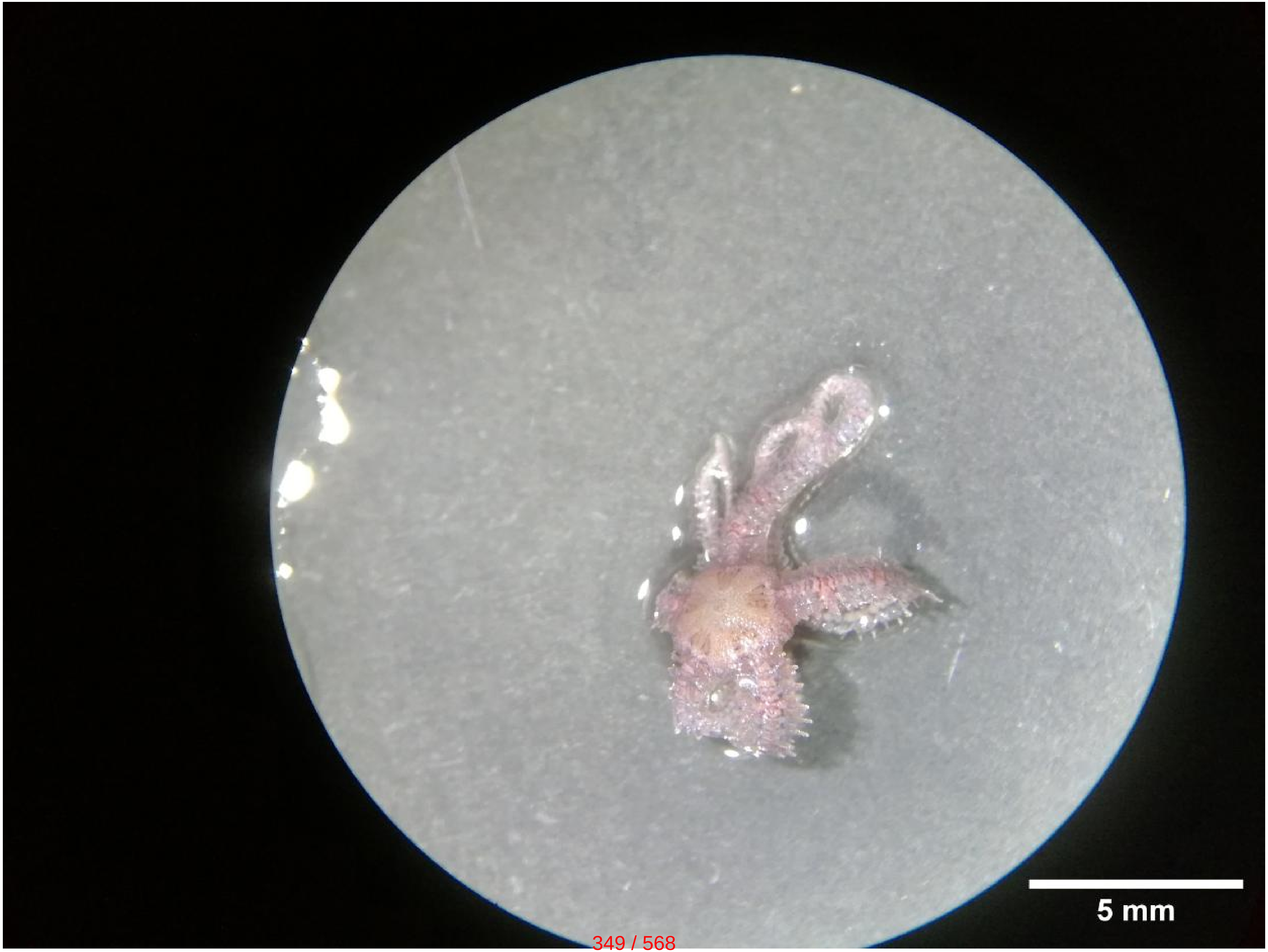
5 mm

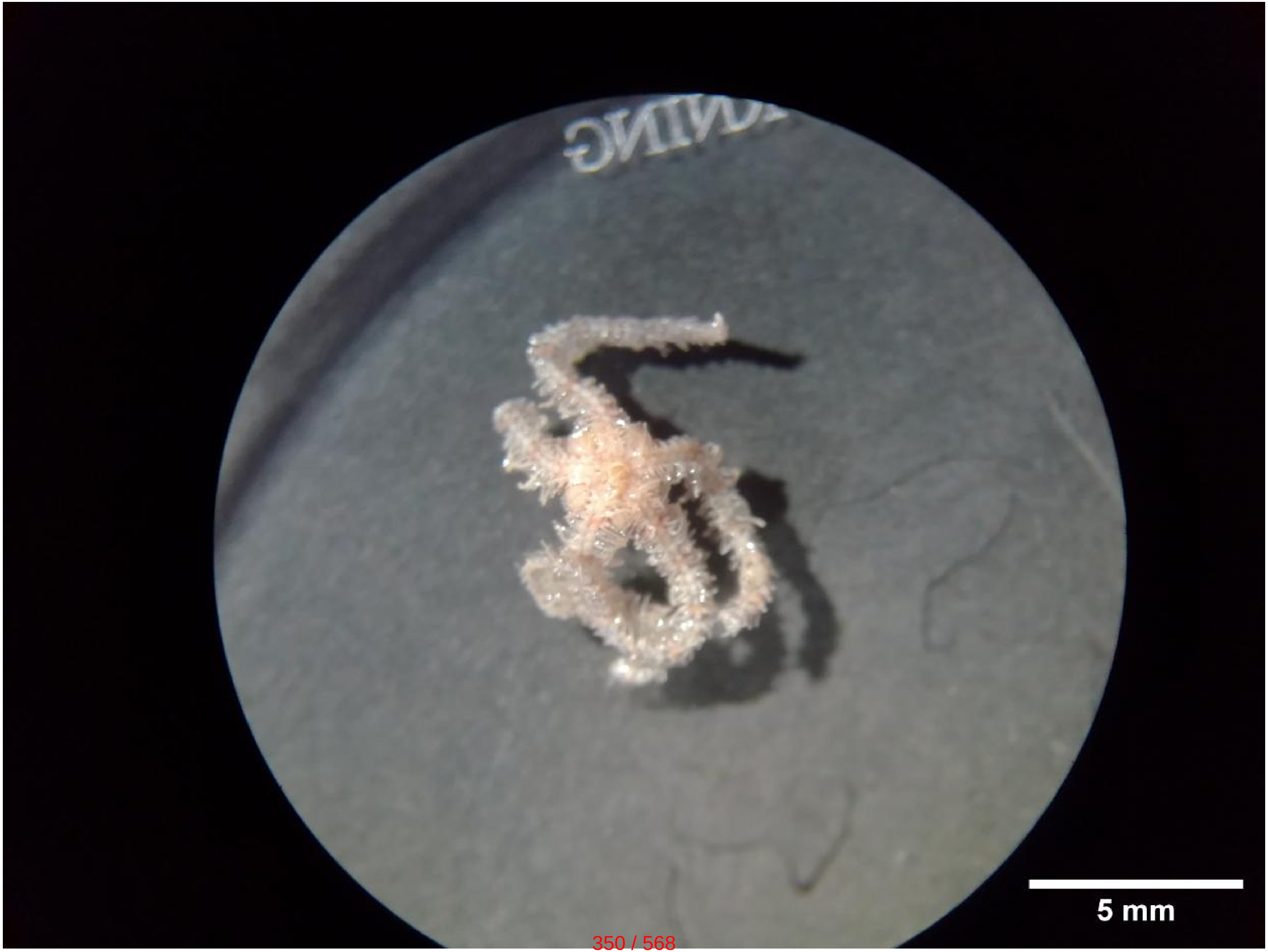
5 mm

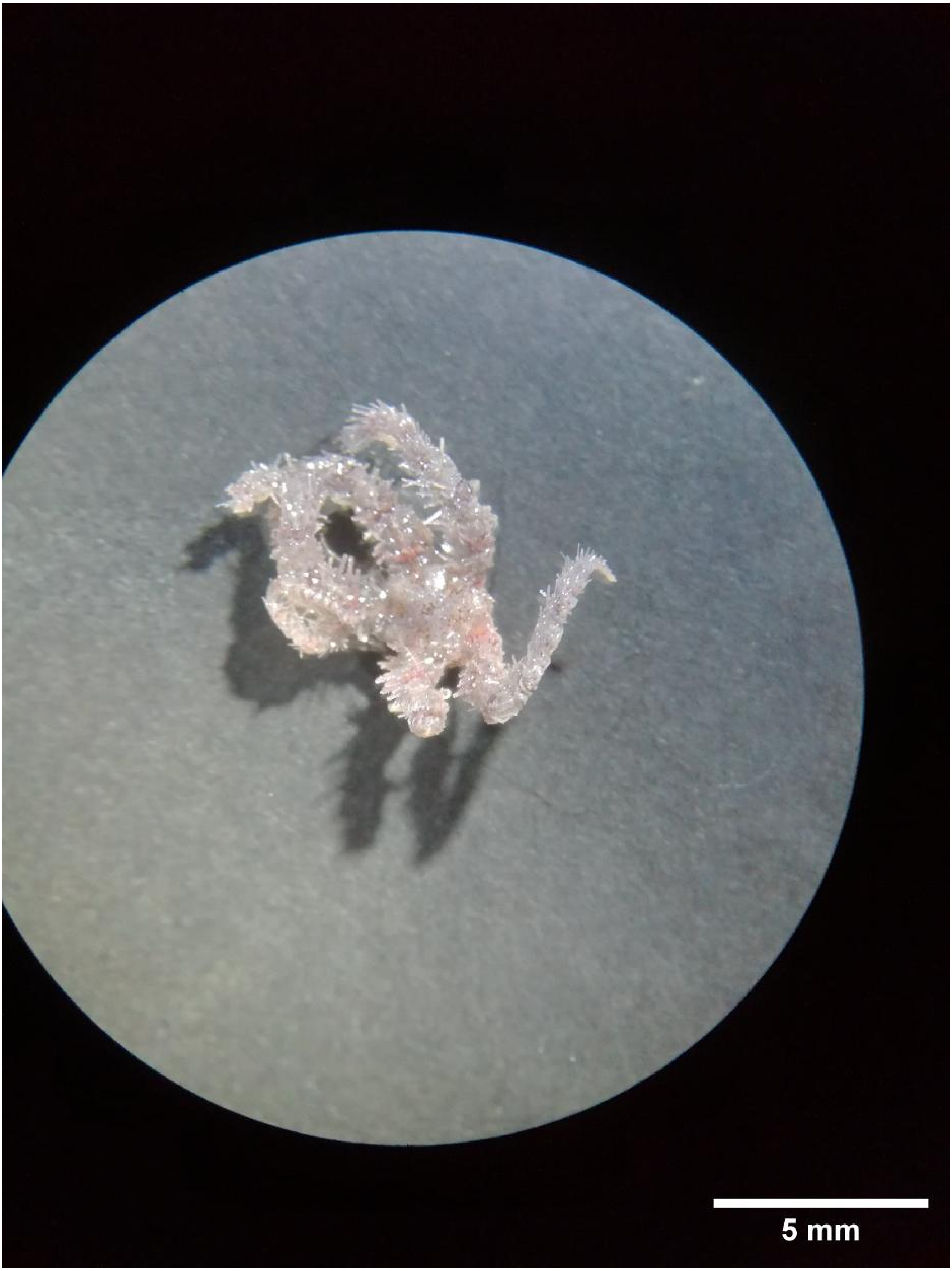
5 mm

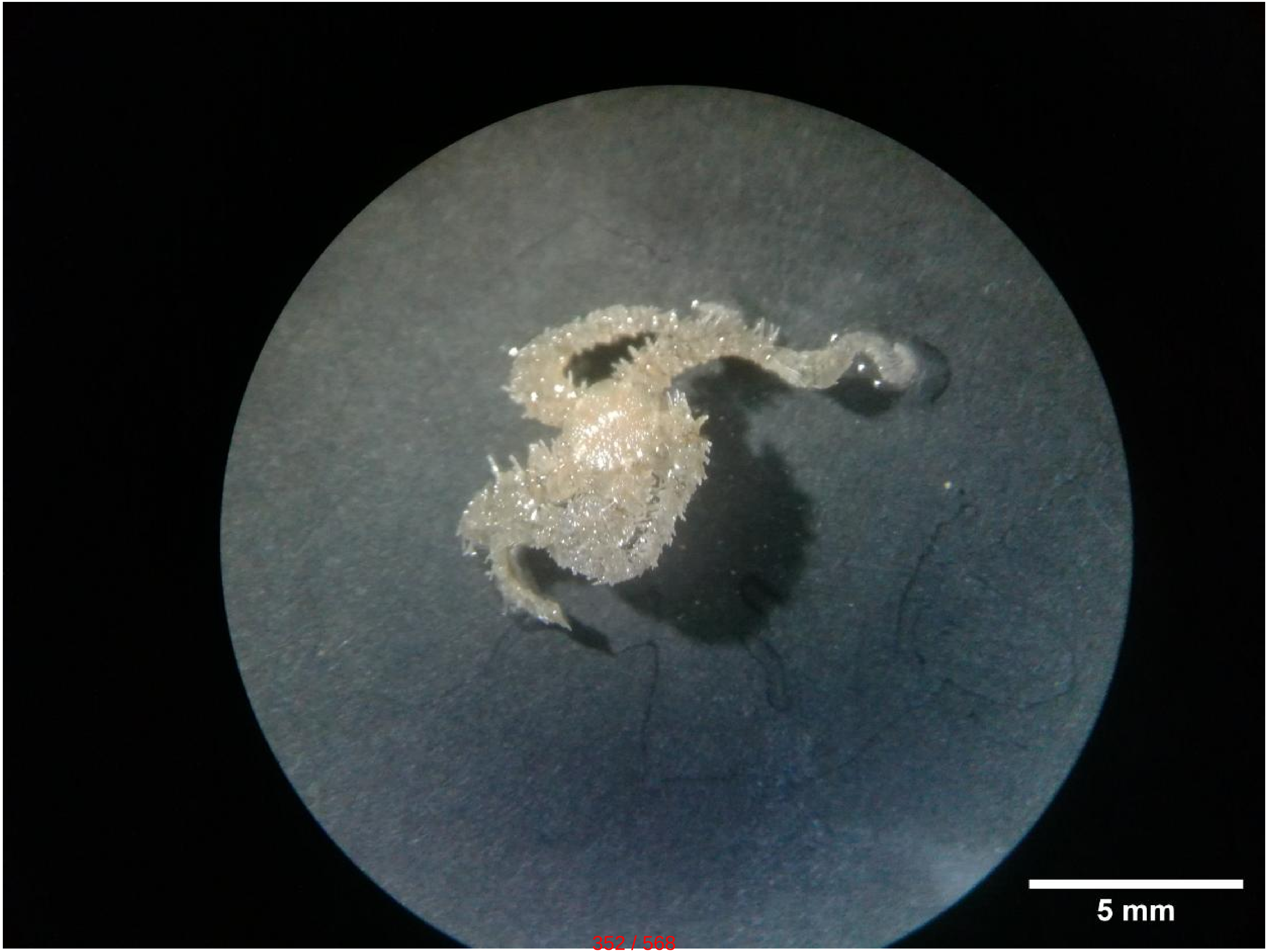
5 mm

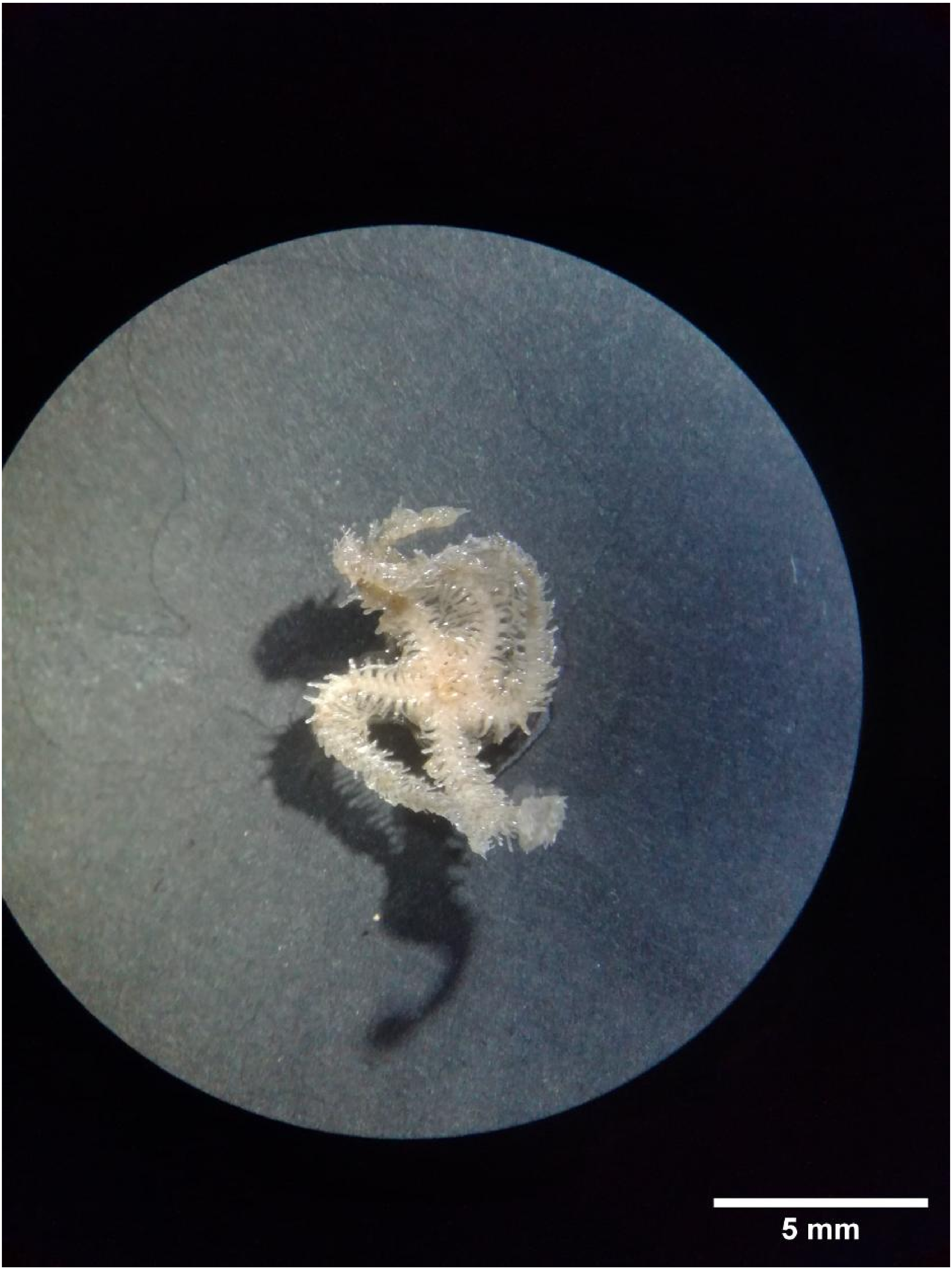
5 mm

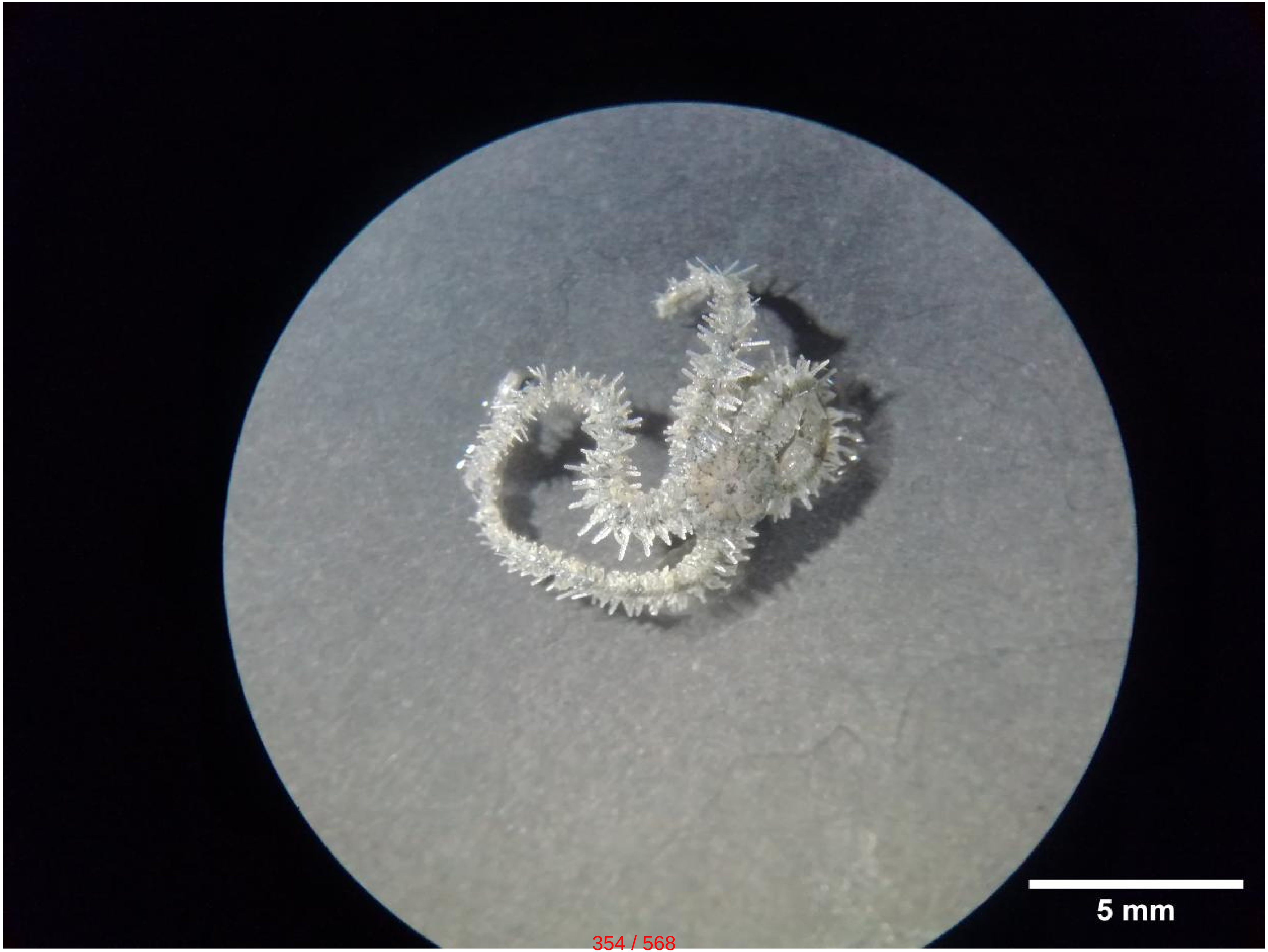
5 mm

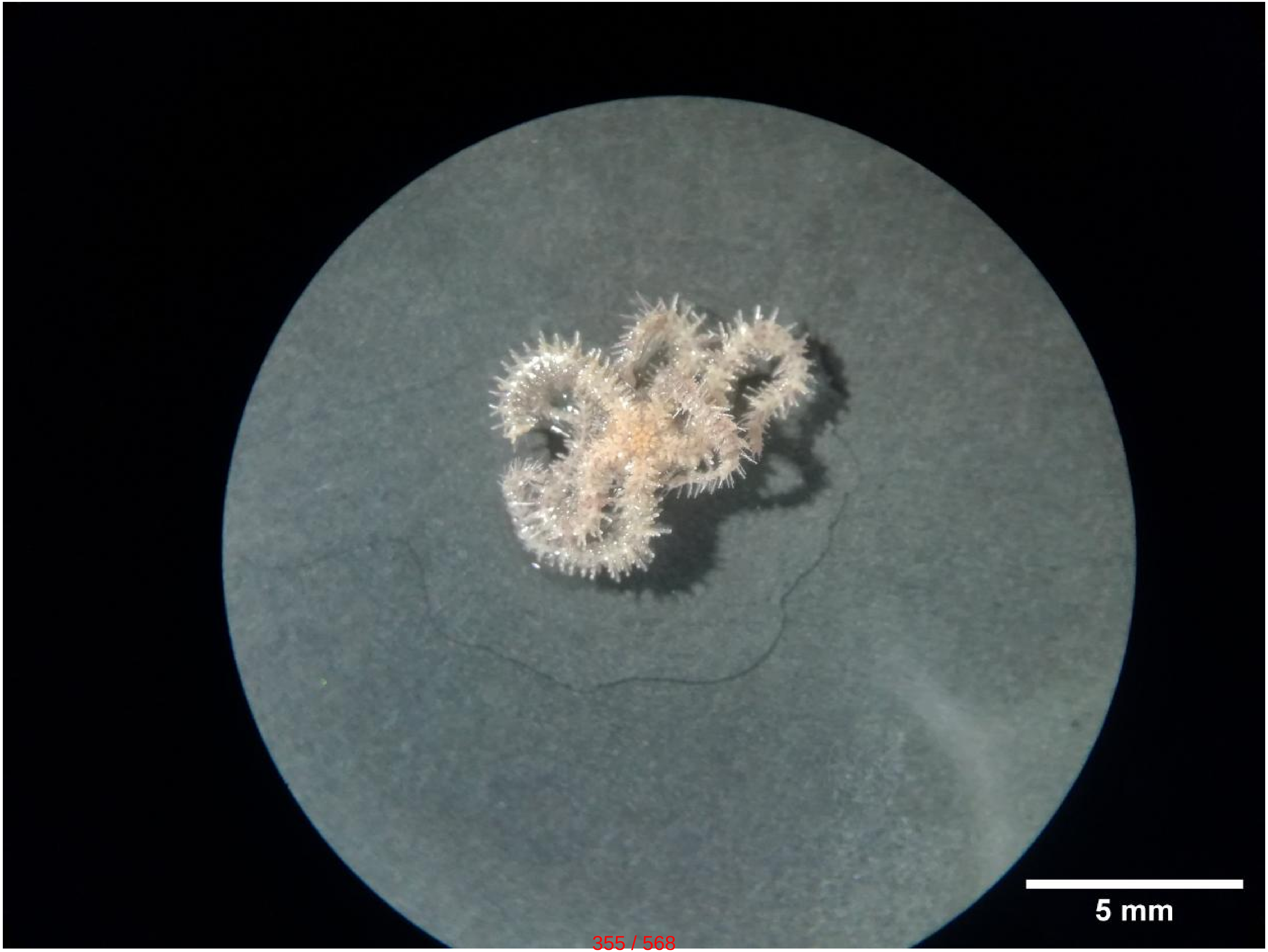
5 mm

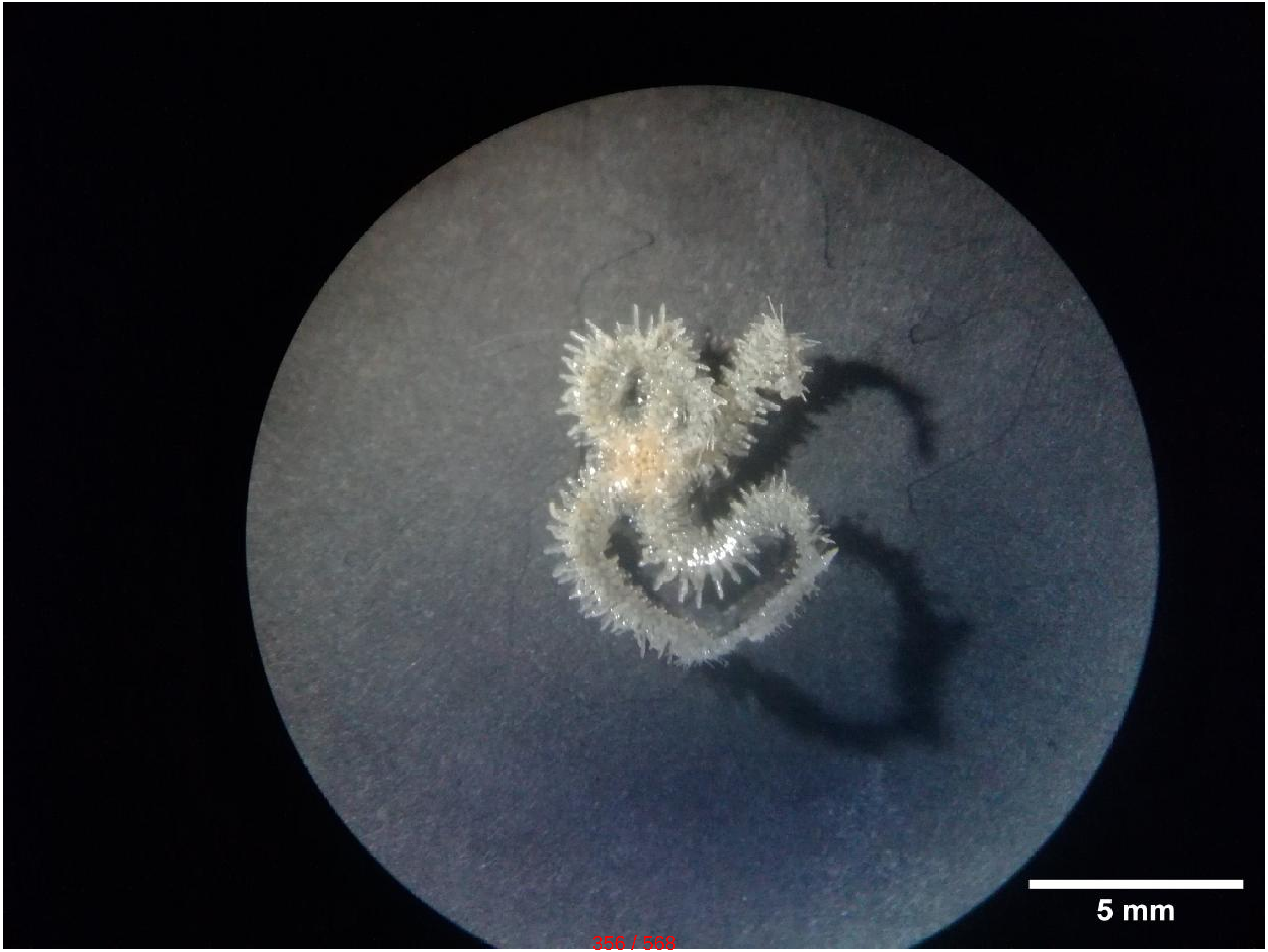
5 mm

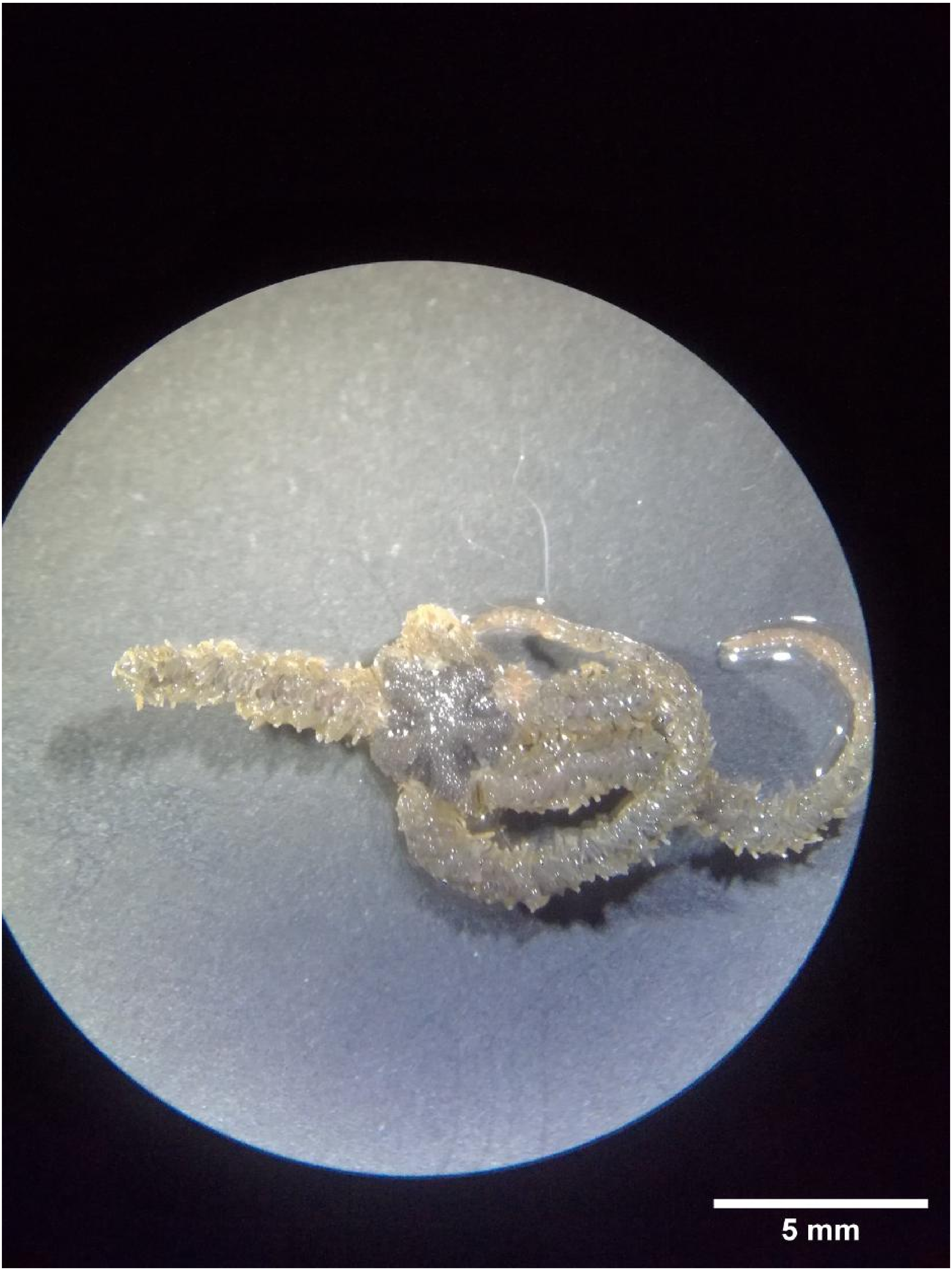
5 mm

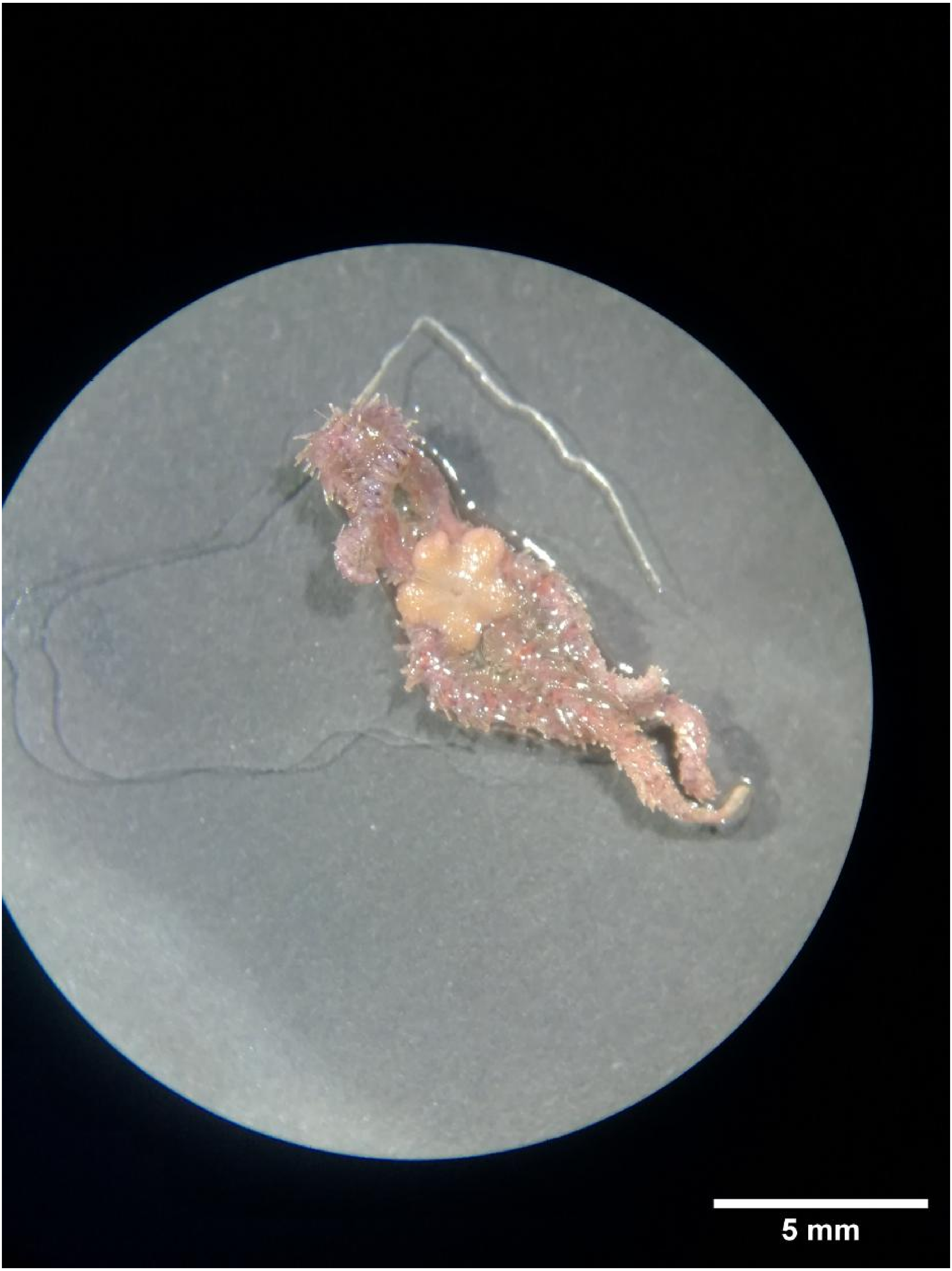
5 mm

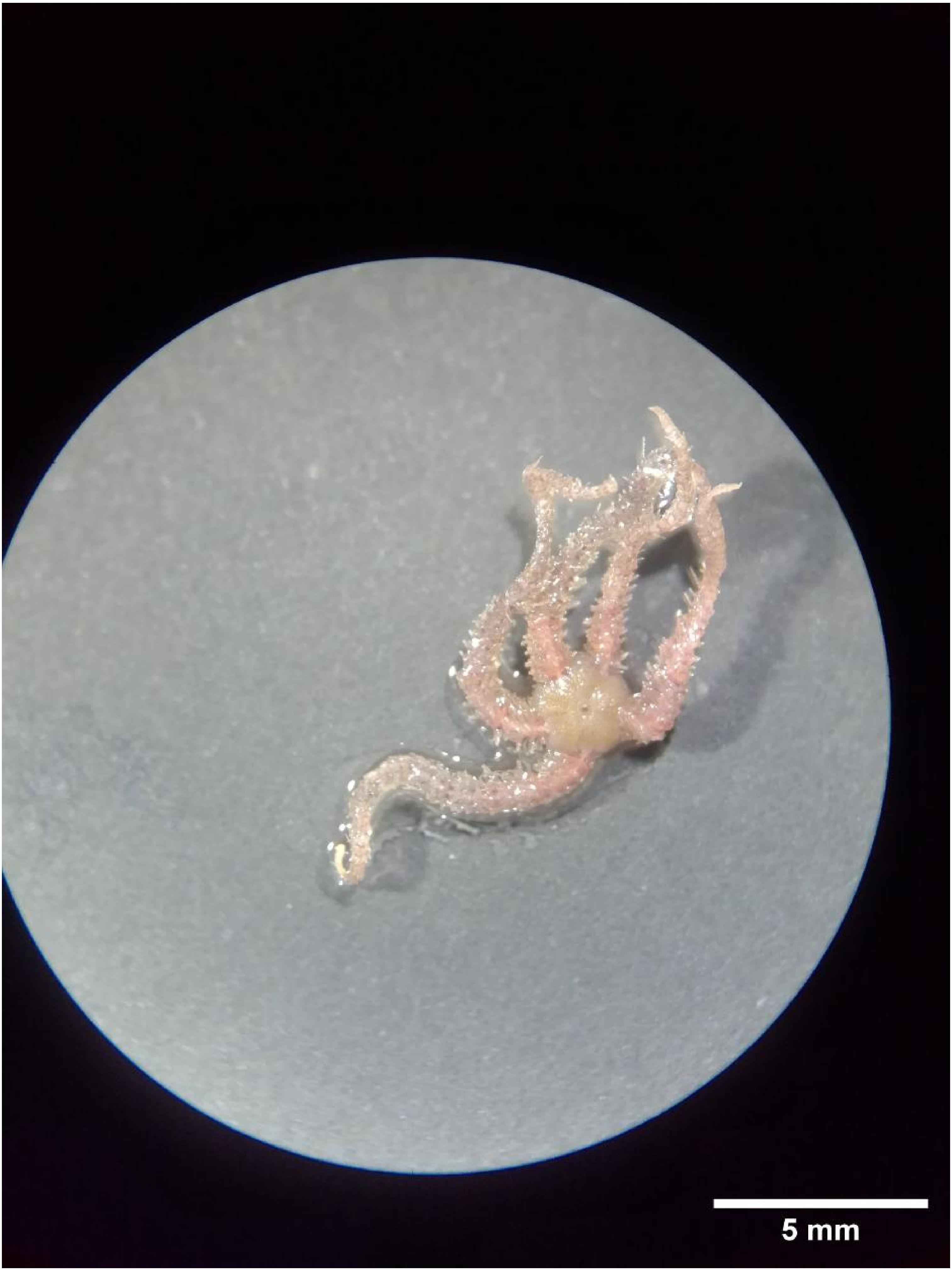
5 mm

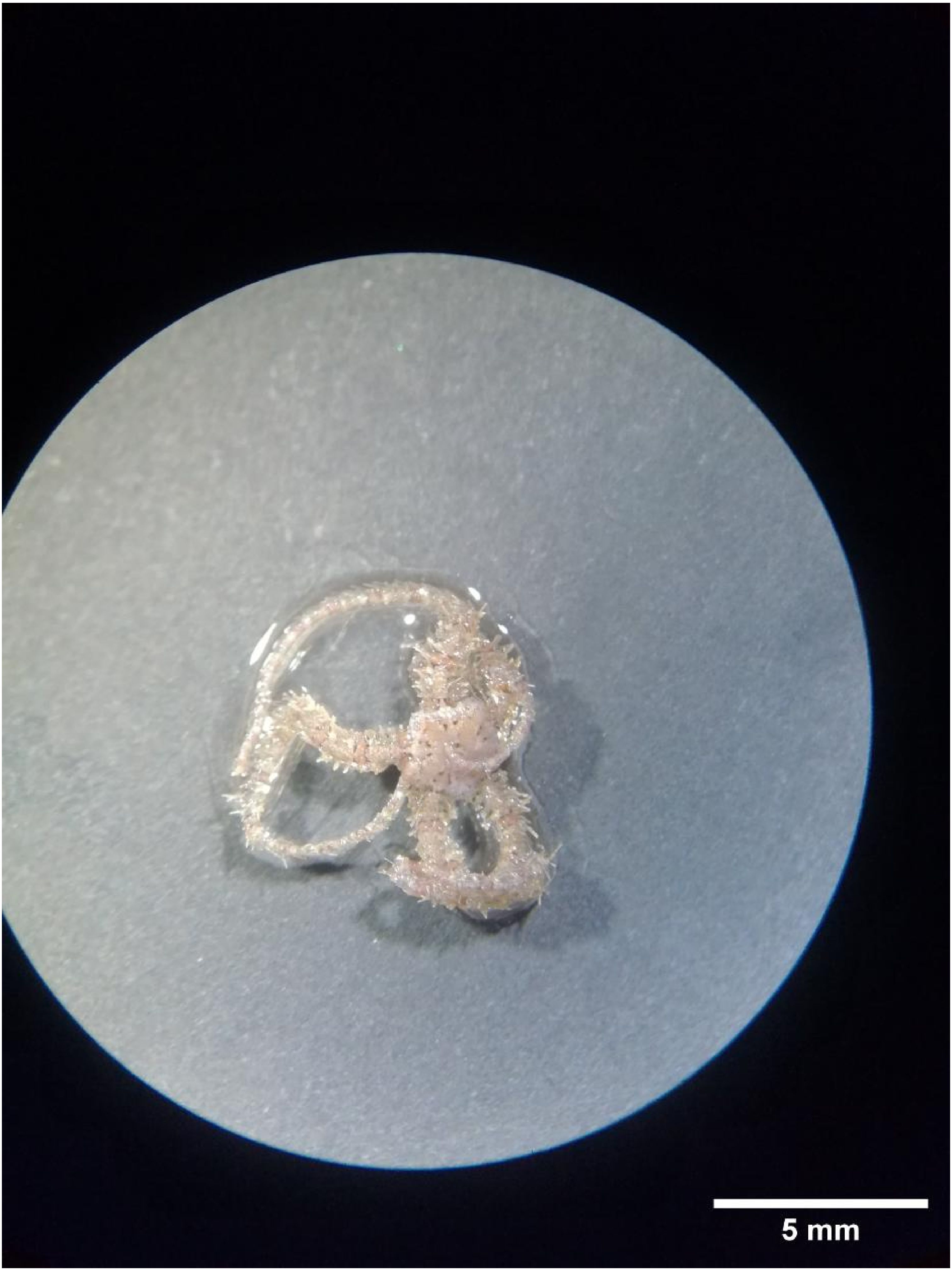
5 mm

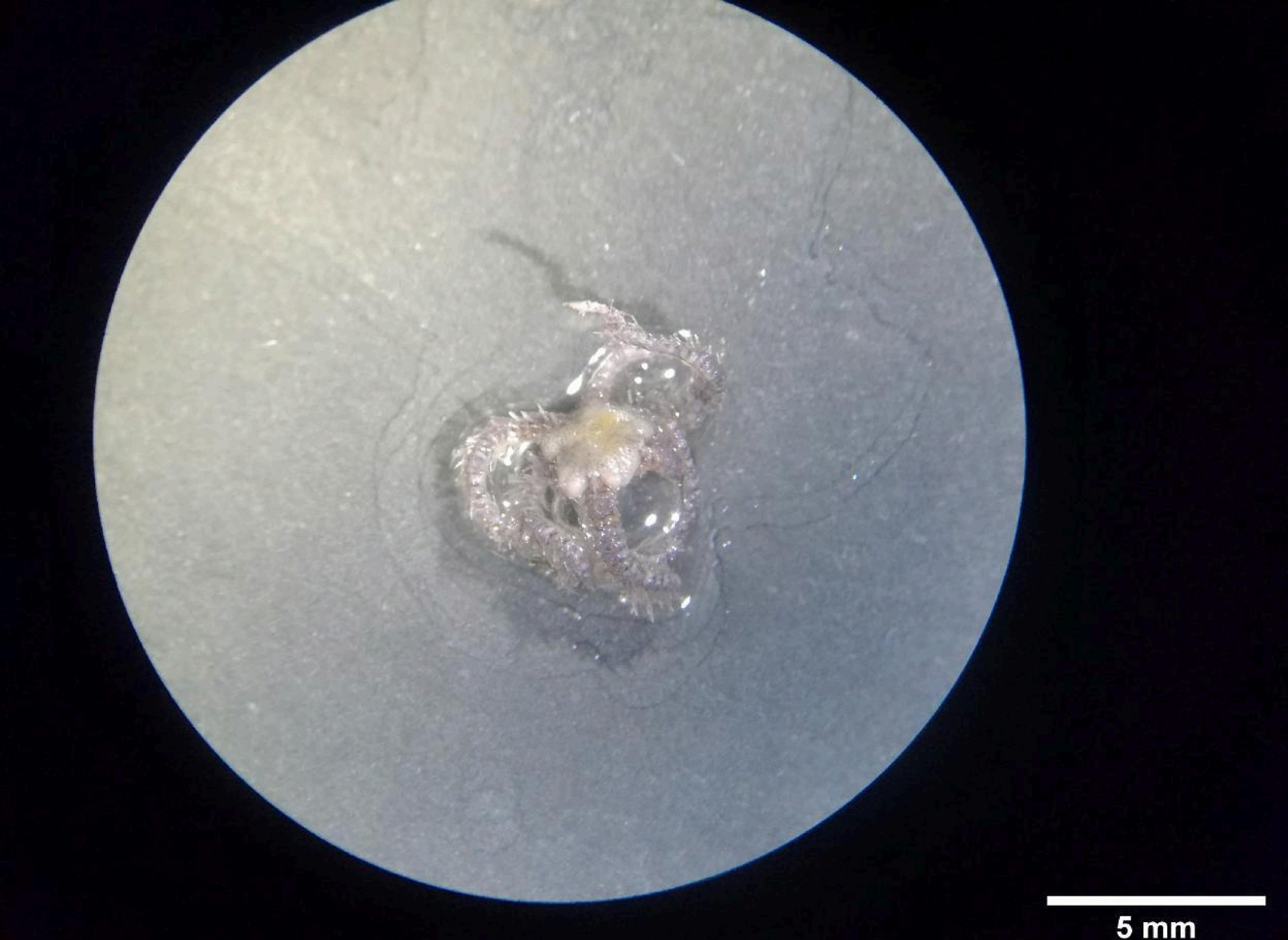
5 mm

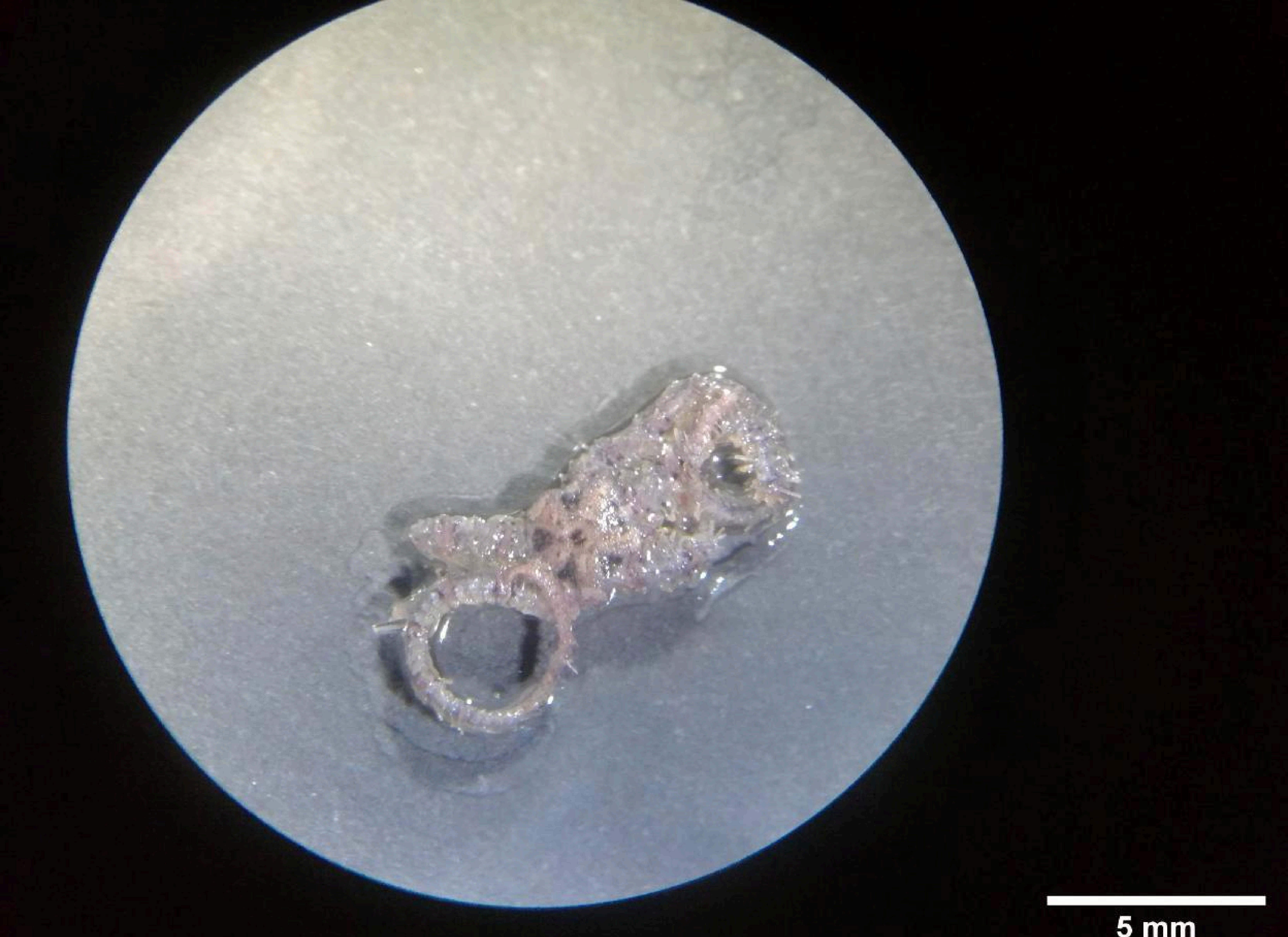
5 mm

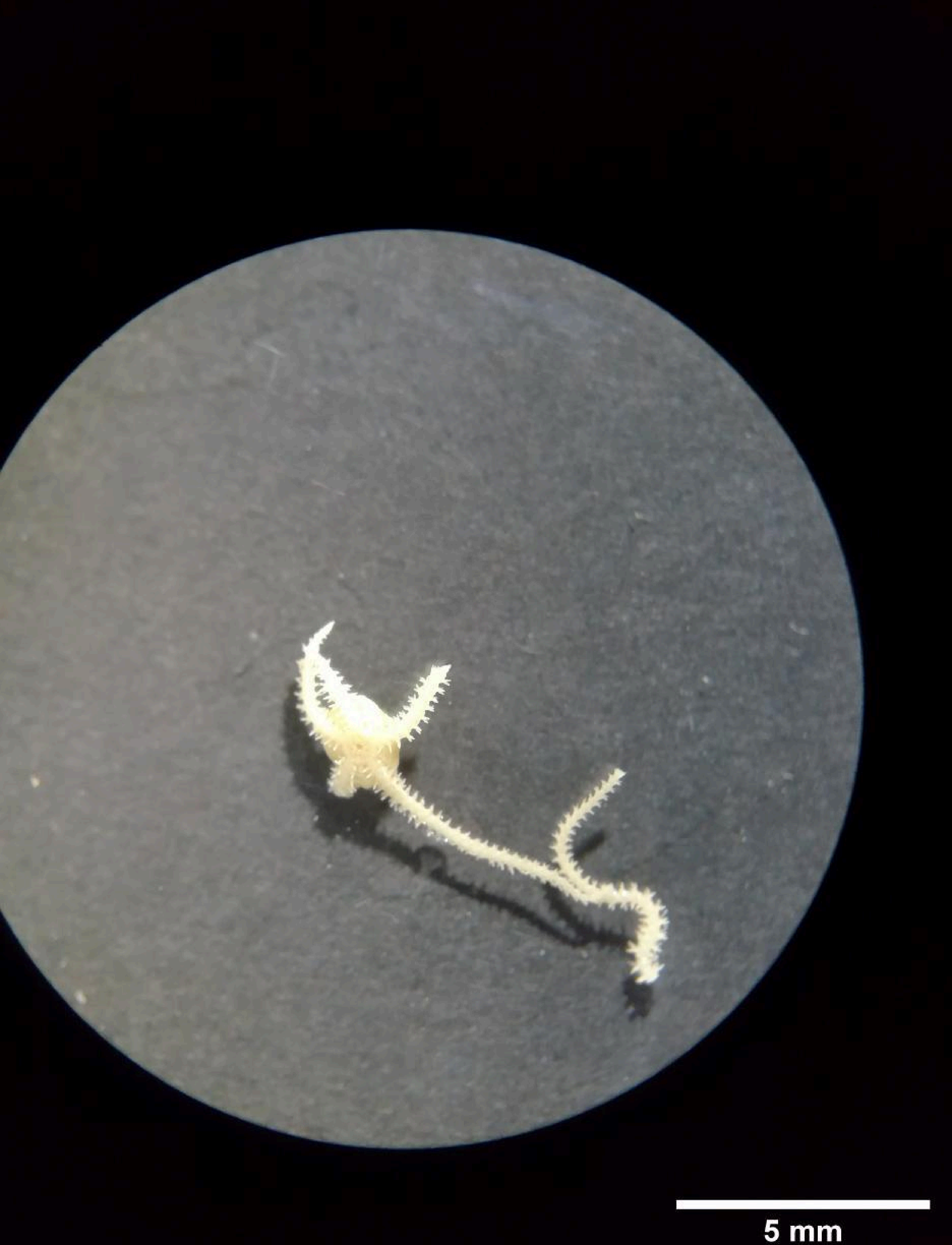
5 mm

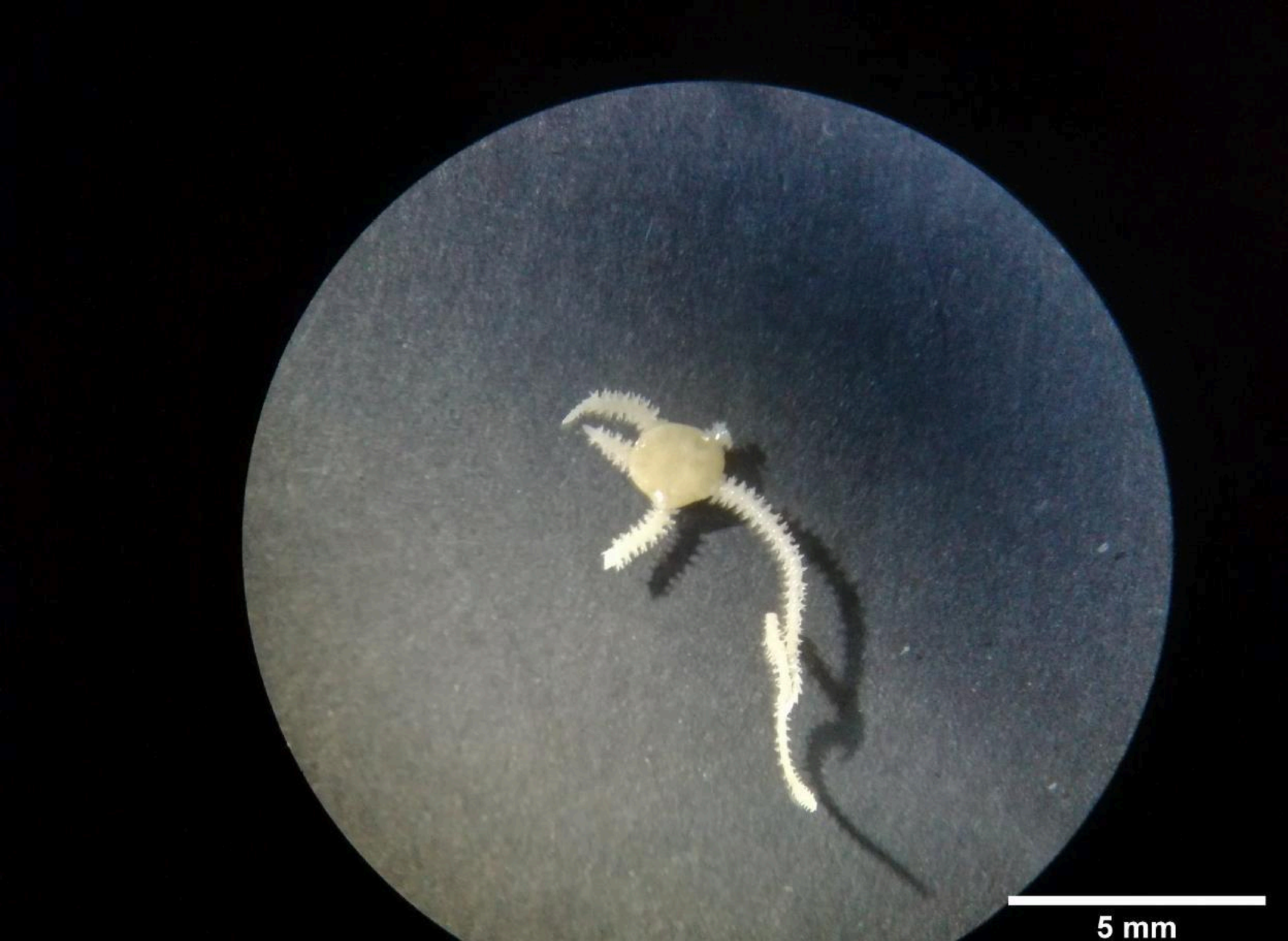
5 mm

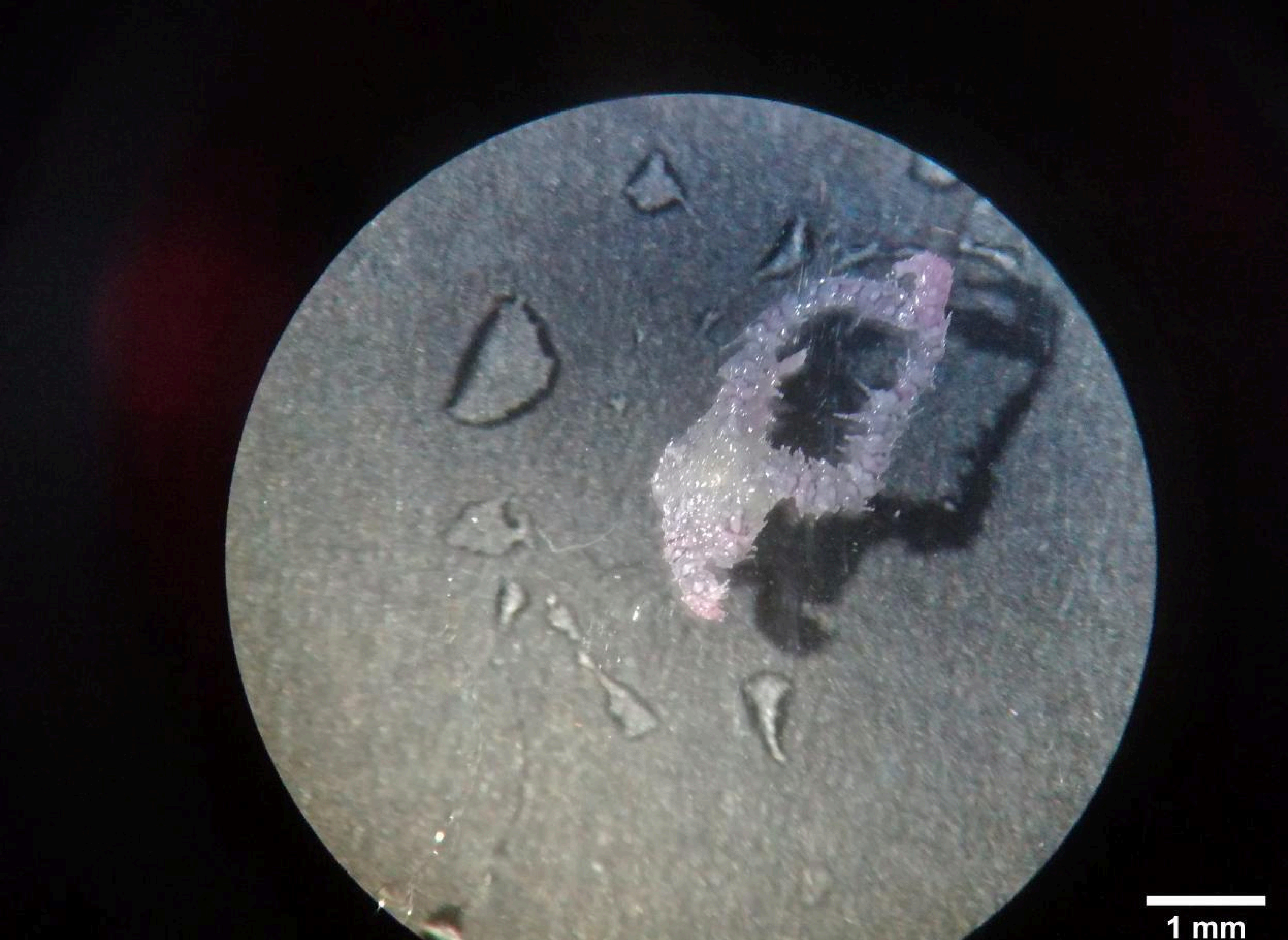
1 mm

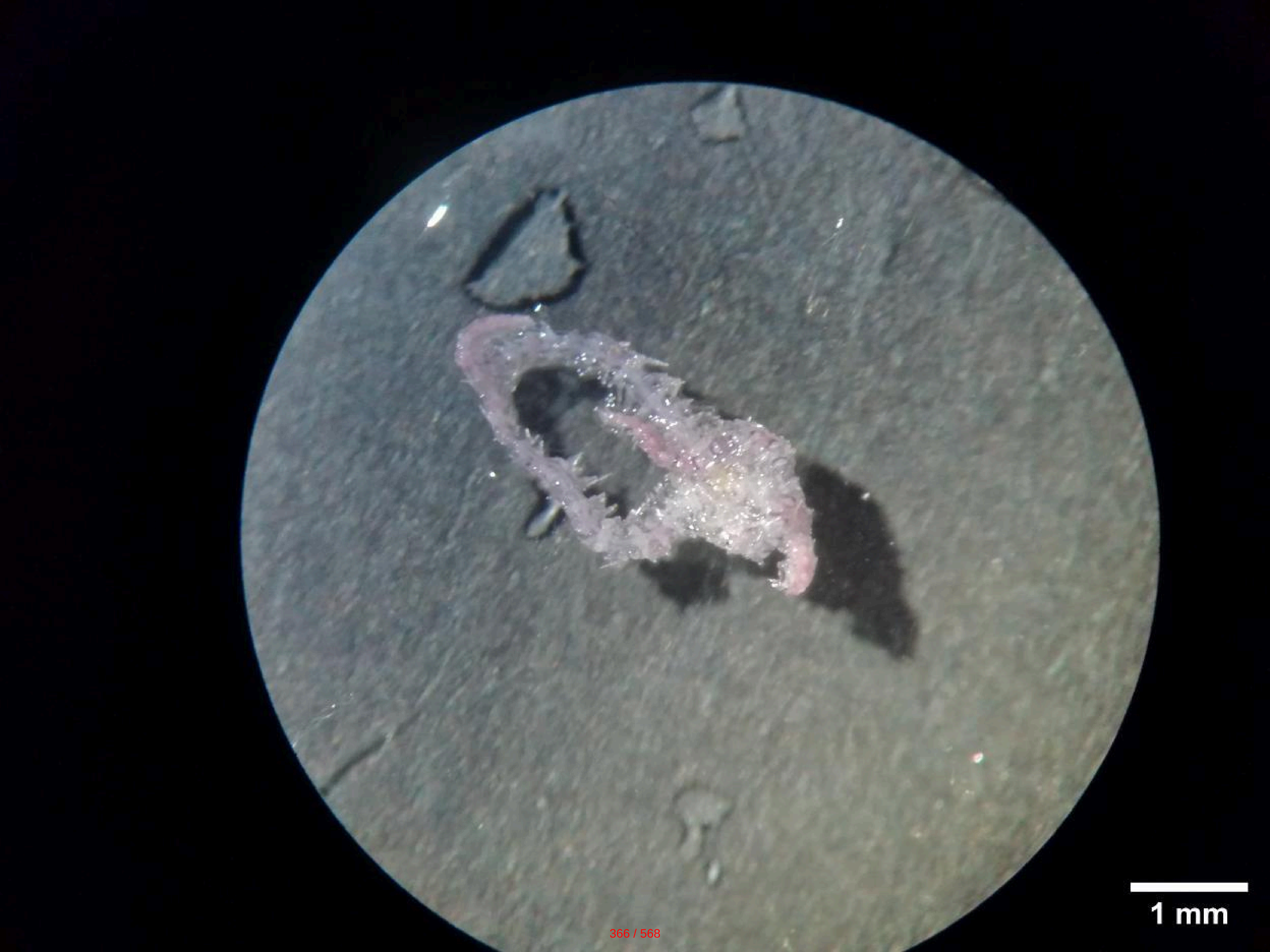
1 mm

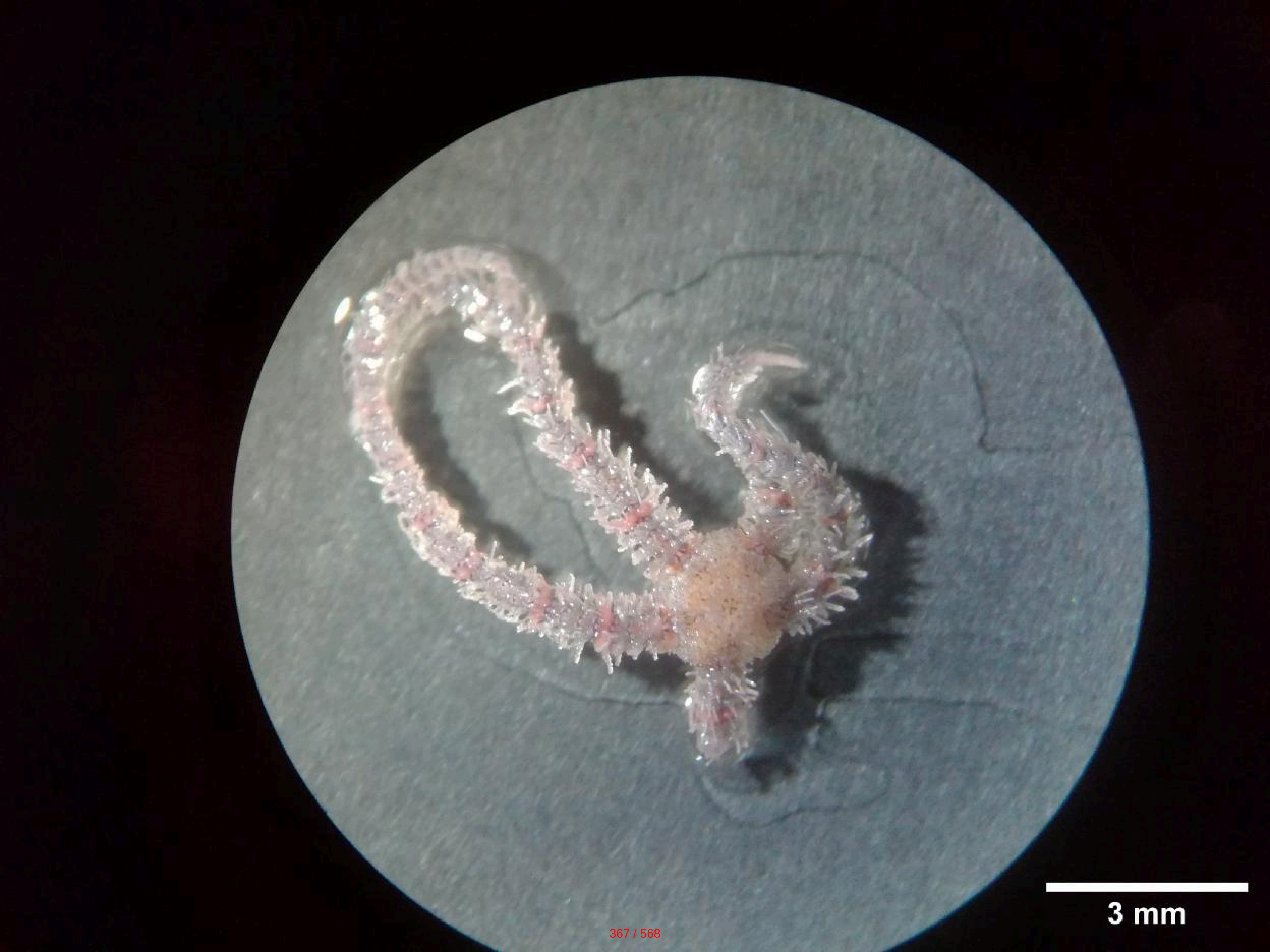
3 mm

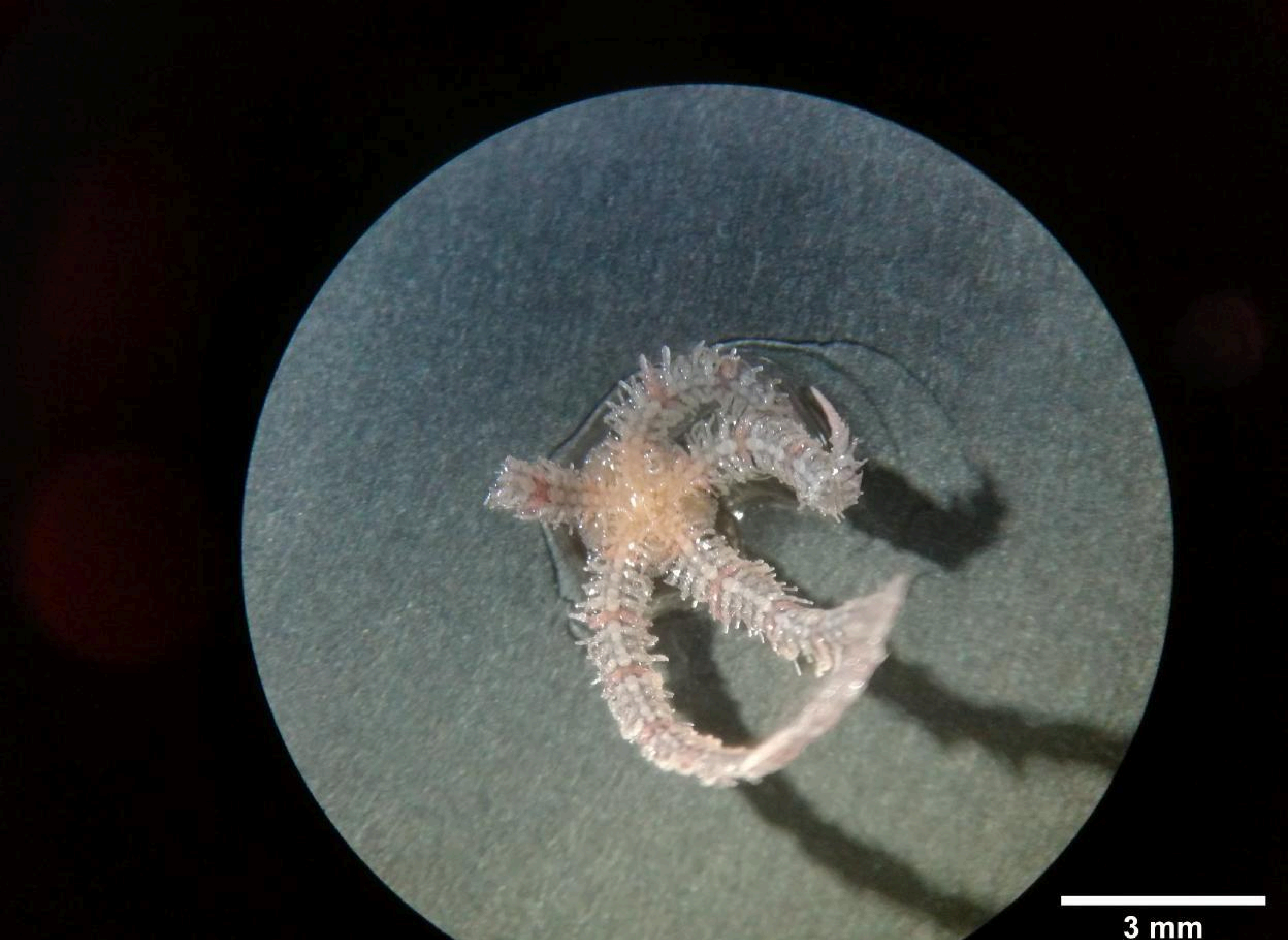
3 mm

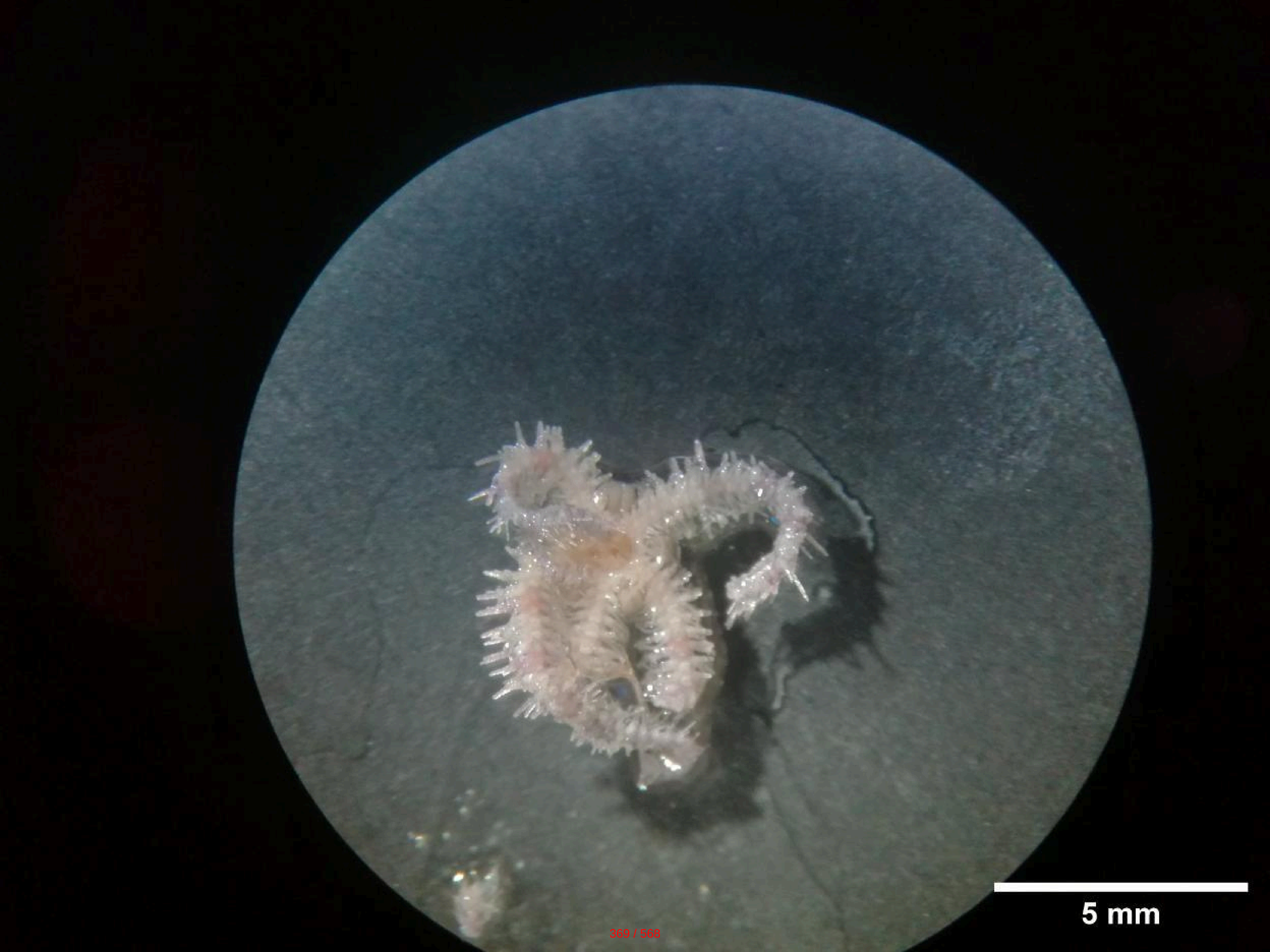
5 mm

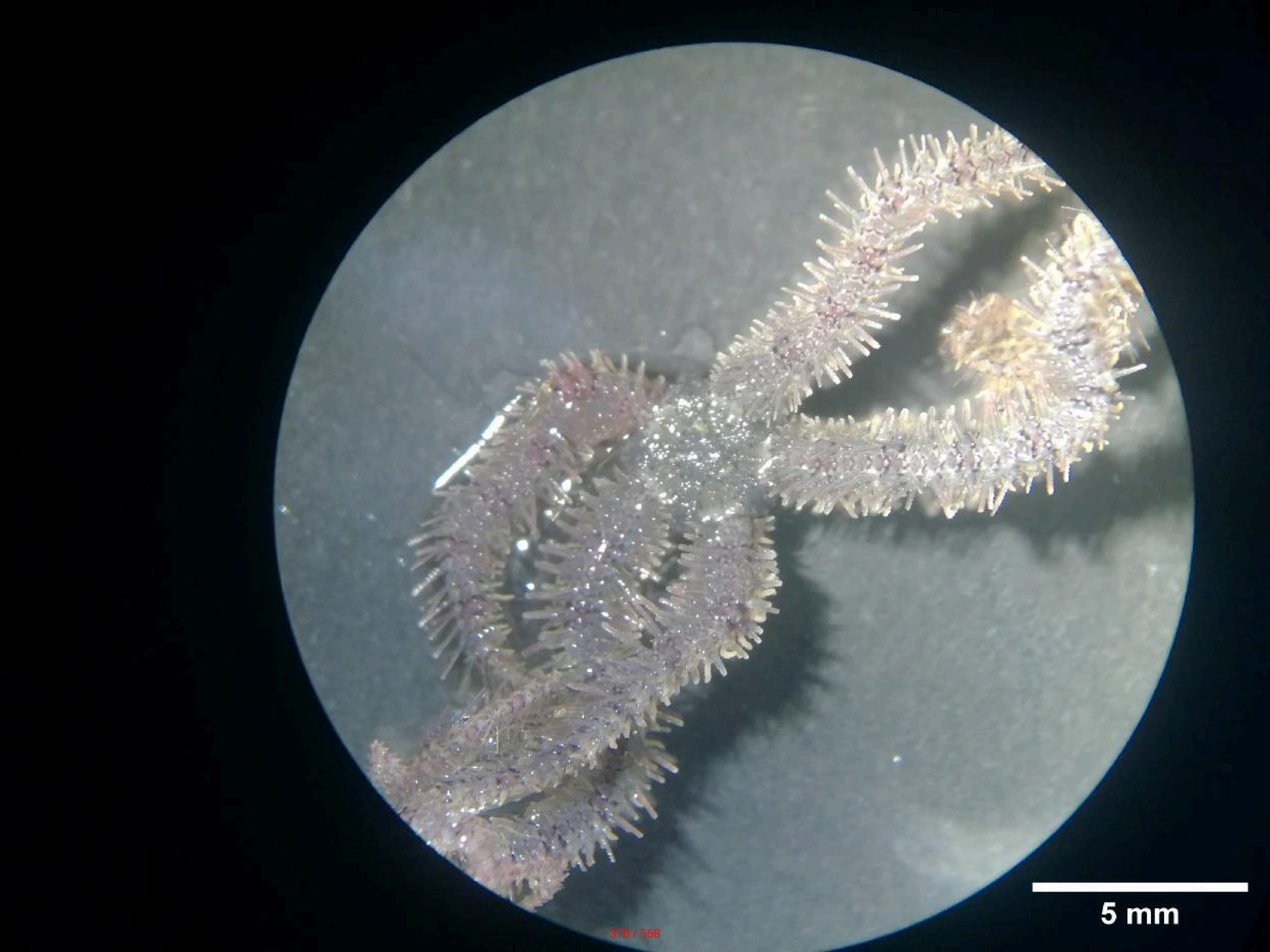
5 mm

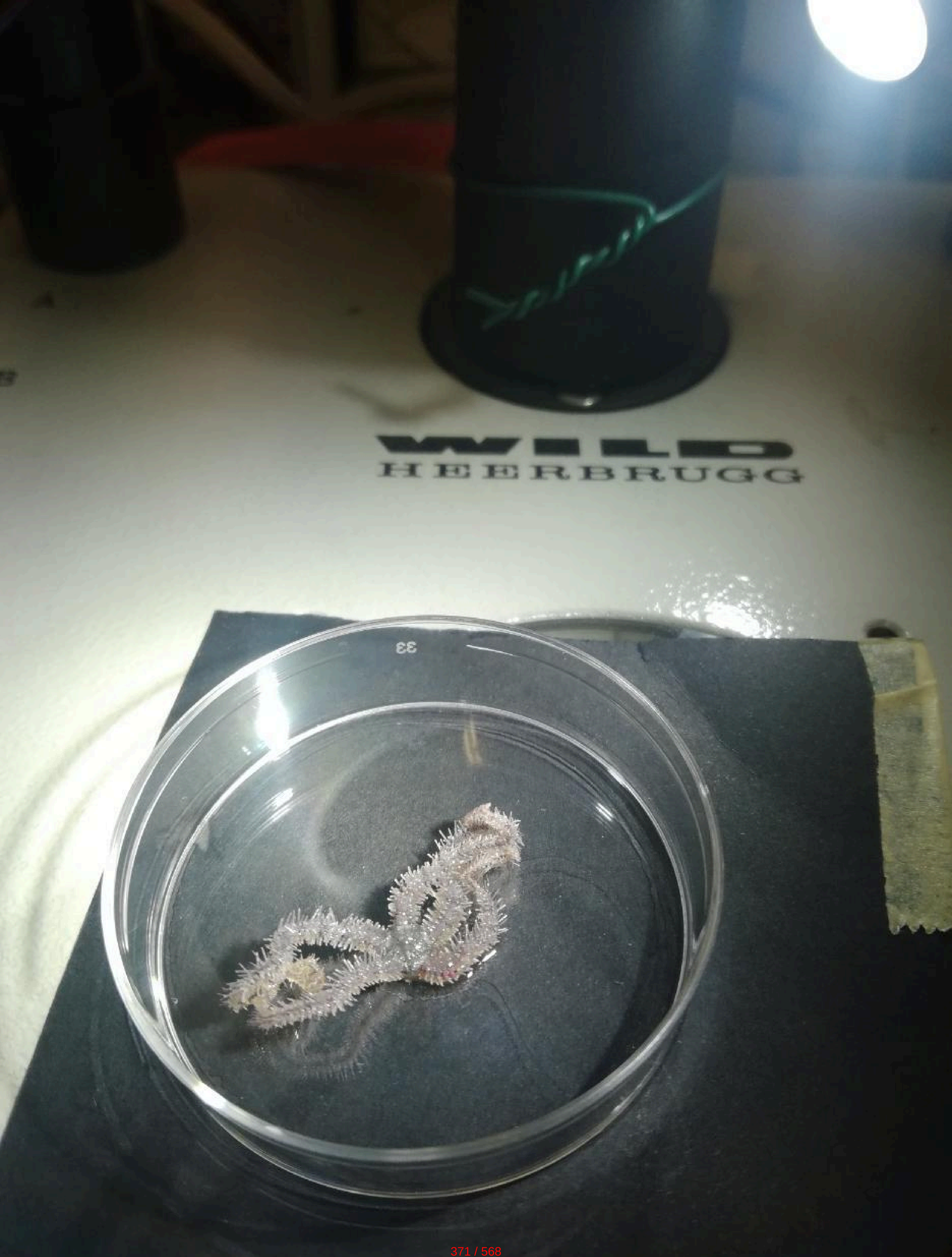
WILD
HEERBRUGG

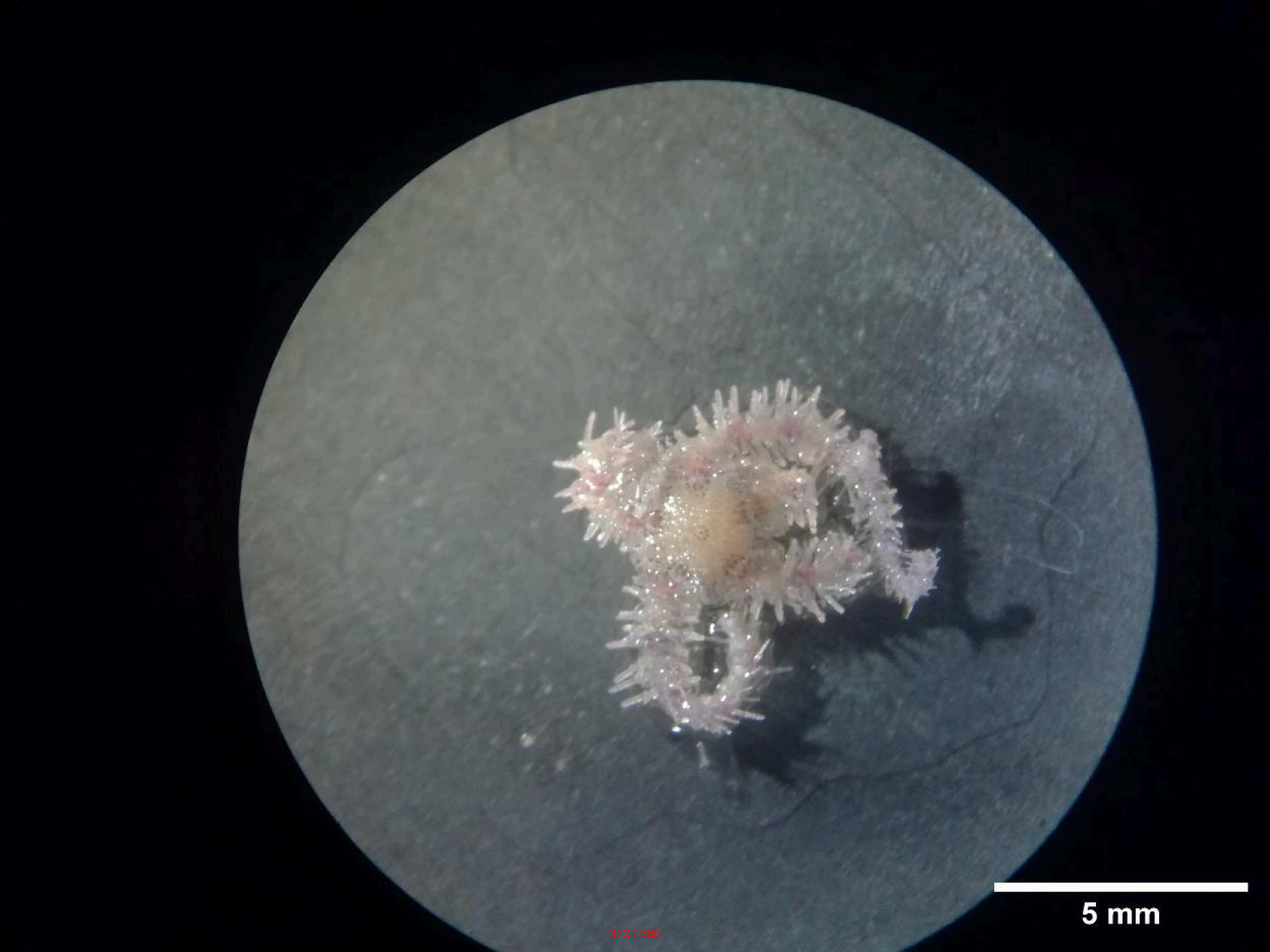
5 mm

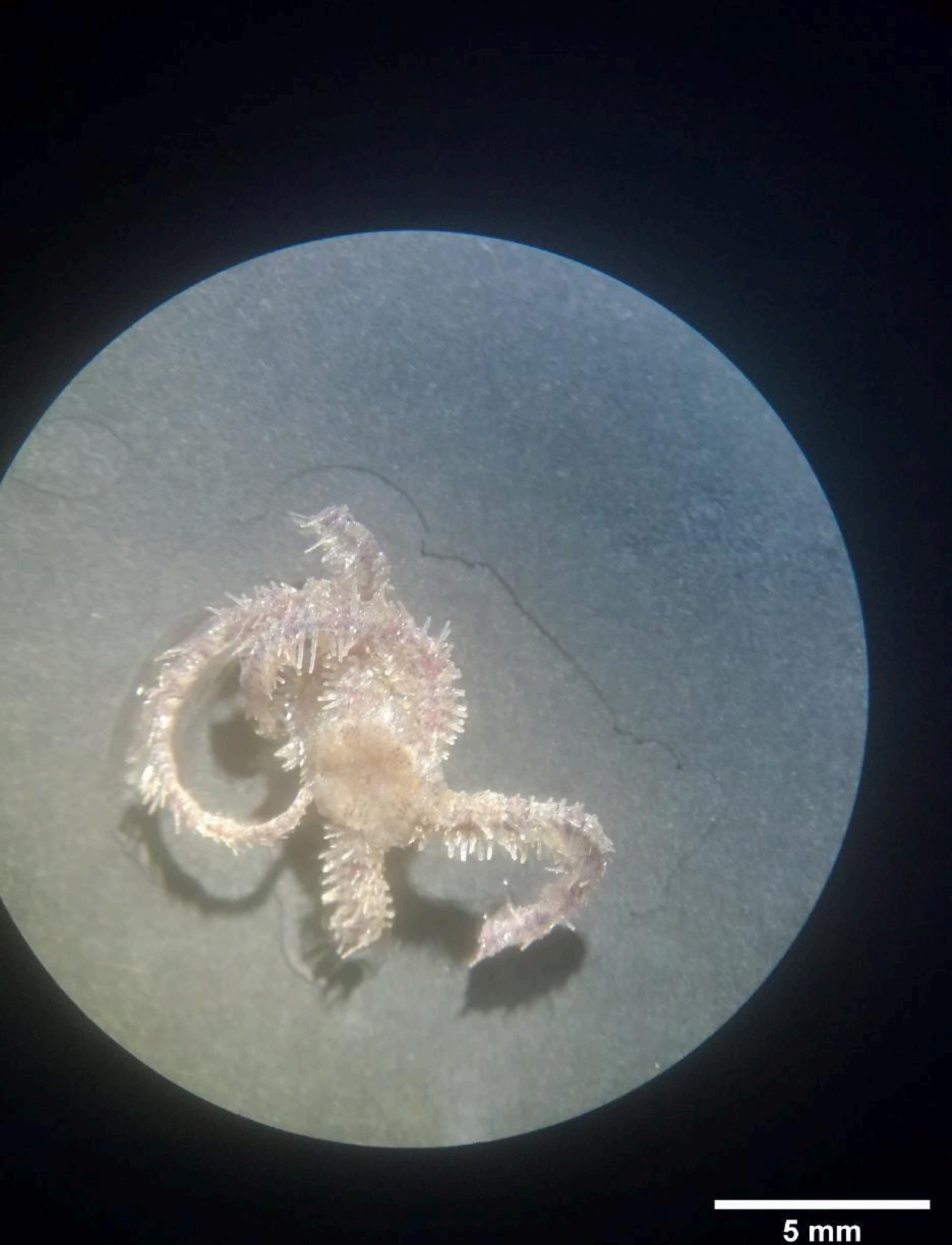
5 mm

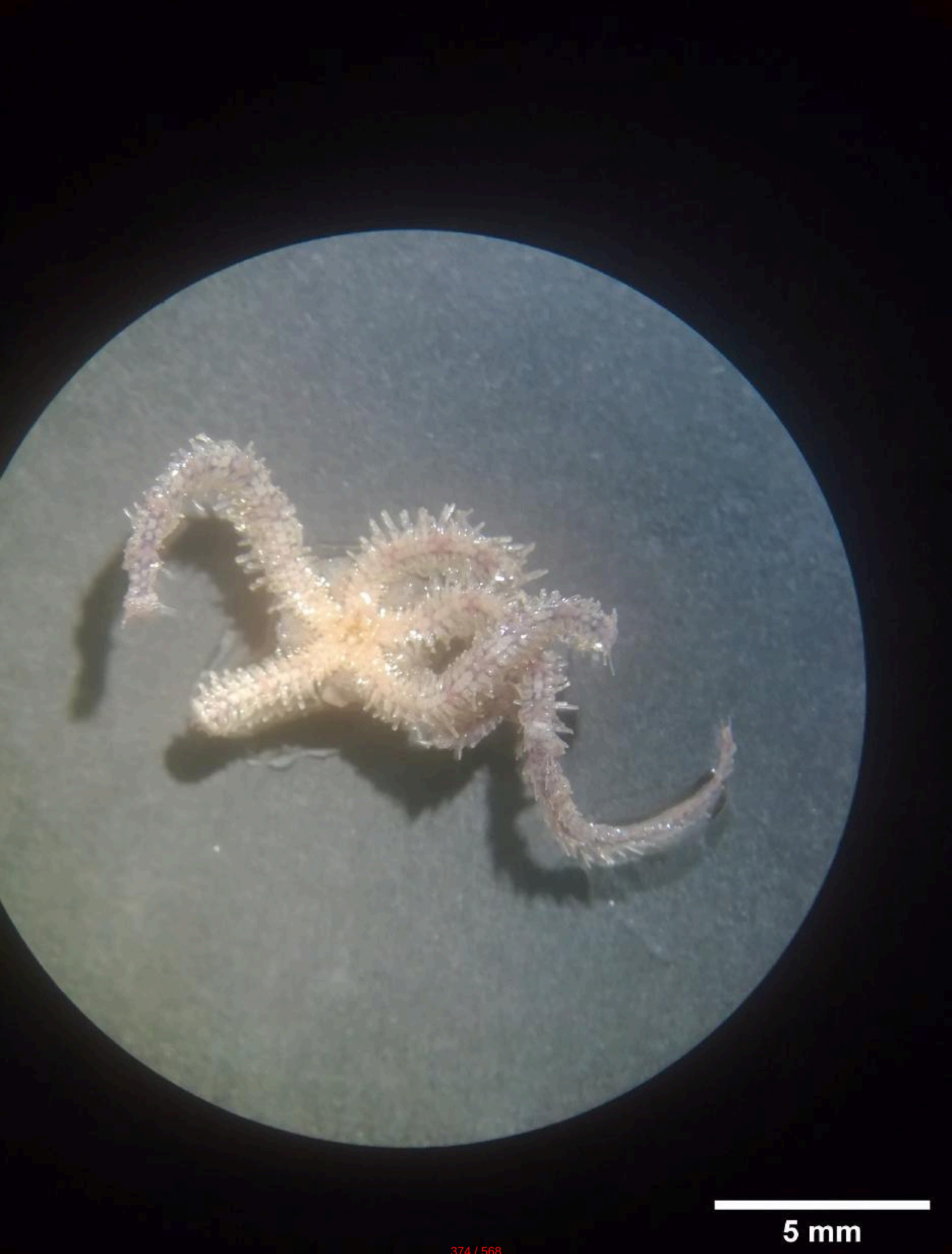
5 mm

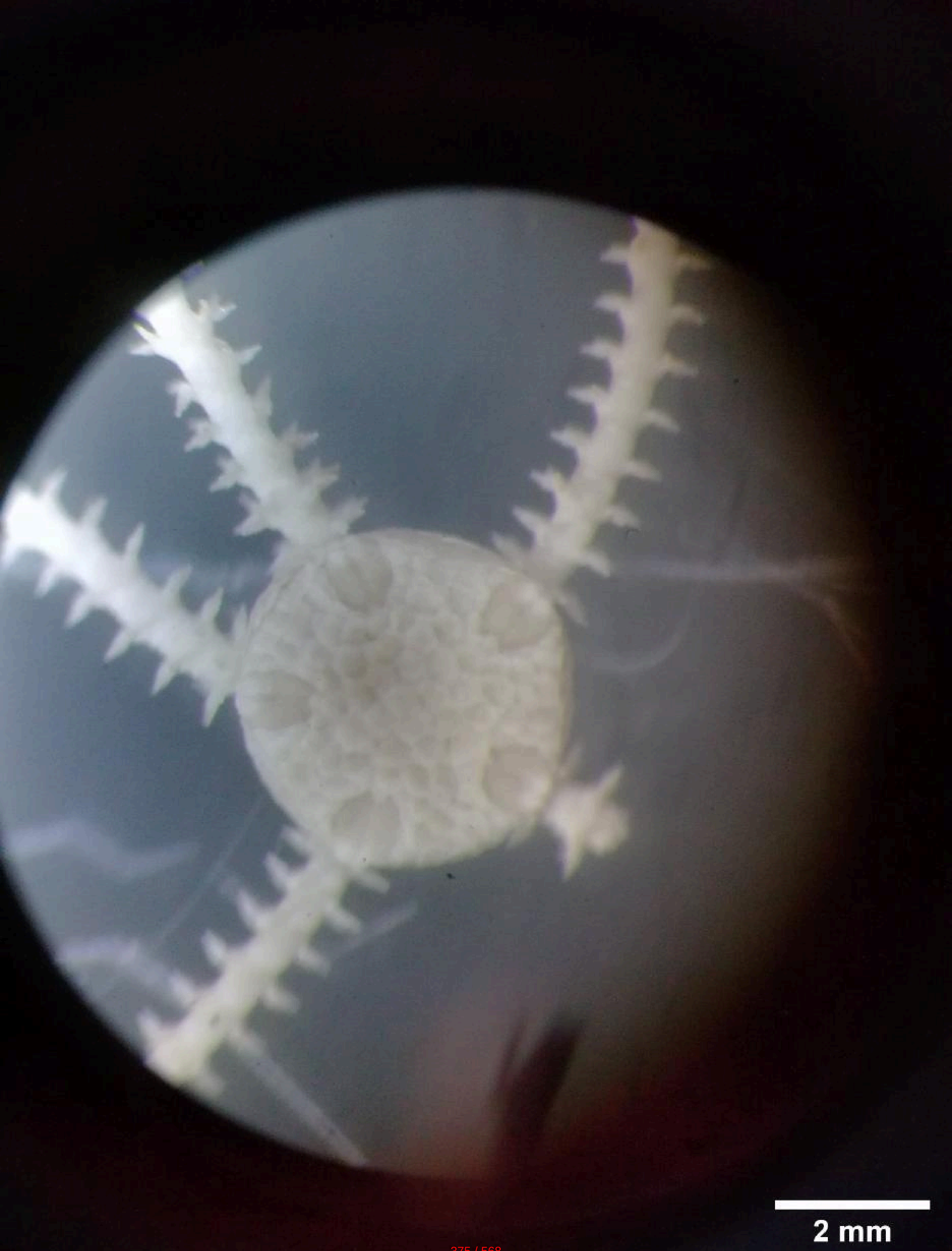
2 mm

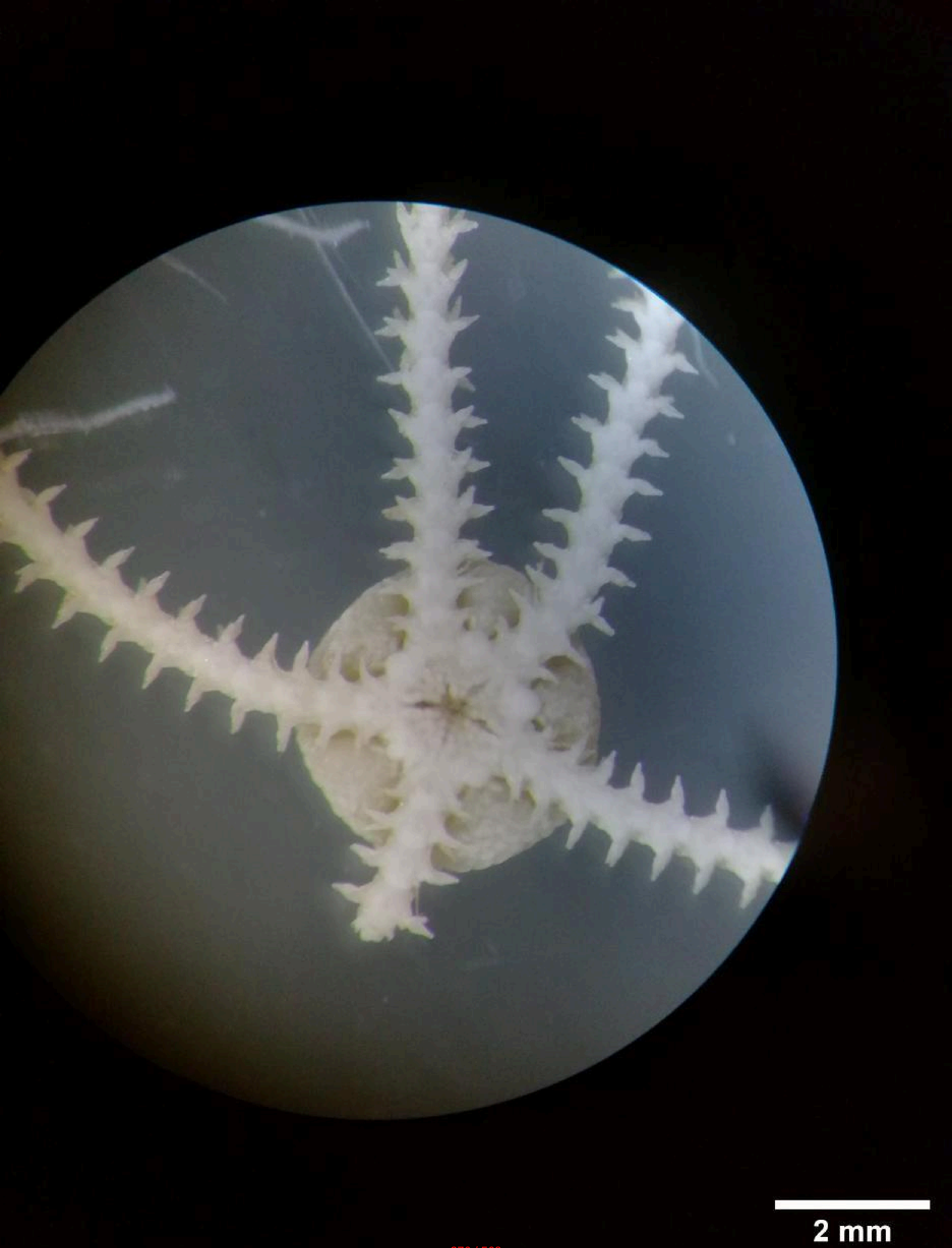
2 mm

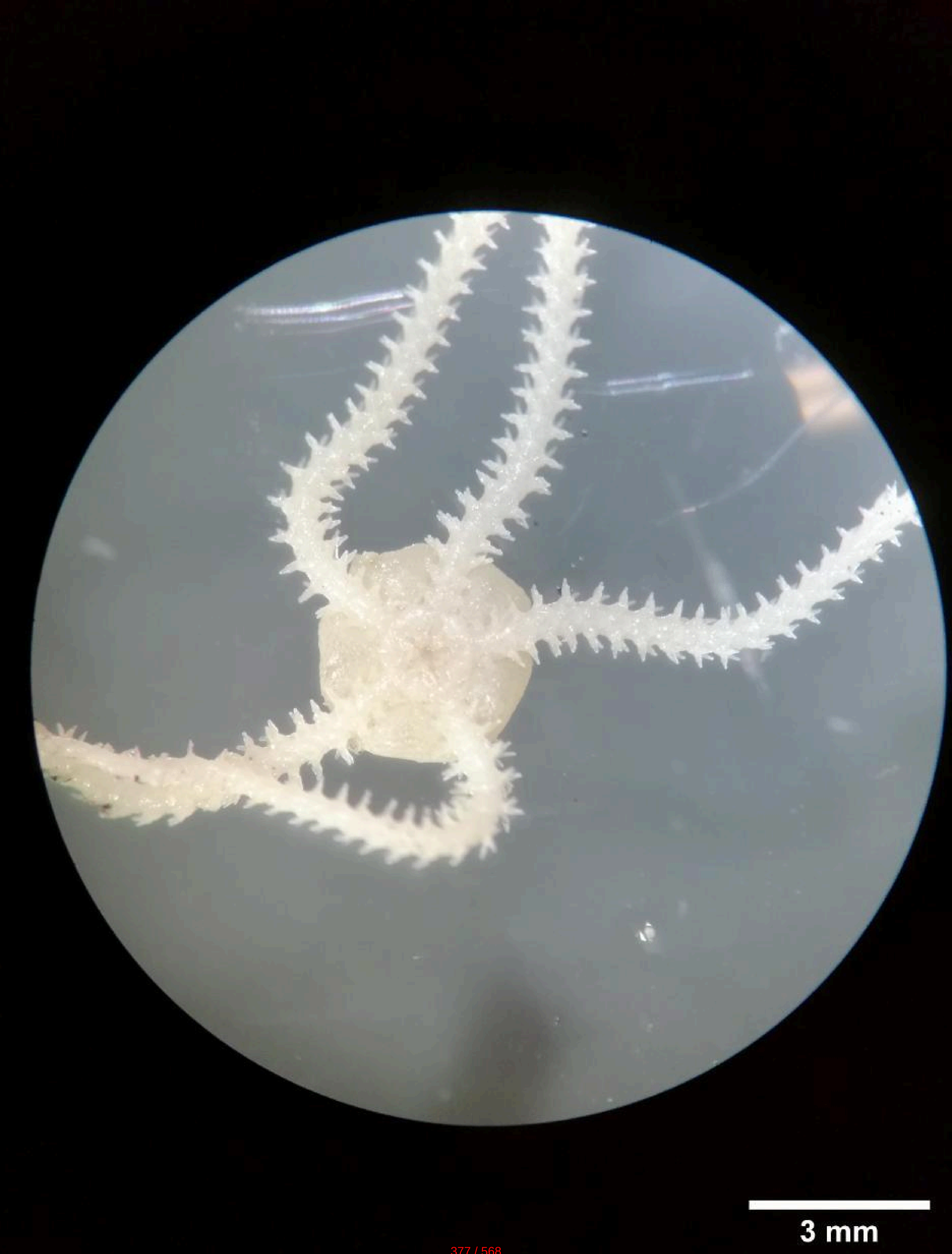
3 mm

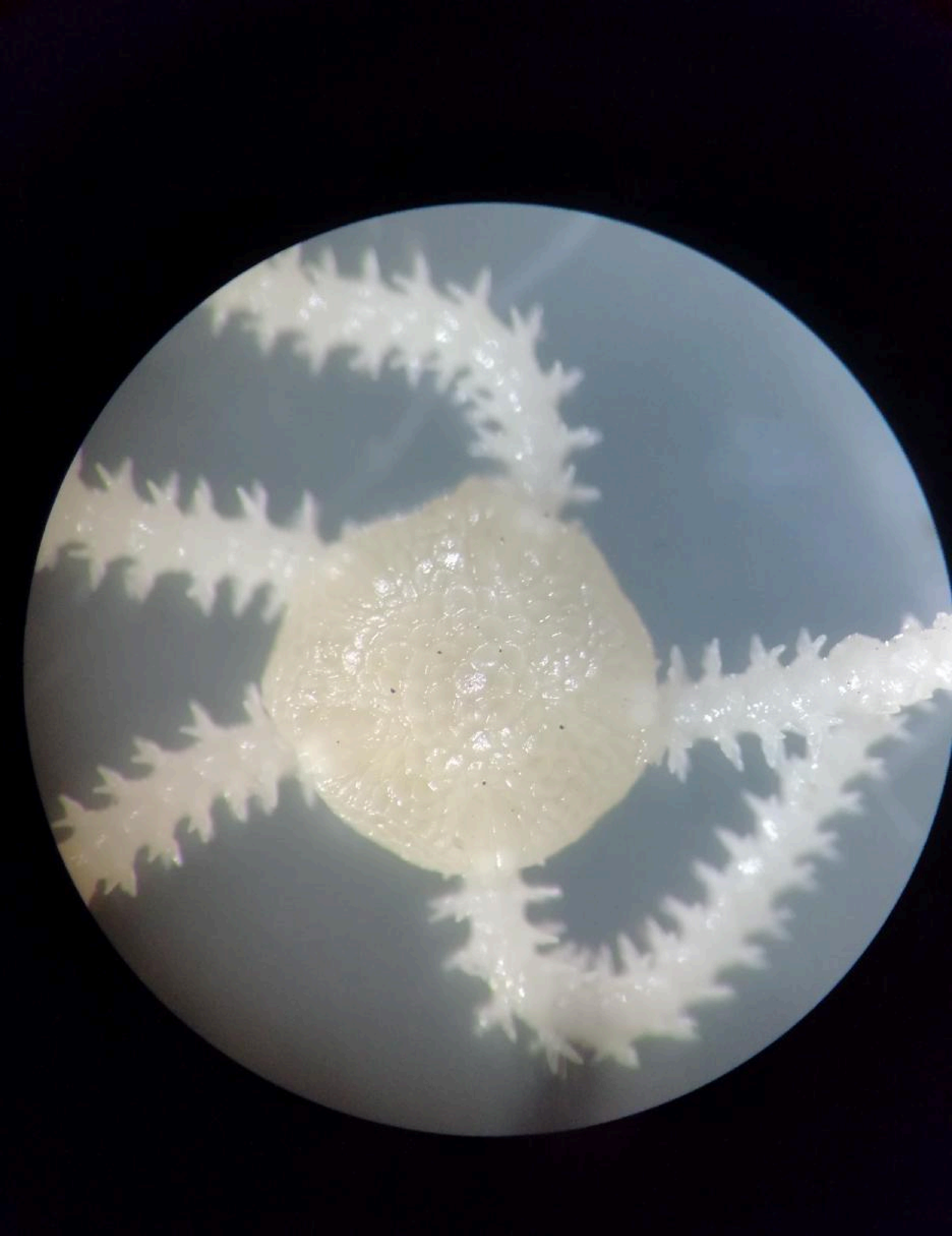

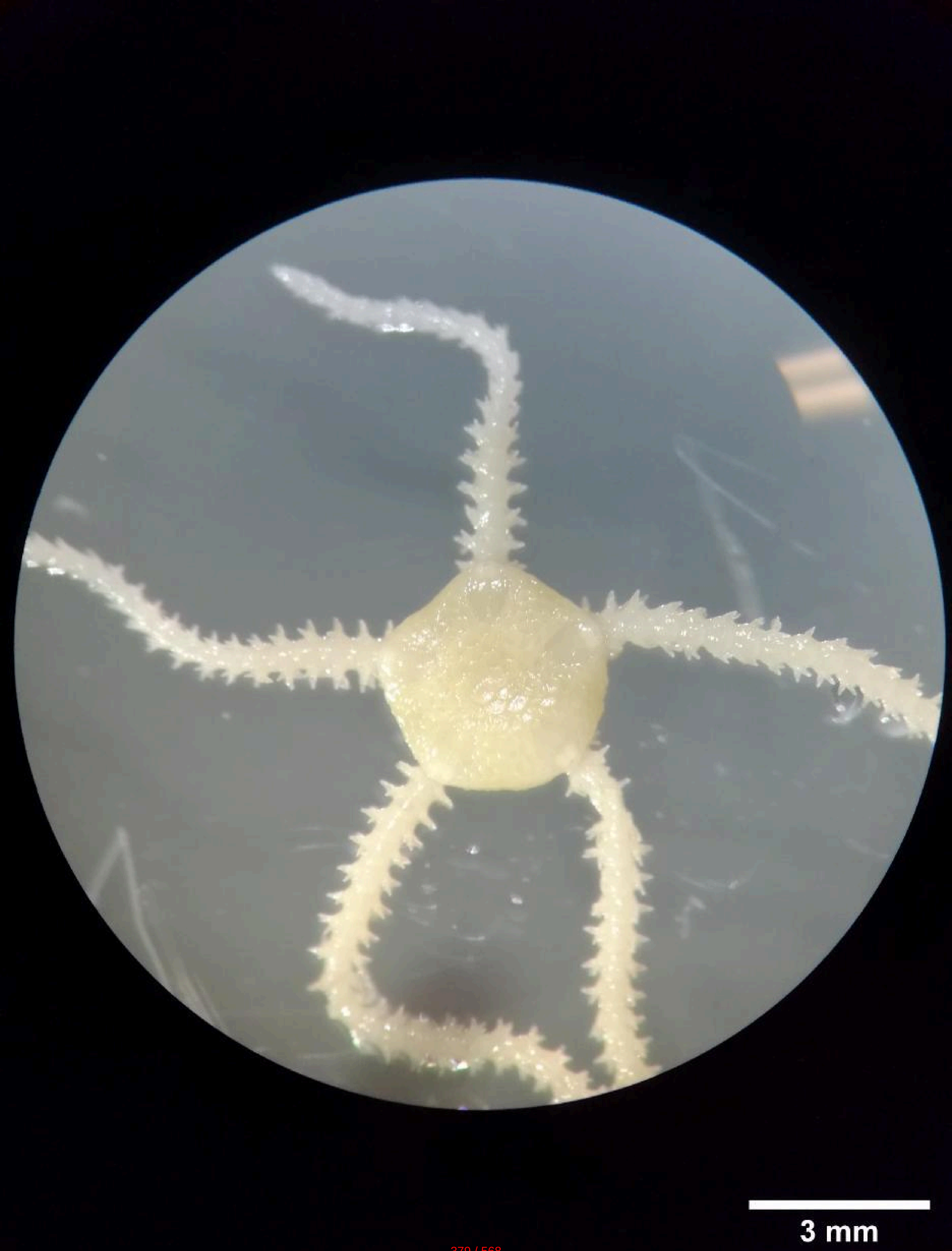
3 mm

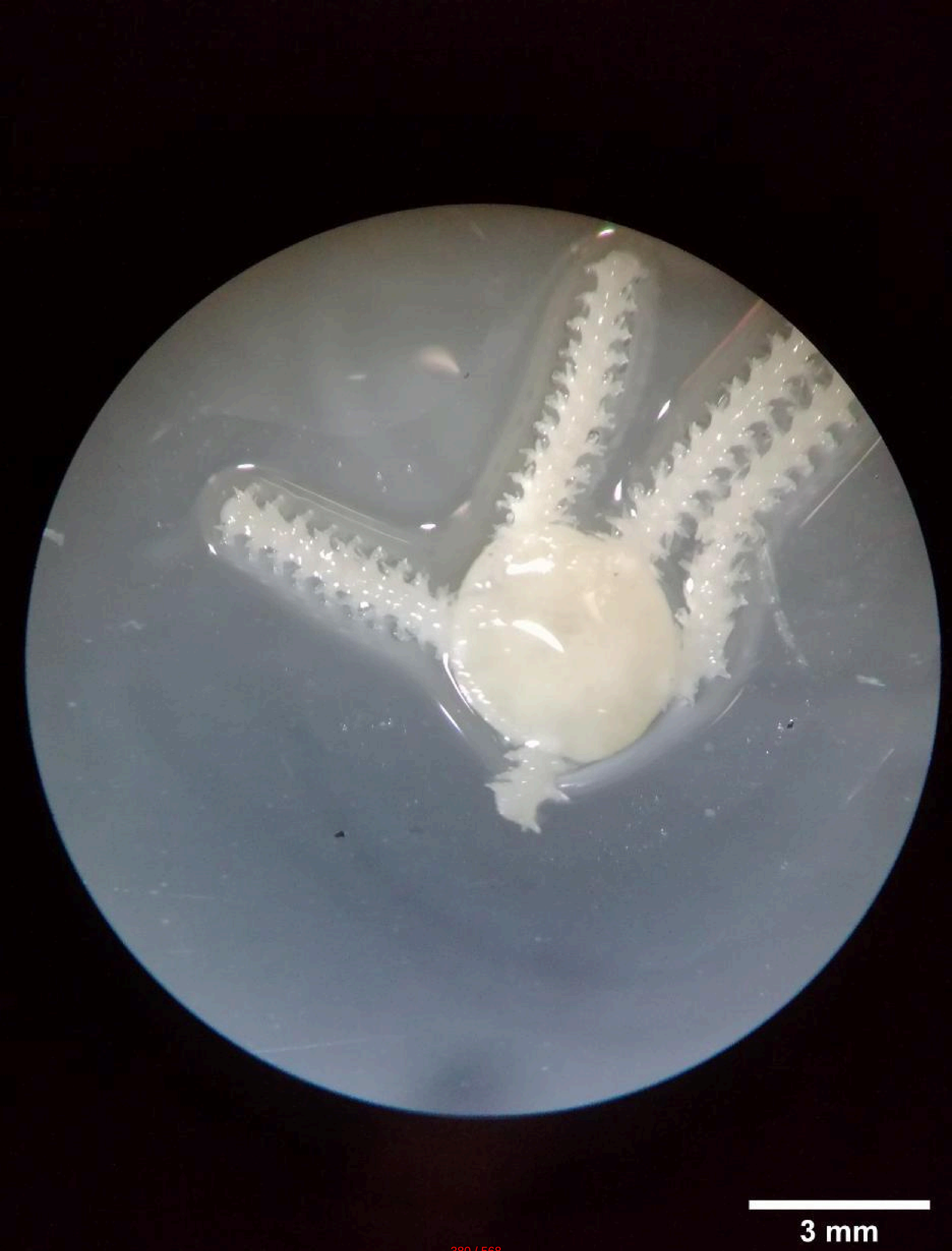
3 mm

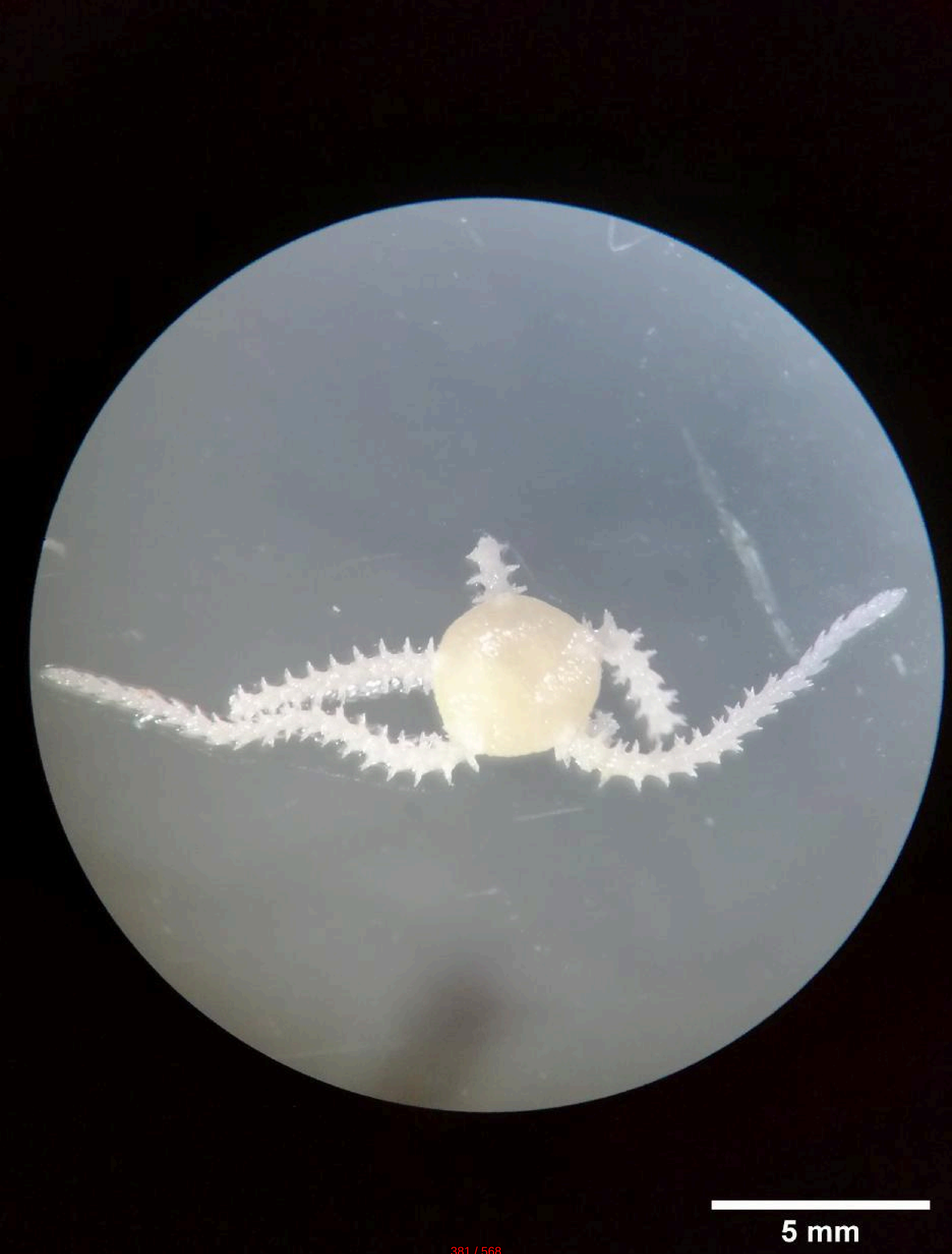
5 mm

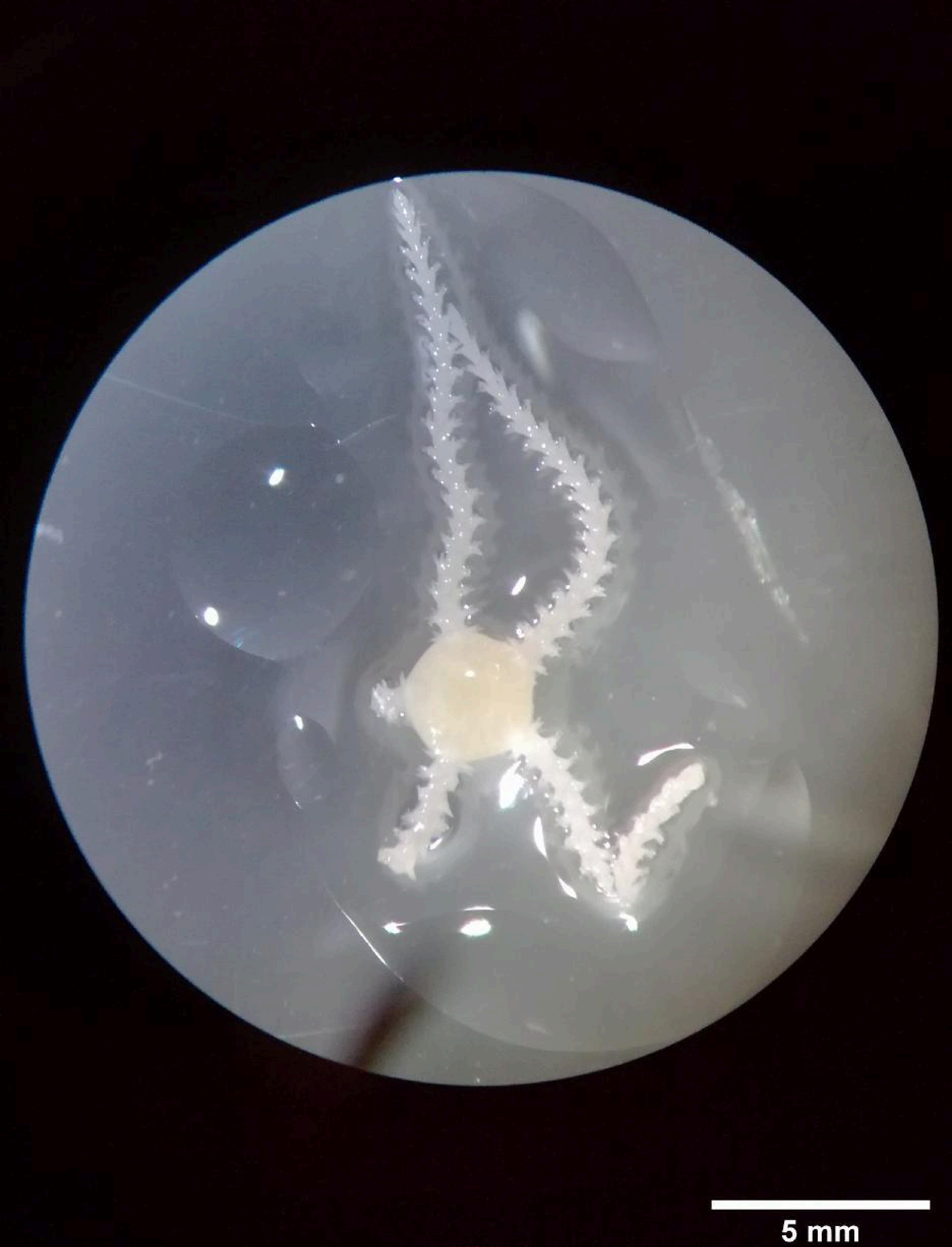
5 mm

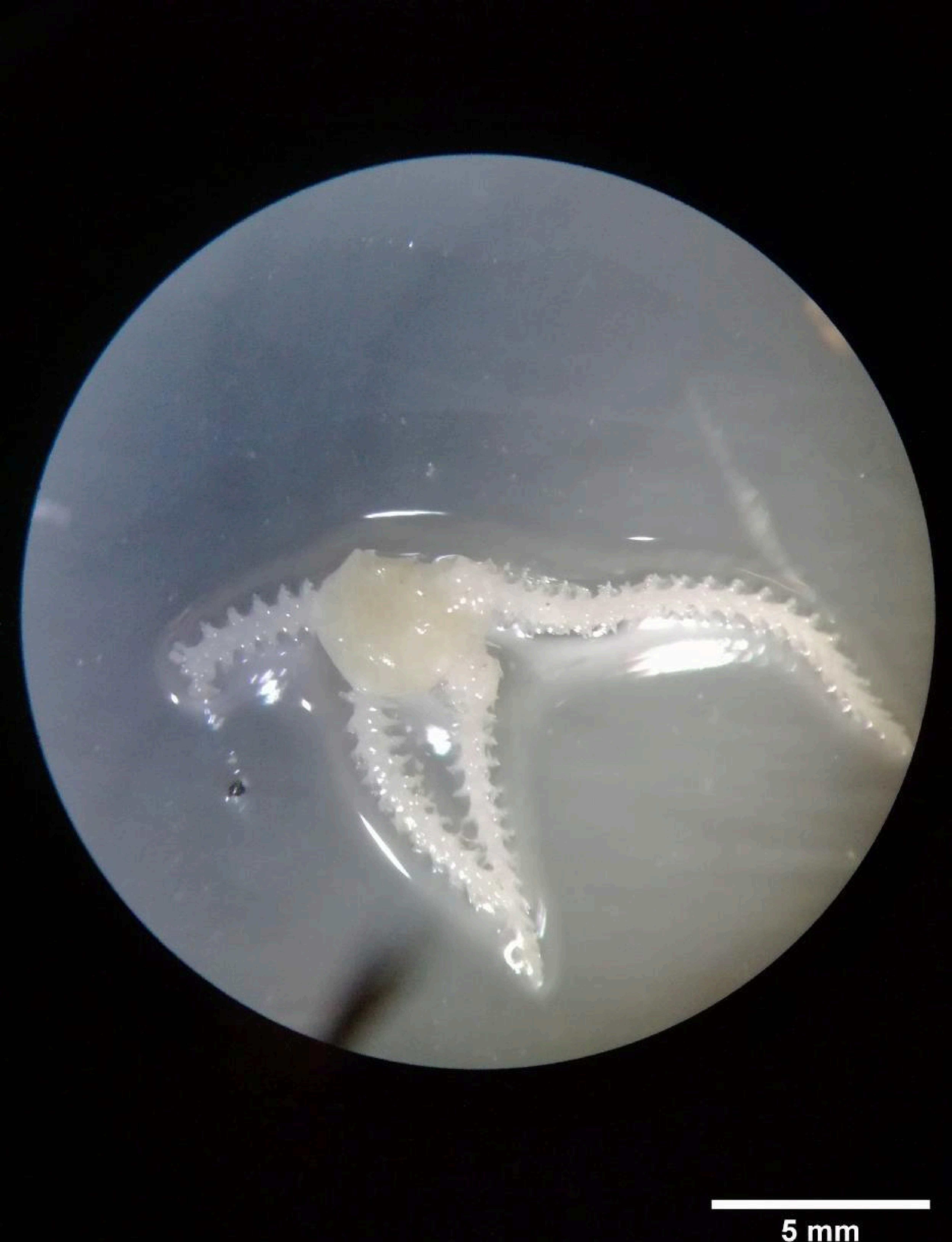
5 mm

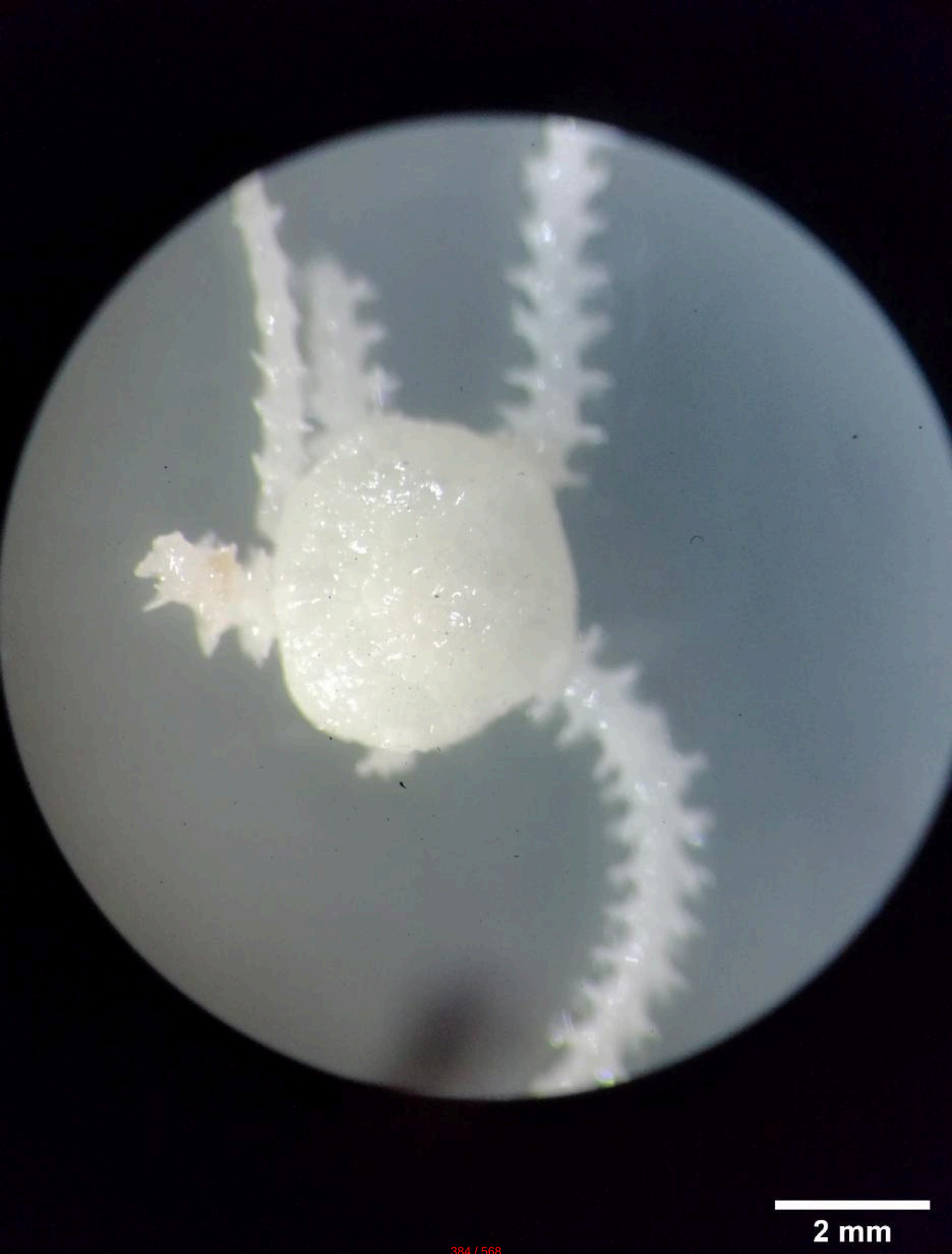
2 mm

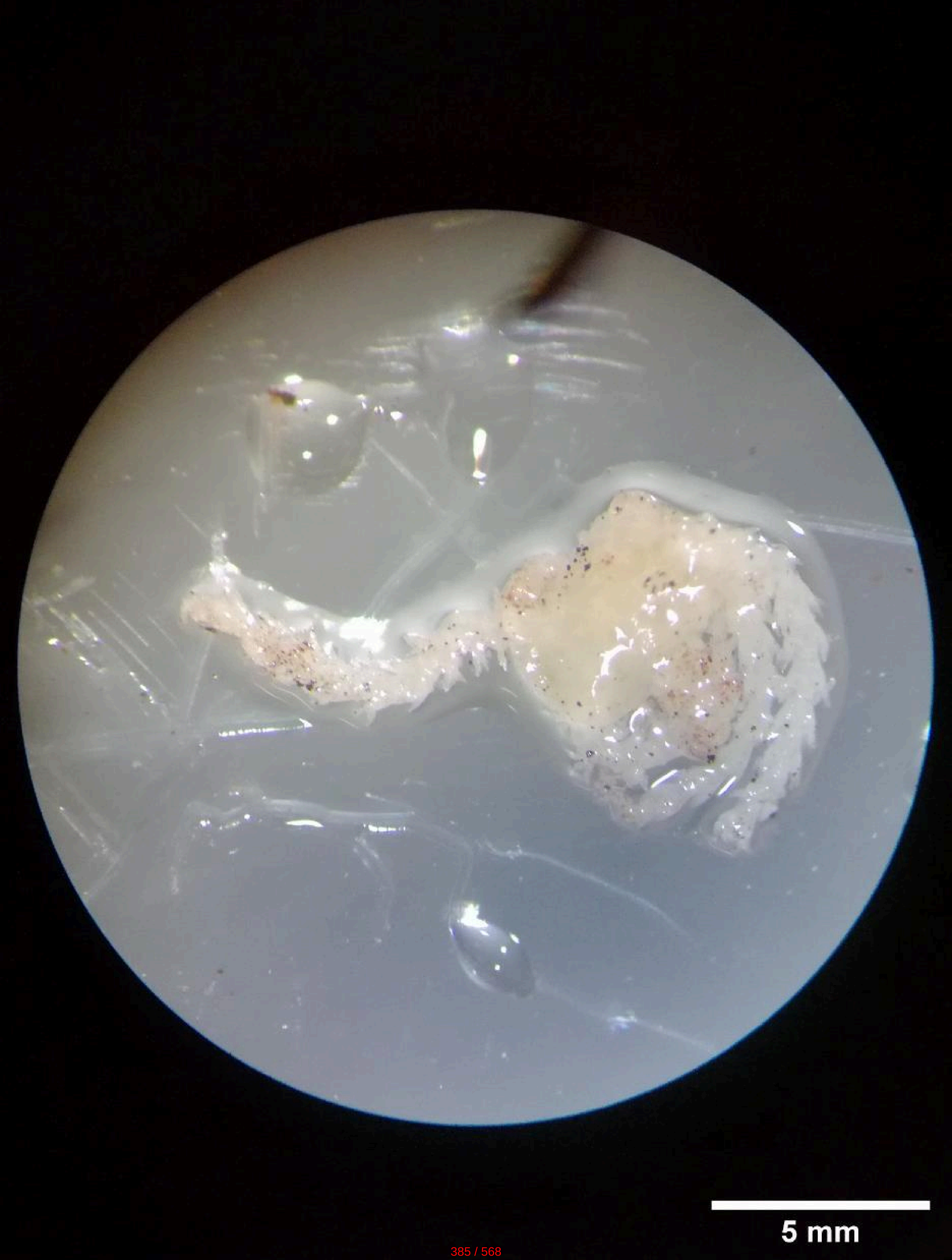
5 mm

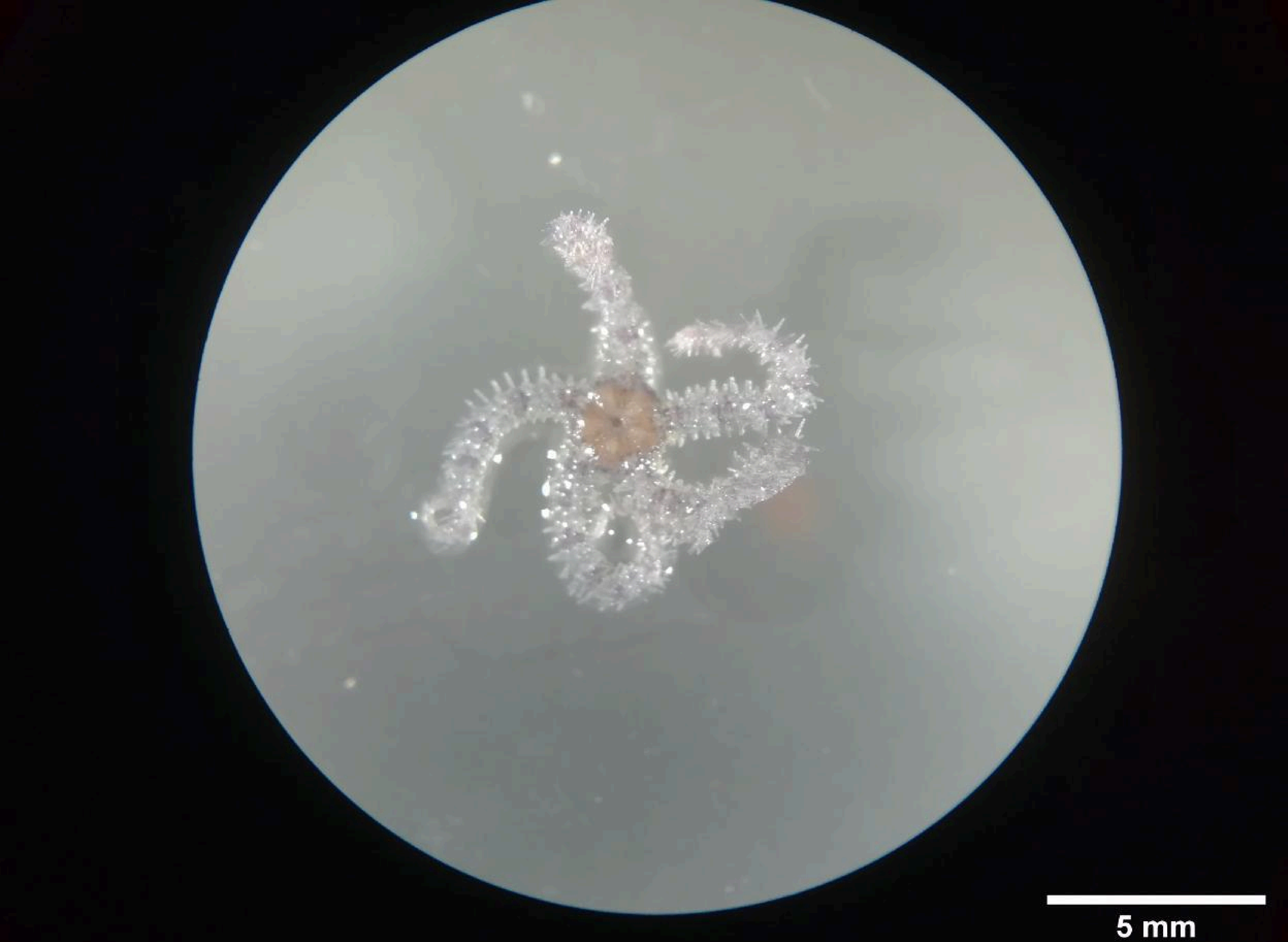
5 mm

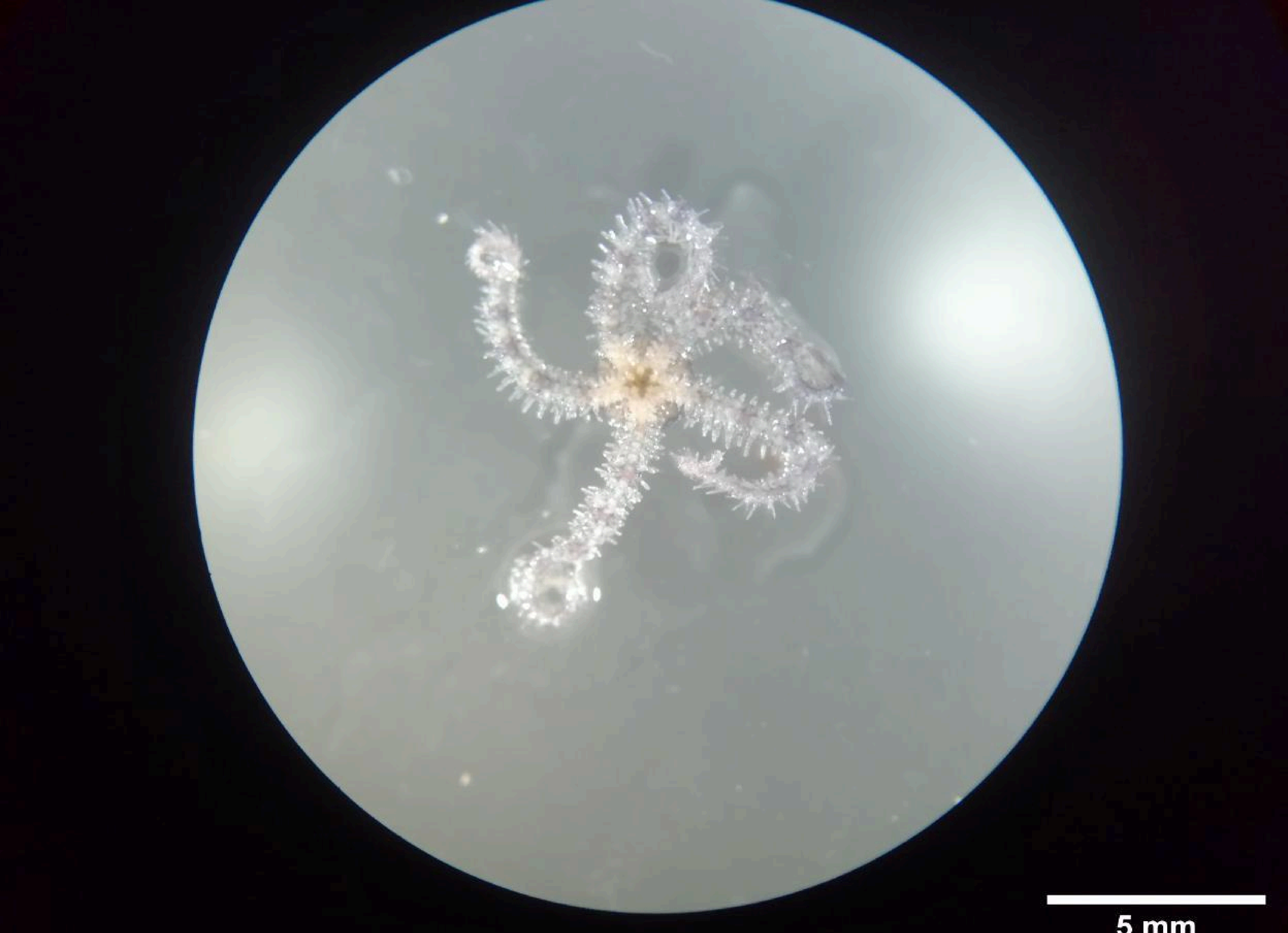
5 mm

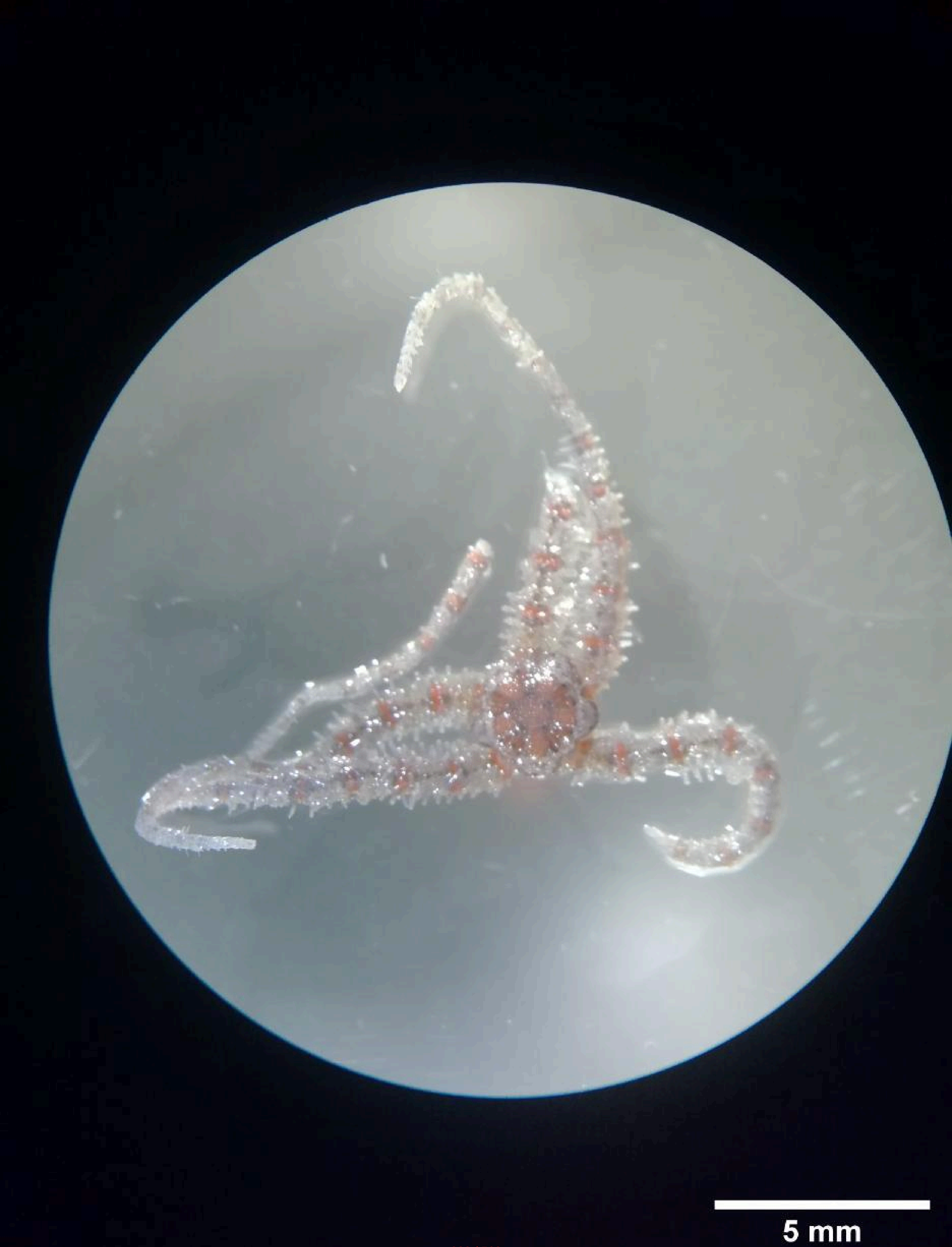
5 mm

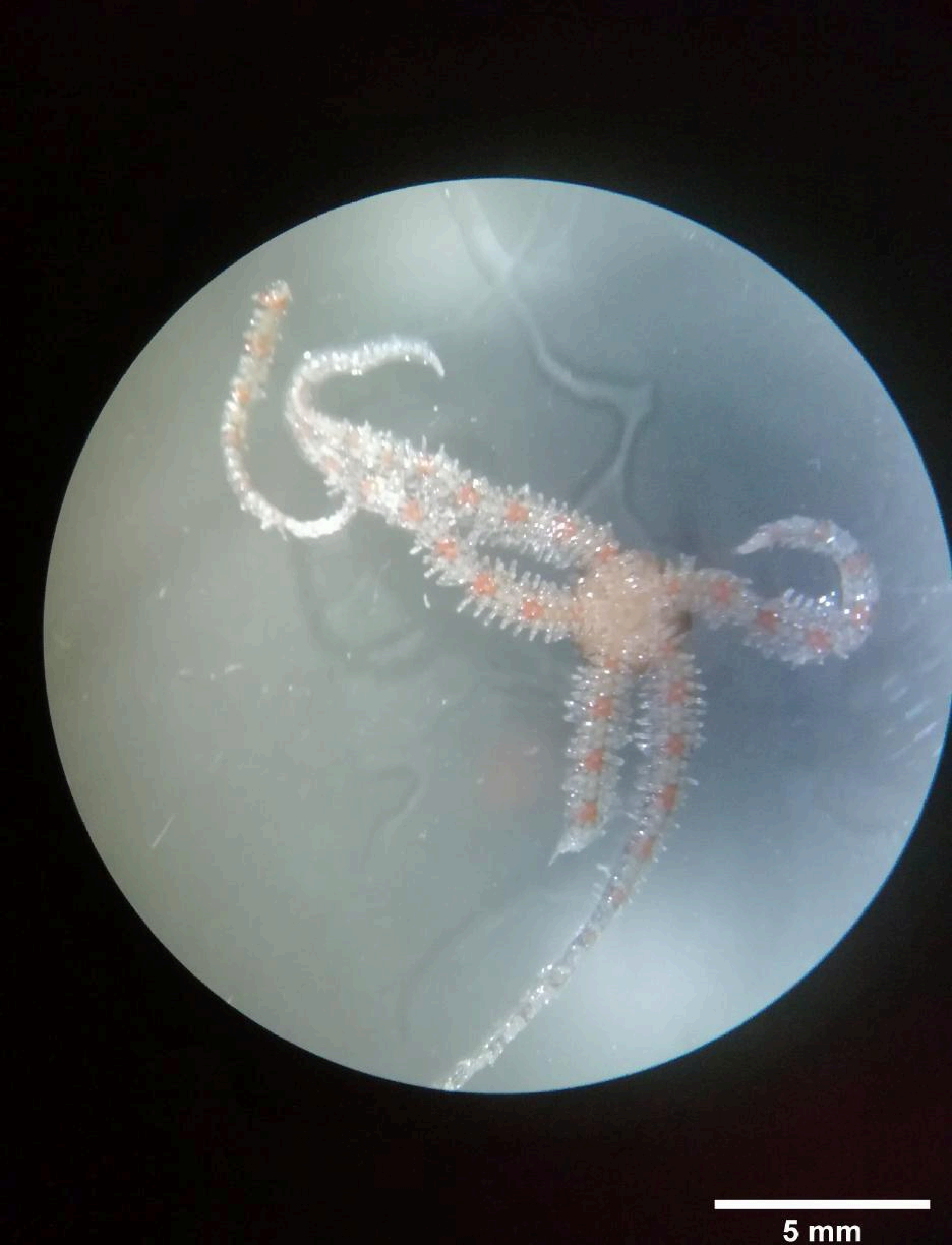
5 mm

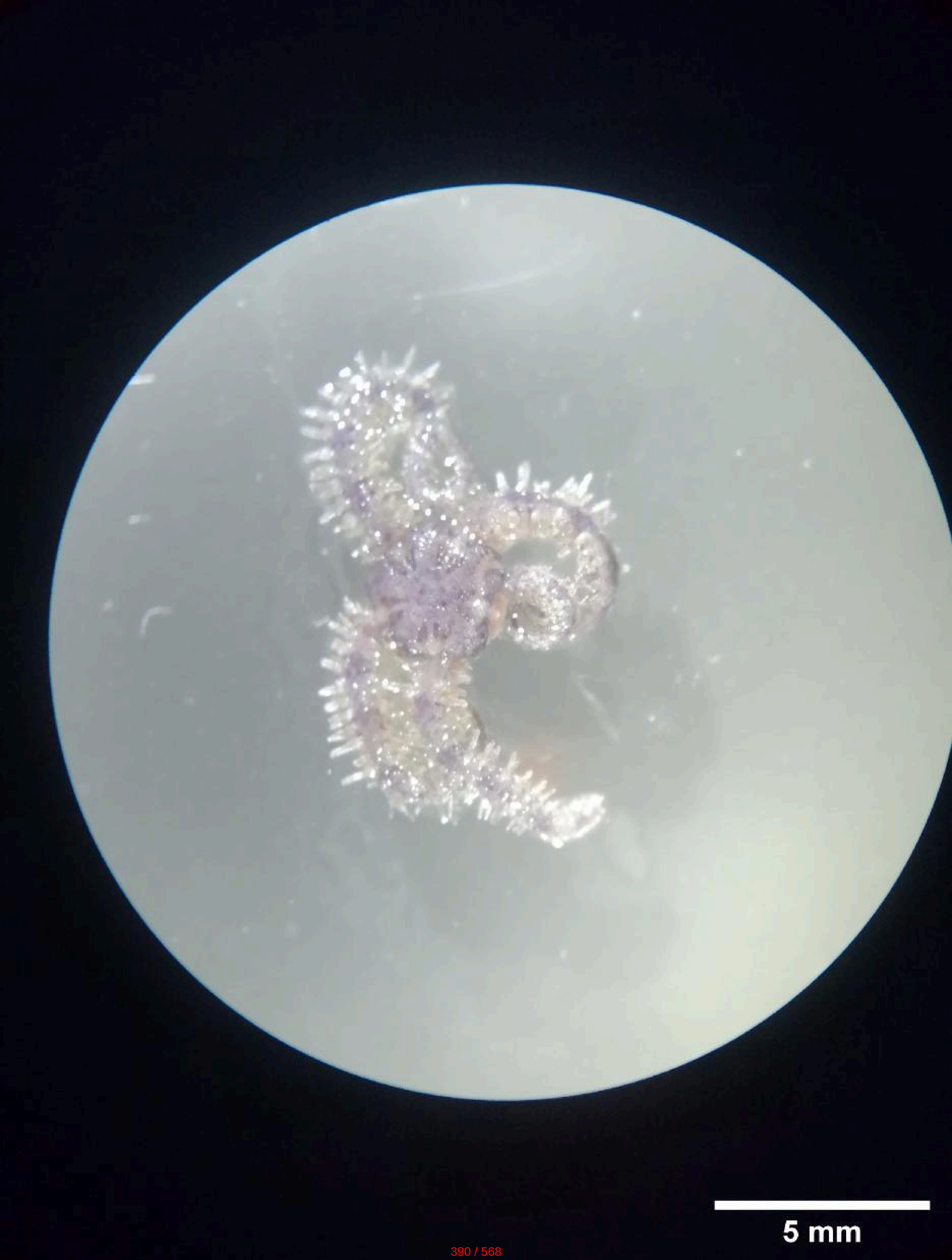
5 mm

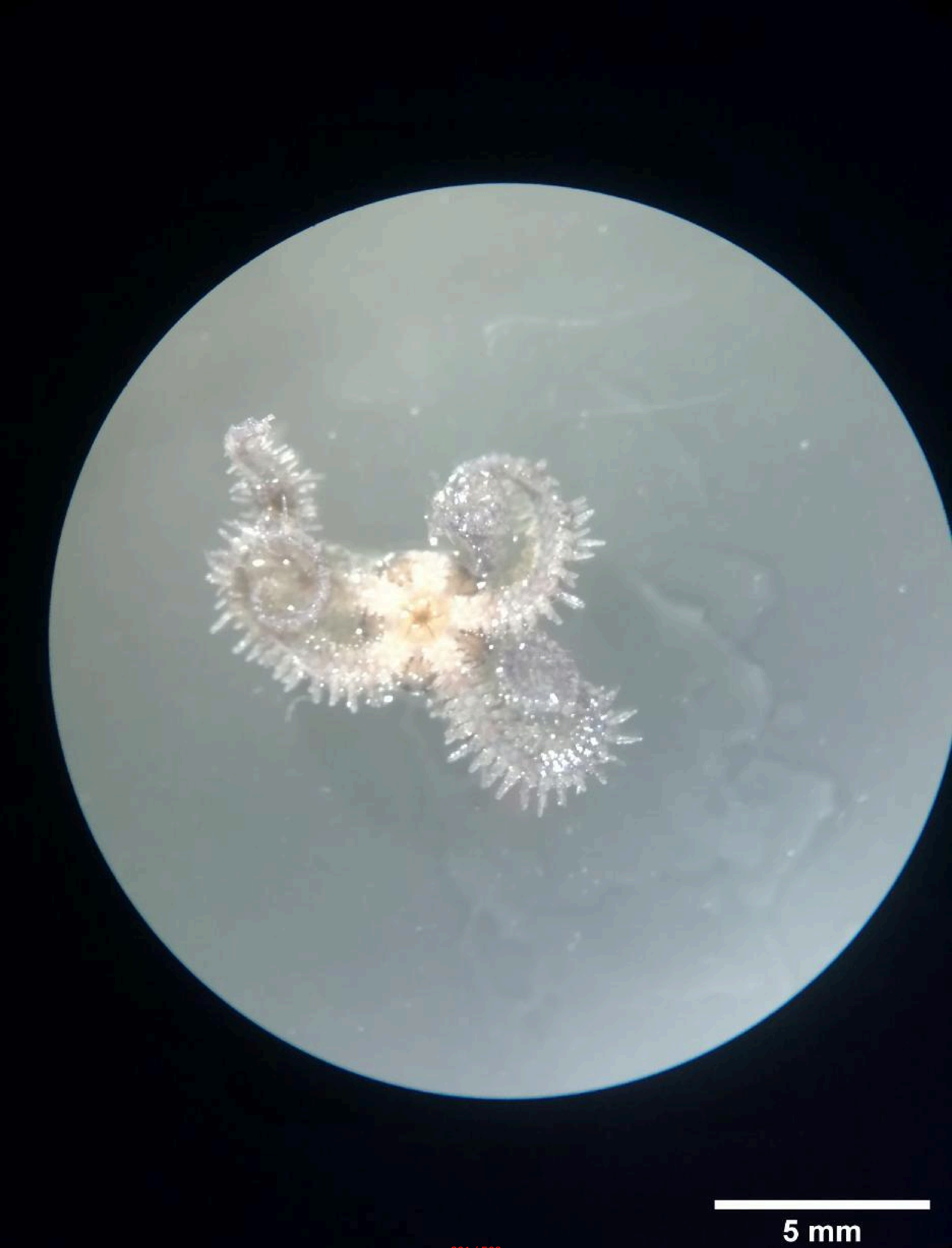
5 mm

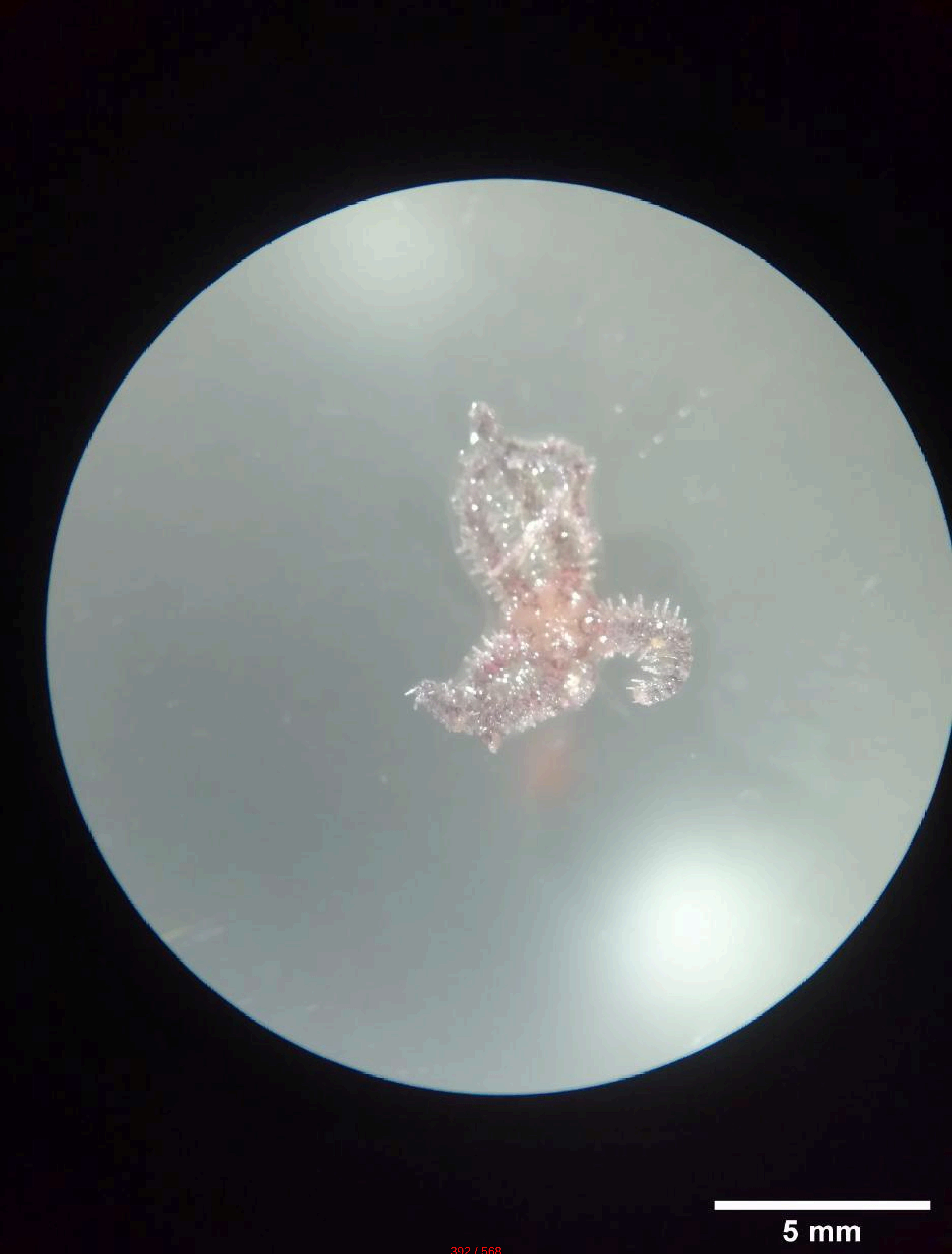
5 mm

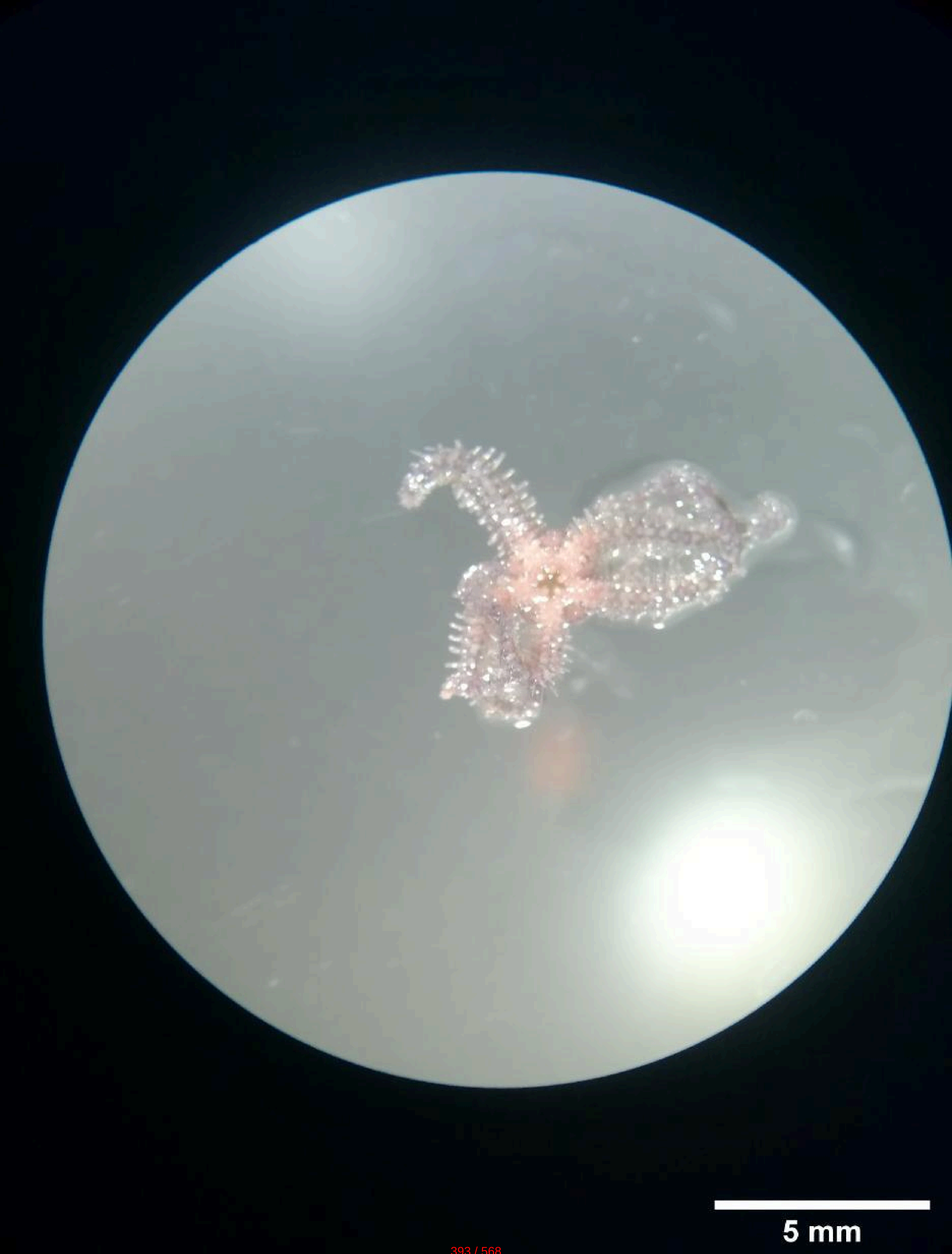
5 mm

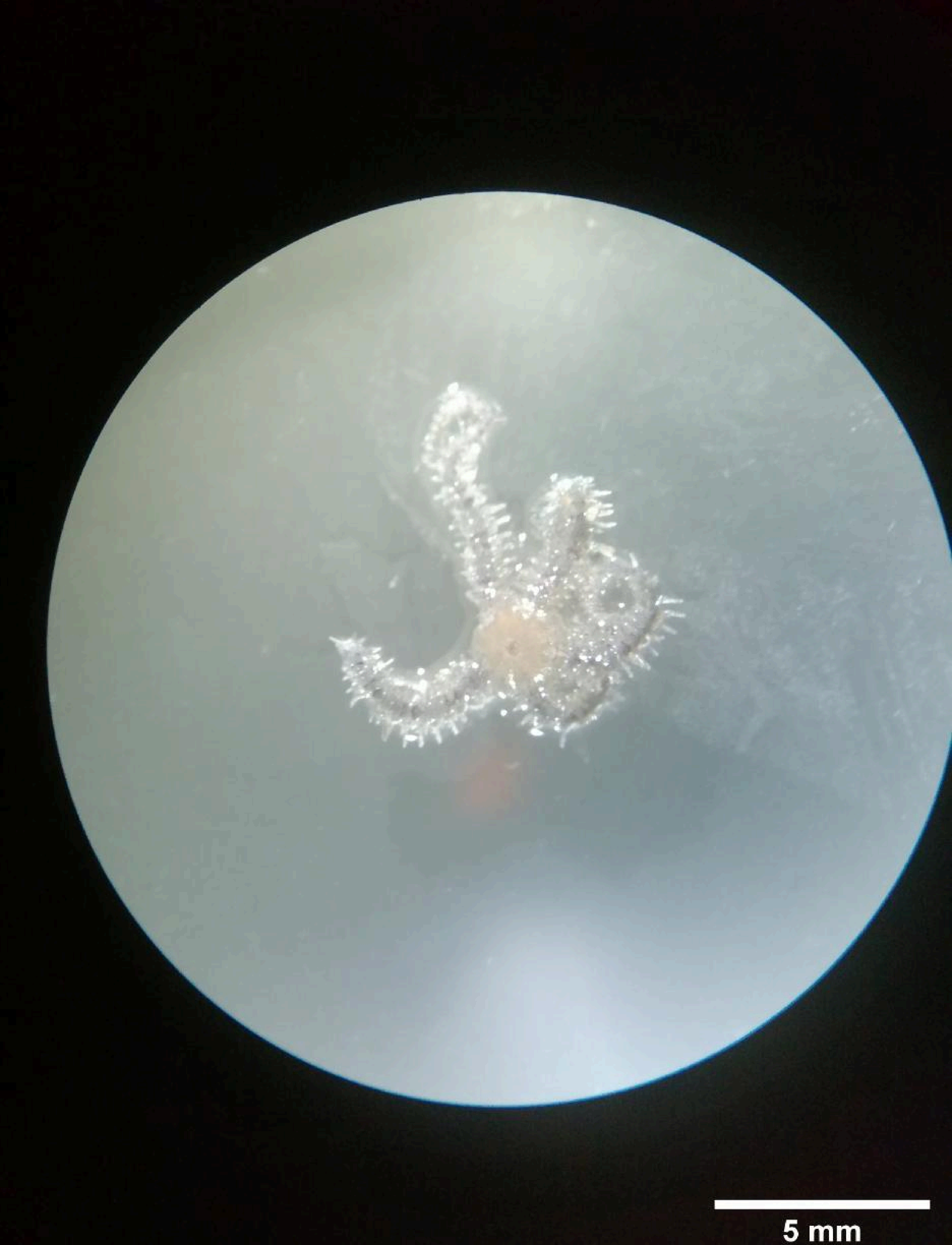
5 mm

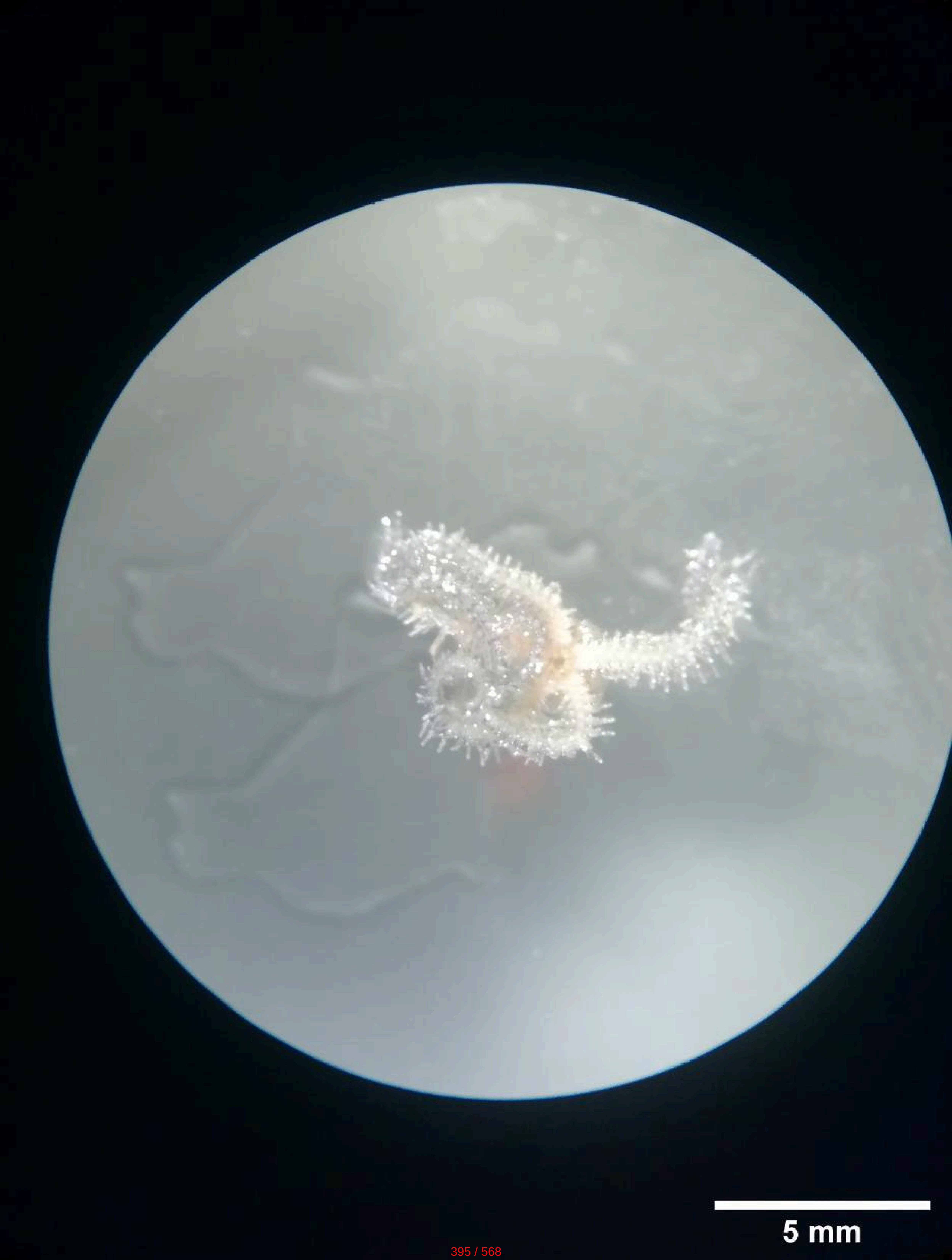
5 mm

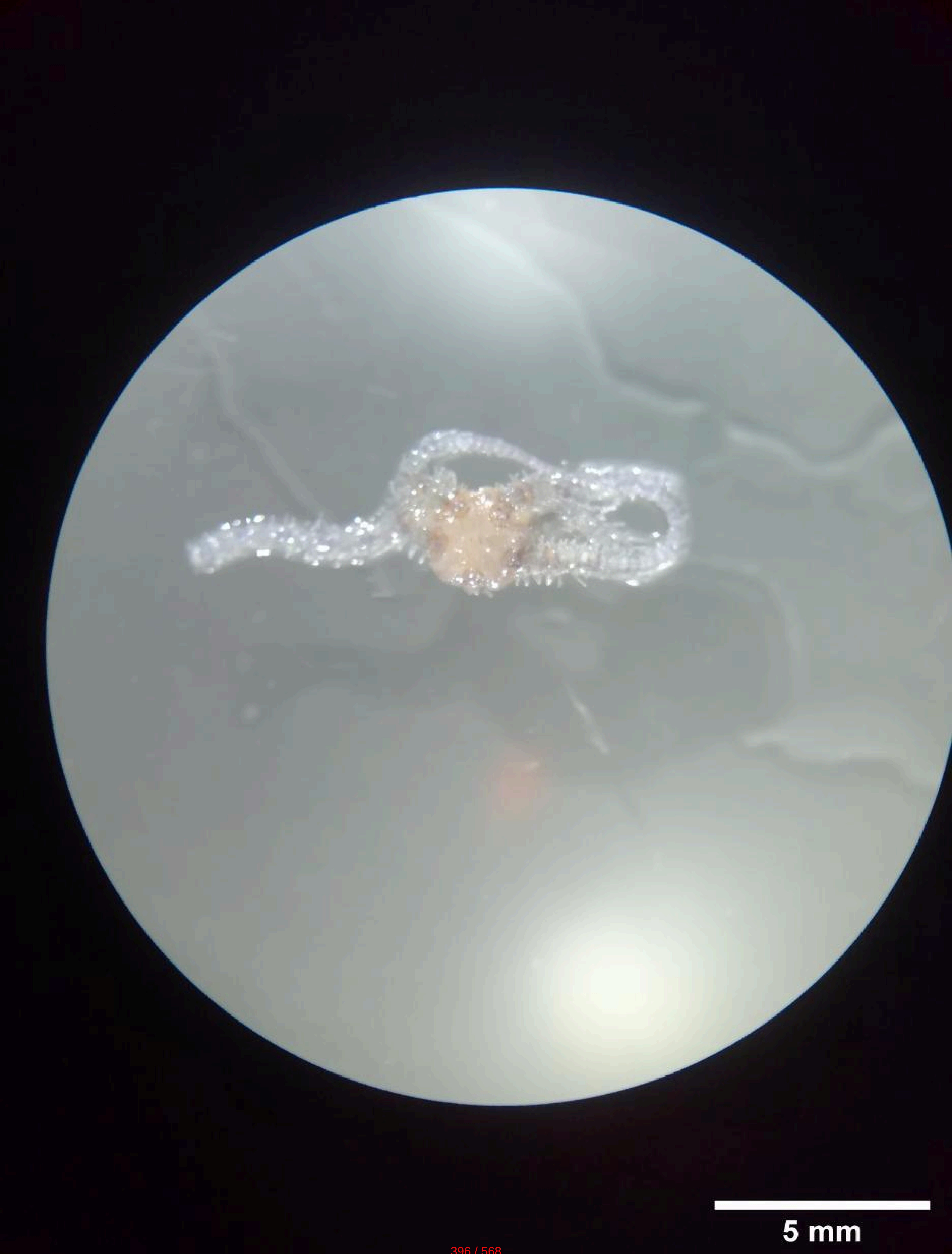
5 mm

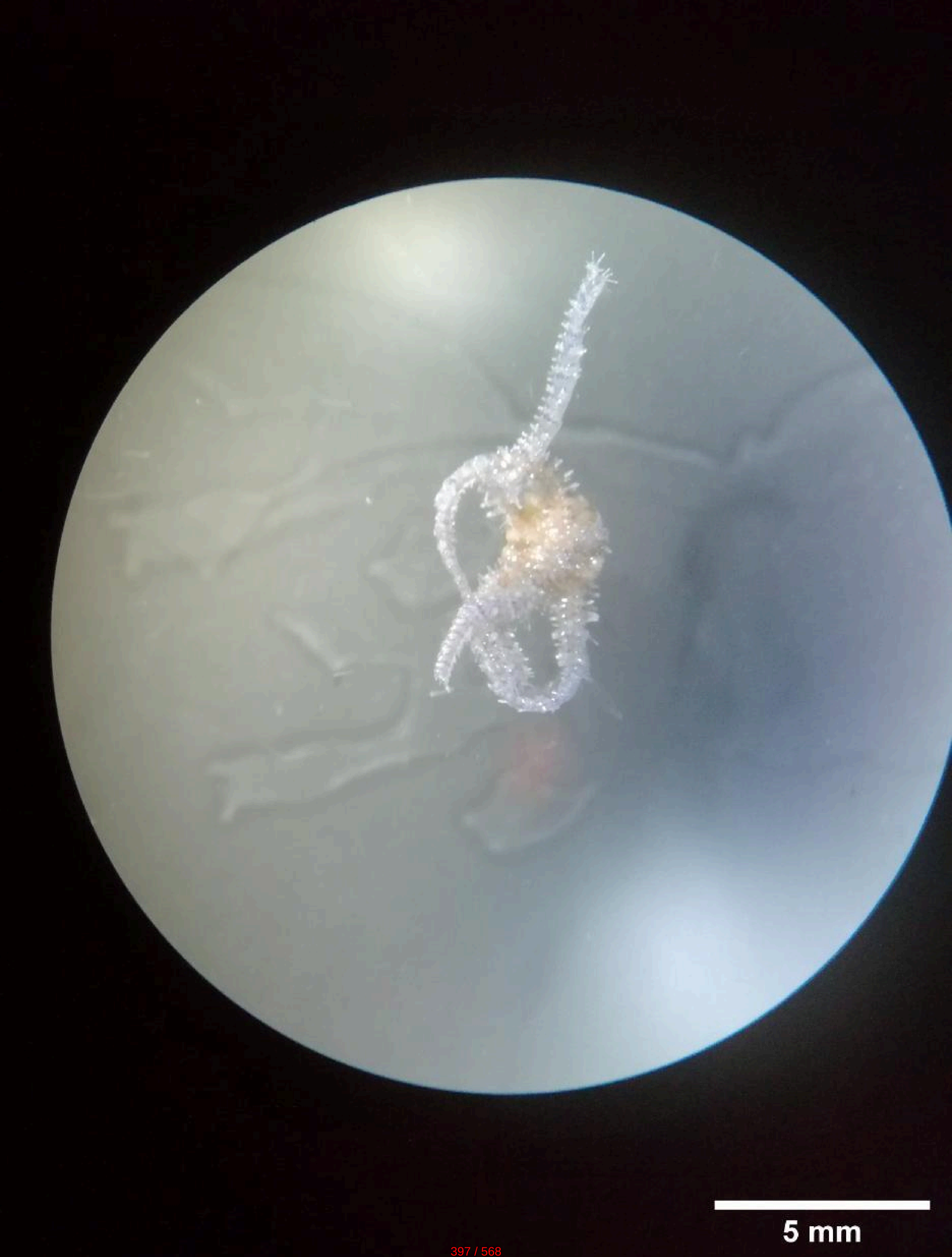
5 mm

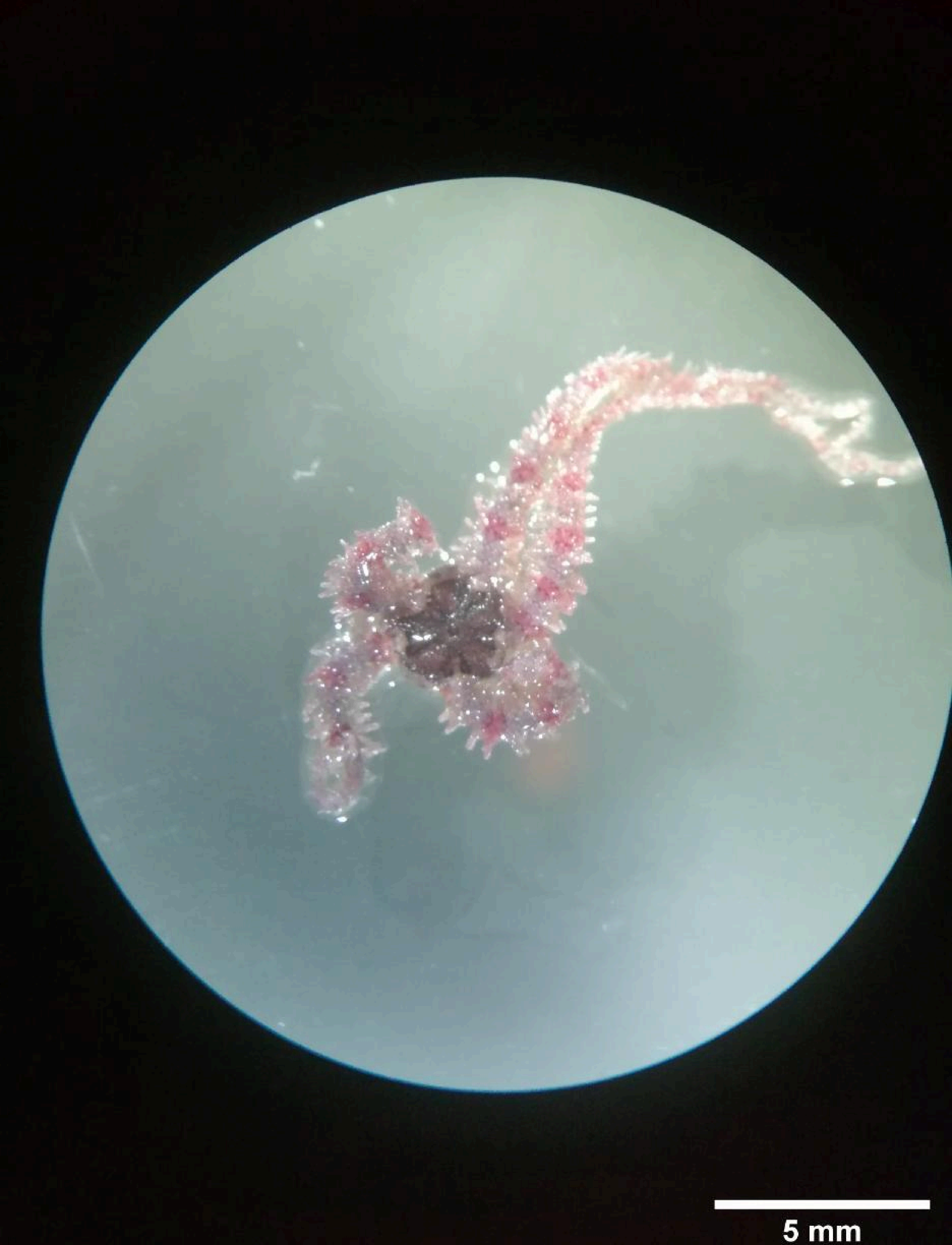
5 mm

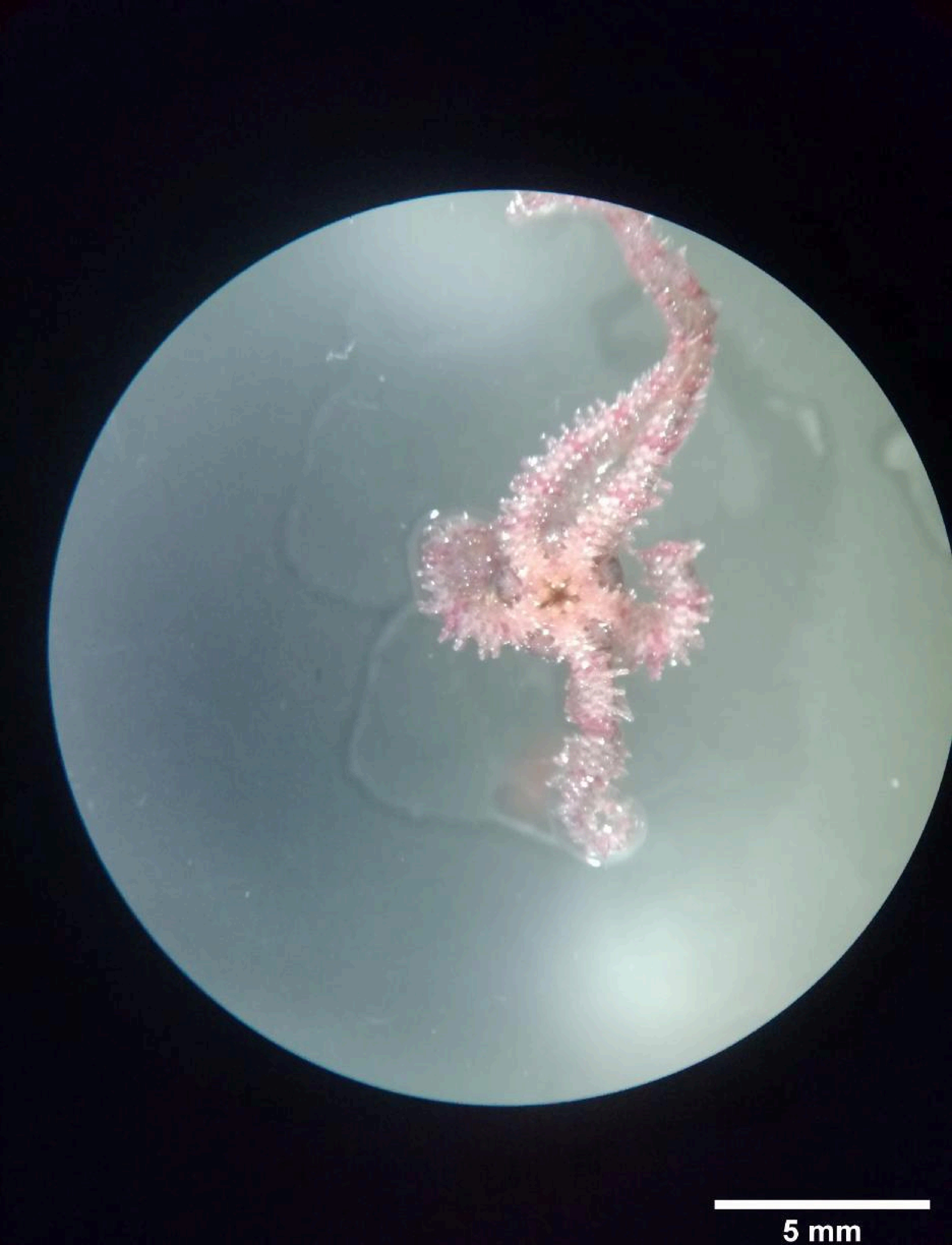
5 mm

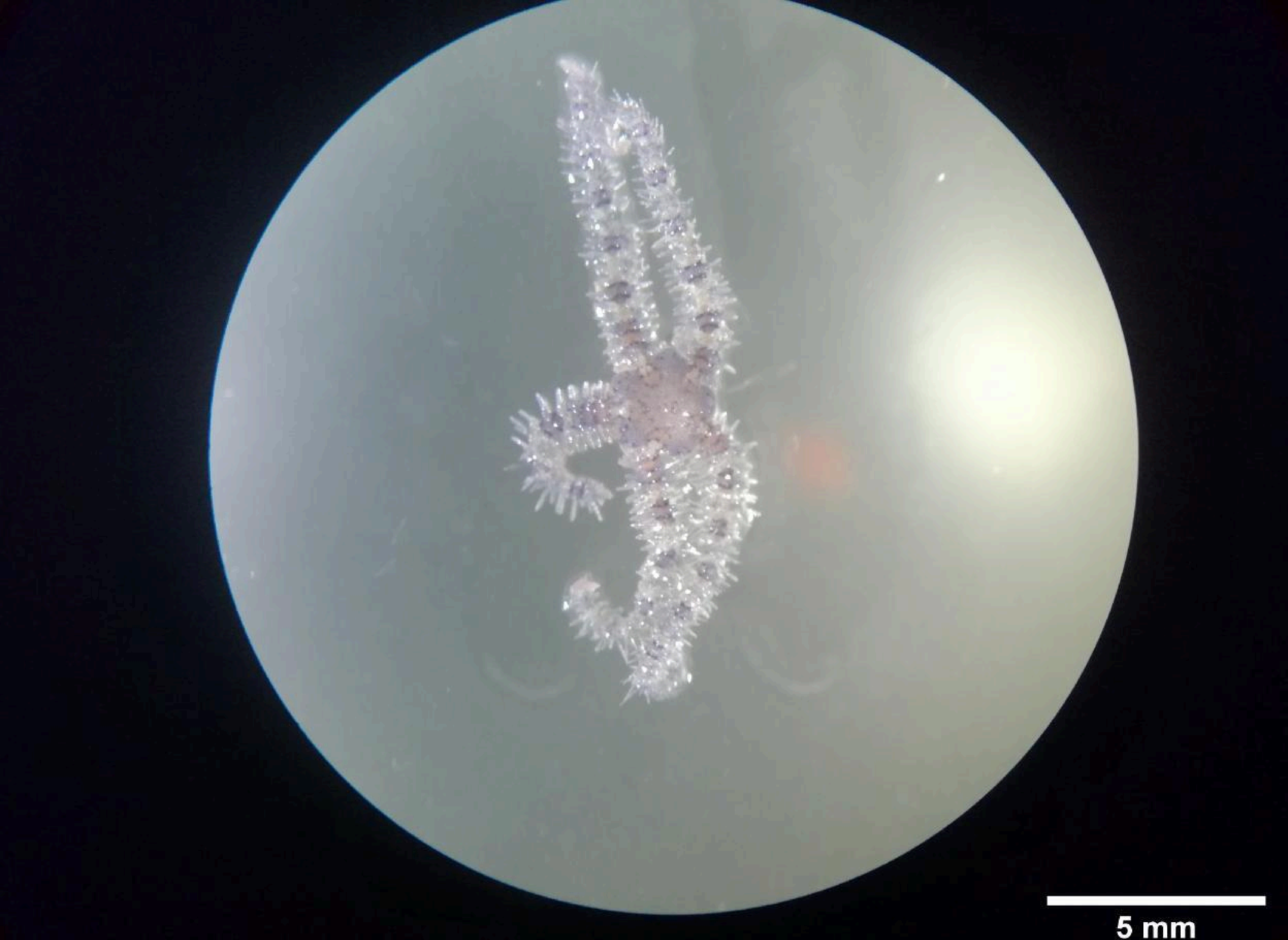
5 mm

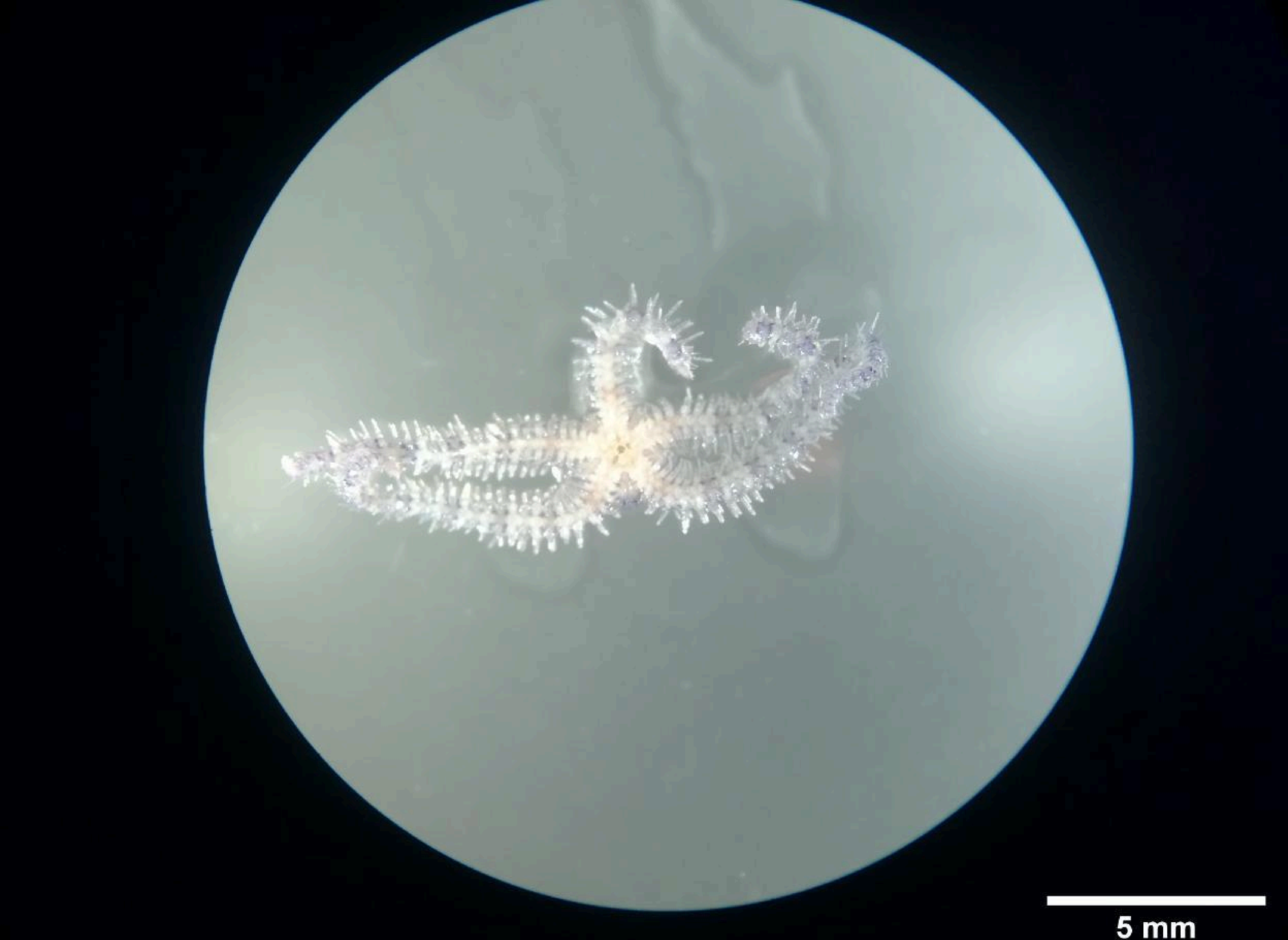
5 mm

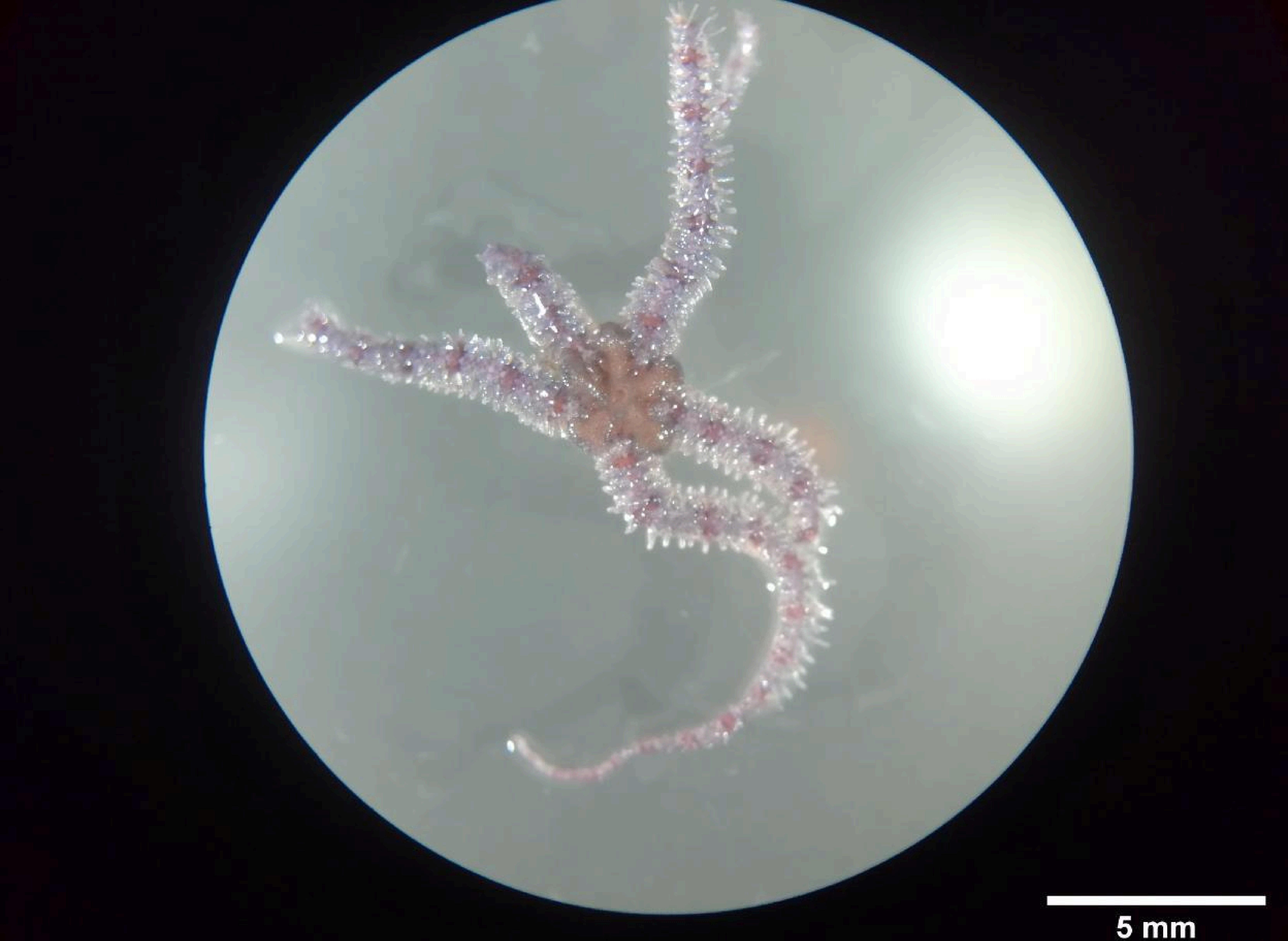
5 mm

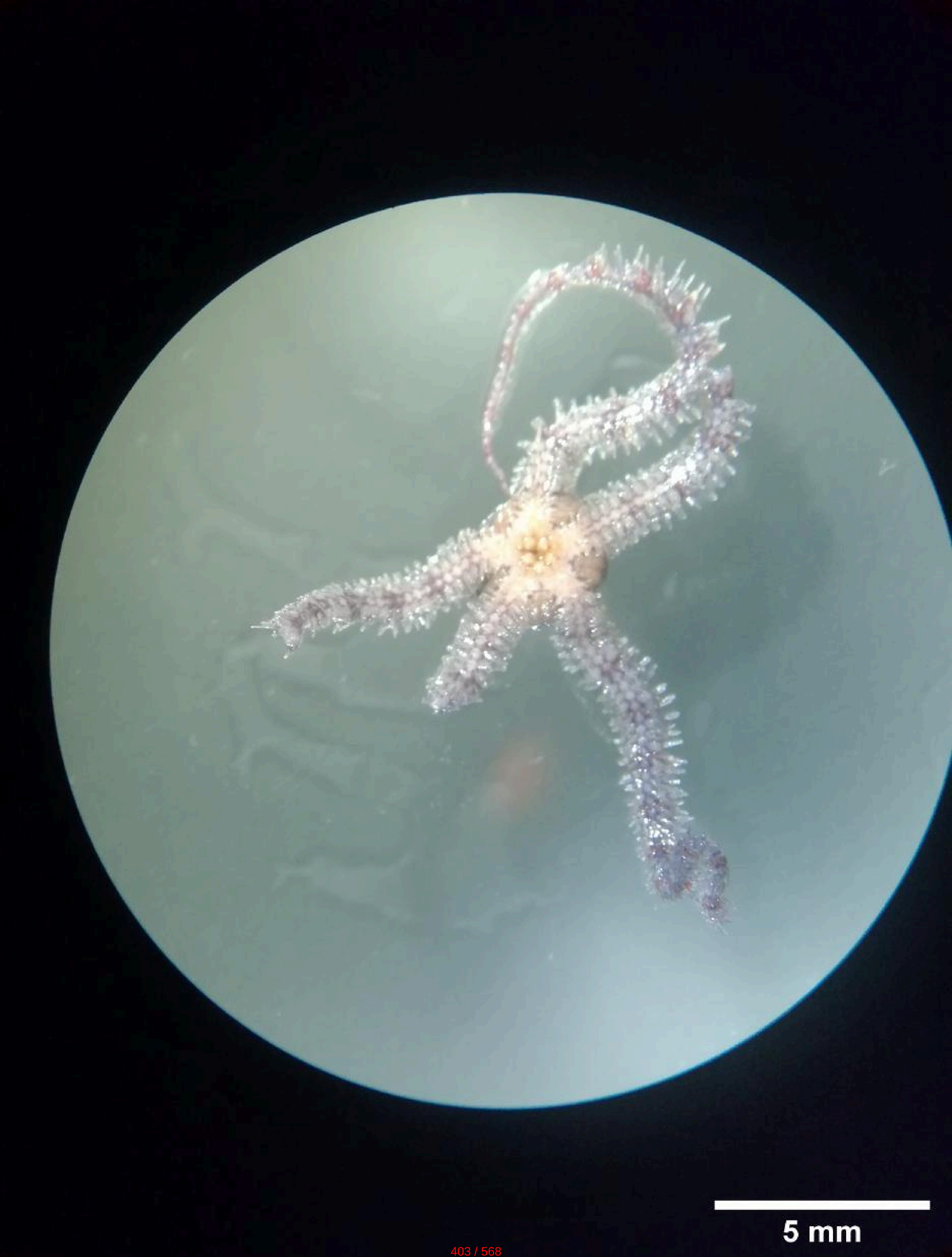
5 mm

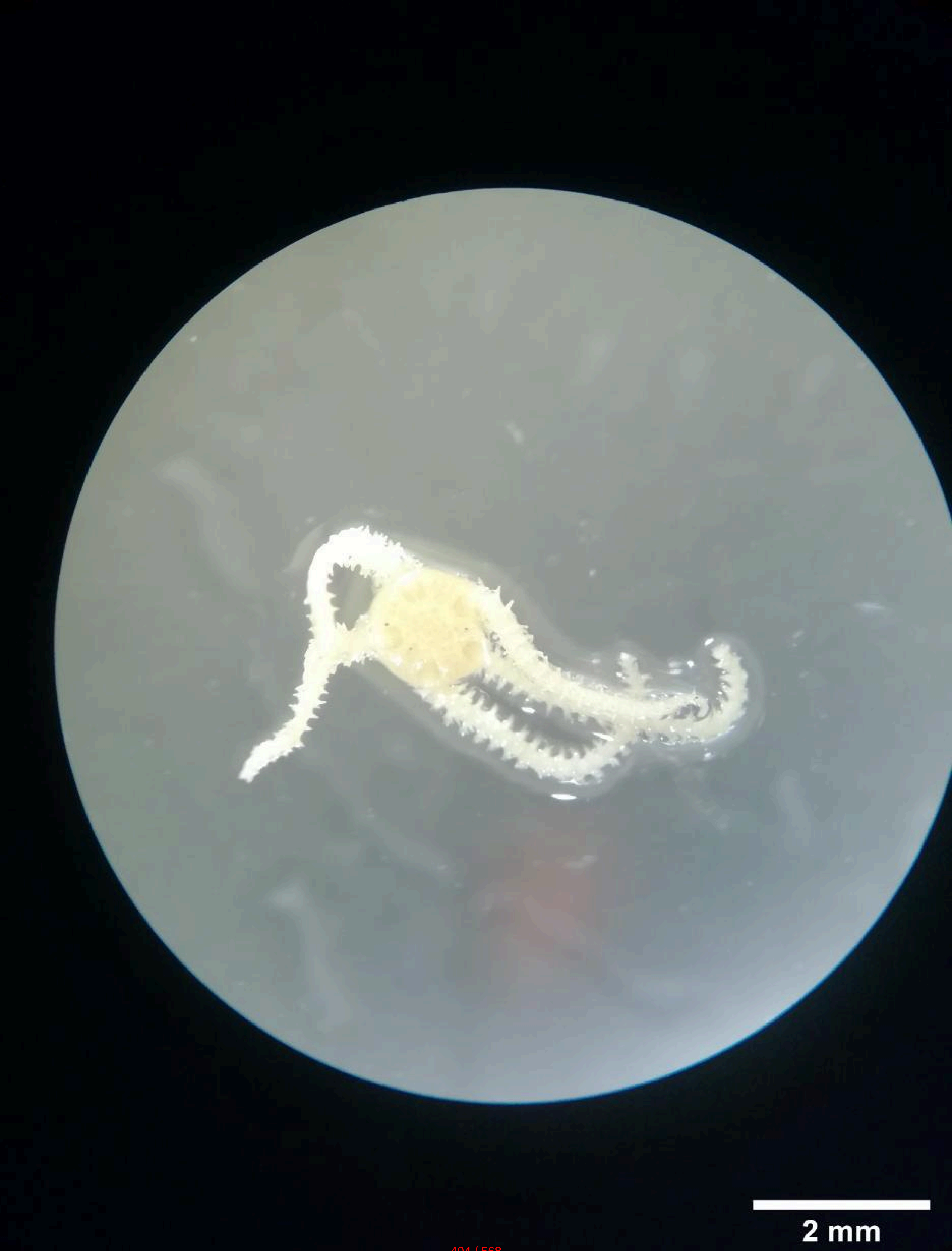
2 mm

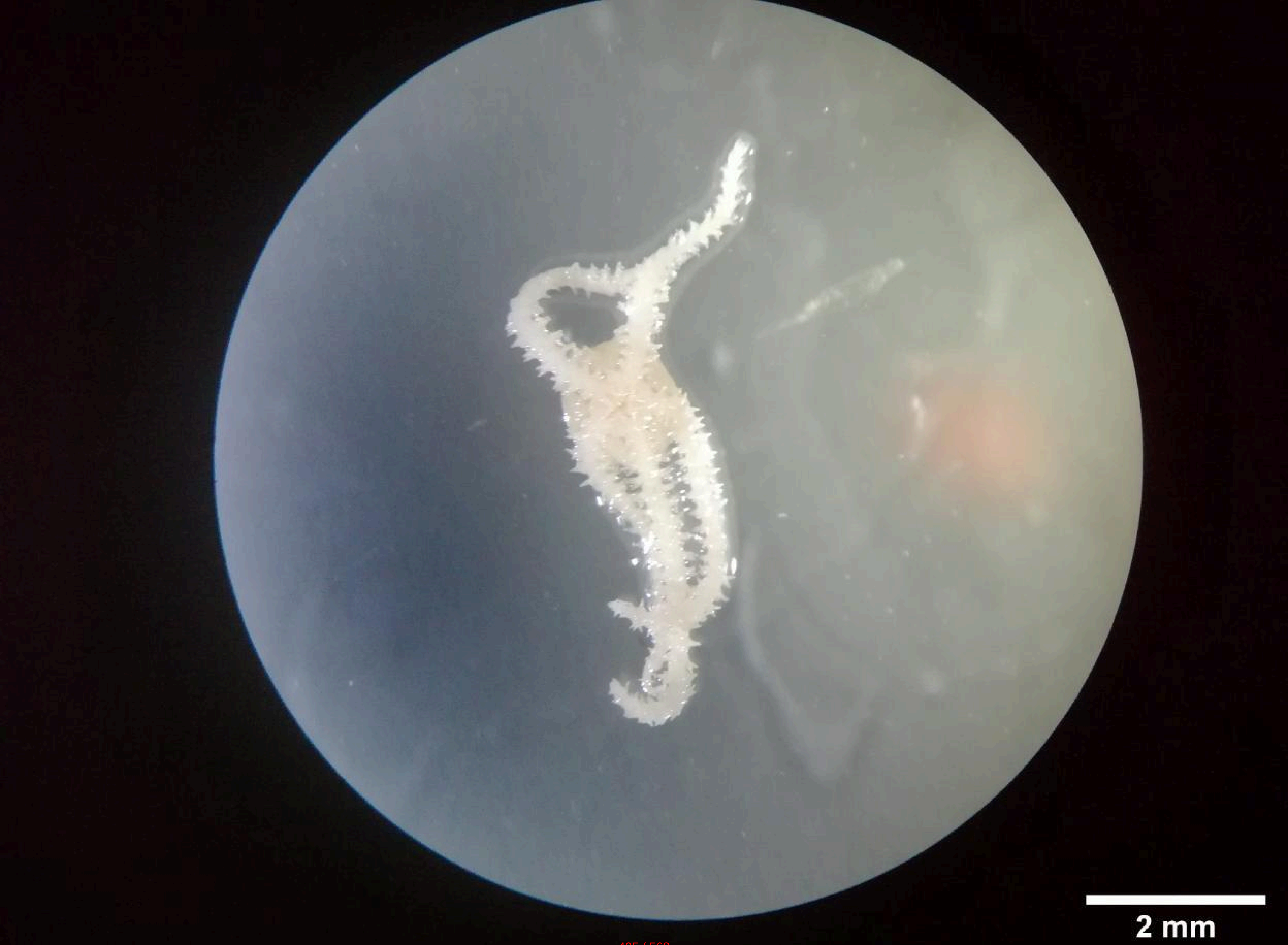
2 mm

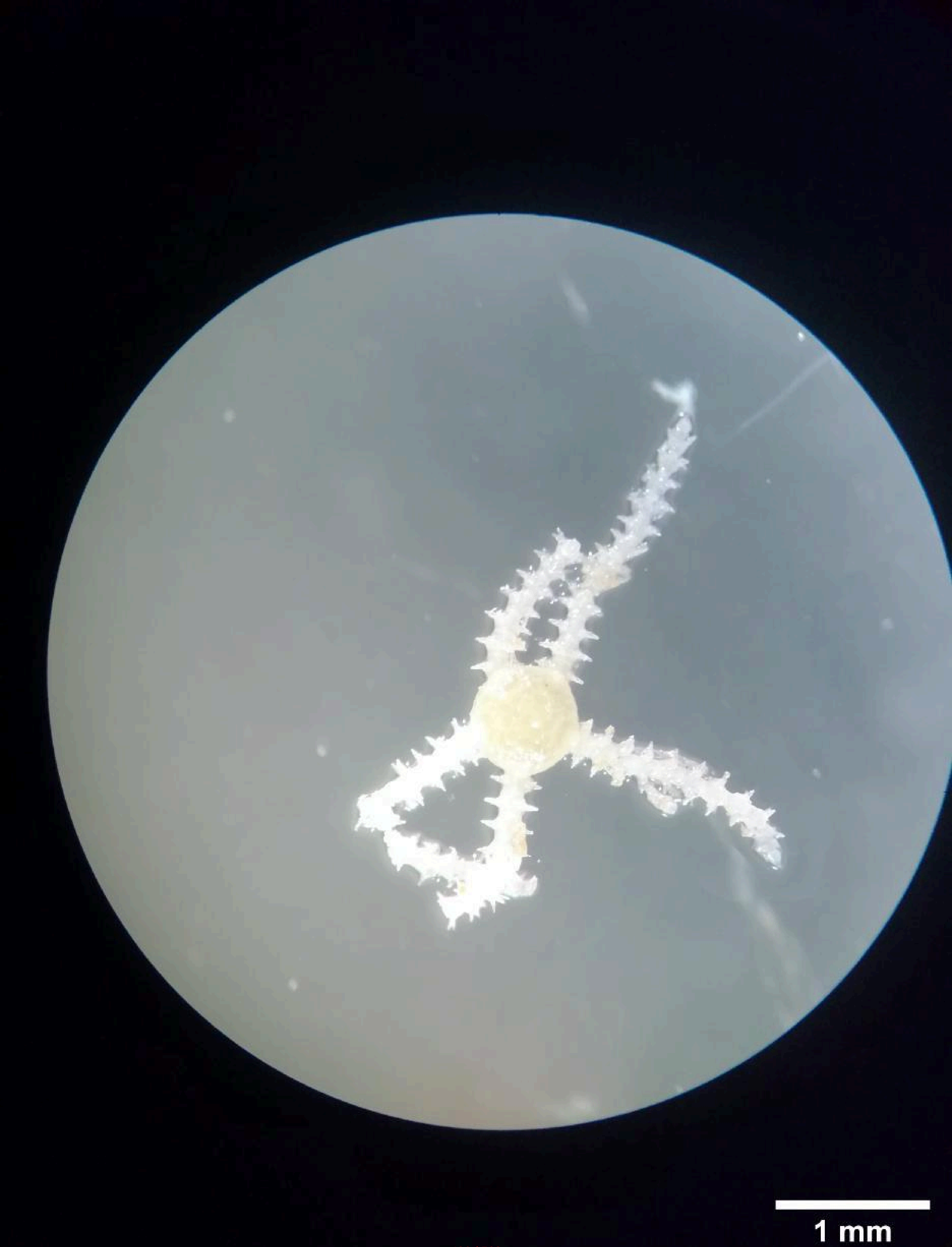
1 mm

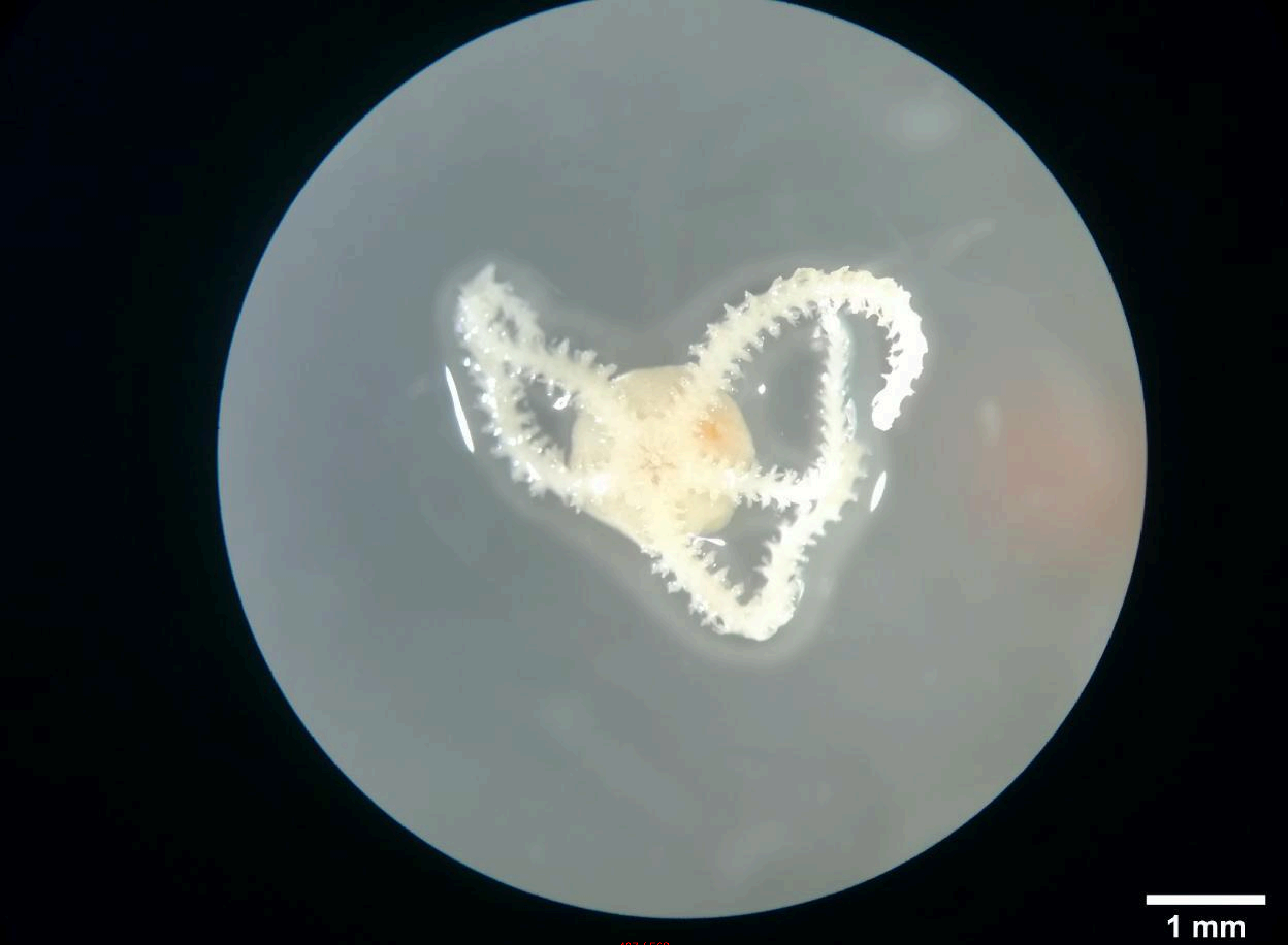
1 mm

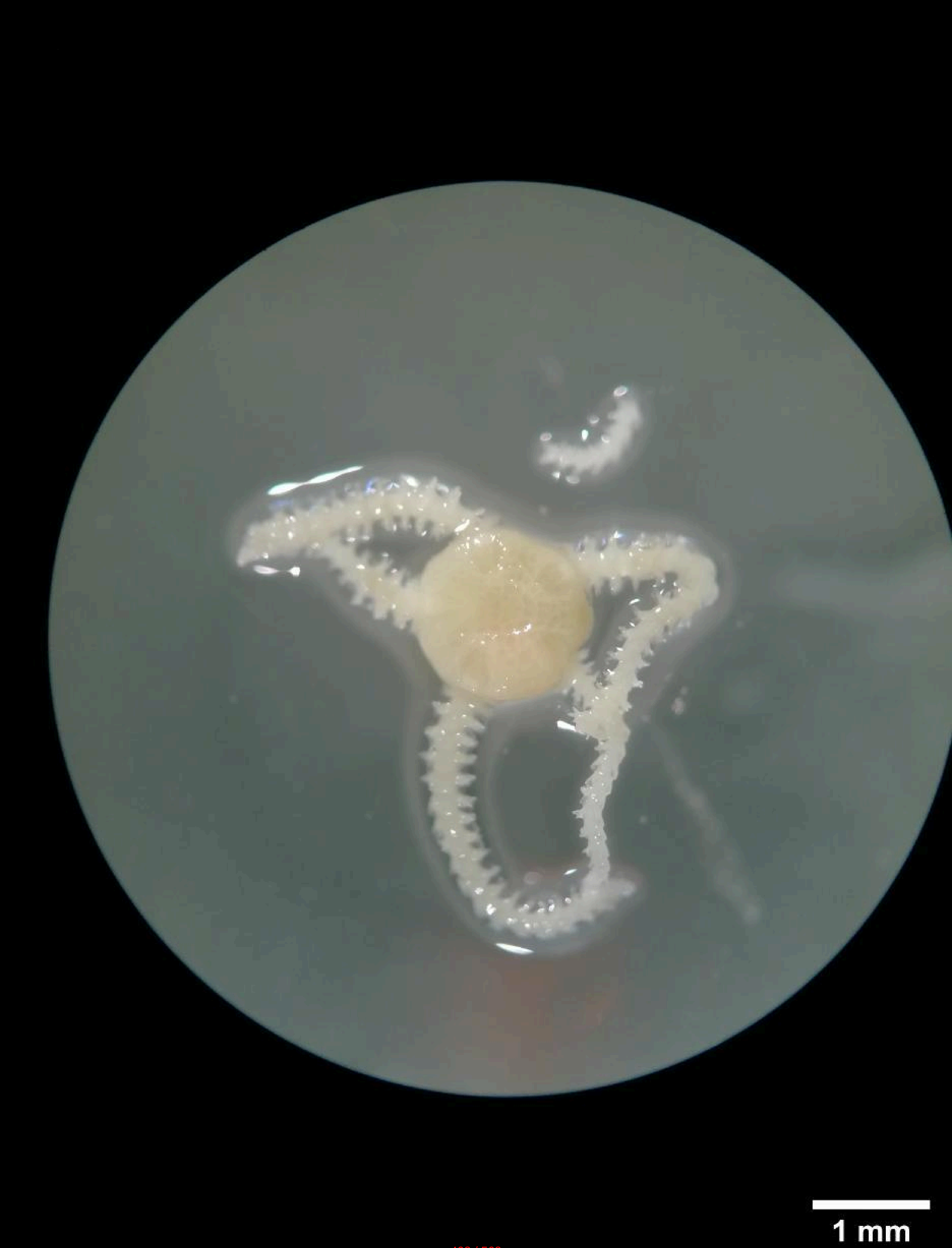
1 mm

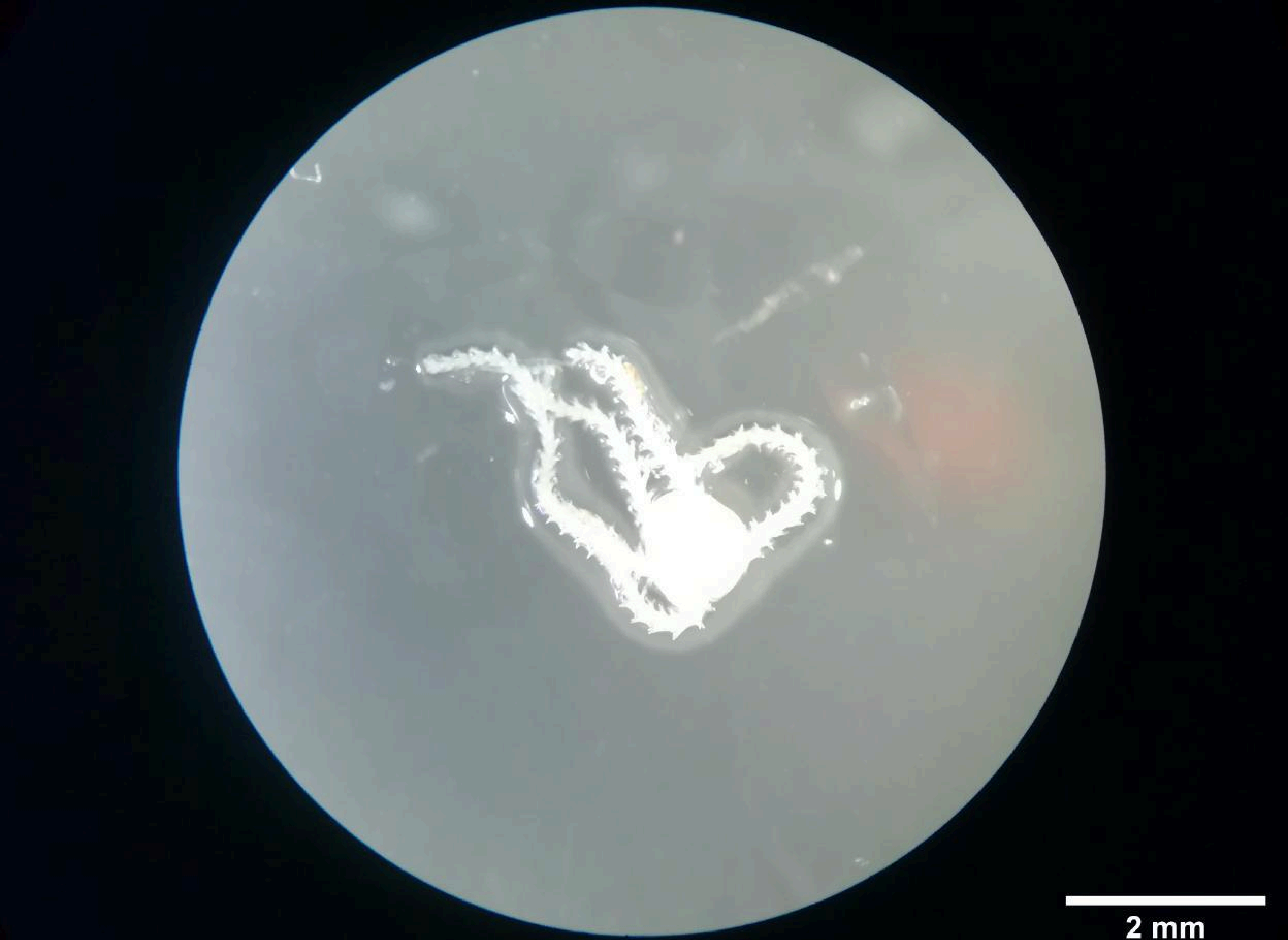
2 mm

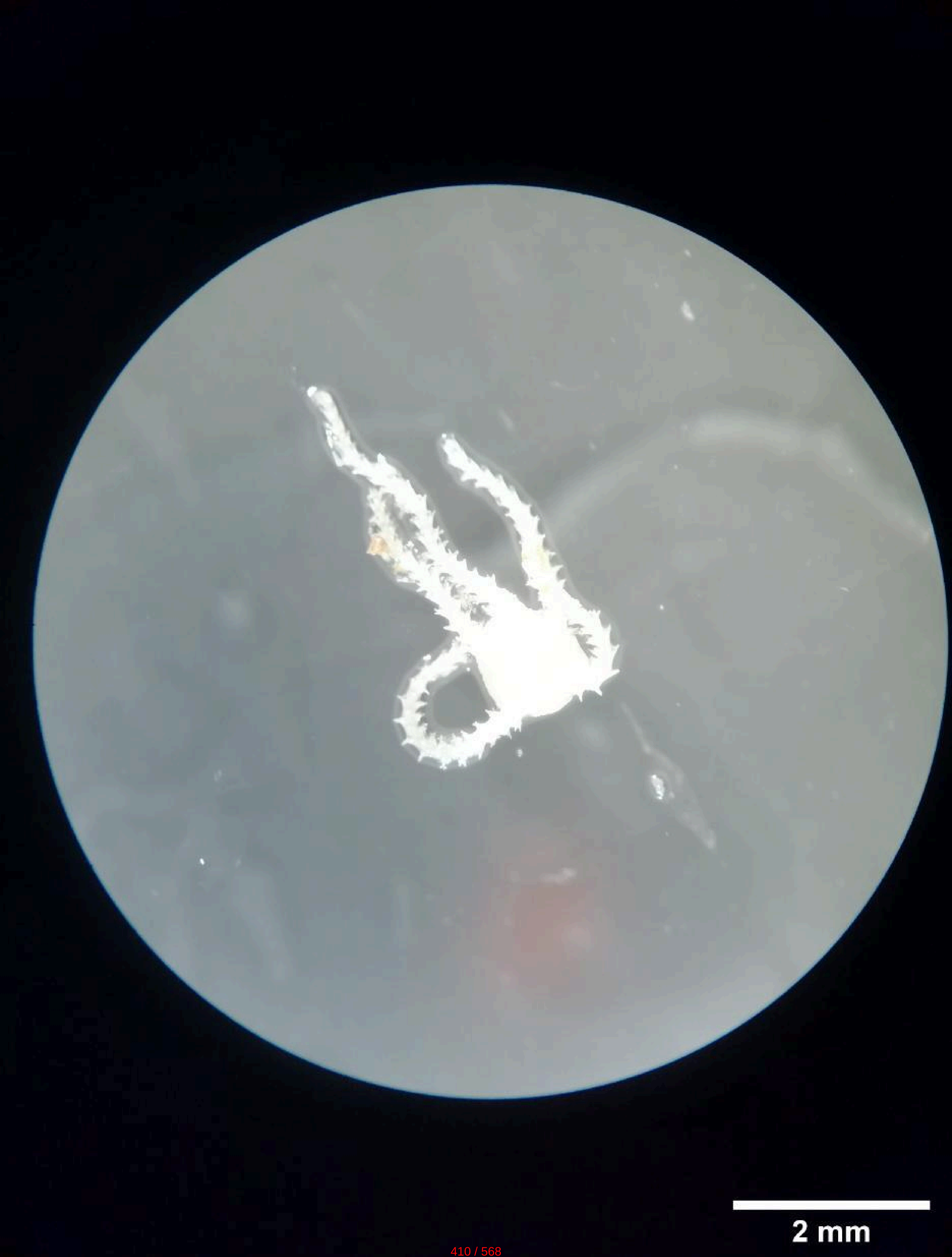
2 mm

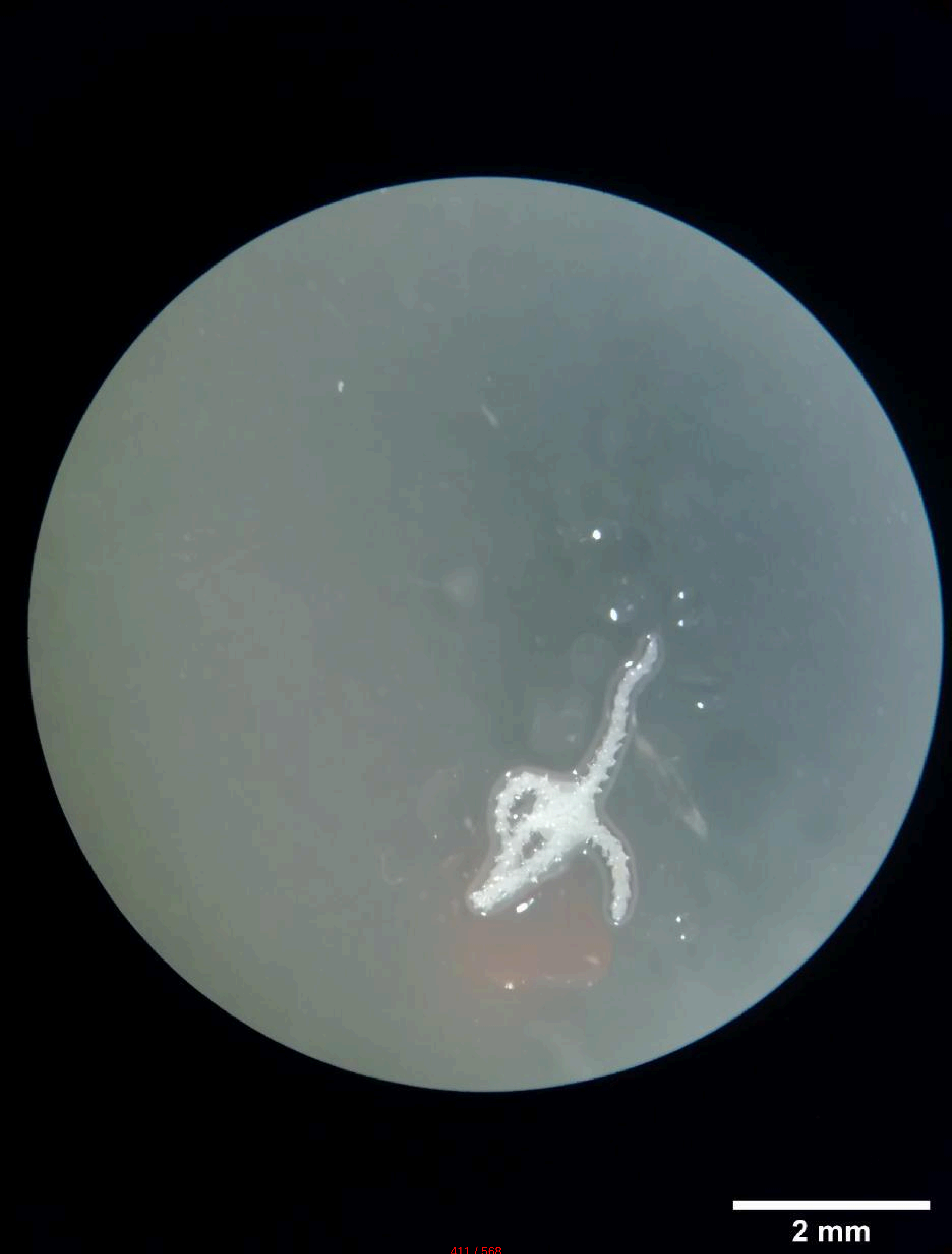
2 mm

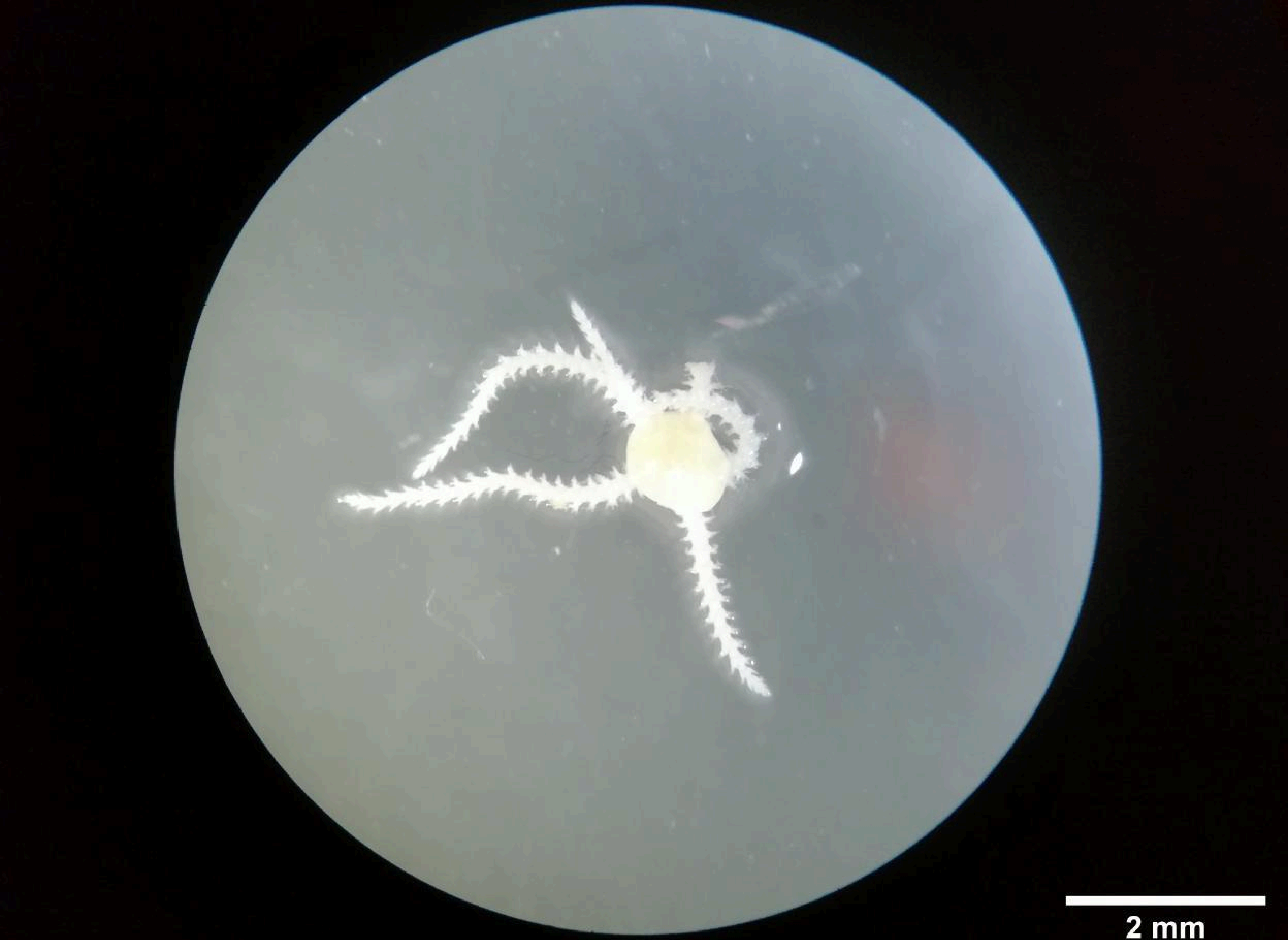
2 mm

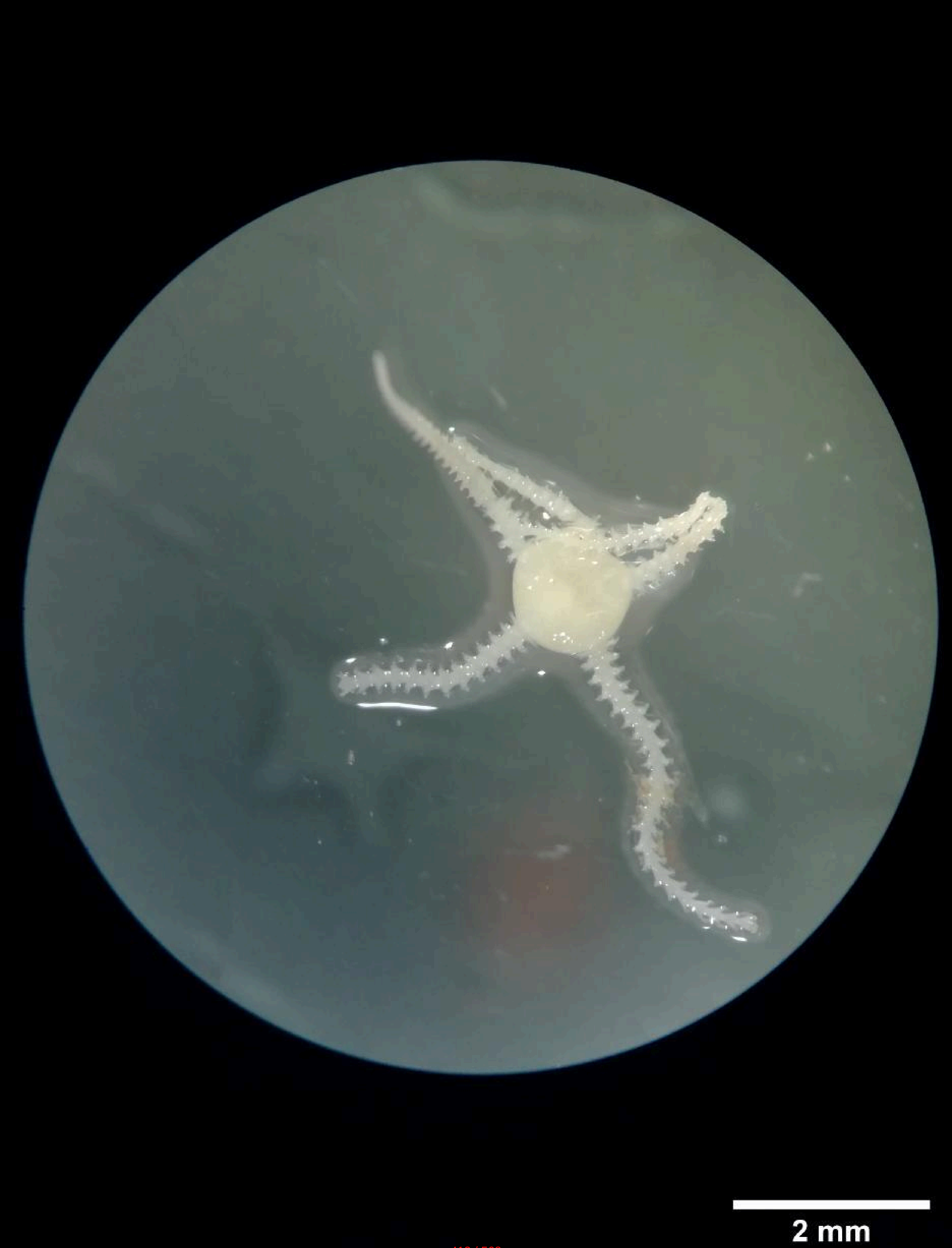
2 mm

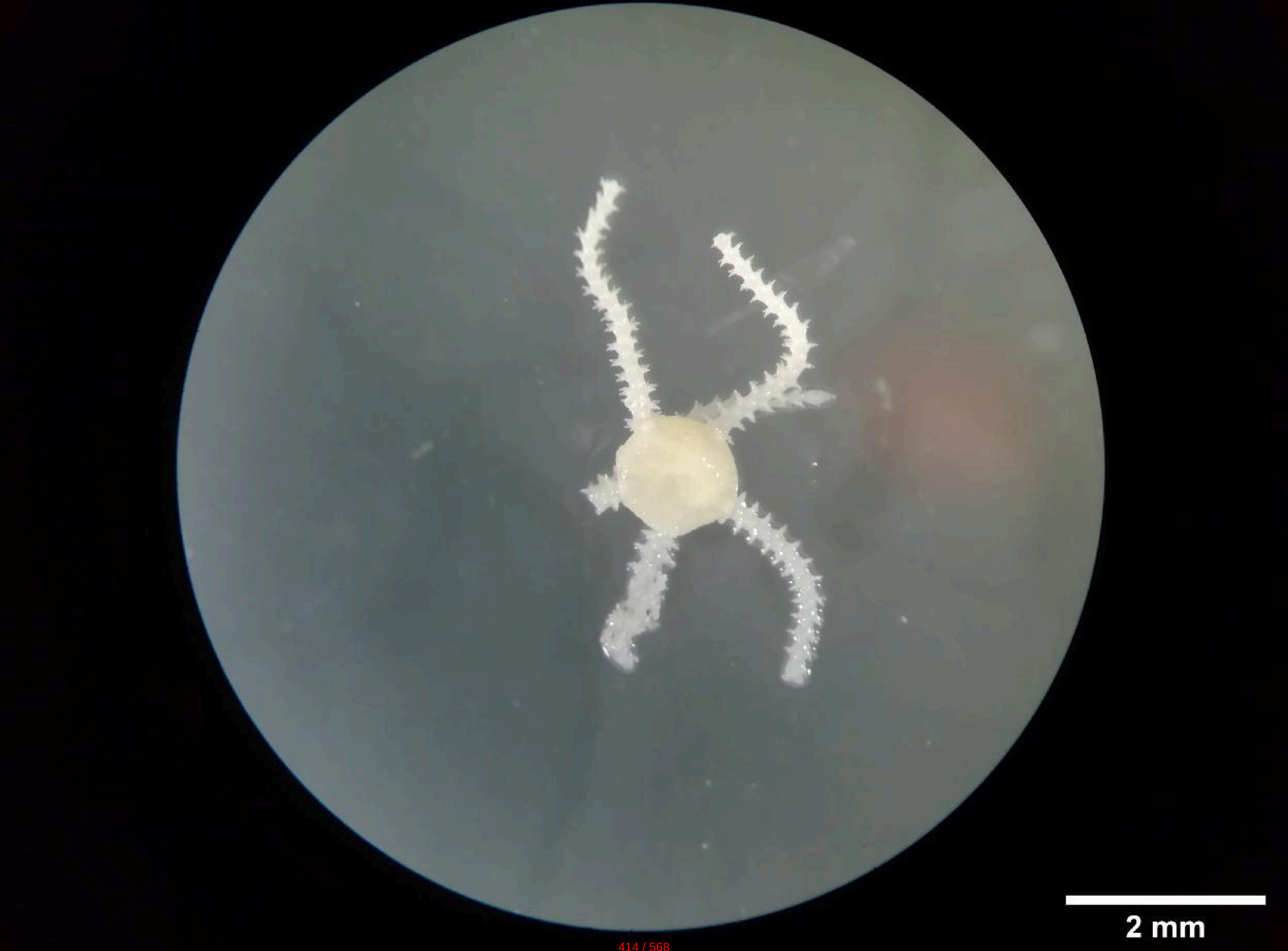
2 mm

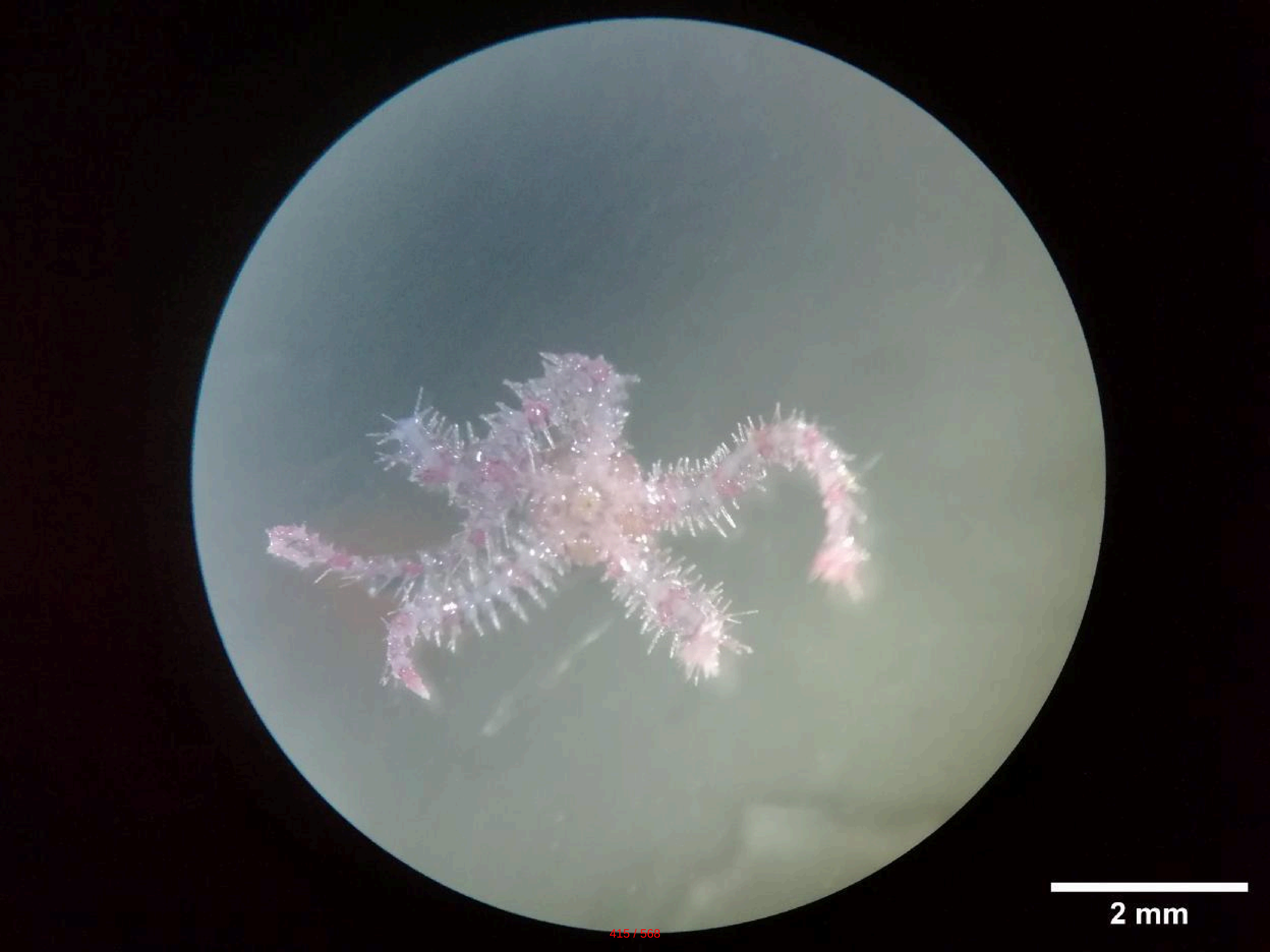
2 mm

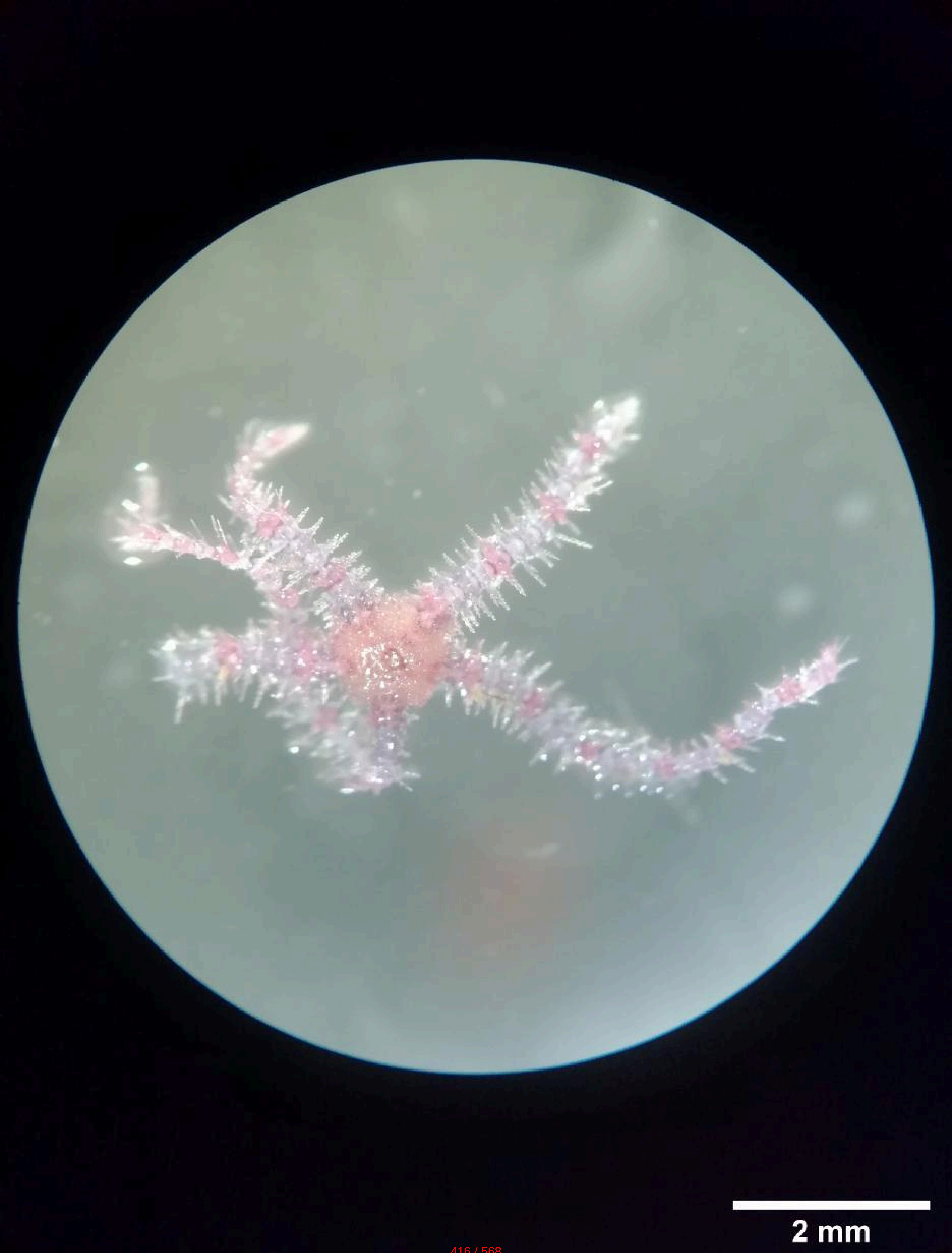
2 mm

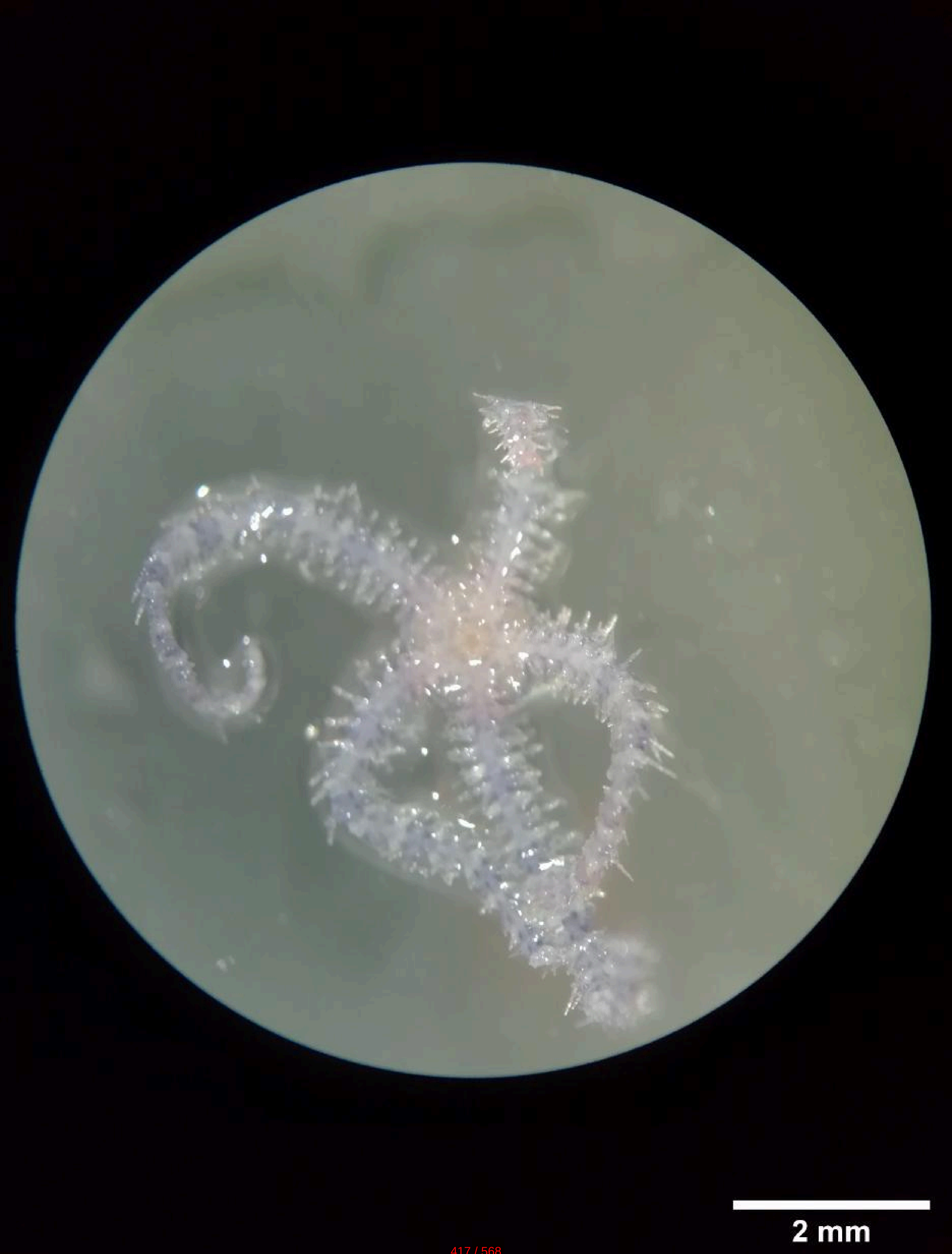
2 mm

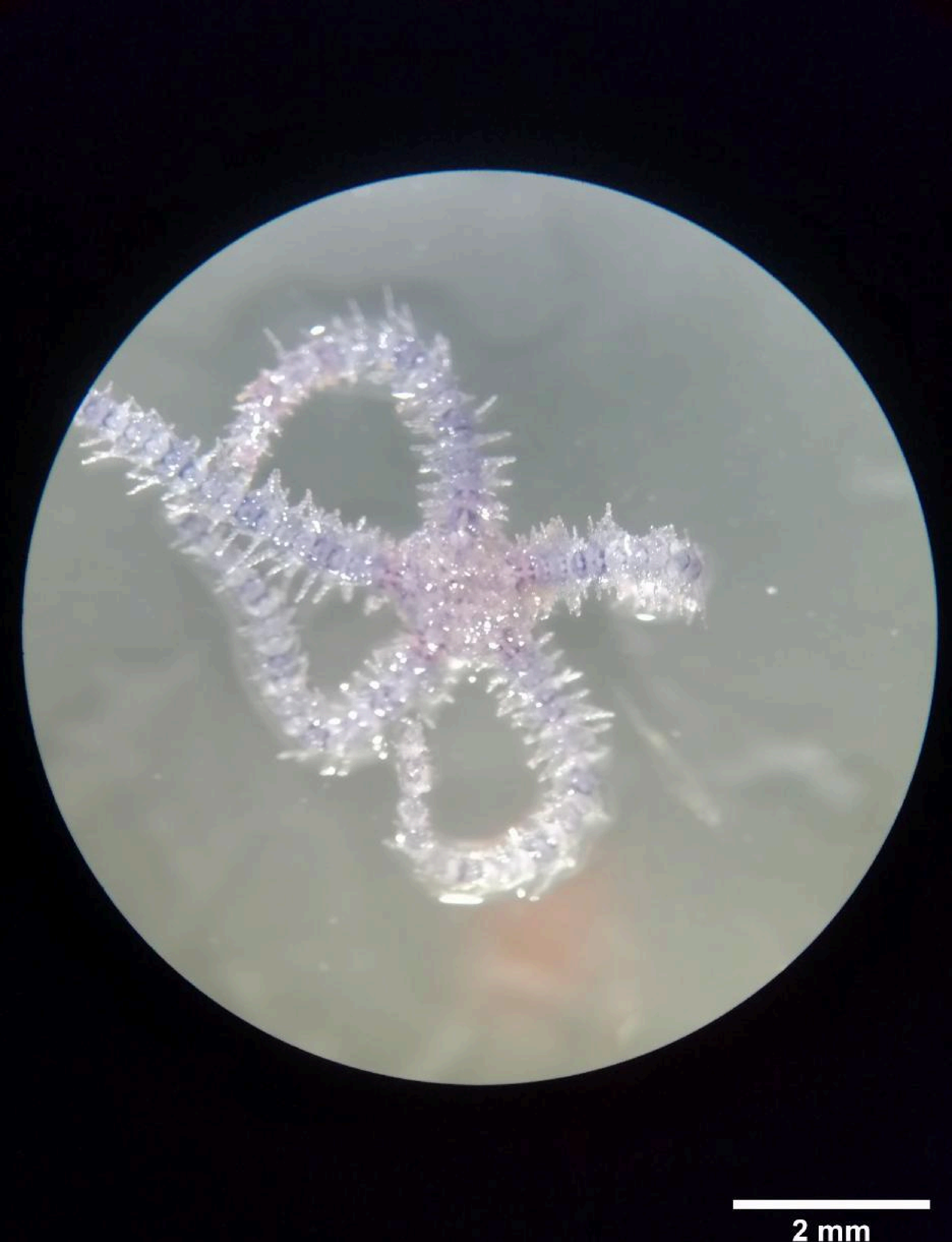
2 mm

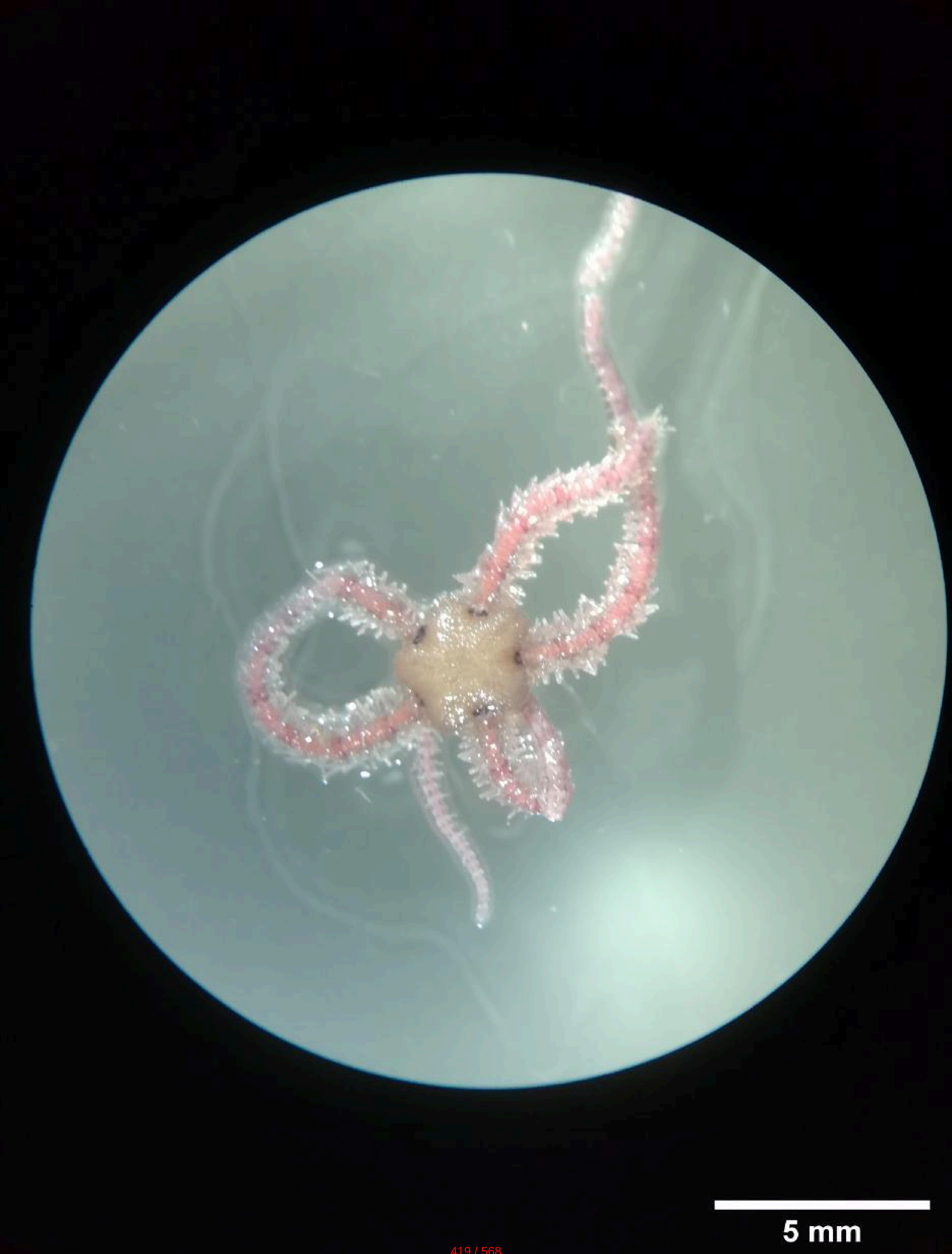
5 mm

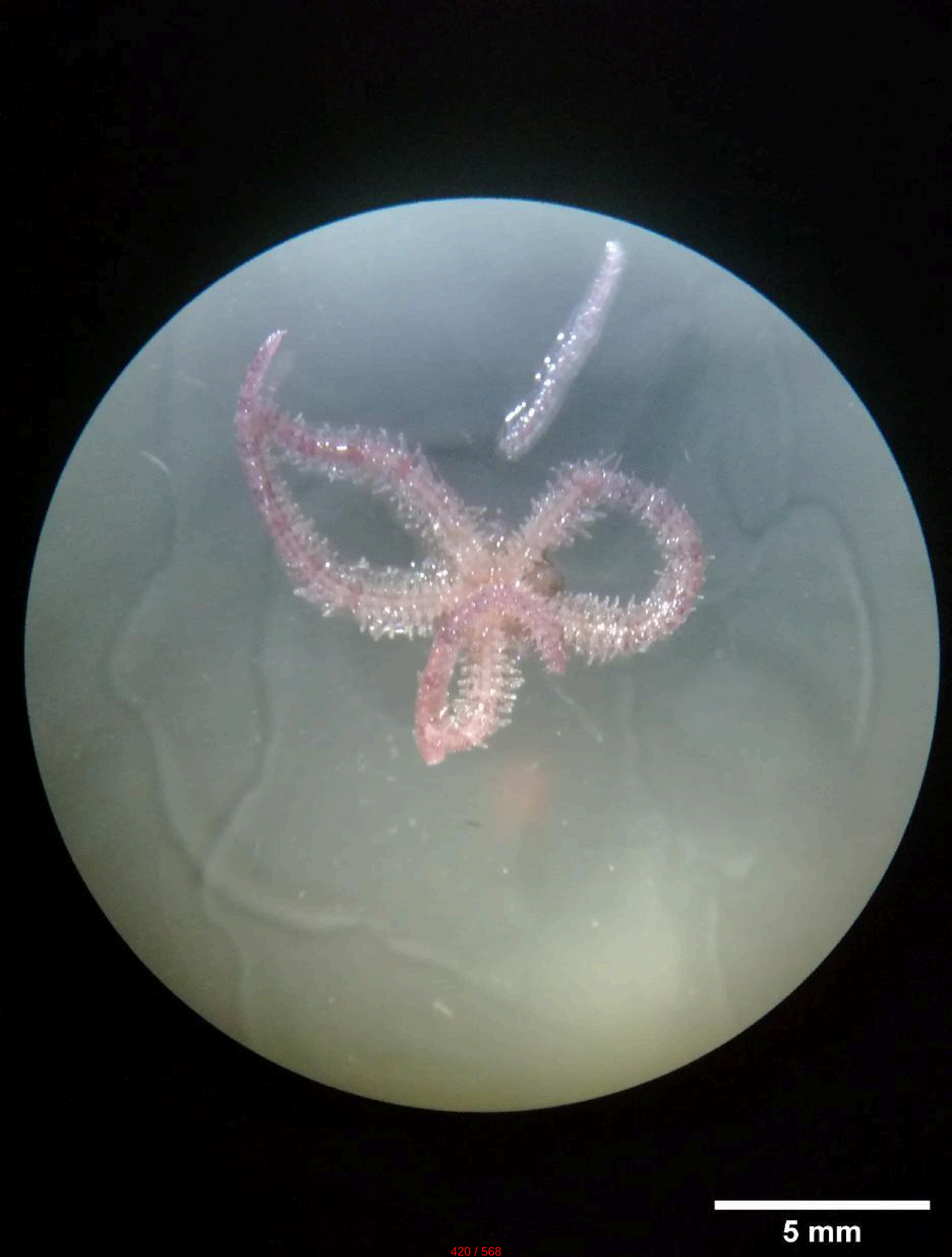
5 mm

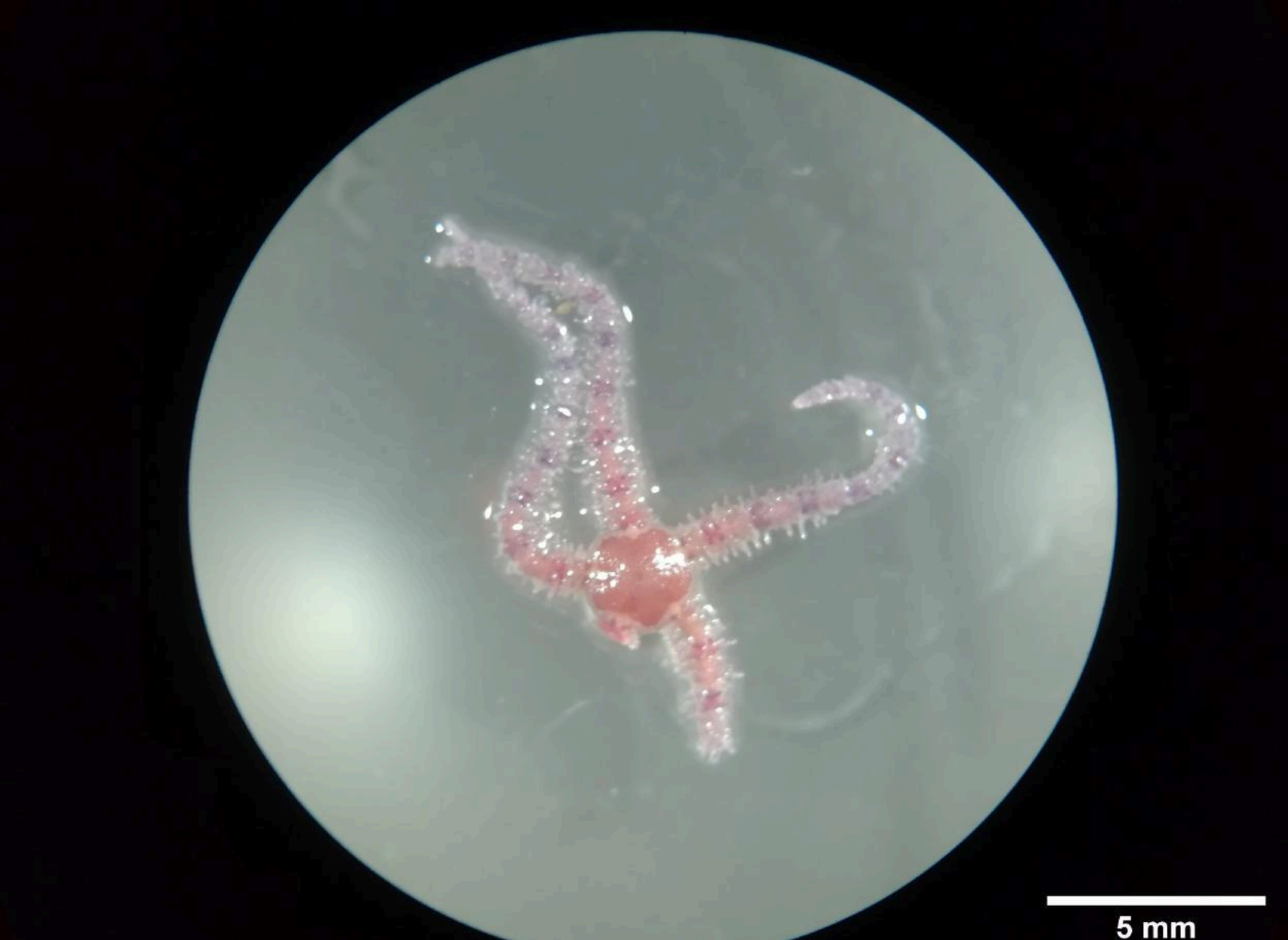
5 mm

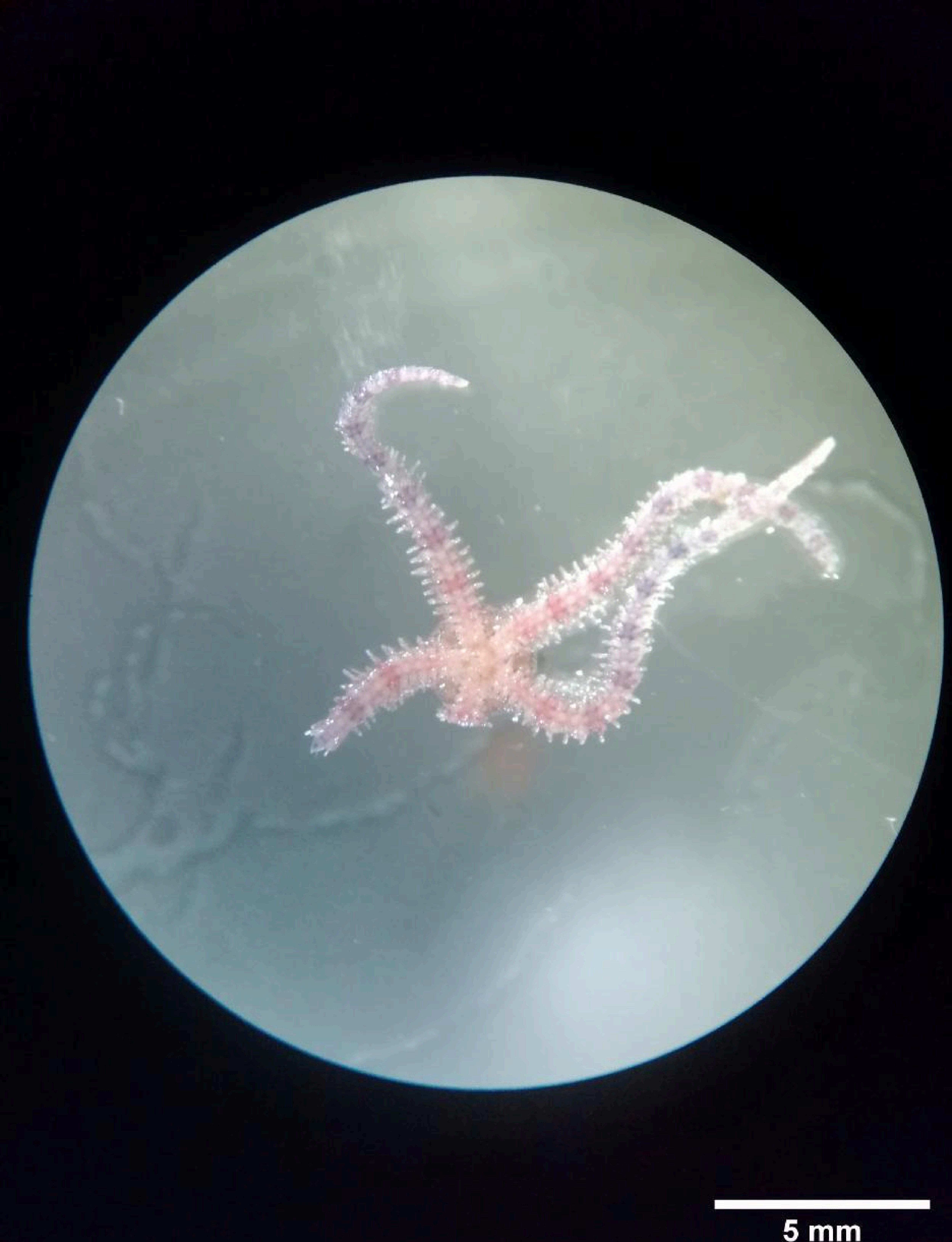
5 mm

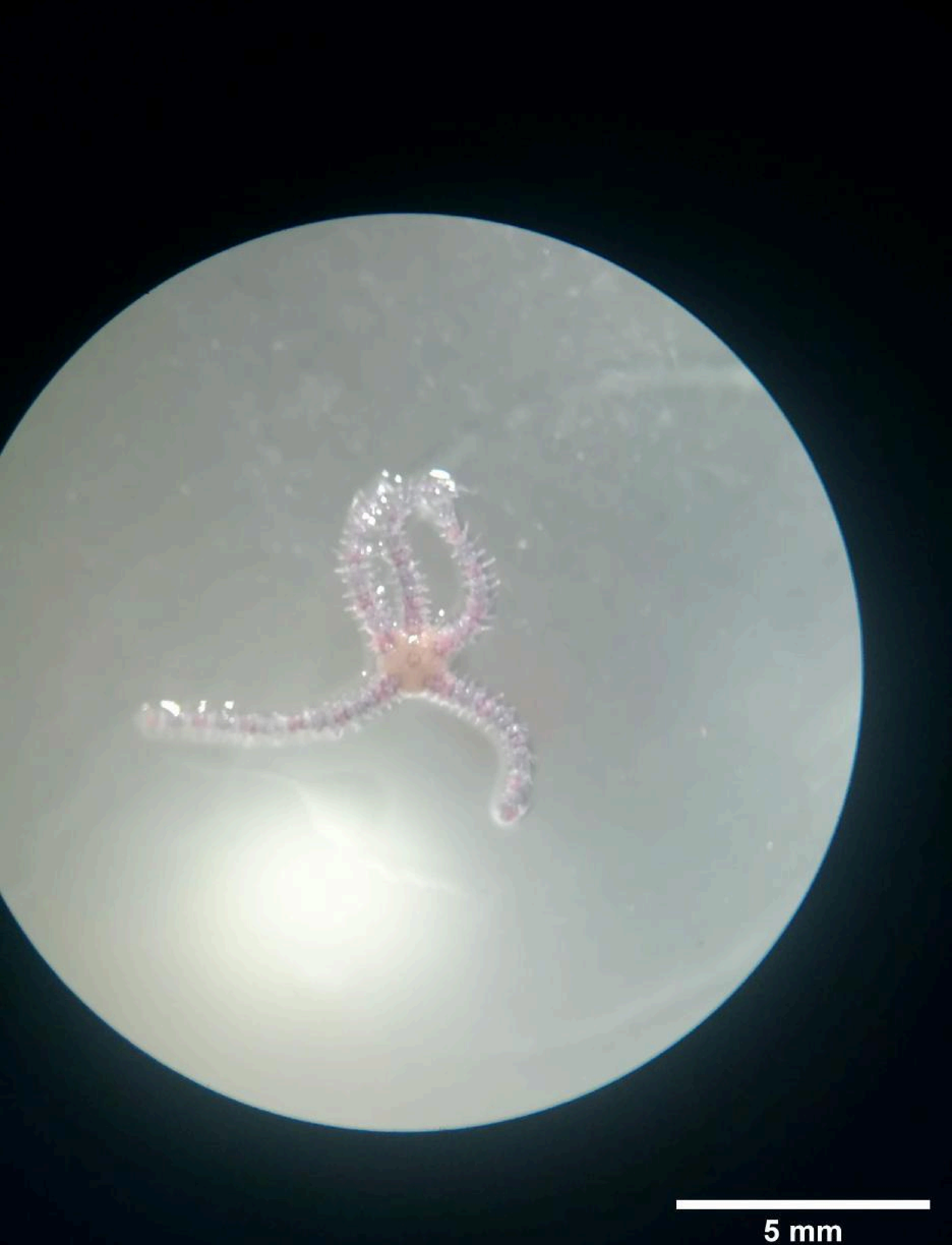
5 mm

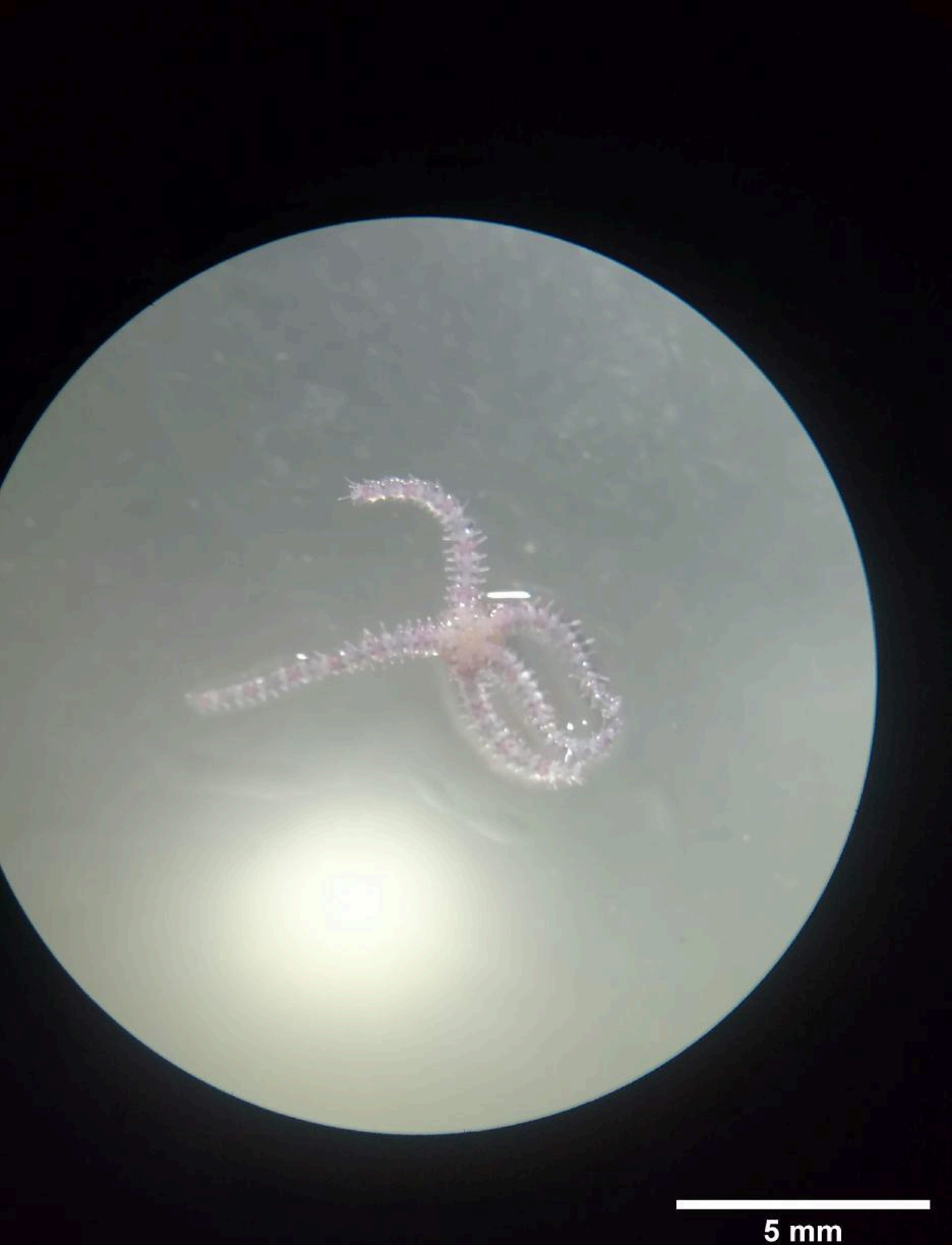
5 mm

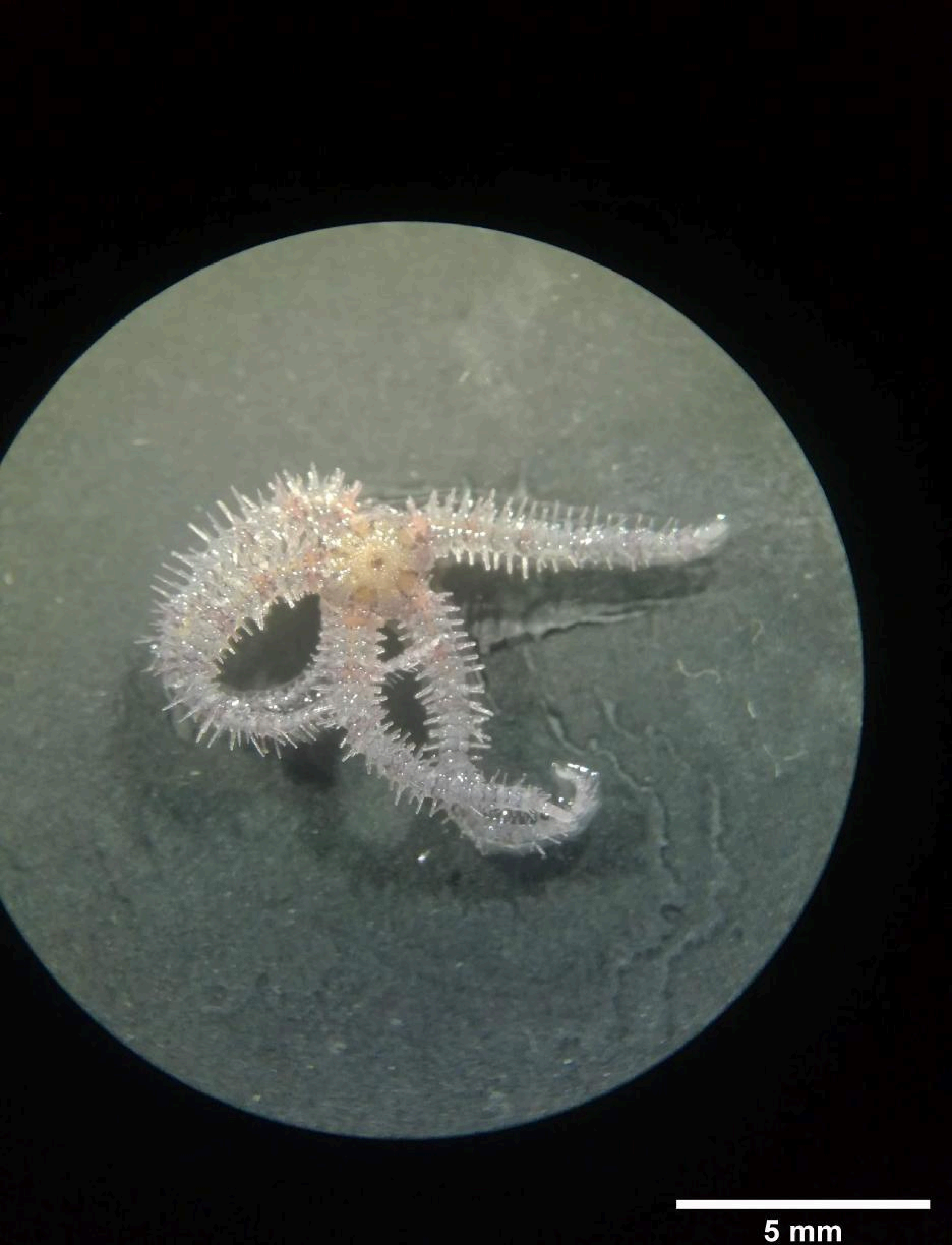
5 mm

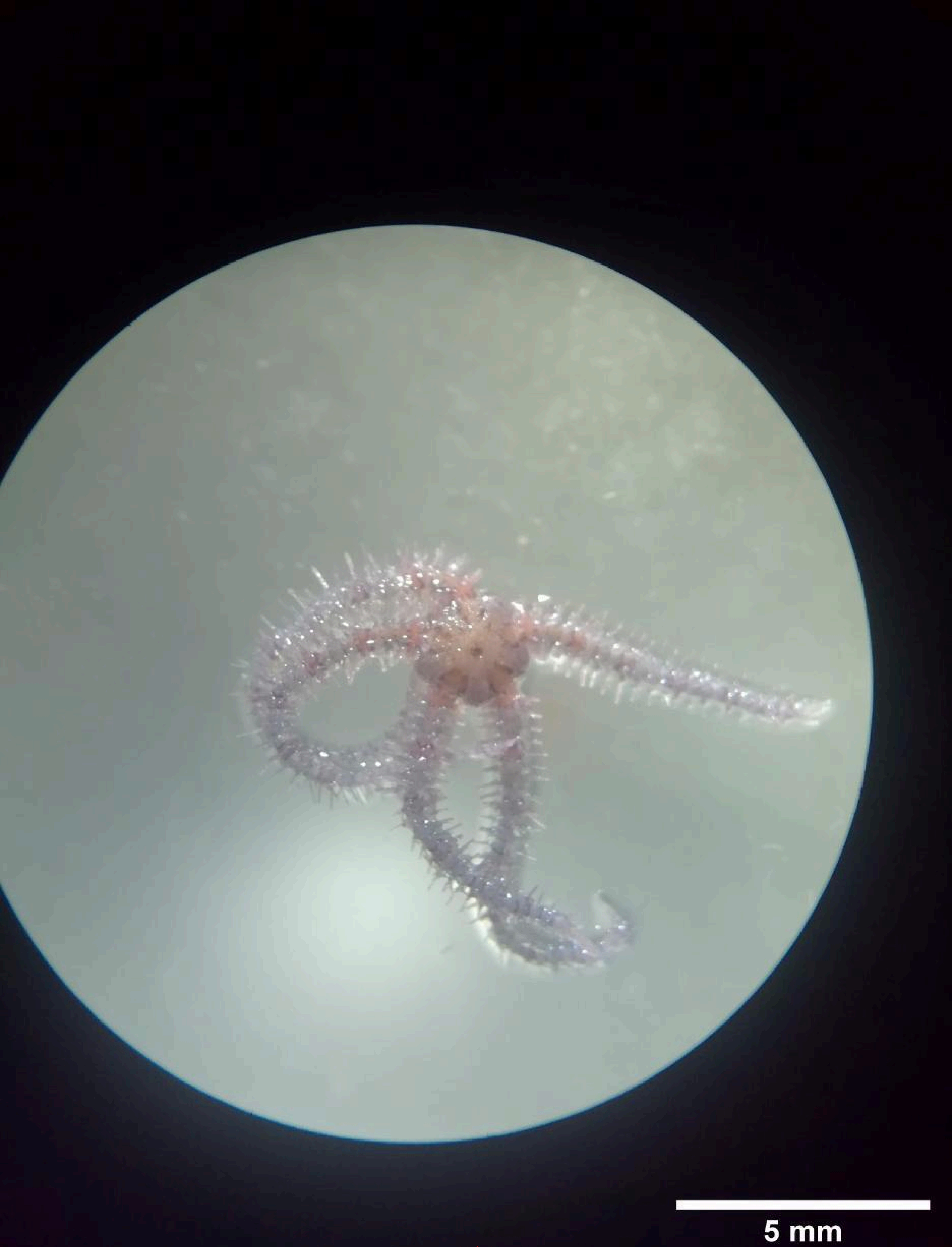
5 mm

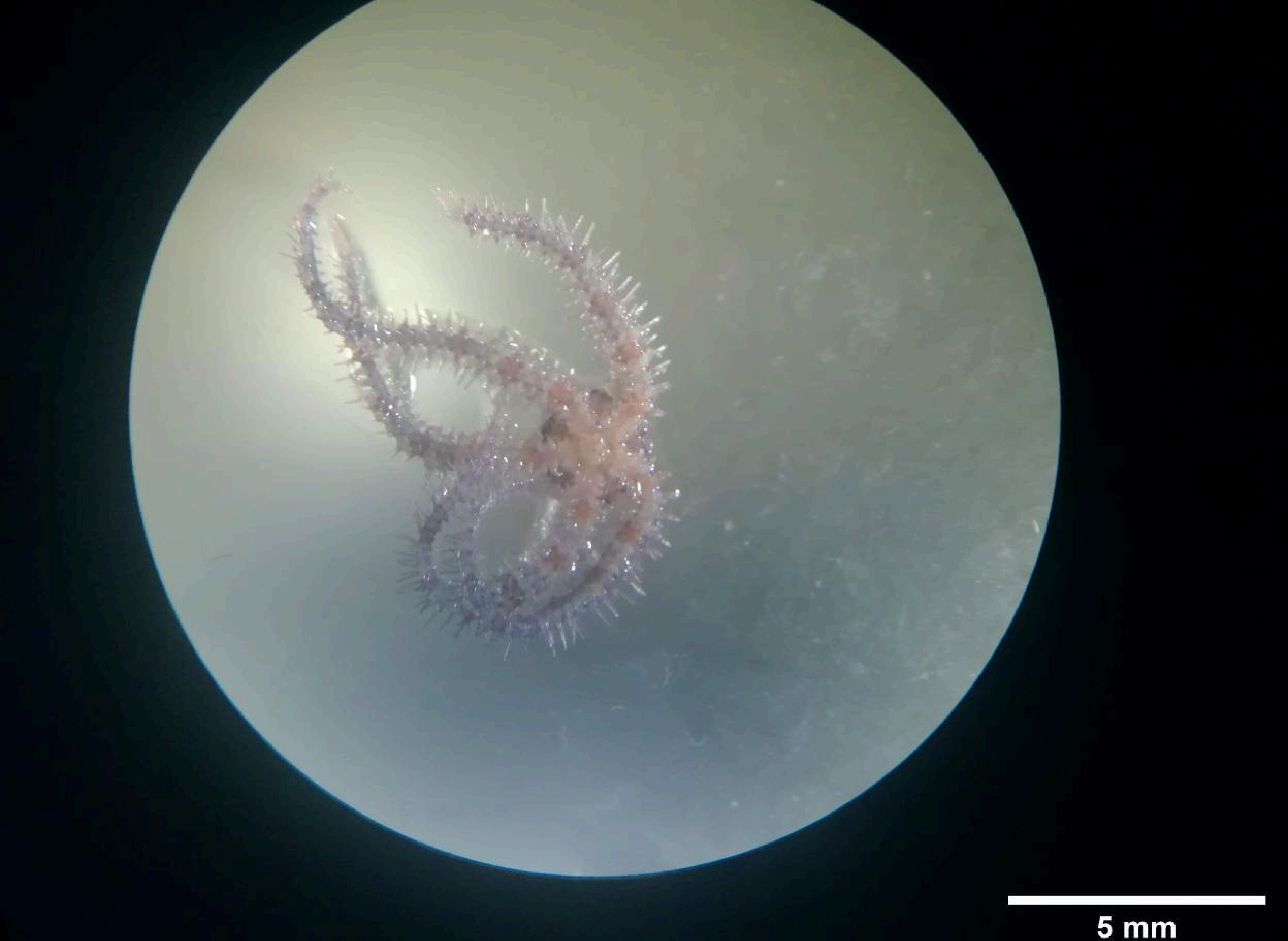
5 mm

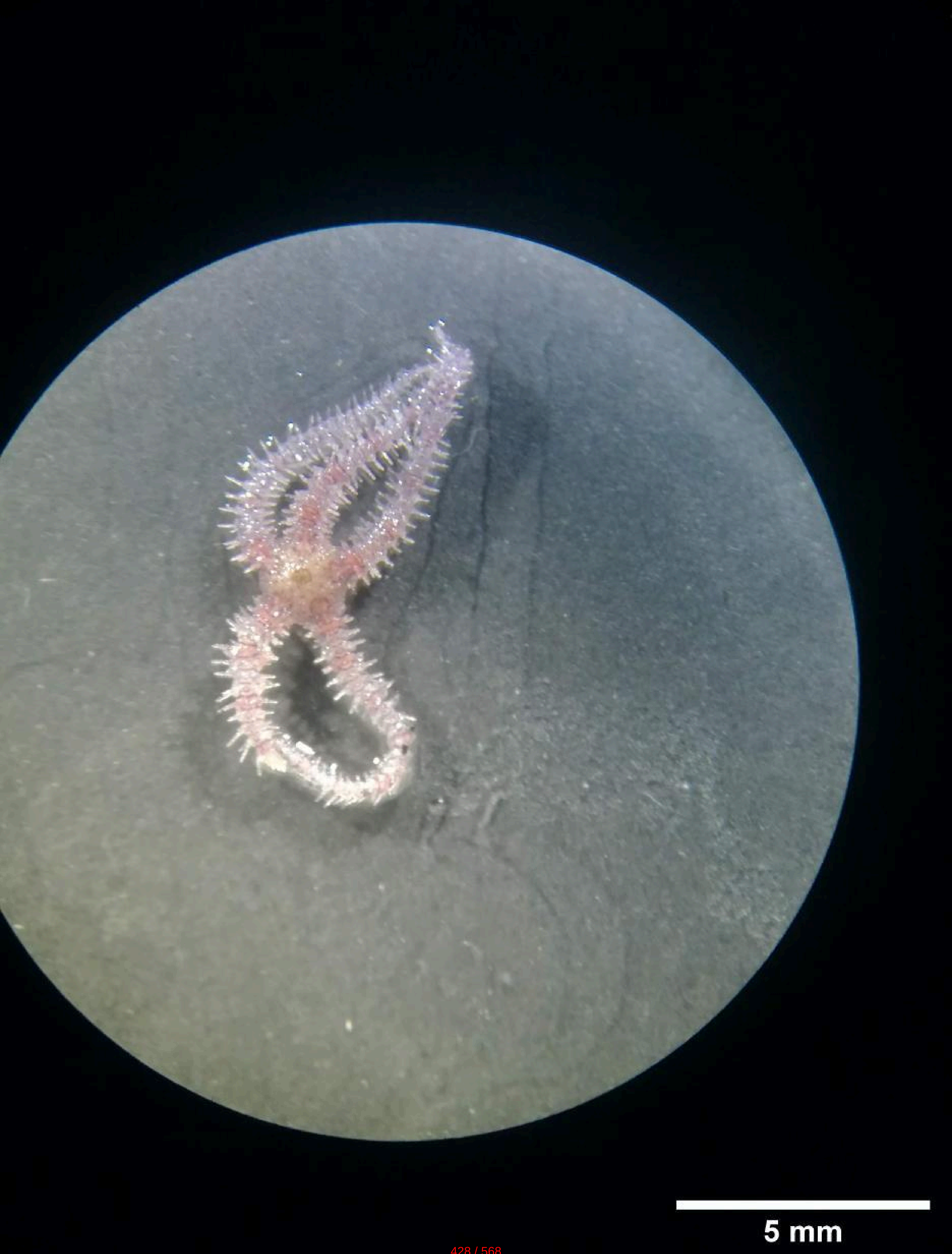
5 mm

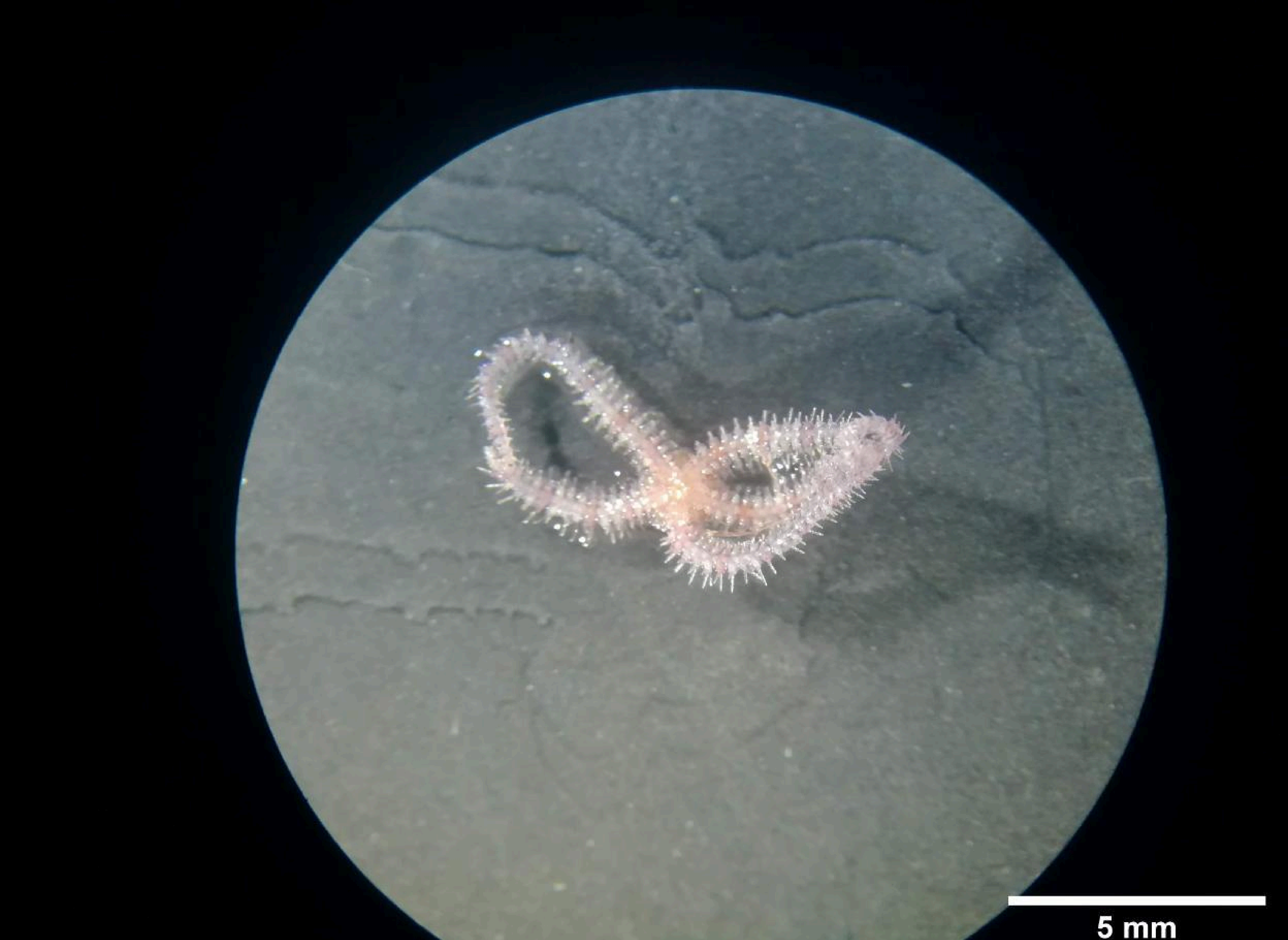
5 mm

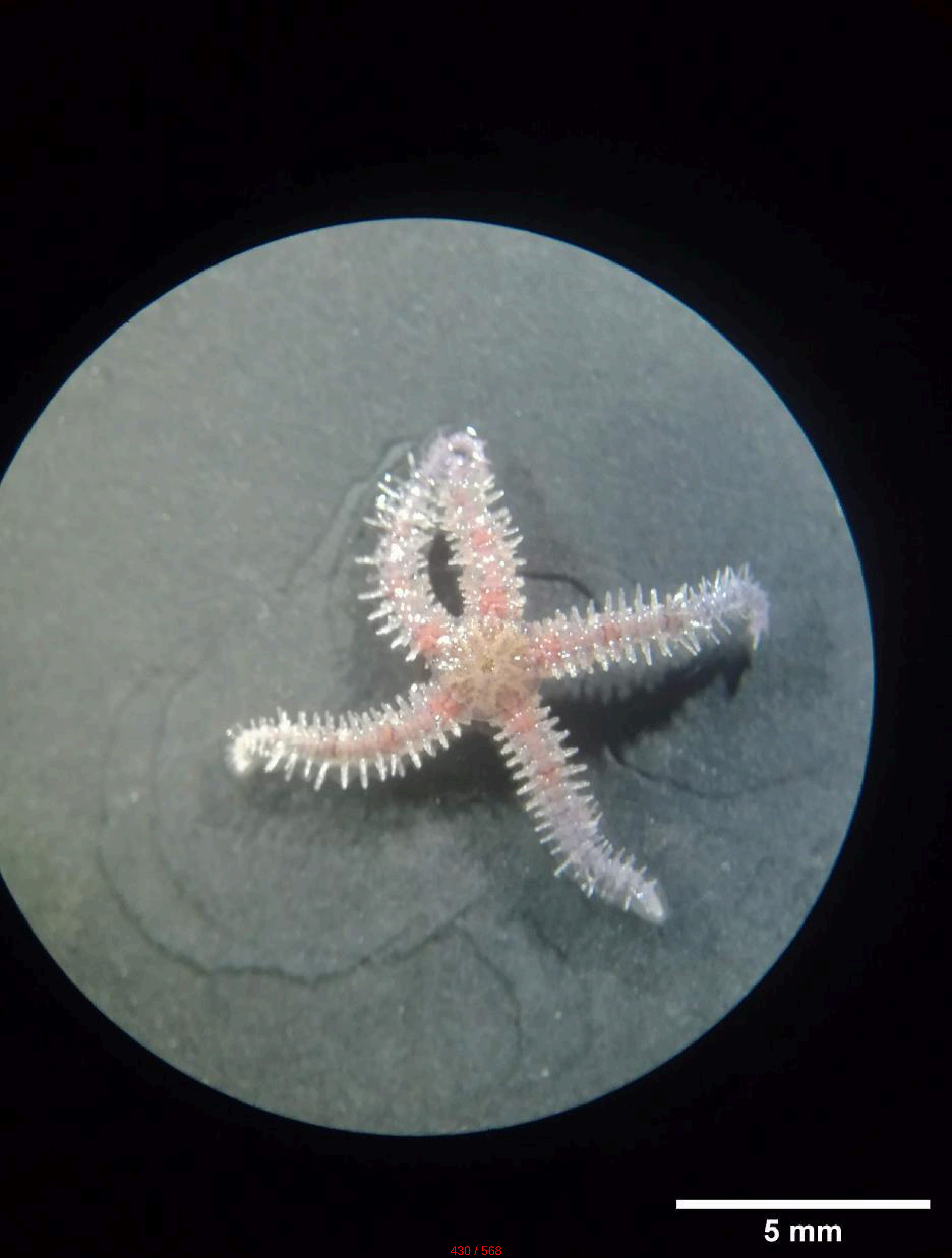
5 mm

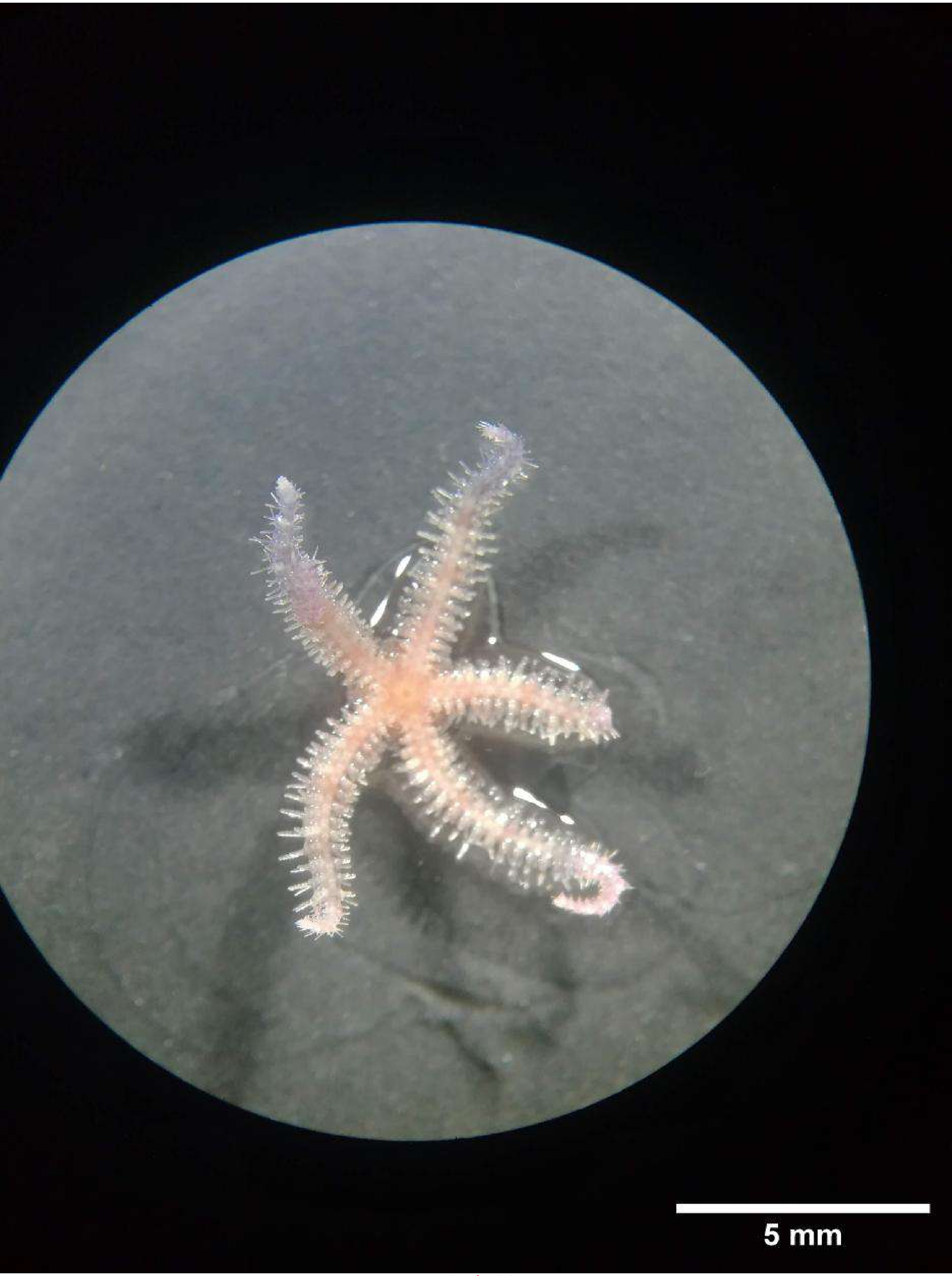
5 mm

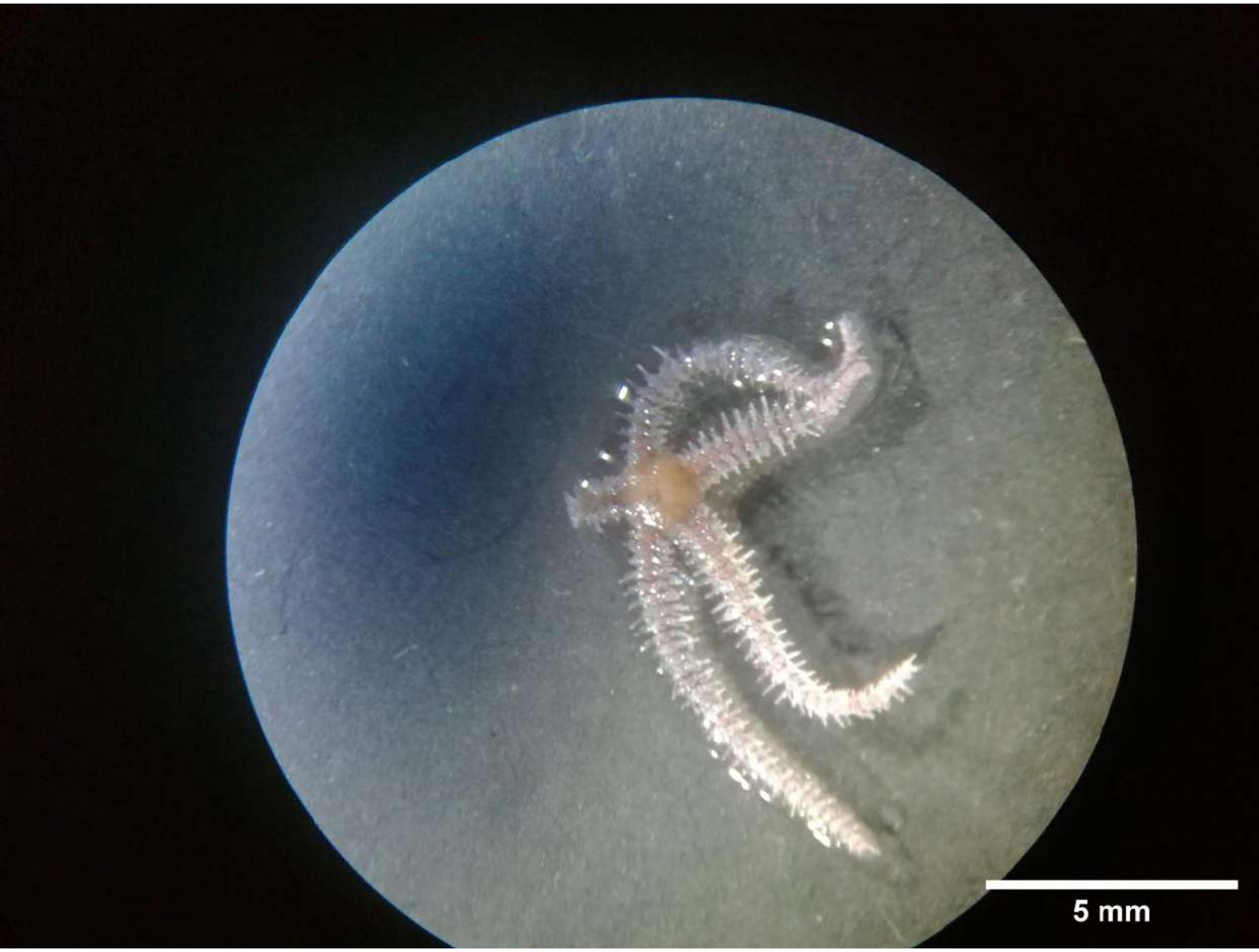
5 mm

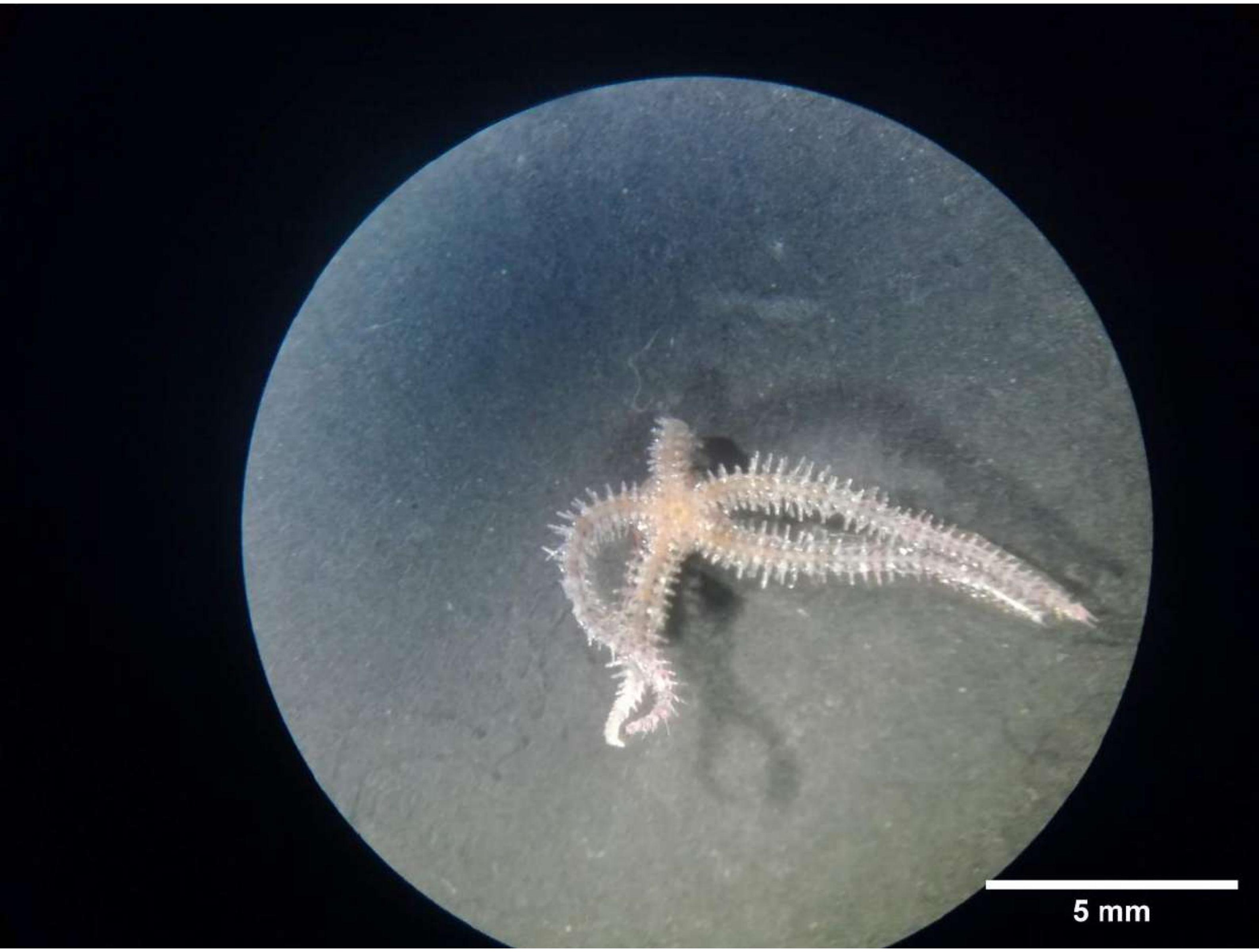
5 mm

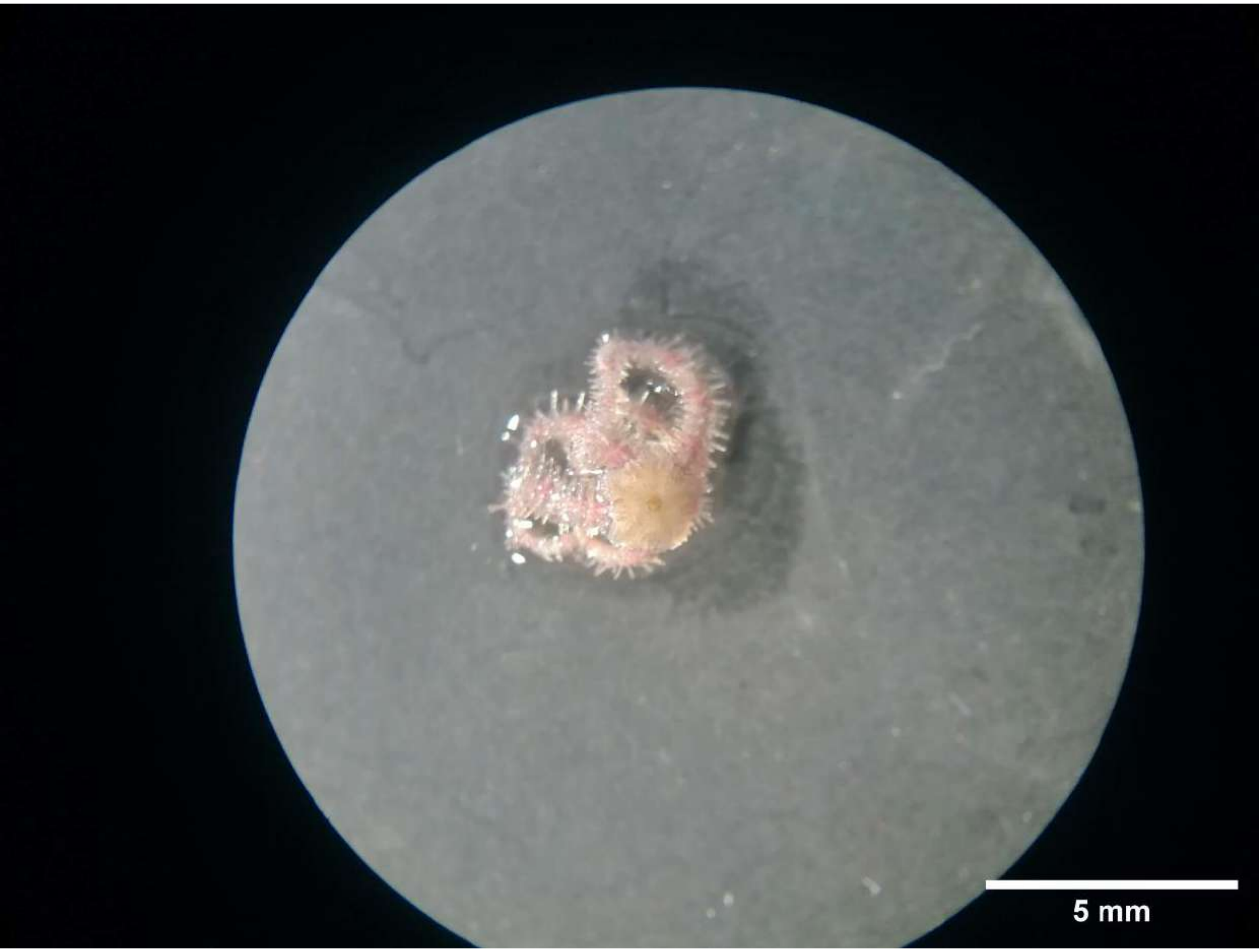
5 mm

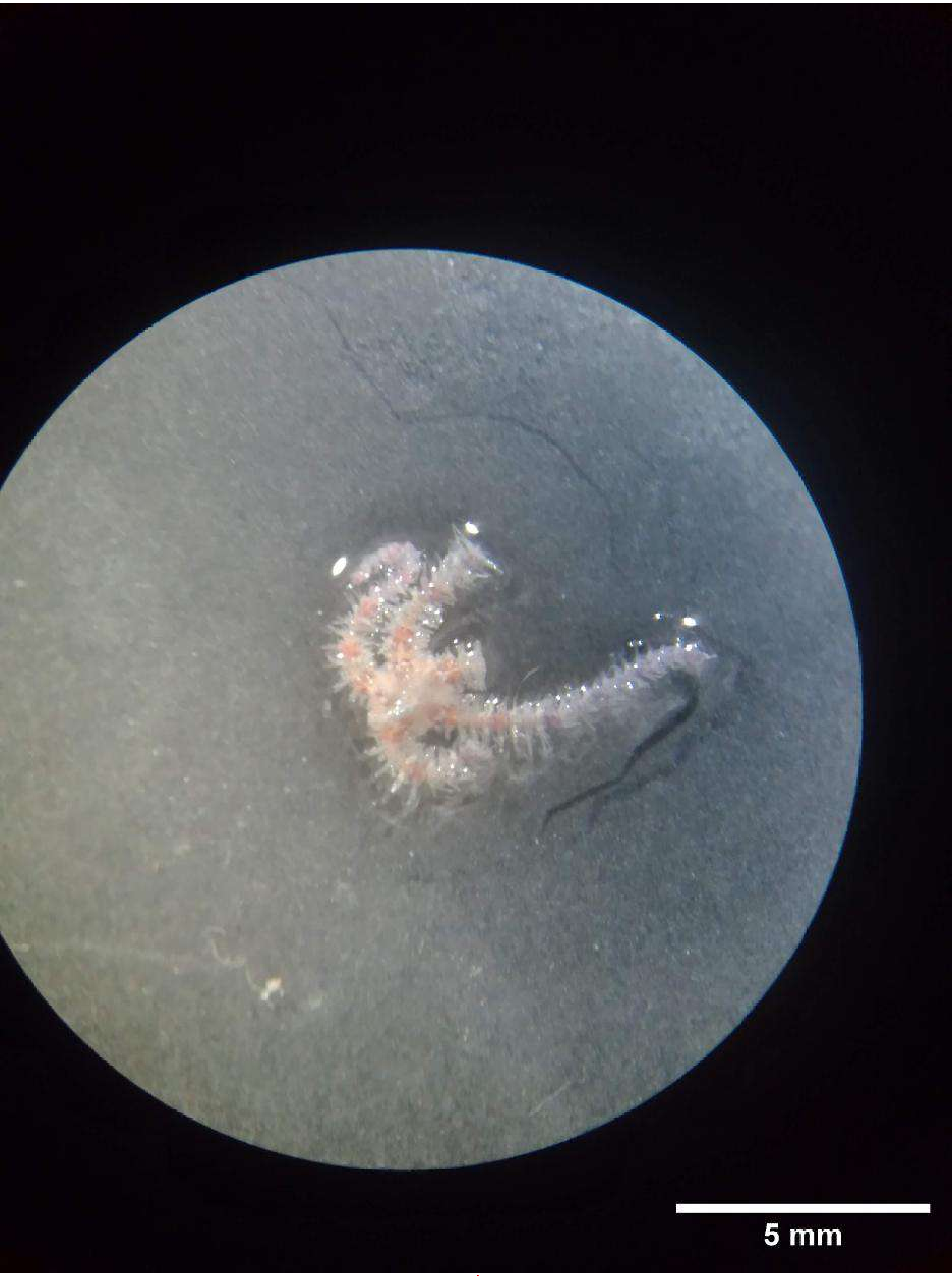
5 mm

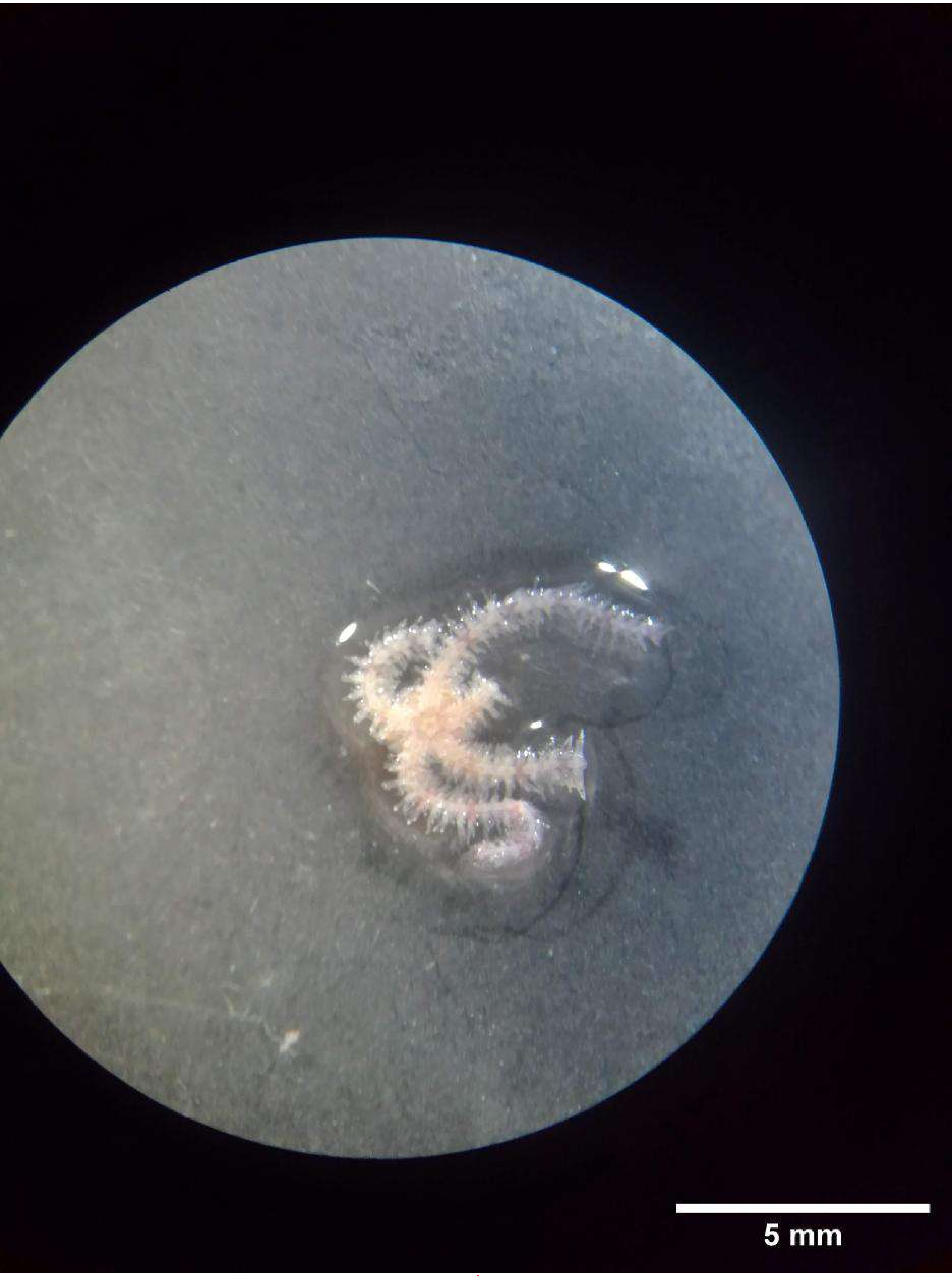
5 mm

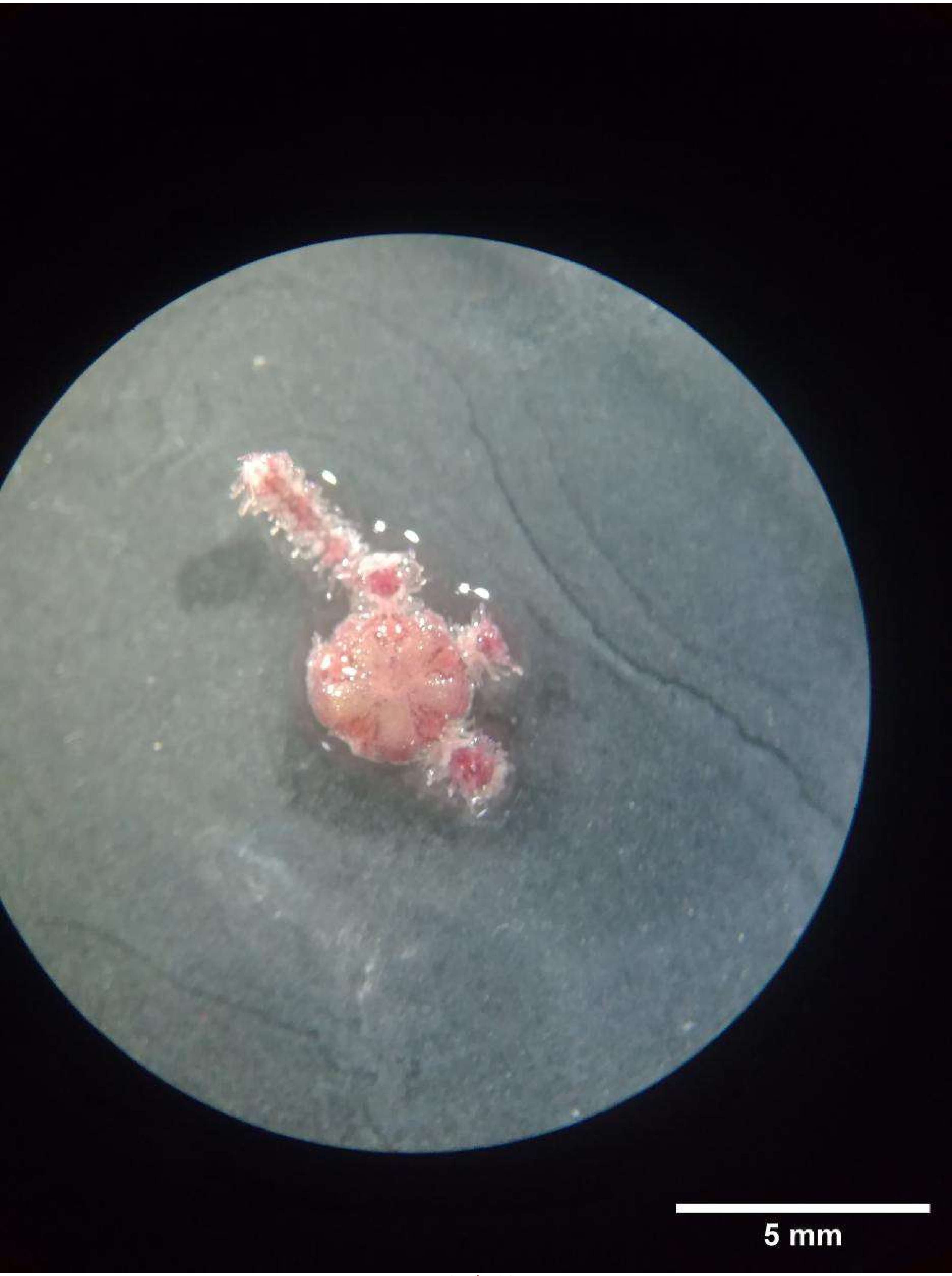
5 mm

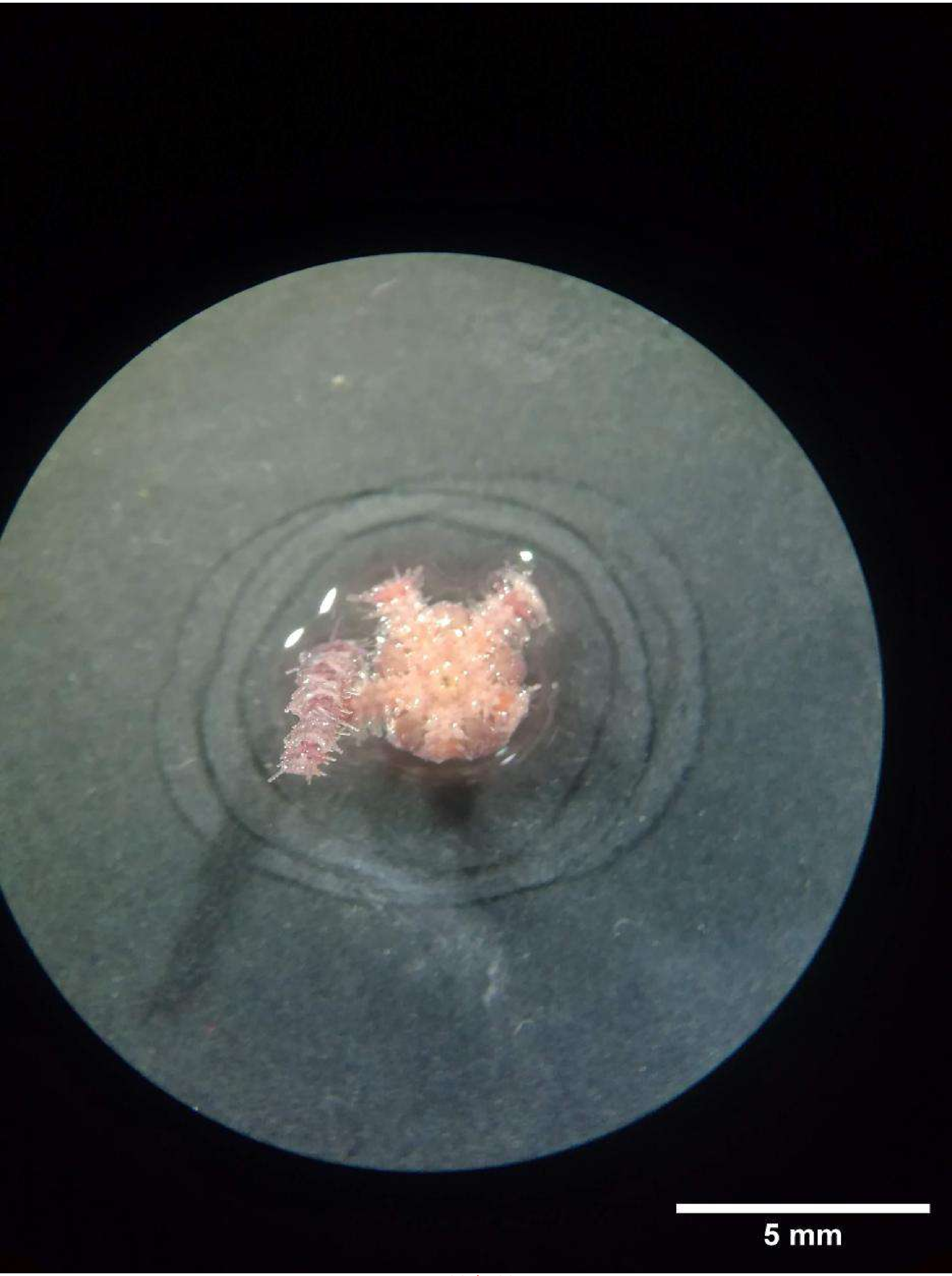
5 mm

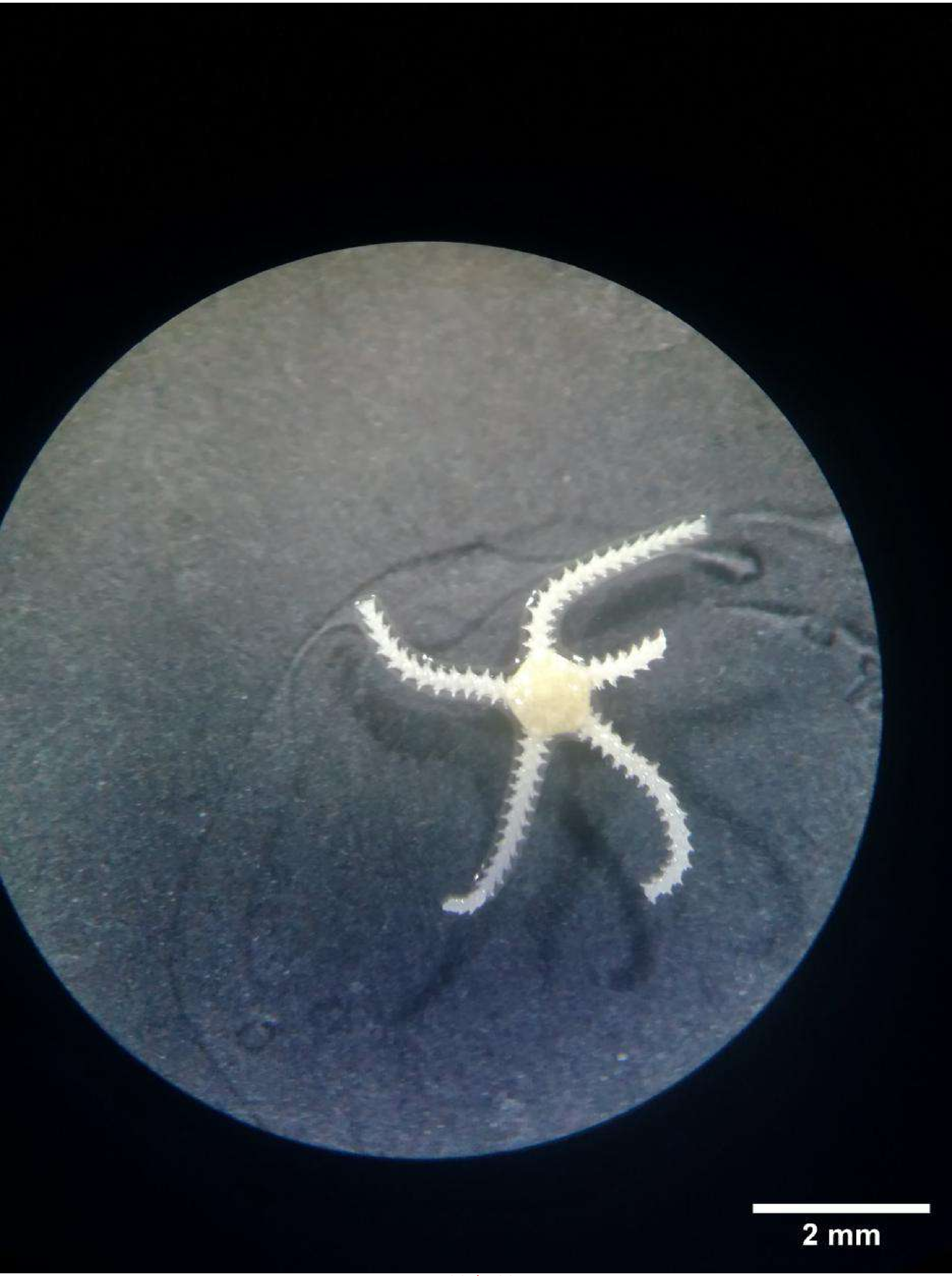
2 mm

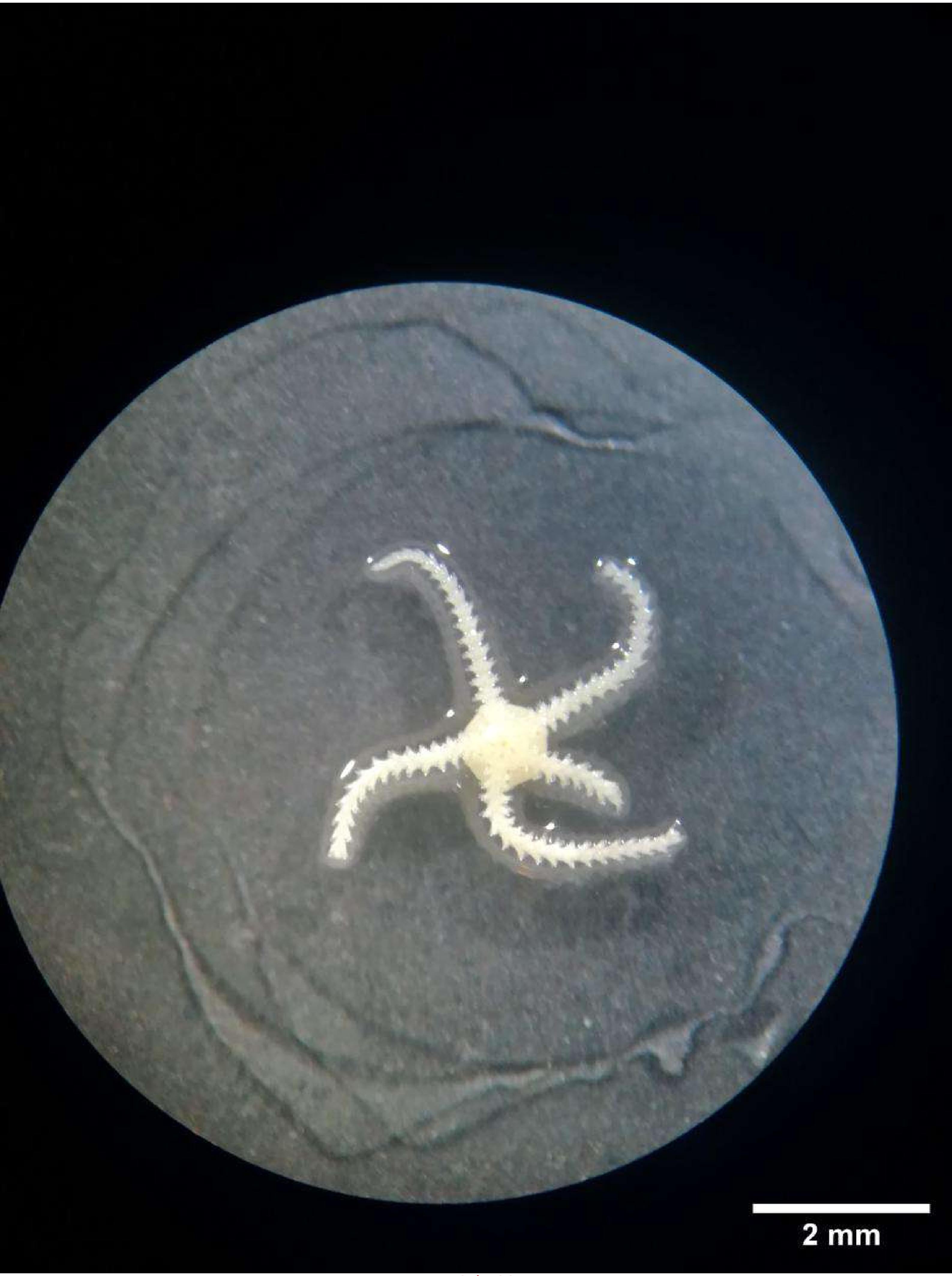
2 mm

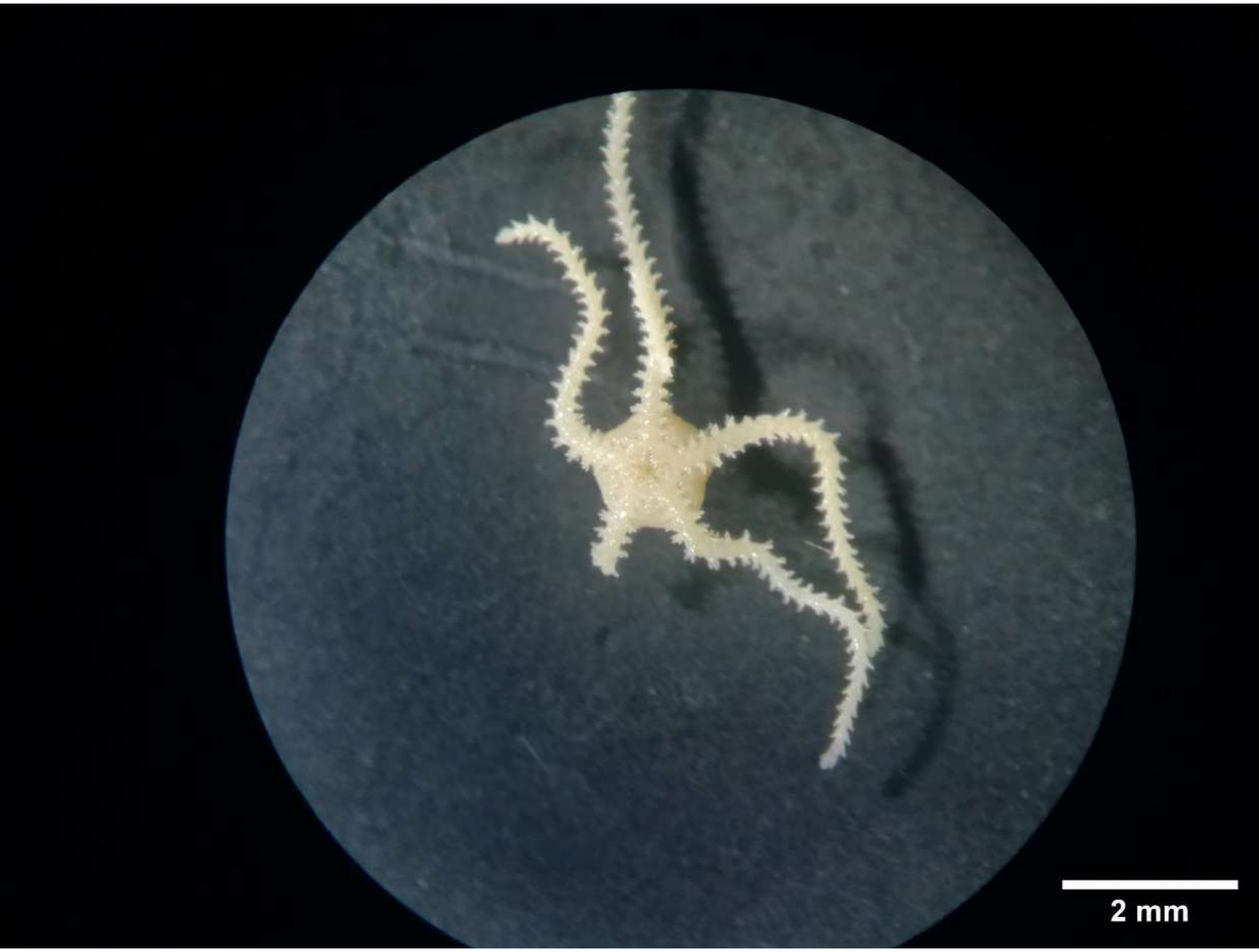
2 mm

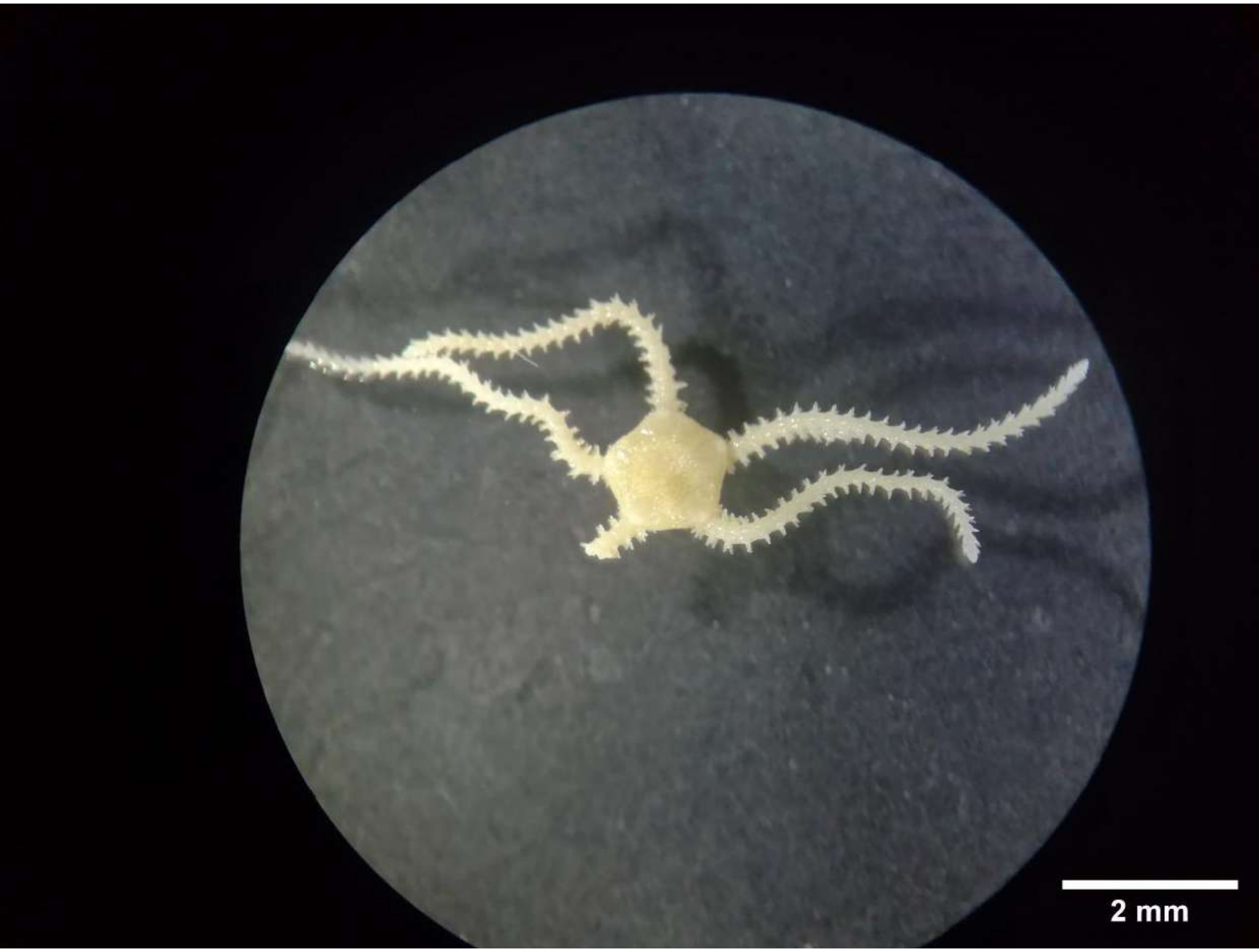
2 mm

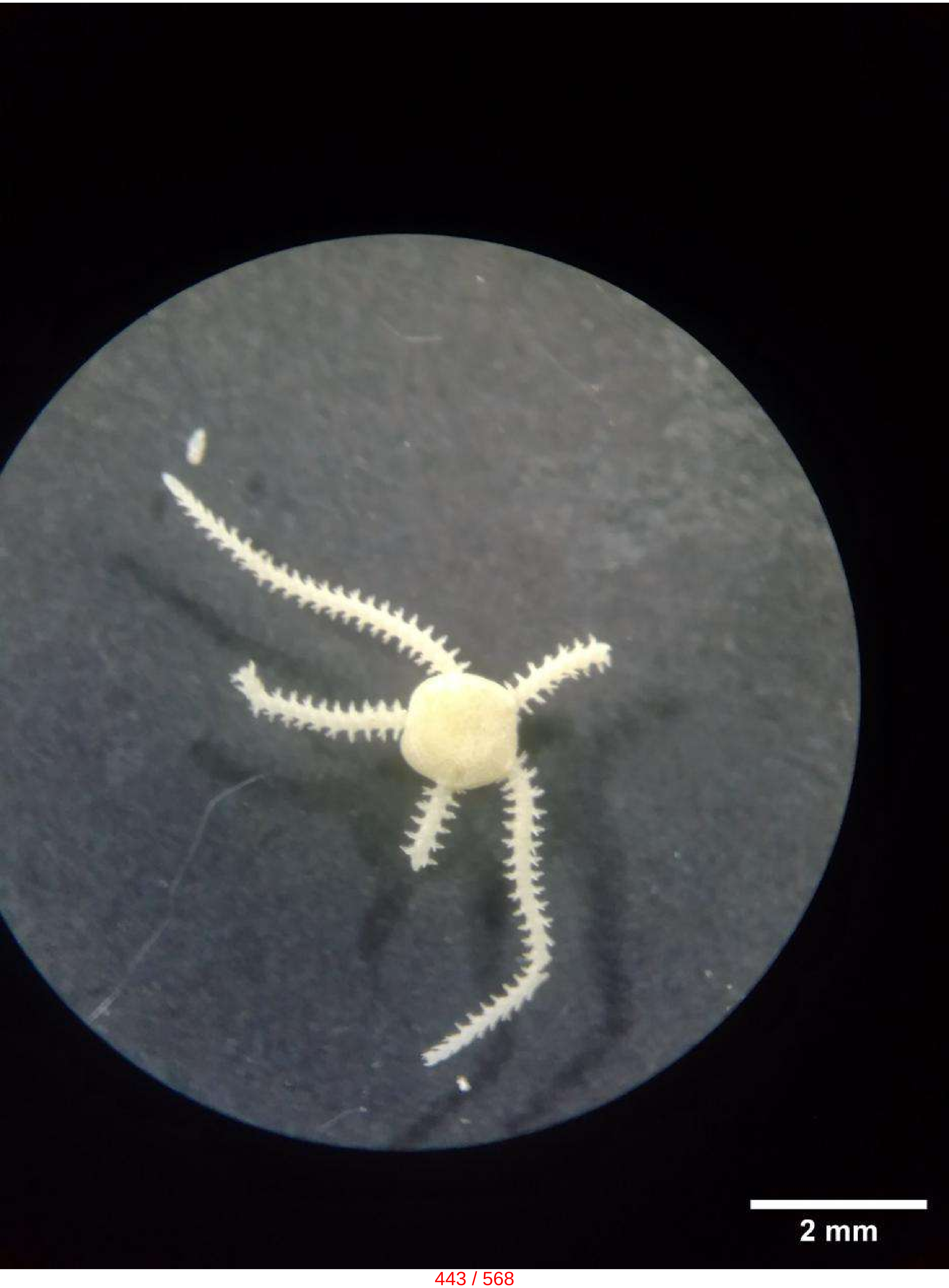
2 mm

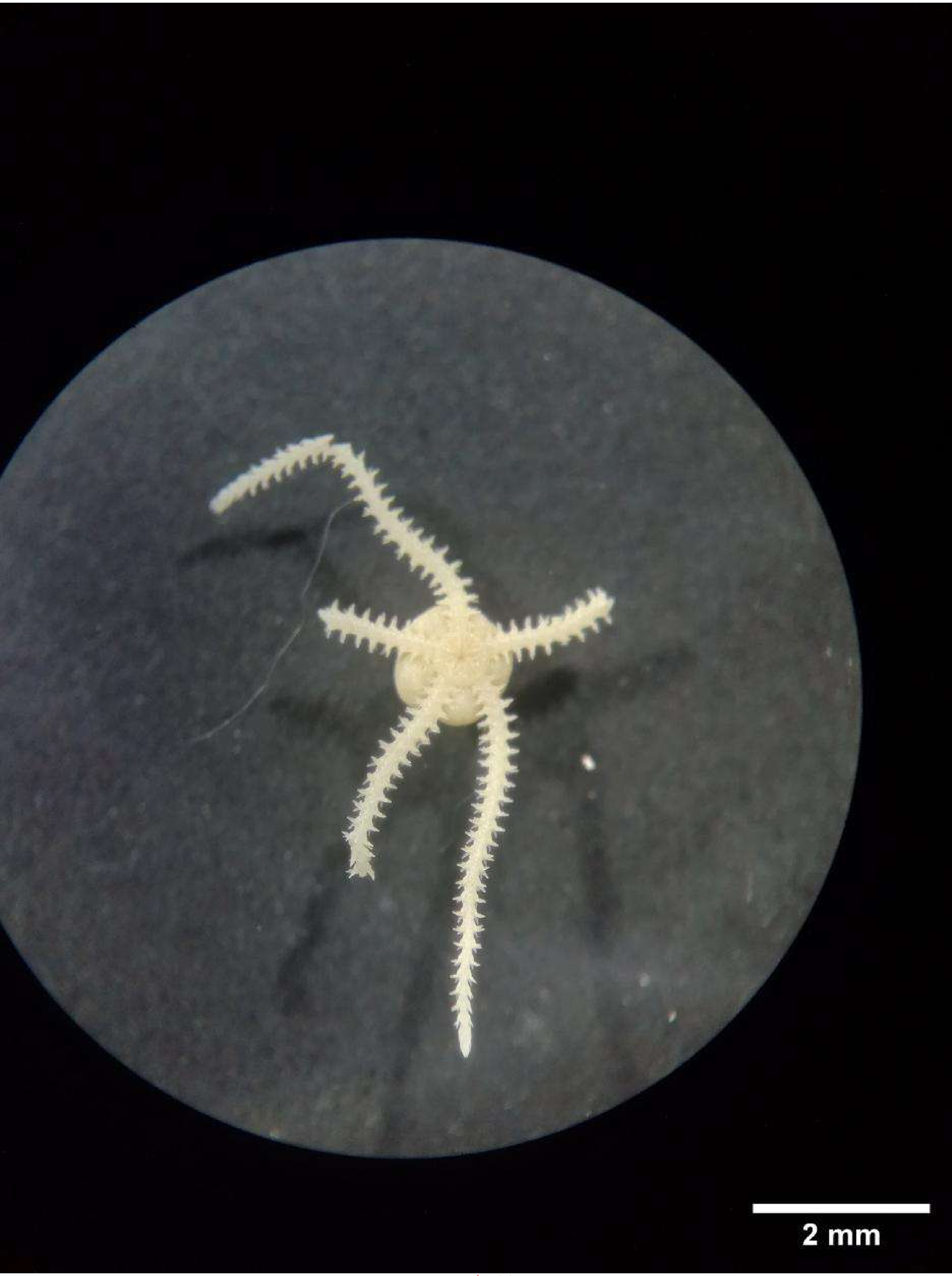
2 mm

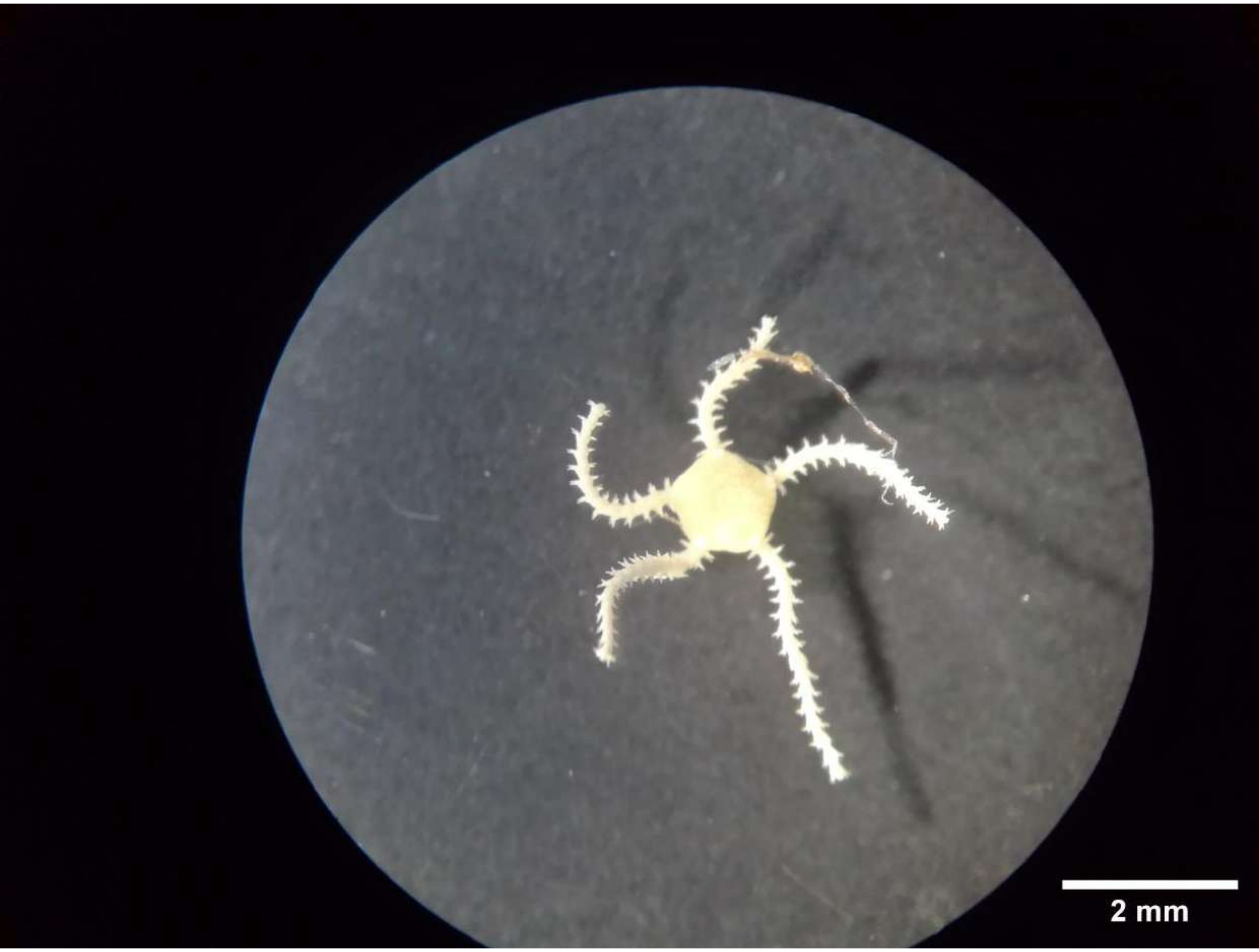
2 mm

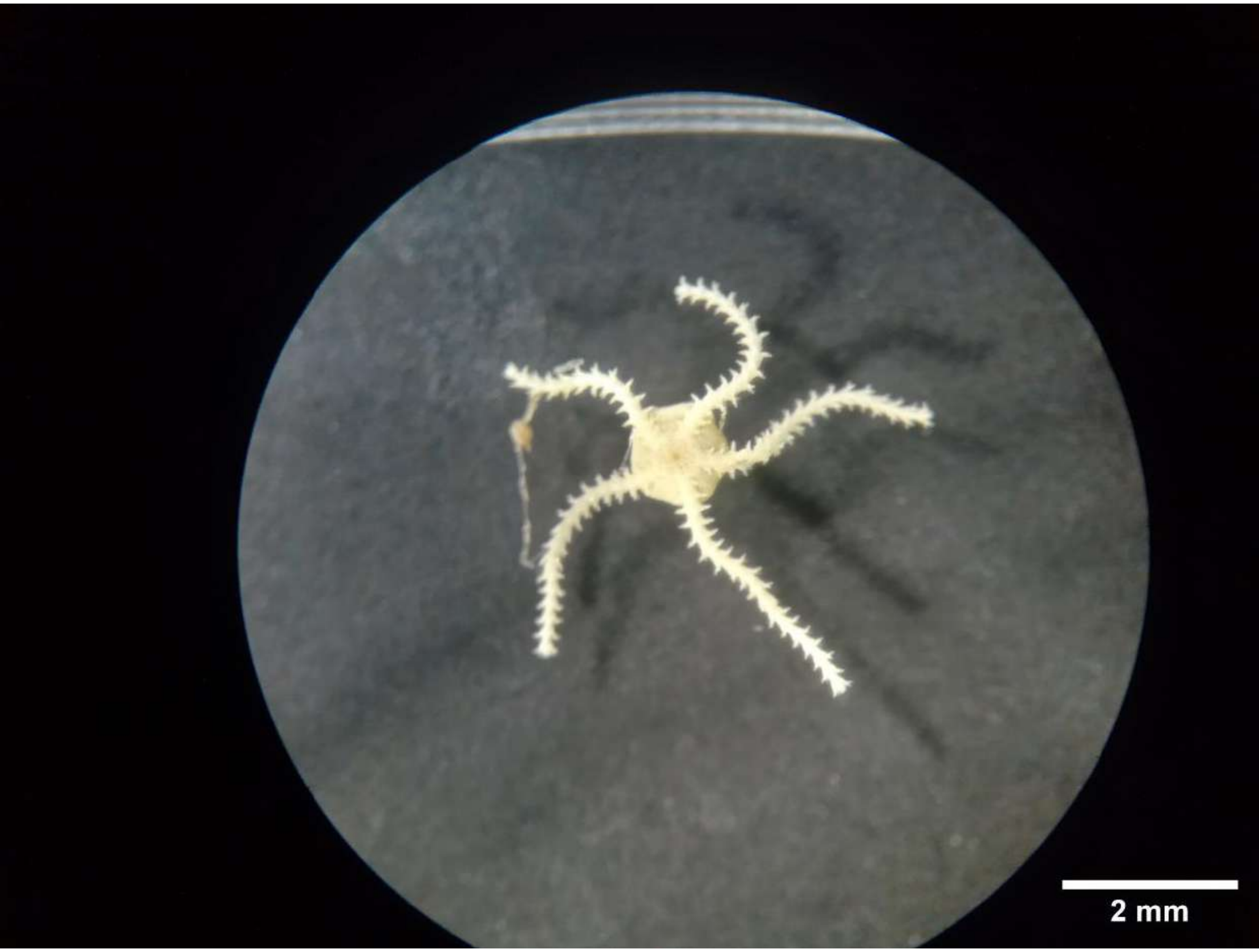
2 mm

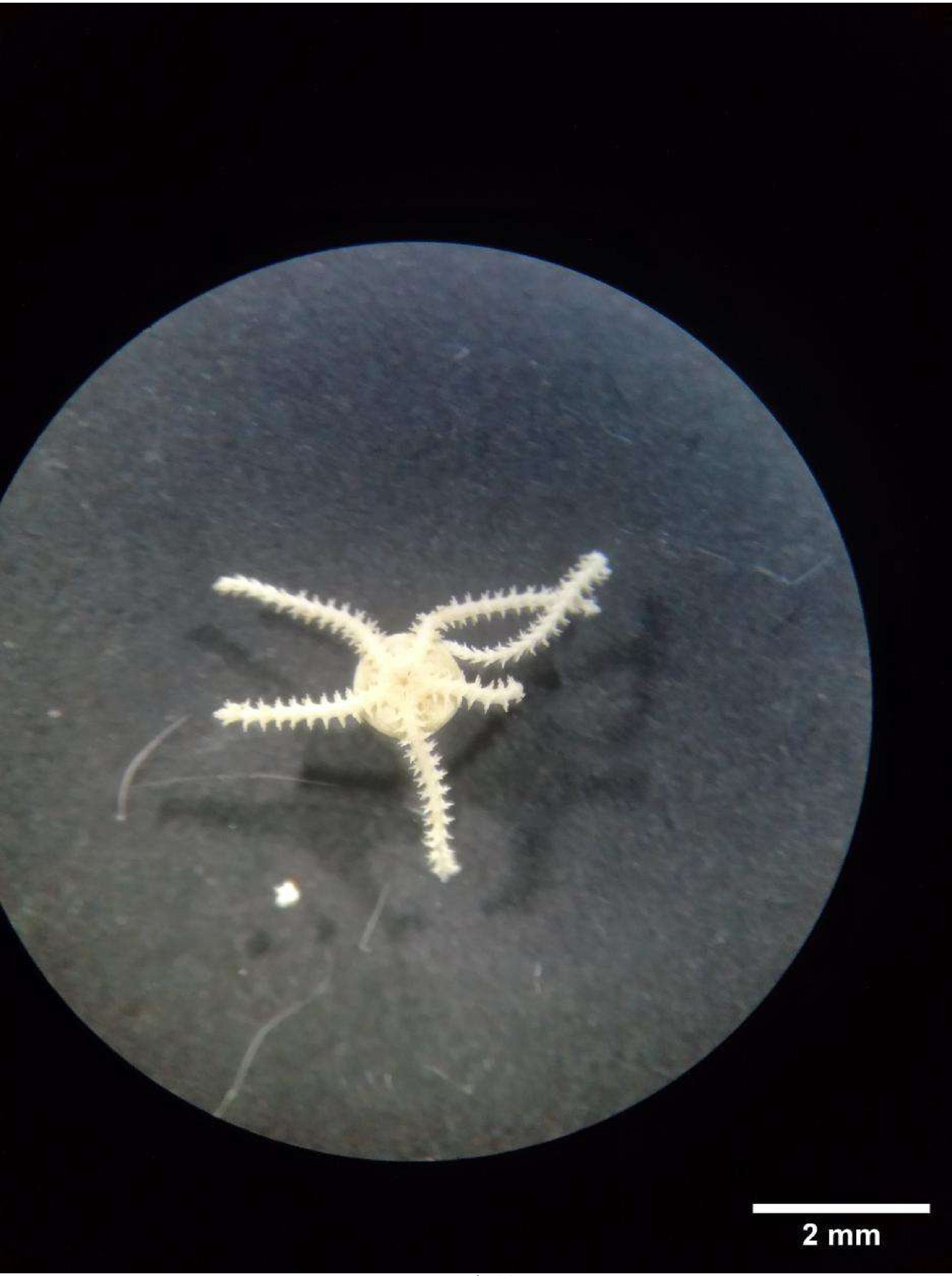
2 mm

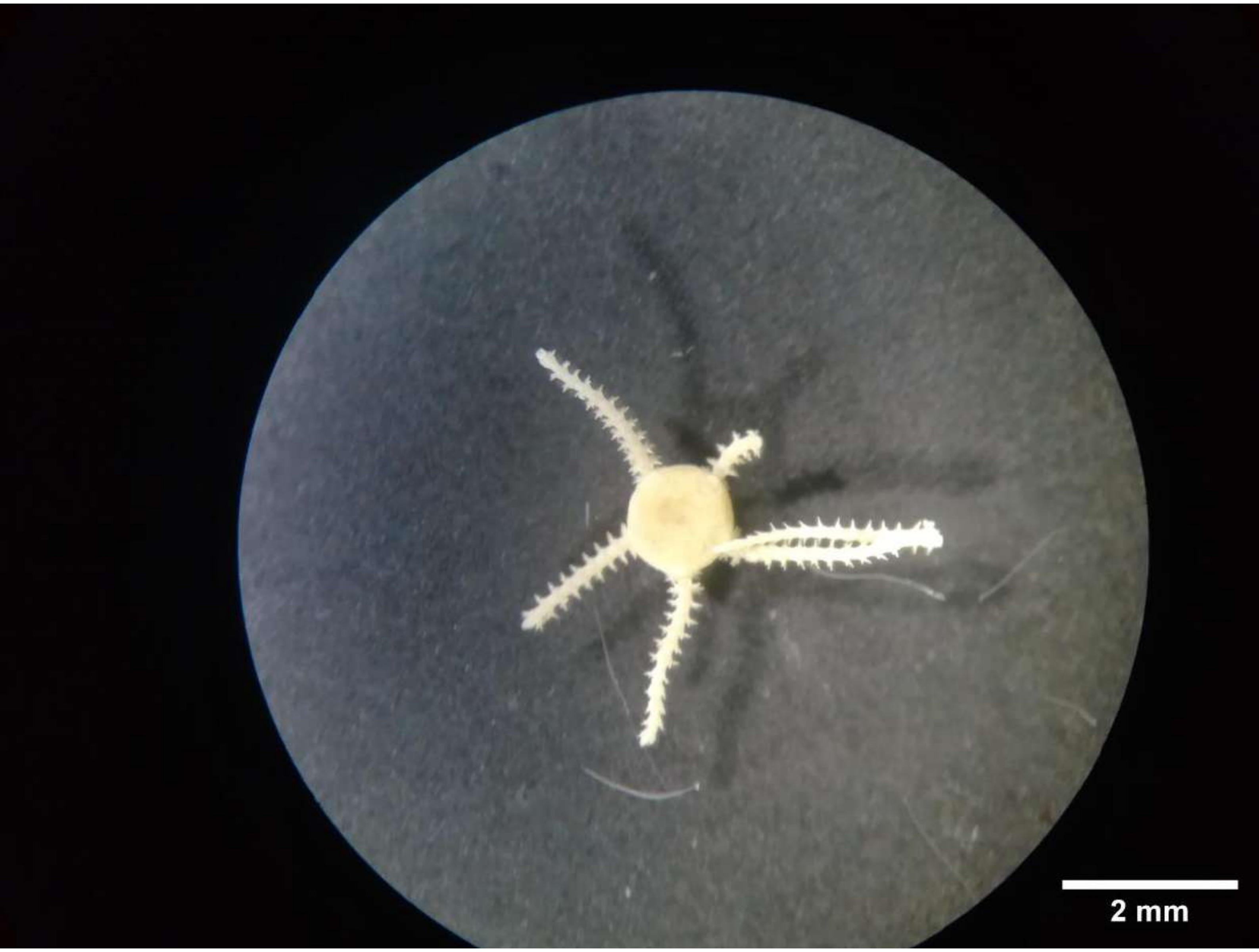
2 mm

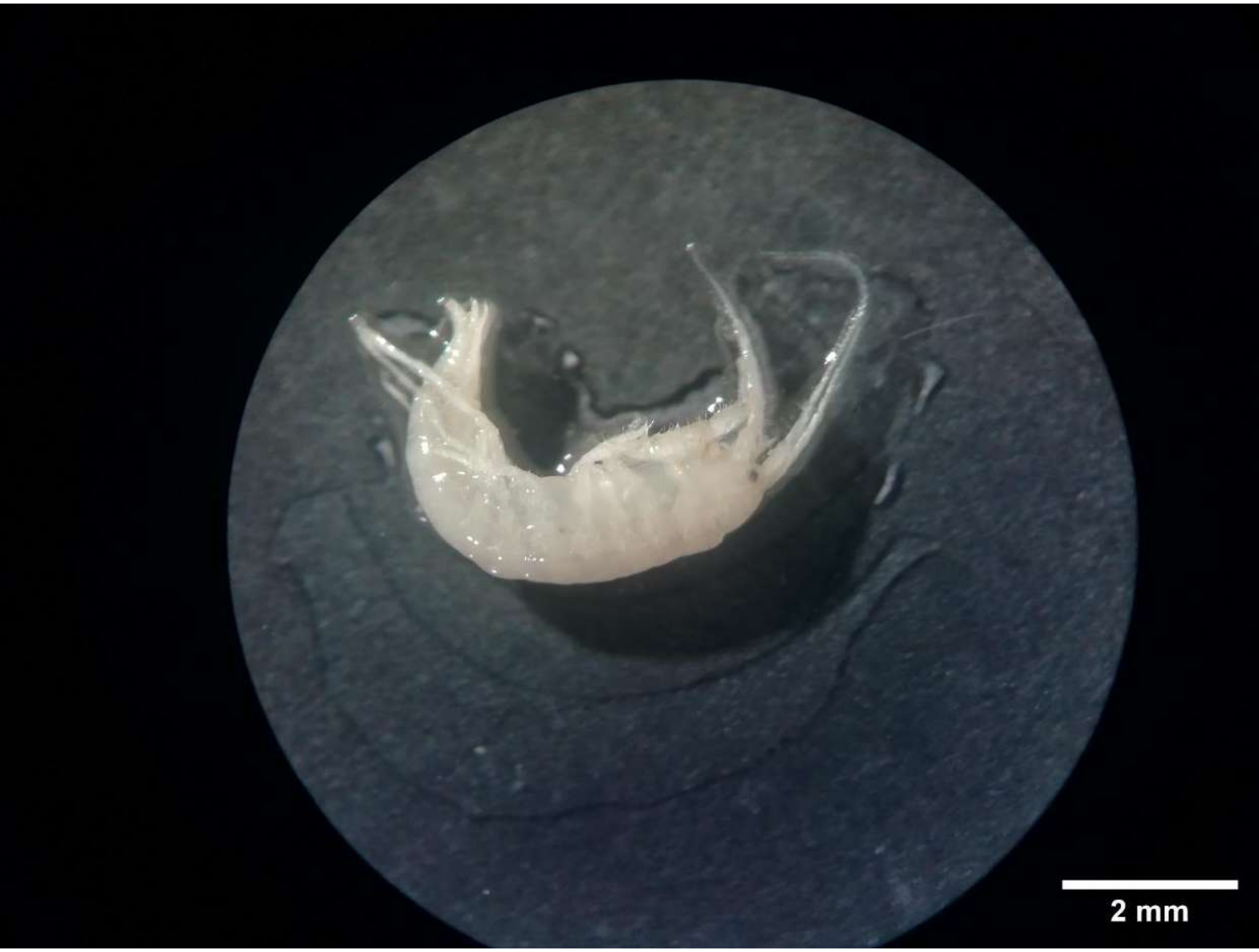
2 mm

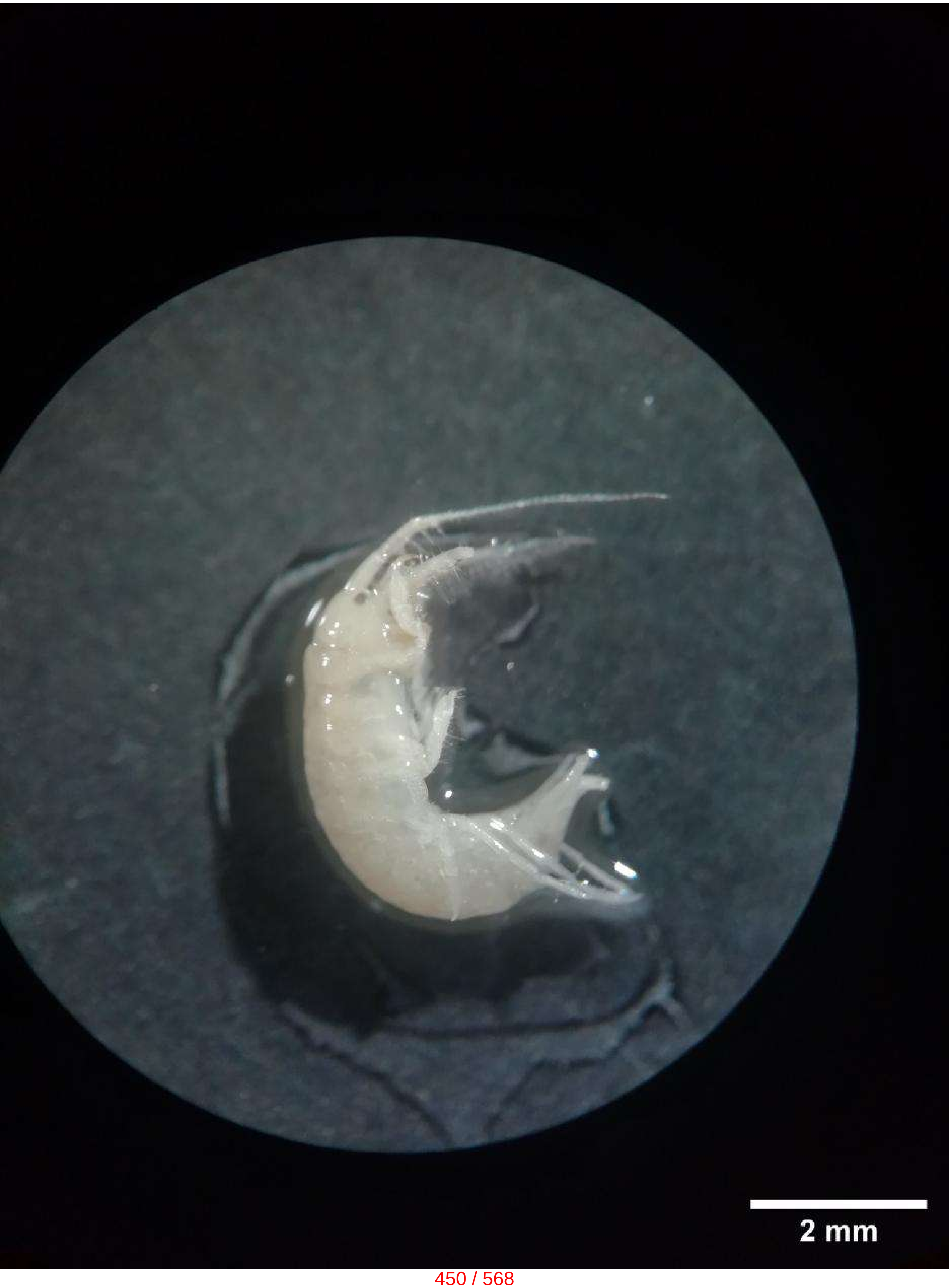
2 mm

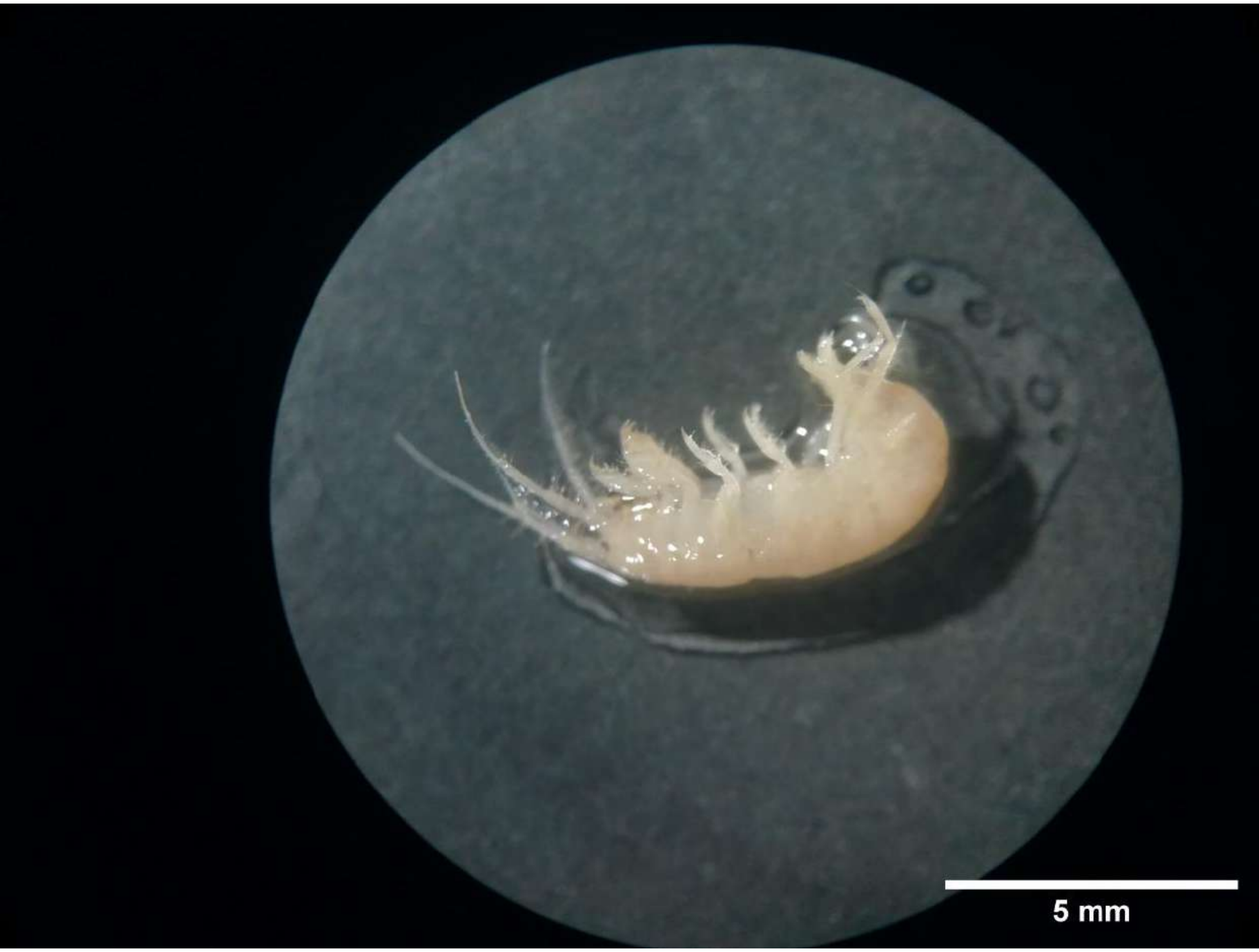
5 mm

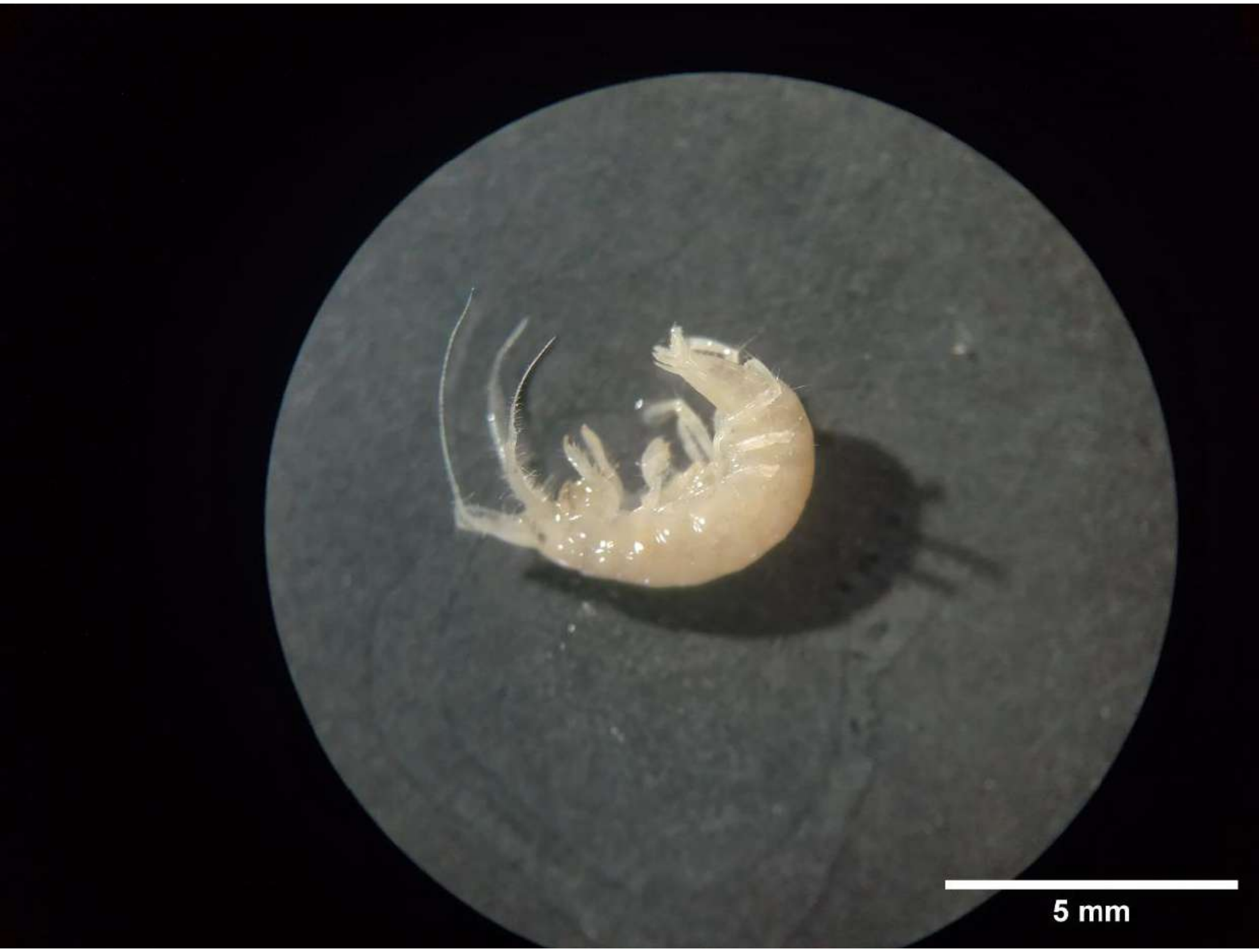
5 mm

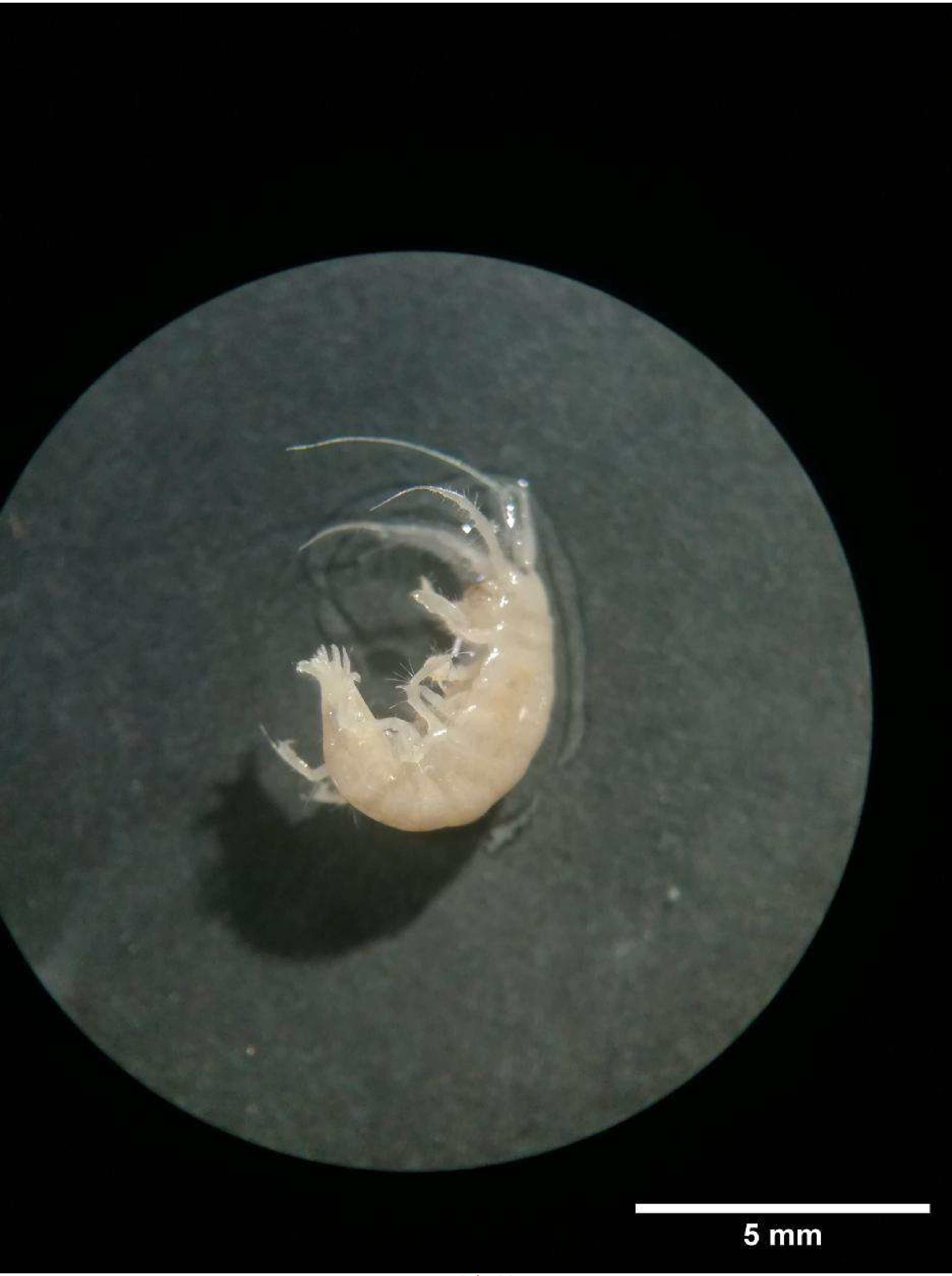
5 mm

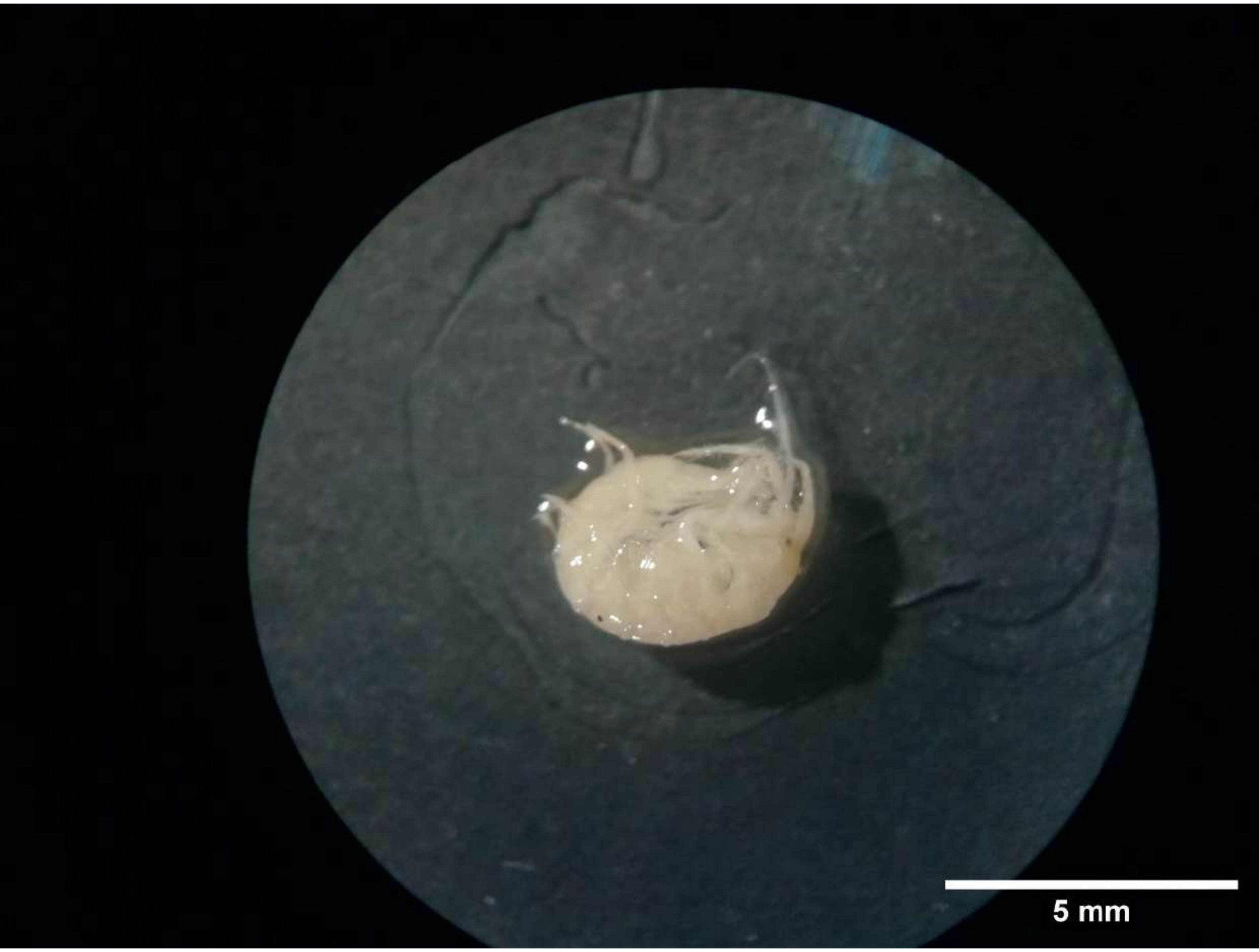
5 mm

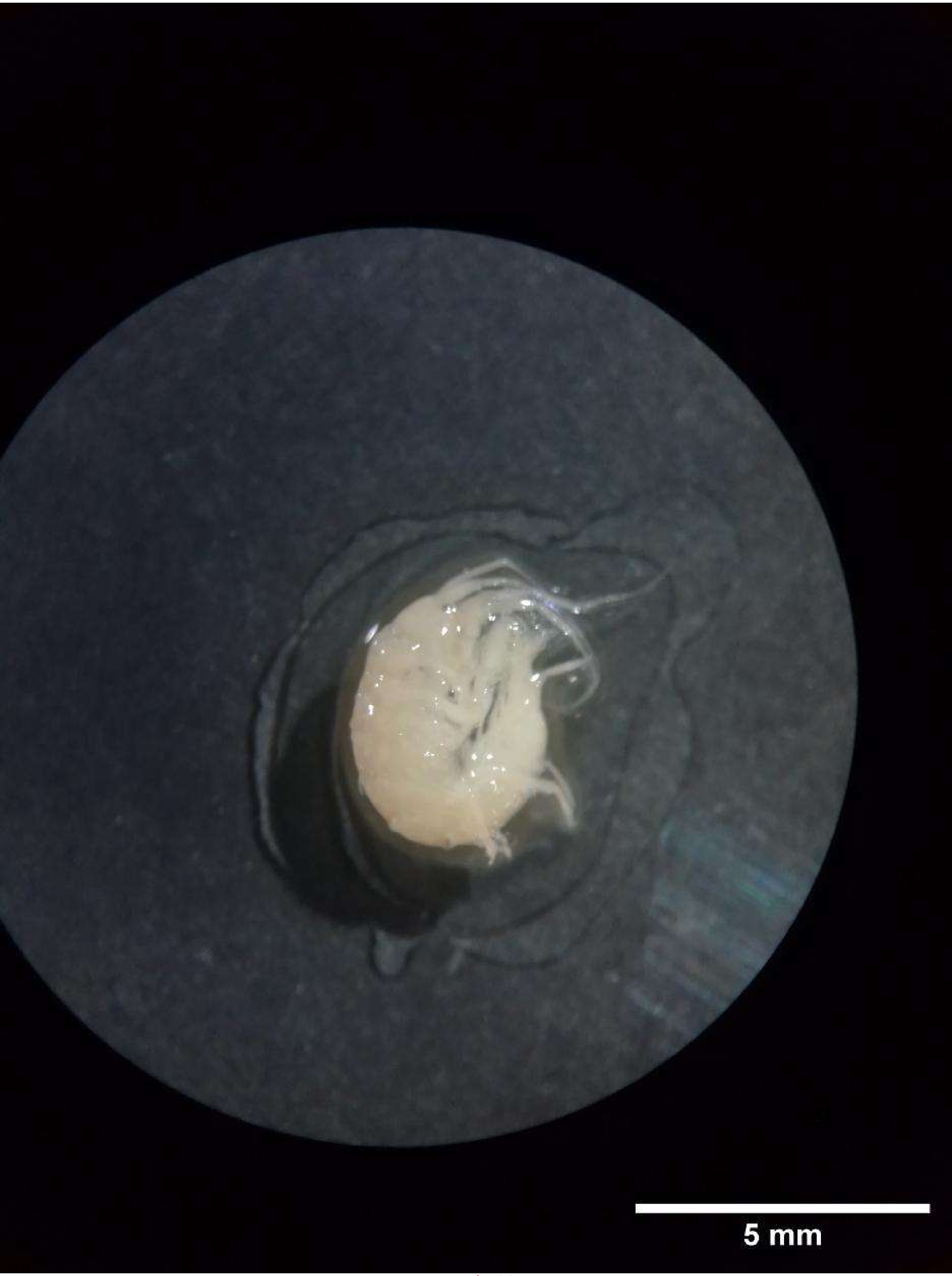
5 mm

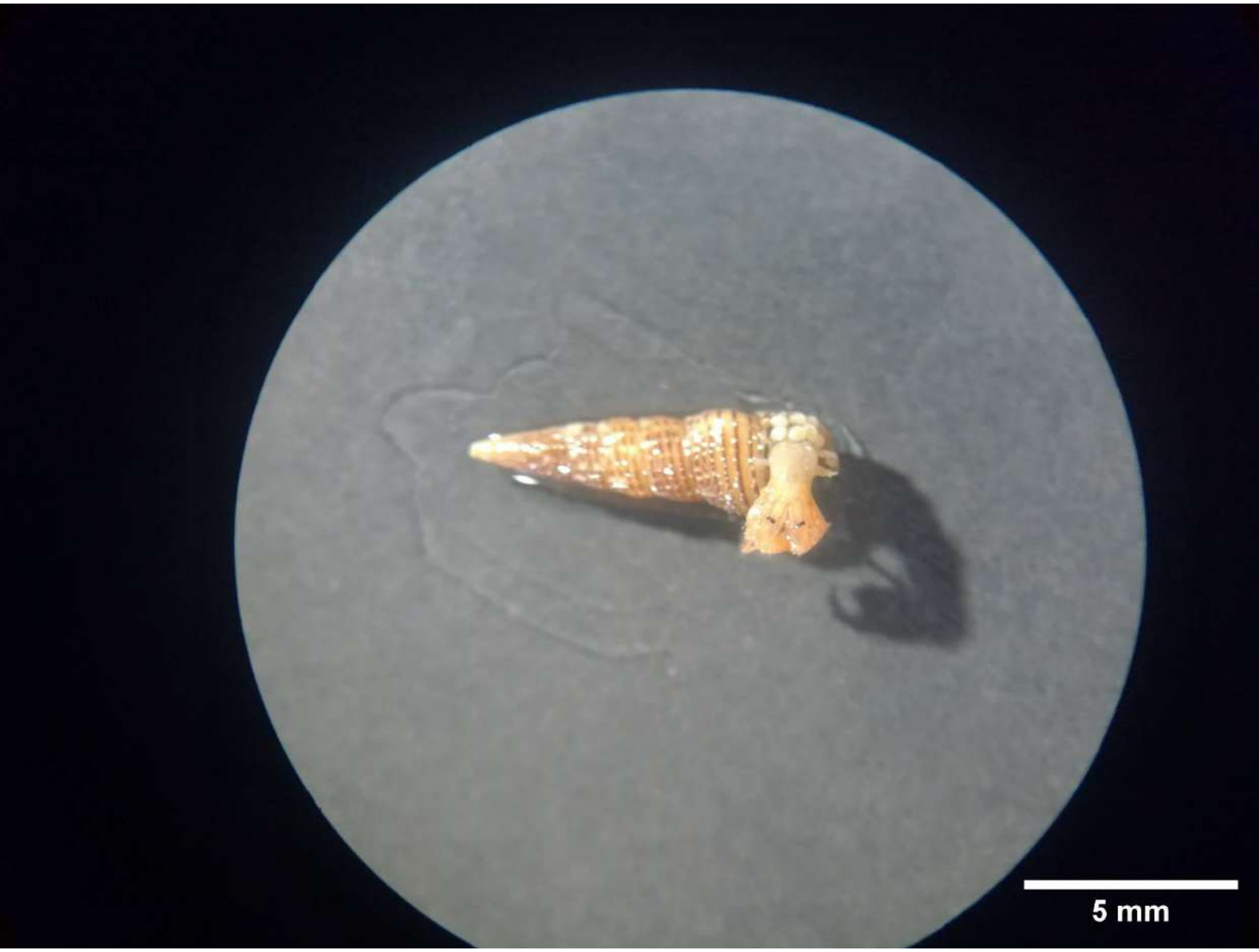
5 mm

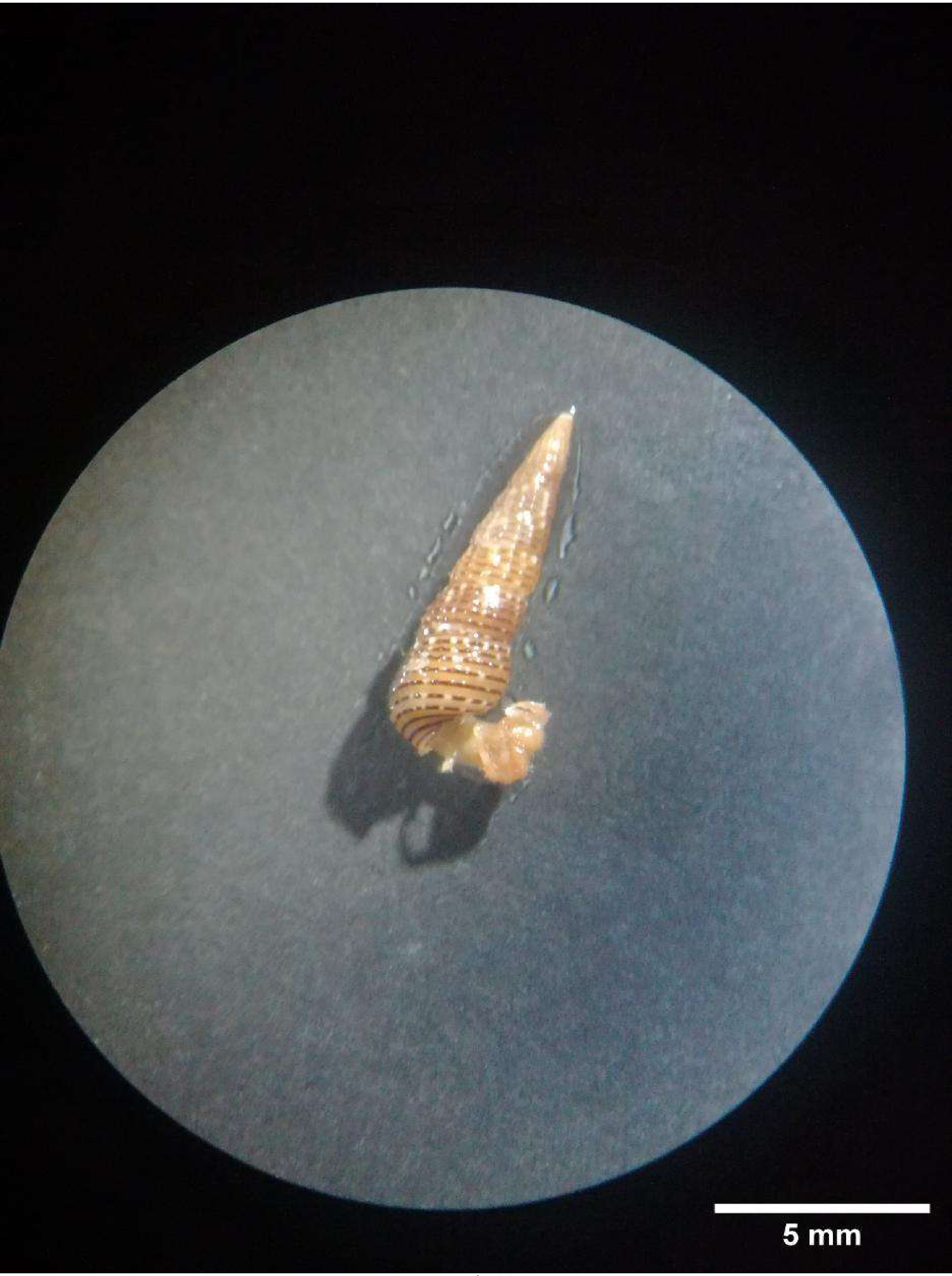
5 mm

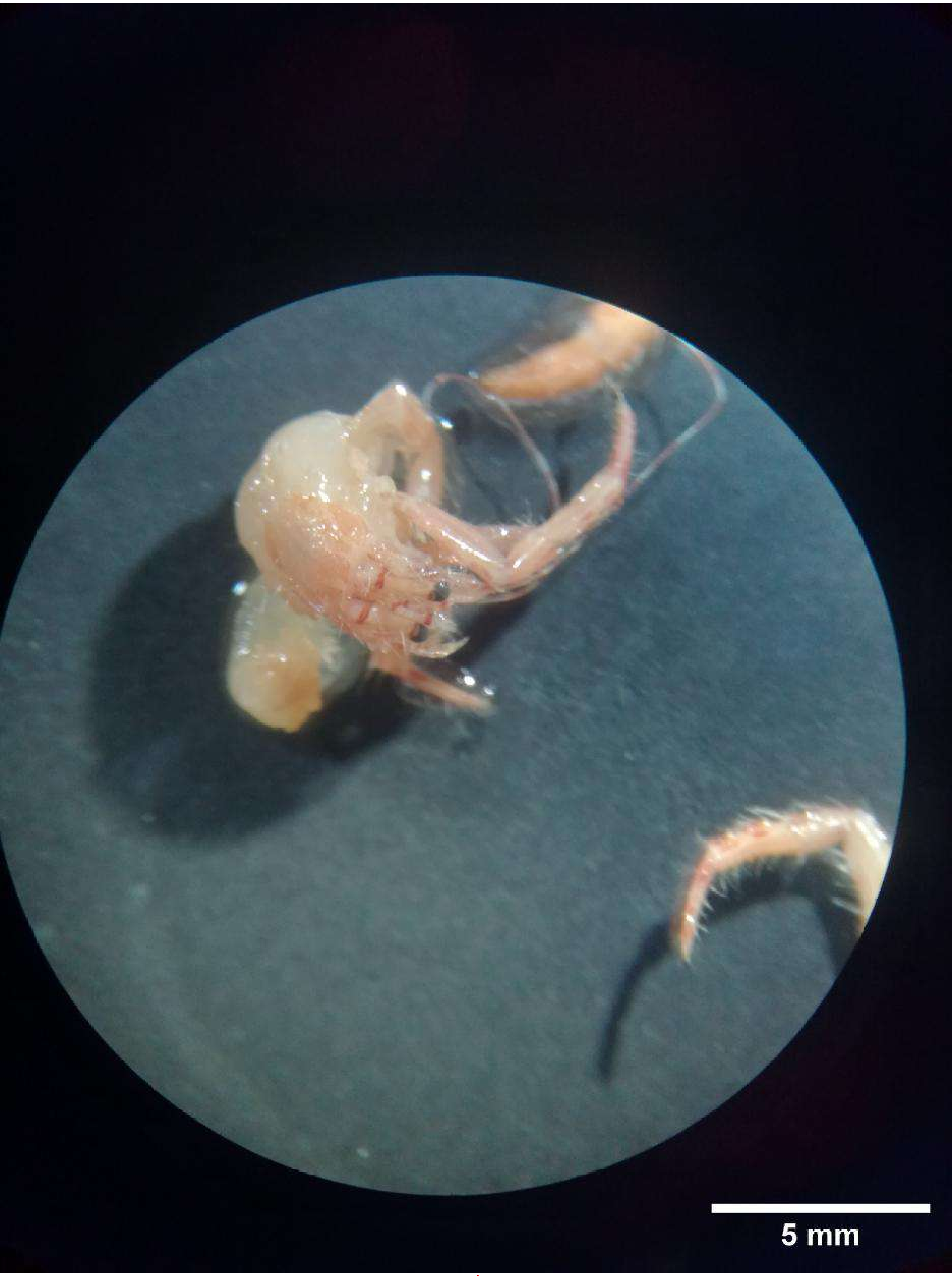
5 mm

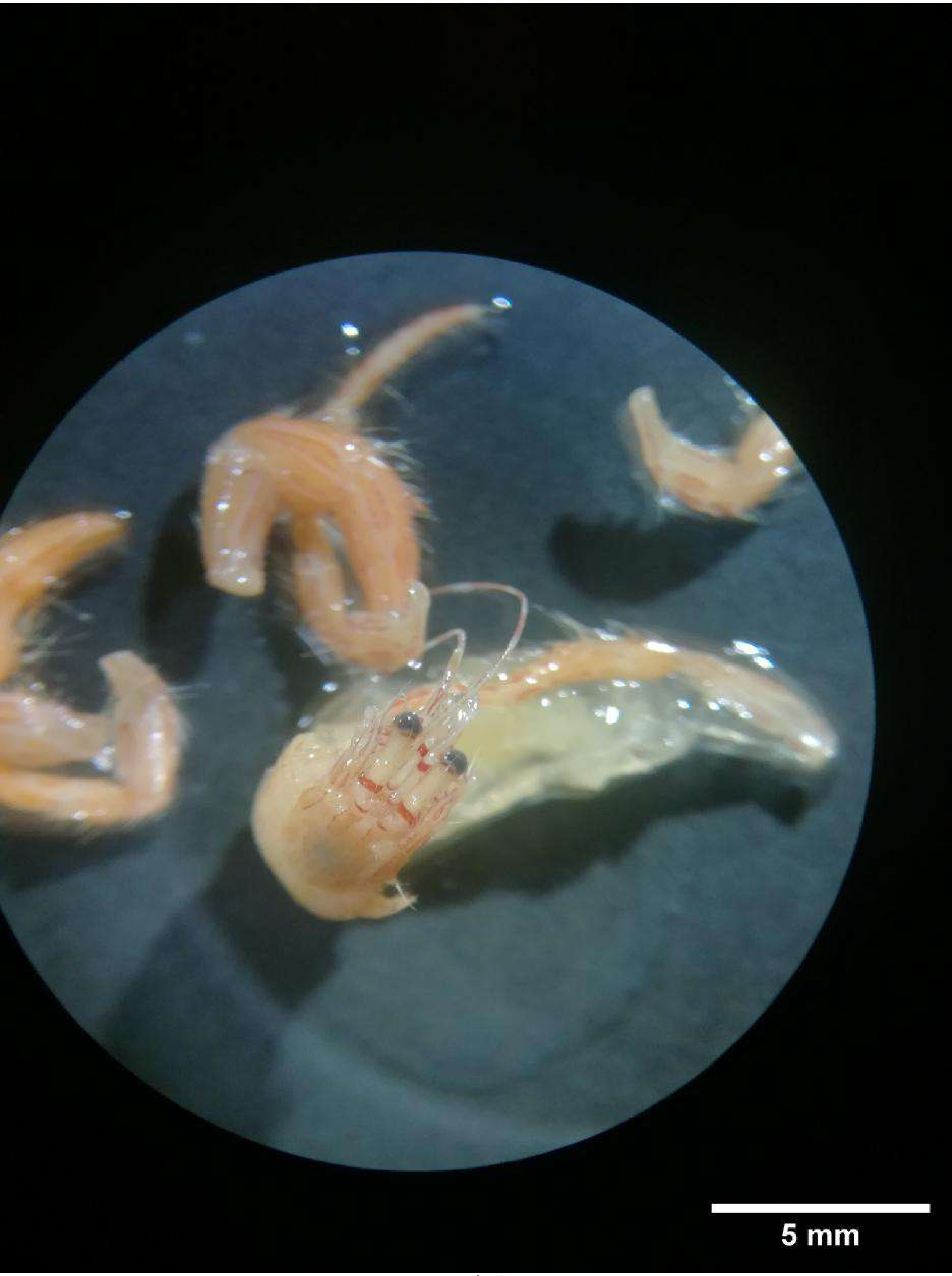
5 mm

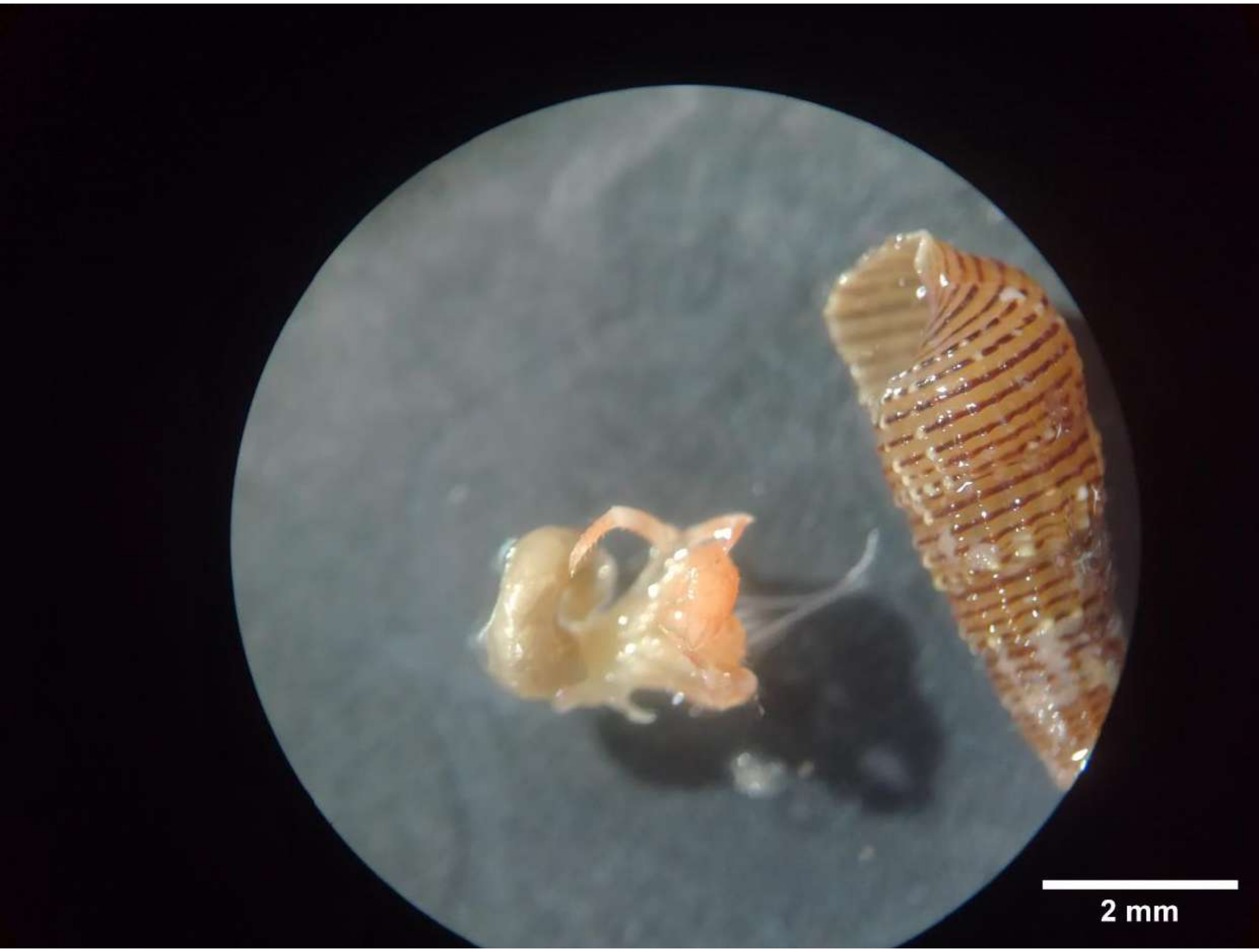
2 mm

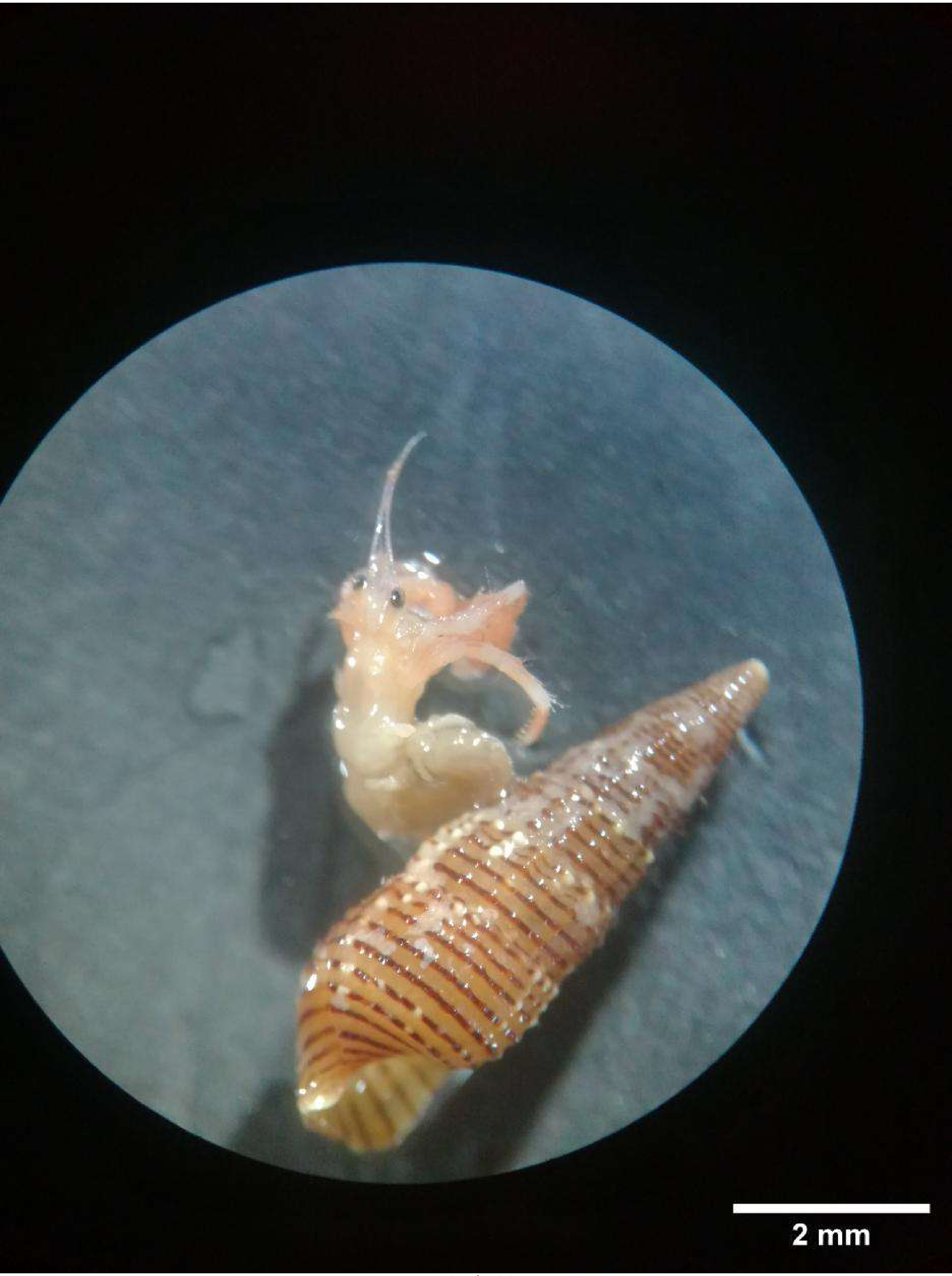
2 mm

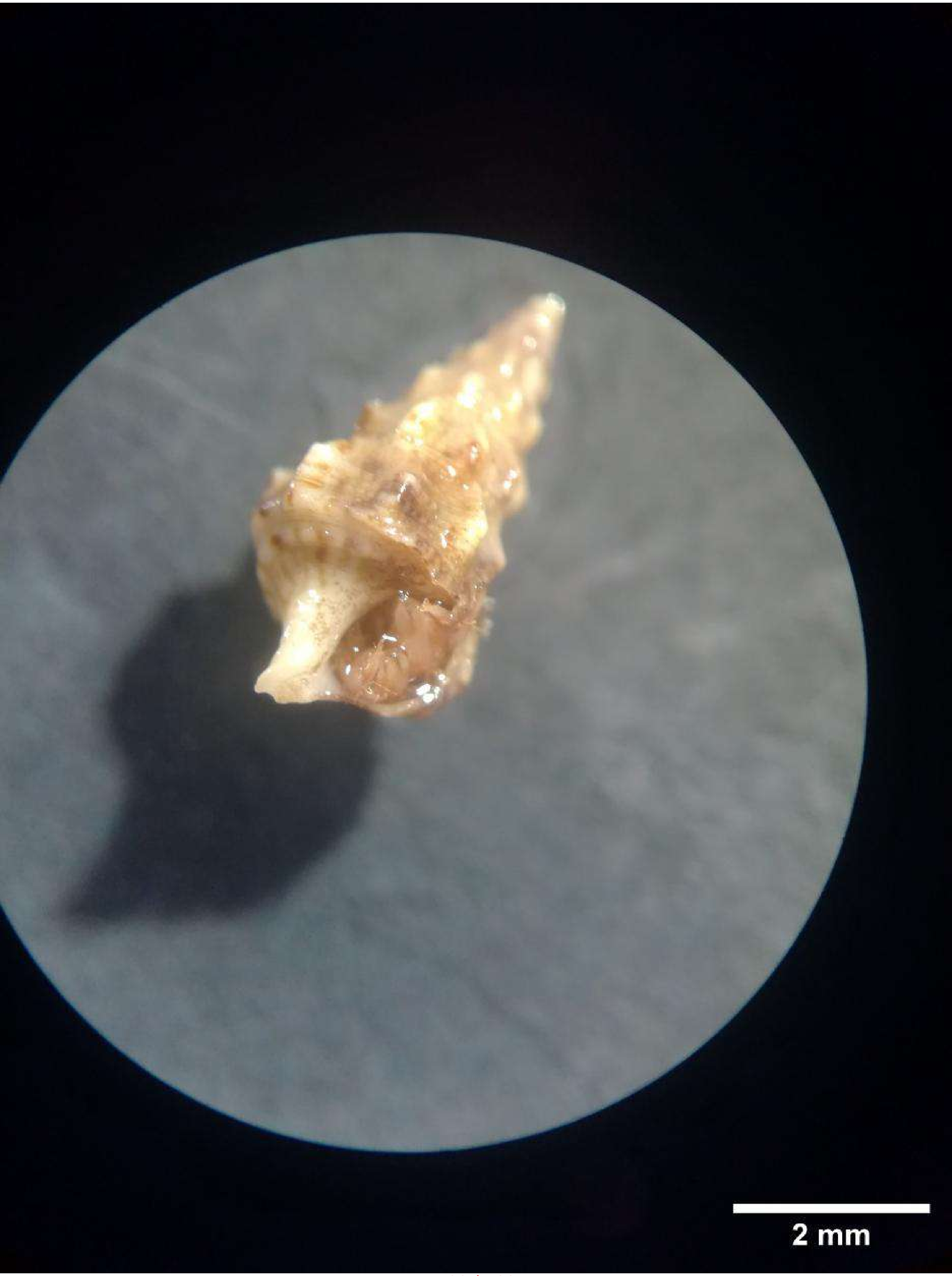
2 mm

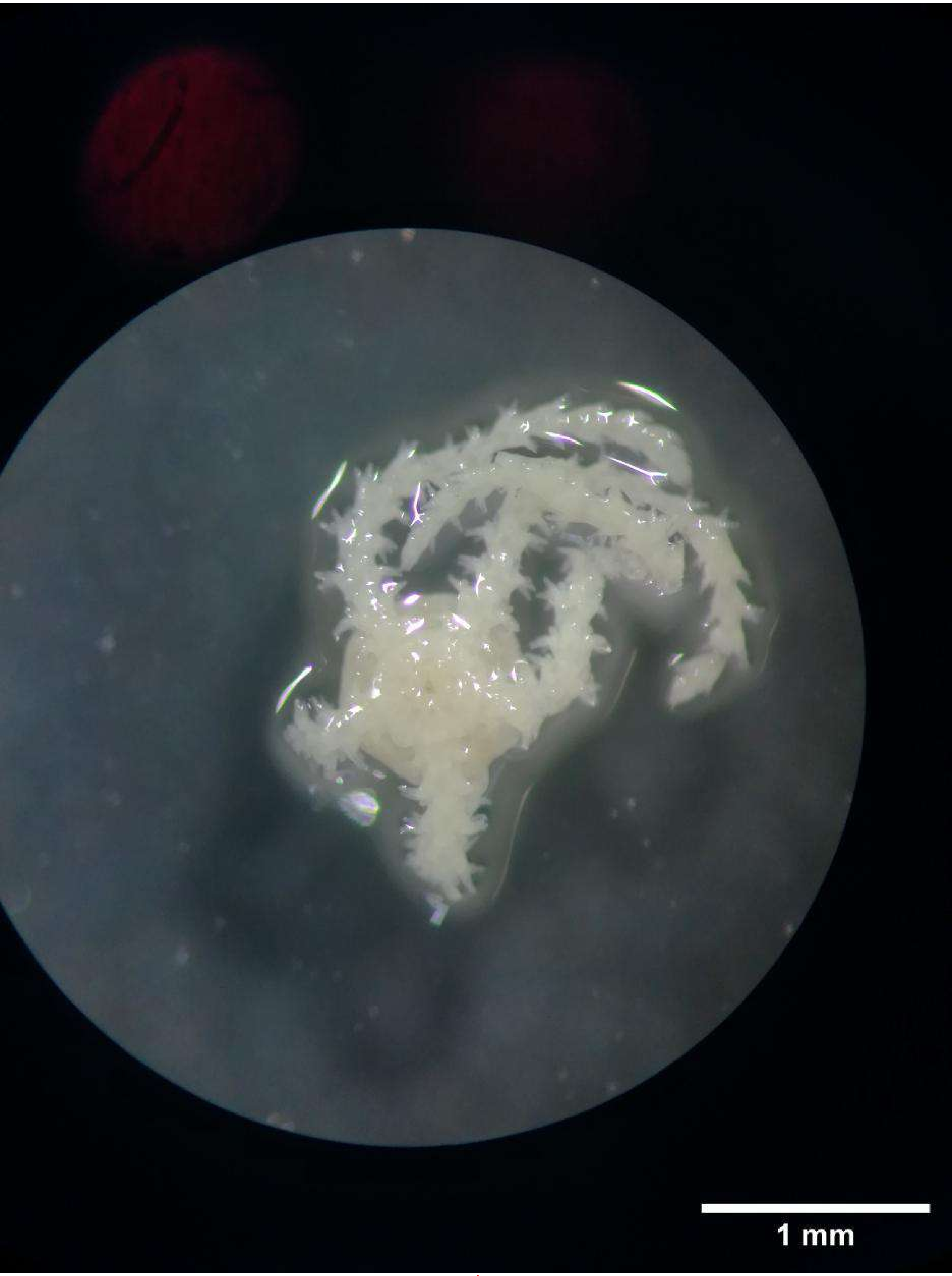
1 mm

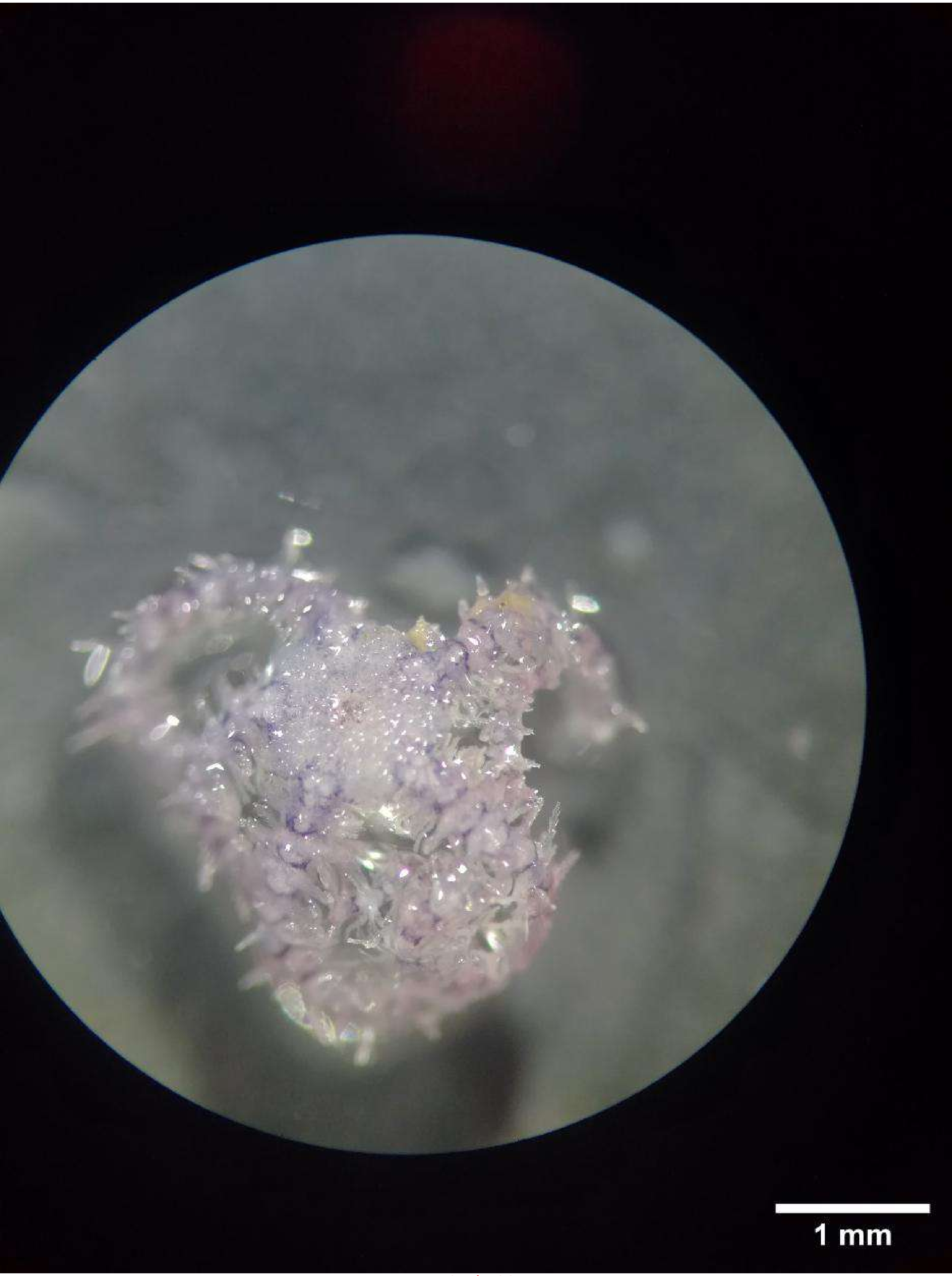
1 mm

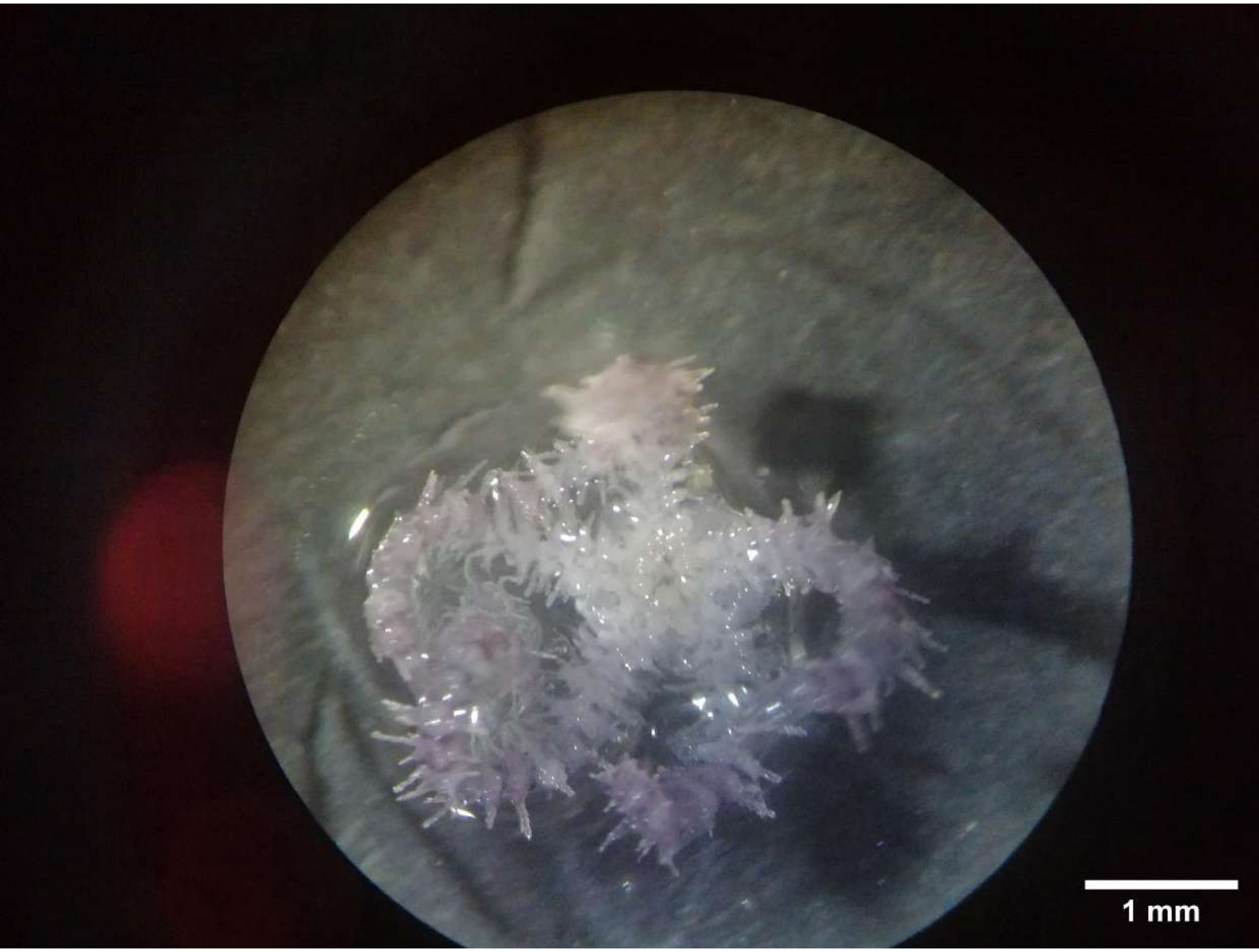
1 mm

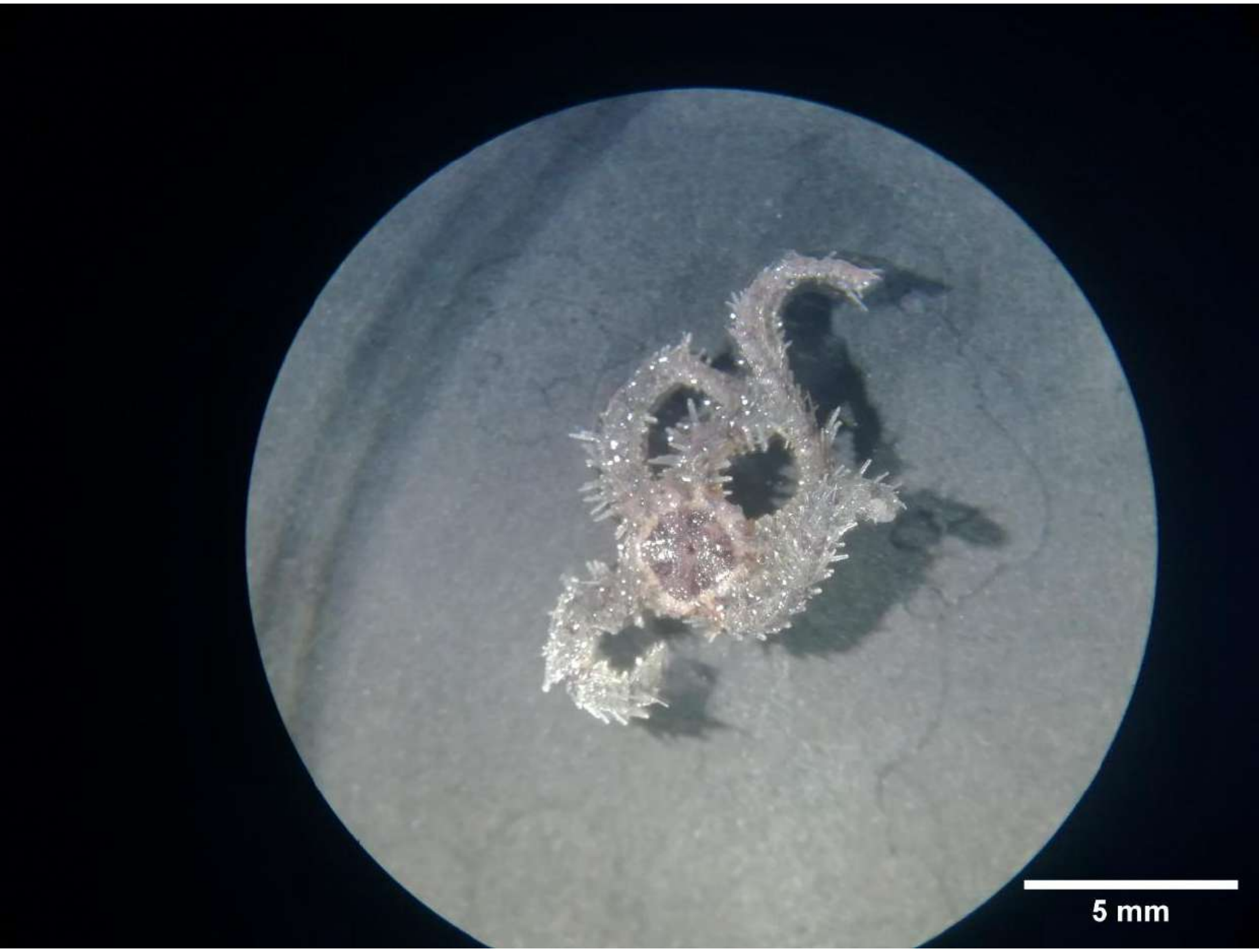
5 mm

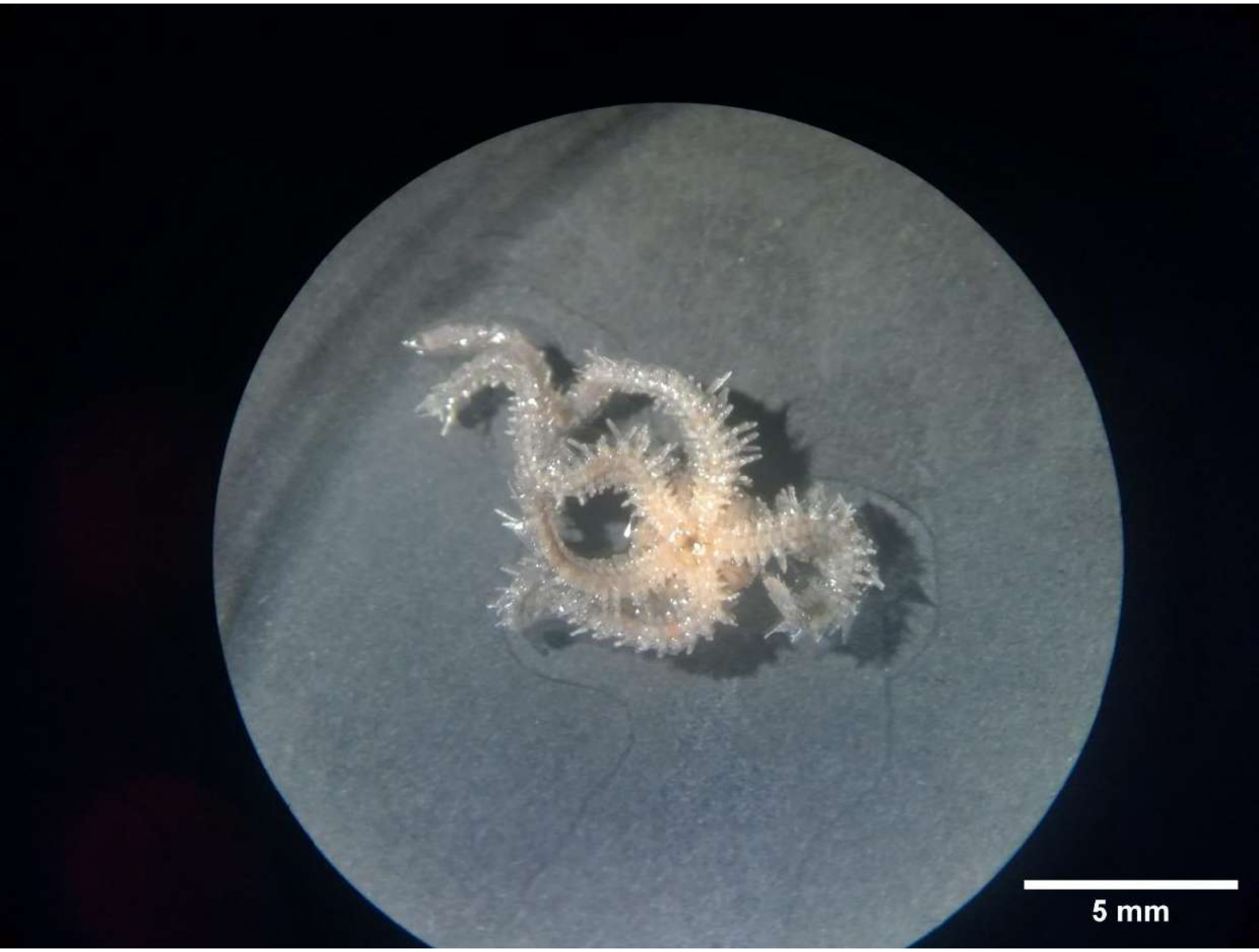
5 mm

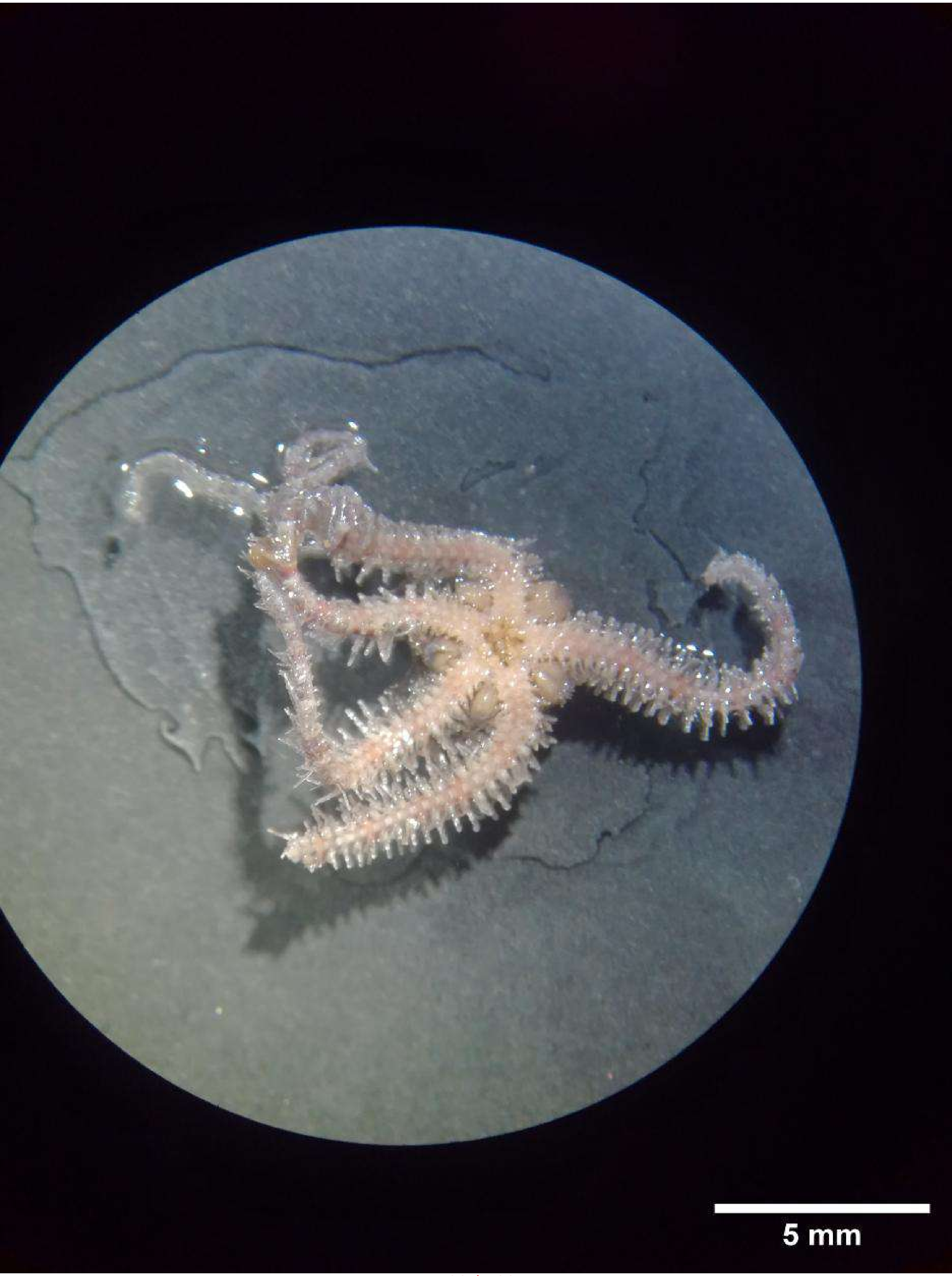
5 mm

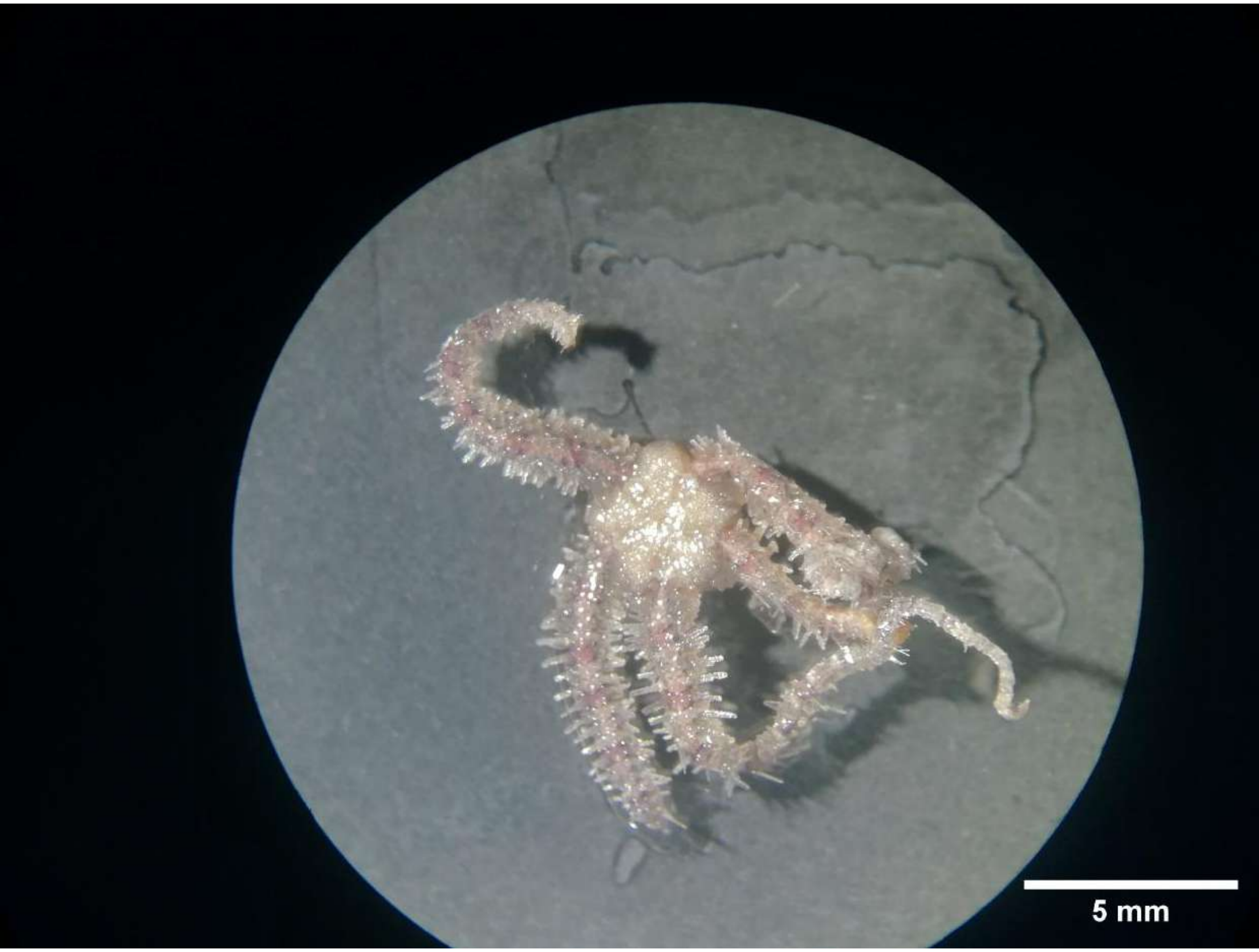
5 mm

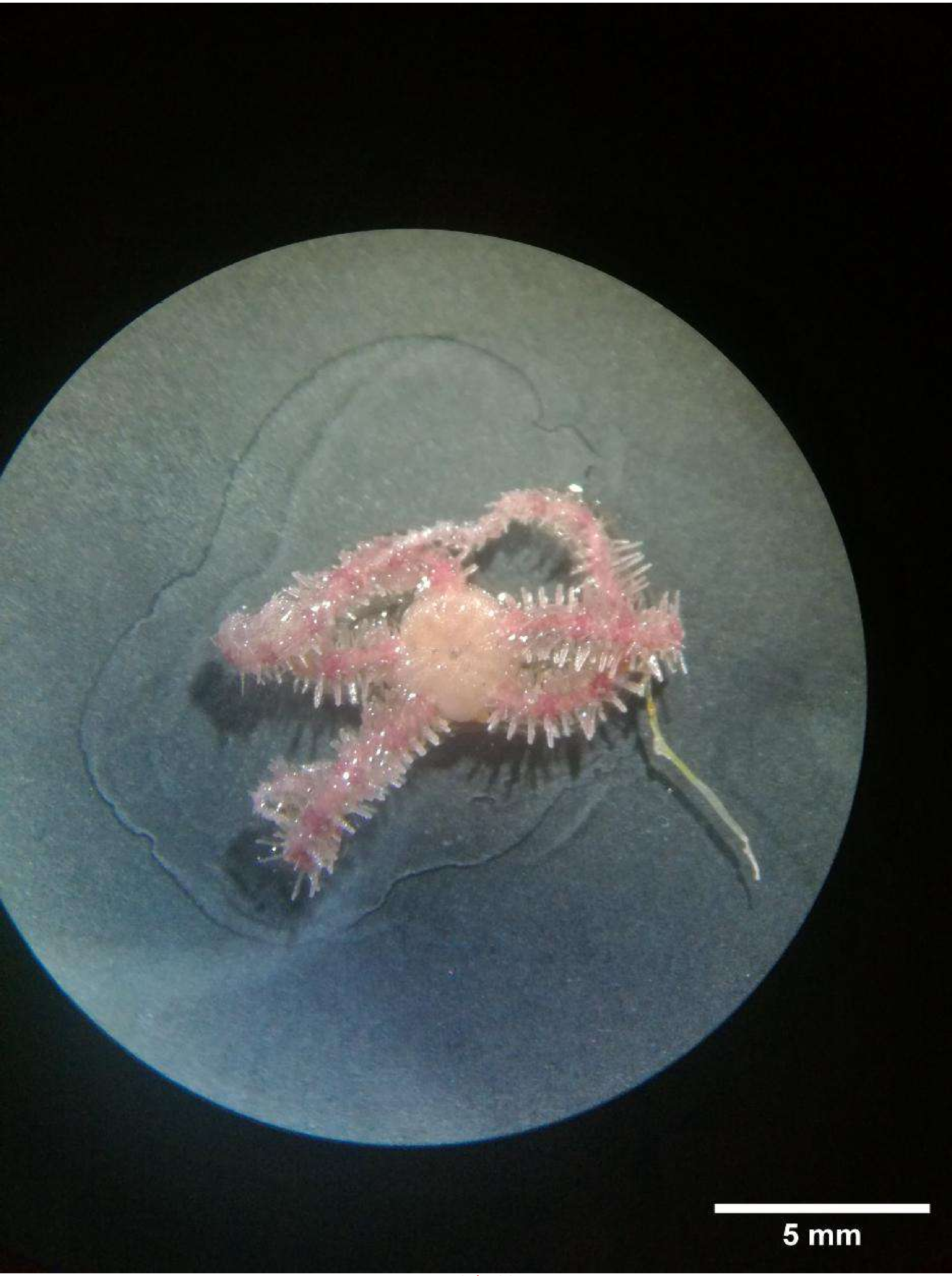
5 mm

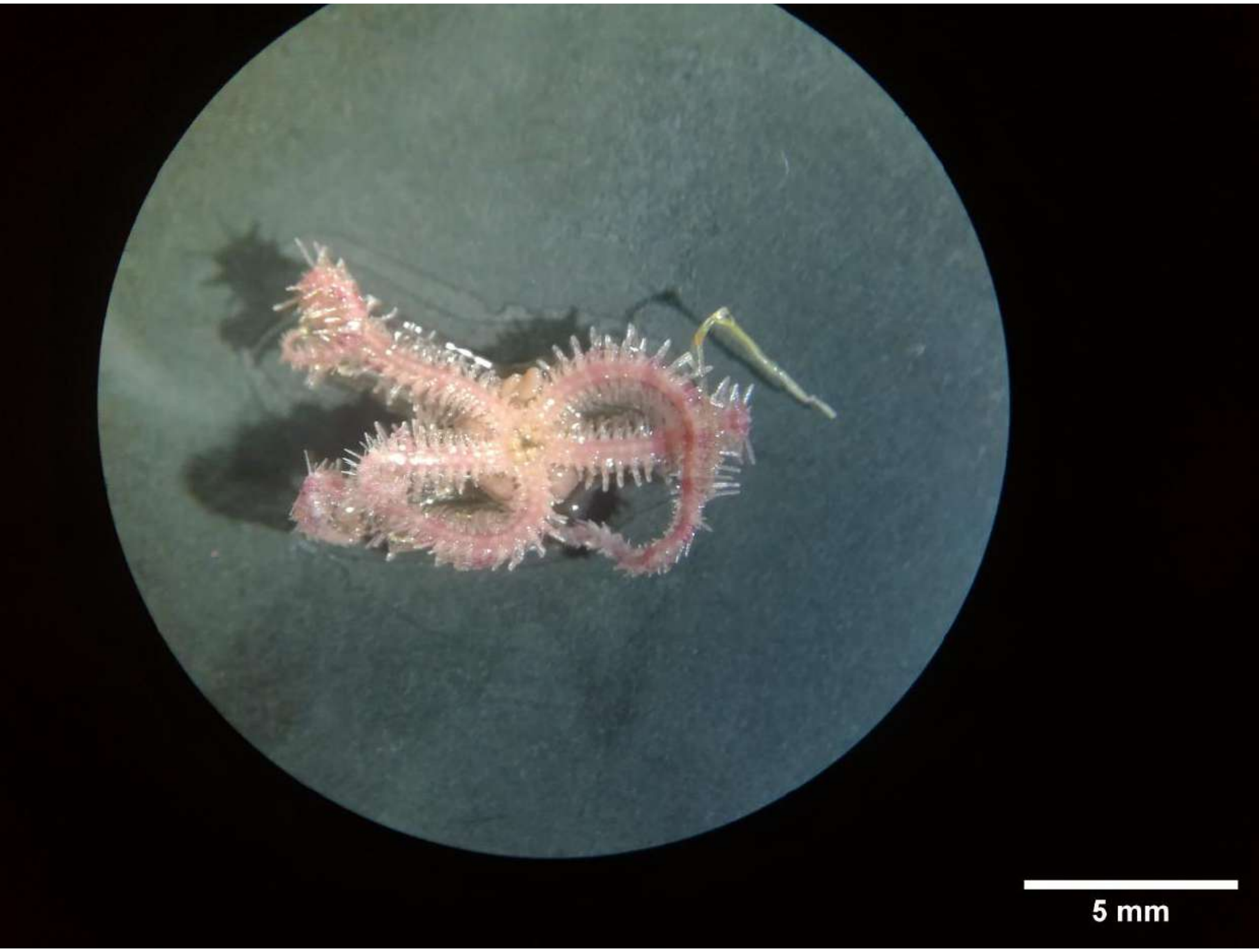
5 mm

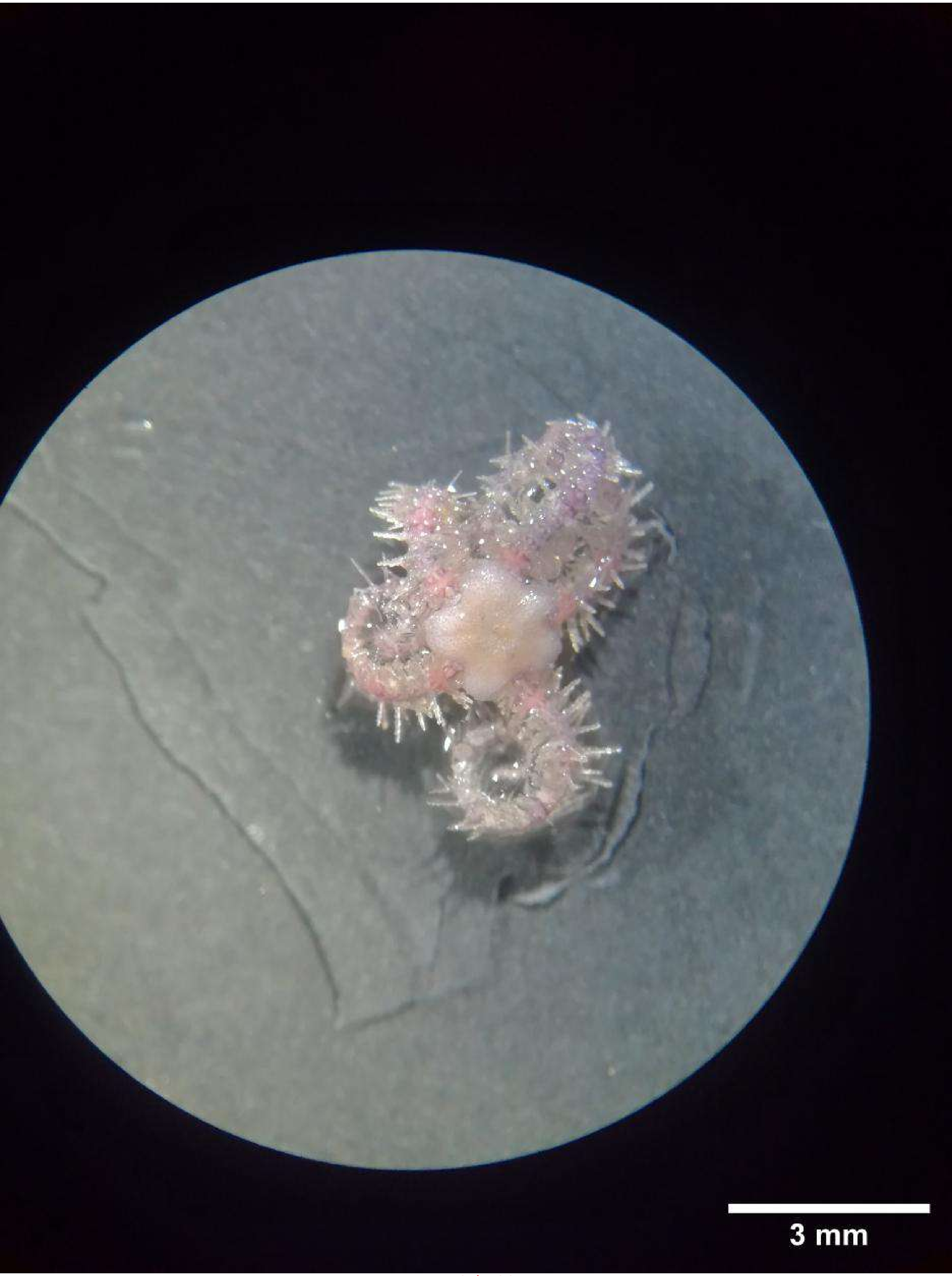
3 mm

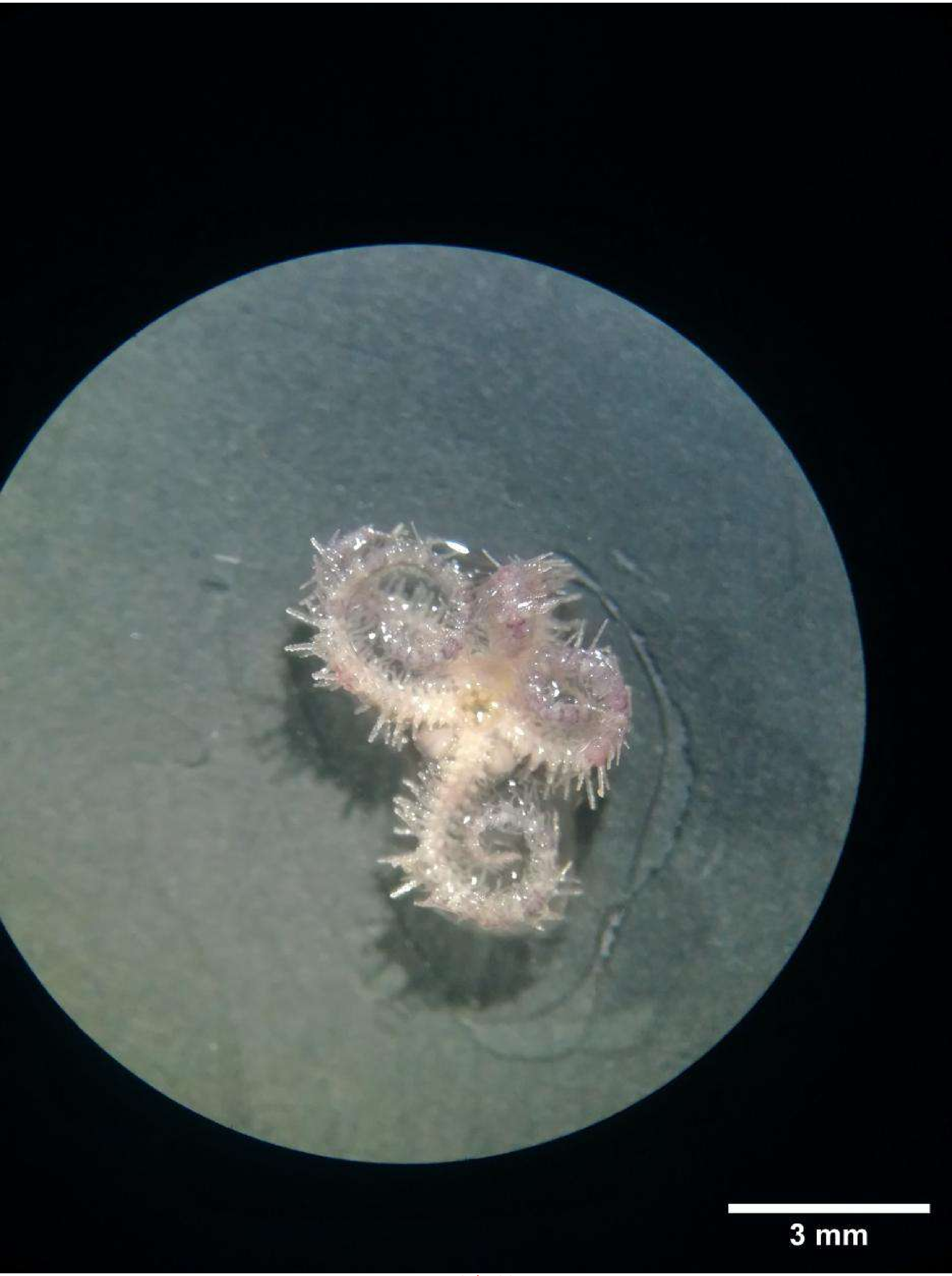
3 mm

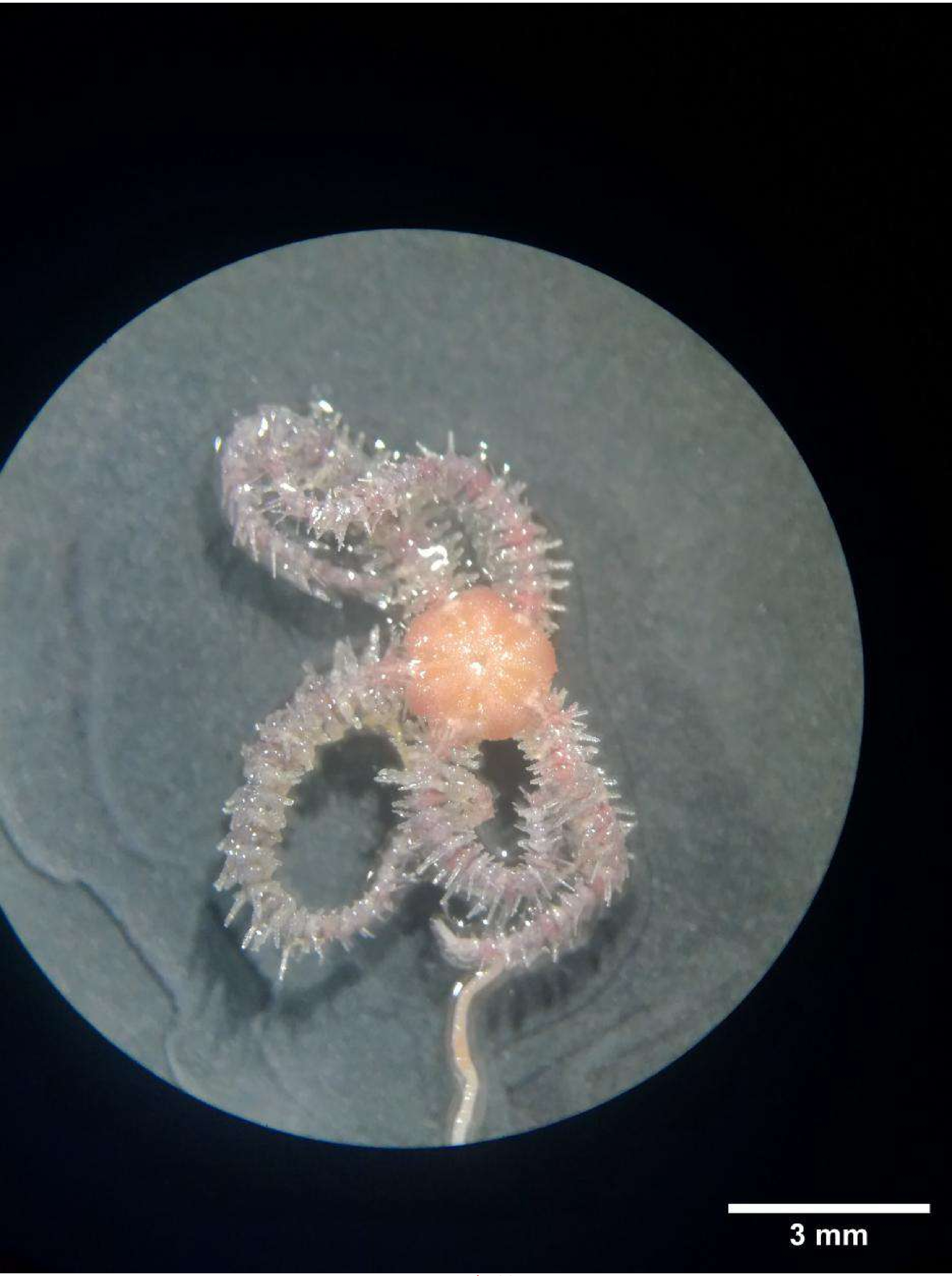
3 mm

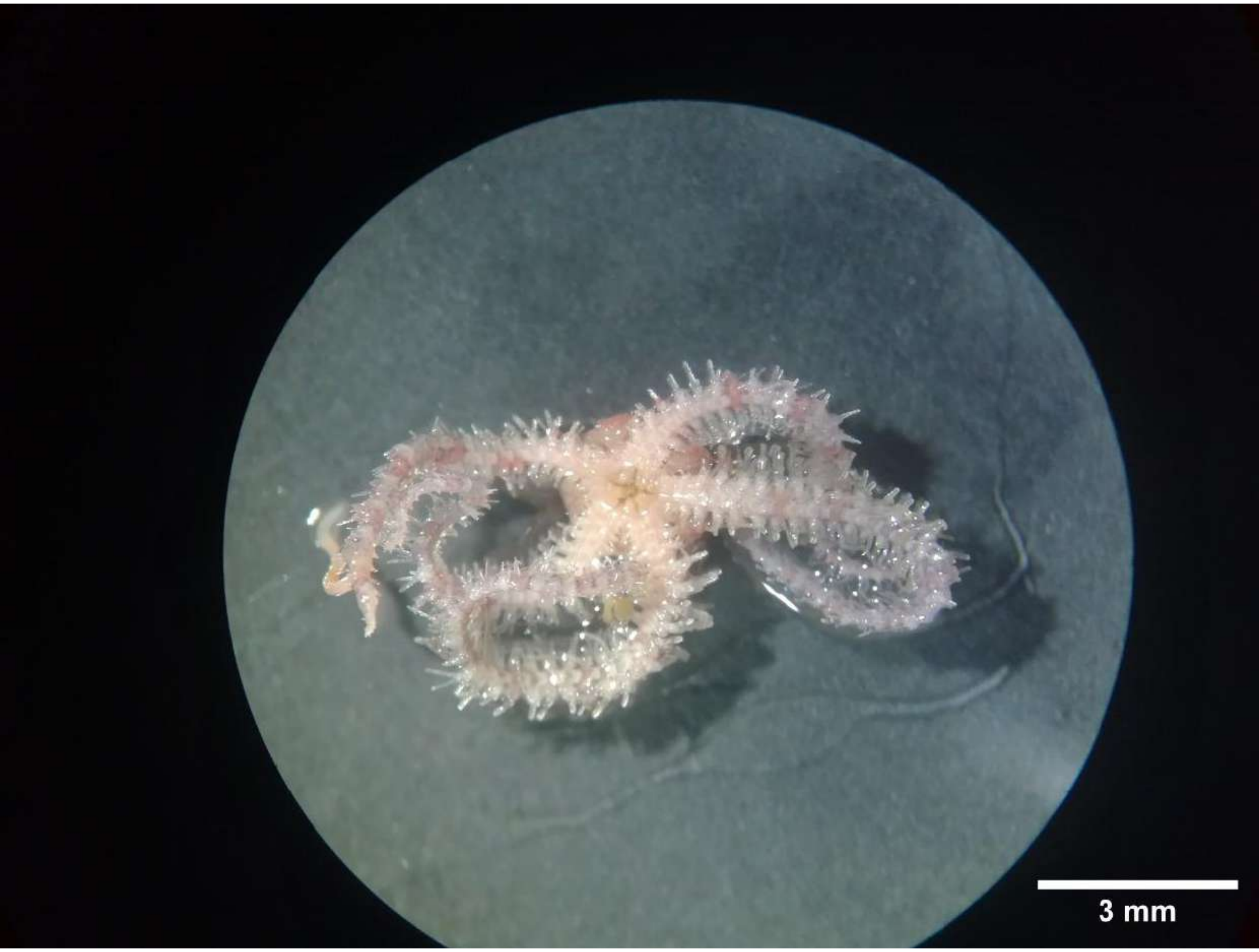
3 mm

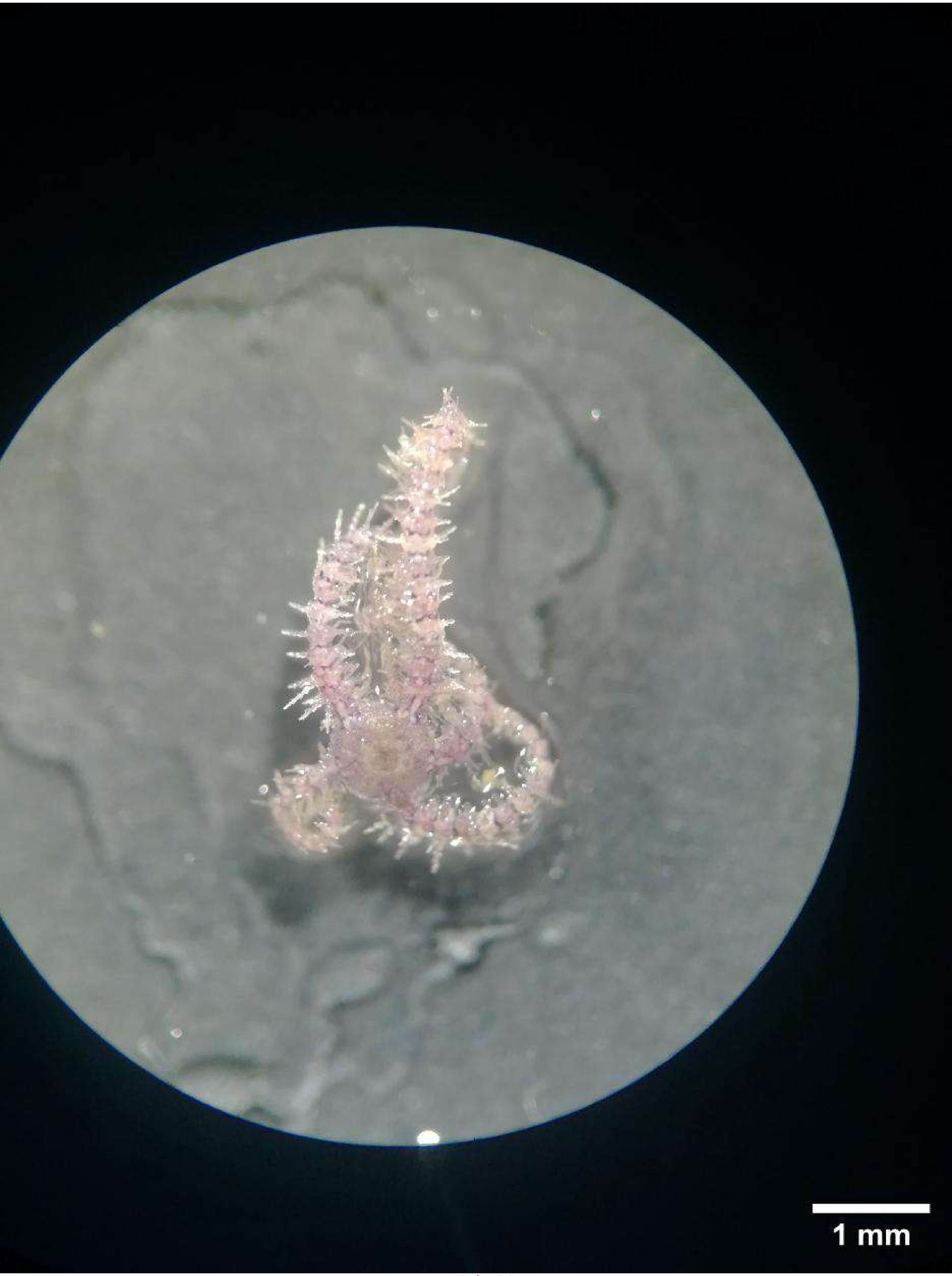
1 mm

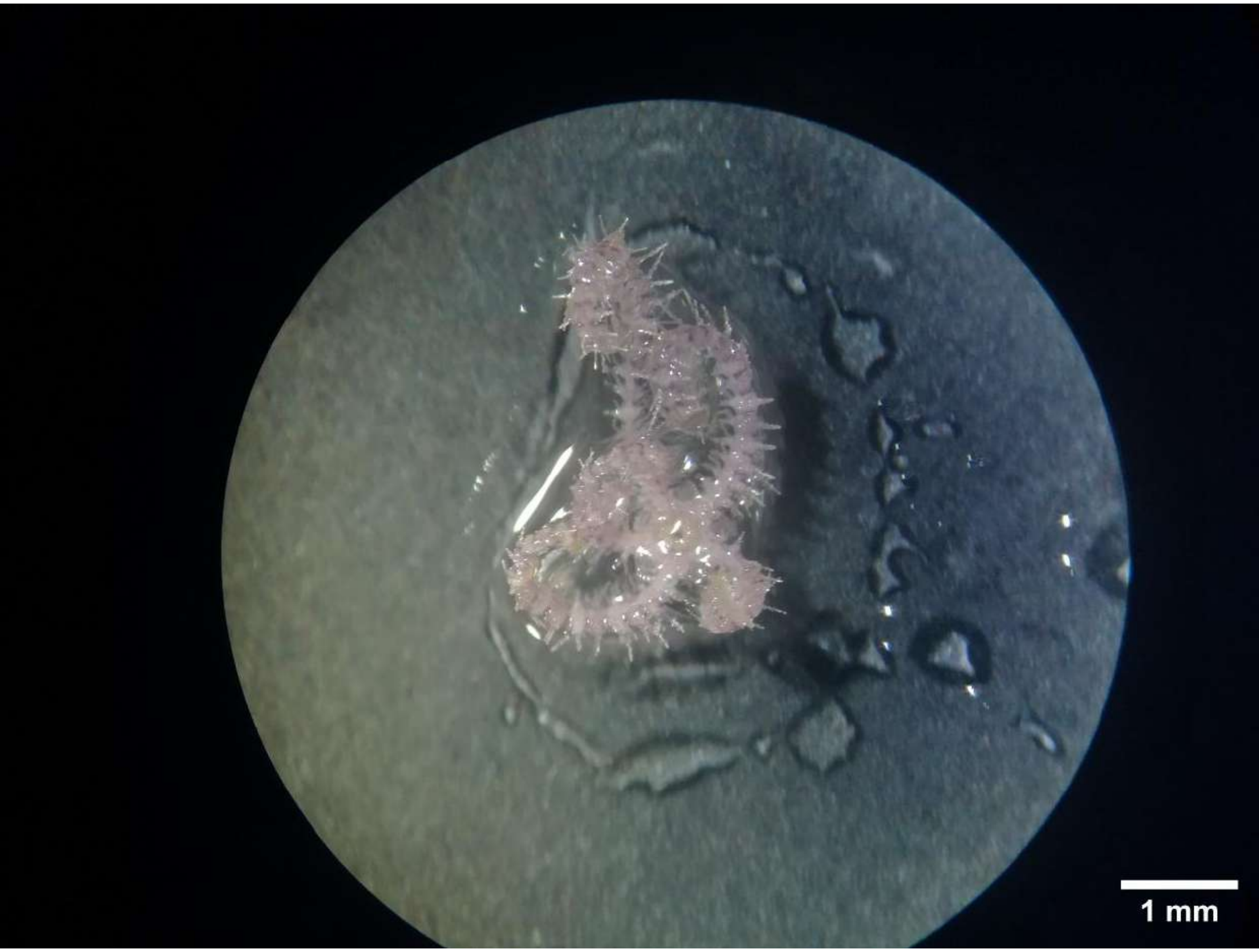
1 mm

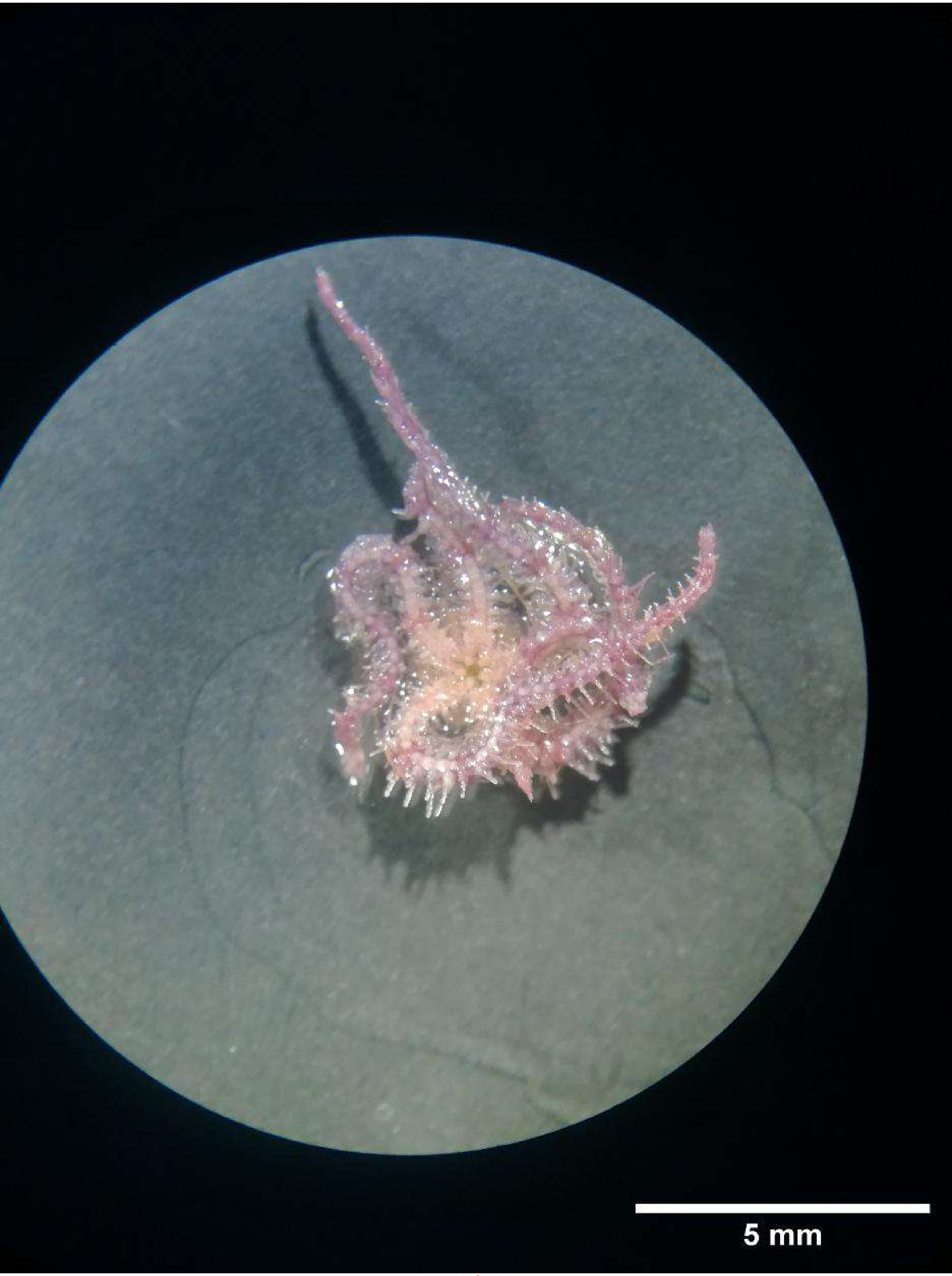
5 mm

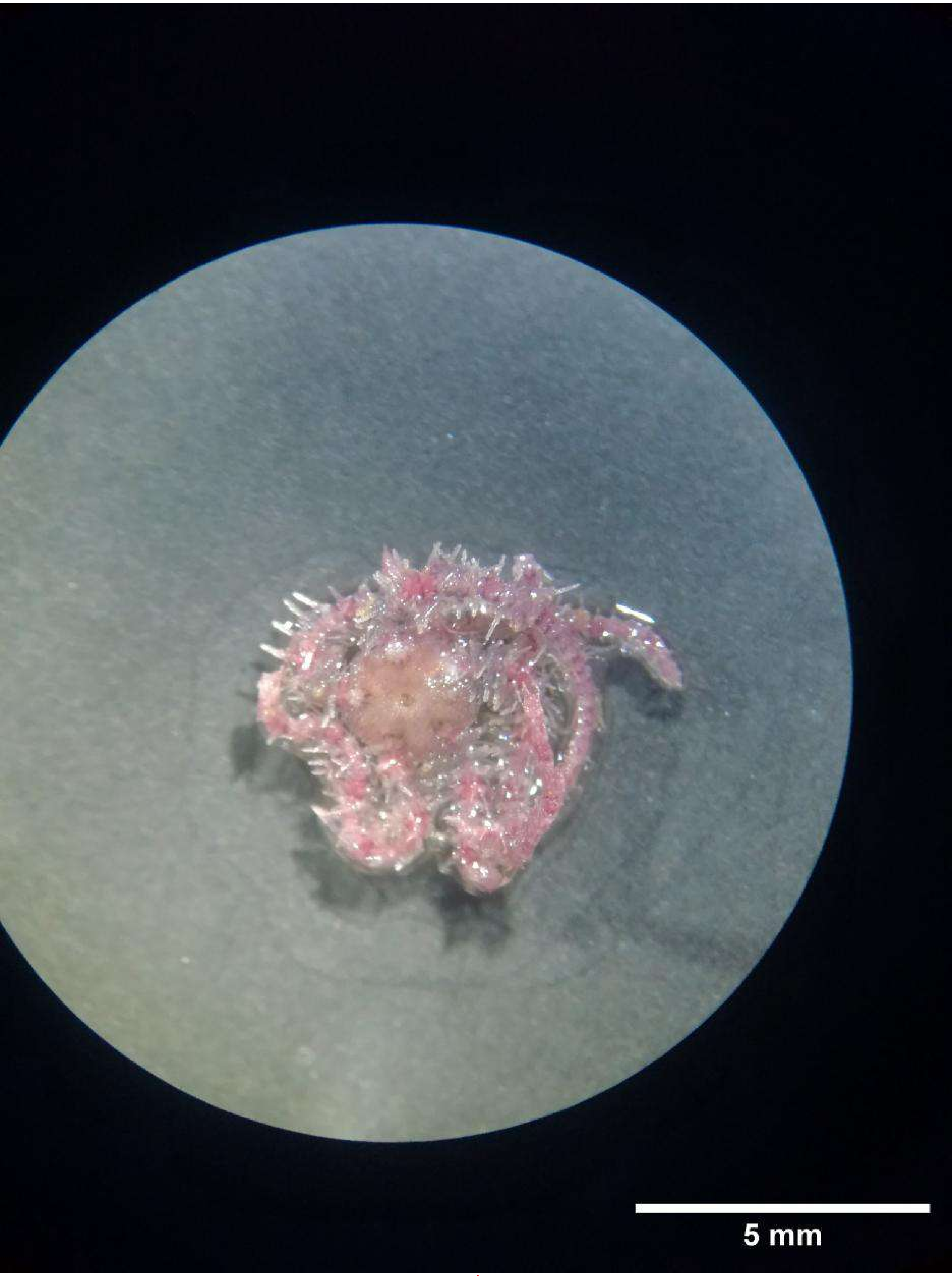
5 mm

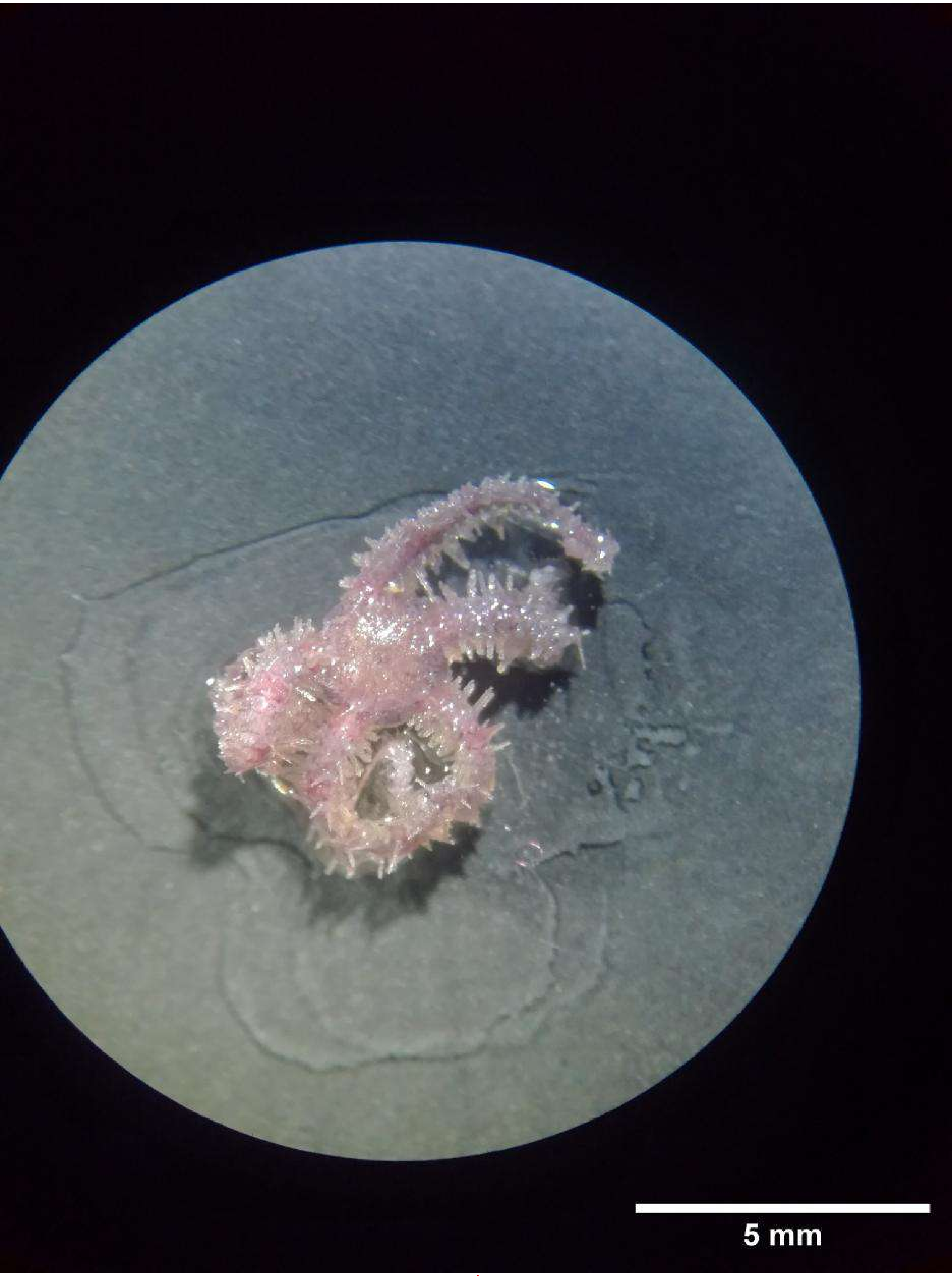
5 mm

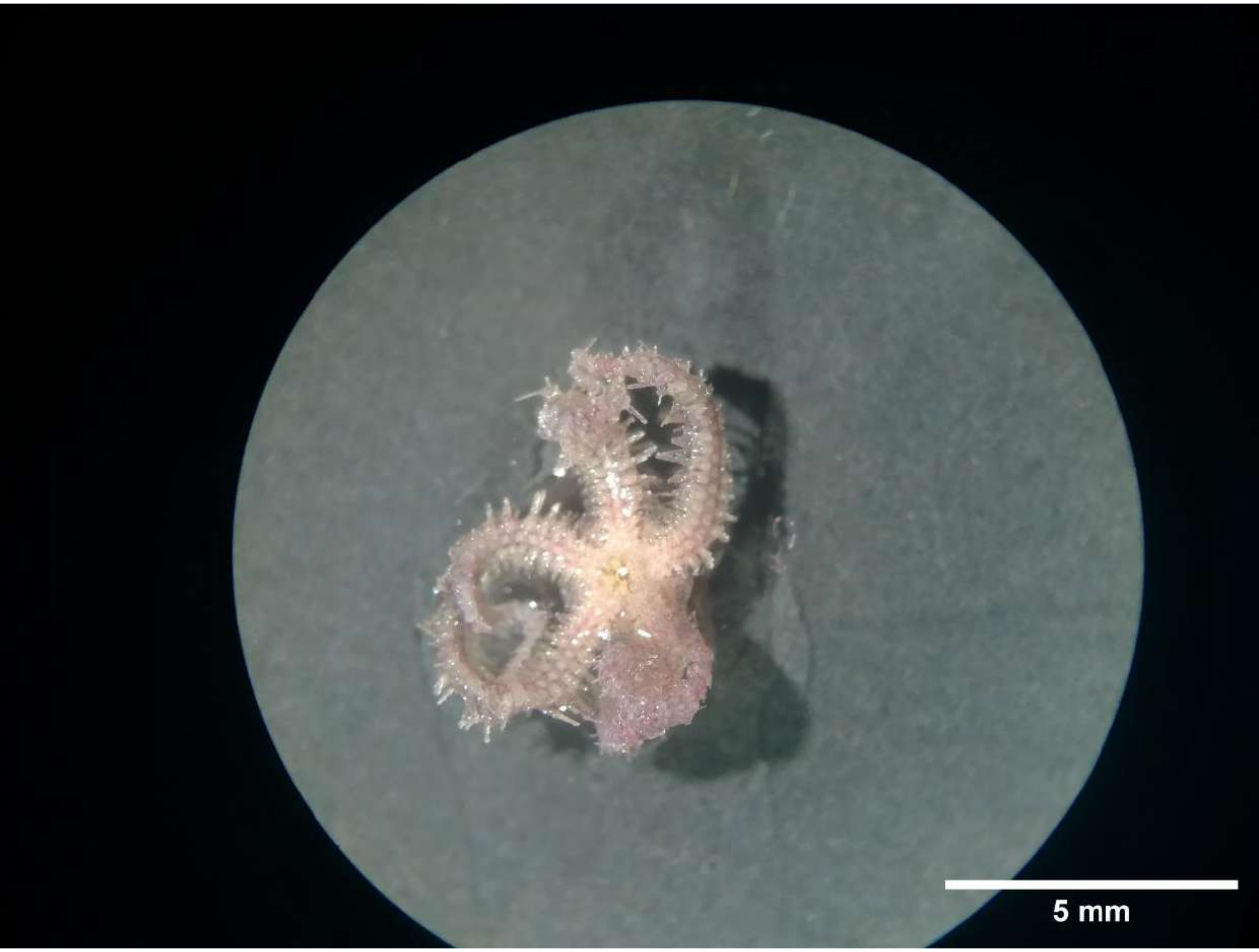
5 mm

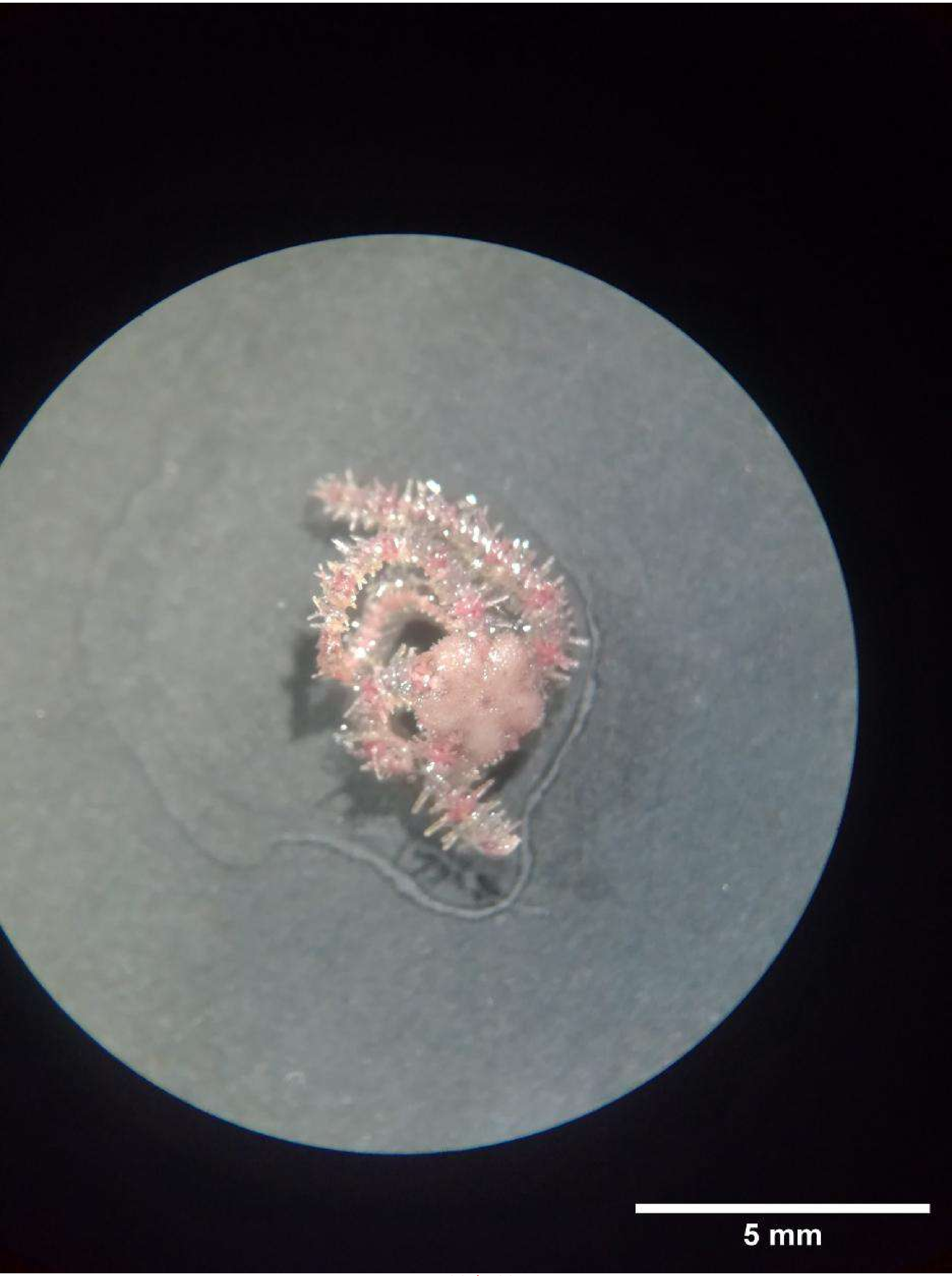
5 mm

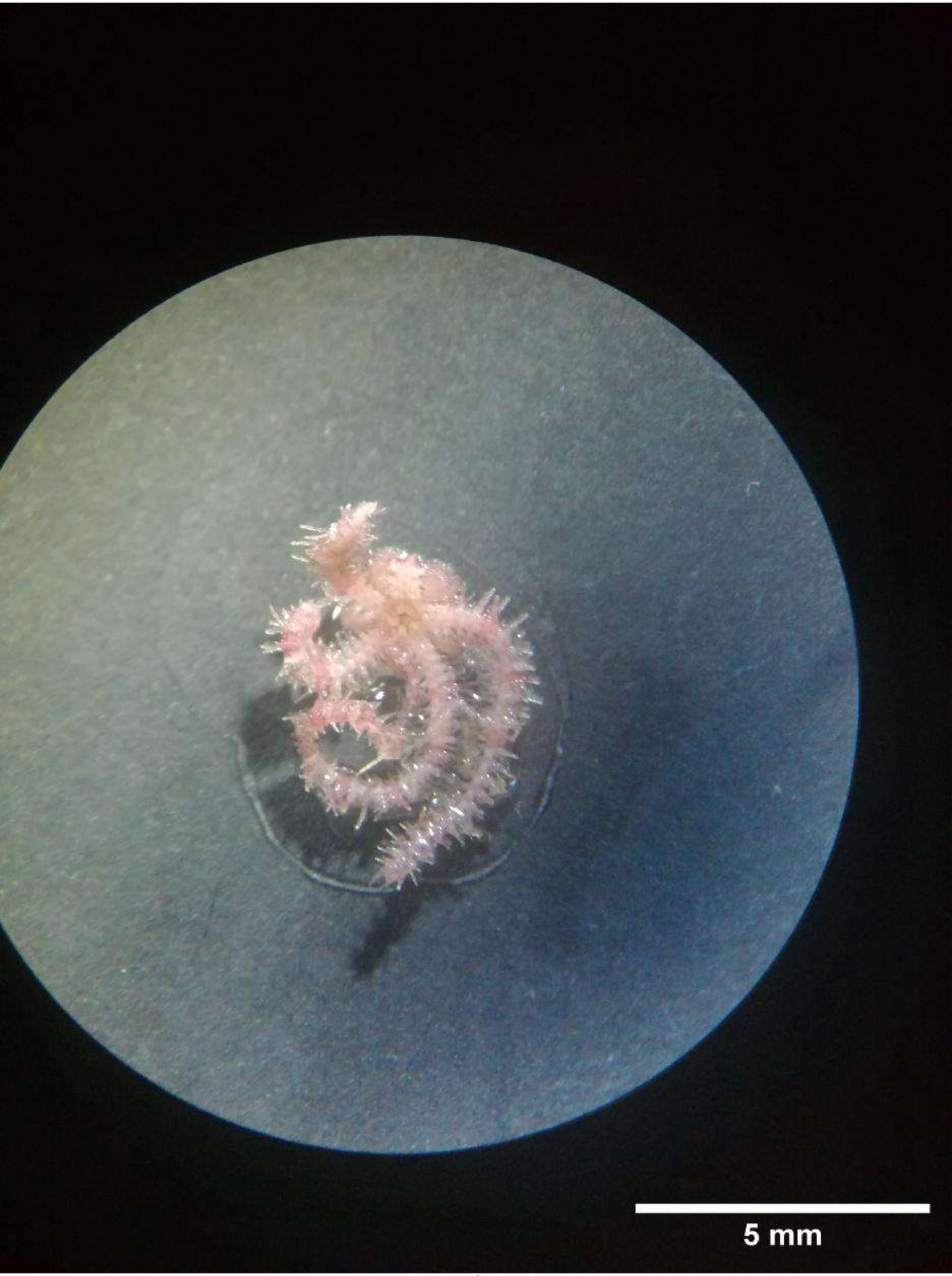
5 mm

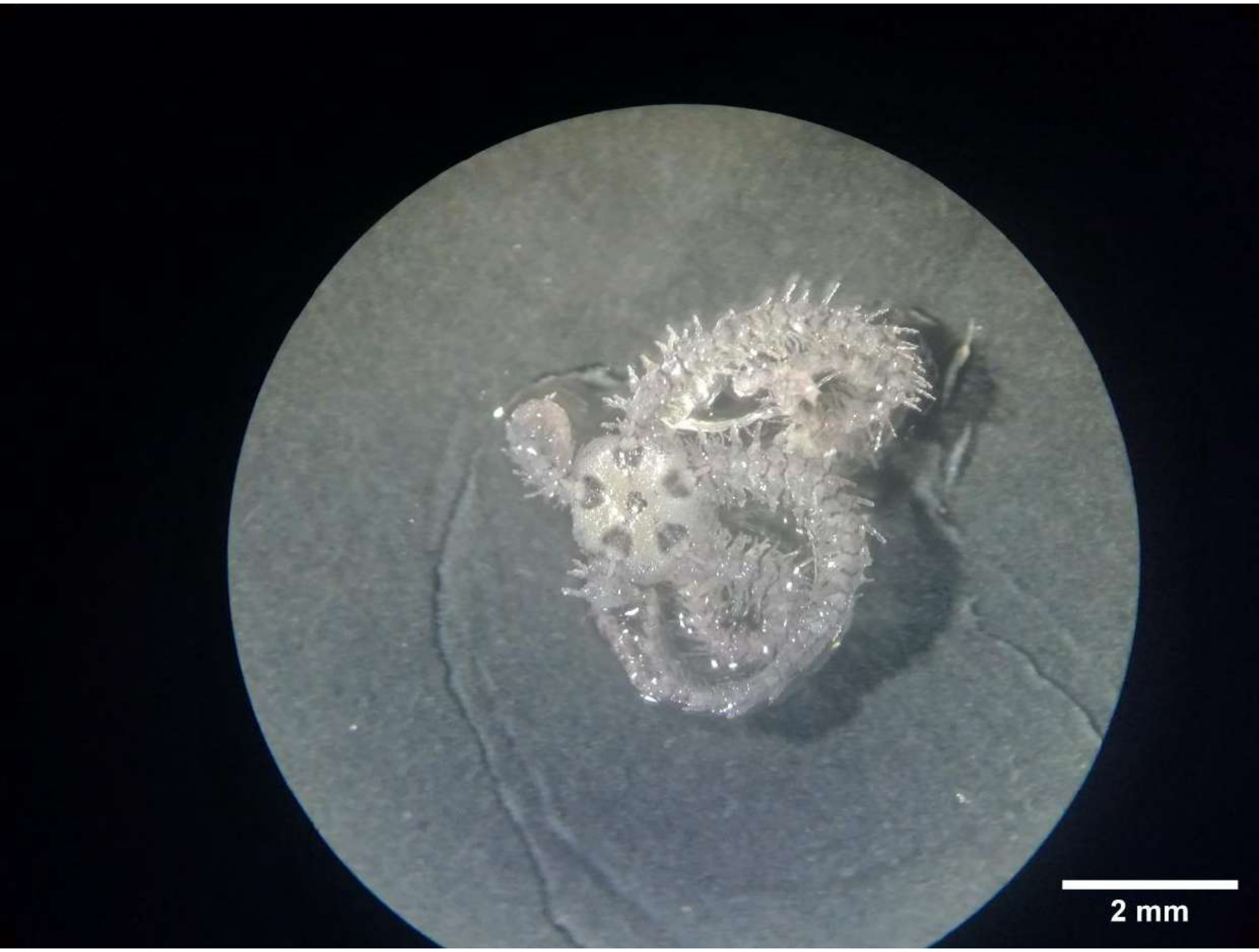
2 mm

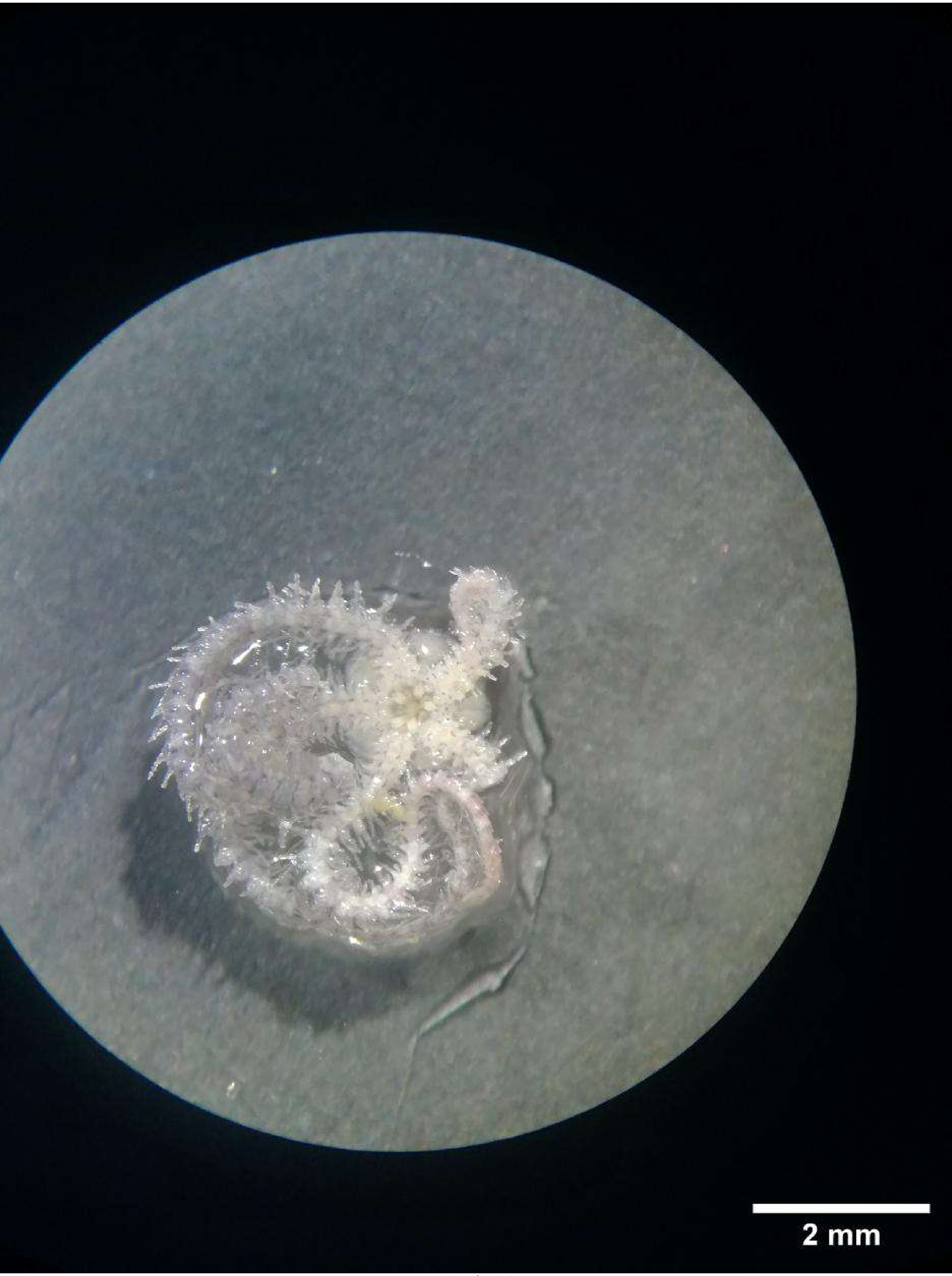
2 mm

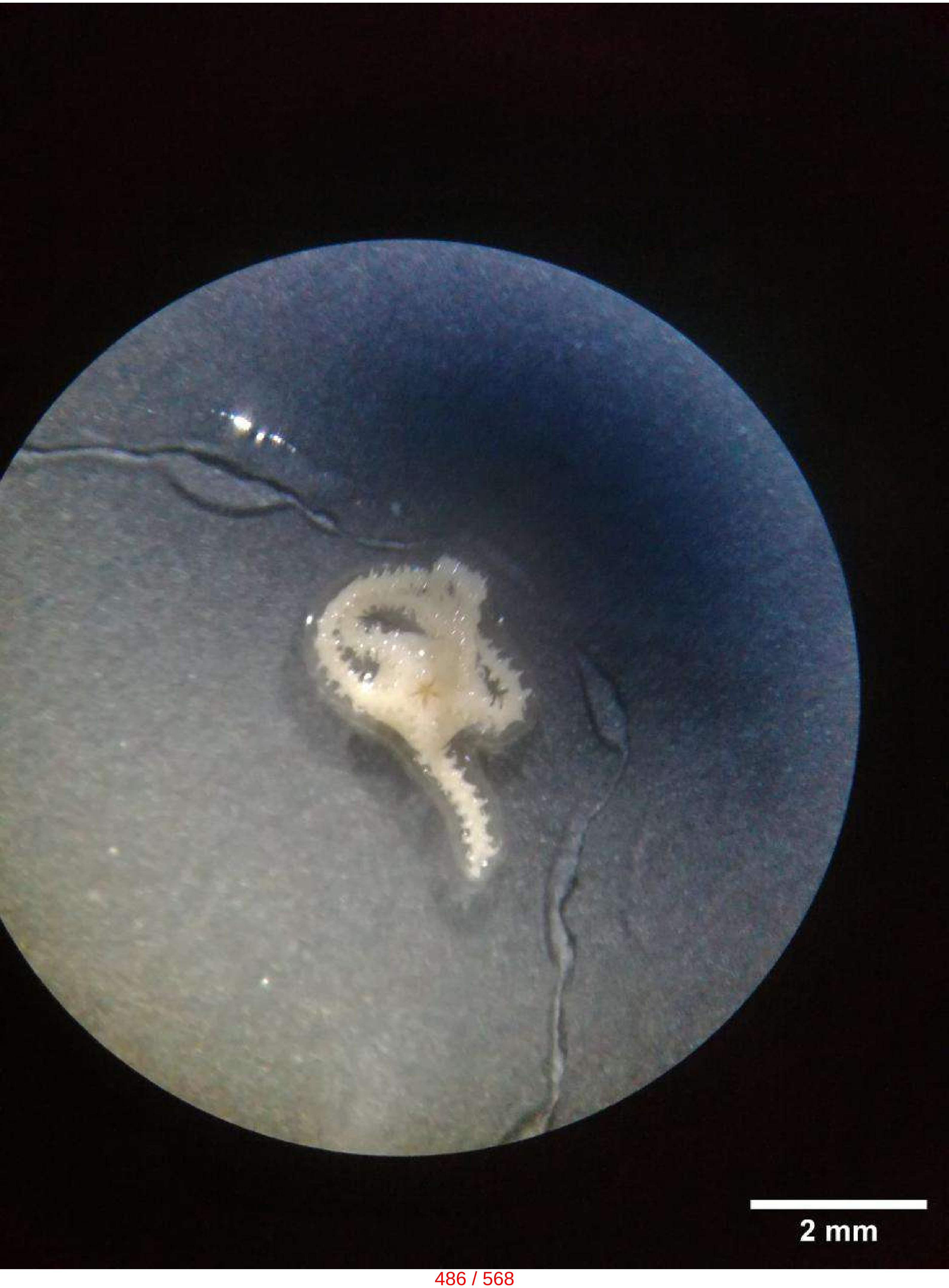
2 mm

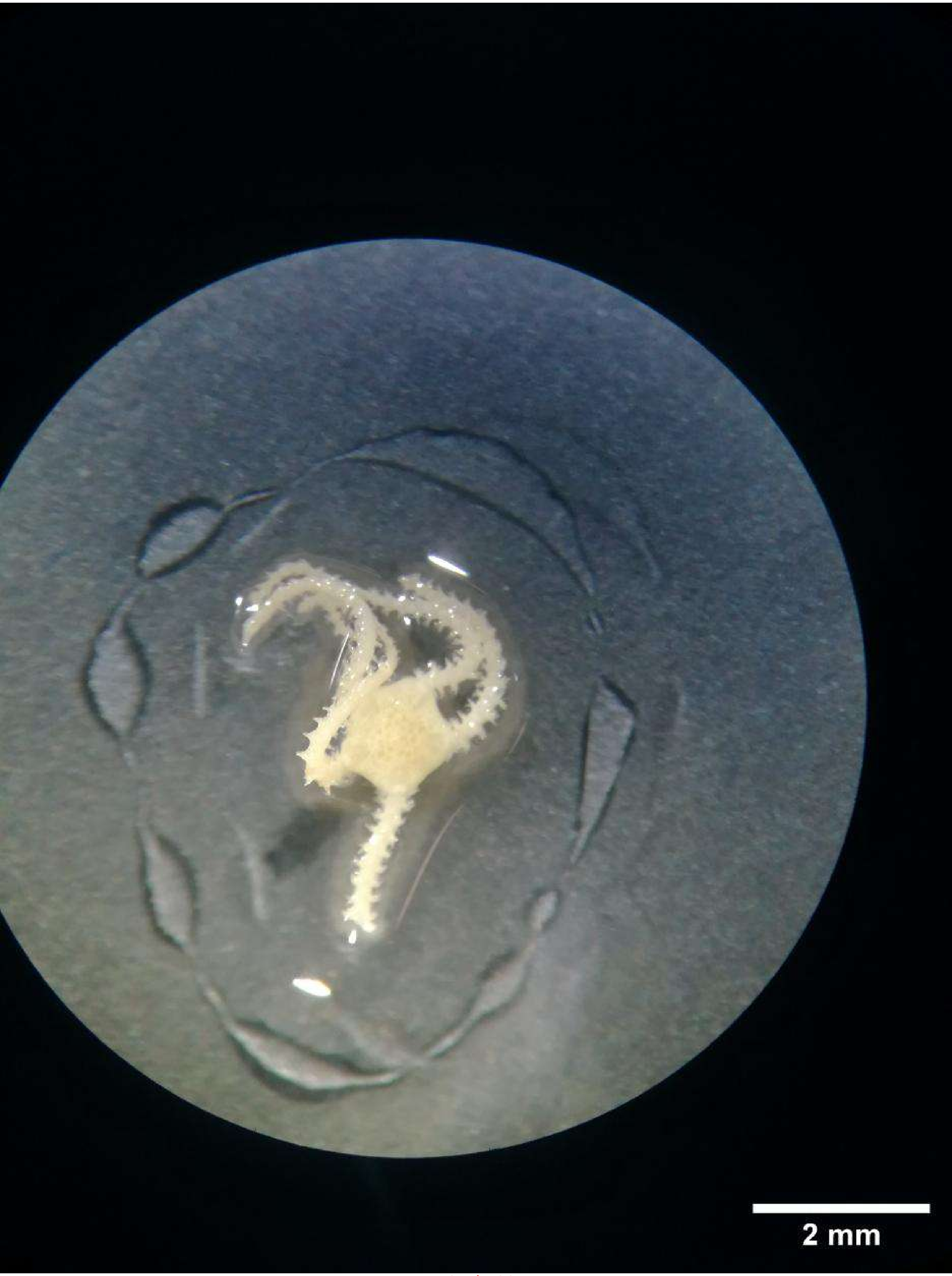
2 mm

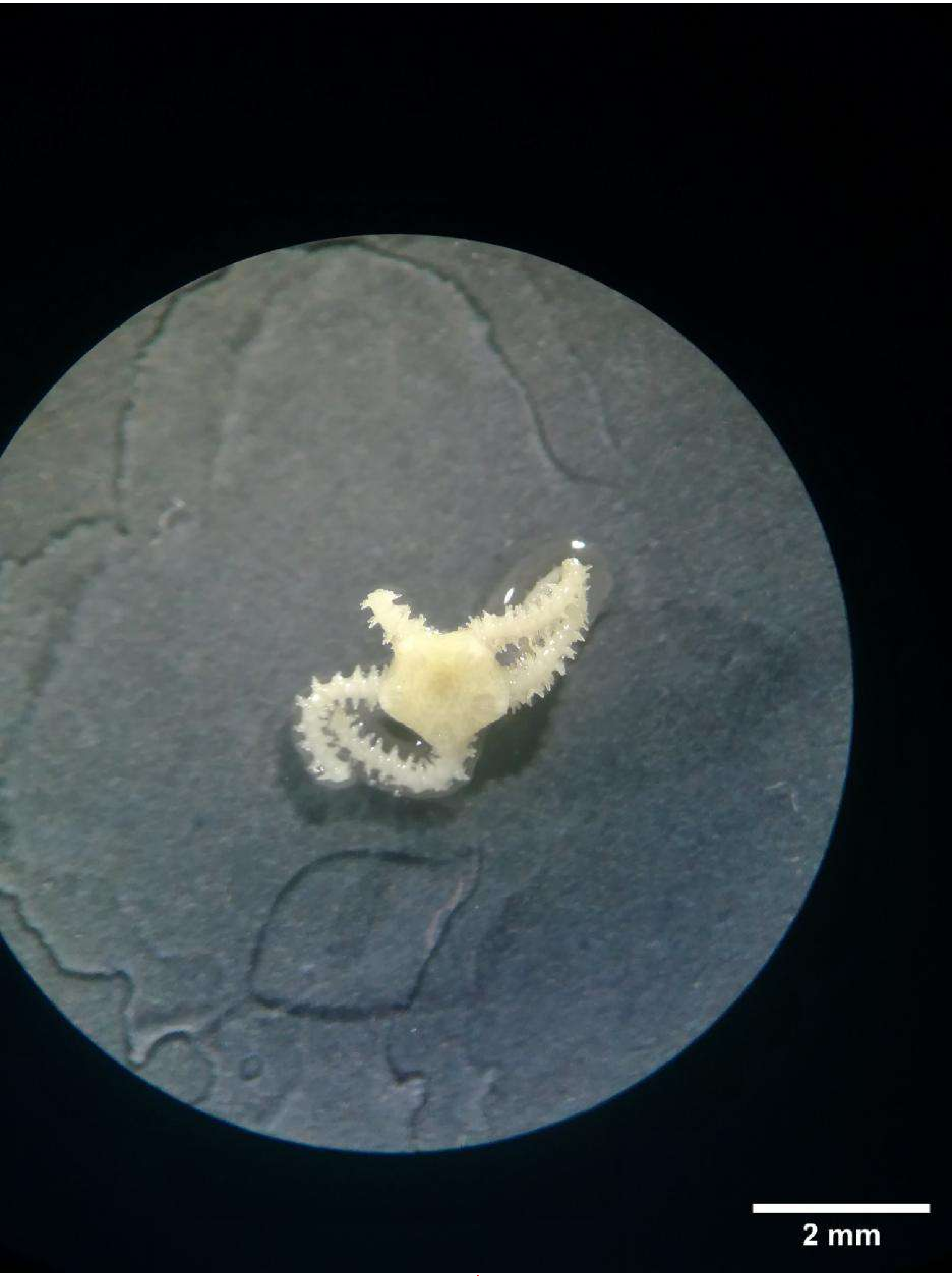
2 mm

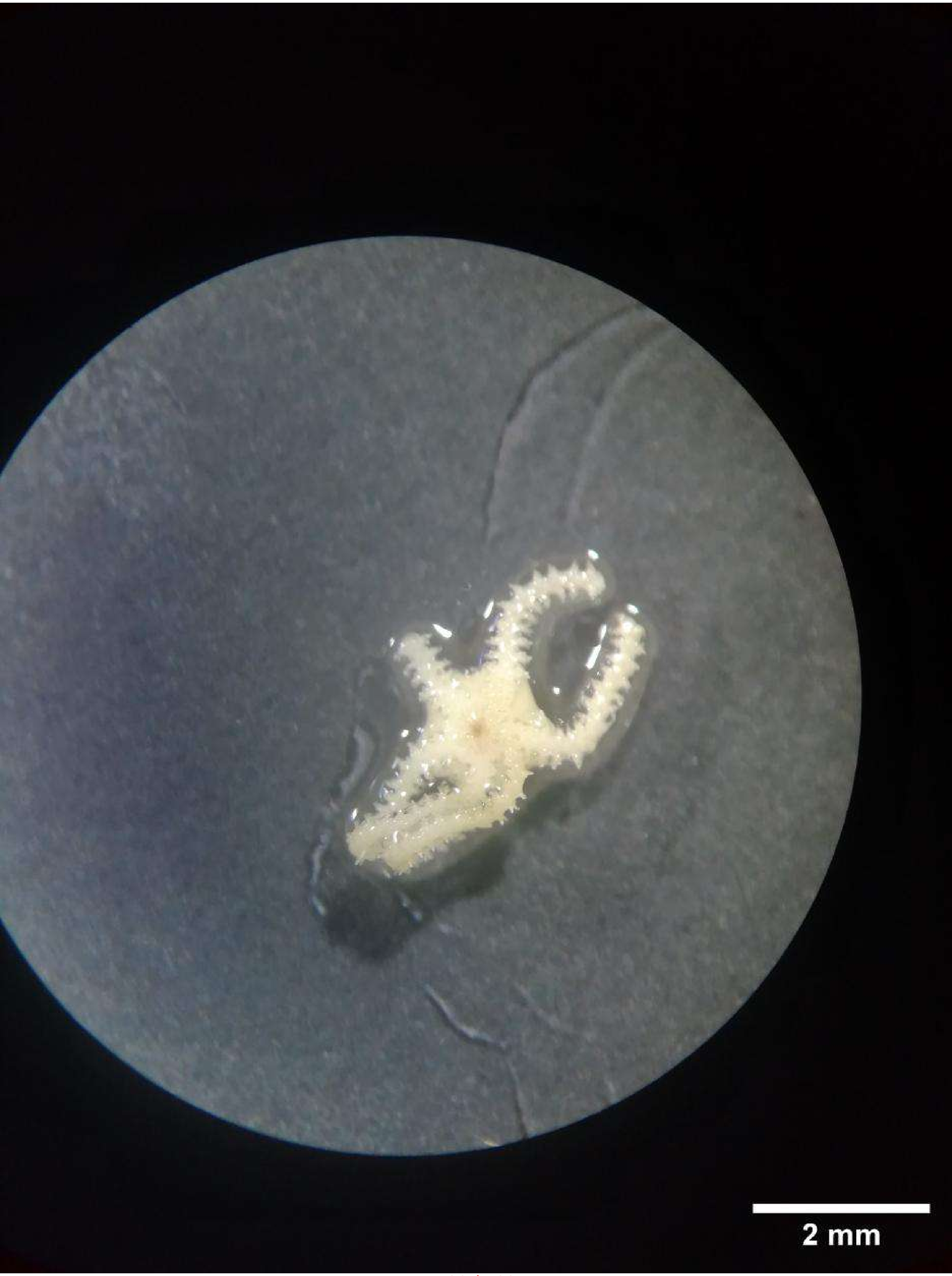
2 mm

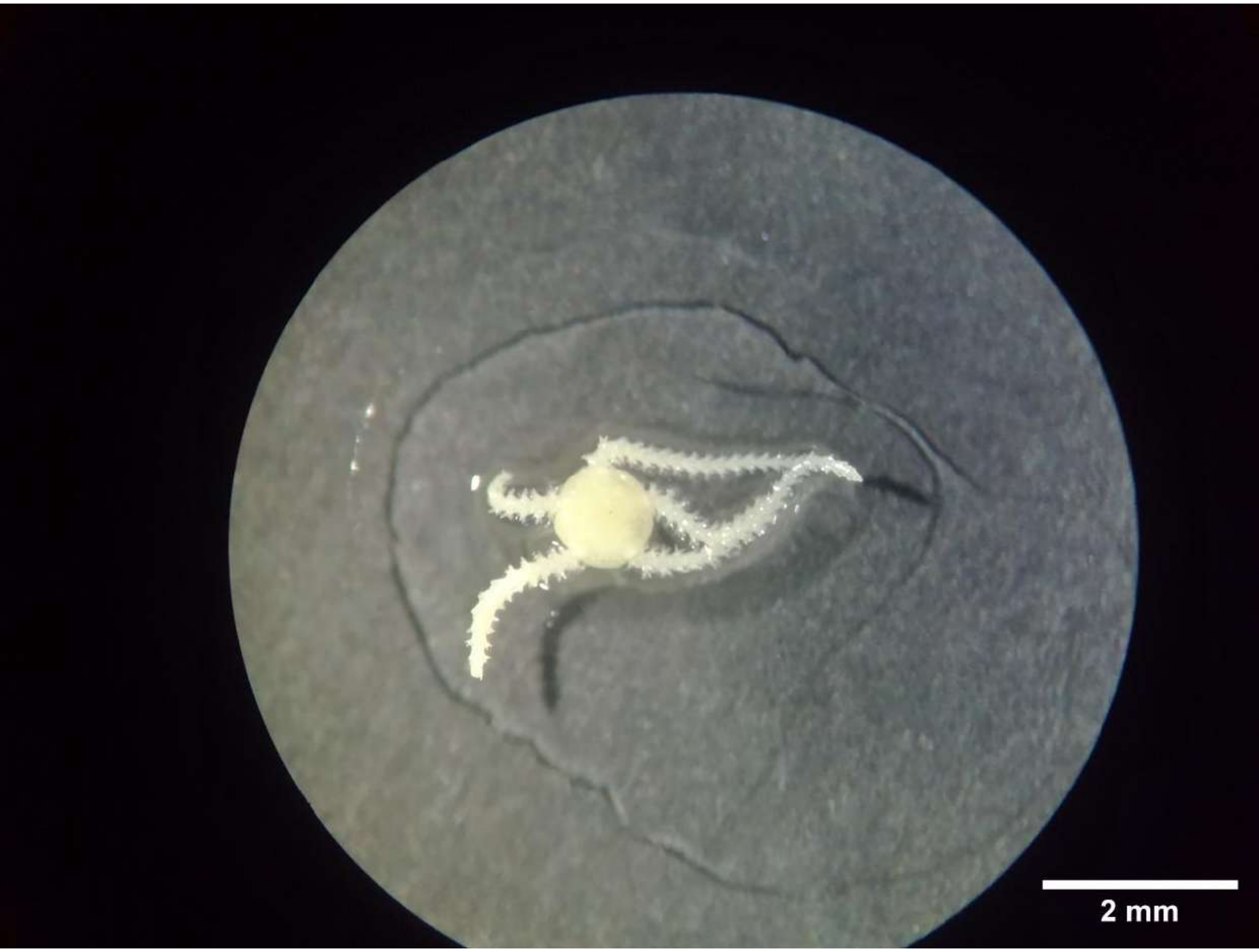
2 mm

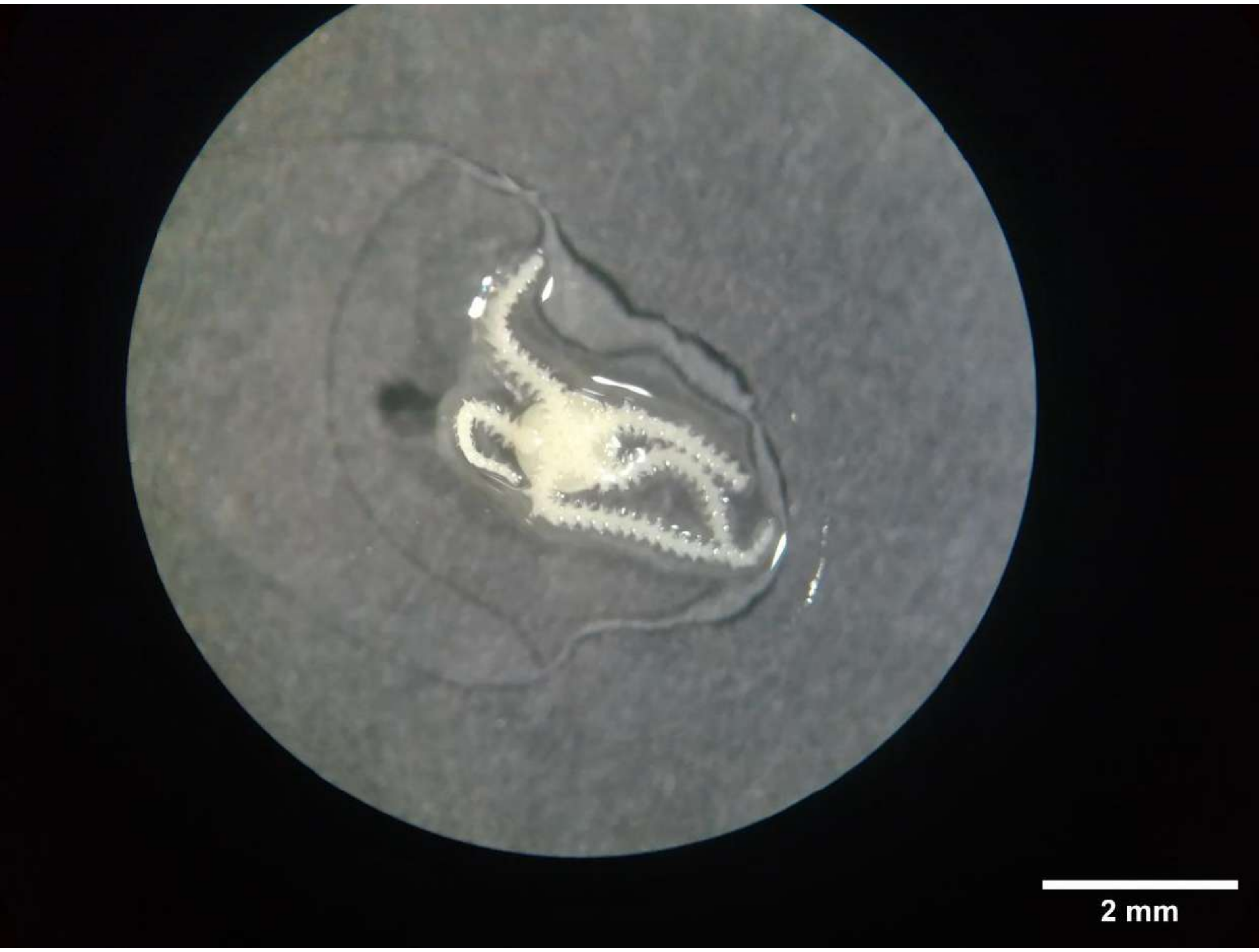
2 mm

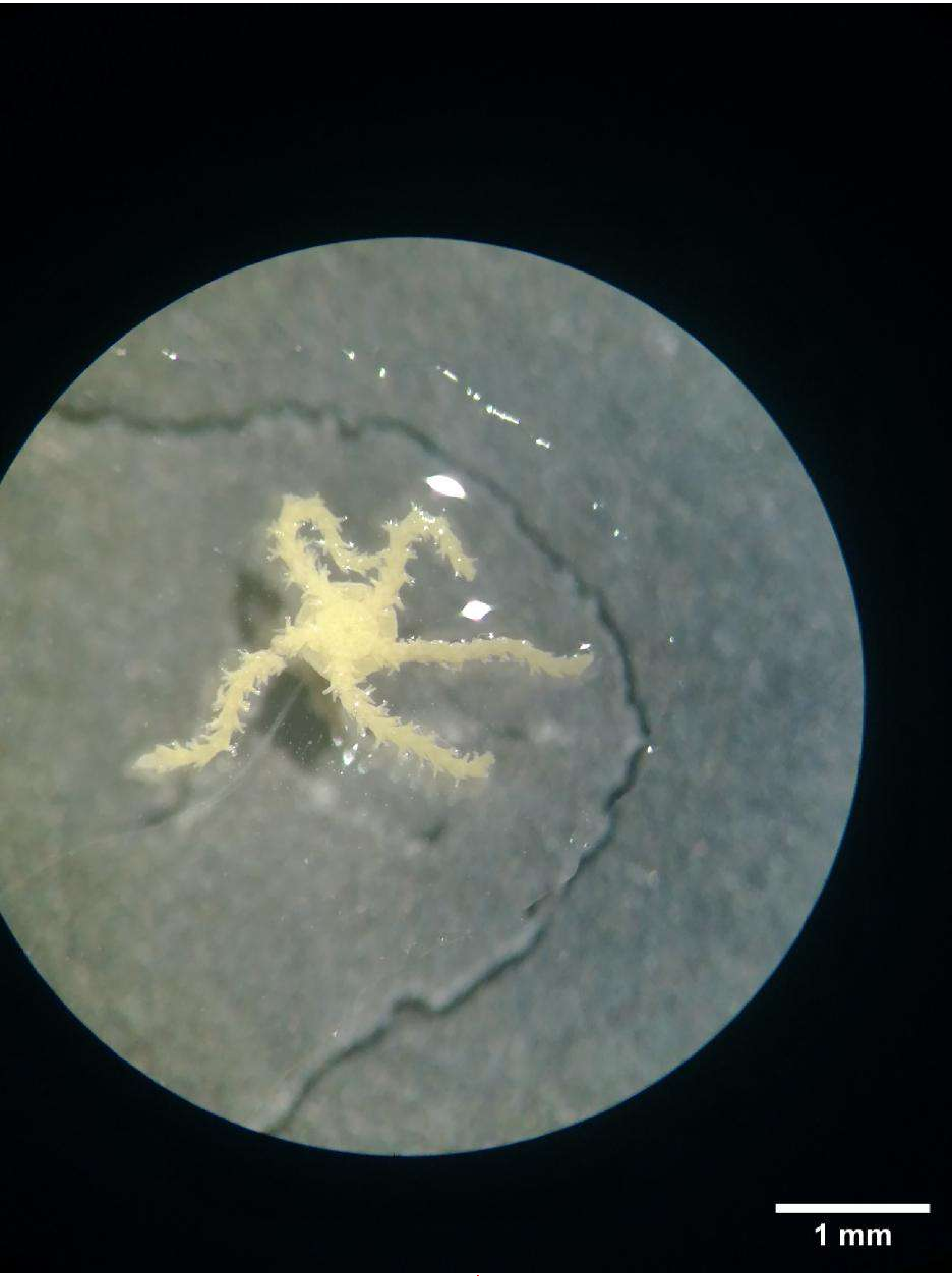
1 mm

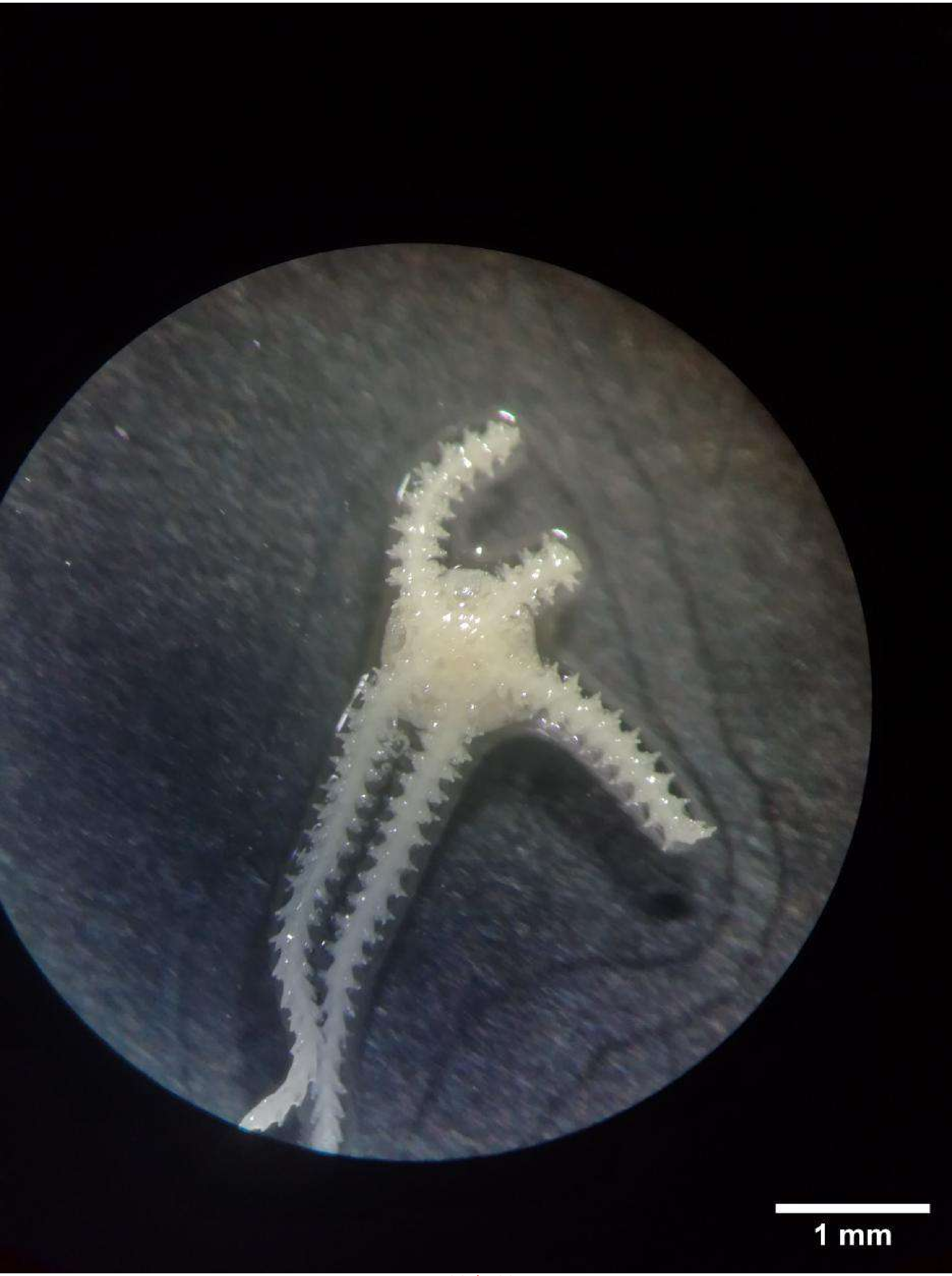
1 mm

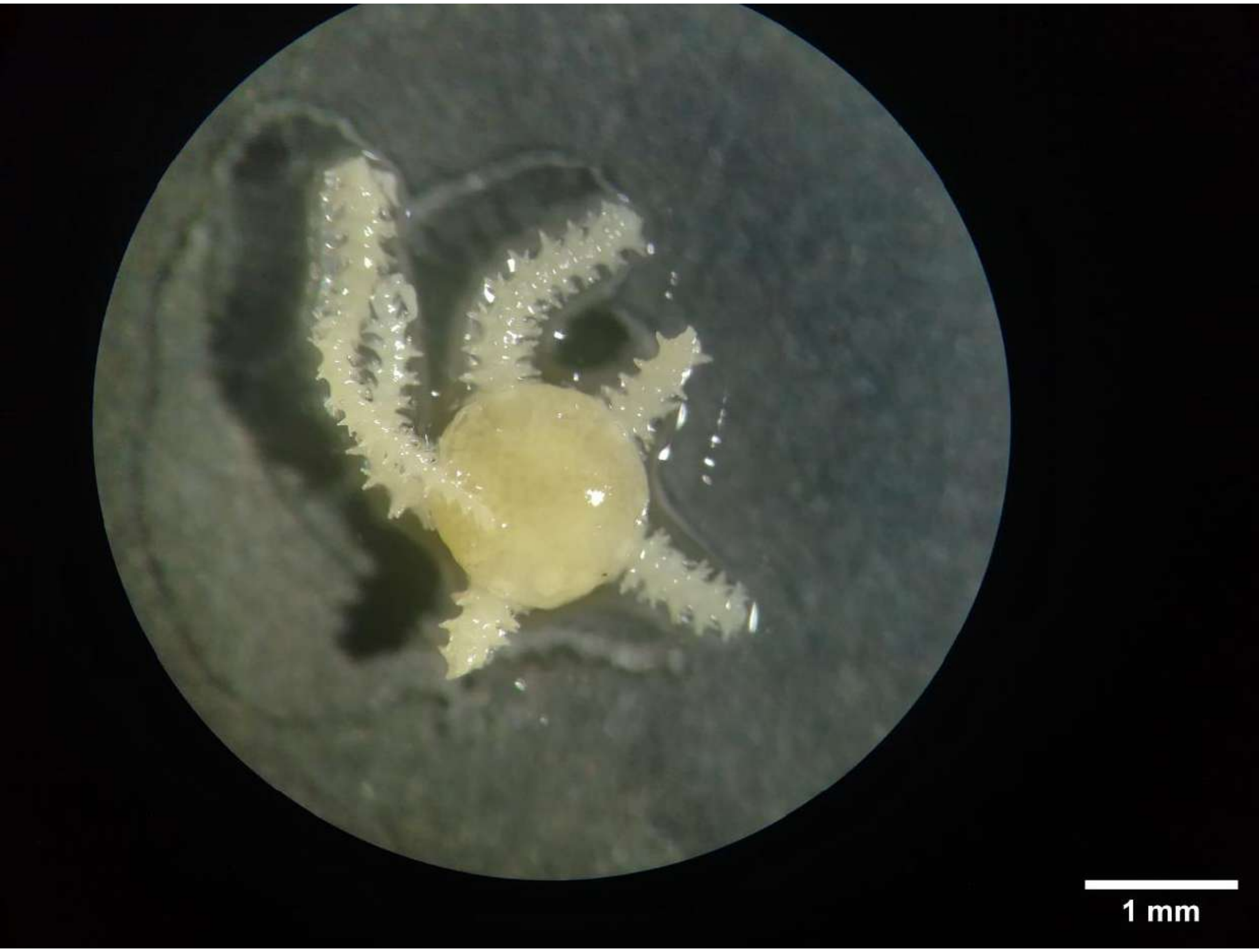
1 mm

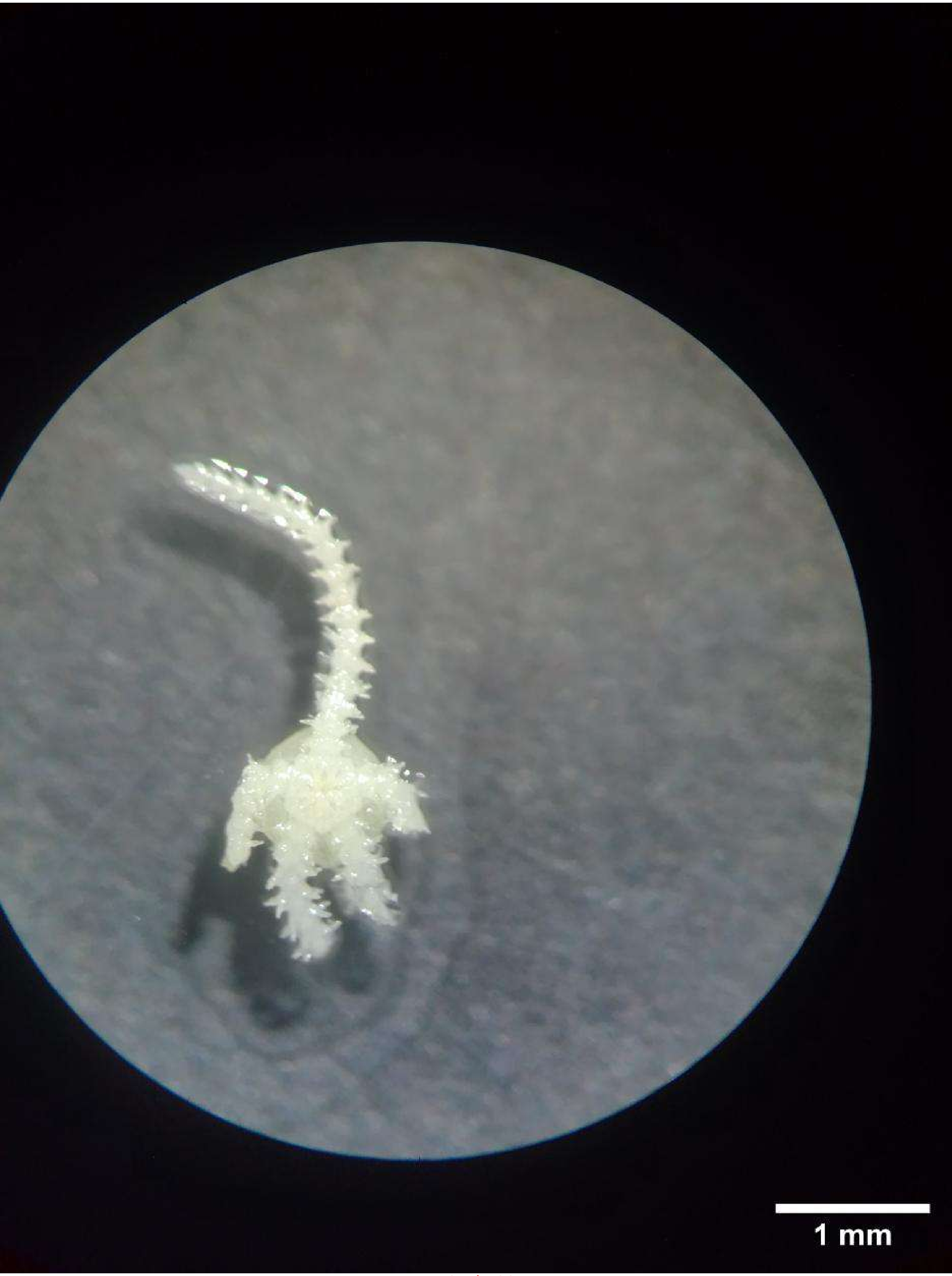
1 mm

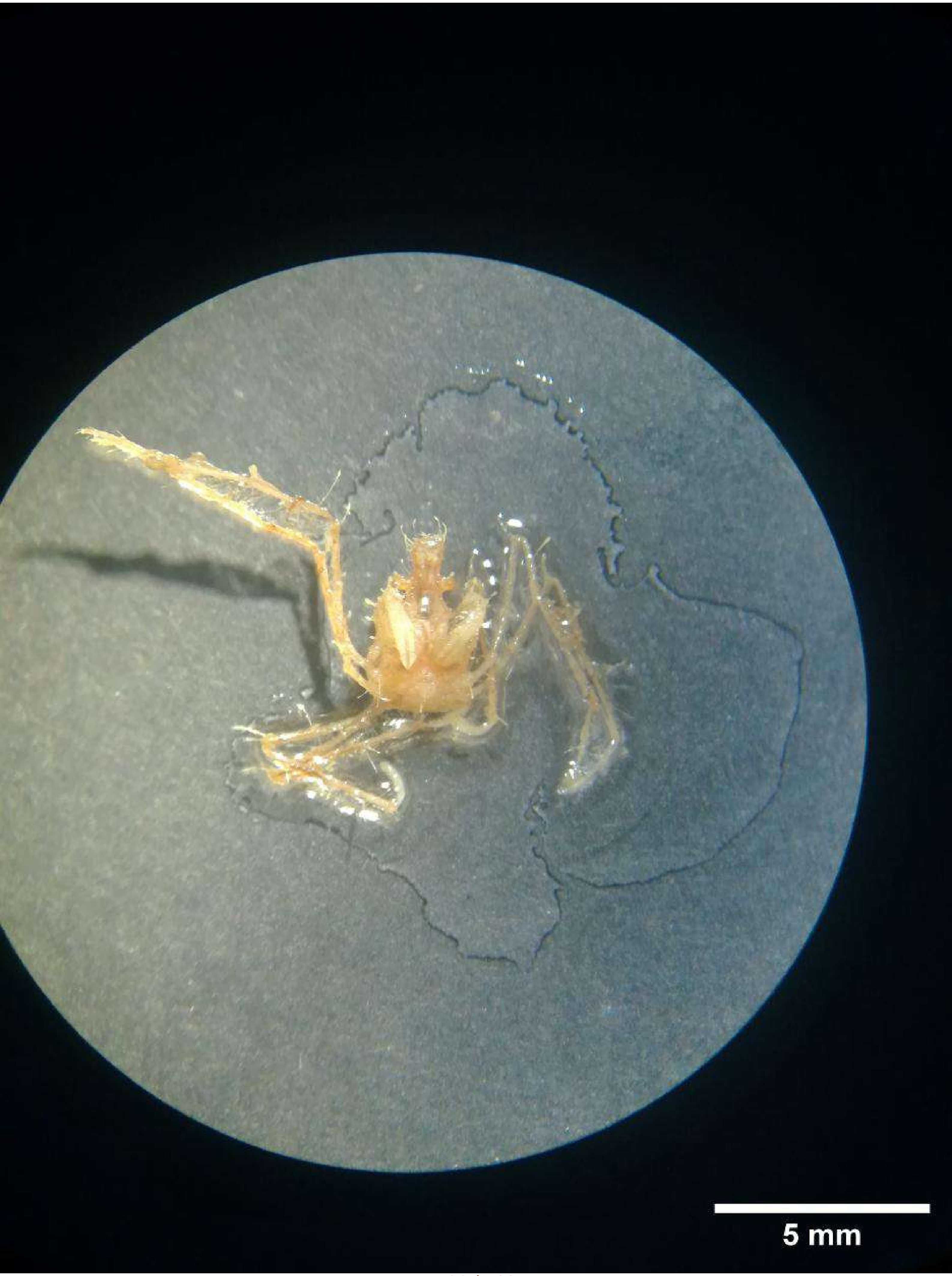
5 mm

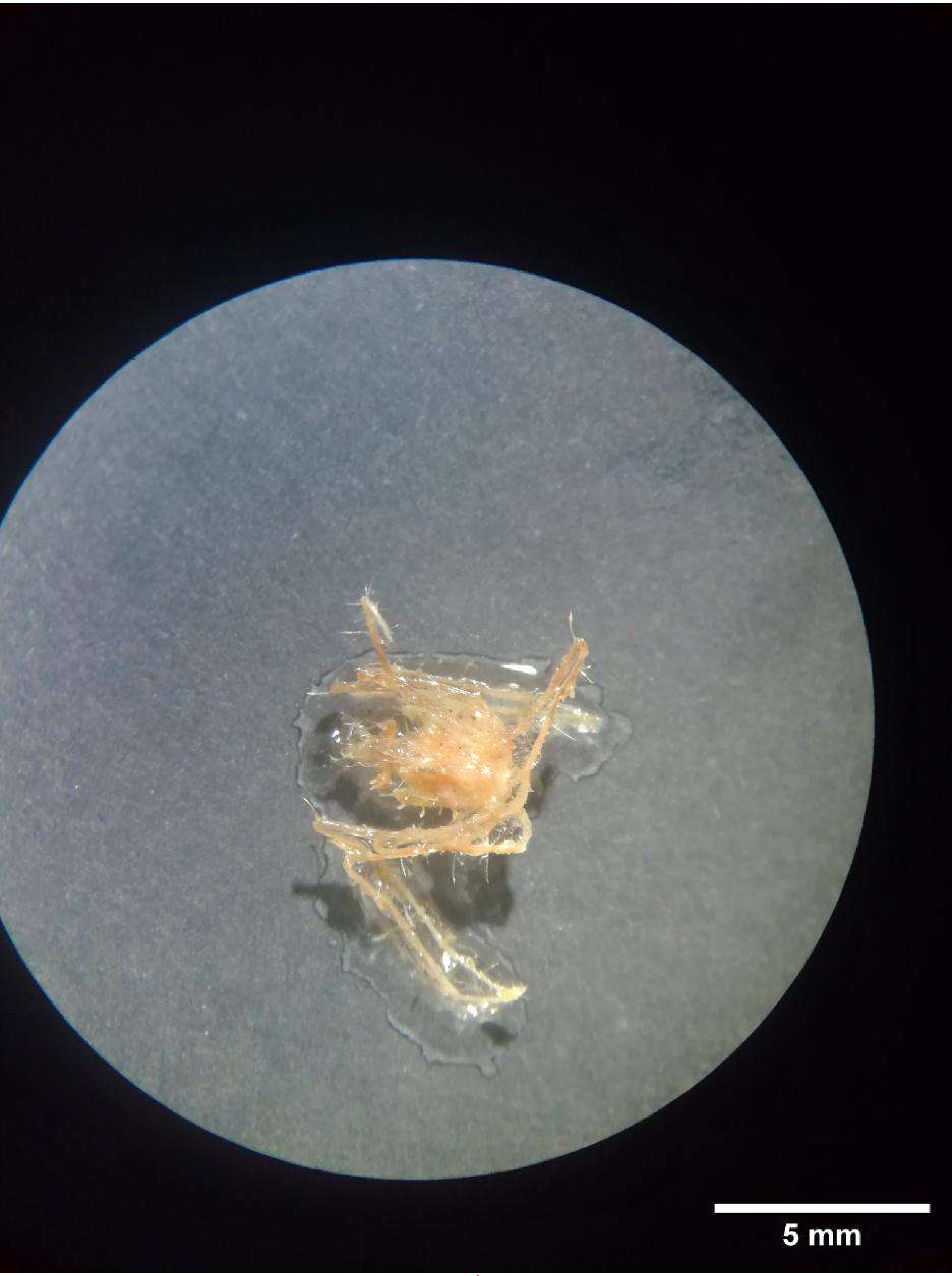
5 mm

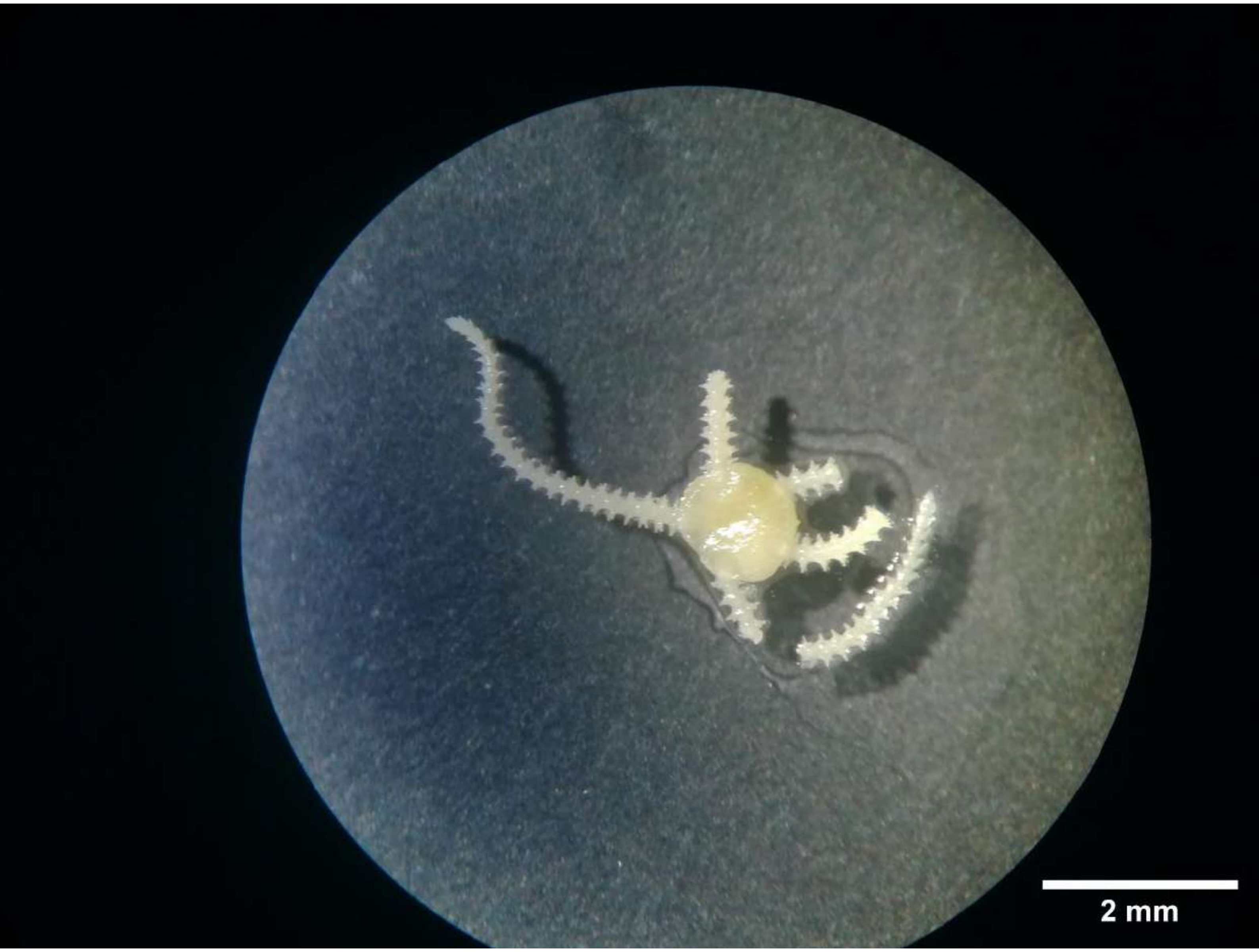
2 mm

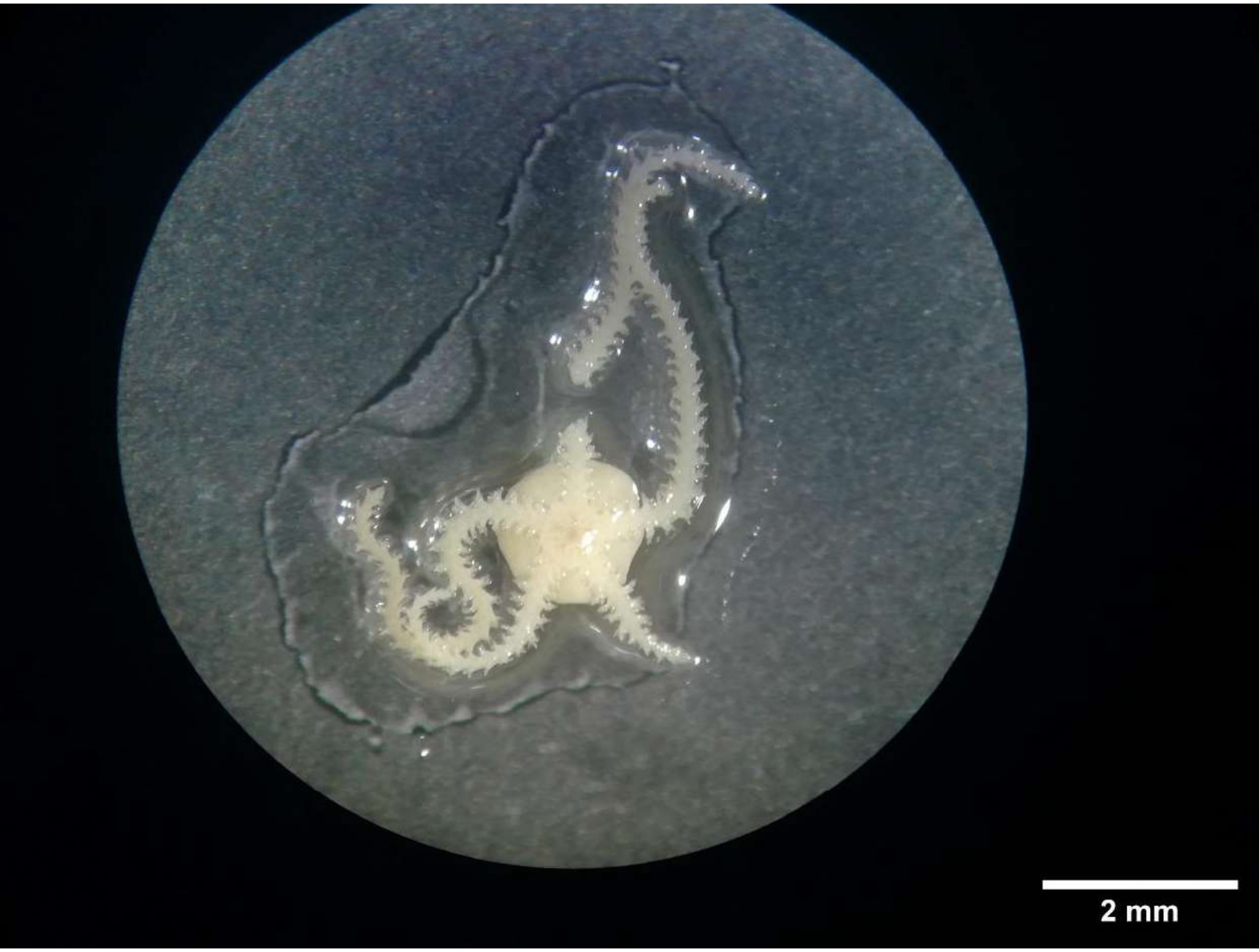
2 mm

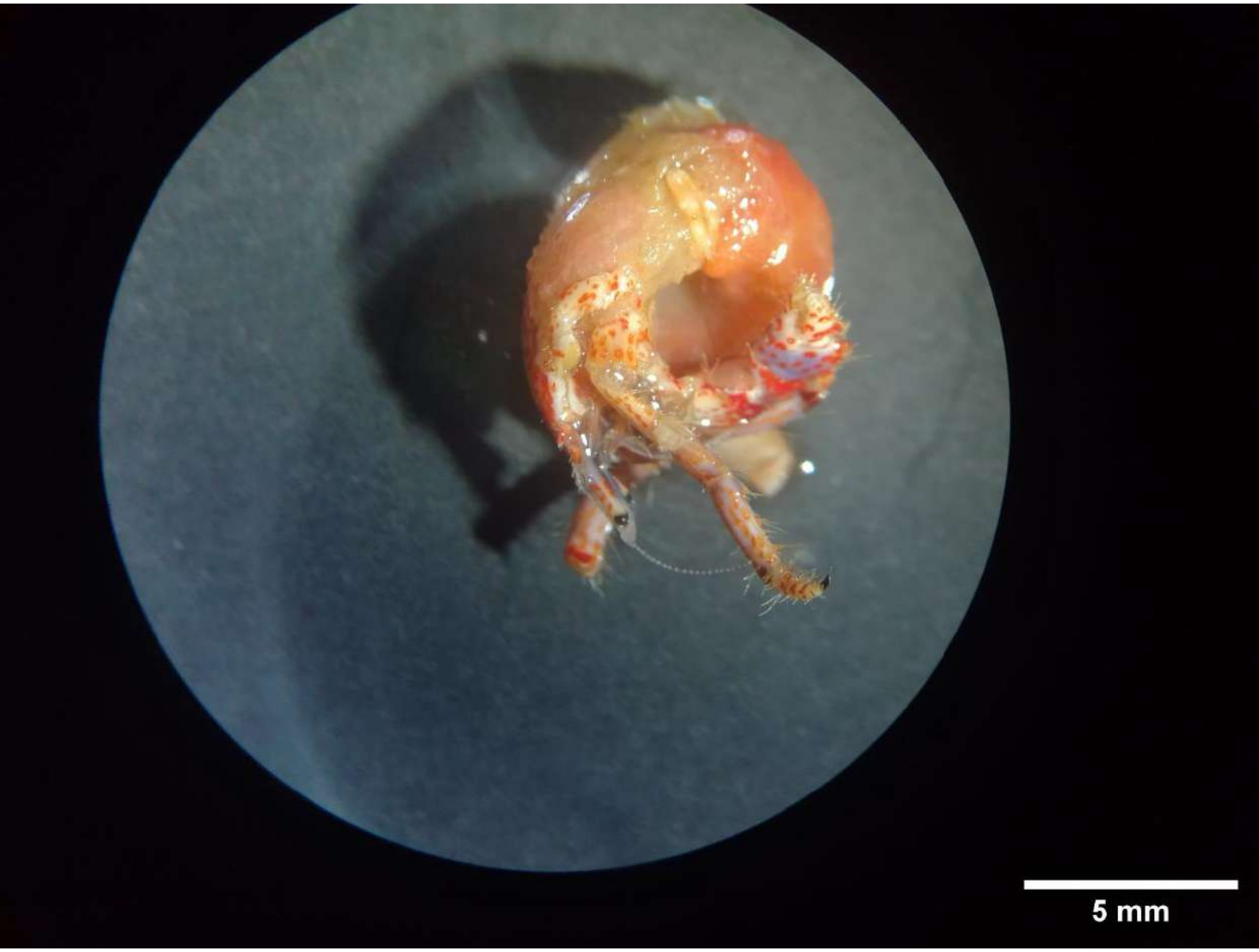
5 mm

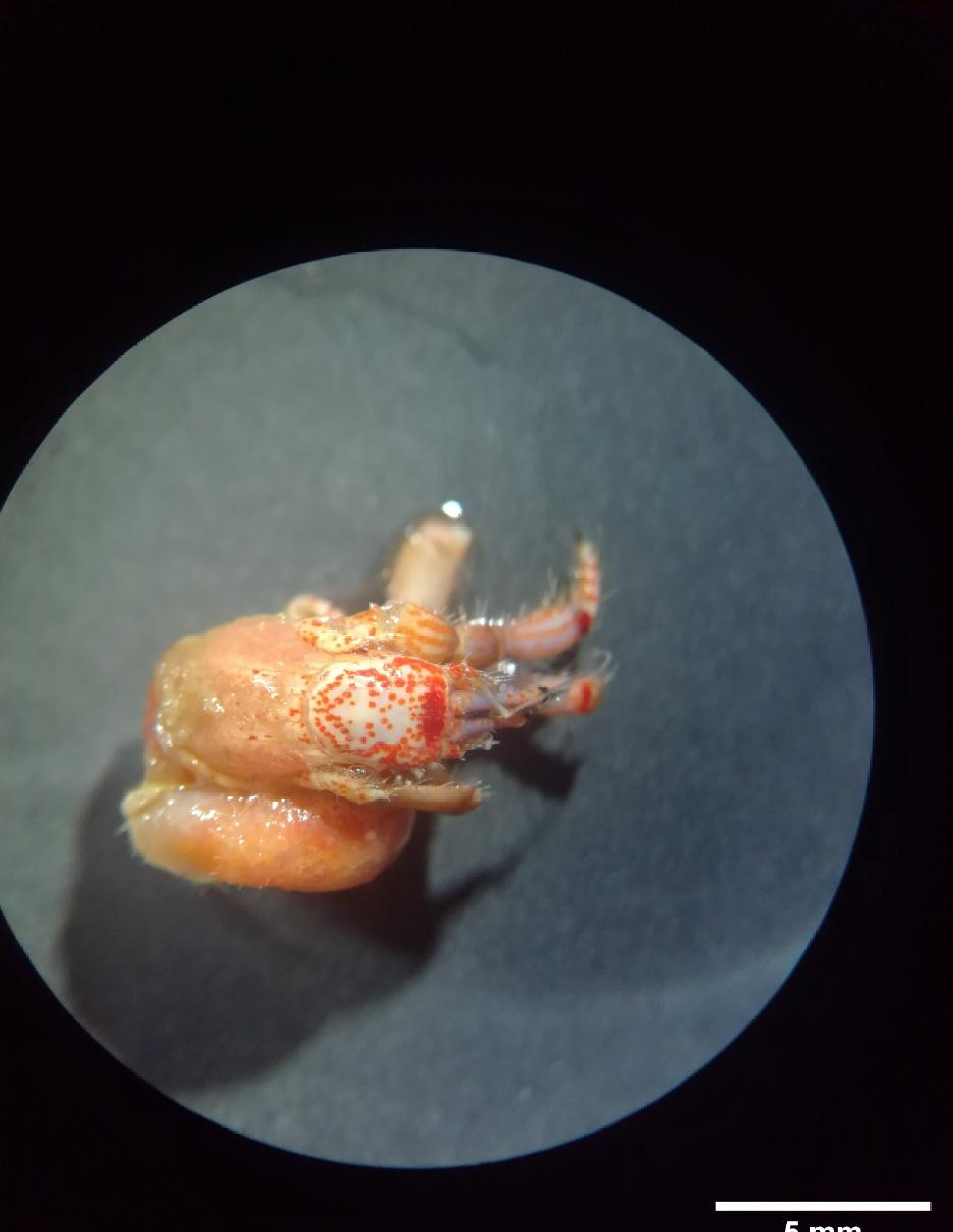
5 mm

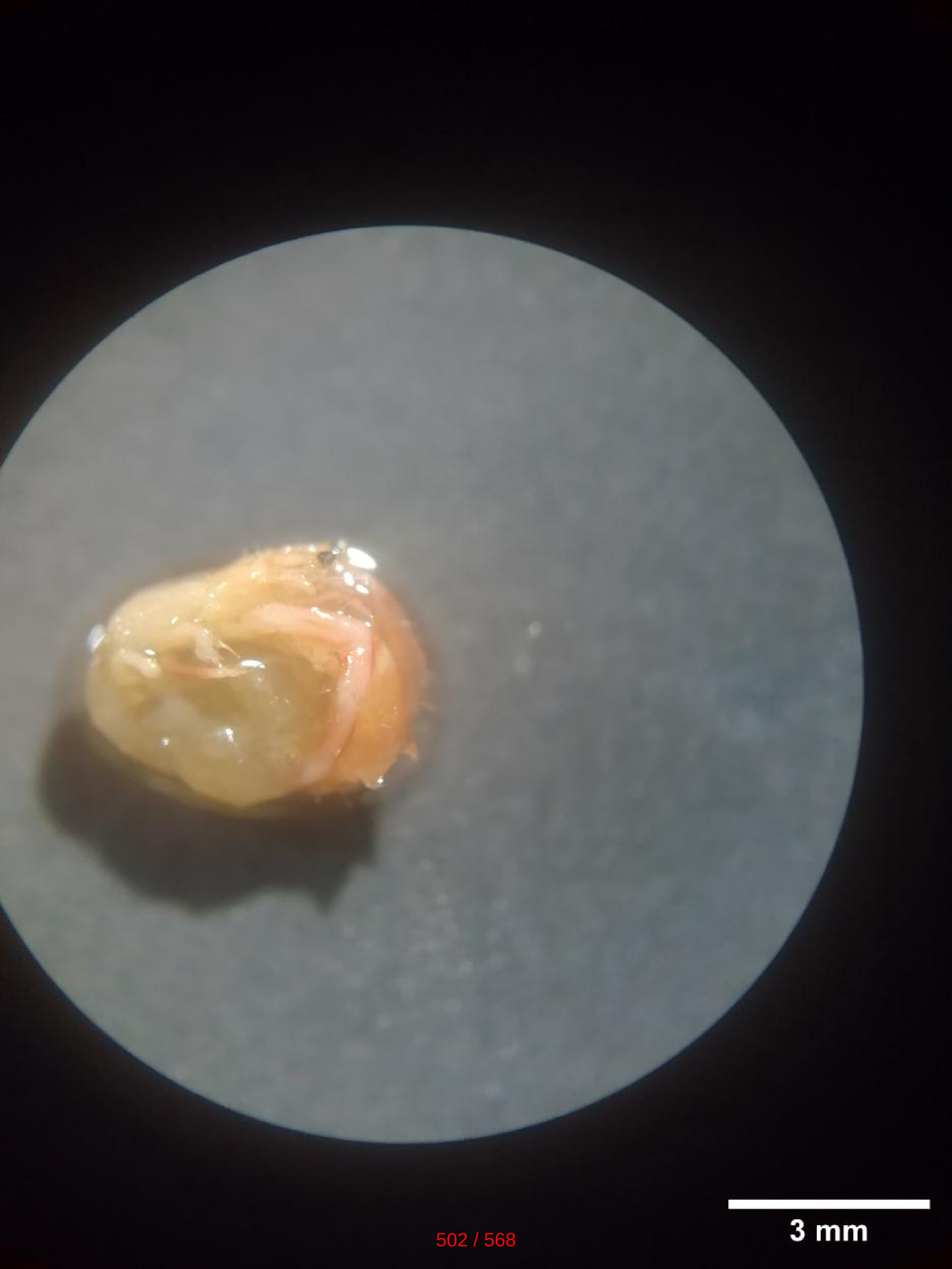
3 mm

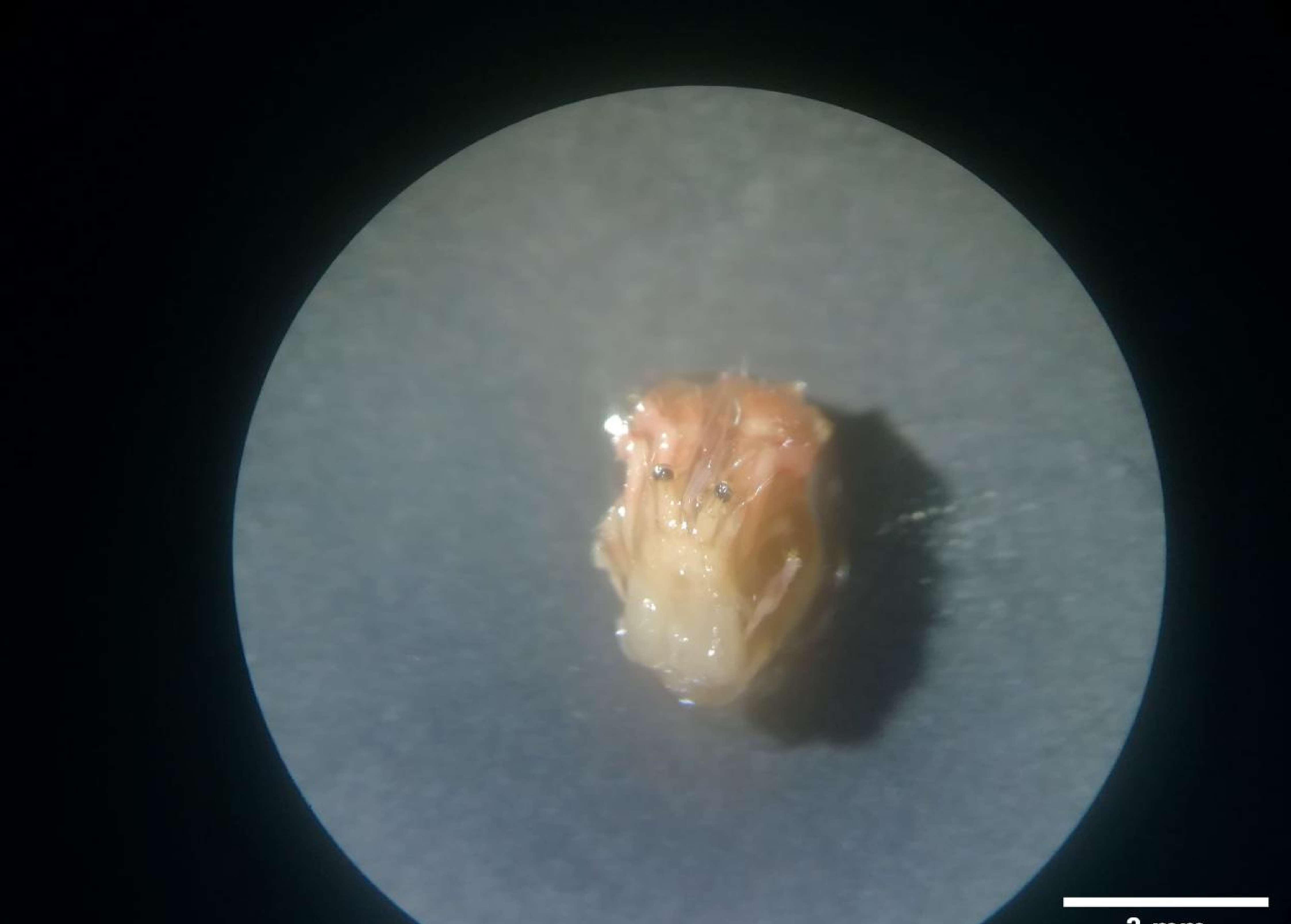
3 mm

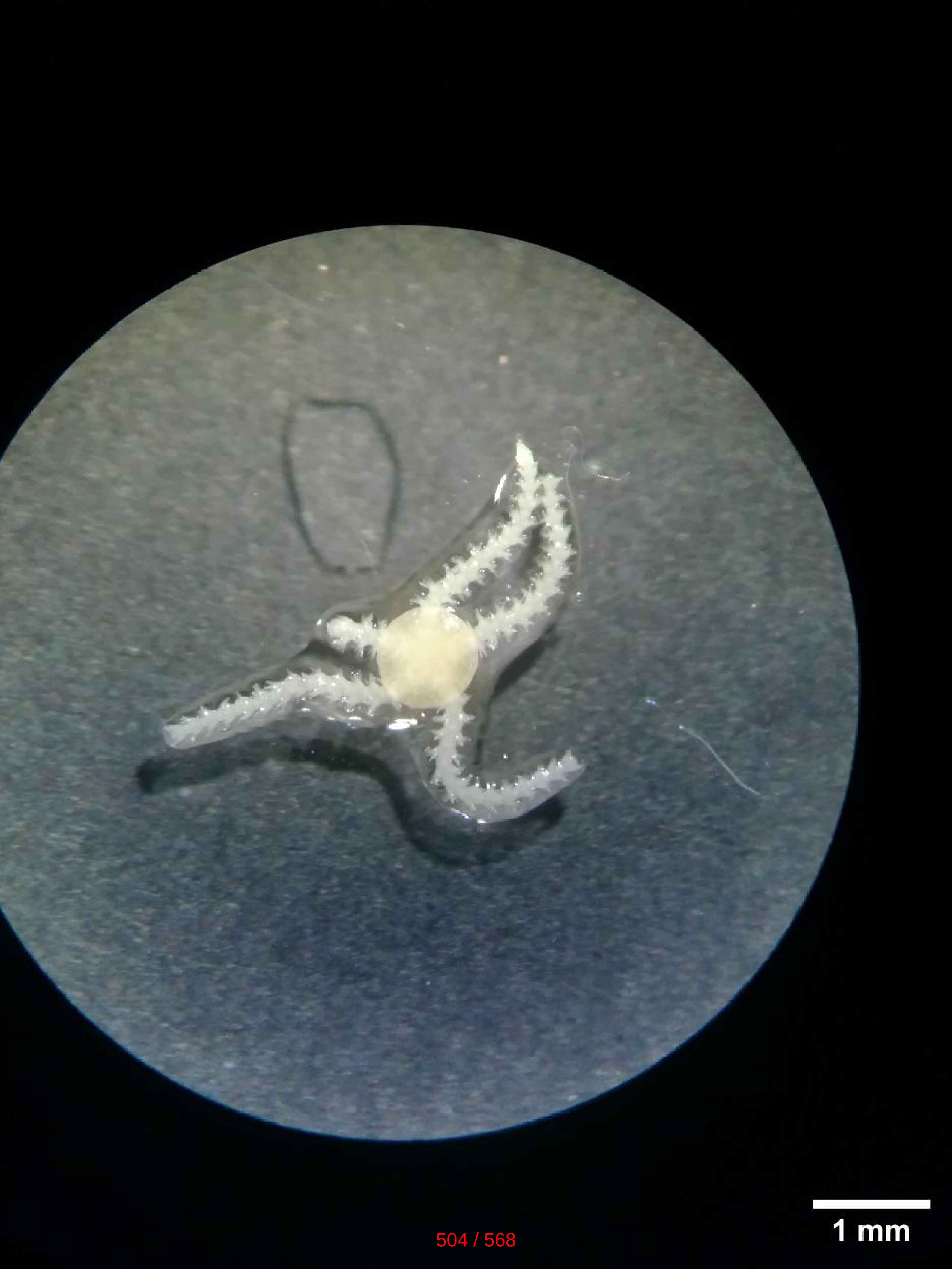
1 mm

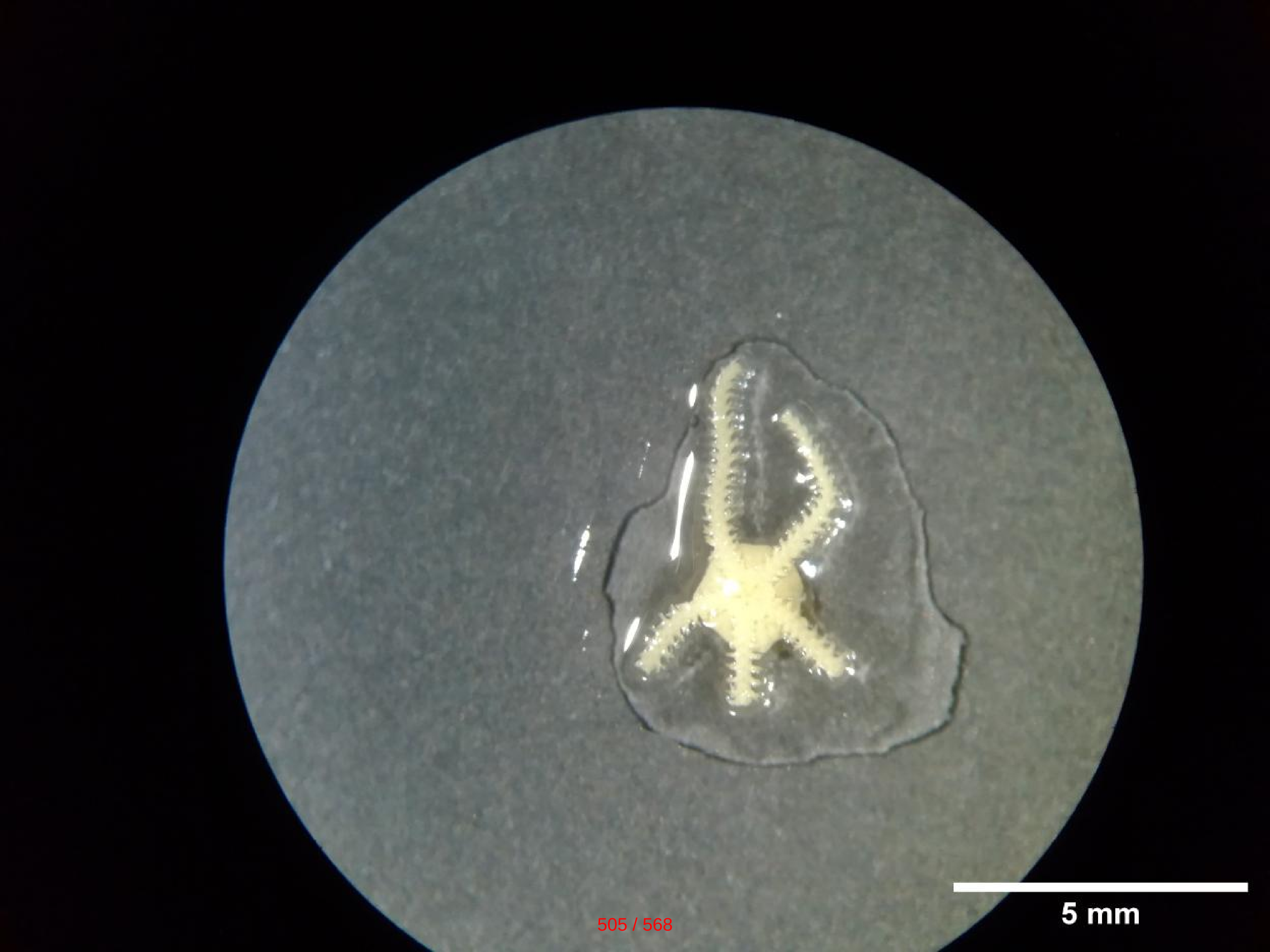
5 mm

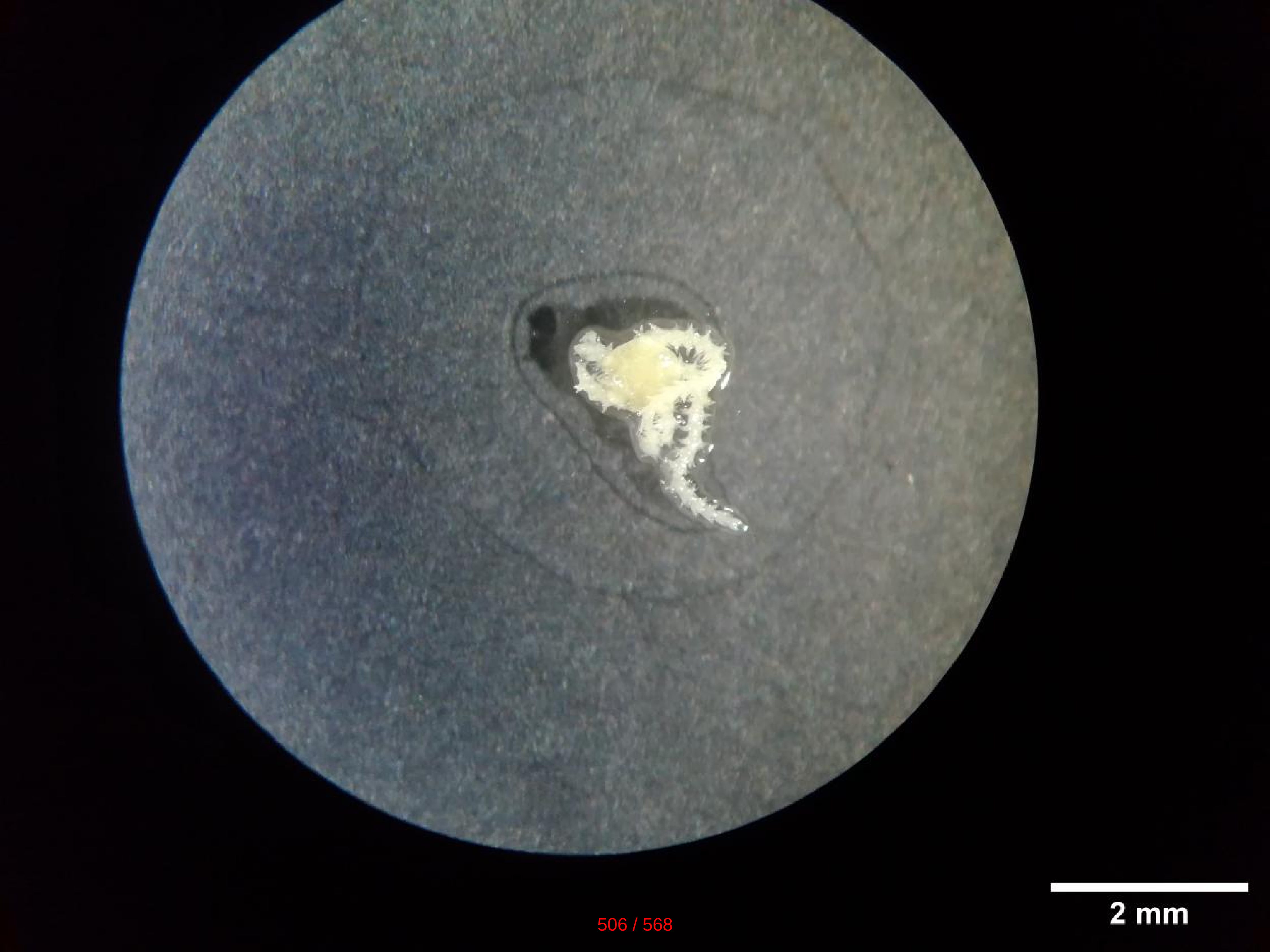
2 mm

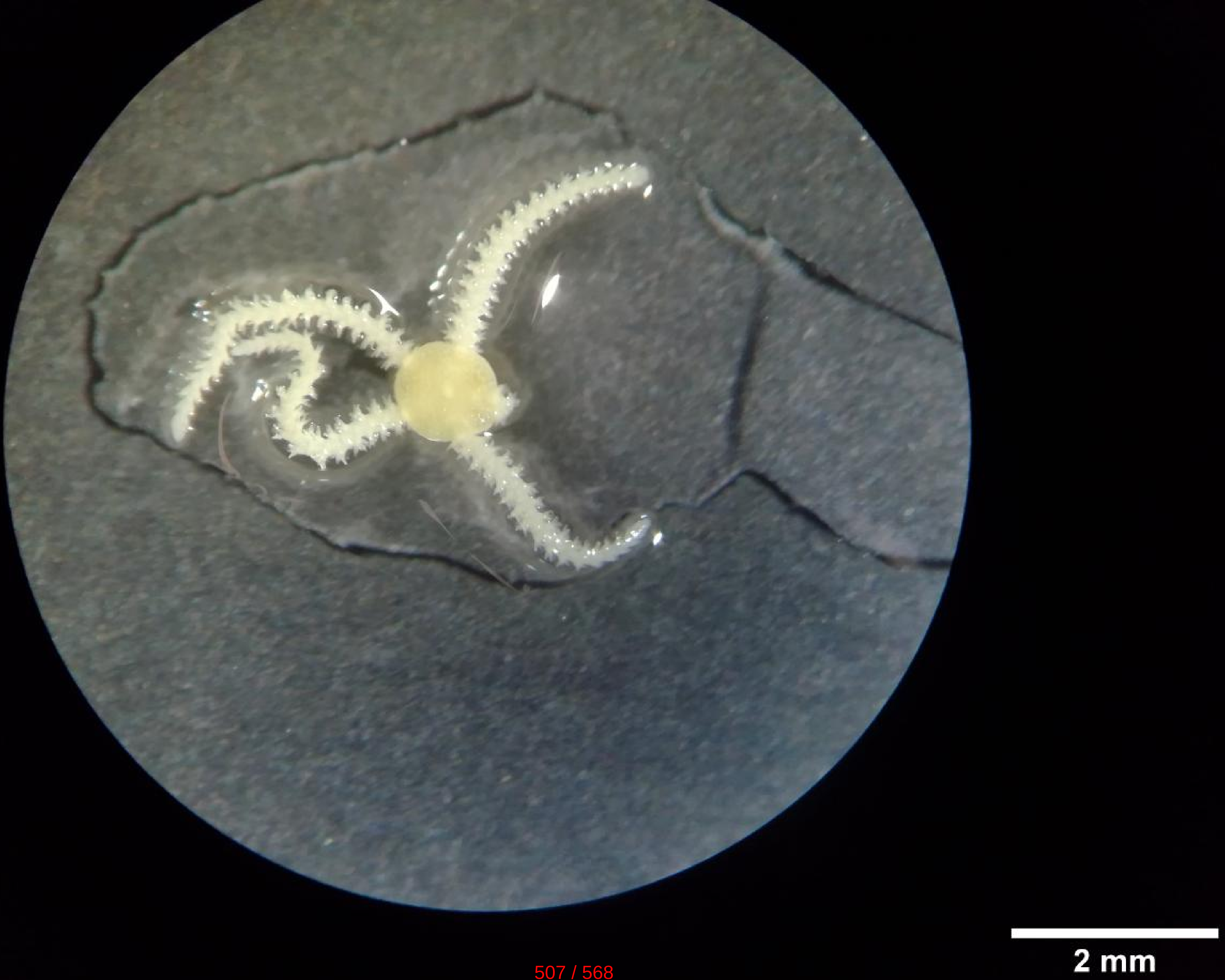
2 mm

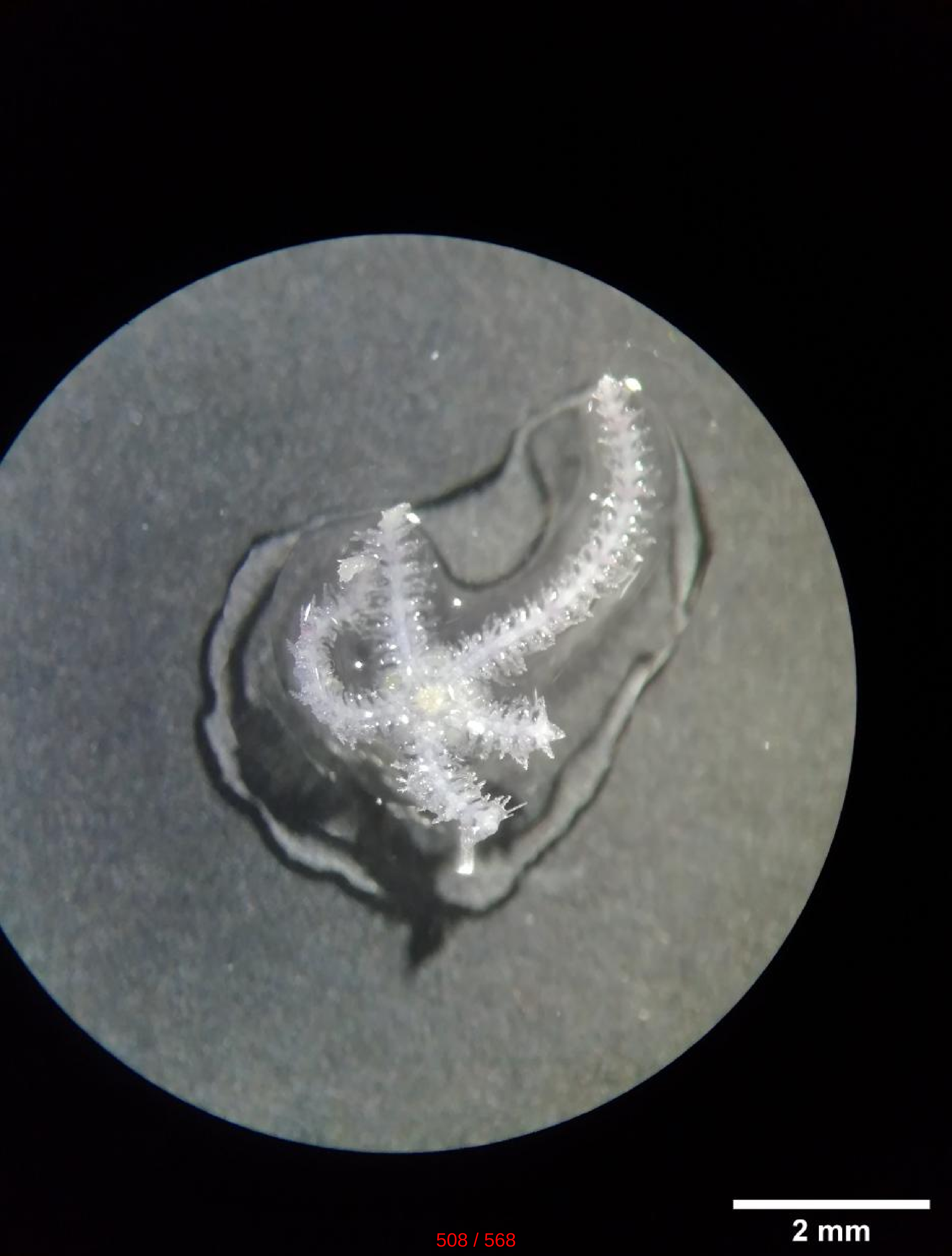
2 mm

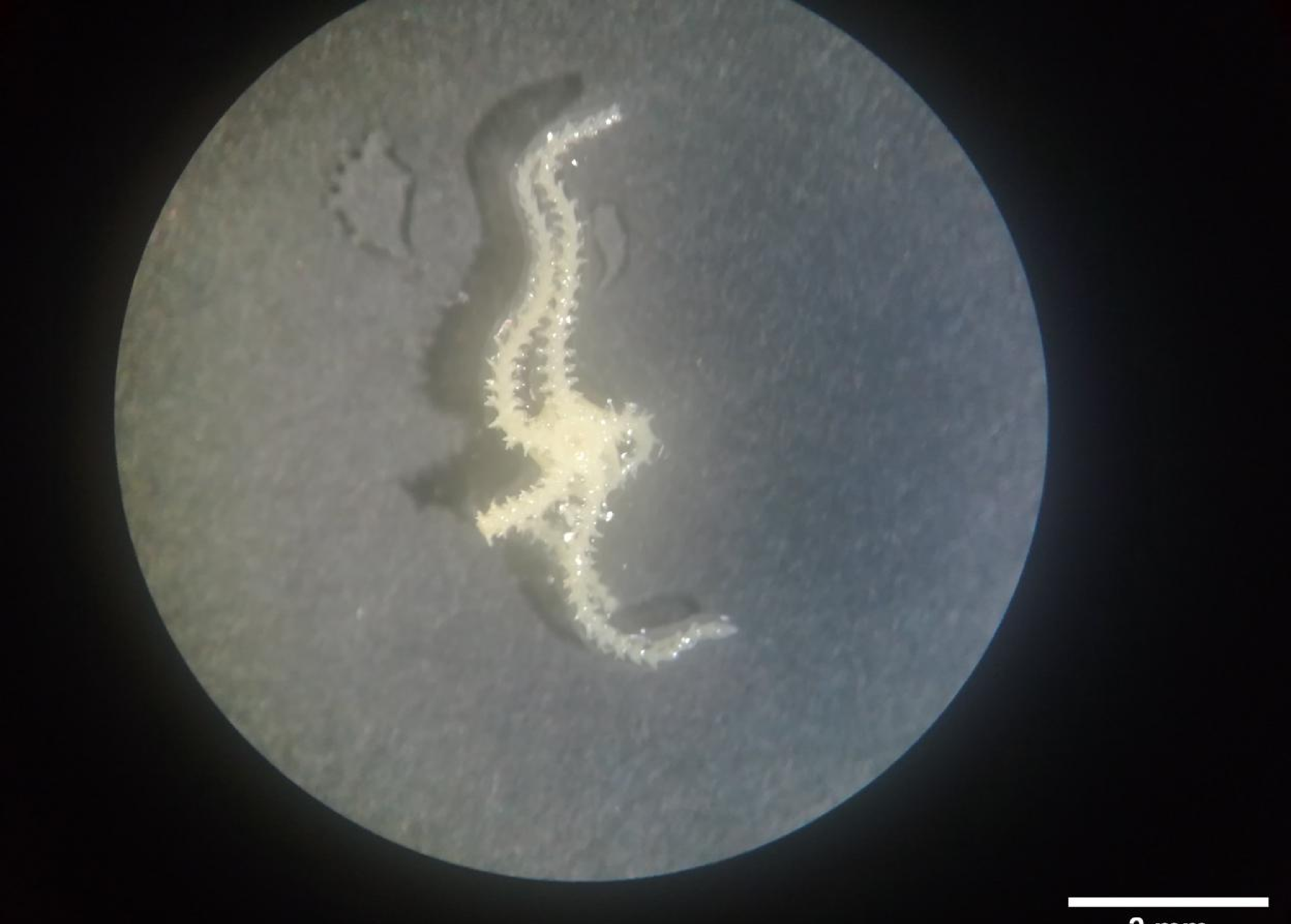
2 mm

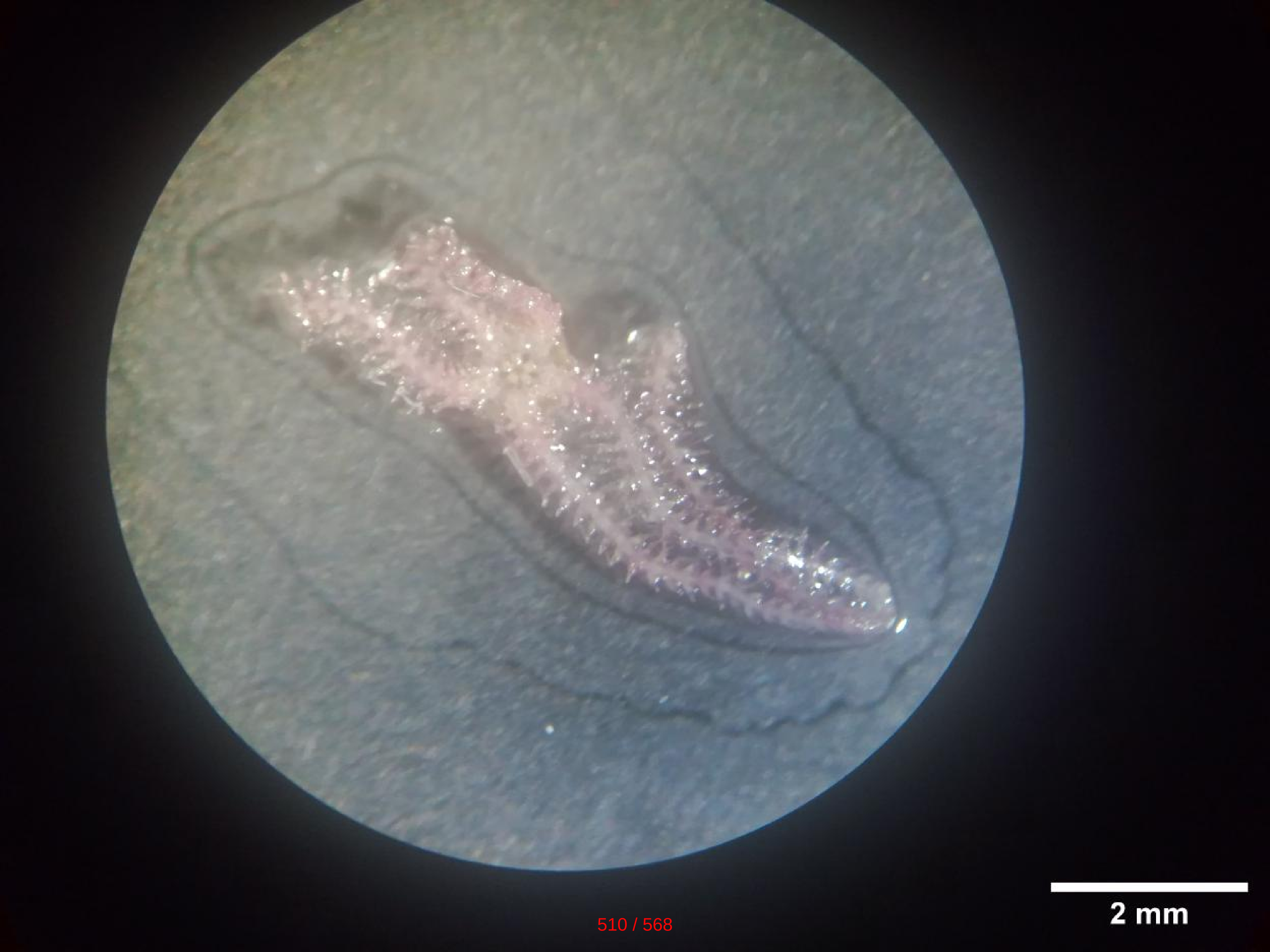
2 mm

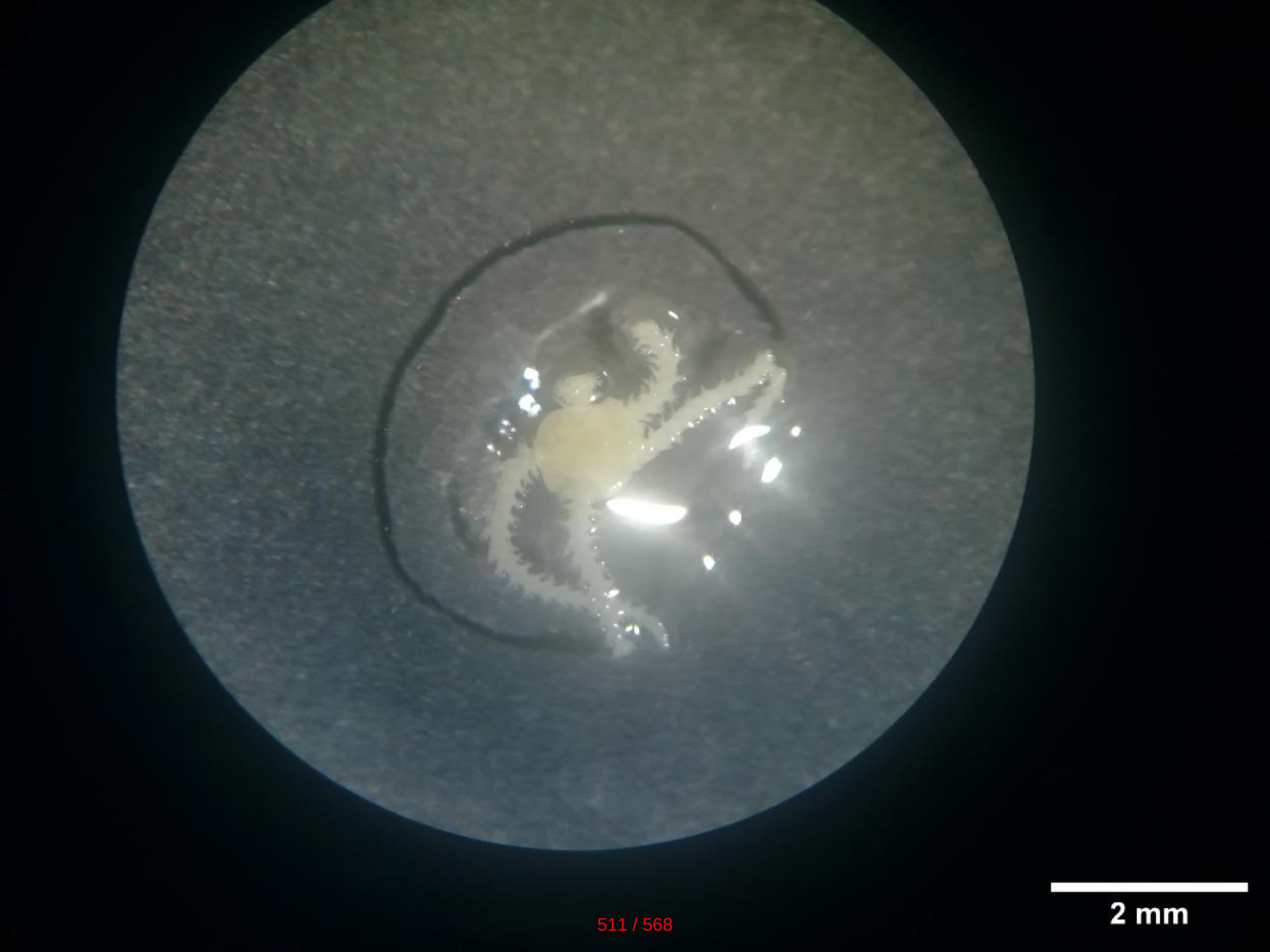
2 mm

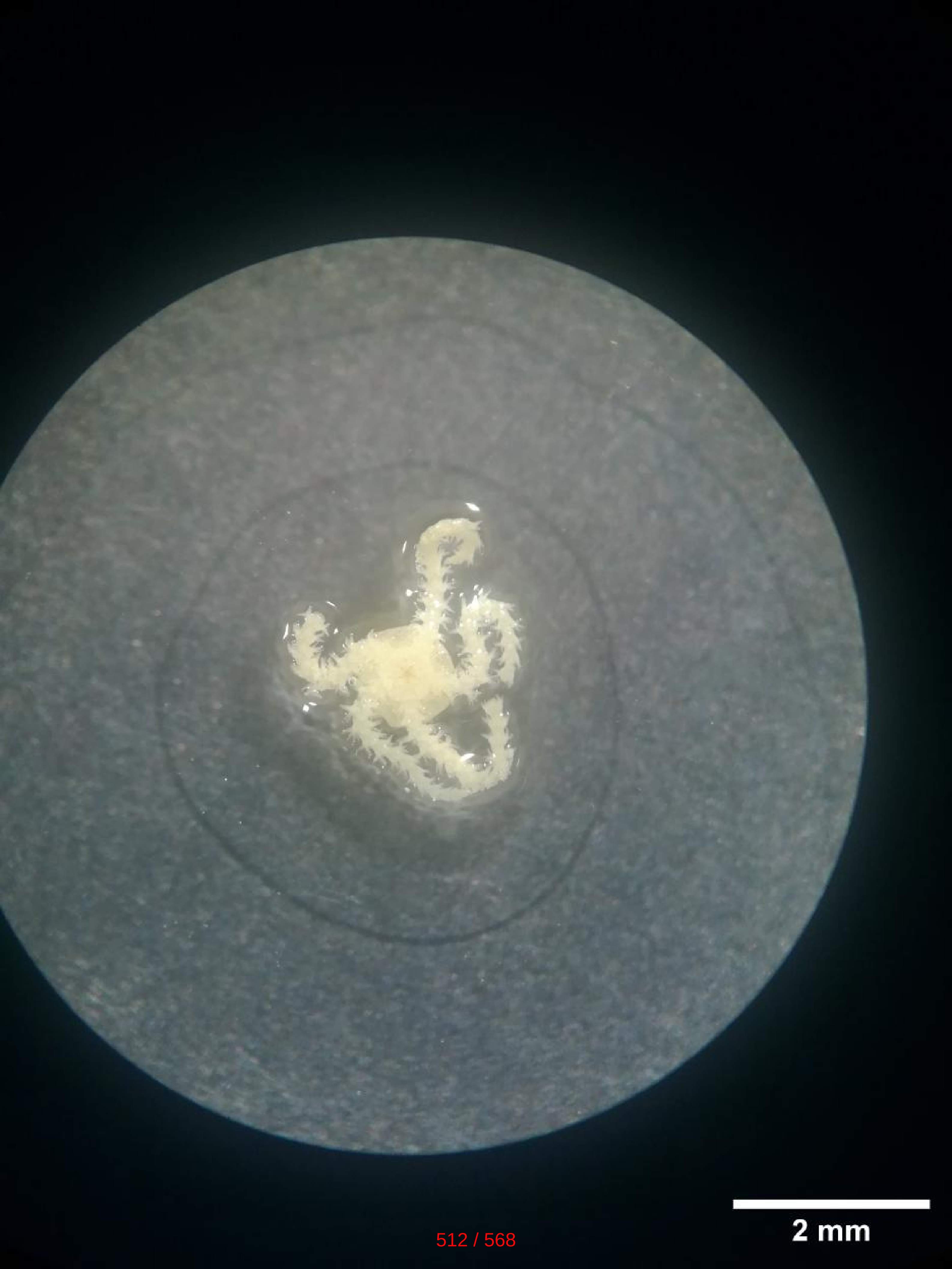
2 mm

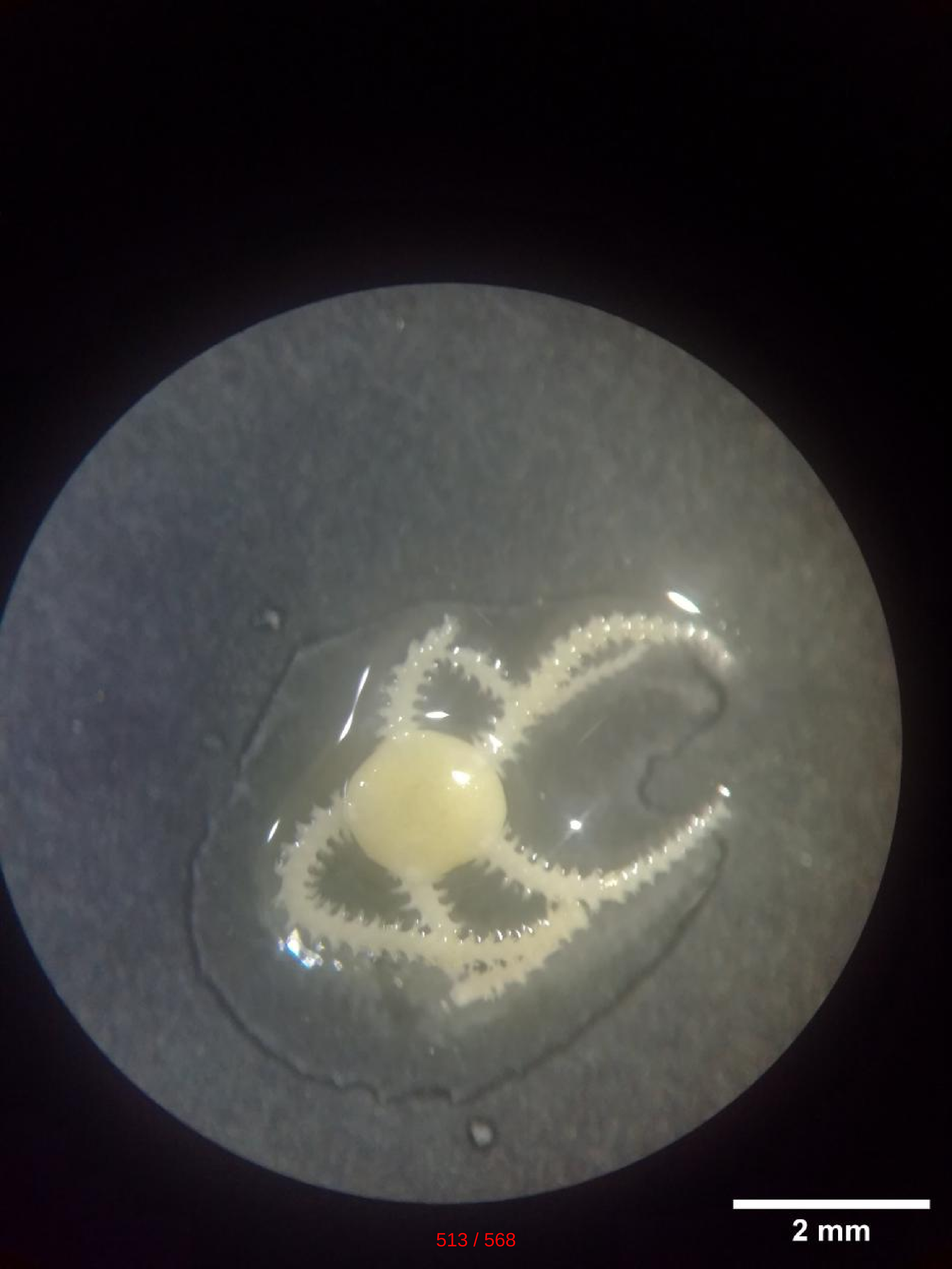
2 mm

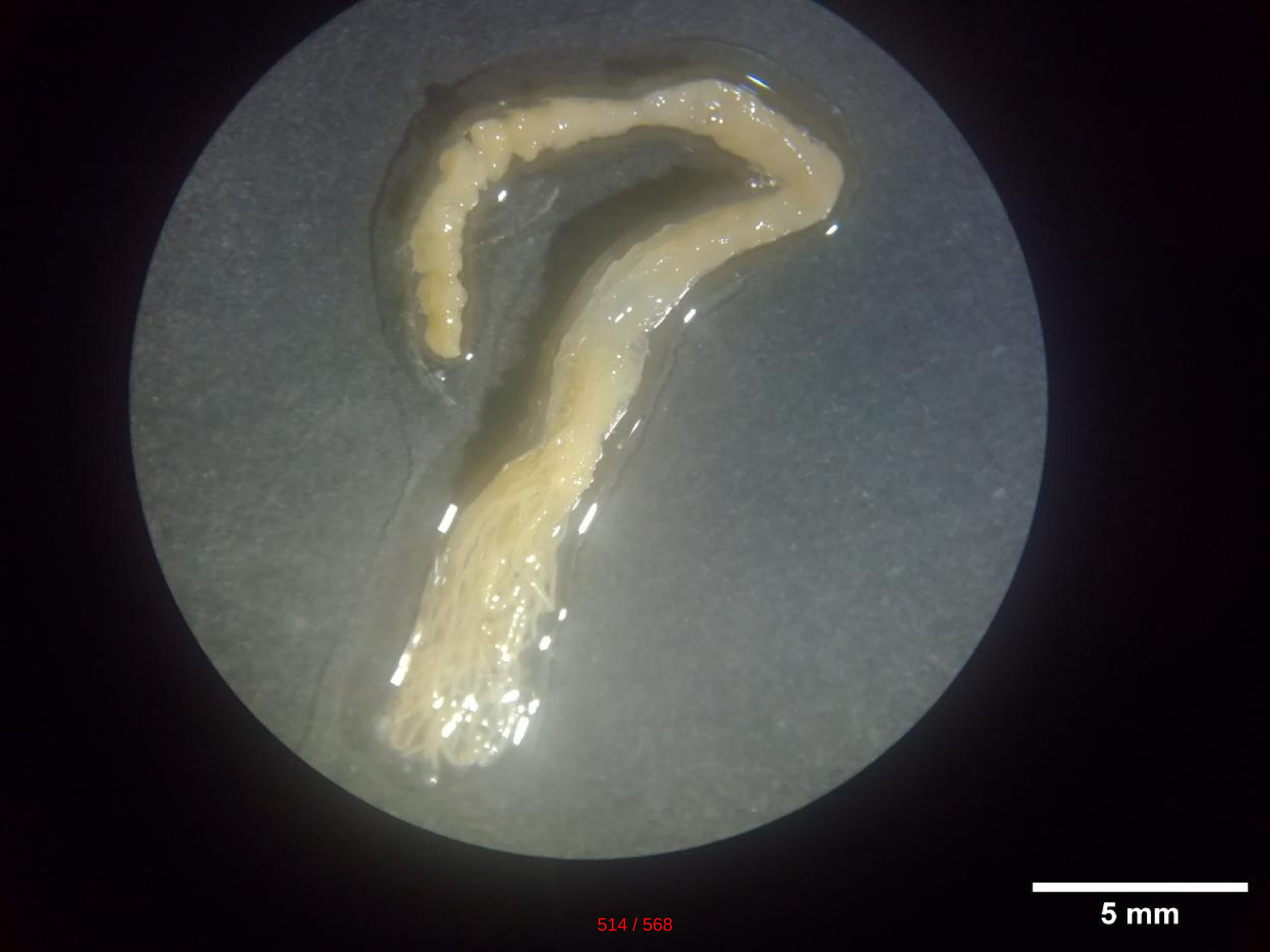
5 mm

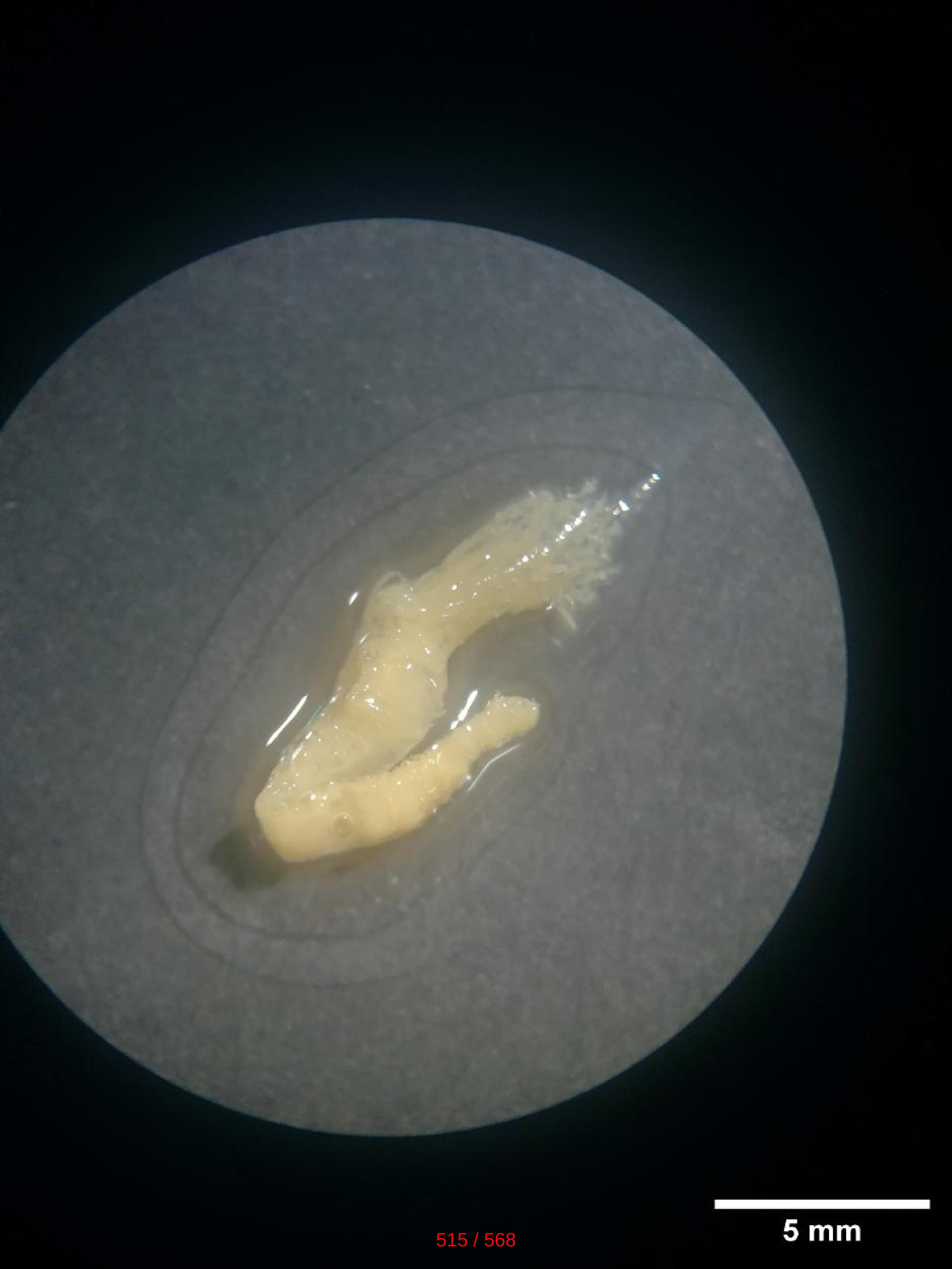
5 mm

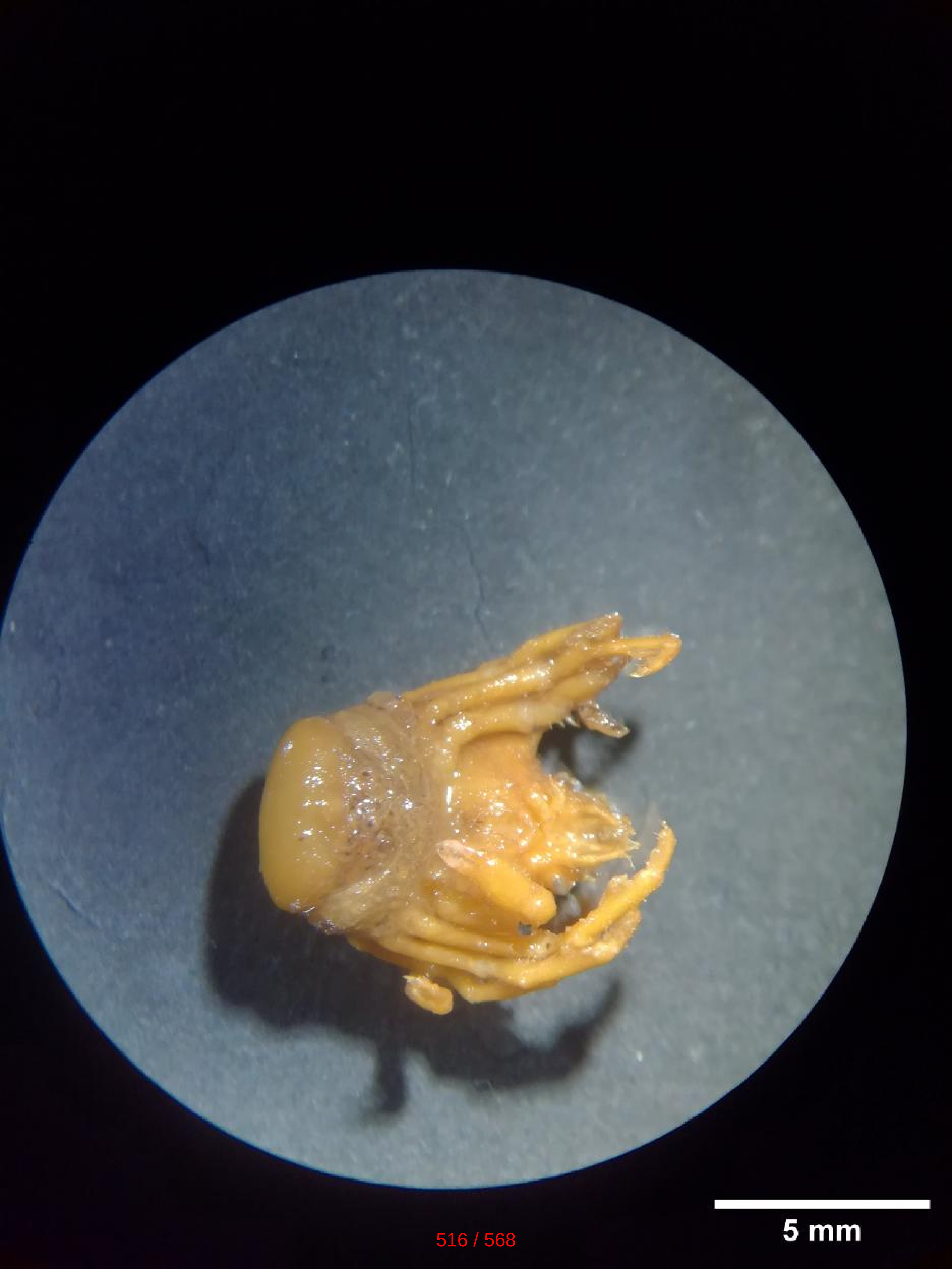
5 mm

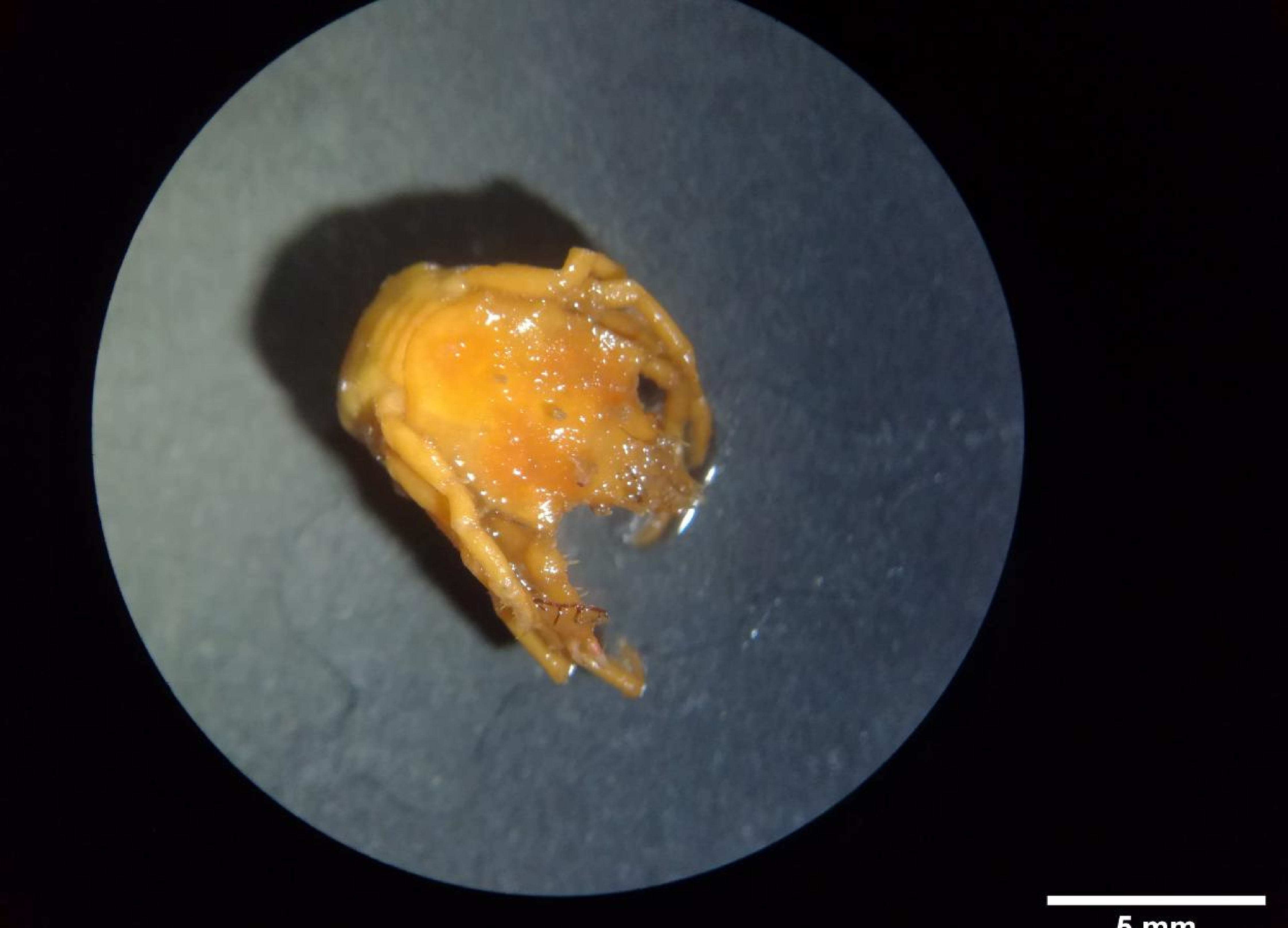
5 mm

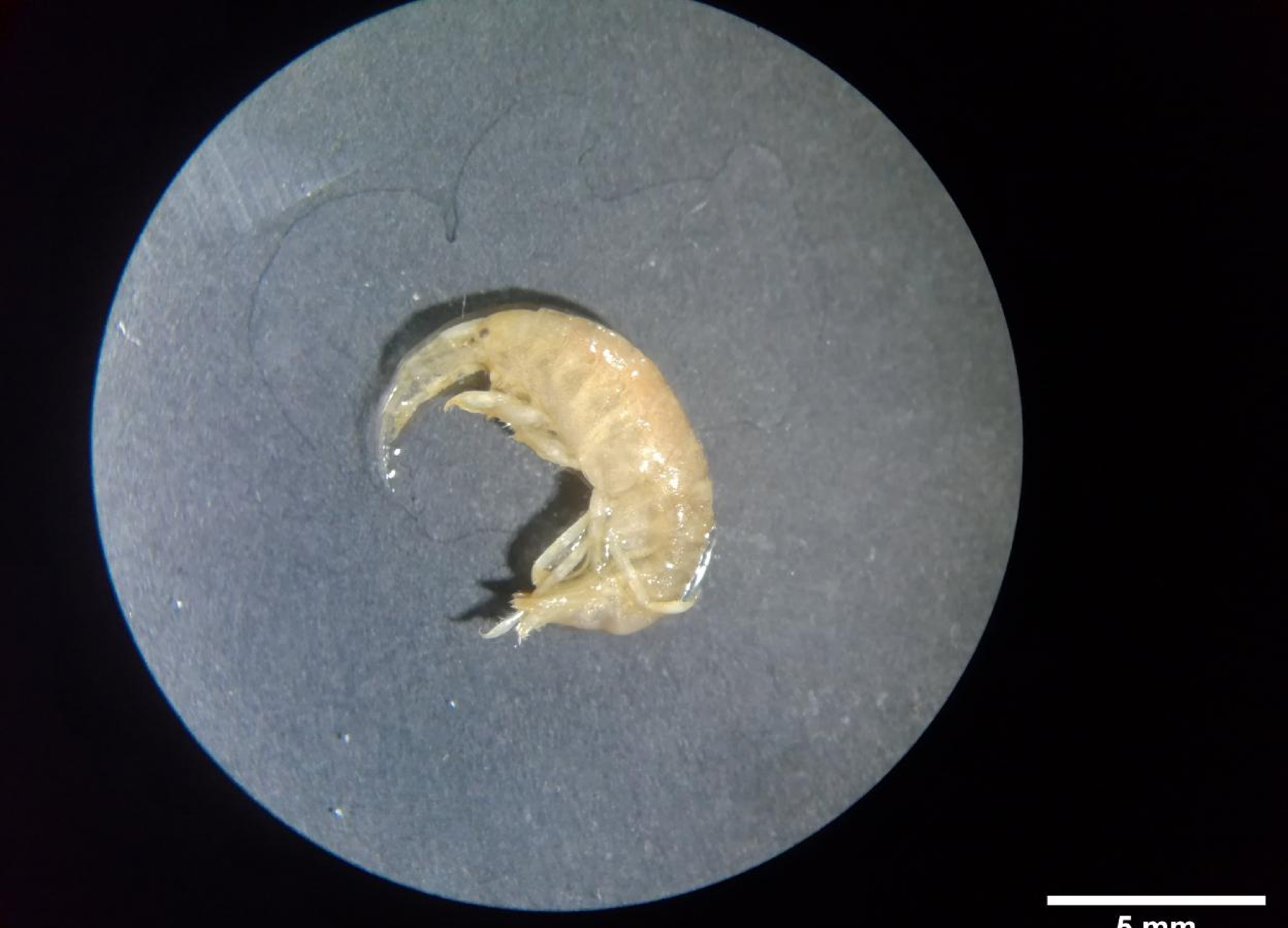
5 mm

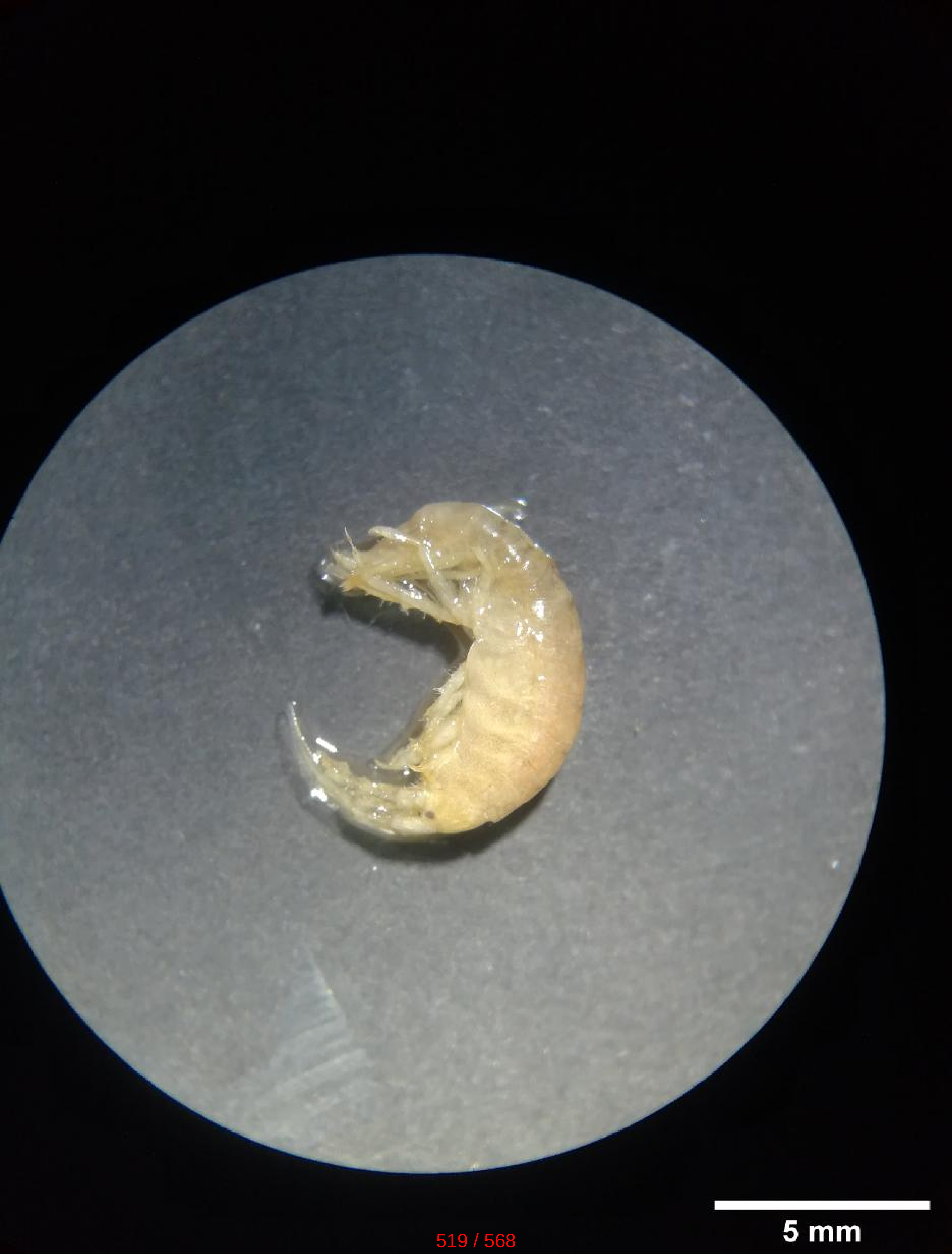
5 mm

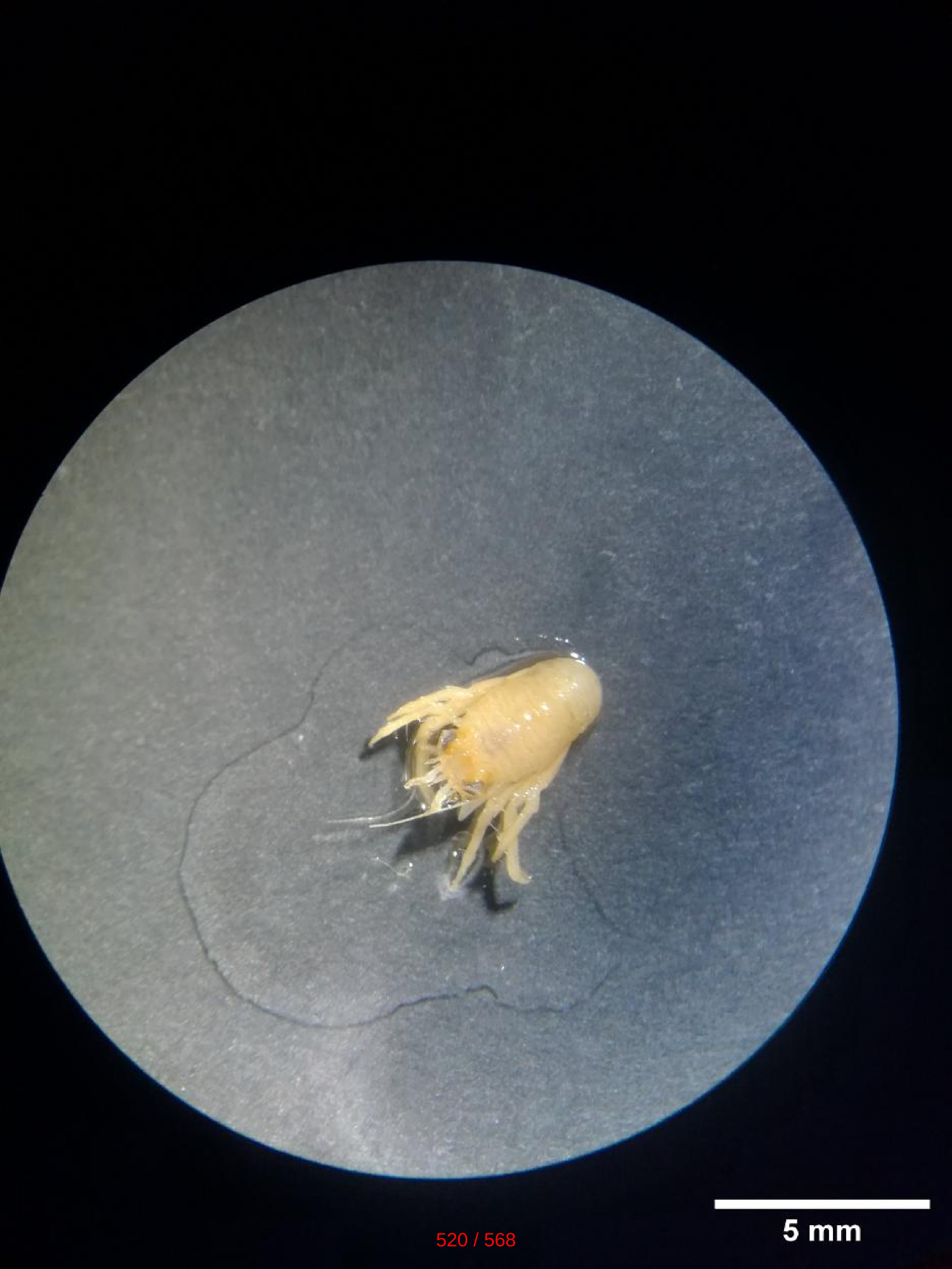
5 mm

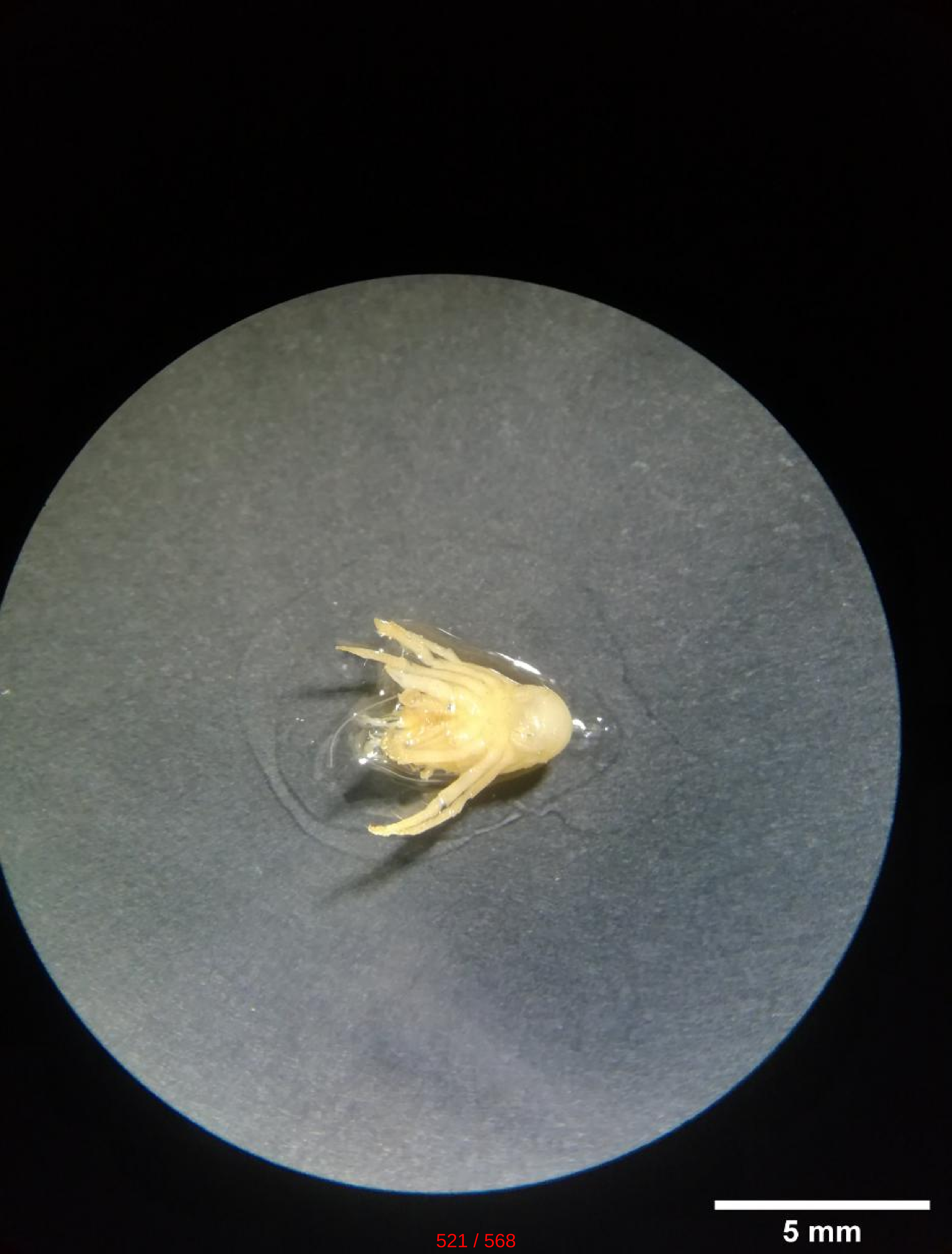
5 mm

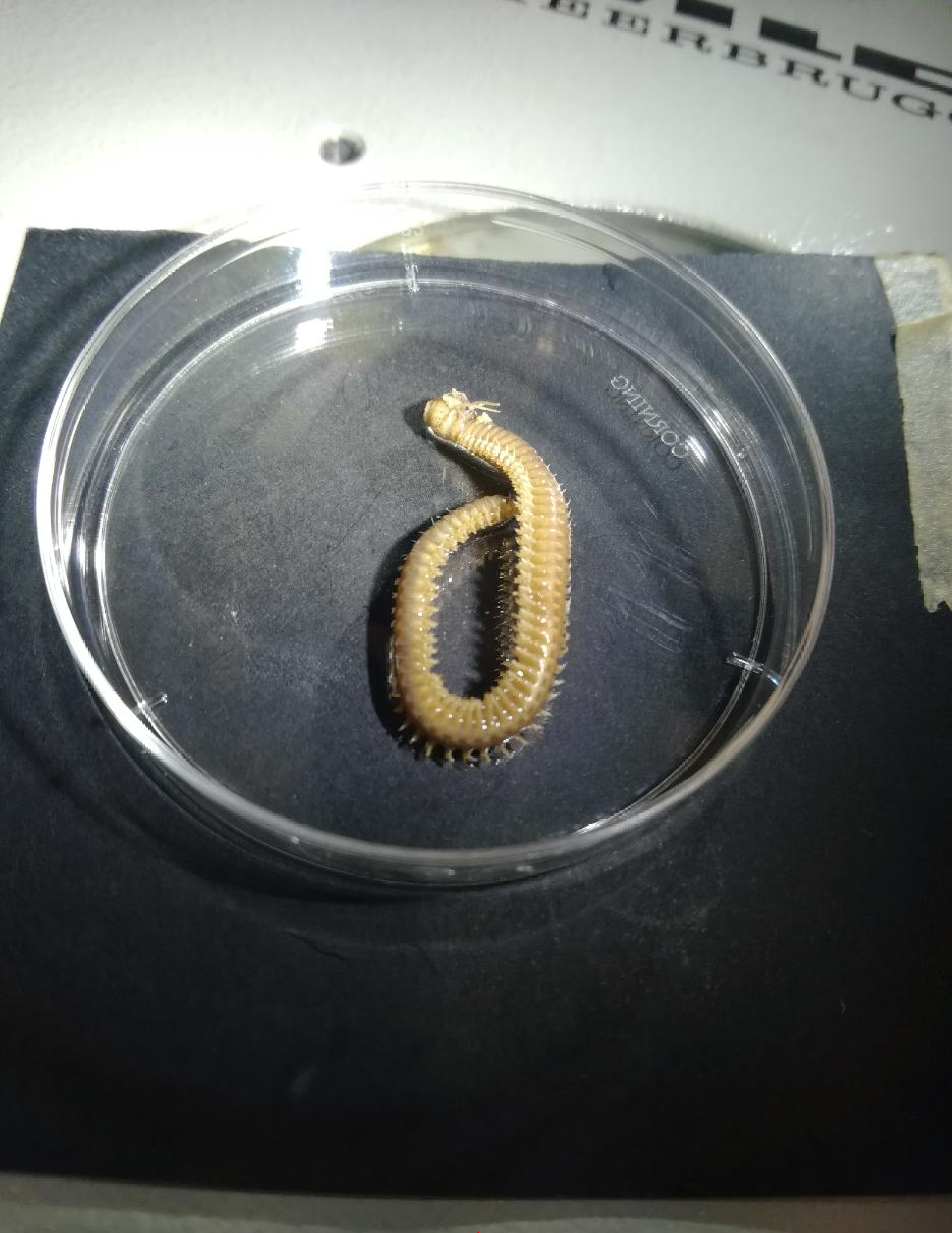
CORNING

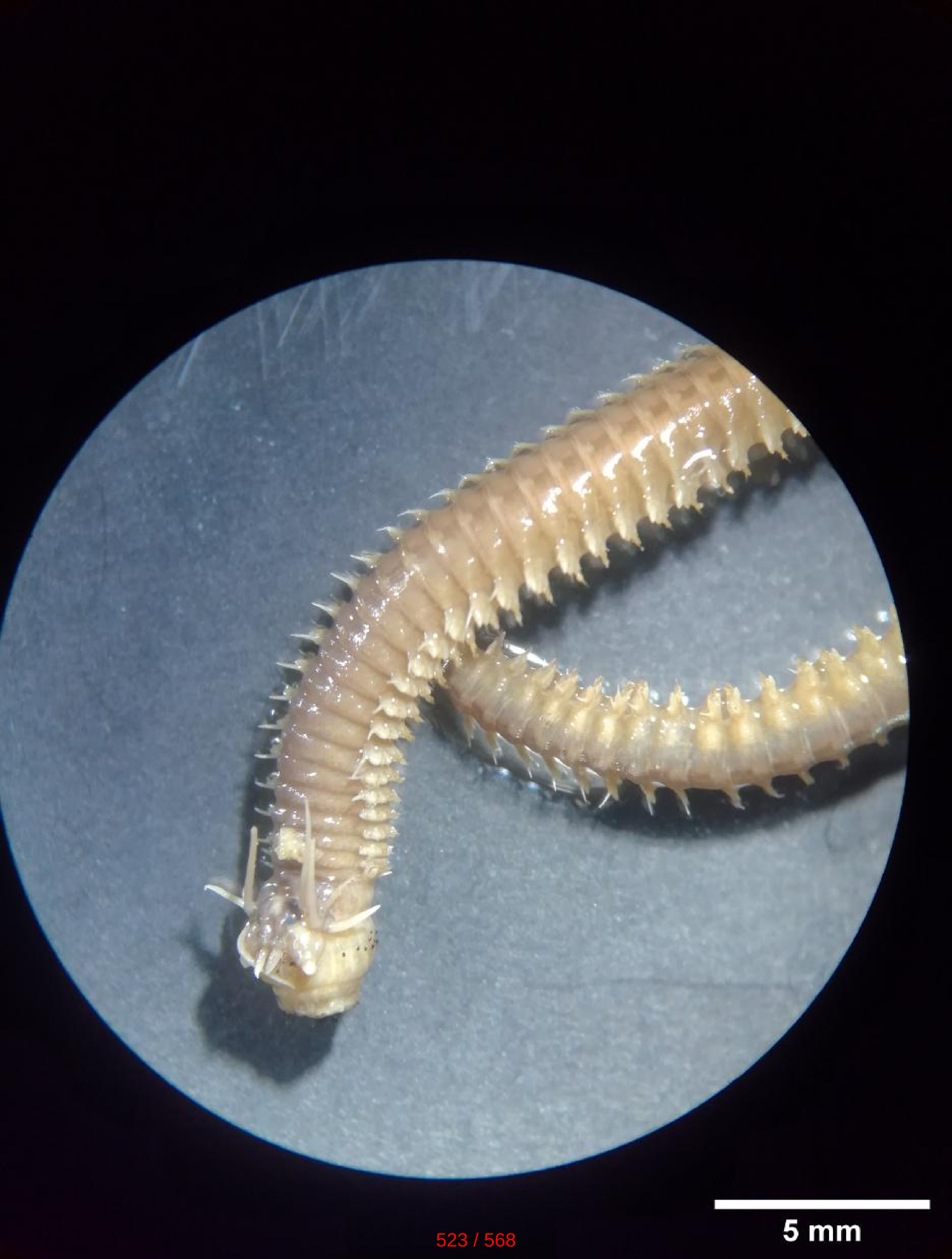
5 mm

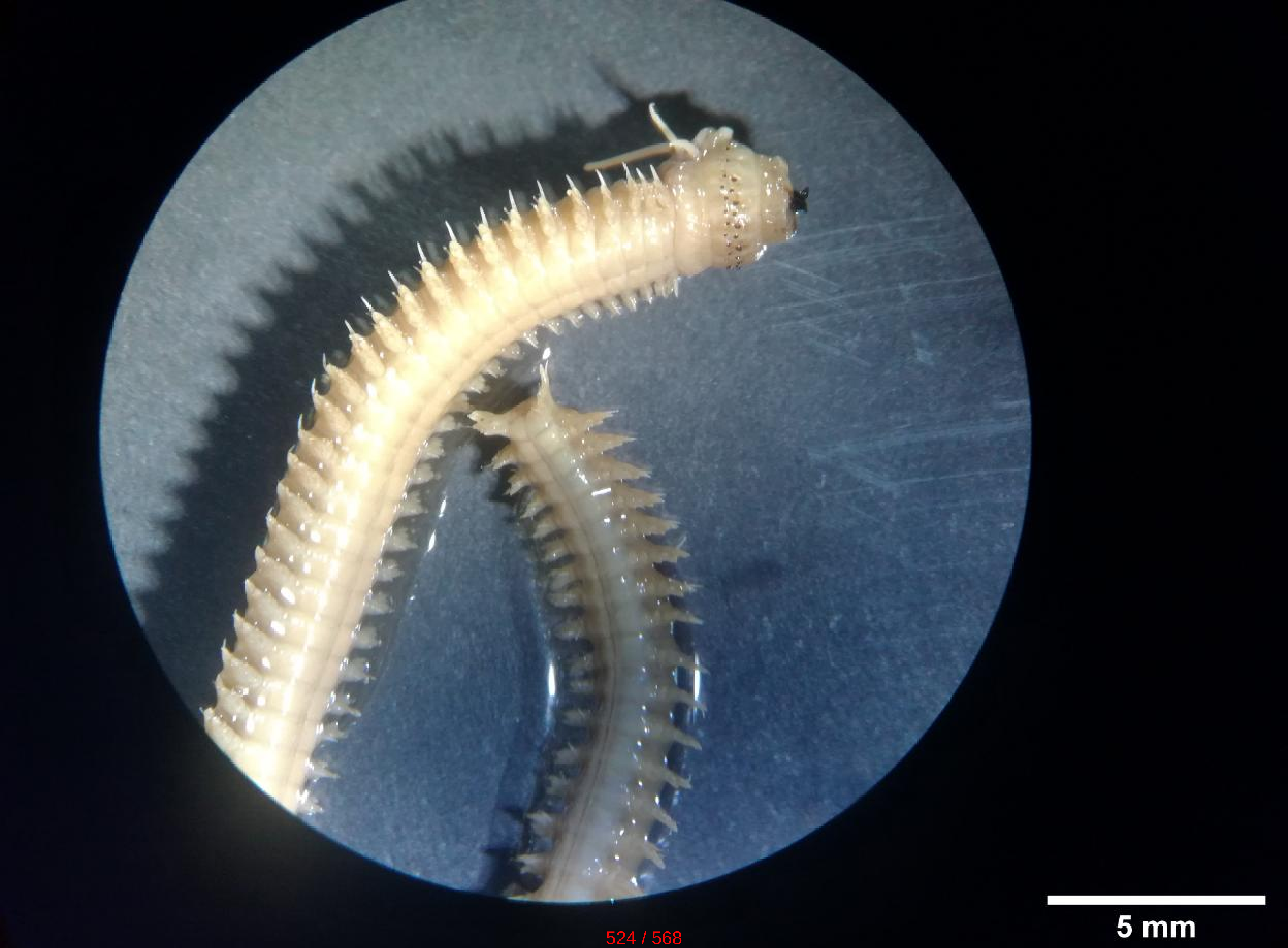
5 mm

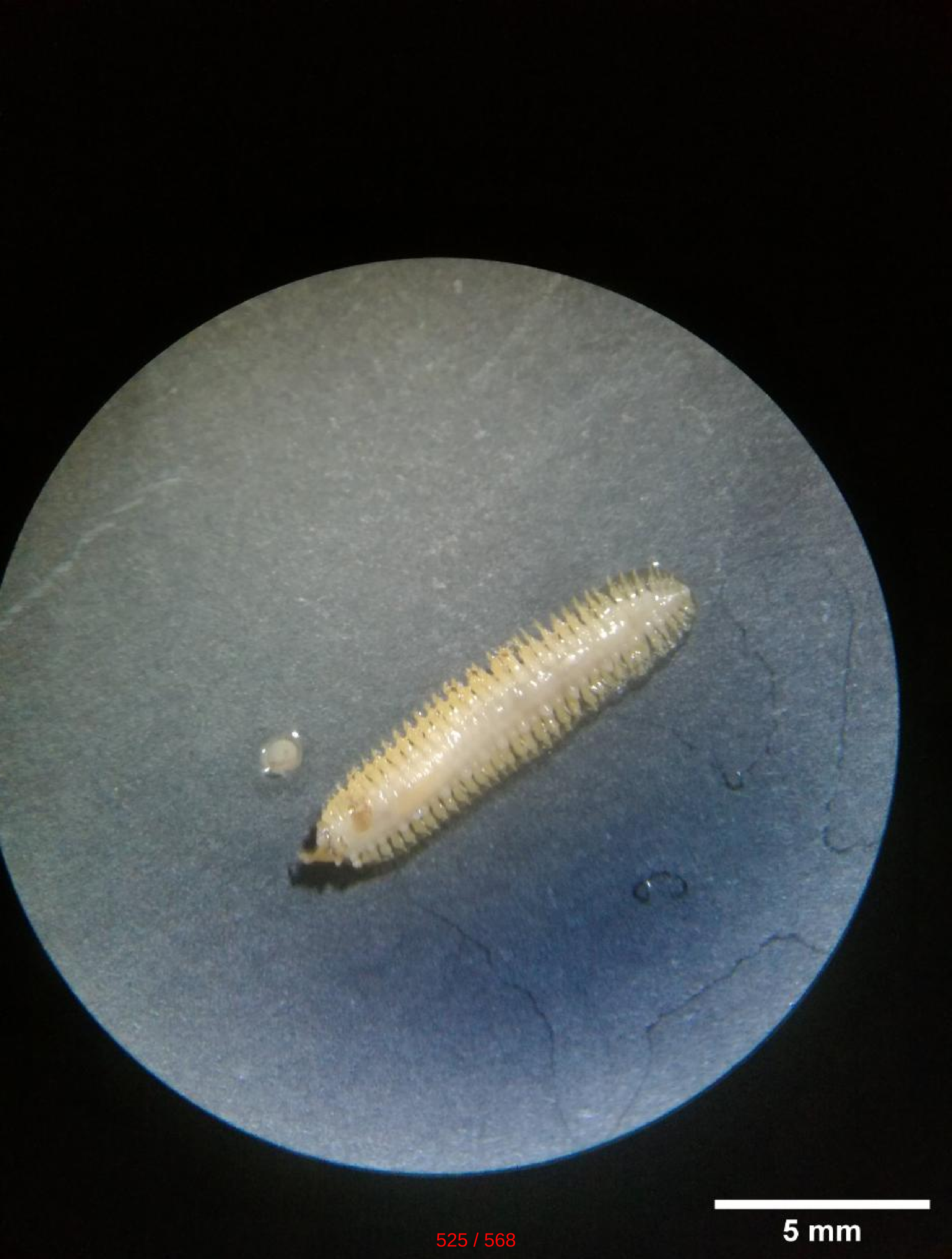
5 mm

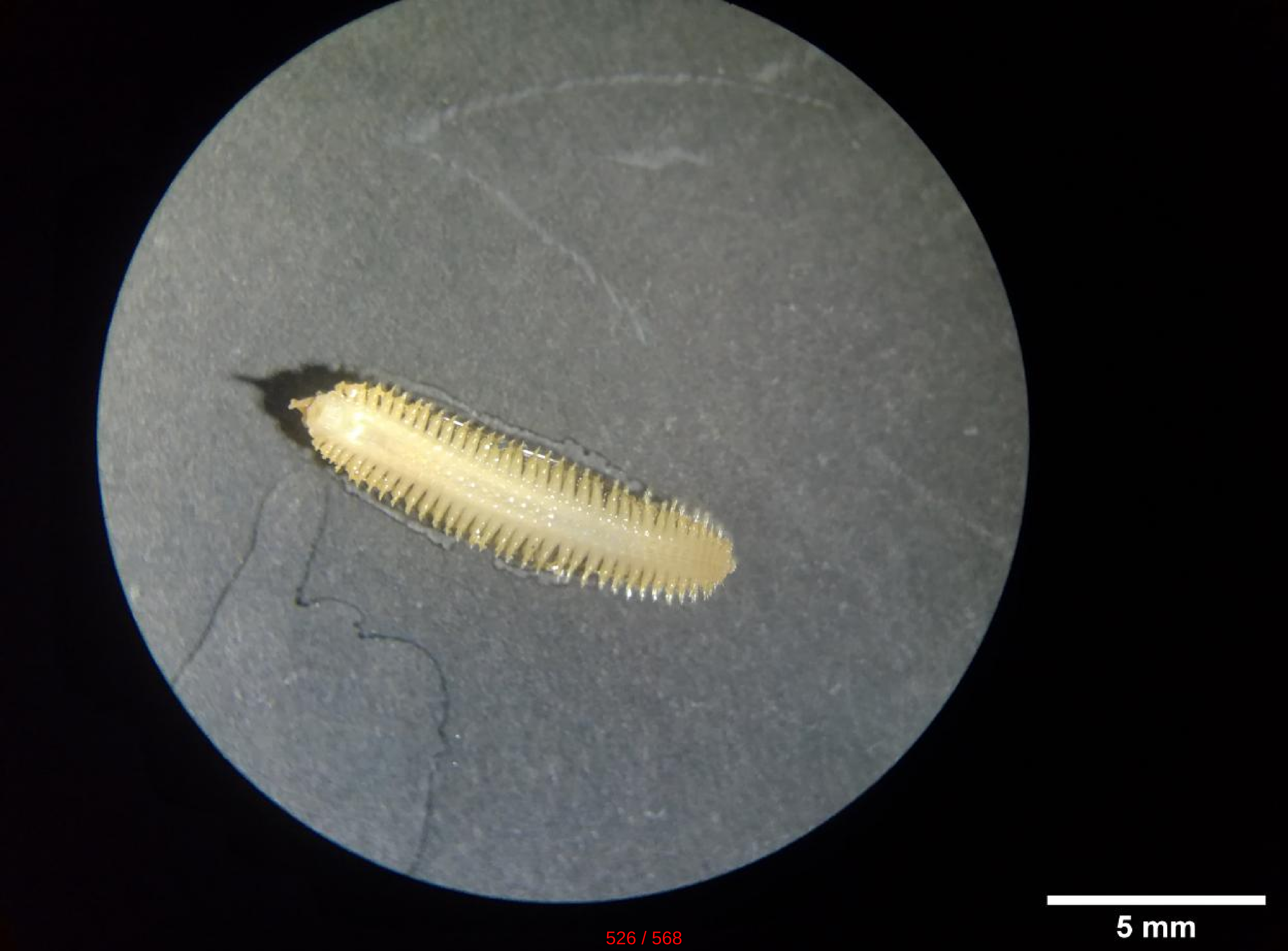
5 mm

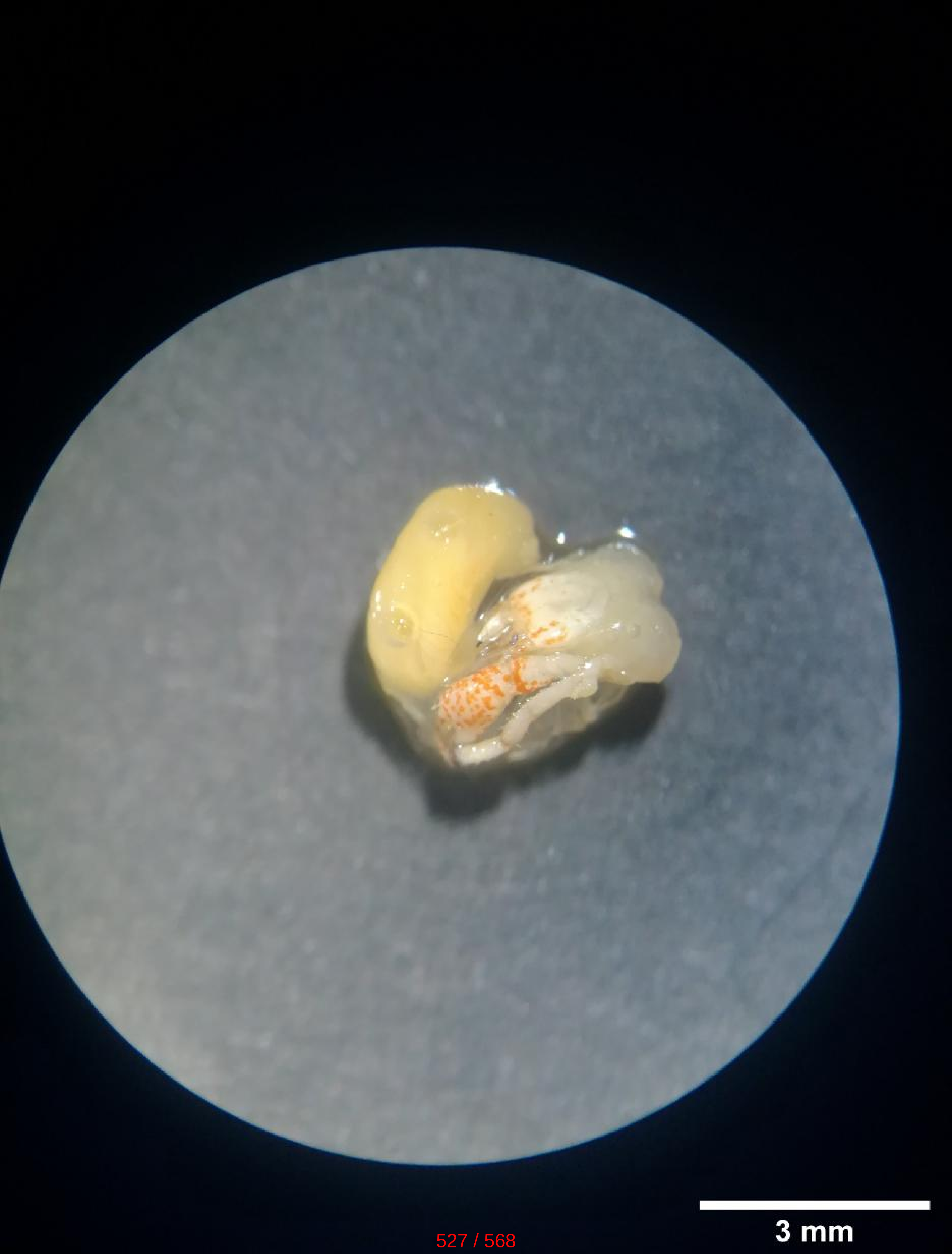
3 mm

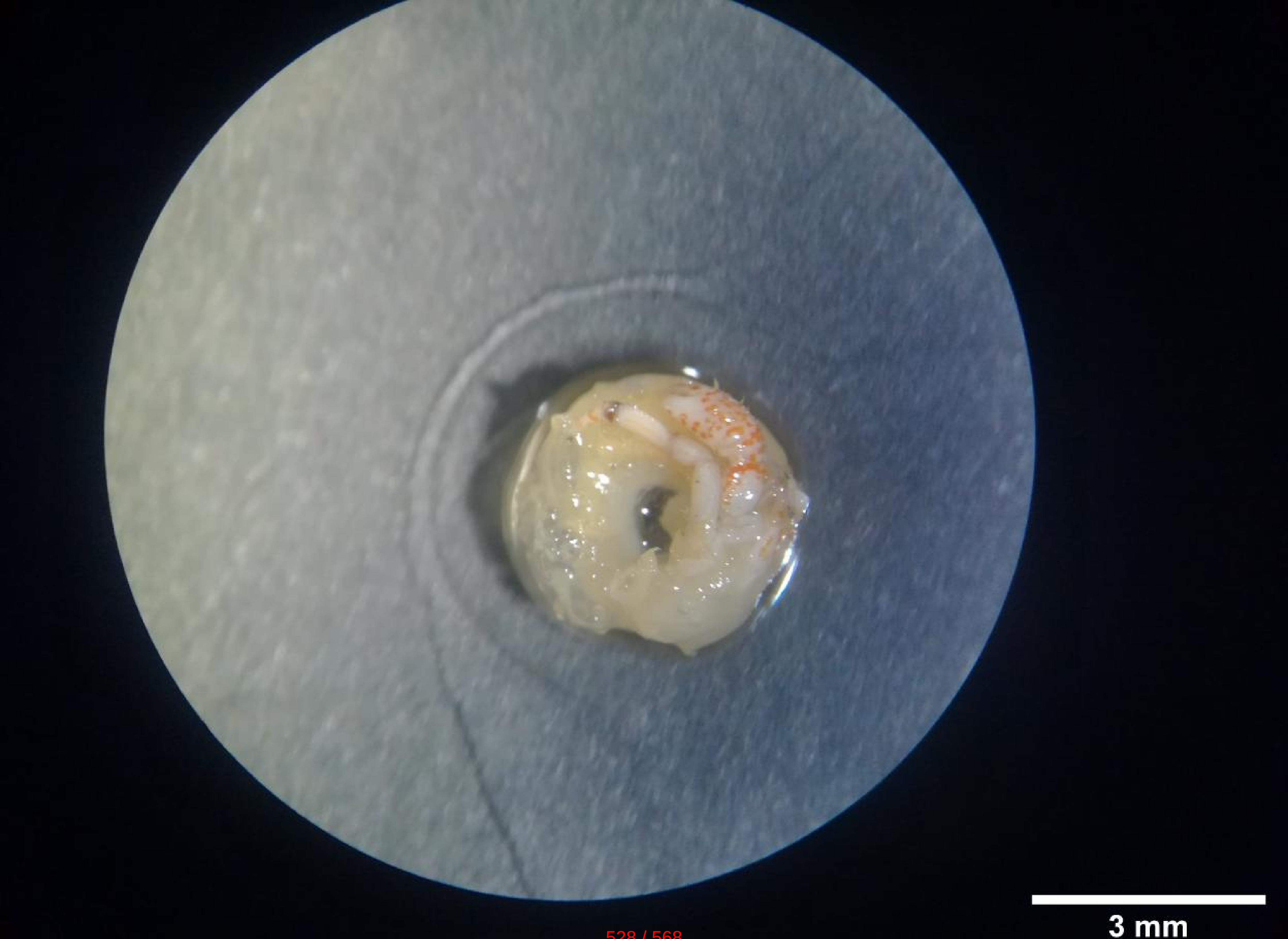
3 mm

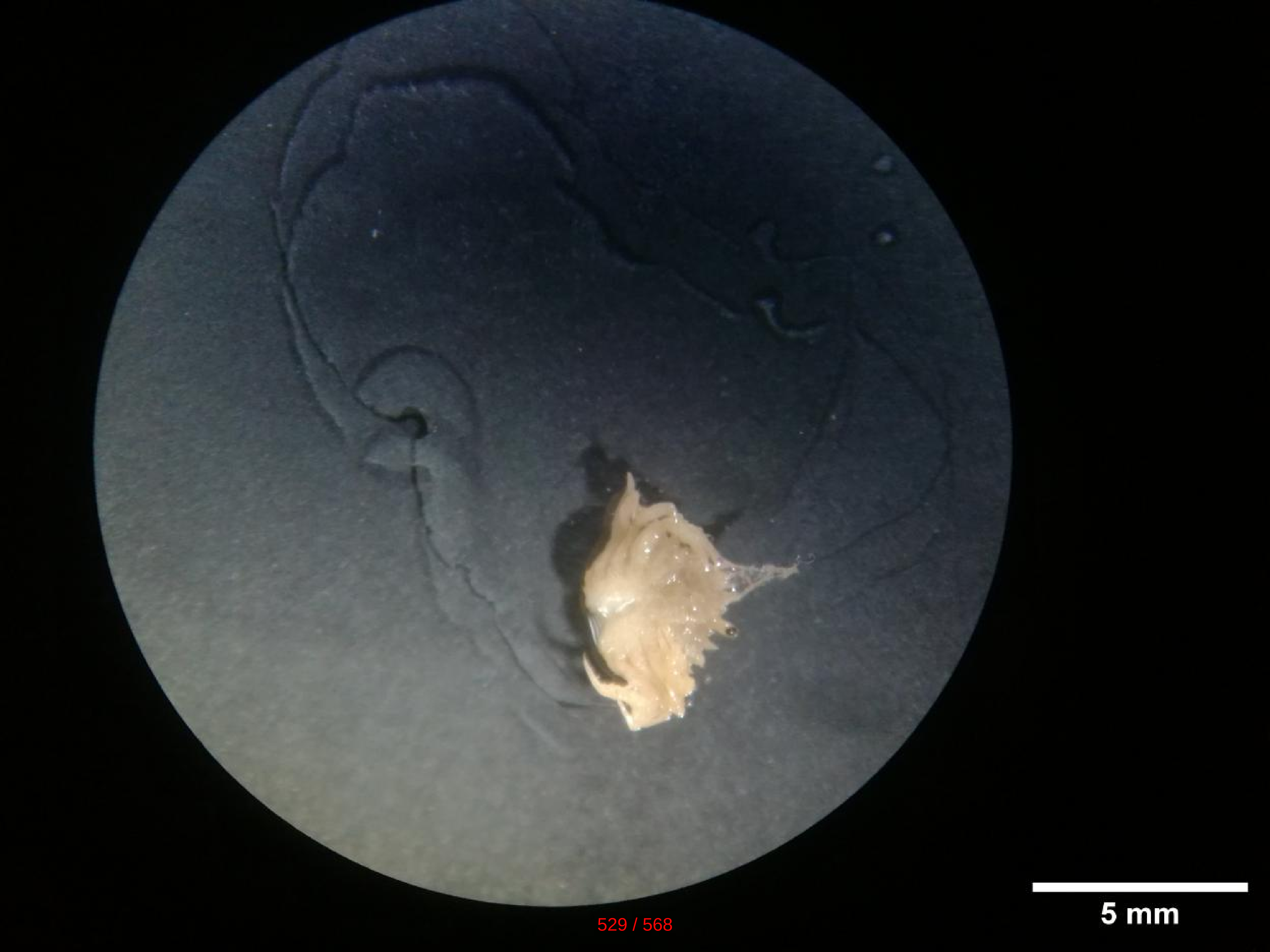
5 mm

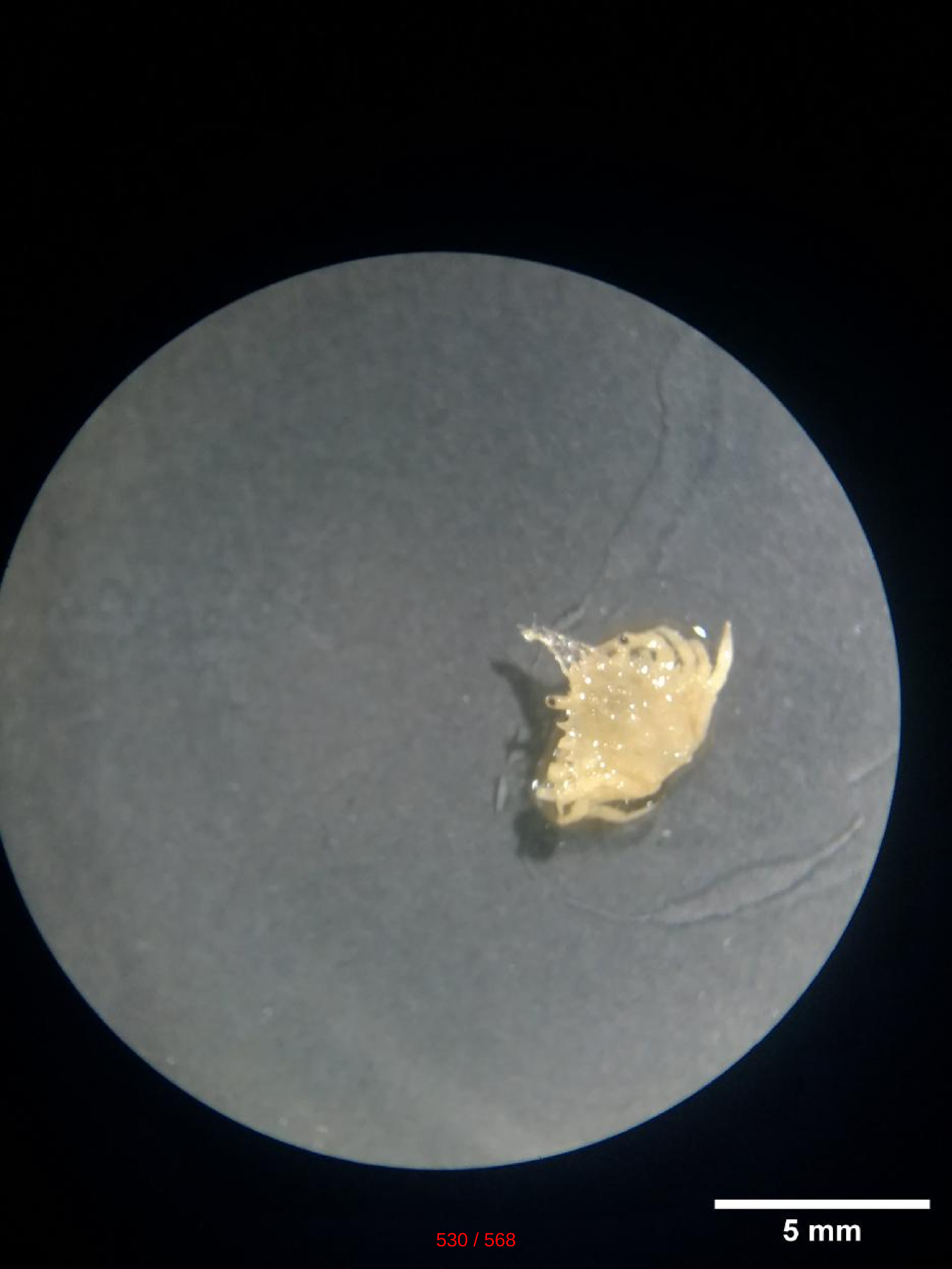
5 mm

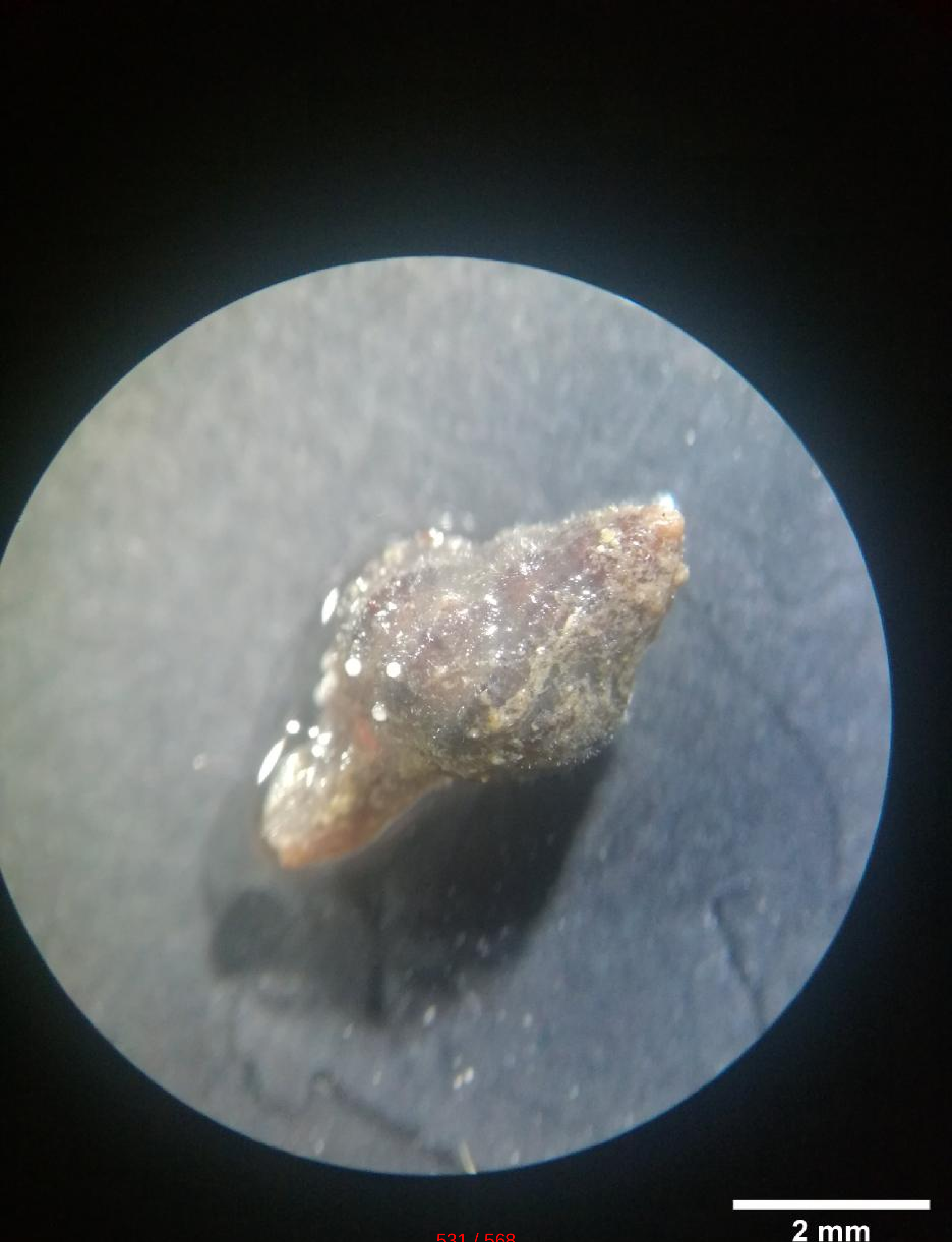
2 mm

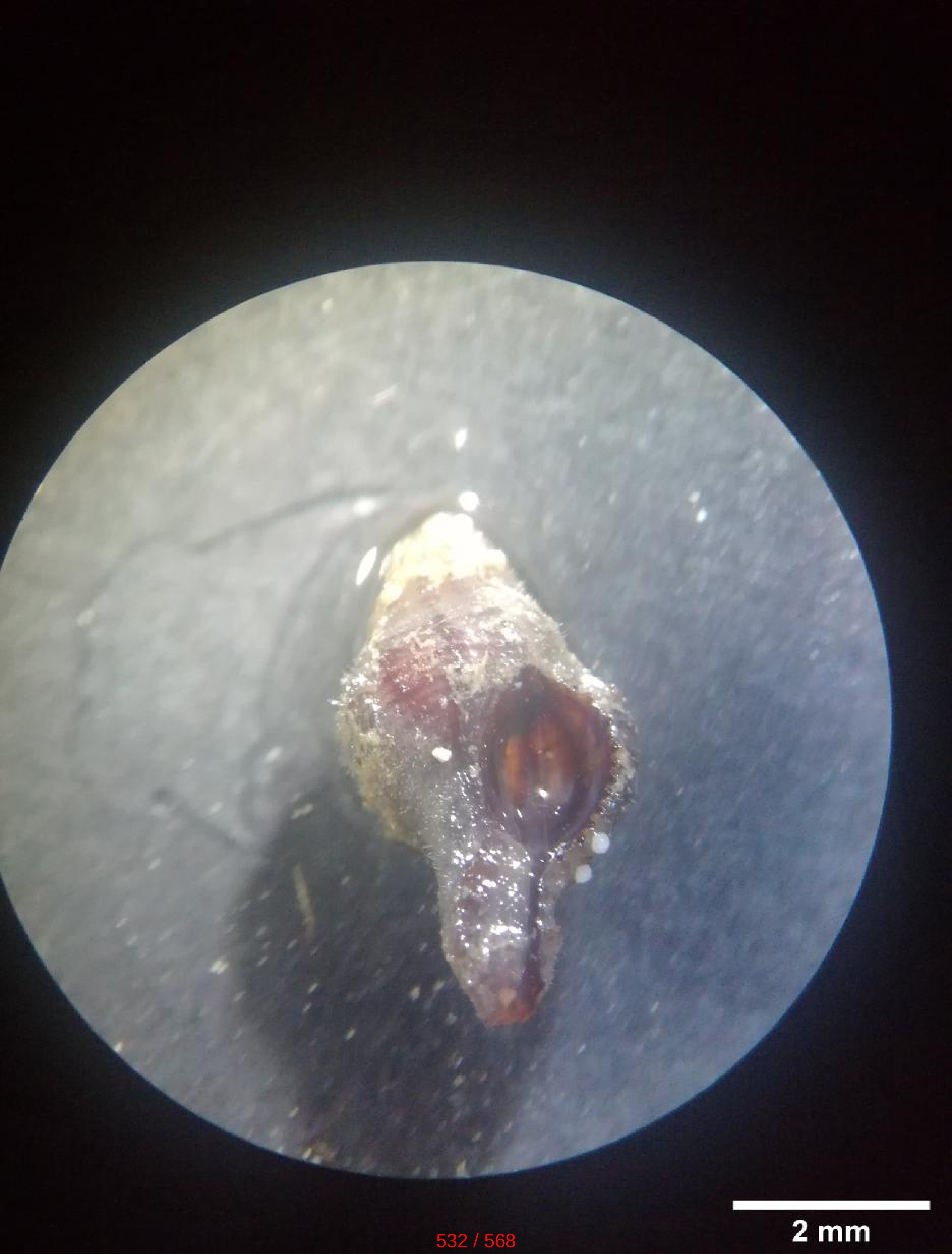
2 mm

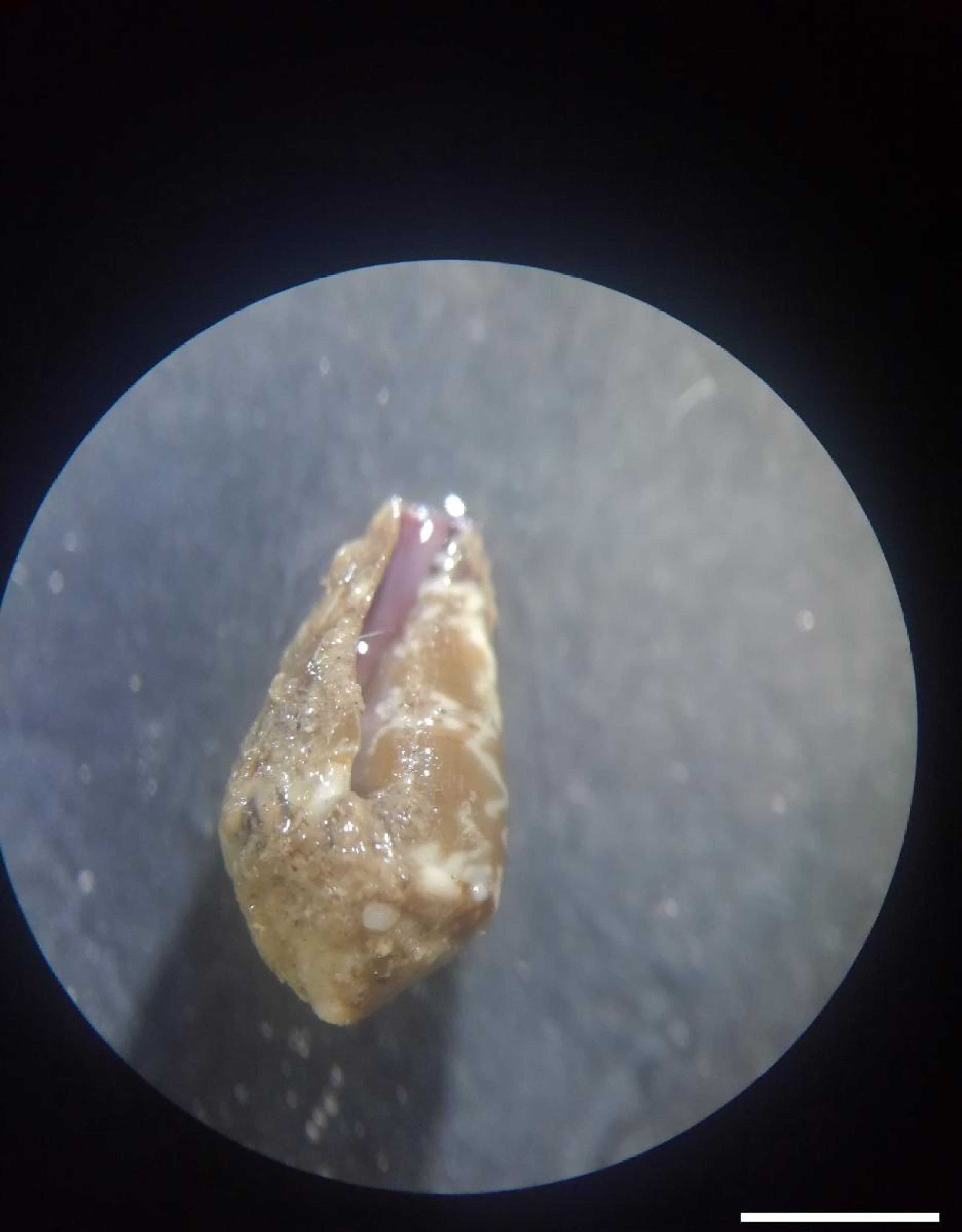
2 mm

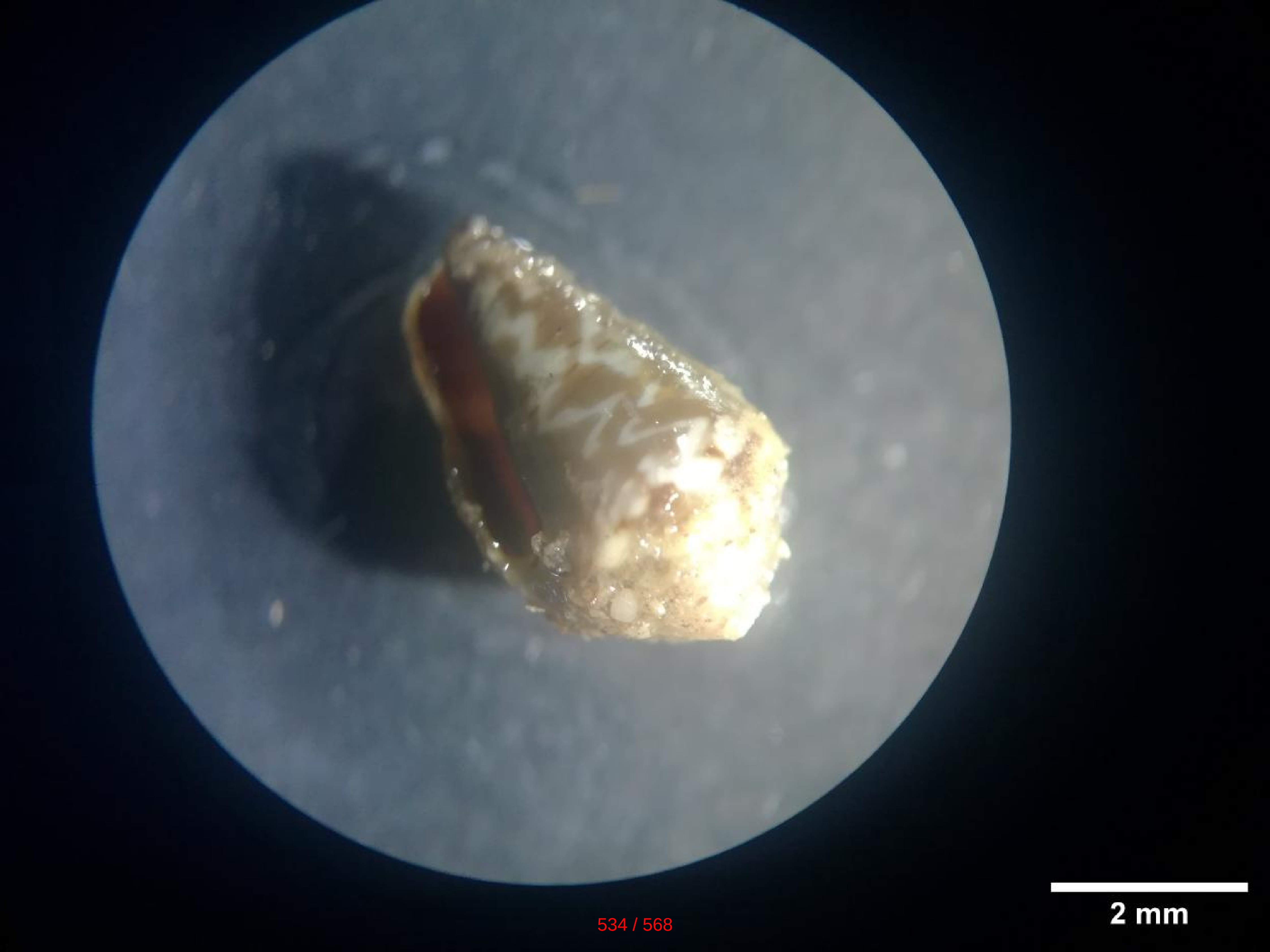
2 mm

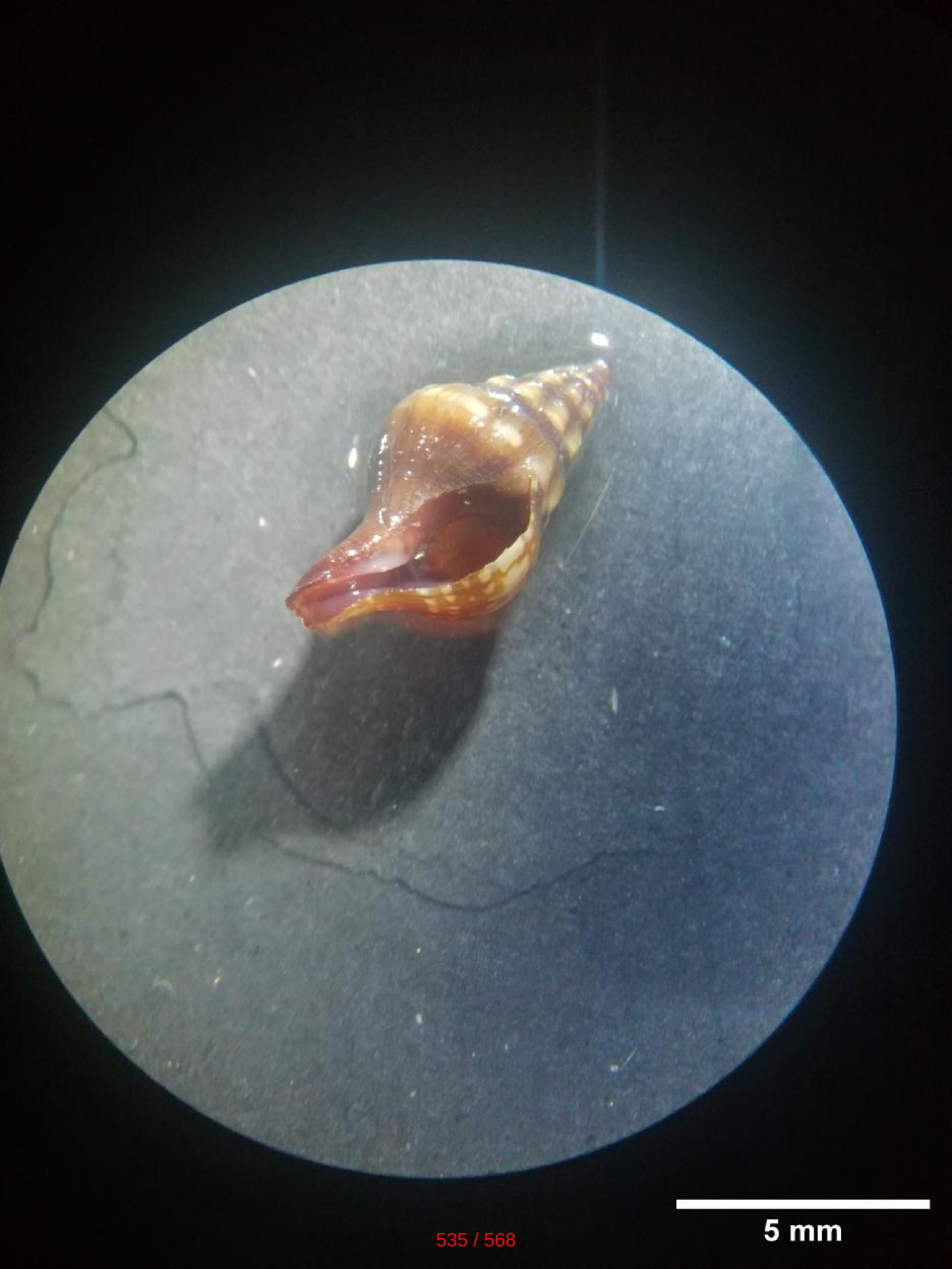
5 mm

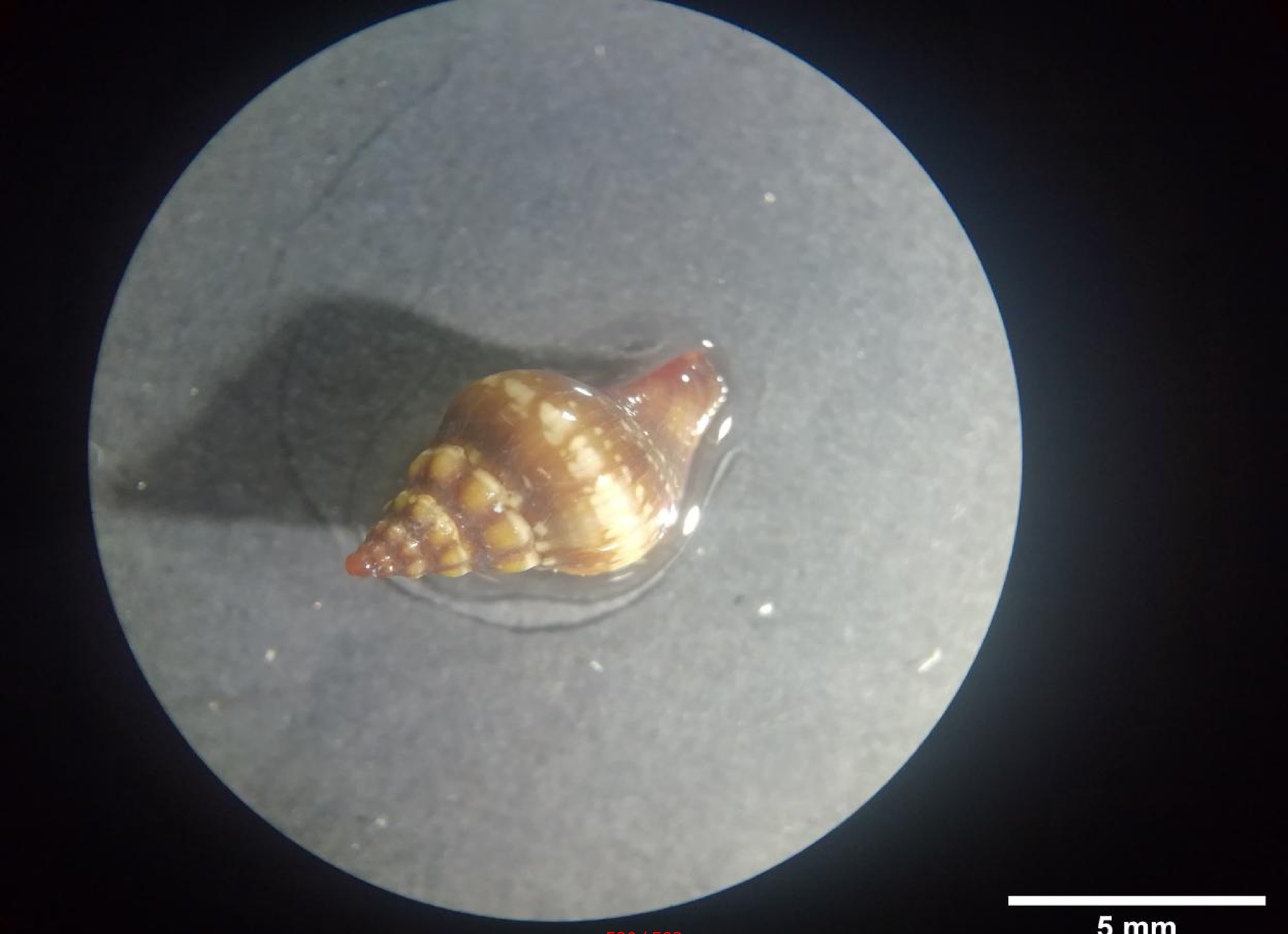
5 mm

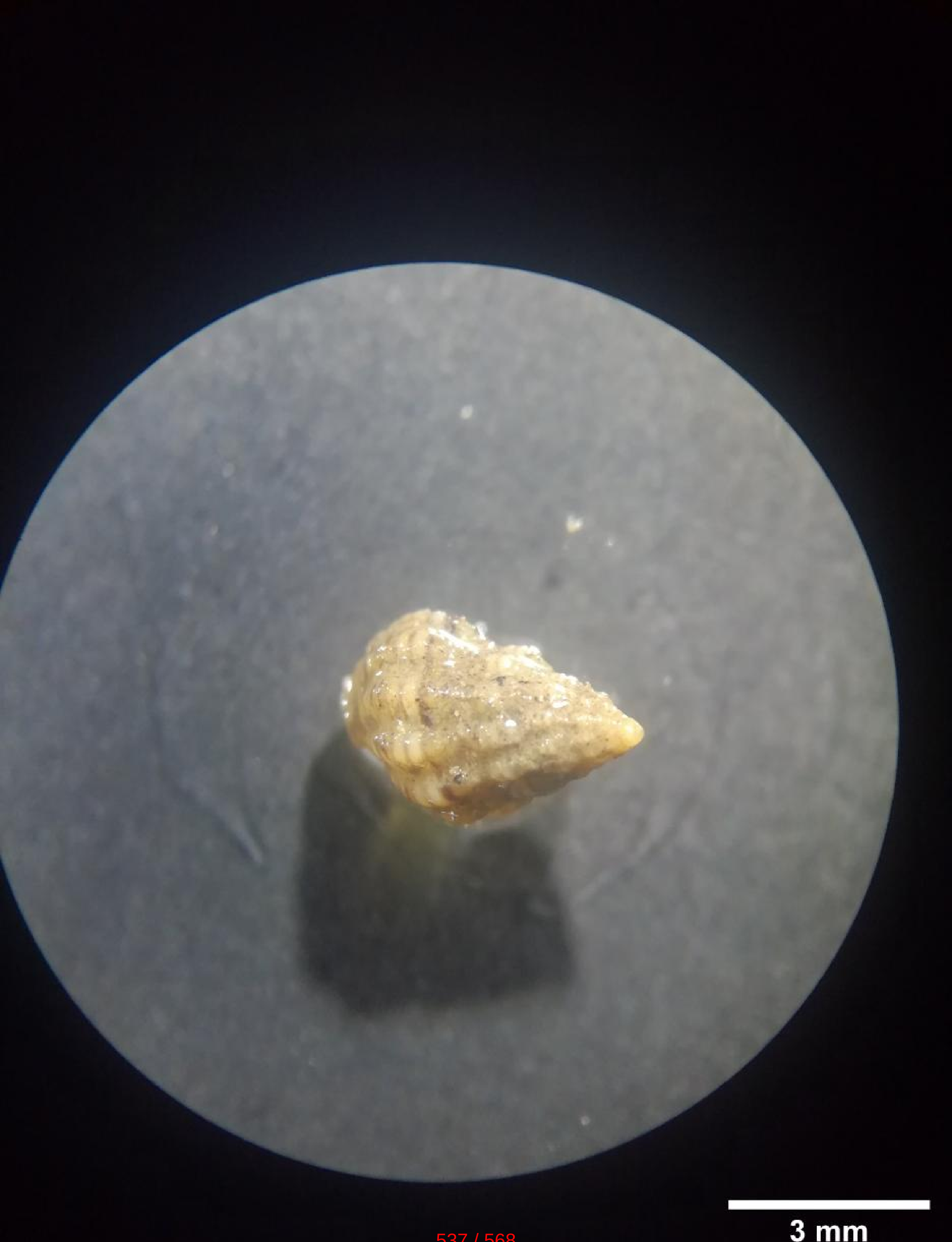
3 mm

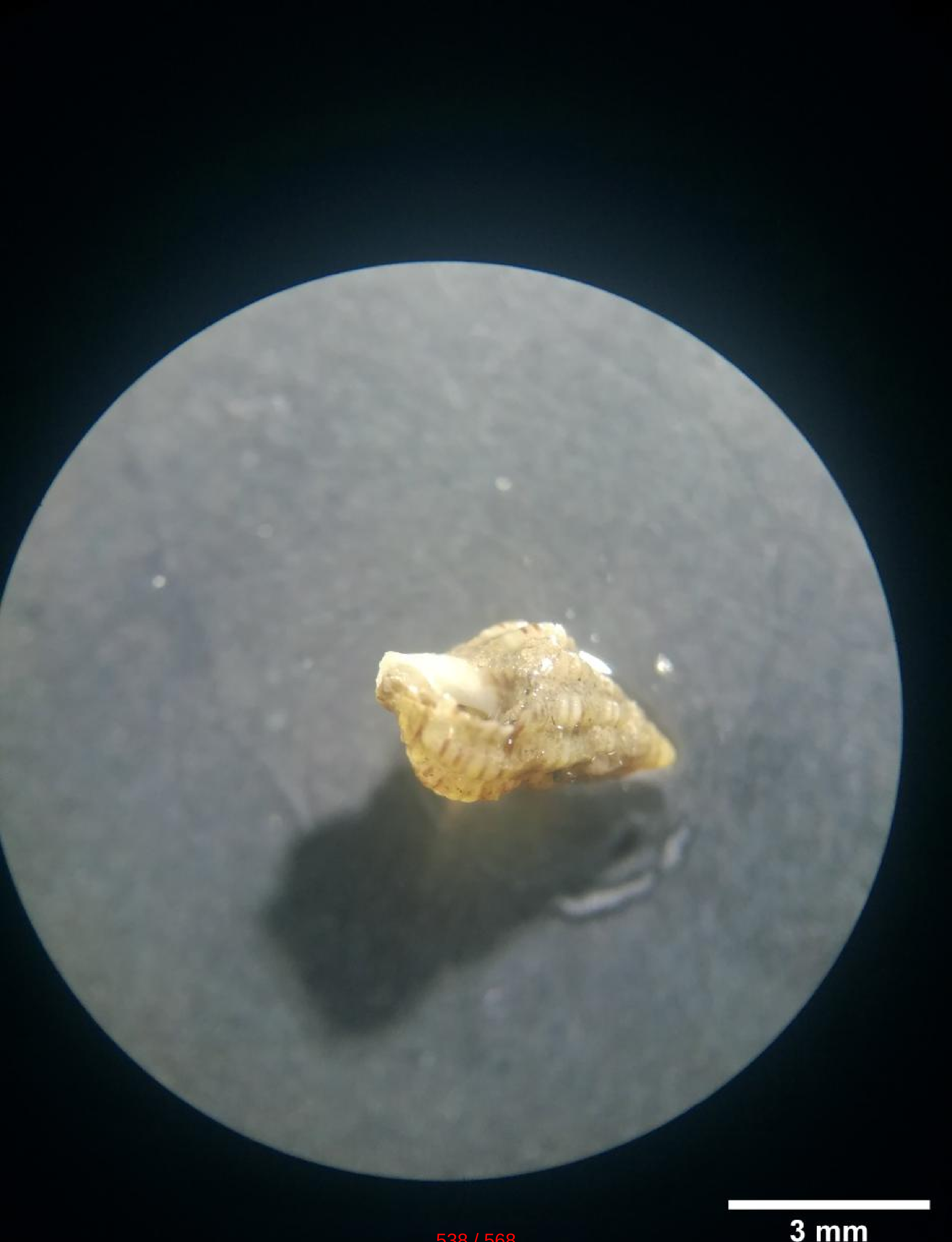
3 mm

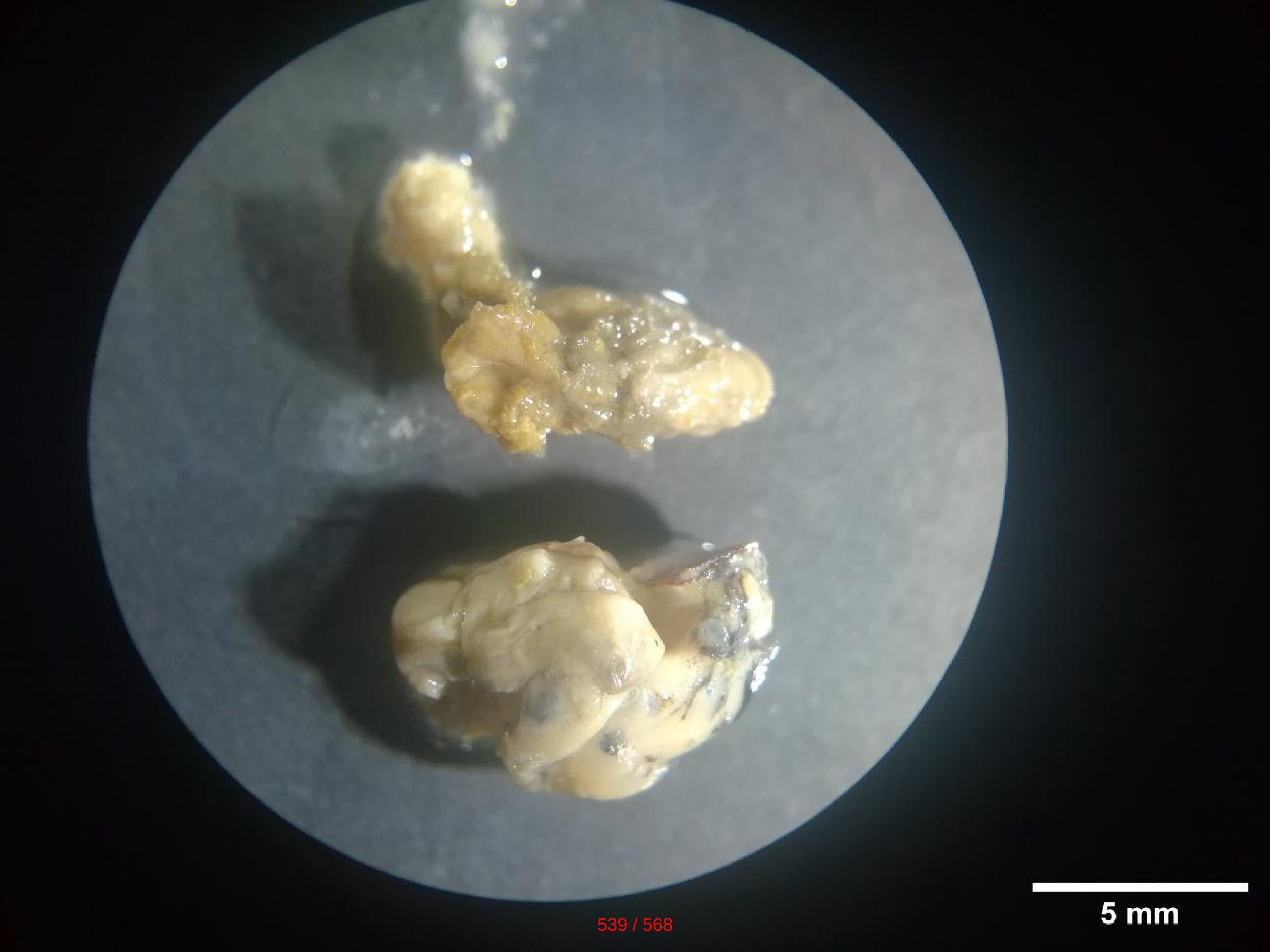
5 mm

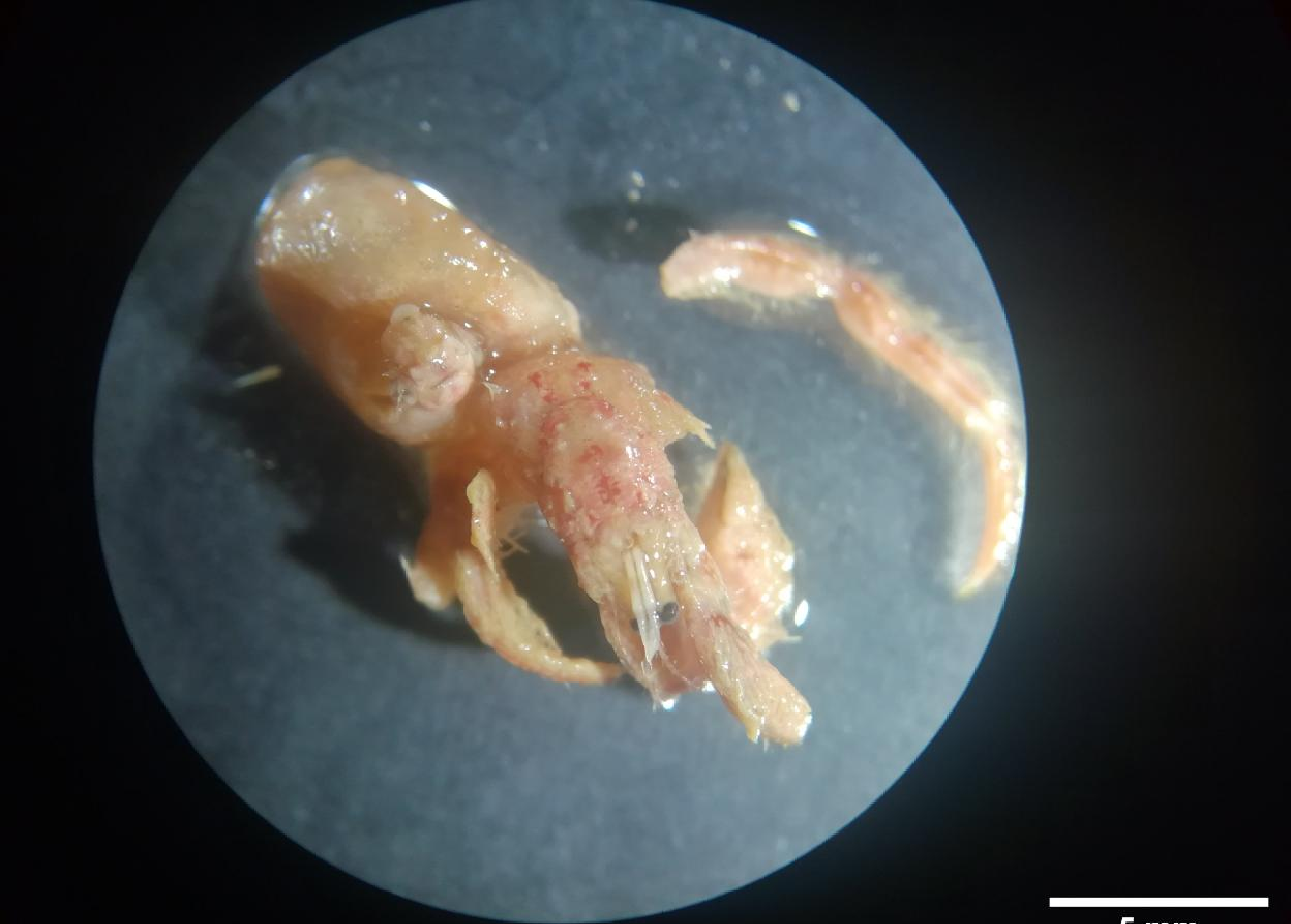
5 mm

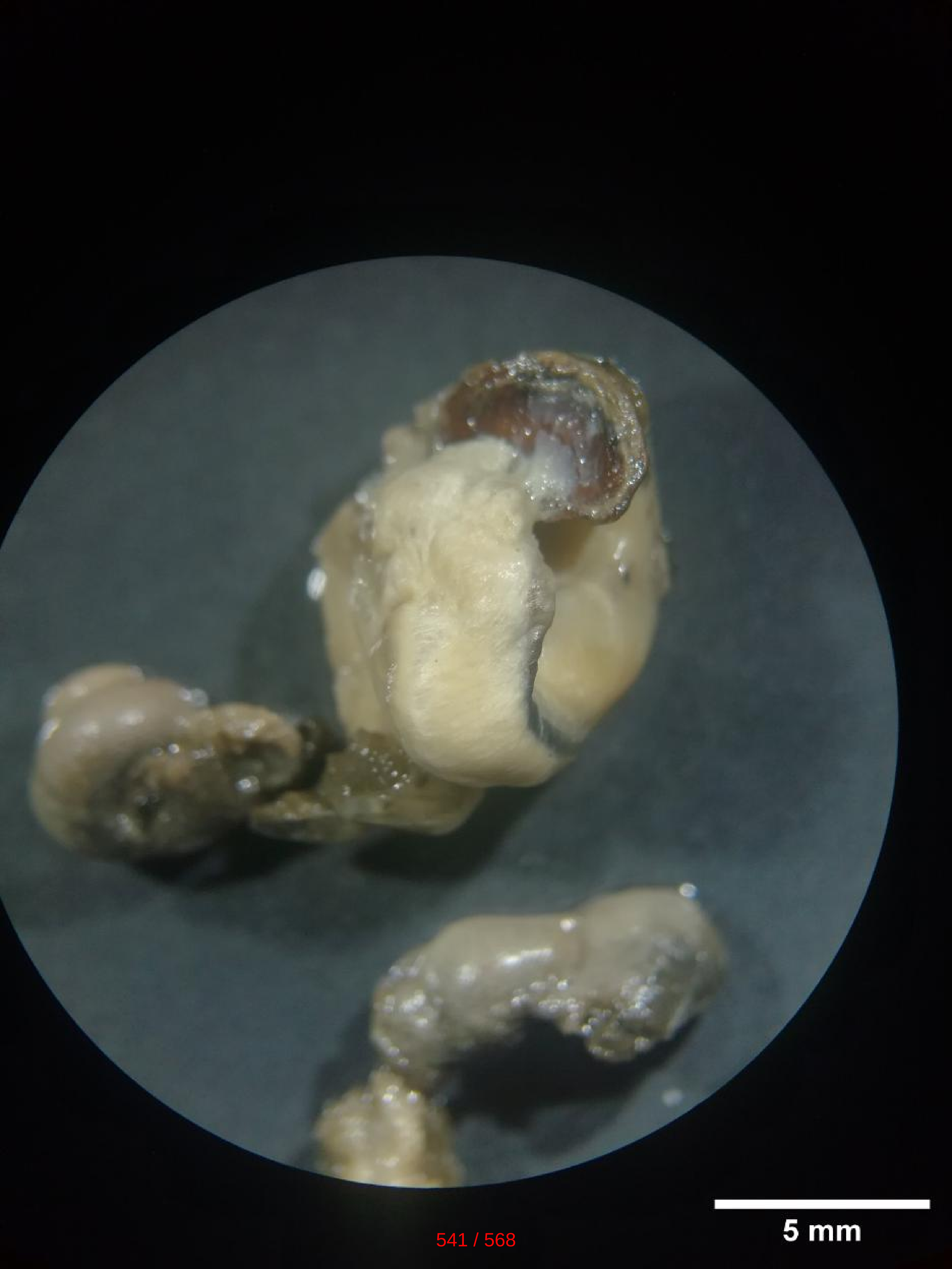
5 mm

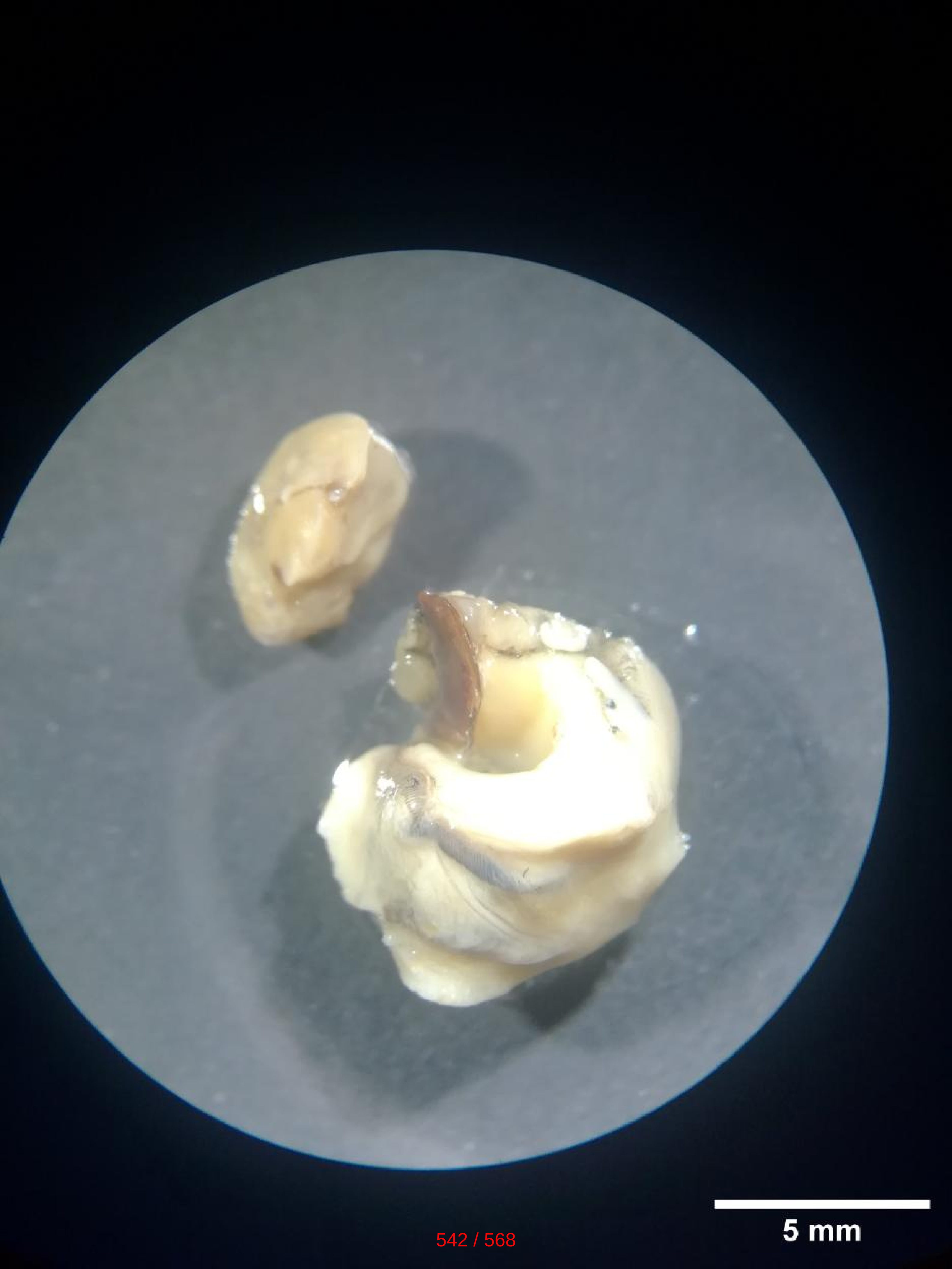
5 mm

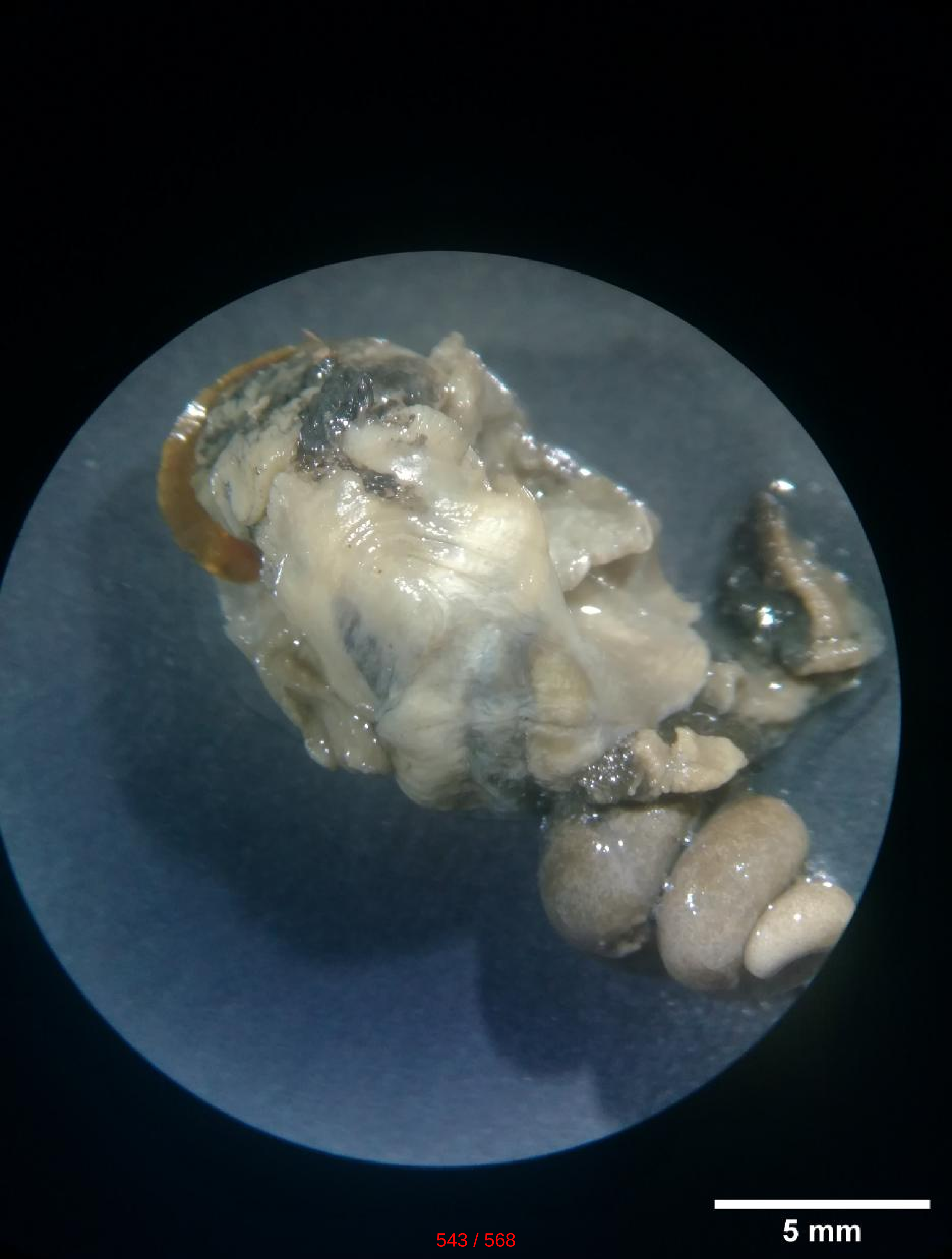
5 mm

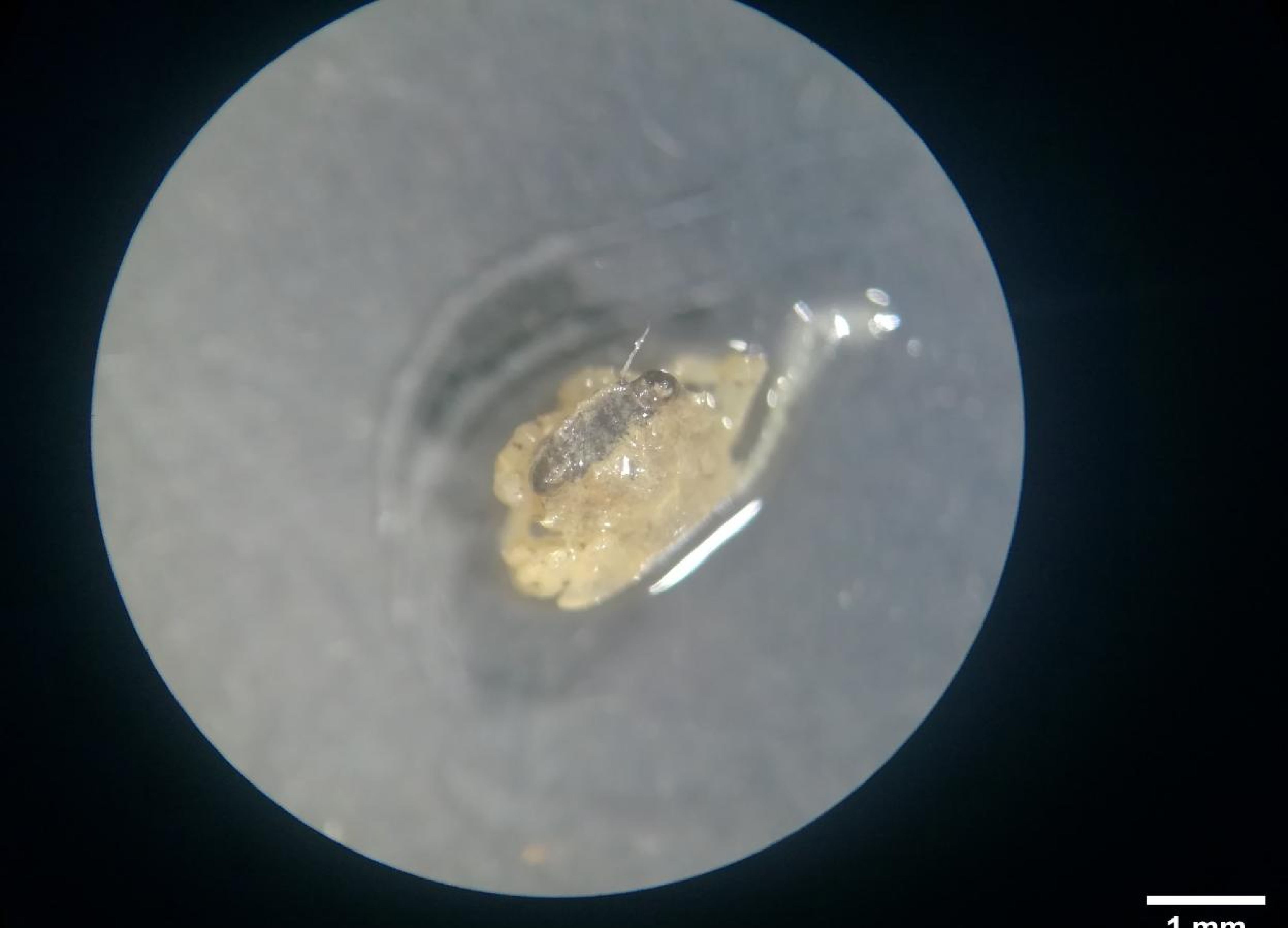
1 mm

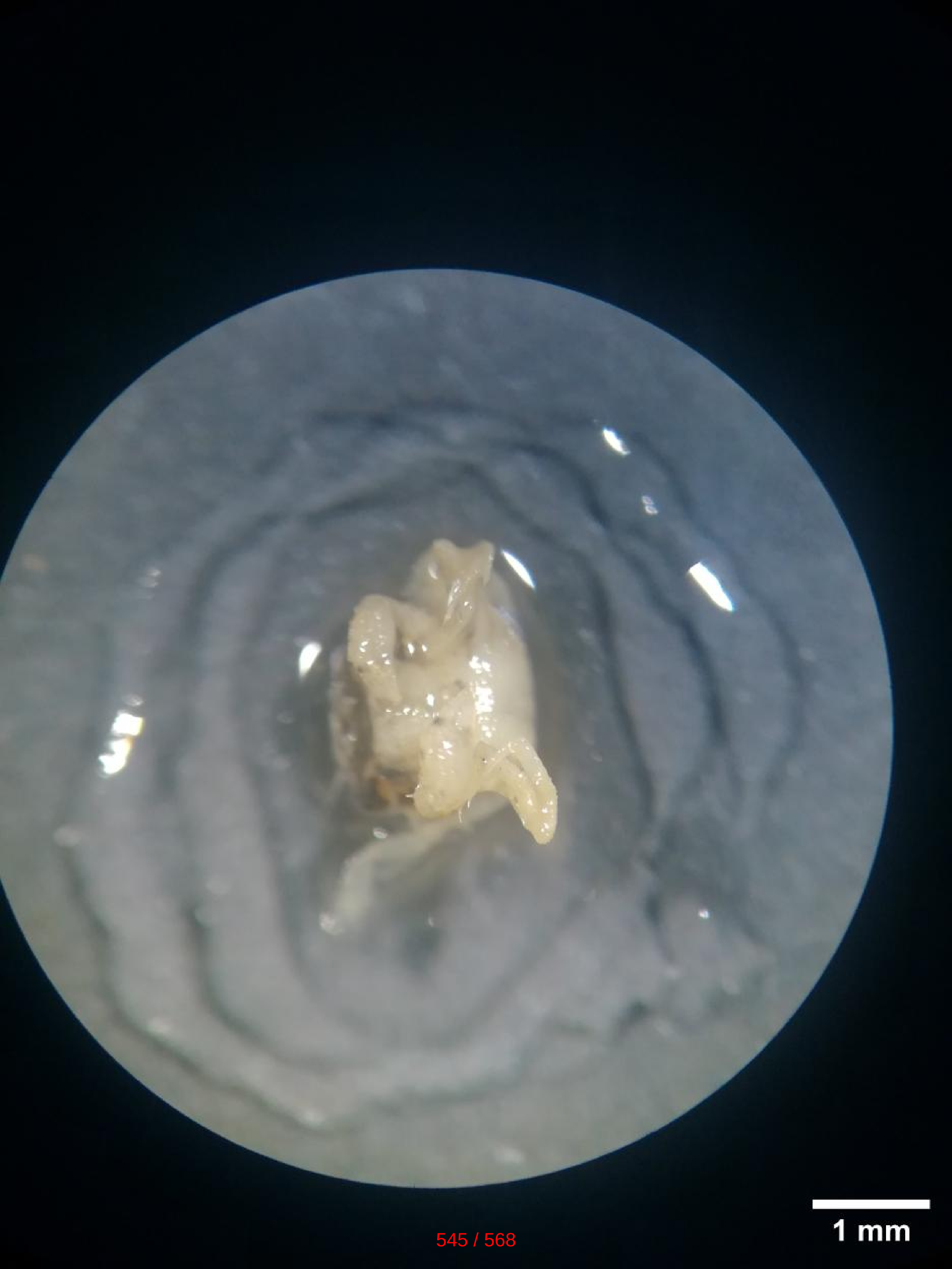
1 mm

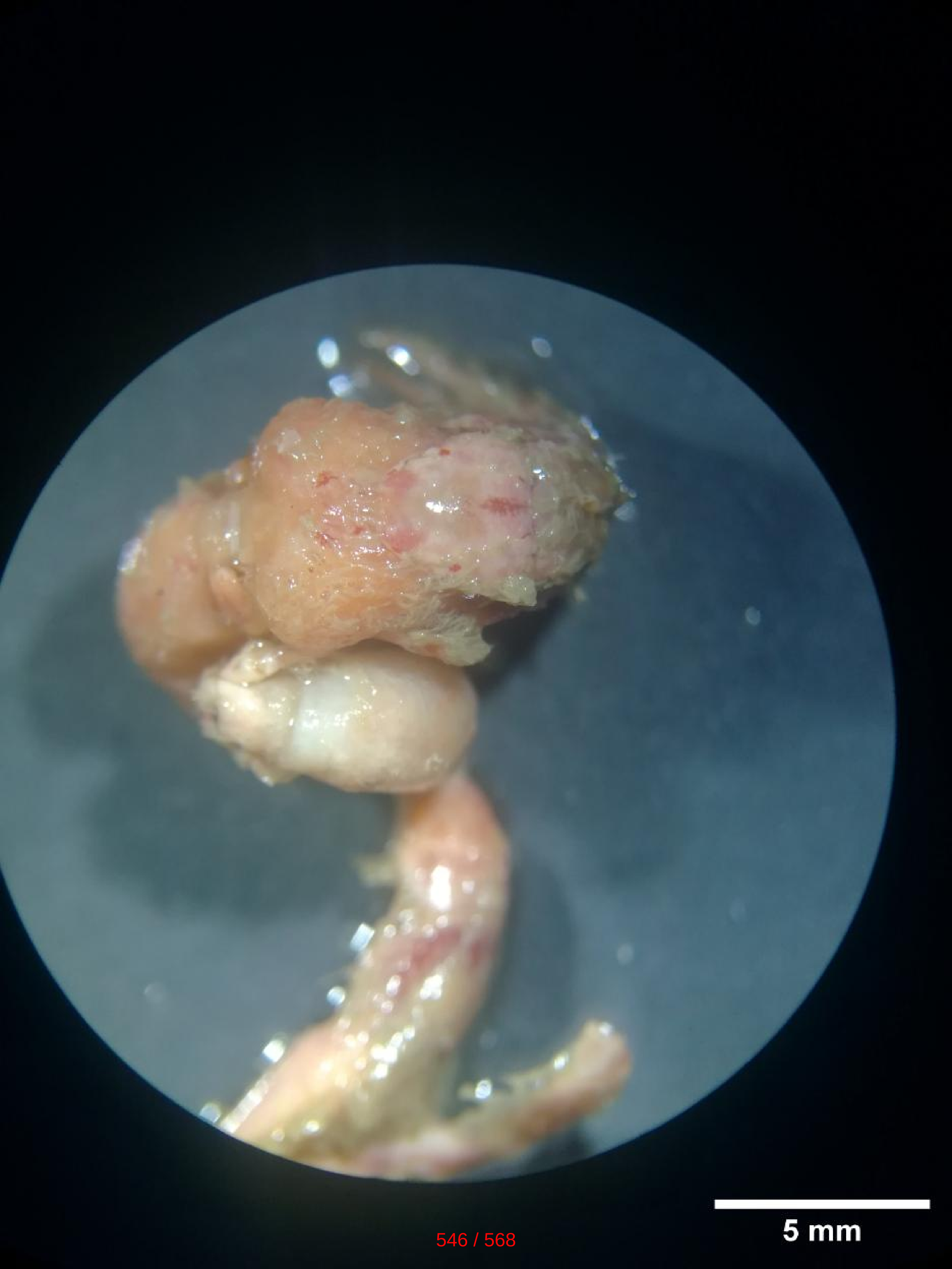
5 mm

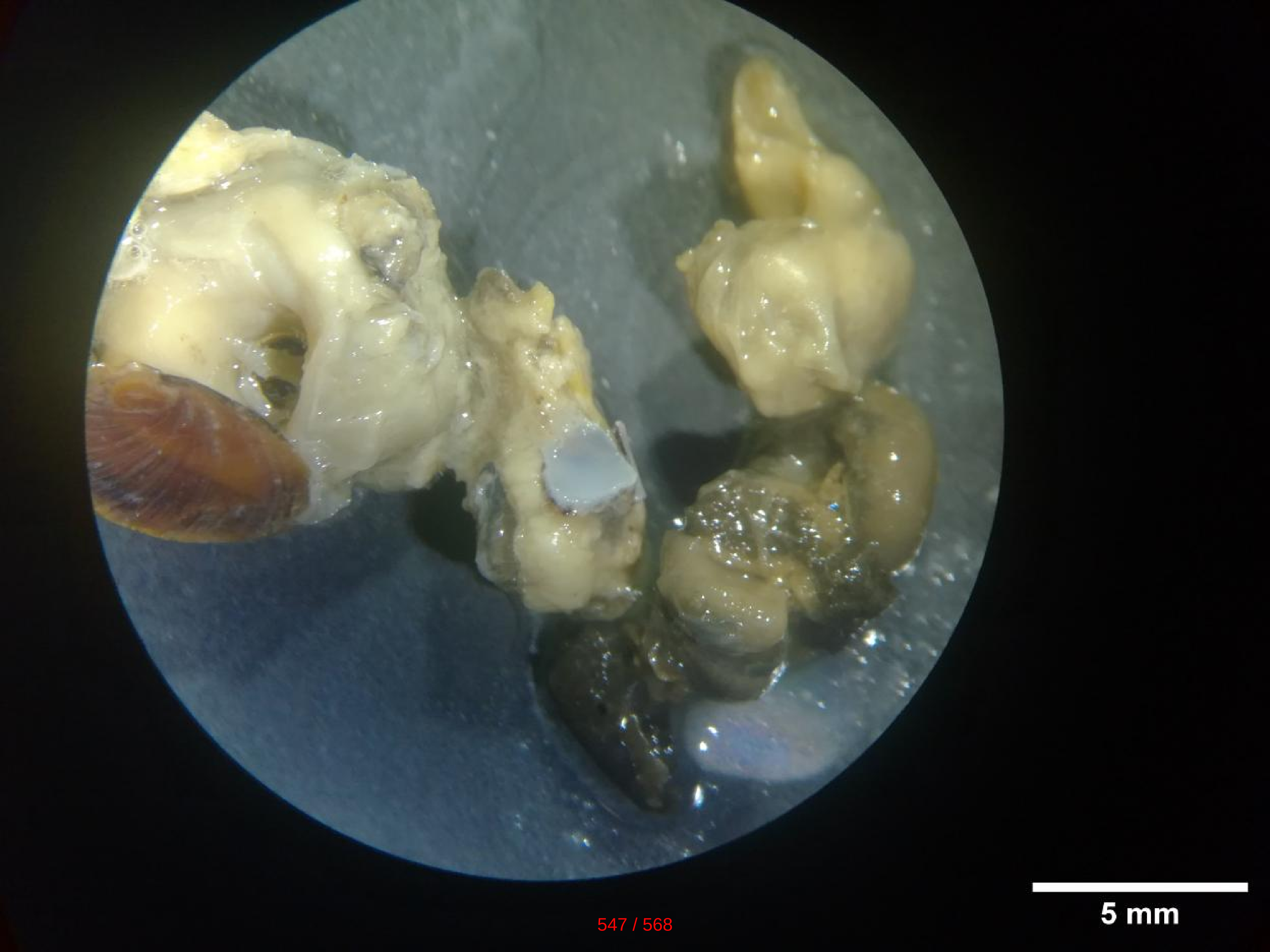
5 mm

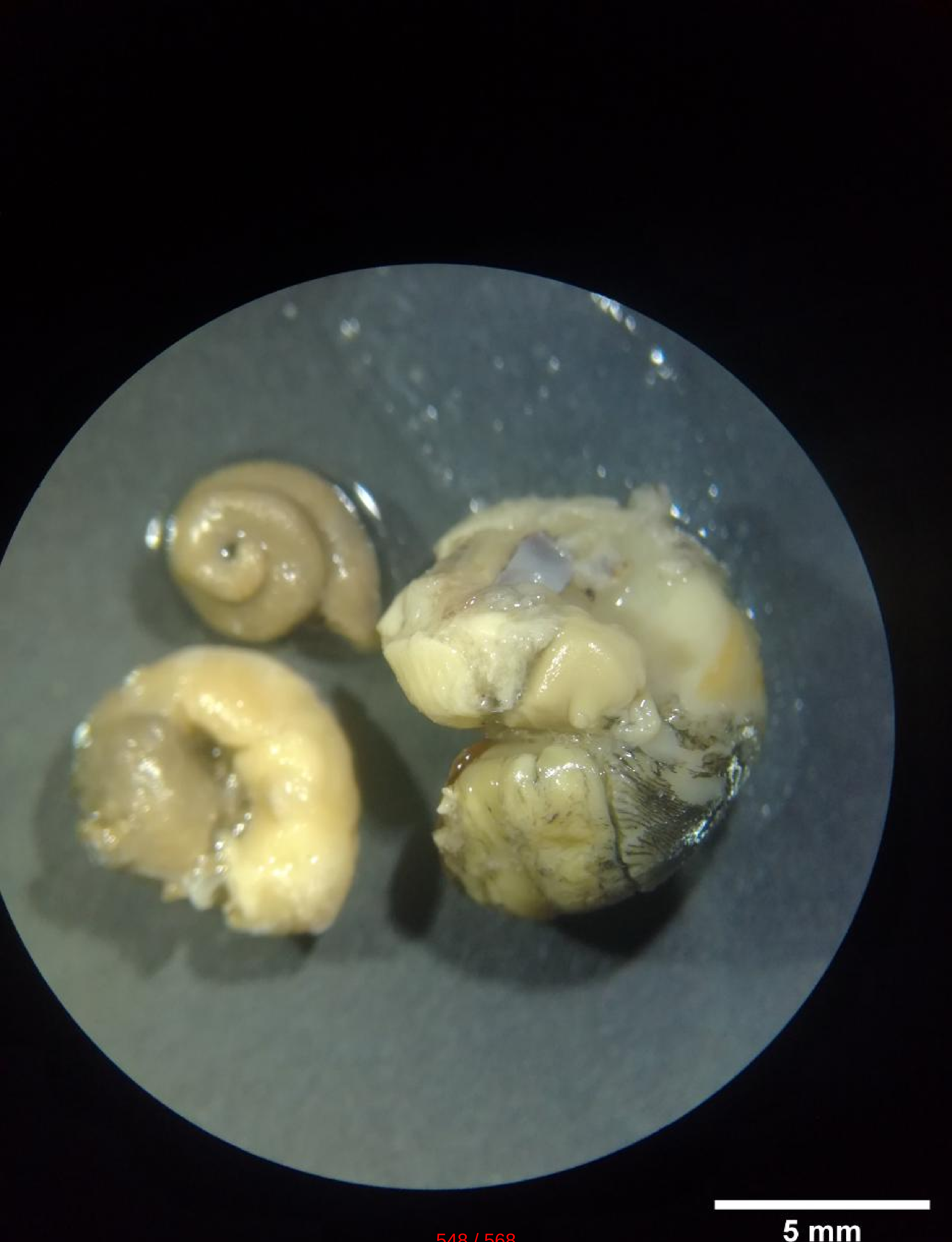
5 mm

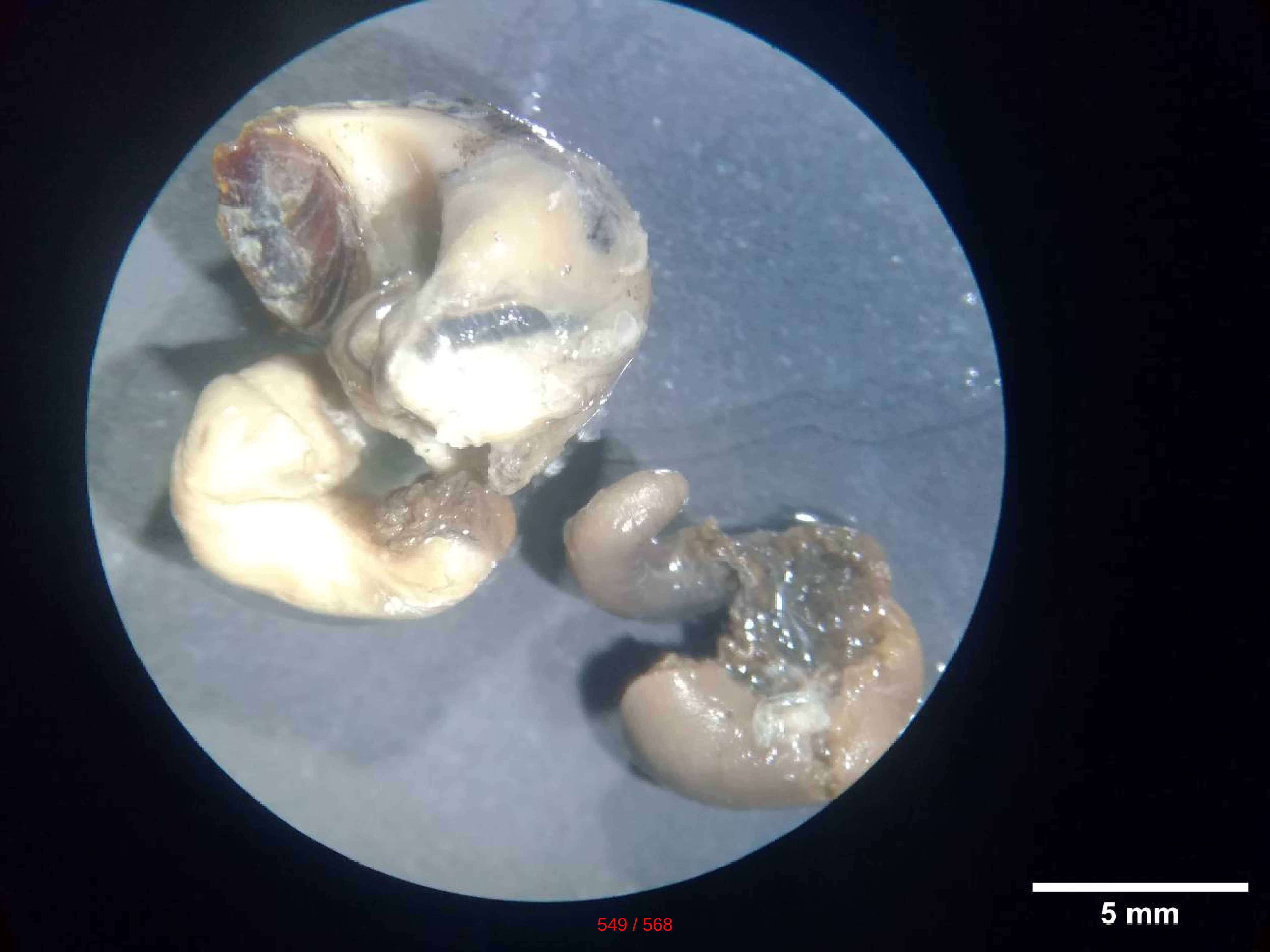
5 mm

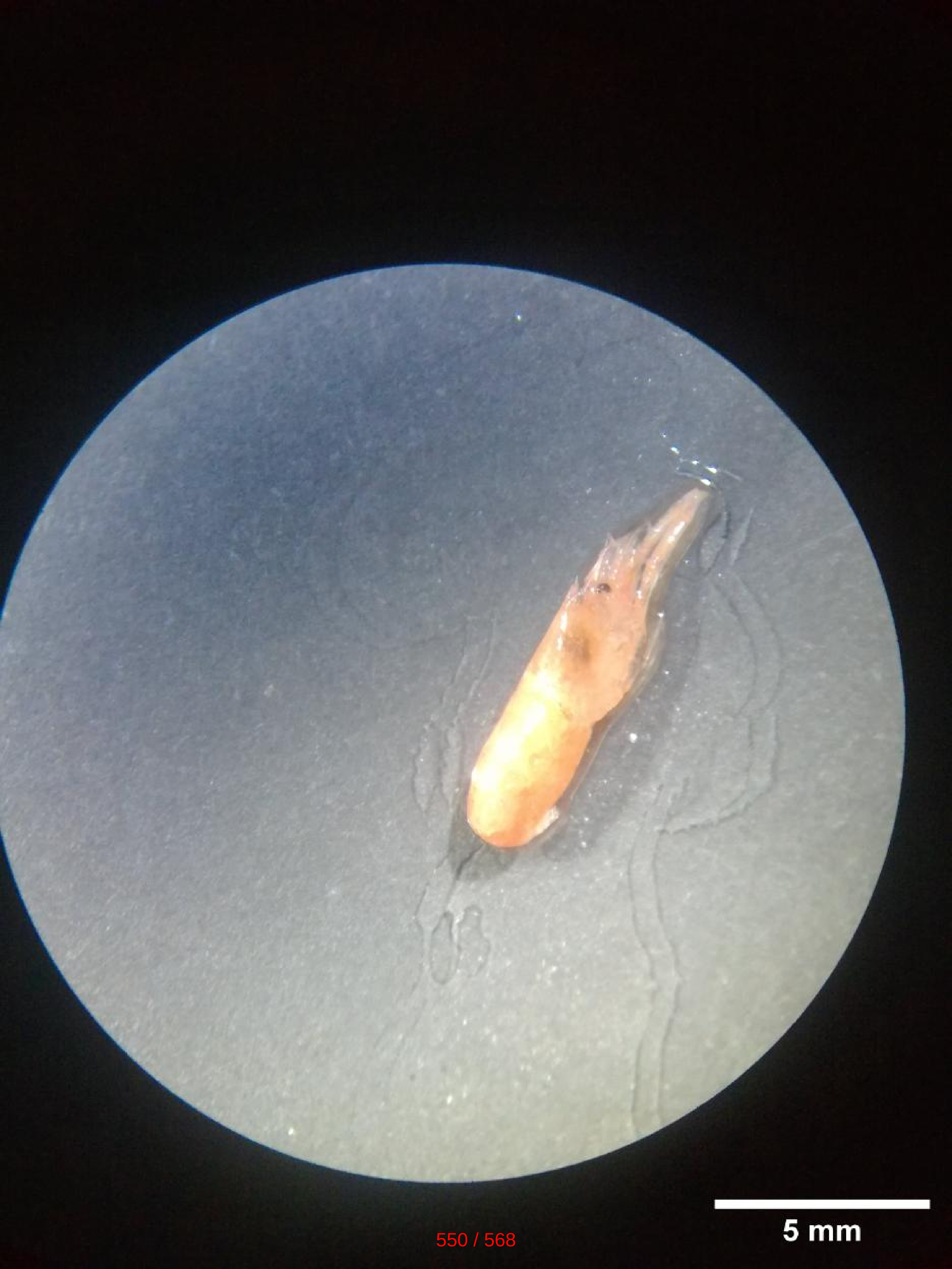
5 mm

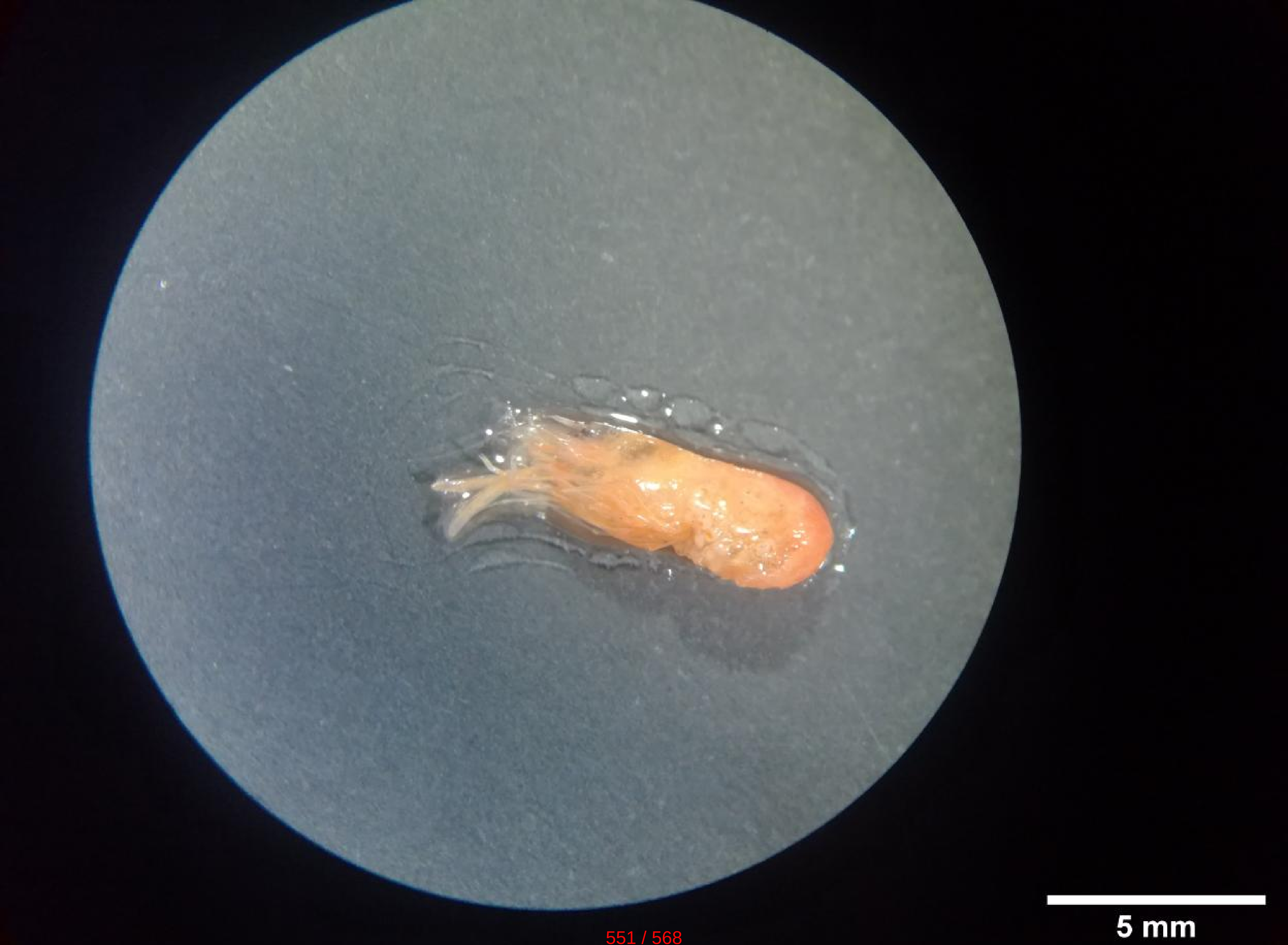
5 mm

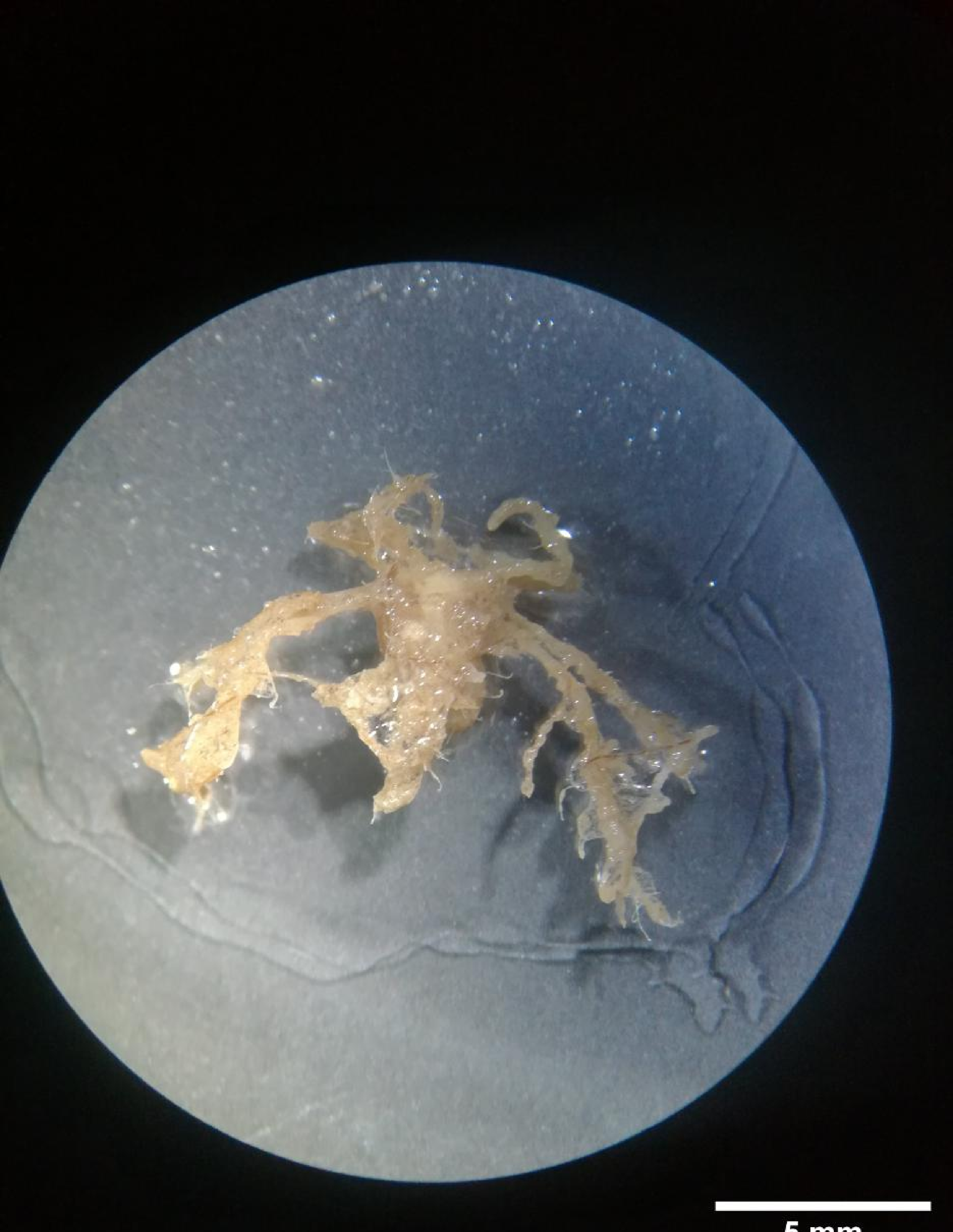
5 mm

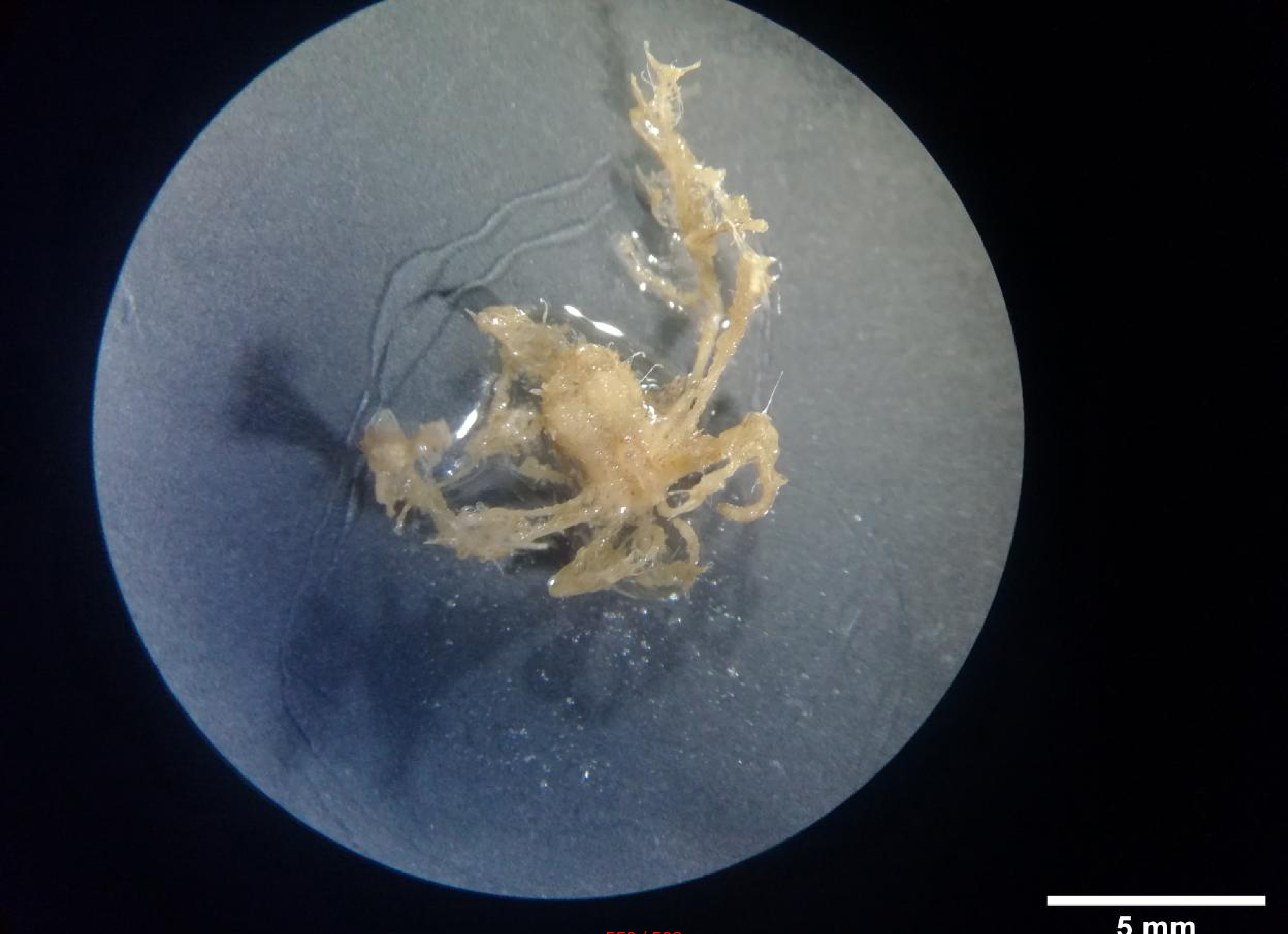
5 mm

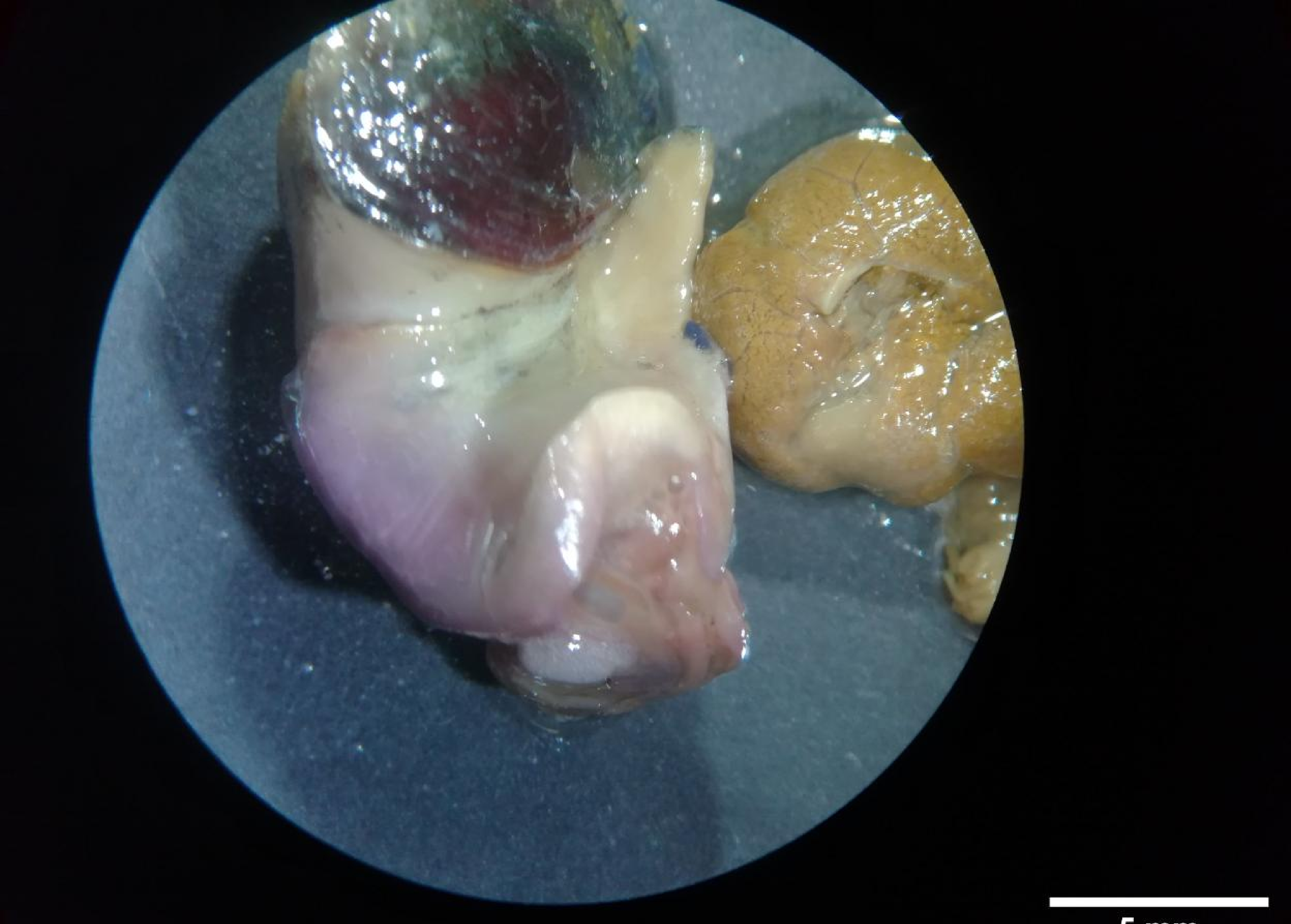
5 mm

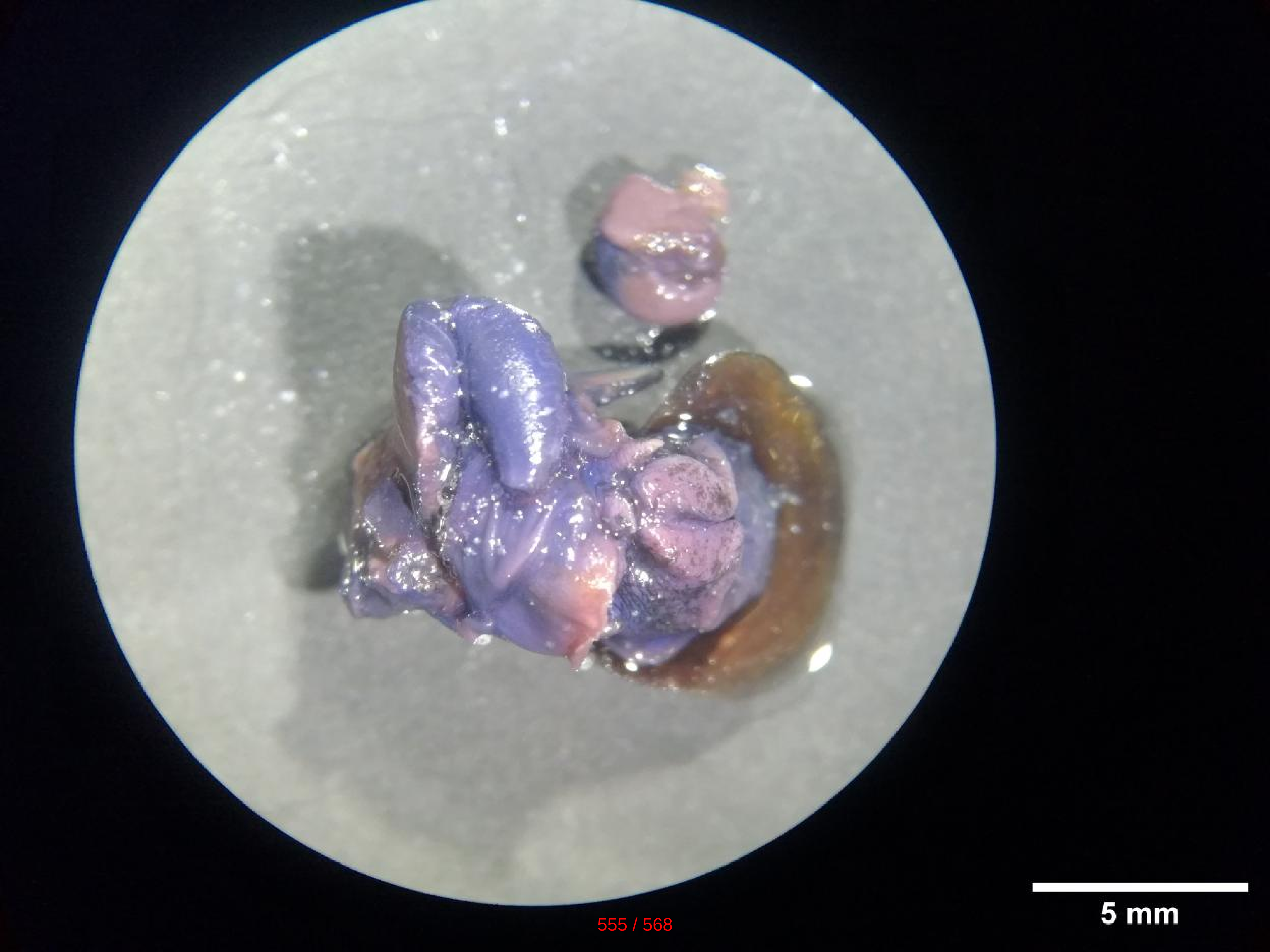
5 mm

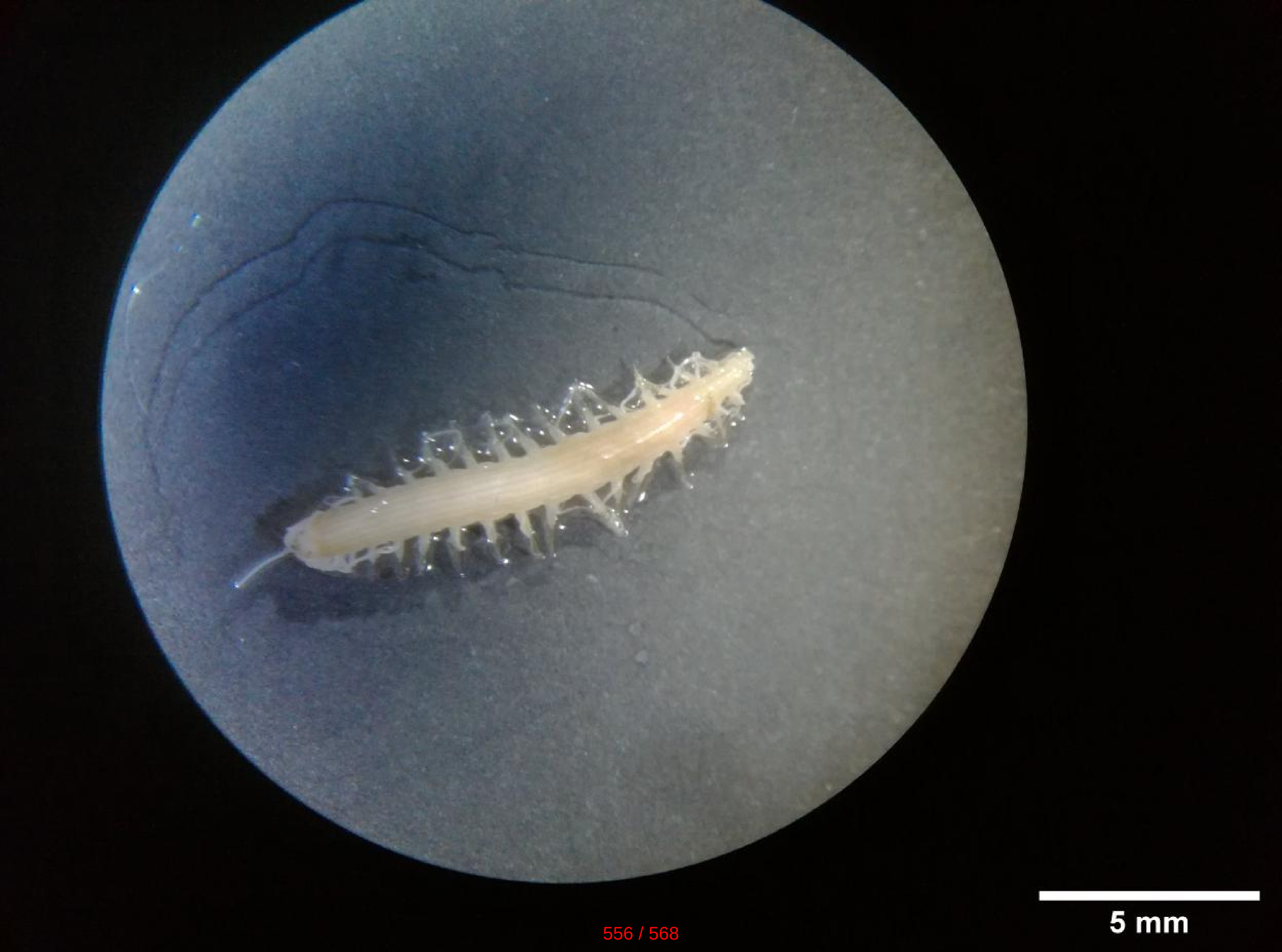
5 mm

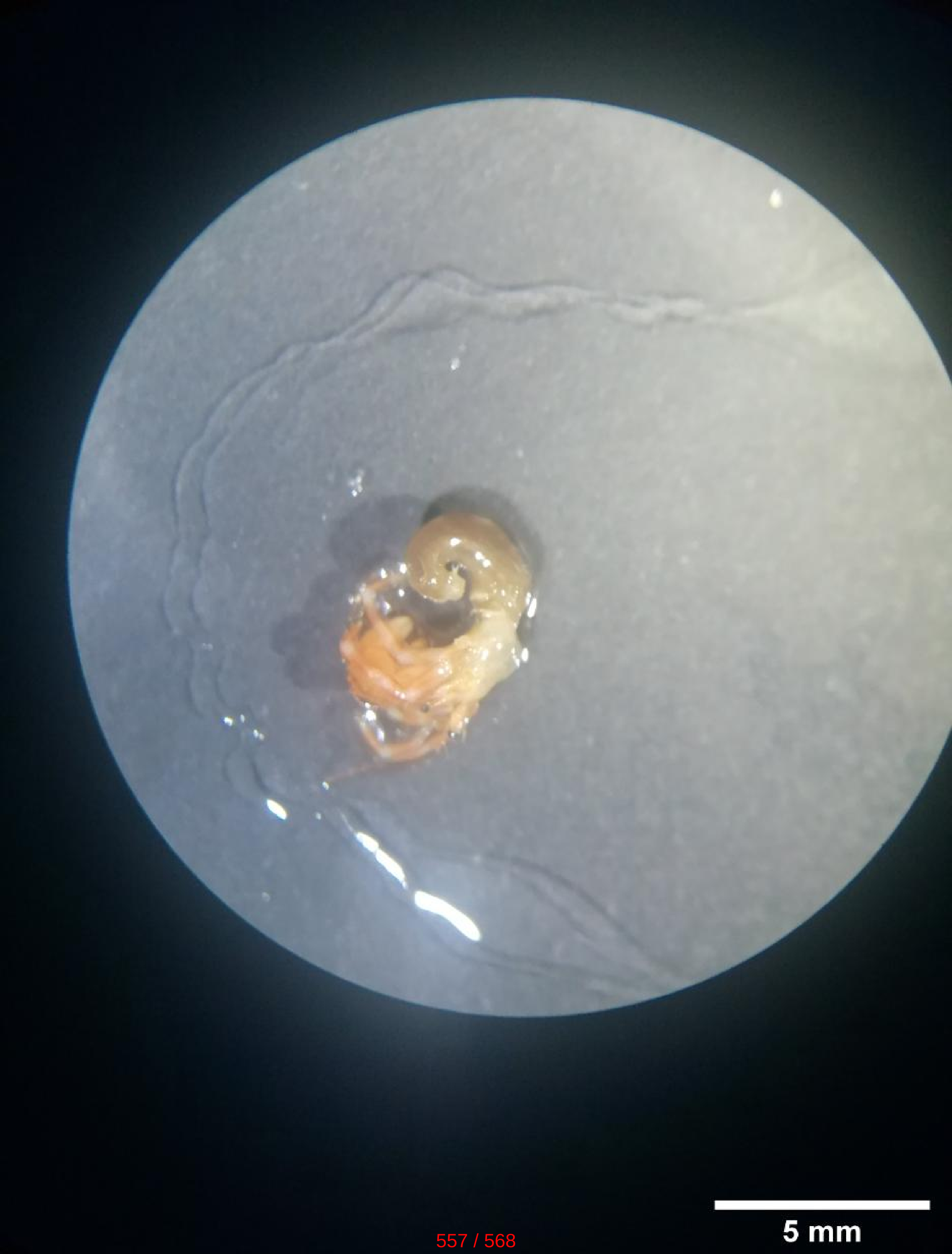
5 mm

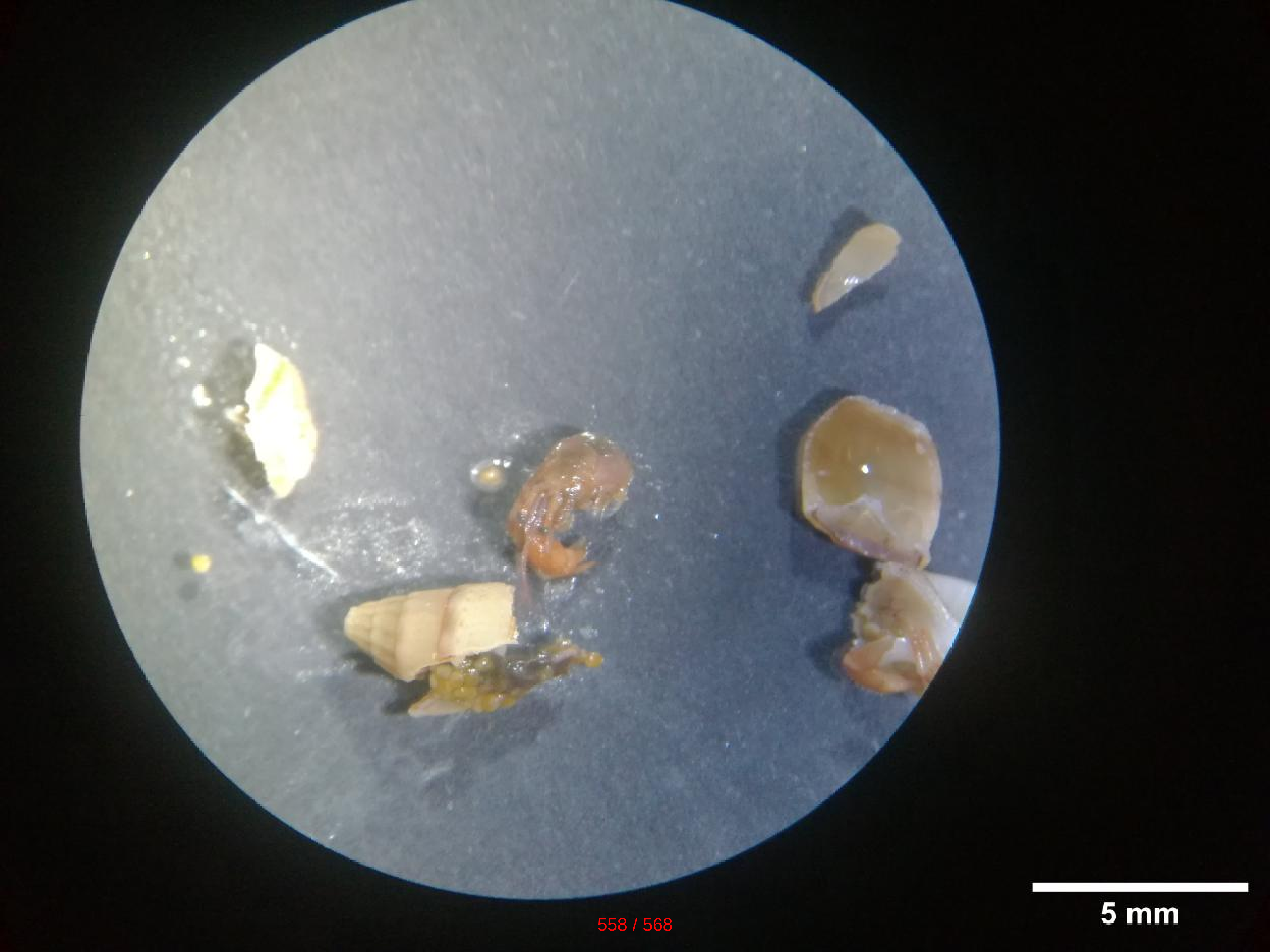
5 mm

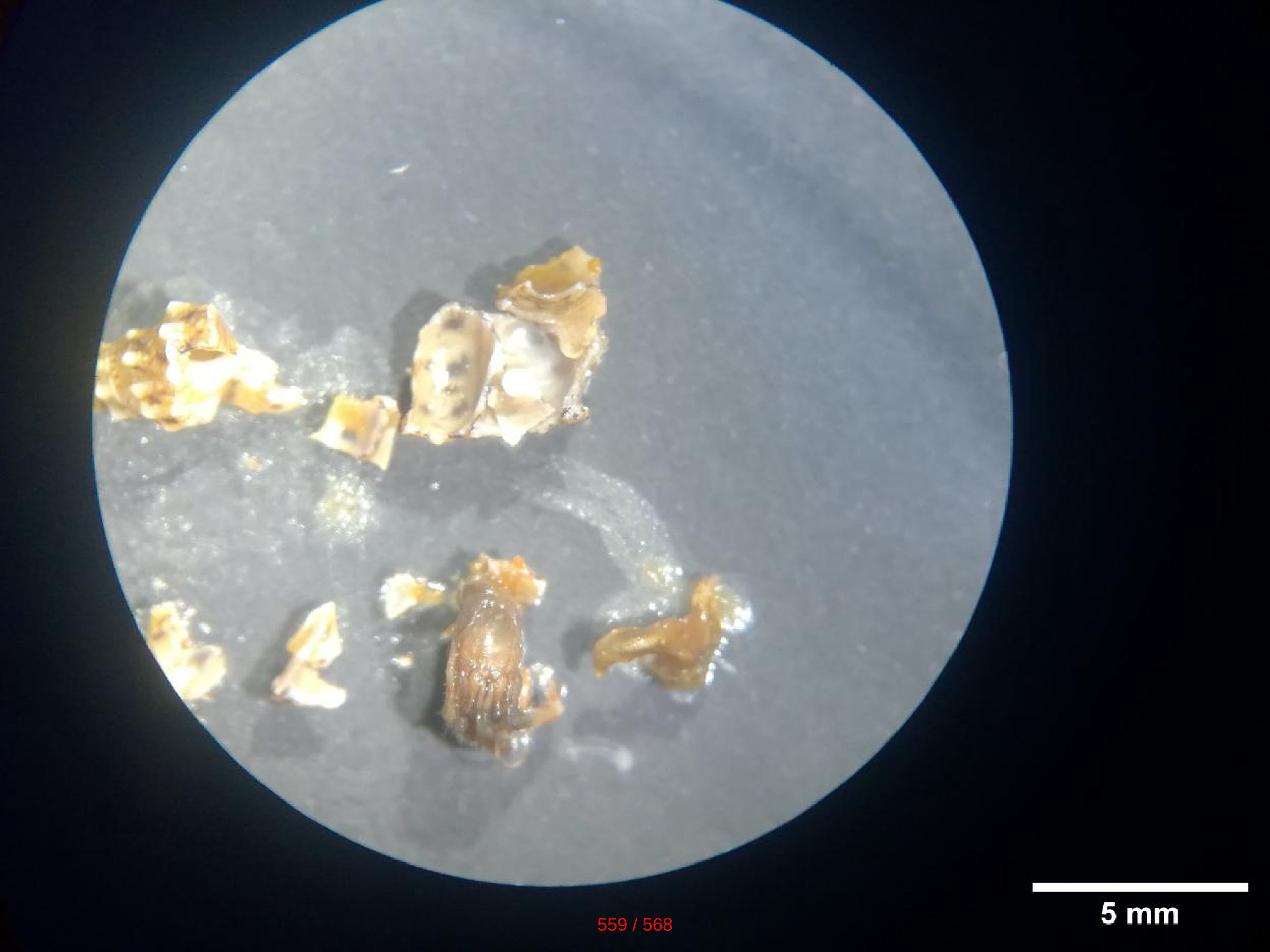
5 mm

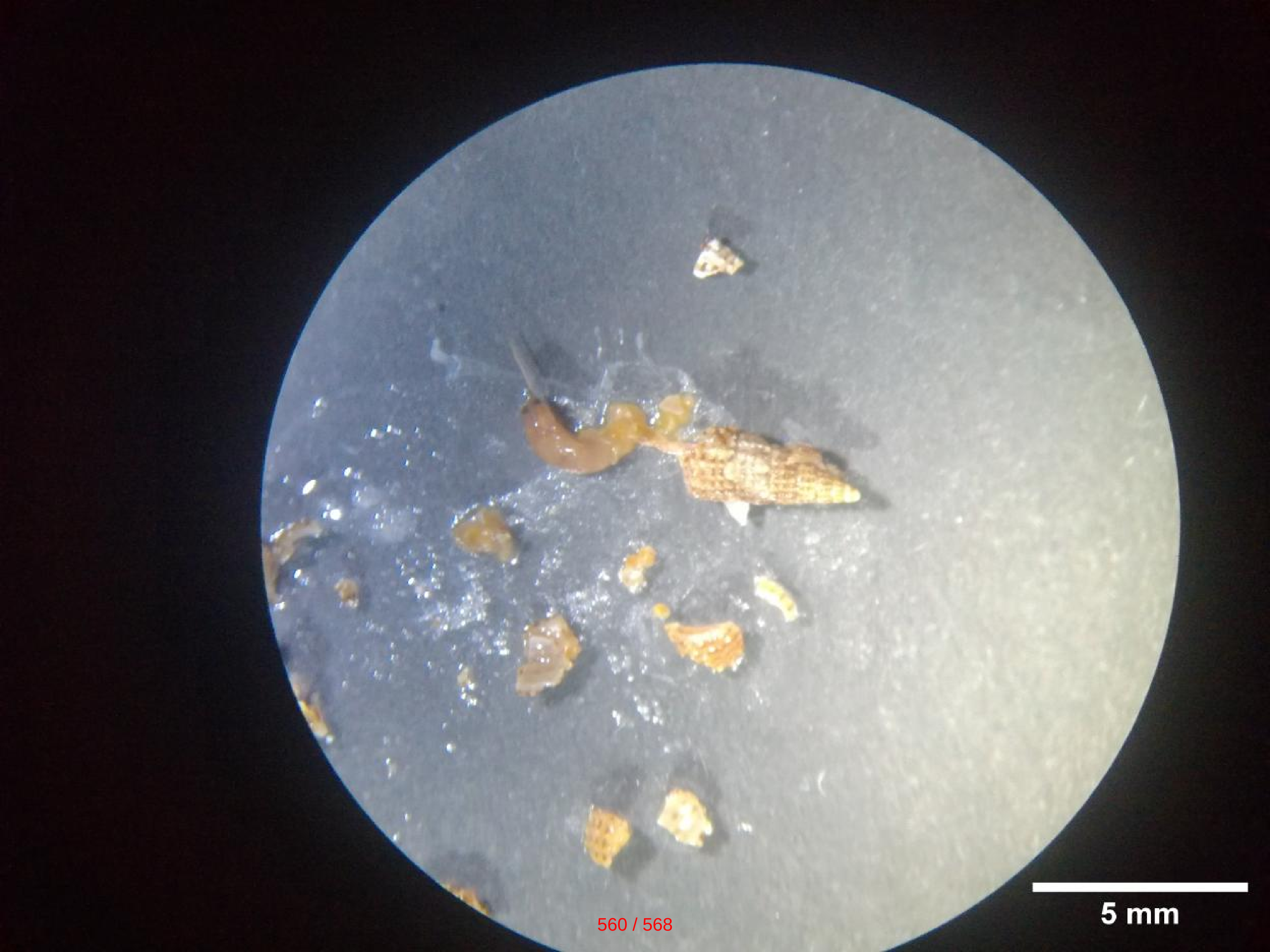
5 mm

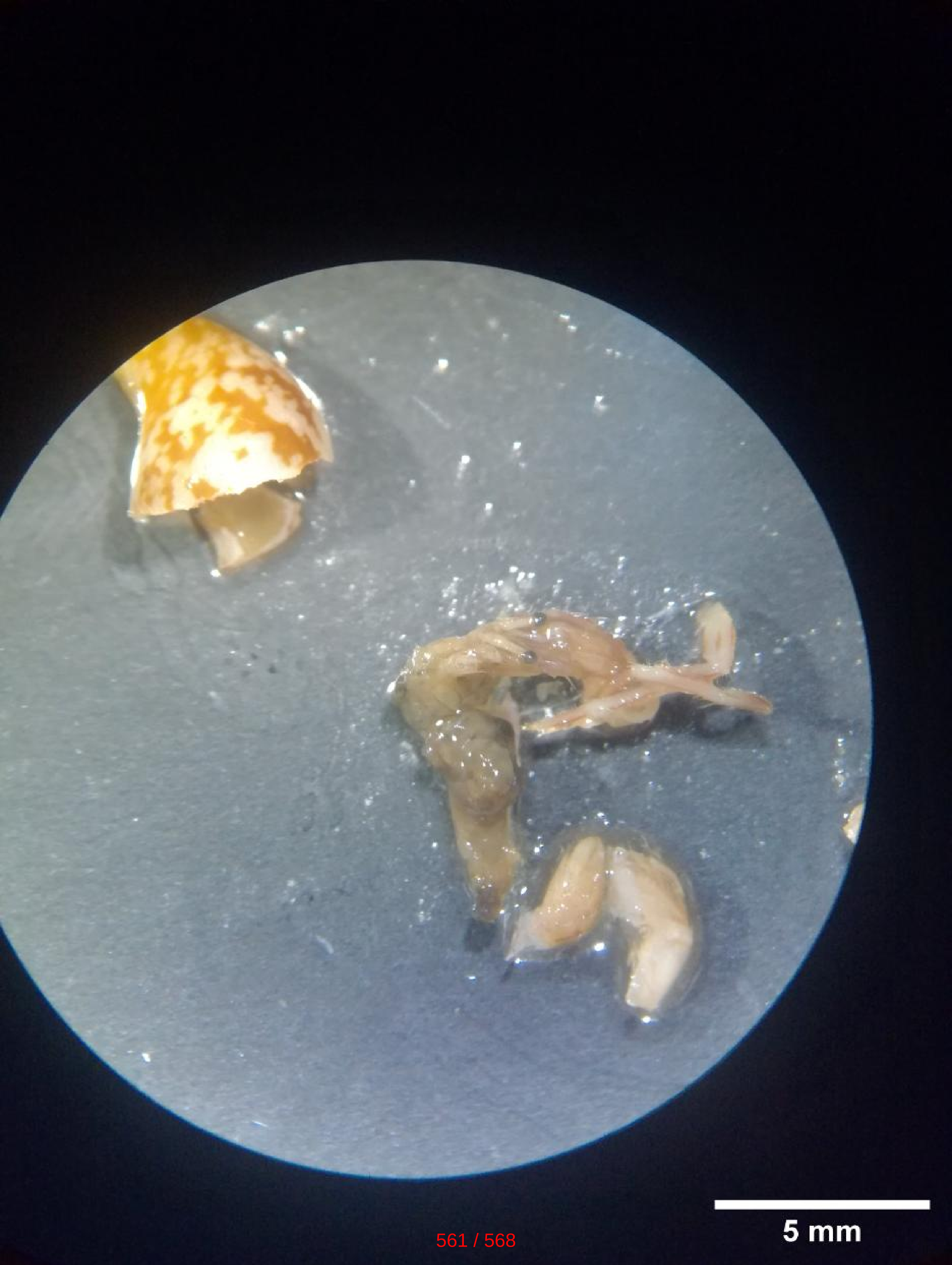
5 mm

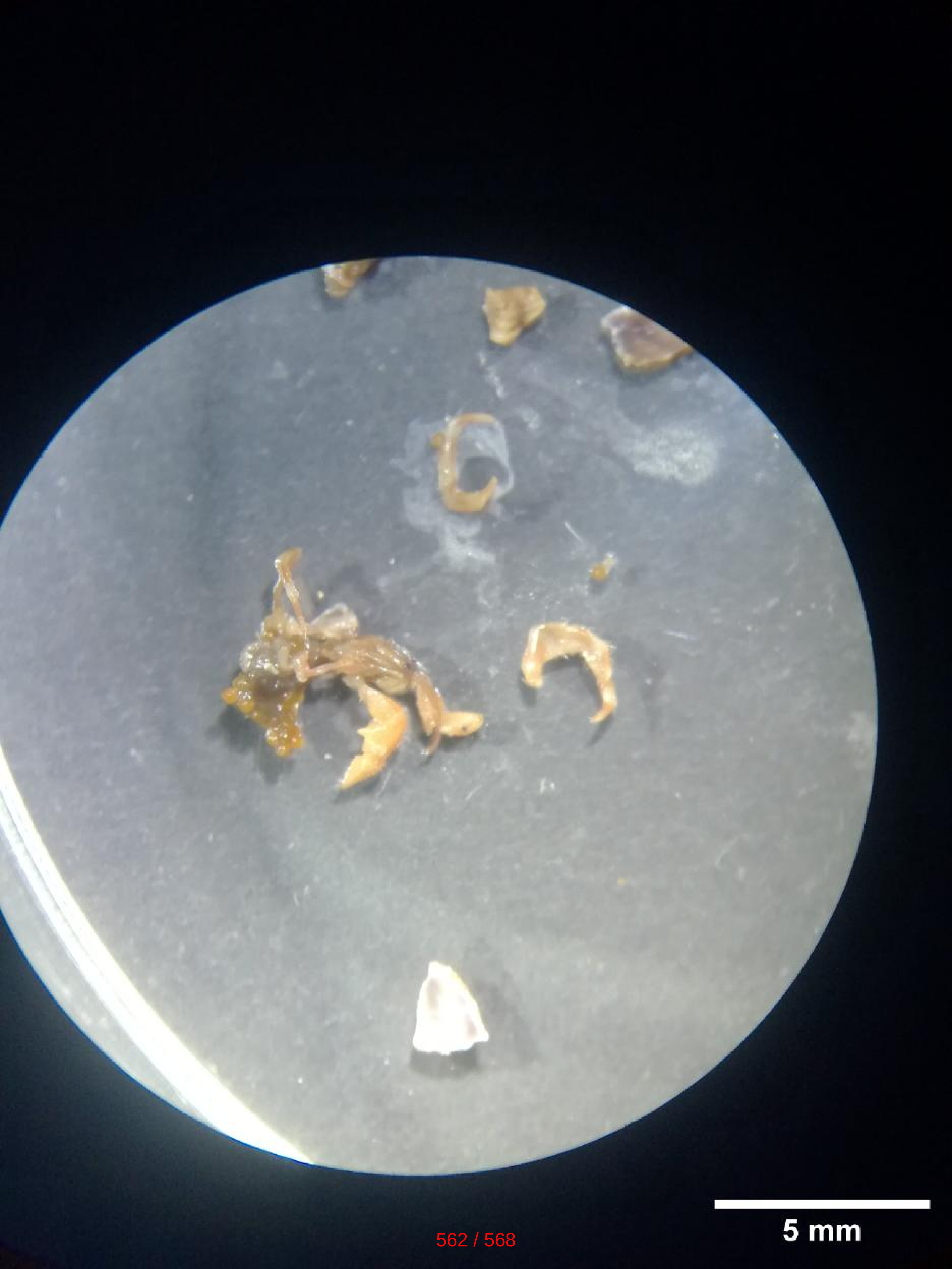
5 mm

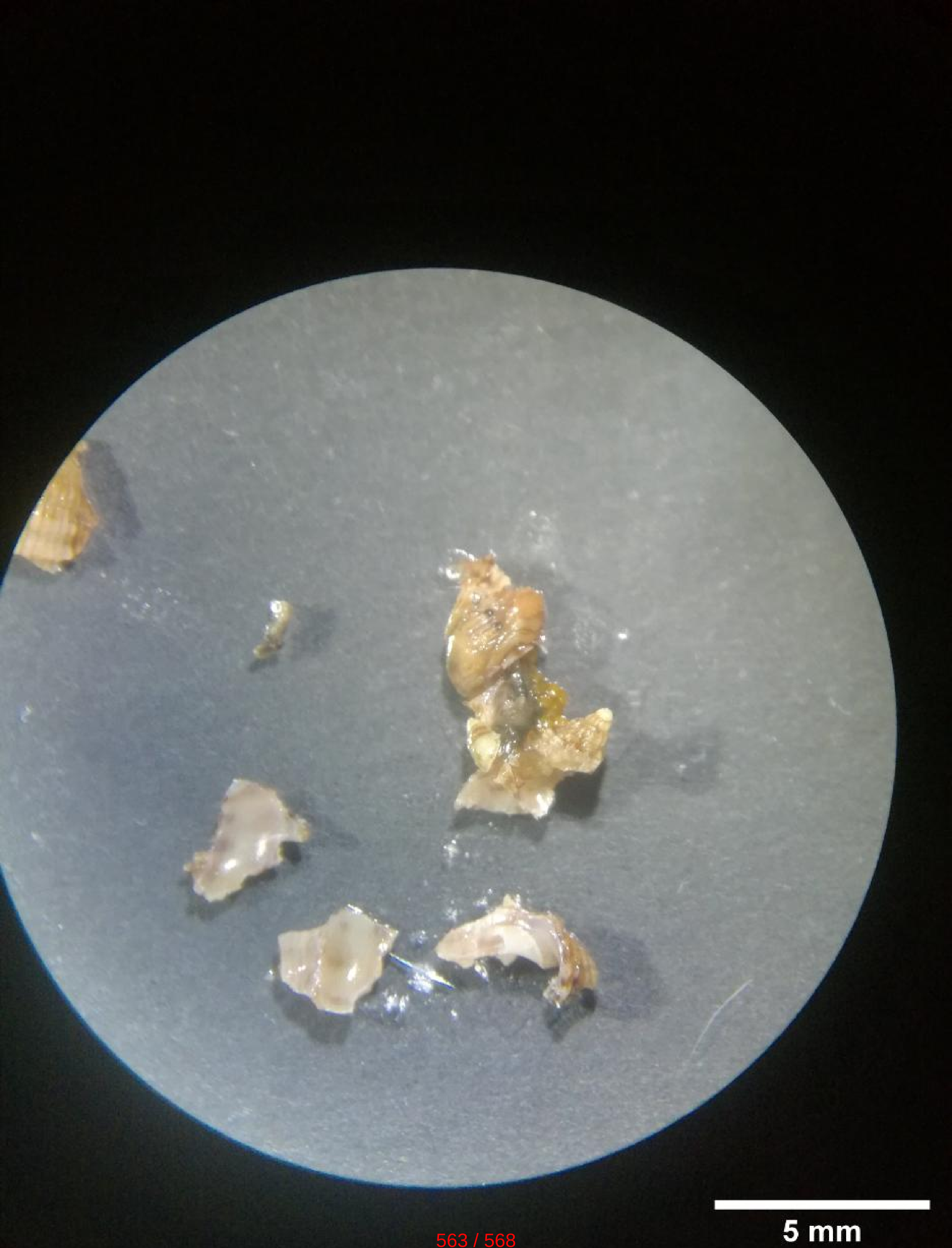
5 mm

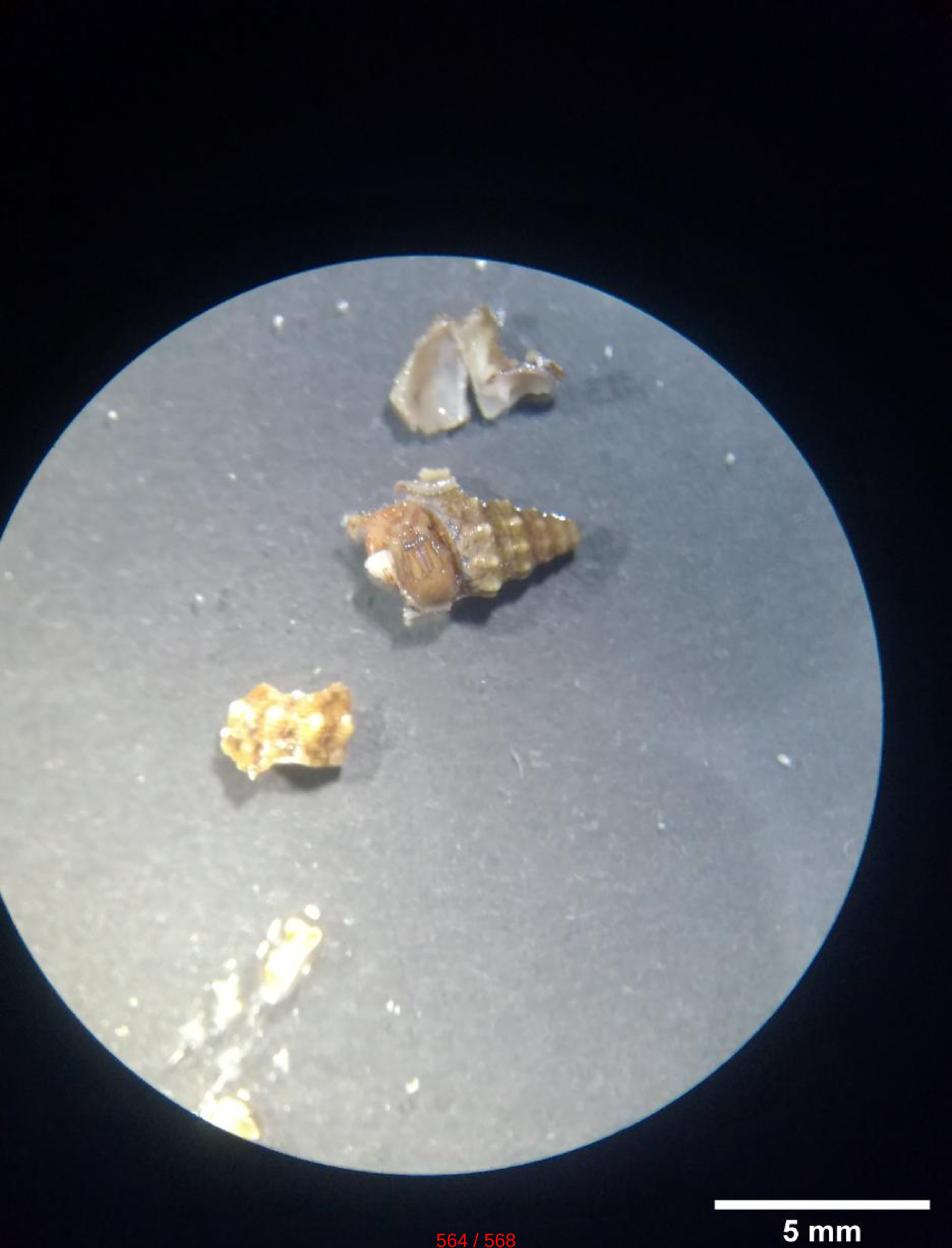
5 mm

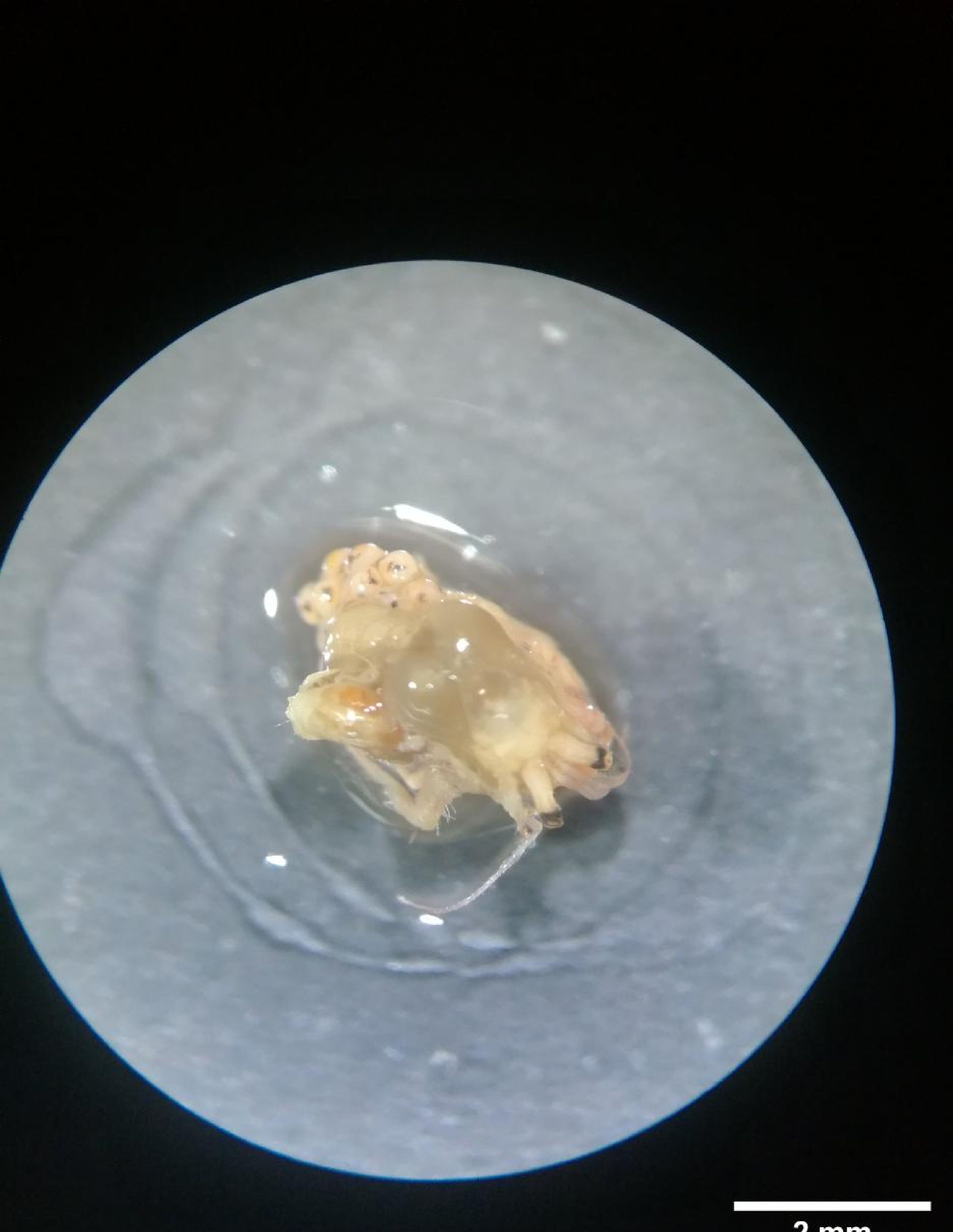
2 mm

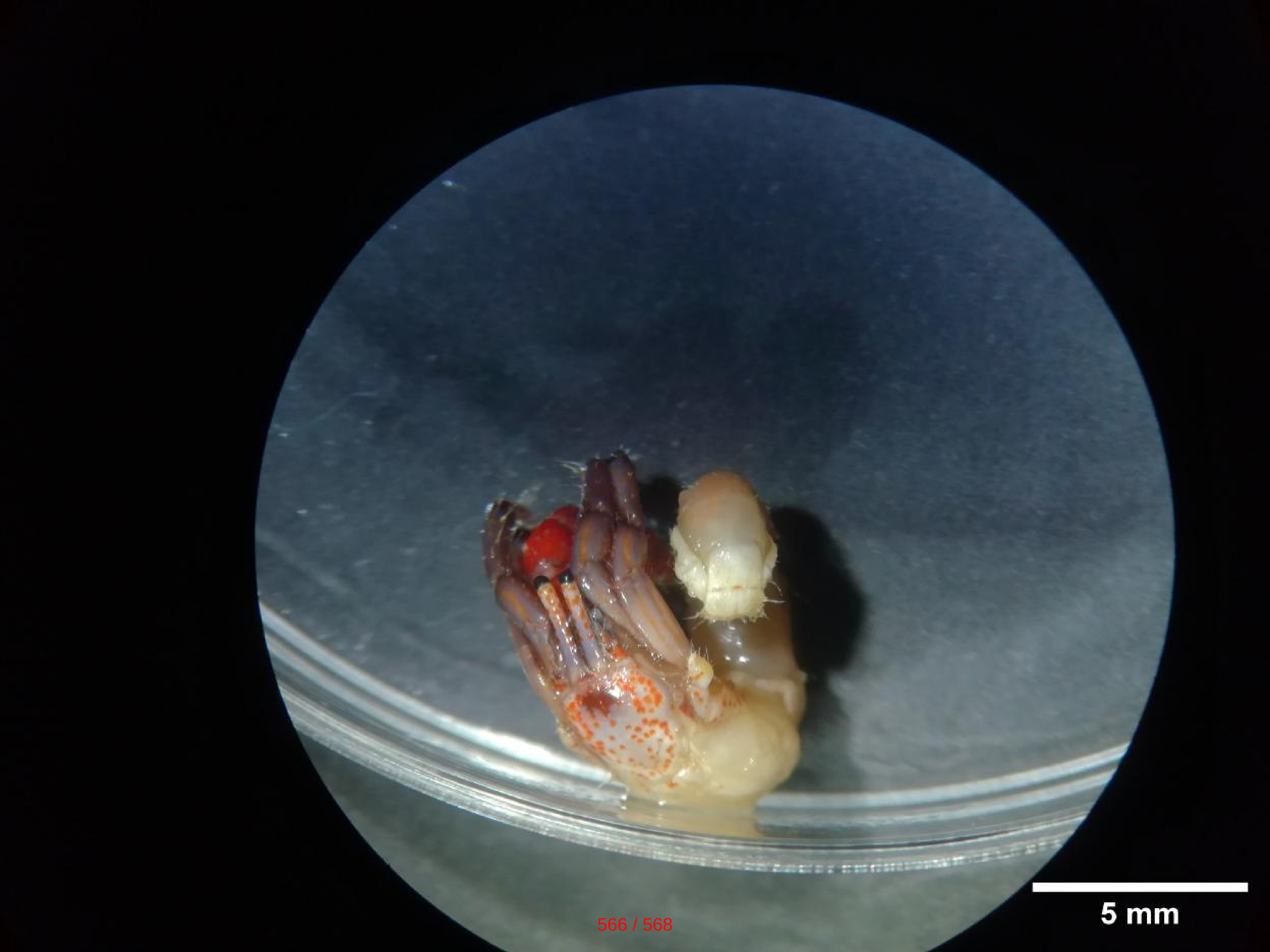
5 mm

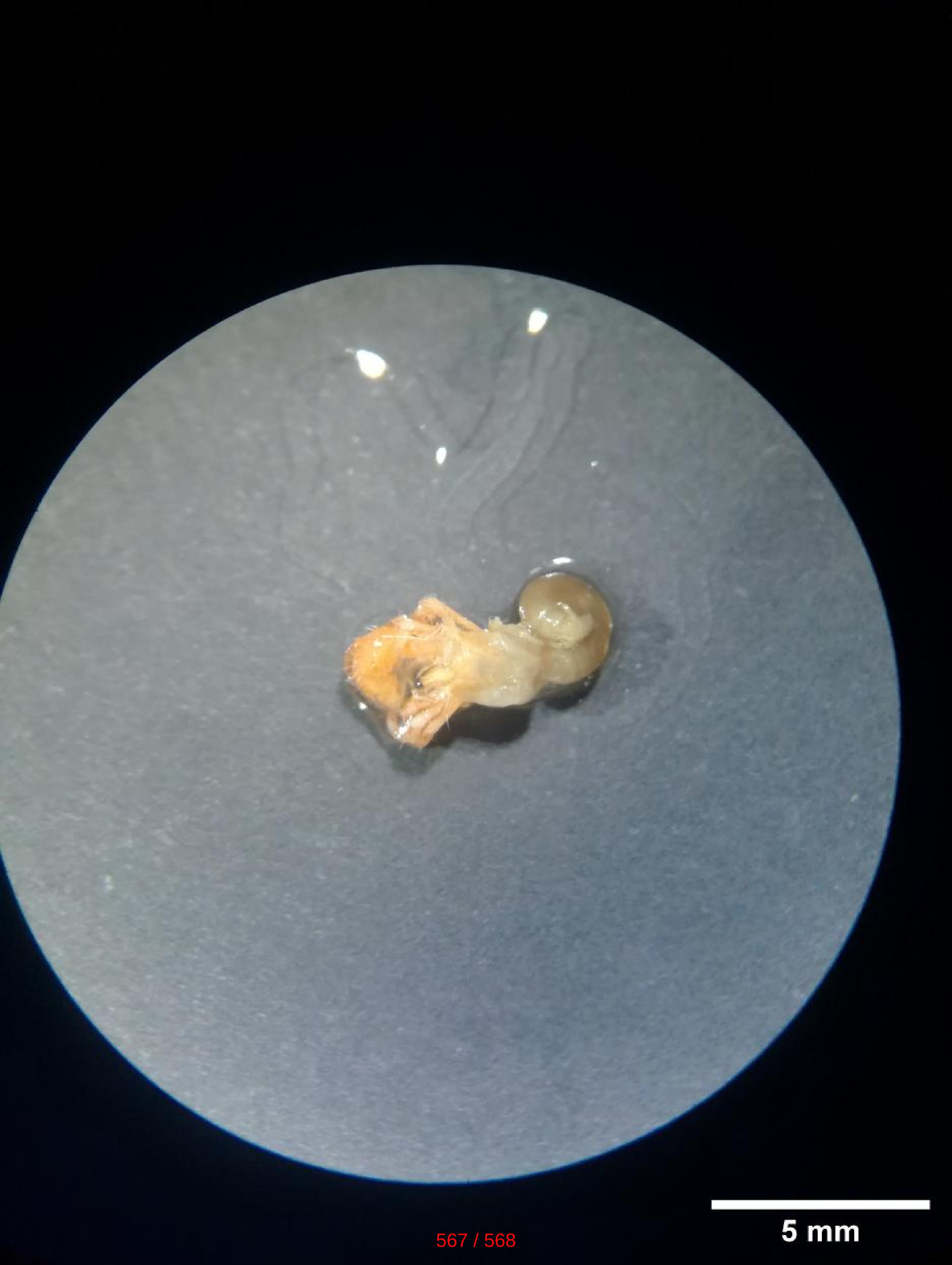
5 mm

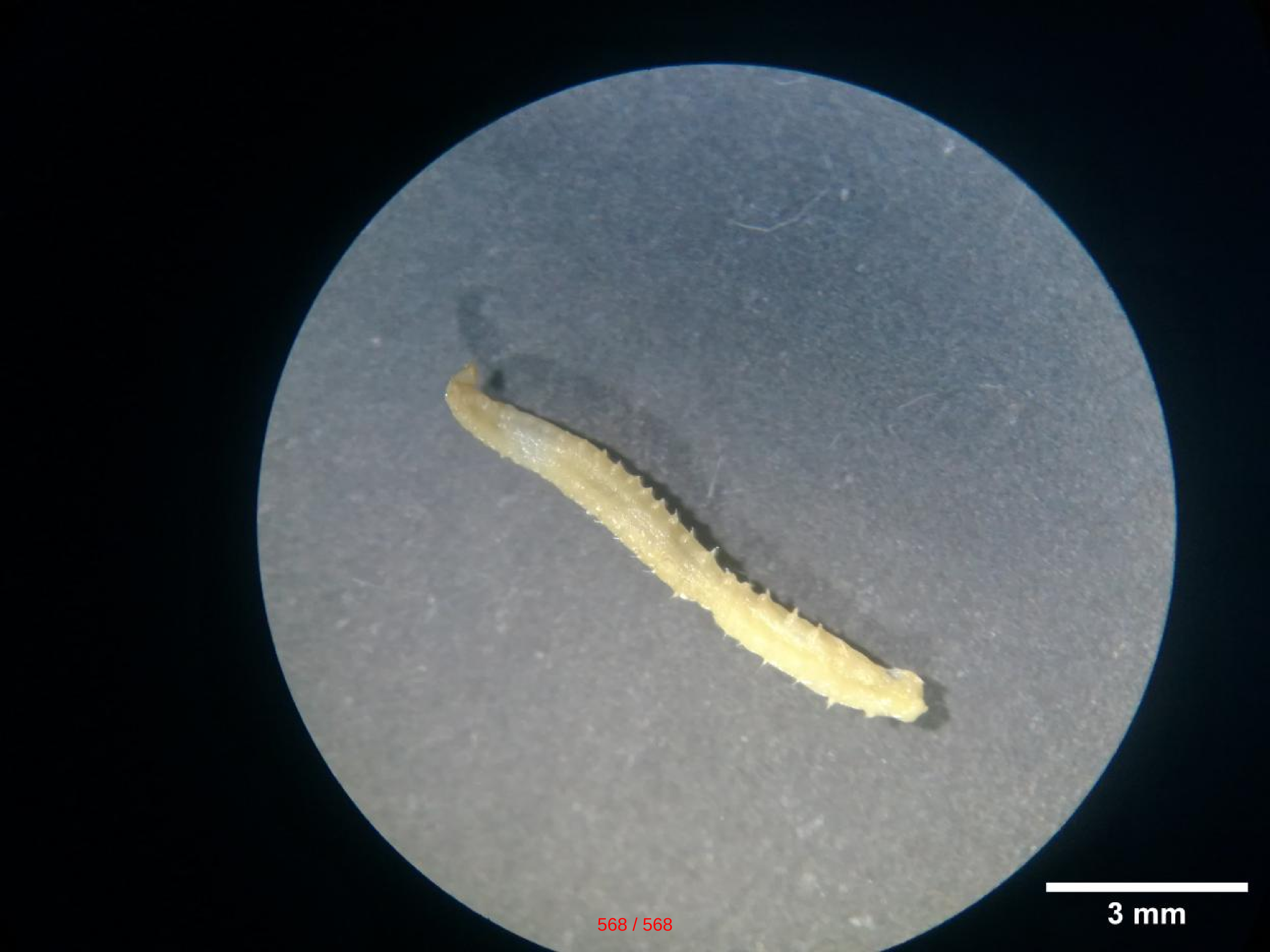
3 mm
